# RISCI - Repeat Induced Sequence Changes Identifier: a comprehensive, comparative genomics-based, *in silico *subtractive hybridization pipeline to identify repeat induced sequence changes in closely related genomes

**DOI:** 10.1186/1471-2105-11-609

**Published:** 2010-12-26

**Authors:** Vipin Singh, Rakesh K Mishra

**Affiliations:** 1 Centre for Cellular and Molecular Biology, Uppal Road, Hyderabad 500007, India

## Abstract

**Background -:**

The availability of multiple whole genome sequences has facilitated *in silico *identification of fixed and polymorphic transposable elements (TE). Whereas polymorphic loci serve as makers for phylogenetic and forensic analysis, fixed species-specific transposon insertions, when compared to orthologous loci in other closely related species, may give insights into their evolutionary significance. Besides, TE insertions are not isolated events and are frequently associated with subtle sequence changes concurrent with insertion or post insertion. These include duplication of target site, 3' and 5' flank transduction, deletion of the target locus, 5' truncation or partial deletion and inversion of the transposon, and post insertion changes like inter or intra element recombination, disruption etc. Although such changes have been studied independently, no automated platform to identify differential transposon insertions and the associated array of sequence changes in genomes of the same or closely related species is available till date. To this end, we have designed RISCI - 'Repeat Induced Sequence Changes Identifier' - a comprehensive, comparative genomics-based, *in silico *subtractive hybridization pipeline to identify differential transposon insertions and associated sequence changes using specific alignment signatures, which may then be examined for their downstream effects.

**Results -:**

We showcase the utility of RISCI by comparing full length and truncated L1HS and AluYa5 retrotransposons in the reference human genome with the chimpanzee genome and the alternate human assemblies (Celera and HuRef). Comparison of the reference human genome with alternate human assemblies using RISCI predicts 14 novel polymorphisms in full length L1HS, 24 in truncated L1HS and 140 novel polymorphisms in AluYa5 insertions, besides several insertion and post insertion changes. We present comparison with two previous studies to show that RISCI predictions are broadly in agreement with earlier reports. We also demonstrate its versatility by comparing various strains of *Mycobacterium tuberculosis *for IS 6100 insertion polymorphism.

**Conclusions -:**

RISCI combines comparative genomics with subtractive hybridization, inferring changes only when exclusive to one of the two genomes being compared. The pipeline is generic and may be applied to most transposons and to any two or more genomes sharing high sequence similarity. Such comparisons, when performed on a larger scale, may pull out a few critical events, which may have seeded the divergence between the two species under comparison.

## Background

Mobile or transposable elements (TEs) are DNA sequences that have the ability to hop (transpose) in the genome, within their cell of origin. TEs constitute a highly diverse class of repeat elements [1,2] and have been reported in all genomes sequenced till date except *Plasmodium falciparum *[[3], reviewed in [4]]. Based on the mechanism of transposition [reviewed in [5]], TEs are broadly divided into two classes - Class I or Retrotransposons and Class II or DNA transposons. Retrotransposons transpose via an RNA intermediate which is reverse transcribed and integrated into the genome, thereby duplicating the element (copy paste mechanism). DNA transposons, on the other hand, excise from their source locus to reinsert at a new one without the involvement of an RNA intermediate (cut paste mechanism) [1].

TEs represent miniature genomes with a versatile repertoire of cis regulatory elements and/or trans acting factors. Long relegated as selfish DNA [6,7], they are turning out to be a treasure trove of genomic novelties as their impact on host genome evolution is beginning to be understood [8-13]. Besides serving as an inexhaustible source of novel genes and exons [13-20], gene functions [21-23], and regulatory motifs and signals [24-27], the insertion of a transposon at a locus may change its properties drastically with local and/or long range or global consequences [10,28-31]. These changes are more palpable when a transposon insertion results in gene disruption and is manifested as a disease condition [32-34]. Such insertions may be subject to negative selection and lost in due course [35].

Most transposon insertions that persist are, therefore, either silent or result in subtle and/or adaptive changes. The cumulative impact of these subtle changes may account for the observed phenotypic, physiological and behavioral differences between closely related genomes that share a high degree of sequence similarity [36]. Notable examples include human-specific inactivation of the CMP-N-acetylneuraminic acid hydroxylase gene via Alu- mediated replacement resulting in widespread biochemical difference between human and non human primates [37] and the loss of exon 34 of tropleolastin gene in human via an Alu recombination-mediated deletion [38].

The challenge, then, is to selectively identify these differential insertions and the consequent alteration of the target locus. To this end, we have designed RISCI - "Repeat Induced Sequence Changes Identifier", a comprehensive comparative genomics based *in silico *subtractive hybridization pipeline to identify such changes, if exclusive to one of the two genomes being compared. It is modeled on LINEs or Long Interspersed Nuclear Elements (non Long Terminal Repeat retrotransposons) [reviewed in [39]], since they display a wide array of sequence changes upon insertion, such as target site duplication, 3'and 5' flank transduction, deletion of target locus upon insertion, inversion and truncation of repeat sequence during transposition besides post insertion modifications like disruption and recombination [40]. In the test dataset of 302 full length L1HS elements (LINE1- Human Specific) in the reference human genome, RISCI predicted and confirmed 26 human-specific 3' flank transduction events (in comparison with the chimpanzee genome), predicted 14 novel insertion polymorphism (compared to alternate human assemblies - Celera and HuRef), 1 inter element recombination in the human genome resulting in the loss of 13.4 kb of sequence and 4 inter element recombination events in the chimpanzee genome. 42 Human specific 3' flank transduction and at least 24 novel polymorphic insertions, besides several recombination events were inferred from analysis of truncated L1HS retrotransposons. RISCI also predicted 140 novel AluYa5 polymorphic insertions in the reference human genome (in comparison with alternate human assemblies - Celera and HuRef).

## Results

**Figure 1:**
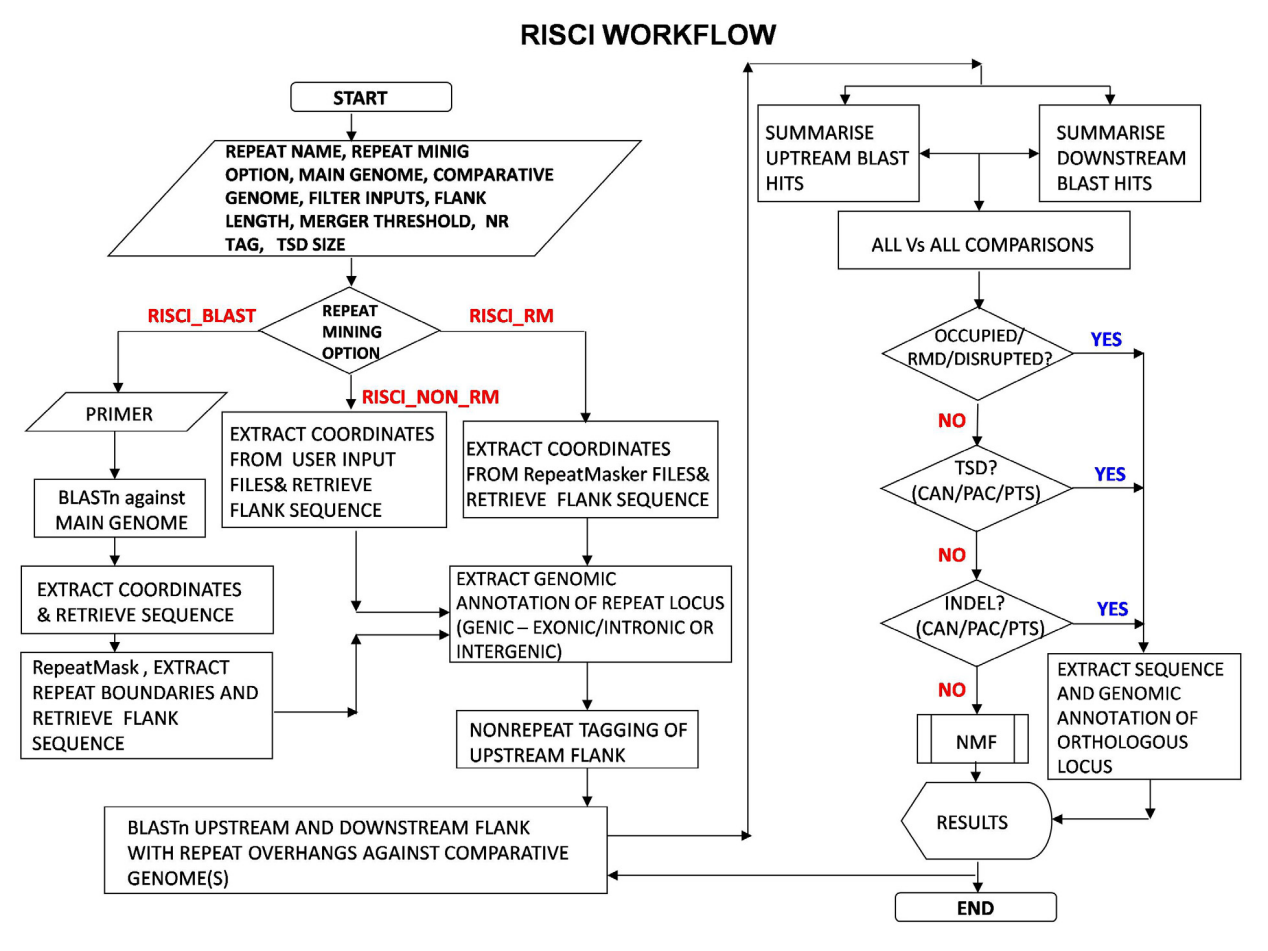
**RISCI flow chart depicting basic steps of the pipeline**. Major inputs include the repeat name (as identified by Repeat Masker), repeat mining option, filter inputs, flank length (default 5000 bases), merger threshold (default 50 bases), size of non-repeat tag (default 500 bases), maximum target site duplication (TSD) size (default 50 bases) and speed options. The genomic annotation of the repeat locus is parsed from the gen bank file if made available. The upstream sequence is tagged with user defined length of non repeat sequence wherever possible (default 500 bases). The upstream and downstream flanks, each carrying 50 base overhang into the repeat, are BLASTed separately against the comparative genome(s) and the BLAST alignment files summarized. For each repeat locus in the main genome, all upstream blast hits are compared against downstream blast hits in the same orientation and sequentially checked for shared ancestry (occupied), post insertion changes (recombination-mediated deletions, disruptions etc.), target site duplication (orthologous locus empty) and classified as CAN, PAC or PTS. If no matches for TSD are found both on the corresponding chromosomal homologue as well as on other chromosomes, the locus is checked for miscellaneous events (INDELs) like insertion-mediated deletion or parallel insertion or insertion deletion on corresponding chromosomal homolog, else the locus is reported as no match found (NMF). In the final results file, RISCI annotation for each locus, genomic annotation of the repeat loci in the main or reference genome and of the orthologous locus in the comparative genome, percentage repeat content of the flanks, Blastn coordinates (query and subject) for the flanks, size of TSD or INDEL or RMD are reported. In case of TSD, sequence of 5' and 3' TSD in the reference genome and of the lone copy of TSD in the comparative genome are also reported.

RISCI is a comparative genomics-based pipeline which sequentially picks the transposon loci in one genome ('Reference ' or 'Main' genome), using one of the three repeat mining options (see materials and methods), and precisely zooms into the corresponding orthologous loci in other genome(s) ('Comparative genome(s)') using user defined length of flanks (default 5000 bases) extending 50 bases into the transposon (repeat overhangs) and Blastn [41]. It then infers the nature of alteration either at the transposon locus in the reference genome or the ortholog in the comparative genome(s), based on event specific-alignment signatures (discussed below). The genomic context (intergenic or genic, if genic - exonic or intronic) of the transposon locus in the reference genome and the ortholog in the comparative genome(s) is also integrated by parsing the annotation files, if available. For each transposon locus in the reference genome, RISCI sequentially assesses whether the orthologous locus in the comparative genome is occupied (indicating shared ancestry), has undergone post insertion changes, or is empty. If empty, RISCI infers insertion-associated sequence changes based on the location of target site duplication (TSD - discussed later). If TSD is not found, the orthologous locus is checked for insertion-mediated deletion or parallel independent insertions or insertion deletion at the orthologous locus (Figure 1).

RISCI was tested on full length (>6 Kb) (Table 1, Additional files 1, 2 and 3) and truncated L1HS elements (Table 1, Additional files 2, 4, 5 and 6) and AluYa5 (Table 1, Additional files 7, 8, 9 and 10) human-specific retrotransposons with the reference human genome [42] as the reference or main genome and the reference chimpanzee [43] and alternate human assemblies, Celera [44] and HuRef [45], as the comparative genomes. RISCI predicted several polymorphic loci in reference human genome comparison with the alternate human assemblies (Additional files 11 and 12). To test the efficacy of RISCI, we present a comparison with the data of Mills et al (Additional file 13) and partially recapitulate a study published earlier by Sen et al [46] (Additional files 14 and 15). Further, to demonstrate that RISCI can handle other transposon classes in other related genomes as well, we present a preliminary analysis checking for presence-absence of IS element (DNA transposon) in various strains of *Mycobacterium tuberculosis *(Additional file 16). We describe here in details the findings of a study on full length and truncated L1HS and AluYa5 retrotransposons.

**Table 1:** RISCI annotates the transposon locus in the main genome or the orthologous locus in the comparative genome into several classes based on specific alignment signatures.

Data set		L1HS (Full length)			L1HS (Truncated)			AluYa5 (all)		
Reference genome - Reference human genome		Comparative genomes			Comparative genomes			Comparative genomes		
**Class**	**RISCI annotation**	**Chimp**	**Celera**	**HuRef**	**Chimp**	**Celera**	**HuRef**	**Chimp**	**Celera**	**HuRef**
										
**Shared ancestry**	OCCUPIED	1	217	171	274	1227	1174	314	3529	3334
										
**Post insertion changes**	C_DISRUPTED_M_INTER_RMD	1	7	43	16	8	11	5	9	6
	C_INTER_RMD_M_DISRUPTED	4	12	11	32	13	21	90	22	74
	M_INTRA_RMD	0	0	0	32	7	36	0	0	0
	C_INTRA_RMD	0	15	24	10	3	12	0	0	0
										
**Orthologous locus empty**	CAN	170	27	25	426	62	76	3132	326	420
	PAC	68	9	10	109	16	22	54	2	4
	PTS	32	3	3	78	12	14	23	2	4
										
**INDELS**	INDEL_CAN	3	2	7	43	4	6	164	34	79
	INDEL_PAC	6	1	1	14	1	2	7	1	0
	INDEL_PTS	9	5	5	28	6	3	96	24	53
										
**Others**	TWIN PRIMING	0	0	0	142	17	24	0	0	0
**Others**	FRAGMENTED	0	0	0	14	1	1	6	1	1
**NMF**	NMF	8	4	2	203	44	19	165	106	81
										
**Total**		302	302	302	1421	1421	1421	4056	4056	4056

### 1. Full length L1HS elements

302 full length (> = 6 kb) L1HS elements were identified using the RISCI_RM option for repeat mining (See materials and methods). Among these, RISCI identified 100 insertions as genic (all intronic). Unless otherwise stated, the inferences refer to the transposon locus in the reference or main genome (Table 1, Additional file 1).

#### Inferences based on the orthologous locus in the reference chimpanzee genome

##### a. Shared ancestry

**Figure 2:**
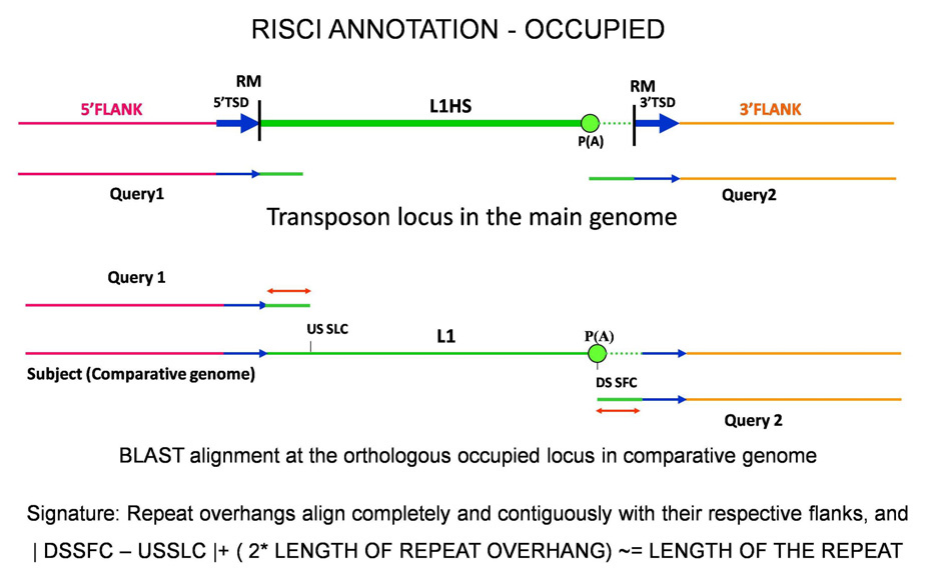
**Alignment signatures for shared ancesstory or OCCUPIED loci in the comparative genome**. Complete alignment of the repeat overhangs contiguously with their respective flanks and the separation between the flanks within 100 base range of the transposon length in the main genome. From left to right - Pink line - upstream flank, blue arrows - Target site duplication sequence, Green line - repeat sequence, dotted lines- poly (A) tails, orange line - downstream flank. DSSFC - downstream sequence subject first coordinate, Query1 - 5' or upstream flank with 50 bp repeat overhang, Query2 - 3' or downstream flank with 50 bp repeat overhang, RM - RepeatMasker start and end coordinates, USSLC - upstream sequence subject last coordinate.

Retrotransposons represent identity by descent markers and are largely homoplasy free [[47] and references therein, [48]]. Therefore, the orthologous locus is considered to have shared ancestry and is annotated as "OCCUPIED" if the repeat overhangs align completely and contiguously with their respective flanks in the comparative genome and the separation between the upstream and downstream flanks is approximately equal (± 100) to the size of the transposon in the reference genome (Figure 2). It is in context to add that the homoplasy free attribute of retrotransposon markers has been questioned occasionally [49,50].

Only 1 locus, L1HS_4_31 (see materials and methods for nomenclature of repeat locus), was found to be occupied in chimpanzee, L1HS being human-specific.

##### b. Post insertion changes

two major types of post insertion changes are possible viz. recombination and disruption.

**Figure 3:**
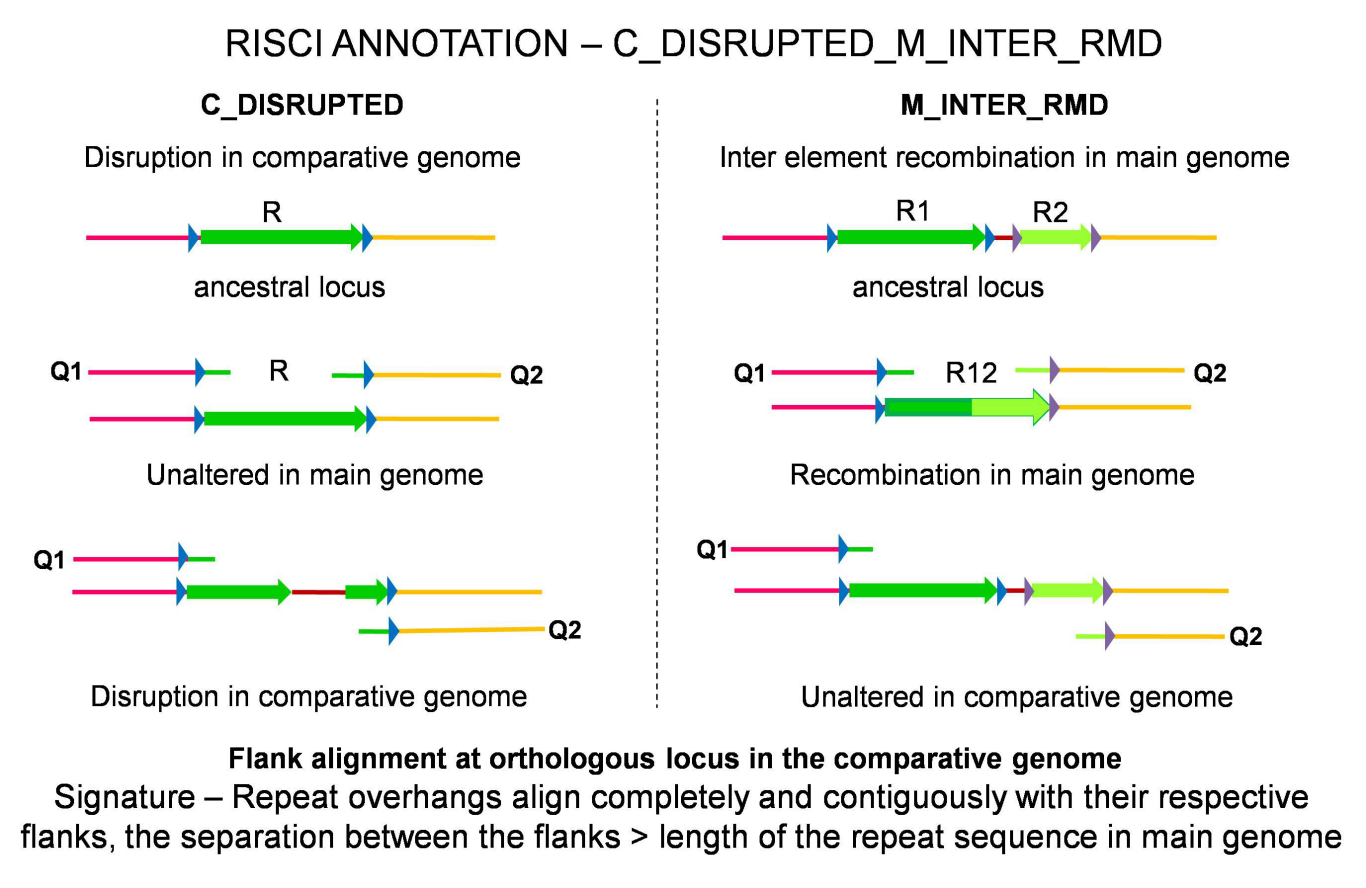
**Alignment signatures for M_INTER_RMD (Inter element recombination in main genome) or C_DISRUPTED (Disruption in comparative genome)**. In both cases, the repeat overhangs align completely and contiguously with their respective flanks and the separation between Q1 and Q2 is greater than the length of the transposon in the main genome. Left panel - Lone transposon in main genome (R1 - green arrow) disrupted by insertion of exogenous sequence (brown line) in the comparative genome. Q1 - upstream flank with 50 base repeat overhang. Q2 - downstream flank with 50 base repeat overhang. Right panel-From left to right - Pink line - 5' flank of R1, blue triangles - target site duplications of R1, green arrow - first repeat copy (R1), brown line - intervening sequence, grey triangles - target site duplications of R2, light green arrow - second repeat copy (R2 - same color indicating homology), orange line - 3' flank of R2. R12 - recombined repeat with a consequential loss of one copy of homologous region (R2) and the intervening sequence. Note that the 5' TSD of R12 comes from R1 (blue triangle) and the 3' TSD comes from R2 (grey triangle).

Homology-based recombination between two similarly oriented repeats on a chromosome results in loss of the intervening sequence and one copy of the homologous sequence. The recombination event may be exclusive to the main or reference genome - M_INTER_RMD (Main genome INTER element Recombination Mediated Deletion) or to the comparative genome, C_INTER_RMD (Comparative genome INTER element Recombination Mediated Deletion). In M_INTER_RMD, the repeat overhangs align completely and contiguously with their respective flanks in the comparative genome (assuming that the insertions are not specific to the reference genome), the separation between the flanks is greater than the size of the repeat in the reference genome and the transposon in the reference genome aligns completely (full length) with one of the two transposon copies in the comparative genome (Figure 3). A similar alignment is obtained in case the transposon locus is disrupted in the comparative genome (C_DISRUPTED). However, in this case, the transposon in the main or reference genome does not show full length alignment with any of the two repeats in the comparative genome (Figure 3). Based on the alignment signatures, the locus is annotated as C_DISRUPTED_M_INTER_RMD and resolved later by pair-wise blast between the transposon in the main genome and the orthologous locus in the comparative genome. L1HS_4_29c was annotated as C_DISRUPTED_M_INTER_RMD, and was shown to be a disruption due to Ns in chimpanzee.

Disruptions in main genome are resolved using specific alignment signatures by the RISCI defragmentation module (discussed later). On the other hand, if the repeat overhangs align completely and contiguously with their respective flanks in the comparative genome, but the separation between the flanks is less than the transposon locus in the main genome, the locus is annotated as C_INTRA_RMD (intra-element recombination mediated deletion in comparative genome). No C_INTRA_RMD event was identified in chimpanzee.

**Figure 4:**
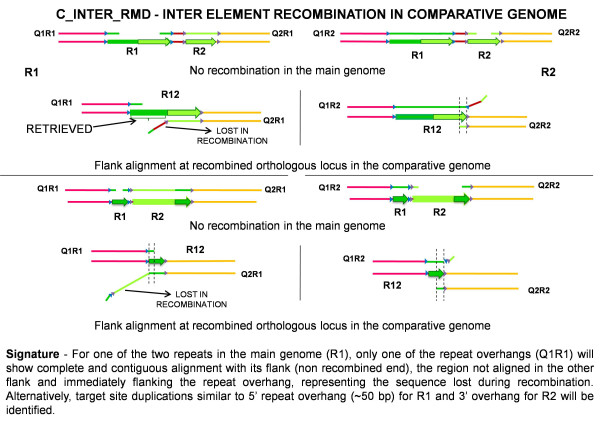
**Alignment signatures for C_INTER_RMD (Inter element recombination in the comparative genome)**. Two possible recombination scenarios and the possible alignments for R1 and R2, which recombine in the comparative genome to form R12 are shown. From left to right - Pink line - 5' flank of R1, blue triangles - target site duplications of R1, green arrow - first repeat copy (R1), brown line - intervening sequence, grey triangles - target site duplications of R2, light green arrow - second repeat copy (R2), orange line - 3' flank of R2. Region of homology between R1 and R2 shown in light green in top panel and dark green in bottom panel, R12 - recombined repeat with a consequential loss of one copy of the homologous region and the intervening sequence. Note that the 5' TSD of R12 comes from R1 (blue triangle) and the 3' TSD comes from R2 (grey triangle). Q1R1 - 5' flank of R1 with 50 bp repeat overhang, Q1R2-5' flank of R2 with 50 bp repeat overhang, Q2R1 - 3' flank of R1 with 50 bp repeat overhang, Q2R2 - 3' flank of R2 with 50 bp repeat overhang. As can be seen from the figure, the flank size should be sufficiently long to span the entire intervening region and other copy of the repeat to pick up such changes.

C_INTER_RMD presents more complex signatures. Given sufficient flank length (large enough to span beyond the two repeats in question in the reference genome), such events can also be identified by RISCI. For one repeat in the main genome (R1), only one of the repeat overhangs shows complete and contiguous alignment with the flank (non recombined end). The region immediately flanking the repeat overhang and not aligned in the other flank represents the sequence lost during recombination (Figure 4). For the other repeat (R2), an overlap between upstream and downstream query in the repeat overhang is seen. Alternatively, overlap between upstream and downstream query in the 5' repeat overhang for one repeat, and 3' overhang for the other repeat may also be identified (Figure 4). A disruption specific to the reference genome, the orthologous locus in the comparative genome being occupied and intact also gives a similar signature (Additional file 17, Figure S1). Therefore, RISCI classifies such loci as C_INTER_RMD_M_DISRUPTED.

Contrary to expectations of no C_INTER_RMD events in chimpanzee, 4 such recombination events (L1HS_ 2_14, 3_13, 5_3 and 12_10) were reported with high RISCI scores (refer methods) and low N-scores (%Ns in a sequence). For each of these loci, 5' truncated L1 element was found in close proximity downstream of the transposon locus in the human genome. All retrieved orthologous loci in chimpanzee aligned with the L1HS sequence in the human genome except L1HS_5_3. This sequence was, however, annotated as L1MA9 by RepeatMasker suggesting homology with L1HS sequence. 1586 bases of intervening sequence in L1HS_3_13 were lost in recombination. In the other three cases the recombining repeats were located next to each other.

The fact that an orthologous locus each in chimpanzee was found to be occupied and disrupted and 4 orthologous loci showed recombination suggests that though largely human specific, as evidenced by the large number of empty alleles in chimpanzee, L1HS predate human chimpanzee divergence, as has been reported earlier [51]

##### c. Inferences based on empty allele at the orthologous locus

Target site duplication (TSD) upon transposon insertion is almost universal [1]. Exceptions include DIRS (Dictyostelium Interspersed Repeats) among retrotransposons [52] and Crypton [53] and Helitron [54] super families of DNA transposons. Loci not found to be occupied or altered post insertion in the comparative genome(s) are then screened for the empty locus using a novel TSD finding strategy.

**Figure 5:**
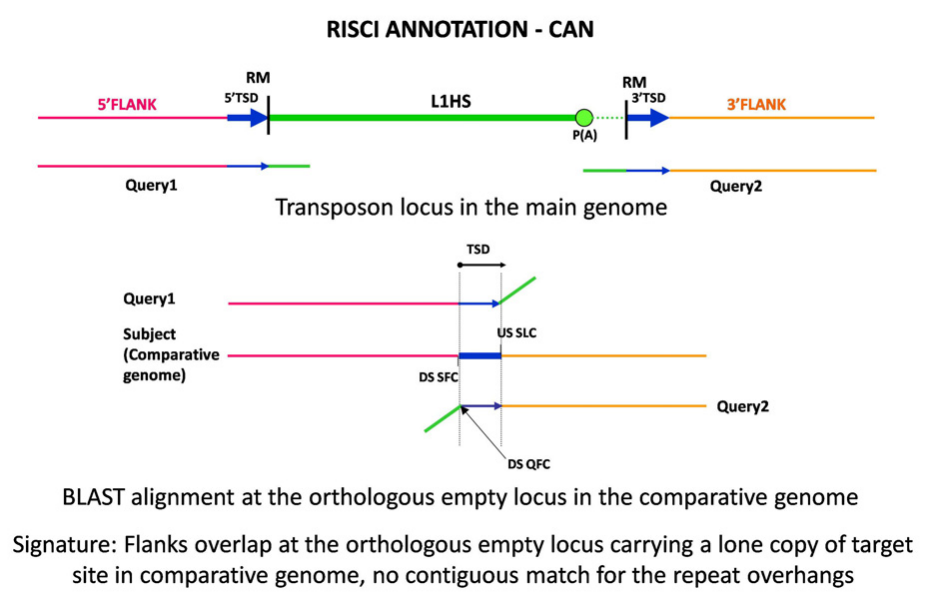
**Alignment signature for CAN (canonical transposition)**. Query1 and Query2 show an overlap in the region of target site duplication at the orthologous empty locus, while the repeat overhangs do not align (represented by oblique green lines) and DSQFC is < 71 or A and AT score > 0.65 or 0.90 respectively. From left to right - Pink line - upstream flank, blue arrows and line - target site duplication sequence, green line - repeat sequence, dotted lines- poly (A) tails, orange line - downstream flank. DSQFC - downstream sequence query first coordinate, DSSFC - downstream sequence subject first coordinate, Query1 - 5' or upstream flank with 50 bp repeat overhang, Query2 - 3' or downstream flank with 50 bp repeat overhang, RM - RepeatMasker start and end coordinates, USSLC - upstream sequence subject last coordinate.

**Figure 6:**
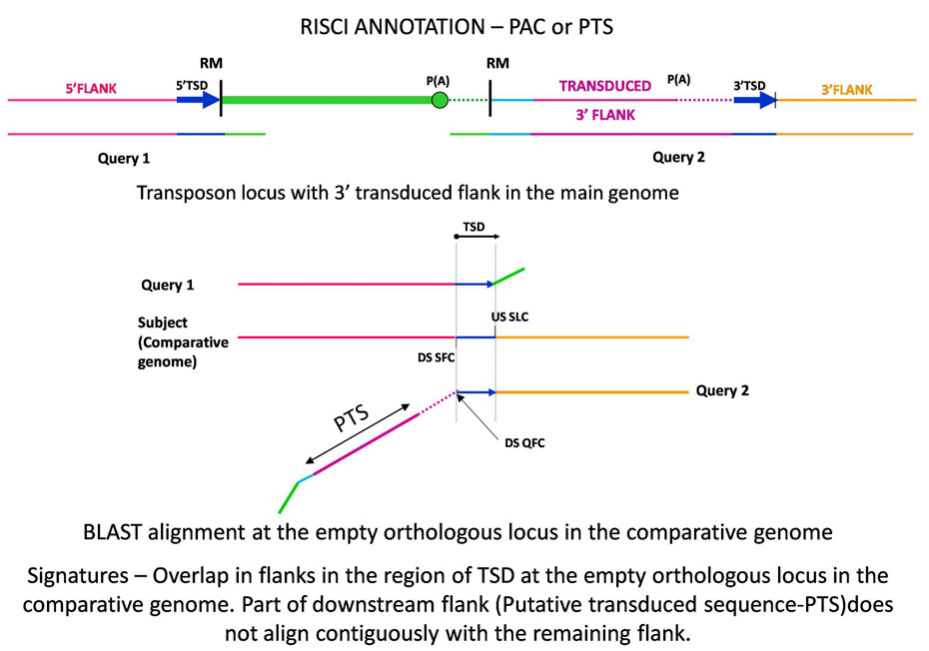
**Alignment signatures for PAC and PTS**. Query 1 aligns at the orthologous empty locus starting from the TSD upwards. Likwise, Query 2 aligns from the TSD, downwards. The region of Query 2 preceding the TSD (represented by oblique line), which does not find a contiguous match, consists of the 3' repeat overhang and the misannotated poly A tail or the transduced flank depending on the A and AT scores. From left to right - Pink line - upstream flank, blue arrows and line-target site duplication sequence, green line - repeat sequence, dotted lines- poly (A) tails, dark pink line - transduced flank, orange line - downstream flank. DSQFC- downstream sequence query first coordinate, DSSFC - downstream sequence subject first coordinate, PTS - putative transduced sequence, Query1 - 5' or upstream flank with 50 bp repeat overhang, Query2 - 3' or downstream flank with 50 bp repeat overhang, RM - RepeatMasker start and end coordinates, USSLC - upstream sequence subject last coordinate.

The rationale behind this strategy is that since both the upstream and downstream flanks of the transposon carry the target site duplication sequence, of which only one copy is present at the orthologous empty locus in the comparative genome, when the upstream and downstream flanks are separately blasted against the comparative genome, the flanks would show an overlap in the comparative genome in the region of the TSD (Figures 5 and 6). The TSD sequence is thus used as a clamp to accurately identify the empty orthologous locus in the comparative genome(s). A TSD size of zero is allowed to accommodate endonuclease independent L1 insertions [55] and transposons which do not duplicate target site. RISCI further classifies the transposition event in the reference genome as canonical (excusive mobilization of the transposon sequence) or non canonical (transposition with flank transduction), based on the position of the TSD in the downstream flank. TSDs were identified for 270 loci in chimpanzee.

Canonical transposition

The 3' end of non LTR retrotransposons are generally under or overestimated by RepeatMasker since they end in highly variable poly-A tails. To accommodate this anomaly, even if the TSD is found 20 bases downstream of the RepeatMasker annotated 3' end, the retrotransposition event is annotated as CAN (Canonical). 170 loci in the reference human genome were annotated as CAN (Figure 5).

Additionally, the RNA transcription machinery occasionally skips the retrotransposon's weak polyadenylation signal resulting in a readthrough transcript. This transcript when subsequently integrated at another locus effectively duplicates the original 3' flank to the extent of the readthrough [56-58]. This mechanism may lead to exon shuffling [58,59] and gene duplication [60]. Therefore, in non-LTR retrotransposons where the TSD is found beyond 20 base pairs of the RepeatMasker annotated 3' end, the unmatched region beyond the repeat overhang till the beginning of the TSD may either represent a grossly misannotated poly-A tail or a true 3' transduced flank (Figure 6).

If the A-score (∑A/length of unmatched downstream sequence) > 0.65 or AT-score (∑(A+T)/length of unmatched downstream sequence) is > 0.90, the transposition is annotated as PAC (Poly A Canonical-canonical transposition with a grossly misannotated poly A tail). The score thresholds were fixed on the basis of empirical observations and may be reset by the user. 68 Loci were annotated as PAC. It is important to restate here that both CAN and PAC represent canonical insertions (exclusive mobilization of transposons sequence). RISCI thus precisely defines transposition boundaries in the reference genome if the orthologous locus is empty in the comparative genome, providing an improvement over RepeatMasker annotations (Additional file 17 Figures S2 and S3). The remaining 32 loci, for which TSDs were identified, qualify as putative 3' flank transduction events and are annotated as PTS (loci with Putative Transduced Sequence, Figure 6).

3' flank transduction

**Figure 7:**
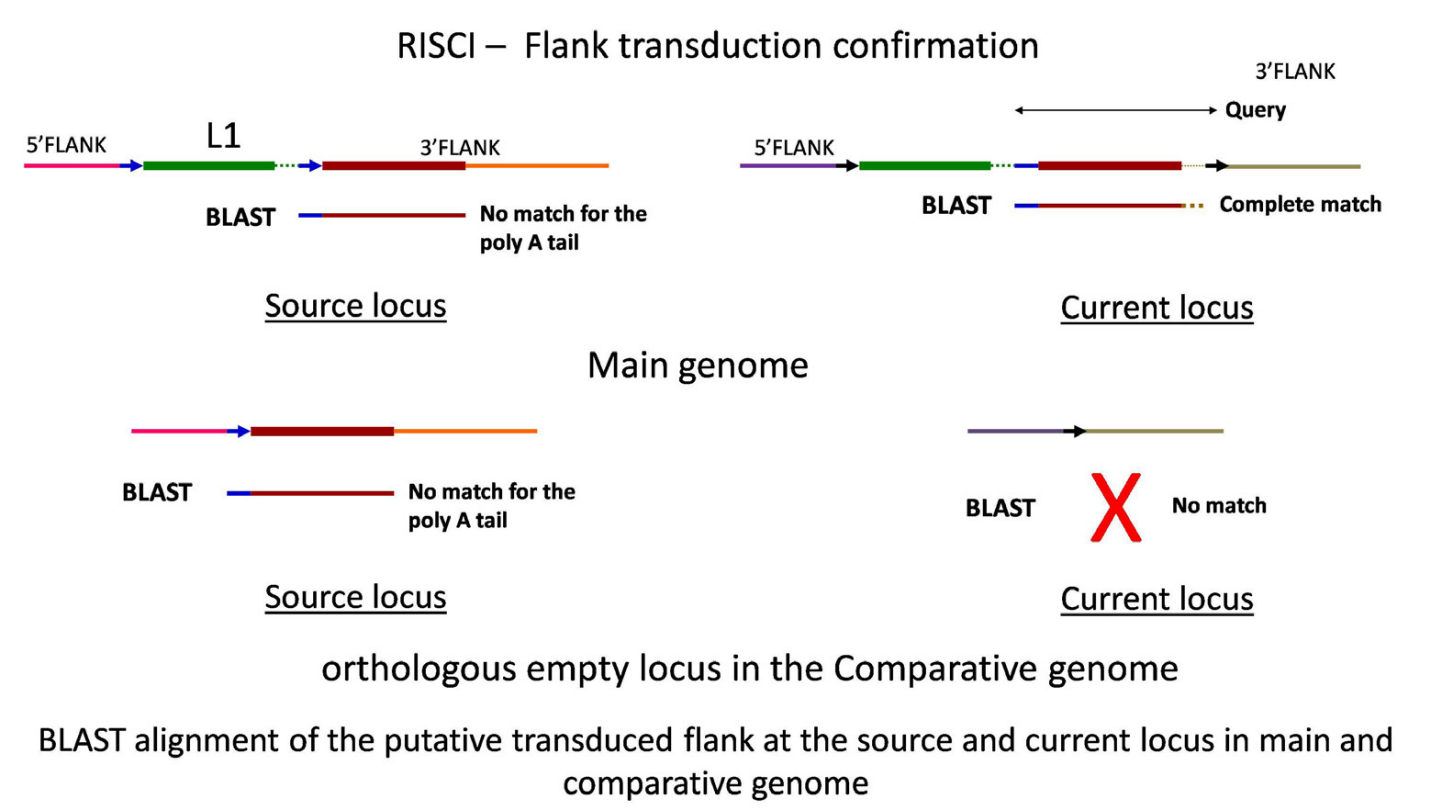
**Signatures of true 3' flank transduction**. The source locus in the main genome consists of an L1 element with TSDs (blue arrows) which moves to the target or current locus along with a part of the 3' flank (brown bold line) forming new TSDs (black arrows). In contrast, no flank transduction takes place in the current locus in the comparative genome. As indicated, the query consists of one copy of the original TSD (blue line), the transduced flank (brown bold line) and the second poly A tail (dotted brown line). When blasted on the main genome, at least two hits are obtained - one complete match at the current locus and one almost complete match (barring the poly A tail) at the source locus. In the comparative genome, no match is found at the orthologous current locus (since no transposition event has taken place for lack of L1 element at the source locus as shown here, or otherwise). The match at the source locus in the comparative genome is similar to the match at the source locus in the main genome and on corresponding chromosomal homologue. RISCI has an inbuilt module for 3' flank transduction confirmation which enlists the putative transduced sequence, the number of blast hits obtained in the main and comparative genomes and the most probable source locus in the two genomes in case multiple hits are obtained. From left to right - Pink line - 5' flank at the source locus, blue arrows - TSDs at the source locus, green line - repeat, dotted line - poly A tail, brown and orange lines - 3' flank at the source locus, purple line - 5' flank at the current locus, black arrows - TSDs at the current locus, grey line - 3' flank at the current locus.

RISCI has inbuilt confirmation module for 3' flank transductions. A putative transduced flank is confirmed as a true transduction event when it has at least two non-redundant Blast high-scoring segment pairs (HSPs) in the reference genome - one from where the sequence is picked - target or current locus (complete match), and the other from where it has moved to the target locus - source locus (partial - no match for the polyA tail), and/or one hit (partial) in the comparative genome on the chromosomal homolog corresponding to the source locus in the reference or main genome (Figure 7).

Of the 32 loci predicted as PTS, the source locus was unambiguously identified for 23 both in the main genome and the comparative genome. For another 3 (L1HS_5_18c, 9_8 and 18_10), the source locus in human was clear and the only hit in chimpanzee was partial but on the chromosome corresponding to the identified source locus in the main genome. The source locus for L1HS_7_14 in chimpanzee is ambiguous. No matches in chimpanzee were found for L1HS_1_24c. The A-score or AT-score of L1HS_4_22, L1HS_18_7 and L1HS_X_9c were very close to the threshold and actually represent misannotated poly-A tails. L1HS_8_6c is falsely reported as PTS. The length of the confirmed transduced flanks ranged from 50 bp to 1600 bp. (Additional file 2).

5' flank transductions

5' flank transductions occur when a strong upstream promoter drives transcription into the L1 sequence. In such cases the 5' TSD is found slightly upstream of the actual L1 5' end. Template switching [61-63] may also result in formation of 5' TSD upstream of the transposon 5' end. Of the 12 reported 5' flank transductions by RISCI, 4 (L1HS_ 7_11, 11_10c, 15_1c and X_19c) were found to satisfy flank transduction criteria (mentioned earlier) and represent confirmed 5' flank transductions (Additional file 3). In the remaining cases, the putative transduced flank was a repeat sequence with multiple hits and may have come to occupy the current locus either as a consequence of 5' flank transduction or insertion into the 5' end of L1. The possibility of template switching is minimal since L1 reverse transcriptase is known to have low processivity.

##### c. Insertion-mediated deletion or parallel independent insertions or insertion-deletions

**Figure 8:**
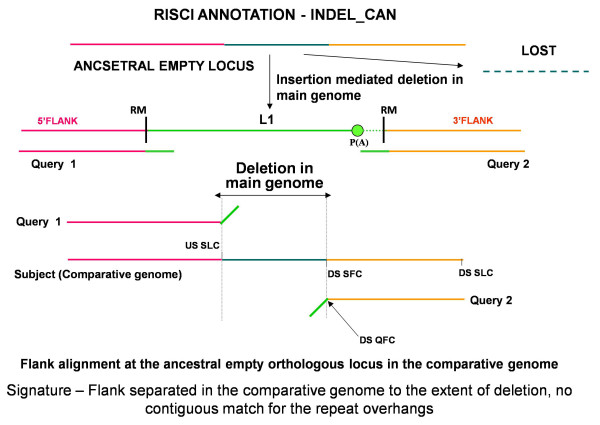
**Alignment signatures for Insertion-mediated deletion with exclusive mobilization of the transposon**. Insertion-mediated deletion results in replacement of original sequence by the transposon without the duplication of target site. On alignment at the orthologous locus in the comparative genome, no match is found for the repeat overhangs (represented by oblique green lines) and the upstream and downstream flanks are separated by a sequence stretch representing the original sequence lost upon insertion of the repeat in the main genome. Ancestral locus - from left to right - Pink line - 5' flank, turquoise line - sequence lost on L1 insertion, orange line - 3' flank. DS QFC - downstream sequence query first coordinate, DS SFC - downstream sequence subject first coordinate, Query1 - 5' or upstream flank with 50 bp repeat overhang, Query2 - 3' or downstream flank with 50 bp repeat overhang, RM - RepeatMasker start and end coordinates, USSLC - upstream sequence subject last coordinate.

**Figure 9:**
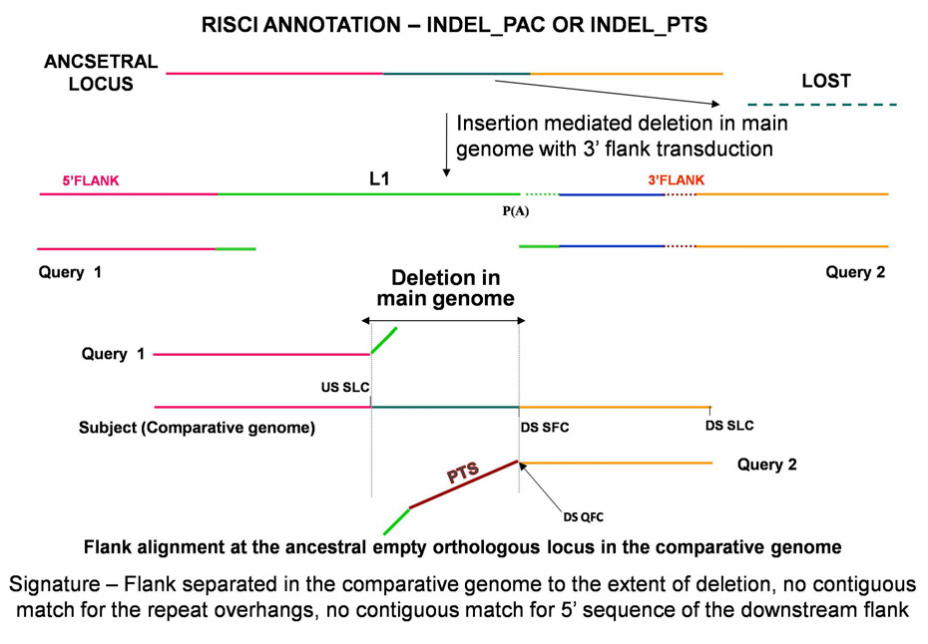
**Alignment signatures for insertion-mediated deletion concurrent with 3' flank transduction**. Ancestral locus - from left to right - Pink line - 5' flank, turquoise line - sequence lost on L1 insertion, orange line - 3' flank. Green line - repeat sequence, brown line - transduced sequence, dotted lines - poly A tails. DS QFC - downstream sequence query first coordinate, DS SFC - downstream sequence subject first coordinate, Query1 - 5' or upstream flank with 50 bp repeat overhang, Query2 - 3' or downstream flank with 50 bp repeat overhang, RM - RepeatMasker start and end coordinates, USSLC - upstream sequence subject last coordinate. Insertion mediated deletion results in replacement of earlier sequence by the repeat with transduced flank (brown line) without the duplication of target site. On alignment at the orthologous locus in the comparative genome, no match is found for the repeat overhangs (represented by oblique green lines) and the transduced sequence, and the upstream and downstream flanks are separated by a sequence stretch representing the sequence lost upon insertion of the repeat in the main genome. Depending on the A and AT scores of the unmatched portion of the downstream flank, RISCI annotates the locus as INDEL_PAC or INDEL_PTS.

Retrotransposons like L1s and Alus have been reported to occasionally cause deletions at the target site in cell culture assays as well as by comparative genomics approaches [64-66]. Additionally, though rare, parallel independent insertion at the same locus in the comparative genome is also possible [67,68]. The orthologous locus may also undergo independent changes (insertion, deletions or gene conversions). In all cases the upstream and downstream flanks in the comparative genome are separated from each other by the extent of deletion or parallel insertion or other changes and the repeat overhangs do not align contiguously with their respective flanks (Figures 8 and 9) as opposed to recombination.

**Figure 10:**
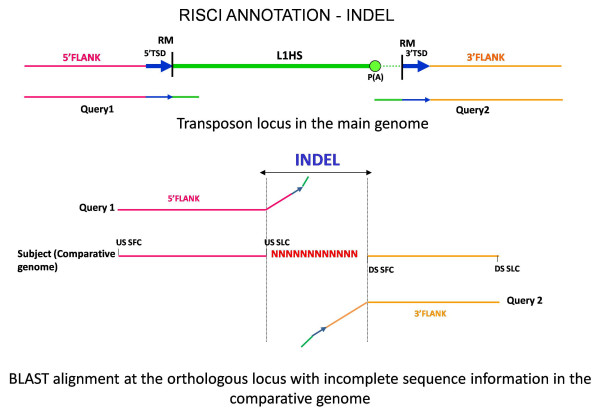
**Misannotation because of sequencing gaps**. Since a certain amount of sequence information at the orthologous locus is missing and substituted by approximate number of Ns, only partial matches for the upstream and downstream sequences are obtained resulting in false annotation by RISCI. From left to right - Pink line - upstream flank, blue arrows - Target site duplication sequence, Green line - repeat sequence, dotted lines- poly (A) tails, orange line - downstream flank. DSSFC - downstream sequence subject first coordinate, Query1 - 5' or upstream flank with 50 bp repeat overhang, Query2 - 3' or downstream flank with 50 bp repeat overhang, RM - RepeatMasker start and end coordinates, USSLC - upstream sequence subject last coordinate.

As in normal transposition, insertion-mediated deletions may result from a normal (CAN) or 3' misannotated (PAC) or readthrough transcript (PTS). Hence INDELs are sub annotated as INDEL_CAN (Figure 8), INDEL_PAC and INDEL_PTS (Figure 9), depending on how far from the annotated 3' end of the repeat does the match for the downstream flank starts. Most INDEL predictions by RISCI are a consequence of substitution of actual sequence by an estimated number of Ns (Figure 10). If the N-score is less than 10 and the locus annotated as "INDEL_PTS", the PTS is also retrieved and confirmed as in normal 3' flank transduction.

It is important to mention that though annotated only after exclusion of all other possibilities and two rounds of check, INDEL annotations per se have relatively relaxed criteria of the flanks being separated by a maximum of 10000 bases and at least a 1000 base query coverage in case of INDEL_PTS. Given the high repeat content of the flanks, random matches may not be ruled out. User discretion is, therefore, advised while dealing with INDELs and INDEL_PTS in particular.

18 INDELS were reported. Of these, 9 had N-scores approximately greater than 10 (ranging from ~ 9.22 to 100) or N-stretch at the 3' end (L1HS_9_1c) of sequence, resulting in misannotation. TSDs were not found in the reference genome (checked by blast2 between 500 bp of upstream flank and 2500 bp of downstream flank) for L1HS_10_9c, 12_8c, 18_9c, 20_2 and 22_2c leaving only two possibilities. The indel sequences either represent the sequences deleted during L1 insertion in human or the intervening sequence between two L1s which recombine to form the present L1 in the main genome. In comparison with Celera and HuRef genomes, L1HS_18_9c was definitively identified as M_INTER_RMD (recombined L1 in the main genome). The fact that the intervening sequence in Celera and HuRef genomes showed high similarity with the INDEL sequence in the chimpanzee genome unambiguously suggests that this sequence is ancestral to human specific L1 insertions and the subsequent recombination. The other four loci were either non differential (OCCUPIED) in Celera and HuRef genomes or had high N-scores and hence cannot be definitively classified as insertion-mediated deletions.

TSDs were identified in the reference genome (checked by blast2 as above) for L1HS4_3c, 4_19c, 7_7 and 10_1 immediately before and after the transposon. Intriguingly though, both L1HS_4_19c and 7_7 were annotated as INDEL_PTS by RISCI and the flank transductions were confirmed (Table 2). This might just be coincidental. However, the fact that the only blast hit in chimpanzee corresponds to the source locus chromosome in the human genome and that the sequence carries a poly A-stretch for which no match is found at the source locus in both human and chimpanzee genomes unambiguously links the transposition of this sequence with the preceding L1HS. This is suggestive of an insertion-mediated deletion mechanism with duplication of the target site in the main genome. It is important to note here that both L1HS4_19c and L1HS7_7 are insertions into intronic region of genes *HSD17B11 *(alias *DHRS8*) and *AUTS2 *respectively.

**Table 2:** Target and source locus for the 3' transduced flank in the main (human) and comparative genomes (chimpanzee) for loci annotated as INDEL_PTS

GENOME	LOCUS	L1HS	CHR	CONTIG	ORIENT	QFC	QLC	SFC	SLC	Source-Genic/Intergenic (based on CDS)
human	Target	L1HS_4_19c	4	NC_000004	Minus	1	91	88487299	88487209	
human	Source	L1HS_4_19c	4	NC_000004	Plus	16	91	88496516	88496591	*HSD17B11*, INTRON 5
Chimp	Source	L1HS_4_19c	4	NC_006471	Plus	16	91	90265309	90265384	*DHRS8*, INTRONS 4,5
										
human	Target	L1HS_7_7	7	NC_000007	Plus	1	619	69306280	69306898	
human	Source	L1HS_7_7	5	NC_000005	Minus	3	606	140466972	140466369	Intergenic
Chimp	Source	L1HS_7_7	5	NC_006472	Minus	3	606	142876752	142876149	Intergenic

RISCI confirmation of 3' flank transduction concurrent with insertion mediated deletion (INDEL_PTS) by unambiguous identification of the source locus in main and comparative genomes. As expected, no hit is found for the target locus in the comparative genome, and one to one chromosomal correspondence for the source locus in main and comparative genome is noticed. Also the source locus hit both in the main genome and in the comparative genome is shorter than the target hit since no match is found for the poly A tail at the source locus. CHR - chromosome, ORIENT - orientation, QFC - Query first coordinate, QLC - Query last coordinate, SFC - Subject first coordinate, SLC - Subject last coordinate. Source loci found within genes (and within exons or introns if CDS coordinates are available are also reported). *HSD17B11 *and *DHRS8 *are aliases of eachother.

#### Inferences based on comparisons with Celera and HuRef genomes

In contrast to the chimpanzee genome, 217 loci in the Celera and 171 in the HuRef genome were annotated as OCCUPIED. Among these, 149 loci were commonly occupied in all 3 human genomes representing the more ancestral or fixed loci. 57 Of these were insertions into genes. Though not informative for phylogenetic studies, some of these may have evolutionary significance. TSDs were identified for 39 elements in Celera and 38 in HuRef assembly comparisons (Table 1). These represent recent and, therefore, polymorphic insertions in the human genome, amenable to phylogenetic studies. Of these, 27 in Celera and 25 in HuRef were canonical insertions in the reference human genome, 9 in Celera and 10 in HuRef had misannotated poly A tails (PAC) and 3 each were annotated as PTS (3' flank transduction). All the 3 PTS in Celera and 2 in HuRef were confirmed by RISCI. As mentioned in comparison with chimpanzee (Additional file 2), X_9c in HuRef has A-score (0.61) close to the threshold (0.65). 5' flank transduction was predicted for L1HS _1_5c, 4_35 and 15_1c both in Celera and HuRef, and the source locus was unambiguously identified for L1HS_15_1c both in Celera and HuRef (Additional file 3). Multiple hits were obtained for the other two, both in reference and comparative genomes.

7 C_DISRUPTED_M_INTER_RMD were reported in comparison with the Celera genome, of which L1HS_18_9c is M_INTER_RMD, with full length L1s at the 5' and 3' end at the orthologous locus in both Celera and HuRef resulting in loss of 13.8 kb of sequence (6 kb L1HS and 7.8 kb of intervening sequence). Additional L1 sequence was found at the 5' end of L1HS_1_6 (N-score - 0.3) and 3' end of L1HS_11_6 (N-score -0). These may be true insertions into pre-existing repeats. Others had very high N-scores. Of the 12 C_INTER_RMD reported, only 5 had N-score < 10, 3 of which had Ns either at the 5' or 3' end of the sequence. For the remaining 2 (L1HS_5_15 and 16_2C), Ns were strategically located at the 3' (L1HS_5_15) or 5' (L1HS_16_2c) end of partial L1 sequence, followed by partial duplication of the upstream (L1HS_5_15) or downstream (L1HS_16_2c) sequence in the ortholog, clearly suggesting errors in assembly. 15 C_INTRA_RMD were reported in Celera genome. 4 had N-score less than 10, and two of these (L1HS_2_16 and L1HS_6_2) were less than 5000 bases (full length L1 is 6 kb) and may represent true intra element recombination. 8 INDELS are reported in comparison with Celera genome. Only 1 had low N-score (0) and represents an occupied locus misannotated as INDEL because of partial match for the 3' repeat overhang.

43 C_DISRUPTED_M_INTER_RMD were reported by RISCI in the HuRef assembly. L1HS_18_9c (N-score 1.1), as mentioned earlier, is a recombined L1 in the human genome with clear full length L1s at either end. All others, except L1HS_11_6, appear to be a consequence of assembly errors. Even when the N-scores were lower than 0.5 (L1HS_ 1_3, 1_18c, 1_25c, 4_27, 5_18c, 5_23c, 6_7, 7_1, 13_7c, 16_2c, 16_4c and 17_1), no non L1 sequence was reported by RepeatMasker and there was a distinct overlap in the L1 sequence before and after the N-stretch pointing to problems in assembly. L1HS_11_6 appears to have been disrupted by insertion of a truncated L1 sequence in the opposite orientation.

11 C_INTER_RMD are reported in HuRef. 8 Of these had N-scores > 10 or N-stretch at the 5' or 3' end of the retrieved sequence. As in the Celera assembly, the N-stretch is placed next to the partial L1HS sequence, followed by duplication of the upstream sequence in L1HS_4_4, 5_15 and 10_1, indicating errors in assembly. 24 C_INTRA_RMD were reported in HuRef. Only three (L1HS_3_13, 7_9 and 11_1) of these were less than 5000 bases, had low N-scores and may possibly be true intra element recombinations.

13 INDELs were reported in the HuRef assembly. Of these, 9 either had N-score >10 or had N-stretch at the 5' (L1HS_ 8_6c) or 3' end (L1HS_ 8_5 and 12_9) of the indel sequence. L1HS_1_2c, 1_11 and 13_8c represent occupied loci but are classified as INDEL because of partial or no match for the 3' repeat overhang, possibly because of the decay of the poly-A tail or the 3' target site duplication. L1HS_11_11 presents an interesting case. In the HuRef genome, it is annotated as 9 bp (N-score 0.0) INDEL with almost full query coverage for upstream and downstream flanks. However, in the chimpanzee genome the orthologous locus is annotated as CAN with a TSD of 18 bp, which suggests that L1 insertion-mediated deletion of the ancestral locus did not take place and that the orthologous empty locus in the HuRef genome has undergone independent changes.

### 2. Analysis of truncated repeats

Retrotransposons get truncated in several ways e.g. 5' truncation because of low processivity of reverse transcriptase and competition by RNAse H in LINES, twin priming [69] resulting in loss of intermediate sequence and inversion of the 5' end, looping of m RNA resulting in loss of intermediate sequence without inversion of the 5' end [65] etc. Besides, false truncations may also result from disruption of the full length insertions. True truncations and disruptions pose stiff challenges to repeat detection and annotation programs. The two parts of a disrupted transposon may frequently get annotated as different repeats and small truncated repeats may escape detection or be misannotated [70]. RISCI has special modules for analysis of such repeats.

#### a. Defragmentation module

**Figure 11:**
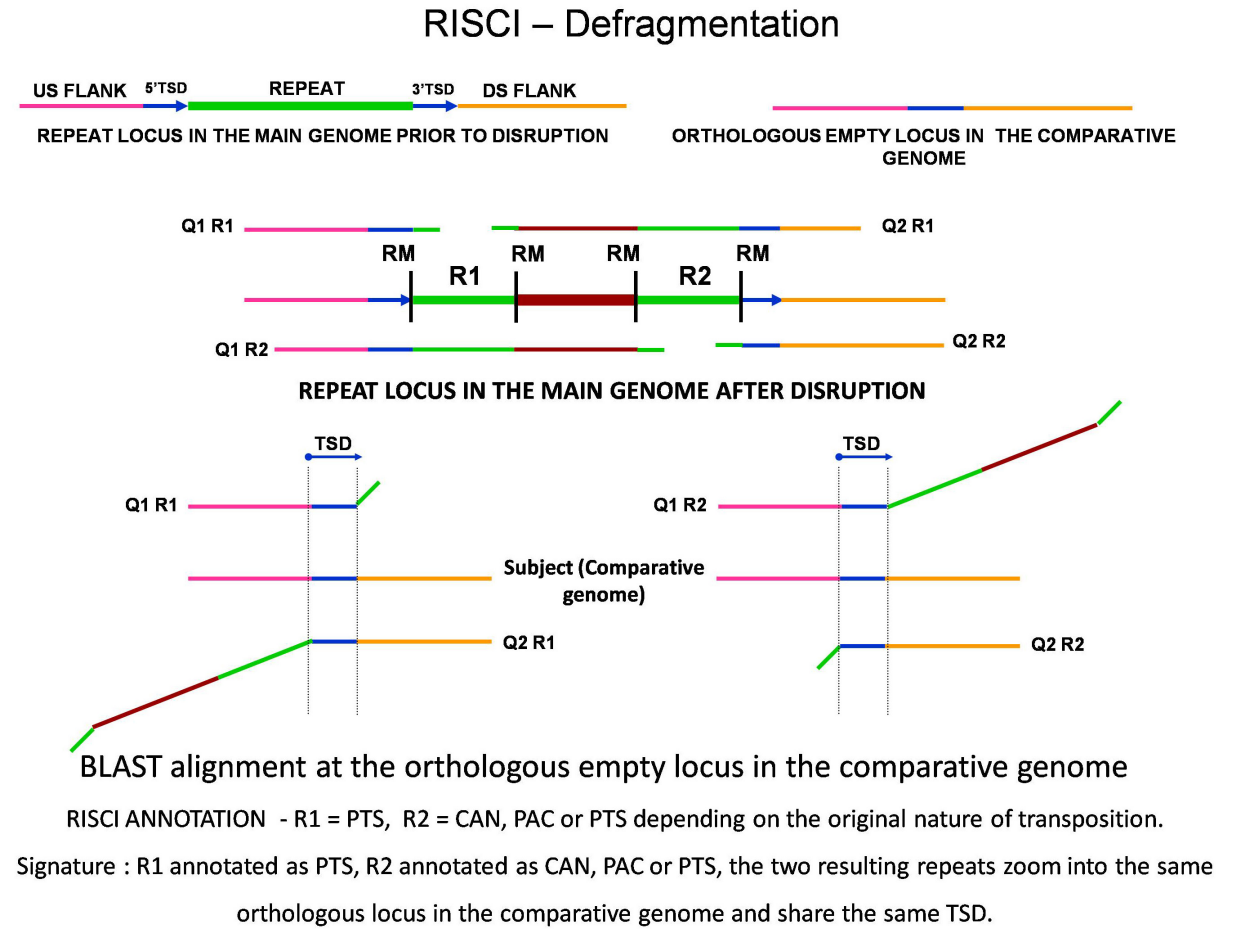
**Alignment signatures for defragmentation by RISCI**. The original repeat is disrupted into R1 and R2 due to insertion of an exogenous sequence (brown bold line). Blastn alignments for R1 and R2 at the orthologous empty locus in the comparative genome are shown. Query 2 of R1 (Query2 R1), starting from R1 and downstream, consists of the 50 base overhang into the 3' end of R1, the exogenous sequence (brown line), R2, 3' target site duplication and its downstream flank, of which, only the TSD and its downstream flank find a match at the orthologous empty locus (parallel lines) resulting in PTS annotation for R1. For R2, starting from R2 upstream, Query 1 (Query1 R2) consists of 50 bp overhang into 5' end of R2, the exogenous insertion, R1, 5' TSD and its upstream flank, of which only the TSD and its upstream flank find a match at the orthologous empty locus (parallel lines). RISCI annotation of R2 depends on the nature of the original transposition event and RepeatMasker estimation of the 3' end of repeat (CAN, PAC or PTS). The flank length is crucial in indentifying such events and must be long enough to span the exogenous sequence, R1 or R2 and their respective flanks. From left to right - pink line - 5' flank of the original repeat before disruption, blue arrows and lines - TSDs, R1 - first fragment, brown bold line - exogenous insertion, R2 - second fragment, orange line - 3' flank of original repeat. Query1 R1 - upstream flank of R1 with 50 base overhang into R1, Query2 R1, downstream flank of R1 with 50 base overhang into R1, Query1 R2 - upstream flank of R2 with 50 base overhang into R2, Query2 R2 - downstream flank of R2 with 50 base overhang into R2, TSD - target site duplication sequence.

Defragmentation refers to the identification of the constituent parts of a disrupted or partially deleted repeat in the genome. All disrupted or partially deleted parts of a parent repeat would be in the same orientation, annotated as independent repeats by RepeatMasker, and the target site duplication would be located at the first (5' end) and the last fragment (3' end) of the disrupted repeat. If the orthologous locus in the comparative genome is empty, the upstream and downstream flanks for each fragment would show an overlap in the region of the single copy of the TSD in the comparative genome (Figure 11). In case of a parent repeat fragmented into two, the first half would be annotated as PTS (false annotation) and the second half as CAN, PAC or PTS (depending on mobilization of exclusive repeat sequence or also of the 3' flank) by RISCI and the two would share the same TSD (Figure 11). In the final results file, names of all fragments of a disrupted repeat are concatenated and marked by "!" suffix. As can be seen, the flank length is crucial to read these signatures and only small disruptions can be identified in this manner. To identify large disruptions, blast HSPs of the upstream flank of a repeat locus, for which no annotation is assigned by RISCI, are compared with the blast HSPs of the downstream flanks of all repeat loci in the same orientation downstream of this locus to check for the TSD in the comparative genome. RISCI identified 14 repeat disruptions in the reference genome (Additional file 4) in the analysis of truncated L1HSs (< 6000 bases-reference human genome Vs chimpanzee genome).

#### b. Identifying inversions using RISCI

**Figure 12:**
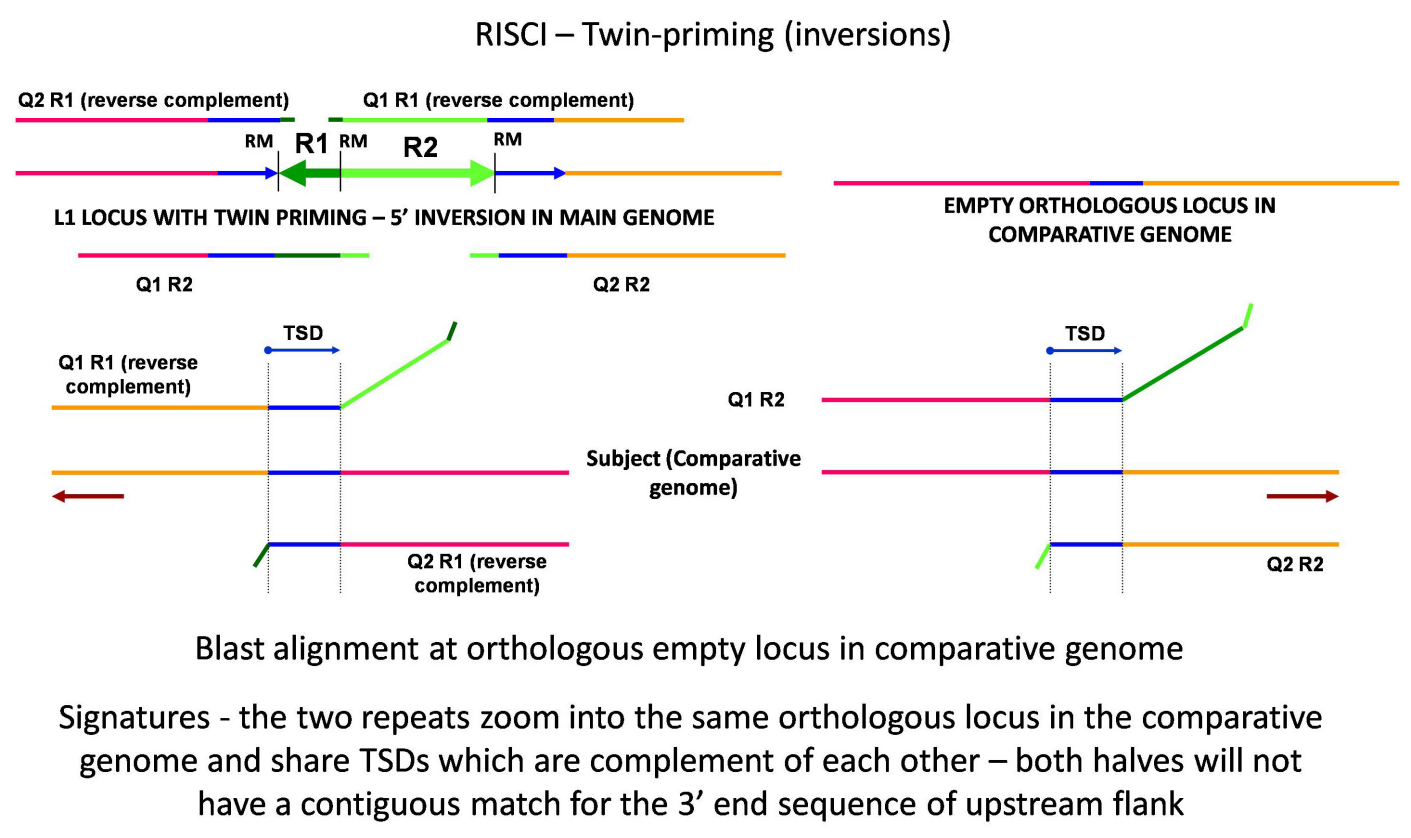
**Alignment signatures for twin priming**. Twin priming results in inversion of the 5' end of L1 (R1) as opposed to its 3' end (R2), the arrowheads indicating opposite orientations. Since Query1 R1 and Query2 R1 are in opposite orientation to Query1 R2 and Query2 R2, the alignment of query sequences at the orthologous empty locus in the comparative genome is in opposite orientations (indicated by opposite facing brown arrows). Also for Query1 R1, no match is found for 50 base overhang into R1, and the R2 sequence. Similarly, for Query1 R2, no match is found for 50 base overhang into R2, and the R1 sequence. Since the alignments are in opposite orientations, the TSDs identified are reverse complements of each other. From left to right - Pink line - 5' flank of the repeat, blue arrows and lines - target site duplication sequence, R1 - inverted 5' sequence, R2 - normal 3' sequence, orange line - 3' flank of the repeat. Query1 R1 - upstream flank of R1 with 50 base overhang into R1, Query2 R1, downstream flank of R1 with 50 base overhang into R1, Query1 R2 - upstream flank of R2 with 50 base overhang into R2, Query2 R2 - downstream flank of R2 with 50 base overhang into R2. In the final result file, inversions are indicated by '*' suffixed to the repeat name.

Owing to twin priming [69], LINE insertion may result in inversion of the 5' end sequence and truncated insertions. In such cases, the 5' end is in opposite orientation to the 3' end and each is annotated as a separate repeat by RepeatMasker. The two repeats share the same TSD (in opposite orientations) at the orthologous empty locus in the comparative genome and show an alignment similar to 5' flank transduction (Figure 12). In the final result file names of the elements of a twin priming event are concatenated and suffixed by "*". 142, 17 and 24 twin priming events were identified in the reference human genome when compared to chimpanzee, Celera and HuRef genomes, respectively. As expected, no twin priming was reported in AluYa5 comparisons since probability of a twin priming event is directly proportional to the length of the template.

It may be noted that since both disruptions and twin priming events are identified in a secondary screening based on the primary annotations by RISCI, misannotations are possible if one of the two constituents of a disruption or twin priming event is not annotated to the same repeat class by RepeatMasker.

### 2.1 Truncated L1HS analysis

A total of 1421 truncated L1HS elements (< 6 kb) were mined by RISCI in the reference human genome by using the RISCI_RM option (direct parsing of repeat coordinates from pre-masked files). However, 1421 does not represent the true number of truncated L1HS elements in the human genome. Twin primed L1HS elements are counted as two despite being the constituent parts of a single parent. Likewise, disrupted L1HS elements are also counted twice. On the other hand, some of the truncated L1HS elements may escape detection or may be misannotated as L1HS. Unless otherwise stated, the inferences refer to the transposon locus in the reference or main (reference human) genome (Table 1, Additional files 4, 5 and 6).

#### Inferences based on the orthologous locus in the reference chimpanzee genome

##### a) Shared ancestry

274 loci were found to be occupied at the orthologous loci in chimpanzee. This partly reflects the problem of truncated repeat misannotation, as also the fact that L1 insertions may not be truly human-specific. Most repeat annotation programs rely on homology to consensus sequences and characteristic nucleotides substitutions to classify a given repeat into a particular class and subclass. However, in the case of truncated repeats the quality of annotation is compromised for lack of sequence information, frequently leading to misannotation. This becomes strikingly evident in the case of twin priming and repeat disruption events, where constituent parts of the same repeat are assigned to different subclasses.

##### b) Post insertion changes

**Figure 13:**
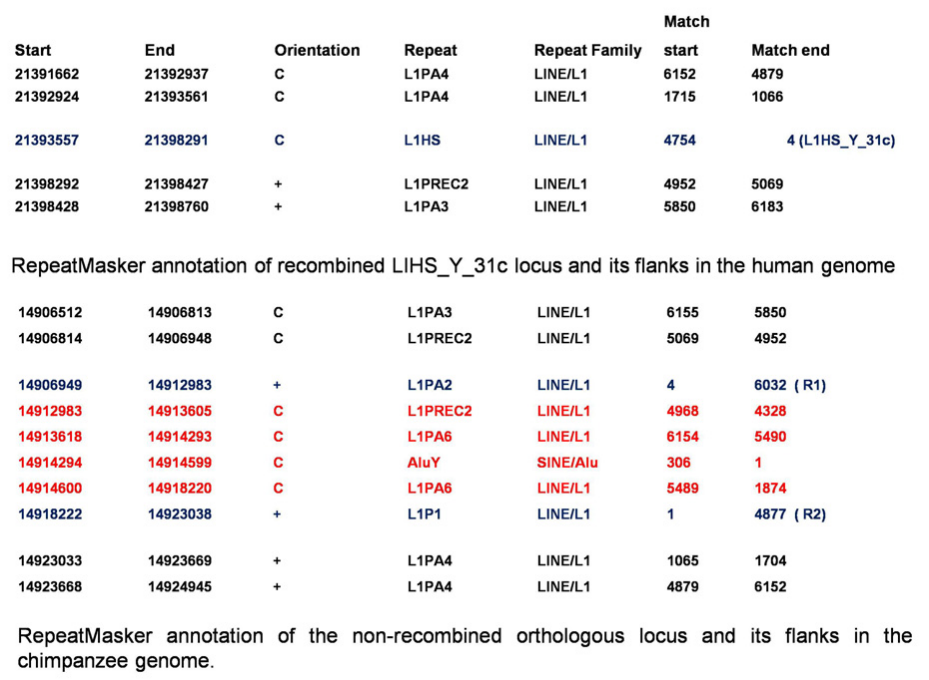
**Repeat Masker annotations for the recombined locus in the human genome and the original non-recombined ortholog in chimpanzee - Please note that the ortholog in chimpanzee was identified in the opposite orientation and should therefore be read in reverse direction when comparing with the human locus**. Loci marked as R1 and R2 (in blue) in chimpanzee annotation represent the loci that recombine in the human genome to give rise to the Y_31c locus. The region marked in red represents the intervening sequence lost in recombination.

Both recombination and disruptions were reported by RISCI. The details may be referred to in Additional files 1 and 2. 16 C_DISRUPTED_M_INTER_RMD events were inferred on the basis of alignments obtained at the orthologous loci in chimpanzee. Since the RepeatMasker files for both reference human and reference chimpanzee genomes were available, we pulled out the repeat annotations for the locus and its flank in the human genome and the identified ortholog and flanks in the chimpanzee genome to confirm recombination (Additional file 1). For example, Y_31c represents a perfect case of inter element recombination in the human (reference or main) genome (M_INTER_RMD) and preservation of the ancestral locus in chimpanzee. The orthologous locus in chimpanzee has no Ns and partially homologous sequences at the 5' and 3' ends (Figure 13, Additional file 2). The recombination between the two results in loss of 11,354 bases in the human genome.

N-scores ranging from 0.36 to 8.11 were found for the remaining 11 loci. L1HS_1_28, 1_40, 8_35, 9_25, 11_17c, 14_35 and 18_38 also represent M_INTER_RMD. In each of the above cases, stretches homologous to the repeat locus in the reference genome were present at 5' and 3' ends of the identified ortholog, and recombination resulted in the loss of one copy equivalent of the homologous sequence and the intervening sequence. However, in most of these cases (except L1HS_9_25 and 18_38) Ns were strategically located in between the two potential homologous stretches of L1s in chimpanzee which recombine to form the lone L1 in the human genome. L1HS 9_25 and 18_38 result from recombination between distant L1s leading to loss of more than 5 kb of intervening sequence.

L1HS_11_4 on the other hand represents minor disruption (C_DISRUPTED) of the orthologous locus in the chimpanzee genome. L1HS_4_84c, 7_73 and 11_4 represent occupied loci in chimpanzee, but are annotated so because of overrepresentation of Ns and misannotation of boundaries by RepeatMasker. L1HS_17_2 is doubtful. The remaining 4 (L1HS 2_72c, 5_46c, 7_29 and X_63c) had N-scores greater than 10 and were not considered further.

32 C_INTER_RMD_M_DISRUPTED events were identified in chimpanzee of which 9 (L1HS 1_61, 1_63, 3_17c, 6_19c, 6_41, 7_21, 8_19, 16_16c and 19_12c) were found to be true inter element recombination events in chimpanzee (C_INTER_RMD). On closer inspection, another 14 loci were found to be disrupted in the human (reference) genome (M_DISRUPTED), with only one of the two fragments annotated as L1HS (except 13_34c and 13_35c). These include L1HS 1_45, 3_7c, 3_57, 4_59, 4_114c, 4_130c, 4_134, 5_54c, 6_38, 6_71, 7_67, 12_31c, 13_34c and 13_35c. Alu element insertion into the parent L1 was the most common cause of disruption. Intriguingly, Alu showed preferential insertion around 300 bases starting from the 5' end of L1. L1HS_6_38 harbors an SVA insertion. Three (L1HS 6_56, L1HS 7_52 and 8_32c) of the identified orthologs had high N-scores. The orthologous loci for 16_11 and X_85 are actually occupied but were annotated so since no contiguous match is found for one of the two repeat overhangs. The remaining 5 loci, L1HS2_3c, 3_14c, 14_20 and 16_24 are difficult to explain. L1HS_2_3c may be a result of parallel independent insertions. L1HS_3_14c is annotated as C_INTER_RMD_M_DISRUPTED in Celera and HuRef comparisons as well and the separation between the flanks is identical. There is homologous L1 sequence in the opposite orientation immediately downstream where recombination may have taken place in these genomes to give rise to the present ortholog. The ortholog for 14_20 has N-stretch at its 3' end, confounding the analysis and, 16_24 locus in the human genome has several Alus inserted into an L1 cluster. The ortholog in chimpanzee is also similar.

10 orthologs were annotated as C_INRA_RMD. L1HS_3_24c presents a picture perfect C_INTRA_RMD event. The identified ortholog has no Ns in the chimpanzee genome. The L1 locus in the human genome is annotated as

36637972   36641523   C   L1HS   LINE/L1   (1)   6154   2621

The RepeatMasker annotation for the orthologous locus in chimpanzee is

37462397   37462754   C   L1PA3   LINE/L1   (0)   6155   5837

37462759   37462898   C   L1P1   LINE/L1   (3397)   2749   2611

This clearly suggests intra element recombination resulting in the loss of 3076 bases of L1 sequence in chimpanzee. Orthologs for L1HS 2_15, 4_51, 10_30c, 14_31, 15_23 and X_45c had low N-scores but the breakpoint was located in Ns. If the Ns are truly representative, these represent true intra element recombination events. L1HS_3_10, X_96 and X_105 had N-scores > 10 and were discarded.

Another 32 loci were annotated as M_INTRA_RMD (Intra element recombination mediated deletion in the reference or main genome). 6 of these had N-score greater than 10 and were not considered. L1HS_4_5 (N-score-0) presents a perfect M_INTRA_RMD event. The RepeatMasker annotation for the complete locus in the human genome is -

13409700-13411042   + L1HS LINE/L1    1   1334 (L1HS_4_5)

3411031-13415298    + L1PA3 LINE/L1   1901   6168

And the identified orthologous locus in chimpanzee is annotated as -

13672242   13678285   +   L1PA3   LINE/L1    1   6045

This very clearly suggests that the ancestral full length insertion in the human genome has undergone intra element recombination resulting in loss of intervening sequence between regions of micro-homology and producing 2 truncated elements, only one of which is annotated as L1HS. Similarly, L1HS_3_118, 3_88c, 4_4c, 4_129, 7_19c, 8_5 and 13_18 have N-scores of 0 and represent confirmed M_INTRA_RMD loci. L1HS_10_39 and 11_25 represent special cases where the recombined locus has further undergone disruption in the human genome, while full length L1 element is conserved in chimpanzee. Ns were found at the breakpoint for L1HS_1_13, 1_48, 3_54, 3_83, 9_44, 10_29c, 16_23 and 18_22, confounding the analysis. L1HS_3_20c, 4_52, 5_52, 5_93c, 8_50c and 12_11 are falsely reported as M_INTRA_RMD and are probably parallel independent insertions.

##### c) Inferences based on empty allele at the orthologous locus

TSDs were identified for 763 loci. Among these, 138 were annotated as twin priming events and 12 were annotated as disruptions. Thus, effectively 613 empty orthologous loci were found in chimpanzee. These were further subdivided into three classes based on the position of the 3' TSD and sequence composition of the stretch between the annotated 3' end of L1 and start of the 3' TSD.

Canonical transposition

426 (of 613) loci in the reference human genome were annotated as CAN - exclusive mobilization of the transposon sequence (Figure 5). Another 109 loci were annotated as PAC (Canonical with a misannotated 3'end, Figure 6).

Non-canonical transposition (3' flank transduction)

The remaining 78 loci qualified as putative 3' flank transduction events and were annotated as PTS (loci with Putative Transduced Sequence). The source locus was unambiguously identified for 42 both in the human and chimpanzee genomes. The source locus was clearly identified in the human genome for L1HS_3_80, 11_43 and 15_1 but no matches were found in the chimpanzee genome. Another 13 loci, (L1HS_1_103, 2_43, 3_26, 5_3, 9_22, 11_11c, 11_34, 12_7, 14_28, 20_19, 21_12, X_50 and X_97), represent twin primed or disrupted L1s in the human genome for which only one of the two constituents is annotated as L1HS by RepeatMasker, leading to misannotation by RISCI. For another 4 (L1HS_2_51, 5_61, 8_40 and X_60) matches were not found for one of the two constituent halves leading to misannotation by RISCI. The A-score and/or AT-score of L1HS_1_58, 1_75, 1_79, 4_21, 4_93 and 9_45 were very close to the threshold and represent marginally misannotated poly-A tails. The PTS was very small for X_84 (20 bases). The PTS for another 6 (L1HS_1_29, 2_32, 2_45, 4_33, 7_24c, X_33) was repeat rich preventing identification of the source locus. The remaining 2 (L1HS_3_39 and 5_22c) are misannotated as PTS by RISCI. The length of the confirmed transduced flanks ranged from 30 bp to 2100 bases (Additional file 2).

##### Insertion-mediated deletion or parallel independent insertions or insertion-deletions

86 INDELS (43 INDEL_CAN, 14 INDEL_PAC and 28 INDEL_PTS) were reported. Of the 44 loci annotated as INDEL_CAN, 4 had N-score above 10. Of the remaining 40, for 24 loci (L1HS_1_47, 1_84c, 3_2, 3_5, 4_58c, 5_5c, 5_51c, 5_65c, 7_7c, 7_10, 8_27, 8_41, 8_42c, 8_43c, 10_24, 12_14, 12_27, 15_18, 18_5c, 18_8, 20_3, X_13, X_72 and X_114), the flanks were separated by less than 50 bases and probably represent insertion-mediated deletions. Of these, 3, (L1HS_7_7c, 8_41, 8_42c), were earlier reported by Han et al. L1HS_1_3 is a false positive. L1HS_1_69c is peculiar since the L1 insertion in chimpanzee is slightly smaller than the insertion in human suggesting parallel independent insertion post divergence of human and chimpanzee genomes. N-stretch at the beginning of the identified ortholog for L1HS_1_86c confounds its analysis. L1HS_9_31 represents an occupied locus, but is annotated as INDEL_CAN for lack of complete matches for the repeat overhangs. L1HS_2_55 and 3_53 insertions in the human genome result in deletion of 385 and 69 bases of non repeat sequence respectively. L1HS_11_41c actually represents a recombination event in the human genome (M_INTER_RMD) but is annotated as INDEL_CAN for lack of complete match for the 3' overhang. L1HS_10_43c has been earlier reported as confirmed L1 insertion-mediated deletion. The identified orthologs for L1HS_2_83, 3_108, 4_48, 16_25, 22_2c and Y_14c are repeat rich and could either represent sequences deleted upon L1 insertion in the human genome or parallel independent insertions. L1HS_4_74 has very low query coverage for the 5' flank and may be a false positive. L1HS_7_11 also has very low query coverage for the 5' flank and an N-score ~10 and therefore discarded.

14 orthologs were annotated as INDEL_PAC. Of these, 2 had N-score > 10 and were not considered further. Of the remaining 12, 8 (L1HS_2_18c, 3_48, 3_74, 4_37, 5_12, 7_45c, 12_38 and 18_18) had the flanks separated by not more than 50 bases and most likely represent insertion-mediated deletion. L1HS_11_62 and 16_1 (16_1 - also reported as insertion-mediated deletion earlier by Han et al.) have RISCI score of 100 and almost full query coverage and represent insertion-mediated deletions. L1HS_7_47 and 8_21c have low RISCI score and are doubtful.

28 loci were annotated as INDEL_PTS by RISCI. Of these, 15 had N-scores lower than or equal to 10. Most of the transduced sequence is repetitive in nature and could not be traced to the source locus.

#### Inferences based on comparisons with Celera and HuRef genome

In contrast to the chimpanzee genome, 1227 loci in the Celera and 1174 in the HuRef genome were annotated as OCCUPIED (Additional file 4). Among these, 1107 loci were commonly occupied in all 3 human genomes representing the more ancestral or fixed loci. Of these, 382 were inserted in genes.

8 C_DISRUPTED_M_INTER_RMD were reported in comparison with Celera genome. Of these, 4 have N-score below 10, 3 of which (L1HS 4_117c, 8_26 and 11_41c) are true inter-element recombination in the human genome. The recombining L1s were separated by 437 and 1216 bases in L1HS_8_26 and 11_41c respectively, and adjacent to each other in L1HS_4_117c. L1HS_2_42 represents a minor disruption of the parent repeat (C_DISRUPTED) in the Celera genome. Of the 13 C_INTER_RMD_M_DISRUPTED reported, 9 had N-score below 10. Of these, 12_41 is a confirmed inter-element recombination (C_INTER_RMD) in the Celera genome. L1HS_4_8, 4_9, 18_2c and 18_3c represent disruption in one of the two halves of a twin-primed L1 in the human genome (M_DISRUPTED). 11_30c is actually OCCUPIED but misannotated due to lack of match for the 3' repeat overhang. L1HS_3_14c, 4_23c and 7_15c are annotated as C_INTER_RMD_M_DISRUPTED, but the region of homology where recombination may have taken place is not apparent. 3 C_INTRA_RMD events identified in Celera have varying length N-stretch and are possibly assembly errors. Of the 7 M_INTRA_RMD loci, only one had an N-score <10 (N-score = 0) and represents true M_INTRA_RMD event (Additional files 4, 5 and 6).

11 C_DISRUPTED_M_INTER_RMD were reported by RISCI in the HuRef assembly 5 of which had N-scores less than 10 (Additional files 1 and 2). Of these L1HS_2_41, 9_49c, 14_20 and 18_5c represent inter element recombination in the human genome. L1HS_11_57 is doubtful. 1 C_INTER_RMD_M_DISRUPTED are reported in HuRef assembly. Of these, 5 had N-score > 10. Of the remaining 16, 6 (L1HS_4_29, 5_80, 5_97, 18_12c, 20_16c and Y_19) had Ns at the 5' or the 3' end of the identified ortholog. These are most likely to be OCCUPIED loci but annotated so for lack of match to one of the repeat overhangs due to Ns. L1HS_11_30c is also OCCUPIED but misannotated. L1HS_1_63 represents inter element recombination in the HuRef genome. L1HS_2_49c, 2_50c, 4_8, 4_9, 13_34c, 18_2c and 18_3c represent disruptions in the main genome (M_DISRUPTED). L1HS_3_14, as mentioned earlier, is annotated as C_INTER_RMD_M_DISRUPTED in all the three comparative genomes. However, the region of homology where recombination takes place is not apparent. 12 C_INTRA_RMDs were reported in HuRef. 7 had low N-scores. Of these, the orthologs for L1HS_10_25 and 13_20 have low N-scores and differ considerably from reference human insertion and may represent true intra element recombination in HuRef. Of the 36 reported M_INTRA_RMD events, only 6 had N-score less than 10. L1HS_2_51c, 5_93c and 8_5 represent true M_INTRA_RMD events. A longer length L1 was found at the orthologous locus in HuRef for each of these and the L1 sequence from the main genome matched perfectly either to the 5' or the 3' end of ortholog.

TSDs were identified for 90 elements in Celera and 112 in comparison with HuRef assembly. These represent recent and, therefore, polymorphic insertions in the human genome, amenable to phylogenetic studies. Of these, 62 in Celera and 76 in HuRef comparisons were canonical insertions in the reference human genome, 16 in Celera and 22 in HuRef had misannotated poly A tails (PAC) and 12 loci in Celera and 14 in HuRef were annotated as PTS (3' flank transduction). The source locus in the reference genome and comparative genomes was unambiguously identified for 6 (L1HS_10_28, 18_12c, 4_92, 5_74, 7_32 and X_113) loci in Celera and 5 (L1HS_4_92, 5_74, 6_12c, 7_32 and 4_83) in HuRef (Additional file 2). The PTS sequence for others was repeat-rich, preventing identification of the source locus.

9 INDELS were reported in comparison with the Celera genome. 5 had N-scores less than ten. Of the 3 loci annotated as INDEL_CAN or INDEL_PAC, L1HS_4_37 (annotated as INDEL_CAN in Chimpanzee and HuRef as well) and X_72 represent insertion mediated deletions. The ortholog identified for L1HS_5_93c has Ns at the beginning of the sequence confounding the analysis. Of the 6 loci annotated as INDEL_PTS, 2 had N-score < 10. L1HS_6_12c was found to true and the source locus for the PTS was also unambiguously identified. L1HS_12_42 may be false positive. 11 INDELS were reported in the HuRef assembly. Of these, 6 had N-score below 10. Three of the remaining 5 loci (L1HS_9_31, X_45c and Y_9) have Ns either in the beginning or end of the ortholog sequence. L1HS_4_37 represents insertion-mediated deletion. Y_30c is a false positive.

17 twin-priming events and 1 disruption were identified in Celera comparisons since most loci are nondifferential. 24 twin priming events and 1 disruption were identified in HuRef genome.

### 3. Analysis of AluYa5 retrotransposons

A total of 4056 (full length and truncated) AluYa5 elements were mined by RISCI in the reference human genome by using the RISCI_RM (direct parsing of repeat coordinates from pre-masked files) option. Using an arbitrary threshold of 285 bases, 3418 qualified as full length and 638 as truncated. 1594 of all Alus were inserted into genes in the reference human genome (5' UTR or intronic). Unless otherwise stated, the inferences refer to the transposon locus in the reference (reference human) genome (Table 1, Additional files 7, 8, 9 and 10).

#### Inferences based on the orthologous locus in the reference chimpanzee genome

##### a) Shared ancestry

314 loci were found to be occupied at the orthologous loci in chimpanzee.

##### b) Post insertion changes

5 loci were annotated as C_DISRUPTED_M_INTER_RMD. Of these, 2 (Alu_1_38 and X_18c) had N-scores > 10 and were not considered further. Of the remaining 3, 2 (Alu_6_210 and 16_96c) were confirmed as M_INTER_RMD, while Alu_17_2 represents a truncated insertion in human and full length insertion in chimpanzee. A recombination between Alu monomers may be responsible for this situation. 90 C_INTER_RMD_M_DISRUPTED events were identified in chimpanzee. Of these, 72 were found to be true inter-element recombination (C_INTER_RMD) events in chimpanzee (Additinal files 3, 4). Another 8 (AluYa5_5_145c, 6_27c, 6_226c, 11_160c, 17_5, 20_69, 22_19, 22_31 were found to be OCCUPIED but were annotated so for lack of almost perfect match for the repeat overhangs. AluYa5_2_250 has Ns at the beginning of the identified ortholog and hence misannotated. It too is likely to be occupied. The remaining 7 (7_95c, 15_89, 17_100, 17_105, 19_57, 20_26, 7_95) are doubtful. As expected, no C_INTRA_RMD event was identified. M_INTRA_RMD option was inactivated for this run.

##### Inferences based on empty allele at the orthologous locus

TSDs were identified for 3209 loci, of which 3132 loci were annotated as CAN, 54 as PAC and 23 as PTS. However, all 23 predicted transduced sequences were either repeat rich or were too small to facilitate identification of source locus (Additional file 5).

##### Insertion-mediated deletion or parallel independent insertions or insertion-deletions

267 loci (164 INDEL_CAN, 7 INDEL_PAC and 96 INDEL_PTS) were annotated as INDELS. Of the 171 INDEL_CAN or INDEL_PAC, 132 had N-score less than 10. At least 60 of these (marked in blue) appear to be insertion-mediated deletions. Another 13 are recombination-mediated deletions, misannotated as INDEL_CAN for lack of match for the repeat overhang (marked in red or brown) (Additional files 7, 8 and 9). Of the 96 loci annotated as INDEL_PTS, 34 had N-score less than 10. As mentioned earlier, we advise user discretion while dealing with INDEL_PTS. Most of these may result from RISCI trudging into loci that are not truly orthologous for lack of sequence (substituted by Ns) at the actual orthologous locus.

#### Inferences based on comparisons with Celera and HuRef genomes

In contrast to the chimpanzee genome, 3530 and 3335 loci were found to be OCCUPIED in Celera and HuRef genomes respectively (Additional file 7).

9 loci in Celera and 6 in HuRef were annotated as C_DISRUPTED_M_INTER_RMD. All 8 orthologous loci in Celera (N-score < 10) and 4 in HuRef (N-score < 10) had homologous Alu sequences at the 5' and the 3' end, confirming inter-element recombination in the human genome. 22 in Celera and 74 in HuRef were annotated as C_INTER_RMD_M_DISRUPTED. Of these, 13 in Celera had N-score < 10. Of these, 2 (AluYa5_3_94c and 18_41c) had Ns at the beginning or end of the identified ortholog. Of the remaining 11, 7 were confirmed as C_INTER_RMD. Other 3, AluYa5_2_181, 6_204 and 22_19, were found to be occupied. AluYa5_5_222c is doubtful. Of the 74 loci in HuRef, 46 had N-scores <10. Of these 46, 29 had Ns at the beginning or end of the identified ortholog sequence and are likely to be occupied in HuRef. AluYa5_2_67c, 8_19, 9_172c, 16_26, 16_67, 17_48 and 19_28 are true inter Alu recombinations in the HuRef genome. The orthologous locus identified for AluYa5_16_26, 16_28c, 16_37 and 16_38 was the same. 6 loci were found to be OCCUPIED but missannotaed as C_INTER_RMD_M_DISRUPTED for lack of match for one of the repeat overhangs.

330 (326 CAN, 2 PAC and 2 PTS) loci in Celera and 428 (420 CAN, 4 PAC and 4 PTS) in HuRef were found to be empty. 59 INDELS (34 INDEL_CAN, 1 INDEL_PAC and 24 INDEL_PTS) were reported in Celera genome. 19 of these had N-scores less than 10. Of these, 4 (AluYa5 3_54, 4_120c, 11_26 and X_7) had Ns at either the beginning or the end of the identified ortholog confounding the analysis. AluYa5 2_322c (10 bp), 4_245 (913 bp), 8_149 (3 bp), 15_74 (25 bp) and X_75 (1966 bp), represent insertion-mediated deletions. The orthologs for Alu_4_194c and 14_98c have full length Alu sequence at the 5' end followed by non Alu sequence suggesting gene conversion, while 6_52 represents parallel insertion of LTR sequence Alu_2_59 possibly results from recombination between Alu monomers. 132 (79 INDEL_CAN and 53 INDEL_PTS) in HuRef were reported. 33 of these had N-score less than 10. Of these, AluYa5_2_59, 2_322c, 4_194c, 4_245, 6_52, 8_149, 14_98c and 15_74 are exactly similar to Celera orthologs as described above. Another 7 (AluYa5_1_313, 5_10, 10_109c, 13_23, 14_100, 20_71c and X_4) had Ns either in the beginning or end of the ortholog sequence leading to misannotation.

### Novel polymorphism

A total of 45 polymorphic sites were identified in comparison with the Celera and HuRef assemblies. Of these 32 were common to both Celera and HuRef, while for others the orthologous locus was empty either in Celera or HuRef assembly. To ascertain how many of the 45 polymorphisms were novel, we cross checked with the L1 insertion polymorphism data in dbRIP by using its recently incorporated 'Position mapping' utility [71]. Of the 45 polymorphic sites reported, 14 did not find a match in the dbRIP recently updated data and are novel (Table 3, Additional files 11 and 12). Of these, 9 had RISCI score of 100 (unique ortholog identified). Likewise, for truncated L1HS, of the 113 empty orthologous loci either in Celera or HuRef or in both, 47 were not found in dbRIP. 24 of these had RISCI score of 100. Of the 435 AluYa5 loci for which an empty ortholog was identified in Celera or HuRef genomes or in both, 140 are not mentioned in dbRIP. All of these had RISCI score of 100 suggesting unambiguity in identifying the ortholog (Additional file 12). The polymorphic sites essentially represent insertions in the reference human genome but absent in Celera or HuRef or in both.

**Table 3:** Novel polymorphic loci predicted by RISCI for full length L1HS by comparison of reference human genome with the alternate human genomes

LOCUS	Ortholog empty in	R-score	hg18 coordinates	Match in dbRIP
L1HS_1_4c	HuRef	80	chr1:81177500-81183677	NA
L1HS_1_5c	Celera, HuRef	100	chr1:84290051-84296742	NA
L1HS_4_3c	Celera, HuRef	100	chr4:18688621-18694707	NA
L1HS_4_13c	Celera, HuRef	56.5	chr4:75861787-75867832	NA
L1HS_5_24	HuRef	100	chr5:177131852-177137889	NA
L1HS_7_10c	Celera, HuRef	100	chr7:96313896-96319990	NA
L1HS_9_2c	Celera, HuRef	100	chr9:46329639-46335695	NA
L1HS_11_4c	Celera, HuRef	57	chr11:48825824-48831881	NA
L1HS_11_12	Celera, HuRef	99	chr11:92793798-92799846	NA
L1HS_14_1c	Celera, HuRef	88	chr14:18130292-18136344	NA
L1HS_X_9c	HuRef	100	chrX:65317263-65323363	NA
L1HS_Y_1	Celera	100	chrY:3371591-3378526	NA
L1HS_Y_2c	Celera	100	chrY:4876952-4882987	NA
L1HS_Y_3c	Celera	100	chrY:5534205-5540267	NA

Details for these loci may be referred to in Additional file 1.

## Discussion

### Salient features of RISCI

RISCI offers both whole genome as well specific region analyses. It runs on contig as well as on assembled chromosome sequence, allows multiple genome comparisons, offers three repeat mining utilities (RISCI_RM, RISCI_NON_RM and RISCI_BLAST, and two filters 'length' and 'gene' (see materials and methods). Wherever possible, the upstream query sequence is tagged with a user defined length of non repeat sequence (default- 500 bp) to avoid spurious hits (see materials and methods). In most cases this non repeat tag forms a part of the upstream blast hit used in RISCI annotation (Additional File 17 Figure S4). RISCI also uses improvised soft masking (see materials and methods) to arrive at the orthologous locus in the comparative genome. The blast databases of the genomes are made with the - o option set to T to enable use of fastacmd so as to speedily retrieve flank sequence from the reference genome and the ortholog sequence from the comparative genome. A merger option is also provided so as to merge BLAST hits in the comparative genome if the gap between two similarly oriented Blast HSPs is not greater than the user defined length (default 50 bp) both in terms of the query and subject coordinates. A scoring scheme has also been implemented to assign confidence scores in cases where multiple orthologous loci are predicted (see materials and methods). As mentioned above, specialized modules to take care of complications involved in truncated repeat analysis are inbuilt. Confirmation module for flank transduction is also inbuilt in RISCI. Besides, 3 speed options are inbuilt (Table 4).

**Table 4:** Details of Speed options in RISCI

Parameters	Fast	Medium	Slow
Blast -v	2	3	5
			
Maximum no of Blast HSPs compared	100 (in each orientation)	500 (in each orientation)	10000 (in each orientation)
			
Pros and cons	fastest, least accurate	Fast, reasonably accurate	Most accurate

For each of the speed options (Fast, Medium and Slow), speed may be further enhanced by selecting for "STOP AT FIRST MATCH (SFM)" as opposed to "ALL AGAINST ALL COMPARISONS". SFM option stops further comparisons as soon as the first match conforming to any of the RISCI alignment signatures is found. To avoid orientation bias, the control shifts between plus and minus hits every 15 hits. The scoring scheme becomes redundant since only one match is allowed, and hence duplications cannot be identified with SFM option.

Medium speed option with SFM off and merger on is recommended.

### Comparison with other tools

To the best of our knowledge, no *in silico *tool comparable to RISCI is available till date. However, several experimental strategies to identify potential polymorphic sites with respect to transposon insertion have been suggested in recent years. These include TGDA (Targeted Genomic Difference Analysis)[72], diffIR [73], and a new general approach to identify insertion deletion polymorphisms [74]. Whole genome *in silico *comparison strategies have also been used earlier but have been restricted to specific goals like identifying novel polymorphisms [75,76] or insertion-mediated deletions [65] or recombination-mediated deletions [77,78]. Bennet et al made an automated pipeline to identify indel and transposon polymorphism from sequence traces [79]. Mills et al identified 11000 transposon copied that are differentially present in the human and chimpanzee genomes [80] (Refer Additional file 13 for comparison of RISCI data with that of Mills et al). Recently, Xing et al combined computational and experimental analyses to identify structural variations in the HuRef genome [81]. As has been mentioned earlier, RISCI is more comprehensive and provides a one-stop platform to identify a wide array of sequence changes, besides polymorphism, presenting a more holistic and comparative view of sequence changes occurring as a consequence of transposon insertions, which may then be examined for their downstream effects.

### RISCI validation

We estimated the accuracy of RISCI indirectly by comparing the TSDs obtained for the same locus in Celera or HuRef genome with those obtained in chimpanzee for full length L1HS. Of the 45 polymorphic loci identified in the human genome (Additional files 11 and 12), TSDs were predicted for 42 in the chimpanzee genome. Of these, 33 loci had exactly identical TSDs in the human and chimpanzee genomes. Of the remaining 9, 4 differed by not more than 2 nucleotides either at the 5' or the 3' end (data available on request). Even when there were large differences in the size of TSD (> 2 bases), the relative query coverage was almost similar. Given that the human and chimpanzee diverged some 6 million years ago and have undergone independent evolutionary pressures and consequent changes, the tendency of target site duplications to decay, as also the possibilities of miniscule errors in the assembly, the accuracy still comes to approximately 88.09% ((37/42)*100). A similar figure was obtained for truncated L1HS and AluYa5 analyses. Also, of the 32 predicted 3' flank transduction events in the chimpanzee genome for full length L1HS, the source locus was unambiguously identified for 23 both in human and chimpanzee genome and for 28 in the human genome alone (87.5% accuracy).

We also estimated the accuracy of RISCI by partially recapitulating the analysis done by Sen et al [46]. The recombined Alu coordinates (hg16) were picked from this study and converted to hg18 coordinates using the liftover utility at the UCSC genome browser. 4 of the converted coordinates did not harbor an Alu within ±50 bases and were dropped from the analysis. Of the remaining 488, Alu start and end coordinates coincided exactly with the converted hg18 coordinates for 472 loci. Thus, a total of 488 loci were fed into RISCI using the RISCI_NON_RM module (Repeat coordinates input directly by the user), 1000 base flanks retrieved, and the orthologous locus in chimpanzee zoomed into using the pan Tro 2.1 assembly as the blast database (Additional files 14 and 15).

9 identified orthologs were annotated as OCCUPIED of which 7 were confirmed by comparing the RepeatMasker annotations of the locus in the human genome and the orthologous locus in chimpanzee (Additional file 6). These include AluYa5_7_174c, 7_180, 10_224c, 10_239, 16_350, 19_407 and 20_458c. The match for 3' flank of Alu_20_458c is very small (200 bases) and may be a false positive. Alu_17_353 is actually a recombination event falsely classified as OCCUPIED. No matches were found for 16 loci in the chimpanzee genome (it may be noted that we have not included the random sequence files in our blast database for chimpanzee). As expected, of the remaining 463 loci, a major fraction (398 of 463 - 85.96%) were annotated as C_DISRUPTED_M_INTER_RMD by RISCI (actually M_INTER_RMD). Another 58 loci were annotated as INDEL_CAN. Of these 46 actually represent M_INTER_RMD (Additional file 6), but were annotated as INDEL_CAN for lack of near complete match for repeat overhangs (at least 35 of 50 bases). For the remaining 12 INDEL_CAN, the putative regions of homology where recombination in human takes place resulting in the current status are not apparent. All of the 6 loci annotated as INDEL_PTS had N-scores > 5 (4 had N-scores > 10) and were not considered further. 1 orthologous locus was falsely interpreted as C_INTER_RMD_M_DISRUPTED by RISCI. Thus, a total of 445 (398 M_INTER_RMD, Alu_17_353 and 46 INDEL_CAN) identified orthologous loci represent inter Alu recombination in the human genome (445/463 = 96.1%).

To further demonstrate the versatility of RISCI, we used it for a preliminary analysis of insertion polymorphism of IS element 6110 (DNA transposon) using *Mycobacterium tuberculosis *h37rv as the reference genome and related strains *Mycobacterium tuberculosis *h37ra, cdc1551 and and F11 as comparative genomes. 2000 base of flanks with 50 base overhangs into the repeat were used to zoom into the orthologous locus in the comparative genomes (Additional file 16).

### RISCI Limitations

Accuracy of RISCI predictions is a function of the sequence quality of the genomes being compared, as also of the quality of repeat annotation by RepeatMasker. In the absence of the true orthologous sequence in the comparative genome, RISCI may trudge to other loci, given the high repeat content (full length L1HS - 52.4%, truncated L1HS - 60.2% and AluYa5 - 51.4% - 5 kb up and downstream) of the flanks. Lack of sequence information substituted by an estimated number of Ns is a major spoil sport resulting in misannotations (Figure 10). It may also be noted that results for some loci may change depending on the speed options selected.

### RISCI availability

RISCI may be downloaded from http://www.ccmb.res.in/rakeshmishra/tools.html (RISCI.tar.gz). It is a collection of several scripts written in perl v5.8.5 for ia64-linux-thread-multi and is compatible to LINUX OS. A sample of RISCI run (L1HS.tar.gz) discussed in this paper, may also be downloaded. RISCI requires a prior installation of the EMBOSS module, RepeatMasker and BLAST for execution. A detailed help file is available with the package for assistance of new users and can be accessed at http://www.ccmb.res.in/rakeshmishra/tools/RISCI_Readme.htm.

## Conclusion

The availability of multiple whole genome sequences of the same and different species presents us with an unprecedented opportunity to compare and infer intra species and inter-species structural variations introduced by transposon. We present an automated pipeline to identify fixed and differential transposon insertions and a wide array of transposon induced sequence changes in closely related genomes. We illustrate the utility of the pipeline by comparing the reference human genome with the reference chimpanzee genome and alternate human assemblies (Celera and HuRef) taking L1HS and AluYa5 as representative transposons. We also show that though modeled on LINES, the pipeline is generic and may be applied to most transposons and any two or more genomes which share high sequence similarity. We believe that such comparisons, when done on a larger scale may pull out a few critical events which may have seeded the divergence between the two species under comparison.

## Methods

### Resources

The reference human genome (Build 36.1), alternate human assemblies - Celera and HuRef and the reference chimpanzee genome (pan Tro 2.1) were downloaded from genomes folder of NCBI ftp site ftp://ftp.ncbi.nih.gov. The corresponding RepeatMasker files (hg18) for reference human genome were downloaded from UCSC genome ftp site ftp http://hgdownload.cse.ucsc.edu from goldenPath/hg18/bigZips directory. The corresponding Genbank files (NC_000001 to NC_00000024 - reference human genome, AC_000044 to AC_000067 - Celera genome, AC_000133 to AC_000156 - HuRef assembly and NC_006468 - NC_006492 - reference chimpanzee genome) were downloaded from NCBI. Emboss was installed (downloaded from http://emboss.sourceforge.net/download/on the local bioinformatics server and integrated into RISCI. NCBI standalone blast http://www.ncbi.nlm.nih.gov/BLAST/download.shtml and RepeatMasker http://www.repeatmasker.org/RMDownload.html were locally installed on the bioinformatics server and integrated into RISCI.

### Nomenclature of the Repeat locus

Each transposon locus in the reference or main genome is named according to the chromosome on which it is found as also the order in which it is found. Thus L1HS_1_1 represents the first annotated L1HS on chromosome 1. A suffix "c' is added if the transposon is on the complementary strand.

### Repeat Mining utilities

RISCI offers three modules to mine out user defined transposon and its flanks from the reference or main genome.

#### a) RISCI_RM

Mines out user defined transposon and the repeat annotation of the upstream and downstream flanks from pre- masked RepeatMasker files (.out files) of the main genome.

#### b) RISCI_BLAST

In case of non availability of the RepeatMasker files of the main genome, the transposon and its flanks are mined using repeat specific tag sequences. The tag is typically an oligonucleotide 18-22 bp long carrying the repeat specific signatures, preferably towards the 3' end. This sequence is then Blasted on to the reference genome. Sufficiently long flanking sequence for exact matches found in the main genome is retrieved and RepeatMasked to precisely define the repeat coordinates. The repeat and user defined length of flanks are then retrieved for blast against the comparative genome(s).

#### c) RISCI_NON_RM

The user may directly input the repeat coordinates in the specified format (refer Readme file) using this option.

### Non repeat tagging of upstream flank

The repeat annotation of the upstream flank is parsed so as to check for the nearest user defined length of non repeat tag (NRT) starting from the 3' end. If a non repeat sequence greater than the NRT is found immediately upstream of the transposon, it is used to query the comparative genome. Otherwise, the length of non-repeat sequence between successive repeats is checked till a non-repeat sequence greater than NRT is found. This sequence consisting of the non-repeat tag at the 5' end and the downstream repeat(s) serves as the upstream query sequence. If the non repeat tag is not found, the entire sequence is used as upstream query for Blast against the comparative genome(s) (Additional file 17 Figure S3).

### Blastn database

Blast database of main and comparative genomes were made using the formatdb command -o option set to T. This enables the use of fastacmd command by RISCI to retrieve sequences directly from the blast database, which is faster.

### Improvised soft masking

Based on the RepeatMasker coordinates, the retrieved sequence is soft masked. 50 bases at the 5' and 3' end of each repeat and 500 bases immediately upstream and downstream of the transposon locus are, however, encrypted in upper case letters.

### Blastn

Upstream and downstream flanks with 50 base overhangs into the respective ends of the repeat are blasted separately against the comparative genome and the blast results summarized into the following heads - element, contig, chromosome, orientation, query first coordinate (QFC), query last coordinate (QLC), subject first coordinate (SFC) and subject last coordinate (SLC). If no match is found in the first round of Blastn, a second round of blast is done with the - U option activated. This enforces masking of sequence in lower case letters effectively reducing spurious hits. -v option and the number of hits compared to zoom onto the orthologous locus depend on the speed option chosen.

### Blast HSP parser

For each repeat element, all upstream hits are compared to all downstream hits in the same orientation. If the upstream and downstream hits are on the same chromosome, same orientation and the same contig, and

1. the repeat overhangs align completely and contiguously with the flanks on the comparative genome and (1) the difference between the upstream SLC and the downstream SFC is within 100 bp range of the repeat length in the reference genome, the locus is annotated as OCCUPIED; (2) the difference is greater than repeat length +100 and less than 25000, the locus is annotated as C_DISRUPTED_M_INTER_RMD; (3) the difference is less than repeatlength-100, the locus is annotated as C_INTRA_RMD; (4) only one of the repeat overhangs aligns completely and contiguously with its flank or the overlap between the hits is equal to or greater than the length of the repeat overhangs, the locus is annotated as C_INTER_RMD.

2. [upstream SLC - downstream SFC >-1] (for plus orientation hits) or [downstream SFC-upstream SLC >-1] (for minus orientation hits), and less than the maximum TSD size input, the locus is annotated as CAN, PAC or PTS depending on the QFC of the downstream flank and the A and AT content of the unmatched region in the downstream flank. The orthologous locus is first checked for in the corresponding chromosomal homologue in the comparative genome. In case no match is found, the orthologous locus is checked for TSD on other chromosomes as well.

If no matches corresponding to shared ancestry, post insertion changes and empty orthologous loci are found in the first round of comparison, another round of comparison with Blast option - U activated, facilitating lower case filtering of FASTA sequences, is performed before checking for INDELS.

3. the difference between the upstream SLC and downstream SFC is less than 10,000 bp, and the repeat overhangs are not contiguous with the flanks the locus is annotated as "INDEL". For each of the above RISCI annotations, both upstream and downstream matches should be greater than 99 bases and at least one of them should be greater than 500 bases.

### RISCI score (R-score)

If only one locus in the comparative genome satisfies RISCI annotation conditions, it is allotted a default score of 100. In case of multiple RISCI matches, the default score for each match is 50 and is incremented by 1/2 of the percentage of query match length/total query length. Match with maximum score is then picked up as final RISCI hit and displayed in the main result file. Other hits with their respective scores are also written on to a log file for reference. Please note that the scoring scheme becomes redundant if 'SFM' speed option (refer Table 4) is selected.

### Blast HSP merger module

As mentioned, hits not separated by a distance greater than user defined threshold both in terms of query and subject coordinates may be merged by selecting for the merger option. The threshold is set at 50 but can also be defined by the user.

### Speed optimization

Several speed optimization strategies have been implemented so as make RISCI faster without compromising significantly on the sensitivity. 'fastacmd' command was used to directly retrieve sequences from reference and comparative genomes from respective blast databases. Where ever possible, the upstream query sequence is tagged with a non repeat tag effectively making the upstream query sequence shorter and reducing the number of spurious hits. Since a repeat overhang of 50 bp is integral to both upstream and downstream flanks, while summarizing the blast file, only hits > 52 bases are picked up to void hits to the repeat overhangs alone. In blastn, the -v option is varied according to the speed option selected. This reduces the number of blast hits for comparison in case of repeat-rich flanks.

## Authors' contributions

VS conceived, designed and implemented the study, and wrote the manuscript. RKM helped in data analysis and manuscript preparation. All authors have read and approved the manuscript.

## Authors' information

Dr Singh can also be contacted at the following email: ashvip@gmail.com

## Supplementary Material

Additional file 1 Full length L1HS results Full length L1HS results for reference human genome comparison with chimpanzee, Celera and HuRef genomes Click here for file

Additional file 2 **3' flank transduction results**. Target and source locus for the 3' transduced flanks in reference and comparative genomes for full length and truncated L1HS Click here for file

Additional file 3 **5' flank transduction results**. Target and source locus for the 5' transduced flanks in reference and comparative genomes for full L1HS Click here for file

Additional file 4 **Truncated L1HS results**. Truncated L1HS results for reference human genome comparison with chimpanzee, Celera and HuRef genomes Click here for file

Additional file 5 **RepeatMasker annotations of recombined loci for truncated L1HS**. RepeatMasker annotations of the repeat locus and its flanks in the reference genome and of the identified ortholog and its flanks in the comparative genomes to identify putative regions of homology where recombination takes place. Click here for file

Additional file 6 **Pairwise alignment and RepeatMasker annotation of repeat locus in main genome and the ortholog in comparative genome for truncated L1HS**. Summary of pair-wise alignments between the truncated L1HS locus in the reference genome and the identified ortholog in the comparative genome and its RepeatMasker annotation to confirm RISCI annotation. Click here for file

Additional file 7 **AluYa5 results**. AluYa5 (full length and truncated) results for reference human genome comparison with chimpanzee, Celera and HuRef genomes Click here for file

Additional file 8 **RepeatMasker annotations of recombined loci for AluYa5**. RepeatMasker annotation of the repeat locus and in flanks in the reference genome and of the identified ortholog and its flanks in the comparative genomes to identify putative regions of homology where recombination takes place Click here for file

Additional file 9 **Pairwise alignment and RepeatMasker annotation of repeat locus in main genome and the ortholog in comparative genome for truncated AluYa5**. Summary of pair-wise alignments between the AluYa5 loci in the reference genome and the identified orthologs in the comparative genome and its RepeatMasker annotation to confirm RISCI annotation. Click here for file

Additional file 10 **3' flank transduction results for AluYa5 (Reference human vs chimpanzee)**. Output file of the 3' flank transduction confirmation module of RISCI - includes the putative transduced flank in EMBL format, RepeatMasker annotation for the same and BLAST hits in reference and comparative genome. Click here for file

Additional file 11 **Pre-reported polymorphisms identified by RISCI**. List of polymorphic loci reported by RISCI in human genome comparisons and also reported in dbRIP Click here for file

Additional file 12 **RISCI predicted novel polymorphisms**. List of novel polymorphisms predicted by RISCI (not reported in dbRIP) Click here for file

Additional file 13 **RISCI validation by comparison with earlier studies**. Comparison of RISCI results with Mills et al data Click here for file

Additional file 14 **RISCI validation by comparison with earlier studies**. Comparison of RISCI results with Sen et al data Click here for file

Additional file 15 **RISCI validation**. Alu loci mentioned in Sen et al and annotated as INDELs by RISCI. RepeatMasker annotation of the repeat locus and in flanks in the reference genome and of the identified ortholog and its flanks in the comparative genomes to identify putative regions of homology where recombination takes place Click here for file

Additional file 16 **RISCI results for IS element insertion polymorphism in Mycobacterium tuberculosis strains**. RISCI results for IS element 6110 insertion polymorphism in Mycobacterium tuberculosis strains-reference genome Mycobacterium tuberculosis (Mtb) h37rv, comparative genomes - Mtb f11, Mtb cdc1551 and Mtb h37ra Click here for file

Additional file 17 **Additional figures**. Figures S1 - Alignment signatures for M_DISRUPTED, S2 - RISCI facilitates precise demarcation of transposon boundaries, S3 - Variation in RepeatMasker annotated boundaries and RISCI predicted boundary, S4 - The non repeat tag forms a part of upstream query for most loci. Click here for file

## References

[B1] Wicker T Sabot F Hua-Van A Bennetzen JL Capy P Chalhoub B A unified classification system for eukaryotic transposable elements Nat Rev Genet 2007 8 973 982 10.1038/nrg2165 17984973

[B2] Kapitonov VV Jurka J A universal classification of eukaryotic transposable elements implemented in Repbase Nat Rev Genet 2008 9 411 412 10.1038/nrg2165-c1 18421312

[B3] Gardner MJ Hall N Fung E White O Berriman M Hyman RW Genome sequence of the human malaria parasite Plasmodium falciparum Nature 2002 419 498 511 10.1038/nature01097 12368864 PMC3836256

[B4] Hua-Van A Le RA Maisonhaute C Capy P Abundance, distribution and dynamics of retrotransposable elements and transposons: similarities and differences Cytogenet Genome Res 2005 110 426 440 10.1159/000084975 16093695

[B5] Curcio MJ Derbyshire KM The outs and ins of transposition: from mu to kangaroo Nat Rev 2003 4 1 13 Ref Type: Generic 10.1038/nrm1241 14682279

[B6] Orgel LE Crick FH Selfish DNA: the ultimate parasite Nature 1980 284 604 607 10.1038/284604a0 7366731

[B7] Doolittle WF Sapienza C Selfish genes, the phenotype paradigm and genome evolution Nature 1980 284 601 603 10.1038/284601a0 6245369

[B8] Bowen NJ Jordan IK Transposable elements and the evolution of eukaryotic complexity Curr Issues Mol Biol 2002 4 65 76 12074196

[B9] Kazazian HH Jr Mobile elements: drivers of genome evolution Science 2004 303 1626 1632 10.1126/science.1089670 15016989

[B10] von SR Shapiro JA How repeated retroelements format genome function Cytogenet Genome Res 2005 110 108 116 10.1159/000084942 16093662

[B11] Hedges DJ Batzer MA From the margins of the genome: mobile elements shape primate evolution Bioessays 2005 27 785 794 10.1002/bies.20268 16015599

[B12] Jurka J Kapitonov VV Kohany O Jurka MV Repetitive sequences in complex genomes: structure and evolution Annu Rev Genomics Hum Genet 2007 8 241 259 10.1146/annurev.genom.8.080706.092416 17506661

[B13] Feschotte C Pritham EJ DNA transposons and the evolution of eukaryotic genomes Annu Rev Genet 2007 41 331 368 10.1146/annurev.genet.40.110405.090448 18076328 PMC2167627

[B14] Volff JN Turning junk into gold: domestication of transposable elements and the creation of new genes in eukaryotes Bioessays 2006 28 913 922 10.1002/bies.20452 16937363

[B15] Smit AF Interspersed repeats and other mementos of transposable elements in mammalian genomes Curr Opin Genet Dev 1999 9 657 663 10.1016/S0959-437X(99)00031-3 10607616

[B16] Miller WJ McDonald JF Pinsker W Molecular domestication of mobile elements Genetica 1997 100 261 270 10.1023/A:1018306317836 9440279

[B17] Miller WJ McDonald JF Nouaud D Anxolabehere D Molecular domestication--more than a sporadic episode in evolution Genetica 1999 107 197 207 10.1023/A:1004070603792 10952213

[B18] Quesneville H Nouaud D Anxolabehere D Recurrent recruitment of the THAP DNA-binding domain and molecular domestication of the P-transposable element Mol Biol Evol 2005 22 741 746 10.1093/molbev/msi064 15574804

[B19] Piriyapongsa J Polavarapu N Borodovsky M McDonald J Exonization of the LTR transposable elements in human genome BMC Genomics 2007 8 291 10.1186/1471-2164-8-291 17725822 PMC2008291

[B20] Bejerano G Lowe CB Ahituv N King B Siepel A Salama SR A distal enhancer and an ultraconserved exon are derived from a novel retroposon Nature 2006 441 87 90 10.1038/nature04696 16625209

[B21] Tiedge H Chen W Brosius J Primary structure, neural-specific expression, and dendritic location of human BC200 RNA J Neurosci 1993 13 2382 2390 7684772 10.1523/JNEUROSCI.13-06-02382.1993 PMC6576500

[B22] Lunyak VV Prefontaine GG Nunez E Cramer T Ju BG Ohgi KA Developmentally regulated activation of a SINE B2 repeat as a domain boundary in organogenesis Science 2007 317 248 251 10.1126/science.1140871 17626886

[B23] Smith AM Sanchez MJ Follows GA Kinston S Donaldson IJ Green AR A novel mode of enhancer evolution: the Tal1 stem cell enhancer recruited a MIR element to specifically boost its activity Genome Res 2008 18 1422 1432 10.1101/gr.077008.108 18687876 PMC2527711

[B24] Jordan IK Rogozin IB Glazko GV Koonin EV Origin of a substantial fraction of human regulatory sequences from transposable elements Trends Genet 2003 19 68 72 10.1016/S0168-9525(02)00006-9 12547512

[B25] Wang T Zeng J Lowe CB Sellers RG Salama SR Yang M Species-specific endogenous retroviruses shape the transcriptional network of the human tumor suppressor protein p53 Proc Natl Acad Sci USA 2007 104 18613 18618 10.1073/pnas.0703637104 18003932 PMC2141825

[B26] Marino-Ramirez L Lewis KC Landsman D Jordan IK Transposable elements donate lineage-specific regulatory sequences to host genomes Cytogenet Genome Res 2005 110 333 341 10.1159/000084965 16093685 PMC1803082

[B27] Thornburg BG Gotea V Makalowski W Transposable elements as a significant source of transcription regulating signals Gene 2006 365 104 110 10.1016/j.gene.2005.09.036 16376497

[B28] Speek M Antisense promoter of human L1 retrotransposon drives transcription of adjacent cellular genes Mol Cell Biol 2001 21 1973 1985 10.1128/MCB.21.6.1973-1985.2001 11238933 PMC86790

[B29] Medstrand P van de Lagemaat LN Dunn CA Landry JR Svenback D Mager DL Impact of transposable elements on the evolution of mammalian gene regulation Cytogenet Genome Res 2005 110 342 352 10.1159/000084966 16093686

[B30] Feschotte C Transposable elements and the evolution of regulatory networks Nat Rev Genet 2008 9 397 405 10.1038/nrg2337 18368054 PMC2596197

[B31] Tomilin NV Regulation of mammalian gene expression by retroelements and non-coding tandem repeats Bioessays 2008 30 338 348 10.1002/bies.20741 18348251

[B32] Kazazian HH Jr Mobile elements and disease Curr Opin Genet Dev 1998 8 343 350 10.1016/S0959-437X(98)80092-0 9690999

[B33] Callinan PA Batzer MA Retrotransposable elements and human disease Genome Dyn 2006 1 104 115 full_text 18724056 10.1159/000092503

[B34] Belancio VP Hedges DJ Deininger P Mammalian non-LTR retrotransposons: for better or worse, in sickness and in health Genome Res 2008 18 343 358 10.1101/gr.5558208 18256243

[B35] Boissinot S Entezam A Furano AV Selection against deleterious LINE-1-containing loci in the human lineage Mol Biol Evol 2001 18 926 935 11371580 10.1093/oxfordjournals.molbev.a003893

[B36] De S Teichmann SA Babu MM The impact of genomic neighborhood on the evolution of human and chimpanzee transcriptome Genome Res 2009 19 785 794 10.1101/gr.086165.108 19233772 PMC2675967

[B37] Hayakawa T Satta Y Gagneux P Varki A Takahata N Alu-mediated inactivation of the human CMP- N-acetylneuraminic acid hydroxylase gene Proc Natl Acad Sci USA 2001 98 11399 11404 10.1073/pnas.191268198 11562455 PMC58741

[B38] Szabo Z Levi-Minzi SA Christiano AM Struminger C Stoneking M Batzer MA Sequential loss of two neighboring exons of the tropoelastin gene during primate evolution J Mol Evol 1999 49 664 671 10.1007/PL00006587 10552047

[B39] Ostertag EM Kazazian HH Jr Biology of mammalian L1 retrotransposons Annu Rev Genet 2001 35 501 538 10.1146/annurev.genet.35.102401.091032 11700292

[B40] Cordaux R Batzer MA The impact of retrotransposons on human genome evolution Nat Rev Genet 2009 10 691 703 10.1038/nrg2640 19763152 PMC2884099

[B41] Altschul SF Gish W Miller W Myers EW Lipman DJ Basic local alignment search tool J Mol Biol 1990 215 403 410 2231712 10.1016/S0022-2836(05)80360-2

[B42] Lander ES Linton LM Birren B Nusbaum C Zody MC Baldwin J Initial sequencing and analysis of the human genome Nature 2001 409 860 921 10.1038/35057062 11237011

[B43] The Chimpanzee Sequencing and analysis consortium Initial sequence of the chimpanzee genome and comparison with the human genome Nature 2005 437 69 87 10.1038/nature04072 16136131

[B44] Venter JC Adams MD Myers EW Li PW Mural RJ Sutton GG The sequence of the human genome Science 2001 291 1304 1351 10.1126/science.1058040 11181995

[B45] Levy S Sutton G Ng PC Feuk L Halpern AL Walenz BP The diploid genome sequence of an individual human PLoS Biol 2007 5 e254 10.1371/journal.pbio.0050254 17803354 PMC1964779

[B46] Sen SK Han K Wang J Lee J Wang H Callinan PA Human genomic deletions mediated by recombination between Alu elements Am J Hum Genet 2006 79 41 53 10.1086/504600 16773564 PMC1474114

[B47] Sheen FM Sherry ST Risch GM Robichaux M Nasidze I Stoneking M Reading between the LINEs: human genomic variation induced by LINE-1 retrotransposition Genome Res 2000 10 1496 1508 10.1101/gr.149400 11042149 PMC310943

[B48] Ho HJ Ray DA Salem AH Myers JS Batzer MA Straightening out the LINEs: LINE-1 orthologous loci Genomics 2005 85 201 207 10.1016/j.ygeno.2004.10.016 15676278

[B49] The Chimpanzee Sequencing and analysis consortium Initial sequence of the chimpanzee genome and comparison with the human genome Nature 2005 437 69 87 10.1038/nature04072 16136131

[B50] van de Lagemaat LN Gagnier L Medstrand P Mager DL Genomic deletions and precise removal of transposable elements mediated by short identical DNA segments in primates Genome Res 2005 15 1243 1249 10.1101/gr.3910705 16140992 PMC1199538

[B51] Mills RE Bennett EA Iskow RC Luttig CT Tsui C Pittard WS Recently mobilized transposons in the human and chimpanzee genomes Am J Hum Genet 2006 78 671 679 10.1086/501028 16532396 PMC1424692

[B52] Poulter RT Goodwin TJ DIRS-1 and the other tyrosine recombinase retrotransposons Cytogenet Genome Res 2005 110 575 588 10.1159/000084991 16093711

[B53] Goodwin TJ Butler MI Poulter RT Cryptons: a group of tyrosine-recombinase-encoding DNA transposons from pathogenic fungi Microbiology 2003 149 3099 3109 10.1099/mic.0.26529-0 14600222

[B54] Kapitonov VV Jurka J Helitrons on a roll: eukaryotic rolling-circle transposons Trends Genet 2007 23 521 529 10.1016/j.tig.2007.08.004 17850916

[B55] Sen SK Huang CT Han K Batzer MA Endonuclease-independent insertion provides an alternative pathway for L1 retrotransposition in the human genome Nucleic Acids Res 2007 35 3741 3751 10.1093/nar/gkm317 17517773 PMC1920257

[B56] Pickeral OK Makalowski W Boguski MS Boeke JD Frequent human genomic DNA transduction driven by LINE-1 retrotransposition Genome Res 2000 10 411 415 10.1101/gr.10.4.411 10779482 PMC310862

[B57] Goodier JL Ostertag EM Kazazian HH Jr Transduction of 3'-flanking sequences is common in L1 retrotransposition Hum Mol Genet 2000 9 653 657 10.1093/hmg/9.4.653 10699189

[B58] Moran JV DeBerardinis RJ Kazazian HH Jr Exon shuffling by L1 retrotransposition Science 1999 283 1530 1534 10.1126/science.283.5407.1530 10066175

[B59] Boeke JD Pickeral OK Retroshuffling the genomic deck Nature 1999 398 108 9 10.1038/18118 10086353

[B60] Xing J Wang H Belancio VP Cordaux R Deininger PL Batzer MA Emergence of primate genes by retrotransposon-mediated sequence transduction Proc Natl Acad Sci USA 2006 103 17608 17613 10.1073/pnas.0603224103 17101974 PMC1693794

[B61] Buzdin A Ustyugova S Gogvadze E Vinogradova T Lebedev Y Sverdlov E A new family of chimeric retrotranscripts formed by a full copy of U6 small nuclear RNA fused to the 3' terminus of l1 Genomics 2002 80 402 406 10.1006/geno.2002.6843 12376094

[B62] Buzdin AA Retroelements and formation of chimeric retrogenes Cell Mol Life Sci 2004 61 2046 2059 10.1007/s00018-004-4041-z 15316654 PMC11138840

[B63] Buzdin A Gogvadze E Kovalskaya E Volchkov P Ustyugova S Illarionova A The human genome contains many types of chimeric retrogenes generated through in vivo RNA recombination Nucleic Acids Res 2003 31 4385 4390 10.1093/nar/gkg496 12888497 PMC169886

[B64] Gilbert N Lutz-Prigge S Moran JV Genomic deletions created upon LINE-1 retrotransposition Cell 2002 110 315 325 10.1016/S0092-8674(02)00828-0 12176319

[B65] Han K Sen SK Wang J Callinan PA Lee J Cordaux R Genomic rearrangements by LINE-1 insertion-mediated deletion in the human and chimpanzee lineages Nucleic Acids Res 2005 33 4040 4052 10.1093/nar/gki718 16034026 PMC1179734

[B66] Callinan PA Wang J Herke SW Garber RK Liang P Batzer MA Alu retrotransposition-mediated deletion J Mol Biol 2005 348 791 800 10.1016/j.jmb.2005.02.043 15843013

[B67] Roy-Engel AM Carroll ML El-Sawy M Salem AH Garber RK Nguyen SV Non-traditional Alu evolution and primate genomic diversity J Mol Biol 2002 316 1033 1040 10.1006/jmbi.2001.5380 11884141

[B68] Vincent BJ Myers JS Ho HJ Kilroy GE Walker JA Watkins WS Following the LINEs: an analysis of primate genomic variation at human-specific LINE-1 insertion sites Mol Biol Evol 2003 20 1338 1348 10.1093/molbev/msg146 12777507

[B69] Ostertag EM Kazazian HH Jr Twin priming: a proposed mechanism for the creation of inversions in L1 retrotransposition Genome Res 2001 11 2059 2065 10.1101/gr.205701 11731496 PMC311219

[B70] Bergman CM Quesneville H Discovering and detecting transposable elements in genome sequences Brief Bioinform 2007 8 382 392 10.1093/bib/bbm048 17932080

[B71] Wang J Song L Grover D Azrak S Batzer MA Liang P dbRIP: a highly integrated database of retrotransposon insertion polymorphisms in humans Hum Mutat 2006 27 323 329 10.1002/humu.20307 16511833 PMC1855216

[B72] Buzdin A Khodosevich K Mamedov I Vinogradova T Lebedev Y Hunsmann G A technique for genome-wide identification of differences in the interspersed repeats integrations between closely related genomes and its application to detection of human-specific integrations of HERV-K LTRs Genomics 2002 79 413 422 10.1006/geno.2002.6705 11863371

[B73] Mamedov I Batrak A Buzdin A Arzumanyan E Lebedev Y Sverdlov ED Genome-wide comparison of differences in the integration sites of interspersed repeats between closely related genomes Nucleic Acids Res 2002 30 e71 10.1093/nar/gnf071 12136119 PMC135772

[B74] Mamedov IZ Arzumanyan ES Amosova AL Lebedev YB Sverdlov ED Whole-genome experimental identification of insertion/deletion polymorphisms of interspersed repeats by a new general approach Nucleic Acids Res 2005 33 e16 10.1093/nar/gni018 15673711 PMC548376

[B75] Wang J Song L Gonder MK Azrak S Ray DA Batzer MA Whole genome computational comparative genomics: A fruitful approach for ascertaining Alu insertion polymorphisms Gene 2006 365 11 20 10.1016/j.gene.2005.09.031 16376498 PMC1847407

[B76] Konkel MK Wang J Liang P Batzer MA Identification and characterization of novel polymorphic LINE-1 insertions through comparison of two human genome sequence assemblies Gene 2007 390 28 38 10.1016/j.gene.2006.07.040 17034961

[B77] Han K Lee J Meyer TJ Wang J Sen SK Srikanta D Alu recombination-mediated structural deletions in the chimpanzee genome PLoS Genet 2007 3 1939 1949 10.1371/journal.pgen.0030184 17953488 PMC2041999

[B78] Han K Lee J Meyer TJ Remedios P Goodwin L Batzer MA L1 recombination-associated deletions generate human genomic variation Proc Natl Acad Sci USA 2008 105 19366 19371 10.1073/pnas.0807866105 19036926 PMC2614767

[B79] Bennett EA Coleman LE Tsui C Pittard WS Devine SE Natural genetic variation caused by transposable elements in humans Genetics 2004 168 933 951 10.1534/genetics.104.031757 15514065 PMC1448813

[B80] Mills RE Bennett EA Iskow RC Luttig CT Tsui C Pittard WS Recently mobilized transposons in the human and chimpanzee genomes Am J Hum Genet 2006 78 671 679 10.1086/501028 16532396 PMC1424692

[B81] Xing J Zhang Y Han K Salem AH Sen SK Huff CD Mobile elements create structural variation: analysis of a complete human genome Genome Res 2009 19 1516 1526 10.1101/gr.091827.109 19439515 PMC2752133

