# Supplementary material for: RISCI - Repeat Induced Sequence Changes Identifier: a comprehensive, comparative genomics-based, in silico subtractive hybridization pipeline to identify repeat induced sequence changes in closely related genomes
Source: BMC Bioinformatics. 2010 Dec 26;11:609. doi: 10.1186/1471-2105-11-609 (PMC3024322; doi:10.1186/1471-2105-11-609)
Supplement: Additional file 5 — RepeatMasker annotations of recombined loci for truncated L1HS. RepeatMasker annotations of the repeat locus and its flanks in the reference genome and of the identified ortholog and its flanks in the comparative genomes to identify putative regions of homology where recombination takes place. [file 1471-2105-11-609-S5.DOC]

**Additional file 5 - Recombination mediated deletions – Truncated L1HS elements Reference human genome comparison with Chimpanzee, Celera and HuRef genomes.**

**Note that premasked files were available only for reference human and Chmpanzee. These were directly parsed to extract repeat masker annotation of the repeat locus in the reference human genome and of the ortholog in the comparative genome. However, for Celera and HuRef genomes, the orthlog and flanking sequence was retrieved and annotated by RepeatMasker online.**

Repeat masker annotation of the locus in the main genome and its flanks is listed first, followed by the Repeat masker annotation of the identified ortholog in the comparative genome and its flanks.

R1,R2 – PUTATIVE REGIONS OF HOMOLOGY WHICH RECOMBINE TO GIVE R12 (M_INTER_RMD or C_INTER_RMD).

In case of disruptions, R1 and R2 represent disrupted halves of the parent repeat, R represents undisrupted repeat and D represents the disruptive sequence. (M_DISRUPTED OR C_DISRUPTED).

In case of intra element recombination (M_INTRA_RMD or C_INTRA_RMD), R12 represent the 2 resulting copies arising out of intra element recombination and R represents the intact repeat.

**Reference human Vs Chimpanzee**

**L1HS_1_13 56327899-56330505 M_INTRA_RMD**

**56324539 56325159 + L1M5 LINE/L1 5098 5727**

**56325173 56325278 + L1ME3B LINE/L1 4600 4701**

**56325279 56325647 + L1PA11 LINE/L1 5794 6173**

**56325648 56325687 + L1ME3B LINE/L1 4702 4740**

**56325718 56325808 + L1ME3B LINE/L1 5755 5857**

**56325809 56326103 C AluSp SINE/Alu 297 1**

**56326104 56326302 + L1ME3B LINE/L1 5858 6096**

**56326396 56327164 C L1MD2 LINE/L1 1521 651**

**56327223 56327517 + AluSx SINE/Alu 1 296**

**56327575 56327876 + AluSx SINE/Alu 1 303**

**56327899 56330505 + L1HS LINE/L1 2 2607 ( L1HS_1_13 ) (R12)**

**56330507 56331226 + L1PA3 LINE/L1 5569 6288 (R12)**

**56331404 56331500 C L1MD2 LINE/L1 408 313**

**56331720 56332173 + LTR16C LTR/ERVL 11 488**

**56332228 56332410 + MIRb SINE/MIR 8 204**

**56332849 56332932 C MIR3 SINE/MIR 197 116**

**56332961 56333266 + L3 LINE/CR1 4144 4486**

**56333626 56334033 + LTR16B LTR/ERVL 39 452**

**56334514 56334934 C MLT1F LTR/MaLR 479 3**

**Ortholog in Chimp 57100076-57106538 Plus Nscore 0.34**

**N positions 57103775-57103784; 57103806-57103807; 57105481-57105490;**

**57096695 57097335 + L1M5 LINE/L1 5074 5727**

**57097349 57097454 + L1ME3B LINE/L1 4600 4701**

**57097455 57097823 + L1PA11 LINE/L1 5794 6173**

**57097824 57097863 + L1ME3B LINE/L1 4702 4740**

**57097894 57097984 + L1ME3B LINE/L1 5755 5857**

**57097985 57098281 C AluSp SINE/Alu 299 1**

**57098282 57098483 + L1ME3B LINE/L1 5858 6095**

**57098580 57099345 C L1MD2 LINE/L1 1507 651**

**57099404 57099698 + AluSx SINE/Alu 1 296**

**57099756 57100066 + AluSx SINE/Alu 1 312**

**57100076 57105480 + L1Pt LINE/L1 2 5020 (R1,R2)**

**57105501 57107124 + L1PA3 LINE/L1 4515 6155 (R1,R2) Broken due to Ns, overlap at Ns**

**57107152 57107182 + (TAAA)n Simple_repeat 2 32**

**57107193 57107226 + (A)n Simple_repeat 1 34**

**57107227 57107348 C L1MD2 LINE/L1 408 291**

**57107433 57107557 C L1MD2 LINE/L1 408 286**

**57107749 57108201 + LTR16C LTR/ERVL 11 488**

**57108256 57108438 + MIRb SINE/MIR 8 204**

**57108877 57108960 C MIR3 SINE/MIR 197 116**

**57108989 57109294 + L3 LINE/CR1 4144 4486**

**57109654 57110059 + LTR16B LTR/ERVL 39 449**

**57110544 57110963 C MLT1F LTR/MaLR 479 3**

**_________________________________________________________________________**

**L1HS_1_28 73431855-73433278 C_DISRUPTED_M_INTER_RMD**

**73429034 73429396 C THE1B LTR/MaLR 364 1**

**73429397 73429422 C MLT1A0-int LTR/MaLR 1674 1653**

**73429543 73429964 + MLT-int LTR/MaLR 972 1456**

**73429962 73430118 C 7SK RNA 238 59**

**73430124 73430383 + MLT1A0-int LTR/MaLR 1438 1706**

**73430371 73430635 + MLT1A0 LTR/MaLR 97 365**

**73430637 73430937 C MLT1A0-int LTR/MaLR 298 2**

**73430939 73431280 C MLT1A0 LTR/MaLR 365 17**

**73431283 73431337 C MLT1A0 LTR/MaLR 55 1**

**73431443 73431859 + L1ME1 LINE/L1 4261 4694**

**73431855 73433278 + L1HS LINE/L1 1716 3139 ( L1HS_1_28 ) (R12)**

**73433308 73433655 C MLT1E LTR/MaLR 654 284**

**73433752 73434067 C AluY SINE/Alu 310 1**

**73434087 73434147 C MLT1F2 LTR/MaLR 60 1**

**73434149 73434201 + L1MD LINE/L1 4653 4708**

**73434202 73434510 + AluSx SINE/Alu 1 311**

**73434511 73434774 + L1ME1 LINE/L1 4782 5068**

**73434775 73435069 + AluJb SINE/Alu 12 306**

**73435070 73436159 + L1ME1 LINE/L1 5069 6233**

**Ortholog in Chimp 74467496-74470238 Plus Nscore 0.36**

**N positions 74469657-74469666;**

**74464674 74465036 C THE1B LTR/MaLR 364 1**

**74465037 74465062 C MLT1A0-int LTR/MaLR 1674 1653**

**74465182 74465607 + MLT-int LTR/MaLR 967 1456**

**74465605 74465760 C 7SK RNA 238 59**

**74465766 74466025 + MLT1A0-int LTR/MaLR 1438 1706**

**74466013 74466277 + MLT1A0 LTR/MaLR 97 365**

**74466279 74466579 C MLT1A0-int LTR/MaLR 298 2**

**74466581 74466922 C MLT1A0 LTR/MaLR 365 17**

**74466925 74466979 C MLT1A0 LTR/MaLR 55 1**

**74467109 74467500 + L1ME1 LINE/L1 4215 4621**

**74467496 74469656 + L1Pt LINE/L1 1717 3881 (R1)**

**74469639 74470238 + L1P1 LINE/L1 2663 3262 (R2)**

**74470268 74470726 C MLT1E LTR/MaLR 593 79**

**74470727 74471043 C AluY SINE/Alu 311 1**

**74471044 74471123 C MLT1E LTR/MaLR 78 1**

**74471125 74471177 + L1MD LINE/L1 4653 4704**

**74471178 74471486 + AluSx SINE/Alu 1 309**

**74471487 74471749 + L1ME1 LINE/L1 4704 4995**

**74471750 74472044 + AluJb SINE/Alu 12 306**

**74472045 74473135 + L1ME1 LINE/L1 4996 6160**

**_________________________________________________________________________**

**L1HS_1_40c 90456915-90457988 C_DISRUPTED_M_INTER_RMD**

**90450702 90451006 C AluSx SINE/Alu 298 1**

**90451007 90452796 C L1MEc LINE/L1 1892 16**

**90452840 90456910 C L1PA3 LINE/L1 6184 2110**

**90456915 90457988 C L1HS LINE/L1 1082 5 ( L1HS_1_40c ) (R12)**

**90458978 90459074 C MIR3 SINE/MIR 154 57**

**90460075 90460172 C MER91A DNA/Tip100 129 33**

**90460579 90460648 + MIR SINE/MIR 2 70**

**90460636 90461034 + L2 LINE/L2 2529 2958**

**90461042 90461887 C MER21B LTR/ERV1 862 15**

**90461888 90461917 + (CATATA)n Simple_repeat 4 33**

**90461933 90462220 + L2 LINE/L2 2960 3268**

**90462261 90462541 C AluSx SINE/Alu 307 27**

**90462585 90462952 + THE1B LTR/MaLR 3 364**

**90462953 90464421 + THE1B-int LTR/MaLR 1 1516**

**Ortholog in Chimp 91675231-91676492 Minus Nscore 0.79**

**N positions 91676234-91676243;**

**91668377 91669250 C L1MEc LINE/L1 2691 1893**

**91669251 91669560 C AluSx SINE/Alu 300 1**

**91669561 91671350 C L1MEc LINE/L1 1892 16**

**91671435 91675226 C L1PA3 LINE/L1 6139 2110**

**91675231 91676233 C L1Pt LINE/L1 1083 76 (R2)**

**91676244 91676492 C L1Pt LINE/L1 255 5 (R1)**

**91677482 91677578 C MIR3 SINE/MIR 154 57**

**91678579 91678669 C MER91 DNA/Tip100 129 38**

**91679083 91679152 + MIR SINE/MIR 2 70**

**91679140 91679538 + L2 LINE/L2 2529 2958**

**91679546 91680380 C MER21B LTR/ERV1 862 27**

**91680381 91680405 + (TA)n Simple_repeat 1 25**

**91680406 91680443 + (CA)n Simple_repeat 2 40**

**91680460 91680742 + L2 LINE/L2 2960 3268**

**91680783 91680859 C Alu SINE/Alu 295 221**

**91681595 91683042 + THE1B-int LTR/MaLR 18 1516**

**_________________________________________________________________________**

**L1HS_1_45 93747746-93749829 M_DISRUPTED**

**93743360 93743922 C LTR10C LTR/ERV1 591 1**

**93743923 93744121 C AluSg SINE/Alu 199 1**

**93744237 93744287 + AT_rich Low_complexity 1 51**

**93745202 93745253 + (CA)n Simple_repeat 2 53**

**93745254 93745315 + (TC)n Simple_repeat 1 62**

**93745794 93746093 C AluSx SINE/Alu 300 1**

**93746097 93746119 + AT_rich Low_complexity 1 23**

**93746212 93746497 + AluSx SINE/Alu 1 286**

**93746894 93747189 C MLT1K LTR/MaLR 591 233**

**93747275 93747369 C MLT1I LTR/MaLR 409 315**

**93747746 93749829 + L1HS LINE/L1 4 2093 ( L1HS_1_45 ) (R1)**

**93749830 93750133 + AluYa5 SINE/Alu 1 304 (D)**

**93750134 93754072 + L1PA3 LINE/L1 2094 6032 (R2)**

**93754420 93754494 C tRNA-Cys-TGY tRNA 74 1**

**93754679 93754863 + AluSq SINE/Alu 1 195**

**93755132 93755172 + CT-rich Low_complexity 3 43**

**93755174 93755455 C AluSx SINE/Alu 311 35**

**93755467 93755635 C FRAM SINE/Alu 166 1**

**93755712 93755812 C MER58A DNA/MER1_type 141 39**

**93755993 93756176 + MIRb SINE/MIR 50 257**

**Ortholog in Chimp 95008405-95010448 Plus Nscore 0.59 (94008405-95015146 - Nscore 17.87)**

**N positions 95008965-95008965; 95008975-95008975; 95009015-95009024;**

**95003576 95004138 C LTR10C LTR/ERV1 591 1**

**95004139 95004337 C AluSg SINE/Alu 199 1**

**95004453 95004503 + AT_rich Low_complexity 1 51**

**95005414 95005463 + (CA)n Simple_repeat 2 51**

**95005464 95005497 + (TC)n Simple_repeat 1 34**

**95005976 95006275 C AluSx SINE/Alu 300 1**

**95006976 95007162 + AluSg/x SINE/Alu 108 293**

**95007552 95007847 C MLT1K LTR/MaLR 591 233**

**95008405 95012316 + L1Pt LINE/L1 4 3983 (R) (BROKEN BECAUSE OF Ns)**

**95013531 95015146 + L1PA3 LINE/L1 4396 6032 (R)**

**95015493 95015567 C tRNA-Cys-TGY tRNA 74 1**

**95015752 95015936 + AluSq SINE/Alu 1 195**

**95016229 95016273 + CT-rich Low_complexity 2 45**

**95016274 95016555 C AluSx SINE/Alu 311 35**

**95016567 95016735 C FRAM SINE/Alu 166 1**

**95016812 95016912 C MER58A DNA/MER1_type 141 39**

**95017093 95017276 + MIRb SINE/MIR 50 257**

**_________________________________________________________________________**

**L1HS_1_48 98826806-98830180 M_INTRA_RMD**

**98820690 98820880 C L1P LINE/L1 5319 5130**

**98820881 98820924 + (TG)n Simple_repeat 1 44**

**98820925 98821635 C L1P LINE/L1 5129 4407**

**98821641 98821891 + Tigger2 DNA/MER2_type 87 343**

**98821892 98824268 + L1PA10 LINE/L1 3372 5788**

**98824269 98824578 + AluSg SINE/Alu 1 306**

**98824579 98824964 + L1PA10 LINE/L1 5789 6167**

**98824973 98825717 + Tigger2 DNA/MER2_type 339 337**

**98825718 98826033 + AluJo SINE/Alu 1 304**

**98826034 98826411 + Tigger2 DNA/MER2_type 338 694**

**98826412 98826702 C AluJo SINE/Alu 285 1**

**98826703 98826795 + Tigger2 DNA/MER2_type 695 786**

**98826806 98830180 + L1HS LINE/L1 12 3389 ( L1HS_1_48 )**

**98830177 98831188 + L1PA2 LINE/L1 5146 6155**

**98831196 98832387 + Tigger2 DNA/MER2_type 775 2718**

**98832388 98832434 + MLT2B1 LTR/ERVL 521 567**

**98832435 98832870 C L1MD LINE/L1 2863 2429**

**98832903 98833632 C L1MA3 LINE/L1 6305 5592**

**98833633 98833939 C AluSx SINE/Alu 306 1**

**98833940 98834354 C L1P4 LINE/L1 5607 5199**

**98835133 98835466 + L1M5 LINE/L1 4425 4739**

**98835472 98835563 + (TA)n Simple_repeat 1 91**

**98836008 98836123 + MLT1I LTR/MaLR 12 131**

**98836256 98836469 C MIR SINE/MIR 237 7**

**98836484 98836834 + MLT1A0 LTR/MaLR 1 365**

**98838327 98838685 C MSTD LTR/MaLR 396 1**

**98839138 98839165 + AT_rich Low_complexity 1 28**

**Ortholog in Chimp 100150551-100158165 Plus Nscore 9.51**

**N positions 100151331-100151340; 100153307-100154020;**

**100145534 100145563 + (TG)n Simple_repeat 1 30**

**100145564 100146250 C L1P LINE/L1 5126 4429**

**100146251 100146528 + Tigger2 DNA/MER2_type 66 343**

**100146529 100147954 + L1P3 LINE/L1 3372 4802**

**100149224 100149455 + Tigger2 DNA/MER2_type 107 330**

**100149456 100149776 + AluJo SINE/Alu 1 308**

**100149777 100150155 + Tigger2 DNA/MER2_type 331 694**

**100150156 100150446 C AluJo SINE/Alu 285 2**

**100150447 100150540 + Tigger2 DNA/MER2_type 695 786**

**100150552 100151330 + L1Pt LINE/L1 13 792 (R1)**

**100151341 100151987 + L1PA11 LINE/L1 5135 5788 (D)**

**100151988 100152303 + AluSg SINE/Alu 1 302 (D)**

**100152304 100152692 + L1PA11 LINE/L1 5789 6172 (D)**

**100152701 100153302 + Tigger2 DNA/MER2_type 339 195 (D)**

**100154021 100159175 + L1PA2 LINE/L1 880 6032 (R2)**

**100159181 100160374 + Tigger2 DNA/MER2_type 775 2718**

**100160375 100160421 + MLT2B1 LTR/ERVL 521 567**

**100160422 100160857 C L1MD LINE/L1 2863 2431**

**100160892 100161599 C L1MA3 LINE/L1 6305 5592**

**100161620 100161926 C AluSx SINE/Alu 306 1**

**100161927 100162343 C L1MA3 LINE/L1 5607 5199**

**100162787 100162816 + AT_rich Low_complexity 1 30**

**100163121 100163265 + L1M5 LINE/L1 4425 4572**

**100163266 100163295 + (TA)n Simple_repeat 2 31**

**100163296 100163458 + L1M5 LINE/L1 4573 4738**

**100163464 100163555 + (TA)n Simple_repeat 1 91**

**100163989 100164114 + MLT1I LTR/MaLR 1 131**

**100164247 100164460 C MIR SINE/MIR 237 7**

**100164475 100164824 + MLT1A0 LTR/MaLR 1 365**

**100166328 100166686 C MSTD LTR/MaLR 396 1**

**100167138 100167165 + AT_rich Low_complexity 1 28**

**_________________________________________________________________________**

**L1HS_1_61 106769783-106770468 C_INTER_RMD_M_DISRUPTED**

**106760330 106761102 C L2 LINE/L2 2398 1467**

**106761926 106762021 C LTR52 LTR/ERVL 147 49**

**106762031 106765236 + L1MA2 LINE/L1 17 3174**

**106765237 106765275 + (TTA)n Simple_repeat 2 40**

**106765276 106765301 + L1MA2 LINE/L1 3175 3178**

**106765302 106765324 + (TG)n Simple_repeat 2 24**

**106765325 106765526 + L1MA2 LINE/L1 3179 3343**

**106765527 106765878 + L1PA10 LINE/L1 5794 6152**

**106765879 106766113 + L1MA2 LINE/L1 3344 3523**

**106766108 106766884 + L1MA2 LINE/L1 4199 5166**

**106766885 106767174 + AluY SINE/Alu 7 296**

**106767175 106769661 + L1MA2 LINE/L1 5167 7671**

**106769665 106769782 + AluSp SINE/Alu 1 120**

**106769783 106770468 + L1HS LINE/L1 5477 6155 ( L1HS_1_61 ) (R1)**

**106770520 106770543 + (TAAAA)n Simple_repeat 2 25**

**106770564 106770944 + L1PA5 LINE/L1 5770 6151 (R2)**

**106770950 106771074 + AluSg/x SINE/Alu 130 254**

**106771075 106771353 + (TA)n Simple_repeat 1 277**

**106771354 106771793 + THE1B-int LTR/MaLR 1146 1580**

**106771794 106772146 + THE1B LTR/MaLR 1 364**

**106772194 106773238 C L1MEc LINE/L1 2475 1393**

**106773271 106773296 + AT_rich Low_complexity 1 26**

**106773380 106773743 C THE1B LTR/MaLR 364 1**

**106774718 106775121 + MLT2A2 LTR/ERVL 1 413**

**106775127 106775270 + (TA)n Simple_repeat 2 155**

**106775287 106775591 + (TA)n Simple_repeat 1 334**

**106775679 106776246 C MLT1G LTR/MaLR 595 2**

**106776399 106776484 + AluSc SINE/Alu 212 297**

**106776486 106777147 C L2 LINE/L2 3095 2367**

**106777875 106777909 + AT_rich Low_complexity 1 35**

**106778027 106778274 + MIR SINE/MIR 7 241**

**106778658 106778848 C MIRb SINE/MIR 239 47**

**106779103 106779392 + AluSx SINE/Alu 1 290**

**106779424 106779833 C MER57A LTR/ERV1 403 3**

**Ortholog in Chimp 108095368-108095651 Plus Nscore 0.00**

**N positions**

**108085925 108086697 C L2 LINE/L2 2398 1467**

**108087521 108087616 C LTR52 LTR/ERVL 147 49**

**108087626 108090828 + L1MA3 LINE/L1 17 3174**

**108090829 108090861 + (TTA)n Simple_repeat 2 34**

**108090862 108090887 + L1MA3 LINE/L1 3175 3178**

**108090888 108090916 + (TG)n Simple_repeat 2 30**

**108090917 108091118 + L1MA3 LINE/L1 3179 3343**

**108091119 108091466 + L1PA10 LINE/L1 5794 6152**

**108091467 108091700 + L1MA3 LINE/L1 3344 3523**

**108091695 108092470 + L1MA3 LINE/L1 4199 5165**

**108092471 108092761 + AluY SINE/Alu 1 292**

**108092762 108095246 + L1MA3 LINE/L1 5166 7671**

**108095254 108095367 + AluSp SINE/Alu 5 120**

**108095368 108096033 + L1PA5 LINE/L1 5477 6152 (R12)**

**108096034 108096161 + AluSp SINE/Alu 121 252**

**108096162 108096283 + (TA)n Simple_repeat 1 121**

**108096288 108096733 + THE1B-int LTR/MaLR 1139 1580**

**108096734 108097084 + THE1B LTR/MaLR 1 364**

**108097132 108098114 C L1MEc LINE/L1 2475 1462**

**108098213 108098238 + AT_rich Low_complexity 1 26**

**108098322 108098684 C THE1B LTR/MaLR 364 1**

**108099657 108100050 + MLT2A2 LTR/ERVL 1 399**

**108100062 108100390 + (TA)n Simple_repeat 1 357**

**108100396 108100479 + (TTATA)n Simple_repeat 1 84**

**108100480 108100656 + (TATAA)n Simple_repeat 1 179**

**108100673 108100818 + (TATAA)n Simple_repeat 4 159**

**108100819 108100845 + AT_rich Low_complexity 1 27**

**108100846 108100900 + MLT2A2 LTR/ERVL 506 560**

**108100912 108100960 + AT_rich Low_complexity 1 49**

**108100973 108101124 + L1P3 LINE/L1 4890 5032**

**108101601 108102167 C MLT1G LTR/MaLR 595 2**

**108102320 108102405 + AluSc SINE/Alu 212 297**

**108102407 108103099 C L2 LINE/L2 3095 2322**

**108103795 108103829 + AT_rich Low_complexity 1 35**

**108103941 108104229 + MIR SINE/MIR 5 272**

**108104557 108104765 C MIRb SINE/MIR 260 47**

**_________________________________________________________________________**

**L1HS_1_63 113685753-113689935 C_INTER_RMD_M_DISRUPTED**

**113676749 113676954 + MIRb SINE/MIR 2 232**

**113677097 113677430 + L2 LINE/L2 3054 3378**

**113679033 113679160 + L1M5 LINE/L1 5691 5823**

**113679198 113680003 C Tigger1 DNA/MER2_type 2418 1589**

**113680004 113680303 C AluSp SINE/Alu 304 2**

**113680304 113681904 C Tigger1 DNA/MER2_type 1588 1**

**113682091 113682392 C AluSp SINE/Alu 299 1**

**113683352 113683405 + (TG)n Simple_repeat 2 55**

**113683409 113683483 + L2 LINE/L2 3301 3374**

**113683523 113683577 + AluSx SINE/Alu 245 299**

**113683579 113684447 + L1MB2 LINE/L1 5230 6135**

**113685480 113685748 C L2 LINE/L2 3352 3083**

**113685753 113689935 + L1HS LINE/L1 1995 6155 ( L1HS_1_63 ) (R1)**

**113689938 113690958 + L1PA4 LINE/L1 5128 6155 (R2)**

**113690974 113691114 C L2 LINE/L2 3061 2910**

**113691263 113691538 C AluSg SINE/Alu 303 11**

**113691591 113691900 C AluJo SINE/Alu 301 1**

**113692262 113692340 + MIR SINE/MIR 1 78**

**113692671 113692720 + (TGGA)n Simple_repeat 4 53**

**113693058 113693159 + AluSg/x SINE/Alu 135 236**

**113693161 113693323 + MIR SINE/MIR 50 231**

**113693398 113693651 C L2 LINE/L2 2964 2711**

**113693652 113693960 C AluY SINE/Alu 306 1**

**113695067 113695163 C MIRm SINE/MIR 255 157**

**113696363 113696683 + MER44A DNA/MER2_type 1 336**

**113697350 113697992 C L1MD3 LINE/L1 7995 7346**

**113698870 113699175 + AluJb SINE/Alu 1 308**

**Ortholog in Chimp 124346587-124349743 Minus Nscore 0.00**

**N positions**

**124337354 124337657 C AluJb SINE/Alu 308 1**

**124338535 124339172 + L1MD3 LINE/L1 7346 7995**

**124339839 124340159 C MER44A DNA/MER2_type 336 1**

**124341353 124341441 + L2 LINE/L2 3252 3341**

**124342563 124342869 + AluY SINE/Alu 1 304**

**124342870 124343150 + L2 LINE/L2 2711 3008**

**124343182 124343373 C MIRb SINE/MIR 273 57**

**124343375 124343476 C AluSg/x SINE/Alu 236 135**

**124343814 124343863 + (TCCA)n Simple_repeat 4 53**

**124344194 124344272 C MIR SINE/MIR 78 1**

**124344634 124344941 + AluJo SINE/Alu 1 301**

**124344994 124345260 + AluSg SINE/Alu 11 294**

**124345406 124345554 + L2 LINE/L2 2907 3066**

**124345565 124349744 C L1PA4 LINE/L1 6155 1995 (R12)**

**124349749 124350017 + L2 LINE/L2 3083 3352**

**124351050 124351918 C L1MB2 LINE/L1 6135 5230**

**124351920 124351974 C Alu SINE/Alu 299 245**

**124352014 124352088 C L2 LINE/L2 3374 3301**

**124352092 124352129 + (CA)n Simple_repeat 2 39**

**124353091 124353394 + AluSp SINE/Alu 1 300**

**124353581 124355008 + Tigger1 DNA/MER2_type 1 1415**

**124355432 124355508 + Tigger1 DNA/MER2_type 1513 1588**

**124355509 124355809 + AluSp SINE/Alu 1 304**

**124355810 124356615 + Tigger1 DNA/MER2_type 1589 2418**

**124356653 124356780 C L1M5 LINE/L1 5823 5691**

**124358386 124358717 C L2 LINE/L2 3378 3054**

**_________________________________________________________________________**

**L1HS_2_3 2240463-2242344 C_INTER_RMD_M_DISRUPTED**

**2229796 2233590 + L1MA2 LINE/L1 2111 6304**

**2233674 2233737 + AT_rich Low_complexity 1 64**

**2234613 2234727 + GA-rich Low_complexity 2 117**

**2235741 2235764 + AT_rich Low_complexity 1 24**

**2235900 2235946 + A-rich Low_complexity 1 47**

**2237303 2237403 + MIR SINE/MIR 158 262**

**2239124 2239340 + MER58A DNA/MER1_type 1 224**

**2239801 2239947 C MER5A DNA/MER1_type 157 6**

**2240183 2240247 + Alu SINE/Alu 9 73**

**2240253 2240432 + L1MA9 LINE/L1 6004 6183**

**2240424 2240473 C L1HS LINE/L1 4431 4384**

**2240463 2242344 + L1HS LINE/L1 4432 6312 ( L1HS_2_3 )**

**2242534 2242730 + MIRb SINE/MIR 32 220**

**2242954 2243086 + MIR SINE/MIR 60 192**

**2243902 2243953 + (TG)n Simple_repeat 2 53**

**2244882 2244964 + L2 LINE/L2 3336 3419**

**2245110 2245165 + AluYc3 SINE/Alu 238 293**

**2245166 2245178 + AluYc3 SINE/Alu 272 283**

**2245190 2245488 + AluY SINE/Alu 1 298**

**2245695 2245734 + (TA)n Simple_repeat 2 41**

**2246687 2246744 + L1PA5 LINE/L1 6092 6150**

**2246809 2246924 C MIRb SINE/MIR 146 16**

**2247708 2247800 + MIRb SINE/MIR 26 120**

**2247801 2248086 C AluSq SINE/Alu 304 1**

**2248087 2248230 + MIRb SINE/MIR 121 267**

**2248429 2248564 + MIR SINE/MIR 84 237**

**2248842 2249077 C MIRb SINE/MIR 238 2**

**2249410 2249769 + THE1B LTR/MaLR 1 364**

**2249993 2250209 C MIR SINE/MIR 232 21**

**Ortholog in Chimp 2221514-2221647 Plus Nscore 0.00**

**N positions**

**2212489 2212614 + GA-rich Low_complexity 2 129**

**2213260 2213392 + GA-rich Low_complexity 2 137**

**2214405 2214427 + AT_rich Low_complexity 1 23**

**2214563 2214610 + (A)n Simple_repeat 1 45**

**2215936 2216036 + MIR SINE/MIR 158 262**

**2216412 2216463 C L4 LINE/RTE 1948 1897**

**2217756 2217972 + MER58A DNA/MER1_type 1 224**

**2218433 2218579 C MER5A DNA/MER1_type 157 6**

**2218815 2219107 + AluSc SINE/Alu 9 300**

**2219707 2219850 C AluSc SINE/Alu 309 166**

**2219852 2220599 + L1PA7 LINE/L1 5405 6154**

**2221069 2221647 + L1MA9 LINE/L1 5730 6312**

**2221837 2222033 + MIRb SINE/MIR 32 220**

**2222248 2222453 + MIR_Mars SINE/MIR 57 260**

**2223202 2223251 + (TG)n Simple_repeat 2 51**

**2223958 2223978 + AT_rich Low_complexity 1 21**

**2224143 2224262 + L2 LINE/L2 3307 3419**

**2224408 2224463 + Alu SINE/Alu 238 293**

**2224464 2224480 + Alu SINE/Alu 272 287**

**2224484 2224783 + AluY SINE/Alu 1 300**

**2225985 2226042 + L1PA5 LINE/L1 6092 6150**

**2226107 2226222 C MIRb SINE/MIR 146 16**

**2227007 2227099 + MIRb SINE/MIR 26 116**

**2227100 2227380 C AluSq SINE/Alu 299 1**

**2227381 2227525 + MIRb SINE/MIR 117 268**

**2227724 2227859 + MIR SINE/MIR 84 237**

**2228137 2228372 C MIRb SINE/MIR 238 2**

**2228705 2229064 + THE1B LTR/MaLR 1 364**

**2229288 2229504 C MIR SINE/MIR 232 21**

**_________________________________________________________________________**

**L1HS_2_15 49162749-49166150 C_INTRA_RMD**

**49156575 49156823 + L1ME3B LINE/L1 3350 3590**

**49156824 49157178 C THE1B LTR/MaLR 364 1**

**49157179 49158723 C MLT-int LTR/MaLR 1735 2**

**49158726 49159024 C THE1B LTR/MaLR 364 71**

**49159046 49161311 + L1ME3B LINE/L1 3588 5946**

**49161312 49161877 + MER74A LTR/ERVL 1 558**

**49161878 49161951 + L1ME3B LINE/L1 5947 6028**

**49162657 49162754 + L1HS LINE/L1 1 98**

**49162749 49166150 + L1HS LINE/L1 615 4029 ( L1HS_2_15 ) (R1,R2)**

**49166149 49166192 C L1P1 LINE/L1 2747 2704**

**49166193 49166764 + L1PA4 LINE/L1 5578 6150**

**49166828 49167075 + Charlie8 DNA/MER1_type 19 271**

**49167162 49167270 C MER5B DNA/MER1_type 178 66**

**49167271 49167576 C AluSq SINE/Alu 312 1**

**49167577 49167641 C MER5B DNA/MER1_type 65 1**

**49167714 49167741 + (T)n Simple_repeat 1 28**

**49168062 49168204 C L2 LINE/L2 3419 3267**

**49168414 49168584 C L2 LINE/L2 3419 3239**

**49168595 49168810 C MIRb SINE/MIR 249 30**

**49169289 49169348 + L3 LINE/CR1 3825 3884**

**49169737 49169757 + (TATAA)n Simple_repeat 3 23**

**49170633 49170717 C MIRb SINE/MIR 261 177**

**49170768 49170809 + (TG)n Simple_repeat 2 43**

**49170812 49171959 C L1ME1 LINE/L1 6110 4951**

**49171960 49172239 C AluJb SINE/Alu 287 1**

**Ortholog in Chimp 50257934-50261219 Plus Nscore 0.30**

**N positions 50258721-50258730;**

**50251790 50252034 + L1ME3B LINE/L1 3349 3589**

**50252036 50252393 C THE1B LTR/MaLR 364 1**

**50252394 50253933 C MLT-int LTR/MaLR 1735 2**

**50253936 50254234 C THE1B LTR/MaLR 364 71**

**50254385 50256495 + L1ME3B LINE/L1 3764 5944**

**50256496 50257061 + MER74A LTR/ERVL 1 558**

**50257062 50257135 + L1ME3B LINE/L1 5945 6028**

**50257842 50257939 + L1Pt LINE/L1 1 98**

**50257909 50261219 + L1Pt LINE/L1 579 4029 (R12) (NOTE THAT THE LENGTH IS SMALLER THAN THE HUMAN ORTHOLOG)**

**50261218 50261261 C L1P1 LINE/L1 2747 2704**

**50261262 50261834 + L1PA4 LINE/L1 5578 6150**

**50261917 50262145 + Charlie8 DNA/MER1_type 28 260**

**50262238 50262346 C MER5B DNA/MER1_type 178 66**

**50262347 50262668 C AluSx SINE/Alu 312 1**

**50262669 50262733 C MER5B DNA/MER1_type 65 1**

**50262808 50262845 + (T)n Simple_repeat 1 38**

**50263167 50263309 C L2 LINE/L2 3419 3267**

**50263519 50263689 C L2 LINE/L2 3419 3239**

**50263700 50263915 C MIRb SINE/MIR 249 30**

**50264394 50264453 + L3 LINE/CR1 3825 3884**

**50264842 50264867 + AT_rich Low_complexity 1 26**

**50265727 50265811 C MIRb SINE/MIR 261 177**

**50265862 50265885 + (TG)n Simple_repeat 2 25**

**50265888 50267027 C L1ME1 LINE/L1 6110 4951**

**50267028 50267308 C AluJb SINE/Alu 288 1**

**_________________________________________________________________________**

**L1HS_3_7c 11505147-11508964 C_INTER_RMD_M_DISRUPTED**

**11496128 11496162 + (TAAAAA)n Simple_repeat 2 35**

**11496404 11496704 C AluY SINE/Alu 297 1**

**11496728 11497061 + AluJo SINE/Alu 3 300**

**11497573 11497644 C L2 LINE/L2 3373 3296**

**11497650 11497823 + L1MB3 LINE/L1 6006 6182**

**11498209 11498442 + MIRb SINE/MIR 14 254**

**11499269 11499477 C L1ME3 LINE/L1 6162 5953**

**11500586 11500894 + AluSx SINE/Alu 1 312**

**11500917 11500993 + L3 LINE/CR1 3525 3606**

**11501024 11503297 C L1PA3 LINE/L1 6155 3867 (R2)**

**11503302 11504773 C SVA Other 1386 393 (D)**

**11505147 11508964 C L1HS LINE/L1 3758 1 ( L1HS_3_7c ) (R1)**

**11508967 11510066 C LTR1D LTR/ERV1 978 1**

**11510076 11510511 + L3 LINE/CR1 3635 4178**

**11510512 11510993 C LOR1a LTR/ERV1 497 1**

**11510994 11511265 + L3 LINE/CR1 4179 4432**

**11511266 11511563 + AluSg SINE/Alu 1 292**

**11511564 11511615 + L3 LINE/CR1 4433 4484**

**11512414 11512549 C MIR SINE/MIR 239 99**

**11512878 11512915 + CT-rich Low_complexity 4 40**

**11513322 11513624 C AluSx SINE/Alu 312 1**

**11513704 11513831 + AluSx SINE/Alu 1 128**

**11513832 11513866 + (TA)n Simple_repeat 2 36**

**11513867 11514029 + AluSx SINE/Alu 129 303**

**11514614 11514655 + (CATA)n Simple_repeat 1 44**

**Ortholog in Chimp 11804325-11808145 Minus Nscore 0.00**

**N positions**

**11794987 11795444 C MLT1K LTR/MaLR 587 154**

**11796357 11796399 + (CA)n Simple_repeat 2 44**

**11797012 11797046 + (TAAAAA)n Simple_repeat 2 35**

**11797288 11797601 C AluY SINE/Alu 308 1**

**11797625 11797960 + AluJo SINE/Alu 3 302**

**11798551 11798724 + L1MB3 LINE/L1 6006 6182**

**11799102 11799322 + MIRb SINE/MIR 14 241**

**11800161 11800368 C L1ME3 LINE/L1 6162 5953**

**11801611 11801916 + AluSx SINE/Alu 1 309**

**11801938 11802014 + L3 LINE/CR1 3525 3606**

**11802030 11802057 + (TTTTA)n Simple_repeat 5 32**

**11802064 11808145 C L1PA3 LINE/L1 6140 1 (R12)**

**11808148 11809237 C LTR1D LTR/ERV1 978 1**

**11809247 11809457 + L3_Mars LINE/CR1 3563 3789**

**11809580 11809682 + L3 LINE/CR1 3999 4103**

**11809683 11810163 C LOR1a LTR/ERV1 497 1**

**11810164 11810436 + L3 LINE/CR1 4104 4357**

**11810437 11810734 + AluSg SINE/Alu 2 293**

**11810735 11810786 + L3 LINE/CR1 4358 4409**

**11811603 11811745 C MIR SINE/MIR 216 85**

**11812050 11812086 + CT-rich Low_complexity 1 36**

**11812489 11812791 C AluSx SINE/Alu 312 1**

**11812871 11813179 + AluSx SINE/Alu 1 303**

**11813764 11813801 + (CATA)n Simple_repeat 3 40**

**_________________________________________________________________________**

**L1HS_3_14c 22066888-22069693 C_INTER_RMD_M_DISRUPTED**

**22057719 22057907 C MLT1K LTR/MaLR 273 69**

**22057981 22058465 + L3 LINE/CR1 3748 4243**

**22058647 22058850 + MIR SINE/MIR 35 262**

**22060019 22060131 C L2 LINE/L2 3407 3288**

**22060135 22060437 + MLT1F2 LTR/MaLR 4 323**

**22060447 22060600 C L2 LINE/L2 1957 1803**

**22060937 22060963 + (CAAAAA)n Simple_repeat 4 30**

**22061065 22061330 C L2 LINE/L2 1752 1458**

**22061452 22061522 + AluSp/q SINE/Alu 226 296**

**22061523 22061584 C L1M2 LINE/L1 4326 4264**

**22061616 22061797 C L1M2 LINE/L1 5133 4941**

**22063602 22065510 C L1MA8 LINE/L1 6289 4316**

**22065511 22065836 + AluSq SINE/Alu 1 327**

**22065837 22066576 C L1MA8 LINE/L1 4315 3551**

**22066580 22066854 + AluJo SINE/Alu 1 279**

**22066855 22066887 + (CAAA)n Simple_repeat 1 33**

**22066888 22069693 C L1HS LINE/L1 3437 329 ( L1HS_3_14c )**

**22069692 22072804 + L1HS LINE/L1 3064 6176**

**22073187 22073315 C MLT1H LTR/MaLR 533 406**

**22073404 22073481 C MLT1H2 LTR/MaLR 161 84**

**22073622 22073874 + MIRb SINE/MIR 14 262**

**22074010 22074309 C AluSx SINE/Alu 296 1**

**22074491 22074521 + (TGAA)n Simple_repeat 3 33**

**22075113 22075135 + AT_rich Low_complexity 1 23**

**22075176 22075197 + AT_rich Low_complexity 1 22**

**22075816 22075885 C MER5B DNA/MER1_type 74 4**

**22075963 22076050 C Charlie2 DNA/MER1_type 263 176**

**22076077 22076177 C Charlie2 DNA/MER1_type 110 5**

**22076180 22076259 + L2 LINE/L2 3302 3387**

**22076260 22076829 + MLT2B2 LTR/ERVL 1 515**

**22076830 22076860 + L2 LINE/L2 3388 3419**

**22077195 22077229 + AT_rich Low_complexity 1 35**

**22077403 22077456 + AT_rich Low_complexity 1 54**

**22077613 22077635 + (TTAAA)n Simple_repeat 4 26**

**22077816 22078127 + L2 LINE/L2 2626 2965**

**22078168 22078487 + L2 LINE/L2 3127 3415**

**22078543 22078851 C AluJb SINE/Alu 291 1**

**Ortholog in Chimp 22610755-22611218 Minus Nscore 0.00**

**N positions**

**22601817 22602300 + L3 LINE/CR1 3748 4243**

**22602415 22602639 + MIR SINE/MIR 11 262**

**22603810 22603922 C L2 LINE/L2 3407 3288**

**22603952 22604264 + MLT1F2 LTR/MaLR 4 381**

**22604269 22604416 C L2 LINE/L2 1951 1803**

**22604752 22604778 + (CAAAAA)n Simple_repeat 4 30**

**22604879 22605144 C L2 LINE/L2 1752 1458**

**22605266 22605336 + AluSp/q SINE/Alu 226 296**

**22605337 22605398 C L1M2 LINE/L1 4326 4264**

**22605430 22605609 C L1M3 LINE/L1 5130 4938**

**22607127 22607147 + AT_rich Low_complexity 1 21**

**22607411 22609319 C L1MA8 LINE/L1 6289 4313**

**22609320 22609635 + AluSq SINE/Alu 1 317**

**22609636 22610453 C L1MA8 LINE/L1 4312 3559**

**22610454 22610753 + AluJo SINE/Alu 1 305**

**22610754 22610966 C L1MA8 LINE/L1 3558 3376**

**22611089 22611152 + AT_rich Low_complexity 1 64**

**22611598 22611726 C MLT1H LTR/MaLR 533 406**

**22611815 22611915 C MLT1H2 LTR/MaLR 161 65**

**22612039 22612266 + MIRb SINE/MIR 27 248**

**22612415 22612713 C AluSx SINE/Alu 295 1**

**22612895 22612925 + (TGAA)n Simple_repeat 3 33**

**22613517 22613539 + AT_rich Low_complexity 1 23**

**22613580 22613601 + AT_rich Low_complexity 1 22**

**22614220 22614289 C MER5B DNA/MER1_type 74 4**

**22614367 22614454 C Charlie2 DNA/MER1_type 263 176**

**22614481 22614581 C Charlie2 DNA/MER1_type 110 5**

**22614584 22614663 + L2 LINE/L2 3302 3387**

**22614664 22615233 + MLT2B2 LTR/ERVL 1 515**

**22615234 22615264 + L2 LINE/L2 3388 3419**

**22615599 22615633 + AT_rich Low_complexity 1 35**

**22616196 22616505 + L2 LINE/L2 2626 2965**

**22616549 22616861 + L2 LINE/L2 3130 3415**

**22616917 22617218 C AluJb SINE/Alu 291 1**

**22617446 22617958 + MLT1F1 LTR/MaLR 1 567**

**22618010 22618148 + MIRb SINE/MIR 89 229**

**22618476 22618903 + LTR16A LTR/ERVL 13 450**

**22618912 22619111 C ERVL-B4 LTR/ERVL 930 727**

**22619282 22619776 + L1MA7 LINE/L1 5629 6119**

**22619842 22619965 + MIR SINE/MIR 1 126**

**_________________________________________________________________________**

**L1HS_3_17c 25715952-25718569 C_INTER_RMD_M_DISRUPTED**

**25711370 25711583 C L1PREC2 LINE/L1 4684 4481**

**25711619 25711889 + AluJb SINE/Alu 24 293**

**25712452 25712533 + MIR SINE/MIR 54 138**

**25712627 25712934 C AluJb SINE/Alu 307 1**

**25713087 25713107 + AT_rich Low_complexity 1 21**

**25713308 25713524 C MIRb SINE/MIR 249 10**

**25713621 25713702 + GA-rich Low_complexity 3 81**

**25714741 25715218 C L1PA3 LINE/L1 6154 5677 (R2)**

**25715231 25715252 + AT_rich Low_complexity 1 22**

**25715315 25715468 C ERVL-E LTR/ERVL 5276 5122**

**25715500 25715921 C ERVL-E LTR/ERVL 5032 4590**

**25715952 25718569 C L1HS LINE/L1 6155 3538 ( L1HS_3_17c ) (R1)**

**25720466 25720773 + AluSx SINE/Alu 1 309**

**25721360 25721402 + AT_rich Low_complexity 1 43**

**25721435 25721547 + L1ME4a LINE/L1 5978 6100**

**25721567 25721605 + AT_rich Low_complexity 1 39**

**25721878 25722037 C MIRb SINE/MIR 258 85**

**25722529 25722729 + L2 LINE/L2 3225 3419**

**25723554 25723641 C MIRm SINE/MIR 247 156**

**25723644 25723741 C L2 LINE/L2 1984 1887**

**25723994 25724078 + MIR3 SINE/MIR 70 159**

**25724080 25724260 C MER5A DNA/MER1_type 189 8**

**25725402 25725432 + (T)n Simple_repeat 1 31**

**25725617 25726320 + L1ME3B LINE/L1 5217 5976**

**25726403 25726679 + AluJb SINE/Alu 1 306**

**25726692 25726874 + THE1C LTR/MaLR 1 183**

**25726875 25727157 C AluSx SINE/Alu 295 1**

**25727158 25727346 + THE1C LTR/MaLR 184 375**

**Ortholog in Chimp 26317977-26320115 Minus Nscore 0.00**

**N positions**

**26314128 26314350 C L1PREC2 LINE/L1 4690 4465**

**26314373 26314643 + AluJb SINE/Alu 24 293**

**26315206 26315287 + MIR SINE/MIR 54 138**

**26315382 26315694 C AluJb SINE/Alu 312 1**

**26315847 26315867 + AT_rich Low_complexity 1 21**

**26316068 26316281 C MIRb SINE/MIR 249 14**

**26316381 26316462 + GA-rich Low_complexity 3 84**

**26317500 26320115 C L1PA3 LINE/L1 6154 3538 (R12)**

**26322018 26322321 + AluSx SINE/Alu 1 305**

**26322908 26322950 + AT_rich Low_complexity 1 43**

**26322983 26323095 + L1ME4a LINE/L1 5978 6100**

**26323106 26323153 + AT_rich Low_complexity 1 48**

**26323427 26323585 C MIRb SINE/MIR 257 85**

**26324077 26324277 + L2 LINE/L2 3225 3419**

**26325191 26325288 C L2 LINE/L2 1984 1887**

**26325534 26325625 + MIR3 SINE/MIR 73 159**

**26325627 26325807 C MER5A DNA/MER1_type 189 8**

**26326942 26326963 + (TTTA)n Simple_repeat 2 23**

**26327150 26327856 + L1ME3B LINE/L1 5217 5976**

**26327939 26328219 + AluJb SINE/Alu 1 310**

**26328226 26328408 + THE1C LTR/MaLR 1 182**

**26328409 26328690 C AluSx SINE/Alu 295 2**

**26328691 26328880 + THE1C LTR/MaLR 183 375**

**_________________________________________________________________________**

**L1HS_3_20c 29124607-29128608 M_INTRA_RMD**

**29120364 29120583 C MER58A DNA/MER1_type 222 1**

**29121141 29121161 + AT_rich Low_complexity 1 21**

**29122122 29122328 + MIR SINE/MIR 1 208**

**29122345 29122484 C MER5A1 DNA/MER1_type 146 1**

**29122532 29122576 + (CAT)n Simple_repeat 1 45**

**29122764 29122891 + L2 LINE/L2 2326 2460**

**29123305 29123954 + L1M1 LINE/L1 4307 4955**

**29123955 29123994 + (TC)n Simple_repeat 1 40**

**29123995 29124608 + L1M1 LINE/L1 4956 5607**

**29124607 29128608 C L1HS LINE/L1 4024 2 ( L1HS_3_20c )**

**29128615 29128711 C L1MEc LINE/L1 1546 1450**

**29128779 29129448 C HAL1 LINE/L1 1344 640**

**29129565 29129886 C HAL1 LINE/L1 417 83**

**29129893 29130196 + AluSx SINE/Alu 1 302**

**29130592 29130645 + (TG)n Simple_repeat 1 54**

**29131346 29131591 C MIR SINE/MIR 262 5**

**29132607 29132875 + AluSg SINE/Alu 14 292**

**29132891 29132913 + AT_rich Low_complexity 1 23**

**29132976 29133271 + MLT1A0 LTR/MaLR 1 326**

**29133612 29133796 C LTR33A LTR/ERVL 496 299**

**Ortholog in Chimp 29782380-29788390 Minus Nscore 0.00**

**N positions**

**29778139 29778358 C MER58A DNA/MER1_type 222 1**

**29778915 29778935 + AT_rich Low_complexity 1 21**

**29779894 29780103 + MIR SINE/MIR 1 218**

**29780104 29780256 C MER5A1 DNA/MER1_type 159 1**

**29780257 29780315 + THER1_MD SINE/MIR 219 274**

**29780395 29780458 + L2 LINE/L2 3277 3341**

**29780781 29780804 + (A)n Simple_repeat 1 24**

**29781082 29782381 + L1MA4 LINE/L1 4307 5607**

**29782380 29784999 C L1PA3 LINE/L1 4147 1528**

**29784995 29787017 + L1PA3 LINE/L1 4132 6155**

**29787021 29787410 + L1MA4 LINE/L1 5595 5991**

**29787422 29787447 + AT_rich Low_complexity 1 26**

**29787578 29789226 C HAL1 LINE/L1 2393 640**

**29789343 29789664 C HAL1 LINE/L1 417 83**

**29789671 29789975 + AluSx SINE/Alu 1 307**

**29790373 29790418 + (TG)n Simple_repeat 1 46**

**29791119 29791361 C MIR SINE/MIR 262 8**

**29792380 29792651 + AluSg SINE/Alu 14 295**

**29792665 29792687 + AT_rich Low_complexity 1 23**

**29792750 29793045 + MLT1A0 LTR/MaLR 1 326**

**29793383 29793471 C LTR33A LTR/ERVL 496 393**

**_________________________________________________________________________**

**L1HS_3_24c 36637972-36641523 C_INTRA_RMD**

**36634339 36634515 C MIRb SINE/MIR 197 14**

**36635470 36635695 C MER46C DNA/MER2_type 330 100**

**36637142 36637402 C L1PA5 LINE/L1 6154 5894**

**36637424 36637492 + Tigger1 DNA/MER2_type 2351 2418**

**36637972 36641523 C L1HS LINE/L1 6154 2621 ( L1HS_3_24c ) (R1,R2)**

**36641560 36641641 + L1MC4 LINE/L1 7955 8037**

**36641780 36641925 + MIRb SINE/MIR 101 236**

**36644232 36644269 + GA-rich Low_complexity 1 38**

**36644541 36644671 + AluJb SINE/Alu 179 311**

**36644684 36644726 + (GAAA)n Simple_repeat 2 42**

**Ortholog in Chimp 37462401-37462886 Minus Nscore 0.00**

**N positions**

**37457561 37457659 + (TA)n Simple_repeat 2 97**

**37458716 37458953 C MIRb SINE/MIR 264 13**

**37459916 37460129 C MER46C DNA/MER2_type 317 100**

**37461580 37461849 C L1PA5 LINE/L1 6154 5887**

**37461861 37461929 + Tigger1 DNA/MER2_type 2351 2418**

**37462397 37462754 C L1PA3 LINE/L1 6155 5837 (R12)**

**37462759 37462898 C L1P1 LINE/L1 2749 2611 (R12)**

**37462923 37463004 + L1MC4 LINE/L1 7955 8037**

**37463143 37463288 + MIRb SINE/MIR 101 236**

**37465594 37465631 + GA-rich Low_complexity 1 38**

**37465903 37466032 + AluJb SINE/Alu 179 312**

**37466046 37466092 + (GAAA)n Simple_repeat 2 46**

**_________________________________________________________________________**

**L1HS_3_54 98842962-98846616 M_INTRA_RMD**

**98834024 98834112 + L2 LINE/L2 3291 3377**

**98835618 98835839 + MIR SINE/MIR 13 262**

**98836088 98836397 + LTR16A LTR/ERVL 52 379**

**98836397 98836703 C Charlie1a DNA/MER1_type 334 42**

**98836925 98837366 + L1MB8 LINE/L1 5715 6178**

**98837549 98837674 C MIRb SINE/MIR 262 115**

**98838512 98838534 + AT_rich Low_complexity 1 23**

**98839922 98839943 + AT_rich Low_complexity 1 22**

**98841131 98841154 + AT_rich Low_complexity 1 24**

**98842018 98842329 + AluSp SINE/Alu 1 298**

**98842359 98842637 C L2 LINE/L2 2779 2502**

**98842962 98846616 + L1HS LINE/L1 1 3661 ( L1HS_3_54 ) (R12)**

**98846617 98847212 + L1PA2 LINE/L1 5559 6155 (R12)**

**98847952 98848006 C Charlie7 DNA/MER1_type 97 38**

**98848103 98848747 C MER70B LTR/ERVL 578 1**

**98850611 98850959 C THE1B LTR/MaLR 363 3**

**98851299 98851326 + (CA)n Simple_repeat 1 28**

**98852286 98852409 + AluSg/x SINE/Alu 178 301**

**98853014 98853038 + AT_rich Low_complexity 1 25**

**98853376 98853613 C L1PA16 LINE/L1 6163 5924**

**98854152 98854445 + MLT1L LTR/MaLR 314 615**

**98854672 98855821 + L1MA9 LINE/L1 5114 6311**

**Ortholog in Chimp 101503376-101508555 Plus Nscore 0.39**

**N positions 101503886-101503895; 101504930-101504939;**

**101494430 101494518 + L2 LINE/L2 3291 3377**

**101496028 101496249 + MIR SINE/MIR 13 262**

**101496418 101496493 C LTR16A LTR/ERVL 445 364**

**101496497 101496806 + LTR16A LTR/ERVL 52 379**

**101496806 101497113 C Charlie1a DNA/MER1_type 334 42**

**101497335 101497776 + L1MB8 LINE/L1 5715 6178**

**101497955 101498076 C THER1_MD SINE/MIR 273 127**

**101498924 101498946 + AT_rich Low_complexity 1 23**

**101500330 101500351 + AT_rich Low_complexity 1 22**

**101501538 101501561 + AT_rich Low_complexity 1 24**

**101501878 101501907 + AT_rich Low_complexity 1 30**

**101502425 101502742 + AluSp SINE/Alu 1 299**

**101502772 101503050 C L2 LINE/L2 2779 2502**

**101503377 101503901 + L1Pt LINE/L1 2 520 (R1,R2)**

**101503903 101504929 + L1Pt LINE/L1 1351 2377 (R1,R2)**

**101504942 101509144 + L1PA2 LINE/L1 1823 6032 (R1,R2)**

**101509886 101509940 C Charlie7 DNA/MER1_type 97 38**

**101510037 101510676 C MER70B LTR/ERVL 578 1**

**101512541 101512889 C THE1B LTR/MaLR 363 3**

**101514209 101514340 + AluSg/x SINE/Alu 178 309**

**101514945 101514969 + AT_rich Low_complexity 1 25**

**101515307 101515543 C L1PA16 LINE/L1 6163 5924**

**101516136 101516429 + MLT1L LTR/MaLR 314 615**

**101516663 101517812 + L1MA9 LINE/L1 5114 6311**

**_________________________________________________________________________**

**L1HS_3_57 104434312-104435487 M_DISRUPTED**

**104421983 104428010 + L1PA2 LINE/L1 4 6032**

**104430325 104430656 C MLT1C LTR/MaLR 354 4**

**104430910 104430961 + AT_rich Low_complexity 1 52**

**104431063 104431298 C MIR SINE/MIR 239 5**

**104431354 104431383 + AT_rich Low_complexity 1 30**

**104434173 104434311 + AluY SINE/Alu 1 138**

**104434312 104435487 + L1HS LINE/L1 1786 2961 ( L1HS_3_57 ) (R1)**

**104435488 104435796 + AluY SINE/Alu 1 307 (D)**

**104435797 104438873 + L1PA2 LINE/L1 3085 6155 (R2)**

**104438883 104439059 + AluY SINE/Alu 127 303**

**104440589 104441054 C LTR40b LTR/ERVL 462 3**

**104441811 104441845 + AT_rich Low_complexity 1 35**

**104442215 104442346 C Charlie8 DNA/MER1_type 271 139**

**104444037 104444621 C MER77 LTR/ERVL 607 1**

**Ortholog in Chimp 107247821-107248983 Plus Nscore 0.00**

**N positions**

**107239052 107239072 + AT_rich Low_complexity 1 21**

**107239080 107239588 C L2 LINE/L2 3419 2878**

**107240299 107240369 + A-rich Low_complexity 2 74**

**107240440 107241063 + HAL1b LINE/L1 742 1394**

**107243841 107244166 C MLT1C LTR/MaLR 354 4**

**107244420 107244471 + AT_rich Low_complexity 1 52**

**107244576 107244808 C MIR SINE/MIR 236 5**

**107244864 107244893 + AT_rich Low_complexity 1 30**

**107247682 107247820 + AluY SINE/Alu 1 138**

**107247821 107252062 + L1PA3 LINE/L1 1909 6155 (R)**

**107252066 107252246 + AluY SINE/Alu 127 307**

**107253777 107254236 C LTR40b LTR/ERVL 462 3**

**107254992 107255027 + AT_rich Low_complexity 1 36**

**107257217 107257801 C MER77 LTR/ERVL 607 1**

**_________________________________________________________________________**

**L1HS_3_83 133338444-133343287 M_INTRA_RMD**

**133328875 133330099 + L1PA3 LINE/L1 4929 6155**

**133330175 133330423 + MLT1H LTR/MaLR 23 262**

**133330486 133330621 + MLT1H LTR/MaLR 410 542**

**133330754 133331201 + MLT2B2 LTR/ERVL 3 514**

**133332122 133332333 C MIRb SINE/MIR 253 40**

**133332874 133333048 C MIRb SINE/MIR 258 45**

**133333776 133334318 + L1ME3B LINE/L1 5353 5888**

**133336710 133336835 + L1MB8 LINE/L1 6044 6167**

**133336854 133336880 + AT_rich Low_complexity 1 27**

**133336939 133337151 + MIRb SINE/MIR 41 253**

**133338147 133338355 + L1ME4a LINE/L1 5575 5804**

**133338444 133343287 + L1HS LINE/L1 42 4892 ( L1HS_3_83 ) (R12)**

**133343288 133343603 + AluYa5 SINE/Alu 1 310 (D)**

**133343604 133344755 + L1PA3 LINE/L1 5016 6152 (R12)**

**133344868 133344896 + AT_rich Low_complexity 1 29**

**133345490 133345524 + (A)n Simple_repeat 1 35**

**133345559 133345714 + L1ME4a LINE/L1 5957 6120**

**133345734 133346009 + AluSc SINE/Alu 34 302**

**133346011 133346328 + AluSg SINE/Alu 1 317**

**133346678 133346790 C L2 LINE/L2 3345 3233**

**133346791 133347015 C MER20 DNA/MER1_type 219 1**

**133347016 133347281 C L2 LINE/L2 3232 2942**

**133347443 133347902 + MLT1J1 LTR/MaLR 2 444**

**133348707 133348826 C MER112 DNA/MER1_type 196 74**

**133348836 133349581 + L1ME2 LINE/L1 5301 6113**

**133349883 133349966 C MIRb SINE/MIR 197 118**

**133350914 133351052 + MIRb SINE/MIR 77 215**

**133351153 133351257 C L2 LINE/L2 3418 3309**

**133351366 133351699 + LTR16A LTR/ERVL 97 428**

**133351700 133352065 + MLT2D LTR/ERVL 1 360**

**133352087 133352139 + (TC)n Simple_repeat 1 53**

**133352140 133352178 + (TG)n Simple_repeat 2 40**

**Ortholog in Chimp 136680168-136686781 Plus Nscore 0.15**

**N positions 136683001-136683010;**

**136670599 136671824 + L1PA3 LINE/L1 4929 6154**

**136671990 136672136 + MLT1H LTR/MaLR 108 249**

**136672477 136672922 + MLT2B2 LTR/ERVL 3 514**

**136673842 136674053 C MIRb SINE/MIR 253 40**

**136674599 136674813 C MIRb SINE/MIR 258 9**

**136675502 136676044 + L1ME3B LINE/L1 5353 5888**

**136677958 136678035 C L2 LINE/L2 3162 3083**

**136678435 136678560 + L1MB8 LINE/L1 6044 6167**

**136678579 136678605 + AT_rich Low_complexity 1 27**

**136678664 136678876 + MIRb SINE/MIR 41 253**

**136679872 136680079 + L1ME4a LINE/L1 5575 5804**

**136680167 136683000 + L1Pt LINE/L1 41 2878 (R) (BROKEN DUE TO Ns)**

**136683011 136687931 + L1PA3 LINE/L1 1104 6029 (R) (Overlap at Ns)**

**136688044 136688072 + AT_rich Low_complexity 1 29**

**136688668 136688689 + (A)n Simple_repeat 1 22**

**136688729 136688829 + L1ME4a LINE/L1 5957 6055**

**136688904 136689195 + AluSc SINE/Alu 34 318**

**136689197 136689502 + AluSg SINE/Alu 1 307**

**136689885 136689963 C L2 LINE/L2 3308 3233**

**136689964 136690188 C MER20 DNA/MER1_type 219 1**

**136690189 136690454 C L2 LINE/L2 3232 2942**

**136690616 136691075 + MLT1J1 LTR/MaLR 2 444**

**136691880 136692026 C MER112 DNA/MER1_type 196 48**

**136692027 136692788 + L1ME2 LINE/L1 5319 6294**

**136693071 136693202 C MIRb SINE/MIR 198 42**

**136694098 136694236 + MIRb SINE/MIR 77 215**

**136694337 136694441 C L2 LINE/L2 3418 3309**

**136694550 136694883 + LTR16A LTR/ERVL 97 428**

**136694884 136695236 + MLT2D LTR/ERVL 1 349**

**136695302 136695326 + (TG)n Simple_repeat 2 26**

**136695443 136695725 + MER45B DNA/Tip100 1 308**

**136695723 136695780 + MER45B DNA/Tip100 251 308**

**136695778 136696143 + MER45B DNA/Tip100 251 607**

**_________________________________________________________________________**

**L1HS_3_88c 136203780-136207508 M_INTRA_RMD**

**136197368 136197470 C L1ME4a LINE/L1 6101 5985**

**136197720 136198018 C AluJb SINE/Alu 305 1**

**136198025 136198364 C L1ME4a LINE/L1 5664 5313**

**136198839 136199105 C HAL1b LINE/L1 988 711**

**136200295 136200341 + L2 LINE/L2 3299 3345**

**136203267 136203458 C L1MC4a LINE/L1 5898 5703**

**136203460 136203681 + L1MB4 LINE/L1 5650 5869**

**136203689 136203779 + MSTA LTR/MaLR 1 100**

**136203780 136207508 C L1HS LINE/L1 3730 10 ( L1HS_3_88c ) (R12)**

**136207509 136207740 + THE1D LTR/MaLR 146 381**

**136207743 136208035 + L1MB4 LINE/L1 5869 6179**

**136208091 136208210 + FLAM_A SINE/Alu 11 130**

**136208220 136208499 C L1MC4a LINE/L1 5709 5409**

**136209213 136209439 C L3 LINE/CR1 3736 3512**

**136209759 136209980 C MIR SINE/MIR 262 13**

**136210660 136210749 C MER5B DNA/MER1_type 177 83**

**136210804 136210881 C MER5B DNA/MER1_type 76 1**

**136211422 136211732 C HAL1b LINE/L1 1990 1648**

**136211801 136211974 C HAL1b LINE/L1 1511 1321**

**Ortholog in Chimp 139591280-139597362 Minus Nscore 0.00**

**N positions**

**139584872 139584977 C L1ME4a LINE/L1 6101 5985**

**139585131 139585225 C L1MC LINE/L1 5749 5658**

**139585226 139585524 C AluJb SINE/Alu 305 1**

**139585531 139585869 C L1M5 LINE/L1 5664 5313**

**139585964 139586250 C L1M5 LINE/L1 5155 4860**

**139586334 139586610 C HAL1b LINE/L1 1002 711**

**139587795 139587855 + L2 LINE/L2 3299 3363**

**139590767 139590958 C L1MC4a LINE/L1 5898 5703**

**139590960 139591181 + L1MB4 LINE/L1 5650 5869**

**139591189 139591335 + THE1D LTR/MaLR 1 168**

**139591345 139597362 C L1PA3 LINE/L1 6032 10 (R1,R2)**

**139597363 139597594 + THE1D LTR/MaLR 146 381**

**139597597 139597889 + L1MB4 LINE/L1 5869 6179**

**139597945 139598064 + FLAM_A SINE/Alu 11 130**

**139598074 139598351 C L1MC4a LINE/L1 5709 5409**

**139599066 139599296 C L3 LINE/CR1 3736 3512**

**139599459 139599520 + L3 LINE/CR1 2207 2268**

**139599616 139599838 C MIR SINE/MIR 262 13**

**139600518 139600608 C MER5B DNA/MER1_type 177 83**

**139600663 139600740 C MER5B DNA/MER1_type 76 1**

**139601278 139601588 C HAL1b LINE/L1 1990 1648**

**139601657 139601829 C HAL1b LINE/L1 1511 1321**

**139601838 139601935 C MER5A1 DNA/MER1_type 160 74**

**139601936 139602239 C AluSq SINE/Alu 306 1**

**139602240 139602313 C MER5A1 DNA/MER1_type 73 7**

**139602414 139602740 C HAL1b LINE/L1 1251 958**

**139605770 139605837 + LTR67 LTR/ERVL 490 557**

**139605942 139606055 C L2 LINE/L2 3414 3235**

**_________________________________________________________________________**

**L1HS_3_118 189836527-189837415 M_INTRA_RMD**

**189827412 189827578 C Arthur1 DNA/Tip100 3785 3597**

**189827581 189831676 + L1PA11 LINE/L1 2461 6701**

**189831129 189831184 C MADE1 DNA/Mariner 80 25**

**189831692 189831868 C Arthur1 DNA/Tip100 3609 3431**

**189831902 189832525 C Arthur1 DNA/Tip100 741 35**

**189832791 189832910 C L2 LINE/L2 3184 3064**

**189834752 189834920 C MIRm SINE/MIR 276 84**

**189834921 189835444 + MER1A DNA/MER1_type 1 529**

**189835445 189835471 C MIRm SINE/MIR 83 60**

**189835606 189835628 + AT_rich Low_complexity 1 23**

**189836239 189836294 + L2 LINE/L2 3320 3374**

**189836527 189837415 + L1HS LINE/L1 3 889 ( L1HS_3_118 ) (R12)**

**189837418 189840995 + L1PA3 LINE/L1 2561 6137 (R12)**

**189840996 189841020 + (TAA)n Simple_repeat 3 27**

**189843137 189843433 + AluSp SINE/Alu 1 298**

**189843600 189843755 C MIR3 SINE/MIR 202 38**

**189844608 189844785 C L2 LINE/L2 3158 2973**

**189845372 189845603 + MIRb SINE/MIR 29 267**

**189845616 189845692 C L3 LINE/CR1 4366 4280**

**189845818 189846030 C L3 LINE/CR1 4177 3951**

**Ortholog in Chimp 194235028-194237447 Plus Nscore 0.00**

**N positions**

**194225824 194226088 C Arthur1 DNA/Tip100 3850 3597**

**194226091 194230183 + L1PA11 LINE/L1 2461 6701**

**194229637 194229692 C MADE1 DNA/Mariner 80 25**

**194230198 194230370 C Arthur1 DNA/Tip100 3609 3431**

**194230404 194231027 C Arthur1 DNA/Tip100 741 35**

**194231293 194231412 C L2 LINE/L2 3184 3064**

**194233253 194233421 C MIRm SINE/MIR 276 84**

**194233422 194233945 + MER1A DNA/MER1_type 1 529**

**194233946 194233972 C MIRm SINE/MIR 83 60**

**194234107 194234129 + AT_rich Low_complexity 1 23**

**194234719 194234786 + MIR SINE/MIR 184 256**

**194235028 194241022 + L1PA3 LINE/L1 3 6014 (R1,R2)**

**194241023 194241044 + (TAA)n Simple_repeat 3 24**

**194243155 194243459 + AluSp SINE/Alu 1 306**

**194243626 194243754 C MIR3 SINE/MIR 202 67**

**194244634 194244811 C L2 LINE/L2 3158 2973**

**194245393 194245624 + MIRb SINE/MIR 29 267**

**194245637 194245709 C L3 LINE/CR1 4366 4284**

**194245793 194246044 C L3 LINE/CR1 4233 3951**

**194246306 194246336 + (A)n Simple_repeat 1 31**

**_________________________________________________________________________**

**L1HS_4_4c 13275194-13275977 M_INTRA_RMD**

**13268614 13268953 + MER113 DNA/MER1_type 49 416**

**13268954 13269218 + AluY SINE/Alu 30 294**

**13269219 13269309 + MER113 DNA/MER1_type 417 511**

**13269312 13269602 + AluJb SINE/Alu 1 288**

**13269718 13269826 C MIR3 SINE/MIR 202 82**

**13269851 13269899 + (TG)n Simple_repeat 2 50**

**13271071 13271160 C MIR SINE/MIR 261 166**

**13271736 13271873 C MIRb SINE/MIR 215 83**

**13271947 13271974 + (TTA)n Simple_repeat 3 30**

**13271975 13275190 C L1PA3 LINE/L1 6137 2919 (R12)**

**13275194 13275977 C L1HS LINE/L1 785 3 ( L1HS_4_4c ) (R12)**

**13276069 13276432 C MLT1A0 LTR/MaLR 365 1**

**13276440 13277080 C MLT1A0-int LTR/MaLR 1727 880**

**13277087 13277599 + LTR3B_ LTR/ERVK 1 492**

**13277668 13278507 C MLT1A0-int LTR/MaLR 845 2**

**13278508 13278879 C MLT1A0 LTR/MaLR 363 1**

**13278972 13278999 + AT_rich Low_complexity 1 28**

**13279002 13279137 C MIR SINE/MIR 145 10**

**13279984 13280286 C AluY SINE/Alu 301 1**

**13280360 13280381 C MIRb SINE/MIR 262 245**

**13280382 13280739 C MLT1A0 LTR/MaLR 365 1**

**Ortholog in Chimp 13519378-13522161 Minus Nscore 0.00**

**N positions**

**13513413 13513481 + MER113 DNA/MER1_type 417 489**

**13513506 13513806 + AluJb SINE/Alu 1 298**

**13513912 13514020 C MIR3 SINE/MIR 202 82**

**13514045 13514093 + (TG)n Simple_repeat 2 50**

**13514779 13514827 + L2 LINE/L2 3369 3417**

**13514872 13515028 + MIR SINE/MIR 34 193**

**13515051 13515159 C L2 LINE/L2 3378 3243**

**13515265 13515354 C MIR SINE/MIR 261 166**

**13515930 13516067 C MIRb SINE/MIR 215 83**

**13516141 13516162 + (TTA)n Simple_repeat 3 24**

**13516163 13522161 C L1PA3 LINE/L1 6014 3 (R1,R2)**

**13522253 13522616 C MLT1A0 LTR/MaLR 365 1**

**13522624 13523264 C MLT1A0-int LTR/MaLR 1727 880**

**13523271 13523783 + LTR3B_ LTR/ERVK 1 492**

**13523917 13524391 C MLT1A0-int LTR/MaLR 801 303**

**13524421 13524782 C MLT1A0 LTR/MaLR 353 1**

**13524905 13525040 C MIR SINE/MIR 145 10**

**13525584 13525755 C L2 LINE/L2 3167 2989**

**13525885 13526191 C AluY SINE/Alu 305 1**

**13526265 13526286 C MIRb SINE/MIR 262 245**

**13526287 13526644 C MLT1A0 LTR/MaLR 365 1**

**_________________________________________________________________________**

**L1HS_4_5 13409700-13411042 M_INTRA_RMD**

**13404638 13405125 C Charlie1b DNA/MER1_type 523 2**

**13405141 13405296 + MIR SINE/MIR 65 244**

**13405512 13405705 C MIRb SINE/MIR 211 3**

**13406298 13406539 C MIRb SINE/MIR 254 2**

**13406962 13406995 + AT_rich Low_complexity 1 34**

**13407382 13407549 C MIRb SINE/MIR 196 14**

**13408580 13408841 + GA-rich Low_complexity 1 259**

**13409255 13409565 C L4 LINE/RTE 1715 1417**

**13409700 13411042 + L1HS LINE/L1 1 1334 ( L1HS_4_5 ) (R12)**

**13411031 13415298 + L1PA3 LINE/L1 1901 6168 (R12)**

**13415670 13415954 C AluSc SINE/Alu 304 20**

**13416145 13416312 C MER5A DNA/MER1_type 186 21**

**13416331 13416420 C L4 LINE/RTE 1368 1268**

**13416431 13416542 C MIRb SINE/MIR 265 162**

**13416591 13416724 C MIR SINE/MIR 168 21**

**13416832 13417007 C MER58A DNA/MER1_type 224 47**

**13417265 13417402 + L2 LINE/L2 2742 2891**

**13418256 13418514 + L2 LINE/L2 3120 3358**

**Ortholog in Chimp 13672242-13674030 Plus Nscore 0.00**

**N positions**

**13667246 13667734 C Charlie1b DNA/MER1_type 523 2**

**13667750 13667922 + MIR SINE/MIR 65 272**

**13668133 13668314 C MIRb SINE/MIR 199 3**

**13668907 13669147 C MIRb SINE/MIR 254 2**

**13669570 13669591 + AT_rich Low_complexity 1 22**

**13669701 13669728 + (T)n Simple_repeat 1 28**

**13671120 13671383 + GA-rich Low_complexity 1 261**

**13671797 13672107 C L4 LINE/RTE 1715 1417**

**13672242 13678285 + L1PA3 LINE/L1 1 6045 (R1,R2)**

**13678406 13678427 + AT_rich Low_complexity 1 22**

**13678672 13678961 C AluSc SINE/Alu 309 20**

**13679154 13679319 C MER5A DNA/MER1_type 184 21**

**13679338 13679427 C L4 LINE/RTE 1368 1268**

**13679441 13679553 C MIR SINE/MIR 258 152**

**13679598 13679726 C MIRb SINE/MIR 172 21**

**13679749 13679806 C L4 LINE/RTE 1218 1162**

**13679834 13680009 C MER58A DNA/MER1_type 224 47**

**13680274 13680411 + L2 LINE/L2 2742 2891**

**13681523 13682028 + (TA)n Simple_repeat 1 511**

**_________________________________________________________________________**

**L1HS_4_51 70564317-70567872 C_INTRA_RMD**

**70558105 70558785 C LTR12D LTR/ERV1 687 3**

**70558786 70561025 + L1PBa LINE/L1 857 3356**

**70561026 70561074 + (TAGA)n Simple_repeat 4 52**

**70561075 70561194 + L1PBa LINE/L1 3357 3489**

**70561193 70562034 C L1P2 LINE/L1 888 27**

**70562022 70564314 + L1P2 LINE/L1 889 3191**

**70564317 70567872 + L1HS LINE/L1 2 3559 ( L1HS_4_51 ) (R1,R2)**

**70568136 70568193 + L1MC LINE/L1 4401 4458**

**70568829 70570087 + L1MA1 LINE/L1 17 1272**

**70570088 70570396 + AluJb SINE/Alu 1 309**

**70570397 70574608 + L1MA1 LINE/L1 1273 5378**

**Ortholog in Chimp 60959220-60962534 Minus Nscore 2.41**

**N positions 60961648-60961727;**

**60952489 60956702 C L1MA1 LINE/L1 5378 1273**

**60956703 60957012 C AluJb SINE/Alu 310 1**

**60957013 60958270 C L1MA1 LINE/L1 1272 17**

**60959222 60961609 C L1Pt LINE/L1 3557 1148 (R12)**

**60961728 60962534 C L1Pt LINE/L1 809 2 (R12)**

**60962537 60963133 C L1P1 LINE/L1 3191 2595**

**60963195 60964100 C L1P1 LINE/L1 2304 1396**

**60964465 60965157 C L1P2 LINE/L1 1604 889**

**60965145 60965986 + L1P2 LINE/L1 27 888**

**60965985 60966104 C L1PBa LINE/L1 3491 3372**

**60966105 60966150 + (TCTA)n Simple_repeat 3 48**

**60966151 60968388 C L1PBa LINE/L1 3371 857**

**60968389 60969069 + LTR12D LTR/ERV1 3 686**

**_________________________________________________________________________**

**L1HS_4_52c 70915703-70919982 M_INTRA_RMD**

**70907696 70907840 + MER5A1 DNA/MER1_type 9 157**

**70908230 70908250 + AT_rich Low_complexity 1 21**

**70909036 70909436 C MER92A LTR/ERV1 399 1**

**70910117 70910475 C L1PB1 LINE/L1 6867 6509**

**70910476 70910544 + (TA)n Simple_repeat 1 69**

**70910545 70914128 C L1PB1 LINE/L1 6508 2906**

**70914129 70914444 C AluSc SINE/Alu 281 1**

**70914445 70915703 C L1PB1 LINE/L1 2905 1563**

**70915703 70919982 C L1HS LINE/L1 4280 1 ( L1HS_4_52c )**

**70920061 70920218 C MIRb SINE/MIR 195 30**

**70920264 70920744 C MLT1D LTR/MaLR 502 1**

**70921672 70922926 + L1PA4 LINE/L1 4930 6152**

**70923710 70924244 C L2 LINE/L2 3414 2802**

**70924976 70925061 + AT_rich Low_complexity 1 86**

**70925258 70925371 + L2 LINE/L2 2245 2361**

**70925711 70925792 + GA-rich Low_complexity 1 82**

**70926549 70926841 + AluJo SINE/Alu 6 293**

**70927206 70927228 + AT_rich Low_complexity 1 23**

**70927375 70927583 C L1PA8 LINE/L1 6171 5954**

**70927660 70927838 C MER5A DNA/MER1_type 183 1**

**Ortholog in Chimp 60593498-60600888 Plus Nscore 0.00**

**N positions**

**60585690 60585868 + MER5A DNA/MER1_type 1 183**

**60585945 60586153 + L1PA8 LINE/L1 5954 6171**

**60586295 60586317 + AT_rich Low_complexity 1 23**

**60586682 60586972 C AluJo SINE/Alu 293 6**

**60587056 60587125 + T-rich Low_complexity 2 69**

**60587726 60587807 + CT-rich Low_complexity 1 82**

**60588147 60588257 C L2 LINE/L2 2356 2245**

**60588509 60588538 + AT_rich Low_complexity 1 30**

**60589272 60589804 + L2 LINE/L2 2802 3414**

**60590589 60591810 C L1PA4 LINE/L1 6152 4930**

**60592531 60592646 C L3 LINE/CR1 4485 4366**

**60592737 60593218 + MLT1D LTR/MaLR 1 502**

**60593264 60593464 + MIRb SINE/MIR 30 243**

**60593498 60599515 + L1PA3 LINE/L1 1 6032**

**60599799 60599969 C MIRb SINE/MIR 245 59**

**60600002 60600042 + (TG)n Simple_repeat 2 42**

**60600301 60600363 + (TA)n Simple_repeat 1 62**

**60600540 60600719 + L1PB1 LINE/L1 3 439**

**60600718 60602148 + L1PB1 LINE/L1 1393 2905**

**60602149 60602470 + AluSc SINE/Alu 1 285**

**60602471 60606070 + L1PB1 LINE/L1 2906 6508**

**60606071 60606219 + (TA)n Simple_repeat 2 150**

**60606220 60606413 + L1PB1 LINE/L1 6509 6730**

**60606510 60606793 + L1PB1 LINE/L1 6584 6867**

**60607483 60607556 + MER92A LTR/ERV1 1 74**

**60607576 60607976 + MER92A LTR/ERV1 1 399**

**60608766 60608786 + AT_rich Low_complexity 1 21**

**60609177 60609321 C MER5A1 DNA/MER1_type 157 9**

**_________________________________________________________________________**

**L1HS_4_59 82293934-82295070 C_INTER_RMD_M_DISRUPTED**

**82284789 82285431 C L1PA5 LINE/L1 6151 5509**

**82286143 82286225 + LTR33 LTR/ERVL 1 92**

**82286814 82287000 C L2 LINE/L2 3411 3230**

**82287043 82287165 C MER94 DNA/AcHobo 134 1**

**82287890 82287959 + MIR3 SINE/MIR 93 170**

**82287960 82288174 + L1MC5 LINE/L1 7638 7860**

**82288203 82288238 + AT_rich Low_complexity 1 36**

**82289627 82289761 + MER104 DNA/Tc2 18 162**

**82290254 82290343 + (CAGAGA)n Simple_repeat 2 92**

**82291471 82291499 + AT_rich Low_complexity 1 29**

**82292901 82292925 + AT_rich Low_complexity 1 25**

**82293510 82293617 C MIR3 SINE/MIR 201 86**

**82293934 82295070 + L1HS LINE/L1 3 1137 ( L1HS_4_59 ) (R1)**

**82295071 82295375 + AluYa5 SINE/Alu 1 301 (D)**

**82295376 82300322 + L1PA3 LINE/L1 1138 6054 (R2)**

**82301540 82302147 C MLT1E2 LTR/MaLR 625 1**

**82302274 82302419 + MIRb SINE/MIR 119 263**

**82302474 82302796 + MLT1J1 LTR/MaLR 58 471**

**82303582 82303720 C Charlie9 DNA/MER1_type 2659 2528**

**82304042 82304081 + (CA)n Simple_repeat 2 41**

**Ortholog in Chimp 49246821-49247947 Minus Nscore 0.00 (49241166-49247947 - Nscore 0.15)**

**N positions**

**49237769 49237907 + Charlie9 DNA/MER1_type 2528 2659**

**49238379 49238449 + MIRm SINE/MIR 108 181**

**49238691 49239015 C MLT1J1 LTR/MaLR 473 58**

**49239069 49239215 C MIRb SINE/MIR 264 119**

**49239342 49239949 + MLT1E2 LTR/MaLR 1 625**

**49241166 49242424 C L1PA3 LINE/L1 6177 4892**

**49242457 49247947 C L1P1 LINE/L1 5498 3 (R BROKEN DUE TO Ns)**

**49248311 49248371 + L3 LINE/CR1 4422 4486**

**49248956 49248980 + AT_rich Low_complexity 1 25**

**49250095 49250128 + AT_rich Low_complexity 1 34**

**49251537 49251626 + (TCTCTG)n Simple_repeat 2 92**

**49252119 49252253 C MER104 DNA/Tc2 162 18**

**49253641 49253676 + AT_rich Low_complexity 1 36**

**49253705 49253919 C L1MC5 LINE/L1 7860 7638**

**49253920 49253989 C MIR3 SINE/MIR 170 93**

**49254714 49254836 + MER94 DNA/AcHobo 1 134**

**49254879 49255065 + L2 LINE/L2 3230 3411**

**49255654 49255736 C LTR33 LTR/ERVL 92 1**

**49256448 49257090 + L1PA5 LINE/L1 5509 6151**

**_________________________________________________________________________**

**L1HS_4_84c 111550299-111552050 C_DISRUPTED_M_INTER_RMD**

**111544803 111545232 C Tigger2a DNA/MER2_type 434 2**

**111545834 111546453 + MER4A LTR/ERV1 1 625**

**111546455 111550299 C L1PA2 LINE/L1 6155 2309**

**111550299 111552050 C L1HS LINE/L1 1756 4 ( L1HS_4_84c )**

**111552058 111552113 + MER4A LTR/ERV1 609 664**

**111553245 111553302 + MIRb SINE/MIR 201 260**

**111553311 111553733 C MLT1E2 LTR/MaLR 618 146**

**111553777 111554193 C MLT1C LTR/MaLR 467 86**

**111554194 111554501 C AluSx SINE/Alu 312 1**

**111554502 111554555 C MLT1C LTR/MaLR 85 36**

**111555367 111555535 + MIRb SINE/MIR 27 198**

**111555625 111555645 + AT_rich Low_complexity 1 21**

**111555801 111555846 C tRNA-Gln-CAG tRNA 46 1**

**111555911 111556211 C AluJo SINE/Alu 306 1**

**111556614 111556867 + AluY SINE/Alu 39 302**

**Ortholog in Chimp 113857858-113859756 Minus Nscore 6.79**

**N positions 113858597-113858724; 113858525-113858525;**

**113851877 113852184 C AluSg SINE/Alu 309 1**

**113852255 113852392 C MIRb SINE/MIR 212 71**

**113853117 113853546 C Tigger2a DNA/MER2_type 434 2**

**113854148 113854766 + MER4A LTR/ERV1 1 624**

**113854767 113854863 C L1Pt LINE/L1 6153 6056**

**113855310 113855662 + L1ME3B LINE/L1 5547 5916**

**113855746 113857062 C L1PA5 LINE/L1 6141 4824**

**113857537 113857858 C L1PA5 LINE/L1 2630 2309**

**113857858 113859756 C L1PA5 LINE/L1 1879 4 (OCCUPIED)**

**113859764 113859819 + MER4A LTR/ERV1 609 664**

**113860088 113860146 + L3_Mars LINE/CR1 3033 3098**

**113860955 113861010 + MIR SINE/MIR 200 257**

**113861018 113861440 C MLT1E2 LTR/MaLR 618 146**

**113861484 113861898 C MLT1C LTR/MaLR 467 86**

**113861899 113862206 C AluSx SINE/Alu 312 1**

**113862207 113862260 C MLT1C LTR/MaLR 85 36**

**113865341 113865651 C AluY SINE/Alu 311 1**

**_________________________________________________________________________**

**L1HS_4_114 147803889-147804215 C_INTER_RMD_M_DISRUPTED**

**147795751 147795788 + GC_rich Low_complexity 1 38**

**147797462 147797546 + (TA)n Simple_repeat 2 86**

**147797547 147797572 + (TG)n Simple_repeat 2 28**

**147798137 147798385 + MER112 DNA/MER1_type 10 261**

**147798711 147799004 C L1PA16 LINE/L1 6165 5858**

**147799086 147799248 + L2 LINE/L2 3227 3408**

**147799513 147799849 + L4 LINE/RTE 1510 1871**

**147799887 147800130 + MIRb SINE/MIR 9 265**

**147803253 147803289 + CT-rich Low_complexity 3 39**

**147803397 147803445 + AT_rich Low_complexity 1 49**

**147803675 147803811 C MIR3 SINE/MIR 204 55**

**147803889 147804215 + L1HS LINE/L1 1 327 ( L1HS_4_114 ) (R1)**

**147804216 147804524 + AluYa5 SINE/Alu 1 309 (D)**

**147804525 147810242 + L1PA3 LINE/L1 328 6032 (R2)**

**147811081 147811224 C L1ME4a LINE/L1 6119 5959**

**147811225 147811542 C AluSx SINE/Alu 302 1**

**147811543 147811722 C L1ME4a LINE/L1 5958 5768**

**147811759 147811856 C FLAM_A SINE/Alu 110 13**

**147812215 147812417 + MLT1K LTR/MaLR 218 417**

**147812439 147812535 + GA-rich Low_complexity 1 94**

**Ortholog in Chimp 150706845-150707155 Plus Nscore 0.00**

**N positions**

**150698592 150698629 + GC_rich Low_complexity 1 38**

**150700475 150700511 + (TA)n Simple_repeat 2 38**

**150701089 150701339 + MER112 DNA/MER1_type 8 261**

**150701661 150701955 C L1PA16 LINE/L1 6166 5858**

**150702037 150702199 + L2 LINE/L2 3227 3408**

**150702464 150702672 + L4 LINE/RTE 1510 1738**

**150702838 150703081 + MIRb SINE/MIR 9 265**

**150706211 150706247 + CT-rich Low_complexity 3 39**

**150706631 150706767 C MIR3 SINE/MIR 204 55**

**150706845 150709686 + L1Pt LINE/L1 1 2843 (R) BROKEN DUE TO Ns**

**150709681 150714057 + L1PA3 LINE/L1 1427 6032 (R) OVERLAP AT Ns**

**150714905 150715050 C L1ME4a LINE/L1 6121 5970**

**150715051 150715371 C AluSx SINE/Alu 305 1**

**150715372 150715551 C L1ME4a LINE/L1 5969 5768**

**150715552 150715683 C FLAM_A SINE/Alu 140 13**

**_________________________________________________________________________**

**L1HS_4_129 168008182-168009360 M_INTRA_RMD**

**168002082 168002348 + MLT2D LTR/ERVL 1 293**

**168002349 168002373 + (TCTG)n Simple_repeat 2 26**

**168002382 168002475 + (TA)n Simple_repeat 2 95**

**168002477 168002561 + MLT2B3 LTR/ERVL 479 555**

**168003537 168003921 C MER7A DNA/MER2_type 346 2**

**168005374 168005407 + (TA)n Simple_repeat 1 34**

**168007105 168007154 C MADE1 DNA/Mariner 77 34**

**168007309 168008050 C L1MB2 LINE/L1 6168 5421**

**168008182 168009360 + L1HS LINE/L1 3 1180 ( L1HS_4_129 ) (R12)**

**168009361 168010031 + L1PA3 LINE/L1 5480 6150 (R12)**

**168010064 168010330 + MER72 LTR/ERV1 48 329**

**168010321 168010494 + MER72 LTR/ERV1 546 722**

**168011327 168011626 + AluSx SINE/Alu 1 301**

**168011727 168011804 + MADE2 DNA/Mariner 1 80**

**168012627 168012799 + LTR29 LTR/ERV1 2 195**

**168012858 168012923 + LTR49 LTR/ERV1 522 591**

**168013432 168013606 C MER5A DNA/MER1_type 167 10**

**Ortholog in Chimp 171182353-171187702 Plus Nscore 0.00**

**N positions**

**171176274 171176540 + MLT2D LTR/ERVL 1 293**

**171176541 171176565 + (TCTG)n Simple_repeat 2 26**

**171176620 171176638 + Ricksha DNA/MuDR 848 863**

**171176639 171176692 + (TCTA)n Simple_repeat 1 54**

**171176693 171176734 + Ricksha DNA/MuDR 864 906**

**171177711 171177966 C Tigger3(Golem) DNA/MER2_type 3028 2771**

**171177964 171178103 C Tigger3(Golem) DNA/MER2_type 142 2**

**171179548 171179577 + (TA)n Simple_repeat 1 30**

**171181481 171182222 C L1MB2 LINE/L1 6168 5421**

**171182223 171182243 + AT_rich Low_complexity 1 21**

**171182353 171188376 + L1PA3 LINE/L1 3 6032 (R1,R2)**

**171188389 171188655 + MER72 LTR/ERV1 48 329**

**171188646 171188819 + MER72 LTR/ERV1 546 722**

**171189649 171189951 + AluSx SINE/Alu 1 304**

**171190052 171190129 + MADE2 DNA/Mariner 1 80**

**171190950 171191122 + LTR29 LTR/ERV1 2 195**

**171191181 171191246 + LTR49 LTR/ERV1 522 591**

**171191756 171191936 C MER5A DNA/MER1_type 167 10**

**171191937 171192014 C L2 LINE/L2 3372 3290**

**171192031 171192088 + L1MA6 LINE/L1 5847 5911**

**171192119 171192518 + L1MA6 LINE/L1 5894 6296**

**171192538 171192823 + AluSc SINE/Alu 1 286**

**171192825 171192935 + (TCTA)n Simple_repeat 4 114**

**171194161 171194230 + MIRm SINE/MIR 205 275**

**171194990 171195369 + MER7A DNA/MER2_type 1 331**

**_________________________________________________________________________**

**L1HS_4_130c 168506431-168507287 C_INTER_RMD_M_DISRUPTED**

**168497339 168497642 + AluSg SINE/Alu 1 302**

**168497686 168498525 + L1MCc LINE/L1 1833 2753**

**168498536 168498691 + FRAM SINE/Alu 1 156**

**168498711 168499078 + Tigger5b DNA/MER2_type 22 418**

**168499227 168499630 + L1MCc LINE/L1 2928 3341**

**168499691 168499808 C AluJb SINE/Alu 118 1**

**168499809 168499856 + L1MCc LINE/L1 3430 3482**

**168500210 168500592 C MSTA LTR/MaLR 428 1**

**168500680 168501238 C L1PA16 LINE/L1 6157 5593**

**168501243 168506404 C L1PA3 LINE/L1 6032 858 (R2)**

**168506405 168506430 + (ATG)n Simple_repeat 1 26 (D)**

**168506431 168507287 C L1HS LINE/L1 857 2 ( L1HS_4_130c ) (R1)**

**168507287 168508598 C L1PA16 LINE/L1 5608 4332**

**168509114 168509167 + AT_rich Low_complexity 1 54**

**168509324 168509618 C AluSg SINE/Alu 297 1**

**168509835 168510096 C AluY SINE/Alu 299 40**

**168510563 168510818 + Tigger7 DNA/MER2_type 1 267**

**168510818 168510914 + Tigger7 DNA/MER2_type 2394 2490**

**168511304 168511347 C L1P5 LINE/L1 5686 5643**

**168511348 168511445 + L1PB1 LINE/L1 6052 6150**

**168511784 168511854 + AluY SINE/Alu 234 304**

**168511858 168511913 + L1ME4a LINE/L1 6063 6118**

**168511925 168512043 + L1M5 LINE/L1 3331 3450**

**168512187 168512225 C Tigger1 DNA/MER2_type 2238 2208**

**168512226 168512524 + AluSg SINE/Alu 1 303**

**168512525 168513222 C Tigger1 DNA/MER2_type 2207 1524**

**168513288 168513312 + AT_rich Low_complexity 1 25**

**168513369 168513419 + L1M1 LINE/L1 3704 3754**

**168513418 168513929 C L1MEc LINE/L1 2760 2241**

**168513931 168514132 C L1MEc LINE/L1 2234 2033**

**168514195 168514322 + (TA)n Simple_repeat 1 129**

**168514440 168514557 C L1MEc LINE/L1 1362 1246**

**168514652 168514808 C L1MEc LINE/L1 1034 873**

**168514798 168515593 + L1M3 LINE/L1 2390 3077**

**168515594 168515617 + (TAAAA)n Simple_repeat 2 25**

**168515618 168516847 + L1M3 LINE/L1 3078 4329**

**Ortholog in Chimp 171691526-171692393 Minus Nscore 0.00 (17686135-171692393 Nscore -27)**

**N positions**

**171682410 171682768 C MLT1D LTR/MaLR 505 132**

**171682796 171683098 + AluSg SINE/Alu 1 301**

**171683142 171683980 + L1MCc LINE/L1 1833 2753**

**171683991 171684146 + FRAM SINE/Alu 1 156**

**171684166 171684534 + Tigger5b DNA/MER2_type 22 418**

**171684682 171685066 + L1MCc LINE/L1 2934 3303**

**171685145 171685262 C AluJb SINE/Alu 118 1**

**171685263 171685310 + L1MCc LINE/L1 3430 3482**

**171685664 171686047 C MSTA LTR/MaLR 428 1**

**171686135 171686630 C L1PA16 LINE/L1 6157 5659 (R)**

**171687457 171689877 C L1P1 LINE/L1 5396 2927 (R) BROKEN DUE TO Ns**

**171690794 171692393 C L1Pt LINE/L1 1601 2 (R)**

**171692393 171693704 C L1PA16 LINE/L1 5608 4332**

**171694220 171694273 + AT_rich Low_complexity 1 54**

**171694430 171694724 C AluSg SINE/Alu 297 1**

**171694936 171695196 C AluY SINE/Alu 299 40**

**171695658 171695913 + Tigger7 DNA/MER2_type 1 267**

**171695913 171696009 + Tigger7 DNA/MER2_type 2394 2490**

**171696398 171696441 C L1P5 LINE/L1 5686 5643**

**171696442 171696539 + L1PB1 LINE/L1 6052 6150**

**171696877 171696938 + Alu SINE/Alu 234 295**

**171696942 171696997 + L1ME4a LINE/L1 6063 6118**

**171697009 171697127 + L1M5 LINE/L1 3331 3450**

**171697271 171697309 C Tigger1 DNA/MER2_type 2238 2208**

**171697310 171697608 + AluSg SINE/Alu 1 303**

**171697609 171698307 C Tigger1 DNA/MER2_type 2207 1524**

**171698373 171698397 + AT_rich Low_complexity 1 25**

**171698454 171698504 + L1P3 LINE/L1 3704 3754**

**171698503 171699014 C L1MEc LINE/L1 2760 2241**

**171699016 171699217 C L1MEc LINE/L1 2234 2033**

**171699280 171699409 + (TA)n Simple_repeat 1 131**

**171699528 171699647 C L1MEc LINE/L1 1362 1244**

**171699740 171699896 C L1MEc LINE/L1 1034 873**

**171699868 171700686 + L1P4 LINE/L1 2375 3071**

**171700687 171700710 + (TAAAA)n Simple_repeat 2 25**

**171700711 171701918 + L1M2 LINE/L1 3068 4317**

**_________________________________________________________________________**

**L1HS_4_134 177849935-177850260 C_INTER_RMD_M_DISRUPTED**

**177840798 177841103 + AluSx SINE/Alu 1 306**

**177841188 177841221 + AT_rich Low_complexity 1 34**

**177842886 177843172 C AluSx SINE/Alu 288 3**

**177844224 177844731 C L2 LINE/L2 2202 1616**

**177846397 177846617 C L1ME4a LINE/L1 5884 5663**

**177846726 177846801 + CT-rich Low_complexity 3 77**

**177846928 177847123 C L1MC4a LINE/L1 6815 6618**

**177848493 177848633 C L1MC4a LINE/L1 6004 5849**

**177849060 177849184 + Charlie9 DNA/MER1_type 1 123**

**177849935 177850260 + L1HS LINE/L1 2 326 ( L1HS_4_134 ) (R1)**

**177850261 177850577 + AluYa5 SINE/Alu 1 317 (D)**

**177850578 177856277 + L1PA3 LINE/L1 327 6014 (R2)**

**177856278 177856315 + (TAA)n Simple_repeat 3 40**

**177856548 177856579 + (CATATA)n Simple_repeat 3 34**

**177856591 177857034 C L1MA9 LINE/L1 6312 5827**

**177858283 177858573 + AluSx SINE/Alu 1 295**

**177859083 177859428 + L1MC2 LINE/L1 5976 6344**

**Ortholog in Chimp 181177517-181177827 Plus Nscore 0.00 (181177517-181184605 Nscore 5.78)**

**N positions**

**181168392 181168693 + AluSx SINE/Alu 1 303**

**181168778 181168812 + AT_rich Low_complexity 1 35**

**181170475 181170760 C AluSx SINE/Alu 288 4**

**181171811 181172305 C L2 LINE/L2 2202 1632**

**181173982 181174203 C L1ME4a LINE/L1 5885 5663**

**181174312 181174391 + CT-rich Low_complexity 3 81**

**181174518 181174720 C L1MC4a LINE/L1 6815 6605**

**181176069 181176212 C L1MC4a LINE/L1 6004 5849**

**181176642 181176766 + Charlie9 DNA/MER1_type 1 123**

**181177517 181180702 + L1Pt LINE/L1 2 3162 (R) (BROKEN DUE TO Ns)**

**181180691 181184605 + L1PA3 LINE/L1 2232 6137 (R) (OVERLAP AT Ns)**

**181184606 181184643 + (TAA)n Simple_repeat 3 40**

**181184878 181184909 + (TA)n Simple_repeat 1 32**

**181184921 181185366 C L1MA9 LINE/L1 6312 5825**

**181186614 181186905 + AluSx SINE/Alu 1 296**

**_________________________________________________________________________**

**L1HS_5_52 66081868-66085803 M_INTRA_RMD**

**66072631 66072977 + L2 LINE/L2 2971 3370**

**66072978 66073167 C MER58A DNA/MER1_type 224 1**

**66074003 66074032 + AT_rich Low_complexity 1 30**

**66074575 66074785 + L2 LINE/L2 3013 3243**

**66075400 66075429 + AT_rich Low_complexity 1 30**

**66075589 66075692 + CT-rich Low_complexity 3 106**

**66076042 66076444 C MSTA LTR/MaLR 428 1**

**66076605 66076808 C MIRb SINE/MIR 248 13**

**66077343 66077456 + L2 LINE/L2 3257 3410**

**66077555 66077745 + MIR3 SINE/MIR 16 208**

**66077946 66078104 + MIRb SINE/MIR 5 175**

**66078682 66078729 + (TG)n Simple_repeat 2 49**

**66079309 66079477 C MER20 DNA/MER1_type 219 29**

**66079749 66079884 + L2 LINE/L2 3288 3419**

**66079964 66080061 + CT-rich Low_complexity 1 98**

**66080062 66080371 C AluSc SINE/Alu 309 1**

**66080471 66080563 C L4 LINE/RTE 1429 1334**

**66080966 66081179 C L4 LINE/RTE 1004 790**

**66081206 66081388 C MIR SINE/MIR 258 67**

**66081868 66085803 + L1HS LINE/L1 1 3934 ( L1HS_5_52 ) (R12)**

**66086067 66086104 + AT_rich Low_complexity 1 38**

**66086176 66086910 C L2 LINE/L2 3419 2500**

**66087229 66087415 C L1MA8 LINE/L1 6288 6091**

**66089097 66089166 C L4 LINE/RTE 1954 1889**

**66089537 66089647 C L4 LINE/RTE 1621 1510**

**66089955 66090001 + AT_rich Low_complexity 1 47**

**66090002 66090164 C FRAM SINE/Alu 163 1**

**66090177 66090377 C L4 LINE/RTE 1036 851**

**66090866 66091080 C MER112 DNA/MER1_type 240 1**

**66091628 66091661 C L1ME2 LINE/L1 6153 6126**

**66091662 66091710 + (TTTA)n Simple_repeat 2 50**

**66091711 66091997 C AluSc SINE/Alu 282 1**

**66091998 66092305 C L1ME2 LINE/L1 6125 5827**

**66092305 66092327 C L1ME2 LINE/L1 5610 5593**

**66092328 66092557 + L1MA5A LINE/L1 6063 6296**

**66092558 66092846 C L1ME2 LINE/L1 5592 5305**

**66094522 66094776 C MIR SINE/MIR 262 1**

**Ortholog in Chimp 48901404-48908030 Minus Nscore 2.57**

**N positions 48904385-48904544; 48902987-48902996;**

**48892429 48892683 + MIR SINE/MIR 1 262**

**48894358 48894646 + L1ME2 LINE/L1 5305 5592**

**48894647 48894879 C L1MA5A LINE/L1 6296 6058**

**48894880 48894893 + L1ME2 LINE/L1 5593 5606**

**48894897 48895204 + L1ME2 LINE/L1 5827 6125**

**48895205 48895526 + AluSc SINE/Alu 1 317**

**48895527 48895558 + L1ME2 LINE/L1 6126 6153**

**48896106 48896310 + MER112 DNA/MER1_type 1 223**

**48896809 48897009 + L4 LINE/RTE 851 1036**

**48897022 48897184 + FRAM SINE/Alu 1 163**

**48897185 48897231 + AT_rich Low_complexity 1 47**

**48897539 48897649 + L4 LINE/RTE 1510 1621**

**48897815 48898000 C L1MC4 LINE/L1 8016 7803**

**48898006 48898075 + L4 LINE/RTE 1889 1954**

**48899774 48899974 + L1MA8 LINE/L1 6076 6288**

**48900295 48901031 + L2 LINE/L2 2500 3419**

**48901464 48902986 C L1PA2 LINE/L1 6153 4630 (R1,R2) (Overlap at Ns)**

**48903001 48904309 C L1P1 LINE/L1 5258 3915 (R1,R2) (Broken due to Ns)**

**48904545 48908030 C L1Pt LINE/L1 3495 1 (R1,R2)**

**48908486 48908697 + MIR SINE/MIR 51 260**

**48909333 48909425 + L4 LINE/RTE 1334 1429**

**48909525 48909839 + AluSc SINE/Alu 1 309**

**48909840 48909858 + (GGAA)n Simple_repeat 1 19**

**48909859 48910036 + (GGGA)n Simple_repeat 1 178**

**48910037 48910080 + (GGAA)n Simple_repeat 4 46**

**48910160 48910295 C L2 LINE/L2 3419 3288**

**48910525 48910735 + MER20 DNA/MER1_type 1 219**

**48911320 48911339 + (CA)n Simple_repeat 2 21**

**48911917 48912061 C MIRb SINE/MIR 175 19**

**48912276 48912446 C MIR3 SINE/MIR 208 34**

**48913213 48913416 + MIRb SINE/MIR 13 248**

**48913580 48913982 + MSTA LTR/MaLR 1 428**

**48914332 48914435 + GA-rich Low_complexity 3 106**

**48914595 48914624 + AT_rich Low_complexity 1 30**

**48915230 48915440 C L2 LINE/L2 3243 3013**

**48915983 48916012 + AT_rich Low_complexity 1 30**

**48916848 48917037 + MER58A DNA/MER1_type 1 224**

**_________________________________________________________________________**

**L1HS_5_54c 77916886-77917213 C_INTER_RMD_M_DISRUPTED**

**77907593 77907893 + AluY SINE/Alu 1 301**

**77908341 77908403 C L1PA15 LINE/L1 6165 6103**

**77908929 77908979 C L3b LINE/CR1 4393 4342**

**77910165 77910200 + (CA)n Simple_repeat 1 36**

**77910812 77910853 + GA-rich Low_complexity 1 42**

**77910921 77916574 C L1PA3 LINE/L1 6032 328 (R2)**

**77916575 77916885 C AluYa5 SINE/Alu 310 1 (D)**

**77916886 77917213 C L1HS LINE/L1 327 1 ( L1HS_5_54c ) (R1)**

**77917269 77917292 + (CAT)n Simple_repeat 3 26**

**77917296 77917419 C MIRb SINE/MIR 253 129**

**77917420 77917445 + (TTGGGG)n Simple_repeat 2 27**

**77917446 77917496 C MIRb SINE/MIR 128 71**

**77917765 77917870 + MIRb SINE/MIR 96 201**

**77919751 77919914 C FRAM SINE/Alu 159 1**

**77919998 77920097 + MIR SINE/MIR 37 135**

**77920276 77920406 + MIR SINE/MIR 97 230**

**77920557 77920712 C MIRb SINE/MIR 199 7**

**77920768 77921062 + AluSc SINE/Alu 1 295**

**77921296 77921432 + L2 LINE/L2 3239 3378**

**77921781 77921806 + (CAA)n Simple_repeat 2 27**

**77924250 77924276 + (CA)n Simple_repeat 2 28**

**77924307 77924333 + (CA)n Simple_repeat 2 28**

**77924442 77924607 + L1MB3 LINE/L1 6000 6181**

**77925598 77925714 C MIR SINE/MIR 194 61**

**77925912 77925943 + MIR SINE/MIR 77 108**

**Ortholog in Chimp 37241511-37241822 Plus Nscore 0.00**

**N positions**

**37232924 37232955 C MIR SINE/MIR 108 77**

**37233117 37233333 + MIR SINE/MIR 2 271**

**37234260 37234425 C L1MB3 LINE/L1 6181 6000**

**37236907 37236929 + (TTG)n Simple_repeat 2 24**

**37237278 37237414 C L2 LINE/L2 3378 3239**

**37237648 37237950 C AluSc SINE/Alu 303 1**

**37238006 37238161 + MIRb SINE/MIR 7 199**

**37238278 37238437 C MIRb SINE/MIR 274 104**

**37238616 37238715 C MIR SINE/MIR 135 37**

**37238800 37238965 + FRAM SINE/Alu 1 166**

**37240848 37240953 C MIRb SINE/MIR 201 96**

**37241222 37241272 + MIRb SINE/MIR 70 127**

**37241273 37241298 + (CCCCAA)n Simple_repeat 2 27**

**37241299 37241422 + MIRb SINE/MIR 128 252**

**37241448 37241475 + (GGA)n Simple_repeat 2 29**

**37241511 37246779 + L1Pt LINE/L1 1 5272 (R)**

**37246790 37247526 + L1Pt LINE/L1 507 1241 (DISRUPTED IN CHIMP AS WELL)**

**37247537 37248328 + L1PA3 LINE/L1 5362 6155 (R)**

**37248393 37248434 + CT-rich Low_complexity 1 42**

**37249046 37249077 + (TG)n Simple_repeat 1 32**

**37250262 37250312 + L3b LINE/CR1 4342 4393**

**_________________________________________________________________________**

**L1HS_5_93c 161890327-161891261 M_INTRA_RMD**

**161881102 161881412 C L1MA4A LINE/L1 6299 5984**

**161881415 161882530 C L1M2 LINE/L1 4026 2908**

**161882531 161882553 + (TTTG)n Simple_repeat 2 24**

**161882554 161882877 C L1M2 LINE/L1 2907 2585**

**161882878 161883176 C AluY SINE/Alu 299 1**

**161883177 161885911 C L1M2 LINE/L1 2584 13**

**161885919 161885944 + AT_rich Low_complexity 1 26**

**161885945 161886066 C AluY SINE/Alu 122 1**

**161886179 161886485 C AluSx SINE/Alu 297 1**

**161886872 161887046 C MIRb SINE/MIR 201 26**

**161887972 161888194 + MIRb SINE/MIR 23 252**

**161888434 161888755 C L2 LINE/L2 3406 3025**

**161889081 161889328 + L1M4b LINE/L1 46 304**

**161889414 161889605 + L1M4b LINE/L1 762 947**

**161890099 161890324 + L1M4b LINE/L1 1205 1449**

**161890327 161891261 C L1HS LINE/L1 937 4 ( L1HS_5_93c )**

**161891262 161892684 + L1M3 LINE/L1 2624 4076**

**161892685 161892999 + AluSg SINE/Alu 1 308**

**161893000 161893054 + L1M3 LINE/L1 4077 4091**

**161893055 161893430 C L1MA5A LINE/L1 6287 5912**

**161893431 161893926 + L1M3 LINE/L1 4092 4598**

**161893924 161894487 + L1M4 LINE/L1 3816 4487**

**161894547 161894697 C L1M5 LINE/L1 5379 5217**

**161894740 161894817 + L2 LINE/L2 2947 3032**

**161894818 161895106 + AluY SINE/Alu 1 305**

**161895107 161895477 + L2 LINE/L2 3033 3410**

**Ortholog in Chimp 164654248-164660125 Minus Nscore 1.70**

**N positions 164655385-164655484;**

**164645008 164645318 C L1MA4A LINE/L1 6299 5984**

**164645321 164646791 C L1M2 LINE/L1 4026 2585**

**164646792 164647090 C AluY SINE/Alu 299 1**

**164647091 164649817 C L1M2 LINE/L1 2584 13**

**164649840 164649969 C AluY SINE/Alu 130 1**

**164650083 164650391 C AluSx SINE/Alu 300 1**

**164650657 164650769 C MER117 DNA/MER1_type 169 55**

**164650802 164650976 C MIRb SINE/MIR 201 26**

**164651902 164652124 + MIRb SINE/MIR 23 252**

**164652360 164652681 C L2 LINE/L2 3406 3025**

**164653007 164653254 + L1M4b LINE/L1 46 304**

**164653340 164653531 + L1M4b LINE/L1 762 947**

**164654022 164654264 + L1M4b LINE/L1 1205 1467**

**164654307 164654633 + L1M4b LINE/L1 1587 3495**

**164654635 164655382 C L1PA2 LINE/L1 6032 5284**

**164655485 164660125 C L1PA2 LINE/L1 4646 4**

**164660126 164661553 + L1M4b LINE/L1 3484 4936**

**164661554 164661871 + AluSg SINE/Alu 1 310**

**164661872 164661914 + L1M4b LINE/L1 4937 4956**

**164661915 164662303 C L1MA5A LINE/L1 6294 5911**

**164662304 164662798 + L1M4b LINE/L1 4957 5458**

**164662796 164663189 + L1M4 LINE/L1 3816 4230**

**164663209 164663359 + L1M4 LINE/L1 4334 4487**

**164663611 164663689 + L2 LINE/L2 2946 3032**

**164663690 164663959 + AluY SINE/Alu 1 295**

**164663960 164664331 + L2 LINE/L2 3033 3410**

**_________________________________________________________________________**

**L1HS_6_19c 50719695-50724460 C_INTER_RMD_M_DISRUPTED**

**50710554 50711127 + L1MCc LINE/L1 5122 6930**

**50711138 50711283 + GA-rich Low_complexity 1 147**

**50711965 50712203 C L1MA5 LINE/L1 5945 5705**

**50712204 50712418 + L1MA5 LINE/L1 6083 6297**

**50712422 50712615 + MIRb SINE/MIR 60 260**

**50714737 50714813 C HAL1 LINE/L1 833 757**

**50716630 50716834 + MLT1J2 LTR/MaLR 241 448**

**50716978 50717054 C MIRb SINE/MIR 253 183**

**50718414 50718717 + L1PB4 LINE/L1 5846 6153**

**50718738 50719035 + AluY SINE/Alu 1 297**

**50719306 50719679 C L1PA6 LINE/L1 6143 5770 (R2)**

**50719695 50724460 C L1HS LINE/L1 6155 1427 ( L1HS_6_19c ) (R1)**

**50724608 50724950 + THE1B LTR/MaLR 5 362**

**50724953 50726539 + THE1B-int LTR/MaLR 1 1580**

**50726540 50726900 + THE1B LTR/MaLR 1 361**

**50726933 50727909 C L2 LINE/L2 1225 223**

**50730160 50730302 + L3b LINE/CR1 4109 4261**

**50730557 50730973 + MSTB LTR/MaLR 4 426**

**50731112 50731344 C MLT1J1 LTR/MaLR 432 205**

**50732178 50732206 + (TTTTTG)n Simple_repeat 2 31**

**50732274 50732334 + L2 LINE/L2 3350 3419**

**50733392 50733491 C L2 LINE/L2 3364 3260**

**Ortholog in Chimp 51790403-51794773 Minus Nscore 0.00**

**N positions**

**51780977 51781437 + L1MCc LINE/L1 5123 5508**

**51781434 51781549 + L1MA9 LINE/L1 6023 6135**

**51781560 51781701 + GA-rich Low_complexity 1 143**

**51782383 51782621 C L1MA5 LINE/L1 5945 5705**

**51782622 51782836 + L1MA5 LINE/L1 6083 6297**

**51782840 51783033 + MIRb SINE/MIR 60 260**

**51785438 51785514 C HAL1 LINE/L1 833 757**

**51787333 51787537 + MLT1J2 LTR/MaLR 241 448**

**51787681 51787757 C MIRb SINE/MIR 253 183**

**51789143 51789439 + L1PB4 LINE/L1 5851 6153**

**51789461 51789758 + AluY SINE/Alu 1 297**

**51790030 51794773 C L1PA6 LINE/L1 6142 1427 (R12)**

**51794921 51795263 + THE1B LTR/MaLR 5 362**

**51795267 51796853 + THE1B-int LTR/MaLR 2 1580**

**51796854 51797213 + THE1B LTR/MaLR 1 361**

**51797246 51798265 C L2 LINE/L2 1225 178**

**51800474 51800616 + L3b LINE/CR1 4109 4261**

**51800871 51801286 + MSTB LTR/MaLR 4 426**

**51801425 51801657 C MLT1J1 LTR/MaLR 432 205**

**51802570 51802630 + L2 LINE/L2 3350 3419**

**51803688 51803787 C L2 LINE/L2 3364 3260**

**_________________________________________________________________________**

**L1HS_6_38 75251034-75255225 C_INTER_RMD_M_DISRUPTED**

**75241916 75242367 + L1PA13 LINE/L1 5708 6159**

**75242371 75242395 + AT_rich Low_complexity 1 25**

**75242558 75242632 + L2 LINE/L2 3300 3378**

**75242675 75243050 C L2 LINE/L2 3419 3026**

**75243386 75243483 + L2 LINE/L2 3276 3405**

**75243739 75243779 + AT_rich Low_complexity 1 41**

**75244573 75244776 + MIR SINE/MIR 15 253**

**75244957 75245168 + L2 LINE/L2 3167 3402**

**75245272 75245326 + AT_rich Low_complexity 1 55**

**75245966 75246139 C HSAT4 Satellite/centr 178 1**

**75246718 75247363 + MER82 DNA/MER2_type 6 652**

**75247491 75247514 + AT_rich Low_complexity 1 24**

**75247705 75247752 + AT_rich Low_complexity 1 48**

**75248407 75248535 C MIR SINE/MIR 170 14**

**75248786 75248823 + (TG)n Simple_repeat 2 39**

**75249106 75249327 C MLT1B LTR/MaLR 344 137**

**75249330 75250437 C L1MC1 LINE/L1 6472 5412**

**75250439 75250726 C L1MC1 LINE/L1 3907 3597**

**75250703 75250966 C L1MC1 LINE/L1 1588 1305**

**75250968 75251064 + L1HS LINE/L1 2 98**

**75251034 75255225 + L1HS LINE/L1 578 4792 ( L1HS_6_38 ) (R1)**

**75255237 75256021 + SVA Other 631 1422 (D)**

**75256022 75257275 + L1PA3 LINE/L1 4906 6155 (R2)**

**75257276 75258582 C L1MC1 LINE/L1 1318 1**

**75260235 75260275 + (TAGA)n Simple_repeat 4 45**

**75260472 75260832 C L1ME3A LINE/L1 6144 5756**

**75260906 75261133 + (TA)n Simple_repeat 1 231**

**75261140 75261574 C L1ME3A LINE/L1 5361 4927**

**75261573 75264191 C L1PA5 LINE/L1 6154 3557**

**75264190 75264448 C L1ME3A LINE/L1 4912 4655**

**Ortholog in Chimp 75328425-75332666 Plus Nscore 0.00**

**N positions**

**75319314 75319765 + L1PA13 LINE/L1 5708 6159**

**75319769 75319793 + AT_rich Low_complexity 1 25**

**75319956 75320030 + L2 LINE/L2 3300 3378**

**75320073 75320448 C L2 LINE/L2 3419 3029**

**75320780 75320877 + L2 LINE/L2 3276 3405**

**75321133 75321173 + AT_rich Low_complexity 1 41**

**75321967 75322169 + MIR SINE/MIR 15 253**

**75322349 75322560 + L2 LINE/L2 3166 3402**

**75322664 75322722 + AT_rich Low_complexity 1 59**

**75323239 75323361 C L1MEc LINE/L1 1906 1772**

**75323362 75323535 C HSAT4 Satellite/centr 178 1**

**75324118 75324763 + MER82 DNA/MER2_type 6 652**

**75324886 75324909 + AT_rich Low_complexity 1 24**

**75325100 75325146 + AT_rich Low_complexity 1 47**

**75325803 75325931 C MIR SINE/MIR 170 14**

**75326180 75326215 + (TG)n Simple_repeat 2 37**

**75326476 75326499 + (T)n Simple_repeat 1 24**

**75326500 75326724 C MLT1B LTR/MaLR 344 137**

**75326727 75327833 C L1MC1 LINE/L1 6472 5412**

**75327835 75328117 C L1MC1 LINE/L1 3907 3597**

**75328116 75328357 C L1MC1 LINE/L1 1561 1305**

**75328360 75328455 + L1Pt LINE/L1 3 98**

**75328450 75333911 + L1PA3 LINE/L1 616 6032 (R)**

**75333916 75335228 C L1MC1 LINE/L1 1316 1**

**75336875 75336915 + (TAGA)n Simple_repeat 4 45**

**75337112 75337464 C L1ME3A LINE/L1 4849 4469**

**75337482 75337586 + (TATG)n Simple_repeat 3 108**

**75337593 75337768 + (TA)n Simple_repeat 1 177**

**75337775 75338206 C L1ME3A LINE/L1 4066 3632**

**75338205 75340780 C L1PA5 LINE/L1 6154 3557**

**75340779 75341029 C L1ME3A LINE/L1 3617 3359**

**75341038 75341151 C L1MB1 LINE/L1 6163 6044**

**75341368 75345986 C L1PA2 LINE/L1 6032 1358**

**_________________________________________________________________________**

**L1HS_6_41 79549565-79552974 C_INTER_RMD_M_DISRUPTED**

**79540570 79541416 + HAL1 LINE/L1 993 1920**

**79541617 79542065 C LTR16B LTR/ERVL 464 28**

**79542237 79542257 + AT_rich Low_complexity 1 21**

**79543453 79543692 C MLT1J LTR/MaLR 255 4**

**79544289 79544342 + AT_rich Low_complexity 1 54**

**79544766 79544800 + AT_rich Low_complexity 1 35**

**79545602 79545912 C L3 LINE/CR1 2292 1958**

**79546137 79546227 C MIR SINE/MIR 262 172**

**79546882 79549075 + L1PBa1 LINE/L1 1 2237**

**79549073 79549563 + L1PBa1 LINE/L1 3018 3508**

**79549565 79552974 + L1HS LINE/L1 2 3413 ( L1HS_6_41 )**

**79552972 79553040 + L1M5 LINE/L1 4324 4371**

**79553041 79553685 C L1PA8 LINE/L1 6162 5519**

**79553686 79554524 + L1M5 LINE/L1 4372 5280**

**79554525 79554571 C MLT2F LTR/ERVL 563 517**

**79554572 79554649 + (TA)n Simple_repeat 2 79**

**79554650 79554861 C MLT2F LTR/ERVL 616 376**

**79554890 79555345 C MLT1C LTR/MaLR 467 9**

**79555354 79555661 C MLT2F LTR/ERVL 327 1**

**79555662 79555883 + L1M5 LINE/L1 5285 5501**

**79556030 79556530 + MLT1D LTR/MaLR 7 505**

**79556819 79556947 + AluJo SINE/Alu 2 130**

**79556962 79559065 + L1PA13 LINE/L1 4047 6156**

**79559066 79559221 + AluJo SINE/Alu 129 293**

**79559282 79559591 C AluSx SINE/Alu 309 2**

**79560053 79560114 + MER91C DNA/Tip100 40 105**

**79560390 79560768 C L1PREC2 LINE/L1 5721 5340**

**79560772 79561566 + L1PA3 LINE/L1 5358 6152**

**79561575 79561718 C L1PA13 LINE/L1 5970 5826**

**79561720 79562057 + MER1B DNA/MER1_type 1 331**

**Ortholog in Chimp 79777832-79778610 Plus Nscore 0.00**

**N positions**

**79768833 79769679 + HAL1 LINE/L1 993 1920**

**79769880 79770328 C LTR16B LTR/ERVL 464 28**

**79770500 79770520 + AT_rich Low_complexity 1 21**

**79771715 79771954 C MLT1J LTR/MaLR 255 4**

**79772551 79772604 + AT_rich Low_complexity 1 54**

**79773831 79774180 C L3 LINE/CR1 2227 1849**

**79774404 79774494 C MIR SINE/MIR 262 172**

**79775149 79777342 + L1PBa1 LINE/L1 6 2487**

**79777340 79777831 + L1PBa1 LINE/L1 3268 3759**

**79777831 79778605 + L1Pt LINE/L1 1 772**

**79778608 79778989 C L1PA8 LINE/L1 5900 5519**

**79778991 79779836 + L1MD LINE/L1 4357 5280**

**79779837 79780139 C MLT2F LTR/ERVL 663 374**

**79780168 79780623 C MLT1C LTR/MaLR 467 9**

**79780632 79780939 C MLT2F LTR/ERVL 327 1**

**79780940 79781161 + L1M5 LINE/L1 5285 5501**

**79781308 79781808 + MLT1D LTR/MaLR 7 505**

**79781810 79781963 C MIR_Mars SINE/MIR 169 2**

**79782097 79782225 + AluJo SINE/Alu 2 130**

**79782240 79784343 + L1PA13 LINE/L1 4047 6156**

**79784344 79784499 + AluJo SINE/Alu 129 293**

**79784562 79784874 C AluSx SINE/Alu 312 2**

**79785336 79785397 + MER91C DNA/Tip100 40 105**

**79785673 79785946 C L1PREC2 LINE/L1 5721 5447**

**79786495 79786585 C L1P LINE/L1 6049 5956**

**79786584 79787396 + L1PA3 LINE/L1 5340 6152**

**79787405 79787548 C L1P LINE/L1 5970 5826**

**79787550 79787887 + MER1B DNA/MER1_type 1 331**

**_________________________________________________________________________**

**L1HS_6_71 117244380-117246108 C_INTER_RMD_M_DISRUPTED**

**117235506 117235694 C MER58C DNA/MER1_type 215 1**

**117235822 117235866 + T-rich Low_complexity 1 45**

**117235881 117235944 + MIRb SINE/MIR 8 74**

**117236764 117236962 + L2 LINE/L2 2833 3037**

**117238059 117238147 + L2 LINE/L2 3330 3418**

**117238264 117238391 + L2 LINE/L2 2931 3082**

**117238410 117238432 + AT_rich Low_complexity 1 23**

**117238578 117238658 + AT_rich Low_complexity 1 81**

**117238668 117240498 + L1MA4A LINE/L1 1092 2896**

**117240498 117242623 + L1PA6 LINE/L1 4024 6154**

**117242625 117243056 + L1MA4A LINE/L1 2878 3308**

**117243057 117243345 + AluSx SINE/Alu 1 289**

**117243346 117243688 + L1MA4A LINE/L1 3309 3655**

**117243693 117243936 C MSTA LTR/MaLR 428 184**

**117243939 117244310 C MSTA-int LTR/MaLR 1651 1274**

**117244380 117246108 + L1HS LINE/L1 1 1735 ( L1HS_6_71 ) (R1)**

**117246109 117246418 + AluYa5 SINE/Alu 1 310 (D)**

**117246419 117250728 + L1PA2 LINE/L1 1736 6031 (R2)**

**117250748 117250777 + (A)n Simple_repeat 1 30**

**117250778 117250943 C MSTA-int LTR/MaLR 723 534**

**117250944 117250969 + (TTTG)n Simple_repeat 2 28**

**117250970 117251505 C MSTA-int LTR/MaLR 533 1**

**117251506 117251922 C MSTA LTR/MaLR 428 1**

**117251923 117255206 + L1MA4A LINE/L1 3655 7002**

**Ortholog in Chimp 118793866-118795582 Plus Nscore 0.00 (118793866-118799869 Nscore 8.31)**

**N positions**

**118784844 118784907 + MIRb SINE/MIR 8 74**

**118785727 118786019 + L2 LINE/L2 2833 3165**

**118787239 118787327 + L2 LINE/L2 3330 3418**

**118787927 118787953 + AT_rich Low_complexity 1 27**

**118788095 118788175 + AT_rich Low_complexity 1 81**

**118788185 118790008 + L1MA4A LINE/L1 1092 2896**

**118790008 118792110 + L1PA6 LINE/L1 4024 6154**

**118792112 118792543 + L1MA4A LINE/L1 2878 3308**

**118792544 118792832 + AluSx SINE/Alu 1 289**

**118792833 118793175 + L1MA4A LINE/L1 3309 3655**

**118793180 118793423 C MSTA LTR/MaLR 428 184**

**118793426 118793796 C MSTA-int LTR/MaLR 1651 1274**

**118793866 118798663 + L1Pt LINE/L1 1 4805 (R)**

**118799167 118799869 + L1PA2 LINE/L1 5453 6154 (R) BROKEN DUE TO Ns**

**118799888 118799907 + (A)n Simple_repeat 1 20**

**118799908 118800631 C MSTA-int LTR/MaLR 723 1**

**118800632 118801048 C MSTA LTR/MaLR 428 1**

**118801049 118804331 + L1MA4A LINE/L1 3655 7002**

**118804410 118804517 + L2 LINE/L2 3302 3412**

**_________________________________________________________________________**

**L1HS_7_19c 43421104-43423176 M_INTRA_RMD**

**43412052 43412488 + LTR47B LTR/ERVL 1 443**

**43412760 43413040 + AluSx SINE/Alu 1 290**

**43414557 43414831 + AluJb SINE/Alu 33 304**

**43414929 43415070 + MIRb SINE/MIR 2 140**

**43415829 43415947 C MIRb SINE/MIR 129 3**

**43416313 43416661 C MLT1A LTR/MaLR 335 1**

**43416679 43418549 C L1MEb LINE/L1 3560 1698**

**43418550 43418584 + (TTTA)n Simple_repeat 2 36**

**43418588 43421102 C L1PA2 LINE/L1 6144 3630 (R12)**

**43421104 43423176 C L1HS LINE/L1 2076 3 ( L1HS_7_19c ) (R12)**

**43423178 43423473 C AluSq SINE/Alu 297 1**

**43423474 43423705 C L1MEb LINE/L1 1618 1402**

**43423706 43423827 + FLAM_A SINE/Alu 1 123**

**43423828 43424005 C L1MEb LINE/L1 1401 1232**

**43424006 43424444 C MLT1C LTR/MaLR 467 1**

**43424445 43425442 C L1MEb LINE/L1 1231 156**

**43425672 43425835 + L1M5 LINE/L1 3826 3989**

**43425836 43426424 C LTR10C LTR/ERV1 591 1**

**Ortholog in Chimp 40791320-40794823 Plus Nscore 0.00**

**N positions**

**40788081 40788669 + LTR10C LTR/ERV1 1 591**

**40788670 40788830 C L1M5 LINE/L1 3989 3829**

**40789063 40790060 + L1MEb LINE/L1 156 1231**

**40790061 40790498 + MLT1C LTR/MaLR 1 467**

**40790499 40790667 + L1MEb LINE/L1 1232 1401**

**40790668 40790789 C FLAM_A SINE/Alu 123 1**

**40790790 40790994 + L1MEb LINE/L1 1402 1591**

**40791022 40791318 + AluSq SINE/Alu 1 297**

**40791320 40797334 + L1PA2 LINE/L1 3 6021 (R1,R2)**

**40797338 40797368 + (TAAA)n Simple_repeat 2 32**

**40797369 40799233 + L1MEb LINE/L1 1698 3560**

**40799357 40799658 + MLT1A LTR/MaLR 26 335**

**40800015 40800142 + MIRb SINE/MIR 6 129**

**40800900 40801041 C MIRb SINE/MIR 140 2**

**40801139 40801450 C AluJb SINE/Alu 304 1**

**40802954 40803235 C AluSq SINE/Alu 292 1**

**40803507 40803943 C LTR47B LTR/ERVL 443 1**

**_________________________________________________________________________**

**L1HS_7_21 46826880-46831780 C_INTER_RMD_M_DISRUPTED**

**46818889 46818917 + AT_rich Low_complexity 1 29**

**46821269 46821319 + (GAAA)n Simple_repeat 2 52**

**46821522 46821834 + AluSq SINE/Alu 1 312**

**46821891 46821935 + AT_rich Low_complexity 1 45**

**46822001 46822145 C MER5A1 DNA/MER1_type 152 7**

**46822187 46822323 + MER58A DNA/MER1_type 30 167**

**46822483 46822875 + MLT1A LTR/MaLR 9 374**

**46823096 46823179 + GA-rich Low_complexity 2 81**

**46823238 46823455 + L1MC4 LINE/L1 1207 1421**

**46823493 46823578 + MER94 DNA/AcHobo 49 134**

**46823581 46824011 + L1MC4 LINE/L1 1498 1939**

**46824184 46824580 C LTR16C LTR/ERVL 491 81**

**46824639 46824676 + (TA)n Simple_repeat 2 39**

**46824764 46824845 + L1MDa LINE/L1 8 88**

**46824846 46824904 + (TA)n Simple_repeat 1 59**

**46824905 46825910 + L1MDa LINE/L1 89 1212**

**46825911 46826879 C LTR13A LTR/ERVK 966 1**

**46826880 46831780 + L1HS LINE/L1 1213 6545 ( L1HS_7_21 ) (R1)**

**46831778 46832208 + L1MDa LINE/L1 1614 2076 (R2)**

**46832237 46833223 + L1M LINE/L1 127 1161**

**46833223 46833376 + L1M LINE/L1 2530 2673**

**46833395 46833598 + L1PB1 LINE/L1 3 200**

**46833599 46837052 + L1PB1 LINE/L1 634 4462**

**46837058 46837152 + L1PB1 LINE/L1 6402 6519**

**46837153 46837209 + (TAGA)n Simple_repeat 4 60**

**46837210 46837554 + L1PB1 LINE/L1 6520 6865**

**46837555 46837588 + AT_rich Low_complexity 1 34**

**46837672 46837943 + L1M LINE/L1 2714 2994**

**46838021 46838250 + L1M LINE/L1 5453 5688**

**46838251 46838961 + MER44D DNA/MER2_type 1 704**

**46838962 46839815 + L1MC3 LINE/L1 5271 6116**

**46839837 46839920 C MER34 LTR/ERV1 537 451**

**46839942 46840145 + THE1B LTR/MaLR 1 212**

**46840153 46840496 C MER34-int LTR/ERV1 968 640**

**46840502 46841551 + L1MC3 LINE/L1 6137 7214**

**Ortholog in Chimp 47596826-47597521 Plus Nscore 0.00**

**N positions**

**47591273 47591312 + (A)n Simple_repeat 1 40**

**47591513 47591827 + AluSq SINE/Alu 1 313**

**47591902 47591944 + AT_rich Low_complexity 1 43**

**47592010 47592154 C MER5A1 DNA/MER1_type 152 7**

**47592196 47592332 + MER58A DNA/MER1_type 30 167**

**47592492 47592884 + MLT1A LTR/MaLR 9 374**

**47593004 47593134 + MER58C DNA/MER1_type 6 132**

**47593145 47593188 + (GAA)n Simple_repeat 1 42**

**47593247 47593464 + L1MC4 LINE/L1 1207 1421**

**47593502 47593587 + MER94 DNA/AcHobo 49 134**

**47593590 47594020 + L1MC4 LINE/L1 1498 1939**

**47594193 47594595 C LTR16C LTR/ERVL 491 72**

**47594645 47594669 + AT_rich Low_complexity 1 25**

**47594749 47595856 + L1M LINE/L1 8 1212**

**47595857 47596825 C LTR13A LTR/ERVK 966 1**

**47596826 47597543 + L1M LINE/L1 1213 1968 (R12)**

**47597527 47597730 + L1PB1 LINE/L1 3 200**

**47597731 47601211 + L1PB1 LINE/L1 634 4462**

**47601217 47601311 + L1PB1 LINE/L1 6402 6519**

**47601312 47601375 + (TAGA)n Simple_repeat 4 68**

**47601376 47601721 + L1PB1 LINE/L1 6520 6865**

**47601722 47601757 + AT_rich Low_complexity 1 36**

**47601871 47602111 + L1M LINE/L1 2722 2967**

**47602170 47602194 + (CA)n Simple_repeat 1 25**

**47602195 47602424 + L1M LINE/L1 5426 5661**

**47602425 47603135 + MER44D DNA/MER2_type 1 704**

**47603136 47603990 + L1MC3 LINE/L1 5271 6116**

**47604012 47604095 C MER34 LTR/ERV1 537 451**

**47604117 47604321 + THE1B LTR/MaLR 1 212**

**47604329 47604672 C MER34-int LTR/ERV1 968 640**

**47604678 47605727 + L1MC3 LINE/L1 6137 7214**

**47605792 47605899 + L1MC3 LINE/L1 7201 7299**

**47605923 47606576 + MER21C LTR/ERV1 120 795**

**_________________________________________________________________________**

**L1HS_7_67 140106910-140108875 C_INTER_RMD_M_DISRUPTED**

**140097809 140097976 C MIR3 SINE/MIR 199 3**

**140098220 140098460 C Charlie1 DNA/MER1_type 2759 2504**

**140098484 140098593 C FLAM_A SINE/Alu 119 7**

**140098607 140098724 C Charlie1 DNA/MER1_type 2483 2366**

**140101301 140101400 C AluSg/x SINE/Alu 282 183**

**140101402 140101495 + L2 LINE/L2 3321 3414**

**140103215 140103393 C L3 LINE/CR1 4010 3831**

**140103568 140103856 C AluSc SINE/Alu 289 1**

**140104127 140104281 C L3 LINE/CR1 3468 3303**

**140104731 140105013 C AluJo SINE/Alu 293 11**

**140105047 140105641 C L1ME2 LINE/L1 6153 5487**

**140105662 140105954 C AluSp SINE/Alu 294 1**

**140106002 140106074 C L2 LINE/L2 2209 2122**

**140106075 140106225 C MER5A1 DNA/MER1_type 160 1**

**140106226 140106399 C L2 LINE/L2 2121 1980**

**140106400 140106708 + AluSx SINE/Alu 1 309**

**140106709 140106877 C L2 LINE/L2 1979 1817**

**140106910 140108875 + L1HS LINE/L1 1116 3083 ( L1HS_7_67 ) (R1)**

**140108876 140109778 C SVA Other 1386 508 (D)**

**140109779 140112719 + L1PA3 LINE/L1 3203 6153 (R2)**

**140112752 140112822 C L1MC4 LINE/L1 8001 7931**

**140112860 140113118 C AluJb SINE/Alu 292 1**

**140113140 140113259 C FLAM_C SINE/Alu 122 3**

**140113269 140113572 C AluJb SINE/Alu 304 1**

**140113588 140113956 C L2 LINE/L2 1767 1338**

**140114020 140114178 + MIR SINE/MIR 79 240**

**140114394 140114510 + L1ME4a LINE/L1 5974 6099**

**140114620 140114765 + MSTB2 LTR/MaLR 1 185**

**140114766 140115065 C AluJo SINE/Alu 296 1**

**140115066 140115342 + MSTB2 LTR/MaLR 186 456**

**140115352 140115646 C AluSx SINE/Alu 294 1**

**140115819 140115973 C L1MC4a LINE/L1 5893 5723**

**140116007 140116141 C AluJb SINE/Alu 136 3**

**140116310 140116421 C L1MC4a LINE/L1 5521 5405**

**140116923 140117228 + AluSx SINE/Alu 1 311**

**140117468 140117760 C AluJb SINE/Alu 297 3**

**Ortholog in Chimp 141421553-141423507 Plus Nscore 0.00 (141421553-141426103 - Nscore 16.3)**

**N positions**

**141412459 141412626 C MIR3 SINE/MIR 199 3**

**141412870 141413131 C Charlie1 DNA/MER1_type 2759 2491**

**141413132 141413247 C FLAM_A SINE/Alu 119 1**

**141413248 141413372 C Charlie1 DNA/MER1_type 2490 2366**

**141415953 141416052 C AluSg/x SINE/Alu 282 183**

**141416054 141416147 + L2 LINE/L2 3321 3414**

**141416748 141416822 C L2 LINE/L2 1710 1632**

**141417839 141418046 C L3 LINE/CR1 3963 3756**

**141418221 141418509 C AluSc SINE/Alu 289 1**

**141418780 141418934 C L3 LINE/CR1 3393 3228**

**141419382 141419664 C AluJo SINE/Alu 293 11**

**141419698 141420288 C L1ME2 LINE/L1 6153 5487**

**141420309 141420601 C AluSp SINE/Alu 294 1**

**141420629 141420721 C L2 LINE/L2 2228 2122**

**141420722 141420872 C MER5A1 DNA/MER1_type 160 1**

**141420873 141421046 C L2 LINE/L2 2121 1980**

**141421047 141421351 + AluSx SINE/Alu 1 304**

**141421352 141421520 C L2 LINE/L2 1979 1817**

**141421553 141424433 + L1Pt LINE/L1 1117 3994 (R)**

**141425379 141426103 + L1PA3 LINE/L1 5415 6153 (R) - BROKEN DUE TO Ns**

**141426136 141426206 C L1MC4 LINE/L1 8001 7931**

**141426245 141426499 C AluJb SINE/Alu 291 4**

**141426524 141426643 C FLAM_C SINE/Alu 122 3**

**141426653 141426956 C AluJb SINE/Alu 304 1**

**141426972 141427340 C L2 LINE/L2 1767 1338**

**141427404 141427562 + MIR SINE/MIR 79 240**

**141427778 141427894 + L1ME4a LINE/L1 5974 6099**

**141428003 141428148 + MSTB2 LTR/MaLR 1 185**

**141428149 141428448 C AluJo SINE/Alu 296 1**

**141428449 141428706 + MSTB2 LTR/MaLR 186 452**

**141428757 141428966 + MIR SINE/MIR 28 240**

**141429182 141429298 + L1ME4a LINE/L1 5974 6099**

**141429407 141429552 + MSTB2 LTR/MaLR 1 185**

**141429553 141429853 C AluJo SINE/Alu 296 1**

**141429854 141430130 + MSTB2 LTR/MaLR 186 456**

**141430140 141430436 C AluSx SINE/Alu 296 1**

**141430610 141430763 C L1ME3B LINE/L1 5894 5725**

**141430797 141430931 C AluJb SINE/Alu 136 3**

**141431100 141431211 C L1ME3B LINE/L1 5521 5405**

**141431709 141432016 + AluSx SINE/Alu 1 311**

**141432259 141432551 C AluJb SINE/Alu 297 3**

**_________________________________________________________________________**

**L1HS_7_73 144889523-144892621 C_DISRUPTED_M_INTER_RMD**

**144883301 144883656 C L1ME3B LINE/L1 5385 5027**

**144884223 144884596 + MLT2C1 LTR/ERVL 1 397**

**144884664 144884869 C HAL1 LINE/L1 1054 844**

**144885344 144885877 C L1MA8 LINE/L1 6291 5769**

**144885879 144886493 C L1M3e LINE/L1 638 5**

**144887193 144889526 C L1PA3 LINE/L1 6155 3824**

**144889523 144892621 + L1HS LINE/L1 594 3697 ( L1HS_7_73 )**

**144892971 144892991 + AT_rich Low_complexity 1 21**

**144893526 144893822 C AluSg SINE/Alu 297 1**

**144894215 144894254 + AT_rich Low_complexity 1 40**

**144894288 144894582 + L2 LINE/L2 2930 3251**

**144894589 144894811 C MER58A DNA/MER1_type 224 1**

**144895983 144896569 C MLT1E2 LTR/MaLR 617 1**

**144896815 144896842 + AT_rich Low_complexity 1 28**

**144896885 144897000 + FLAM_C SINE/Alu 3 119**

**144897002 144897029 + (TAA)n Simple_repeat 3 31**

**144897411 144897773 C MER46B DNA/MER2_type 231 1**

**144897782 144897909 + L3 LINE/CR1 3995 4133**

**144898014 144898034 + AT_rich Low_complexity 1 21**

**144898110 144898305 C L2 LINE/L2 3412 3251**

**Ortholog in Chimp 146100140-146103474 Plus Nscore 0.33**

**N positions 146102292-146102292; 146102372-146102381;**

**146093889 146094279 C L1M LINE/L1 5426 5027**

**146094846 146095219 + MLT2C1 LTR/ERVL 1 397**

**146095316 146095492 C HAL1 LINE/L1 1031 844**

**146095968 146096495 C L1MA8 LINE/L1 6291 5769**

**146096497 146097111 C L1M3e LINE/L1 638 5**

**146097812 146100143 C L1PA3 LINE/L1 6155 3824**

**146100140 146103474 + L1Pt LINE/L1 595 3697 (OCCUPIED)**

**146103826 146103846 + AT_rich Low_complexity 1 21**

**146104375 146104394 + (TTA)n Simple_repeat 2 21**

**146104395 146104688 C AluSg SINE/Alu 294 1**

**146105081 146105120 + AT_rich Low_complexity 1 40**

**146105154 146105405 + L2 LINE/L2 2930 3198**

**146105455 146105677 C MER58A DNA/MER1_type 224 1**

**146106849 146107438 C MLT1E2 LTR/MaLR 617 1**

**146107684 146107729 + AT_rich Low_complexity 1 46**

**146107754 146107869 + FLAM_C SINE/Alu 3 119**

**146107871 146107907 + (TAA)n Simple_repeat 3 40**

**146108289 146108651 C MER46B DNA/MER2_type 231 1**

**146108660 146108787 + L3 LINE/CR1 3995 4133**

**146108895 146108915 + AT_rich Low_complexity 1 21**

**146108991 146109186 C L2 LINE/L2 3412 3251**

**_________________________________________________________________________**

**L1HS_8_5 16572000-16575946 M_INTRA_RMD**

**16566302 16566502 + MLT1E1A LTR/MaLR 27 254**

**16566503 16566812 C AluJb SINE/Alu 312 5**

**16566813 16567197 + MLT1E1A LTR/MaLR 255 697**

**16567212 16567334 C L2 LINE/L2 2152 2017**

**16567716 16567744 + (CA)n Simple_repeat 2 30**

**16567908 16567949 + (CA)n Simple_repeat 1 42**

**16569139 16569247 + GA-rich Low_complexity 2 108**

**16569249 16569585 C MLT2C1 LTR/ERVL 349 1**

**16570504 16570727 + AluJo SINE/Alu 12 237**

**16571373 16571393 + AT_rich Low_complexity 1 21**

**16571602 16571719 + L1PB3 LINE/L1 6032 6147**

**16571741 16571911 + L1ME4a LINE/L1 5709 5887**

**16572000 16575946 + L1HS LINE/L1 2 3954 ( L1HS_8_5 ) (R12)**

**16575937 16577170 + L1PA2 LINE/L1 4921 6154 (R12)**

**16577186 16577219 + AT_rich Low_complexity 1 34**

**16577585 16577662 + (CA)n Simple_repeat 1 78**

**16578847 16579178 C MLT1J2 LTR/MaLR 380 2**

**16579206 16579728 + L1ME4a LINE/L1 5296 5840**

**16580447 16580659 C MIR SINE/MIR 230 15**

**16581001 16581027 + (TG)n Simple_repeat 2 28**

**16581908 16581942 + AT_rich Low_complexity 1 35**

**16582417 16582535 + MER5B DNA/MER1_type 4 127**

**Ortholog in Chimp 12834553-12839345 Plus Nscore 0.00**

**N positions**

**12829352 12829737 + MLT1E1A LTR/MaLR 255 697**

**12829752 12829874 C L2 LINE/L2 2152 2017**

**12830454 12830489 + (CA)n Simple_repeat 2 37**

**12831696 12831804 + GA-rich Low_complexity 2 108**

**12831806 12832142 C MLT2C1 LTR/ERVL 349 1**

**12833061 12833284 + AluJo SINE/Alu 12 237**

**12834154 12834271 + L1PB3 LINE/L1 6032 6147**

**12834293 12834464 + L1ME4a LINE/L1 5709 5887**

**12834553 12840566 + L1PA2 LINE/L1 2 6032 (R1,R2)**

**12840570 12840603 + AT_rich Low_complexity 1 34**

**12840980 12841059 + (CA)n Simple_repeat 1 80**

**12842237 12842574 C MLT1J2 LTR/MaLR 325 2**

**12842752 12842781 + AT_rich Low_complexity 1 30**

**12842815 12843124 + L1ME4a LINE/L1 5523 5840**

**12843815 12844053 C MIR SINE/MIR 272 15**

**12844395 12844415 + (TG)n Simple_repeat 2 22**

**12845296 12845330 + AT_rich Low_complexity 1 35**

**12845801 12845919 + MER5B DNA/MER1_type 4 127**

**12845920 12845946 + AT_rich Low_complexity 1 27**

**12846703 12846948 + AluSx SINE/Alu 58 303**

**12846967 12847433 C MLT1C LTR/MaLR 467 1**

**12847538 12847654 + MIRb SINE/MIR 92 221**

**12847674 12847841 C MIR3 SINE/MIR 163 1**

**12848065 12848103 + (TG)n Simple_repeat 1 38**

**_________________________________________________________________________**

**L1HS_8_19 40837918-40839258 C_INTER_RMD_M_DISRUPTED**

**40828842 40828965 C L1MA5A LINE/L1 6045 5929**

**40828966 40829014 + L1MD3 LINE/L1 7942 7988**

**40829016 40829950 C Charlie1a DNA/MER1_type 1245 302**

**40829951 40830255 + AluSg SINE/Alu 1 304**

**40830256 40830519 C Charlie1a DNA/MER1_type 301 51**

**40830520 40830891 C MSTB LTR/MaLR 426 1**

**40830892 40830939 C Charlie1a DNA/MER1_type 50 3**

**40832356 40832666 C AluJo SINE/Alu 298 1**

**40833906 40834063 + FAM SINE/Alu 1 44**

**40834137 40834832 + L1ME2 LINE/L1 5431 6155**

**40836130 40836165 + (CA)n Simple_repeat 2 37**

**40836667 40836820 + MIR SINE/MIR 4 172**

**40837641 40837909 + L2 LINE/L2 3116 3415**

**40837918 40839258 + L1HS LINE/L1 4815 6154 ( L1HS_8_19 ) (R1)**

**40839275 40839660 + L1PA5 LINE/L1 5770 6154 (R2)**

**40839665 40840303 C L2 LINE/L2 3066 2401**

**40840605 40840727 + L2 LINE/L2 3255 3373**

**40842289 40842462 C MIRb SINE/MIR 187 11**

**40843467 40843546 + (CATA)n Simple_repeat 4 84**

**40843667 40843831 + L2 LINE/L2 2561 2765**

**40843832 40844043 C MER30 DNA/MER1_type 230 1**

**40844044 40844523 + L2 LINE/L2 2766 3264**

**40844524 40844619 + L1PA8 LINE/L1 6077 6172**

**40844620 40844752 + L2 LINE/L2 3265 3401**

**40846090 40846254 + MIRb SINE/MIR 34 200**

**40846268 40846334 + L2 LINE/L2 3306 3385**

**40846422 40846538 C L3b LINE/CR1 4254 4132**

**40846627 40847165 + MER34C_ LTR/ERV1 1 585**

**Ortholog in Chimp 37586064-37587019 Plus Nscore 0.00**

**N positions**

**37576963 37577086 C L1MA5A LINE/L1 6045 5929**

**37577087 37577135 + L1MD3 LINE/L1 7942 7990**

**37577137 37578073 C Charlie1a DNA/MER1_type 1245 302**

**37578074 37578378 + AluSg SINE/Alu 1 304**

**37578379 37578644 C Charlie1a DNA/MER1_type 301 51**

**37578645 37579016 C MSTB LTR/MaLR 426 1**

**37579017 37579064 C Charlie1a DNA/MER1_type 50 3**

**37579731 37579756 + (CA)n Simple_repeat 1 26**

**37580487 37580798 C AluJo SINE/Alu 299 1**

**37582038 37582200 + FAM SINE/Alu 1 49**

**37582281 37582976 + L1ME2 LINE/L1 5431 6155**

**37584274 37584311 + (CA)n Simple_repeat 2 39**

**37584813 37584966 + MIRb SINE/MIR 4 176**

**37585787 37586055 + L2 LINE/L2 3116 3415**

**37586092 37587404 + L1PA5 LINE/L1 4836 6153 (R12)**

**37587408 37588045 C L2 LINE/L2 3066 2401**

**37588347 37588458 + L2 LINE/L2 3255 3364**

**37590031 37590204 C MIRb SINE/MIR 187 11**

**37591207 37591282 + (CATA)n Simple_repeat 4 80**

**37591403 37591566 + L2 LINE/L2 2561 2765**

**37591567 37591783 C MER30 DNA/MER1_type 230 1**

**37591784 37592263 + L2 LINE/L2 2766 3264**

**37592264 37592359 + L1PA8 LINE/L1 6077 6172**

**37592360 37592495 + L2 LINE/L2 3265 3401**

**37593821 37594027 + MIRb SINE/MIR 23 226**

**37594011 37594077 + L2 LINE/L2 3306 3385**

**37594174 37594282 C L3b LINE/CR1 4246 4132**

**37594371 37594909 + MER34C_ LTR/ERV1 1 585**

**_________________________________________________________________________**

**L1HS_8_35 90592964-90595078 C_DISRUPTED_M_INTER_RMD**

**90586949 90587009 + L1ME3B LINE/L1 1443 1504**

**90587312 90587693 + L1ME3B LINE/L1 1980 2513**

**90587685 90587761 + L1ME3B LINE/L1 4899 4975**

**90587803 90588820 + L1ME3B LINE/L1 5097 6161**

**90588821 90589116 + AluJo SINE/Alu 1 296**

**90589117 90589909 + L1ME3B LINE/L1 6165 7007**

**90590359 90590668 + AluYa5 SINE/Alu 1 310**

**90590699 90590872 + L2 LINE/L2 2980 3160**

**90590950 90592968 C L1PA3 LINE/L1 6154 4128**

**90592964 90595078 + L1HS LINE/L1 1171 3285 ( L1HS_8_35 ) (R12)**

**90595083 90595265 + L2 LINE/L2 3241 3417**

**90595315 90595362 + AT_rich Low_complexity 1 48**

**90596537 90596560 + (T)n Simple_repeat 1 24**

**90596798 90596922 C L2 LINE/L2 3417 3289**

**90597004 90598009 + L2 LINE/L2 1753 2860**

**90598269 90598476 C L2 LINE/L2 2324 2107**

**90598570 90599001 + Tigger2a DNA/MER2_type 2 433**

**90599244 90599396 C L1ME3B LINE/L1 5923 5767**

**90599397 90599742 C MLT1A LTR/MaLR 374 1**

**90599743 90600207 C L1ME3B LINE/L1 5766 5283**

**90600281 90600730 C MLT1C LTR/MaLR 467 3**

**Ortholog in Chimp 88297893-88300710 Plus Nscore 0.39**

**N positions 88300002-88300011; 88300034-88300034;**

**88292182 88292558 + L1ME3B LINE/L1 1980 2507**

**88292556 88292632 + L1ME3B LINE/L1 4899 4975**

**88292674 88293691 + L1ME3B LINE/L1 5097 6161**

**88293692 88293987 + AluJo SINE/Alu 1 296**

**88293988 88294778 + L1ME3B LINE/L1 6165 7007**

**88295252 88295412 + L2 LINE/L2 2980 3150**

**88295502 88296045 C L1PA3 LINE/L1 6155 5610**

**88296404 88297897 C L1PA3 LINE/L1 5629 4128**

**88297893 88299944 + L1Pt LINE/L1 1172 3233 (R1)**

**88300012 88300710 + L1P1 LINE/L1 2711 3408 (R2)**

**88300715 88300897 + L2 LINE/L2 3241 3417**

**88300947 88300994 + AT_rich Low_complexity 1 48**

**88302115 88302141 + (TC)n Simple_repeat 1 27**

**88302175 88302207 + (TAGA)n Simple_repeat 1 33**

**88302457 88302581 C L2 LINE/L2 3417 3289**

**88303428 88303454 + (TC)n Simple_repeat 1 27**

**88303488 88303532 + (TAGA)n Simple_repeat 1 45**

**88303782 88303906 C L2 LINE/L2 3417 3289**

**88303991 88304995 + L2 LINE/L2 1753 2860**

**88305255 88305427 C L2 LINE/L2 2324 2142**

**88305557 88305988 + Tigger2a DNA/MER2_type 2 433**

**88306231 88306383 C L1ME3B LINE/L1 5923 5767**

**88306384 88306729 C MLT1A LTR/MaLR 374 1**

**_________________________________________________________________________**

**L1HS_8_50c 122411300-122416228 M_INTRA_RMD**

**122407492 122407551 C L2 LINE/L2 2012 1953**

**122407710 122408010 C AluSq SINE/Alu 302 1**

**122409019 122409053 + AT_rich Low_complexity 1 35**

**122409104 122409212 + MIR3 SINE/MIR 83 204**

**122409510 122409724 + MIR SINE/MIR 8 238**

**122410186 122410258 + LTR33A LTR/ERVL 441 523**

**122410347 122410435 + L2 LINE/L2 3112 3229**

**122410436 122410737 C AluSg SINE/Alu 301 1**

**122410738 122410928 + L2 LINE/L2 3230 3418**

**122410930 122411058 C MIR SINE/MIR 242 114**

**122411300 122416228 C L1HS LINE/L1 4932 13 ( L1HS_8_50c )**

**122417739 122418033 C AluSx SINE/Alu 301 3**

**122419233 122419408 C AluJb SINE/Alu 311 137**

**122419453 122419555 + MIR3 SINE/MIR 54 162**

**122421942 122422246 C LTR16B LTR/ERVL 464 98**

**122422363 122423080 C L1MA8 LINE/L1 6288 5563**

**122423566 122423613 C MER53 DNA 129 82**

**122423614 122423634 + (CAAA)n Simple_repeat 1 21**

**122423635 122423714 C MER53 DNA 81 2**

**122424221 122424341 C L2 LINE/L2 3419 3293**

**122424608 122424640 + AT_rich Low_complexity 1 33**

**122424794 122425120 C L2 LINE/L2 3378 3032**

**Ortholog in Chimp 120844736-120852156 Minus Nscore 0.00**

**N positions**

**120840924 120840984 C L2 LINE/L2 2012 1952**

**120841142 120841443 C AluSq SINE/Alu 302 1**

**120841712 120841797 + L3 LINE/CR1 1103 1187**

**120842450 120842484 + AT_rich Low_complexity 1 35**

**120842535 120842643 + MIR3 SINE/MIR 83 204**

**120842949 120843155 + MIR SINE/MIR 16 238**

**120843618 120843690 + LTR33A LTR/ERVL 441 523**

**120843868 120844173 C AluSg SINE/Alu 305 1**

**120844178 120844364 + L2 LINE/L2 3234 3419**

**120844366 120844494 C MIR SINE/MIR 242 114**

**120844650 120844771 C MIR_Mars SINE/MIR 141 22**

**120845381 120845408 + (CAAAA)n Simple_repeat 3 30**

**120845885 120846015 + MIRb SINE/MIR 21 153**

**120846051 120846100 + (CA)n Simple_repeat 1 50**

**120846113 120846142 + AT_rich Low_complexity 1 30**

**120846143 120852156 C L1PA3 LINE/L1 6029 13**

**120853667 120853961 C AluSx SINE/Alu 301 3**

**120855165 120855340 C AluJb SINE/Alu 311 137**

**120855385 120855487 + MIR3 SINE/MIR 54 162**

**120857875 120858037 C LTR16B LTR/ERVL 464 275**

**120858296 120859013 C L1MA8 LINE/L1 6288 5563**

**120859575 120859644 C MER53 DNA 70 1**

**120860151 120860271 C L2 LINE/L2 3419 3293**

**120860539 120860571 + AT_rich Low_complexity 1 33**

**120860743 120861055 C L2 LINE/L2 3356 3032**

**120861125 120861246 C L2 LINE/L2 2902 2768**

**_________________________________________________________________________**

**L1HS_9_5c 7853205-7857520 M_INTRA_RMD**

**7843823 7844272 C MER31B LTR/ERV1 466 1**

**7844273 7844309 + (TCCA)n Simple_repeat 1 38**

**7844826 7845081 C MIRb SINE/MIR 256 4**

**7845506 7845676 C MIRb SINE/MIR 222 48**

**7845797 7845982 + MER104 DNA/Tc2 3 179**

**7846229 7846528 + AluSq SINE/Alu 1 302**

**7846939 7847097 + MIRb SINE/MIR 64 193**

**7847516 7847596 + L2 LINE/L2 3282 3378**

**7848877 7849112 + MIRb SINE/MIR 3 239**

**7849114 7849152 + (TTCA)n Simple_repeat 1 39**

**7849445 7849632 + MER5A DNA/MER1_type 3 189**

**7850848 7850992 C L2 LINE/L2 3378 3229**

**7851706 7851939 + MIR SINE/MIR 41 262**

**7852020 7852285 + L1M5 LINE/L1 3959 4228**

**7852286 7852643 + Charlie3 DNA/MER1_type 24 2712**

**7852644 7852831 + L1M5 LINE/L1 4225 4432**

**7852890 7853195 + AluY SINE/Alu 1 303**

**7853205 7857520 C L1HS LINE/L1 4971 612 ( L1HS_9_5c ) (5' end of Twin primed L1)**

**7857505 7858562 + L1PA3 LINE/L1 5095 6154 (3 ' end)**

**7858630 7858755 C L1M5 LINE/L1 4742 4603**

**7859156 7859343 + MLT1J2 LTR/MaLR 1 209**

**7859550 7859757 + L1MEd LINE/L1 1025 1233**

**7860242 7860297 + AT_rich Low_complexity 1 56**

**7860344 7860504 + (TA)n Simple_repeat 1 170**

**7860514 7860585 + (ATATG)n Simple_repeat 4 75**

**7860611 7860716 + (TA)n Simple_repeat 1 110**

**7862206 7862276 + Charlie9 DNA/MER1_type 2 73**

**7862856 7863038 C MIRb SINE/MIR 200 1**

**7863797 7864111 C AluSx SINE/Alu 312 1**

**7865649 7866025 + THE1D LTR/MaLR 1 381**

**7866028 7867650 + MLT1D-int LTR/MaLR 2 1735**

**Ortholog in Chimp 7984340-7989203 Minus Nscore 4.26**

**N positions 7988151-7988357;**

**7975292 7975547 C MIRb SINE/MIR 256 4**

**7975648 7975668 + AT_rich Low_complexity 1 21**

**7975922 7976131 C MIRb SINE/MIR 260 48**

**7976252 7976437 + MER104 DNA/Tc2 3 179**

**7976684 7976982 + AluSq SINE/Alu 1 301**

**7977394 7977552 + MIRb SINE/MIR 64 193**

**7977971 7978051 + L2 LINE/L2 3282 3378**

**7980032 7980263 + MIRb SINE/MIR 7 239**

**7980265 7980299 + (TTCA)n Simple_repeat 1 35**

**7980589 7980755 + MER5A DNA/MER1_type 3 168**

**7981343 7981362 + (CA)n Simple_repeat 2 21**

**7981995 7982139 C L2 LINE/L2 3378 3229**

**7982853 7983086 + MIR SINE/MIR 41 262**

**7983167 7983432 + L1M5 LINE/L1 3959 4228**

**7983433 7983790 + Charlie3 DNA/MER1_type 24 2712**

**7983791 7983977 + L1M5 LINE/L1 4225 4432**

**7984036 7984366 + AluY SINE/Alu 1 328**

**7984372 7988148 C L1Pt LINE/L1 4970 1151**

**7988384 7989203 C L1Pt LINE/L1 1467 613 (OCCUPIED - complications due to Ns)**

**7989188 7990243 + L1PA3 LINE/L1 5095 6153**

**7990335 7990467 C L1M5 LINE/L1 4718 4559**

**7990836 7991023 + MLT1J2 LTR/MaLR 1 209**

**7991228 7991437 + L1MEd LINE/L1 1023 1233**

**7991922 7991974 + AT_rich Low_complexity 1 53**

**7992000 7992165 + (TA)n Simple_repeat 2 178**

**7992211 7992398 + (TATAA)n Simple_repeat 1 179**

**7992449 7992554 + (TA)n Simple_repeat 1 110**

**7993077 7993385 C AluY SINE/Alu 309 1**

**7994351 7994421 + Charlie9 DNA/MER1_type 2 73**

**7995003 7995189 C MIRb SINE/MIR 200 4**

**7995949 7996263 C AluSx SINE/Alu 312 1**

**7997802 7998178 + THE1D LTR/MaLR 1 381**

**7998181 7999802 + MLT1D-int LTR/MaLR 2 1735**

**_________________________________________________________________________**

**L1HS_9_25 67998937-68001980 C_DISRUPTED_M_INTER_RMD**

**67993046 67993138 + MER63D DNA/AcHobo 331 1061**

**67993188 67993455 + L2 LINE/L2 2858 3155**

**67993456 67993787 C MLT1D LTR/MaLR 505 70**

**67993827 67994103 + L2 LINE/L2 3151 3419**

**67994319 67994443 C FLAM_C SINE/Alu 125 1**

**67997512 67997628 + MARNA DNA/Mariner 94 220**

**67997659 67997852 + MIRb SINE/MIR 16 228**

**67998664 67998710 + (TTCA)n Simple_repeat 3 48**

**67998937 68001980 + L1HS LINE/L1 4 3047 ( L1HS_9_25 ) (R12)**

**68001996 68003998 C CER Satellite 2005 1**

**Ortholog in Chimp 136131508-136145140 Minus Nscore 1.29**

**N positions 136136565-136136740;**

**136123889 136127297 + CER Satellite 1 3397**

**136127918 136131492 + CER Satellite 1 3588**

**136131508 136132412 C L1P1 LINE/L1 3170 2266 (R2)**

**136132414 136133526 + L1PA4 LINE/L1 5034 6147**

**136133544 136133799 C L2 LINE/L2 3115 2800**

**136133800 136134041 + AluSx SINE/Alu 58 300**

**136134042 136134595 C L2 LINE/L2 2799 2213**

**136135323 136135468 C L2 LINE/L2 2944 2799**

**136136093 136136547 + L1P1 LINE/L1 4951 5403**

**136136755 136137952 + L1PA3 LINE/L1 4951 6155**

**136137964 136137984 + AT_rich Low_complexity 1 21**

**136139093 136139117 + AT_rich Low_complexity 1 25**

**136139123 136145138 C L1PA3 LINE/L1 6027 6 (R1)**

**136145367 136145413 + (TGAA)n Simple_repeat 3 48**

**136146225 136146418 C MIRb SINE/MIR 228 16**

**136146445 136146565 C MARNA DNA/Mariner 224 94**

**136147870 136147892 + (CA)n Simple_repeat 2 24**

**136147893 136147916 + (GA)n Simple_repeat 2 26**

**136149322 136149347 + (A)n Simple_repeat 1 26**

**136149646 136149769 + FLAM_C SINE/Alu 2 125**

**136149985 136150261 C L2 LINE/L2 3419 3151**

**136150301 136150563 + MLT1D LTR/MaLR 70 347**

**136150566 136150632 + MLT1E2 LTR/MaLR 560 626**

**136150633 136150735 C L2 LINE/L2 3155 3052**

**136150950 136151042 C MER63 DNA/AcHobo 1061 957**

**_________________________________________________________________________**

**L1HS_9_44 105039589-105043574 M_INTRA_RMD**

**105034132 105034187 + L1MB5 LINE/L1 6118 6173**

**105034675 105034699 + (T)n Simple_repeat 1 25**

**105035510 105035551 + L2 LINE/L2 2941 2984**

**105035552 105035585 + (TTTA)n Simple_repeat 2 35**

**105035594 105035852 C AluSx SINE/Alu 260 1**

**105035853 105035942 + L2 LINE/L2 2990 3075**

**105036068 105036370 C AluSx SINE/Alu 305 1**

**105036748 105036885 C L2 LINE/L2 3245 3106**

**105036886 105037190 + AluSg SINE/Alu 1 303**

**105037191 105037852 C L2 LINE/L2 3105 2393**

**105038475 105038500 + (TG)n Simple_repeat 1 26**

**105038947 105039051 C L2 LINE/L2 2373 2275**

**105039361 105039584 + L1PA13 LINE/L1 5891 6122**

**105039589 105043574 + L1HS LINE/L1 2 3987 ( L1HS_9_44 ) (R12)**

**105043587 105043727 + L1PA2 LINE/L1 6050 6190 (R12)**

**105043728 105043771 + L1PREC2 LINE/L1 6112 6160**

**105044754 105044822 C L2 LINE/L2 3353 3285**

**105045010 105045190 C LTR16D LTR/ERVL 325 140**

**105045925 105045945 + AT_rich Low_complexity 1 21**

**105046049 105046600 C MER41A LTR/ERV1 554 1**

**105046863 105046913 + (TG)n Simple_repeat 2 52**

**105046941 105046983 + MIR SINE/MIR 118 156**

**105047029 105047085 + Tigger5 DNA/MER2_type 2345 2401**

**105047086 105047164 C Tigger5 DNA/MER2_type 79 1**

**105048458 105048732 + L1ME3A LINE/L1 5881 6162**

**Ortholog in Chimp 102541038-102547579 Plus Nscore 0.17**

**N positions 102543959-102543959; 102543999-102544008;**

**102535601 102535656 + L1MB5 LINE/L1 6118 6173**

**102536956 102537009 + L2 LINE/L2 2928 2984**

**102537010 102537043 + (TTTA)n Simple_repeat 2 35**

**102537052 102537310 C AluSx SINE/Alu 260 1**

**102537311 102537399 + L2 LINE/L2 2990 3075**

**102537525 102537826 C AluSx SINE/Alu 304 1**

**102538200 102538337 C L2 LINE/L2 3245 3106**

**102538338 102538638 + AluSg SINE/Alu 1 299**

**102538639 102539300 C L2 LINE/L2 3105 2393**

**102540396 102540500 C L2 LINE/L2 2373 2275**

**102540810 102541033 + L1PA13 LINE/L1 5891 6122**

**102541038 102543992 + L1Pt LINE/L1 2 2926 (R1,R2)**

**102544014 102547683 + L1PA2 LINE/L1 2464 6155 (R1,R2) (BREAK DUE TO Ns, OVERLAP AT Ns)**

**102547684 102547714 + AT_rich Low_complexity 1 31**

**102548746 102548814 C L2 LINE/L2 3353 3285**

**102549002 102549182 C LTR16D LTR/ERVL 325 140**

**102550038 102550589 C MER41A LTR/ERV1 554 1**

**102550837 102550918 + (TATATG)n Simple_repeat 2 82**

**102550937 102550979 + MIR SINE/MIR 118 156**

**102551075 102551424 C MER41A LTR/ERV1 349 1**

**102551672 102551753 + (TA)n Simple_repeat 2 84**

**102551772 102551814 + MIR SINE/MIR 118 156**

**102551860 102551916 + Tigger5 DNA/MER2_type 2345 2401**

**102551917 102551995 C Tigger5 DNA/MER2_type 79 1**

**102553289 102553564 + L1ME3A LINE/L1 5881 6162**

**_________________________________________________________________________**

**L1HS_10_29c 83191402-83191711 M_INTRA_RMD**

**83183635 83183677 + AT_rich Low_complexity 1 43**

**83183707 83183974 C L1ME3A LINE/L1 6158 5891**

**83183972 83184552 + L1MD2 LINE/L1 5778 6356**

**83184972 83185487 + L1MC4 LINE/L1 6458 7059**

**83185482 83185613 + L1MC4 LINE/L1 7338 7479**

**83185614 83185982 C THE1B LTR/MaLR 362 1**

**83185983 83186390 + L1MC4 LINE/L1 7480 7899**

**83187060 83187363 + AluY SINE/Alu 10 313**

**83187779 83188064 C HAL1 LINE/L1 1639 1365**

**83188121 83188312 C L1MA4 LINE/L1 6300 6104**

**83188398 83188438 + AT_rich Low_complexity 1 41**

**83188593 83189130 C L1MC4a LINE/L1 7808 7230**

**83189138 83189408 C MLT1E LTR/MaLR 593 318**

**83189515 83190084 C L1MC4a LINE/L1 5855 5254**

**83190099 83190342 C L1MC4a LINE/L1 5228 4993**

**83190346 83191410 C L1PA2 LINE/L1 6183 5118 (R12)**

**83191402 83191711 C L1HS LINE/L1 321 13 ( L1HS_10_29c ) (R12)**

**83191711 83191949 C L1MC4a LINE/L1 4995 4768**

**83191950 83192248 C AluSg SINE/Alu 298 1**

**83192249 83192521 C L1MC4a LINE/L1 4767 4491**

**83192598 83192770 C L1M1 LINE/L1 4001 3829**

**83192854 83194017 C L1MA2 LINE/L1 7723 6570**

**83194018 83194437 + MER61C LTR/ERV1 1 428**

**83194438 83194478 C L1MA2 LINE/L1 6569 6525**

**83194472 83194618 C L1MA2 LINE/L1 5906 5760**

**83194615 83197667 C L1MA2 LINE/L1 6515 3668**

**83197668 83197973 C AluSx SINE/Alu 308 1**

**83197974 83198286 C L1MA2 LINE/L1 3667 3361**

**83198303 83199170 C L1MA2 LINE/L1 2638 1756**

**83199171 83199468 C AluSp SINE/Alu 300 2**

**83199469 83201165 C L1MA2 LINE/L1 1755 13**

**Ortholog in Chimp 81473574-81479354 Minus Nscore 1.70**

**N positions 81478627-81478636; 81474015-81474102;**

**81465638 81465680 + AT_rich Low_complexity 1 43**

**81465710 81465969 C L1ME3A LINE/L1 6158 5899**

**81465981 81466418 + L1MD2 LINE/L1 5783 6227**

**81466716 81466755 + AT_rich Low_complexity 1 40**

**81467226 81467666 + L1MC4 LINE/L1 6514 7059**

**81467661 81467792 + L1MC4 LINE/L1 7338 7479**

**81467793 81468161 C THE1B LTR/MaLR 362 1**

**81468162 81468569 + L1MC4 LINE/L1 7480 7899**

**81469239 81469540 + AluY SINE/Alu 10 311**

**81469955 81470240 C HAL1 LINE/L1 1639 1365**

**81470297 81470488 C L1MA4 LINE/L1 6300 6104**

**81470574 81470614 + AT_rich Low_complexity 1 41**

**81470769 81471306 C L1MC4a LINE/L1 7808 7230**

**81471314 81471584 C MLT1E LTR/MaLR 593 318**

**81471691 81472260 C L1MC4a LINE/L1 5855 5254**

**81472264 81472526 C L1MC4a LINE/L1 5234 4981**

**81472549 81474014 C L1PA2 LINE/L1 6029 4563 (R1,R2) (Broken due to Ns)**

**81474104 81478012 C L1PA2 LINE/L1 3909 3 (R1,R2)**

**81478637 81479354 C L1Pt LINE/L1 727 13**

**81479354 81479586 C L1MC4a LINE/L1 4995 4768**

**81479587 81479883 C AluSg SINE/Alu 296 1**

**81479884 81480156 C L1MC4a LINE/L1 4767 4491**

**81480232 81480404 C L1M1 LINE/L1 4001 3829**

**81480488 81481651 C L1MA2 LINE/L1 7723 6570**

**81481652 81482071 + MER61C LTR/ERV1 1 428**

**81482072 81482116 C L1MA2 LINE/L1 6569 6525**

**81482110 81482256 C L1MA2 LINE/L1 5906 5760**

**81482253 81485311 C L1MA2 LINE/L1 6515 3668**

**81485312 81485616 C AluSx SINE/Alu 307 1**

**81485617 81485932 C L1MA2 LINE/L1 3667 3361**

**81485949 81486809 C L1MA2 LINE/L1 2638 1770**

**81486810 81487110 C AluSp SINE/Alu 303 2**

**81487111 81488809 C L1MA2 LINE/L1 1769 13**

**_________________________________________________________________________**

**L1HS_10_30c 83518118-83522977 C_INTRA_RMD**

**83512015 83512817 + L1M5 LINE/L1 3118 3878**

**83512851 83512880 + AT_rich Low_complexity 1 30**

**83512977 83518073 C L1PA8A LINE/L1 6314 1135**

**83518074 83518103 + (TTG)n Simple_repeat 1 30**

**83518118 83522977 C L1HS LINE/L1 4862 1 ( L1HS_10_30c ) (R1,R2)**

**83522982 83523223 C L1M5 LINE/L1 3227 2972**

**83523910 83523939 + AT_rich Low_complexity 1 30**

**83523965 83524242 C AluSx SINE/Alu 276 1**

**83524784 83525096 C AluSx SINE/Alu 312 1**

**83527259 83527408 + MER5B DNA/MER1_type 28 177**

**83527791 83528487 + MER44C DNA/MER2_type 1 733**

**Ortholog in Chimp 81799614-81803737 Minus Nscore 0.24**

**N positions 81802138-81802147;**

**81793028 81793825 + L1MD LINE/L1 3118 3873**

**81793864 81793893 + AT_rich Low_complexity 1 30**

**81794697 81798197 C L1PA8A LINE/L1 6102 2501**

**81798198 81798220 + (TTTA)n Simple_repeat 2 24**

**81798221 81799569 C L1PA8A LINE/L1 2500 1135**

**81799570 81799599 + (TTG)n Simple_repeat 1 30**

**81799614 81802137 C L1Pt LINE/L1 4862 2336 (R12)**

**81802137 81803737 C L1Pt LINE/L1 1609 1 (R12)**

**81804673 81804702 + AT_rich Low_complexity 1 30**

**81804741 81805019 C AluSx SINE/Alu 276 1**

**81805721 81805779 + (T)n Simple_repeat 1 55**

**81805780 81805874 C SVA_B Other 854 760**

**81805888 81806188 C AluSx SINE/Alu 300 1**

**81808350 81808499 + MER5B DNA/MER1_type 28 177**

**81808880 81809567 + MER44C DNA/MER2_type 1 733**

**81809612 81809637 + (TTTG)n Simple_repeat 2 27**

**_________________________________________________________________________**

**L1HS_10_39 109213285-109214060 M_INTRA_RMD**

**109211688 109212086 C MLT1E2 LTR/MaLR 402 1**

**109212128 109212162 + (CA)n Simple_repeat 1 35**

**109212196 109212263 + L1MEc LINE/L1 1214 1274**

**109212264 109212570 + AluSx SINE/Alu 1 307**

**109212571 109212817 + L1MEc LINE/L1 1146 1488**

**109212846 109213213 + L1MEc LINE/L1 1480 1886**

**109213285 109214060 + L1HS LINE/L1 1 767 ( L1HS_10_39 ) (R12)**

**109214058 109214132 + L1PA2 LINE/L1 2767 2841 (R12)**

**109214133 109214446 + AluYb8 SINE/Alu 1 314 (D)**

**109214447 109217770 + L1PA2 LINE/L1 2842 6155 (R12)**

**109217810 109218151 + L1MD LINE/L1 3976 4367**

**109218727 109218822 + ERVL-E LTR/ERVL 462 557**

**109218961 109219635 C MER54B LTR/ERVL 793 114**

**109219817 109220098 + AluSp SINE/Alu 1 281**

**109220100 109220122 + (TAAA)n Simple_repeat 2 24**

**109220498 109220659 C MER58A DNA/MER1_type 167 1**

**109220939 109221568 C LTR9B LTR/ERV1 644 1**

**109222681 109222820 + MIRb SINE/MIR 107 257**

**109222908 109223047 + MER5A DNA/MER1_type 35 186**

**109223053 109223347 C L3 LINE/CR1 4447 4129**

**Ortholog in Chimp 108013378-108016078 Plus Nscore 0.00**

**N positions**

**108011778 108012175 C MLT1E2 LTR/MaLR 402 1**

**108012217 108012251 + (CA)n Simple_repeat 1 35**

**108012288 108012355 + L1M4c LINE/L1 1122 1176**

**108012356 108012662 + AluSx SINE/Alu 1 307**

**108012663 108013305 + L1M4c LINE/L1 1177 1947**

**108013377 108019406 + L1PA2 LINE/L1 1 6032 (R) -no disruption**

**108019445 108019788 + L1MD LINE/L1 3976 4367**

**108020366 108020461 + ERVL-E LTR/ERVL 462 557**

**108020602 108021277 C MER54B LTR/ERVL 793 114**

**108021459 108021764 + AluSp SINE/Alu 1 306**

**108022140 108022301 C MER58A DNA/MER1_type 167 1**

**108022581 108023209 C LTR9B LTR/ERV1 644 1**

**108024323 108024462 + MIRb SINE/MIR 107 257**

**108024550 108024689 + MER5A DNA/MER1_type 35 186**

**108024695 108025010 C L3 LINE/CR1 4447 4112**

**_________________________________________________________________________**

**L1HS_11_4 5892300-5896254 C_DISRUPTED_M_INTER_RMD**

**5882406 5886820 C L1PB1 LINE/L1 6867 2393**

**5886821 5886850 + (TTAG)n Simple_repeat 3 32**

**5886851 5887119 C AluSx SINE/Alu 277 1**

**5887120 5888727 C L1PB1 LINE/L1 2392 620**

**5888708 5888879 C L1PBa1 LINE/L1 442 51**

**5888963 5889152 C L1M5 LINE/L1 3217 3024**

**5889168 5889310 C MSTD-int LTR/MaLR 146 4**

**5889313 5889705 C MSTD LTR/MaLR 394 1**

**5890251 5890387 + L1PA3 LINE/L1 6016 6152**

**5891158 5891390 C MIR SINE/MIR 252 12**

**5891499 5891611 C LTR67 LTR/ERVL 553 440**

**5892300 5896254 + L1HS LINE/L1 13 3980 ( L1HS_11_4 )**

**5896261 5896347 + LTR26 LTR/ERV1 516 603**

**5896370 5896653 + L1MEa LINE/L1 188 466**

**5896656 5896743 + L1HS LINE/L1 8 98**

**5896738 5897627 + L1P1 LINE/L1 617 1491**

**5897626 5899662 + L1PA4 LINE/L1 4114 6153**

**5899646 5899668 + AT_rich Low_complexity 1 23**

**5899669 5900208 + L1MEa LINE/L1 456 993**

**5900198 5902757 + L1M1 LINE/L1 27 2713**

**Ortholog in Chimp 5758689-5762758 Plus Nscore 0.00**

**N positions**

**5749969 5753214 C L1PB1 LINE/L1 5686 2393**

**5753215 5753502 C AluSx SINE/Alu 296 1**

**5753503 5755110 C L1PB1 LINE/L1 2392 620**

**5755091 5755239 C L1PB1 LINE/L1 200 51**

**5755347 5755539 C L1M5 LINE/L1 3217 3024**

**5755555 5755699 C MSTD-int LTR/MaLR 146 2**

**5755708 5756092 C MSTD LTR/MaLR 387 1**

**5756638 5756774 + L1PA3 LINE/L1 6016 6152**

**5756775 5756802 + AT_rich Low_complexity 1 28**

**5757553 5757785 C MIR SINE/MIR 252 12**

**5757891 5758004 C LTR67 LTR/ERVL 553 439**

**5758689 5762758 + L1P1 LINE/L1 13 4103 (OCCUPIED)**

**5762765 5762851 + LTR26 LTR/ERV1 516 603**

**5762874 5763158 + L1MEa LINE/L1 188 467**

**5763165 5763249 + L1Pt LINE/L1 12 98**

**5763244 5764128 + L1Pt LINE/L1 494 1368**

**5764127 5766166 + L1PA4 LINE/L1 4114 6153**

**5766173 5766712 + L1MEa LINE/L1 456 993**

**5766702 5769257 + L1M1 LINE/L1 27 2713**

**_________________________________________________________________________**

**L1HS_11_17c 24146892-24148613 C_DISRUPTED_M_INTER_RMD**

**24140844 24140914 C MER94 DNA/AcHobo 128 57**

**24141141 24141393 + L2 LINE/L2 3125 3419**

**24142597 24142726 + CT-rich Low_complexity 4 136**

**24143311 24143332 + AT_rich Low_complexity 1 22**

**24143614 24143755 C MIR SINE/MIR 236 93**

**24144507 24144655 C L1ME3A LINE/L1 6161 5991**

**24144656 24144962 + AluSx SINE/Alu 1 312**

**24144963 24145546 C L1ME3A LINE/L1 5990 5375**

**24145573 24145610 + AT_rich Low_complexity 1 38**

**24145730 24145831 C L1MDa LINE/L1 181 82**

**24146224 24146244 + AT_rich Low_complexity 1 21**

**24146892 24148613 C L1HS LINE/L1 3583 1867 ( L1HS_11_17c ) (R12)**

**24149259 24149636 C MLT1B LTR/MaLR 390 4**

**24149675 24149771 C L2 LINE/L2 3372 3274**

**24150443 24151620 C L1ME2 LINE/L1 6162 4873**

**24151621 24152038 C MER61B LTR/ERV1 427 1**

**24152039 24152330 C L1ME2 LINE/L1 4872 4576**

**24152381 24152982 C L1ME2 LINE/L1 4017 3350**

**24153116 24153136 + AT_rich Low_complexity 1 21**

**24153863 24154177 C AluY SINE/Alu 310 1**

**24154601 24154652 + AT_rich Low_complexity 1 52**

**Ortholog in Chimp 24250587-24252910 Minus Nscore 6.45**

**N positions 24251211-24251360;**

**24244630 24244880 + L2 LINE/L2 3125 3417**

**24245181 24245222 + AT_rich Low_complexity 1 42**

**24246084 24246188 + CT-rich Low_complexity 4 108**

**24247094 24247270 C MIR SINE/MIR 249 71**

**24248185 24248333 C L1ME3A LINE/L1 6161 5995**

**24248334 24248639 + AluSx SINE/Alu 1 311**

**24248640 24249240 C L1ME3A LINE/L1 5994 5375**

**24249267 24249304 + AT_rich Low_complexity 1 38**

**24249428 24249529 C L1MDa LINE/L1 181 82**

**24249792 24249864 + A-rich Low_complexity 1 74**

**24250587 24251210 C L1P1 LINE/L1 3706 3086 (R2)**

**24251410 24252910 C L1Pt LINE/L1 3406 1868 (R1)**

**24253555 24253910 C MLT1B LTR/MaLR 390 20**

**24253971 24254067 C L2 LINE/L2 3372 3274**

**24254736 24255907 C L1ME2 LINE/L1 6162 4873**

**24255908 24256325 C MER61B LTR/ERV1 427 1**

**24256326 24256617 C L1ME2 LINE/L1 4872 4576**

**24256664 24257260 C L1ME2 LINE/L1 4015 3350**

**24257394 24257418 + AT_rich Low_complexity 1 25**

**24258140 24258447 C AluY SINE/Alu 300 1**

**24258869 24258916 + AT_rich Low_complexity 1 48**

**_________________________________________________________________________**

**L1HS_11_25c 34669416-34670953 M_INTRA_RMD**

**34662827 34663064 C L1M LINE/L1 5193 4969**

**34663278 34663356 C L1M LINE/L1 4654 4572**

**34663869 34663910 + (CA)n Simple_repeat 1 42**

**34664191 34664816 C L1ME3B LINE/L1 6003 5320**

**34664881 34664940 + MLT1A LTR/MaLR 34 93**

**34664960 34665091 C L1PB1 LINE/L1 6150 6018**

**34665092 34668951 C L1PA2 LINE/L1 6152 2289 (R12)**

**34668947 34669335 + L1P1 LINE/L1 4448 4836 (D)**

**34669336 34669421 + L1P1 LINE/L1 4837 4922 (D)**

**34669416 34670953 C L1HS LINE/L1 1550 12 ( L1HS_11_25c ) (R12)**

**34670951 34672365 C L1MEc LINE/L1 5364 3855**

**34672385 34674150 C L1M2 LINE/L1 4958 3150**

**34674154 34674667 C L1PA15 LINE/L1 6150 5639**

**34674676 34674906 C L1M4c LINE/L1 1048 806**

**34675153 34675547 + LTR16A1 LTR/ERVL 45 457**

**34675833 34675861 + AT_rich Low_complexity 1 29**

**34676042 34676332 C AluSg SINE/Alu 290 1**

**34676386 34676803 C L1MEc LINE/L1 3890 3471**

**34676809 34677086 + L1PB1 LINE/L1 5867 6146**

**Ortholog in Chimp 34964438-34966583 Minus Nscore 0.00**

**N positions**

**34957782 34958191 C L1M LINE/L1 5360 4969**

**34958401 34958502 C L1M LINE/L1 4687 4572**

**34959015 34959049 + (CA)n Simple_repeat 1 36**

**34959330 34959972 C L1ME3B LINE/L1 6076 5367**

**34959976 34960220 + (TA)n Simple_repeat 1 249**

**34960227 34960308 + (TTTA)n Simple_repeat 2 81**

**34960272 34960311 + (T)n Simple_repeat 1 40**

**34960323 34960421 + MLT1A LTR/MaLR 1 93**

**34960441 34960572 C L1PB1 LINE/L1 6150 6018**

**34960573 34966583 C L1PA2 LINE/L1 6030 12 (R1,R2)**

**34966581 34967993 C L1ME3B LINE/L1 5364 3855**

**34968013 34969781 C L1M2 LINE/L1 4958 3150**

**34969785 34970298 C L1PA15 LINE/L1 6150 5639**

**34970797 34971180 + LTR16A1 LTR/ERVL 61 457**

**34971466 34971494 + AT_rich Low_complexity 1 29**

**34971675 34971965 C AluSg SINE/Alu 290 1**

**34972019 34972142 C L1ME3B LINE/L1 3890 3757**

**34972143 34972162 + (TTTTC)n Simple_repeat 1 20**

**34972163 34972441 C L1ME3B LINE/L1 3756 3471**

**34972447 34972724 + L1PB1 LINE/L1 5867 6146**

**_________________________________________________________________________**

**L1HS_12_31c 64235466-64235787 C_INTER_RMD_M_DISRUPTED**

**64227887 64227988 + MIRm SINE/MIR 3 113**

**64228061 64228375 + AluSx SINE/Alu 1 307**

**64228614 64228635 + AT_rich Low_complexity 1 22**

**64228777 64228812 + (TG)n Simple_repeat 2 37**

**64229216 64229364 + MIR SINE/MIR 100 248**

**64229372 64229402 + (CAT)n Simple_repeat 3 33**

**64229468 64235155 C L1PA3 LINE/L1 6018 317 (R2)**

**64235156 64235465 C AluYa5 SINE/Alu 310 1 (D)**

**64235466 64235787 C L1HS LINE/L1 316 3 ( L1HS_12_31c ) (R1)**

**64235933 64235981 + GA-rich Low_complexity 3 51**

**64236201 64236340 C MIR3 SINE/MIR 133 1**

**64237317 64237937 + MER41B LTR/ERV1 1 634**

**64238103 64238403 C HAL1 LINE/L1 559 249**

**64238406 64238435 + AT_rich Low_complexity 1 30**

**64238487 64238622 + LTR67 LTR/ERVL 64 209**

**64238679 64238865 + LTR67 LTR/ERVL 353 540**

**64239221 64239253 + (TTA)n Simple_repeat 2 34**

**64242047 64242673 + MER41B LTR/ERV1 1 626**

**64243017 64243172 + MIR3 SINE/MIR 4 160**

**64243622 64243759 + GA-rich Low_complexity 3 141**

**64243760 64243798 + (CAG)n Simple_repeat 2 40**

**64243895 64243997 C MIR SINE/MIR 200 83**

**64244729 64244892 + MER45A DNA/Tip100 2 177**

**Ortholog in Chimp 23900259-23900568 Plus Nscore 0.00 (23900259-23906508 Nscore - 0.32)**

**N positions**

**23891811 23891929 + MIR SINE/MIR 83 222**

**23892032 23892119 + (TTC)n Simple_repeat 1 88**

**23892899 23893054 C MIR3 SINE/MIR 160 4**

**23893372 23894006 C MER41B LTR/ERV1 634 1**

**23896789 23896821 + (TAA)n Simple_repeat 2 34**

**23897177 23897363 C LTR67 LTR/ERVL 540 353**

**23897420 23897555 C LTR67 LTR/ERVL 209 64**

**23897607 23897635 + AT_rich Low_complexity 1 29**

**23897639 23897939 + HAL1 LINE/L1 249 559**

**23898105 23898725 C MER41B LTR/ERV1 634 1**

**23899702 23899843 + MIR3 SINE/MIR 1 135**

**23900066 23900114 + CT-rich Low_complexity 3 51**

**23900259 23906227 + L1PA3 LINE/L1 3 5792 (R)**

**23906509 23906536 + (CA)n Simple_repeat 2 29**

**23906678 23906698 + AT_rich Low_complexity 1 21**

**23906937 23907251 C AluSx SINE/Alu 307 1**

**23907324 23907425 C MIRm SINE/MIR 113 3**

**_________________________________________________________________________**

**L1HS_13_18 79624042-79625783 M_INTRA_RMD**

**79618779 79618898 C L1MA8 LINE/L1 6288 6166**

**79618899 79619212 C AluY SINE/Alu 311 1**

**79619213 79619317 C L1MA8 LINE/L1 6165 6073**

**79619318 79619705 C THE1D LTR/MaLR 381 1**

**79619706 79619891 C L1MA8 LINE/L1 6072 5888**

**79619962 79620074 + L2 LINE/L2 3281 3396**

**79620136 79620769 + Tigger1 DNA/MER2_type 28 666**

**79620770 79621004 + MER46A DNA/MER2_type 2 236**

**79621005 79621450 + Tigger1 DNA/MER2_type 667 1243**

**79621814 79621839 + (T)n Simple_repeat 1 26**

**79622739 79622824 C Tigger1 DNA/MER2_type 2418 2331**

**79623062 79623135 C L2 LINE/L2 3419 3335**

**79623147 79623175 + (TTTTG)n Simple_repeat 3 31**

**79623755 79624041 C MLT1J LTR/MaLR 337 23**

**79624042 79625783 + L1HS LINE/L1 5 1751 ( L1HS_13_18 ) (R12)**

**79625782 79628554 + L1PA2 LINE/L1 3377 6153 (R12)**

**79628652 79628751 C L3 LINE/CR1 3486 3382**

**79628988 79629284 + AluSq SINE/Alu 1 299**

**79630112 79630171 + (GGAA)n Simple_repeat 3 62**

**79630311 79630609 + AluSx SINE/Alu 1 300**

**79631165 79631273 C L2 LINE/L2 3373 3255**

**79631542 79631832 + AluJb SINE/Alu 1 298**

**79632893 79633368 + L1ME1 LINE/L1 5668 6148**

**79633369 79633772 C MSTA LTR/MaLR 428 1**

**79633773 79633788 + L1ME1 LINE/L1 6149 6160**

**79633797 79633944 C MIR SINE/MIR 216 65**

**79634342 79634652 C AluSg SINE/Alu 308 1**

**Ortholog in Chimp 80546735-80549984 Plus Nscore 0.00**

**N positions**

**80542409 80542594 C L1MA8 LINE/L1 6072 5888**

**80542665 80542777 + L2 LINE/L2 3281 3396**

**80542827 80544150 + Tigger1 DNA/MER2_type 1 1243**

**80544514 80544537 + (T)n Simple_repeat 1 24**

**80545437 80545522 C Tigger1 DNA/MER2_type 2418 2331**

**80545760 80545833 C L2 LINE/L2 3419 3335**

**80545845 80545873 + (TTTTG)n Simple_repeat 3 31**

**80546452 80546730 C MLT1J LTR/MaLR 337 33**

**80546731 80552762 + L1PA2 LINE/L1 1 6031 (R1,R2)**

**80552849 80552948 C L3 LINE/CR1 3486 3382**

**80553185 80553482 + AluSq SINE/Alu 1 300**

**80554310 80554361 + (GGAA)n Simple_repeat 3 54**

**80554501 80554801 + AluSx SINE/Alu 1 301**

**80555357 80555465 C L2 LINE/L2 3373 3255**

**80555734 80556024 + AluJb SINE/Alu 1 298**

**80557085 80557560 + L1ME1 LINE/L1 5668 6148**

**80557561 80557964 C MSTA LTR/MaLR 428 1**

**80557965 80557980 + L1ME1 LINE/L1 6149 6160**

**80557989 80558136 C MIR SINE/MIR 216 65**

**80558526 80558836 C AluSg SINE/Alu 308 1**

**_________________________________________________________________________**

**L1HS_13_34c 104832362-104832716 C_INTER_RMD_M_DISRUPTED**

**104822440 104823711 + L1MA8 LINE/L1 5005 6288**

**104823756 104824417 + L1MB8 LINE/L1 5490 6172**

**104825989 104826019 + AT_rich Low_complexity 1 31**

**104826083 104826214 + L1M5 LINE/L1 5543 5680**

**104826885 104827036 + MLT1J LTR/MaLR 246 404**

**104828400 104828421 + AT_rich Low_complexity 1 22**

**104828422 104828699 C AluJb SINE/Alu 289 1**

**104829051 104829546 C L3 LINE/CR1 4315 3792**

**104830124 104830146 + AT_rich Low_complexity 1 23**

**104830508 104830875 C MSTA LTR/MaLR 428 2**

**104832362 104832716 C L1HS LINE/L1 933 589 ( L1HS_13_34c ) (R2)**

**104832717 104833016 C AluYg6 SINE/Alu 301 1 (D)**

**104833017 104833602 C L1HS LINE/L1 588 4 (R1)**

**104833603 104838696 + L1PA3 LINE/L1 926 6031 (3' end of twinprimed L1)**

**104838792 104838836 + AT_rich Low_complexity 1 45**

**104840075 104840095 + AT_rich Low_complexity 1 21**

**104840181 104840344 C MER5B DNA/MER1_type 162 4**

**Ortholog in Chimp 106338811-106339167 Minus Nscore 0.00**

**N positions**

**106328893 106330156 + L1MA8 LINE/L1 5022 6288**

**106330179 106330211 + (TA)n Simple_repeat 1 33**

**106330213 106330874 + L1MB8 LINE/L1 5490 6172**

**106331401 106331514 C MIRb SINE/MIR 202 84**

**106332447 106332477 + AT_rich Low_complexity 1 31**

**106332541 106332669 + L1M5 LINE/L1 5543 5680**

**106333315 106333483 + MLT1J LTR/MaLR 232 404**

**106334847 106334868 + AT_rich Low_complexity 1 22**

**106334869 106335146 C AluJb SINE/Alu 289 1**

**106335498 106335994 C L3 LINE/CR1 4315 3792**

**106336572 106336594 + AT_rich Low_complexity 1 23**

**106336956 106337323 C MSTA LTR/MaLR 415 2**

**106338811 106339738 C L1Pt LINE/L1 933 4 (R)**

**106339739 106344835 + L1PA3 LINE/L1 927 6031**

**106344931 106344975 + AT_rich Low_complexity 1 45**

**106346211 106346231 + AT_rich Low_complexity 1 21**

**106346317 106346480 C MER5B DNA/MER1_type 162 4**

**_________________________________________________________________________**

**L1HS_13_35c 104833017-104833602 C_INTER_RMD_M_DISRUPTED**

**104823756 104824417 + L1MB8 LINE/L1 5490 6172**

**104825989 104826019 + AT_rich Low_complexity 1 31**

**104826083 104826214 + L1M5 LINE/L1 5543 5680**

**104826885 104827036 + MLT1J LTR/MaLR 246 404**

**104828400 104828421 + AT_rich Low_complexity 1 22**

**104828422 104828699 C AluJb SINE/Alu 289 1**

**104829051 104829546 C L3 LINE/CR1 4315 3792**

**104830124 104830146 + AT_rich Low_complexity 1 23**

**104830508 104830875 C MSTA LTR/MaLR 428 2**

**104832362 104832716 C L1HS LINE/L1 933 589 (R2)**

**104832717 104833016 C AluYg6 SINE/Alu 301 1 (D)**

**104833017 104833602 C L1HS LINE/L1 588 4 ( L1HS_13_35c ) (R1)**

**104833603 104838696 + L1PA3 LINE/L1 926 6031 (3' end of twim primed L1)**

**104838792 104838836 + AT_rich Low_complexity 1 45**

**104840075 104840095 + AT_rich Low_complexity 1 21**

**104840181 104840344 C MER5B DNA/MER1_type 162 4**

**104842455 104842643 + ERVL LTR/ERVL 1772 1965**

**Ortholog in Chimp 106339171-106339738 Minus Nscore 0.00**

**N positions**

**106330179 106330211 + (TA)n Simple_repeat 1 33**

**106330213 106330874 + L1MB8 LINE/L1 5490 6172**

**106331401 106331514 C MIRb SINE/MIR 202 84**

**106332447 106332477 + AT_rich Low_complexity 1 31**

**106332541 106332669 + L1M5 LINE/L1 5543 5680**

**106333315 106333483 + MLT1J LTR/MaLR 232 404**

**106334847 106334868 + AT_rich Low_complexity 1 22**

**106334869 106335146 C AluJb SINE/Alu 289 1**

**106335498 106335994 C L3 LINE/CR1 4315 3792**

**106336572 106336594 + AT_rich Low_complexity 1 23**

**106336956 106337323 C MSTA LTR/MaLR 415 2**

**106338811 106339738 C L1Pt LINE/L1 933 4 (R)**

**106339739 106344835 + L1PA3 LINE/L1 927 6031**

**106344931 106344975 + AT_rich Low_complexity 1 45**

**106346211 106346231 + AT_rich Low_complexity 1 21**

**106346317 106346480 C MER5B DNA/MER1_type 162 4**

**106348588 106348776 + ERVL LTR/ERVL 1772 1965**

**_________________________________________________________________________**

**L1HS_14_20 43136854-43141041 C_INTER_RMD_M_DISRUPTED**

**43126610 43127935 C L1M5 LINE/L1 5387 3974**

**43128571 43128591 + AT_rich Low_complexity 1 21**

**43131277 43131301 + AT_rich Low_complexity 1 25**

**43131302 43131673 C L1PB1 LINE/L1 6151 5776**

**43131674 43131768 + (TA)n Simple_repeat 1 95**

**43131769 43134088 C L1PB1 LINE/L1 5801 3567**

**43136175 43136849 C MER89-int LTR/ERV1 6073 5358**

**43136854 43141041 + L1HS LINE/L1 1 4202 ( L1HS_14_20 )**

**43141038 43142354 + L1PA2 LINE/L1 4837 6153**

**43142356 43143019 C MER89-int LTR/ERV1 5363 4660**

**43143030 43143404 C MER89-int LTR/ERV1 4547 4170**

**43143442 43143636 + L1MCa LINE/L1 292 712**

**43143897 43144419 + L1MCa LINE/L1 555 1091**

**43144421 43144603 + L1M2 LINE/L1 2 185**

**43144619 43144704 + L1M2 LINE/L1 587 672**

**43145165 43145209 + (TCTA)n Simple_repeat 4 50**

**43145243 43145605 + L1PA8 LINE/L1 5805 6168**

**43145915 43145985 + AT_rich Low_complexity 1 71**

**43146641 43146661 + AT_rich Low_complexity 1 21**

**43146741 43146810 + AT_rich Low_complexity 1 70**

**43146888 43147056 C L1ME1 LINE/L1 6149 5981**

**43147057 43147263 C MER30 DNA/MER1_type 230 1**

**43147264 43147577 C L1ME1 LINE/L1 5980 5657**

**43147578 43147973 C MLT1B LTR/MaLR 390 4**

**43148042 43148234 C L1PA16 LINE/L1 6162 5955**

**43148235 43150654 C L1PA15-16 LINE/L1 3398 941**

**Ortholog in Chimp 42672359-42676314 Plus Nscore 0.25**

**N positions 42676294-42676303;**

**42662152 42663474 C L1M5 LINE/L1 5387 3974**

**42664111 42664131 + AT_rich Low_complexity 1 21**

**42666816 42666837 + AT_rich Low_complexity 1 22**

**42666838 42667209 C L1PB1 LINE/L1 6151 5776**

**42667210 42667290 + (TA)n Simple_repeat 1 81**

**42667291 42669605 C L1PB1 LINE/L1 5801 3567**

**42671680 42672354 C MER89-int LTR/ERV1 6073 5358**

**42672359 42676293 + L1Pt LINE/L1 1 3926**

**42676309 42676814 + L1PA2 LINE/L1 5646 6153**

**42676818 42677485 C MER89-int LTR/ERV1 5365 4660**

**42677496 42677871 C MER89-int LTR/ERV1 4547 4170**

**42677909 42678141 + L1MCa LINE/L1 286 716**

**42678363 42678890 + L1MCa LINE/L1 555 1091**

**42678892 42679074 + L1M2 LINE/L1 2 185**

**42679090 42679175 + L1M2 LINE/L1 587 672**

**42679636 42679688 + (TCTA)n Simple_repeat 4 58**

**42679722 42680084 + L1PA8 LINE/L1 5805 6168**

**42680394 42680464 + AT_rich Low_complexity 1 71**

**42681120 42681140 + AT_rich Low_complexity 1 21**

**42681220 42681289 + AT_rich Low_complexity 1 70**

**42681358 42681538 C L1ME1 LINE/L1 6158 5981**

**42681539 42681744 C MER30 DNA/MER1_type 230 1**

**42681745 42682058 C L1ME1 LINE/L1 5980 5657**

**42682059 42682456 C MLT1B LTR/MaLR 390 4**

**42682525 42682717 C L1PA16 LINE/L1 6162 5955**

**42682718 42685141 C L1PA15-16 LINE/L1 3398 941**

**42685142 42688585 + HERV4_I LTR/ERV1 2834 6268**

**_________________________________________________________________________**

**L1HS_14_31 61822791-61825189 C_INTRA_RMD**

**61817019 61817224 + MIRb SINE/MIR 16 248**

**61817199 61817251 + L2 LINE/L2 3370 3419**

**61818019 61818301 C MLT1A LTR/MaLR 374 1**

**61818929 61819322 + MLT1B LTR/MaLR 1 390**

**61819482 61819766 + AluY SINE/Alu 1 280**

**61819767 61819799 + (CAA)n Simple_repeat 1 33**

**61820419 61820611 + MLT1A0 LTR/MaLR 1 186**

**61820607 61820676 + MLT1A0 LTR/MaLR 298 371**

**61820927 61822796 C L1PA3 LINE/L1 6195 4326**

**61822791 61825189 + L1HS LINE/L1 1809 4211 ( L1HS_14_31 ) (R1,R2)**

**61825216 61825682 C L2 LINE/L2 3419 2875**

**61825750 61825900 + MER5A1 DNA/MER1_type 6 159**

**61826112 61826456 + MLT1J2 LTR/MaLR 54 444**

**61826461 61826596 C MER5A DNA/MER1_type 185 64**

**61826597 61826707 C L1PA5 LINE/L1 6146 6036**

**61826708 61826768 C MER5A DNA/MER1_type 63 3**

**61827964 61828434 C L2 LINE/L2 3403 2893**

**61828699 61829283 C L1P4 LINE/L1 4779 4193**

**61829273 61829898 C HAL1 LINE/L1 2480 1818**

**61829925 61829973 C HAL1 LINE/L1 1715 1666**

**61829974 61830266 C AluSc SINE/Alu 295 1**

**61830267 61830396 C HAL1 LINE/L1 1665 1536**

**61830548 61831197 C HAL1 LINE/L1 1558 849**

**Ortholog in Chimp 61705520-61707613 Plus Nscore 0.48**

**N positions 61706143-61706152;**

**61699617 61699901 + AluY SINE/Alu 1 280**

**61699902 61699955 + (CAA)n Simple_repeat 1 54**

**61700575 61700767 + MLT1A0 LTR/MaLR 1 186**

**61700763 61700832 + MLT1A0 LTR/MaLR 298 371**

**61701083 61701778 C L1PA3 LINE/L1 6200 5506**

**61702893 61704216 + L1P1 LINE/L1 2139 3473**

**61704252 61705525 C L1P2 LINE/L1 5598 4326**

**61705520 61707613 + L1Pt LINE/L1 1810 4211 (R12) (NOTE THAT THERE IS ~300 DIFFERENCE BETWEEN R1-R2 AND R12)**

**61707640 61708107 C L2 LINE/L2 3419 2875**

**61708175 61708325 + MER5A1 DNA/MER1_type 6 159**

**61708537 61708881 + MLT1J2 LTR/MaLR 54 444**

**61708886 61709010 C MER5A DNA/MER1_type 185 64**

**61709011 61709129 C L1PA5 LINE/L1 6154 6036**

**61709130 61709190 C MER5A DNA/MER1_type 63 3**

**61710386 61710818 C L2 LINE/L2 3403 2938**

**61711121 61711708 C L1P4 LINE/L1 4779 4193**

**61711698 61712323 C HAL1 LINE/L1 2480 1818**

**61712350 61712398 C HAL1 LINE/L1 1715 1666**

**61712399 61712686 C AluSc SINE/Alu 292 1**

**61712687 61712816 C HAL1 LINE/L1 1665 1536**

**61712968 61713641 C HAL1 LINE/L1 1558 811**

**_________________________________________________________________________**

**L1HS_14_35 70011674-70013407 C_DISRUPTED_M_INTER_RMD**

**70008247 70008770 + L1ME2 LINE/L1 5585 6154**

**70009141 70009161 + AT_rich Low_complexity 1 21**

**70009444 70010305 C MER21A LTR/ERV1 929 17**

**70010306 70010336 + (TG)n Simple_repeat 2 32**

**70010337 70010371 + (TA)n Simple_repeat 1 38**

**70010372 70010387 C MER21A LTR/ERV1 16 1**

**70010519 70010553 + (TG)n Simple_repeat 2 37**

**70010672 70010853 + L1MB4 LINE/L1 7 181**

**70010862 70011597 + L1MB4 LINE/L1 927 1662**

**70011602 70011673 C MLT-int LTR/MaLR 572 497**

**70011674 70013407 + L1HS LINE/L1 1 1734 ( L1HS_14_35 ) (R12)**

**70013410 70014998 + SVA Other 3 1379**

**70015005 70019358 + L1PA2 LINE/L1 1720 6069**

**70019364 70019452 C MLT-int LTR/MaLR 505 415**

**Ortholog in Chimp 70116445-70119007 Plus Nscore 6.25**

**N positions 70117309-70117468;**

**70112999 70113522 + L1ME2 LINE/L1 5585 6154**

**70114195 70115054 C MER21A LTR/ERV1 929 24**

**70115055 70115097 + (TG)n Simple_repeat 2 44**

**70115106 70115144 + AT_rich Low_complexity 1 39**

**70115281 70115315 + (TG)n Simple_repeat 2 37**

**70115434 70115615 + L1MB4 LINE/L1 7 181**

**70115624 70116368 + L1MB4 LINE/L1 927 1662**

**70116373 70116444 C MLT-int LTR/MaLR 572 497**

**70116446 70117252 + L1Pt LINE/L1 2 809 (R1)**

**70117544 70119008 + L1Pt LINE/L1 213 1736 (R2)**

**70119010 70119059 + (TCTCCC)n Simple_repeat 3 53**

**70119069 70119431 + SVA_B Other 1 362**

**70121118 70123184 + L1Pt LINE/L1 2289 4338**

**70124536 70125063 + L1PA2 LINE/L1 5661 6188**

**_________________________________________________________________________**

**L1HS_15_23 71612037-71615599 C_INTRA_RMD**

**71606022 71606374 C L1MEc LINE/L1 982 619**

**71606375 71606505 C FLAM_C SINE/Alu 128 1**

**71606506 71607078 C L1MEc LINE/L1 618 47**

**71607317 71607933 + L1MC5 LINE/L1 6837 7427**

**71607934 71608240 + MER58B DNA/MER1_type 1 340**

**71608241 71608520 + L1MC5 LINE/L1 7428 7713**

**71608521 71608831 C AluY SINE/Alu 310 1**

**71608832 71609039 + L1MC5 LINE/L1 7714 7906**

**71609087 71609291 + L2 LINE/L2 3121 3342**

**71609776 71610055 + AluJo SINE/Alu 3 296**

**71611169 71611304 + L2 LINE/L2 2008 2147**

**71611367 71611377 + L1HS LINE/L1 1462 1472**

**71611378 71612037 C L1PA2 LINE/L1 6155 5496**

**71612037 71615599 + L1HS LINE/L1 1471 5048 ( L1HS_15_23 ) (R1,R2)**

**71615904 71615981 C L2 LINE/L2 3197 3113**

**71616270 71616405 C L1PA16 LINE/L1 6157 6021**

**71616427 71616633 C L2 LINE/L2 3419 3205**

**71616881 71616955 + L2 LINE/L2 2958 3035**

**71616961 71617054 C L1MB1 LINE/L1 6163 6062**

**71617315 71617404 C L1MC4a LINE/L1 5933 5843**

**71617409 71617601 + Charlie5 DNA/MER1_type 1 200**

**71617600 71617845 + Charlie5 DNA/MER1_type 2352 2605**

**71617846 71618148 C AluY SINE/Alu 304 1**

**71618149 71618176 + Charlie5 DNA/MER1_type 2606 2624**

**71618195 71618510 C L1MC4a LINE/L1 5816 5497**

**71618677 71618865 C AluY SINE/Alu 307 119**

**71620320 71620347 + AT_rich Low_complexity 1 28**

**71620584 71621039 + Charlie4a DNA/MER1_type 14 508**

**71621210 71621251 + AT_rich Low_complexity 1 42**

**71621343 71621434 C MLT1J LTR/MaLR 511 415**

**71621443 71621754 + AluSx SINE/Alu 1 312**

**Ortholog in Chimp 71326913-71329979 Plus Nscore 0.33**

**N positions 71329368-71329377;**

**71320891 71321243 C L1MEc LINE/L1 982 619**

**71321244 71321376 C FLAM_C SINE/Alu 133 1**

**71321377 71321949 C L1MEc LINE/L1 618 47**

**71322188 71322802 + L1MC5 LINE/L1 6837 7427**

**71322803 71323109 + MER58B DNA/MER1_type 1 340**

**71323110 71323380 + L1MC5 LINE/L1 7428 7711**

**71323381 71323718 C AluY SINE/Alu 337 1**

**71323719 71323926 + L1MC5 LINE/L1 7712 7906**

**71323974 71324178 + L2 LINE/L2 3121 3342**

**71324654 71324933 + AluJo SINE/Alu 3 296**

**71326048 71326183 + L2 LINE/L2 2008 2147**

**71326256 71326913 C L1PA2 LINE/L1 6153 5496**

**71326914 71329367 + L1Pt LINE/L1 1473 3929 (R12)**

**71329428 71329979 + L1Pt LINE/L1 4467 5048 (R12)**

**71330284 71330361 C L2 LINE/L2 3197 3113**

**71330649 71330784 C L1PA16 LINE/L1 6157 6021**

**71330806 71330993 C L2 LINE/L2 3419 3225**

**71331261 71331331 + L2 LINE/L2 2964 3035**

**71331337 71331476 C L1MB1 LINE/L1 6163 6010**

**71331696 71331785 C L1MC4a LINE/L1 5933 5843**

**71331790 71331977 + Charlie5 DNA/MER1_type 1 200**

**71331976 71332220 + Charlie5 DNA/MER1_type 2352 2598**

**71332221 71332525 C AluY SINE/Alu 306 1**

**71332526 71332553 + Charlie5 DNA/MER1_type 2599 2624**

**71332572 71332919 C L1MC4a LINE/L1 5818 5469**

**71333052 71333237 C AluY SINE/Alu 304 119**

**71334683 71334710 + AT_rich Low_complexity 1 28**

**71334943 71335398 + Charlie4a DNA/MER1_type 14 508**

**71335570 71335611 + AT_rich Low_complexity 1 42**

**71335703 71335794 C MLT1J LTR/MaLR 511 415**

**71335803 71336113 + AluSx SINE/Alu 1 311**

**_________________________________________________________________________**

**L1HS_16_11c 34626287-34629551 C_INTER_RMD_M_DISRUPTED**

**34617639 34617663 + (CAA)n Simple_repeat 2 26**

**34619228 34619531 C AluSx SINE/Alu 300 1**

**34621390 34622400 + L1M4 LINE/L1 3398 4478**

**34625588 34625907 C HSAT6 Satellite 321 1**

**34625996 34626251 C MSTA LTR/MaLR 285 1**

**34626287 34629551 C L1HS LINE/L1 4281 1018 ( L1HS_16_11c )**

**34629552 34630187 + L1PA3 LINE/L1 5520 6155**

**34630206 34630522 + AluY SINE/Alu 1 311**

**34631962 34632046 C MER5B DNA/MER1_type 177 91**

**34632088 34634039 + L1PB4 LINE/L1 4189 6156**

**34634101 34634130 + AT_rich Low_complexity 1 30**

**34634131 34634369 + (TA)n Simple_repeat 1 256**

**34634375 34634655 C AluSx SINE/Alu 278 1**

**34634998 34635027 + (TTTA)n Simple_repeat 3 32**

**34636282 34636491 + L1MA9 LINE/L1 4251 4446**

**34636492 34636795 + AluSg SINE/Alu 1 305**

**34636796 34636843 + L1MA9 LINE/L1 4447 4485**

**34636844 34637154 + AluY SINE/Alu 1 311**

**34637159 34637186 + (CAAG)n Simple_repeat 1 28**

**34637187 34638315 + L1MA9 LINE/L1 4486 5671**

**34638378 34638840 + L1MA9 LINE/L1 5805 6277**

**Ortholog in Chimp 45014197-45017443 Plus Nscore 0.31**

**N positions 45014314-45014323;**

**45005066 45005540 C L1MA9 LINE/L1 6288 5805**

**45005578 45006791 C L1MA9 LINE/L1 5697 4477**

**45006792 45007101 C AluY SINE/Alu 310 1**

**45007102 45007148 C L1MA9 LINE/L1 4476 4447**

**45007149 45007442 C AluSg SINE/Alu 296 1**

**45007443 45007652 C L1MA9 LINE/L1 4446 4251**

**45008334 45008393 + GC_rich Low_complexity 1 60**

**45008909 45008942 + (TAAA)n Simple_repeat 3 36**

**45009252 45009529 + AluSx SINE/Alu 1 278**

**45009535 45009840 + (TA)n Simple_repeat 1 327**

**45009853 45009911 + AT_rich Low_complexity 1 59**

**45009973 45011923 C L1PB4 LINE/L1 6156 4189**

**45013494 45013810 C AluY SINE/Alu 311 1**

**45013832 45014208 C L1PA3 LINE/L1 6155 5774**

**45014324 45017443 + L1P1 LINE/L1 1281 4404 (OCCUPIED)**

**45017479 45017734 + MSTA LTR/MaLR 1 285**

**45017823 45018142 C HSAT6 Satellite 311 1**

**45021329 45022337 C L1M4 LINE/L1 4478 3398**

**45024197 45024496 + AluSx SINE/Alu 1 297**

**45026058 45026091 + (TTG)n Simple_repeat 2 35**

**_________________________________________________________________________**

**L1HS_16_16c 57585219-57585770 C_INTER_RMD_M_DISRUPTED**

**57574936 57577167 + L1M3b LINE/L1 2259 4474**

**57577173 57579399 + L1MA7 LINE/L1 93 2232**

**57579400 57579648 C AluJo SINE/Alu 262 1**

**57579649 57582010 + L1MA7 LINE/L1 2233 4502**

**57582011 57582877 + L1MA7 LINE/L1 4849 5708**

**57582877 57584761 C L1P2 LINE/L1 1921 22**

**57584762 57584806 + (TTTA)n Simple_repeat 2 48**

**57584809 57585180 C L1PA4 LINE/L1 6147 5776 (R2)**

**57585181 57585214 + (TTTA)n Simple_repeat 2 36**

**57585219 57585770 C L1HS LINE/L1 6154 5603 ( L1HS_16_16c ) (R1)**

**57585794 57586649 + L1MA7 LINE/L1 5698 6596**

**57586651 57587262 + L1PB1 LINE/L1 5470 6107**

**57587266 57587284 + (TAAAAA)n Simple_repeat 2 20**

**57587285 57587730 + L1MA7 LINE/L1 6580 7011**

**57587790 57587910 C MIR SINE/MIR 144 20**

**57588519 57588716 C MIRb SINE/MIR 211 8**

**57589106 57589135 + (T)n Simple_repeat 1 30**

**57589425 57589445 + AT_rich Low_complexity 1 21**

**57589495 57589675 + MIRb SINE/MIR 39 265**

**57589771 57590067 C AluY SINE/Alu 297 1**

**57590380 57590434 + (TCTA)n Simple_repeat 4 58**

**57590435 57590724 C AluSq SINE/Alu 282 1**

**57591550 57591772 C MIRb SINE/MIR 251 5**

**57591909 57591932 + (TC)n Simple_repeat 1 24**

**57591933 57591965 + (CA)n Simple_repeat 1 34**

**57591967 57592046 + L2 LINE/L2 2377 2457**

**57592078 57592366 + L2 LINE/L2 2530 2835**

**57592417 57592605 C AluY SINE/Alu 298 110**

**57592732 57592865 + L2 LINE/L2 3041 3157**

**57592892 57594098 C L1PA4 LINE/L1 6155 4944**

**57594201 57594313 + L2 LINE/L2 3288 3412**

**57594321 57594651 C LTR16A LTR/ERVL 415 35**

**57594659 57594818 C MER63 DNA/AcHobo 165 6**

**Ortholog in Chimp 58497652-58497821 Minus Nscore 0.00**

**N positions**

**58487393 58489624 + L1M3b LINE/L1 2295 4474**

**58489630 58491852 + L1MA7 LINE/L1 93 2226**

**58491853 58492101 C AluJo SINE/Alu 262 1**

**58492102 58494472 + L1MA7 LINE/L1 2227 4502**

**58494473 58495338 + L1MA7 LINE/L1 4849 5708**

**58495338 58497235 C L1P2 LINE/L1 1921 19**

**58497236 58497274 + AT_rich Low_complexity 1 39**

**58497277 58497821 C L1PA4 LINE/L1 6147 5603 (R12)**

**58497845 58498699 + L1MA7 LINE/L1 5698 6596**

**58498701 58499312 + L1PB1 LINE/L1 5470 6107**

**58499316 58499334 + (TAAAAA)n Simple_repeat 2 20**

**58499335 58499777 + L1MA7 LINE/L1 6580 7011**

**58499838 58499972 C MIR SINE/MIR 148 11**

**58500566 58500763 C MIRb SINE/MIR 211 8**

**58501468 58501488 + AT_rich Low_complexity 1 21**

**58501538 58501718 + MIRb SINE/MIR 39 265**

**58501814 58502115 C AluY SINE/Alu 298 1**

**58502423 58502743 C AluSq SINE/Alu 315 1**

**58503569 58503791 C MIRb SINE/MIR 251 5**

**58503928 58503983 + (TC)n Simple_repeat 1 56**

**58503984 58504010 + (CA)n Simple_repeat 1 28**

**58504012 58504091 + L2 LINE/L2 2377 2457**

**58504123 58504412 + L2 LINE/L2 2530 2835**

**58504463 58504648 C AluY SINE/Alu 295 110**

**58504774 58504907 + L2 LINE/L2 3041 3157**

**58504935 58506128 C L1PA4 LINE/L1 6155 4944**

**58506297 58506342 + L2 LINE/L2 3370 3412**

**58506350 58506680 C LTR16A LTR/ERVL 415 35**

**58506688 58506847 C MER63 DNA/AcHobo 165 6**

**_________________________________________________________________________**

**L1HS_16_23 79318488-79322115 M_INTRA_RMD**

**79315012 79315222 + MER58A DNA/MER1_type 6 224**

**79315225 79315351 + GA-rich Low_complexity 1 124**

**79315375 79315739 + GA-rich Low_complexity 1 353**

**79315994 79316133 + MER112 DNA/MER1_type 110 258**

**79316185 79316565 + L2 LINE/L2 2690 3089**

**79316566 79316619 + (CA)n Simple_repeat 2 55**

**79316620 79316932 + L2 LINE/L2 3090 3378**

**79317502 79317603 + L1MC5 LINE/L1 7809 7913**

**79318014 79318164 + L1MC3 LINE/L1 6705 6857**

**79318204 79318481 + AluJb SINE/Alu 1 296**

**79318488 79322115 + L1HS LINE/L1 7 3631 ( L1HS_16_23 )**

**79322113 79322525 + L1ME1 LINE/L1 5750 6161**

**79322515 79323414 + HAL1 LINE/L1 953 1881**

**79323584 79323707 + MER104 DNA/Tc2 1 125**

**79323890 79324024 + HAL1b LINE/L1 1852 1989**

**79325170 79325373 + L2 LINE/L2 2700 2914**

**79325669 79325726 + L4 LINE/RTE 946 1003**

**79326028 79326103 C L2 LINE/L2 3378 3306**

**79326785 79326926 + MIR SINE/MIR 108 260**

**79327951 79328007 + MIR SINE/MIR 1 58**

**Ortholog in Chimp 80826702-80835146 Plus Nscore 8.85**

**N positions 80832198-80832353; 80833567-80834157;**

**80823202 80823417 + MER58A DNA/MER1_type 3 224**

**80823420 80823551 + GA-rich Low_complexity 1 129**

**80823566 80823935 + GA-rich Low_complexity 1 358**

**80824211 80824350 + MER112 DNA/MER1_type 110 258**

**80824402 80824781 + L2 LINE/L2 2690 3089**

**80824782 80824826 + (CA)n Simple_repeat 2 46**

**80824827 80825139 + L2 LINE/L2 3090 3378**

**80825684 80825810 + L1MC5 LINE/L1 7776 7913**

**80826220 80826365 + L1MC3 LINE/L1 6705 6851**

**80826368 80826402 + (CAA)n Simple_repeat 1 36**

**80826417 80826695 + AluJo SINE/Alu 1 297**

**80826702 80832197 + L1Pt LINE/L1 7 5505**

**80832380 80833544 + L1ME1 LINE/L1 3443 4667**

**80834165 80835555 + L1ME1 LINE/L1 4742 6161**

**80835569 80836450 + HAL1 LINE/L1 975 1881**

**80836620 80836739 + MER104 DNA/Tc2 1 125**

**80838216 80838419 + L2 LINE/L2 2700 2914**

**80839081 80839156 C L2 LINE/L2 3378 3306**

**80839763 80839783 + AT_rich Low_complexity 1 21**

**80839842 80839981 + MIR SINE/MIR 108 260**

**80841006 80841062 + MIR SINE/MIR 1 58**

**80841082 80841199 + FLAM_C SINE/Alu 1 118**

**80841208 80841329 C Charlie2 DNA/MER1_type 1998 1862**

**80841361 80841652 C Charlie2 DNA/MER1_type 324 16**

**_________________________________________________________________________**

**L1HS_16_24 80568460-80569573 C_INTER_RMD_M_DISRUPTED**

**80559167 80560594 C L1M LINE/L1 5663 4202**

**80560595 80560979 + MSTB LTR/MaLR 1 426**

**80560980 80563334 C L1M1 LINE/L1 4201 1175**

**80563431 80563502 + C-rich Low_complexity 2 70**

**80563503 80563573 C L1M3c LINE/L1 1453 1381**

**80563591 80563642 + (CA)n Simple_repeat 2 53**

**80563651 80563927 C AluJo SINE/Alu 287 12**

**80563943 80563971 + AT_rich Low_complexity 1 29**

**80564092 80564306 + L1M5 LINE/L1 3702 3970**

**80564312 80564524 + L1PA16 LINE/L1 1 218**

**80564506 80565635 + L1PA16 LINE/L1 845 1991**

**80565636 80565937 + AluY SINE/Alu 1 306**

**80565938 80567839 + L1PA16 LINE/L1 1992 3903**

**80567840 80568149 C AluSg SINE/Alu 310 1**

**80568150 80568459 + L1PA16 LINE/L1 3904 4210**

**80568460 80569573 + L1HS LINE/L1 1 1114 ( L1HS_16_24 ) (R1)**

**80569574 80569891 + AluYb8 SINE/Alu 1 318 (D)**

**80569892 80570964 + L1HS LINE/L1 1115 2180 (R2)**

**80570969 80573538 + L1PA3 LINE/L1 4283 6858**

**80573536 80575974 + L1PA16 LINE/L1 4195 6752**

**80575975 80576281 C AluSx SINE/Alu 304 1**

**80576282 80576404 + L1PA16 LINE/L1 6753 6869**

**80576425 80576917 + L1M5 LINE/L1 3957 4461**

**80576918 80577218 + AluSx SINE/Alu 1 293**

**80577219 80577426 + L1M5 LINE/L1 4462 4659**

**80577584 80578212 + L1M5 LINE/L1 4878 5584**

**80578327 80578587 + MLT1J LTR/MaLR 224 615**

**Ortholog in Chimp 82124509-82125613 Plus Nscore 0.00**

**N positions**

**82115403 82117846 C L1M4c LINE/L1 4197 1175**

**82117849 82117940 + CT-rich Low_complexity 3 96**

**82118010 82118065 + (CA)n Simple_repeat 2 57**

**82118074 82118360 C AluJo SINE/Alu 287 2**

**82118587 82118661 + L1M5 LINE/L1 3799 3873**

**82118741 82118955 + L1PA15-16 LINE/L1 1 218**

**82118937 82120065 + L1PA15-16 LINE/L1 845 1991**

**82120066 82120368 + AluY SINE/Alu 1 304**

**82120369 82122292 + L1PA15-16 LINE/L1 1992 3904**

**82122300 82122575 C AluSg SINE/Alu 307 32**

**82122590 82123887 + L1PA16 LINE/L1 2615 3905**

**82123888 82124197 C AluSg SINE/Alu 310 1**

**82124198 82124506 + L1PA16 LINE/L1 3906 4209**

**82124509 82130044 + L1Pt LINE/L1 1 5534**

**82130029 82132203 + L1PA3 LINE/L1 3982 6155**

**82132203 82134639 + L1PA16 LINE/L1 4198 6752**

**_________________________________________________________________________**

**L1HS_17_2 3198430-3202087 C_DISRUPTED_M_INTER_RMD**

**3187327 3193435 C L1PA5 LINE/L1 6146 5**

**3194174 3194196 + MLT1K LTR/MaLR 38 60**

**3194197 3194706 + LTR55 LTR/ERV 1 548**

**3194707 3194915 + MLT1K LTR/MaLR 61 301**

**3194975 3195056 C MLT1K LTR/MaLR 453 372**

**3195226 3195983 + HAL1 LINE/L1 637 1454**

**3196039 3196161 + L1MEb LINE/L1 3 126**

**3196181 3196291 C L1MA7 LINE/L1 5882 5771**

**3196292 3196393 C L1MA2 LINE/L1 6304 6202**

**3196396 3196471 + MLT1K LTR/MaLR 220 297**

**3196541 3197229 C L1MA2 LINE/L1 6197 5509**

**3197232 3197287 C L1M LINE/L1 6826 6771**

**3197288 3198430 C L1PA3 LINE/L1 6155 5013**

**3198430 3202087 + L1HS LINE/L1 1220 4878 ( L1HS_17_2 ) (R12)**

**3202086 3203719 C L1M LINE/L1 6783 5283**

**3203720 3203991 C L1MA1 LINE/L1 6302 6020**

**3203992 3204355 C L1M LINE/L1 5282 4929**

**3204356 3204445 C AluJb SINE/Alu 301 211**

**3204446 3205472 C L1M LINE/L1 4928 3928**

**3205473 3205505 + (TC)n Simple_repeat 2 34**

**3205506 3206535 C L1M1 LINE/L1 3925 2889**

**3206536 3206640 + L1M1 LINE/L1 3356 3482**

**3207445 3207608 + L1MC1 LINE/L1 5837 5999**

**3207491 3207752 + L1MB3 LINE/L1 5881 6144**

**3207758 3207889 C AluJo SINE/Alu 136 9**

**3207898 3207941 + L1MB4 LINE/L1 6136 6179**

**Ortholog in Chimp 3387661-3395127 Plus Nscore 7.55**

**N positions 3389554-3390107; 3390945-3390954;**

**3377473 3382676 C L1PA5 LINE/L1 5228 4**

**3383414 3383436 + MLT1K LTR/MaLR 38 60**

**3383437 3383947 + LTR55 LTR/ERV 1 548**

**3383948 3384156 + MLT1K LTR/MaLR 61 301**

**3384168 3384297 C MLT1K LTR/MaLR 506 372**

**3384467 3385214 + HAL1 LINE/L1 637 1454**

**3385308 3385392 + L1MEa LINE/L1 43 126**

**3385412 3385522 C L1MA7 LINE/L1 5882 5771**

**3385523 3385624 C L1MA2 LINE/L1 6304 6202**

**3385627 3385702 + MLT1K LTR/MaLR 220 297**

**3385772 3386463 C L1MA2 LINE/L1 6197 5509**

**3386466 3386521 C L1M LINE/L1 5407 5352**

**3386519 3387661 C L1PA3 LINE/L1 6155 5013**

**3387661 3389553 + L1P1 LINE/L1 1343 3237**

**3390108 3390183 + L1P1 LINE/L1 4174 4249**

**3390174 3390457 C L1P1 LINE/L1 3981 3695**

**3390456 3390955 + L1P1 LINE/L1 3977 4480**

**3390962 3394302 + L1PA2 LINE/L1 2796 6155**

**3394359 3395127 + L1P1 LINE/L1 4233 5001**

**3395126 3396744 C L1M1 LINE/L1 5367 3835**

**3396764 3396976 C L1M1 LINE/L1 5457 5283**

**3396977 3397249 C L1MA1 LINE/L1 6302 6020**

**3397250 3399772 C L1M1 LINE/L1 5282 2889**

**3397614 3397703 C AluJb SINE/Alu 301 211**

**3399775 3399877 + L1M1 LINE/L1 3355 3482**

**3400728 3400989 + L1MB3 LINE/L1 5881 6144**

**3400995 3401126 C AluJo SINE/Alu 136 9**

**_________________________________________________________________________**

**L1HS_18_22c 39855243-39858789 M_INTRA_RMD**

**39850781 39851569 C L1PA17 LINE/L1 6165 5336**

**39851571 39851797 + L1P4 LINE/L1 3111 3339**

**39851798 39852115 + AluSx SINE/Alu 1 308**

**39852116 39852578 + L1P4 LINE/L1 3340 3801**

**39852622 39852825 C L1M5 LINE/L1 4069 3848**

**39852906 39853044 C L1MDa LINE/L1 2847 2692**

**39853045 39853366 C AluSx SINE/Alu 303 1**

**39853367 39854106 C L1MDa LINE/L1 2691 1849**

**39854562 39854582 + AT_rich Low_complexity 1 21**

**39854653 39855242 C L1PA5 LINE/L1 6154 5565**

**39855243 39858789 C L1HS LINE/L1 3571 1 ( L1HS_18_22c )**

**39860354 39860477 + MIR SINE/MIR 123 252**

**39860489 39860579 + L2 LINE/L2 3328 3419**

**39860974 39861064 + MSTA LTR/MaLR 338 428**

**39861129 39861924 C THE1-int LTR/MaLR 1514 732**

**39862220 39862976 C MSTA-int LTR/MaLR 784 1**

**39862977 39863354 C MSTA LTR/MaLR 428 1**

**39863752 39863842 + MIR SINE/MIR 41 137**

**39864000 39864980 + L1M1 LINE/L1 2592 3618**

**Ortholog in Chimp 40180532-40188625 Minus Nscore 9.80**

**N positions 40185686-40186468; 40184730-40184739;**

**40176075 40176863 C L1PA17 LINE/L1 6165 5336**

**40176865 40177091 + L1P4 LINE/L1 3111 3339**

**40177092 40177410 + AluSx SINE/Alu 1 309**

**40177411 40177875 + L1P4 LINE/L1 3340 3801**

**40177919 40178162 C L1M5 LINE/L1 4069 3808**

**40178203 40178356 C L1MDa LINE/L1 2847 2690**

**40178357 40178664 C AluSx SINE/Alu 289 1**

**40178665 40179405 C L1MDa LINE/L1 2774 1849**

**40179861 40179881 + AT_rich Low_complexity 1 21**

**40179951 40184700 C L1PA5 LINE/L1 6031 1226**

**40184740 40185685 C L1Pt LINE/L1 3154 2200**

**40186469 40188625 C L1Pt LINE/L1 2161 1**

**40190078 40190298 + MIRb SINE/MIR 21 260**

**40190306 40190396 + L2 LINE/L2 3328 3419**

**40190782 40190880 + MSTA LTR/MaLR 329 428**

**40190945 40191738 C THE1-int LTR/MaLR 1514 732**

**40192033 40192789 C MSTA-int LTR/MaLR 784 1**

**40192790 40193169 C MSTA LTR/MaLR 428 1**

**40193567 40193657 + MIR SINE/MIR 41 137**

**40193815 40194795 + L1M1 LINE/L1 2592 3620**

**_________________________________________________________________________**

**L1HS_18_38 63421851-63423817 C_DISRUPTED_M_INTER_RMD**

**63416347 63416713 C MLT1A LTR/MaLR 374 4**

**63416800 63416906 + MIRm SINE/MIR 14 133**

**63416907 63416927 + AT_rich Low_complexity 1 21**

**63416928 63417236 C AluY SINE/Alu 308 1**

**63417239 63417307 + MIR3 SINE/MIR 119 187**

**63419248 63420412 + Tigger1 DNA/MER2_type 1 1189**

**63420410 63420603 + Tigger1 DNA/MER2_type 2184 2380**

**63420673 63420700 + (TG)n Simple_repeat 1 28**

**63421170 63421205 + (TTTTTA)n Simple_repeat 2 36**

**63421307 63421629 + MLT1B LTR/MaLR 1 459**

**63421631 63421667 + (A)n Simple_repeat 1 37**

**63421851 63423817 + L1HS LINE/L1 2 1972 ( L1HS_18_38 )**

**63423818 63423938 + L1PA4 LINE/L1 6031 6152**

**63423939 63424335 + MLT2A1 LTR/ERVL 13 503**

**63424336 63424358 + (CA)n Simple_repeat 2 24**

**63424476 63424524 + (TA)n Simple_repeat 2 48**

**63424976 63425323 C THE1D LTR/MaLR 381 1**

**63425702 63425912 + MIRb SINE/MIR 56 268**

**63427375 63427458 + CT-rich Low_complexity 4 90**

**63427950 63427978 + (CAAAA)n Simple_repeat 2 30**

**63428405 63428546 C L2 LINE/L2 3414 3266**

**63429340 63429370 + AT_rich Low_complexity 1 31**

**Ortholog in Chimp 64292213-64298265 Plus Nscore 8.11**

**N positions 64292393-64292863; 64294442-64294451; 64297798-64297807;**

**64286712 64287078 C MLT1A LTR/MaLR 374 4**

**64287165 64287271 + MIRm SINE/MIR 14 133**

**64287272 64287298 + AT_rich Low_complexity 1 27**

**64287299 64287606 C AluY SINE/Alu 307 1**

**64289601 64290765 + Tigger1 DNA/MER2_type 1 1189**

**64290763 64290956 + Tigger1 DNA/MER2_type 2184 2380**

**64291026 64291057 + (TG)n Simple_repeat 1 32**

**64291527 64291564 + (T)n Simple_repeat 1 38**

**64291663 64291985 + MLT1B LTR/MaLR 1 459**

**64291987 64292030 + A-rich Low_complexity 1 44**

**64292212 64292392 + L1Pt LINE/L1 1 181 (R1) (BROKEN BECAUSE OF Ns)**

**64292864 64294441 + L1Pt LINE/L1 278 1858 (R1)**

**64294452 64295464 + Tigger1 DNA/MER2_type 174 1189**

**64295462 64295655 + Tigger1 DNA/MER2_type 2184 2380**

**64295725 64295752 + (TG)n Simple_repeat 1 28**

**64296221 64296244 + (T)n Simple_repeat 1 24**

**64296343 64296665 + MLT1B LTR/MaLR 1 459**

**64296667 64296708 + (A)n Simple_repeat 1 42**

**64296890 64297797 + L1Pt LINE/L1 1 910**

**64297808 64298265 + L1Pt LINE/L1 1515 1972 (R2)**

**64298266 64298391 + L1PA4 LINE/L1 6031 6155**

**64298392 64298776 + MLT2A1 LTR/ERVL 13 404**

**64298785 64298805 + (CA)n Simple_repeat 2 22**

**64298923 64298963 + (TA)n Simple_repeat 2 40**

**64299406 64299753 C THE1D LTR/MaLR 381 1**

**64300096 64300342 + MIRb SINE/MIR 19 268**

**64301814 64301896 + CT-rich Low_complexity 2 80**

**64302390 64302415 + (CAAAA)n Simple_repeat 2 27**

**64302871 64303012 C L2 LINE/L2 3414 3266**

**64303806 64303834 + AT_rich Low_complexity 1 29**

**_________________________________________________________________________**

**L1HS_19_12c 59783480-59789179 C_INTER_RMD_M_DISRUPTED**

**59775052 59775087 + AT_rich Low_complexity 1 36**

**59776277 59776552 C AluJo SINE/Alu 286 11**

**59778931 59779002 + GA-rich Low_complexity 2 72**

**59781415 59783471 C L1PA6 LINE/L1 6148 4068 (R2)**

**59783480 59789179 C L1HS LINE/L1 6155 5 ( L1HS_19_12c ) (R1)**

**59791259 59791364 C MER63 DNA/AcHobo 1014 909**

**59791602 59791899 + AluY SINE/Alu 2 296**

**59791955 59792244 C AluSp SINE/Alu 291 1**

**59792290 59792580 C MLT1C LTR/MaLR 269 1**

**59792620 59792792 + AluSg/x SINE/Alu 123 295**

**59792793 59792913 + L1M4 LINE/L1 4421 4541**

**59792915 59792951 + MLT2A2 LTR/ERVL 525 561**

**59792961 59793151 C L1P4c LINE/L1 764 1**

**59794059 59794343 + LTR42 LTR/ERVL 3 267**

**59794347 59794490 + MER65D LTR/ERV1 4 147**

**59794544 59794720 + MER65D LTR/ERV1 157 341**

**59794733 59795252 C MER21A LTR/ERV1 923 376**

**59795258 59795499 C MER21A LTR/ERV1 264 1**

**59795500 59795586 + MER65D LTR/ERV1 342 427**

**59795587 59795825 + MER8 DNA/MER2_type 1 239**

**59795826 59795862 + MER65D LTR/ERV1 428 463**

**59795885 59796104 + LTR42 LTR/ERVL 265 491**

**59796511 59796623 C MIRb SINE/MIR 181 68**

**Ortholog in Chimp 60308686-60311189 Minus Nscore 0.00**

**N positions**

**60299903 60299938 + AT_rich Low_complexity 1 36**

**60301508 60301783 C AluJo SINE/Alu 286 11**

**60306629 60310172 C L1PA6 LINE/L1 6148 2571 (R12- further undergone intra-element recombination)**

**60310169 60311189 C L1P2 LINE/L1 1057 5 (R12- further undergone intra-element recombination)**

**60313264 60313369 C MER63 DNA/AcHobo 1014 909**

**60313607 60313907 + AluY SINE/Alu 2 298**

**60313965 60314040 C AluSp/q SINE/Alu 289 213**

**60314681 60314965 C AluSx SINE/Alu 285 1**

**60315011 60315302 C MLT1C LTR/MaLR 269 1**

**60315342 60315514 + AluSg/x SINE/Alu 123 295**

**60315515 60315644 + L1M4 LINE/L1 4421 4549**

**60315681 60315873 C L1P4c LINE/L1 759 1**

**60315916 60316241 C L1M4 LINE/L1 3488 3155**

**60316782 60317067 + LTR42 LTR/ERVL 3 267**

**60317071 60317214 + MER65D LTR/ERV1 4 147**

**60317268 60317444 + MER65D LTR/ERV1 157 341**

**60317457 60317964 C MER21A LTR/ERV1 923 389**

**60317948 60318223 C MER21A LTR/ERV1 285 5**

**60318218 60318307 + MER65D LTR/ERV1 337 427**

**60318308 60318546 + MER8 DNA/MER2_type 1 239**

**60318547 60318583 + MER65D LTR/ERV1 428 463**

**60318606 60318819 + LTR42 LTR/ERVL 265 491**

**60319218 60319338 C MIRb SINE/MIR 194 68**

**_________________________________________________________________________**

**L1HS_X_45c 70935033-70938407 C_INTRA_RMD**

**70929042 70930266 + L1MD LINE/L1 3475 4704**

**70930267 70930577 + AluSc SINE/Alu 6 315**

**70930578 70930600 + L1MD LINE/L1 4705 4726**

**70930601 70930893 C AluJo SINE/Alu 311 38**

**70930894 70931028 + L1MD LINE/L1 4727 4860**

**70931038 70931323 C L1MB5 LINE/L1 6165 5889**

**70931324 70931633 C AluJb SINE/Alu 305 1**

**70931634 70932669 C L1MB5 LINE/L1 5888 4843**

**70932669 70933786 + L1M3e LINE/L1 1200 2352**

**70933787 70934057 C AluSq SINE/Alu 270 1**

**70934058 70934286 + L1M3e LINE/L1 2353 2590**

**70934321 70934639 C AluJb SINE/Alu 309 1**

**70934640 70935039 + L1M3e LINE/L1 3008 3429**

**70935033 70938407 C L1HS LINE/L1 4274 895 ( L1HS_X_45c ) (R1,R2)**

**70938407 70940163 + L1PA4 LINE/L1 4395 6151**

**70940154 70940358 + L1M3 LINE/L1 3555 3770**

**70940410 70941770 + L1M1 LINE/L1 297 1678**

**70941768 70942638 + L1M3e LINE/L1 3586 4467**

**70942627 70942856 + L1M3e LINE/L1 4972 5193**

**70942896 70942933 + (TA)n Simple_repeat 2 39**

**70942934 70944190 C L1PA6 LINE/L1 6140 4884**

**70944190 70945499 + L1PA6 LINE/L1 3550 4861**

**Ortholog in Chimp 71122692-71125420 Minus Nscore 1.10**

**N positions 71124693-71124722;**

**71121787 71121910 + L1M3e LINE/L1 2412 2544**

**71121896 71121941 + L1M3e LINE/L1 2968 3014**

**71121991 71122298 C AluJb SINE/Alu 298 1**

**71122299 71122698 + L1M3e LINE/L1 3008 3429**

**71122692 71124692 C L1Pt LINE/L1 4274 2270 (R12)**

**71124723 71125420 C L1Pt LINE/L1 1593 896 (R12)**

**71125420 71127175 + L1PA4 LINE/L1 4395 6151**

**71127166 71127383 + L1M3 LINE/L1 3555 3770**

**71127435 71128795 + L1M1 LINE/L1 297 1678**

**71128793 71129663 + L1M3e LINE/L1 3586 4467**

**71129652 71129881 + L1M3e LINE/L1 4972 5193**

**71129921 71129963 + (TA)n Simple_repeat 2 44**

**71129964 71130104 C L1PA6 LINE/L1 6135 5994**

**71130661 71131588 C L1PA6 LINE/L1 5811 4884**

**_________________________________________________________________________**

**L1HS_X_85 108033780-108039316 C_INTER_RMD_M_DISRUPTED**

**108023320 108025407 + L1M3e LINE/L1 10 2517**

**108025454 108025815 + THE1B LTR/MaLR 1 362**

**108025818 108027336 + THE1B-int LTR/MaLR 1 1580**

**108027337 108027699 + THE1B LTR/MaLR 1 364**

**108027700 108028185 + L1M1 LINE/L1 2676 3168**

**108028188 108030669 + L1M1 LINE/L1 3329 5742**

**108030670 108030886 + L1PB1 LINE/L1 5934 6151**

**108030887 108031875 + L1M1 LINE/L1 5743 6742**

**108031879 108033770 + L1PB1 LINE/L1 6 1915**

**108033780 108039316 + L1HS LINE/L1 11 5662 ( L1HS_X_85 )**

**108039318 108039516 + HERVH LTR/ERV1 5626 5824**

**108039511 108039886 + HERVH LTR/ERV1 7324 7713**

**108039887 108040290 + LTR7 LTR/ERV1 1 407**

**108040291 108040318 + (GAAAA)n Simple_repeat 3 30**

**108040319 108040691 + L1PA5 LINE/L1 5777 6149**

**108040699 108044754 + L1PB1 LINE/L1 1898 6045**

**108044761 108045244 + L1PA10 LINE/L1 5693 6177**

**108045264 108045405 + MLT1A LTR/MaLR 185 341**

**108045414 108046264 + L1PB1 LINE/L1 6036 6867**

**108046260 108046848 + L1MA8 LINE/L1 5296 5918**

**108046849 108047099 + L1MA8 LINE/L1 6031 6290**

**108047100 108047167 + L1MA4 LINE/L1 5919 6021**

**108047488 108048343 + L2 LINE/L2 2025 2956**

**Ortholog in Chimp 108418801-108424245 Plus Nscore 3.36**

**N positions 108421001-108421171; 108421231-108421231; 108421234-108421234; 108424224-108424233;**

**108408036 108410111 + L1M3e LINE/L1 10 2517**

**108410158 108410519 + THE1B LTR/MaLR 1 362**

**108410522 108412039 + THE1B-int LTR/MaLR 1 1580**

**108412040 108412419 + THE1B LTR/MaLR 1 364**

**108412420 108412905 + L1M1 LINE/L1 2676 3168**

**108412908 108416490 + L1M1 LINE/L1 3329 6749**

**108416490 108416988 + L1PB1 LINE/L1 6 542**

**108417499 108418791 + L1PB1 LINE/L1 621 1915**

**108418801 108424208 + L1Pt LINE/L1 11 5382 (OCCUPIED)**

**108424246 108424396 + HERVH LTR/ERV1 5674 5824**

**108424391 108424766 + HERVH LTR/ERV1 7324 7713**

**108424767 108425129 + LTR7 LTR/ERV1 1 380**

**108425244 108426614 + L1PA5 LINE/L1 4743 6150**

**108426622 108430671 + L1PB1 LINE/L1 1898 6045**

**108430678 108431176 + L1PA10 LINE/L1 5693 6192**

**108431196 108431336 + MLT1A LTR/MaLR 185 341**

**108431347 108432197 + L1PB1 LINE/L1 6036 6867**

**108432207 108432587 + L1MA8 LINE/L1 5319 5725**

**108433078 108433318 + L1MA8 LINE/L1 6031 6290**

**_________________________________________________________________________**

**L1HS_Y_31c 21393557-21398291 C_DISRUPTED_M_INTER_RMD**

**21386975 21388798 + L1PA14 LINE/L1 1497 4850**

**21388816 21389753 + L1P2 LINE/L1 9 975**

**21389755 21391639 + L1P2 LINE/L1 1 1887**

**21391662 21392937 C L1PA4 LINE/L1 6152 4879**

**21392924 21393561 C L1PA4 LINE/L1 1715 1066**

**21393557 21398291 C L1HS LINE/L1 4754 4 ( L1HS_Y_31c ) (R12)**

**21398292 21398427 + L1PREC2 LINE/L1 4952 5069**

**21398428 21398760 + L1PA3 LINE/L1 5850 6183**

**21398761 21399036 + L1PREC2 LINE/L1 5070 5386**

**21399037 21399335 C AluY SINE/Alu 299 1**

**21399336 21399420 + L1PA15 LINE/L1 5387 5464**

**21399421 21399716 C AluSp SINE/Alu 299 1**

**21399717 21399881 + L1PA15 LINE/L1 5465 5611**

**21399882 21400184 + AluY SINE/Alu 1 303**

**21400185 21400253 + L1PA15 LINE/L1 5612 5680**

**21400254 21400283 + (CA)n Simple_repeat 2 32**

**21400284 21400366 + L1PA15 LINE/L1 5681 5768**

**21400367 21400668 C AluY SINE/Alu 301 1**

**21400669 21401062 + L1PA15 LINE/L1 5769 6162**

**21402358 21402558 C L1PB4 LINE/L1 5976 5774**

**21402571 21402710 + L1PB4 LINE/L1 6017 6156**

**21402768 21403059 + AluSg SINE/Alu 1 294**

**21403079 21403099 + AT_rich Low_complexity 1 21**

**21403703 21404725 C L1PREC2 LINE/L1 6159 5112**

**Ortholog in Chimp 14906949-14923038 Plus Nscore 0.00**

**N positions**

**14900419 14901831 + L1PREC2 LINE/L1 4551 6159**

**14902435 14902455 + AT_rich Low_complexity 1 21**

**14902475 14902770 C AluSg SINE/Alu 298 1**

**14902828 14902967 C L1PB4 LINE/L1 6156 6017**

**14902980 14903177 + L1PB4 LINE/L1 5774 5973**

**14904494 14904887 C L1PA15 LINE/L1 6163 5770**

**14904888 14905195 + AluY SINE/Alu 1 308**

**14905196 14905271 C L1PA15 LINE/L1 5769 5698**

**14905272 14905305 + (TG)n Simple_repeat 2 36**

**14905306 14905372 C L1PA15 LINE/L1 5697 5618**

**14905373 14905675 C AluY SINE/Alu 303 1**

**14905676 14905840 C L1PA15 LINE/L1 5617 5466**

**14905841 14906138 + AluSp SINE/Alu 1 301**

**14906139 14906511 C L1PA15 LINE/L1 5465 5070**

**14906512 14906813 C L1PA3 LINE/L1 6155 5850**

**14906814 14906948 C L1PREC2 LINE/L1 5069 4952**

**14906949 14912983 + L1PA2 LINE/L1 4 6032 (R1)**

**14912983 14913605 C L1PREC2 LINE/L1 4968 4328**

**14913618 14914293 C L1PA6 LINE/L1 6154 5490**

**14914294 14914599 C AluY SINE/Alu 306 1**

**14914600 14918220 C L1PA6 LINE/L1 5489 1874**

**14918222 14923038 + L1P1 LINE/L1 1 4877 (R2)**

**14923033 14923669 + L1PA4 LINE/L1 1065 1704**

**14923668 14924945 + L1PA4 LINE/L1 4879 6152**

**14924968 14926857 C L1PA6 LINE/L1 1887 1**

**14926859 14927796 C L1P2 LINE/L1 975 9**

**14927814 14929402 C L1PA14 LINE/L1 4850 3245**

**_________________________________________________________________________**

**Supplementary file 1 - Recombination mediated deletions – Truncated L1HS elements, Human Vs Celera comparison**

L1HS_3_14c C_INTER_RMD ??

189 7.4 0.0 0.0 chr3 22060937 22060963 (177440864) + (CAAAAA)n Simple_repeat 4 30 (0) 37

450 32.2 11.7 0.8 chr3 22061065 22061330 (177440497) C L2 LINE/L2 (1667) 1752 1458 36

466 12.7 0.0 0.0 chr3 22061452 22061522 (177440305) + AluSp/q SINE/Alu 226 296 (17) 38

300 14.5 1.6 0.0 chr3 22061523 22061584 (177440243) C L1M2 LINE/L1 (1817) 4326 4264 39

728 20.1 7.7 1.6 chr3 22061616 22061797 (177440030) C L1M2 LINE/L1 (1010) 5133 4941 40

7282 15.7 6.0 3.6 chr3 22063602 22065510 (177436317) C L1MA8 LINE/L1 (2) 6289 4316 41

2028 12.4 0.3 0.0 chr3 22065511 22065836 (177435991) + AluSq SINE/Alu 1 327 (0) 42

7282 17.9 8.8 3.6 chr3 22065837 22066576 (177435251) C L1MA8 LINE/L1 (1828) 4315 3551 41

1379 22.6 1.4 0.0 chr3 22066580 22066854 (177434973) + AluJo SINE/Alu 1 279 (33) 43

231 6.1 0.0 0.0 chr3 22066855 22066887 (177434940) + (CAAA)n Simple_repeat 1 33 (0) 44

**16108 0.3 0.0 0.0 chr3 22066888 22069693 (177432134) C L1HS LINE/L1 (2586) 3437 329 45 (R1)**

**20482 0.2 0.0 0.0 chr3 22069692 22072804 (177429023) + L1HS LINE/L1 3064 6176 (0) 46 (R2)**

335 28.2 3.1 3.9 chr3 22073187 22073315 (177428512) C MLT1H LTR/MaLR (16) 533 406 47

245 33.3 0.0 0.0 chr3 22073404 22073481 (177428346) C MLT1H2 LTR/MaLR (323) 161 84 48

646 29.4 3.1 5.3 chr3 22073622 22073874 (177427953) + MIRb SINE/MIR 14 262 (0) 49

2103 12.5 0.0 1.3 chr3 22074010 22074309 (177427518) C AluSx SINE/Alu (16) 296 1 50

216 9.7 0.0 0.0 chr3 22074491 22074521 (177427306) + (TGAA)n Simple_repeat 3 33 (0) 51

23 0.0 0.0 0.0 chr3 22075113 22075135 (177426692) + AT_rich Low_complexity 1 23 (0) 52

22 0.0 0.0 0.0 chr3 22075176 22075197 (177426630) + AT_rich Low_complexity 1 22 (0) 53

274 23.2 2.9 1.4 chr3 22075816 22075885 (177425942) C MER5B DNA/MER1_type (104) 74 4 54

282 29.6 0.0 0.0 chr3 22075963 22076050 (177425777) C Charlie2 DNA/MER1_type (2598) 263 176 55

288 24.5 7.9 3.0 chr3 22076077 22076177 (177425650) C Charlie2 DNA/MER1_type (2751) 110 5 55

Ortholog in Celera

SW perc perc perc query position in query matching repeat position in repeat

score div. del. ins. sequence begin end (left) repeat class/family begin end (left) ID

5371 15.8 6.9 3.0 gi|89161204:22028483-22038898 1666 3574 (6842) C L1MA8 LINE/L1 (2) 6289 4316 1

2095 12.4 0.3 0.0 gi|89161204:22028483-22038898 3575 3900 (6516) + AluSq SINE/Alu 1 313 (0) 2

3013 18.1 7.7 5.1 gi|89161204:22028483-22038898 3901 4640 (5776) C L1MA8 LINE/L1 (1828) 4315 3551 1

1507 22.6 1.4 0.0 gi|89161204:22028483-22038898 4644 4918 (5498) + AluJo SINE/Alu 1 279 (33) 3

231 6.1 0.0 0.0 gi|89161204:22028483-22038898 4919 4951 (5465) + (CAAA)n Simple_repeat 1 33 (0) 4

**871 16.3 6.2 0.5 gi|89161204:22028483-22038898 4992 5170 (5246) C L1MA8 LINE/L1 (2585) 3558 3370 1 (R12 ? )**

23 53.3 0.0 0.0 gi|89161204:22028483-22038898 5320 5349 (5067) + AT_rich Low_complexity 1 30 (0) 5

337 26.9 0.8 2.6 gi|89161204:22028483-22038898 5803 5931 (4485) C MLT1H2 LTR/ERVL-MaLR (16) 533 382 6

230 31.2 3.0 0.0 gi|89161204:22028483-22038898 6020 6120 (4296) C MLT1J2 LTR/ERVL-MaLR (328) 161 65 7

654 29.4 3.1 5.4 gi|89161204:22028483-22038898 6238 6490 (3926) + MIRb SINE/MIR 14 262 (0) 8

2188 12.3 0.0 1.4 gi|89161204:22028483-22038898 6626 6925 (3491) C AluSx SINE/Alu (16) 296 1 9

216 9.7 0.0 0.0 gi|89161204:22028483-22038898 7107 7137 (3279) + (TGAA)n Simple_repeat 3 33 (0) 10

25 56.0 0.0 0.0 gi|89161204:22028483-22038898 7728 7752 (2664) + AT_rich Low_complexity 1 25 (0) 11

22 54.5 0.0 0.0 gi|89161204:22028483-22038898 7792 7813 (2603) + AT_rich Low_complexity 1 22 (0) 12

230 26.7 10.0 0.0 gi|89161204:22028483-22038898 7826 7915 (2501) + Tigger16a DNA/TcMar-Tigger 742 840 (93) 13

267 23.2 2.9 1.4 gi|89161204:22028483-22038898 8432 8501 (1915) C MER5B DNA/hAT-Charlie (104) 74 4 14

284 29.6 0.0 0.0 gi|89161204:22028483-22038898 8579 8666 (1750) C Charlie2a DNA/hAT-Charlie (2598) 263 176 15

284 24.4 7.9 2.8 gi|89161204:22028483-22038898 8693 8793 (1623) C Charlie2a DNA/hAT-Charlie (2751) 110 5 15

423 18.8 11.6 5.0 gi|89161204:22028483-22038898 8796 8875 (1541) + L2a LINE/L2 3308 3394 (32) 16

2509 18.0 1.4 12.2 gi|89161204:22028483-22038898 8876 9445 (971) + MLT2B2 LTR/ERVL 1 515 (0) 17

423 18.8 11.6 5.0 gi|89161204:22028483-22038898 9446 9476 (940) + L2a LINE/L2 3395 3426 (0) 16

21 60.0 0.0 0.0 gi|89161204:22028483-22038898 9811 9845 (571) + AT_rich Low_complexity 1 35 (0) 18

26 81.5 0.0 0.0 gi|89161204:22028483-22038898 10019 10072 (344) + AT_rich Low_complexity 1 54 (0) 19

207 0.0 0.0 0.0 gi|89161204:22028483-22038898 10229 10251 (165) + (TTAAA)n Simple_repeat 4 26 (0) 20

_________________________________________________________________________________

L1HS_4_8 (Reference Human) M_DISRUPTED

203 33.3 5.3 6.1 chr4 16547199 16547329 (174725734) C MIR3 SINE/MIR (31) 177 48 220

704 17.0 0.0 0.9 chr4 16547348 16547460 (174725603) + FLAM_A SINE/Alu 8 119 (23) 221

2055 14.4 0.0 0.6 chr4 16547480 16547793 (174725270) C AluSx SINE/Alu (0) 312 1 222

286 34.1 3.2 2.4 chr4 16547995 16548120 (174724943) + MIRb SINE/MIR 40 166 (102) 223

1723 13.4 1.0 3.1 chr4 16548440 16548732 (174724331) + AluJb SINE/Alu 23 309 (3) 224

295 20.8 8.3 0.0 chr4 16550074 16550145 (174722918) + L1ME4a LINE/L1 5715 5792 (329) 225

365 30.1 7.1 5.2 chr4 16550581 16550888 (174722175) + L1ME4a LINE/L1 5789 6102 (19) 225

253 33.0 5.5 0.0 chr4 16551405 16551495 (174721568) C MIR3 SINE/MIR (46) 162 67 226

225 34.2 2.5 5.8 chr4 16551517 16551637 (174721426) C L2 LINE/L2 (5) 3373 3257 227

196 25.6 0.0 0.0 chr4 16551805 16551847 (174721216) + L2 LINE/L2 3373 3415 (4) 228

27 6.2 0.0 0.0 chr4 16551854 16551901 (174721162) + AT_rich Low_complexity 1 48 (0) 229

27 8.7 0.0 0.0 chr4 16551944 16552012 (174721051) + AT_rich Low_complexity 1 69 (0) 230

207 33.8 8.4 0.0 chr4 16552058 16552128 (174720935) C MIRb SINE/MIR (29) 239 163 231

523 33.1 7.9 4.7 chr4 16552156 16552409 (174720654) C MIR SINE/MIR (0) 262 1 232

404 13.4 10.4 0.0 chr4 16553284 16553350 (174719713) C MLT1C LTR/MaLR (0) 467 394 233

**19171 0.6 0.1 0.7 chr4 16553498 16555351 (174717712) + L1HS LINE/L1 3 1857 (4175) 234 (R1) (L1HS_4_8)**

**2782 0.7 0.0 0.0 chr4 16555352 16555647 (174717416) + AluYb8 SINE/Alu 23 318 (0) 235 (D)**

**29360 0.5 0.0 0.0 chr4 16555648 16559834 (174713229) + L1HS LINE/L1 1858 6031 (1) 236 (R2)**

2548 15.3 2.2 0.0 chr4 16559835 16560240 (174712823) C MLT1C LTR/MaLR (52) 415 1 233

2433 8.3 0.0 0.0 chr4 16560272 16560572 (174712491) C AluY SINE/Alu (9) 302 2 237

1921 12.0 0.0 9.6 chr4 16560600 16560913 (174712150) + AluSx SINE/Alu 1 284 (28) 238

182 3.7 3.7 0.0 chr4 16560914 16560940 (174712123) + (TAA)n Simple_repeat 2 29 (0) 239

2195 9.0 1.4 0.0 chr4 16560960 16561248 (174711815) C L1PA10 LINE/L1 (248) 5920 5628 240

2605 16.2 1.0 5.6 chr4 16561719 16562221 (174710842) + MLT1D LTR/MaLR 26 505 (0) 241

189 13.9 0.0 2.7 chr4 16563100 16563136 (174709927) C MIRm SINE/MIR (11) 265 230 242

213 3.9 0.0 0.0 chr4 16564221 16564246 (174708817) + (A)n Simple_repeat 1 26 (0) 243

2192 16.9 5.8 6.2 chr4 16565149 16565582 (174707481) C MSTB1 LTR/MaLR (0) 432 1 244

1257 16.7 1.2 7.4 chr4 16565590 16565847 (174707216) C MSTB-int LTR/MaLR (7) 1644 1403 244

3213 22.0 1.7 0.3 chr4 16565845 16566505 (174706558) C MSTB-int LTR/MaLR (981) 670 1 244

523 17.2 2.1 0.0 chr4 16566510 16566602 (174706461) C MSTB1 LTR/MaLR (335) 97 3 245

2464 19.1 2.9 0.0 chr4 16566759 16567172 (174705891) C MSTB LTR/MaLR (0) 426 1 244

Celera ortholog (5 kb upstream and downstream of the identified ortholog)

1078 14.6 1.1 3.3 gi|89161206:17404459-17416251 2 186 (11607) + AluJb SINE/Alu 129 309 (3) 1

271 19.4 8.3 0.0 gi|89161206:17404459-17416251 1528 1599 (10194) + L1MC LINE/L1 5713 5790 (2092) 2

684 25.6 3.6 1.5 gi|89161206:17404459-17416251 1612 1805 (9988) + LTR79 LTR/ERVL 20 217 (310) 3

357 26.1 18.9 0.5 gi|89161206:17404459-17416251 1837 2032 (9761) + LTR79 LTR/ERVL 296 527 (0) 3

224 33.0 5.5 0.0 gi|89161206:17404459-17416251 2859 2949 (8844) C MIR3 SINE/MIR (46) 162 67 4

205 22.7 0.0 0.0 gi|89161206:17404459-17416251 3258 3301 (8492) + L2c LINE/L2 3340 3383 (4) 5

27 72.9 0.0 0.0 gi|89161206:17404459-17416251 3308 3355 (8438) + AT_rich Low_complexity 1 48 (0) 6

27 89.9 0.0 0.0 gi|89161206:17404459-17416251 3398 3466 (8327) + AT_rich Low_complexity 1 69 (0) 7

183 33.8 8.4 0.0 gi|89161206:17404459-17416251 3512 3582 (8211) C MIRb SINE/MIR (29) 239 163 8

454 33.6 4.8 4.8 gi|89161206:17404459-17416251 3633 3863 (7930) C MIR SINE/MIR (31) 231 1 9

394 13.4 10.4 0.0 gi|89161206:17404459-17416251 4738 4804 (6989) C MLT1C LTR/ERVL-MaLR (0) 467 394 10

**29228 0.6 0.0 0.0 gi|89161206:17404459-17416251 4952 10979 (814) + L1HS LINE/L1 126 6154 (1) 11 (R)**

2534 15.3 2.2 0.0 gi|89161206:17404459-17416251 10980 11385 (408) C MLT1C LTR/ERVL-MaLR (52) 415 1 10

2450 8.3 0.0 0.0 gi|89161206:17404459-17416251 11417 11717 (76) C AluY SINE/Alu (9) 302 2 12

359 14.3 0.0 0.0 gi|89161206:17404459-17416251 11745 11793 (0) + AluYk11 SINE/Alu 1 49 (263) 13

__________________________________________________________________________________________________________________________

L1HS_4_9 (Reference Human) M_DISRUPTED

203 33.3 5.3 6.1 chr4 16547199 16547329 (174725734) C MIR3 SINE/MIR (31) 177 48 220

704 17.0 0.0 0.9 chr4 16547348 16547460 (174725603) + FLAM_A SINE/Alu 8 119 (23) 221

2055 14.4 0.0 0.6 chr4 16547480 16547793 (174725270) C AluSx SINE/Alu (0) 312 1 222

286 34.1 3.2 2.4 chr4 16547995 16548120 (174724943) + MIRb SINE/MIR 40 166 (102) 223

1723 13.4 1.0 3.1 chr4 16548440 16548732 (174724331) + AluJb SINE/Alu 23 309 (3) 224

295 20.8 8.3 0.0 chr4 16550074 16550145 (174722918) + L1ME4a LINE/L1 5715 5792 (329) 225

365 30.1 7.1 5.2 chr4 16550581 16550888 (174722175) + L1ME4a LINE/L1 5789 6102 (19) 225

253 33.0 5.5 0.0 chr4 16551405 16551495 (174721568) C MIR3 SINE/MIR (46) 162 67 226

225 34.2 2.5 5.8 chr4 16551517 16551637 (174721426) C L2 LINE/L2 (5) 3373 3257 227

196 25.6 0.0 0.0 chr4 16551805 16551847 (174721216) + L2 LINE/L2 3373 3415 (4) 228

27 6.2 0.0 0.0 chr4 16551854 16551901 (174721162) + AT_rich Low_complexity 1 48 (0) 229

27 8.7 0.0 0.0 chr4 16551944 16552012 (174721051) + AT_rich Low_complexity 1 69 (0) 230

207 33.8 8.4 0.0 chr4 16552058 16552128 (174720935) C MIRb SINE/MIR (29) 239 163 231

523 33.1 7.9 4.7 chr4 16552156 16552409 (174720654) C MIR SINE/MIR (0) 262 1 232

404 13.4 10.4 0.0 chr4 16553284 16553350 (174719713) C MLT1C LTR/MaLR (0) 467 394 233

**19171 0.6 0.1 0.7 chr4 16553498 16555351 (174717712) + L1HS LINE/L1 3 1857 (4175) 234 (R1)**

**2782 0.7 0.0 0.0 chr4 16555352 16555647 (174717416) + AluYb8 SINE/Alu 23 318 (0) 235 (D)**

**29360 0.5 0.0 0.0 chr4 16555648 16559834 (174713229) + L1HS LINE/L1 1858 6031 (1) 236 (R2) (L1HS_4_9)**

2548 15.3 2.2 0.0 chr4 16559835 16560240 (174712823) C MLT1C LTR/MaLR (52) 415 1 233

2433 8.3 0.0 0.0 chr4 16560272 16560572 (174712491) C AluY SINE/Alu (9) 302 2 237

1921 12.0 0.0 9.6 chr4 16560600 16560913 (174712150) + AluSx SINE/Alu 1 284 (28) 238

182 3.7 3.7 0.0 chr4 16560914 16560940 (174712123) + (TAA)n Simple_repeat 2 29 (0) 239

2195 9.0 1.4 0.0 chr4 16560960 16561248 (174711815) C L1PA10 LINE/L1 (248) 5920 5628 240

2605 16.2 1.0 5.6 chr4 16561719 16562221 (174710842) + MLT1D LTR/MaLR 26 505 (0) 241

189 13.9 0.0 2.7 chr4 16563100 16563136 (174709927) C MIRm SINE/MIR (11) 265 230 242

213 3.9 0.0 0.0 chr4 16564221 16564246 (174708817) + (A)n Simple_repeat 1 26 (0) 243

2192 16.9 5.8 6.2 chr4 16565149 16565582 (174707481) C MSTB1 LTR/MaLR (0) 432 1 244

1257 16.7 1.2 7.4 chr4 16565590 16565847 (174707216) C MSTB-int LTR/MaLR (7) 1644 1403 244

3213 22.0 1.7 0.3 chr4 16565845 16566505 (174706558) C MSTB-int LTR/MaLR (981) 670 1 244

523 17.2 2.1 0.0 chr4 16566510 16566602 (174706461) C MSTB1 LTR/MaLR (335) 97 3 245

2464 19.1 2.9 0.0 chr4 16566759 16567172 (174705891) C MSTB LTR/MaLR (0) 426 1 244

Celera ortholog (5 kb upstream and downstream of the identified ortholog)

1078 14.6 1.1 3.3 gi|89161206:17404459-17416251 2 186 (11607) + AluJb SINE/Alu 129 309 (3) 1

271 19.4 8.3 0.0 gi|89161206:17404459-17416251 1528 1599 (10194) + L1MC LINE/L1 5713 5790 (2092) 2

684 25.6 3.6 1.5 gi|89161206:17404459-17416251 1612 1805 (9988) + LTR79 LTR/ERVL 20 217 (310) 3

357 26.1 18.9 0.5 gi|89161206:17404459-17416251 1837 2032 (9761) + LTR79 LTR/ERVL 296 527 (0) 3

224 33.0 5.5 0.0 gi|89161206:17404459-17416251 2859 2949 (8844) C MIR3 SINE/MIR (46) 162 67 4

205 22.7 0.0 0.0 gi|89161206:17404459-17416251 3258 3301 (8492) + L2c LINE/L2 3340 3383 (4) 5

27 72.9 0.0 0.0 gi|89161206:17404459-17416251 3308 3355 (8438) + AT_rich Low_complexity 1 48 (0) 6

27 89.9 0.0 0.0 gi|89161206:17404459-17416251 3398 3466 (8327) + AT_rich Low_complexity 1 69 (0) 7

183 33.8 8.4 0.0 gi|89161206:17404459-17416251 3512 3582 (8211) C MIRb SINE/MIR (29) 239 163 8

454 33.6 4.8 4.8 gi|89161206:17404459-17416251 3633 3863 (7930) C MIR SINE/MIR (31) 231 1 9

394 13.4 10.4 0.0 gi|89161206:17404459-17416251 4738 4804 (6989) C MLT1C LTR/ERVL-MaLR (0) 467 394 10

**29228 0.6 0.0 0.0 gi|89161206:17404459-17416251 4952 10979 (814) + L1HS LINE/L1 126 6154 (1) 11 (R)**

2534 15.3 2.2 0.0 gi|89161206:17404459-17416251 10980 11385 (408) C MLT1C LTR/ERVL-MaLR (52) 415 1 10

2450 8.3 0.0 0.0 gi|89161206:17404459-17416251 11417 11717 (76) C AluY SINE/Alu (9) 302 2 12

359 14.3 0.0 0.0 gi|89161206:17404459-17416251 11745 11793 (0) + AluYk11 SINE/Alu 1 49 (263) 13

___________________________________________________________________________________________________________________________

L1HS_4_23c (Reference human)

234 24.6 1.8 0.0 chr4 45746438 45746494 (145526569) + L2 LINE/L2 3362 3419 (0) 320

283 29.3 4.9 2.0 chr4 45747436 45747615 (145525448) + MIRb SINE/MIR 30 262 (0) 321

21 0.0 0.0 0.0 chr4 45748531 45748551 (145524512) + AT_rich Low_complexity 1 21 (0) 322

792 32.0 5.7 6.6 chr4 45748621 45749132 (145523931) + MLT1J LTR/MaLR 5 511 (0) 323

221 31.9 5.8 0.0 chr4 45749760 45749828 (145523235) + MIR SINE/MIR 49 121 (141) 324

617 27.6 16.2 1.7 chr4 45749923 45750268 (145522795) C L1M5 LINE/L1 (3282) 2864 2469 325

**8995 0.1 0.0 0.0 chr4 45750761 45751768 (145521295) C L1HS LINE/L1 (1132) 5014 4007 326 (R2)**

**7982 0.3 0.0 0.1 chr4 45751763 45752952 (145520111) + L1HS LINE/L1 5028 6216 (0) 326 (R1)**

680 37.3 5.6 2.6 chr4 45752953 45753508 (145519555) + L2 LINE/L2 2851 3416 (3) 327

354 15.2 0.0 0.0 chr4 45753769 45753827 (145519236) C L1M1 LINE/L1 (705) 5600 5542 328

1006 30.3 8.6 6.9 chr4 45755493 45756167 (145516896) C L1ME3B LINE/L1 (9) 6231 5552 329

1743 13.7 0.0 8.2 chr4 45756168 45756486 (145516577) C AluSx SINE/Alu (19) 293 1 330

1006 29.4 9.7 5.2 chr4 45756487 45756810 (145516253) C L1ME3B LINE/L1 (689) 5551 5205 329

240 3.5 0.0 0.0 chr4 45757028 45757056 (145516007) + (TG)n Simple_repeat 2 30 (0) 331

215 29.9 0.0 5.4 chr4 45757648 45757739 (145515324) + MIRb SINE/MIR 112 198 (70) 332

Ortholog in Celera

244 22.8 1.8 0.0 gi|89161206:46501288-46511292 625 681 (9324) + L2a LINE/L2 3369 3426 (0) 1

283 27.7 5.7 1.6 gi|89161206:46501288-46511292 1615 1802 (8203) + MIRb SINE/MIR 16 262 (0) 2

21 52.4 0.0 0.0 gi|89161206:46501288-46511292 2718 2738 (7267) + AT_rich Low_complexity 1 21 (0) 3

644 32.2 5.9 6.7 gi|89161206:46501288-46511292 2808 3319 (6686) + MLT1J LTR/ERVL-MaLR 5 512 (0) 4

218 31.9 5.8 0.0 gi|89161206:46501288-46511292 3947 4015 (5990) + MIR SINE/MIR 49 121 (141) 5

613 27.2 14.8 1.4 gi|89161206:46501288-46511292 4110 4459 (5546) C L1M5 LINE/L1 (3282) 2864 2469 6

**454 4.2 1.2 0.0 gi|89161206:46501288-46511292 4940 5004 (5001) C L1P1 LINE/L1 (1116) 5030 4962 7 (R12)**

659 37.6 5.4 1.9 gi|89161206:46501288-46511292 5006 5501 (4504) + L2a LINE/L2 2911 3423 (3) 8

354 15.2 0.0 0.0 gi|89161206:46501288-46511292 5762 5820 (4185) C L1M1 LINE/L1 (702) 5600 5542 9

967 29.9 8.2 5.6 gi|89161206:46501288-46511292 7486 8160 (1845) C L1ME3B LINE/L1 (11) 6231 5550 10

1880 12.5 0.0 8.9 gi|89161206:46501288-46511292 8161 8479 (1526) C AluSx1 SINE/Alu (19) 293 1 11

967 28.9 9.6 4.6 gi|89161206:46501288-46511292 8480 8803 (1202) C L1ME3B LINE/L1 (693) 5549 5205 10

240 3.5 0.0 0.0 gi|89161206:46501288-46511292 9021 9049 (956) + (TG)n Simple_repeat 2 30 (0) 12

215 30.0 0.0 5.8 gi|89161206:46501288-46511292 9641 9732 (273) + MIRb SINE/MIR 112 198 (70) 13

____________________________________________________________________________

L1HS_7_15c (Reference Human genome)

667 17.7 7.1 0.0 chr7 31486660 31486800 (127334624) + ORSL DNA/Tip100 1 151 (124) 9

716 22.2 0.0 0.0 chr7 31486808 31486942 (127334482) C AluJb SINE/Alu (176) 136 2 10

785 13.6 6.8 0.0 chr7 31486946 31487077 (127334347) + ORSL DNA/Tip100 135 275 (0) 9

404 25.8 10.4 0.0 chr7 31487453 31487635 (127333789) + MIR SINE/MIR 20 239 (29) 11

25 0.0 0.0 0.0 chr7 31487681 31487705 (127333719) + AT_rich Low_complexity 1 25 (0) 12

1688 20.4 3.8 2.8 chr7 31488292 31488684 (127332740) + MER31A LTR/ERV1 89 485 (0) 13

227 34.5 15.7 2.0 chr7 31488790 31489088 (127332336) C L2 LINE/L2 (799) 2620 2281 14

221 22.6 0.0 1.9 chr7 31489665 31489718 (127331706) + MIRb SINE/MIR 116 168 (100) 15

**214 34.8 0.0 0.0 chr7 31490140 31490208 (127331216) + L2 LINE/L2 3349 3417 (2) 16 (R1)**

**583 12.6 1.1 0.0 chr7 31490218 31490304 (127331120) + L1PB1 LINE/L1 5613 5700 (451) 17**

**8236 0.4 0.0 0.0 chr7 31490305 31491778 (127329646) C L1HS LINE/L1 (0) 6196 4723 18 (R2)**

3182 9.7 1.1 1.3 chr7 31491779 31492240 (127329184) + L1PB1 LINE/L1 5687 6147 (4) 17

218 22.2 2.2 0.0 chr7 31492267 31492311 (127329113) + L2 LINE/L2 3316 3361 (17) 19

339 31.4 7.9 0.7 chr7 31492529 31492696 (127328728) C MIR SINE/MIR (80) 186 1 20

363 29.4 3.7 3.9 chr7 31492823 31493014 (127328410) + MIR SINE/MIR 20 210 (58) 21

21 3.6 0.0 0.0 chr7 31493078 31493105 (127328319) + AT_rich Low_complexity 1 28 (0) 22

262 26.8 5.3 3.0 chr7 31493785 31493915 (127327509) + L2 LINE/L2 2701 2834 (585) 23

226 31.5 16.1 0.0 chr7 31494113 31494242 (127327182) + MIRb SINE/MIR 3 153 (115) 24

797 17.1 2.9 2.3 chr7 31494262 31494435 (127326989) + MER96B DNA/MER1_type? 5 179 (238) 25

569 24.0 15.0 3.4 chr7 31494511 31494777 (127326647) + MER5C DNA/MER1_type 27 324 (0) 26

686 32.2 9.4 2.8 chr7 31495095 31495585 (127325839) C L2 LINE/L2 (0) 3419 2889 27

2211 12.5 0.0 0.0 chr7 31495714 31496018 (127325406) C AluSx SINE/Alu (7) 305 1 28

Ortholog in Celera

650 26.1 5.7 8.6 gi|89161211:31507470-31517763 714 1011 (9283) + MLT1B LTR/ERVL-MaLR 34 323 (67) 1

287 24.8 8.6 0.0 gi|89161211:31507470-31517763 1213 1329 (8965) + MIRc SINE/MIR 25 151 (117) 2

660 17.7 7.1 0.0 gi|89161211:31507470-31517763 1580 1720 (8574) + ORSL DNA/hAT-Tip100 1 151 (124) 3

734 22.1 0.0 0.0 gi|89161211:31507470-31517763 1728 1863 (8431) C AluJb SINE/Alu (176) 136 1 4

779 13.6 6.8 0.0 gi|89161211:31507470-31517763 1866 1997 (8297) + ORSL DNA/hAT-Tip100 135 275 (0) 5

395 25.1 9.0 0.0 gi|89161211:31507470-31517763 2373 2505 (7789) + MIR SINE/MIR 20 177 (91) 6

25 40.0 0.0 0.0 gi|89161211:31507470-31517763 2599 2623 (7671) + AT_rich Low_complexity 1 25 (0) 7

25 64.0 0.0 0.0 gi|89161211:31507470-31517763 2601 2625 (7669) + AT_rich Low_complexity 1 25 (0) 8

1729 20.8 3.3 2.3 gi|89161211:31507470-31517763 3212 3604 (6690) + MER31A LTR/ERV1 89 485 (0) 9

323 33.5 1.2 1.8 gi|89161211:31507470-31517763 3845 4008 (6286) C L2 LINE/L2 (976) 2443 2281 10

294 29.1 0.0 1.2 gi|89161211:31507470-31517763 4585 4671 (5623) + MIRc SINE/MIR 113 198 (70) 11

**2907 0.3 0.0 0.0 gi|89161211:31507470-31517763 5022 5343 (4951) C L1P1 LINE/L1 (1102) 5044 4723 12 (R12)**

3181 9.7 1.1 1.3 gi|89161211:31507470-31517763 5344 5805 (4489) + L1PB1 LINE/L1 5687 6147 (4) 13

222 20.0 2.2 0.0 gi|89161211:31507470-31517763 5832 5876 (4418) + L2b LINE/L2 3313 3358 (17) 14

333 31.4 7.9 0.6 gi|89161211:31507470-31517763 6094 6261 (4033) C MIR SINE/MIR (82) 186 1 15

547 32.0 1.1 0.0 gi|89161211:31507470-31517763 6406 6580 (3714) + MIRc SINE/MIR 41 217 (51) 16

21 78.6 0.0 0.0 gi|89161211:31507470-31517763 6643 6670 (3624) + AT_rich Low_complexity 1 28 (0) 17

260 26.8 5.3 3.0 gi|89161211:31507470-31517763 7350 7480 (2814) + L2 LINE/L2 2701 2834 (585) 18

254 28.1 16.4 0.0 gi|89161211:31507470-31517763 7680 7807 (2487) + MIRc SINE/MIR 2 150 (118) 19

800 17.1 2.9 2.3 gi|89161211:31507470-31517763 7827 8000 (2294) + MER96B DNA/hAT 5 179 (238) 20

614 25.1 9.0 1.4 gi|89161211:31507470-31517763 8076 8342 (1952) + MER5C DNA/hAT-Charlie 27 313 (0) 21

504 29.5 9.7 4.0 gi|89161211:31507470-31517763 8660 9150 (1144) C L2a LINE/L2 (0) 3426 2889 22

2226 12.5 0.0 0.0 gi|89161211:31507470-31517763 9279 9583 (711) C AluSx SINE/Alu (7) 305 1 23

231 23.5 0.0 0.0 gi|89161211:31507470-31517763 10055 10105 (189) + MLT1K LTR/ERVL-MaLR 528 578 (17) 24

______________________________________________________________________________

L1HS_11_30c (Reference human genome) 0CCUPIED

679 33.5 10.4 5.3 chr11 41041361 41042133 (93410251) C L2 LINE/L2 (18) 3401 2562 525

306 20.6 0.0 1.4 chr11 41042697 41042765 (93409619) + MSTA LTR/MaLR 2 69 (359) 526

21581 14.9 2.1 5.8 chr11 41042766 41046236 (93406148) + L1PA8 LINE/L1 22 3376 (2955) 527

2085 12.9 0.6 2.5 chr11 41046237 41046553 (93405831) + AluSx SINE/Alu 1 311 (1) 528

21581 10.1 1.3 1.4 chr11 41046554 41049554 (93402830) + L1PA8 LINE/L1 3377 6356 (1) 527

5051 13.3 18.2 0.0 chr11 41049576 41050497 (93401887) + Tigger1 DNA/MER2_type 1149 2238 (180) 529

1190 18.9 8.1 6.2 chr11 41050507 41050827 (93401557) + THE1D LTR/MaLR 55 381 (0) 526

1845 19.8 5.4 4.4 chr11 41051235 41051662 (93400722) C MSTB1 LTR/MaLR (0) 432 1 530

243 6.1 0.0 0.0 chr11 41053136 41053168 (93399216) + (T)n Simple_repeat 1 33 (0) 531

**742 0.0 0.0 0.0 chr11 41053176 41053262 (93399122) C L1HS LINE/L1 (0) 6155 6069 532 (L1HS_11_30c)**

2148 31.8 8.4 5.8 chr11 41053605 41054440 (93397944) + L2 LINE/L2 1669 2529 (890) 533

1487 17.9 18.7 4.3 chr11 41054441 41054814 (93397570) C MSTA LTR/MaLR (0) 428 1 534

3201 31.6 7.0 3.7 chr11 41054815 41056492 (93395892) C MLT-int LTR/MaLR (0) 1735 2 535

1828 17.6 12.5 1.0 chr11 41056495 41056878 (93395506) C MSTA LTR/MaLR (0) 428 1 536

2148 31.9 7.6 4.5 chr11 41056879 41057288 (93395096) + L2 LINE/L2 2530 2955 (464) 533

1424 13.6 3.6 0.9 chr11 41057800 41058022 (93394362) + MER30 DNA/MER1_type 2 230 (0) 537

2356 9.7 0.0 0.3 chr11 41058469 41058778 (93393606) C AluSp SINE/Alu (4) 309 1 538

222 20.4 0.0 0.0 chr11 41059841 41059884 (93392500) + (CA)n Simple_repeat 2 45 (0) 539

23 0.0 0.0 0.0 chr11 41059899 41059921 (93392463) + AT_rich Low_complexity 1 23 (0) 540

189 0.0 0.0 0.0 chr11 41060554 41060574 (93391810) + (T)n Simple_repeat 1 21 (0) 541

519 24.0 11.7 6.1 chr11 41060604 41060834 (93391550) C MIR SINE/MIR (15) 247 4 542

21 0.0 0.0 0.0 chr11 41061793 41061813 (93390571) + AT_rich Low_complexity 1 21 (0) 543

666 27.5 2.0 3.0 chr11 41062345 41062543 (93389841) + MIRb SINE/MIR 14 210 (58) 544

21 0.0 0.0 0.0 chr11 41062672 41062692 (93389692) + AT_rich Low_complexity 1 21 (0) 545

2288 10.6 0.0 0.6 chr11 41062693 41063006 (93389378) + AluSx SINE/Alu 1 312 (0) 546

485 28.7 9.4 1.3 chr11 41063568 41063726 (93388658) + MIRb SINE/MIR 68 239 (29) 547

Orhtholog in Celera

6068 10.1 1.6 0.1 gi|89161188:41245026-41255090 1 1393 (8672) + L1PA8 LINE/L1 4759 6171 (1) 1

763 14.9 10.6 0.0 gi|89161188:41245026-41255090 1415 1555 (8510) + Tigger1 DNA/TcMar-Tigger 1149 1304 (1114) 2 *

4322 13.9 4.4 0.0 gi|89161188:41245026-41255090 1534 2244 (7821) + Tigger1 DNA/TcMar-Tigger 1317 2058 (360) 3

600 11.8 4.3 0.0 gi|89161188:41245026-41255090 2242 2334 (7731) + Tigger1 DNA/TcMar-Tigger 2140 2236 (182) 4 *

1171 19.6 8.1 6.1 gi|89161188:41245026-41255090 2346 2666 (7399) + THE1D LTR/ERVL-MaLR 55 381 (0) 5

511 28.3 0.6 2.9 gi|89161188:41245026-41255090 2884 3061 (7004) + Tigger9a DNA/TcMar-Tigger 558 731 (1) 6

1847 19.6 5.4 4.4 gi|89161188:41245026-41255090 3074 3501 (6564) C MSTB1 LTR/ERVL-MaLR (0) 432 1 7

294 8.7 0.0 0.0 gi|89161188:41245026-41255090 4975 5020 (5045) + (T)n Simple_repeat 1 46 (0) 8

**722 1.1 0.0 0.0 gi|89161188:41245026-41255090 5028 5114 (4951) C L1HS LINE/L1 (0) 6155 6069 9 (OCCUPIED)**

1136 31.4 6.5 4.7 gi|89161188:41245026-41255090 5457 6291 (3774) + L2 LINE/L2 1669 2523 (896) 10

1151 21.6 6.7 4.7 gi|89161188:41245026-41255090 6294 6667 (3398) C MSTA LTR/ERVL-MaLR (0) 381 1 11

7039 17.8 1.1 0.9 gi|89161188:41245026-41255090 6668 8194 (1871) C MSTA-int LTR/ERVL-MaLR (0) 1580 96 11

1055 13.0 0.0 0.0 gi|89161188:41245026-41255090 8192 8345 (1720) C MSTA-int LTR/ERVL-MaLR (1424) 156 3 11 *

1786 17.6 12.5 0.9 gi|89161188:41245026-41255090 8348 8731 (1334) C MSTA LTR/ERVL-MaLR (0) 428 1 11

912 33.1 4.7 1.9 gi|89161188:41245026-41255090 8739 9141 (924) + L2 LINE/L2 2545 2958 (461) 10

290 35.3 7.6 1.5 gi|89161188:41245026-41255090 9372 9621 (444) + LTR85a LTR/Gypsy? 289 553 (149) 12

1417 13.6 3.6 0.9 gi|89161188:41245026-41255090 9653 9875 (190) + MER30 DNA/hAT-Charlie 2 230 (0) 13

______________________________________________________________________________

L1HS_12_41c (Reference human genome) C_INTER_RMD

7775 23.0 8.0 3.2 chr12 86656449 86657589 (45691945) + L1MC3 LINE/L1 6625 7777 (8) 134

1751 16.4 2.5 0.0 chr12 86660380 86660665 (45688869) C AluJb SINE/Alu (19) 293 1 138

1095 31.3 6.4 3.6 chr12 86662548 86663015 (45686519) + MLT1K LTR/MaLR 1 481 (110) 139

714 28.8 10.0 0.5 chr12 86663296 86663515 (45686019) + MIR SINE/MIR 10 250 (12) 140

37 5.2 0.0 0.0 chr12 86663528 86663585 (45685949) + AT_rich Low_complexity 1 58 (0) 141

201 37.9 2.9 1.0 chr12 86663736 86663839 (45685695) C L2 LINE/L2 (17) 3402 3297 142

184 30.4 3.6 0.0 chr12 86663922 86663977 (45685557) C L2 LINE/L2 (211) 3167 3110 142

27 4.9 0.0 0.0 chr12 86664583 86664623 (45684911) + AT_rich Low_complexity 1 41 (0) 143

21 0.0 0.0 0.0 chr12 86665451 86665471 (45684063) + AT_rich Low_complexity 1 21 (0) 144

**9646 0.2 0.0 0.0 chr12 86665472 86667330 (45682204) C L1HS LINE/L1 (3) 6152 4294 1 (L1HS_12_41c) (R2)**

**6299 0.7 0.0 0.0 chr12 86667329 86668039 (45681495) + L1HS LINE/L1 3568 4278 (1868) 145**

**193 2.9 5.9 0.0 chr12 86668344 86668377 (45681157) + (TTA)n Simple_repeat 1 36 (0) 146**

**8849 2.9 0.0 0.0 chr12 86668379 86670176 (45679358) C L1PA3 LINE/L1 (18) 6137 4340 147 (R1)**

3494 2.2 0.2 0.0 chr12 86670177 86670585 (45678949) + L1PA3 LINE/L1 3902 4311 (1835) 147

216 33.9 3.2 8.1 chr12 86671399 86671584 (45677950) C L2 LINE/L2 (740) 2679 2503 148

1565 27.5 9.1 3.2 chr12 86671741 86672682 (45676852) + L1MD LINE/L1 3033 4065 (2081) 149

429 33.9 10.4 0.0 chr12 86672689 86672918 (45676616) C L2 LINE/L2 (1074) 2345 2092 148

2030 13.4 0.3 1.9 chr12 86672923 86673233 (45676301) + AluSq SINE/Alu 3 308 (5) 150

709 15.0 1.6 0.8 chr12 86673684 86673811 (45675723) + L1MB3 LINE/L1 6054 6182 (1) 151

423 22.3 1.8 1.8 chr12 86673815 86673928 (45675606) + Charlie2 DNA/MER1_type 2707 2820 (42) 152

2348 7.0 0.0 2.9 chr12 86674354 86674663 (45674871) + AluY SINE/Alu 1 301 (10) 153

Ortholog in Celera

1005 13.2 0.0 0.0 gi|89161189:87807275-87819039 10 145 (11620) C AluJb SINE/Alu (176) 136 1 1

1075 31.5 6.4 3.1 gi|89161189:87807275-87819039 2028 2495 (9270) + MLT1K LTR/ERVL-MaLR 1 483 (112) 2

698 28.8 10.0 0.4 gi|89161189:87807275-87819039 2776 2995 (8770) + MIR SINE/MIR 10 250 (12) 3

37 79.3 0.0 0.0 gi|89161189:87807275-87819039 3008 3065 (8700) + AT_rich Low_complexity 1 58 (0) 4

304 38.4 7.4 3.2 gi|89161189:87807275-87819039 3216 3457 (8308) C L2b LINE/L2 (17) 3358 3107 5

180 0.0 0.0 0.0 gi|89161189:87807275-87819039 4065 4084 (7681) + (T)n Simple_repeat 1 20 (0) 6

**8849 2.4 0.0 0.0 gi|89161189:87807275-87819039 4952 6764 (5001) C L1HS LINE/L1 (3) 6152 4340 7 (R12)**

3494 2.2 0.2 0.0 gi|89161189:87807275-87819039 6765 7173 (4592) + L1HS LINE/L1 3902 4311 (1835) 7

213 34.1 3.2 8.5 gi|89161189:87807275-87819039 7987 8172 (3593) C L2b LINE/L2 (740) 2679 2503 5

982 26.3 8.4 5.0 gi|89161189:87807275-87819039 8329 9137 (2628) + L1M5 LINE/L1 3033 3883 (2263) 8

238 25.7 8.0 0.0 gi|89161189:87807275-87819039 9158 9270 (2495) + L1M5 LINE/L1 3944 4065 (2081) 8

301 34.4 9.6 0.0 gi|89161189:87807275-87819039 9277 9506 (2259) C L2b LINE/L2 (1074) 2345 2092 5

2168 12.5 0.3 2.0 gi|89161189:87807275-87819039 9511 9821 (1944) + AluSq2 SINE/Alu 3 308 (4) 9

708 15.0 1.6 0.8 gi|89161189:87807275-87819039 10272 10399 (1366) + L1MB3 LINE/L1 6054 6182 (1) 10

422 22.3 1.8 1.8 gi|89161189:87807275-87819039 10403 10516 (1249) + Charlie2b DNA/hAT-Charlie 2627 2740 (42) 11

2466 6.8 0.0 3.0 gi|89161189:87807275-87819039 10942 11251 (514) + AluY SINE/Alu 1 301 (10) 12

469 13.8 11.7 0.0 gi|89161189:87807275-87819039 11662 11755 (10) C ORSL DNA/hAT-Tip100 (13) 262 158 13

________________________________________________________________________________________________

L1HS_18_2c (Reference human genome) M_DISRUPTED

5028 15.3 2.3 5.0 chr18 823890 824715 (75292438) C MER49 LTR/ERV1 (36) 887 79 603

2203 10.6 0.7 0.0 chr18 824716 825007 (75292146) + AluSq SINE/Alu 6 299 (14) 605

5028 15.3 2.3 5.0 chr18 825008 825051 (75292102) C MER49 LTR/ERV1 (845) 78 35 603

347 13.9 9.7 0.0 chr18 825053 825124 (75292029) C L1MB2 LINE/L1 (213) 5958 5880 606

1670 17.7 3.8 0.0 chr18 825140 825427 (75291726) C AluJb SINE/Alu (13) 299 1 607

194 27.9 0.0 0.0 chr18 825428 825470 (75291683) + MER5B DNA/MER1_type 136 178 (0) 608

1322 3.6 0.0 1.2 chr18 825474 825639 (75291514) + L1PA2 LINE/L1 5992 6155 (0) 609

2039 13.2 3.1 0.0 chr18 825655 825942 (75291211) + AluSx SINE/Alu 1 297 (15) 610

220 30.1 0.0 1.4 chr18 825944 826017 (75291136) C MER5B DNA/MER1_type (32) 146 74 611

2026 14.3 1.0 0.0 chr18 826409 826715 (75290438) C AluSq SINE/Alu (3) 310 1 612

**4690 1.7 0.0 1.9 chr18 826734 827111 (75290042) C L1HS LINE/L1 (1) 6154 5785 613 (R1) (L1HS_18_2c)**

**2903 0.7 0.0 0.0 chr18 827112 827418 (75289735) C AluYb8 SINE/Alu (11) 307 1 614 (D)**

**4690 1.7 0.0 1.9 chr18 827419 827581 (75289572) C L1HS LINE/L1 (371) 5784 5623 613 (R2)**

817 1.1 0.0 0.0 chr18 827582 827670 (75289483) + L1P2 LINE/L1 5523 5611 (544) 615

391 30.0 4.6 0.0 chr18 827787 827916 (75289237) + MIR SINE/MIR 110 245 (17) 616

1979 12.4 3.9 2.6 chr18 828425 828731 (75288422) C AluSx SINE/Alu (1) 311 1 617

2216 10.6 7.2 2.6 chr18 828988 829335 (75287818) C THE1B LTR/MaLR (0) 364 1 618

1088 20.0 0.4 6.0 chr18 830458 830691 (75286462) + AluJo SINE/Alu 1 221 (91) 619

506 19.8 0.0 0.0 chr18 830694 830779 (75286374) C AluJ/FLAM SINE/Alu (225) 87 2 620

444 29.7 8.7 0.9 chr18 830927 831157 (75285996) C MIR SINE/MIR (12) 250 2 621

460 29.4 3.2 6.4 chr18 831384 831441 (75285712) + L2 LINE/L2 2930 2979 (440) 622

3506 6.5 4.4 10.0 chr18 831442 831609 (75285544) + LTR2C LTR/ERV1 4 172 (329) 623

261 0.0 0.0 0.0 chr18 831610 831638 (75285515) + (TAA)n Simple_repeat 2 30 (0) 624

Ortholog in Celera

1381 16.9 1.1 3.9 gi|89161196:706482-716803 84 348 (9974) C MER44B DNA/TcMar-Tigger (0) 550 293 1

1169 19.8 2.2 2.2 gi|89161196:706482-716803 747 968 (9354) C MER44B DNA/TcMar-Tigger (250) 300 79 1

2343 9.4 0.0 0.7 gi|89161196:706482-716803 970 1268 (9054) C AluSx1 SINE/Alu (15) 297 1 2

299 21.7 0.0 0.0 gi|89161196:706482-716803 1269 1328 (8994) + L1MD3 LINE/L1 5894 5953 (2042) 3

423 29.9 11.1 2.8 gi|89161196:706482-716803 1373 1724 (8598) C L2c LINE/L2 (896) 2523 2173 4

5021 15.4 2.3 5.1 gi|89161196:706482-716803 1745 1784 (8538) C MER49 LTR/ERV1 (2) 921 882 5

1847 15.1 2.6 2.2 gi|89161196:706482-716803 1785 2095 (8227) + AluJo SINE/Alu 1 312 (0) 6

5021 15.4 2.3 5.1 gi|89161196:706482-716803 2096 2920 (7402) C MER49 LTR/ERV1 (42) 881 71 5

2210 10.6 0.7 0.0 gi|89161196:706482-716803 2921 3212 (7110) + AluSq2 SINE/Alu 6 299 (13) 7

5021 15.4 2.3 5.1 gi|89161196:706482-716803 3213 3256 (7066) C MER49 LTR/ERV1 (853) 70 35 5

342 13.9 9.7 0.0 gi|89161196:706482-716803 3258 3329 (6993) C L1MB2 LINE/L1 (213) 5958 5880 8

1760 17.2 3.8 0.0 gi|89161196:706482-716803 3345 3634 (6688) C AluJb SINE/Alu (11) 301 1 9

222 25.6 0.0 0.0 gi|89161196:706482-716803 3635 3677 (6645) C MER5B DNA/hAT-Charlie (135) 43 1 10

1320 3.6 0.0 1.2 gi|89161196:706482-716803 3681 3846 (6476) + L1PA2 LINE/L1 5992 6155 (0) 11

2037 13.2 3.1 0.0 gi|89161196:706482-716803 3862 4149 (6173) + AluSz SINE/Alu 1 297 (15) 12

228 30.1 0.0 1.4 gi|89161196:706482-716803 4151 4224 (6098) C MER5B DNA/hAT-Charlie (32) 146 74 13

199 33.1 4.5 4.5 gi|89161196:706482-716803 4335 4467 (5855) C L2 LINE/L2 (1327) 2092 1960 14

2185 13.6 0.0 0.0 gi|89161196:706482-716803 4616 4925 (5397) C AluSq2 SINE/Alu (2) 310 1 15

**4770 1.5 0.0 0.0 gi|89161196:706482-716803 4951 5483 (4839) C L1HS LINE/L1 (0) 6155 5623 16 (R)**

817 1.1 0.0 0.0 gi|89161196:706482-716803 5484 5572 (4750) + L1HS LINE/L1 5523 5611 (544) 16

189 37.5 0.0 1.8 gi|89161196:706482-716803 5620 5676 (4646) + MIR SINE/MIR 6 61 (201) 17

437 30.0 4.6 0.0 gi|89161196:706482-716803 5689 5818 (4504) + MIR SINE/MIR 110 245 (17) 17

2121 11.4 4.0 0.3 gi|89161196:706482-716803 6327 6625 (3697) C AluSz SINE/Alu (2) 310 1 18

2161 11.2 7.2 2.5 gi|89161196:706482-716803 6882 7229 (3093) C THE1B LTR/ERVL-MaLR (0) 364 1 19

191 31.6 5.1 0.8 gi|89161196:706482-716803 7237 7354 (2968) + MIR3 SINE/MIR 57 179 (29) 20

1078 18.0 0.4 11.3 gi|89161196:706482-716803 8352 8596 (1726) + AluJo SINE/Alu 1 221 (91) 21

554 19.5 0.0 0.0 gi|89161196:706482-716803 8599 8685 (1637) C AluJo SINE/Alu (225) 87 1 22

422 29.7 8.7 0.8 gi|89161196:706482-716803 8832 9062 (1260) C MIR SINE/MIR (12) 250 2 23

471 31.9 1.6 6.2 gi|89161196:706482-716803 9265 9346 (976) + L2b LINE/L2 2903 2979 (440) 24

3525 5.9 4.4 10.6 gi|89161196:706482-716803 9347 9514 (808) + LTR2C LTR/ERV1 4 172 (329) 25

207 0.0 0.0 0.0 gi|89161196:706482-716803 9515 9537 (785) + (TAA)n Simple_repeat 2 24 (0) 26

3525 5.9 4.4 10.6 gi|89161196:706482-716803 9538 9896 (426) + LTR2C LTR/ERV1 173 500 (1) 25

471 33.0 3.8 5.1 gi|89161196:706482-716803 9897 10174 (148) + L2b LINE/L2 2977 3276 (111) 24

373 21.3 2.7 0.0 gi|89161196:706482-716803 10228 10302 (20) + MER91A DNA/hAT-Tip100 19 95 (101) 27

________________________________________________________________________________________________

L1HS_18_3c (Reference human genome) (M_DISRUPTED)

347 13.9 9.7 0.0 chr18 825053 825124 (75292029) C L1MB2 LINE/L1 (213) 5958 5880 606

1670 17.7 3.8 0.0 chr18 825140 825427 (75291726) C AluJb SINE/Alu (13) 299 1 607

194 27.9 0.0 0.0 chr18 825428 825470 (75291683) + MER5B DNA/MER1_type 136 178 (0) 608

1322 3.6 0.0 1.2 chr18 825474 825639 (75291514) + L1PA2 LINE/L1 5992 6155 (0) 609

2039 13.2 3.1 0.0 chr18 825655 825942 (75291211) + AluSx SINE/Alu 1 297 (15) 610

220 30.1 0.0 1.4 chr18 825944 826017 (75291136) C MER5B DNA/MER1_type (32) 146 74 611

2026 14.3 1.0 0.0 chr18 826409 826715 (75290438) C AluSq SINE/Alu (3) 310 1 612

**4690 1.7 0.0 1.9 chr18 826734 827111 (75290042) C L1HS LINE/L1 (1) 6154 5785 613 (R1)**

**2903 0.7 0.0 0.0 chr18 827112 827418 (75289735) C AluYb8 SINE/Alu (11) 307 1 614 (D)**

**4690 1.7 0.0 1.9 chr18 827419 827581 (75289572) C L1HS LINE/L1 (371) 5784 5623 613 (R2) (L1HS_18_3c)**

817 1.1 0.0 0.0 chr18 827582 827670 (75289483) + L1P2 LINE/L1 5523 5611 (544) 615

391 30.0 4.6 0.0 chr18 827787 827916 (75289237) + MIR SINE/MIR 110 245 (17) 616

1979 12.4 3.9 2.6 chr18 828425 828731 (75288422) C AluSx SINE/Alu (1) 311 1 617

2216 10.6 7.2 2.6 chr18 828988 829335 (75287818) C THE1B LTR/MaLR (0) 364 1 618

1088 20.0 0.4 6.0 chr18 830458 830691 (75286462) + AluJo SINE/Alu 1 221 (91) 619

506 19.8 0.0 0.0 chr18 830694 830779 (75286374) C AluJ/FLAM SINE/Alu (225) 87 2 620

444 29.7 8.7 0.9 chr18 830927 831157 (75285996) C MIR SINE/MIR (12) 250 2 621

Ortholog in Celera

342 13.9 9.7 0.0 gi|89161196:706482-716803 3258 3329 (6993) C L1MB2 LINE/L1 (213) 5958 5880 8

1760 17.2 3.8 0.0 gi|89161196:706482-716803 3345 3634 (6688) C AluJb SINE/Alu (11) 301 1 9

222 25.6 0.0 0.0 gi|89161196:706482-716803 3635 3677 (6645) C MER5B DNA/hAT-Charlie (135) 43 1 10

1320 3.6 0.0 1.2 gi|89161196:706482-716803 3681 3846 (6476) + L1PA2 LINE/L1 5992 6155 (0) 11

2037 13.2 3.1 0.0 gi|89161196:706482-716803 3862 4149 (6173) + AluSz SINE/Alu 1 297 (15) 12

228 30.1 0.0 1.4 gi|89161196:706482-716803 4151 4224 (6098) C MER5B DNA/hAT-Charlie (32) 146 74 13

199 33.1 4.5 4.5 gi|89161196:706482-716803 4335 4467 (5855) C L2 LINE/L2 (1327) 2092 1960 14

2185 13.6 0.0 0.0 gi|89161196:706482-716803 4616 4925 (5397) C AluSq2 SINE/Alu (2) 310 1 15

**4770 1.5 0.0 0.0 gi|89161196:706482-716803 4951 5483 (4839) C L1HS LINE/L1 (0) 6155 5623 16 (R)**

817 1.1 0.0 0.0 gi|89161196:706482-716803 5484 5572 (4750) + L1HS LINE/L1 5523 5611 (544) 16

189 37.5 0.0 1.8 gi|89161196:706482-716803 5620 5676 (4646) + MIR SINE/MIR 6 61 (201) 17

437 30.0 4.6 0.0 gi|89161196:706482-716803 5689 5818 (4504) + MIR SINE/MIR 110 245 (17) 17

2121 11.4 4.0 0.3 gi|89161196:706482-716803 6327 6625 (3697) C AluSz SINE/Alu (2) 310 1 18

2161 11.2 7.2 2.5 gi|89161196:706482-716803 6882 7229 (3093) C THE1B LTR/ERVL-MaLR (0) 364 1 19

191 31.6 5.1 0.8 gi|89161196:706482-716803 7237 7354 (2968) + MIR3 SINE/MIR 57 179 (29) 20

1078 18.0 0.4 11.3 gi|89161196:706482-716803 8352 8596 (1726) + AluJo SINE/Alu 1 221 (91) 21

554 19.5 0.0 0.0 gi|89161196:706482-716803 8599 8685 (1637) C AluJo SINE/Alu (225) 87 1 22

422 29.7 8.7 0.8 gi|89161196:706482-716803 8832 9062 (1260) C MIR SINE/MIR (12) 250 2 23

_________________________________________________________________________________________

L1HS_4_117c (Reference human genome) M_INTER_RMD

41 4.8 0.0 0.0 chr4 157184766 157184827 (34088236) + AT_rich Low_complexity 1 62 (0) 24

1870 18.7 3.0 2.1 chr4 157185187 157185656 (34087407) C MLT2A2 LTR/ERVL (79) 482 1 25

354 26.0 0.0 4.9 chr4 157185770 157185850 (34087213) + LTR16D LTR/ERVL 575 651 (4) 26

1885 15.6 8.4 0.0 chr4 157186035 157186368 (34086695) + MER57A LTR/ERV1 42 403 (0) 27

267 20.0 0.0 0.0 chr4 157187075 157187119 (34085944) C LTR16A1 LTR/ERVL (2) 455 411 28

475 5.6 1.4 1.4 chr4 157187134 157187205 (34085858) + (TTCC)n Simple_repeat 1 72 (0) 29

272 13.7 0.0 1.9 chr4 157187198 157187249 (34085814) + (TTCCC)n Simple_repeat 4 54 (0) 30

465 30.5 16.0 3.1 chr4 157187277 157187601 (34085462) C LTR16A1 LTR/ERVL (90) 367 1 28

21 0.0 0.0 0.0 chr4 157188800 157188820 (34084243) + AT_rich Low_complexity 1 21 (0) 31

**8016 0.4 0.0 0.0 chr4 157188972 157190069 (34082994) C L1HS LINE/L1 (576) 5579 4482 32 (L1HS_4_117c) (R12)**

5179 0.9 0.0 0.0 chr4 157190070 157190650 (34082413) + L1HS LINE/L1 5575 6155 (0) 32

24 3.2 0.0 0.0 chr4 157190651 157190681 (34082382) + AT_rich Low_complexity 1 31 (0) 33

587 10.1 0.0 0.0 chr4 157190730 157190818 (34082245) + L1MA5 LINE/L1 6212 6300 (0) 34

30 2.7 0.0 0.0 chr4 157191792 157191828 (34081235) + AT_rich Low_complexity 1 37 (0) 35

24 5.3 0.0 0.0 chr4 157192119 157192156 (34080907) + AT_rich Low_complexity 1 38 (0) 36

2195 11.8 0.0 0.3 chr4 157192159 157192457 (34080606) + AluSg SINE/Alu 3 300 (10) 37

1332 15.8 6.2 2.2 chr4 157194262 157194533 (34078530) + L1MC3 LINE/L1 7337 7619 (166) 38

758 28.1 12.1 3.5 chr4 157194534 157194880 (34078183) + L2 LINE/L2 3043 3419 (0) 39

232 22.9 4.8 1.2 chr4 157195781 157195864 (34077199) + MER5A DNA/MER1_type 97 183 (6) 40

29 0.0 0.0 0.0 chr4 157196118 157196146 (34076917) + AT_rich Low_complexity 1 29 (0) 41

2030 8.7 6.0 0.0 chr4 157196182 157196447 (34076616) C AluSc SINE/Alu (27) 282 1 42

258 34.4 7.0 3.2 chr4 157196532 157196687 (34076376) C L3b LINE/CR1 (319) 4189 4028 43

L1HS_4_117c Ortholog in Celera

41 79.0 0.0 0.0 gi|89161206:154308367-154317552 760 821 (8365) + AT_rich Low_complexity 1 62 (0) 3

644 34.4 1.4 0.9 gi|89161206:154308367-154317552 932 1151 (8035) + LTR16D2 LTR/ERVL 138 358 (214) 4

1882 18.9 2.7 1.7 gi|89161206:154308367-154317552 1181 1650 (7536) C MLT2A2 LTR/ERVL (79) 482 1 5

462 30.0 9.1 2.4 gi|89161206:154308367-154317552 1651 1844 (7342) + LTR16D2 LTR/ERVL 465 671 (4) 4

1365 14.8 4.0 0.9 gi|89161206:154308367-154317552 2029 2362 (6824) + MER57B2 LTR/ERV1 42 385 (0) 6

275 20.0 0.0 0.0 gi|89161206:154308367-154317552 3069 3113 (6073) C LTR16A1 LTR/ERVL (2) 454 410 7

365 5.8 0.0 1.9 gi|89161206:154308367-154317552 3135 3187 (5999) + (TTCC)n Simple_repeat 1 52 (0) 8

276 17.0 0.0 0.0 gi|89161206:154308367-154317552 3178 3230 (5956) + C-rich Low_complexity 125 177 (0) 9 *

593 30.8 15.1 2.2 gi|89161206:154308367-154317552 3259 3583 (5603) C LTR16A1 LTR/ERVL (90) 366 1 7

22 54.5 0.0 0.0 gi|89161206:154308367-154317552 4780 4801 (4385) + AT_rich Low_complexity 1 22 (0) 10

**1802 0.5 0.5 0.0 gi|89161206:154308367-154317552 4952 5157 (4029) C L1P1 LINE/L1 (576) 5579 5373 11 * (R2)**

**8059 0.3 0.0 0.0 gi|89161206:154308367-154317552 5155 6235 (2951) C L1HS LINE/L1 (593) 5562 4482 12 (R1)**

5176 0.9 0.0 0.0 gi|89161206:154308367-154317552 6236 6816 (2370) + L1HS LINE/L1 5575 6155 (0) 12

25 56.2 0.0 0.0 gi|89161206:154308367-154317552 6817 6848 (2338) + AT_rich Low_complexity 1 32 (0) 13

587 10.1 0.0 0.0 gi|89161206:154308367-154317552 6897 6985 (2201) + L1MA5 LINE/L1 6212 6300 (0) 14

30 59.5 0.0 0.0 gi|89161206:154308367-154317552 7959 7995 (1191) + AT_rich Low_complexity 1 37 (0) 15

24 52.6 0.0 0.0 gi|89161206:154308367-154317552 8286 8323 (863) + AT_rich Low_complexity 1 38 (0) 16

2273 11.7 0.0 0.3 gi|89161206:154308367-154317552 8326 8625 (561) + AluSg SINE/Alu 3 301 (9) 17

__________________________________________________________________________________________________________________________________

L1HS_8_26 Reference human genome M_INTER_RMD

2015 18.3 5.0 0.1 chr8 66463716 66464149 (79810677) + MER21A LTR/ERV1 484 923 (8) 195

910 14.8 5.1 1.1 chr8 66464162 66464339 (79810487) C MER65D LTR/ERV1 (132) 341 157 196

475 22.7 2.1 1.4 chr8 66464393 66464535 (79810291) C MER65D LTR/ERV1 (326) 147 4 196

500 24.6 1.0 13.4 chr8 66464544 66464745 (79810081) C LTR42 LTR/ERVL (235) 260 84 197

2020 11.1 11.4 0.0 chr8 66465154 66465494 (79809332) C MSTA LTR/MaLR (48) 380 1 198

188 28.1 3.7 10.3 chr8 66465680 66465786 (79809040) C MIR SINE/MIR (10) 252 153 199

2113 10.6 2.6 3.2 chr8 66465809 66466119 (79808707) + AluSc SINE/Alu 1 309 (0) 200

1202 1.2 0.1 0.0 chr8 66466274 66466434 (79808392) C L1P2 LINE/L1 (390) 5764 5603 201

**3682 0.2 0.0 0.5 chr8 66466435 66466886 (79807940) + L1HS LINE/L1 5740 6191 (0) 202 (R12)**

1081 20.5 1.8 1.4 chr8 66466915 66467132 (79807694) + MER20 DNA/MER1_type 1 219 (0) 203

353 31.4 1.0 1.0 chr8 66467166 66467268 (79807558) + L2 LINE/L2 3256 3358 (20) 204

240 20.4 0.0 1.8 chr8 66468426 66468480 (79806346) C MLT2B2 LTR/ERVL (0) 556 503 205

264 15.2 1.5 2.9 chr8 66468481 66468548 (79806278) + (TA)n Simple_repeat 2 68 (0) 206

2432 17.6 1.2 7.0 chr8 66468549 66469050 (79805776) C MLT2B2 LTR/ERVL (40) 475 3 205

22 0.0 0.0 0.0 chr8 66469356 66469377 (79805449) + AT_rich Low_complexity 1 22 (0) 207

2045 26.3 12.4 2.4 chr8 66470393 66471369 (79803457) C L2 LINE/L2 (56) 3363 2282 208

1893 18.0 2.4 2.6 chr8 66471427 66471795 (79803031) + Charlie1b DNA/MER1_type 103 480 (43) 209

L1HS_8_26 Ortholog in Celera

2003 17.8 4.3 0.0 gi|89161214:62289528-62298770 2268 2666 (6577) + MER21A LTR/ERVL 508 923 (8) 6

903 15.9 5.1 1.1 gi|89161214:62289528-62298770 2679 2856 (6387) C MER65D LTR/ERV1 (132) 341 157 7

475 22.7 2.1 1.4 gi|89161214:62289528-62298770 2910 3052 (6191) C MER65D LTR/ERV1 (326) 147 4 7

432 23.1 2.8 0.9 gi|89161214:62289528-62298770 3154 3262 (5981) C LTR42 LTR/ERVL (301) 194 84 8

1359 11.8 9.6 1.4 gi|89161214:62289528-62298770 3671 4011 (5232) C MSTA LTR/ERVL-MaLR (48) 380 1 9

185 28.7 1.2 10.7 gi|89161214:62289528-62298770 4197 4278 (4965) C MIR SINE/MIR (10) 252 178 10

2163 10.3 2.6 3.6 gi|89161214:62289528-62298770 4326 4636 (4607) + AluSc SINE/Alu 1 308 (1) 11

1202 1.2 0.1 0.0 gi|89161214:62289528-62298770 4791 4951 (4292) C L1HS LINE/L1 (390) 5764 5603 12

**3762 0.0 0.0 0.0 gi|89161214:62289528-62298770 4952 5367 (3876) + L1HS LINE/L1 5740 6155 (0) 12 (R1)**

**411 2.1 0.0 0.0 gi|89161214:62289528-62298770 5368 5415 (3828) + (A)n Simple_repeat 1 48 (0) 13**

**3572 0.2 0.0 0.5 gi|89161214:62289528-62298770 5852 6245 (2998) + L1HS LINE/L1 5751 6144 (11) 14 (R2)**

350 2.1 2.1 0.0 gi|89161214:62289528-62298770 6246 6292 (2951) + (TAAA)n Simple_repeat 2 49 (0) 15

1080 20.5 1.8 1.4 gi|89161214:62289528-62298770 6321 6538 (2705) + MER20 DNA/hAT-Charlie 1 219 (0) 16

295 35.3 1.0 1.0 gi|89161214:62289528-62298770 6572 6674 (2569) + L2b LINE/L2 3253 3355 (20) 17

240 20.4 0.0 1.9 gi|89161214:62289528-62298770 7832 7886 (1357) C MLT2B3 LTR/ERVL (0) 556 503 18

242 16.0 0.0 2.0 gi|89161214:62289528-62298770 7904 7954 (1289) + (TA)n Simple_repeat 2 51 (0) 19

2255 20.4 3.9 0.7 gi|89161214:62289528-62298770 7955 8456 (787) C MLT2B3 LTR/ERVL (40) 522 3 18

23 34.8 0.0 0.0 gi|89161214:62289528-62298770 8761 8783 (460) + AT_rich Low_complexity 1 23 (0) 20

_________________________________________________________________________

L1HS_11_41c (Reference human genome) M_INTER_RMD

2330 9.4 0.0 1.0 chr11 59565615 59565925 (74886459) + AluSx SINE/Alu 1 308 (4) 148

1238 8.9 26.0 3.7 chr11 59565942 59566187 (74886197) + AluYa5 SINE/Alu 1 301 (9) 149

2326 7.9 1.6 0.0 chr11 59566948 59567252 (74885132) C AluY SINE/Alu (1) 310 1 150

215 32.2 3.8 5.3 chr11 59568270 59568400 (74883984) C MIR SINE/MIR (121) 141 13 151

469 7.3 14.6 0.0 chr11 59569732 59569813 (74882571) C L1MB3 LINE/L1 (3) 6180 6087 152

410 14.2 0.7 5.3 chr11 59569961 59570109 (74882275) + (TCTCTG)n Simple_repeat 1 142 (0) 153

630 14.9 0.0 2.8 chr11 59570110 59570288 (74882096) + (TC)n Simple_repeat 1 174 (0) 154

22 0.0 0.0 0.0 chr11 59572046 59572067 (74880317) + AT_rich Low_complexity 1 22 (0) 155

429 27.8 9.3 8.8 chr11 59572238 59572430 (74879954) C MIR SINE/MIR (67) 195 2 156

**12256 1.8 0.0 0.0 chr11 59572704 59575033 (74877351) C L1HS LINE/L1 (0) 6203 3873 157 (R12)**

7211 1.5 0.6 0.0 chr11 59575038 59575901 (74876483) + L1PA3 LINE/L1 5284 6152 (3) 158

253 16.7 1.8 1.8 chr11 59576255 59576309 (74876075) C MER5B DNA/MER1_type (0) 178 124 159

2102 13.3 0.7 1.0 chr11 59576320 59576622 (74875762) + AluSx SINE/Alu 1 302 (10) 160

39 2.2 0.0 0.0 chr11 59577523 59577568 (74874816) + AT_rich Low_complexity 1 46 (0) 161

3118 8.8 5.6 0.2 chr11 59577569 59578035 (74874349) + L1MA1 LINE/L1 5806 6297 (5) 162

2351 8.6 0.3 0.0 chr11 59578049 59578350 (74874034) + AluSc SINE/Alu 1 303 (6) 163

828 19.3 4.1 4.1 chr11 59578407 59578649 (74873735) + L1MA9 LINE/L1 6046 6288 (24) 164

366 28.2 6.4 0.0 chr11 59578750 59578859 (74873525) C MIRb SINE/MIR (48) 220 104 165

2146 14.2 0.0 0.0 chr11 59579583 59579891 (74872493) + AluSx SINE/Alu 1 309 (3) 166

L1HS_11_41c Ortholog in Celera

212 32.3 3.8 5.4 gi|89161188:57173918-57185787 518 648 (11222) C MIR SINE/MIR (121) 141 13 1

467 7.3 14.6 0.0 gi|89161188:57173918-57185787 1980 2061 (9809) C L1MB3 LINE/L1 (3) 6180 6087 2

523 15.5 1.0 4.6 gi|89161188:57173918-57185787 2209 2475 (9395) + (TCTCTG)n Simple_repeat 1 257 (0) 3

456 17.1 0.0 0.0 gi|89161188:57173918-57185787 2451 2561 (9309) + CT-rich Low_complexity 70 180 (0) 4 *

23 39.1 0.0 0.0 gi|89161188:57173918-57185787 4294 4316 (7554) + AT_rich Low_complexity 1 23 (0) 5

423 28.3 9.8 7.5 gi|89161188:57173918-57185787 4427 4678 (7192) C MIR SINE/MIR (6) 262 2 6

315 8.5 0.0 0.0 gi|89161188:57173918-57185787 4952 4998 (6872) + (T)n Simple_repeat 1 47 (0) 7

**8034 0.9 0.0 0.0 gi|89161188:57173918-57185787 5000 6276 (5594) C L1HS LINE/L1 (0) 6155 4879 8 (R2)**

**292 11.5 0.0 0.0 gi|89161188:57173918-57185787 6329 6380 (5490) + L1MB2 LINE/L1 6115 6166 (5) 9**

**501 33.9 8.1 0.0 gi|89161188:57173918-57185787 6395 6615 (5255) C MLT2F LTR/ERVL (236) 427 189 10**

**459 26.6 4.8 0.8 gi|89161188:57173918-57185787 6639 6763 (5107) C LTR33C LTR/ERVL (486) 143 14 11**

**708 27.5 3.8 1.6 gi|89161188:57173918-57185787 6767 7006 (4864) + L1M5 LINE/L1 5456 5700 (494) 12**

**2097 11.1 0.0 0.4 gi|89161188:57173918-57185787 7108 7378 (4492) + AluSz SINE/Alu 8 277 (35) 13**

**270 0.0 0.0 0.0 gi|89161188:57173918-57185787 7382 7411 (4459) + (CAAA)n Simple_repeat 1 30 (0) 14**

**11915 3.1 0.1 0.0 gi|89161188:57173918-57185787 7492 8919 (2951) C L1PA3 LINE/L1 (845) 5301 3873 15 (R1)**

7545 1.5 0.6 0.0 gi|89161188:57173918-57185787 8924 9787 (2083) + L1PA3 LINE/L1 5284 6152 (3) 15

270 16.7 1.8 1.8 gi|89161188:57173918-57185787 10141 10195 (1675) C MER5B DNA/hAT-Charlie (0) 178 124 16

2148 13.0 0.7 1.0 gi|89161188:57173918-57185787 10206 10505 (1365) + AluSz6 SINE/Alu 1 299 (13) 17

39 73.9 0.0 0.0 gi|89161188:57173918-57185787 11411 11456 (414) + AT_rich Low_complexity 1 46 (0) 18

2817 8.0 5.3 0.2 gi|89161188:57173918-57185787 11457 11870 (0) + L1MA1 LINE/L1 5806 6240 (62) 19

**Recombination mediated deletions – Truncated L1HS elements, Human Vs Celera comparison**

L1HS_1_63 Reference human genome C_INTER_RMD

2310 8.3 1.0 0.0 chr1 113680004 113680303 (133569416) C AluSp SINE/Alu (9) 304 2 480

15442 12.6 1.7 1.3 chr1 113680304 113681904 (133567815) C Tigger1 DNA/MER2_type (830) 1588 1 479

2265 10.1 0.3 1.3 chr1 113682091 113682392 (133567327) C AluSp SINE/Alu (14) 299 1 481

444 3.7 0.0 0.0 chr1 113683352 113683405 (133566314) + (TG)n Simple_repeat 2 55 (0) 482

324 25.7 0.0 1.3 chr1 113683409 113683483 (133566236) + L2 LINE/L2 3301 3374 (4) 483

314 14.6 0.0 0.0 chr1 113683523 113683577 (133566142) + AluSx SINE/Alu 245 299 (3) 484

4399 17.2 5.0 1.4 chr1 113683579 113684447 (133565272) + L1MB2 LINE/L1 5230 6135 (36) 485

360 39.5 5.2 4.8 chr1 113685480 113685748 (133563971) C L2 LINE/L2 (26) 3352 3083 486

**26479 3.4 0.1 0.2 chr1 113685753 113689935 (133559784) + L1HS LINE/L1 1995 6155 (0) 487 (R1)**

**7259 3.6 0.9 0.0 chr1 113689938 113690958 (133558761) + L1PA4 LINE/L1 5128 6155 (0) 488 (R2)**

233 32.4 9.2 1.4 chr1 113690974 113691114 (133558605) C L2 LINE/L2 (317) 3061 2910 486

1989 10.5 6.2 0.0 chr1 113691263 113691538 (133558181) C AluSg SINE/Alu (7) 303 11 489

1703 16.4 1.0 3.9 chr1 113691591 113691900 (133557819) C AluJo SINE/Alu (11) 301 1 490

297 29.5 0.0 1.3 chr1 113692262 113692340 (133557379) + MIR SINE/MIR 1 78 (184) 491

450 0.0 0.0 0.0 chr1 113692671 113692720 (133556999) + (TGGA)n Simple_repeat 4 53 (0) 492

843 6.9 0.0 0.0 chr1 113693058 113693159 (133556560) + AluSg/x SINE/Alu 135 236 (76) 493

387 25.5 12.9 1.2 chr1 113693161 113693323 (133556396) + MIR SINE/MIR 50 231 (31) 491

214 37.2 5.9 5.9 chr1 113693398 113693651 (133556068) C L2 LINE/L2 (455) 2964 2711 494

2507 5.2 0.3 1.3 chr1 113693652 113693960 (133555759) C AluY SINE/Alu (5) 306 1 495

262 35.4 3.1 1.0 chr1 113695067 113695163 (133554556) C MIRm SINE/MIR (21) 255 157 496

1204 18.7 8.1 3.4 chr1 113696363 113696683 (133553036) + MER44A DNA/MER2_type 1 336 (3) 497

Orhtolog in HuRef

7602 11.3 1.2 1.5 gi|157704448:111737462-111750565 1 1114 (11990) C Tigger1 DNA/TcMar-Tigger (1308) 1110 1 1

2314 9.9 0.3 1.3 gi|157704448:111737462-111750565 1301 1602 (11502) C AluSp SINE/Alu (14) 299 1 2

189 28.0 1.8 15.5 gi|157704448:111737462-111750565 2532 2562 (10542) + L2b LINE/L2 3287 3316 (71) 3

345 4.7 0.0 0.0 gi|157704448:111737462-111750565 2563 2605 (10499) + (TG)n Simple_repeat 2 44 (0) 4

300 27.6 0.2 1.8 gi|157704448:111737462-111750565 2606 2683 (10421) + L2b LINE/L2 3303 3383 (4) 3

353 10.6 0.0 0.0 gi|157704448:111737462-111750565 2723 2769 (10335) + AluJo SINE/Alu 245 291 (21) 5

3677 17.6 4.6 1.6 gi|157704448:111737462-111750565 2779 3647 (9457) + L1MB2 LINE/L1 5230 6135 (36) 6

383 33.0 8.1 5.4 gi|157704448:111737462-111750565 4680 4951 (8153) C L2c LINE/L2 (26) 3361 3083 7

**26405 4.1 0.3 0.2 gi|157704448:111737462-111750565 4952 9124 (3980) + L1PA4 LINE/L1 1995 6155 (0) 8 (R12)**

211 33.0 9.6 2.6 gi|157704448:111737462-111750565 9135 9280 (3824) C L2c LINE/L2 (322) 3065 2910 7

2032 10.9 6.2 0.0 gi|157704448:111737462-111750565 9429 9702 (3402) C AluSg SINE/Alu (9) 301 11 9

1824 16.2 0.3 3.3 gi|157704448:111737462-111750565 9755 10063 (3041) C AluJo SINE/Alu (11) 301 2 10

297 29.5 0.0 1.3 gi|157704448:111737462-111750565 10426 10504 (2600) + MIR SINE/MIR 1 78 (184) 11

414 0.0 0.0 0.0 gi|157704448:111737462-111750565 10835 10880 (2224) + (TGGA)n Simple_repeat 4 49 (0) 12

895 6.9 0.0 0.0 gi|157704448:111737462-111750565 11218 11319 (1785) + AluSx SINE/Alu 135 236 (76) 13

397 24.8 12.9 1.1 gi|157704448:111737462-111750565 11321 11483 (1621) + MIR SINE/MIR 50 231 (31) 11

202 37.9 5.5 5.9 gi|157704448:111737462-111750565 11558 11812 (1292) C L2 LINE/L2 (455) 2964 2711 14

2592 4.9 0.3 1.3 gi|157704448:111737462-111750565 11813 12119 (985) C AluY SINE/Alu (7) 304 1 15

___________________________________________________________________________

L1HS_2_41 Reference human genome M_INTER_RMD

2081 20.9 3.1 7.1 chr2 109287006 109287329 (133663820) C L1MB1 LINE/L1 (51) 6138 5804 665

355 22.7 11.6 1.6 chr2 109287500 109287620 (133663529) C Charlie5 DNA/MER1_type (2471) 141 9 666

9571 1.5 0.0 0.0 chr2 109290311 109292221 (133658928) C L1PA2 LINE/L1 (0) 6155 4244 667

**17275 1.7 0.5 0.0 chr2 109292220 109294476 (133656673) + L1HS LINE/L1 1845 4112 (1911) 668 (L1HS_2_41) (R12)**

2336 20.9 4.1 3.8 chr2 109295239 109295849 (133655300) C L1MB5 LINE/L1 (0) 6174 5562 669

287 26.1 0.0 0.0 chr2 109297925 109297993 (133653156) C MER3 DNA/MER1_type (140) 69 1 1

Ortholog in HuReF

511 6.5 0.0 0.0 gi|157724517:103517969-103531048 1 62 (13018) C L1MA7 LINE/L1 (426) 5865 5804 1

369 18.8 3.5 0.0 gi|157724517:103517969-103531048 242 326 (12754) C Charlie5 DNA/hAT-Charlie (2492) 132 45 2

10143 1.4 0.1 0.0 gi|157724517:103517969-103531048 3044 4953 (8127) C L1PA2 LINE/L1 (0) 6155 4244 3

**2469 2.7 2.5 0.0 gi|157724517:103517969-103531048 4952 5398 (7682) + L1HS LINE/L1 1968 2425 (3721) 4 * (R1)**

**3759 1.5 2.4 0.0 gi|157724517:103517969-103531048 5419 5875 (7205) C L1P1 LINE/L1 (1435) 4711 4244 5 ***

**18297 1.7 0.5 0.0 gi|157724517:103517969-103531048 5874 8129 (4951) + L1P1 LINE/L1 1968 4235 (1911) 5 (R2)**

2355 20.3 4.6 3.8 gi|157724517:103517969-103531048 8893 9500 (3580) C L1MB5 LINE/L1 (0) 6174 5562 6

294 26.1 0.0 0.0 gi|157724517:103517969-103531048 11576 11644 (1436) C MER3 DNA/hAT-Charlie (140) 69 1 7

__________________________________________________________________________________

L1HS_2_49c Reference human genome M_DISRUPTED

1290 25.2 14.7 6.5 chr2 129881918 129882899 (113068250) C L1M4b LINE/L1 (3935) 3252 2065 407

1474 22.9 2.3 5.0 chr2 129882900 129883295 (113067854) C MLT1B LTR/MaLR (3) 387 3 409

906 28.3 10.4 6.6 chr2 129883296 129883441 (113067708) C L1MEc LINE/L1 (4963) 1437 1294 410

456 30.4 4.5 2.4 chr2 129883441 129883686 (113067463) C L1M5 LINE/L1 (736) 5504 5254 411

648 29.4 10.0 4.1 chr2 129884022 129884650 (113066499) C L1MC4 LINE/L1 (4355) 1860 1196 412

3280 2.1 0.3 0.3 chr2 129884651 129885037 (113066112) C L1PA3 LINE/L1 (0) 6155 5769 413

648 29.4 10.0 4.1 chr2 129885038 129885094 (113066055) C L1MC4 LINE/L1 (5020) 1195 1133 412

**28729 0.4 0.0 0.1 chr2 129885097 129889422 (113061727) C L1HS LINE/L1 (0) 6032 1715 414 (L1HS_2_49c) (R1)**

**2813 0.7 0.0 0.3 chr2 129889423 129889730 (113061419) + AluYa5 SINE/Al 1 307 (3) 415 (D)**

**18979 0.3 0.1 0.4 chr2 129889731 129891443 (113059706) C L1HS LINE/L1 (4318) 1714 2 416 (R2)**

1482 20.7 6.8 0.3 chr2 129892380 129892733 (113058416) C L1MB7 LINE/L1 (203) 5981 5605 417

622 16.1 0.0 0.0 chr2 129892755 129892866 (113058283) C L1MB7 LINE/L1 (706) 5472 5361 417

460 24.1 22.9 3.6 chr2 129892877 129893212 (113057937) C L1M4c LINE/L1 (4223) 2423 2013 418

4565 23.8 5.6 2.7 chr2 129893209 129894517 (113056632) C L1M4c LINE/L1 (5200) 1342 3 418

303 26.3 3.1 1.0 chr2 129894678 129894773 (113056376) C L1MEe LINE/L1 (4645) 209 112 419

2406 9.1 0.0 0.0 chr2 129895079 129895376 (113055773) + AluSc SINE/Alu 6 303 (6) 420

923 33.5 8.8 1.9 chr2 129895379 129895904 (113055245) + L1MCc LINE/L1 49 610 (5813) 421

289 32.5 4.0 6.2 chr2 129896078 129896254 (113054895) + Kanga1 DNA/Tc2 534 706 (1192) 422

795 31.8 7.0 3.3 chr2 129896337 129896762 (113054387) + L2 LINE/L2 193 634 (2785) 423

Ortholog in HuRef

954 18.9 0.5 0.0 gi|157724517:122471407-122485672 5 194 (14072) C L1M4c LINE/L1 (2191) 4191 4001 1

28 64.3 0.0 0.0 gi|157724517:122471407-122485672 196 223 (14043) + AT_rich Low_complexity 1 28 (0) 2

26 50.0 0.0 0.0 gi|157724517:122471407-122485672 201 226 (14040) + AT_rich Low_complexity 1 26 (0) 3 *

26 87.0 0.0 0.0 gi|157724517:122471407-122485672 237 290 (13976) + AT_rich Low_complexity 1 54 (0) 4

1010 23.5 4.6 8.6 gi|157724517:122471407-122485672 291 700 (13566) C L1M4c LINE/L1 (2414) 3968 3574 1 *

2586 21.8 2.4 1.7 gi|157724517:122471407-122485672 689 1388 (12878) C L1M4c LINE/L1 (3142) 3240 2536 1

1127 21.1 7.0 9.9 gi|157724517:122471407-122485672 1391 1756 (12510) C MLT1A0 LTR/ERVL-MaLR (0) 396 1 5

1707 26.5 4.5 5.6 gi|157724517:122471407-122485672 1760 2741 (11525) C L1M4c LINE/L1 (3856) 2526 1439 1

1470 22.9 2.3 5.2 gi|157724517:122471407-122485672 2742 3137 (11129) C MLT1B LTR/ERVL-MaLR (3) 387 3 6

1707 24.0 2.8 5.3 gi|157724517:122471407-122485672 3138 3287 (10979) C L1M4c LINE/L1 (4946) 1438 1299 1

630 28.1 3.3 1.2 gi|157724517:122471407-122485672 3287 3531 (10735) C L1MC4 LINE/L1 (670) 5571 5322 7 *

661 30.4 7.8 3.6 gi|157724517:122471407-122485672 3867 4447 (9819) C L1MC4 LINE/L1 (4355) 1860 1257 7

3317 1.6 0.3 0.3 gi|157724517:122471407-122485672 4500 4886 (9380) C L1PA3 LINE/L1 (0) 6155 5769 8

297 10.9 0.0 0.0 gi|157724517:122471407-122485672 4896 4950 (9316) + (T)n Simple_repeat 1 55 (0) 9

**28768 0.3 0.0 0.0 gi|157724517:122471407-122485672 4952 10981 (3285) C L1HS LINE/L1 (0) 6155 125 10 (R)**

1473 19.4 5.3 0.3 gi|157724517:122471407-122485672 11918 12238 (2028) C L1MB7 LINE/L1 (203) 5981 5645 11

621 16.1 0.0 0.0 gi|157724517:122471407-122485672 12293 12404 (1862) C L1MB7 LINE/L1 (712) 5472 5361 11

435 26.1 6.7 3.1 gi|157724517:122471407-122485672 12415 12746 (1520) C L1M4 LINE/L1 (4223) 2785 2330 12

4420 25.2 5.3 2.9 gi|157724517:122471407-122485672 12747 14055 (211) C L1M4 LINE/L1 (5200) 1704 365 12

249 12.2 0.0 0.0 gi|157724517:122471407-122485672 14216 14256 (10) C L1MEg LINE/L1 (4733) 1351 1311 13

___________________________________________________________________________________________________________________________

L1HS_2_50c Reference human genome M_DISRUPTED

1290 25.2 14.7 6.5 chr2 129881918 129882899 (113068250) C L1M4b LINE/L1 (3935) 3252 2065 407

1474 22.9 2.3 5.0 chr2 129882900 129883295 (113067854) C MLT1B LTR/MaLR (3) 387 3 409

906 28.3 10.4 6.6 chr2 129883296 129883441 (113067708) C L1MEc LINE/L1 (4963) 1437 1294 410

456 30.4 4.5 2.4 chr2 129883441 129883686 (113067463) C L1M5 LINE/L1 (736) 5504 5254 411

648 29.4 10.0 4.1 chr2 129884022 129884650 (113066499) C L1MC4 LINE/L1 (4355) 1860 1196 412

3280 2.1 0.3 0.3 chr2 129884651 129885037 (113066112) C L1PA3 LINE/L1 (0) 6155 5769 413

648 29.4 10.0 4.1 chr2 129885038 129885094 (113066055) C L1MC4 LINE/L1 (5020) 1195 1133 412

**28729 0.4 0.0 0.1 chr2 129885097 129889422 (113061727) C L1HS LINE/L1 (0) 6032 1715 414 (R1)**

**2813 0.7 0.0 0.3 chr2 129889423 129889730 (113061419) + AluYa5 SINE/Alu 1 307 (3) 415 (D)**

**18979 0.3 0.1 0.4 chr2 129889731 129891443 (113059706) C L1HS LINE/L1 (4318) 1714 2 416 (R2) (L1HS_2_50C)**

1482 20.7 6.8 0.3 chr2 129892380 129892733 (113058416) C L1MB7 LINE/L1 (203) 5981 5605 417

622 16.1 0.0 0.0 chr2 129892755 129892866 (113058283) C L1MB7 LINE/L1 (706) 5472 5361 417

460 24.1 22.9 3.6 chr2 129892877 129893212 (113057937) C L1M4c LINE/L1 (4223) 2423 2013 418

4565 23.8 5.6 2.7 chr2 129893209 129894517 (113056632) C L1M4c LINE/L1 (5200) 1342 3 418

303 26.3 3.1 1.0 chr2 129894678 129894773 (113056376) C L1MEe LINE/L1 (4645) 209 112 419

2406 9.1 0.0 0.0 chr2 129895079 129895376 (113055773) + AluSc SINE/Alu 6 303 (6) 420

923 33.5 8.8 1.9 chr2 129895379 129895904 (113055245) + L1MCc LINE/L1 49 610 (5813) 421

289 32.5 4.0 6.2 chr2 129896078 129896254 (113054895) + Kanga1 DNA/Tc2 534 706 (1192) 422

795 31.8 7.0 3.3 chr2 129896337 129896762 (113054387) + L2 LINE/L2 193 634 (2785) 423

Ortholog in HuRef

954 18.9 0.5 0.0 gi|157724517:122471407-122485672 5 194 (14072) C L1M4c LINE/L1 (2191) 4191 4001 1

28 64.3 0.0 0.0 gi|157724517:122471407-122485672 196 223 (14043) + AT_rich Low_complexity 1 28 (0) 2

26 50.0 0.0 0.0 gi|157724517:122471407-122485672 201 226 (14040) + AT_rich Low_complexity 1 26 (0) 3 *

26 87.0 0.0 0.0 gi|157724517:122471407-122485672 237 290 (13976) + AT_rich Low_complexity 1 54 (0) 4

1010 23.5 4.6 8.6 gi|157724517:122471407-122485672 291 700 (13566) C L1M4c LINE/L1 (2414) 3968 3574 1 *

2586 21.8 2.4 1.7 gi|157724517:122471407-122485672 689 1388 (12878) C L1M4c LINE/L1 (3142) 3240 2536 1

1127 21.1 7.0 9.9 gi|157724517:122471407-122485672 1391 1756 (12510) C MLT1A0 LTR/ERVL-MaLR (0) 396 1 5

1707 26.5 4.5 5.6 gi|157724517:122471407-122485672 1760 2741 (11525) C L1M4c LINE/L1 (3856) 2526 1439 1

1470 22.9 2.3 5.2 gi|157724517:122471407-122485672 2742 3137 (11129) C MLT1B LTR/ERVL-MaLR (3) 387 3 6

1707 24.0 2.8 5.3 gi|157724517:122471407-122485672 3138 3287 (10979) C L1M4c LINE/L1 (4946) 1438 1299 1

630 28.1 3.3 1.2 gi|157724517:122471407-122485672 3287 3531 (10735) C L1MC4 LINE/L1 (670) 5571 5322 7 *

661 30.4 7.8 3.6 gi|157724517:122471407-122485672 3867 4447 (9819) C L1MC4 LINE/L1 (4355) 1860 1257 7

3317 1.6 0.3 0.3 gi|157724517:122471407-122485672 4500 4886 (9380) C L1PA3 LINE/L1 (0) 6155 5769 8

297 10.9 0.0 0.0 gi|157724517:122471407-122485672 4896 4950 (9316) + (T)n Simple_repeat 1 55 (0) 9

**28768 0.3 0.0 0.0 gi|157724517:122471407-122485672 4952 10981 (3285) C L1HS LINE/L1 (0) 6155 125 10 (R)**

1473 19.4 5.3 0.3 gi|157724517:122471407-122485672 11918 12238 (2028) C L1MB7 LINE/L1 (203) 5981 5645 11

621 16.1 0.0 0.0 gi|157724517:122471407-122485672 12293 12404 (1862) C L1MB7 LINE/L1 (712) 5472 5361 11

435 26.1 6.7 3.1 gi|157724517:122471407-122485672 12415 12746 (1520) C L1M4 LINE/L1 (4223) 2785 2330 12

4420 25.2 5.3 2.9 gi|157724517:122471407-122485672 12747 14055 (211) C L1M4 LINE/L1 (5200) 1704 365 12

249 12.2 0.0 0.0 gi|157724517:122471407-122485672 14216 14256 (10) C L1MEg LINE/L1 (4733) 1351 1311 13

___________________________________________________________________________________________________________________________

L1HS_3_14c Reference human genome C_INTER_RMD ??

189 7.4 0.0 0.0 chr3 22060937 22060963 (177440864) + (CAAAAA)n Simple_repeat 4 30 (0) 37

450 32.2 11.7 0.8 chr3 22061065 22061330 (177440497) C L2 LINE/L2 (1667) 1752 1458 36

466 12.7 0.0 0.0 chr3 22061452 22061522 (177440305) + AluSp/q SINE/Alu 226 296 (17) 38

300 14.5 1.6 0.0 chr3 22061523 22061584 (177440243) C L1M2 LINE/L1 (1817) 4326 4264 39

728 20.1 7.7 1.6 chr3 22061616 22061797 (177440030) C L1M2 LINE/L1 (1010) 5133 4941 40

7282 15.7 6.0 3.6 chr3 22063602 22065510 (177436317) C L1MA8 LINE/L1 (2) 6289 4316 41

2028 12.4 0.3 0.0 chr3 22065511 22065836 (177435991) + AluSq SINE/Alu 1 327 (0) 42

7282 17.9 8.8 3.6 chr3 22065837 22066576 (177435251) C L1MA8 LINE/L1 (1828) 4315 3551 41

1379 22.6 1.4 0.0 chr3 22066580 22066854 (177434973) + AluJo SINE/Alu 1 279 (33) 43

231 6.1 0.0 0.0 chr3 22066855 22066887 (177434940) + (CAAA)n Simple_repeat 1 33 (0) 44

**16108 0.3 0.0 0.0 chr3 22066888 22069693 (177432134) C L1HS LINE/L1 (2586) 3437 329 45 (R1) (L1HS_3_14c)**

**20482 0.2 0.0 0.0 chr3 22069692 22072804 (177429023) + L1HS LINE/L1 3064 6176 (0) 46 (R2)**

335 28.2 3.1 3.9 chr3 22073187 22073315 (177428512) C MLT1H LTR/MaLR (16) 533 406 47

245 33.3 0.0 0.0 chr3 22073404 22073481 (177428346) C MLT1H2 LTR/MaLR (323) 161 84 48

646 29.4 3.1 5.3 chr3 22073622 22073874 (177427953) + MIRb SINE/MIR 14 262 (0) 49

2103 12.5 0.0 1.3 chr3 22074010 22074309 (177427518) C AluSx SINE/Alu (16) 296 1 50

216 9.7 0.0 0.0 chr3 22074491 22074521 (177427306) + (TGAA)n Simple_repeat 3 33 (0) 51

23 0.0 0.0 0.0 chr3 22075113 22075135 (177426692) + AT_rich Low_complexity 1 23 (0) 52

22 0.0 0.0 0.0 chr3 22075176 22075197 (177426630) + AT_rich Low_complexity 1 22 (0) 53

274 23.2 2.9 1.4 chr3 22075816 22075885 (177425942) C MER5B DNA/MER1_type (104) 74 4 54

282 29.6 0.0 0.0 chr3 22075963 22076050 (177425777) C Charlie2 DNA/MER1_type (2598) 263 176 55

288 24.5 7.9 3.0 chr3 22076077 22076177 (177425650) C Charlie2 DNA/MER1_type (2751) 110 5 55

Ortholog in HuRef

5342 15.8 6.9 3.0 gi|157731950:22036680-22048120 1666 3574 (7867) C L1MA8 LINE/L1 (2) 6289 4316 1

2095 12.4 0.3 0.0 gi|157731950:22036680-22048120 3575 3900 (7541) + AluSq SINE/Alu 1 313 (0) 2

3013 18.1 7.7 5.1 gi|157731950:22036680-22048120 3901 4640 (6801) C L1MA8 LINE/L1 (1828) 4315 3551 1

1507 22.6 1.4 0.0 gi|157731950:22036680-22048120 4644 4918 (6523) + AluJo SINE/Alu 1 279 (33) 3

231 6.1 0.0 0.0 gi|157731950:22036680-22048120 4919 4951 (6490) + (CAAA)n Simple_repeat 1 33 (0) 4

**871 16.3 6.2 0.5 gi|157731950:22036680-22048120 4992 5170 (6271) C L1MA8 LINE/L1 (2585) 3558 3370 1 (R12 ????)**

23 53.3 0.0 0.0 gi|157731950:22036680-22048120 5320 5349 (6092) + AT_rich Low_complexity 1 30 (0) 5

337 26.9 0.8 2.6 gi|157731950:22036680-22048120 5803 5931 (5510) C MLT1H2 LTR/ERVL-MaLR (16) 533 382 6

230 31.2 3.0 0.0 gi|157731950:22036680-22048120 6020 6120 (5321) C MLT1J2 LTR/ERVL-MaLR (328) 161 65 7

654 29.4 3.1 5.4 gi|157731950:22036680-22048120 6238 6490 (4951) + MIRb SINE/MIR 14 262 (0) 8

2196 12.3 0.0 1.4 gi|157731950:22036680-22048120 6626 6926 (4515) C AluSx SINE/Alu (15) 297 1 9

216 9.7 0.0 0.0 gi|157731950:22036680-22048120 7108 7138 (4303) + (TGAA)n Simple_repeat 3 33 (0) 10

25 56.0 0.0 0.0 gi|157731950:22036680-22048120 7729 7753 (3688) + AT_rich Low_complexity 1 25 (0) 11

22 54.5 0.0 0.0 gi|157731950:22036680-22048120 7793 7814 (3627) + AT_rich Low_complexity 1 22 (0) 12

230 26.7 10.0 0.0 gi|157731950:22036680-22048120 7827 7916 (3525) + Tigger16a DNA/TcMar-Tigger 742 840 (93) 13

267 23.2 2.9 1.4 gi|157731950:22036680-22048120 8433 8502 (2939) C MER5B DNA/hAT-Charlie (104) 74 4 14

284 29.6 0.0 0.0 gi|157731950:22036680-22048120 8580 8667 (2774) C Charlie2a DNA/hAT-Charlie (2598) 263 176 15

_________________________________________________________________________

L1HS_4_8 (Reference human genome) M_DISRUPTED

203 33.3 5.3 6.1 chr4 16547199 16547329 (174725734) C MIR3 SINE/MIR (31) 177 48 220

704 17.0 0.0 0.9 chr4 16547348 16547460 (174725603) + FLAM_A SINE/Alu 8 119 (23) 221

2055 14.4 0.0 0.6 chr4 16547480 16547793 (174725270) C AluSx SINE/Alu (0) 312 1 222

286 34.1 3.2 2.4 chr4 16547995 16548120 (174724943) + MIRb SINE/MIR 40 166 (102) 223

1723 13.4 1.0 3.1 chr4 16548440 16548732 (174724331) + AluJb SINE/Alu 23 309 (3) 224

295 20.8 8.3 0.0 chr4 16550074 16550145 (174722918) + L1ME4a LINE/L1 5715 5792 (329) 225

365 30.1 7.1 5.2 chr4 16550581 16550888 (174722175) + L1ME4a LINE/L1 5789 6102 (19) 225

253 33.0 5.5 0.0 chr4 16551405 16551495 (174721568) C MIR3 SINE/MIR (46) 162 67 226

225 34.2 2.5 5.8 chr4 16551517 16551637 (174721426) C L2 LINE/L2 (5) 3373 3257 227

196 25.6 0.0 0.0 chr4 16551805 16551847 (174721216) + L2 LINE/L2 3373 3415 (4) 228

27 6.2 0.0 0.0 chr4 16551854 16551901 (174721162) + AT_rich Low_complexity 1 48 (0) 229

27 8.7 0.0 0.0 chr4 16551944 16552012 (174721051) + AT_rich Low_complexity 1 69 (0) 230

207 33.8 8.4 0.0 chr4 16552058 16552128 (174720935) C MIRb SINE/MIR (29) 239 163 231

523 33.1 7.9 4.7 chr4 16552156 16552409 (174720654) C MIR SINE/MIR (0) 262 1 232

404 13.4 10.4 0.0 chr4 16553284 16553350 (174719713) C MLT1C LTR/MaLR (0) 467 394 233

**19171 0.6 0.1 0.7 chr4 16553498 16555351 (174717712) + L1HS LINE/L1 3 1857 (4175) 234 (R1) (L1HS_4_8)**

**2782 0.7 0.0 0.0 chr4 16555352 16555647 (174717416) + AluYb8 SINE/Alu 23 318 (0) 235 (D)**

**29360 0.5 0.0 0.0 chr4 16555648 16559834 (174713229) + L1HS LINE/L1 1858 6031 (1) 236 (R2)**

2548 15.3 2.2 0.0 chr4 16559835 16560240 (174712823) C MLT1C LTR/MaLR (52) 415 1 233

2433 8.3 0.0 0.0 chr4 16560272 16560572 (174712491) C AluY SINE/Alu (9) 302 2 237

1921 12.0 0.0 9.6 chr4 16560600 16560913 (174712150) + AluSx SINE/Alu 1 284 (28) 238

182 3.7 3.7 0.0 chr4 16560914 16560940 (174712123) + (TAA)n Simple_repeat 2 29 (0) 239

2195 9.0 1.4 0.0 chr4 16560960 16561248 (174711815) C L1PA10 LINE/L1 (248) 5920 5628 240

2605 16.2 1.0 5.6 chr4 16561719 16562221 (174710842) + MLT1D LTR/MaLR 26 505 (0) 241

189 13.9 0.0 2.7 chr4 16563100 16563136 (174709927) C MIRm SINE/MIR (11) 265 230 242

213 3.9 0.0 0.0 chr4 16564221 16564246 (174708817) + (A)n Simple_repeat 1 26 (0) 243

2192 16.9 5.8 6.2 chr4 16565149 16565582 (174707481) C MSTB1 LTR/MaLR (0) 432 1 244

1257 16.7 1.2 7.4 chr4 16565590 16565847 (174707216) C MSTB-int LTR/MaLR (7) 1644 1403 244

3213 22.0 1.7 0.3 chr4 16565845 16566505 (174706558) C MSTB-int LTR/MaLR (981) 670 1 244

523 17.2 2.1 0.0 chr4 16566510 16566602 (174706461) C MSTB1 LTR/MaLR (335) 97 3 245

2464 19.1 2.9 0.0 chr4 16566759 16567172 (174705891) C MSTB LTR/MaLR (0) 426 1 244

Ortholog in HuRef

1159 14.6 1.1 3.3 gi|157734150:16293153-16304946 2 186 (11608) + AluJb SINE/Alu 129 309 (3) 1

184 31.8 1.5 3.0 gi|157734150:16293153-16304946 266 333 (11461) + L2c LINE/L2 3289 3355 (32) 2

276 19.4 8.3 0.0 gi|157734150:16293153-16304946 1528 1599 (10195) + L1MC LINE/L1 5713 5790 (2092) 3

691 25.6 3.6 1.5 gi|157734150:16293153-16304946 1612 1805 (9989) + LTR79 LTR/ERVL 20 217 (310) 4

356 26.1 18.9 0.5 gi|157734150:16293153-16304946 1837 2032 (9762) + LTR79 LTR/ERVL 296 527 (0) 4

250 32.5 12.3 0.7 gi|157734150:16293153-16304946 2859 2996 (8798) C MIR3 SINE/MIR (46) 162 12 5

189 37.6 0.8 3.4 gi|157734150:16293153-16304946 2971 3091 (8703) C L2a LINE/L2 (5) 3421 3304 6 *

217 22.7 0.0 0.0 gi|157734150:16293153-16304946 3258 3301 (8493) + L2c LINE/L2 3340 3383 (4) 2

27 72.9 0.0 0.0 gi|157734150:16293153-16304946 3308 3355 (8439) + AT_rich Low_complexity 1 48 (0) 7

27 89.9 0.0 0.0 gi|157734150:16293153-16304946 3398 3466 (8328) + AT_rich Low_complexity 1 69 (0) 8

203 33.8 8.4 0.0 gi|157734150:16293153-16304946 3512 3582 (8212) C MIRb SINE/MIR (29) 239 163 9

510 33.6 4.8 4.8 gi|157734150:16293153-16304946 3633 3863 (7931) C MIR SINE/MIR (31) 231 1 10

398 13.4 10.4 0.0 gi|157734150:16293153-16304946 4738 4804 (6990) C MLT1C LTR/ERVL-MaLR (0) 467 394 11

**19295 0.6 0.3 0.0 gi|157734150:16293153-16304946 4952 8097 (3697) + L1HS LINE/L1 126 3282 (2864) 12 (R)**

**18409 0.5 0.0 0.0 gi|157734150:16293153-16304946 8249 11054 (740) + L1HS LINE/L1 3347 6152 (3) 12 (R) BROKEN DUE TO Ns**

2544 15.3 2.2 0.0 gi|157734150:16293153-16304946 11055 11460 (334) C MLT1C LTR/ERVL-MaLR (52) 415 1 11

2604 8.3 0.0 0.0 gi|157734150:16293153-16304946 11492 11792 (2) C AluY SINE/Alu (9) 302 2 13

_____________________________________________________________________________________________

L1HS_4_9 (Reference human genome) M_DISRUPTED

203 33.3 5.3 6.1 chr4 16547199 16547329 (174725734) C MIR3 SINE/MIR (31) 177 48 220

704 17.0 0.0 0.9 chr4 16547348 16547460 (174725603) + FLAM_A SINE/Alu 8 119 (23) 221

2055 14.4 0.0 0.6 chr4 16547480 16547793 (174725270) C AluSx SINE/Alu (0) 312 1 222

286 34.1 3.2 2.4 chr4 16547995 16548120 (174724943) + MIRb SINE/MIR 40 166 (102) 223

1723 13.4 1.0 3.1 chr4 16548440 16548732 (174724331) + AluJb SINE/Alu 23 309 (3) 224

295 20.8 8.3 0.0 chr4 16550074 16550145 (174722918) + L1ME4a LINE/L1 5715 5792 (329) 225

365 30.1 7.1 5.2 chr4 16550581 16550888 (174722175) + L1ME4a LINE/L1 5789 6102 (19) 225

253 33.0 5.5 0.0 chr4 16551405 16551495 (174721568) C MIR3 SINE/MIR (46) 162 67 226

225 34.2 2.5 5.8 chr4 16551517 16551637 (174721426) C L2 LINE/L2 (5) 3373 3257 227

196 25.6 0.0 0.0 chr4 16551805 16551847 (174721216) + L2 LINE/L2 3373 3415 (4) 228

27 6.2 0.0 0.0 chr4 16551854 16551901 (174721162) + AT_rich Low_complexity 1 48 (0) 229

27 8.7 0.0 0.0 chr4 16551944 16552012 (174721051) + AT_rich Low_complexity 1 69 (0) 230

207 33.8 8.4 0.0 chr4 16552058 16552128 (174720935) C MIRb SINE/MIR (29) 239 163 231

523 33.1 7.9 4.7 chr4 16552156 16552409 (174720654) C MIR SINE/MIR (0) 262 1 232

404 13.4 10.4 0.0 chr4 16553284 16553350 (174719713) C MLT1C LTR/MaLR (0) 467 394 233

**19171 0.6 0.1 0.7 chr4 16553498 16555351 (174717712) + L1HS LINE/L1 3 1857 (4175) 234 (R1) (L1HS_4_9)**

**2782 0.7 0.0 0.0 chr4 16555352 16555647 (174717416) + AluYb8 SINE/Alu 23 318 (0) 235 (D)**

**29360 0.5 0.0 0.0 chr4 16555648 16559834 (174713229) + L1HS LINE/L1 1858 6031 (1) 236 (R2)**

2548 15.3 2.2 0.0 chr4 16559835 16560240 (174712823) C MLT1C LTR/MaLR (52) 415 1 233

2433 8.3 0.0 0.0 chr4 16560272 16560572 (174712491) C AluY SINE/Alu (9) 302 2 237

1921 12.0 0.0 9.6 chr4 16560600 16560913 (174712150) + AluSx SINE/Alu 1 284 (28) 238

182 3.7 3.7 0.0 chr4 16560914 16560940 (174712123) + (TAA)n Simple_repeat 2 29 (0) 239

2195 9.0 1.4 0.0 chr4 16560960 16561248 (174711815) C L1PA10 LINE/L1 (248) 5920 5628 240

2605 16.2 1.0 5.6 chr4 16561719 16562221 (174710842) + MLT1D LTR/MaLR 26 505 (0) 241

189 13.9 0.0 2.7 chr4 16563100 16563136 (174709927) C MIRm SINE/MIR (11) 265 230 242

213 3.9 0.0 0.0 chr4 16564221 16564246 (174708817) + (A)n Simple_repeat 1 26 (0) 243

2192 16.9 5.8 6.2 chr4 16565149 16565582 (174707481) C MSTB1 LTR/MaLR (0) 432 1 244

1257 16.7 1.2 7.4 chr4 16565590 16565847 (174707216) C MSTB-int LTR/MaLR (7) 1644 1403 244

3213 22.0 1.7 0.3 chr4 16565845 16566505 (174706558) C MSTB-int LTR/MaLR (981) 670 1 244

523 17.2 2.1 0.0 chr4 16566510 16566602 (174706461) C MSTB1 LTR/MaLR (335) 97 3 245

2464 19.1 2.9 0.0 chr4 16566759 16567172 (174705891) C MSTB LTR/MaLR (0) 426 1 244

Ortholog in HuRef

1159 14.6 1.1 3.3 gi|157734150:16293153-16304946 2 186 (11608) + AluJb SINE/Alu 129 309 (3) 1

184 31.8 1.5 3.0 gi|157734150:16293153-16304946 266 333 (11461) + L2c LINE/L2 3289 3355 (32) 2

276 19.4 8.3 0.0 gi|157734150:16293153-16304946 1528 1599 (10195) + L1MC LINE/L1 5713 5790 (2092) 3

691 25.6 3.6 1.5 gi|157734150:16293153-16304946 1612 1805 (9989) + LTR79 LTR/ERVL 20 217 (310) 4

356 26.1 18.9 0.5 gi|157734150:16293153-16304946 1837 2032 (9762) + LTR79 LTR/ERVL 296 527 (0) 4

250 32.5 12.3 0.7 gi|157734150:16293153-16304946 2859 2996 (8798) C MIR3 SINE/MIR (46) 162 12 5

189 37.6 0.8 3.4 gi|157734150:16293153-16304946 2971 3091 (8703) C L2a LINE/L2 (5) 3421 3304 6 *

217 22.7 0.0 0.0 gi|157734150:16293153-16304946 3258 3301 (8493) + L2c LINE/L2 3340 3383 (4) 2

27 72.9 0.0 0.0 gi|157734150:16293153-16304946 3308 3355 (8439) + AT_rich Low_complexity 1 48 (0) 7

27 89.9 0.0 0.0 gi|157734150:16293153-16304946 3398 3466 (8328) + AT_rich Low_complexity 1 69 (0) 8

203 33.8 8.4 0.0 gi|157734150:16293153-16304946 3512 3582 (8212) C MIRb SINE/MIR (29) 239 163 9

510 33.6 4.8 4.8 gi|157734150:16293153-16304946 3633 3863 (7931) C MIR SINE/MIR (31) 231 1 10

398 13.4 10.4 0.0 gi|157734150:16293153-16304946 4738 4804 (6990) C MLT1C LTR/ERVL-MaLR (0) 467 394 11

**19295 0.6 0.3 0.0 gi|157734150:16293153-16304946 4952 8097 (3697) + L1HS LINE/L1 126 3282 (2864) 12 (R)**

**18409 0.5 0.0 0.0 gi|157734150:16293153-16304946 8249 11054 (740) + L1HS LINE/L1 3347 6152 (3) 12 (R) BROKEN DUE TO Ns**

2544 15.3 2.2 0.0 gi|157734150:16293153-16304946 11055 11460 (334) C MLT1C LTR/ERVL-MaLR (52) 415 1 11

2604 8.3 0.0 0.0 gi|157734150:16293153-16304946 11492 11792 (2) C AluY SINE/Alu (9) 302 2 13

__________________________________________________________________________

L1HS_4_29 Reference human genome

2221 10.8 0.0 0.0 chr4 48106376 48106661 (143166402) + AluSx SINE/Alu 1 286 (26) 166

2628 15.2 5.3 3.1 chr4 48106662 48107309 (143165754) C L1MB7 LINE/L1 (478) 5706 5040 162

186 18.9 0.0 0.0 chr4 48107730 48107766 (143165297) + CT-rich Low_complexity 1 37 (0) 167

198 7.1 0.0 0.0 chr4 48107870 48107897 (143165166) + (TG)n Simple_repeat 2 29 (0) 168

920 11.6 1.3 1.3 chr4 48108145 48108311 (143164752) C L1MB7 LINE/L1 (1089) 5057 4881 162

459 0.0 0.0 0.0 chr4 48109140 48109190 (143163873) + (CA)n Simple_repeat 2 52 (0) 169

1667 19.0 0.7 0.7 chr4 48109720 48110016 (143163047) + AluSx SINE/Alu 1 297 (15) 170

313 29.0 4.2 3.5 chr4 48110060 48110202 (143162861) + MIR3 SINE/MIR 21 164 (44) 171

23 5.4 0.0 0.0 chr4 48110770 48110806 (143162257) + AT_rich Low_complexity 1 37 (0) 172

11771 0.5 0.0 0.0 chr4 48111332 48113422 (143159641) C L1HS LINE/L1 (0) 6155 4065 173

**10629 0.4 0.3 0.0 chr4 48113419 48114647 (143158416) + L1HS LINE/L1 2780 4012 (2134) 173 (L1HS_4_29)**

3013 17.4 6.6 10.9 chr4 48114645 48115527 (143157536) C L1MD LINE/L1 (1762) 4384 3540 174

910 18.0 9.4 3.1 chr4 48115556 48115865 (143157198) C L1ME1 LINE/L1 (189) 5975 5665 175

2223 11.5 0.0 0.3 chr4 48115869 48116174 (143156889) C AluSx SINE/Alu (4) 308 4 176

900 20.0 1.2 0.0 chr4 48116178 48116337 (143156726) C AluSg/x SINE/Alu (15) 297 136 177

274 35.0 1.7 2.5 chr4 48117623 48117742 (143155321) C MER5A DNA/MER1_type (3) 186 68 178

1703 15.5 3.2 0.0 chr4 48117766 48118058 (143155005) C AluJb SINE/Alu (0) 324 23 179

195 8.2 4.0 2.0 chr4 48118059 48118092 (143154971) + (TTTG)n Simple_repeat 2 36 (0) 180

617 32.4 8.1 1.0 chr4 48118216 48118424 (143154639) C MIRb SINE/MIR (43) 225 2 181

23 0.0 0.0 0.0 chr4 48120916 48120938 (143152125) + AT_rich Low_complexity 1 23 (0) 182

Ortholog in HuRef

414 0.0 0.0 0.0 gi|157734150:47734030-47745251 690 735 (10487) + (CA)n Simple_repeat 2 47 (0) 1

1847 17.0 0.7 0.7 gi|157734150:47734030-47745251 1262 1555 (9667) + AluSz6 SINE/Alu 1 294 (18) 2

311 29.0 4.2 3.5 gi|157734150:47734030-47745251 1599 1741 (9481) + MIR3 SINE/MIR 21 164 (44) 3

23 67.6 0.0 0.0 gi|157734150:47734030-47745251 2309 2345 (8877) + AT_rich Low_complexity 1 37 (0) 4

271 26.1 1.1 1.1 gi|157734150:47734030-47745251 2708 2796 (8426) C MIRc SINE/MIR (4) 264 176 5

11783 0.5 0.0 0.0 gi|157734150:47734030-47745251 2864 4955 (6267) C L1HS LINE/L1 (0) 6155 4065 6

10677 0.4 0.0 0.1 gi|157734150:47734030-47745251 4952 6185 (5037) + L1HS LINE/L1 2780 4012 (2134) 6 *

**364 14.5 0.0 5.8 gi|157734150:47734030-47745251 6223 6295 (4927) C L1MD LINE/L1 (1860) 4286 4218 7**

**222 15.3 1.6 3.3 gi|157734150:47734030-47745251 6333 6393 (4829) + CT-rich Low_complexity 121 180 (0) 8 (D)**

**2777 17.6 4.4 0.0 gi|157734150:47734030-47745251 6395 7008 (4214) C L1MD LINE/L1 (1951) 4195 3540 7**

913 22.5 7.9 6.9 gi|157734150:47734030-47745251 7015 7344 (3878) C L1ME2 LINE/L1 (164) 6000 5668 9

2318 11.4 0.0 0.3 gi|157734150:47734030-47745251 7348 7654 (3568) C AluSx SINE/Alu (3) 309 4 10

994 20.0 1.2 0.0 gi|157734150:47734030-47745251 7658 7817 (3405) C AluSx SINE/Alu (15) 297 136 11

314 35.9 1.7 2.5 gi|157734150:47734030-47745251 9103 9222 (2000) C MER5A DNA/hAT-Charlie (3) 186 68 12

1853 14.8 3.2 0.0 gi|157734150:47734030-47745251 9261 9538 (1684) C AluJb SINE/Alu (3) 309 23 13

185 7.7 2.6 0.0 gi|157734150:47734030-47745251 9539 9572 (1650) + (TTTG)n Simple_repeat 2 36 (0) 14

606 32.4 8.1 0.9 gi|157734150:47734030-47745251 9696 9904 (1318) C MIRb SINE/MIR (43) 225 2 15

________________________________________________________________________________________________________________________________

L1HS_5_80 Reference human genome OCCUPIED

40 0.0 0.0 0.0 chr5 135769370 135769409 (45088457) + AT_rich Low_complexity 1 40 (0) 193

2897 11.6 7.9 5.6 chr5 135770157 135770747 (45087119) C MER4B LTR/ERV1 (7) 893 1 194

487 32.0 5.9 6.7 chr5 135770949 135771186 (45086680) + MIRb SINE/MIR 3 238 (30) 195

992 19.9 5.7 3.1 chr5 135771191 135771418 (45086448) C MIR SINE/MIR (5) 257 24 196

259 40.0 4.3 0.0 chr5 135772467 135772581 (45085285) + L2 LINE/L2 3231 3350 (28) 197

426 25.7 8.6 0.0 chr5 135772600 135772704 (45085162) + MER81 DNA/AcHobo 1 114 (0) 198

4291 1.0 0.0 2.4 chr5 135773770 135774032 (45083834) C L1P1 LINE/L1 (413) 5742 5491 199

2853 1.3 0.0 0.0 chr5 135774033 135774342 (45083524) C AluYa5 SINE/Alu (0) 310 1 200

4291 0.7 0.0 0.8 chr5 135774343 135774615 (45083251) C L1P1 LINE/L1 (665) 5490 5218 199

**4221 0.7 0.0 0.0 chr5 135774616 135775060 (45082806) + L1HS LINE/L1 5710 6154 (1) 201 (L1HS_5_80)**

23 0.0 0.0 0.0 chr5 135775061 135775083 (45082783) + AT_rich Low_complexity 1 23 (0) 202

23 0.0 0.0 0.0 chr5 135775362 135775384 (45082482) + AT_rich Low_complexity 1 23 (0) 203

201 14.7 0.0 0.0 chr5 135775449 135775482 (45082384) + (TTC)n Simple_repeat 1 34 (0) 204

858 10.3 0.0 0.0 chr5 135775483 135775598 (45082268) C AluSg/x SINE/Alu (0) 312 197 205

196 18.6 4.7 0.0 chr5 135775632 135775674 (45082192) + L2 LINE/L2 3120 3164 (214) 206

232 40.5 1.8 3.6 chr5 135776410 135776632 (45081234) C MIRb SINE/MIR (46) 222 4 207

207 0.0 0.0 0.0 chr5 135777117 135777139 (45080727) + (TTTTTG)n Simple_repeat 2 24 (0) 208

661 17.8 9.5 0.6 chr5 135777311 135777468 (45080398) C Charlie4 DNA/MER1_type (16) 1945 1774 209

579 9.0 0.0 0.0 chr5 135777469 135777546 (45080320) + LTR5_Hs LTR/ERVK 1 78 (890) 210

Ortholog in HuRef

2381 12.9 6.1 4.4 gi|157734151:130929868-130940065 545 972 (9226) C MER4D1 LTR/ERV1 (7) 893 459 1

761 15.9 2.7 5.0 gi|157734151:130929868-130940065 951 1135 (9063) C MER4D1 LTR/ERV1 (719) 181 1 1 *

479 32.0 5.9 6.8 gi|157734151:130929868-130940065 1337 1574 (8624) + MIRb SINE/MIR 3 238 (30) 2

982 19.9 5.7 3.0 gi|157734151:130929868-130940065 1579 1806 (8392) C MIR SINE/MIR (5) 257 24 3

337 36.5 4.3 0.0 gi|157734151:130929868-130940065 2855 2969 (7229) + L2b LINE/L2 3228 3347 (28) 4

420 24.8 8.6 0.0 gi|157734151:130929868-130940065 2988 3092 (7106) + MER81 DNA/hAT-Blackjack 1 114 (0) 5

190 32.2 6.4 11.1 gi|157734151:130929868-130940065 3717 3999 (6199) C L2c LINE/L2 (175) 3212 2942 6

4297 1.0 0.0 2.2 gi|157734151:130929868-130940065 4158 4419 (5779) C L1P1 LINE/L1 (413) 5742 5481 7

3059 1.3 0.0 0.0 gi|157734151:130929868-130940065 4421 4730 (5468) C AluYa5 SINE/Alu (0) 310 1 8

4297 0.7 0.0 0.7 gi|157734151:130929868-130940065 4731 5001 (5197) C L1P1 LINE/L1 (675) 5480 5220 7

**2122 0.9 0.0 0.0 gi|157734151:130929868-130940065 5022 5278 (4920) + L1HS LINE/L1 5929 6155 (0) 9 (OCCUPIED)**

212 31.3 6.4 9.5 gi|157734151:130929868-130940065 5484 5656 (4542) + L2c LINE/L2 2916 3083 (304) 10

966 9.5 0.0 0.0 gi|157734151:130929868-130940065 5678 5793 (4405) C AluSz6 SINE/Alu (0) 312 197 11

189 32.5 13.6 2.9 gi|157734151:130929868-130940065 5827 6023 (4175) + L2c LINE/L2 3117 3317 (70) 10

227 40.5 1.8 3.6 gi|157734151:130929868-130940065 6604 6826 (3372) C MIRb SINE/MIR (46) 222 4 12

207 0.0 0.0 0.0 gi|157734151:130929868-130940065 7311 7333 (2865) + (TTTTTG)n Simple_repeat 2 24 (0) 13

651 17.8 9.5 0.6 gi|157734151:130929868-130940065 7505 7662 (2536) C Charlie4a DNA/hAT-Charlie (16) 492 321 14

579 9.0 0.0 0.0 gi|157734151:130929868-130940065 7663 7740 (2458) + LTR5_Hs LTR/ERVK 1 78 (890) 15

5377 4.6 0.3 0.0 gi|157734151:130929868-130940065 7741 8387 (1811) C HERVK-int LTR/ERVK (1973) 5563 4915 16

247 15.9 2.3 0.0 gi|157734151:130929868-130940065 8388 8431 (1767) C LTR5A LTR/ERVK (988) 45 1 17

675 23.8 1.2 0.0 gi|157734151:130929868-130940065 8451 8618 (1580) C Charlie4a DNA/hAT-Charlie (315) 193 24 14

600 30.8 8.2 6.5 gi|157734151:130929868-130940065 8631 9057 (1141) + LTR33B LTR/ERVL 56 489 (14) 18

221 34.3 0.0 0.0 gi|157734151:130929868-130940065 9147 9213 (985) + MIRb SINE/MIR 156 222 (46) 19

_______________________________________________________________________________________________________________________

L1HS_5_97c Reference human genome OCCUPIED

262 31.1 10.5 4.6 chr5 172638460 172638678 (8219188) + L3 LINE/CR1 4174 4405 (84) 27

612 22.1 12.0 1.4 chr5 172638719 172638860 (8219006) C MIR SINE/MIR (0) 262 106 28

385 31.1 7.9 0.7 chr5 172638913 172639064 (8218802) + MIR SINE/MIR 2 164 (98) 29

953 20.0 0.0 4.1 chr5 172639085 172639256 (8218610) C FRAM SINE/Alu (0) 166 2 30

328 29.2 8.3 2.3 chr5 172639696 172639828 (8218038) + MIRb SINE/MIR 7 147 (121) 31

1444 16.7 0.0 0.0 chr5 172639833 172640053 (8217813) + AluJo SINE/Alu 81 301 (11) 32

324 27.6 0.9 7.9 chr5 172640073 172640186 (8217680) + MIRb SINE/MIR 163 268 (0) 31

2554 5.7 0.0 1.3 chr5 172640542 172640845 (8217021) + AluSc SINE/Alu 1 300 (9) 33

**2262 5.2 0.3 0.7 chr5 172641129 172641419 (8216447) C L1HS LINE/L1 (0) 6155 5866 34 (L1HS_5_97c)**

2353 9.6 1.0 0.7 chr5 172641907 172642211 (8215655) C AluSq SINE/Alu (7) 306 1 35

201 14.7 0.0 0.0 chr5 172642257 172642290 (8215576) + (GAA)n Simple_repeat 3 36 (0) 36

562 31.0 9.4 0.8 chr5 172642296 172642529 (8215337) C MIRb SINE/MIR (6) 262 9 37

448 13.6 1.1 1.1 chr5 172643035 172643123 (8214743) + GA-rich Low_complexity 2 90 (0) 38

329 16.2 1.5 0.0 chr5 172643970 172644037 (8213829) + (TCCA)n Simple_repeat 2 70 (0) 39

707 19.2 0.6 0.0 chr5 172644055 172644231 (8213635) + (TCCA)n Simple_repeat 3 180 (0) 39

920 15.8 0.7 0.0 chr5 172644250 172644799 (8213067) + (TCCA)n Simple_repeat 1 554 (0) 39

2223 12.9 3.9 3.3 chr5 172644831 172645192 (8212674) + THE1B LTR/MaLR 1 364 (0) 40

678 26.2 7.4 1.2 chr5 172645221 172645382 (8212484) + MIRb SINE/MIR 86 257 (11) 41

832 16.1 0.0 0.8 chr5 172645616 172645746 (8212120) C FLAM_C SINE/Alu (6) 137 8 42

204 6.7 0.0 0.0 chr5 172645887 172645916 (8211950) + (CAAAA)n Simple_repeat 1 30 (0) 43

574 27.6 11.6 1.0 chr5 172646112 172646309 (8211557) C MIRb SINE/MIR (8) 260 42 44

Ortholog in HuRef

603 22.1 12.0 1.3 gi|157734151:167798528-167808761 2545 2686 (7548) C MIR SINE/MIR (0) 262 106 7

372 27.6 2.5 6.5 gi|157734151:167798528-167808761 2739 2906 (7328) + MIR SINE/MIR 2 175 (93) 8

998 21.5 0.0 0.0 gi|157734151:167798528-167808761 2911 3082 (7152) C FRAM SINE/Alu (3) 173 2 9

313 29.2 8.3 2.1 gi|157734151:167798528-167808761 3522 3654 (6580) + MIRb SINE/MIR 7 147 (121) 10

1370 18.1 0.0 0.0 gi|157734151:167798528-167808761 3659 3879 (6355) + AluJo SINE/Alu 81 301 (11) 11

307 28.8 0.9 8.5 gi|157734151:167798528-167808761 3899 4012 (6222) + MIRb SINE/MIR 163 268 (0) 10

2490 5.7 0.0 1.4 gi|157734151:167798528-167808761 4368 4667 (5567) + AluSc SINE/Alu 1 296 (13) 12

**2264 1.9 0.0 0.4 gi|157734151:167798528-167808761 4952 5213 (5021) C L1HS LINE/L1 (0) 6155 5895 13 (OCCUPIED)**

357 26.9 4.8 2.3 gi|157734151:167798528-167808761 5318 5467 (4767) C X7B_LINE LINE/CR1 (97) 170 18 14

2312 9.6 1.0 0.7 gi|157734151:167798528-167808761 5701 6002 (4232) C AluSq SINE/Alu (9) 304 2 15

201 14.7 0.0 0.0 gi|157734151:167798528-167808761 6049 6082 (4152) + (GAA)n Simple_repeat 3 36 (0) 16

551 31.0 9.4 0.8 gi|157734151:167798528-167808761 6088 6321 (3913) C MIRb SINE/MIR (6) 262 9 17

244 22.5 0.0 2.0 gi|157734151:167798528-167808761 6828 6877 (3357) + G-rich Low_complexity 11 59 (0) 18

381 2.2 0.0 0.0 gi|157734151:167798528-167808761 6878 6923 (3311) + (GA)n Simple_repeat 1 46 (0) 19

734 20.4 0.7 0.0 gi|157734151:167798528-167808761 7764 8150 (2084) + (TCCA)n Simple_repeat 1 389 (0) 20

394 27.9 0.8 0.0 gi|157734151:167798528-167808761 8154 8421 (1813) + (CCCA)n Simple_repeat 1 270 (0) 21

824 15.7 0.6 0.0 gi|157734151:167798528-167808761 8434 8599 (1635) + (TCCA)n Simple_repeat 2 168 (0) 22

2191 12.8 3.9 3.3 gi|157734151:167798528-167808761 8631 8992 (1242) + THE1B LTR/ERVL-MaLR 1 364 (0) 23

675 25.6 7.4 1.2 gi|157734151:167798528-167808761 9021 9182 (1052) + MIRb SINE/MIR 86 257 (11) 24

844 16.5 0.0 0.8 gi|157734151:167798528-167808761 9414 9546 (688) C FLAM_C SINE/Alu (4) 139 8 25

204 6.7 0.0 0.0 gi|157734151:167798528-167808761 9687 9716 (518) + (CAAAA)n Simple_repeat 1 30 (0) 26

560 27.5 11.6 0.9 gi|157734151:167798528-167808761 9912 10109 (125) C MIRb SINE/MIR (8) 260 42 27

_________________________________________________________________________________

L1HS_9_49c M_INTER_RMD Reference human genome

201 15.4 0.0 0.0 chr9 140191494 140191532 (81720) + GA-rich Low_complexity 3 41 (0) 423

217 33.6 0.0 0.0 chr9 140193106 140193218 (80034) + C-rich Low_complexity 2 114 (0) 424

653 21.8 2.6 0.0 chr9 140195853 140196008 (77244) C L1MB4 LINE/L1 (2) 6178 6019 425

250 28.6 6.6 7.9 chr9 140196025 140196252 (77000) + L1M5 LINE/L1 5126 5350 (796) 426

4193 21.7 8.4 1.9 chr9 140196285 140198193 (75059) C L1MB3 LINE/L1 (1) 6182 4145 427

**15436 0.6 0.0 0.0 chr9 140198198 140200747 (72505) C L1HS LINE/L1 (0) 6155 3606 428 (L1HS_9_49c) (R12)**

3882 21.4 5.8 5.0 chr9 140200738 140202004 (71248) C L1MB3 LINE/L1 (1985) 4155 2879 427

6374 5.7 5.8 1.6 chr9 140203286 140203545 (69707) C L1PA7 LINE/L1 (5) 6149 5895 429

2107 13.1 0.0 0.3 chr9 140203546 140203859 (69393) C AluSp SINE/Alu (0) 313 1 430

11589 7.3 2.3 0.7 chr9 140203860 140205923 (67329) C L1PA7 LINE/L1 (260) 5894 3784 429

6916 20.2 4.7 2.8 chr9 140205926 140207780 (65472) C L1P3 LINE/L1 (4647) 1836 10 431

Ortholog in HuRef

610 21.8 2.6 0.0 gi|157734174:110541137-110554118 2607 2762 (10220) C L1MB4 LINE/L1 (2) 6178 6019 1

422 30.3 4.4 5.8 gi|157734174:110541137-110554118 2779 3006 (9976) + L1M5 LINE/L1 5126 5350 (796) 2

2124 21.6 6.9 1.2 gi|157734174:110541137-110554118 3082 4911 (8071) C L1MB3 LINE/L1 (75) 6108 4181 3

**6922 1.3 0.0 0.0 gi|157734174:110541137-110554118 4928 5785 (7197) C L1HS LINE/L1 (0) 6155 5322 4 (R2)**

**15010 0.6 0.0 0.0 gi|157734174:110541137-110554118 5806 8031 (4951) C L1HS LINE/L1 (324) 5831 3606 5 (R1)**

3604 21.6 4.9 4.1 gi|157734174:110541137-110554118 8022 9270 (3712) C L1MB3 LINE/L1 (1985) 4155 2898 3 *

3597 5.9 3.6 2.7 gi|157734174:110541137-110554118 10571 10830 (2152) C L1PA7 LINE/L1 (5) 6149 5890 6

2272 13.1 0.0 0.3 gi|157734174:110541137-110554118 10831 11144 (1838) C AluSp SINE/Alu (0) 313 1 7

9371 7.1 2.9 0.7 gi|157734174:110541137-110554118 11145 12982 (0) C L1PA7 LINE/L1 (265) 5889 4010 6

______________________________________________________________________________________

L1HS_11_30c Reference human genome OCCUPIED

679 33.5 10.4 5.3 chr11 41041361 41042133 (93410251) C L2 LINE/L2 (18) 3401 2562 525

306 20.6 0.0 1.4 chr11 41042697 41042765 (93409619) + MSTA LTR/MaLR 2 69 (359) 526

21581 14.9 2.1 5.8 chr11 41042766 41046236 (93406148) + L1PA8 LINE/L1 22 3376 (2955) 527

2085 12.9 0.6 2.5 chr11 41046237 41046553 (93405831) + AluSx SINE/Alu 1 311 (1) 528

21581 10.1 1.3 1.4 chr11 41046554 41049554 (93402830) + L1PA8 LINE/L1 3377 6356 (1) 527

5051 13.3 18.2 0.0 chr11 41049576 41050497 (93401887) + Tigger1 DNA/MER2_type 1149 2238 (180) 529

1190 18.9 8.1 6.2 chr11 41050507 41050827 (93401557) + THE1D LTR/MaLR 55 381 (0) 526

1845 19.8 5.4 4.4 chr11 41051235 41051662 (93400722) C MSTB1 LTR/MaLR (0) 432 1 530

243 6.1 0.0 0.0 chr11 41053136 41053168 (93399216) + (T)n Simple_repeat 1 33 (0) 531

**742 0.0 0.0 0.0 chr11 41053176 41053262 (93399122) C L1HS LINE/L1 (0) 6155 6069 532 (L1HS_11_30c)**

2148 31.8 8.4 5.8 chr11 41053605 41054440 (93397944) + L2 LINE/L2 1669 2529 (890) 533

1487 17.9 18.7 4.3 chr11 41054441 41054814 (93397570) C MSTA LTR/MaLR (0) 428 1 534

3201 31.6 7.0 3.7 chr11 41054815 41056492 (93395892) C MLT-int LTR/MaLR (0) 1735 2 535

1828 17.6 12.5 1.0 chr11 41056495 41056878 (93395506) C MSTA LTR/MaLR (0) 428 1 536

2148 31.9 7.6 4.5 chr11 41056879 41057288 (93395096) + L2 LINE/L2 2530 2955 (464) 533

1424 13.6 3.6 0.9 chr11 41057800 41058022 (93394362) + MER30 DNA/MER1_type 2 230 (0) 537

2356 9.7 0.0 0.3 chr11 41058469 41058778 (93393606) C AluSp SINE/Alu (4) 309 1 538

222 20.4 0.0 0.0 chr11 41059841 41059884 (93392500) + (CA)n Simple_repeat 2 45 (0) 539

23 0.0 0.0 0.0 chr11 41059899 41059921 (93392463) + AT_rich Low_complexity 1 23 (0) 540

Ortholog in HuRef

6068 10.0 1.7 0.1 gi|157704452:40799169-40809234 1 1305 (8761) + L1PA8 LINE/L1 4847 6171 (1) 1

740 15.6 10.6 0.0 gi|157704452:40799169-40809234 1327 1467 (8599) + Tigger1 DNA/TcMar-Tigger 1149 1304 (1114) 2 *

5516 14.1 3.5 0.0 gi|157704452:40799169-40809234 1446 2334 (7732) + Tigger1 DNA/TcMar-Tigger 1317 2236 (182) 3

1171 19.6 8.1 6.1 gi|157704452:40799169-40809234 2346 2666 (7400) + THE1D LTR/ERVL-MaLR 55 381 (0) 4

511 28.3 0.6 2.9 gi|157704452:40799169-40809234 2884 3061 (7005) + Tigger9a DNA/TcMar-Tigger 558 731 (1) 5

1847 19.6 5.4 4.4 gi|157704452:40799169-40809234 3074 3501 (6565) C MSTB1 LTR/ERVL-MaLR (0) 432 1 6

294 8.7 0.0 0.0 gi|157704452:40799169-40809234 4975 5020 (5046) + (T)n Simple_repeat 1 46 (0) 7

**722 1.1 0.0 0.0 gi|157704452:40799169-40809234 5029 5115 (4951) C L1HS LINE/L1 (0) 6155 6069 8 (OCCUPIED)**

1131 31.4 6.4 4.6 gi|157704452:40799169-40809234 5458 6292 (3774) + L2 LINE/L2 1669 2523 (896) 9

1151 21.6 6.7 4.7 gi|157704452:40799169-40809234 6295 6668 (3398) C MSTA LTR/ERVL-MaLR (0) 381 1 10

7039 17.8 1.1 0.9 gi|157704452:40799169-40809234 6669 8195 (1871) C MSTA-int LTR/ERVL-MaLR (0) 1580 96 10

1055 13.0 0.0 0.0 gi|157704452:40799169-40809234 8193 8346 (1720) C MSTA-int LTR/ERVL-MaLR (1424) 156 3 10 *

1806 17.4 12.5 0.9 gi|157704452:40799169-40809234 8349 8732 (1334) C MSTA LTR/ERVL-MaLR (0) 428 1 10

912 33.1 4.7 1.9 gi|157704452:40799169-40809234 8740 9142 (924) + L2 LINE/L2 2545 2958 (461) 9

290 35.3 7.6 1.5 gi|157704452:40799169-40809234 9373 9622 (444) + LTR85a LTR/Gypsy? 289 553 (149) 11

1417 13.6 3.6 0.9 gi|157704452:40799169-40809234 9654 9876 (190) + MER30 DNA/hAT-Charlie 2 230 (0) 12

_______________________________________________________________________________________

L1HS_11_57 Reference human genome ?????

667 10.9 3.4 2.8 chr11 114213853 114214031 (20238353) + (TA)n Simple_repeat 1 180 (0) 336

347 26.0 3.7 6.6 chr11 114214169 114214304 (20238080) + L1ME4a LINE/L1 5737 5868 (253) 329

467 35.3 10.6 5.1 chr11 114214427 114214859 (20237525) C L2 LINE/L2 (4) 3374 2918 337

400 25.7 15.1 5.9 chr11 114214958 114215555 (20236829) C L1MEd LINE/L1 (5221) 1236 698 338

17473 1.2 0.2 0.0 chr11 114215715 114218894 (20233490) C L1PA2 LINE/L1 (0) 6193 3122 339

**8866 1.6 0.2 0.0 chr11 114218890 114220188 (20232196) + L1HS LINE/L1 1715 3015 (3008) 340 (R12)**

644 28.6 9.4 5.0 chr11 114220681 114221063 (20231321) C L2 LINE/L2 (541) 2878 2479 337

324 30.1 10.3 5.8 chr11 114221385 114221539 (20230845) + L2 LINE/L2 3212 3373 (5) 341

213 9.4 0.0 0.0 chr11 114223336 114223367 (20229017) + (CA)n Simple_repeat 2 33 (0) 342

248 25.7 1.3 1.3 chr11 114223516 114223590 (20228794) + GA-rich Low_complexity 2 76 (0) 343

324 25.9 0.0 1.8 chr11 114224286 114224450 (20227934) + GA-rich Low_complexity 3 164 (0) 344

709 19.4 18.0 4.1 chr11 114224553 114224746 (20227638) + MER58A DNA/MER1_type 4 224 (0) 345

2302 11.9 0.0 0.0 chr11 114224902 114225213 (20227171) C AluSx SINE/Alu (0) 312 1 346

290 16.0 0.0 0.0 chr11 114226029 114226078 (20226306) C MLT1H2 LTR/MaLR (331) 153 104 347

24 0.0 0.0 0.0 chr11 114226909 114226932 (20225452) + AT_rich Low_complexity 1 24 (0) 348

207 22.0 0.0 0.0 chr11 114227225 114227274 (20225110) + CT-rich Low_complexity 4 53 (0) 349

Ortholog in HuRef

432 9.8 0.9 4.8 gi|157704452:110637712-110655588 14 120 (17757) + (TA)n Simple_repeat 2 104 (0) 1

307 27.7 1.5 4.5 gi|157704452:110637712-110655588 238 373 (17504) + L1ME4a LINE/L1 5740 5871 (253) 2

490 36.1 8.9 5.7 gi|157704452:110637712-110655588 496 924 (16953) C L2b LINE/L2 (4) 3371 2930 3

565 25.6 11.8 1.5 gi|157704452:110637712-110655588 1025 1450 (16427) C L1MEg LINE/L1 (4841) 1243 769 4

23 34.8 0.0 0.0 gi|157704452:110637712-110655588 1787 1809 (16068) + AT_rich Low_complexity 1 23 (0) 5

16342 1.2 0.2 0.0 gi|157704452:110637712-110655588 1815 4546 (13331) C L1PA2 LINE/L1 (0) 6155 3419 6

721 1.2 0.0 0.0 gi|157704452:110637712-110655588 4545 4629 (13248) + L1P1 LINE/L1 2211 2295 (3851) 7 *

2429 0.7 0.0 0.0 gi|157704452:110637712-110655588 4650 4956 (12921) C L1PA2 LINE/L1 (2718) 3428 3122 6 *

**3019 1.6 0.0 0.0 gi|157704452:110637712-110655588 4952 5323 (12554) + L1HS LINE/L1 1715 2086 (3946) 8 * (R1)**

**5939 2.7 0.0 0.0 gi|157704452:110637712-110655588 5316 6049 (11828) + L1HS LINE/L1 353 1086 (4946) 9**

**10474 1.0 0.3 0.1 gi|157704452:110637712-110655588 6070 7387 (10490) C L1P1 LINE/L1 (705) 5450 4130 10**

**13520 1.6 0.1 0.0 gi|157704452:110637712-110655588 7408 9926 (7951) + L1HS LINE/L1 618 3138 (3008) 11 (R2)**

515 28.1 8.1 5.0 gi|157704452:110637712-110655588 10419 10801 (7076) C L2b LINE/L2 (541) 2878 2479 3

321 33.4 2.9 6.0 gi|157704452:110637712-110655588 11141 11277 (6600) + L2b LINE/L2 3238 3370 (5) 12

231 8.8 0.0 0.0 gi|157704452:110637712-110655588 13074 13107 (4770) + (CA)n Simple_repeat 2 35 (0) 13

248 25.7 1.3 1.3 gi|157704452:110637712-110655588 13256 13330 (4547) + GA-rich Low_complexity 2 76 (0) 14

200 26.1 4.6 0.0 gi|157704452:110637712-110655588 13962 14026 (3851) + L2b LINE/L2 3307 3374 (1) 15

282 25.4 0.0 2.4 gi|157704452:110637712-110655588 14062 14190 (3687) + A-rich Low_complexity 3 128 (0) 16

595 19.3 18.0 3.6 gi|157704452:110637712-110655588 14293 14486 (3391) + MER58A DNA/hAT-Charlie 4 224 (0) 17

2336 11.9 0.0 0.0 gi|157704452:110637712-110655588 14642 14952 (2925) C AluSx SINE/Alu (0) 312 2 18

205 35.0 11.3 6.8 gi|157704452:110637712-110655588 15140 15280 (2597) + MIRc SINE/MIR 4 150 (118) 19

244 20.0 0.0 0.0 gi|157704452:110637712-110655588 15769 15818 (2059) C MLT1H2 LTR/ERVL-MaLR (336) 153 104 20

24 58.3 0.0 0.0 gi|157704452:110637712-110655588 16649 16672 (1205) + AT_rich Low_complexity 1 24 (0) 21

207 22.0 0.0 0.0 gi|157704452:110637712-110655588 16965 17014 (863) + C-rich Low_complexity 128 177 (0) 22

924 22.8 0.9 1.4 gi|157704452:110637712-110655588 17193 17406 (471) + MER20 DNA/hAT-Charlie 7 219 (0) 23

255 20.8 0.0 0.0 gi|157704452:110637712-110655588 17684 17736 (141) + (TCCA)n Simple_repeat 1 53 (0) 24

__________________________________________________________________________

L1HS_13_34c Reference human genome M_DISRUPTED

2759 19.2 6.3 3.2 chr13 104823756 104824417 (9318563) + L1MB8 LINE/L1 5490 6172 (6) 134

24 3.2 0.0 0.0 chr13 104825989 104826019 (9316961) + AT_rich Low_complexity 1 31 (0) 135

266 29.8 5.3 0.8 chr13 104826083 104826214 (9316766) + L1M5 LINE/L1 5543 5680 (441) 136

261 35.6 6.6 2.0 chr13 104826885 104827036 (9315944) + MLT1J LTR/MaLR 246 404 (107) 137

22 0.0 0.0 0.0 chr13 104828400 104828421 (9314559) + AT_rich Low_complexity 1 22 (0) 138

1469 18.9 5.0 1.1 chr13 104828422 104828699 (9314281) C AluJb SINE/Alu (23) 289 1 139

907 30.1 10.5 4.8 chr13 104829051 104829546 (9313434) C L3 LINE/CR1 (174) 4315 3792 140

23 0.0 0.0 0.0 chr13 104830124 104830146 (9312834) + AT_rich Low_complexity 1 23 (0) 141

1903 15.4 17.4 1.4 chr13 104830508 104830875 (9312105) C MSTA LTR/MaLR (0) 428 2 142

**7426 4.9 0.0 1.3 chr13 104832362 104832716 (9310264) C L1HS LINE/L1 (5099) 933 589 143 (R2)**

**2741 1.0 0.3 0.0 chr13 104832717 104833016 (9309964) C AluYg6 SINE/Alu (10) 301 1 144 (D)**

**7426 4.9 0.0 1.3 chr13 104833017 104833602 (9309378) C L1HS LINE/L1 (5444) 588 4 143 (R1)**

**27369 2.6 0.3 0.0 chr13 104833603 104838696 (9304284) + L1PA3 LINE/L1 926 6031 (1) 145 (Remaining part of twin pried L1)**

24 6.7 0.0 0.0 chr13 104838792 104838836 (9304144) + AT_rich Low_complexity 1 45 (0) 146

21 0.0 0.0 0.0 chr13 104840075 104840095 (9302885) + AT_rich Low_complexity 1 21 (0) 147

253 38.0 0.6 3.7 chr13 104840181 104840344 (9302636) C MER5B DNA/MER1_type (16) 162 4 148

376 32.2 5.8 3.2 chr13 104842455 104842643 (9300337) + ERVL LTR/ERVL 1772 1965 (3792) 149

1561 25.1 4.3 0.0 chr13 104842823 104842877 (9300103) + MER97c DNA/MER1_type? 184 240 (850) 150

408 9.7 0.0 0.0 chr13 104842878 104842939 (9300041) + (TA)n Simple_repeat 1 62 (0) 151

Ortholog in HuRef

22 59.1 0.0 0.0 gi|157704454:86620367-86630664 990 1011 (9287) + AT_rich Low_complexity 1 22 (0) 1

1549 18.7 5.0 1.0 gi|157704454:86620367-86630664 1012 1289 (9009) C AluJb SINE/Alu (23) 289 1 2

618 30.6 10.3 7.1 gi|157704454:86620367-86630664 1641 2136 (8162) C L3 LINE/CR1 (174) 4315 3792 3

23 43.5 0.0 0.0 gi|157704454:86620367-86630664 2714 2736 (7562) + AT_rich Low_complexity 1 23 (0) 4

1844 15.4 17.4 1.2 gi|157704454:86620367-86630664 3098 3465 (6833) C MSTA LTR/ERVL-MaLR (0) 428 2 5

**7131 4.8 0.0 0.4 gi|157704454:86620367-86630664 4952 5885 (4413) C L1HS LINE/L1 (5099) 1056 127 6 (R)**

**26173 2.6 0.3 0.0 gi|157704454:86620367-86630664 5886 10298 (0) + L1HS LINE/L1 1049 5473 (682) 6 (Remaining part of twin primed L1)**

__________________________________________________________________________________

L1HS_14_20 M_INTER_RMD

1600 21.6 7.1 9.4 chr14 43126088 43126553 (63242032) C MER76 LTR/ERVL (235) 456 2 78

1264 30.9 10.9 4.2 chr14 43126610 43127935 (63240650) C L1M5 LINE/L1 (759) 5387 3974 80

21 0.0 0.0 0.0 chr14 43128571 43128591 (63239994) + AT_rich Low_complexity 1 21 (0) 81

25 0.0 0.0 0.0 chr14 43131277 43131301 (63237284) + AT_rich Low_complexity 1 25 (0) 82

2241 13.3 1.9 0.8 chr14 43131302 43131673 (63236912) C L1PB1 LINE/L1 (0) 6151 5776 83

453 18.9 0.0 0.0 chr14 43131674 43131768 (63236817) + (TA)n Simple_repeat 1 95 (0) 84

10167 11.8 2.1 5.3 chr14 43131769 43134088 (63234497) C L1PB1 LINE/L1 (350) 5801 3567 83

2334 25.7 1.5 1.4 chr14 43136175 43136849 (63231736) C MER89-int LTR/ERV1 (728) 6073 5358 85

**18477 3.3 0.3 0.0 chr14 43136854 43141041 (63227544) + L1HS LINE/L1 1 4202 (1821) 86 (L1HS_14_20) (R12)**

7871 1.8 0.0 0.0 chr14 43141038 43142354 (63226231) + L1PA2 LINE/L1 4837 6153 (2) 87

1701 31.4 7.4 1.4 chr14 43142356 43143019 (63225566) C MER89-int LTR/ERV1 (1438) 5363 4660 85

950 29.7 3.7 2.9 chr14 43143030 43143404 (63225181) C MER89-int LTR/ERV1 (2254) 4547 4170 85

490 28.5 3.0 2.7 chr14 43143442 43143636 (63224949) + L1MCa LINE/L1 292 712 (5606) 88

1181 26.0 8.4 3.9 chr14 43143897 43144419 (63224166) + L1MCa LINE/L1 555 1091 (4955) 88

1047 16.5 1.1 0.6 chr14 43144421 43144603 (63223982) + L1M2 LINE/L1 2 185 (6664) 89

339 29.1 0.0 0.0 chr14 43144619 43144704 (63223881) + L1M2 LINE/L1 587 672 (6177) 89

271 4.4 4.4 0.0 chr14 43145165 43145209 (63223376) + (TCTA)n Simple_repeat 4 50 (0) 90

Ortholog in HuRef

6123 11.9 2.8 1.6 gi|157704455:24171539-24185952 1 1360 (13054) C L1PB LINE/L1 (476) 5675 4302 1

354 16.7 2.8 0.0 gi|157704455:24171539-24185952 1369 1440 (12974) + L1PB LINE/L1 3449 3522 (2624) 2

4554 12.1 1.1 1.4 gi|157704455:24171539-24185952 1456 2186 (12228) C L1PB LINE/L1 (1851) 4295 3567 1

2337 25.7 7.4 1.3 gi|157704455:24171539-24185952 4273 4947 (9467) C MER89-int LTR/ERV1 (728) 6073 5358 3

**18173 3.3 0.3 0.1 gi|157704455:24171539-24185952 4952 9106 (5308) + L1HS LINE/L1 124 4291 (1855) 4 (R1)**

**2926 2.1 0.0 0.0 gi|157704455:24171539-24185952 9127 9463 (4951) + L1PA2 LINE/L1 3989 4325 (1821) 5 * (R2)**

7871 1.8 0.0 0.0 gi|157704455:24171539-24185952 9460 10776 (3638) + L1PA2 LINE/L1 4837 6153 (2) 5

938 31.7 7.4 1.3 gi|157704455:24171539-24185952 10778 11441 (2973) C MER89-int LTR/ERV1 (1438) 5363 4660 3

941 29.7 3.7 2.9 gi|157704455:24171539-24185952 11452 11826 (2588) C MER89-int LTR/ERV1 (2254) 4547 4170 3

466 30.4 1.5 8.8 gi|157704455:24171539-24185952 11864 12058 (2356) + L1MCb LINE/L1 908 1089 (6039) 6

1156 26.6 7.9 3.2 gi|157704455:24171539-24185952 12319 12841 (1573) + L1MCb LINE/L1 932 1468 (4955) 6

1046 16.5 1.1 0.5 gi|157704455:24171539-24185952 12843 13025 (1389) + L1M2 LINE/L1 2 185 (6664) 7

339 29.1 0.0 0.0 gi|157704455:24171539-24185952 13041 13126 (1288) + L1M2 LINE/L1 587 672 (6177) 7

239 5.4 2.7 0.0 gi|157704455:24171539-24185952 13595 13631 (783) + (TCTA)n Simple_repeat 4 41 (0) 8

2745 6.9 0.3 0.0 gi|157704455:24171539-24185952 13665 14027 (387) + L1PA8 LINE/L1 5805 6168 (4) 9

36 81.7 0.0 0.0 gi|157704455:24171539-24185952 14337 14407 (7) + AT_rich Low_complexity 1 71 (0) 10

________________________________________________________________________________

L1HS_18_2c M_DISRUPTED

2203 10.6 0.7 0.0 chr18 824716 825007 (75292146) + AluSq SINE/Alu 6 299 (14) 605

5028 15.3 2.3 5.0 chr18 825008 825051 (75292102) C MER49 LTR/ERV1 (845) 78 35 603

347 13.9 9.7 0.0 chr18 825053 825124 (75292029) C L1MB2 LINE/L1 (213) 5958 5880 606

1670 17.7 3.8 0.0 chr18 825140 825427 (75291726) C AluJb SINE/Alu (13) 299 1 607

194 27.9 0.0 0.0 chr18 825428 825470 (75291683) + MER5B DNA/MER1_type 136 178 (0) 608

1322 3.6 0.0 1.2 chr18 825474 825639 (75291514) + L1PA2 LINE/L1 5992 6155 (0) 609

2039 13.2 3.1 0.0 chr18 825655 825942 (75291211) + AluSx SINE/Alu 1 297 (15) 610

220 30.1 0.0 1.4 chr18 825944 826017 (75291136) C MER5B DNA/MER1_type (32) 146 74 611

2026 14.3 1.0 0.0 chr18 826409 826715 (75290438) C AluSq SINE/Alu (3) 310 1 612

**4690 1.7 0.0 1.9 chr18 826734 827111 (75290042) C L1HS LINE/L1 (1) 6154 5785 613 (R1) L1HS_18_2c**

**2903 0.7 0.0 0.0 chr18 827112 827418 (75289735) C AluYb8 SINE/Alu (11) 307 1 614 (D)**

**4690 1.7 0.0 1.9 chr18 827419 827581 (75289572) C L1HS LINE/L1 (371) 5784 5623 613 (R2)**

817 1.1 0.0 0.0 chr18 827582 827670 (75289483) + L1P2 LINE/L1 5523 5611 (544) 615

391 30.0 4.6 0.0 chr18 827787 827916 (75289237) + MIR SINE/MIR 110 245 (17) 616

1979 12.4 3.9 2.6 chr18 828425 828731 (75288422) C AluSx SINE/Alu (1) 311 1 617

2216 10.6 7.2 2.6 chr18 828988 829335 (75287818) C THE1B LTR/MaLR (0) 364 1 618

1088 20.0 0.4 6.0 chr18 830458 830691 (75286462) + AluJo SINE/Alu 1 221 (91) 619

Ortholog in HuRef

2210 10.6 0.7 0.0 gi|157715044:792004-802325 2922 3213 (7109) + AluSq2 SINE/Alu 6 299 (13) 7

5021 15.4 2.3 5.1 gi|157715044:792004-802325 3214 3257 (7065) C MER49 LTR/ERV1 (853) 70 35 5

342 13.9 9.7 0.0 gi|157715044:792004-802325 3259 3330 (6992) C L1MB2 LINE/L1 (213) 5958 5880 8

1760 17.2 3.8 0.0 gi|157715044:792004-802325 3346 3635 (6687) C AluJb SINE/Alu (11) 301 1 9

222 25.6 0.0 0.0 gi|157715044:792004-802325 3636 3678 (6644) C MER5B DNA/hAT-Charlie (135) 43 1 10

1342 3.1 0.6 0.0 gi|157715044:792004-802325 3682 3844 (6478) + L1PA2 LINE/L1 5992 6155 (0) 11

2037 13.2 3.1 0.0 gi|157715044:792004-802325 3862 4149 (6173) + AluSz SINE/Alu 1 297 (15) 12

228 30.1 0.0 1.4 gi|157715044:792004-802325 4151 4224 (6098) C MER5B DNA/hAT-Charlie (32) 146 74 13

199 33.1 4.5 4.5 gi|157715044:792004-802325 4335 4467 (5855) C L2 LINE/L2 (1327) 2092 1960 14

2185 13.6 0.0 0.0 gi|157715044:792004-802325 4616 4925 (5397) C AluSq2 SINE/Alu (2) 310 1 15

**4770 1.5 0.0 0.0 gi|157715044:792004-802325 4951 5483 (4839) C L1HS LINE/L1 (0) 6155 5623 16 (R)**

817 1.1 0.0 0.0 gi|157715044:792004-802325 5484 5572 (4750) + L1HS LINE/L1 5523 5611 (544) 16

189 37.5 0.0 1.8 gi|157715044:792004-802325 5620 5676 (4646) + MIR SINE/MIR 6 61 (201) 17

437 30.0 4.6 0.0 gi|157715044:792004-802325 5689 5818 (4504) + MIR SINE/MIR 110 245 (17) 17

2121 11.4 4.0 0.3 gi|157715044:792004-802325 6327 6625 (3697) C AluSz SINE/Alu (2) 310 1 18

2161 11.2 7.2 2.5 gi|157715044:792004-802325 6882 7229 (3093) C THE1B LTR/ERVL-MaLR (0) 364 1 19

191 31.6 5.1 0.8 gi|157715044:792004-802325 7237 7354 (2968) + MIR3 SINE/MIR 57 179 (29) 20

1078 18.0 0.4 11.3 gi|157715044:792004-802325 8362 8606 (1716) + AluJo SINE/Alu 1 221 (91) 21

___________________________________________________________________________

L1HS_18_3c M_DISRUPTED

2203 10.6 0.7 0.0 chr18 824716 825007 (75292146) + AluSq SINE/Alu 6 299 (14) 605

5028 15.3 2.3 5.0 chr18 825008 825051 (75292102) C MER49 LTR/ERV1 (845) 78 35 603

347 13.9 9.7 0.0 chr18 825053 825124 (75292029) C L1MB2 LINE/L1 (213) 5958 5880 606

1670 17.7 3.8 0.0 chr18 825140 825427 (75291726) C AluJb SINE/Alu (13) 299 1 607

194 27.9 0.0 0.0 chr18 825428 825470 (75291683) + MER5B DNA/MER1_type 136 178 (0) 608

1322 3.6 0.0 1.2 chr18 825474 825639 (75291514) + L1PA2 LINE/L1 5992 6155 (0) 609

2039 13.2 3.1 0.0 chr18 825655 825942 (75291211) + AluSx SINE/Alu 1 297 (15) 610

220 30.1 0.0 1.4 chr18 825944 826017 (75291136) C MER5B DNA/MER1_type (32) 146 74 611

2026 14.3 1.0 0.0 chr18 826409 826715 (75290438) C AluSq SINE/Alu (3) 310 1 612

**4690 1.7 0.0 1.9 chr18 826734 827111 (75290042) C L1HS LINE/L1 (1) 6154 5785 613 (R1)**

**2903 0.7 0.0 0.0 chr18 827112 827418 (75289735) C AluYb8 SINE/Alu (11) 307 1 614 (D)**

**4690 1.7 0.0 1.9 chr18 827419 827581 (75289572) C L1HS LINE/L1 (371) 5784 5623 613 (R2) (L1HS_18_3c)**

817 1.1 0.0 0.0 chr18 827582 827670 (75289483) + L1P2 LINE/L1 5523 5611 (544) 615

391 30.0 4.6 0.0 chr18 827787 827916 (75289237) + MIR SINE/MIR 110 245 (17) 616

1979 12.4 3.9 2.6 chr18 828425 828731 (75288422) C AluSx SINE/Alu (1) 311 1 617

2216 10.6 7.2 2.6 chr18 828988 829335 (75287818) C THE1B LTR/MaLR (0) 364 1 618

1088 20.0 0.4 6.0 chr18 830458 830691 (75286462) + AluJo SINE/Alu 1 221 (91) 619

Ortholog in HuRef

2210 10.6 0.7 0.0 gi|157715044:792004-802325 2922 3213 (7109) + AluSq2 SINE/Alu 6 299 (13) 7

5021 15.4 2.3 5.1 gi|157715044:792004-802325 3214 3257 (7065) C MER49 LTR/ERV1 (853) 70 35 5

342 13.9 9.7 0.0 gi|157715044:792004-802325 3259 3330 (6992) C L1MB2 LINE/L1 (213) 5958 5880 8

1760 17.2 3.8 0.0 gi|157715044:792004-802325 3346 3635 (6687) C AluJb SINE/Alu (11) 301 1 9

222 25.6 0.0 0.0 gi|157715044:792004-802325 3636 3678 (6644) C MER5B DNA/hAT-Charlie (135) 43 1 10

1342 3.1 0.6 0.0 gi|157715044:792004-802325 3682 3844 (6478) + L1PA2 LINE/L1 5992 6155 (0) 11

2037 13.2 3.1 0.0 gi|157715044:792004-802325 3862 4149 (6173) + AluSz SINE/Alu 1 297 (15) 12

228 30.1 0.0 1.4 gi|157715044:792004-802325 4151 4224 (6098) C MER5B DNA/hAT-Charlie (32) 146 74 13

199 33.1 4.5 4.5 gi|157715044:792004-802325 4335 4467 (5855) C L2 LINE/L2 (1327) 2092 1960 14

2185 13.6 0.0 0.0 gi|157715044:792004-802325 4616 4925 (5397) C AluSq2 SINE/Alu (2) 310 1 15

**4770 1.5 0.0 0.0 gi|157715044:792004-802325 4951 5483 (4839) C L1HS LINE/L1 (0) 6155 5623 16 (R)**

817 1.1 0.0 0.0 gi|157715044:792004-802325 5484 5572 (4750) + L1HS LINE/L1 5523 5611 (544) 16

189 37.5 0.0 1.8 gi|157715044:792004-802325 5620 5676 (4646) + MIR SINE/MIR 6 61 (201) 17

437 30.0 4.6 0.0 gi|157715044:792004-802325 5689 5818 (4504) + MIR SINE/MIR 110 245 (17) 17

2121 11.4 4.0 0.3 gi|157715044:792004-802325 6327 6625 (3697) C AluSz SINE/Alu (2) 310 1 18

2161 11.2 7.2 2.5 gi|157715044:792004-802325 6882 7229 (3093) C THE1B LTR/ERVL-MaLR (0) 364 1 19

191 31.6 5.1 0.8 gi|157715044:792004-802325 7237 7354 (2968) + MIR3 SINE/MIR 57 179 (29) 20

1078 18.0 0.4 11.3 gi|157715044:792004-802325 8362 8606 (1716) + AluJo SINE/Alu 1 221 (91) 21

___________________________________________________________________________

L1HS_18_5c - M_INTER_RMD

330 2.6 0.0 0.0 chr18 5318410 5318448 (70798705) + (GGAA)n Simple_repeat 2 40 (0) 419

6147 10.8 0.8 1.7 chr18 5318449 5318717 (70798436) + L1PA11 LINE/L1 5909 6171 (3) 417

2197 11.0 1.0 0.6 chr18 5318800 5319110 (70798043) C AluSx SINE/Alu (0) 312 1 420

392 31.0 5.1 7.4 chr18 5319383 5319678 (70797475) + L1ME3A LINE/L1 691 979 (3875) 421

321 27.5 2.2 2.2 chr18 5319881 5320014 (70797139) + L1MC4 LINE/L1 7153 7286 (756) 422

2351 26.3 8.4 1.3 chr18 5320031 5321150 (70796003) + L1ME3A LINE/L1 3402 4582 (276) 421

2193 10.7 0.0 1.3 chr18 5321151 5321452 (70795701) + AluY SINE/Alu 1 298 (13) 423

2351 25.0 9.3 2.0 chr18 5321453 5321686 (70795467) + L1ME3A LINE/L1 4583 4849 (9) 421

435 26.2 5.3 4.6 chr18 5321723 5321874 (70795279) C MLT1L LTR/MaLR (440) 175 23 424

**12130 0.6 0.0 0.7 chr18 5322408 5324600 (70792553) C L1HS LINE/L1 (0) 6200 4026 425 (R12)**

225 32.0 0.0 3.0 chr18 5325457 5325556 (70791597) + HAL1 LINE/L1 2236 2332 (175) 426

4368 22.3 3.6 1.4 chr18 5326060 5327482 (70789671) C ERVL-D LTR/ERVL (4205) 1509 5 427

559 11.2 1.9 0.0 chr18 5327541 5327647 (70789506) + (TCTA)n Simple_repeat 2 110 (0) 428

1701 18.6 1.6 0.7 chr18 5327650 5327958 (70789195) C MLT2D LTR/ERVL (100) 314 3 427

2091 18.1 4.4 4.2 chr18 5327965 5328467 (70788686) C L1M4 LINE/L1 (746) 5400 4897 429

2199 12.7 1.5 0.3 chr18 5328468 5328792 (70788361) + MSTA LTR/MaLR 1 329 (99) 430

963 8.6 3.1 0.0 chr18 5328986 5329113 (70788040) + MSTA LTR/MaLR 297 428 (0) 430

623 18.3 0.8 0.0 chr18 5329118 5329237 (70787916) C L1M4 LINE/L1 (1250) 4896 4776 429

1604 24.5 7.9 4.2 chr18 5329246 5329765 (70787388) C LTR55 LTR/ERV (8) 540 2 431

Ortholog in HuRef

6115 10.4 0.8 1.9 gi|157715044:5291178-5304664 1 643 (12844) + L1PA11 LINE/L1 5268 5908 (266) 1

2178 13.6 0.7 1.0 gi|157715044:5291178-5304664 644 953 (12534) + AluSx SINE/Alu 2 310 (2) 2

330 2.6 0.0 0.0 gi|157715044:5291178-5304664 954 992 (12495) + (GGAA)n Simple_repeat 2 40 (0) 3

6115 10.4 0.8 1.9 gi|157715044:5291178-5304664 993 1261 (12226) + L1PA11 LINE/L1 5909 6171 (3) 1

2304 10.6 1.0 0.6 gi|157715044:5291178-5304664 1344 1654 (11833) C AluSz SINE/Alu (0) 312 1 4

321 27.5 2.2 2.2 gi|157715044:5291178-5304664 2426 2559 (10928) + L1MC4 LINE/L1 7153 7286 (756) 5

1258 30.1 3.0 0.4 gi|157715044:5291178-5304664 2576 3016 (10471) + L1ME3 LINE/L1 4714 5165 (981) 6

2190 23.8 7.5 1.6 gi|157715044:5291178-5304664 3029 3695 (9792) + L1ME3 LINE/L1 5194 5894 (268) 6

2274 10.2 0.0 1.7 gi|157715044:5291178-5304664 3696 3999 (9488) + AluSc8 SINE/Alu 1 299 (13) 7

2190 26.9 10.7 1.9 gi|157715044:5291178-5304664 4000 4244 (9243) + L1ME3 LINE/L1 5895 6172 (1) 6

438 27.9 1.8 10.3 gi|157715044:5291178-5304664 4254 4421 (9066) C MLT1L LTR/ERVL-MaLR (433) 177 23 8

495 4.5 0.0 0.0 gi|157715044:5291178-5304664 4955 5020 (8467) + (TTTTA)n Simple_repeat 4 69 (0) 9

**5991 1.3 0.3 0.0 gi|157715044:5291178-5304664 5021 5703 (7784) C L1HS LINE/L1 (16) 6139 5455 10 (R1)**

**1743 24.1 4.3 0.4 gi|157715044:5291178-5304664 5759 6266 (7221) C L1MEf LINE/L1 (5175) 1465 938 11**

**1105 23.4 4.8 0.0 gi|157715044:5291178-5304664 6281 6528 (6959) C L1MEf LINE/L1 (5640) 907 648 11**

**10488 0.2 0.0 0.0 gi|157715044:5291178-5304664 7357 8536 (4951) C L1P1 LINE/L1 (941) 5205 4026 12 (R2)**

225 32.0 0.0 3.1 gi|157715044:5291178-5304664 9395 9494 (3993) + HAL1 LINE/L1 2236 2332 (175) 13

3524 22.8 3.5 1.1 gi|157715044:5291178-5304664 9998 11420 (2067) C ERVL-E-int LTR/ERVL (4205) 1509 5 14

559 11.2 1.9 0.0 gi|157715044:5291178-5304664 11479 11585 (1902) + (TCTA)n Simple_repeat 2 110 (0) 15

1696 18.6 1.6 0.6 gi|157715044:5291178-5304664 11588 11896 (1591) C MLT2D LTR/ERVL (100) 314 3 16

2083 17.8 4.4 4.2 gi|157715044:5291178-5304664 11903 12405 (1082) C L1M4 LINE/L1 (746) 5400 4897 17

2155 12.0 0.3 0.3 gi|157715044:5291178-5304664 12406 12715 (772) + MSTA LTR/ERVL-MaLR 1 310 (118) 18

274 22.3 9.9 5.1 gi|157715044:5291178-5304664 12763 12913 (574) + L1ME2z LINE/L1 6285 6442 (2) 19

969 8.5 3.1 0.0 gi|157715044:5291178-5304664 12922 13050 (437) + MSTA LTR/ERVL-MaLR 296 428 (0) 20

624 18.3 0.8 0.0 gi|157715044:5291178-5304664 13055 13174 (313) C L1M4 LINE/L1 (1250) 4896 4776 17

551 27.5 11.8 1.7 gi|157715044:5291178-5304664 13183 13464 (23) C LTR55 LTR/ERV (8) 540 235 21

_____________________________________________________________________________________________

L1HS_18_12c OCCUPIED

1355 18.5 4.0 0.4 chr18 20155323 20155550 (55961603) C AluJb SINE/Alu (76) 236 1 593

313 19.3 0.0 0.0 chr18 20155558 20155614 (55961539) + Tigger5 DNA/MER2_type 2346 2402 (0) 594

428 33.3 1.3 1.3 chr18 20155890 20156038 (55961115) C L2 LINE/L2 (609) 2810 2662 595

2413 6.6 0.0 3.8 chr18 20156069 20156382 (55960771) C AluY SINE/Alu (9) 302 1 596

214 26.4 8.1 2.7 chr18 20156728 20156801 (55960352) + L2 LINE/L2 3341 3418 (1) 597

246 22.7 4.7 9.3 chr18 20156934 20157040 (55960113) + L1M5 LINE/L1 4519 4620 (1526) 598

993 14.5 0.0 0.0 chr18 20157233 20157370 (55959783) + AluSx SINE/Alu 1 138 (174) 599

910 7.0 0.0 0.0 chr18 20157371 20157485 (55959668) C AluSg1 SINE/Alu (194) 115 1 600

**4589 0.2 0.0 0.0 chr18 20157511 20158019 (55959134) C L1HS LINE/L1 (0) 6155 5647 601 (L1HS_18_12c)**

1325 9.8 0.0 0.0 chr18 20158023 20158195 (55958958) + AluSx SINE/Alu 119 291 (18) 599

252 0.0 0.0 0.0 chr18 20158418 20158445 (55958708) + (A)n Simple_repeat 1 28 (0) 602

23 0.0 0.0 0.0 chr18 20158561 20158583 (55958570) + AT_rich Low_complexity 1 23 (0) 603

1246 13.3 0.0 1.1 chr18 20158584 20158765 (55958388) + AluSx SINE/Alu 121 300 (12) 604

2126 10.2 0.0 0.3 chr18 20158768 20159076 (55958077) + AluSc SINE/Alu 1 308 (0) 605

1087 28.6 9.7 1.1 chr18 20159204 20159745 (55957408) + L2 LINE/L2 2827 3419 (0) 606

778 16.5 3.1 0.0 chr18 20159887 20160013 (55957140) C FLAM_C SINE/Alu (2) 131 1 607

2126 11.6 3.3 0.0 chr18 20161059 20161359 (55955794) C AluSx SINE/Alu (1) 311 1 608

2119 13.3 0.3 0.0 chr18 20161377 20161670 (55955483) C AluSx SINE/Alu (17) 295 1 609

Ortholog in HuRef

1410 18.4 4.0 0.4 gi|157715044:18754480-18764940 2962 3189 (7272) C AluJb SINE/Alu (76) 236 1 12

313 19.3 0.0 0.0 gi|157715044:18754480-18764940 3197 3253 (7208) + MER47A DNA/TcMar-Tigger 306 362 (4) 13

428 33.3 1.3 1.3 gi|157715044:18754480-18764940 3529 3677 (6784) C L2a LINE/L2 (609) 2810 2662 9

2520 6.3 0.0 4.0 gi|157715044:18754480-18764940 3708 4023 (6438) C AluY SINE/Alu (7) 304 1 14

220 25.7 7.8 2.9 gi|157715044:18754480-18764940 4333 4442 (6019) + L2a LINE/L2 3273 3425 (1) 15

242 22.8 4.7 9.8 gi|157715044:18754480-18764940 4575 4681 (5780) + L1M5 LINE/L1 4519 4620 (1526) 16

937 14.1 0.0 0.0 gi|157715044:18754480-18764940 4874 5001 (5460) + AluSz SINE/Alu 1 128 (184) 17

**4238 0.2 0.0 0.0 gi|157715044:18754480-18764940 5022 5510 (4951) C L1HS LINE/L1 (20) 6135 5647 18 (OCCUPIED)**

1354 9.2 0.6 0.0 gi|157715044:18754480-18764940 5514 5686 (4775) + AluSz SINE/Alu 121 294 (18) 17

243 0.0 0.0 0.0 gi|157715044:18754480-18764940 5909 5935 (4526) + (A)n Simple_repeat 1 27 (0) 19

22 36.4 0.0 0.0 gi|157715044:18754480-18764940 6051 6072 (4389) + AT_rich Low_complexity 1 22 (0) 20

1249 13.7 0.0 1.1 gi|157715044:18754480-18764940 6073 6254 (4207) + AluSx3 SINE/Alu 120 299 (13) 21

2215 10.2 0.0 0.3 gi|157715044:18754480-18764940 6257 6565 (3896) + AluSc SINE/Alu 1 309 (0) 22

698 28.6 8.3 5.0 gi|157715044:18754480-18764940 6712 7234 (3227) + L2a LINE/L2 2856 3426 (0) 23

808 16.5 3.1 0.0 gi|157715044:18754480-18764940 7376 7502 (2959) C FLAM_C SINE/Alu (12) 131 1 24

2175 11.3 3.3 0.0 gi|157715044:18754480-18764940 8548 8849 (1612) C AluSz SINE/Alu (0) 312 1 25

2181 12.9 0.3 0.0 gi|157715044:18754480-18764940 8867 9160 (1301) C AluSx1 SINE/Alu (17) 295 1 26

2231 7.7 1.9 8.2 gi|157715044:18754480-18764940 9489 9811 (650) C AluSc8 SINE/Alu (8) 304 1 27

306 25.2 3.0 0.0 gi|157715044:18754480-18764940 9859 9957 (504) C L1M4 LINE/L1 (1252) 4894 4793 28

399 31.9 2.2 0.7 gi|157715044:18754480-18764940 9960 10095 (366) C L2c LINE/L2 (217) 3170 3033 29

1499 16.4 3.5 0.9 gi|157715044:18754480-18764940 10144 10456 (5) + MER33 DNA/hAT-Charlie 1 321 (3) 30

________________________________________________________________________________________

L1HS_20_16c OCCUPIED

435 35.2 12.0 3.4 chr20 37855957 37856687 (24579277) C L2 LINE/L2 (58) 3361 2573 756

319 27.6 14.4 2.9 chr20 37857401 37857584 (24578380) + MIRb SINE/MIR 2 191 (71) 757

346 35.0 10.6 0.0 chr20 37858797 37858956 (24577008) C MIR3 SINE/MIR (22) 186 10 758

2060 24.9 14.9 3.7 chr20 37859874 37860639 (24575325) C MER21B LTR/ERV1 (11) 852 1 759

185 30.4 5.4 0.0 chr20 37860931 37860986 (24574978) + L2 LINE/L2 3238 3296 (82) 760

3867 19.1 3.5 3.5 chr20 37861016 37861152 (24574812) C L1MC4 LINE/L1 (0) 8042 7900 761

255 7.9 0.0 0.0 chr20 37861153 37861190 (24574774) + (CA)n Simple_repeat 2 39 (0) 762

3867 19.1 3.5 3.5 chr20 37861191 37861975 (24573989) C L1MC4 LINE/L1 (143) 7899 7120 761

**9346 2.2 0.8 0.0 chr20 37863552 37864713 (24571251) C L1HS LINE/L1 (4380) 1652 482 763**

24063 1.9 0.0 0.1 chr20 37864714 37868453 (24567511) + L1PA2 LINE/L1 2418 6155 (0) 764

25 3.1 0.0 0.0 chr20 37868896 37868927 (24567037) + AT_rich Low_complexity 1 32 (0) 765

301 28.7 10.6 0.8 chr20 37868958 37869080 (24566884) + MIRb SINE/MIR 63 197 (71) 766

350 25.5 15.3 5.3 chr20 37869162 37869389 (24566575) + MLT1E2 LTR/MaLR 2 252 (374) 767

424 5.4 0.0 0.0 chr20 37869399 37869454 (24566510) C L1P1 LINE/L1 (391) 5764 5709 768

293 0.0 0.0 2.6 chr20 37869472 37869509 (24566455) + (TAA)n Simple_repeat 3 39 (0) 769

1349 23.1 6.3 0.9 chr20 37869517 37869848 (24566116) + MLT1E2 LTR/MaLR 269 618 (8) 767

282 28.7 9.0 1.4 chr20 37869883 37870027 (24565937) C MIR SINE/MIR (5) 257 102 770

283 11.6 0.0 0.0 chr20 37870470 37870512 (24565452) C MER96B DNA/MER1_type? (185) 232 190 771

635 18.7 10.4 2.0 chr20 37870849 37871050 (24564914) + MER96B DNA/MER1_type? 198 416 (1) 772

Ortholog in HuRef

335 35.0 10.6 0.0 gi|157726890:35164031-35174988 197 356 (10602) C MIR3 SINE/MIR (22) 186 10 1

1473 22.1 12.5 6.8 gi|157726890:35164031-35174988 1267 1817 (9141) C MER21C LTR/ERVL (35) 903 267 2

850 20.6 5.2 0.2 gi|157726890:35164031-35174988 1806 2047 (8911) C MER21C LTR/ERVL (584) 354 1 3 *

234 23.8 18.0 1.3 gi|157726890:35164031-35174988 2268 2395 (8563) + L2c LINE/L2 3185 3333 (54) 4

4104 19.1 3.5 3.5 gi|157726890:35164031-35174988 2416 2552 (8406) C L1MC4 LINE/L1 (0) 8042 7900 5

255 7.9 0.0 0.0 gi|157726890:35164031-35174988 2553 2590 (8368) + (CA)n Simple_repeat 2 39 (0) 6

4104 19.1 3.5 3.5 gi|157726890:35164031-35174988 2591 3375 (7583) C L1MC4 LINE/L1 (143) 7899 7120 5

**8153 2.2 0.9 0.0 gi|157726890:35164031-35174988 4952 5932 (5026) C L1HS LINE/L1 (4380) 1652 663 7 *(OCCUPIED)**

24610 2.4 0.2 0.1 gi|157726890:35164031-35174988 5930 9664 (1294) + L1PA2 LINE/L1 2414 6155 (0) 8

25 81.2 0.0 0.0 gi|157726890:35164031-35174988 10107 10138 (820) + AT_rich Low_complexity 1 32 (0) 9

301 25.0 4.2 0.0 gi|157726890:35164031-35174988 10220 10291 (667) + MIRb SINE/MIR 123 197 (71) 10

331 25.9 16.2 5.2 gi|157726890:35164031-35174988 10373 10600 (358) + MLT1E2 LTR/ERVL-MaLR 2 253 (374) 11

444 5.4 0.0 0.0 gi|157726890:35164031-35174988 10610 10665 (293) C L1P1 LINE/L1 (391) 5764 5709 12

414 0.0 0.0 0.0 gi|157726890:35164031-35174988 10683 10728 (230) + (TAA)n Simple_repeat 3 48 (0) 13

934 24.8 8.1 0.4 gi|157726890:35164031-35174988 10736 10958 (0) + MLT1E2 LTR/ERVL-MaLR 270 509 (118) 11
