# Supplementary material for: RISCI - Repeat Induced Sequence Changes Identifier: a comprehensive, comparative genomics-based, in silico subtractive hybridization pipeline to identify repeat induced sequence changes in closely related genomes
Source: BMC Bioinformatics. 2010 Dec 26;11:609. doi: 10.1186/1471-2105-11-609 (PMC3024322; doi:10.1186/1471-2105-11-609)
Supplement: Additional file 6 — Pairwise alignment and RepeatMasker annotation of repeat locus in main genome and the ortholog in comparative genome for truncated L1HS. Summary of pair-wise alignments between the truncated L1HS locus in the reference genome and the identified ortholog in the comparative genome and its RepeatMasker annotation to confirm RISCI annotation. [file 1471-2105-11-609-S6.DOC]

**Additional file 6 -Truncated L1HS comparison with Chimpanzee genome**

**The file summarizes the blast2 result of the identified ortholog in the comparative genome with the L1HS repeat in the reference or main genome and also gives the repeat masker annotation of the identified orthologous locus, along with N details.**

**OFC – Ortholog start coordinate, OLC – ortholog end coordinate, RFC – Repeat start coordinate, RLC – Repeat end coordinate**

**1. L1HS_10_1**

**Ortholog annotation INDEL_PTS Length 1195 nscore 2.51 NPOSITIONS 1 30 ;**

**Repeat length (main genome) 199**

**Blast2 Results -**

**OFC OLC RFC RLC**

**no hits found**

**Ortholog Repeat Masker annotation**

**There were no repetitive sequences detected in /home/vipin/L1HS_TR_CHR/Chimp/CONFIRMATION/L1HS_TR_INDEL_SEQUENCES/L1HS_10_1**

**_________________________________________________________________________**

**2. L1HS_10_10**

**Ortholog annotation INDEL_PTS Length 321 nscore 0.00 NPOSITIONS NA**

**Repeat length (main genome) 78**

**Blast2 Results -**

**OFC OLC RFC RLC**

**no hits found**

**Ortholog Repeat Masker annotation**

**SW perc perc perc query position in query matching repeat position in repeat**

**score div. del. ins. sequence begin end (left) repeat class/family begin end (left) ID**

**2608 5.5 0.0 0.0 L1HS_10_10 1 308 (13) C AluY SINE/Alu (3) 308 1 1**

**_________________________________________________________________________**

**3. L1HS_10_29c**

**Ortholog annotation M_INTRA_RMD Length 5781 nscore 1.70 NPOSITIONS 719 728 ; 5253 5340 ;**

**Repeat length (main genome) 310**

**Blast2 Results -**

**OFC OLC RFC RLC**

**1 306 1 306**

**1354 1659 1 306**

**Ortholog Repeat Masker annotation**

**SW perc perc perc query position in query matching repeat position in repeat**

**score div. del. ins. sequence begin end (left) repeat class/family begin end (left) ID**

**5870 4.3 0.0 0.6 L1HS_10_29c 1 718 (5063) + L1HS LINE/L1 13 726 (5306) 1**

**18449 2.6 0.0 0.1 L1HS_10_29c 1343 5251 (530) + L1HS LINE/L1 126 4032 (2114) 2**

**3702 2.7 0.2 0.2 L1HS_10_29c 5341 5781 (0) + L1HS LINE/L1 4686 5126 (1020) 2**

**_________________________________________________________________________**

**4. L1HS_10_30c**

**Ortholog annotation C_INTRA_RMD Length 4124 nscore 0.24 NPOSITIONS 1591 1600 ;**

**Repeat length (main genome) 4860**

**Blast2 Results -**

**OFC OLC RFC RLC**

**1 1590 1 1596**

**1641 4124 2375 4860**

**Ortholog Repeat Masker annotation**

**SW perc perc perc query position in query matching repeat position in repeat**

**score div. del. ins. sequence begin end (left) repeat class/family begin end (left) ID**

**13750 2.6 0.5 0.1 L1HS_10_30c 1 1590 (2534) + L1HS LINE/L1 124 1720 (4435) 1**

**21374 2.9 0.2 0.1 L1HS_10_30c 1601 4124 (0) + L1HS LINE/L1 2459 4985 (1161) 1**

**_________________________________________________________________________**

**5. L1HS_10_32**

**Ortholog annotation INDEL_PTS Length 2980 nscore 0.00 NPOSITIONS NA**

**Repeat length (main genome) 397**

**Blast2 Results -**

**OFC OLC RFC RLC**

**Ortholog Repeat Masker annotation**

**SW perc perc perc query position in query matching repeat position in repeat**

**score div. del. ins. sequence begin end (left) repeat class/family begin end (left) ID**

**339 27.1 0.9 2.7 L1HS_10_32 47 156 (2824) + L1M4 LINE/L1 4080 4187 (1959) 1**

**988 19.8 0.0 0.6 L1HS_10_32 366 538 (2442) C AluJb SINE/Alu (17) 295 124 2**

**559 16.8 11.5 2.5 L1HS_10_32 1781 1902 (1078) + L1M2 LINE/L1 4073 4205 (1938) 3**

**4540 19.6 3.0 0.7 L1HS_10_32 1954 2949 (31) + L1M4 LINE/L1 4314 5332 (814) 1**

**198 10.3 0.0 0.0 L1HS_10_32 2952 2980 (0) + (T)n Simple_repeat 1 29 (0) 4**

**_________________________________________________________________________**

**6. L1HS_10_39**

**Ortholog annotation M_INTRA_RMD Length 2701 nscore 0.00 NPOSITIONS NA**

**Repeat length (main genome) 776**

**Blast2 Results -**

**OFC OLC RFC RLC**

**1 62 2 62**

**64 765 74 776**

**Ortholog Repeat Masker annotation**

**SW perc perc perc query position in query matching repeat position in repeat**

**score div. del. ins. sequence begin end (left) repeat class/family begin end (left) ID**

**18526 2.8 0.1 0.1 L1HS_10_39 1 2701 (0) + L1HS LINE/L1 125 2826 (3320) 1**

**_________________________________________________________________________**

**7. L1HS_10_43c**

**Ortholog annotation INDEL_CAN Length 2212 nscore 0.00 NPOSITIONS NA**

**Repeat length (main genome) 299**

**Blast2 Results -**

**OFC OLC RFC RLC**

**Ortholog Repeat Masker annotation**

**SW perc perc perc query position in query matching repeat position in repeat**

**score div. del. ins. sequence begin end (left) repeat class/family begin end (left) ID**

**215 26.3 0.0 1.7 L1HS_10_43c 789 846 (1366) C MIRm SINE/MIR (4) 272 216 1**

**2361 11.0 0.0 0.0 L1HS_10_43c 1054 1363 (849) + AluSg SINE/Alu 1 310 (0) 2**

**243 15.4 0.0 0.0 L1HS_10_43c 1388 1426 (786) C tRNA-Ile-ATA tRNA (38) 39 1 3**

**265 21.2 16.7 0.0 L1HS_10_43c 1453 1518 (694) + MIR SINE/MIR 18 94 (168) 4**

**2226 12.5 0.0 0.0 L1HS_10_43c 1717 2028 (184) + AluSx SINE/Alu 1 312 (0) 5**

**_________________________________________________________________________**

**8. L1HS_11_17c**

**Ortholog annotation C_DISRUPTED_M_INTER_RMD Length 2324 nscore 6.45 NPOSITIONS 1551 1700 ;**

**Repeat length (main genome) 1722**

**Blast2 Results -**

**OFC OLC RFC RLC**

**1 1387 1 1394**

**1709 2321 1105 1719**

**Ortholog Repeat Masker annotation**

**SW perc perc perc query position in query matching repeat position in repeat**

**score div. del. ins. sequence begin end (left) repeat class/family begin end (left) ID**

**10468 3.6 2.6 0.0 L1HS_11_17c 1 1501 (823) + L1P1 LINE/L1 1990 3529 (2617) 1**

**4722 4.1 0.6 1.1 L1HS_11_17c 1701 2324 (0) + L1P1 LINE/L1 3086 3706 (2440) 2**

**_________________________________________________________________________**

**9. L1HS_11_25c**

**Ortholog annotation M_INTRA_RMD Length 2146 nscore 0.00 NPOSITIONS NA**

**Repeat length (main genome) 1538**

**Blast2 Results -**

**OFC OLC RFC RLC**

**1 1531 1 1538**

**Ortholog Repeat Masker annotation**

**SW perc perc perc query position in query matching repeat position in repeat**

**score div. del. ins. sequence begin end (left) repeat class/family begin end (left) ID**

**17033 3.7 0.4 0.1 L1HS_11_25c 1 2146 (0) + L1HS LINE/L1 135 2288 (3858) 1**

**_________________________________________________________________________**

**10. L1HS_11_4**

**Ortholog annotation C_DISRUPTED_M_INTER_RMD Length 4069 nscore 0.00 NPOSITIONS NA**

**Repeat length (main genome) 3955**

**Blast2 Results -**

**OFC OLC RFC RLC**

**1 740 1 747**

**880 4069 759 3954**

**Ortholog Repeat Masker annotation**

**SW perc perc perc query position in query matching repeat position in repeat**

**score div. del. ins. sequence begin end (left) repeat class/family begin end (left) ID**

**17645 6.2 0.5 0.0 L1HS_11_4 1 4069 (0) + L1P1 LINE/L1 13 4102 (2044) 1**

**_________________________________________________________________________**

**11. L1HS_11_41c**

**Ortholog annotation INDEL_CAN Length 6520 nscore 0.00 NPOSITIONS NA**

**Repeat length (main genome) 2330**

**Blast2 Results -**

**OFC OLC RFC RLC**

**1 1428 1 1428**

**Ortholog Repeat Masker annotation**

**SW perc perc perc query position in query matching repeat position in repeat**

**score div. del. ins. sequence begin end (left) repeat class/family begin end (left) ID**

**11899 3.3 0.1 0.0 L1HS_11_41c 1 1428 (5092) + L1P1 LINE/L1 3873 5301 (845) 1**

**234 0.0 0.0 0.0 L1HS_11_41c 1509 1534 (4986) + (TTTG)n Simple_repeat 1 26 (0) 2**

**2016 11.8 0.0 0.4 L1HS_11_41c 1538 1808 (4712) C AluSx SINE/Alu (35) 277 8 3**

**683 27.8 4.6 2.5 L1HS_11_41c 1910 2149 (4371) C L1M5 LINE/L1 (494) 5700 5456 4**

**985 29.5 13.4 6.6 L1HS_11_41c 2156 2761 (3759) + MLT2F LTR/ERVL 17 663 (0) 5**

**232 22.5 6.5 4.3 L1HS_11_41c 4038 4130 (2390) + MLT1J LTR/MaLR 418 512 (0) 6**

**669 32.5 8.6 1.9 L1HS_11_41c 4610 4970 (1550) + L2 LINE/L2 2489 2873 (546) 7**

**2153 11.6 0.7 0.3 L1HS_11_41c 5417 5710 (810) C AluSq SINE/Alu (18) 295 1 8**

**202 31.1 0.0 1.6 L1HS_11_41c 5731 5792 (728) + MIRc SINE/MIR 86 146 (122) 9**

**2163 14.5 5.1 0.0 L1HS_11_41c 5793 6165 (355) C L1MA6 LINE/L1 (462) 5838 5447 10**

**1336 14.7 16.6 4.0 L1HS_11_41c 6158 6519 (1) + L1MA6 LINE/L1 5898 6297 (0) 10 ***

**_________________________________________________________________________**

**12. L1HS_11_47**

**Ortholog annotation C_INTER_RMD_M_DISRUPTED Length 4378 nscore 2.28 NPOSITIONS 1372 1471 ;**

**Repeat length (main genome) 1391**

**Blast2 Results -**

**OFC OLC RFC RLC**

**1 1371 1 1373**

**Ortholog Repeat Masker annotation**

**SW perc perc perc query position in query matching repeat position in repeat**

**score div. del. ins. sequence begin end (left) repeat class/family begin end (left) ID**

**6269 3.5 0.5 0.1 L1HS_11_47 1 1371 (3007) + L1P1 LINE/L1 1487 2859 (3287) 1**

**1098 22.6 0.5 1.4 L1HS_11_47 1656 1866 (2512) C MER58A DNA/MER1_type (3) 221 13 2**

**1547 20.1 11.3 3.8 L1HS_11_47 1916 2313 (2065) + MSTC LTR/MaLR 1 428 (0) 3**

**483 32.0 6.3 1.0 L1HS_11_47 3024 3228 (1150) + MIRb SINE/MIR 38 253 (15) 4**

**193 32.9 19.7 4.0 L1HS_11_47 3606 3757 (621) C MIRb SINE/MIR (89) 179 4 5**

**424 27.6 3.7 5.9 L1HS_11_47 3818 3952 (426) C MIRb SINE/MIR (48) 220 89 6**

**311 26.5 18.6 0.8 L1HS_11_47 4257 4374 (4) C MIRb SINE/MIR (18) 250 112 7**

**_________________________________________________________________________**

**13. L1HS_11_62**

**Ortholog annotation INDEL_PAC Length 111 nscore 0.00 NPOSITIONS NA**

**Repeat length (main genome) 1680**

**Blast2 Results -**

**OFC OLC RFC RLC**

**no hits found**

**Ortholog Repeat Masker annotation**

**SW perc perc perc query position in query matching repeat position in repeat**

**score div. del. ins. sequence begin end (left) repeat class/family begin end (left) ID**

**668 9.8 0.0 1.0 L1HS_11_62 8 110 (1) + (GAAA)n Simple_repeat 4 105 (0) 1**

**_________________________________________________________________________**

**14. L1HS_12_11**

**Ortholog annotation INDEL_PTS Length 3559 nscore 0.28 NPOSITIONS 3338 3347 ;**

**Repeat length (main genome) 888**

**Blast2 Results -**

**OFC OLC RFC RLC**

**1699 2588 1 888**

**Ortholog Repeat Masker annotation**

**SW perc perc perc query position in query matching repeat position in repeat**

**score div. del. ins. sequence begin end (left) repeat class/family begin end (left) ID**

**2711 23.8 6.3 1.5 L1HS_12_11 12 728 (2831) C L1ME3A LINE/L1 (3) 6170 5420 1**

**445 29.8 3.9 3.4 L1HS_12_11 879 1106 (2453) C MLT1H2 LTR/MaLR (199) 350 85 2**

**232 26.9 1.5 0.0 L1HS_12_11 1122 1188 (2371) C MLT1H2 LTR/MaLR (470) 79 12 2**

**1239 23.0 7.8 0.5 L1HS_12_11 1200 1571 (1988) C L1ME3A LINE/L1 (743) 5403 5005 1**

**13689 3.8 0.1 0.1 L1HS_12_11 1699 3326 (233) + L1HS LINE/L1 3 1629 (4403) 3**

**_________________________________________________________________________**

**15. L1HS_12_2**

**Ortholog annotation INDEL_PTS Length 117 nscore 0.00 NPOSITIONS NA**

**Repeat length (main genome) 324**

**Blast2 Results -**

**OFC OLC RFC RLC**

**Ortholog Repeat Masker annotation**

**SW perc perc perc query position in query matching repeat position in repeat**

**score div. del. ins. sequence begin end (left) repeat class/family begin end (left) ID**

**423 0.0 0.0 0.0 L1HS_12_2 66 112 (5) + (TG)n Simple_repeat 2 48 (0) 1**

**_________________________________________________________________________**

**16. L1HS_12_30**

**Ortholog annotation C_INTER_RMD Length 2312 nscore 0.43 NPOSITIONS 11 20 ;**

**Repeat length (main genome) 2678**

**Blast2 Results -**

**OFC OLC RFC RLC**

**115 141 476 502**

**256 2312 625 2678**

**Ortholog Repeat Masker annotation**

**SW perc perc perc query position in query matching repeat position in repeat**

**score div. del. ins. sequence begin end (left) repeat class/family begin end (left) ID**

**17537 3.7 1.0 0.4 L1HS_12_30 74 2312 (0) + L1P1 LINE/L1 2458 4710 (1436) 1**

**_________________________________________________________________________**

**17. L1HS_12_31c**

**Ortholog annotation C_INTER_RMD_M_DISRUPTED Length 310 nscore 0.00 NPOSITIONS NA**

**Repeat length (main genome) 322**

**Blast2 Results -**

**OFC OLC RFC RLC**

**1 310 1 306**

**Ortholog Repeat Masker annotation**

**SW perc perc perc query position in query matching repeat position in repeat**

**score div. del. ins. sequence begin end (left) repeat class/family begin end (left) ID**

**2474 6.1 0.3 0.0 L1HS_12_31c 1 310 (0) + L1P1 LINE/L1 3 313 (5842) 1**

**_________________________________________________________________________**

**18. L1HS_13_18**

**Ortholog annotation M_INTRA_RMD Length 3249 nscore 0.00 NPOSITIONS NA**

**Repeat length (main genome) 1742**

**Blast2 Results -**

**OFC OLC RFC RLC**

**1 1746 1 1742**

**Ortholog Repeat Masker annotation**

**SW perc perc perc query position in query matching repeat position in repeat**

**score div. del. ins. sequence begin end (left) repeat class/family begin end (left) ID**

**18274 2.9 0.0 0.0 L1HS_13_18 1 3249 (0) + L1HS LINE/L1 128 3377 (2769) 1**

**_________________________________________________________________________**

**19. L1HS_13_34c**

**Ortholog annotation C_INTER_RMD_M_DISRUPTED Length 357 nscore 0.00 NPOSITIONS NA**

**Repeat length (main genome) 355**

**Blast2 Results -**

**OFC OLC RFC RLC**

**3 342 1 340**

**Ortholog Repeat Masker annotation**

**SW perc perc perc query position in query matching repeat position in repeat**

**score div. del. ins. sequence begin end (left) repeat class/family begin end (left) ID**

**2937 3.9 0.0 0.6 L1HS_13_34c 1 357 (0) + L1HS LINE/L1 578 932 (5100) 1**

**_________________________________________________________________________**

**20. L1HS_13_35c**

**Ortholog annotation C_INTER_RMD_M_DISRUPTED Length 568 nscore 0.00 NPOSITIONS NA**

**Repeat length (main genome) 586**

**Blast2 Results -**

**OFC OLC RFC RLC**

**1 568 1 568**

**Ortholog Repeat Masker annotation**

**SW perc perc perc query position in query matching repeat position in repeat**

**score div. del. ins. sequence begin end (left) repeat class/family begin end (left) ID**

**4535 5.1 0.0 0.2 L1HS_13_35c 1 568 (0) + L1HS LINE/L1 4 570 (5462) 1**

**_________________________________________________________________________**

**21. L1HS_14_20**

**Ortholog annotation C_INTER_RMD_M_DISRUPTED Length 3956 nscore 0.25 NPOSITIONS 3936 3945 ;**

**Repeat length (main genome) 4188**

**Blast2 Results -**

**OFC OLC RFC RLC**

**1 651 1 651**

**667 3903 654 3884**

**Ortholog Repeat Masker annotation**

**SW perc perc perc query position in query matching repeat position in repeat**

**score div. del. ins. sequence begin end (left) repeat class/family begin end (left) ID**

**17883 3.3 0.1 0.3 L1HS_14_20 1 3935 (21) + L1HS LINE/L1 124 4049 (2097) 1**

**_________________________________________________________________________**

**22. L1HS_14_21c**

**Ortholog annotation INDEL_PTS Length 136 nscore 0.00 NPOSITIONS NA**

**Repeat length (main genome) 787**

**Blast2 Results -**

**OFC OLC RFC RLC**

**no hits found**

**Ortholog Repeat Masker annotation**

**SW perc perc perc query position in query matching repeat position in repeat**

**score div. del. ins. sequence begin end (left) repeat class/family begin end (left) ID**

**1016 8.5 0.0 0.0 L1HS_14_21c 1 130 (6) + AluSp/q SINE/Alu 166 295 (18) 1**

**_________________________________________________________________________**

**23. L1HS_14_31**

**Ortholog annotation C_INTRA_RMD Length 2093 nscore 0.48 NPOSITIONS 624 633 ;**

**Repeat length (main genome) 2399**

**Blast2 Results -**

**OFC OLC RFC RLC**

**1 623 1 623**

**634 2093 939 2398**

**Ortholog Repeat Masker annotation**

**SW perc perc perc query position in query matching repeat position in repeat**

**score div. del. ins. sequence begin end (left) repeat class/family begin end (left) ID**

**3589 4.2 0.0 0.0 L1HS_14_31 1 623 (1470) + L1HS LINE/L1 1932 2554 (3592) 1**

**12042 3.1 0.3 0.0 L1HS_14_31 634 2093 (0) + L1HS LINE/L1 2870 4333 (1813) 1**

**_________________________________________________________________________**

**24. L1HS_14_35**

**Ortholog annotation C_DISRUPTED_M_INTER_RMD Length 2562 nscore 6.25 NPOSITIONS 865 1024 ;**

**Repeat length (main genome) 1734**

**Blast2 Results -**

**OFC OLC RFC RLC**

**2 808 2 808**

**1286 1534 445 694**

**1538 2562 709 1733**

**Ortholog Repeat Masker annotation**

**SW perc perc perc query position in query matching repeat position in repeat**

**score div. del. ins. sequence begin end (left) repeat class/family begin end (left) ID**

**6251 5.2 0.2 0.2 L1HS_14_35 2 808 (1754) + L1HS LINE/L1 2 808 (5224) 1**

**10773 3.9 2.6 0.0 L1HS_14_35 1145 2562 (0) + L1HS LINE/L1 279 1733 (4299) 2**

**_________________________________________________________________________**

**25. L1HS_15_23**

**Ortholog annotation C_INTRA_RMD Length 3066 nscore 0.33 NPOSITIONS 2456 2465 ;**

**Repeat length (main genome) 3563**

**Blast2 Results -**

**OFC OLC RFC RLC**

**1 2455 1 2450**

**2603 3066 3099 3562**

**Ortholog Repeat Masker annotation**

**SW perc perc perc query position in query matching repeat position in repeat**

**score div. del. ins. sequence begin end (left) repeat class/family begin end (left) ID**

**15469 2.2 0.1 0.0 L1HS_15_23 1 2455 (611) + L1HS LINE/L1 1594 4052 (2094) 1**

**3869 2.5 2.7 0.0 L1HS_15_23 2544 3066 (0) + L1HS LINE/L1 4634 5170 (976) 1**

**_________________________________________________________________________**

**26. L1HS_16_1**

**Ortholog annotation INDEL_PAC Length 117 nscore 0.00 NPOSITIONS NA**

**Repeat length (main genome) 240**

**Blast2 Results -**

**OFC OLC RFC RLC**

**no hits found**

**Ortholog Repeat Masker annotation**

**There were no repetitive sequences detected in /home/vipin/L1HS_TR_CHR/Chimp/CONFIRMATION/L1HS_TR_INDEL_SEQUENCES/L1HS_16_1**

**_________________________________________________________________________**

**27. L1HS_16_11c**

**Ortholog annotation C_INTER_RMD_M_DISRUPTED Length 3246 nscore 0.31 NPOSITIONS 118 127 ;**

**Repeat length (main genome) 3265**

**Blast2 Results -**

**OFC OLC RFC RLC**

**128 3246 145 3264**

**Ortholog Repeat Masker annotation**

**SW perc perc perc query position in query matching repeat position in repeat**

**score div. del. ins. sequence begin end (left) repeat class/family begin end (left) ID**

**17824 4.1 0.2 0.1 L1HS_16_11c 128 3246 (0) + L1P1 LINE/L1 1281 4403 (1743) 1**

**_________________________________________________________________________**

**28. L1HS_16_16c**

**Ortholog annotation C_INTER_RMD_M_DISRUPTED Length 170 nscore 0.00 NPOSITIONS NA**

**Repeat length (main genome) 552**

**Blast2 Results -**

**OFC OLC RFC RLC**

**1 170 1 170**

**Ortholog Repeat Masker annotation**

**SW perc perc perc query position in query matching repeat position in repeat**

**score div. del. ins. sequence begin end (left) repeat class/family begin end (left) ID**

**1353 6.5 0.0 0.0 L1HS_16_16c 1 170 (0) + L1P2 LINE/L1 5603 5772 (383) 1**

**_________________________________________________________________________**

**29. L1HS_16_23**

**Ortholog annotation M_INTRA_RMD Length 8444 nscore 8.85 NPOSITIONS 5497 5652 ; 6866 7456 ;**

**Repeat length (main genome) 3628**

**Blast2 Results -**

**OFC OLC RFC RLC**

**1 3623 1 3628**

**Ortholog Repeat Masker annotation**

**SW perc perc perc query position in query matching repeat position in repeat**

**score div. del. ins. sequence begin end (left) repeat class/family begin end (left) ID**

**27226 3.4 0.1 0.0 L1HS_16_23 1 5496 (2948) + L1HS LINE/L1 130 5628 (527) 1**

**3809 23.8 6.5 1.4 L1HS_16_23 5679 6843 (1601) + L1M5 LINE/L1 3443 4667 (1479) 2**

**2550 19.3 5.4 2.0 L1HS_16_23 7464 8444 (0) + L1M5 LINE/L1 4742 5754 (425) 2**

**_________________________________________________________________________**

**30. L1HS_16_24**

**Ortholog annotation C_INTER_RMD_M_DISRUPTED Length 1105 nscore 0.00 NPOSITIONS NA**

**Repeat length (main genome) 1114**

**Blast2 Results -**

**OFC OLC RFC RLC**

**1 1101 1 1100**

**Ortholog Repeat Masker annotation**

**SW perc perc perc query position in query matching repeat position in repeat**

**score div. del. ins. sequence begin end (left) repeat class/family begin end (left) ID**

**9013 5.3 0.1 0.2 L1HS_16_24 1 1105 (0) + L1HS LINE/L1 1 1104 (4928) 1**

**_________________________________________________________________________**

**31. L1HS_16_25**

**Ortholog annotation INDEL_CAN Length 2343 nscore 0.00 NPOSITIONS NA**

**Repeat length (main genome) 1073**

**Blast2 Results -**

**OFC OLC RFC RLC**

**1 1067 7 1073**

**Ortholog Repeat Masker annotation**

**SW perc perc perc query position in query matching repeat position in repeat**

**score div. del. ins. sequence begin end (left) repeat class/family begin end (left) ID**

**11417 2.9 0.0 0.0 L1HS_16_25 1 2343 (0) + L1HS LINE/L1 1238 3579 (2567) 1**

**_________________________________________________________________________**

**32. L1HS_16_26c**

**Ortholog annotation INDEL_PTS Length 3643 nscore 0.00 NPOSITIONS NA**

**Repeat length (main genome) 606**

**Blast2 Results -**

**OFC OLC RFC RLC**

**Ortholog Repeat Masker annotation**

**SW perc perc perc query position in query matching repeat position in repeat**

**score div. del. ins. sequence begin end (left) repeat class/family begin end (left) ID**

**5034 8.1 0.0 0.4 L1HS_16_26c 1 693 (2950) C L1P1 LINE/L1 (2127) 4019 3330 1**

**599 18.6 0.9 0.0 L1HS_16_26c 694 806 (2837) + L1MC3 LINE/L1 6160 6273 (1512) 2**

**1750 18.9 3.2 4.5 L1HS_16_26c 808 1207 (2436) + L1MA9 LINE/L1 5880 6274 (38) 3**

**6477 19.7 6.6 2.3 L1HS_16_26c 1269 1546 (2097) + L1MC3 LINE/L1 6261 6550 (1235) 2**

**1955 4.9 0.0 0.0 L1HS_16_26c 1547 1791 (1852) + L1PA4 LINE/L1 5908 6152 (3) 4**

**6477 19.7 6.6 2.3 L1HS_16_26c 1792 2975 (668) + L1MC3 LINE/L1 6551 7785 (0) 2**

**26 0.0 0.0 0.0 L1HS_16_26c 3065 3090 (553) + AT_rich Low_complexity 1 26 (0) 5**

**376 30.9 3.6 3.6 L1HS_16_26c 3316 3510 (133) + MLT1N2 LTR/MaLR 347 541 (21) 6**

**_________________________________________________________________________**

**33. L1HS_17_2**

**Ortholog annotation C_DISRUPTED_M_INTER_RMD Length 7466 nscore 7.55 NPOSITIONS 1894 2447 ; 3285 3294 ;**

**Repeat length (main genome) 3658**

**Blast2 Results -**

**OFC OLC RFC RLC**

**1 1885 1 1887**

**2448 2521 2831 2904**

**2797 3284 2637 3126**

**3302 5502 1455 3658**

**6699 7466 2890 3657**

**Ortholog Repeat Masker annotation**

**SW perc perc perc query position in query matching repeat position in repeat**

**score div. del. ins. sequence begin end (left) repeat class/family begin end (left) ID**

**9196 3.0 0.1 0.0 L1HS_17_2 1 1893 (5573) + L1P1 LINE/L1 1343 3237 (2909) 1**

**615 5.3 0.0 0.0 L1HS_17_2 2448 2523 (4943) + L1P1 LINE/L1 4174 4249 (1897) 1 ***

**2435 0.7 1.1 0.0 L1HS_17_2 2514 2797 (4669) C L1P1 LINE/L1 (2165) 3981 3695 2 ***

**4156 4.4 0.8 0.0 L1HS_17_2 2796 3295 (4171) + L1P1 LINE/L1 3977 4480 (1666) 2**

**21660 2.3 0.6 0.0 L1HS_17_2 3302 6642 (824) + L1PA2 LINE/L1 2796 6155 (0) 3**

**6519 2.3 0.0 0.0 L1HS_17_2 6699 7466 (0) + L1P1 LINE/L1 4233 5000 (1146) 1**

**_________________________________________________________________________**

**34. L1HS_18_22c**

**Ortholog annotation M_INTRA_RMD Length 8094 nscore 9.80 NPOSITIONS 2158 2940 ; 3887 3896 ;**

**Repeat length (main genome) 3547**

**Blast2 Results -**

**OFC OLC RFC RLC**

**1 2157 1 2152**

**2941 3877 2191 3124**

**4086 6212 1427 3543**

**Ortholog Repeat Masker annotation**

**SW perc perc perc query position in query matching repeat position in repeat**

**score div. del. ins. sequence begin end (left) repeat class/family begin end (left) ID**

**17312 4.7 0.3 0.2 L1HS_18_22c 1 2157 (5937) + L1HS LINE/L1 124 2284 (3862) 1**

**7430 2.8 1.2 0.2 L1HS_18_22c 2941 3886 (4208) + L1HS LINE/L1 2323 3277 (2869) 1**

**26907 3.3 0.8 0.1 L1HS_18_22c 3958 8094 (0) + L1HS LINE/L1 1402 5572 (583) 2**

**_________________________________________________________________________**

**35. L1HS_18_38**

**Ortholog annotation C_DISRUPTED_M_INTER_RMD Length 6052 nscore 8.11 NPOSITIONS 181 651 ; 2230 2239 ; 5586 5595 ;**

**Repeat length (main genome) 1967**

**Blast2 Results -**

**OFC OLC RFC RLC**

**1 180 1 179**

**652 2229 275 1852**

**4679 5585 1 905**

**5596 6052 1510 1966**

**Ortholog Repeat Masker annotation**

**SW perc perc perc query position in query matching repeat position in repeat**

**score div. del. ins. sequence begin end (left) repeat class/family begin end (left) ID**

**1417 6.1 0.0 0.0 L1HS_18_38 1 180 (5872) + L1HS LINE/L1 2 181 (5851) 1**

**13127 3.9 0.2 0.1 L1HS_18_38 652 2229 (3823) + L1HS LINE/L1 277 1857 (4175) 1**

**5721 16.3 2.2 1.9 L1HS_18_38 2240 3252 (2800) + Tigger1 DNA/MER2_type 174 1189 (1229) 2**

**1033 16.7 2.6 1.0 L1HS_18_38 3250 3443 (2609) + Tigger1 DNA/MER2_type 2184 2380 (38) 2 ***

**252 0.0 0.0 0.0 L1HS_18_38 3513 3540 (2512) + (TG)n Simple_repeat 1 28 (0) 3**

**183 4.2 0.0 0.0 L1HS_18_38 4009 4032 (2020) + (T)n Simple_repeat 1 24 (0) 4**

**924 23.3 12.7 5.9 L1HS_18_38 4131 4453 (1599) + MLT1B LTR/MaLR 1 459 (46) 5**

**216 14.3 0.0 0.0 L1HS_18_38 4455 4496 (1556) + (A)n Simple_repeat 1 42 (0) 6**

**7465 4.1 0.2 0.1 L1HS_18_38 4678 5585 (467) + L1HS LINE/L1 124 1032 (5123) 7**

**3834 3.3 0.0 0.0 L1HS_18_38 5596 6052 (0) + L1HS LINE/L1 1638 2094 (4061) 7**

**_________________________________________________________________________**

**36. L1HS_19_12c**

**Ortholog annotation C_INTER_RMD_M_DISRUPTED Length 2504 nscore 0.00 NPOSITIONS NA**

**Repeat length (main genome) 5700**

**Blast2 Results -**

**OFC OLC RFC RLC**

**1 1021 1 1021**

**1037 2504 2563 4036**

**Ortholog Repeat Masker annotation**

**SW perc perc perc query position in query matching repeat position in repeat**

**score div. del. ins. sequence begin end (left) repeat class/family begin end (left) ID**

**7251 9.8 4.3 1.2 L1HS_19_12c 1 1021 (1483) + L1P2 LINE/L1 5 1057 (1218) 1 ***

**10794 8.7 0.7 0.0 L1HS_19_12c 1018 2504 (0) + L1P2 LINE/L1 2571 4067 (2079) 1**

**_________________________________________________________________________**

**37. L1HS_1_13**

**Ortholog annotation M_INTRA_RMD Length 6463 nscore 0.34 NPOSITIONS 3700 3709 ; 3731 3732 ; 5406 5415 ;**

**Repeat length (main genome) 2607**

**Blast2 Results -**

**OFC OLC RFC RLC**

**1 2616 1 2607**

**Ortholog Repeat Masker annotation**

**SW perc perc perc query position in query matching repeat position in repeat**

**score div. del. ins. sequence begin end (left) repeat class/family begin end (left) ID**

**18267 3.0 1.4 0.3 L1HS_1_13 1 3556 (2907) + L1HS LINE/L1 125 3718 (2428) 1**

**14691 2.9 0.2 0.1 L1HS_1_13 3710 5422 (1041) + L1P1 LINE/L1 3447 5162 (984) 2**

**7145 2.0 1.3 0.0 L1HS_1_13 5426 6463 (0) + L1HS LINE/L1 4515 5568 (587) 1**

**_________________________________________________________________________**

**38. L1HS_1_21c**

**Ortholog annotation C_INTER_RMD_M_DISRUPTED Length 9871 nscore 0.00 NPOSITIONS NA**

**Repeat length (main genome) 2176**

**Blast2 Results -**

**OFC OLC RFC RLC**

**3346 3449 9 112**

**4221 4277 592 648**

**4301 4326 672 697**

**4529 4589 775 835**

**4641 4691 863 913**

**4908 4960 1133 1185**

**5097 5885 1321 2110**

**5915 5946 2133 2164**

**7571 8340 1 773**

**8470 9871 775 2176**

**Ortholog Repeat Masker annotation**

**SW perc perc perc query position in query matching repeat position in repeat**

**score div. del. ins. sequence begin end (left) repeat class/family begin end (left) ID**

**2169 13.1 0.3 0.0 L1HS_1_21c 780 1077 (8794) + AluSx SINE/Alu 1 299 (13) 1**

**545 29.4 7.4 1.1 L1HS_1_21c 1387 1575 (8296) + L2 LINE/L2 2632 2832 (587) 2**

**376 25.3 10.4 0.6 L1HS_1_21c 1577 1739 (8132) + L2 LINE/L2 2781 2959 (460) 3**

**406 28.6 7.1 0.7 L1HS_1_21c 1901 2055 (7816) C Charlie4z DNA/MER1_type (2) 165 1 4**

**365 35.2 8.6 5.0 L1HS_1_21c 2144 2180 (7691) C L4 LINE/RTE (691) 1338 1300 5**

**2226 16.8 1.4 0.3 L1HS_1_21c 2181 2539 (7332) C MLT1A0 LTR/MaLR (2) 363 1 6**

**365 35.2 8.6 5.0 L1HS_1_21c 2540 2956 (6915) C L4 LINE/RTE (730) 1299 868 5**

**12748 9.4 1.1 1.3 L1HS_1_21c 3337 7566 (2305) + L1P3 LINE/L1 1 4211 (2250) 7**

**18214 5.8 0.3 0.3 L1HS_1_21c 7568 9871 (0) + L1P1 LINE/L1 1 2303 (3843) 8**

**_________________________________________________________________________**

**39. L1HS_1_23c**

**Ortholog annotation C_INTER_RMD_M_DISRUPTED Length 58785 nscore 11.01 NPOSITIONS 6670 7009 ; 10537 11281 ; 14711 15454 ; 16760 18209 ; 19982 19983 ; 20038 20047 ; 21540 23157 ; 25652 25809 ; 31000 31546 ; 39442 39471 ; 47931 48756 ;**

**Repeat length (main genome) 2499**

**Blast2 Results -**

**OFC OLC RFC RLC**

**2 693 1 693**

**690 2483 701 2491**

**19890 19979 2128 2220**

**35097 35662 1563 2129**

**35965 36322 2129 2488**

**38860 39437 1713 2290**

**39472 39694 2272 2495**

**55746 55987 820 1061**

**55988 57415 1075 2498**

**Ortholog Repeat Masker annotation**

**SW perc perc perc query position in query matching repeat position in repeat**

**score div. del. ins. sequence begin end (left) repeat class/family begin end (left) ID**

**14594 1.8 0.5 0.6 L1HS_1_23c 1 2546 (56239) + L1PA2 LINE/L1 3652 6155 (0) 1**

**262 36.9 8.3 5.8 L1HS_1_23c 2643 3162 (55623) C L2 LINE/L2 (472) 2947 2415 2**

**273 14.6 0.0 0.0 L1HS_1_23c 3425 3472 (55313) + (TG)n Simple_repeat 2 49 (0) 3**

**819 27.7 11.5 4.3 L1HS_1_23c 4672 5018 (53767) + L2a LINE/L2 3049 3420 (6) 4**

**2666 26.4 13.9 5.8 L1HS_1_23c 5194 6376 (52409) + LTR78 LTR/ERV1 1 1278 (26) 5**

**515 30.9 5.1 2.8 L1HS_1_23c 6393 6608 (52177) C MIR SINE/MIR (1) 261 41 6**

**987 11.3 0.0 2.1 L1HS_1_23c 7010 7154 (51631) C AluSq/x SINE/Alu (170) 142 1 7**

**1768 14.7 0.7 0.4 L1HS_1_23c 7208 7480 (51305) C AluJb SINE/Alu (36) 276 3 8**

**25 0.0 0.0 0.0 L1HS_1_23c 7487 7511 (51274) + AT_rich Low_complexity 1 25 (0) 9**

**2270 7.3 0.0 0.3 L1HS_1_23c 7512 7801 (50984) C AluY SINE/Alu (22) 289 1 10**

**852 29.2 1.8 0.9 L1HS_1_23c 7865 8085 (50700) + MIRb SINE/MIR 10 232 (36) 11**

**317 18.1 22.9 0.0 L1HS_1_23c 8397 8479 (50306) + Tigger13a DNA/MER2_type 192 293 (478) 12**

**3242 11.4 4.7 1.6 L1HS_1_23c 8845 9212 (49573) + MER75 DNA/PiggyBac 1 380 (134) 13**

**1891 9.9 14.0 0.9 L1HS_1_23c 9213 9547 (49238) + THE1D LTR/MaLR 3 381 (0) 14**

**3242 11.4 4.7 1.6 L1HS_1_23c 9548 9670 (49115) + MER75 DNA/PiggyBac 381 507 (7) 13**

**720 23.6 3.5 2.4 L1HS_1_23c 10013 10181 (48604) C MIR3 SINE/MIR (30) 178 8 15**

**699 28.2 7.6 0.5 L1HS_1_23c 10199 10408 (48377) C MIR SINE/MIR (0) 262 38 16**

**227 28.0 10.1 2.8 L1HS_1_23c 11351 11567 (47218) + L1M5 LINE/L1 2895 3127 (3019) 17**

**550 19.3 3.0 10.7 L1HS_1_23c 11679 11846 (46939) C MIR SINE/MIR (0) 262 108 18**

**389 30.9 5.2 3.1 L1HS_1_23c 11917 12204 (46581) + L1M5 LINE/L1 3342 3637 (2509) 17**

**284 16.1 0.0 0.0 L1HS_1_23c 12202 12263 (46522) C L1MB5 LINE/L1 (0) 6174 6113 19 ***

**1670 28.9 7.7 3.2 L1HS_1_23c 12255 13260 (45525) + L1M5 LINE/L1 3625 4658 (1488) 17**

**248 28.1 0.0 0.0 L1HS_1_23c 13520 13583 (45202) + L1M3 LINE/L1 4948 5011 (1129) 20**

**984 19.4 11.9 0.4 L1HS_1_23c 13612 13880 (44905) + Charlie4a DNA/MER1_type 119 418 (90) 21**

**27 4.9 0.0 0.0 L1HS_1_23c 13881 13921 (44864) + AT_rich Low_complexity 1 41 (0) 22**

**6598 2.2 0.1 0.8 L1HS_1_23c 13922 14710 (44075) C L1PA3 LINE/L1 (3) 6152 5369 23**

**10470 2.8 0.2 0.2 L1HS_1_23c 15483 16759 (42026) C L1PA3 LINE/L1 (1874) 4272 2995 23**

**5487 5.1 1.7 0.1 L1HS_1_23c 18210 18917 (39868) C L1PA3 LINE/L1 (5311) 844 126 23**

**443 20.2 4.0 2.0 L1HS_1_23c 18925 19025 (39760) + Charlie4a DNA/MER1_type 406 508 (0) 21**

**352 31.0 15.3 4.5 L1HS_1_23c 19026 19444 (39341) C L2 LINE/L2 (1318) 2101 1638 24**

**255 6.1 0.0 0.0 L1HS_1_23c 19819 19851 (38934) + (TG)n Simple_repeat 2 34 (0) 25**

**980 5.8 3.6 0.7 L1HS_1_23c 19890 20028 (38757) + L1PA7 LINE/L1 5784 5926 (228) 26**

**10455 3.5 0.1 0.1 L1HS_1_23c 20048 21539 (37246) C L1HS LINE/L1 (3674) 2472 981 27**

**758 31.1 5.5 3.2 L1HS_1_23c 23608 23955 (34830) C MARNA DNA/Mariner (25) 561 206 28**

**231 36.9 4.0 3.2 L1HS_1_23c 24007 24132 (34653) C L2 LINE/L2 (469) 2950 2824 29**

**268 26.9 15.1 1.9 L1HS_1_23c 24183 24288 (34497) C L3 LINE/CR1 (1123) 3366 3247 30**

**293 32.4 2.8 0.9 L1HS_1_23c 24811 24916 (33869) C L2a LINE/L2 (0) 3426 3319 31**

**202 37.1 9.8 0.0 L1HS_1_23c 25008 25139 (33646) + L2b LINE/L2 2782 2926 (493) 32 ***

**239 28.1 4.7 0.0 L1HS_1_23c 25137 25200 (33585) + L2a LINE/L2 3360 3426 (0) 33**

**220 31.7 5.9 8.8 L1HS_1_23c 26612 26773 (32012) + MIRb SINE/MIR 2 165 (103) 34**

**292 22.9 10.7 1.2 L1HS_1_23c 26855 26938 (31847) C MIR SINE/MIR (19) 243 152 35**

**431 21.9 0.0 1.2 L1HS_1_23c 27015 27097 (31688) C AluJ/FLAM SINE/Alu (228) 84 3 36**

**231 11.4 0.0 0.0 L1HS_1_23c 27308 27342 (31443) + (CATATA)n Simple_repeat 2 36 (0) 37**

**256 40.7 0.0 3.6 L1HS_1_23c 27477 27588 (31197) + L2b LINE/L2 3038 3145 (230) 32**

**789 9.0 0.0 0.0 L1HS_1_23c 27634 27744 (31041) + (TTCC)n Simple_repeat 4 114 (0) 38**

**1844 15.0 1.0 2.2 L1HS_1_23c 28004 28360 (30425) C AluJo SINE/Alu (0) 312 3 39**

**458 28.9 2.5 0.0 L1HS_1_23c 28368 28488 (30297) + MLT1I LTR/MaLR 34 157 (254) 40**

**254 27.5 2.3 8.1 L1HS_1_23c 28762 28848 (29937) + L2a LINE/L2 3340 3421 (5) 41**

**257 12.9 5.7 0.0 L1HS_1_23c 29045 29114 (29671) + (TA)n Simple_repeat 1 74 (0) 42**

**690 36.9 10.4 2.2 L1HS_1_23c 29435 30221 (28564) + L2a LINE/L2 798 1649 (1770) 43**

**510 33.8 1.9 7.1 L1HS_1_23c 30282 30692 (28093) C L2 LINE/L2 (1156) 2263 1874 44**

**2232 10.6 0.7 0.3 L1HS_1_23c 30695 30997 (27788) + AluSc SINE/Alu 1 304 (5) 45**

**1490 9.2 0.0 0.0 L1HS_1_23c 31547 31741 (27044) C AluY SINE/Alu (116) 195 1 46**

**384 36.5 2.7 2.4 L1HS_1_23c 31744 32077 (26708) + L2a LINE/L2 2254 2588 (831) 43**

**375 26.9 16.1 4.8 L1HS_1_23c 32059 32333 (26452) + L2a LINE/L2 3074 3424 (2) 43 ***

**501 28.6 10.4 1.6 L1HS_1_23c 32329 32520 (26265) C MIRc SINE/MIR (0) 268 60 47**

**189 12.1 0.0 0.0 L1HS_1_23c 32720 32752 (26033) + (GAAAA)n Simple_repeat 2 34 (0) 48**

**554 26.9 16.3 0.5 L1HS_1_23c 32758 32959 (25826) + MIRb SINE/MIR 34 267 (1) 49**

**480 28.7 4.7 0.0 L1HS_1_23c 33391 33519 (25266) C MIRb SINE/MIR (46) 222 88 50**

**309 33.6 1.3 1.3 L1HS_1_23c 33698 33851 (24934) C MER5A DNA/MER1_type (10) 179 26 51**

**888 26.2 2.7 3.4 L1HS_1_23c 34465 34756 (24029) C L1MA9 LINE/L1 (38) 6274 5985 52**

**526 13.1 6.5 0.0 L1HS_1_23c 34764 34931 (23854) + (TA)n Simple_repeat 1 179 (0) 53**

**569 14.8 3.4 2.3 L1HS_1_23c 34941 35094 (23691) C L1MA9 LINE/L1 (246) 5948 5793 52**

**6885 6.6 0.8 0.8 L1HS_1_23c 35097 35661 (23124) + L1PA7 LINE/L1 5215 5779 (375) 54**

**2359 7.5 0.0 0.3 L1HS_1_23c 35662 35957 (22828) + AluY SINE/Alu 1 295 (16) 55**

**6885 6.3 0.7 0.9 L1HS_1_23c 35958 36333 (22452) + L1PA7 LINE/L1 5780 6154 (0) 54**

**971 22.4 2.8 2.4 L1HS_1_23c 36410 36660 (22125) + Tigger13a DNA/MER2_type 117 368 (403) 56**

**2740 17.5 3.0 1.1 L1HS_1_23c 36661 37198 (21587) C L1M3 LINE/L1 (511) 5801 5254 57**

**6048 18.6 1.8 1.9 L1HS_1_23c 37213 38478 (20307) C L1M3 LINE/L1 (1424) 4716 3451 57**

**1683 19.2 0.0 0.7 L1HS_1_23c 38479 38777 (20008) C AluJo SINE/Alu (15) 297 1 58**

**6048 18.6 1.8 1.9 L1HS_1_23c 38778 38815 (19970) C L1M3 LINE/L1 (2690) 3450 3414 57**

**5989 4.5 0.4 6.3 L1HS_1_23c 38860 39694 (19091) + L1PA4 LINE/L1 5369 6153 (2) 59**

**387 30.1 5.3 4.7 L1HS_1_23c 39860 40030 (18755) + L2b LINE/L2 2657 2828 (591) 60**

**316 28.8 0.0 0.0 L1HS_1_23c 40033 40105 (18680) + L2a LINE/L2 3350 3422 (4) 61**

**608 34.9 4.8 7.4 L1HS_1_23c 40201 40621 (18164) + L2b LINE/L2 2948 3357 (18) 60**

**552 29.1 7.4 0.5 L1HS_1_23c 40785 40974 (17811) + L2 LINE/L2 2656 2858 (561) 62**

**337 23.8 0.0 2.9 L1HS_1_23c 42141 42190 (16595) C L1MC4 LINE/L1 (0) 8042 7993 63**

**1786 17.3 1.3 1.0 L1HS_1_23c 42191 42493 (16292) C AluJb SINE/Alu (8) 304 1 64**

**337 23.8 0.0 2.9 L1HS_1_23c 42494 42546 (16239) C L1MC4 LINE/L1 (50) 7992 7942 63**

**192 4.0 0.0 0.0 L1HS_1_23c 42586 42610 (16175) + (TC)n Simple_repeat 2 26 (0) 65**

**194 22.9 1.4 0.0 L1HS_1_23c 42611 42680 (16105) + (TA)n Simple_repeat 1 71 (0) 66**

**4851 23.0 3.0 3.0 L1HS_1_23c 42689 42861 (15924) C L1MC4 LINE/L1 (144) 7898 7725 63**

**1832 15.5 12.9 0.6 L1HS_1_23c 42862 43218 (15567) C MER57B1 LTR/ERV1 (0) 401 1 67**

**4851 23.0 3.0 3.0 L1HS_1_23c 43219 44222 (14563) C L1MC4 LINE/L1 (318) 7724 6721 63**

**325 23.5 3.5 0.0 L1HS_1_23c 44788 44872 (13913) + MER103 DNA 77 164 (2) 68**

**2011 11.6 2.5 0.0 L1HS_1_23c 45364 45647 (13138) C AluSg SINE/Alu (19) 291 1 69**

**608 30.0 4.9 8.2 L1HS_1_23c 48917 49341 (9444) + MLT1I LTR/MaLR 1 411 (0) 70**

**226 32.0 1.3 0.0 L1HS_1_23c 49771 49845 (8940) + MIRc SINE/MIR 12 87 (181) 71**

**409 23.4 11.0 3.4 L1HS_1_23c 50057 50202 (8583) C MIRc SINE/MIR (108) 160 4 72**

**180 35.3 15.0 0.8 L1HS_1_23c 50594 50713 (8072) + MIRc SINE/MIR 2 138 (130) 73**

**189 30.5 18.8 3.8 L1HS_1_23c 50951 51083 (7702) + L2c LINE/L2 3185 3337 (50) 74**

**2602 14.5 2.8 1.2 L1HS_1_23c 51144 51570 (7215) + Tigger2a DNA/MER2_type 1 434 (0) 75**

**1429 25.8 0.8 3.8 L1HS_1_23c 51605 51995 (6790) C MamRep137 DNA/TcMar? (1) 443 65 76**

**1286 20.7 11.7 1.7 L1HS_1_23c 52140 52429 (6356) C Ricksha_c DNA/MuDR (1383) 665 347 77**

**344 35.5 4.1 3.2 L1HS_1_23c 52522 52742 (6043) + LTR33A_ LTR/ERVL 204 426 (105) 78**

**234 34.2 8.6 2.0 L1HS_1_23c 52962 53158 (5627) C LTR78 LTR/ERV1 (357) 947 738 79**

**305 34.2 6.5 7.9 L1HS_1_23c 54209 54232 (4553) + L1M5 LINE/L1 3643 3666 (2480) 80**

**2016 16.5 2.1 0.0 L1HS_1_23c 54233 54572 (4213) C Tigger3a DNA/MER2_type (0) 348 2 81**

**305 34.2 6.5 7.9 L1HS_1_23c 54573 55219 (3566) + L1M5 LINE/L1 3667 4306 (1840) 80**

**1810 15.9 3.9 0.0 L1HS_1_23c 55220 55502 (3283) C AluJb SINE/Alu (18) 294 1 82**

**305 34.2 6.5 7.9 L1HS_1_23c 55503 55661 (3124) + L1M5 LINE/L1 4307 4462 (1684) 80**

**7497 4.5 0.8 0.1 L1HS_1_23c 55728 57415 (1370) + L1PA4 LINE/L1 4452 6154 (1) 83**

**539 25.7 5.5 0.0 L1HS_1_23c 57522 57704 (1081) + L1M5 LINE/L1 4467 4659 (1487) 80**

**25 7.5 0.0 0.0 L1HS_1_23c 57842 57894 (891) + AT_rich Low_complexity 1 53 (0) 84**

**634 28.1 14.0 0.4 L1HS_1_23c 58287 58515 (270) + L1M5 LINE/L1 5397 5656 (468) 80**

**225 13.7 1.3 7.6 L1HS_1_23c 58533 58611 (174) + (TA)n Simple_repeat 2 75 (0) 85**

**_________________________________________________________________________**

**40. L1HS_1_28**

**Ortholog annotation M_INTER_RMD Length 2742 nscore 0.36 NPOSITIONS 2162 2171 ;**

**Repeat length (main genome) 1424**

**Blast2 Results -**

**OFC OLC RFC RLC**

**1 1424 1 1424**

**2172 2742 853 1423**

**Ortholog Repeat Masker annotation**

**SW perc perc perc query position in query matching repeat position in repeat**

**score div. del. ins. sequence begin end (left) repeat class/family begin end (left) ID**

**15171 3.7 0.3 0.0 L1HS_1_28 1 2161 (581) + L1P1 LINE/L1 1839 4004 (2142) 1**

**4720 5.0 0.0 0.0 L1HS_1_28 2144 2742 (0) + L1P1 LINE/L1 2663 3261 (2885) 1 ***

**_________________________________________________________________________**

**41. L1HS_1_3**

**Ortholog annotation INDEL_CAN Length 3110 nscore 0.00 NPOSITIONS NA**

**Repeat length (main genome) 122**

**Blast2 Results -**

**OFC OLC RFC RLC**

**2716 2827 5 116**

**Ortholog Repeat Masker annotation**

**SW perc perc perc query position in query matching repeat position in repeat**

**score div. del. ins. sequence begin end (left) repeat class/family begin end (left) ID**

**4255 8.8 0.8 0.0 L1HS_1_3 1 589 (2521) + THE1A-int LTR/MaLR 987 1580 (0) 1**

**2591 10.7 0.3 0.0 L1HS_1_3 590 943 (2167) + THE1A LTR/MaLR 1 355 (0) 1**

**785 24.0 10.3 3.7 L1HS_1_3 1250 1327 (1783) C L1M5 LINE/L1 (2107) 4039 3956 2**

**387 0.0 0.0 0.0 L1HS_1_3 1328 1370 (1740) + (TCTA)n Simple_repeat 4 46 (0) 3**

**785 24.0 10.3 3.7 L1HS_1_3 1371 1698 (1412) C L1M5 LINE/L1 (2191) 3955 3606 2**

**374 26.7 0.8 5.5 L1HS_1_3 1874 2000 (1110) + MIR SINE/MIR 101 221 (41) 4**

**3028 7.0 0.0 0.0 L1HS_1_3 2710 3110 (0) + L1P1 LINE/L1 1 401 (5754) 5**

**_________________________________________________________________________**

**42. L1HS_1_40c**

**Ortholog annotation C_DISRUPTED_M_INTER_RMD Length 1262 nscore 0.79 NPOSITIONS 250 259 ;**

**Repeat length (main genome) 1074**

**Blast2 Results -**

**OFC OLC RFC RLC**

**1 249 1 251**

**260 1262 72 1074**

**Ortholog Repeat Masker annotation**

**SW perc perc perc query position in query matching repeat position in repeat**

**score div. del. ins. sequence begin end (left) repeat class/family begin end (left) ID**

**1929 5.6 0.8 0.0 L1HS_1_40c 1 249 (1013) + L1HS LINE/L1 5 255 (5777) 1**

**8165 5.2 0.5 0.1 L1HS_1_40c 260 1262 (0) + L1HS LINE/L1 76 1082 (4950) 1**

**_________________________________________________________________________**

**43. L1HS_1_45**

**Ortholog annotation C_INTER_RMD_M_DISRUPTED Length 2044 nscore 0.59 NPOSITIONS 561 561 ; 571 571 ; 611 620 ;**

**Repeat length (main genome) 2084**

**Blast2 Results -**

**OFC OLC RFC RLC**

**1 610 1 612**

**621 2043 650 2069**

**Ortholog Repeat Masker annotation**

**SW perc perc perc query position in query matching repeat position in repeat**

**score div. del. ins. sequence begin end (left) repeat class/family begin end (left) ID**

**15823 5.3 1.6 0.1 L1HS_1_45 1 2043 (1) + L1HS LINE/L1 4 2078 (3954) 1**

**_________________________________________________________________________**

**44. L1HS_1_48**

**Ortholog annotation M_INTRA_RMD Length 7614 nscore 9.51 NPOSITIONS 781 790 ; 2757 3470 ;**

**Repeat length (main genome) 3375**

**Blast2 Results -**

**OFC OLC RFC RLC**

**2 780 2 778**

**3471 5981 866 3375**

**Ortholog Repeat Masker annotation**

**SW perc perc perc query position in query matching repeat position in repeat**

**score div. del. ins. sequence begin end (left) repeat class/family begin end (left) ID**

**6211 5.7 0.1 0.1 L1HS_1_48 2 780 (6834) + L1HS LINE/L1 13 791 (5241) 1**

**5918 10.1 1.7 0.9 L1HS_1_48 791 1437 (6177) + L1PA11 LINE/L1 5135 5783 (391) 2**

**2198 9.9 0.0 4.4 L1HS_1_48 1438 1753 (5861) + AluSg SINE/Alu 1 302 (8) 3**

**5918 12.2 1.6 1.5 L1HS_1_48 1754 2142 (5472) + L1PA11 LINE/L1 5784 6172 (2) 2**

**3312 17.8 0.7 2.0 L1HS_1_48 2151 2752 (4862) + Tigger2 DNA/MER2_type 339 932 (1786) 4**

**25179 3.0 0.1 0.1 L1HS_1_48 3471 7614 (0) + L1HS LINE/L1 1002 5148 (998) 5**

**_________________________________________________________________________**

**45. L1HS_1_61**

**Ortholog annotation C_INTER_RMD_M_DISRUPTED Length 284 nscore 0.00 NPOSITIONS NA**

**Repeat length (main genome) 686**

**Blast2 Results -**

**OFC OLC RFC RLC**

**1 284 1 284**

**Ortholog Repeat Masker annotation**

**SW perc perc perc query position in query matching repeat position in repeat**

**score div. del. ins. sequence begin end (left) repeat class/family begin end (left) ID**

**2341 4.9 3.2 0.0 L1HS_1_61 1 284 (0) + L1P2 LINE/L1 5477 5769 (385) 1**

**_________________________________________________________________________**

**46. L1HS_1_63**

**Ortholog annotation C_INTER_RMD_M_DISRUPTED Length 3157 nscore 0.00 NPOSITIONS NA**

**Repeat length (main genome) 4183**

**Blast2 Results -**

**OFC OLC RFC RLC**

**7 3155 7 3151**

**Ortholog Repeat Masker annotation**

**SW perc perc perc query position in query matching repeat position in repeat**

**score div. del. ins. sequence begin end (left) repeat class/family begin end (left) ID**

**23869 5.0 0.1 0.3 L1HS_1_63 1 3155 (2) + L1P2 LINE/L1 1996 5125 (1021) 1**

**_________________________________________________________________________**

**47. L1HS_1_69c**

**Ortholog annotation INDEL_CAN Length 1553 nscore 0.00 NPOSITIONS NA**

**Repeat length (main genome) 1811**

**Blast2 Results -**

**OFC OLC RFC RLC**

**1 1429 382 1810**

**Ortholog Repeat Masker annotation**

**SW perc perc perc query position in query matching repeat position in repeat**

**score div. del. ins. sequence begin end (left) repeat class/family begin end (left) ID**

**7990 1.6 0.0 0.0 L1HS_1_69c 1 1429 (124) + L1PA2 LINE/L1 4726 6154 (1) 1**

**219 6.9 0.0 0.0 L1HS_1_69c 1447 1475 (78) + (A)n Simple_repeat 1 29 (0) 2**

**_________________________________________________________________________**

**48. L1HS_1_86c**

**Ortholog annotation INDEL_CAN Length 8077 nscore 0.25 NPOSITIONS 1 10 ; 4870 4879 ;**

**Repeat length (main genome) 764**

**Blast2 Results -**

**OFC OLC RFC RLC**

**1417 2181 1 764**

**Ortholog Repeat Masker annotation**

**SW perc perc perc query position in query matching repeat position in repeat**

**score div. del. ins. sequence begin end (left) repeat class/family begin end (left) ID**

**837 22.9 5.9 0.3 L1HS_1_86c 368 656 (7421) + L1MDa LINE/L1 2027 2331 (4302) 1**

**330 16.7 0.0 0.0 L1HS_1_86c 658 717 (7360) + (TA)n Simple_repeat 1 60 (0) 2**

**376 19.8 3.5 1.8 L1HS_1_86c 718 830 (7247) + L1MDa LINE/L1 2321 2435 (4198) 1 ***

**4790 3.9 0.2 0.0 L1HS_1_86c 829 1417 (6660) C L1HS LINE/L1 (4902) 1253 664 3 ***

**12759 2.8 0.0 0.1 L1HS_1_86c 1417 3820 (4257) + L1HS LINE/L1 1246 3647 (2499) 3**

**2526 1.7 0.0 0.0 L1HS_1_86c 3821 4111 (3966) + L1PA2 LINE/L1 5865 6155 (0) 4**

**6113 5.4 0.1 0.1 L1HS_1_86c 4123 4885 (3192) + L1P1 LINE/L1 3489 4251 (1895) 5 ***

**22311 2.8 0.0 0.0 L1HS_1_86c 4880 8077 (0) + L1PA3 LINE/L1 2711 5908 (247) 6**

**_________________________________________________________________________**

**49. L1HS_20_10c**

**Ortholog annotation INDEL_PTS Length 1372 nscore 0.00 NPOSITIONS NA**

**Repeat length (main genome) 4424**

**Blast2 Results -**

**OFC OLC RFC RLC**

**Ortholog Repeat Masker annotation**

**SW perc perc perc query position in query matching repeat position in repeat**

**score div. del. ins. sequence begin end (left) repeat class/family begin end (left) ID**

**1696 19.3 5.6 1.9 L1HS_20_10c 122 481 (891) + MLT1A LTR/MaLR 1 373 (1) 1**

**809 21.1 10.9 0.5 L1HS_20_10c 570 779 (593) C MIR SINE/MIR (0) 262 31 2**

**_________________________________________________________________________**

**50. L1HS_20_19**

**Ortholog annotation C_INTER_RMD Length 2088 nscore 0.00 NPOSITIONS NA**

**Repeat length (main genome) 3984**

**Blast2 Results -**

**OFC OLC RFC RLC**

**297 1074 1 776**

**1221 1452 778 1008**

**1452 2084 3345 3979**

**Ortholog Repeat Masker annotation**

**SW perc perc perc query position in query matching repeat position in repeat**

**score div. del. ins. sequence begin end (left) repeat class/family begin end (left) ID**

**921 17.6 0.6 3.3 L1HS_20_19 27 208 (1880) + LTR38 LTR/ERV1 378 554 (2) 1**

**8868 5.1 0.0 2.2 L1HS_20_19 297 1455 (633) + L1P1 LINE/L1 2 1135 (5020) 2**

**5206 2.8 0.3 0.0 L1HS_20_19 1452 2088 (0) + L1P1 LINE/L1 3469 4107 (2039) 2 ***

**_________________________________________________________________________**

**51. L1HS_20_1c**

**Ortholog annotation INDEL_CAN Length 188 nscore 0.00 NPOSITIONS NA**

**Repeat length (main genome) 904**

**Blast2 Results -**

**OFC OLC RFC RLC**

**no hits found**

**Ortholog Repeat Masker annotation**

**SW perc perc perc query position in query matching repeat position in repeat**

**score div. del. ins. sequence begin end (left) repeat class/family begin end (left) ID**

**629 15.6 27.7 4.3 L1HS_20_1c 1 188 (0) + MSTA LTR/MaLR 125 356 (72) 1**

**_________________________________________________________________________**

**52. L1HS_20_6**

**Ortholog annotation INDEL_PTS Length 675 nscore 0.00 NPOSITIONS NA**

**Repeat length (main genome) 36**

**Blast2 Results -**

**OFC OLC RFC RLC**

**Ortholog Repeat Masker annotation**

**SW perc perc perc query position in query matching repeat position in repeat**

**score div. del. ins. sequence begin end (left) repeat class/family begin end (left) ID**

**2839 1.3 0.0 0.0 L1HS_20_6 372 675 (0) C AluY SINE/Alu (6) 305 2 1**

**_________________________________________________________________________**

**53. L1HS_22_2c**

**Ortholog annotation INDEL_CAN Length 154 nscore 0.00 NPOSITIONS NA**

**Repeat length (main genome) 340**

**Blast2 Results -**

**OFC OLC RFC RLC**

**no hits found**

**Ortholog Repeat Masker annotation**

**SW perc perc perc query position in query matching repeat position in repeat**

**score div. del. ins. sequence begin end (left) repeat class/family begin end (left) ID**

**1365 1.3 0.0 0.0 L1HS_22_2c 1 154 (0) C L1HS LINE/L1 (4087) 1945 1792 1**

**_________________________________________________________________________**

**54. L1HS_2_15**

**Ortholog annotation C_INTRA_RMD Length 3285 nscore 0.30 NPOSITIONS 788 797 ;**

**Repeat length (main genome) 3402**

**Blast2 Results -**

**OFC OLC RFC RLC**

**1 778 1 773**

**798 3285 914 3401**

**Ortholog Repeat Masker annotation**

**SW perc perc perc query position in query matching repeat position in repeat**

**score div. del. ins. sequence begin end (left) repeat class/family begin end (left) ID**

**6004 4.1 0.6 0.0 L1HS_2_15 1 787 (2498) + L1HS LINE/L1 738 1529 (4626) 1**

**16205 2.5 0.1 0.0 L1HS_2_15 798 3285 (0) + L1HS LINE/L1 1661 4151 (1995) 1**

**_________________________________________________________________________**

**55. L1HS_2_17**

**Ortholog annotation C_INTER_RMD_M_DISRUPTED Length 2304 nscore 0.00 NPOSITIONS NA**

**Repeat length (main genome) 1348**

**Blast2 Results -**

**OFC OLC RFC RLC**

**1 1338 1 1342**

**Ortholog Repeat Masker annotation**

**SW perc perc perc query position in query matching repeat position in repeat**

**score div. del. ins. sequence begin end (left) repeat class/family begin end (left) ID**

**13284 2.5 0.1 0.1 L1HS_2_17 1 1767 (537) + L1HS LINE/L1 702 2469 (3677) 1**

**622 16.8 0.9 0.0 L1HS_2_17 1768 1880 (424) C MER67D LTR/ERV1 (400) 114 1 2**

**638 24.1 1.3 0.6 L1HS_2_17 1886 2044 (260) C L1M4 LINE/L1 (537) 5641 5482 3**

**907 13.7 7.2 10.9 L1HS_2_17 2045 2265 (39) + MLT1A1 LTR/MaLR 1 213 (195) 4**

**_________________________________________________________________________**

**56. L1HS_2_3**

**Ortholog annotation C_INTER_RMD_M_DISRUPTED Length 133 nscore 0.00 NPOSITIONS NA**

**Repeat length (main genome) 1882**

**Blast2 Results -**

**OFC OLC RFC RLC**

**1 133 1749 1881**

**Ortholog Repeat Masker annotation**

**SW perc perc perc query position in query matching repeat position in repeat**

**score div. del. ins. sequence begin end (left) repeat class/family begin end (left) ID**

**708 18.8 0.8 0.0 L1HS_2_3 1 133 (0) + L1MA9 LINE/L1 6178 6311 (1) 1**

**_________________________________________________________________________**

**57. L1HS_2_55**

**Ortholog annotation INDEL_CAN Length 385 nscore 0.00 NPOSITIONS NA**

**Repeat length (main genome) 2958**

**Blast2 Results -**

**OFC OLC RFC RLC**

**no hits found**

**Ortholog Repeat Masker annotation**

**There were no repetitive sequences detected in /home/vipin/L1HS_TR_CHR/Chimp/CONFIRMATION/L1HS_TR_INDEL_SEQUENCES/L1HS_2_55**

**_________________________________________________________________________**

**58. L1HS_2_5c**

**Ortholog annotation C_INTER_RMD_M_DISRUPTED Length 60062 nscore 1.91 NPOSITIONS 366 441 ; 12320 12525 ; 12559 12560 ; 16028 16398 ; 19801 20288 ; 20370 20370 ; 20389 20390 ;**

**Repeat length (main genome) 2032**

**Blast2 Results -**

**OFC OLC RFC RLC**

**58034 60062 3 2031**

**Ortholog Repeat Masker annotation**

**SW perc perc perc query position in query matching repeat position in repeat**

**score div. del. ins. sequence begin end (left) repeat class/family begin end (left) ID**

**1450 7.1 0.0 0.0 L1HS_2_5c 3 185 (59877) + L1PA6 LINE/L1 5833 6015 (139) 1**

**475 24.1 1.5 0.0 L1HS_2_5c 184 320 (59742) + L1P1 LINE/L1 4580 4718 (1428) 2 ***

**250 13.3 0.0 0.0 L1HS_2_5c 319 363 (59699) C L1PREC2 LINE/L1 (3451) 2695 2651 3 ***

**709 13.3 3.0 2.3 L1HS_2_5c 599 729 (59333) + L1PA6 LINE/L1 6023 6154 (0) 4**

**234 28.9 10.5 2.1 L1HS_2_5c 828 1018 (59044) + L1MC4a LINE/L1 5086 5292 (854) 5**

**1119 27.4 16.9 3.1 L1HS_2_5c 1093 1608 (58454) + L1MC4a LINE/L1 5355 5941 (1941) 5**

**210 25.3 3.5 7.1 L1HS_2_5c 1789 1873 (58189) C MIR3 SINE/MIR (13) 195 114 6**

**2364 16.7 11.8 0.2 L1HS_2_5c 1908 2356 (57706) C MLT1D LTR/MaLR (4) 501 1 7**

**3386 13.5 7.1 1.8 L1HS_2_5c 2365 2928 (57134) + MER41E LTR/ERV1 1 594 (1) 8**

**1711 26.1 8.8 4.7 L1HS_2_5c 2958 3992 (56070) C L2a LINE/L2 (54) 3372 2296 9**

**3656 14.2 3.5 2.5 L1HS_2_5c 3994 4621 (55441) C MER41B LTR/ERV1 (0) 634 1 10**

**22 0.0 0.0 0.0 L1HS_2_5c 4835 4856 (55206) + AT_rich Low_complexity 1 22 (0) 11**

**437 29.4 18.8 0.0 L1HS_2_5c 5868 6064 (53998) + MIRb SINE/MIR 9 242 (26) 12**

**605 30.9 7.2 0.5 L1HS_2_5c 7113 7307 (52755) + MLT1L LTR/MaLR 337 544 (66) 13**

**413 18.8 1.2 0.0 L1HS_2_5c 7338 7422 (52640) + MLT1L LTR/MaLR 525 610 (0) 13**

**561 27.8 5.6 0.0 L1HS_2_5c 7818 7961 (52101) + MER91A DNA/Tip100 38 189 (7) 14**

**1107 24.9 9.7 1.5 L1HS_2_5c 8143 8481 (51581) + LTR82A LTR/ERVL 8 374 (498) 15**

**2247 10.5 0.0 1.0 L1HS_2_5c 9072 9370 (50692) C AluSg SINE/Alu (14) 296 1 16**

**380 26.8 9.2 2.9 L1HS_2_5c 9475 9647 (50415) + LTR81A LTR/ERV1? 69 252 (1098) 17**

**267 33.3 16.2 0.7 L1HS_2_5c 9962 10103 (49959) + LTR81A LTR/ERV1? 515 678 (475) 17**

**23 0.0 0.0 0.0 L1HS_2_5c 10368 10390 (49672) + AT_rich Low_complexity 1 23 (0) 18**

**1897 9.9 3.2 2.9 L1HS_2_5c 10741 11218 (48844) C MER51A LTR/ERV1 (0) 634 1 19**

**721 22.9 4.7 0.6 L1HS_2_5c 11219 11389 (48673) + MIR SINE/MIR 64 241 (21) 20**

**29 7.0 0.0 0.0 L1HS_2_5c 11894 11950 (48112) + AT_rich Low_complexity 1 57 (0) 21**

**1764 14.3 1.0 3.1 L1HS_2_5c 12031 12319 (47743) C AluSx SINE/Alu (7) 305 23 22**

**475 28.6 10.7 3.7 L1HS_2_5c 13023 13265 (46797) + MER112 DNA/MER1_type 1 260 (1) 23**

**727 32.8 3.3 0.3 L1HS_2_5c 13298 13658 (46404) + L1ME4a LINE/L1 5532 5903 (221) 24**

**358 18.1 13.5 2.1 L1HS_2_5c 13703 13798 (46264) + L1ME4a LINE/L1 5993 6099 (25) 24**

**2525 18.3 1.2 3.3 L1HS_2_5c 14120 14633 (45429) C MLT1D LTR/MaLR (2) 503 1 25**

**775 21.9 0.0 7.1 L1HS_2_5c 15436 15471 (44591) C AluJo/FRAM SINE/Alu (3) 309 276 26**

**1610 12.9 17.2 0.0 L1HS_2_5c 15472 15727 (44335) + AluJo SINE/Alu 1 300 (2) 27**

**775 21.9 0.0 7.1 L1HS_2_5c 15728 15872 (44190) C FRAM SINE/Alu (37) 139 5 26**

**236 33.7 2.1 1.1 L1HS_2_5c 16443 16535 (43527) + L2c LINE/L2 3060 3153 (234) 28**

**215 27.7 3.1 0.0 L1HS_2_5c 16671 16735 (43327) C MIR SINE/MIR (57) 151 85 29**

**299 31.0 15.3 4.7 L1HS_2_5c 16879 17179 (42883) C L2b LINE/L2 (11) 3376 3044 30**

**225 26.4 6.3 4.2 L1HS_2_5c 17148 17242 (42820) C MIRb SINE/MIR (7) 261 165 31 ***

**193 25.0 0.0 0.0 L1HS_2_5c 17215 17254 (42808) C MIRc SINE/MIR (70) 198 159 32 ***

**737 20.2 12.8 0.5 L1HS_2_5c 17336 17539 (42523) C MIR SINE/MIR (30) 232 4 33**

**566 28.1 0.7 0.0 L1HS_2_5c 17714 17848 (42214) C MIRb SINE/MIR (100) 168 33 31**

**2171 15.4 2.3 0.3 L1HS_2_5c 18452 18803 (41259) + MLT1A0 LTR/MaLR 5 363 (2) 34**

**1934 13.1 0.0 0.4 L1HS_2_5c 19526 19800 (40262) + AluSx SINE/Alu 1 274 (38) 35**

**333 28.6 3.4 0.0 L1HS_2_5c 20579 20697 (39365) C MER102c DNA/MER1_type (0) 331 209 36**

**3320 21.0 0.8 0.8 L1HS_2_5c 20766 21412 (38650) C MER82 DNA/MER2_type (6) 647 1 37**

**302 20.9 26.7 0.9 L1HS_2_5c 21641 21756 (38306) C LTR33A_ LTR/ERVL (362) 169 24 38**

**478 22.3 13.0 3.1 L1HS_2_5c 22745 22906 (37156) + MER5B DNA/MER1_type 1 178 (0) 39**

**234 14.3 20.6 0.0 L1HS_2_5c 23189 23251 (36811) C L2c LINE/L2 (19) 3368 3293 40**

**835 14.5 0.0 5.5 L1HS_2_5c 23293 23438 (36624) C FLAM_C SINE/Alu (5) 138 1 41**

**1268 19.3 10.1 10.4 L1HS_2_5c 23704 24068 (35994) + MLT1A LTR/MaLR 9 372 (2) 42**

**514 24.3 1.9 0.0 L1HS_2_5c 24112 24218 (35844) C MER81 DNA/AcHobo (5) 109 1 43**

**629 17.7 7.6 0.3 L1HS_2_5c 25072 25215 (34847) + MIR SINE/MIR 113 261 (1) 44**

**982 27.2 10.2 8.4 L1HS_2_5c 25361 25822 (34240) C MLT1K LTR/MaLR (115) 480 11 45**

**225 29.2 6.8 9.1 L1HS_2_5c 25902 26033 (34029) C MER5A DNA/MER1_type (24) 165 37 46**

**1489 14.0 9.8 1.6 L1HS_2_5c 26045 26298 (33764) C AluJb SINE/Alu (23) 289 15 47**

**207 11.4 0.0 0.0 L1HS_2_5c 26519 26553 (33509) + (TTCA)n Simple_repeat 4 38 (0) 48**

**272 29.0 1.4 0.0 L1HS_2_5c 26736 26804 (33258) + L2c LINE/L2 3257 3326 (61) 49**

**931 20.2 5.7 0.9 L1HS_2_5c 26848 27057 (33005) C Arthur1C DNA/Tip100 (2) 361 142 50**

**809 13.0 0.9 0.0 L1HS_2_5c 27058 27236 (32826) C FLAM_C SINE/Alu (0) 143 3 51**

**714 25.0 2.4 1.9 L1HS_2_5c 27433 27644 (32418) + LTR33 LTR/ERVL 293 505 (10) 52**

**2156 10.1 0.3 0.3 L1HS_2_5c 27817 28104 (31958) C AluSc SINE/Alu (21) 288 1 53**

**604 24.7 4.8 2.4 L1HS_2_5c 28508 28673 (31389) C MLT1K LTR/MaLR (26) 569 400 54**

**223 25.0 0.0 0.0 L1HS_2_5c 28674 28725 (31337) + CT-rich Low_complexity 1 52 (0) 55**

**373 26.6 10.4 15.6 L1HS_2_5c 28871 29062 (31000) C MLT1K LTR/MaLR (413) 182 1 54**

**223 30.2 7.8 0.0 L1HS_2_5c 29187 29302 (30760) + MIR3 SINE/MIR 60 184 (24) 56**

**3206 15.7 6.3 1.4 L1HS_2_5c 30527 31158 (28904) C Charlie1a DNA/MER1_type (0) 1455 793 57**

**206 23.9 0.0 1.4 L1HS_2_5c 31162 31233 (28829) + (TATAA)n Simple_repeat 4 74 (0) 58**

**2906 20.4 3.1 4.9 L1HS_2_5c 31467 32176 (27886) C Charlie1a DNA/MER1_type (677) 778 82 57**

**481 29.5 29.3 0.3 L1HS_2_5c 32177 32485 (27577) C L2 LINE/L2 (2127) 1292 894 59**

**226 22.1 4.3 0.0 L1HS_2_5c 32516 32588 (27474) C MIRb SINE/MIR (0) 262 182 60**

**22 0.0 0.0 0.0 L1HS_2_5c 32591 32612 (27450) + AT_rich Low_complexity 1 22 (0) 61**

**350 16.2 8.7 1.4 L1HS_2_5c 32618 32686 (27376) + LTR54 LTR/ERV1 5 78 (432) 62**

**414 22.2 6.4 14.1 L1HS_2_5c 32701 33006 (27056) + MER57B2 LTR/ERV1 125 385 (0) 63**

**620 17.2 4.0 2.4 L1HS_2_5c 33147 33271 (26791) C L1M4b LINE/L1 (7076) 258 132 64**

**342 0.0 0.0 0.0 L1HS_2_5c 33512 33549 (26513) + (TA)n Simple_repeat 2 39 (0) 65**

**1639 16.3 1.0 5.0 L1HS_2_5c 33552 33710 (26352) C AluJo SINE/Alu (21) 291 138 66**

**2114 8.7 3.4 3.0 L1HS_2_5c 33711 34006 (26056) C AluY SINE/Alu (14) 297 1 67**

**1639 16.3 1.0 5.0 L1HS_2_5c 34007 34149 (25913) C AluJo SINE/Alu (175) 137 1 66**

**2165 9.4 3.1 0.0 L1HS_2_5c 34348 34633 (25429) C AluSc SINE/Alu (14) 295 1 68**

**837 9.5 0.0 0.0 L1HS_2_5c 34720 34824 (25238) + AluSg/x SINE/Alu 193 297 (15) 69**

**1146 27.3 6.2 1.7 L1HS_2_5c 34852 35257 (24805) + L1MC4a LINE/L1 5643 6066 (1816) 70**

**849 26.7 6.3 0.0 L1HS_2_5c 35403 35623 (24439) C MIRb SINE/MIR (8) 260 26 71**

**698 14.0 0.0 0.0 L1HS_2_5c 35699 35798 (24264) + AluJ SINE/Alu 5 104 (208) 72**

**1876 9.2 14.1 0.7 L1HS_2_5c 35799 36082 (23980) C LTR12C LTR/ERV1 (2) 1576 1255 73**

**5705 9.2 3.7 4.9 L1HS_2_5c 36086 36918 (23144) C LTR12 LTR/ERV1 (3) 823 1 74**

**6493 5.5 0.4 0.0 L1HS_2_5c 36949 37715 (22347) C HERV9-int LTR/ERV1 (3) 8433 7664 74**

**3304 5.3 3.8 1.2 L1HS_2_5c 37714 38135 (21927) C HERVIP10FH-int LTR/ERV1 (3238) 1864 1432 75 ***

**2004 11.3 4.5 1.6 L1HS_2_5c 38118 38495 (21567) C HERVIP10FH-int LTR/ERV1 (5623) 1273 866 75 ***

**41418 5.8 0.2 0.0 L1HS_2_5c 38482 43391 (16671) C HERV9-int LTR/ERV1 (3473) 4963 46 74**

**5351 6.8 3.6 0.0 L1HS_2_5c 43435 44098 (15964) C LTR12_ LTR/ERV1 (0) 688 1 76**

**1085 16.7 3.7 0.5 L1HS_2_5c 44099 44285 (15777) + AluJb SINE/Alu 99 291 (21) 77**

**1076 28.9 2.9 0.0 L1HS_2_5c 44572 44848 (15214) C MER115 DNA/Tip100 (0) 693 409 78**

**2133 13.5 0.3 0.0 L1HS_2_5c 44885 45181 (14881) + AluSx SINE/Alu 1 298 (14) 79**

**3075 7.2 2.7 0.0 L1HS_2_5c 45182 45582 (14480) + L1PA7 LINE/L1 5740 6151 (3) 80**

**965 31.0 8.3 2.1 L1HS_2_5c 45819 46157 (13905) C MER115 DNA/Tip100 (325) 368 9 78**

**226 25.4 2.8 0.0 L1HS_2_5c 46534 46604 (13458) C MLT1M LTR/MaLR (12) 660 588 81**

**1536 21.7 3.9 5.2 L1HS_2_5c 47377 47772 (12290) + MLT1I LTR/MaLR 6 398 (13) 82**

**717 24.3 0.0 17.5 L1HS_2_5c 47773 47897 (12165) + MER5A DNA/MER1_type 1 104 (85) 83**

**1567 15.4 0.0 3.1 L1HS_2_5c 47898 48151 (11911) + AluJo SINE/Alu 1 246 (66) 84**

**717 24.3 0.0 17.5 L1HS_2_5c 48152 48254 (11808) + MER5A DNA/MER1_type 105 189 (0) 83**

**1536 21.7 3.9 5.2 L1HS_2_5c 48255 48265 (11797) + MLT1I LTR/MaLR 399 408 (3) 82**

**299 29.9 4.5 11.5 L1HS_2_5c 48417 48616 (11446) C MLT1M LTR/MaLR (472) 200 15 81**

**268 13.2 0.0 0.0 L1HS_2_5c 49373 49410 (10652) + tRNA-Met_ tRNA 1 38 (38) 85**

**27 2.9 0.0 0.0 L1HS_2_5c 49694 49727 (10335) + AT_rich Low_complexity 1 34 (0) 86**

**2110 12.1 0.7 0.0 L1HS_2_5c 49736 50038 (10024) + AluSx SINE/Alu 1 312 (0) 87**

**344 16.7 1.4 0.0 L1HS_2_5c 50138 50209 (9853) + (TA)n Simple_repeat 1 73 (0) 88**

**1713 16.6 0.3 2.1 L1HS_2_5c 50210 50499 (9563) C AluJo SINE/Alu (27) 285 1 89**

**287 22.5 1.4 2.7 L1HS_2_5c 50527 50599 (9463) C MER5B DNA/MER1_type (0) 178 107 90**

**369 29.5 2.7 2.7 L1HS_2_5c 50673 50822 (9240) C MER65B LTR/ERV1 (118) 355 206 91**

**242 25.8 3.0 0.0 L1HS_2_5c 50888 50953 (9109) C MER65B LTR/ERV1 (384) 147 80 91**

**471 18.9 4.6 6.9 L1HS_2_5c 50960 51090 (8972) C MER65B LTR/ERV1 (403) 128 1 91**

**645 16.0 7.2 12.4 L1HS_2_5c 51126 51198 (8864) + MER53 DNA/hAT 7 76 (117) 92**

**2108 12.8 0.0 0.3 L1HS_2_5c 51199 51519 (8543) + AluSx SINE/Alu 1 312 (0) 93**

**645 16.0 7.2 12.4 L1HS_2_5c 51520 51635 (8427) + MER53 DNA/hAT 77 189 (4) 92**

**4068 3.1 0.2 0.2 L1HS_2_5c 52500 52978 (7084) C L1PA3 LINE/L1 (0) 6155 5677 94**

**3973 3.2 1.0 0.4 L1HS_2_5c 52979 53475 (6587) + L1P1 LINE/L1 2934 3433 (2713) 95**

**516 27.3 6.4 2.0 L1HS_2_5c 54498 54733 (5329) C MIR SINE/MIR (0) 262 19 96**

**244 28.4 12.1 6.1 L1HS_2_5c 55023 55187 (4875) C MamGypLTR2c LTR/Gypsy (726) 342 168 97**

**2124 10.9 0.7 0.0 L1HS_2_5c 55284 55566 (4496) C AluSx SINE/Alu (23) 289 5 98**

**387 20.0 0.0 8.3 L1HS_2_5c 55854 55923 (4139) C MLT2D LTR/ERVL (0) 556 492 99**

**201 3.9 0.0 0.0 L1HS_2_5c 55924 55949 (4113) + (GA)n Simple_repeat 1 26 (0) 100**

**387 20.0 0.0 8.3 L1HS_2_5c 55950 55987 (4075) C MLT2D LTR/ERVL (65) 491 457 99 ***

**1019 16.4 2.2 1.1 L1HS_2_5c 55983 56167 (3895) C MLT2D LTR/ERVL (227) 187 1 99**

**210 36.5 2.1 5.2 L1HS_2_5c 56532 56765 (3297) C L2b LINE/L2 (227) 3160 2931 101**

**2104 12.5 0.0 1.4 L1HS_2_5c 57436 57728 (2334) + AluSx SINE/Alu 1 289 (23) 102**

**434 27.6 15.0 3.5 L1HS_2_5c 57744 57916 (2146) C MIR SINE/MIR (30) 232 40 103**

**17462 3.1 0.1 0.1 L1HS_2_5c 58034 60062 (0) + L1HS LINE/L1 7 2035 (3997) 104**

**_________________________________________________________________________**

**59. L1HS_2_72c**

**Ortholog annotation C_DISRUPTED_M_INTER_RMD Length 3486 nscore 19.10 NPOSITIONS 1209 1873 ; 1987 1987 ;**

**Repeat length (main genome) 3172**

**Blast2 Results -**

**OFC OLC RFC RLC**

**1 1208 1 1217**

**1874 3486 1555 3172**

**Ortholog Repeat Masker annotation**

**SW perc perc perc query position in query matching repeat position in repeat**

**score div. del. ins. sequence begin end (left) repeat class/family begin end (left) ID**

**10069 1.9 0.8 0.1 L1HS_2_72c 1 1208 (2278) + L1HS LINE/L1 755 1971 (4184) 1**

**13376 2.0 0.3 0.0 L1HS_2_72c 1874 3486 (0) + L1HS LINE/L1 2309 3926 (2220) 1**

**_________________________________________________________________________**

**60. L1HS_2_83**

**Ortholog annotation INDEL_CAN Length 5936 nscore 0.00 NPOSITIONS NA**

**Repeat length (main genome) 5932**

**Blast2 Results -**

**OFC OLC RFC RLC**

**1026 5936 1 4902**

**Ortholog Repeat Masker annotation**

**SW perc perc perc query position in query matching repeat position in repeat**

**score div. del. ins. sequence begin end (left) repeat class/family begin end (left) ID**

**234 29.1 0.0 0.0 L1HS_2_83 21 75 (5861) + MamRep605 Unknown 556 610 (266) 1**

**1868 15.8 0.4 0.0 L1HS_2_83 154 432 (5504) + AluSx SINE/Alu 1 280 (32) 2**

**197 3.3 3.3 0.0 L1HS_2_83 434 463 (5473) + (CAAA)n Simple_repeat 1 31 (0) 3**

**23785 6.6 0.9 0.0 L1HS_2_83 886 5936 (0) + L1P1 LINE/L1 2 5096 (1050) 4**

**_________________________________________________________________________**

**61. L1HS_2_88**

**Ortholog annotation C_INTER_RMD_M_DISRUPTED Length 5179 nscore 0.79 NPOSITIONS 3531 3560 ; 5052 5061 ; 5075 5075 ;**

**Repeat length (main genome) 140**

**Blast2 Results -**

**OFC OLC RFC RLC**

**5062 5179 22 139**

**Ortholog Repeat Masker annotation**

**SW perc perc perc query position in query matching repeat position in repeat**

**score div. del. ins. sequence begin end (left) repeat class/family begin end (left) ID**

**242 31.5 5.4 1.6 L1HS_2_88 3 131 (5048) C MER110 LTR/ERV1 (90) 401 268 1**

**206 26.4 9.6 1.4 L1HS_2_88 621 693 (4486) C MIR SINE/MIR (6) 256 178 2**

**2325 12.6 2.8 0.6 L1HS_2_88 698 1049 (4130) + THE1B LTR/MaLR 5 364 (0) 3**

**234 24.5 0.0 0.0 L1HS_2_88 1062 1114 (4065) C MIRc SINE/MIR (131) 137 85 4**

**370 33.2 5.6 0.2 L1HS_2_88 1277 1426 (3753) C MIRc SINE/MIR (83) 193 36 5**

**1026 19.5 9.3 2.5 L1HS_2_88 1901 2149 (3030) C MLT1D LTR/MaLR (0) 505 220 6**

**743 28.7 5.5 4.6 L1HS_2_88 2151 2497 (2682) + L1MC4a LINE/L1 5587 5936 (1946) 7**

**21 0.0 0.0 0.0 L1HS_2_88 2520 2540 (2639) + AT_rich Low_complexity 1 21 (0) 8**

**28 0.0 0.0 0.0 L1HS_2_88 2552 2579 (2600) + AT_rich Low_complexity 1 28 (0) 9**

**746 22.5 9.0 11.0 L1HS_2_88 2694 3047 (2132) C MLT1B LTR/MaLR (0) 390 44 10**

**2575 9.5 12.0 0.0 L1HS_2_88 3048 3447 (1732) + LTR7 LTR/ERV1 1 448 (2) 11**

**400 6.6 14.5 0.0 L1HS_2_88 3450 3525 (1654) + HERVH-int LTR/ERV1 1 87 (7626) 11**

**9126 2.8 1.4 0.0 L1HS_2_88 3642 5050 (129) + L1HS LINE/L1 1082 2513 (3633) 12**

**1010 12.6 0.7 0.0 L1HS_2_88 5045 5179 (0) + L1HS LINE/L1 6 141 (5891) 13 ***

**_________________________________________________________________________**

**62. L1HS_3_10**

**Ortholog annotation C_INTRA_RMD Length 2271 nscore 14.88 NPOSITIONS 1100 1437 ;**

**Repeat length (main genome) 2424**

**Blast2 Results -**

**OFC OLC RFC RLC**

**1 945 1 947**

**1438 2271 1590 2423**

**Ortholog Repeat Masker annotation**

**SW perc perc perc query position in query matching repeat position in repeat**

**score div. del. ins. sequence begin end (left) repeat class/family begin end (left) ID**

**7860 5.7 2.2 0.2 L1HS_3_10 1 1089 (1182) + L1HS LINE/L1 124 1234 (4921) 1**

**4642 3.1 0.0 0.0 L1HS_3_10 1438 2271 (0) + L1HS LINE/L1 1713 2546 (3600) 1**

**_________________________________________________________________________**

**63. L1HS_3_108**

**Ortholog annotation INDEL_CAN Length 5740 nscore 0.00 NPOSITIONS NA**

**Repeat length (main genome) 4901**

**Blast2 Results -**

**OFC OLC RFC RLC**

**Ortholog Repeat Masker annotation**

**SW perc perc perc query position in query matching repeat position in repeat**

**score div. del. ins. sequence begin end (left) repeat class/family begin end (left) ID**

**286 12.1 1.5 1.5 L1HS_3_108 227 293 (5447) + (TAAA)n Simple_repeat 2 68 (0) 1**

**712 30.7 8.2 1.2 L1HS_3_108 410 653 (5087) + MIRb SINE/MIR 8 268 (0) 2**

**35 7.1 0.0 0.0 L1HS_3_108 976 1045 (4695) + AT_rich Low_complexity 1 70 (0) 3**

**1919 14.2 0.0 0.3 L1HS_3_108 1050 1331 (4409) C AluSx SINE/Alu (31) 281 1 4**

**303 30.8 5.5 2.0 L1HS_3_108 1596 1741 (3999) C MIRb SINE/MIR (0) 268 118 5**

**1718 21.9 6.8 3.4 L1HS_3_108 2988 3455 (2285) + MER66B LTR/ERV1 1 484 (2) 6**

**1974 15.4 1.5 0.6 L1HS_3_108 3544 3875 (1865) + MER1B DNA/MER1_type 5 339 (0) 7**

**311 0.0 0.0 2.5 L1HS_3_108 4777 4816 (924) + (ATG)n Simple_repeat 2 40 (0) 8**

**1018 28.7 1.8 6.2 L1HS_3_108 5331 5716 (24) + MLT1D LTR/MaLR 2 370 (135) 9**

**_________________________________________________________________________**

**64. L1HS_3_118**

**Ortholog annotation M_INTRA_RMD Length 2420 nscore 0.00 NPOSITIONS NA**

**Repeat length (main genome) 889**

**Blast2 Results -**

**OFC OLC RFC RLC**

**1 888 1 889**

**Ortholog Repeat Masker annotation**

**SW perc perc perc query position in query matching repeat position in repeat**

**score div. del. ins. sequence begin end (left) repeat class/family begin end (left) ID**

**17866 3.8 0.6 0.0 L1HS_3_118 1 2420 (0) + L1HS LINE/L1 126 2560 (3586) 1**

**_________________________________________________________________________**

**65. L1HS_3_14c**

**Ortholog annotation C_INTER_RMD_M_DISRUPTED Length 464 nscore 0.00 NPOSITIONS NA**

**Repeat length (main genome) 2806**

**Blast2 Results -**

**OFC OLC RFC RLC**

**1 464 2343 2806**

**Ortholog Repeat Masker annotation**

**SW perc perc perc query position in query matching repeat position in repeat**

**score div. del. ins. sequence begin end (left) repeat class/family begin end (left) ID**

**29 7.8 0.0 0.0 L1HS_3_14c 67 130 (334) + AT_rich Low_complexity 1 64 (0) 1**

**867 16.9 6.2 0.6 L1HS_3_14c 246 424 (40) + L1M2 LINE/L1 3370 3558 (2585) 2**

**_________________________________________________________________________**

**66. L1HS_3_17c**

**Ortholog annotation C_INTER_RMD_M_DISRUPTED Length 2139 nscore 0.00 NPOSITIONS NA**

**Repeat length (main genome) 2618**

**Blast2 Results -**

**OFC OLC RFC RLC**

**1 2139 1 2139**

**Ortholog Repeat Masker annotation**

**SW perc perc perc query position in query matching repeat position in repeat**

**score div. del. ins. sequence begin end (left) repeat class/family begin end (left) ID**

**15476 3.2 0.0 0.0 L1HS_3_17c 1 2139 (0) + L1P1 LINE/L1 3538 5676 (479) 1**

**_________________________________________________________________________**

**67. L1HS_3_20c**

**Ortholog annotation M_INTRA_RMD Length 6011 nscore 0.00 NPOSITIONS NA**

**Repeat length (main genome) 4002**

**Blast2 Results -**

**OFC OLC RFC RLC**

**3396 6011 1388 4002**

**Ortholog Repeat Masker annotation**

**SW perc perc perc query position in query matching repeat position in repeat**

**score div. del. ins. sequence begin end (left) repeat class/family begin end (left) ID**

**1029 28.8 9.6 2.8 L1HS_3_20c 1 813 (5198) + HAL1 LINE/L1 1526 2393 (114) 1**

**26 0.0 0.0 0.0 L1HS_3_20c 944 969 (5042) + AT_rich Low_complexity 1 26 (0) 2**

**2321 14.4 1.8 0.0 L1HS_3_20c 981 1370 (4641) C L1MA4 LINE/L1 (309) 5991 5595 3**

**10339 3.5 0.2 0.1 L1HS_3_20c 1374 3396 (2615) C L1PA3 LINE/L1 (0) 6155 4132 4 ***

**16656 3.8 0.0 0.0 L1HS_3_20c 3392 6011 (0) + L1PA3 LINE/L1 1528 4147 (1999) 4**

**_________________________________________________________________________**

**68. L1HS_3_24c**

**Ortholog annotation C_INTRA_RMD Length 486 nscore 0.00 NPOSITIONS NA**

**Repeat length (main genome) 3552**

**Blast2 Results -**

**OFC OLC RFC RLC**

**1 126 1 126**

**133 486 3199 3552**

**Ortholog Repeat Masker annotation**

**SW perc perc perc query position in query matching repeat position in repeat**

**score div. del. ins. sequence begin end (left) repeat class/family begin end (left) ID**

**937 7.0 0.8 0.0 L1HS_3_24c 1 128 (358) + L1P1 LINE/L1 2621 2749 (3397) 1**

**2493 3.1 0.3 10.2 L1HS_3_24c 133 486 (0) + L1PA3 LINE/L1 5837 6155 (0) 2**

**_________________________________________________________________________**

**69. L1HS_3_25c**

**Ortholog annotation C_INTER_RMD_M_DISRUPTED Length 6003 nscore 11.86 NPOSITIONS 833 842 ; 2648 2657 ; 5005 5696 ;**

**Repeat length (main genome) 741**

**Blast2 Results -**

**OFC OLC RFC RLC**

**5746 6003 484 741**

**Ortholog Repeat Masker annotation**

**SW perc perc perc query position in query matching repeat position in repeat**

**score div. del. ins. sequence begin end (left) repeat class/family begin end (left) ID**

**5852 2.8 0.0 0.0 L1HS_3_25c 149 832 (5171) C L1PA3 LINE/L1 (0) 6155 5472 1**

**16992 10.3 0.5 0.9 L1HS_3_25c 843 4987 (1016) C L1PA3 LINE/L1 (217) 5938 1855 2**

**2303 5.6 0.0 0.7 L1HS_3_25c 5697 6003 (0) + L1HS LINE/L1 1408 1712 (4320) 3**

**_________________________________________________________________________**

**70. L1HS_3_33**

**Ortholog annotation INDEL_PTS Length 111 nscore 0.00 NPOSITIONS NA**

**Repeat length (main genome) 102**

**Blast2 Results -**

**OFC OLC RFC RLC**

**no hits found**

**Ortholog Repeat Masker annotation**

**SW perc perc perc query position in query matching repeat position in repeat**

**score div. del. ins. sequence begin end (left) repeat class/family begin end (left) ID**

**219 6.9 0.0 0.0 L1HS_3_33 36 64 (47) + (CA)n Simple_repeat 2 30 (0) 1**

**28 0.0 0.0 0.0 L1HS_3_33 84 111 (0) + AT_rich Low_complexity 1 28 (0) 2**

**_________________________________________________________________________**

**71. L1HS_3_53**

**Ortholog annotation INDEL_CAN Length 72 nscore 0.00 NPOSITIONS NA**

**Repeat length (main genome) 123**

**Blast2 Results -**

**OFC OLC RFC RLC**

**no hits found**

**Ortholog Repeat Masker annotation**

**There were no repetitive sequences detected in /home/vipin/L1HS_TR_CHR/Chimp/CONFIRMATION/L1HS_TR_INDEL_SEQUENCES/L1HS_3_53**

**_________________________________________________________________________**

**72. L1HS_3_54**

**Ortholog annotation M_INTRA_RMD Length 5180 nscore 0.39 NPOSITIONS 511 520 ; 1555 1564 ;**

**Repeat length (main genome) 3655**

**Blast2 Results -**

**OFC OLC RFC RLC**

**2 500 2 494**

**528 1554 1349 2373**

**1601 3402 1858 3655**

**Ortholog Repeat Masker annotation**

**SW perc perc perc query position in query matching repeat position in repeat**

**score div. del. ins. sequence begin end (left) repeat class/family begin end (left) ID**

**3676 5.4 0.0 1.2 L1HS_3_54 2 506 (4674) + L1HS LINE/L1 125 623 (5532) 1**

**6301 2.6 0.1 0.0 L1HS_3_54 528 1554 (3626) + L1HS LINE/L1 1473 2500 (3646) 1**

**26179 2.5 0.2 0.0 L1HS_3_54 1567 5180 (0) + L1HS LINE/L1 1945 5566 (589) 2**

**_________________________________________________________________________**

**73. L1HS_3_57**

**Ortholog annotation C_INTER_RMD_M_DISRUPTED Length 1163 nscore 0.00 NPOSITIONS NA**

**Repeat length (main genome) 1176**

**Blast2 Results -**

**OFC OLC RFC RLC**

**1 1163 1 1163**

**Ortholog Repeat Masker annotation**

**SW perc perc perc query position in query matching repeat position in repeat**

**score div. del. ins. sequence begin end (left) repeat class/family begin end (left) ID**

**8113 3.4 0.0 0.0 L1HS_3_57 1 1163 (0) + L1P1 LINE/L1 1909 3071 (3075) 1**

**_________________________________________________________________________**

**74. L1HS_3_7c**

**Ortholog annotation C_INTER_RMD_M_DISRUPTED Length 3821 nscore 0.00 NPOSITIONS NA**

**Repeat length (main genome) 3818**

**Blast2 Results -**

**OFC OLC RFC RLC**

**1 3821 1 3802**

**Ortholog Repeat Masker annotation**

**SW perc perc perc query position in query matching repeat position in repeat**

**score div. del. ins. sequence begin end (left) repeat class/family begin end (left) ID**

**15920 5.5 0.8 0.8 L1HS_3_7c 1 3255 (566) + L1HS LINE/L1 124 3376 (2770) 1**

**789 4.1 1.0 2.0 L1HS_3_7c 3247 3345 (476) + L1HS LINE/L1 574 671 (5361) 2 ***

**4246 2.5 0.0 0.0 L1HS_3_7c 3334 3821 (0) + L1HS LINE/L1 3379 3866 (2280) 1**

**_________________________________________________________________________**

**75. L1HS_3_83**

**Ortholog annotation M_INTRA_RMD Length 6614 nscore 0.15 NPOSITIONS 2834 2843 ;**

**Repeat length (main genome) 4844**

**Blast2 Results -**

**OFC OLC RFC RLC**

**1 2833 1 2834**

**2844 6614 1059 4830**

**Ortholog Repeat Masker annotation**

**SW perc perc perc query position in query matching repeat position in repeat**

**score div. del. ins. sequence begin end (left) repeat class/family begin end (left) ID**

**17438 3.5 0.2 0.0 L1HS_3_83 1 2833 (3781) + L1HS LINE/L1 165 3001 (3145) 1**

**25030 2.2 0.1 0.0 L1HS_3_83 2844 6614 (0) + L1HS LINE/L1 1226 5001 (1145) 2**

**_________________________________________________________________________**

**76. L1HS_3_88c**

**Ortholog annotation M_INTRA_RMD Length 6083 nscore 0.00 NPOSITIONS NA**

**Repeat length (main genome) 3729**

**Blast2 Results -**

**OFC OLC RFC RLC**

**1 3717 1 3729**

**Ortholog Repeat Masker annotation**

**SW perc perc perc query position in query matching repeat position in repeat**

**score div. del. ins. sequence begin end (left) repeat class/family begin end (left) ID**

**27892 3.1 0.1 0.0 L1HS_3_88c 1 6018 (65) + L1PA3 LINE/L1 133 6155 (0) 1**

**319 13.0 7.4 0.0 L1HS_3_88c 6028 6081 (2) C THE1D LTR/MaLR (213) 168 111 2**

**_________________________________________________________________________**

**77. L1HS_4_11**

**Ortholog annotation INDEL_PTS Length 9651 nscore 0.00 NPOSITIONS NA**

**Repeat length (main genome) 20**

**Blast2 Results -**

**OFC OLC RFC RLC**

**Ortholog Repeat Masker annotation**

**SW perc perc perc query position in query matching repeat position in repeat**

**score div. del. ins. sequence begin end (left) repeat class/family begin end (left) ID**

**1915 11.6 2.2 5.3 L1HS_4_11 29 53 (9598) C AluSx SINE/Alu (2) 310 286 1**

**216 0.0 0.0 0.0 L1HS_4_11 54 77 (9574) + (GTTTG)n Simple_repeat 2 25 (0) 2**

**1915 11.6 2.2 5.3 L1HS_4_11 78 371 (9280) C AluSx SINE/Alu (27) 285 1 1**

**8079 17.6 4.9 2.2 L1HS_4_11 446 2469 (7182) + L1MD LINE/L1 2162 4241 (1905) 3**

**855 15.7 8.0 4.0 L1HS_4_11 2492 2594 (7057) + L1MD LINE/L1 5170 5277 (869) 3**

**2093 13.3 0.0 0.6 L1HS_4_11 2595 2905 (6746) + AluSx SINE/Alu 1 309 (3) 4**

**855 14.4 3.3 4.4 L1HS_4_11 2906 3069 (6582) + L1MD LINE/L1 5278 5446 (796) 3**

**589 28.3 17.4 3.7 L1HS_4_11 3087 3413 (6238) + L2a LINE/L2 3055 3426 (0) 5**

**2369 5.6 4.2 0.0 L1HS_4_11 4324 4611 (5040) + AluY SINE/Alu 1 300 (11) 6**

**3854 8.9 4.5 0.0 L1HS_4_11 5022 5551 (4100) + MER41A LTR/ERV1 1 554 (0) 7**

**870 16.7 11.6 1.2 L1HS_4_11 5576 5739 (3912) + AluJb SINE/Alu 128 308 (4) 8**

**5567 18.2 3.9 4.8 L1HS_4_11 5740 6041 (3610) + L1PA15-16 LINE/L1 112 411 (6442) 9**

**1757 18.3 0.3 0.3 L1HS_4_11 6042 6332 (3319) C AluJb SINE/Alu (21) 291 1 10**

**12552 15.4 2.0 0.9 L1HS_4_11 6333 8864 (787) + L1PA15-16 LINE/L1 412 2853 (4000) 9**

**759 18.4 19.8 0.4 L1HS_4_11 8873 9119 (532) + AluJb SINE/Alu 10 304 (8) 11**

**3100 10.5 3.2 1.9 L1HS_4_11 9120 9651 (0) + L1PA15-16 LINE/L1 2843 3381 (3468) 9**

**_________________________________________________________________________**

**78. L1HS_4_114**

**Ortholog annotation C_INTER_RMD_M_DISRUPTED Length 311 nscore 0.00 NPOSITIONS NA**

**Repeat length (main genome) 327**

**Blast2 Results -**

**OFC OLC RFC RLC**

**1 311 1 311**

**Ortholog Repeat Masker annotation**

**SW perc perc perc query position in query matching repeat position in repeat**

**score div. del. ins. sequence begin end (left) repeat class/family begin end (left) ID**

**2507 4.5 0.3 0.3 L1HS_4_114 1 311 (0) + L1HS LINE/L1 1 311 (5721) 1**

**_________________________________________________________________________**

**79. L1HS_4_129**

**Ortholog annotation M_INTRA_RMD Length 5350 nscore 0.00 NPOSITIONS NA**

**Repeat length (main genome) 1179**

**Blast2 Results -**

**OFC OLC RFC RLC**

**1 1177 1 1179**

**Ortholog Repeat Masker annotation**

**SW perc perc perc query position in query matching repeat position in repeat**

**score div. del. ins. sequence begin end (left) repeat class/family begin end (left) ID**

**27781 3.5 0.2 0.0 L1HS_4_129 1 5349 (1) + L1HS LINE/L1 126 5480 (675) 1**

**_________________________________________________________________________**

**80. L1HS_4_130c**

**Ortholog annotation C_INTER_RMD_M_DISRUPTED Length 868 nscore 0.00 NPOSITIONS NA**

**Repeat length (main genome) 857**

**Blast2 Results -**

**OFC OLC RFC RLC**

**1 857 1 857**

**Ortholog Repeat Masker annotation**

**SW perc perc perc query position in query matching repeat position in repeat**

**score div. del. ins. sequence begin end (left) repeat class/family begin end (left) ID**

**6952 6.2 0.0 0.1 L1HS_4_130c 1 868 (0) + L1HS LINE/L1 2 868 (5164) 1**

**_________________________________________________________________________**

**81. L1HS_4_134**

**Ortholog annotation C_INTER_RMD_M_DISRUPTED Length 311 nscore 0.00 NPOSITIONS NA**

**Repeat length (main genome) 326**

**Blast2 Results -**

**OFC OLC RFC RLC**

**1 311 1 311**

**Ortholog Repeat Masker annotation**

**SW perc perc perc query position in query matching repeat position in repeat**

**score div. del. ins. sequence begin end (left) repeat class/family begin end (left) ID**

**2537 5.8 0.0 0.3 L1HS_4_134 1 311 (0) + L1HS LINE/L1 2 311 (5721) 1**

**_________________________________________________________________________**

**82. L1HS_4_48**

**Ortholog annotation INDEL_CAN Length 7784 nscore 0.13 NPOSITIONS 6278 6287 ;**

**Repeat length (main genome) 2510**

**Blast2 Results -**

**OFC OLC RFC RLC**

**Ortholog Repeat Masker annotation**

**SW perc perc perc query position in query matching repeat position in repeat**

**score div. del. ins. sequence begin end (left) repeat class/family begin end (left) ID**

**17901 3.1 0.3 0.0 L1HS_4_48 1 4047 (3737) C L1HS LINE/L1 (1961) 4185 126 1**

**2203 17.2 1.1 0.8 L1HS_4_48 4139 4502 (3282) C MLT1A0 LTR/MaLR (0) 365 1 2**

**2290 21.8 9.2 4.1 L1HS_4_48 4510 5150 (2634) C MLT1A0-int LTR/MaLR (8) 1727 880 2**

**3277 11.5 1.0 5.1 L1HS_4_48 5157 5669 (2115) + LTR3B_ LTR/ERVK 1 492 (7) 3**

**2500 21.8 5.2 0.0 L1HS_4_48 5803 6297 (1487) C MLT1A0-int LTR/MaLR (934) 801 281 2**

**1788 21.2 1.1 3.6 L1HS_4_48 6307 6668 (1116) C MLT1A0 LTR/MaLR (12) 353 1 4**

**367 35.1 1.5 1.5 L1HS_4_48 6791 6926 (858) C MIR SINE/MIR (117) 145 10 5**

**195 36.7 5.0 0.0 L1HS_4_48 7492 7611 (173) C L2c LINE/L2 (239) 3148 3023 6**

**_________________________________________________________________________**

**83. L1HS_4_4c**

**Ortholog annotation M_INTRA_RMD Length 2784 nscore 0.00 NPOSITIONS NA**

**Repeat length (main genome) 784**

**Blast2 Results -**

**OFC OLC RFC RLC**

**1 775 1 784**

**Ortholog Repeat Masker annotation**

**SW perc perc perc query position in query matching repeat position in repeat**

**score div. del. ins. sequence begin end (left) repeat class/family begin end (left) ID**

**17960 3.5 0.3 0.0 L1HS_4_4c 1 2784 (0) + L1HS LINE/L1 126 2918 (3228) 1**

**_________________________________________________________________________**

**84. L1HS_4_5**

**Ortholog annotation M_INTRA_RMD Length 1788 nscore 0.00 NPOSITIONS NA**

**Repeat length (main genome) 1343**

**Blast2 Results -**

**OFC OLC RFC RLC**

**1 1328 1 1335**

**Ortholog Repeat Masker annotation**

**SW perc perc perc query position in query matching repeat position in repeat**

**score div. del. ins. sequence begin end (left) repeat class/family begin end (left) ID**

**14674 5.3 0.1 0.1 L1HS_4_5 1 1788 (0) + L1HS LINE/L1 1 1788 (4244) 1**

**_________________________________________________________________________**

**85. L1HS_4_51**

**Ortholog annotation C_INTRA_RMD Length 3315 nscore 2.41 NPOSITIONS 808 887 ;**

**Repeat length (main genome) 3556**

**Blast2 Results -**

**OFC OLC RFC RLC**

**1 807 1 807**

**1012 3313 1252 3554**

**Ortholog Repeat Masker annotation**

**SW perc perc perc query position in query matching repeat position in repeat**

**score div. del. ins. sequence begin end (left) repeat class/family begin end (left) ID**

**6855 4.0 0.0 0.0 L1HS_4_51 1 807 (2508) + L1HS LINE/L1 125 931 (5224) 1**

**13118 3.0 0.7 0.0 L1HS_4_51 959 3315 (0) + L1HS LINE/L1 1310 3682 (2464) 1**

**_________________________________________________________________________**

**86. L1HS_4_52c**

**Ortholog annotation M_INTRA_RMD Length 7391 nscore 0.00 NPOSITIONS NA**

**Repeat length (main genome) 4280**

**Blast2 Results -**

**OFC OLC RFC RLC**

**1 4268 1 4280**

**Ortholog Repeat Masker annotation**

**SW perc perc perc query position in query matching repeat position in repeat**

**score div. del. ins. sequence begin end (left) repeat class/family begin end (left) ID**

**25884 3.7 0.2 0.0 L1HS_4_52c 1 6018 (1373) + L1PA3 LINE/L1 124 6155 (0) 1**

**184 32.8 0.0 0.0 L1HS_4_52c 6029 6086 (1305) C MIRc SINE/MIR (38) 230 173 2**

**210 17.6 31.1 0.0 L1HS_4_52c 6218 6291 (1100) + MIRc SINE/MIR 170 266 (2) 3**

**363 34.3 10.5 1.2 L1HS_4_52c 6302 6472 (919) C MIRb SINE/MIR (23) 245 59 4**

**315 4.9 0.0 0.0 L1HS_4_52c 6505 6545 (846) + (TG)n Simple_repeat 2 42 (0) 5**

**180 11.1 0.0 0.0 L1HS_4_52c 6814 6840 (551) + (TATG)n Simple_repeat 2 28 (0) 6**

**239 16.7 8.3 0.0 L1HS_4_52c 6921 6968 (423) C MLT1J1 LTR/MaLR (392) 52 1 7**

**658 21.9 9.5 0.6 L1HS_4_52c 7043 7222 (169) + L1PBa LINE/L1 3 163 (6700) 8 ***

**884 17.3 0.6 0.6 L1HS_4_52c 7221 7389 (2) + L1PBa LINE/L1 1393 1561 (5302) 8**

**_________________________________________________________________________**

**87. L1HS_4_59**

**Ortholog annotation C_INTER_RMD_M_DISRUPTED Length 1127 nscore 0.00 NPOSITIONS NA**

**Repeat length (main genome) 1137**

**Blast2 Results -**

**OFC OLC RFC RLC**

**5 1127 5 1124**

**Ortholog Repeat Masker annotation**

**SW perc perc perc query position in query matching repeat position in repeat**

**score div. del. ins. sequence begin end (left) repeat class/family begin end (left) ID**

**9173 4.6 0.1 0.5 L1HS_4_59 1 1127 (0) + L1HS LINE/L1 3 1124 (4908) 1**

**_________________________________________________________________________**

**88. L1HS_4_73**

**Ortholog annotation C_INTER_RMD_M_DISRUPTED Length 6931 nscore 0.00 NPOSITIONS NA**

**Repeat length (main genome) 2209**

**Blast2 Results -**

**OFC OLC RFC RLC**

**1 2198 1 2206**

**Ortholog Repeat Masker annotation**

**SW perc perc perc query position in query matching repeat position in repeat**

**score div. del. ins. sequence begin end (left) repeat class/family begin end (left) ID**

**25550 3.7 0.5 0.3 L1HS_4_73 1 6011 (920) + L1PA3 LINE/L1 124 6137 (18) 1**

**252 0.0 0.0 0.0 L1HS_4_73 6012 6039 (892) + (TAA)n Simple_repeat 3 30 (0) 2**

**194 0.0 3.9 0.0 L1HS_4_73 6104 6129 (802) + (CAAAAA)n Simple_repeat 2 28 (0) 3**

**360 22.9 4.9 2.1 L1HS_4_73 6130 6272 (659) + L1M5 LINE/L1 3252 3398 (2748) 4**

**1242 26.1 6.8 0.4 L1HS_4_73 6404 6918 (13) + L1M5 LINE/L1 3578 4125 (2021) 4**

**_________________________________________________________________________**

**89. L1HS_4_74**

**Ortholog annotation INDEL_PTS Length 6021 nscore 0.00 NPOSITIONS NA**

**Repeat length (main genome) 98**

**Blast2 Results -**

**OFC OLC RFC RLC**

**5122 5213 7 98**

**Ortholog Repeat Masker annotation**

**SW perc perc perc query position in query matching repeat position in repeat**

**score div. del. ins. sequence begin end (left) repeat class/family begin end (left) ID**

**274 35.6 3.5 0.0 L1HS_4_74 688 802 (5219) + L2a LINE/L2 2737 2855 (564) 1**

**2949 10.2 0.0 0.0 L1HS_4_74 1007 1370 (4651) C THE1B LTR/MaLR (0) 364 1 2**

**390 23.3 9.2 2.5 L1HS_4_74 1382 1549 (4472) C MLT1H LTR/MaLR (292) 197 53 3**

**871 32.9 5.5 3.0 L1HS_4_74 1609 2087 (3934) + L2a LINE/L2 2851 3335 (91) 1**

**862 19.1 1.8 2.4 L1HS_4_74 3376 3541 (2480) C FRAM SINE/Alu (0) 166 2 4**

**203 26.8 0.0 1.8 L1HS_4_74 4271 4327 (1694) + MIRb SINE/MIR 200 255 (13) 5**

**185 15.6 0.0 0.0 L1HS_4_74 4376 4407 (1614) C L2a LINE/L2 (1) 3425 3394 6**

**1167 19.6 1.0 0.0 L1HS_4_74 4693 4901 (1120) C MLT1F1 LTR/MaLR (0) 569 359 7**

**1171 19.1 1.4 0.0 L1HS_4_74 4906 5114 (907) C L1PB4 LINE/L1 (42) 6114 5903 8**

**7016 7.7 0.0 0.8 L1HS_4_74 5117 6021 (0) + L1P1 LINE/L1 2 899 (5256) 9**

**_________________________________________________________________________**

**90. L1HS_4_84c**

**Ortholog annotation C_DISRUPTED_M_INTER_RMD Length 1899 nscore 6.79 NPOSITIONS 1033 1160 ; 1232 1232 ;**

**Repeat length (main genome) 1752**

**Blast2 Results -**

**OFC OLC RFC RLC**

**1 1032 1 1033**

**1161 1899 1013 1752**

**Ortholog Repeat Masker annotation**

**SW perc perc perc query position in query matching repeat position in repeat**

**score div. del. ins. sequence begin end (left) repeat class/family begin end (left) ID**

**11250 14.2 2.8 4.0 L1HS_4_84c 1 1899 (0) + L1P1 LINE/L1 4 1879 (4276) 1**

**_________________________________________________________________________**

**91. L1HS_4_90**

**Ortholog annotation INDEL_PTS Length 5665 nscore 0.00 NPOSITIONS NA**

**Repeat length (main genome) 908**

**Blast2 Results -**

**OFC OLC RFC RLC**

**Ortholog Repeat Masker annotation**

**SW perc perc perc query position in query matching repeat position in repeat**

**score div. del. ins. sequence begin end (left) repeat class/family begin end (left) ID**

**21626 1.5 0.0 0.0 L1HS_4_90 1 4443 (1222) C L1HS LINE/L1 (1578) 4568 125 1**

**555 14.3 0.0 0.0 L1HS_4_90 4450 4533 (1132) + MER45A DNA/Tip100 95 178 (0) 2**

**555 28.6 13.2 0.0 L1HS_4_90 4545 4764 (901) C MIR SINE/MIR (1) 261 13 3**

**247 15.0 0.0 0.0 L1HS_4_90 5025 5064 (601) + Alu SINE/Alu 252 291 (11) 4**

**_________________________________________________________________________**

**92. L1HS_5_17**

**Ortholog annotation INDEL_PTS Length 429 nscore 0.00 NPOSITIONS NA**

**Repeat length (main genome) 416**

**Blast2 Results -**

**OFC OLC RFC RLC**

**Ortholog Repeat Masker annotation**

**SW perc perc perc query position in query matching repeat position in repeat**

**score div. del. ins. sequence begin end (left) repeat class/family begin end (left) ID**

**621 12.2 13.6 2.7 L1HS_5_17 34 143 (286) + FLAM_C SINE/Alu 6 127 (16) 1**

**551 17.2 0.0 0.0 L1HS_5_17 331 429 (0) C AluSg/x SINE/Alu (4) 308 210 2**

**_________________________________________________________________________**

**93. L1HS_5_22c**

**Ortholog annotation C_INTRA_RMD Length 2533 nscore 0.39 NPOSITIONS 2374 2383 ;**

**Repeat length (main genome) 2748**

**Blast2 Results -**

**OFC OLC RFC RLC**

**1 2372 1 2373**

**2389 2533 2598 2747**

**Ortholog Repeat Masker annotation**

**SW perc perc perc query position in query matching repeat position in repeat**

**score div. del. ins. sequence begin end (left) repeat class/family begin end (left) ID**

**15490 3.5 0.5 0.0 L1HS_5_22c 1 2384 (149) + L1HS LINE/L1 1649 4044 (2102) 1**

**1102 3.5 3.5 0.0 L1HS_5_22c 2389 2533 (0) + L1HS LINE/L1 4257 4406 (1740) 1**

**_________________________________________________________________________**

**94. L1HS_5_44c**

**Ortholog annotation INDEL_PTS Length 4163 nscore 0.00 NPOSITIONS NA**

**Repeat length (main genome) 1721**

**Blast2 Results -**

**OFC OLC RFC RLC**

**Ortholog Repeat Masker annotation**

**SW perc perc perc query position in query matching repeat position in repeat**

**score div. del. ins. sequence begin end (left) repeat class/family begin end (left) ID**

**1874 2.3 0.0 0.0 L1HS_5_44c 1 217 (3946) C L1P1 LINE/L1 (4317) 1838 1622 1 ***

**5711 7.6 0.3 0.6 L1HS_5_44c 215 945 (3218) C L1P1 LINE/L1 (5426) 729 1 1**

**1805 14.4 5.5 0.0 L1HS_5_44c 1000 1291 (2872) C AluSx SINE/Alu (4) 308 1 2**

**312 7.0 0.0 0.0 L1HS_5_44c 1773 1815 (2348) + (TG)n Simple_repeat 2 44 (0) 3**

**274 35.5 1.9 0.9 L1HS_5_44c 1822 1929 (2234) + L2b LINE/L2 3263 3371 (4) 4**

**2596 8.9 2.0 0.0 L1HS_5_44c 2284 2631 (1532) + THE1A LTR/MaLR 1 355 (0) 5**

**2654 30.0 16.7 3.2 L1HS_5_44c 2634 4161 (2) + MLT-int LTR/MaLR 2 1735 (0) 6**

**_________________________________________________________________________**

**95. L1HS_5_45c**

**Ortholog annotation C_INTER_RMD Length 429 nscore 0.00 NPOSITIONS NA**

**Repeat length (main genome) 891**

**Blast2 Results -**

**OFC OLC RFC RLC**

**1 36 1 36**

**Ortholog Repeat Masker annotation**

**There were no repetitive sequences detected in /home/vipin/L1HS_TR_CHR/Chimp/CONFIRMATION/L1HS_TR_INDEL_SEQUENCES/L1HS_5_45c**

**_________________________________________________________________________**

**96. L1HS_5_52**

**Ortholog annotation M_INTRA_RMD Length 6627 nscore 2.57 NPOSITIONS 3487 3646 ; 5035 5044 ;**

**Repeat length (main genome) 3936**

**Blast2 Results -**

**OFC OLC RFC RLC**

**1 3486 1 3497**

**3787 3825 3879 3918**

**Ortholog Repeat Masker annotation**

**SW perc perc perc query position in query matching repeat position in repeat**

**score div. del. ins. sequence begin end (left) repeat class/family begin end (left) ID**

**16968 3.2 0.2 0.0 L1HS_5_52 1 3486 (3141) + L1HS LINE/L1 124 3618 (2528) 1**

**9791 2.6 2.7 0.0 L1HS_5_52 3722 5030 (1597) + L1HS LINE/L1 3915 5258 (888) 1**

**7382 2.5 0.1 0.0 L1HS_5_52 5045 6567 (60) + L1PA2 LINE/L1 4630 6153 (2) 2**

**_________________________________________________________________________**

**97. L1HS_5_54c**

**Ortholog annotation C_INTER_RMD_M_DISRUPTED Length 312 nscore 0.00 NPOSITIONS NA**

**Repeat length (main genome) 328**

**Blast2 Results -**

**OFC OLC RFC RLC**

**1 302 1 302**

**Ortholog Repeat Masker annotation**

**SW perc perc perc query position in query matching repeat position in repeat**

**score div. del. ins. sequence begin end (left) repeat class/family begin end (left) ID**

**2546 5.1 0.3 0.0 L1HS_5_54c 1 312 (0) + L1P1 LINE/L1 1 313 (5842) 1**

**_________________________________________________________________________**

**98. L1HS_5_79c**

**Ortholog annotation INDEL_PTS Length 158 nscore 0.00 NPOSITIONS NA**

**Repeat length (main genome) 1160**

**Blast2 Results -**

**OFC OLC RFC RLC**

**no hits found**

**Ortholog Repeat Masker annotation**

**SW perc perc perc query position in query matching repeat position in repeat**

**score div. del. ins. sequence begin end (left) repeat class/family begin end (left) ID**

**426 25.9 2.7 0.0 L1HS_5_79c 2 113 (45) + MIR SINE/MIR 126 240 (22) 1**

**214 5.0 2.4 2.4 L1HS_5_79c 114 154 (4) + (TG)n Simple_repeat 2 42 (0) 2**

**_________________________________________________________________________**

**99. L1HS_5_93c**

**Ortholog annotation M_INTRA_RMD Length 5878 nscore 1.70 NPOSITIONS 4642 4741 ;**

**Repeat length (main genome) 935**

**Blast2 Results -**

**OFC OLC RFC RLC**

**1 936 1 935**

**Ortholog Repeat Masker annotation**

**SW perc perc perc query position in query matching repeat position in repeat**

**score div. del. ins. sequence begin end (left) repeat class/family begin end (left) ID**

**20405 3.7 0.3 0.3 L1HS_5_93c 1 4641 (1237) + L1PA2 LINE/L1 127 4769 (1377) 1**

**6222 3.3 0.1 0.0 L1HS_5_93c 4744 5491 (387) + L1PA2 LINE/L1 5407 6155 (0) 1**

**755 18.8 0.0 1.1 L1HS_5_93c 5493 5670 (208) C L1M4 LINE/L1 (3511) 3133 2958 2**

**244 18.6 5.1 0.0 L1HS_5_93c 5676 5734 (144) C L1M4 LINE/L1 (5108) 1434 1373 2**

**_________________________________________________________________________**

**100. L1HS_6_19c**

**Ortholog annotation C_INTER_RMD_M_DISRUPTED Length 4371 nscore 0.00 NPOSITIONS NA**

**Repeat length (main genome) 4766**

**Blast2 Results -**

**OFC OLC RFC RLC**

**1 921 1 918**

**907 4371 917 4380**

**Ortholog Repeat Masker annotation**

**SW perc perc perc query position in query matching repeat position in repeat**

**score div. del. ins. sequence begin end (left) repeat class/family begin end (left) ID**

**23913 7.5 0.1 0.3 L1HS_6_19c 1 4371 (0) + L1P2 LINE/L1 1427 5769 (385) 1**

**_________________________________________________________________________**

**101. L1HS_6_38**

**Ortholog annotation C_INTER_RMD_M_DISRUPTED Length 4242 nscore 0.00 NPOSITIONS NA**

**Repeat length (main genome) 4192**

**Blast2 Results -**

**OFC OLC RFC RLC**

**1 569 1 566**

**571 4241 515 4184**

**Ortholog Repeat Masker annotation**

**SW perc perc perc query position in query matching repeat position in repeat**

**score div. del. ins. sequence begin end (left) repeat class/family begin end (left) ID**

**256 3.2 0.0 0.0 L1HS_6_38 1 31 (4211) + L1P2 LINE/L1 191 221 (5934) 1 ***

**21797 4.9 0.8 0.9 L1HS_6_38 26 4242 (0) + L1P2 LINE/L1 738 4908 (1238) 1**

**_________________________________________________________________________**

**102. L1HS_6_41**

**Ortholog annotation C_INTER_RMD_M_DISRUPTED Length 779 nscore 0.00 NPOSITIONS NA**

**Repeat length (main genome) 3410**

**Blast2 Results -**

**OFC OLC RFC RLC**

**1 775 1 773**

**Ortholog Repeat Masker annotation**

**SW perc perc perc query position in query matching repeat position in repeat**

**score div. del. ins. sequence begin end (left) repeat class/family begin end (left) ID**

**6036 7.0 0.0 0.5 L1HS_6_41 1 774 (5) + L1HS LINE/L1 2 771 (5261) 1**

**_________________________________________________________________________**

**103. L1HS_6_59**

**Ortholog annotation M_INTRA_RMD Length 7493 nscore 10.57 NPOSITIONS 2220 2229 ; 3890 3938 ; 5624 6282 ; 7420 7493 ;**

**Repeat length (main genome) 3668**

**Blast2 Results -**

**OFC OLC RFC RLC**

**1 2219 1 2223**

**2380 3881 2073 3574**

**3941 5539 2070 3668**

**Ortholog Repeat Masker annotation**

**SW perc perc perc query position in query matching repeat position in repeat**

**score div. del. ins. sequence begin end (left) repeat class/family begin end (left) ID**

**17528 4.5 0.1 0.0 L1HS_6_59 1 2230 (5263) + L1HS LINE/L1 126 2358 (3788) 1**

**12637 2.4 1.4 0.2 L1HS_6_59 2256 3881 (3612) + L1P1 LINE/L1 2056 3698 (2448) 2**

**14002 2.1 0.1 0.1 L1HS_6_59 3939 5623 (1870) + L1P1 LINE/L1 2191 3876 (2270) 3**

**6716 7.2 1.6 0.0 L1HS_6_59 6283 7419 (74) C L1PA6 LINE/L1 (8) 6146 4995 4**

**_________________________________________________________________________**

**104. L1HS_6_71**

**Ortholog annotation C_INTER_RMD_M_DISRUPTED Length 1717 nscore 0.00 NPOSITIONS NA**

**Repeat length (main genome) 1729**

**Blast2 Results -**

**OFC OLC RFC RLC**

**1 1717 1 1717**

**Ortholog Repeat Masker annotation**

**SW perc perc perc query position in query matching repeat position in repeat**

**score div. del. ins. sequence begin end (left) repeat class/family begin end (left) ID**

**14872 2.9 0.3 0.0 L1HS_6_71 1 1717 (0) + L1HS LINE/L1 1 1723 (4309) 1**

**_________________________________________________________________________**

**105. L1HS_6_93**

**Ortholog annotation INDEL_PTS Length 2827 nscore 0.00 NPOSITIONS NA**

**Repeat length (main genome) 1164**

**Blast2 Results -**

**OFC OLC RFC RLC**

**Ortholog Repeat Masker annotation**

**SW perc perc perc query position in query matching repeat position in repeat**

**score div. del. ins. sequence begin end (left) repeat class/family begin end (left) ID**

**3944 7.1 1.5 1.5 L1HS_6_93 1 584 (2243) C L1P1 LINE/L1 (2227) 3919 3336 1 ***

**5056 5.1 0.0 0.0 L1HS_6_93 581 1261 (1566) + L1P1 LINE/L1 2599 3279 (2867) 1**

**226 32.4 1.8 16.3 L1HS_6_93 1271 1436 (1391) C MIRb SINE/MIR (121) 147 6 2**

**300 30.6 19.7 1.6 L1HS_6_93 1485 1675 (1152) C MIRb SINE/MIR (6) 262 38 3**

**_________________________________________________________________________**

**106. L1HS_7_11**

**Ortholog annotation INDEL_PTS Length 4449 nscore 0.22 NPOSITIONS 405 414 ;**

**Repeat length (main genome) 98**

**Blast2 Results -**

**OFC OLC RFC RLC**

**no hits found**

**Ortholog Repeat Masker annotation**

**SW perc perc perc query position in query matching repeat position in repeat**

**score div. del. ins. sequence begin end (left) repeat class/family begin end (left) ID**

**272 27.6 1.5 5.4 L1HS_7_11 145 274 (4175) + ERVL-E-int LTR/ERVL 3809 3933 (1734) 1**

**2531 4.8 0.0 1.4 L1HS_7_11 747 1040 (3409) + AluY SINE/Alu 13 302 (9) 2**

**522 27.1 12.0 6.9 L1HS_7_11 1185 1501 (2948) + ERVL-E-int LTR/ERVL 3809 4141 (1526) 3**

**2476 19.4 1.5 2.3 L1HS_7_11 1896 2369 (2080) + MER70A LTR/ERVL 1 470 (2) 4**

**2057 18.4 5.5 4.3 L1HS_7_11 2382 2598 (1851) C MSTB LTR/MaLR (0) 426 206 5**

**1945 14.0 0.7 0.7 L1HS_7_11 2599 2886 (1563) C AluJb SINE/Alu (18) 294 7 6**

**2057 18.4 5.5 4.3 L1HS_7_11 2887 3089 (1360) C MSTB LTR/MaLR (221) 205 1 5**

**828 15.2 0.0 1.5 L1HS_7_11 3646 3779 (670) + LTR40b LTR/ERVL 331 462 (0) 7**

**460 28.6 5.3 1.8 L1HS_7_11 4059 4343 (106) + L1M5 LINE/L1 3648 3942 (2204) 8**

**370 23.6 0.0 0.0 L1HS_7_11 4361 4449 (0) + L1P2 LINE/L1 1369 1457 (818) 9**

**_________________________________________________________________________**

**107. L1HS_7_19c**

**Ortholog annotation M_INTRA_RMD Length 3504 nscore 0.00 NPOSITIONS NA**

**Repeat length (main genome) 2073**

**Blast2 Results -**

**OFC OLC RFC RLC**

**3 2074 3 2073**

**Ortholog Repeat Masker annotation**

**SW perc perc perc query position in query matching repeat position in repeat**

**score div. del. ins. sequence begin end (left) repeat class/family begin end (left) ID**

**18765 2.1 0.0 0.0 L1HS_7_19c 1 3504 (0) + L1HS LINE/L1 126 3629 (2517) 1**

**_________________________________________________________________________**

**108. L1HS_7_21**

**Ortholog annotation C_INTER_RMD_M_DISRUPTED Length 696 nscore 0.00 NPOSITIONS NA**

**Repeat length (main genome) 4901**

**Blast2 Results -**

**OFC OLC RFC RLC**

**1 423 1 424**

**Ortholog Repeat Masker annotation**

**SW perc perc perc query position in query matching repeat position in repeat**

**score div. del. ins. sequence begin end (left) repeat class/family begin end (left) ID**

**2279 21.9 6.6 1.4 L1HS_7_21 1 696 (0) + L1MDa LINE/L1 1213 1944 (4689) 1**

**_________________________________________________________________________**

**109. L1HS_7_47**

**Ortholog annotation INDEL_PAC Length 639 nscore 0.00 NPOSITIONS NA**

**Repeat length (main genome) 1693**

**Blast2 Results -**

**OFC OLC RFC RLC**

**334 639 5 310**

**Ortholog Repeat Masker annotation**

**SW perc perc perc query position in query matching repeat position in repeat**

**score div. del. ins. sequence begin end (left) repeat class/family begin end (left) ID**

**2168 0.4 0.0 0.0 L1HS_7_47 1 253 (386) C L1P1 LINE/L1 (2702) 3444 3192 1 ***

**3475 1.0 0.0 0.0 L1HS_7_47 250 639 (0) + L1P1 LINE/L1 4434 4823 (1323) 2**

**_________________________________________________________________________**

**110. L1HS_7_5**

**Ortholog annotation INDEL_PTS Length 9604 nscore 0.10 NPOSITIONS 2720 2729 ;**

**Repeat length (main genome) 2995**

**Blast2 Results -**

**OFC OLC RFC RLC**

**Ortholog Repeat Masker annotation**

**SW perc perc perc query position in query matching repeat position in repeat**

**score div. del. ins. sequence begin end (left) repeat class/family begin end (left) ID**

**15938 2.4 0.2 0.0 L1HS_7_5 1 2647 (6957) C L1HS LINE/L1 (2167) 3979 1326 1**

**13605 2.3 0.5 0.1 L1HS_7_5 2730 5069 (4535) C L1HS LINE/L1 (3115) 3031 682 2 ***

**14541 11.7 4.3 2.1 L1HS_7_5 5069 9349 (255) C L1P2 LINE/L1 (1898) 4433 14 3**

**_________________________________________________________________________**

**111. L1HS_7_65c**

**Ortholog annotation C_INTER_RMD_M_DISRUPTED Length 1834 nscore 0.00 NPOSITIONS NA**

**Repeat length (main genome) 1304**

**Blast2 Results -**

**OFC OLC RFC RLC**

**1 1074 1 1074**

**1088 1320 1072 1304**

**Ortholog Repeat Masker annotation**

**SW perc perc perc query position in query matching repeat position in repeat**

**score div. del. ins. sequence begin end (left) repeat class/family begin end (left) ID**

**15294 4.6 0.2 0.1 L1HS_7_65c 1 1834 (0) + L1HS LINE/L1 4 1839 (4193) 1**

**_________________________________________________________________________**

**112. L1HS_7_67**

**Ortholog annotation C_INTER_RMD_M_DISRUPTED Length 1955 nscore 0.00 NPOSITIONS NA**

**Repeat length (main genome) 1966**

**Blast2 Results -**

**OFC OLC RFC RLC**

**1 1955 1 1953**

**Ortholog Repeat Masker annotation**

**SW perc perc perc query position in query matching repeat position in repeat**

**score div. del. ins. sequence begin end (left) repeat class/family begin end (left) ID**

**8717 4.4 0.0 0.0 L1HS_7_67 1 1955 (0) + L1HS LINE/L1 1239 3193 (2953) 1**

**_________________________________________________________________________**

**113. L1HS_7_73**

**Ortholog annotation C_DISRUPTED_M_INTER_RMD Length 3334 nscore 0.33 NPOSITIONS 2153 2153 ; 2233 2242 ;**

**Repeat length (main genome) 3099**

**Blast2 Results -**

**OFC OLC RFC RLC**

**1 2201 1 2198**

**2253 3334 2018 3098**

**Ortholog Repeat Masker annotation**

**SW perc perc perc query position in query matching repeat position in repeat**

**score div. del. ins. sequence begin end (left) repeat class/family begin end (left) ID**

**11963 4.6 0.2 0.0 L1HS_7_73 1 2253 (1081) + L1HS LINE/L1 717 2975 (3171) 1**

**8658 2.4 0.1 0.0 L1HS_7_73 2249 3333 (1) + L1P1 LINE/L1 2733 3818 (2328) 2 ***

**_________________________________________________________________________**

**114. L1HS_8_19**

**Ortholog annotation C_INTER_RMD_M_DISRUPTED Length 956 nscore 0.00 NPOSITIONS NA**

**Repeat length (main genome) 1341**

**Blast2 Results -**

**OFC OLC RFC RLC**

**1 956 1 956**

**Ortholog Repeat Masker annotation**

**SW perc perc perc query position in query matching repeat position in repeat**

**score div. del. ins. sequence begin end (left) repeat class/family begin end (left) ID**

**4377 4.9 0.5 0.0 L1HS_8_19 29 956 (0) + L1P1 LINE/L1 4836 5769 (385) 1**

**_________________________________________________________________________**

**115. L1HS_8_21c**

**Ortholog annotation INDEL_PAC Length 245 nscore 0.00 NPOSITIONS NA**

**Repeat length (main genome) 310**

**Blast2 Results -**

**OFC OLC RFC RLC**

**no hits found**

**Ortholog Repeat Masker annotation**

**SW perc perc perc query position in query matching repeat position in repeat**

**score div. del. ins. sequence begin end (left) repeat class/family begin end (left) ID**

**614 29.7 0.9 4.1 L1HS_8_21c 19 239 (6) + L1MEc LINE/L1 267 480 (5920) 1**

**_________________________________________________________________________**

**116. L1HS_8_35**

**Ortholog annotation C_DISRUPTED_M_INTER_RMD Length 2817 nscore 0.39 NPOSITIONS 2110 2119 ; 2142 2142 ;**

**Repeat length (main genome) 2115**

**Blast2 Results -**

**OFC OLC RFC RLC**

**1 2033 1 2040**

**2120 2817 1418 2114**

**Ortholog Repeat Masker annotation**

**SW perc perc perc query position in query matching repeat position in repeat**

**score div. del. ins. sequence begin end (left) repeat class/family begin end (left) ID**

**9714 3.7 0.5 0.0 L1HS_8_35 1 2052 (765) + L1HS LINE/L1 1294 3356 (2790) 1**

**5364 4.5 0.0 0.1 L1HS_8_35 2120 2817 (0) + L1P1 LINE/L1 2711 3407 (2739) 2**

**_________________________________________________________________________**

**117. L1HS_8_5**

**Ortholog annotation M_INTRA_RMD Length 4792 nscore 0.00 NPOSITIONS NA**

**Repeat length (main genome) 3947**

**Blast2 Results -**

**OFC OLC RFC RLC**

**1 441 1 441**

**472 3937 478 3943**

**Ortholog Repeat Masker annotation**

**SW perc perc perc query position in query matching repeat position in repeat**

**score div. del. ins. sequence begin end (left) repeat class/family begin end (left) ID**

**24053 2.9 0.4 0.1 L1HS_8_5 1 4792 (0) + L1HS LINE/L1 125 4929 (1217) 1**

**_________________________________________________________________________**

**118. L1HS_8_50c**

**Ortholog annotation M_INTRA_RMD Length 7421 nscore 0.00 NPOSITIONS NA**

**Repeat length (main genome) 4929**

**Blast2 Results -**

**OFC OLC RFC RLC**

**1 4902 1 4908**

**Ortholog Repeat Masker annotation**

**SW perc perc perc query position in query matching repeat position in repeat**

**score div. del. ins. sequence begin end (left) repeat class/family begin end (left) ID**

**25760 3.3 0.2 0.1 L1HS_8_50c 1 6014 (1407) + L1PA3 LINE/L1 136 6152 (3) 1**

**23 3.3 0.0 0.0 L1HS_8_50c 6015 6044 (1377) + AT_rich Low_complexity 1 30 (0) 2**

**270 16.0 0.0 0.0 L1HS_8_50c 6057 6106 (1315) + (TG)n Simple_repeat 1 50 (0) 3**

**408 35.9 1.5 0.0 L1HS_8_50c 6142 6272 (1149) C MIRc SINE/MIR (118) 150 18 4**

**189 10.7 0.0 0.0 L1HS_8_50c 6749 6776 (645) + (TTTTG)n Simple_repeat 3 30 (0) 5**

**_________________________________________________________________________**

**119. L1HS_9_12**

**Ortholog annotation M_INTRA_RMD Length 4971 nscore 16.92 NPOSITIONS 4125 4965 ;**

**Repeat length (main genome) 2239**

**Blast2 Results -**

**OFC OLC RFC RLC**

**5 2239 5 2239**

**Ortholog Repeat Masker annotation**

**SW perc perc perc query position in query matching repeat position in repeat**

**score div. del. ins. sequence begin end (left) repeat class/family begin end (left) ID**

**17305 3.9 0.9 0.0 L1HS_9_12 1 4078 (893) + L1HS LINE/L1 124 4237 (1909) 1**

**_________________________________________________________________________**

**120. L1HS_9_22**

**Ortholog annotation INDEL_PTS Length 8487 nscore 0.00 NPOSITIONS NA**

**Repeat length (main genome) 2054**

**Blast2 Results -**

**OFC OLC RFC RLC**

**Ortholog Repeat Masker annotation**

**SW perc perc perc query position in query matching repeat position in repeat**

**score div. del. ins. sequence begin end (left) repeat class/family begin end (left) ID**

**1327 13.4 0.3 0.2 L1HS_9_22 1 8487 (0) + ALR/Alpha Satellite/centr 1 8491 (0) 1**

**_________________________________________________________________________**

**121. L1HS_9_25**

**Ortholog annotation C_DISRUPTED_M_INTER_RMD Length 13633 nscore 1.29 NPOSITIONS 8401 8576 ;**

**Repeat length (main genome) 3044**

**Blast2 Results -**

**OFC OLC RFC RLC**

**3 3044 3 3044**

**12729 13633 2140 3044**

**Ortholog Repeat Masker annotation**

**SW perc perc perc query position in query matching repeat position in repeat**

**score div. del. ins. sequence begin end (left) repeat class/family begin end (left) ID**

**25531 3.8 0.1 0.0 L1HS_9_25 3 6015 (7618) + L1PA3 LINE/L1 129 6147 (8) 1**

**26 3.0 0.0 0.0 L1HS_9_25 6016 6048 (7585) + AT_rich Low_complexity 1 33 (0) 2**

**21 0.0 0.0 0.0 L1HS_9_25 7157 7177 (6456) + AT_rich Low_complexity 1 21 (0) 3**

**7435 2.3 0.4 0.0 L1HS_9_25 7191 8386 (5247) C L1PA3 LINE/L1 (2) 6153 4951 4**

**2830 9.5 0.0 0.4 L1HS_9_25 8594 9048 (4585) C L1P1 LINE/L1 (743) 5403 4951 5**

**230 29.4 6.8 6.8 L1HS_9_25 9673 9818 (3815) + L2 LINE/L2 2799 2944 (475) 6**

**1711 28.7 15.1 3.2 L1HS_9_25 10546 11099 (2534) + L2a LINE/L2 2213 2833 (586) 7**

**1776 12.4 0.4 0.0 L1HS_9_25 11100 11341 (2292) C AluSx SINE/Alu (12) 300 58 8**

**1711 28.8 15.3 3.0 L1HS_9_25 11342 11606 (2027) + L2a LINE/L2 2834 3127 (299) 7**

**7139 3.7 0.1 0.0 L1HS_9_25 11615 12727 (906) C L1PA4 LINE/L1 (8) 6147 5034 9**

**6760 4.9 0.0 0.0 L1HS_9_25 12729 13633 (0) + L1P1 LINE/L1 2266 3170 (2976) 10**

**_________________________________________________________________________**

**122. L1HS_9_31**

**Ortholog annotation INDEL_CAN Length 1469 nscore 0.00 NPOSITIONS NA**

**Repeat length (main genome) 1480**

**Blast2 Results -**

**OFC OLC RFC RLC**

**1 1469 2 1468**

**1442 1469 1453 1480**

**Ortholog Repeat Masker annotation**

**SW perc perc perc query position in query matching repeat position in repeat**

**score div. del. ins. sequence begin end (left) repeat class/family begin end (left) ID**

**6798 7.7 0.8 2.7 L1HS_9_31 1 1459 (10) + L1PA8 LINE/L1 4739 6172 (0) 1**

**_________________________________________________________________________**

**123. L1HS_9_44**

**Ortholog annotation M_INTRA_RMD Length 6542 nscore 0.17 NPOSITIONS 2922 2922 ; 2962 2971 ;**

**Repeat length (main genome) 3986**

**Blast2 Results -**

**OFC OLC RFC RLC**

**1 1002 1 1001**

**1001 2948 969 2917**

**3043 4603 2425 3986**

**Ortholog Repeat Masker annotation**

**SW perc perc perc query position in query matching repeat position in repeat**

**score div. del. ins. sequence begin end (left) repeat class/family begin end (left) ID**

**18101 3.2 0.1 1.0 L1HS_9_44 1 2955 (3587) + L1HS LINE/L1 125 3049 (3097) 1**

**24164 2.7 0.7 0.1 L1HS_9_44 2977 6542 (0) + L1PA3 LINE/L1 2464 6051 (104) 2**

**_________________________________________________________________________**

**124. L1HS_9_5c**

**Ortholog annotation M_INTRA_RMD Length 4864 nscore 4.26 NPOSITIONS 847 1053 ;**

**Repeat length (main genome) 4316**

**Blast2 Results -**

**OFC OLC RFC RLC**

**1 701 1 707**

**1054 4832 535 4315**

**Ortholog Repeat Masker annotation**

**SW perc perc perc query position in query matching repeat position in repeat**

**score div. del. ins. sequence begin end (left) repeat class/family begin end (left) ID**

**5668 5.6 3.7 0.1 L1HS_9_5c 1 803 (4061) + L1HS LINE/L1 612 1443 (4589) 1**

**15605 10.8 0.7 0.1 L1HS_9_5c 1056 4832 (32) + L1HS LINE/L1 1273 5093 (1053) 2**

**_________________________________________________________________________**

**125. L1HS_9_9**

**Ortholog annotation INDEL_PTS Length 9396 nscore 0.11 NPOSITIONS 1426 1435 ;**

**Repeat length (main genome) 1359**

**Blast2 Results -**

**OFC OLC RFC RLC**

**Ortholog Repeat Masker annotation**

**SW perc perc perc query position in query matching repeat position in repeat**

**score div. del. ins. sequence begin end (left) repeat class/family begin end (left) ID**

**7234 7.9 1.4 0.0 L1HS_9_9 1 1016 (8380) C L1P1 LINE/L1 (2201) 3945 2916 1**

**3020 8.2 0.0 0.2 L1HS_9_9 1021 1425 (7971) C L1PA5 LINE/L1 (8) 6146 5743 2**

**6514 8.0 0.8 0.1 L1HS_9_9 1446 2436 (6960) C L1PA5 LINE/L1 (650) 5504 4509 2**

**2197 9.2 0.0 0.0 L1HS_9_9 2434 2737 (6659) C L1P1 LINE/L1 (3212) 2934 2631 1 ***

**242 3.3 0.0 0.0 L1HS_9_9 2747 2776 (6620) + MSTB1 LTR/MaLR 390 419 (13) 3**

**252 14.3 2.0 0.0 L1HS_9_9 2781 2829 (6567) C L1M2 LINE/L1 (539) 5761 5712 4 ***

**5705 19.2 3.0 2.1 L1HS_9_9 2824 4180 (5216) C L1M2a LINE/L1 (2381) 5181 3812 5**

**1359 22.2 0.9 0.9 L1HS_9_9 4190 4535 (4861) + L1M3 LINE/L1 3286 3631 (2509) 6**

**2369 23.7 6.8 3.0 L1HS_9_9 4541 5718 (3678) C L1M2a LINE/L1 (3758) 3807 2550 5**

**1256 19.6 1.1 5.6 L1HS_9_9 5723 6008 (3388) C AluJo SINE/Alu (22) 290 18 7**

**5680 25.1 3.7 2.9 L1HS_9_9 6010 8398 (998) C L1M2a LINE/L1 (4658) 2908 400 5**

**2013 19.6 3.7 11.3 L1HS_9_9 8396 8540 (856) C L1M4 LINE/L1 (1011) 5135 5001 8 ***

**1898 17.7 0.3 1.6 L1HS_9_9 8541 8856 (540) C AluJb SINE/Alu (0) 312 1 9**

**2013 19.6 3.7 11.3 L1HS_9_9 8857 9365 (31) C L1M4 LINE/L1 (1146) 5000 4531 8**

**_________________________________________________________________________**

**126. L1HS_X_19c**

**Ortholog annotation INDEL_PTS Length 573 nscore 0.00 NPOSITIONS NA**

**Repeat length (main genome) 918**

**Blast2 Results -**

**OFC OLC RFC RLC**

**Ortholog Repeat Masker annotation**

**SW perc perc perc query position in query matching repeat position in repeat**

**score div. del. ins. sequence begin end (left) repeat class/family begin end (left) ID**

**2588 5.5 3.4 0.0 L1HS_X_19c 1 326 (247) C MER11B LTR/ERVK (899) 337 1 1**

**1009 14.8 5.3 4.1 L1HS_X_19c 328 573 (0) + LTR49-int LTR/ERV1 5399 5647 (684) 2**

**_________________________________________________________________________**

**127. L1HS_X_33**

**Ortholog annotation C_DISRUPTED_M_INTER_RMD Length 1746 nscore 18.27 NPOSITIONS 219 537 ;**

**Repeat length (main genome) 1394**

**Blast2 Results -**

**OFC OLC RFC RLC**

**1 218 1 218**

**553 1746 200 1393**

**Ortholog Repeat Masker annotation**

**SW perc perc perc query position in query matching repeat position in repeat**

**score div. del. ins. sequence begin end (left) repeat class/family begin end (left) ID**

**1510 14.3 0.0 0.0 L1HS_X_33 3 218 (1528) + L1P2 LINE/L1 6 221 (2054) 1**

**7278 10.7 1.2 1.7 L1HS_X_33 552 1746 (0) + L1P2 LINE/L1 341 1551 (724) 1**

**_________________________________________________________________________**

**128. L1HS_X_38**

**Ortholog annotation INDEL_PTS Length 7191 nscore 6.56 NPOSITIONS 1527 1986 ; 2022 2022 ; 2030 2030 ; 3666 3675 ;**

**Repeat length (main genome) 429**

**Blast2 Results -**

**OFC OLC RFC RLC**

**Ortholog Repeat Masker annotation**

**SW perc perc perc query position in query matching repeat position in repeat**

**score div. del. ins. sequence begin end (left) repeat class/family begin end (left) ID**

**286 24.8 22.4 0.0 L1HS_X_38 59 167 (7024) C MIR SINE/MIR (122) 146 17 1**

**470 38.4 4.0 2.0 L1HS_X_38 448 795 (6396) + L2 LINE/L2 1908 2262 (1157) 2**

**484 22.9 1.9 0.9 L1HS_X_38 805 910 (6281) C L2a LINE/L2 (15) 3411 3305 3**

**228 32.7 1.9 0.0 L1HS_X_38 916 1022 (6169) + L2 LINE/L2 2260 2368 (1051) 2**

**278 34.2 24.3 1.4 L1HS_X_38 1028 1250 (5941) C L2c LINE/L2 (246) 3141 2866 4**

**531 6.1 3.7 0.0 L1HS_X_38 1444 1525 (5666) + AluY SINE/Alu 1 85 (233) 5**

**1503 15.3 0.4 0.0 L1HS_X_38 1987 2221 (4970) + AluSg SINE/Alu 59 294 (16) 6**

**368 33.6 7.8 4.3 L1HS_X_38 2223 2452 (4739) C L1M6 LINE/L1 (1535) 4865 4628 7**

**776 29.3 8.5 3.0 L1HS_X_38 2478 3086 (4105) C L1MC LINE/L1 (1809) 4337 3695 8**

**1174 9.9 14.5 0.5 L1HS_X_38 3233 3425 (3766) + MER4A1 LTR/ERV1 1 220 (252) 9**

**194 0.0 3.9 0.0 L1HS_X_38 3497 3522 (3669) + (TTTTC)n Simple_repeat 2 28 (0) 10**

**2227 8.6 0.6 4.7 L1HS_X_38 3676 3993 (3198) C AluSq SINE/Alu (8) 305 1 11**

**1435 7.3 2.1 0.0 L1HS_X_38 3995 4186 (3005) + MER4A1 LTR/ERV1 277 472 (0) 9**

**585 33.0 5.7 1.6 L1HS_X_38 4236 4672 (2519) C L1M6 LINE/L1 (4943) 1553 1099 7**

**354 4.5 0.0 0.0 L1HS_X_38 4861 4904 (2287) + (TGG)n Simple_repeat 3 46 (0) 12**

**402 32.3 5.7 0.0 L1HS_X_38 4982 5139 (2052) C L1M6 LINE/L1 (5708) 788 622 7**

**635 28.7 4.4 1.0 L1HS_X_38 5384 5587 (1604) C L1M6 LINE/L1 (6285) 211 1 7**

**188 23.6 3.8 10.0 L1HS_X_38 5855 5934 (1257) C L4 LINE/RTE (295) 1734 1660 13**

**21 7.1 0.0 0.0 L1HS_X_38 6314 6355 (836) + AT_rich Low_complexity 1 42 (0) 14**

**2001 12.2 1.4 0.3 L1HS_X_38 6476 6771 (420) C AluSg SINE/Alu (11) 299 1 15**

**2179 9.9 0.3 0.0 L1HS_X_38 6823 7145 (46) C AluSp SINE/Alu (0) 313 1 16**

**_________________________________________________________________________**

**129. L1HS_X_45c**

**Ortholog annotation C_INTRA_RMD Length 2729 nscore 1.10 NPOSITIONS 699 728 ;**

**Repeat length (main genome) 3375**

**Blast2 Results -**

**OFC OLC RFC RLC**

**1 698 1 698**

**729 2729 1374 3375**

**Ortholog Repeat Masker annotation**

**SW perc perc perc query position in query matching repeat position in repeat**

**score div. del. ins. sequence begin end (left) repeat class/family begin end (left) ID**

**5528 5.0 0.0 0.0 L1HS_X_45c 1 698 (2031) + L1HS LINE/L1 1018 1715 (4440) 1**

**16120 3.7 0.3 0.1 L1HS_X_45c 729 2729 (0) + L1HS LINE/L1 2393 4397 (1749) 1**

**_________________________________________________________________________**

**130. L1HS_X_63c**

**Ortholog annotation C_DISRUPTED_M_INTER_RMD Length 2105 nscore 12.21 NPOSITIONS 1607 1863 ;**

**Repeat length (main genome) 1809**

**Blast2 Results -**

**OFC OLC RFC RLC**

**1 1606 1 1611**

**1867 2101 1569 1805**

**Ortholog Repeat Masker annotation**

**SW perc perc perc query position in query matching repeat position in repeat**

**score div. del. ins. sequence begin end (left) repeat class/family begin end (left) ID**

**13485 3.8 0.3 0.1 L1HS_X_63c 1 1606 (499) + L1HS LINE/L1 3 1612 (4420) 1**

**2027 2.5 0.8 0.0 L1HS_X_63c 1865 2105 (0) + L1HS LINE/L1 1568 1810 (4222) 1**

**_________________________________________________________________________**

**131. L1HS_X_69c**

**Ortholog annotation C_INTER_RMD_M_DISRUPTED Length 80452 nscore 8.03 NPOSITIONS 257 266 ; 3166 4527 ; 5824 5833 ; 7586 8408 ; 11226 11393 ; 16400 16494 ; 21750 21759 ; 24105 24429 ; 37411 37420 ; 48724 48724 ; 48742 48742 ; 48770 48770 ; 48800 49968 ; 52756 52935 ; 57522 58753 ; 60760 60769 ; 62547 63294 ; 70834 71066 ; 71125 71125 ; 71134 71134 ; 71136 71136 ; 71138 71138 ; 71141 71143 ; 71146 71147 ; 77538 77601 ;**

**Repeat length (main genome) 2396**

**Blast2 Results -**

**OFC OLC RFC RLC**

**1 256 1 256**

**295 1300 1379 2383**

**1548 1576 1936 1964**

**1642 1673 2029 2060**

**5571 5821 721 971**

**8422 9766 1027 2383**

**10674 11031 1859 2217**

**11139 11177 2309 2347**

**13637 13758 1483 1604**

**13806 14245 1654 2096**

**19728 19754 1576 1602**

**19802 19861 1650 1709**

**26955 27018 1613 1676**

**27767 27795 428 456**

**28060 28106 722 768**

**28231 28355 895 1020**

**28747 28820 1413 1486**

**28917 28944 1577 1604**

**29035 29061 1656 1682**

**29319 29378 1937 1996**

**45804 48186 9 2393**

**Ortholog Repeat Masker annotation**

**SW perc perc perc query position in query matching repeat position in repeat**

**score div. del. ins. sequence begin end (left) repeat class/family begin end (left) ID**

**2259 0.4 0.0 0.0 L1HS_X_69c 1 256 (80196) + L1PA4 LINE/L1 3760 4015 (2131) 1**

**7444 3.8 0.1 0.1 L1HS_X_69c 273 1313 (79139) + L1PA4 LINE/L1 5113 6155 (0) 1**

**4279 15.2 2.2 0.3 L1HS_X_69c 1372 2128 (78324) + L1MA4 LINE/L1 5516 6287 (13) 2**

**827 24.6 6.7 1.3 L1HS_X_69c 2447 2685 (77767) + MLT1L LTR/MaLR 336 587 (23) 3**

**606 31.3 9.0 2.6 L1HS_X_69c 2686 2918 (77534) C MIRb SINE/MIR (1) 267 20 4**

**665 14.6 1.8 0.0 L1HS_X_69c 3056 3165 (77287) C Tigger1 DNA/MER2_type (730) 1688 1577 5**

**25 8.3 0.0 0.0 L1HS_X_69c 4734 4793 (75659) + AT_rich Low_complexity 1 60 (0) 6**

**845 27.8 9.2 5.0 L1HS_X_69c 5029 5410 (75042) + L1MB4 LINE/L1 8 405 (6202) 7**

**1690 13.1 0.0 0.7 L1HS_X_69c 5546 5821 (74631) + L1P1 LINE/L1 4457 4730 (1416) 8**

**5812 15.6 5.3 0.0 L1HS_X_69c 5823 7265 (73187) C Tigger1 DNA/MER2_type (870) 1548 30 5**

**6614 8.1 1.0 0.3 L1HS_X_69c 8422 9781 (70671) + L1PA6 LINE/L1 4786 6154 (0) 9**

**497 21.4 2.9 0.0 L1HS_X_69c 9784 9886 (70566) C MER45R DNA/Tip100 (1474) 107 2 10**

**4287 6.2 0.3 1.2 L1HS_X_69c 10653 11219 (69233) + L1PA8 LINE/L1 5604 6165 (7) 11**

**2153 19.9 2.2 0.1 L1HS_X_69c 11396 11881 (68571) + L1PA10 LINE/L1 2035 2557 (4311) 12**

**642 11.3 0.0 0.0 L1HS_X_69c 11885 11981 (68471) + (GAAA)n Simple_repeat 2 98 (0) 13**

**8005 13.4 1.1 1.1 L1HS_X_69c 11983 13630 (66822) + L1PA10 LINE/L1 2550 4191 (2658) 12**

**5736 12.2 1.5 0.3 L1HS_X_69c 13630 14549 (65903) + L1PA10 LINE/L1 5938 6868 (3) 12 ***

**2861 23.5 7.4 3.6 L1HS_X_69c 14604 15870 (64582) + L1M4 LINE/L1 624 1926 (4720) 14 ***

**15560 18.3 2.9 2.5 L1HS_X_69c 15868 19237 (61215) + L1PA13 LINE/L1 4 3387 (3379) 15**

**6117 13.2 2.4 0.4 L1HS_X_69c 19233 20681 (59771) + L1MA2 LINE/L1 4824 6304 (0) 16 ***

**3900 19.3 4.6 0.1 L1HS_X_69c 20704 21531 (58921) + L1MA9 LINE/L1 5366 6230 (82) 17**

**1193 10.1 6.5 0.5 L1HS_X_69c 21550 21749 (58703) C L1MA2 LINE/L1 (0) 6304 6093 18**

**237 30.5 0.0 0.0 L1HS_X_69c 21760 21818 (58634) C FLAM_A SINE/Alu (72) 60 2 19**

**1875 14.5 0.8 2.3 L1HS_X_69c 22065 22427 (58025) C L1MA2 LINE/L1 (273) 6031 5666 20 ***

**5224 15.3 1.9 0.5 L1HS_X_69c 22397 24104 (56348) C L1PB3 LINE/L1 (3) 6151 4418 21**

**3178 17.6 3.7 0.0 L1HS_X_69c 24494 25142 (55310) C L1PB3 LINE/L1 (1744) 4402 3730 21**

**3493 30.2 2.7 3.4 L1HS_X_69c 25147 26570 (53882) C L1PBa LINE/L1 (5447) 1416 3 22**

**4016 19.4 3.5 2.7 L1HS_X_69c 26633 27730 (52722) + L1MB5 LINE/L1 5054 6165 (9) 23**

**6485 16.2 0.8 1.0 L1HS_X_69c 27731 28997 (51455) + L1MA5A LINE/L1 4148 5411 (886) 24**

**3583 16.1 1.4 2.0 L1HS_X_69c 29017 29850 (50602) + L1MA5A LINE/L1 5397 6297 (0) 24**

**364 37.0 6.1 0.6 L1HS_X_69c 29960 30122 (50330) + MIRc SINE/MIR 2 173 (95) 25**

**1312 30.7 17.8 3.9 L1HS_X_69c 30296 30764 (49688) C L2c LINE/L2 (1) 3386 2847 26**

**1814 12.4 9.9 2.0 L1HS_X_69c 30765 31117 (49335) C THE1D LTR/MaLR (0) 381 1 27**

**1312 33.7 7.5 2.2 L1HS_X_69c 31118 31621 (48831) C L2c LINE/L2 (573) 2846 2316 26**

**509 28.6 6.7 2.4 L1HS_X_69c 32806 32970 (47482) + MIR3 SINE/MIR 34 205 (3) 28**

**1095 24.4 2.0 0.8 L1HS_X_69c 33552 33799 (46653) C MIR SINE/MIR (0) 262 12 29**

**668 29.1 0.0 5.0 L1HS_X_69c 33833 34013 (46439) C MIR3 SINE/MIR (21) 187 16 30**

**181 19.0 2.3 2.3 L1HS_X_69c 35145 35187 (45265) C L2c LINE/L2 (0) 3387 3345 31**

**364 27.9 6.2 5.6 L1HS_X_69c 35322 35465 (44987) C MIR3 SINE/MIR (1) 207 63 32**

**4940 20.8 2.8 4.2 L1HS_X_69c 35614 35918 (44534) C MER45B DNA/Tip100 (0) 1040 740 33**

**1721 18.8 0.0 2.0 L1HS_X_69c 35919 36211 (44241) + AluJb SINE/Alu 1 287 (25) 34**

**4940 20.8 2.8 4.2 L1HS_X_69c 36212 36960 (43492) C MER45B DNA/Tip100 (301) 739 1 33**

**589 20.0 5.3 3.3 L1HS_X_69c 36961 37019 (43433) C L1ME2z LINE/L1 (36) 6408 6348 35**

**2012 13.8 0.0 0.7 L1HS_X_69c 37020 37318 (43134) C AluSx SINE/Alu (15) 297 1 36**

**589 20.0 5.3 3.3 L1HS_X_69c 37319 37408 (43044) C L1ME2z LINE/L1 (97) 6347 6256 35**

**1403 19.8 9.4 1.4 L1HS_X_69c 37748 38110 (42342) C L1ME2z LINE/L1 (77) 6367 5976 37**

**332 26.8 4.8 6.1 L1HS_X_69c 38241 38270 (42182) C MIR SINE/MIR (10) 252 222 38**

**2354 14.3 7.5 0.5 L1HS_X_69c 38271 38670 (41782) + MSTA LTR/MaLR 1 428 (0) 39**

**3828 28.7 11.6 3.8 L1HS_X_69c 38673 40280 (40172) + MSTA-int LTR/MaLR 2 1735 (0) 39**

**2212 19.5 4.4 0.0 L1HS_X_69c 40281 40690 (39762) + MSTA LTR/MaLR 1 428 (0) 39**

**314 23.6 3.0 3.7 L1HS_X_69c 40691 40802 (39650) C MIR SINE/MIR (41) 227 108 38**

**1313 20.9 5.2 0.3 L1HS_X_69c 40973 41318 (39134) + L1MD LINE/L1 4938 5300 (846) 40**

**5161 6.9 0.0 0.0 L1HS_X_69c 41327 42056 (38396) + L1M1 LINE/L1 3541 4270 (3295) 41**

**23617 6.6 0.4 0.1 L1HS_X_69c 42059 48186 (32266) + L1PA4 LINE/L1 2 6154 (1) 42**

**325 31.9 1.7 0.8 L1HS_X_69c 48202 48318 (32134) + L1M2 LINE/L1 4144 4261 (1882) 43**

**1276 10.0 35.0 0.0 L1HS_X_69c 48322 48541 (31911) + AluSq SINE/Alu 2 298 (5) 44**

**273 23.8 5.2 5.2 L1HS_X_69c 48545 48735 (31717) + L1M2 LINE/L1 4249 4439 (1701) 43**

**16593 12.0 0.9 0.4 L1HS_X_69c 49990 52755 (27697) C L1P2 LINE/L1 (978) 5483 2704 45**

**10207 11.9 1.5 0.6 L1HS_X_69c 52936 55147 (25305) C L1P2 LINE/L1 (3771) 2690 470 45**

**6512 16.1 3.0 4.9 L1HS_X_69c 55142 55510 (24942) C L1P4 LINE/L1 (757) 5389 5027 46 ***

**660 23.3 0.0 0.0 L1HS_X_69c 55511 55643 (24809) + FLAM_A SINE/Alu 1 133 (0) 47**

**6512 16.1 3.0 4.9 L1HS_X_69c 55644 56796 (23656) C L1P4 LINE/L1 (1120) 5026 3896 46**

**1098 12.6 0.6 0.6 L1HS_X_69c 56794 56976 (23476) C L1PREC2 LINE/L1 (1411) 4735 4553 48 ***

**4221 3.1 2.9 0.7 L1HS_X_69c 56977 57521 (22931) C L1PA4 LINE/L1 (5) 6150 5594 49**

**2927 17.7 2.5 0.3 L1HS_X_69c 58754 59388 (21064) C L1P4 LINE/L1 (2758) 3388 2740 46 ***

**6335 11.8 0.4 3.2 L1HS_X_69c 59382 60753 (19699) C L1PA7 LINE/L1 (8) 6146 4800 50**

**14924 25.8 0.6 1.3 L1HS_X_69c 60770 64710 (15742) C L1P2 LINE/L1 (813) 5333 1438 51**

**9754 15.7 1.8 0.5 L1HS_X_69c 64730 66939 (13513) C L1PREC2 LINE/L1 (3380) 4761 2520 52**

**461 23.0 0.0 0.0 L1HS_X_69c 66936 67022 (13430) C L1PREC2 LINE/L1 (5523) 2018 1932 52 ***

**278 30.1 0.0 0.0 L1HS_X_69c 67028 67100 (13352) + L1PA13 LINE/L1 1168 1240 (5545) 53**

**3884 17.4 0.4 0.9 L1HS_X_69c 67113 67853 (12599) + L1PA13 LINE/L1 1388 2124 (4661) 53**

**2192 23.8 5.4 0.3 L1HS_X_69c 68018 68603 (11849) C L1PA12 LINE/L1 (5812) 2348 1701 54**

**2043 14.7 0.6 0.3 L1HS_X_69c 68590 68924 (11528) C L1PA12 LINE/L1 (4977) 2004 1669 55 ***

**7132 16.6 1.2 0.7 L1HS_X_69c 68921 70257 (10195) C L1PA12 LINE/L1 (5779) 1762 3 54**

**357 16.7 0.0 1.5 L1HS_X_69c 70260 70326 (10126) + MLT1B LTR/MaLR 325 390 (0) 56**

**33 0.0 0.0 0.0 L1HS_X_69c 70421 70453 (9999) + AT_rich Low_complexity 1 33 (0) 57**

**1182 25.0 2.4 3.6 L1HS_X_69c 70475 70810 (9642) C MLT1B LTR/MaLR (0) 390 59 58**

**561 15.9 8.3 0.0 L1HS_X_69c 71170 71301 (9151) + L1MC4a LINE/L1 7739 7881 (1) 59**

**341 18.2 0.0 0.0 L1HS_X_69c 71492 71557 (8895) + L1PA12 LINE/L1 116 181 (6800) 60**

**293 30.7 0.0 4.3 L1HS_X_69c 71565 71656 (8796) + MIRb SINE/MIR 61 148 (120) 61**

**264 20.5 6.4 6.4 L1HS_X_69c 71745 71838 (8614) + MIRc SINE/MIR 114 207 (61) 62**

**498 32.9 1.2 0.6 L1HS_X_69c 72571 72735 (7717) + MIRc SINE/MIR 28 193 (75) 63**

**295 36.7 1.6 0.0 L1HS_X_69c 73025 73152 (7300) + L2c LINE/L2 3243 3372 (15) 64**

**196 35.9 12.0 0.0 L1HS_X_69c 73261 73352 (7100) C L2b LINE/L2 (9) 3366 3263 65**

**428 27.7 14.0 1.3 L1HS_X_69c 73353 73509 (6943) C MER5B DNA/MER1_type (0) 178 2 66**

**196 35.9 12.0 0.0 L1HS_X_69c 73510 73533 (6919) C L2b LINE/L2 (113) 3262 3236 65**

**1187 19.9 8.9 1.5 L1HS_X_69c 73610 73936 (6516) + L1MDa LINE/L1 1891 2241 (4392) 67**

**1440 24.4 3.9 1.5 L1HS_X_69c 73944 74351 (6101) + L1MD2 LINE/L1 5640 6057 (302) 68 ***

**10761 14.9 0.7 0.8 L1HS_X_69c 74351 77361 (3091) + L1PA10 LINE/L1 8 3033 (3428) 69**

**278 31.6 0.0 2.2 L1HS_X_69c 77602 77737 (2715) + L1P1 LINE/L1 2588 2720 (3426) 70**

**10238 13.8 3.2 1.2 L1HS_X_69c 77747 80450 (2) + L1PBa1 LINE/L1 254 3056 (3806) 71**

**_________________________________________________________________________**

**132. L1HS_X_75c**

**Ortholog annotation INDEL_CAN Length 74 nscore 13.51 NPOSITIONS 1 10 ;**

**Repeat length (main genome) 908**

**Blast2 Results -**

**OFC OLC RFC RLC**

**no hits found**

**Ortholog Repeat Masker annotation**

**SW perc perc perc query position in query matching repeat position in repeat**

**score div. del. ins. sequence begin end (left) repeat class/family begin end (left) ID**

**445 7.9 1.6 0.0 L1HS_X_75c 12 74 (0) C L1M1 LINE/L1 (2200) 3946 3883 1**

**_________________________________________________________________________**

**133. L1HS_X_76**

**Ortholog annotation INDEL_PAC Length 74 nscore 13.51 NPOSITIONS 65 74 ;**

**Repeat length (main genome) 656**

**Blast2 Results -**

**OFC OLC RFC RLC**

**no hits found**

**Ortholog Repeat Masker annotation**

**SW perc perc perc query position in query matching repeat position in repeat**

**score div. del. ins. sequence begin end (left) repeat class/family begin end (left) ID**

**445 7.9 1.6 0.0 L1HS_X_76 1 63 (11) + L1M1 LINE/L1 3883 3946 (2200) 1**

**_________________________________________________________________________**

**134. L1HS_X_85**

**Ortholog annotation C_INTER_RMD_M_DISRUPTED Length 5445 nscore 3.36 NPOSITIONS 2201 2371 ; 2431 2431 ; 2434 2434 ; 5424 5433 ;**

**Repeat length (main genome) 5537**

**Blast2 Results -**

**OFC OLC RFC RLC**

**1 2057 1 2058**

**2378 5266 2185 5082**

**Ortholog Repeat Masker annotation**

**SW perc perc perc query position in query matching repeat position in repeat**

**score div. del. ins. sequence begin end (left) repeat class/family begin end (left) ID**

**1942 12.5 0.0 0.0 L1HS_X_85 1 289 (5156) + L1P1 LINE/L1 11 299 (5856) 1**

**12078 7.7 0.8 0.1 L1HS_X_85 320 2098 (3347) + L1P1 LINE/L1 559 2351 (3795) 1**

**20821 6.5 1.2 0.2 L1HS_X_85 2372 5349 (96) + L1P1 LINE/L1 2420 5430 (724) 1**

**_________________________________________________________________________**

**135. L1HS_Y_14c**

**Ortholog annotation INDEL_CAN Length 5029 nscore 0.00 NPOSITIONS NA**

**Repeat length (main genome) 2067**

**Blast2 Results -**

**OFC OLC RFC RLC**

**Ortholog Repeat Masker annotation**

**SW perc perc perc query position in query matching repeat position in repeat**

**score div. del. ins. sequence begin end (left) repeat class/family begin end (left) ID**

**423 34.1 3.3 2.4 L1HS_Y_14c 3 212 (4817) + MER102a DNA/MER1_type 117 328 (2) 1**

**848 12.6 0.0 3.8 L1HS_Y_14c 285 416 (4613) C AluSq/x SINE/Alu (185) 127 1 2**

**200 34.2 1.4 1.4 L1HS_Y_14c 428 501 (4528) + L2a LINE/L2 3344 3417 (9) 3**

**840 26.1 6.1 2.6 L1HS_Y_14c 1185 1412 (3617) + MIR SINE/MIR 1 236 (26) 4**

**41 2.1 0.0 0.0 L1HS_Y_14c 1606 1653 (3376) + AT_rich Low_complexity 1 48 (0) 5**

**794 26.0 10.1 6.9 L1HS_Y_14c 1789 2041 (2988) C L1M5 LINE/L1 (1777) 4369 4108 6**

**2103 13.5 0.0 0.0 L1HS_Y_14c 2042 2344 (2685) C AluSx SINE/Alu (9) 303 1 7**

**794 26.0 10.1 6.9 L1HS_Y_14c 2345 2673 (2356) C L1M5 LINE/L1 (2039) 4107 3768 6**

**242 14.0 3.5 0.0 L1HS_Y_14c 3440 3496 (1533) + (TCTA)n Simple_repeat 3 61 (0) 8**

**197 32.8 0.0 0.0 L1HS_Y_14c 3741 3807 (1222) + L2c LINE/L2 3092 3158 (229) 9**

**_________________________________________________________________________**

**136. L1HS_Y_15**

**Ortholog annotation INDEL_CAN Length 5029 nscore 0.00 NPOSITIONS NA**

**Repeat length (main genome) 884**

**Blast2 Results -**

**OFC OLC RFC RLC**

**Ortholog Repeat Masker annotation**

**SW perc perc perc query position in query matching repeat position in repeat**

**score div. del. ins. sequence begin end (left) repeat class/family begin end (left) ID**

**197 32.8 0.0 0.0 L1HS_Y_15 1223 1289 (3740) C L2c LINE/L2 (229) 3158 3092 1**

**242 14.0 3.5 0.0 L1HS_Y_15 1534 1590 (3439) + (TAGA)n Simple_repeat 3 61 (0) 2**

**794 26.0 10.1 6.9 L1HS_Y_15 2357 2685 (2344) + L1M5 LINE/L1 3768 4108 (2038) 3**

**2103 13.5 0.0 0.0 L1HS_Y_15 2686 2988 (2041) + AluSx SINE/Alu 1 303 (9) 4**

**794 26.0 10.1 6.9 L1HS_Y_15 2989 3241 (1788) + L1M5 LINE/L1 4109 4369 (1777) 3**

**41 2.1 0.0 0.0 L1HS_Y_15 3377 3424 (1605) + AT_rich Low_complexity 1 48 (0) 5**

**840 26.1 6.1 2.6 L1HS_Y_15 3618 3845 (1184) C MIR SINE/MIR (26) 236 1 6**

**200 34.2 1.4 1.4 L1HS_Y_15 4529 4602 (427) C L2a LINE/L2 (9) 3417 3344 7**

**848 12.6 0.0 3.8 L1HS_Y_15 4614 4745 (284) + AluSq/x SINE/Alu 1 127 (185) 8**

**423 34.1 3.3 2.4 L1HS_Y_15 4818 5027 (2) C MER102a DNA/MER1_type (2) 328 117 9**

**_________________________________________________________________________**

**137. L1HS_Y_31c**

**Ortholog annotation C_DISRUPTED_M_INTER_RMD Length 16089 nscore 0.00 NPOSITIONS NA**

**Repeat length (main genome) 4735**

**Blast2 Results -**

**OFC OLC RFC RLC**

**1 4743 1 4735**

**11280 11305 4 29**

**11306 11343 62 99**

**11349 11927 119 700**

**12124 16089 775 4734**

**Ortholog Repeat Masker annotation**

**SW perc perc perc query position in query matching repeat position in repeat**

**score div. del. ins. sequence begin end (left) repeat class/family begin end (left) ID**

**25475 3.7 0.2 0.3 L1HS_Y_31c 1 6035 (10054) + L1PA2 LINE/L1 127 6155 (0) 1**

**2248 17.1 7.9 5.0 L1HS_Y_31c 6035 6657 (9432) C L1PREC2 LINE/L1 (1178) 4968 4328 2 ***

**6044 9.4 1.1 1.6 L1HS_Y_31c 6670 7345 (8744) C L1PA6 LINE/L1 (0) 6154 5481 3**

**2175 13.4 0.0 0.0 L1HS_Y_31c 7346 7651 (8438) C AluY SINE/Alu (5) 306 1 4**

**19420 11.2 1.3 1.0 L1HS_Y_31c 7652 11272 (4817) C L1PA6 LINE/L1 (674) 5480 1874 3**

**19955 7.1 1.3 0.1 L1HS_Y_31c 11274 16089 (0) + L1P1 LINE/L1 1 4876 (1270) 5**

**_________________________________________________________________________**

**138. L1HS_Y_6**

**Ortholog annotation C_INTER_RMD_M_DISRUPTED Length 59582 nscore 0.17 NPOSITIONS 57932 58031 ;**

**Repeat length (main genome) 846**

**Blast2 Results -**

**OFC OLC RFC RLC**

**1 846 1 846**

**52889 53004 23 138**

**Ortholog Repeat Masker annotation**

**SW perc perc perc query position in query matching repeat position in repeat**

**score div. del. ins. sequence begin end (left) repeat class/family begin end (left) ID**

**24894 4.7 0.7 0.5 L1HS_Y_6 1 6023 (53559) + L1PA3 LINE/L1 124 6155 (0) 1**

**313 24.2 7.2 0.4 L1HS_Y_6 6096 6346 (53236) + L1M5 LINE/L1 4247 4514 (1632) 2**

**991 30.9 5.2 1.9 L1HS_Y_6 6454 7030 (52552) + L1MC LINE/L1 4532 5127 (1019) 3**

**1420 22.9 10.1 0.3 L1HS_Y_6 7049 7416 (52166) C MLT1A1 LTR/MaLR (4) 404 1 4**

**2068 26.9 7.3 2.1 L1HS_Y_6 7418 8074 (51508) C MLT1A1-int LTR/MaLR (1) 1734 1044 4**

**1709 15.7 8.3 2.5 L1HS_Y_6 8110 8272 (51310) + THE1D LTR/MaLR 1 173 (208) 5**

**2355 14.1 1.9 0.0 L1HS_Y_6 8273 8640 (50942) + THE1C LTR/MaLR 1 375 (0) 6**

**1709 15.7 8.3 2.5 L1HS_Y_6 8641 8836 (50746) + THE1D LTR/MaLR 174 381 (0) 5**

**838 26.6 1.6 4.6 L1HS_Y_6 8837 9103 (50479) C MLT1A1-int LTR/MaLR (668) 1067 759 4**

**3326 20.5 2.7 5.1 L1HS_Y_6 9145 9582 (50000) C MLT1A1-int LTR/MaLR (933) 802 375 4**

**2299 9.6 0.0 1.6 L1HS_Y_6 9583 9898 (49684) C AluY SINE/Alu (0) 311 1 7**

**3326 20.5 2.7 5.1 L1HS_Y_6 9899 10280 (49302) C MLT1A1-int LTR/MaLR (1361) 374 2 4**

**861 17.3 0.7 0.0 L1HS_Y_6 10323 10472 (49110) C MLT1A1 LTR/MaLR (74) 334 184 8**

**474 38.9 10.0 2.8 L1HS_Y_6 10473 10832 (48750) + L2b LINE/L2 2984 3369 (6) 9**

**213 14.0 5.3 0.0 L1HS_Y_6 11287 11343 (48239) + (TA)n Simple_repeat 2 61 (0) 10**

**1576 14.9 5.3 0.8 L1HS_Y_6 11447 11709 (47873) + AluSg SINE/Alu 1 275 (35) 11**

**1757 16.6 0.7 1.7 L1HS_Y_6 11813 12106 (47476) C AluJb SINE/Alu (21) 291 1 12**

**21 8.2 0.0 0.0 L1HS_Y_6 12148 12196 (47386) + AT_rich Low_complexity 1 49 (0) 13**

**4125 4.7 0.0 0.0 L1HS_Y_6 12480 12985 (46597) C L1PA3 LINE/L1 (3) 6152 5647 14**

**2119 14.4 0.0 0.3 L1HS_Y_6 13336 13641 (45941) + AluSg SINE/Alu 1 305 (5) 15**

**305 35.8 4.4 5.0 L1HS_Y_6 13888 14046 (45536) C MIR3 SINE/MIR (49) 159 2 16**

**363 32.7 12.8 1.0 L1HS_Y_6 14667 14870 (44712) + L3 LINE/CR1 3735 3962 (527) 17 ***

**537 23.2 5.8 5.0 L1HS_Y_6 14845 15047 (44535) + MIR SINE/MIR 64 268 (0) 18**

**813 35.3 11.7 2.5 L1HS_Y_6 15053 15531 (44051) + L3 LINE/CR1 3966 4488 (1) 17**

**2066 15.1 0.3 0.0 L1HS_Y_6 15804 16115 (43467) + AluSp SINE/Alu 1 313 (0) 19**

**2005 15.5 0.0 1.0 L1HS_Y_6 16173 16479 (43103) + AluSx SINE/Alu 1 304 (8) 20**

**22 5.6 0.0 0.0 L1HS_Y_6 17005 17040 (42542) + AT_rich Low_complexity 1 36 (0) 21**

**505 25.1 4.1 0.0 L1HS_Y_6 17106 17276 (42306) + L1MA4 LINE/L1 5999 6176 (124) 22**

**1126 25.3 4.7 1.8 L1HS_Y_6 17290 17396 (42186) + AluJo SINE/Alu 14 124 (188) 23**

**291 2.8 0.0 0.0 L1HS_Y_6 17397 17432 (42150) + (CA)n Simple_repeat 1 36 (0) 24**

**1126 25.3 4.7 1.8 L1HS_Y_6 17433 17602 (41980) + AluJo SINE/Alu 125 299 (13) 23**

**1366 20.7 3.6 1.4 L1HS_Y_6 17664 17942 (41640) C AluSx SINE/Alu (25) 287 3 25**

**1439 18.3 3.7 0.4 L1HS_Y_6 18433 18701 (40881) + AluSg SINE/Alu 12 289 (21) 26**

**1076 20.7 11.4 2.5 L1HS_Y_6 20625 21026 (38556) + L1MB5 LINE/L1 5254 5691 (483) 27**

**755 26.2 2.3 1.9 L1HS_Y_6 21142 21424 (38158) + L1MB5 LINE/L1 5905 6184 (0) 27**

**1128 28.1 2.3 3.5 L1HS_Y_6 22210 22726 (36856) + L1MA8 LINE/L1 5758 6268 (23) 28**

**6656 7.4 0.1 0.0 L1HS_Y_6 22748 23760 (35822) + L1PA5 LINE/L1 5137 6150 (4) 29**

**22 3.5 0.0 0.0 L1HS_Y_6 23764 23792 (35790) + AT_rich Low_complexity 1 29 (0) 30**

**266 16.7 0.0 0.0 L1HS_Y_6 23843 23884 (35698) + (TA)n Simple_repeat 2 43 (0) 31**

**512 2.4 3.6 1.2 L1HS_Y_6 23898 23980 (35602) + (TTATA)n Simple_repeat 5 89 (0) 32**

**842 7.6 4.6 1.2 L1HS_Y_6 23983 24155 (35427) + (TA)n Simple_repeat 2 180 (0) 33**

**866 26.2 2.7 5.0 L1HS_Y_6 24301 24597 (34985) + AluJb SINE/Alu 14 303 (9) 34**

**314 33.7 5.4 2.4 L1HS_Y_6 24633 24799 (34783) + L1M4c LINE/L1 1192 1363 (5021) 35**

**213 24.7 0.0 9.4 L1HS_Y_6 25545 25629 (33953) + GA-rich Low_complexity 3 79 (0) 36**

**393 9.5 0.0 0.0 L1HS_Y_6 27927 27989 (31593) + (CA)n Simple_repeat 2 64 (0) 37**

**195 29.5 15.6 3.7 L1HS_Y_6 28616 28724 (30858) + MIR3 SINE/MIR 63 184 (24) 38**

**182 12.0 0.0 5.7 L1HS_Y_6 30509 30561 (29021) + CT-rich Low_complexity 1 50 (0) 39**

**310 23.6 5.7 11.3 L1HS_Y_6 30565 30688 (28894) C HAL1-3A_ME LINE/L1 (2309) 549 433 40**

**1088 16.8 0.3 0.2 L1HS_Y_6 32485 44606 (14976) + ALR/Alpha Satellite/centr 1 12138 (0) 41**

**978 15.8 0.4 0.4 L1HS_Y_6 44621 45124 (14458) C ALR/Alpha Satellite/centr (8) 504 1 42**

**1102 15.8 0.2 0.1 L1HS_Y_6 45137 52881 (6701) C ALR/Alpha Satellite/centr (12) 7750 1 42**

**859 9.8 0.0 0.0 L1HS_Y_6 52882 53004 (6578) + L1HS LINE/L1 16 138 (5894) 43**

**302 28.3 12.6 3.7 L1HS_Y_6 53433 53623 (5959) + HAL1-3A_ME LINE/L1 1988 2195 (663) 44**

**183 16.7 0.0 0.0 L1HS_Y_6 54754 54795 (4787) + (CA)n Simple_repeat 1 42 (0) 45**

**298 24.0 0.8 0.8 L1HS_Y_6 54806 54935 (4647) + GA-rich Low_complexity 2 131 (0) 46**

**830 13.3 0.0 0.0 L1HS_Y_6 55476 55595 (3987) C 5S rRNA (0) 121 2 47**

**305 30.4 5.9 8.4 L1HS_Y_6 56389 56454 (3128) + HAL1-3A_ME LINE/L1 1976 2040 (818) 48**

**3503 8.7 0.2 0.0 L1HS_Y_6 56455 56916 (2666) + LTR2 LTR/ERV1 1 463 (0) 49**

**305 30.4 5.9 8.4 L1HS_Y_6 56917 57086 (2496) + HAL1-3A_ME LINE/L1 2041 2206 (652) 48**

**186 7.1 0.0 0.0 L1HS_Y_6 57884 57911 (1671) + (G)n Simple_repeat 1 28 (0) 50**

**6562 3.5 0.6 0.0 L1HS_Y_6 58755 59582 (0) C L1PA2 LINE/L1 (0) 6155 5323 51**

**_________________________________________________________________________**

**Supplementary file 2 -Truncated L1HS comparison with Celera genome.**

**The file summarizes the blast2 result of the identified ortholog for INDELS and recombination, with the L1HS repeat and also gives the repeat masker annotation of the identified orthologous locus, along with N details.**

**1. L1HS_10_30c**

**Ortholog annotation C_INTRA_RMD Length 4162 nscore 5.00 NPOSITIONS 2004 2211 ;**

**Repeat length (main genome) 4860**

**Blast2 Results -**

**OFC OLC RFC RLC**

**1 2003 1 2003**

**2212 4162 2910 4860**

**Ortholog Repeat Masker annotation**

**SW perc perc perc query position in query matching repeat position in repeat**

**score div. del. ins. sequence begin end (left) repeat class/family begin end (left) ID**

**16062 3.2 0.1 0.1 L1HS_10_30c 1 2003 (2159) + L1HS LINE/L1 124 2127 (4028) 1**

**15722 2.3 0.1 0.0 L1HS_10_30c 2212 4162 (0) + L1HS LINE/L1 3034 4985 (1161) 1**

**__________________________________________________________________________________**

**2. L1HS_11_30c**

**Ortholog annotation C_INTER_RMD_M_DISRUPTED Length 113 nscore 0.00 NPOSITIONS NA**

**Repeat length (main genome) 87**

**Blast2 Results -**

**OFC OLC RFC RLC**

**1 87 1 87**

**Ortholog Repeat Masker annotation**

**SW perc perc perc query position in query matching repeat position in repeat**

**score div. del. ins. sequence begin end (left) repeat class/family begin end (left) ID**

**737 1.1 0.0 0.0 L1HS_11_30c 1 87 (26) + L1HS LINE/L1 6069 6155 (0) 1**

**22 0.0 0.0 0.0 L1HS_11_30c 92 113 (0) + AT_rich Low_complexity 1 22 (0) 2**

**__________________________________________________________________________________**

**3. L1HS_11_41c**

**Ortholog annotation C_DISRUPTED_M_INTER_RMD Length 3968 nscore 0.50 NPOSITIONS 2624 2643 ;**

**Repeat length (main genome) 2330**

**Blast2 Results -**

**OFC OLC RFC RLC**

**1 1428 1 1428**

**2644 3968 1006 2330**

**Ortholog Repeat Masker annotation**

**SW perc perc perc query position in query matching repeat position in repeat**

**score div. del. ins. sequence begin end (left) repeat class/family begin end (left) ID**

**11399 3.1 0.1 0.0 L1HS_11_41c 1 1428 (2540) + L1P1 LINE/L1 3873 5301 (845) 1 (R1)**

**270 0.0 0.0 0.0 L1HS_11_41c 1509 1538 (2430) + (TTTG)n Simple_repeat 1 30 (0) 2**

**2018 11.5 0.0 0.4 L1HS_11_41c 1542 1812 (2156) C AluSx SINE/Alu (35) 277 8 3**

**640 26.9 4.6 2.5 L1HS_11_41c 1914 2153 (1815) C L1M5 LINE/L1 (494) 5700 5456 4**

**421 26.6 4.8 0.8 L1HS_11_41c 2157 2281 (1687) + LTR33C LTR/ERVL 14 143 (486) 5**

**450 34.1 8.1 0.0 L1HS_11_41c 2305 2515 (1453) + MLT2F LTR/ERVL 189 416 (247) 6**

**280 11.5 0.0 0.0 L1HS_11_41c 2540 2591 (1377) C L1MB2 LINE/L1 (5) 6166 6115 7**

**7697 0.9 0.0 0.0 L1HS_11_41c 2644 3968 (0) + L1HS LINE/L1 4879 6155 (0) 8 (R2)**

**__________________________________________________________________________________**

**4. L1HS_12_41c**

**Ortholog annotation C_INTER_RMD_M_DISRUPTED Length 1813 nscore 0.00 NPOSITIONS NA**

**Repeat length (main genome) 1859**

**Blast2 Results -**

**OFC OLC RFC RLC**

**1 1813 47 1859**

**Ortholog Repeat Masker annotation**

**SW perc perc perc query position in query matching repeat position in repeat**

**score div. del. ins. sequence begin end (left) repeat class/family begin end (left) ID**

**9062 2.4 0.0 0.0 L1HS_12_41c 1 1813 (0) + L1HS LINE/L1 4340 6152 (3) 1**

**__________________________________________________________________________________**

**5. L1HS_12_42**

**Ortholog annotation INDEL_PTS Length 9891 nscore 0.20 NPOSITIONS 9802 9821 ;**

**Repeat length (main genome) 711**

**Blast2 Results -**

**OFC OLC RFC RLC**

**Ortholog Repeat Masker annotation**

**SW perc perc perc query position in query matching repeat position in repeat**

**score div. del. ins. sequence begin end (left) repeat class/family begin end (left) ID**

**18239 0.8 0.0 0.0 L1HS_12_42 1 4169 (5722) C L1HS LINE/L1 (1851) 4295 127 1**

**3398 18.2 3.9 0.2 L1HS_12_42 4884 6139 (3752) + L1MB3 LINE/L1 4665 5961 (222) 2**

**7816 20.0 4.6 2.0 L1HS_12_42 6166 8379 (1512) + L1M2 LINE/L1 1 2838 (4371) 3**

**225 0.0 0.0 0.0 L1HS_12_42 8380 8404 (1487) + (CAAAA)n Simple_repeat 1 25 (0) 4**

**7816 18.3 4.8 2.2 L1HS_12_42 8405 9384 (507) + L1M2 LINE/L1 2839 3831 (3374) 3**

**1851 17.8 0.3 1.3 L1HS_12_42 9385 9691 (200) + AluJb SINE/Alu 1 304 (8) 5**

**2965 17.4 5.2 2.1 L1HS_12_42 9692 9750 (141) + L1M2 LINE/L1 3832 3891 (3314) 3**

**__________________________________________________________________________________**

**6. L1HS_13_14**

**Ortholog annotation C_DISRUPTED_M_INTER_RMD Length 1040 nscore 1.92 NPOSITIONS 723 742 ;**

**Repeat length (main genome) 385**

**Blast2 Results -**

**OFC OLC RFC RLC**

**1 385 1 385**

**743 1040 87 384**

**Ortholog Repeat Masker annotation**

**SW perc perc perc query position in query matching repeat position in repeat**

**score div. del. ins. sequence begin end (left) repeat class/family begin end (left) ID**

**3544 0.0 0.0 0.0 L1HS_13_14 1 385 (655) + L1HS LINE/L1 5771 6155 (0) 1**

**1022 14.0 0.0 2.3 L1HS_13_14 388 563 (477) + L1MC LINE/L1 5283 5454 (879) 2**

**336 14.5 0.0 3.1 L1HS_13_14 538 601 (439) C L1MC1 LINE/L1 (127) 6206 6145 3 ***

**2750 0.0 0.0 0.0 L1HS_13_14 743 1040 (0) + L1HS LINE/L1 5857 6154 (1) 4**

**__________________________________________________________________________________**

**7. L1HS_18_2c**

**Ortholog annotation C_INTER_RMD_M_DISRUPTED Length 370 nscore 0.00 NPOSITIONS NA**

**Repeat length (main genome) 378**

**Blast2 Results -**

**OFC OLC RFC RLC**

**1 370 9 378**

**Ortholog Repeat Masker annotation**

**SW perc perc perc query position in query matching repeat position in repeat**

**score div. del. ins. sequence begin end (left) repeat class/family begin end (left) ID**

**3321 1.4 0.0 0.0 L1HS_18_2c 1 370 (0) + L1HS LINE/L1 5785 6154 (1) 1**

**__________________________________________________________________________________**

**8. L1HS_18_3c**

**Ortholog annotation C_INTER_RMD_M_DISRUPTED Length 147 nscore 0.00 NPOSITIONS NA**

**Repeat length (main genome) 163**

**Blast2 Results -**

**OFC OLC RFC RLC**

**1 147 1 147**

**Ortholog Repeat Masker annotation**

**SW perc perc perc query position in query matching repeat position in repeat**

**score div. del. ins. sequence begin end (left) repeat class/family begin end (left) ID**

**1347 0.7 0.0 0.0 L1HS_18_3c 1 147 (0) + L1P1 LINE/L1 5623 5769 (386) 1**

**__________________________________________________________________________________**

**9. L1HS_2_42**

**Ortholog annotation C_DISRUPTED_M_INTER_RMD Length 1857 nscore 0.00 NPOSITIONS NA**

**Repeat length (main genome) 1723**

**Blast2 Results -**

**OFC OLC RFC RLC**

**4 777 4 771**

**901 1857 767 1723**

**Ortholog Repeat Masker annotation**

**SW perc perc perc query position in query matching repeat position in repeat**

**score div. del. ins. sequence begin end (left) repeat class/family begin end (left) ID**

**15302 4.3 0.6 0.4 L1HS_2_42 1 1854 (3) + L1P1 LINE/L1 1 1858 (4297) 1**

**__________________________________________________________________________________**

**10. L1HS_3_116c**

**Ortholog annotation C_DISRUPTED_M_INTER_RMD Length 3720 nscore 12.96 NPOSITIONS 638 1119 ;**

**Repeat length (main genome) 3445**

**Blast2 Results -**

**OFC OLC RFC RLC**

**1 637 1 637**

**1120 3720 846 3445**

**Ortholog Repeat Masker annotation**

**SW perc perc perc query position in query matching repeat position in repeat**

**score div. del. ins. sequence begin end (left) repeat class/family begin end (left) ID**

**5434 0.2 0.0 0.0 L1HS_3_116c 1 637 (3083) + L1HS LINE/L1 2711 3347 (2799) 1**

**16291 0.3 0.0 0.0 L1HS_3_116c 1120 3720 (0) + L1HS LINE/L1 3556 6155 (0) 1**

**__________________________________________________________________________________**

**11. L1HS_3_14c**

**Ortholog annotation C_INTER_RMD_M_DISRUPTED Length 464 nscore 0.00 NPOSITIONS NA**

**Repeat length (main genome) 2806**

**Blast2 Results -**

**OFC OLC RFC RLC**

**1 464 2343 2806**

**Ortholog Repeat Masker annotation**

**SW perc perc perc query position in query matching repeat position in repeat**

**score div. del. ins. sequence begin end (left) repeat class/family begin end (left) ID**

**23 3.3 0.0 0.0 L1HS_3_14c 67 96 (368) + AT_rich Low_complexity 1 30 (0) 1**

**881 16.3 6.2 0.6 L1HS_3_14c 246 424 (40) + L1M2 LINE/L1 3370 3558 (2585) 2**

**__________________________________________________________________________________**

**12. L1HS_3_52**

**Ortholog annotation INDEL_CAN Length 3531 nscore 9.97 NPOSITIONS 2323 2674 ;**

**Repeat length (main genome) 2284**

**Blast2 Results -**

**OFC OLC RFC RLC**

**1 2284 1 2284**

**Ortholog Repeat Masker annotation**

**SW perc perc perc query position in query matching repeat position in repeat**

**score div. del. ins. sequence begin end (left) repeat class/family begin end (left) ID**

**13352 0.8 0.0 0.0 L1HS_3_52 1 2284 (1247) + L1HS LINE/L1 3870 6153 (2) 1**

**1080 16.4 0.3 0.0 L1HS_3_52 2681 3531 (0) C ALR/Alpha Satellite/centr (0) 853 1 2**

**__________________________________________________________________________________**

**13. L1HS_4_117c**

**Ortholog annotation C_DISRUPTED_M_INTER_RMD Length 1284 nscore 0.00 NPOSITIONS NA**

**Repeat length (main genome) 1098**

**Blast2 Results -**

**OFC OLC RFC RLC**

**1 1081 1 1081**

**1079 1284 892 1098**

**Ortholog Repeat Masker annotation**

**SW perc perc perc query position in query matching repeat position in repeat**

**score div. del. ins. sequence begin end (left) repeat class/family begin end (left) ID**

**8238 0.3 0.0 0.0 L1HS_4_117c 1 1081 (203) + L1P1 LINE/L1 4482 5562 (593) 1 (R1)**

**1841 0.5 0.5 0.0 L1HS_4_117c 1079 1284 (0) + L1P1 LINE/L1 5373 5579 (576) 2 * (R2)**

**__________________________________________________________________________________**

**14. L1HS_4_23c**

**Ortholog annotation C_INTER_RMD_M_DISRUPTED Length 53 nscore 0.00 NPOSITIONS NA**

**Repeat length (main genome) 1008**

**Blast2 Results -**

**OFC OLC RFC RLC**

**1 53 956 1008**

**Ortholog Repeat Masker annotation**

**SW perc perc perc query position in query matching repeat position in repeat**

**score div. del. ins. sequence begin end (left) repeat class/family begin end (left) ID**

**462 0.0 0.0 0.0 L1HS_4_23c 1 53 (0) + L1P1 LINE/L1 4962 5014 (1132) 1**

**__________________________________________________________________________________**

**15. L1HS_4_69**

**Ortholog annotation C_INTER_RMD Length 1273 nscore 1.57 NPOSITIONS 782 801 ;**

**Repeat length (main genome) 2197**

**Blast2 Results -**

**OFC OLC RFC RLC**

**1 781 1 783**

**Ortholog Repeat Masker annotation**

**SW perc perc perc query position in query matching repeat position in repeat**

**score div. del. ins. sequence begin end (left) repeat class/family begin end (left) ID**

**7031 2.6 0.2 1.1 L1HS_4_69 1 826 (447) + L1P1 LINE/L1 3960 4778 (1368) 1**

**1206 26.0 6.0 2.2 L1HS_4_69 810 1254 (19) + L1MD2 LINE/L1 5423 5905 (454) 2 ***

**__________________________________________________________________________________**

**16. L1HS_4_79c**

**Ortholog annotation C_INTRA_RMD Length 422 nscore 4.74 NPOSITIONS 150 169 ;**

**Repeat length (main genome) 774**

**Blast2 Results -**

**OFC OLC RFC RLC**

**1 149 1 149**

**170 422 522 774**

**Ortholog Repeat Masker annotation**

**SW perc perc perc query position in query matching repeat position in repeat**

**score div. del. ins. sequence begin end (left) repeat class/family begin end (left) ID**

**1350 0.0 0.0 0.0 L1HS_4_79c 1 149 (273) + L1P1 LINE/L1 5386 5534 (621) 1**

**2276 0.4 0.0 0.0 L1HS_4_79c 170 418 (4) + L1HS LINE/L1 5907 6155 (0) 2**

**__________________________________________________________________________________**

**17. L1HS_4_8**

**Ortholog annotation C_INTER_RMD_M_DISRUPTED Length 1841 nscore 0.00 NPOSITIONS NA**

**Repeat length (main genome) 1854**

**Blast2 Results -**

**OFC OLC RFC RLC**

**1 1841 1 1841**

**Ortholog Repeat Masker annotation**

**SW perc perc perc query position in query matching repeat position in repeat**

**score div. del. ins. sequence begin end (left) repeat class/family begin end (left) ID**

**16704 0.7 0.1 0.0 L1HS_4_8 1 1841 (0) + L1HS LINE/L1 3 1844 (4188) 1**

**__________________________________________________________________________________**

**18. L1HS_4_9**

**Ortholog annotation C_INTER_RMD_M_DISRUPTED Length 4173 nscore 0.00 NPOSITIONS NA**

**Repeat length (main genome) 4187**

**Blast2 Results -**

**OFC OLC RFC RLC**

**1 4173 14 4186**

**Ortholog Repeat Masker annotation**

**SW perc perc perc query position in query matching repeat position in repeat**

**score div. del. ins. sequence begin end (left) repeat class/family begin end (left) ID**

**27415 0.6 0.0 0.0 L1HS_4_9 1 4173 (0) + L1HS LINE/L1 1981 6153 (2) 1**

**__________________________________________________________________________________**

**19. L1HS_5_17**

**Ortholog annotation INDEL_PTS Length 431 nscore 0.00 NPOSITIONS NA**

**Repeat length (main genome) 416**

**Blast2 Results -**

**OFC OLC RFC RLC**

**Ortholog Repeat Masker annotation**

**SW perc perc perc query position in query matching repeat position in repeat**

**score div. del. ins. sequence begin end (left) repeat class/family begin end (left) ID**

**663 13.6 12.5 1.7 L1HS_5_17 43 162 (269) + FLAM_C SINE/Alu 6 138 (5) 1**

**557 15.1 0.0 0.0 L1HS_5_17 339 431 (0) C AluSg/x SINE/Alu (6) 306 214 2**

**__________________________________________________________________________________**

**20. L1HS_5_74**

**Ortholog annotation C_INTER_RMD Length 2746 nscore 0.73 NPOSITIONS 2258 2277 ;**

**Repeat length (main genome) 4561**

**Blast2 Results -**

**OFC OLC RFC RLC**

**1 2257 1 2254**

**Ortholog Repeat Masker annotation**

**SW perc perc perc query position in query matching repeat position in repeat**

**score div. del. ins. sequence begin end (left) repeat class/family begin end (left) ID**

**15408 0.7 0.0 0.2 L1HS_5_74 1 2257 (489) + L1HS LINE/L1 1666 3919 (2227) 1**

**1916 16.1 6.7 1.5 L1HS_5_74 2279 2743 (3) C L1M3 LINE/L1 (858) 5454 4966 2**

**__________________________________________________________________________________**

**21. L1HS_5_93c**

**Ortholog annotation INDEL_CAN Length 3780 nscore 0.53 NPOSITIONS 1 20 ;**

**Repeat length (main genome) 935**

**Blast2 Results -**

**OFC OLC RFC RLC**

**Ortholog Repeat Masker annotation**

**SW perc perc perc query position in query matching repeat position in repeat**

**score div. del. ins. sequence begin end (left) repeat class/family begin end (left) ID**

**21407 3.0 0.0 0.2 L1HS_5_93c 21 3397 (383) + L1PA2 LINE/L1 2786 6155 (0) 1**

**781 17.9 1.7 1.1 L1HS_5_93c 3398 3572 (208) C L1M4 LINE/L1 (3511) 3133 2958 2**

**264 18.6 5.1 0.0 L1HS_5_93c 3578 3636 (144) C L1M4 LINE/L1 (5108) 1434 1373 2**

**__________________________________________________________________________________**

**22. L1HS_7_15c**

**Ortholog annotation C_INTER_RMD_M_DISRUPTED Length 342 nscore 5.85 NPOSITIONS 323 342 ;**

**Repeat length (main genome) 1474**

**Blast2 Results -**

**OFC OLC RFC RLC**

**1 322 1 322**

**Ortholog Repeat Masker annotation**

**SW perc perc perc query position in query matching repeat position in repeat**

**score div. del. ins. sequence begin end (left) repeat class/family begin end (left) ID**

**2907 0.3 0.0 0.0 L1HS_7_15c 1 322 (20) + L1P1 LINE/L1 4723 5044 (1102) 1**

**__________________________________________________________________________________**

**23. L1HS_7_55c**

**Ortholog annotation C_INTRA_RMD Length 726 nscore 2.75 NPOSITIONS 474 493 ;**

**Repeat length (main genome) 1057**

**Blast2 Results -**

**OFC OLC RFC RLC**

**1 473 1 473**

**494 726 825 1057**

**Ortholog Repeat Masker annotation**

**SW perc perc perc query position in query matching repeat position in repeat**

**score div. del. ins. sequence begin end (left) repeat class/family begin end (left) ID**

**2811 0.6 0.0 0.0 L1HS_7_55c 1 473 (253) + L1P1 LINE/L1 5099 5571 (584) 1**

**2019 2.1 0.0 0.0 L1HS_7_55c 494 726 (0) + L1PA2 LINE/L1 5923 6155 (0) 2**

**__________________________________________________________________________________**

**24. L1HS_8_26**

**Ortholog annotation C_DISRUPTED_M_INTER_RMD Length 1340 nscore 1.49 NPOSITIONS 881 900 ;**

**Repeat length (main genome) 452**

**Blast2 Results -**

**OFC OLC RFC RLC**

**1 427 1 427**

**901 1340 12 451**

**1303 1329 425 452**

**Ortholog Repeat Masker annotation**

**SW perc perc perc query position in query matching repeat position in repeat**

**score div. del. ins. sequence begin end (left) repeat class/family begin end (left) ID**

**3831 0.0 0.0 0.0 L1HS_8_26 1 464 (876) + L1HS LINE/L1 5740 6155 (0) 1 (R1)**

**3647 0.0 0.0 0.0 L1HS_8_26 901 1340 (0) + L1HS LINE/L1 5751 6155 (0) 2 (R2)**

**__________________________________________________________________________________**

**25. L1HS_8_4**

**Ortholog annotation INDEL_PTS Length 5032 nscore 0.00 NPOSITIONS NA**

**Repeat length (main genome) 1071**

**Blast2 Results -**

**OFC OLC RFC RLC**

**Ortholog Repeat Masker annotation**

**SW perc perc perc query position in query matching repeat position in repeat**

**score div. del. ins. sequence begin end (left) repeat class/family begin end (left) ID**

**17224 5.2 0.0 0.3 L1HS_8_4 1 2560 (2472) C L1P1 LINE/L1 (3593) 2553 1 1**

**2272 14.4 9.7 0.3 L1HS_8_4 3932 4322 (710) C MSTA LTR/MaLR (0) 428 1 2**

**306 6.2 0.0 0.0 L1HS_8_4 4985 5032 (0) C AluYc3 SINE/Alu (16) 284 237 3**

**__________________________________________________________________________________**

**26. L1HS_8_5**

**Ortholog annotation M_INTRA_RMD Length 4796 nscore 0.00 NPOSITIONS NA**

**Repeat length (main genome) 3947**

**Blast2 Results -**

**OFC OLC RFC RLC**

**1 3943 1 3943**

**Ortholog Repeat Masker annotation**

**SW perc perc perc query position in query matching repeat position in repeat**

**score div. del. ins. sequence begin end (left) repeat class/family begin end (left) ID**

**24168 2.6 0.2 0.0 L1HS_8_5 1 4796 (0) + L1HS LINE/L1 125 4929 (1217) 1**

**__________________________________________________________________________________**

**27. L1HS_9_19**

**Ortholog annotation INDEL_PTS Length 99 nscore 0.00 NPOSITIONS NA**

**Repeat length (main genome) 40**

**Blast2 Results -**

**OFC OLC RFC RLC**

**no hits found**

**Ortholog Repeat Masker annotation**

**SW perc perc perc query position in query matching repeat position in repeat**

**score div. del. ins. sequence begin end (left) repeat class/family begin end (left) ID**

**198 0.0 0.0 0.0 L1HS_9_19 1 22 (77) + (TTTTTA)n Simple_repeat 2 23 (0) 1**

**__________________________________________________________________________________**

**Supplementary file 2 - Truncated L1HS comparison with HuRef genome.**

**The file summarizes the blast2 result of the identified ortholog with the L1HS repeat and also gives the repeat masker annotation of the identified orthologous locus, along with N details.**

**1. L1HS_10_25**

**Ortholog annotation C_INTRA_RMD Length 2373 nscore 0.84 NPOSITIONS 857 876 ;**

**Repeat length (main genome) 4911**

**Blast2 Results -**

**OFC OLC RFC RLC**

**1 856 1 855**

**879 2370 3416 4907**

**Ortholog Repeat Masker annotation**

**SW perc perc perc query position in query matching repeat position in repeat**

**score div. del. ins. sequence begin end (left) repeat class/family begin end (left) ID**

**7117 0.6 0.0 0.1 L1HS_10_25 1 856 (1517) + L1HS LINE/L1 1120 1974 (4058) 1**

**7608 0.9 0.0 0.0 L1HS_10_25 877 2370 (3) + L1HS LINE/L1 4656 6149 (6) 2**

**__________________________________________________________________________________**

**2. L1HS_11_30c**

**Ortholog annotation C_INTER_RMD_M_DISRUPTED Length 114 nscore 0.00 NPOSITIONS NA**

**Repeat length (main genome) 87**

**Blast2 Results -**

**OFC OLC RFC RLC**

**1 87 1 87**

**Ortholog Repeat Masker annotation**

**SW perc perc perc query position in query matching repeat position in repeat**

**score div. del. ins. sequence begin end (left) repeat class/family begin end (left) ID**

**737 1.1 0.0 0.0 L1HS_11_30c 1 87 (27) + L1HS LINE/L1 6069 6155 (0) 1**

**22 0.0 0.0 0.0 L1HS_11_30c 93 114 (0) + AT_rich Low_complexity 1 22 (0) 2**

**__________________________________________________________________________________**

**3. L1HS_11_57**

**Ortholog annotation C_DISRUPTED_M_INTER_RMD Length 4974 nscore 0.80 NPOSITIONS 1099 1118 ; 2437 2456 ;**

**Repeat length (main genome) 1299**

**Blast2 Results -**

**OFC OLC RFC RLC**

**1 372 1 372**

**3677 4974 1 1298**

**Ortholog Repeat Masker annotation**

**SW perc perc perc query position in query matching repeat position in repeat**

**score div. del. ins. sequence begin end (left) repeat class/family begin end (left) ID**

**3225 1.6 0.0 0.0 L1HS_11_57 1 372 (4602) + L1HS LINE/L1 1715 2086 (3946) 1 ***

**6360 2.7 0.0 0.0 L1HS_11_57 365 1098 (3876) + L1HS LINE/L1 353 1086 (4946) 2**

**11175 1.0 0.3 0.1 L1HS_11_57 1119 2436 (2538) C L1P1 LINE/L1 (705) 5450 4130 3**

**14449 1.6 0.1 0.0 L1HS_11_57 2457 4974 (0) + L1HS LINE/L1 618 3137 (3009) 4**

**__________________________________________________________________________________**

**4. L1HS_13_20c**

**Ortholog annotation C_INTRA_RMD Length 1548 nscore 1.29 NPOSITIONS 816 835 ;**

**Repeat length (main genome) 2282**

**Blast2 Results -**

**OFC OLC RFC RLC**

**1 809 1 809**

**837 1548 1571 2282**

**Ortholog Repeat Masker annotation**

**SW perc perc perc query position in query matching repeat position in repeat**

**score div. del. ins. sequence begin end (left) repeat class/family begin end (left) ID**

**7305 0.4 0.1 0.0 L1HS_13_20c 1 815 (733) + L1HS LINE/L1 1083 1898 (4257) 1**

**6138 0.6 0.1 0.1 L1HS_13_20c 837 1548 (0) + L1HS LINE/L1 2653 3364 (2782) 1**

**__________________________________________________________________________________**

**5. L1HS_13_34c**

**Ortholog annotation C_INTER_RMD_M_DISRUPTED Length 346 nscore 0.00 NPOSITIONS NA**

**Repeat length (main genome) 355**

**Blast2 Results -**

**OFC OLC RFC RLC**

**1 346 10 355**

**Ortholog Repeat Masker annotation**

**SW perc perc perc query position in query matching repeat position in repeat**

**score div. del. ins. sequence begin end (left) repeat class/family begin end (left) ID**

**2990 1.7 0.0 0.3 L1HS_13_34c 1 346 (0) + L1HS LINE/L1 589 933 (5099) 1**

**__________________________________________________________________________________**

**6. L1HS_13_35c**

**Ortholog annotation C_INTER_RMD Length 574 nscore 0.00 NPOSITIONS NA**

**Repeat length (main genome) 586**

**Blast2 Results -**

**OFC OLC RFC RLC**

**1 574 1 572**

**Ortholog Repeat Masker annotation**

**SW perc perc perc query position in query matching repeat position in repeat**

**score div. del. ins. sequence begin end (left) repeat class/family begin end (left) ID**

**4528 6.3 0.2 0.3 L1HS_13_35c 1 574 (0) + L1P1 LINE/L1 4 576 (5579) 1**

**__________________________________________________________________________________**

**7. L1HS_14_20**

**Ortholog annotation C_DISRUPTED_M_INTER_RMD Length 4511 nscore 0.44 NPOSITIONS 4156 4175 ;**

**Repeat length (main genome) 4188**

**Blast2 Results -**

**OFC OLC RFC RLC**

**1 4155 1 4154**

**4176 4511 3852 4187**

**Ortholog Repeat Masker annotation**

**SW perc perc perc query position in query matching repeat position in repeat**

**score div. del. ins. sequence begin end (left) repeat class/family begin end (left) ID**

**18571 3.3 0.3 0.1 L1HS_14_20 1 4155 (356) + L1HS LINE/L1 124 4291 (1855) 1**

**2985 1.8 0.0 0.0 L1HS_14_20 4176 4510 (1) + L1P1 LINE/L1 3989 4323 (1823) 2**

**__________________________________________________________________________________**

**8. L1HS_15_2**

**Ortholog annotation INDEL_PTS Length 8067 nscore 0.00 NPOSITIONS NA**

**Repeat length (main genome) 627**

**Blast2 Results -**

**OFC OLC RFC RLC**

**Ortholog Repeat Masker annotation**

**SW perc perc perc query position in query matching repeat position in repeat**

**score div. del. ins. sequence begin end (left) repeat class/family begin end (left) ID**

**1096 17.4 0.4 0.5 L1HS_15_2 3 3433 (4634) C ALR/Alpha Satellite/centr (28) 3429 1 1**

**7659 2.2 0.1 0.1 L1HS_15_2 3449 5089 (2978) C L1PA2 LINE/L1 (0) 6155 4534 2**

**798 4.6 0.0 0.0 L1HS_15_2 5082 5189 (2878) C L1PA2 LINE/L1 (1737) 4409 4302 2 ***

**7055 2.6 0.0 0.0 L1HS_15_2 5189 6032 (2035) + L1PA3 LINE/L1 5328 6155 (0) 3**

**1107 16.0 0.1 0.1 L1HS_15_2 6033 8064 (3) C ALR/Alpha Satellite/centr (14) 2033 1 4**

**__________________________________________________________________________________**

**9. L1HS_15_20**

**Ortholog annotation C_INTRA_RMD Length 5213 nscore 0.38 NPOSITIONS 1756 1775 ;**

**Repeat length (main genome) 5416**

**Blast2 Results -**

**OFC OLC RFC RLC**

**1 1755 1 1755**

**1776 5213 1978 5415**

**Ortholog Repeat Masker annotation**

**SW perc perc perc query position in query matching repeat position in repeat**

**score div. del. ins. sequence begin end (left) repeat class/family begin end (left) ID**

**12874 0.4 0.1 0.0 L1HS_15_20 1 1755 (3458) + L1HS LINE/L1 738 2494 (3652) 1**

**22595 0.3 0.0 0.0 L1HS_15_20 1776 5213 (0) + L1HS LINE/L1 2717 6154 (1) 1**

**__________________________________________________________________________________**

**10. L1HS_16_17c**

**Ortholog annotation C_INTER_RMD Length 1080 nscore 1.85 NPOSITIONS 1 20 ;**

**Repeat length (main genome) 1196**

**Blast2 Results -**

**OFC OLC RFC RLC**

**21 1080 138 1196**

**Ortholog Repeat Masker annotation**

**SW perc perc perc query position in query matching repeat position in repeat**

**score div. del. ins. sequence begin end (left) repeat class/family begin end (left) ID**

**9239 0.8 0.0 0.1 L1HS_16_17c 21 1080 (0) + L1P1 LINE/L1 2425 3483 (2663) 1**

**__________________________________________________________________________________**

**11. L1HS_16_18**

**Ortholog annotation C_INTER_RMD Length 1149 nscore 1.74 NPOSITIONS 1 20 ;**

**Repeat length (main genome) 2675**

**Blast2 Results -**

**OFC OLC RFC RLC**

**21 1149 1544 2674**

**Ortholog Repeat Masker annotation**

**SW perc perc perc query position in query matching repeat position in repeat**

**score div. del. ins. sequence begin end (left) repeat class/family begin end (left) ID**

**8038 1.0 0.1 0.0 L1HS_16_18 21 1149 (0) + L1HS LINE/L1 5024 6154 (1) 1**

**__________________________________________________________________________________**

**12. L1HS_16_2**

**Ortholog annotation C_INTRA_RMD Length 1833 nscore 1.09 NPOSITIONS 519 538 ;**

**Repeat length (main genome) 2882**

**Blast2 Results -**

**OFC OLC RFC RLC**

**1 518 1 518**

**539 1833 1588 2881**

**Ortholog Repeat Masker annotation**

**SW perc perc perc query position in query matching repeat position in repeat**

**score div. del. ins. sequence begin end (left) repeat class/family begin end (left) ID**

**4601 0.4 0.0 0.0 L1HS_16_2 1 518 (1315) + L1HS LINE/L1 3274 3791 (2355) 1**

**8085 1.3 0.0 0.1 L1HS_16_2 539 1833 (0) + L1HS LINE/L1 4861 6154 (1) 1**

**__________________________________________________________________________________**

**13. L1HS_18_12c**

**Ortholog annotation C_INTER_RMD_M_DISRUPTED Length 509 nscore 3.93 NPOSITIONS 490 509 ;**

**Repeat length (main genome) 509**

**Blast2 Results -**

**OFC OLC RFC RLC**

**1 489 1 489**

**Ortholog Repeat Masker annotation**

**SW perc perc perc query position in query matching repeat position in repeat**

**score div. del. ins. sequence begin end (left) repeat class/family begin end (left) ID**

**4523 0.2 0.0 0.0 L1HS_18_12c 1 489 (20) + L1HS LINE/L1 5647 6135 (20) 1**

**__________________________________________________________________________________**

**14. L1HS_18_2c**

**Ortholog annotation C_INTER_RMD_M_DISRUPTED Length 370 nscore 0.00 NPOSITIONS NA**

**Repeat length (main genome) 378**

**Blast2 Results -**

**OFC OLC RFC RLC**

**1 370 9 378**

**Ortholog Repeat Masker annotation**

**SW perc perc perc query position in query matching repeat position in repeat**

**score div. del. ins. sequence begin end (left) repeat class/family begin end (left) ID**

**3321 1.4 0.0 0.0 L1HS_18_2c 1 370 (0) + L1HS LINE/L1 5785 6154 (1) 1**

**__________________________________________________________________________________**

**15. L1HS_18_3c**

**Ortholog annotation C_INTER_RMD_M_DISRUPTED Length 147 nscore 0.00 NPOSITIONS NA**

**Repeat length (main genome) 163**

**Blast2 Results -**

**OFC OLC RFC RLC**

**1 147 1 147**

**Ortholog Repeat Masker annotation**

**SW perc perc perc query position in query matching repeat position in repeat**

**score div. del. ins. sequence begin end (left) repeat class/family begin end (left) ID**

**1347 0.7 0.0 0.0 L1HS_18_3c 1 147 (0) + L1P1 LINE/L1 5623 5769 (386) 1**

**__________________________________________________________________________________**

**16. L1HS_18_5c**

**Ortholog annotation C_DISRUPTED_M_INTER_RMD Length 3582 nscore 2.79 NPOSITIONS 1182 1231 ; 2784 2833 ;**

**Repeat length (main genome) 2193**

**Blast2 Results -**

**OFC OLC RFC RLC**

**1 1180 1 1180**

**2834 3029 1430 1627**

**3008 3577 1624 2193**

**3532 3582 2133 2183**

**Ortholog Repeat Masker annotation**

**SW perc perc perc query position in query matching repeat position in repeat**

**score div. del. ins. sequence begin end (left) repeat class/family begin end (left) ID**

**10488 0.2 0.0 0.0 L1HS_18_5c 1 1180 (2402) + L1P1 LINE/L1 4026 5205 (941) 1**

**1921 25.3 4.8 9.5 L1HS_18_5c 2011 2778 (804) + L1MEf LINE/L1 294 1025 (5175) 2**

**5991 1.3 0.3 0.0 L1HS_18_5c 2834 3582 (0) + L1HS LINE/L1 5455 6155 (0) 3**

**__________________________________________________________________________________**

**17. L1HS_18_6**

**Ortholog annotation C_INTRA_RMD Length 3080 nscore 2.05 NPOSITIONS 2244 2306 ;**

**Repeat length (main genome) 3223**

**Blast2 Results -**

**OFC OLC RFC RLC**

**1 2243 1 2243**

**2307 3080 2448 3222**

**Ortholog Repeat Masker annotation**

**SW perc perc perc query position in query matching repeat position in repeat**

**score div. del. ins. sequence begin end (left) repeat class/family begin end (left) ID**

**19464 0.5 0.0 0.0 L1HS_18_6 1 2243 (837) + L1HS LINE/L1 125 2367 (3779) 1**

**6725 0.4 0.1 0.0 L1HS_18_6 2307 3080 (0) + L1HS LINE/L1 2572 3346 (2800) 1**

**__________________________________________________________________________________**

**18. L1HS_1_14**

**Ortholog annotation C_INTER_RMD Length 600 nscore 3.33 NPOSITIONS 581 600 ;**

**Repeat length (main genome) 3830**

**Blast2 Results -**

**OFC OLC RFC RLC**

**1 580 1 579**

**Ortholog Repeat Masker annotation**

**SW perc perc perc query position in query matching repeat position in repeat**

**score div. del. ins. sequence begin end (left) repeat class/family begin end (left) ID**

**5143 0.7 0.0 0.2 L1HS_1_14 1 580 (20) + L1P1 LINE/L1 2326 2904 (3242) 1**

**__________________________________________________________________________________**

**19. L1HS_1_63**

**Ortholog annotation C_INTER_RMD_M_DISRUPTED Length 3152 nscore 0.00 NPOSITIONS NA**

**Repeat length (main genome) 4183**

**Blast2 Results -**

**OFC OLC RFC RLC**

**1 3152 1 3153**

**Ortholog Repeat Masker annotation**

**SW perc perc perc query position in query matching repeat position in repeat**

**score div. del. ins. sequence begin end (left) repeat class/family begin end (left) ID**

**24163 4.4 0.1 0.2 L1HS_1_63 1 3152 (0) + L1P2 LINE/L1 1995 5127 (1019) 1**

**__________________________________________________________________________________**

**20. L1HS_20_16c**

**Ortholog annotation C_INTER_RMD_M_DISRUPTED Length 1006 nscore 1.99 NPOSITIONS 6 25 ;**

**Repeat length (main genome) 1162**

**Blast2 Results -**

**OFC OLC RFC RLC**

**26 1006 182 1162**

**Ortholog Repeat Masker annotation**

**SW perc perc perc query position in query matching repeat position in repeat**

**score div. del. ins. sequence begin end (left) repeat class/family begin end (left) ID**

**8342 2.2 0.9 0.0 L1HS_20_16c 26 1006 (0) + L1HS LINE/L1 663 1652 (4380) 1**

**__________________________________________________________________________________**

**21. L1HS_2_41**

**Ortholog annotation C_DISRUPTED_M_INTER_RMD Length 3177 nscore 0.63 NPOSITIONS 448 467 ;**

**Repeat length (main genome) 2257**

**Blast2 Results -**

**OFC OLC RFC RLC**

**1 447 1 447**

**923 3177 1 2256**

**Ortholog Repeat Masker annotation**

**SW perc perc perc query position in query matching repeat position in repeat**

**score div. del. ins. sequence begin end (left) repeat class/family begin end (left) ID**

**2422 2.7 2.5 0.0 L1HS_2_41 1 447 (2730) + L1HS LINE/L1 1968 2425 (3721) 1**

**3674 1.5 2.4 0.0 L1HS_2_41 468 924 (2253) C L1P1 LINE/L1 (1435) 4711 4244 2 ***

**17908 1.7 0.5 0.0 L1HS_2_41 923 3177 (0) + L1P1 LINE/L1 1968 4234 (1912) 2**

**__________________________________________________________________________________**

**22. L1HS_2_49c**

**Ortholog annotation C_INTER_RMD_M_DISRUPTED Length 4314 nscore 0.00 NPOSITIONS NA**

**Repeat length (main genome) 4326**

**Blast2 Results -**

**OFC OLC RFC RLC**

**1 4314 13 4326**

**Ortholog Repeat Masker annotation**

**SW perc perc perc query position in query matching repeat position in repeat**

**score div. del. ins. sequence begin end (left) repeat class/family begin end (left) ID**

**27589 0.3 0.0 0.0 L1HS_2_49c 1 4314 (0) + L1HS LINE/L1 1843 6155 (0) 1**

**__________________________________________________________________________________**

**23. L1HS_2_50c**

**Ortholog annotation C_INTER_RMD_M_DISRUPTED Length 1705 nscore 0.00 NPOSITIONS NA**

**Repeat length (main genome) 1713**

**Blast2 Results -**

**OFC OLC RFC RLC**

**1 1705 1 1707**

**Ortholog Repeat Masker annotation**

**SW perc perc perc query position in query matching repeat position in repeat**

**score div. del. ins. sequence begin end (left) repeat class/family begin end (left) ID**

**15481 0.3 0.1 0.0 L1HS_2_50c 1 1705 (0) + L1HS LINE/L1 2 1708 (4324) 1**

**__________________________________________________________________________________**

**24. L1HS_2_51c**

**Ortholog annotation M_INTRA_RMD Length 4901 nscore 0.00 NPOSITIONS NA**

**Repeat length (main genome) 3482**

**Blast2 Results -**

**OFC OLC RFC RLC**

**1425 4901 1 3482**

**Ortholog Repeat Masker annotation**

**SW perc perc perc query position in query matching repeat position in repeat**

**score div. del. ins. sequence begin end (left) repeat class/family begin end (left) ID**

**27524 0.5 0.1 0.0 L1HS_2_51c 1 4901 (0) + L1HS LINE/L1 1250 6155 (0) 1**

**__________________________________________________________________________________**

**25. L1HS_2_52c**

**Ortholog annotation INDEL_CAN Length 2548 nscore 0.00 NPOSITIONS NA**

**Repeat length (main genome) 1125**

**Blast2 Results -**

**OFC OLC RFC RLC**

**1 1113 1 1114**

**Ortholog Repeat Masker annotation**

**SW perc perc perc query position in query matching repeat position in repeat**

**score div. del. ins. sequence begin end (left) repeat class/family begin end (left) ID**

**19318 0.6 0.0 0.0 L1HS_2_52c 1 2548 (0) + L1HS LINE/L1 138 2685 (3461) 1**

**__________________________________________________________________________________**

**26. L1HS_3_14c**

**Ortholog annotation C_INTER_RMD_M_DISRUPTED Length 464 nscore 0.00 NPOSITIONS NA**

**Repeat length (main genome) 2806**

**Blast2 Results -**

**OFC OLC RFC RLC**

**1 464 2343 2806**

**Ortholog Repeat Masker annotation**

**SW perc perc perc query position in query matching repeat position in repeat**

**score div. del. ins. sequence begin end (left) repeat class/family begin end (left) ID**

**23 3.3 0.0 0.0 L1HS_3_14c 67 96 (368) + AT_rich Low_complexity 1 30 (0) 1**

**881 16.3 6.2 0.6 L1HS_3_14c 246 424 (40) + L1M2 LINE/L1 3370 3558 (2585) 2**

**__________________________________________________________________________________**

**27. L1HS_3_30**

**Ortholog annotation C_INTRA_RMD Length 2033 nscore 8.41 NPOSITIONS 880 1050 ;**

**Repeat length (main genome) 2141**

**Blast2 Results -**

**OFC OLC RFC RLC**

**1 874 1 874**

**1051 2033 1158 2140**

**Ortholog Repeat Masker annotation**

**SW perc perc perc query position in query matching repeat position in repeat**

**score div. del. ins. sequence begin end (left) repeat class/family begin end (left) ID**

**7752 0.6 0.1 0.0 L1HS_3_30 1 879 (1154) + L1HS LINE/L1 4015 4894 (1252) 1**

**7983 0.7 0.0 0.0 L1HS_3_30 1051 2033 (0) + L1HS LINE/L1 5172 6154 (1) 1**

**__________________________________________________________________________________**

**28. L1HS_3_9c**

**Ortholog annotation C_INTRA_RMD Length 2467 nscore 10.34 NPOSITIONS 1218 1472 ;**

**Repeat length (main genome) 3117**

**Blast2 Results -**

**OFC OLC RFC RLC**

**1 1217 1 1217**

**1473 2467 2123 3117**

**Ortholog Repeat Masker annotation**

**SW perc perc perc query position in query matching repeat position in repeat**

**score div. del. ins. sequence begin end (left) repeat class/family begin end (left) ID**

**10360 3.0 0.0 0.0 L1HS_3_9c 1 1217 (1250) + L1HS LINE/L1 127 1343 (4812) 1**

**8504 2.0 0.1 0.0 L1HS_3_9c 1473 2467 (0) + L1HS LINE/L1 2249 3244 (2902) 1**

**__________________________________________________________________________________**

**29. L1HS_4_129**

**Ortholog annotation C_INTRA_RMD Length 1055 nscore 2.09 NPOSITIONS 366 387 ;**

**Repeat length (main genome) 1179**

**Blast2 Results -**

**OFC OLC RFC RLC**

**1 365 1 365**

**388 1055 511 1178**

**Ortholog Repeat Masker annotation**

**SW perc perc perc query position in query matching repeat position in repeat**

**score div. del. ins. sequence begin end (left) repeat class/family begin end (left) ID**

**2879 6.3 0.3 0.0 L1HS_4_129 1 365 (690) + L1HS LINE/L1 3 368 (5787) 1**

**5672 3.7 0.0 0.0 L1HS_4_129 388 1055 (0) + L1HS LINE/L1 635 1302 (4853) 1**

**__________________________________________________________________________________**

**30. L1HS_4_29**

**Ortholog annotation C_INTER_RMD_M_DISRUPTED Length 1270 nscore 2.76 NPOSITIONS 1236 1270 ;**

**Repeat length (main genome) 1229**

**Blast2 Results -**

**OFC OLC RFC RLC**

**1 1234 1 1229**

**Ortholog Repeat Masker annotation**

**SW perc perc perc query position in query matching repeat position in repeat**

**score div. del. ins. sequence begin end (left) repeat class/family begin end (left) ID**

**10909 0.4 0.0 0.1 L1HS_4_29 1 1234 (36) + L1P1 LINE/L1 2780 4012 (2134) 1**

**__________________________________________________________________________________**

**31. L1HS_4_8**

**Ortholog annotation C_INTER_RMD_M_DISRUPTED Length 1842 nscore 0.00 NPOSITIONS NA**

**Repeat length (main genome) 1854**

**Blast2 Results -**

**OFC OLC RFC RLC**

**1 1842 1 1841**

**Ortholog Repeat Masker annotation**

**SW perc perc perc query position in query matching repeat position in repeat**

**score div. del. ins. sequence begin end (left) repeat class/family begin end (left) ID**

**16728 0.6 0.1 0.1 L1HS_4_8 1 1842 (0) + L1HS LINE/L1 3 1844 (4188) 1**

**__________________________________________________________________________________**

**32. L1HS_4_9**

**Ortholog annotation C_INTER_RMD_M_DISRUPTED Length 4249 nscore 3.55 NPOSITIONS 1292 1442 ;**

**Repeat length (main genome) 4187**

**Blast2 Results -**

**OFC OLC RFC RLC**

**1 1258 14 1276**

**1443 4249 1380 4186**

**Ortholog Repeat Masker annotation**

**SW perc perc perc query position in query matching repeat position in repeat**

**score div. del. ins. sequence begin end (left) repeat class/family begin end (left) ID**

**9767 0.6 0.7 0.0 L1HS_4_9 1 1291 (2958) + L1HS LINE/L1 1981 3282 (2864) 1**

**17979 0.5 0.0 0.0 L1HS_4_9 1443 4249 (0) + L1HS LINE/L1 3347 6153 (2) 1**

**__________________________________________________________________________________**

**33. L1HS_5_80**

**Ortholog annotation C_INTER_RMD_M_DISRUPTED Length 245 nscore 8.16 NPOSITIONS 1 20 ;**

**Repeat length (main genome) 445**

**Blast2 Results -**

**OFC OLC RFC RLC**

**21 241 220 440**

**Ortholog Repeat Masker annotation**

**SW perc perc perc query position in query matching repeat position in repeat**

**score div. del. ins. sequence begin end (left) repeat class/family begin end (left) ID**

**2014 0.9 0.0 0.0 L1HS_5_80 21 245 (0) + L1HS LINE/L1 5929 6153 (2) 1**

**__________________________________________________________________________________**

**34. L1HS_5_93c**

**Ortholog annotation M_INTRA_RMD Length 6403 nscore 0.00 NPOSITIONS NA**

**Repeat length (main genome) 935**

**Blast2 Results -**

**OFC OLC RFC RLC**

**1 924 1 935**

**Ortholog Repeat Masker annotation**

**SW perc perc perc query position in query matching repeat position in repeat**

**score div. del. ins. sequence begin end (left) repeat class/family begin end (left) ID**

**25714 3.4 0.3 0.1 L1HS_5_93c 1 6020 (383) + L1PA2 LINE/L1 127 6155 (0) 1**

**733 17.9 1.7 1.1 L1HS_5_93c 6021 6195 (208) C L1M4 LINE/L1 (3511) 3133 2958 2**

**244 18.6 5.1 0.0 L1HS_5_93c 6201 6259 (144) C L1M4 LINE/L1 (5108) 1434 1373 2**

**__________________________________________________________________________________**

**35. L1HS_5_97c**

**Ortholog annotation C_INTER_RMD_M_DISRUPTED Length 282 nscore 7.09 NPOSITIONS 1 20 ;**

**Repeat length (main genome) 291**

**Blast2 Results -**

**OFC OLC RFC RLC**

**21 282 30 291**

**Ortholog Repeat Masker annotation**

**SW perc perc perc query position in query matching repeat position in repeat**

**score div. del. ins. sequence begin end (left) repeat class/family begin end (left) ID**

**2271 1.9 0.0 0.4 L1HS_5_97c 21 282 (0) + L1HS LINE/L1 5895 6155 (0) 1**

**__________________________________________________________________________________**

**36. L1HS_7_24c**

**Ortholog annotation C_INTRA_RMD Length 1982 nscore 5.05 NPOSITIONS 914 1013 ;**

**Repeat length (main genome) 3434**

**Blast2 Results -**

**OFC OLC RFC RLC**

**1 907 1 908**

**1014 1981 2466 3433**

**Ortholog Repeat Masker annotation**

**SW perc perc perc query position in query matching repeat position in repeat**

**score div. del. ins. sequence begin end (left) repeat class/family begin end (left) ID**

**7863 1.1 0.1 0.1 L1HS_7_24c 1 913 (1069) + L1HS LINE/L1 2720 3632 (2514) 1**

**8062 1.2 0.0 0.0 L1HS_7_24c 1014 1981 (1) + L1HS LINE/L1 5185 6152 (3) 1**

**__________________________________________________________________________________**

**37. L1HS_7_54**

**Ortholog annotation C_INTRA_RMD Length 3266 nscore 2.88 NPOSITIONS 831 924 ;**

**Repeat length (main genome) 3371**

**Blast2 Results -**

**OFC OLC RFC RLC**

**1 830 1 830**

**925 3266 1029 3370**

**Ortholog Repeat Masker annotation**

**SW perc perc perc query position in query matching repeat position in repeat**

**score div. del. ins. sequence begin end (left) repeat class/family begin end (left) ID**

**7150 0.4 0.0 0.0 L1HS_7_54 1 830 (2436) + L1HS LINE/L1 2785 3614 (2532) 1**

**13961 0.7 0.0 0.0 L1HS_7_54 925 3266 (0) + L1HS LINE/L1 3813 6154 (1) 1**

**__________________________________________________________________________________**

**38. L1HS_8_5**

**Ortholog annotation M_INTRA_RMD Length 4796 nscore 0.00 NPOSITIONS NA**

**Repeat length (main genome) 3947**

**Blast2 Results -**

**OFC OLC RFC RLC**

**1 3943 1 3943**

**Ortholog Repeat Masker annotation**

**SW perc perc perc query position in query matching repeat position in repeat**

**score div. del. ins. sequence begin end (left) repeat class/family begin end (left) ID**

**24168 2.8 0.2 0.0 L1HS_8_5 1 4796 (0) + L1HS LINE/L1 125 4929 (1217) 1**

**__________________________________________________________________________________**

**39. L1HS_8_7c**

**Ortholog annotation C_INTER_RMD_M_DISRUPTED Length 870 nscore 13.68 NPOSITIONS 752 870 ;**

**Repeat length (main genome) 877**

**Blast2 Results -**

**OFC OLC RFC RLC**

**1 751 1 752**

**Ortholog Repeat Masker annotation**

**SW perc perc perc query position in query matching repeat position in repeat**

**score div. del. ins. sequence begin end (left) repeat class/family begin end (left) ID**

**6552 1.5 0.4 0.0 L1HS_8_7c 1 751 (119) + L1HS LINE/L1 5273 6030 (125) 1**

**__________________________________________________________________________________**

**40. L1HS_9_19**

**Ortholog annotation INDEL_PTS Length 99 nscore 0.00 NPOSITIONS NA**

**Repeat length (main genome) 40**

**Blast2 Results -**

**OFC OLC RFC RLC**

**no hits found**

**Ortholog Repeat Masker annotation**

**SW perc perc perc query position in query matching repeat position in repeat**

**score div. del. ins. sequence begin end (left) repeat class/family begin end (left) ID**

**198 0.0 0.0 0.0 L1HS_9_19 1 22 (77) + (TTTTTA)n Simple_repeat 2 23 (0) 1**

**__________________________________________________________________________________**

**41. L1HS_9_31**

**Ortholog annotation INDEL_CAN Length 1455 nscore 9.90 NPOSITIONS 1 144 ;**

**Repeat length (main genome) 1480**

**Blast2 Results -**

**OFC OLC RFC RLC**

**145 1455 156 1466**

**1430 1455 1452 1477**

**1430 1455 1453 1478**

**1430 1455 1454 1479**

**1430 1455 1455 1480**

**Ortholog Repeat Masker annotation**

**SW perc perc perc query position in query matching repeat position in repeat**

**score div. del. ins. sequence begin end (left) repeat class/family begin end (left) ID**

**3617 8.6 1.0 0.4 L1HS_9_31 145 962 (493) + L1P3 LINE/L1 4900 5725 (447) 1 ***

**3667 9.0 1.2 0.0 L1HS_9_31 950 1447 (8) + L1PA8 LINE/L1 5669 6172 (0) 2**

**__________________________________________________________________________________**

**42. L1HS_9_40**

**Ortholog annotation C_INTRA_RMD Length 1714 nscore 1.17 NPOSITIONS 1138 1157 ;**

**Repeat length (main genome) 1905**

**Blast2 Results -**

**OFC OLC RFC RLC**

**1 1137 1 1137**

**1164 1714 1354 1904**

**Ortholog Repeat Masker annotation**

**SW perc perc perc query position in query matching repeat position in repeat**

**score div. del. ins. sequence begin end (left) repeat class/family begin end (left) ID**

**10116 0.4 0.0 0.0 L1HS_9_40 1 1137 (577) + L1HS LINE/L1 4251 5387 (759) 1**

**5054 0.5 0.2 0.0 L1HS_9_40 1158 1714 (0) + L1HS LINE/L1 5597 6154 (1) 1**

**__________________________________________________________________________________**

**43. L1HS_9_49c**

**Ortholog annotation C_DISRUPTED_M_INTER_RMD Length 3080 nscore 0.65 NPOSITIONS 2227 2246 ;**

**Repeat length (main genome) 2550**

**Blast2 Results -**

**OFC OLC RFC RLC**

**1 2226 1 2226**

**2247 3080 1717 2550**

**Ortholog Repeat Masker annotation**

**SW perc perc perc query position in query matching repeat position in repeat**

**score div. del. ins. sequence begin end (left) repeat class/family begin end (left) ID**

**15010 0.6 0.0 0.0 L1HS_9_49c 1 2226 (854) + L1HS LINE/L1 3606 5831 (324) 1**

**6953 1.6 0.0 0.0 L1HS_9_49c 2247 3080 (0) + L1HS LINE/L1 5322 6155 (0) 2**

**__________________________________________________________________________________**

**44. L1HS_X_108c**

**Ortholog annotation M_INTRA_RMD Length 1105 nscore 9.59 NPOSITIONS 1 106 ;**

**Repeat length (main genome) 961**

**Blast2 Results -**

**OFC OLC RFC RLC**

**145 1105 1 961**

**Ortholog Repeat Masker annotation**

**SW perc perc perc query position in query matching repeat position in repeat**

**score div. del. ins. sequence begin end (left) repeat class/family begin end (left) ID**

**372 0.0 0.0 0.0 L1HS_X_108c 107 146 (959) C L1P1 LINE/L1 (1213) 4933 4894 1 ***

**8682 0.5 0.1 0.0 L1HS_X_108c 145 1105 (0) + L1P1 LINE/L1 3959 4920 (1226) 1**

**__________________________________________________________________________________**

**45. L1HS_X_117c**

**Ortholog annotation C_INTER_RMD Length 1346 nscore 7.43 NPOSITIONS 1 100 ;**

**Repeat length (main genome) 1832**

**Blast2 Results -**

**OFC OLC RFC RLC**

**115 150 639 674**

**186 319 700 833**

**321 1346 805 1832**

**Ortholog Repeat Masker annotation**

**SW perc perc perc query position in query matching repeat position in repeat**

**score div. del. ins. sequence begin end (left) repeat class/family begin end (left) ID**

**8096 3.0 0.2 2.2 L1HS_X_117c 104 1346 (0) + L1HS LINE/L1 4950 6155 (0) 1**

**__________________________________________________________________________________**

**46. L1HS_X_13**

**Ortholog annotation M_INTRA_RMD Length 2798 nscore 12.29 NPOSITIONS 1 344 ;**

**Repeat length (main genome) 2461**

**Blast2 Results -**

**OFC OLC RFC RLC**

**345 2798 7 2460**

**Ortholog Repeat Masker annotation**

**SW perc perc perc query position in query matching repeat position in repeat**

**score div. del. ins. sequence begin end (left) repeat class/family begin end (left) ID**

**17022 3.5 0.0 0.1 L1HS_X_13 345 2797 (1) + L1HS LINE/L1 130 2581 (3565) 1**

**__________________________________________________________________________________**

**47. L1HS_X_32**

**Ortholog annotation M_INTRA_RMD Length 3002 nscore 18.32 NPOSITIONS 4 553 ;**

**Repeat length (main genome) 2480**

**Blast2 Results -**

**OFC OLC RFC RLC**

**554 3002 31 2479**

**Ortholog Repeat Masker annotation**

**SW perc perc perc query position in query matching repeat position in repeat**

**score div. del. ins. sequence begin end (left) repeat class/family begin end (left) ID**

**16138 2.1 0.0 0.0 L1HS_X_32 555 3002 (0) + L1HS LINE/L1 1590 4036 (2110) 1**

**__________________________________________________________________________________**

**48. L1HS_X_45c**

**Ortholog annotation INDEL_CAN Length 3304 nscore 0.61 NPOSITIONS 1 20 ;**

**Repeat length (main genome) 3375**

**Blast2 Results -**

**OFC OLC RFC RLC**

**Ortholog Repeat Masker annotation**

**SW perc perc perc query position in query matching repeat position in repeat**

**score div. del. ins. sequence begin end (left) repeat class/family begin end (left) ID**

**239 22.6 0.0 0.0 L1HS_X_45c 22 74 (3230) + CT-rich Low_complexity 2 54 (0) 1**

**8450 17.4 2.1 0.1 L1HS_X_45c 77 1510 (1794) C L1M1 LINE/L1 (5800) 1765 303 2**

**8406 17.5 2.4 0.7 L1HS_X_45c 1566 3297 (7) C L1M1 LINE/L1 (2370) 5192 3430 3**

**__________________________________________________________________________________**

**49. L1HS_X_69c**

**Ortholog annotation C_INTER_RMD_M_DISRUPTED Length 603 nscore 0.00 NPOSITIONS NA**

**Repeat length (main genome) 2396**

**Blast2 Results -**

**OFC OLC RFC RLC**

**1 575 1 575**

**Ortholog Repeat Masker annotation**

**SW perc perc perc query position in query matching repeat position in repeat**

**score div. del. ins. sequence begin end (left) repeat class/family begin end (left) ID**

**4650 6.1 0.0 0.0 L1HS_X_69c 1 575 (28) + L1P1 LINE/L1 3760 4334 (1812) 1**

**__________________________________________________________________________________**

**50. L1HS_X_99**

**Ortholog annotation M_INTRA_RMD Length 498 nscore 4.02 NPOSITIONS 316 335 ;**

**Repeat length (main genome) 163**

**Blast2 Results -**

**OFC OLC RFC RLC**

**337 498 1 162**

**Ortholog Repeat Masker annotation**

**SW perc perc perc query position in query matching repeat position in repeat**

**score div. del. ins. sequence begin end (left) repeat class/family begin end (left) ID**

**1003 23.8 5.1 1.1 L1HS_X_99 40 315 (183) + L1MEf LINE/L1 873 1159 (5041) 1**

**1485 0.0 0.0 0.0 L1HS_X_99 337 498 (0) + L1HS LINE/L1 5993 6154 (1) 2**

**__________________________________________________________________________________**

**51. L1HS_Y_19c**

**Ortholog annotation C_INTER_RMD_M_DISRUPTED Length 2812 nscore 9.74 NPOSITIONS 1 274 ;**

**Repeat length (main genome) 2745**

**Blast2 Results -**

**OFC OLC RFC RLC**

**275 2812 208 2745**

**Ortholog Repeat Masker annotation**

**SW perc perc perc query position in query matching repeat position in repeat**

**score div. del. ins. sequence begin end (left) repeat class/family begin end (left) ID**

**13974 5.3 0.0 0.0 L1HS_Y_19c 275 2812 (0) + L1HS LINE/L1 1346 3884 (2262) 1**

**__________________________________________________________________________________**

**52. L1HS_Y_26**

**Ortholog annotation M_INTRA_RMD Length 4126 nscore 7.76 NPOSITIONS 1 320 ;**

**Repeat length (main genome) 3805**

**Blast2 Results -**

**OFC OLC RFC RLC**

**323 4126 1 3804**

**Ortholog Repeat Masker annotation**

**SW perc perc perc query position in query matching repeat position in repeat**

**score div. del. ins. sequence begin end (left) repeat class/family begin end (left) ID**

**16715 3.9 0.2 0.0 L1HS_Y_26 323 4126 (0) + L1HS LINE/L1 129 3937 (2209) 1**

**__________________________________________________________________________________**

**53. L1HS_Y_30c**

**Ortholog annotation INDEL_CAN Length 2195 nscore 0.00 NPOSITIONS NA**

**Repeat length (main genome) 545**

**Blast2 Results -**

**OFC OLC RFC RLC**

**Ortholog Repeat Masker annotation**

**SW perc perc perc query position in query matching repeat position in repeat**

**score div. del. ins. sequence begin end (left) repeat class/family begin end (left) ID**

**17959 3.2 0.2 0.5 L1HS_Y_30c 1 2195 (0) + L1P1 LINE/L1 3133 5320 (826) 1**

**__________________________________________________________________________________**

**54. L1HS_Y_9**

**Ortholog annotation INDEL_CAN Length 5606 nscore 0.36 NPOSITIONS 5581 5600 ;**

**Repeat length (main genome) 123**

**Blast2 Results -**

**OFC OLC RFC RLC**

**no hits found**

**Ortholog Repeat Masker annotation**

**SW perc perc perc query position in query matching repeat position in repeat**

**score div. del. ins. sequence begin end (left) repeat class/family begin end (left) ID**

**1052 16.3 0.2 0.1 L1HS_Y_9 5 1194 (4412) C ALR/Alpha Satellite/centr (0) 1191 1 1**

**1116 16.0 0.1 0.1 L1HS_Y_9 1207 5575 (31) C ALR/Alpha Satellite/centr (12) 4369 1 1**

**__________________________________________________________________________________**
