# Supplementary material for: RISCI - Repeat Induced Sequence Changes Identifier: a comprehensive, comparative genomics-based, in silico subtractive hybridization pipeline to identify repeat induced sequence changes in closely related genomes
Source: BMC Bioinformatics. 2010 Dec 26;11:609. doi: 10.1186/1471-2105-11-609 (PMC3024322; doi:10.1186/1471-2105-11-609)
Supplement: Additional file 8 — RepeatMasker annotations of recombined loci for AluYa5. RepeatMasker annotation of the repeat locus and in flanks in the reference genome and of the identified ortholog and its flanks in the comparative genomes to identify putative regions of homology where recombination takes place [file 1471-2105-11-609-S8.DOC]

**Additional file 8 - Recombination mediated deletions – AluYa5 elements, Human Vs Chimpanzee comparison**

Repeat masker annotation of the locus in the main genome and its flanks is listed first, followed by the Repeat masker annotation of the identified ortholog in the comparative genome and its flanks.

R1,R2 – PUTATIVE REGIONS OF HOMOLOGY WHICH RECOMBINE TO GIVE R12 (M_INTER_RMD or C_INTER_RMD).

In case of disruptions, R1 and R2 represent disrupted halves of the parent repeat, R represents undisrupted repeat and D represents the disruptive sequence. (M_DISRUPTED OR C_DISRUPTED).

In case of intra element recombination (M_INTRA_RMD or C_INTRA_RMD), R12 represent the 2 resulting copies arising out of intra element recombination and R represents the intact repeat.

**AluYa5_1_19 18485981-18486290 C_INTER_RMD**

**18482854 18482982 + MIR SINE/MIR 28 161**

**18482973 18483109 C L2 LINE/L2 3418 3276**

**18483151 18483201 + L2 LINE/L2 3326 3376**

**18483394 18483469 C L2 LINE/L2 3419 3341**

**18483590 18483802 C MIRb SINE/MIR 226 22**

**18484265 18484289 + (TTTTC)n Simple_repeat 2 26**

**18484927 18484973 + MIR SINE/MIR 215 262**

**18484992 18485208 C MIRb SINE/MIR 240 23**

**18485307 18485385 + MIRb SINE/MIR 89 169**

**18485397 18485451 C L2 LINE/L2 3360 3306**

**18485811 18485872 + L2 LINE/L2 3319 3395**

**18485888 18485980 C L2 LINE/L2 3419 3315**

**18485981 18486290 + AluYa5 SINE/Alu 1 310 (AluYa5_1_19) (R1)**

**18486291 18486312 + AT_rich Low_complexity 1 22**

**18486313 18486498 + AluY SINE/Alu 118 304 (R2)**

**18487132 18487238 C MIRb SINE/MIR 196 81**

**18488547 18488637 + MIRb SINE/MIR 69 165**

**18488694 18488920 C MER20 DNA/MER1_type 217 16**

**18490430 18490859 + L2 LINE/L2 2713 3197**

**18491305 18491475 C MIRb SINE/MIR 187 19**

**18491729 18491834 + L2 LINE/L2 3021 3127**

**18492229 18492341 + MIRm SINE/MIR 158 275**

**Ortholog in Chimp 18321320-18321387 Plus Nscore 0.00**

**18318147 18318274 + MIR SINE/MIR 28 161**

**18318265 18318400 C L2 LINE/L2 3418 3276**

**18318442 18318492 + L2 LINE/L2 3326 3376**

**18318685 18318760 C L2 LINE/L2 3419 3341**

**18318881 18319093 C MIRb SINE/MIR 226 22**

**18320182 18320264 + THER1_MD SINE/MIR 179 272**

**18320280 18320498 C MIRb SINE/MIR 240 23**

**18320597 18320675 + MIRb SINE/MIR 89 169**

**18320687 18320741 C L2 LINE/L2 3360 3306**

**18321101 18321162 + L2 LINE/L2 3319 3395**

**18321178 18321270 C L2 LINE/L2 3419 3315**

**18321271 18321577 + AluY SINE/Alu 1 309 (R12)**

**18322208 18322312 C MIRb SINE/MIR 196 81**

**18323621 18323711 + MIRb SINE/MIR 69 165**

**18323768 18323994 C MER20 DNA/MER1_type 217 16**

**18325500 18325929 + L2 LINE/L2 2713 3197**

**18326365 18326535 C MIRb SINE/MIR 187 19**

**18326789 18326894 + L2 LINE/L2 3021 3127**

**18327288 18327400 + MIRm SINE/MIR 158 275**

**__________________________________________________________________________________**

**AluYa5_1_196c 152069565-152069874 C_INTER_RMD**

**152067759 152067841 C L1PA15 LINE/L1 6165 6083**

**152068024 152068324 + AluJb SINE/Alu 1 303**

**152068556 152068841 + AluSg1 SINE/Alu 1 285**

**152068918 152069221 + AluSx SINE/Alu 1 310**

**152069259 152069545 C AluSc SINE/Alu 286 1 (R2)**

**152069565 152069874 C AluYa5 SINE/Alu 310 1 ( AluYa5_1_196c ) (R1)**

**152069875 152070151 C AluJo SINE/Alu 301 21**

**152070247 152070616 + L1PA8 LINE/L1 5806 6172**

**152070618 152070746 C L1MB8 LINE/L1 6172 6043**

**152070965 152071020 + Tigger1 DNA/MER2_type 1 56**

**152071033 152071327 + AluSx SINE/Alu 1 294**

**152071328 152071748 + Tigger1 DNA/MER2_type 563 964**

**152071749 152071964 C L1PA17 LINE/L1 6159 5945**

**Ortholog in Chimp 132964782-132964849 Minus Nscore 0.00**

**132963060 132963142 C L1PA15 LINE/L1 6165 6083**

**132963325 132963624 + AluJb SINE/Alu 1 302**

**132963856 132964141 + AluSg1 SINE/Alu 1 285**

**132964218 132964521 + AluSx SINE/Alu 1 310**

**132964536 132964831 C AluSc SINE/Alu 309 1 (R12)**

**132964834 132965110 C AluJo SINE/Alu 301 21**

**132965206 132965575 + L1PA8 LINE/L1 5806 6172**

**132965580 132965708 C L1MB8 LINE/L1 6172 6043**

**132965927 132965967 + Tigger1 DNA/MER2_type 1 41**

**132965995 132966291 + AluSq SINE/Alu 1 297**

**132966292 132966712 + Tigger1 DNA/MER2_type 563 964**

**132966713 132966928 C L1PA17 LINE/L1 6159 5945**

**___________________________________________________________________________________________**

**AluYa5_1_216c 160525879-160526188 C_INTER_RMD**

**160522043 160522096 + CT-rich Low_complexity 4 56**

**160523104 160523149 + L1MA5A LINE/L1 6244 6292**

**160523301 160523517 + L3b LINE/CR1 4159 4406**

**160524875 160525088 C L3 LINE/CR1 4488 4264**

**160525603 160525726 + (TTCC)n Simple_repeat 2 124**

**160525732 160525877 C AluSq SINE/Alu 281 135 (R2)**

**160525879 160526188 C AluYa5 SINE/Alu 309 1 ( AluYa5_1_216c ) (R1)**

**160527215 160527351 + MIR3 SINE/MIR 42 196**

**160528498 160528538 C L2 LINE/L2 3414 3373**

**160528803 160529028 C MIR SINE/MIR 268 18**

**160529245 160529420 C MIR3 SINE/MIR 201 17**

**160530167 160530286 + MIRm SINE/MIR 25 157**

**160530428 160530561 C LTR16B LTR/ERVL 464 284**

**160530562 160530659 + (GGAA)n Simple_repeat 2 99**

**160530660 160530929 C LTR16B LTR/ERVL 283 6**

**160532419 160532712 + AluSx SINE/Alu 1 295**

**Ortholog in Chimp 141502992-141503071 Minus Nscore 0.00**

**141497032 141497085 + CT-rich Low_complexity 4 56**

**141498093 141498125 + L1MA5A LINE/L1 6244 6276**

**141500526 141500579 + L3b LINE/CR1 4298 4351**

**141501993 141502206 C L3 LINE/CR1 4488 4264**

**141502721 141502836 + (TTCC)n Simple_repeat 2 116**

**141502842 141503120 C AluSq SINE/Alu 281 1 (R12)**

**141505355 141505395 C L2 LINE/L2 3414 3373**

**141505660 141505885 C MIR SINE/MIR 268 18**

**141506102 141506291 C MIR3 SINE/MIR 201 2**

**141507025 141507144 + MIRm SINE/MIR 25 157**

**141507286 141507451 C LTR16B LTR/ERVL 464 276**

**141508195 141508330 C LTR16B LTR/ERVL 146 5**

**141509301 141509357 + T-rich Low_complexity 2 58**

**141509604 141509724 + MIR3 SINE/MIR 2 126**

**141509830 141510123 + AluSx SINE/Alu 1 295**

**___________________________________________________________________________________________**

**AluYa5_1_277 200037605-200037906 C_INTER_RMD**

**200035186 200035212 + (TTTTA)n Simple_repeat 3 29**

**200035329 200035627 + AluSq SINE/Alu 1 299**

**200035723 200036051 C AluJo SINE/Alu 328 1**

**200036138 200036359 + MER58A DNA/MER1_type 2 224**

**200036569 200036878 C AluJb SINE/Alu 305 1**

**200037231 200037366 C AluJo SINE/Alu 134 6**

**200037383 200037517 + L1MD2 LINE/L1 6221 6358**

**200037605 200037906 + AluYa5 SINE/Alu 1 300 ( AluYa5_1_277 ) (R1)**

**200037907 200038079 + AluSq SINE/Alu 134 306 (R2)**

**200038090 200038224 + FLAM_C SINE/Alu 1 133**

**200038250 200038558 + AluSg SINE/Alu 1 309**

**200039167 200039209 + L2 LINE/L2 3375 3417**

**200040331 200040353 + (A)n Simple_repeat 1 23**

**200041105 200041148 C L2 LINE/L2 3163 3120**

**200041392 200041686 + AluJb SINE/Alu 1 297**

**Ortholog in Chimp 181824832-181824911 Plus Nscore 0.00**

**181822357 181822383 + (TTTTA)n Simple_repeat 3 29**

**181822500 181822796 + AluSq SINE/Alu 1 297**

**181822893 181823213 C AluJo SINE/Alu 320 1**

**181823300 181823521 + MER58A DNA/MER1_type 2 224**

**181823731 181824045 C AluJb SINE/Alu 310 1**

**181824409 181824544 C AluJo SINE/Alu 134 6**

**181824561 181824695 + L1MD2 LINE/L1 6221 6358**

**181824783 181825094 + AluSq SINE/Alu 1 312 (R12)**

**181825105 181825239 + FLAM_C SINE/Alu 1 133**

**181825273 181825582 + AluSg SINE/Alu 1 310**

**181826194 181826236 + L2 LINE/L2 3375 3417**

**181827361 181827385 + (A)n Simple_repeat 1 25**

**181828137 181828180 C L2 LINE/L2 3163 3120**

**181828424 181828708 + AluJb SINE/Alu 1 287**

**__________________________________________________________________________________**

**AluYa5_1_286c 205471998-205472303 C_INTER_RMD**

**205464630 205465506 C L1ME3B LINE/L1 6218 5268**

**205466181 205466421 C L1ME3A LINE/L1 6165 5924**

**205466422 205466467 + MADE1 DNA/Mariner 1 46**

**205466468 205466778 C L1ME3A LINE/L1 5923 5611**

**205466779 205467091 C AluJo SINE/Alu 305 1**

**205467092 205467177 C L1ME3A LINE/L1 5608 5523**

**205467182 205467540 C L1ME3B LINE/L1 5178 4819**

**205467911 205468046 + MIR SINE/MIR 18 153**

**205469827 205469991 + MLT1B LTR/MaLR 230 388**

**205469994 205470926 + L1ME3A LINE/L1 5179 6168**

**205470952 205471254 C AluSx SINE/Alu 303 1**

**205471695 205471815 + MSTA LTR/MaLR 252 393**

**205471819 205471997 C AluY SINE/Alu 311 133 (R2)**

**205471998 205472303 C AluYa5 SINE/Alu 306 1 ( AluYa5_1_286c ) (R1)**

**205472305 205472348 + MSTA LTR/MaLR 385 428**

**205473179 205473263 + L2 LINE/L2 1013 1095**

**205473375 205473507 + L2 LINE/L2 2633 2771**

**205473591 205474092 C L1MEb LINE/L1 1382 848**

**205474093 205474375 C AluY SINE/Alu 303 21**

**205474376 205475236 C L1MEb LINE/L1 851 2**

**205475276 205475646 + L2 LINE/L2 3047 3413**

**205475664 205475909 + L1MD2 LINE/L1 4821 5075**

**205475902 205476050 + L1MD2 LINE/L1 4998 5158**

**205476061 205476354 + AluSg1 SINE/Alu 5 295**

**205476357 205476819 + L1MD2 LINE/L1 5775 6210**

**205476994 205479841 + L1MA2 LINE/L1 3471 6299**

**Ortholog in Chimp 187642920-187642997 Minus Nscore 0.00**

**187635408 187636284 C L1ME3B LINE/L1 6218 5268**

**187637106 187637346 C L1ME3A LINE/L1 6165 5924**

**187637347 187637392 + MADE1 DNA/Mariner 1 46**

**187637393 187637703 C L1ME3A LINE/L1 5923 5611**

**187637704 187638005 C AluJo SINE/Alu 294 1**

**187638006 187638091 C L1ME3A LINE/L1 5608 5523**

**187638096 187638454 C L1ME3B LINE/L1 5178 4819**

**187638826 187638961 + MIR SINE/MIR 18 153**

**187640739 187640903 + MLT1B LTR/MaLR 230 388**

**187640906 187641844 + L1ME3A LINE/L1 5179 6168**

**187641870 187642173 C AluSx SINE/Alu 304 1**

**187642614 187642733 + MSTA LTR/MaLR 252 385**

**187642734 187643046 C AluY SINE/Alu 310 1 (R12)**

**187643047 187643091 + MSTA LTR/MaLR 386 428**

**187643927 187644011 + L2 LINE/L2 1013 1095**

**187644123 187644255 + L2 LINE/L2 2633 2771**

**187644339 187644843 C L1MEb LINE/L1 1382 852**

**187644844 187645118 C AluY SINE/Alu 295 21**

**187645119 187645980 C L1MEb LINE/L1 851 2**

**187646020 187646390 + L2 LINE/L2 3047 3413**

**187646408 187646652 + L1MD2 LINE/L1 4821 5075**

**187646645 187646793 + L1MD2 LINE/L1 4998 5158**

**187646800 187647097 + AluSg1 SINE/Alu 1 295**

**187647100 187647562 + L1MD2 LINE/L1 5775 6210**

**187647737 187650778 + L1MA2 LINE/L1 3471 6303**

**__________________________________________________________________________________**

**AluYa5_2_43 32171594-32171906 C_INTER_RMD**

**32168715 32168920 + MER58A DNA/MER1_type 1 216**

**32168962 32169508 C L1MC5 LINE/L1 7779 7221**

**32169750 32169817 + MIR SINE/MIR 79 146**

**32169819 32169870 + (TTA)n Simple_repeat 2 53**

**32169872 32170122 C AluSq SINE/Alu 283 28**

**32170124 32170427 + AluY SINE/Alu 1 302**

**32170446 32170485 C AluSq SINE/Alu 40 1**

**32171299 32171592 + AluSx SINE/Alu 1 295**

**32171594 32171906 + AluYa5 SINE/Alu 2 304 ( AluYa5_2_43 ) (R1)**

**32171907 32172072 + AluSg/x SINE/Alu 135 300 (R2)**

**32172081 32172312 + L2 LINE/L2 3184 3418**

**32172307 32172366 + MIRb SINE/MIR 191 254**

**32172386 32172409 + AT_rich Low_complexity 1 24**

**32172795 32172937 C AluSg/x SINE/Alu 297 155**

**32172948 32173260 C L2 LINE/L2 3185 2847**

**32173261 32173556 + AluSx SINE/Alu 1 297**

**32173557 32173796 C L2 LINE/L2 2846 2598**

**32174023 32174334 + AluJo SINE/Alu 1 305**

**32174395 32174629 C L2 LINE/L2 2979 2756**

**32174728 32175023 + AluSx SINE/Alu 3 298**

**32176255 32176570 C AluY SINE/Alu 311 1**

**32176758 32177027 + AluJo SINE/Alu 2 275**

**32177195 32177235 + AT_rich Low_complexity 1 41**

**32177301 32177350 + T-rich Low_complexity 1 50**

**32178106 32178432 C L1MA5A LINE/L1 6296 5938**

**32178436 32178863 C L1MA5A LINE/L1 5806 5374**

**Ortholog in Chimp 32824258-32824343 Plus Nscore 0.00**

**32821325 32821530 + MER58A DNA/MER1_type 1 216**

**32821547 32822156 C L1MC5 LINE/L1 7801 7189**

**32822361 32822428 + MIR SINE/MIR 79 146**

**32822430 32822472 + (TTA)n Simple_repeat 2 44**

**32822474 32822724 C AluSx SINE/Alu 282 28**

**32822726 32823039 + AluY SINE/Alu 1 312**

**32823047 32823103 C AluSx SINE/Alu 40 1**

**32823242 32823267 + (CATATA)n Simple_repeat 2 27**

**32823900 32824207 + AluSx SINE/Alu 1 309**

**32824209 32824510 + AluSx SINE/Alu 2 296 (R12)**

**32824519 32824751 + L2 LINE/L2 3184 3419**

**32824759 32824816 + L2 LINE/L2 3316 3373**

**32824823 32824845 + AT_rich Low_complexity 1 23**

**32825231 32825373 C AluSg/x SINE/Alu 297 155**

**32825432 32825696 C L2 LINE/L2 3103 2847**

**32825697 32825996 + AluSx SINE/Alu 1 301**

**32825997 32826236 C L2 LINE/L2 2846 2598**

**32826463 32826773 + AluJo SINE/Alu 1 305**

**32826834 32827068 C L2 LINE/L2 2979 2756**

**32827167 32827476 + AluSx SINE/Alu 3 312**

**32828684 32828712 + (T)n Simple_repeat 1 29**

**32829495 32829761 C AluY SINE/Alu 262 1**

**32829949 32830219 + AluJb SINE/Alu 2 275**

**32830387 32830427 + AT_rich Low_complexity 1 41**

**32830493 32830542 + T-rich Low_complexity 1 50**

**__________________________________________________________________________________**

**AluYa5_2_54c 38468000-38468289 C_INTER_RMD_M_DISRUPTED**

**38465912 38466080 C Arthur1 DNA/Tip100 3869 3695**

**38466081 38466393 C AluSx SINE/Alu 309 1**

**38466394 38466559 C Arthur1 DNA/Tip100 3694 3526**

**38466561 38466946 + AluJb SINE/Alu 3 385**

**38466947 38467126 + (GGAA)n Simple_repeat 2 180**

**38467127 38467149 + (GAAA)n Simple_repeat 1 22**

**38467759 38467802 C MIR SINE/MIR 262 218**

**38467826 38467996 C AluSp SINE/Alu 307 137 (R2)**

**38468000 38468289 C AluYa5 SINE/Alu 301 12 ( AluYa5_2_54c ) (R1)**

**38468291 38468474 C MIR SINE/MIR 187 2**

**38468925 38469002 + LTR73 LTR/ERV1 1 81**

**38469003 38469306 + AluSg SINE/Alu 1 303**

**38469307 38469740 + LTR73 LTR/ERV1 82 552**

**38470895 38471003 + L3 LINE/CR1 3525 3633**

**38472021 38472329 + AluJo SINE/Alu 10 308**

**Ortholog in Chimp 39238734-39238802 Minus Nscore 0.00**

**39236201 39236370 C Arthur1 DNA/Tip100 3869 3695**

**39236371 39236685 C AluSx SINE/Alu 311 1**

**39236686 39236851 C Arthur1 DNA/Tip100 3694 3526**

**39236853 39237105 + AluJb SINE/Alu 3 252**

**39237389 39237671 + AluSx SINE/Alu 1 282**

**39237728 39237887 + GA-rich Low_complexity 3 161**

**39238481 39238547 C MIR SINE/MIR 262 188**

**39238548 39238843 C AluSq SINE/Alu 307 12 (R12)**

**39238844 39239028 C MIR SINE/MIR 187 2**

**39239223 39239245 + (T)n Simple_repeat 1 23**

**39239487 39239564 + LTR73 LTR/ERV1 1 81**

**39239565 39239870 + AluSg SINE/Alu 1 305**

**39239871 39240304 + LTR73 LTR/ERV1 82 552**

**39242585 39242897 + AluJo SINE/Alu 10 312**

**39243193 39243430 C AluY SINE/Alu 303 66**

**39243687 39244019 C LTR73 LTR/ERV1 550 203**

**__________________________________________________________________________________**

**AluYa5_2_71c 47571721-47572030 C_INTER_RMD**

**47564671 47564835 + L1ME3B LINE/L1 5040 5226**

**47564848 47564982 + AluJo SINE/Alu 122 274**

**47565018 47565817 + L1MB5 LINE/L1 5270 6144**

**47565746 47565851 + L1MA10 LINE/L1 6224 6334**

**47565854 47566483 + L1ME3B LINE/L1 5340 6165**

**47568953 47569205 + AluY SINE/Alu 1 264**

**47569331 47569447 C MIR SINE/MIR 152 33**

**47570088 47570219 + MIR3 SINE/MIR 4 157**

**47570939 47571248 C AluSx SINE/Alu 312 1**

**47571273 47571539 + HAL1 LINE/L1 1830 2119**

**47571541 47571713 C AluSg/x SINE/Alu 301 129 (R2)**

**47571721 47572030 C AluYa5 SINE/Alu 310 1 ( AluYa5_2_71c ) (R1)**

**47572098 47572385 + AluJo SINE/Alu 6 295**

**47572429 47572604 + HAL1 LINE/L1 2281 2471**

**47572894 47573088 + L1MB8 LINE/L1 5977 6178**

**47573167 47573346 + LTR3 LTR/ERVK 251 432**

**47573353 47573441 C MIRb SINE/MIR 181 82**

**47573451 47573783 C MLT1J LTR/MaLR 443 96**

**47574193 47574259 C MIR SINE/MIR 90 19**

**47575679 47575793 C MIR3 SINE/MIR 192 95**

**47576325 47576848 C MER21C LTR/ERV1 873 339**

**47576849 47577044 + AluJo SINE/Alu 1 195**

**47577060 47577291 C MER21B LTR/ERV1 224 14**

**47578008 47578182 C AluSg/x SINE/Alu 308 134**

**47578183 47578481 C AluSx SINE/Alu 299 1**

**47578614 47579055 + MER31B LTR/ERV1 2 466**

**Ortholog in Chimp 48630049-48630120 Minus Nscore 0.00**

**48623006 48623170 + L1ME3B LINE/L1 5040 5226**

**48623183 48623317 + AluJo SINE/Alu 122 274**

**48623353 48624185 + L1MB8 LINE/L1 5270 6178**

**48624190 48624821 + L1ME3B LINE/L1 5340 6165**

**48627297 48627551 + AluY SINE/Alu 1 264**

**48627677 48627801 C MIR SINE/MIR 152 27**

**48628432 48628581 + MIR3 SINE/MIR 4 175**

**48629282 48629572 C AluSx SINE/Alu 293 1**

**48629597 48629861 + HAL1 LINE/L1 1830 2122**

**48629862 48630167 C AluSg SINE/Alu 304 1 (R12)**

**48630168 48630234 + HAL1 LINE/L1 2123 2184**

**48630235 48630522 + AluJo SINE/Alu 6 295**

**48630523 48630545 + HAL1 LINE/L1 2185 2205**

**48630566 48630741 + HAL1 LINE/L1 2281 2471**

**48631030 48631224 + L1MB8 LINE/L1 5977 6178**

**48631262 48631301 + LTR3 LTR/ERVK 1 45**

**48631301 48631480 + LTR3 LTR/ERVK 249 432**

**48631487 48631575 C MIR SINE/MIR 177 82**

**48631585 48631920 C MLT1J LTR/MaLR 443 96**

**48632330 48632396 C MIR SINE/MIR 90 19**

**48633811 48633908 C MIR3 SINE/MIR 192 77**

**48634478 48634979 C MER21C LTR/ERV1 848 339**

**48634980 48635175 + AluJo SINE/Alu 1 195**

**48635191 48635414 C MER21B LTR/ERV1 224 22**

**48636142 48636325 C AluSg/x SINE/Alu 312 129**

**48636328 48636641 C AluSx SINE/Alu 312 1**

**48636774 48637215 + MER31B LTR/ERV1 2 466**

**__________________________________________________________________________________**

**AluYa5_2_87 54002228-54002521 C_INTER_RMD**

**53995242 53995529 + AluSg SINE/Alu 1 289**

**53995796 53996091 + AluJo SINE/Alu 1 293**

**53996096 53996200 + FLAM_C SINE/Alu 6 117**

**53996430 53996724 + AluSq SINE/Alu 1 291**

**53996727 53996902 + AluSg/x SINE/Alu 122 302**

**53997643 53997701 + AT_rich Low_complexity 1 59**

**53997714 53997777 + AT_rich Low_complexity 1 64**

**53998982 53999233 C AluJb SINE/Alu 309 49**

**54000471 54000498 + AT_rich Low_complexity 1 28**

**54001984 54002185 C AluSg SINE/Alu 202 1**

**54002228 54002521 + AluYa5 SINE/Alu 1 294 ( AluYa5_2_87 ) (R1)**

**54002532 54002832 + AluY SINE/Alu 1 311 (R2)**

**54004282 54004584 + AluSx SINE/Alu 1 312**

**54005266 54005410 C L1PA5 LINE/L1 6153 6009**

**54005416 54006045 + MER82 DNA/MER2_type 1 653**

**54007033 54007304 + AluSx SINE/Alu 1 265**

**54007333 54007643 + AluSx SINE/Alu 1 312**

**54008306 54008610 + AluSx SINE/Alu 1 302**

**54008621 54008664 + AT_rich Low_complexity 1 44**

**54009102 54009406 + MER44A DNA/MER2_type 1 339**

**Ortholog in Chimp 55205248-55205312 Plus Nscore 0.00**

**55198278 55198565 + AluSg SINE/Alu 1 289**

**55198834 55199126 + AluJo SINE/Alu 1 290**

**55199131 55199235 + FLAM_C SINE/Alu 6 117**

**55199464 55199758 + AluSq SINE/Alu 1 291**

**55199761 55199937 + AluSg/x SINE/Alu 122 303**

**55200678 55200736 + AT_rich Low_complexity 1 59**

**55200749 55200798 + AT_rich Low_complexity 1 50**

**55202019 55202270 C AluJb SINE/Alu 309 49**

**55203509 55203536 + AT_rich Low_complexity 1 28**

**55205020 55205221 C AluSg SINE/Alu 202 1**

**55205266 55205561 + AluY SINE/Alu 1 306 (R12)**

**55207002 55207294 + AluSx SINE/Alu 1 304**

**55207974 55208118 C L1PA5 LINE/L1 6153 6009**

**55208124 55208753 + MER82 DNA/MER2_type 1 653**

**55209739 55210010 + AluSx SINE/Alu 1 265**

**55210039 55210345 + AluSx SINE/Alu 1 308**

**55211005 55211309 + AluSx SINE/Alu 1 302**

**55211320 55211363 + AT_rich Low_complexity 1 44**

**55211803 55212107 + MER44A DNA/MER2_type 1 339**

**55212225 55212524 + AluSx SINE/Alu 1 302**

**__________________________________________________________________________________**

**AluYa5_2_109 68245719-68246028 C_INTER_RMD**

**68239527 68239828 C MER7D DNA/MER2_type 303 1**

**68239891 68239915 + (TG)n Simple_repeat 2 26**

**68239931 68239980 C AluSq SINE/Alu 261 211**

**68239984 68240141 C AluSq SINE/Alu 161 1**

**68240601 68240890 C AluY SINE/Alu 303 15**

**68241350 68241476 C MIR3 SINE/MIR 181 45**

**68241570 68241779 + MIRb SINE/MIR 11 260**

**68241849 68241969 C L2 LINE/L2 2377 2253**

**68242489 68242790 + AluSx SINE/Alu 1 298**

**68243422 68243738 C Tigger5a DNA/MER2_type 335 1**

**68243886 68244168 + AluSx SINE/Alu 1 284**

**68244169 68244224 + (CATA)n Simple_repeat 2 57**

**68244844 68245140 + AluJb SINE/Alu 1 298**

**68245154 68245446 + AluJo SINE/Alu 3 296**

**68245470 68245556 C Tigger5 DNA/MER2_type 2406 2320**

**68245604 68245681 C Tigger5 DNA/MER2_type 77 1**

**68245719 68246028 + AluYa5 SINE/Alu 1 310 ( AluYa5_2_109 ) (R1)**

**68246029 68246201 + AluSx SINE/Alu 130 302 (R2)**

**68246203 68246330 + FLAM_C SINE/Alu 1 130**

**68246463 68246825 C L1ME1 LINE/L1 6161 5778**

**68246827 68248613 C Tigger2 DNA/MER2_type 2716 126**

**68248614 68249330 C MER49 LTR/ERV1 923 204**

**68249349 68249552 C Tigger2 DNA/MER2_type 146 690**

**68249553 68249864 C AluY SINE/Alu 311 1**

**68249865 68250550 C Tigger2 DNA/MER2_type 689 1**

**68250551 68250635 C L1ME1 LINE/L1 5783 5698**

**68250934 68251051 + L2 LINE/L2 1821 1942**

**68251054 68251101 C HAL1 LINE/L1 2503 2457**

**68251102 68251405 + AluY SINE/Alu 1 303**

**68251406 68252001 C HAL1 LINE/L1 2456 1816**

**68252037 68252211 C HAL1 LINE/L1 245 45**

**68252248 68252428 C L2 LINE/L2 3082 2865**

**68252431 68253142 C L2 LINE/L2 1159 347**

**Ortholog in Chimp 69650666-69650737 Plus Nscore 0.00**

**69644422 69644725 C MER7D DNA/MER2_type 305 1**

**69644788 69644818 + (TG)n Simple_repeat 2 32**

**69644824 69644873 C AluSq SINE/Alu 261 211**

**69644877 69645037 C AluSq SINE/Alu 161 1**

**69645496 69645785 C AluY SINE/Alu 303 15**

**69646245 69646371 C MIR3 SINE/MIR 181 45**

**69646465 69646677 + MIRb SINE/MIR 11 266**

**69646744 69646864 C L2 LINE/L2 2377 2253**

**69647384 69647685 + AluSx SINE/Alu 1 298**

**69648317 69648633 C Tigger5a DNA/MER2_type 335 1**

**69648781 69649063 + AluSx SINE/Alu 1 284**

**69649064 69649119 + (CATA)n Simple_repeat 2 57**

**69649739 69650035 + AluJb SINE/Alu 1 298**

**69650049 69650341 + AluJo SINE/Alu 3 296**

**69650365 69650451 C Tigger5 DNA/MER2_type 2406 2320**

**69650499 69650579 C Tigger5 DNA/MER2_type 77 1**

**69650617 69650915 + AluSx SINE/Alu 1 305 (R12)**

**69650917 69651046 + FLAM_C SINE/Alu 1 132**

**69651184 69651546 C L1ME1 LINE/L1 6161 5778**

**69651548 69652576 C Tigger2 DNA/MER2_type 2716 901**

**69652580 69652712 + AluY SINE/Alu 169 301**

**69652727 69653335 C HAL1 LINE/L1 2421 1787**

**69653343 69653545 C HAL1 LINE/L1 245 19**

**69653554 69653734 C L2 LINE/L2 3082 2865**

**69653737 69654453 C L2 LINE/L2 1159 347**

**69654619 69654778 + MIRb SINE/MIR 50 259**

**69654846 69654935 C L1MB7 LINE/L1 6168 6069**

**69655095 69655260 C AluJo SINE/Alu 163 1**

**69655520 69655841 C AluSg SINE/Alu 305 1**

**69656227 69656250 + (T)n Simple_repeat 1 24**

**69656309 69656621 + MER2 DNA/MER2_type 1 345**

**69657217 69657498 C AluSx SINE/Alu 281 1**

**69657699 69657998 C Tigger5a DNA/MER2_type 366 13**

**__________________________________________________________________________________**

**AluYa5_2_201c 132857466-132857740 C_INTER_RMD_M_DISRUPTED**

**132850553 132851853 C LOR1a-int LTR/ERV1 5024 3797**

**132851854 132852154 C AluJo SINE/Alu 298 1**

**132852155 132852306 C LOR1a-int LTR/ERV1 3963 3818**

**132852312 132853719 C LOR1a-int LTR/ERV1 1867 458**

**132853800 132854037 C LOR1a-int LTR/ERV1 450 208**

**132854052 132854424 C LOR1a-int LTR/ERV1 371 1**

**132854425 132854594 C LOR1a LTR/ERV1 497 329**

**132854602 132854838 + MER44B DNA/MER2_type 292 547**

**132856064 132856500 C MER31B LTR/ERV1 463 1**

**132857299 132857465 C AluSc SINE/Alu 298 132 (R2)**

**132857466 132857740 C AluYa5 SINE/Alu 310 36 ( AluYa5_2_201c ) (R1)**

**132857959 132858107 C MIRb SINE/MIR 173 23**

**132859584 132859779 + L1MB7 LINE/L1 5933 6137**

**132859837 132859936 C MER81 DNA/AcHobo 109 1**

**132860020 132860196 C MIRb SINE/MIR 181 17**

**132861520 132861874 C L1PB1 LINE/L1 6150 5794**

**132861875 132861897 + (TG)n Simple_repeat 2 24**

**132861898 132863277 C L1PB1 LINE/L1 5793 4413**

**132863278 132863303 + (TTTTA)n Simple_repeat 2 27**

**132863304 132863765 C L1PB1 LINE/L1 4412 3948**

**132864390 132864684 + AluSx SINE/Alu 1 299**

**132864725 132864874 + MER102b DNA/MER1_type 196 335**

**Ortholog in Chimp 136473065-136473107 Minus Nscore 0.00**

**136466139 136467437 C LOR1a-int LTR/ERV1 5024 3765**

**136467438 136467734 C AluJo SINE/Alu 298 1**

**136467735 136467886 C LOR1a-int LTR/ERV1 3963 3818**

**136467887 136469334 C LOR1a-int LTR/ERV1 1873 423**

**136469380 136469617 C LOR1a-int LTR/ERV1 450 208**

**136469636 136470008 C LOR1a-int LTR/ERV1 371 1**

**136470009 136470181 C LOR1a LTR/ERV1 497 326**

**136470171 136470422 + MER44B DNA/MER2_type 277 547**

**136471650 136472083 C MER31B LTR/ERV1 463 1**

**136472901 136473156 C AluSc SINE/Alu 290 36 (R12)**

**136473375 136473523 C MIRb SINE/MIR 173 23**

**136475007 136475195 + L1MB7 LINE/L1 5940 6137**

**136475249 136475353 C MER81 DNA/AcHobo 113 1**

**136475422 136475613 C MIRb SINE/MIR 192 17**

**136476871 136476897 + (TTA)n Simple_repeat 1 27**

**136476947 136477304 C L1PB1 LINE/L1 6150 5791**

**136477583 136478942 C L1PB1 LINE/L1 5772 4413**

**136478943 136478968 + (TTTTA)n Simple_repeat 2 27**

**136478969 136479430 C L1PB1 LINE/L1 4412 3948**

**136480055 136480362 + AluSx SINE/Alu 1 312**

**__________________________________________________________________________________**

**AluYa5_2_250 165204674-165204976 C_INTER_RMD_M_DISRUPTED**

**165201375 165201396 + (GGAA)n Simple_repeat 3 24**

**165201484 165201789 + AluSx SINE/Alu 1 306**

**165201857 165202164 C AluY SINE/Alu 305 1**

**165202184 165202288 + L2 LINE/L2 3259 3377**

**165202380 165202536 C MIRb SINE/MIR 167 3**

**165202963 165203408 C L2 LINE/L2 2636 2152**

**165203944 165203999 + AT_rich Low_complexity 1 56**

**165204322 165204336 C AluSx SINE/Alu 300 286**

**165204337 165204359 + (TTA)n Simple_repeat 2 24**

**165204360 165204640 C AluSx SINE/Alu 285 7**

**165204674 165204976 + AluYa5 SINE/Alu 1 302 ( AluYa5_2_250 )**

**165204977 165205297 C L1PA16 LINE/L1 6137 5816**

**165205555 165205699 + Charlie7 DNA/MER1_type 44 186**

**165205875 165206315 + Charlie7 DNA/MER1_type 1493 1923**

**165206316 165206441 + FLAM_A SINE/Alu 1 126**

**165206442 165206931 + Charlie7 DNA/MER1_type 1924 2468**

**165206948 165207379 C MER57B LTR/ERV1 434 1**

**165207557 165207782 C ORSL DNA/Tip100 273 1**

**165208130 165208620 + L2 LINE/L2 2850 3417**

**165209399 165209587 C L2 LINE/L2 3359 3186**

**165210286 165210649 C MLT1I LTR/MaLR 409 63**

**165210689 165210807 C MIRb SINE/MIR 242 109**

**165210993 165211097 + L2 LINE/L2 3304 3418**

**165211317 165211762 C MLT1C LTR/MaLR 466 1**

**165211775 165211974 + Charlie8 DNA/MER1_type 81 284**

**Ortholog in Chimp 169378116-169378225 Plus Nscore 7.05**

**N positions 169378117-169378126; 169378247-169378247;**

**169370825 169371689 C L1PA4 LINE/L1 6155 5284**

**169371690 169371932 C L1M LINE/L1 3446 3143**

**169371933 169372227 C AluSx SINE/Alu 294 1**

**169372228 169372781 C L1M LINE/L1 3142 2541**

**169373212 169373432 + L4 LINE/RTE 1738 1939**

**169374570 169374883 + AluJb SINE/Alu 1 312**

**169374884 169374945 + (GGAA)n Simple_repeat 3 64**

**169375033 169375338 + AluSx SINE/Alu 1 306**

**169375406 169375708 C AluY SINE/Alu 301 1**

**169375728 169375832 + L2 LINE/L2 3259 3377**

**169375924 169376080 C MIRb SINE/MIR 167 3**

**169376113 169376183 C L2 LINE/L2 3168 3094**

**169376507 169376952 C L2 LINE/L2 2636 2152**

**169377867 169377881 C Alu SINE/Alu 300 286**

**169377882 169377925 + (TTA)n Simple_repeat 2 45**

**169377926 169378116 C AluSg/x SINE/Alu 285 97**

**169378127 169378273 + AluY SINE/Alu 156 302 (Ns at the start of ortholog, most likely OCCUPIED)**

**169378274 169378594 C L1PA16 LINE/L1 6137 5816**

**169378860 169379001 + Charlie7 DNA/MER1_type 47 186**

**169379177 169379617 + Charlie7 DNA/MER1_type 1493 1930**

**169379618 169379743 + FLAM_A SINE/Alu 1 126**

**169379744 169380199 + Charlie7 DNA/MER1_type 1931 2419**

**169380250 169380681 C MER57B LTR/ERV1 434 1**

**169380859 169381084 C ORSL DNA/Tip100 273 1**

**169381432 169381922 + L2 LINE/L2 2850 3417**

**169382706 169382896 C L2 LINE/L2 3359 3186**

**169383597 169383846 C MLT1I LTR/MaLR 409 170**

**169383850 169383929 C MLT1J LTR/MaLR 175 97**

**169383996 169384118 C MIRb SINE/MIR 249 109**

**169384304 169384408 + L2 LINE/L2 3304 3418**

**169384628 169385073 C MLT1C LTR/MaLR 466 1**

**169385086 169385285 + Charlie8 DNA/MER1_type 81 284**

**___________________________________________________________________________________________**

**AluYa5_2_341 219675689-219675997 C_INTER_RMD_M_DISRUPTED**

**219672165 219672382 C MER20 DNA/MER1_type 219 1**

**219672383 219672928 C L2 LINE/L2 893 302**

**219673287 219673593 + AluSx SINE/Alu 1 301**

**219673917 219674091 C L1ME4a LINE/L1 6027 5827**

**219674092 219674380 C AluSx SINE/Alu 289 1**

**219674383 219674673 C AluJo SINE/Alu 295 4**

**219674684 219674989 C AluSc SINE/Alu 298 1**

**219674990 219675125 C L1ME4a LINE/L1 5824 5685**

**219675189 219675348 C L1ME4a LINE/L1 5578 5406**

**219675400 219675688 + 7SLRNA srpRNA 1 311**

**219675689 219675997 + AluYa5 SINE/Alu 2 308 ( AluYa5_2_341 ) (R1)**

**219675998 219676115 + AluSq SINE/Alu 134 251 (R2)**

**219676208 219676234 + AT_rich Low_complexity 1 27**

**219676236 219676541 C AluSx SINE/Alu 306 1**

**219676607 219676822 C MIRb SINE/MIR 264 34**

**219676835 219676912 C MIRb SINE/MIR 105 31**

**219677018 219677052 + (TTTTG)n Simple_repeat 5 41**

**219677108 219677200 + MER91C DNA/Tip100 32 138**

**219678106 219678148 + AT_rich Low_complexity 1 43**

**219678422 219678537 + L2 LINE/L2 3295 3415**

**219678611 219678721 + L2 LINE/L2 3048 3165**

**219678849 219678979 + L2 LINE/L2 3233 3370**

**219678993 219679065 + L2 LINE/L2 3305 3378**

**219680293 219680431 C MIR3 SINE/MIR 185 36**

**Ortholog in Chimp 225036076-225036154 Plus Nscore 0.00**

**225032521 225032738 C MER20 DNA/MER1_type 219 1**

**225032739 225033284 C L2 LINE/L2 893 302**

**225033643 225033942 + AluSx SINE/Alu 1 297**

**225034265 225034439 C L1ME4a LINE/L1 6027 5827**

**225034440 225034728 C AluSx SINE/Alu 289 1**

**225034731 225035021 C AluJo SINE/Alu 295 4**

**225035032 225035327 C AluSc SINE/Alu 296 1**

**225035328 225035670 C L1ME4a LINE/L1 5827 5423**

**225035738 225036025 + 7SLRNA srpRNA 1 293**

**225036026 225036276 + AluSq SINE/Alu 1 251 (R12)**

**225036277 225036308 + FAM SINE/Alu 153 178**

**225036628 225036902 C AluSc SINE/Alu 273 1**

**225036968 225037183 C MIRb SINE/MIR 264 34**

**225037196 225037277 C MIRb SINE/MIR 105 31**

**225037378 225037413 + (TTTTG)n Simple_repeat 5 41**

**225037468 225037561 + MER91C DNA/Tip100 31 138**

**225038465 225038507 + AT_rich Low_complexity 1 43**

**225038781 225038896 + L2 LINE/L2 3295 3415**

**225038970 225039080 + L2 LINE/L2 3048 3165**

**225039208 225039338 + L2 LINE/L2 3233 3370**

**225039352 225039424 + L2 LINE/L2 3305 3378**

**225040652 225040789 C MIR3 SINE/MIR 185 36**

**__________________________________________________________________________________**

**AluYa5_2_346 223191821-223192118 C_INTER_RMD_M_DISRUPTED**

**223185259 223185361 + MIR SINE/MIR 44 151**

**223186203 223186235 + L1MC5 LINE/L1 7700 7730**

**223186236 223186529 + AluSg SINE/Alu 1 303**

**223186530 223186731 + L1MC5 LINE/L1 7731 7926**

**223187216 223187390 C FRAM SINE/Alu 166 1**

**223187450 223187525 C L4 LINE/RTE 1952 1884**

**223187526 223187818 + AluJo SINE/Alu 1 303**

**223187819 223188105 C L4 LINE/RTE 1883 1612**

**223188213 223188675 + L2 LINE/L2 2691 3203**

**223188995 223189268 + L1MC5 LINE/L1 7302 7597**

**223189269 223189530 + AluSc SINE/Alu 40 301**

**223189531 223189614 + L1MC5 LINE/L1 7598 7676**

**223189946 223190224 C AluYb8 SINE/Alu 317 39**

**223190382 223190564 + L1PA16 LINE/L1 5977 6160**

**223190565 223190870 + AluSx SINE/Alu 1 307**

**223191821 223192118 + AluYa5 SINE/Alu 3 295 ( AluYa5_2_346 ) (R1)**

**223192120 223192300 + AluSg/x SINE/Alu 134 312 (R2)**

**223192854 223192875 + AT_rich Low_complexity 1 22**

**223193523 223193583 + MIRb SINE/MIR 189 253**

**223194258 223194482 C AluSx SINE/Alu 294 4**

**223194613 223194670 + MER113 DNA/MER1_type 409 480**

**223194982 223195142 + MIRb SINE/MIR 31 223**

**223195480 223195504 + AT_rich Low_complexity 1 25**

**223195784 223195823 + MIRb SINE/MIR 215 254**

**223195790 223195835 + L2 LINE/L2 3368 3417**

**223198439 223198614 + MER5A1 DNA/MER1_type 1 160**

**223198654 223198828 C MIRb SINE/MIR 260 67**

**223198831 223198903 + L2 LINE/L2 3344 3419**

**223198928 223199231 + AluSx SINE/Alu 1 307**

**Ortholog in Chimp 228614730-228614810 Plus Nscore 0.00**

**228608415 228608517 + MIR_Mars SINE/MIR 44 158**

**228609359 228609391 + L1MC5 LINE/L1 7700 7730**

**228609392 228609687 + AluSg SINE/Alu 1 305**

**228609688 228609888 + L1MC5 LINE/L1 7731 7926**

**228610373 228610547 C FRAM SINE/Alu 166 1**

**228610607 228610682 C L4 LINE/RTE 1952 1884**

**228610683 228610975 + AluJo SINE/Alu 1 303**

**228610976 228611256 C L4 LINE/RTE 1883 1612**

**228611364 228611826 + L2 LINE/L2 2691 3203**

**228612146 228612419 + L1MC5 LINE/L1 7302 7597**

**228612420 228612682 + AluSc SINE/Alu 40 302**

**228612683 228612766 + L1MC5 LINE/L1 7598 7676**

**228613245 228613427 + L1PA16 LINE/L1 5977 6160**

**228613428 228613733 + AluSx SINE/Alu 1 307**

**228614692 228614995 + AluSx SINE/Alu 15 312 (R12)**

**228615554 228615575 + AT_rich Low_complexity 1 22**

**228616224 228616299 + MIRb SINE/MIR 189 271**

**228616959 228617249 C AluSx SINE/Alu 294 4**

**228617381 228617473 + MER113 DNA/MER1_type 409 515**

**228617756 228617916 + MIRb SINE/MIR 31 223**

**228618254 228618278 + AT_rich Low_complexity 1 25**

**228618558 228618597 + MIRb SINE/MIR 215 254**

**228618564 228618609 + L2 LINE/L2 3368 3417**

**228621211 228621386 + MER5A1 DNA/MER1_type 1 160**

**228621404 228621596 C MIRb SINE/MIR 272 67**

**228621599 228621671 + L2 LINE/L2 3344 3419**

**228621696 228621997 + AluSx SINE/Alu 1 305**

**__________________________________________________________________________________**

**AluYa5_2_354c 231910420-231910681 C_INTER_RMD**

**231903262 231903899 C L1MDa LINE/L1 952 196**

**231903900 231904113 + AluJb SINE/Alu 86 298**

**231904114 231904292 C L1MDa LINE/L1 201 21**

**231905199 231905444 C L2 LINE/L2 2347 2084**

**231906098 231906350 C L2 LINE/L2 3007 2740**

**231907160 231907562 C MLT1C LTR/MaLR 459 9**

**231908751 231909012 + AluJo SINE/Alu 1 260**

**231909020 231909079 + (TAAA)n Simple_repeat 2 63**

**231909224 231909388 C MIR SINE/MIR 249 85**

**231909630 231909772 C MER5A DNA/MER1_type 174 23**

**231910227 231910247 + (TA)n Simple_repeat 2 22**

**231910248 231910419 C AluSg/x SINE/Alu 306 134 (R2)**

**231910420 231910681 C AluYa5 SINE/Alu 305 43 ( AluYa5_2_354c ) (R1)**

**231911036 231911149 C Arthur1 DNA/Tip100 3939 3814**

**231911178 231911482 C AluSx SINE/Alu 299 1**

**231911492 231911625 C AluJb SINE/Alu 304 171**

**231911626 231911777 C Arthur1 DNA/Tip100 3801 3642**

**231911831 231911981 C Arthur1 DNA/Tip100 3650 3488**

**231912004 231912300 + AluSg SINE/Alu 1 296**

**231912387 231912546 C FRAM SINE/Alu 167 11**

**231912562 231912613 C Arthur1 DNA/Tip100 3483 3439**

**231912614 231912891 + AluSq SINE/Alu 1 285**

**231912892 231912912 C Arthur1 DNA/Tip100 3438 3437**

**231912913 231913388 C MER34 LTR/ERV1 542 3**

**231913389 231913445 C Arthur1 DNA/Tip100 3436 3377**

**231913455 231913560 C Arthur1 DNA/Tip100 391 286**

**231913561 231913872 + AluSx SINE/Alu 1 312**

**231913879 231914152 + AluJo SINE/Alu 1 287**

**231914344 231914532 C MLT1J LTR/MaLR 202 4**

**231914568 231914634 + (TAGA)n Simple_repeat 4 72**

**231915617 231915703 C Arthur1 DNA/Tip100 210 124**

**231915872 231916166 C AluSp SINE/Alu 306 3**

**231916362 231916580 C L1MD2 LINE/L1 7807 6161**

**231916749 231916870 + FLAM_A SINE/Alu 1 131**

**231916872 231917361 C L1ME3B LINE/L1 5913 5374**

**231917380 231917535 C L1ME3B LINE/L1 5360 5197**

**Ortholog in Chimp 237552782-237552809 Minus Nscore 0.00**

**237545599 237546256 C L1MDa LINE/L1 952 196**

**237546257 237546470 + AluJb SINE/Alu 86 298**

**237546471 237546649 C L1MDa LINE/L1 201 21**

**237547555 237547800 C L2 LINE/L2 2347 2084**

**237548455 237548707 C L2 LINE/L2 3007 2740**

**237549517 237549919 C MLT1C LTR/MaLR 459 9**

**237551105 237551366 + AluJo SINE/Alu 1 260**

**237551374 237551433 + (TAAA)n Simple_repeat 2 63**

**237551523 237551545 + AT_rich Low_complexity 1 23**

**237551558 237551745 C MIRb SINE/MIR 273 92**

**237551987 237552128 C MER5A DNA/MER1_type 174 23**

**237552603 237552846 C AluSg SINE/Alu 299 55 (R12)**

**237553213 237553351 C Arthur1 DNA/Tip100 3939 3796**

**237553355 237553659 C AluSx SINE/Alu 299 1**

**237553669 237553805 C AluJb SINE/Alu 307 171**

**237553806 237553957 C Arthur1 DNA/Tip100 3801 3642**

**237554011 237554161 C Arthur1 DNA/Tip100 3650 3488**

**237554185 237554482 + AluSg SINE/Alu 1 297**

**237554569 237554728 C FRAM SINE/Alu 167 11**

**237554744 237554795 C Arthur1 DNA/Tip100 3483 3439**

**237554796 237555073 + AluSq SINE/Alu 1 285**

**237555074 237555094 C Arthur1 DNA/Tip100 3438 3437**

**237555095 237555571 C MER34 LTR/ERV1 542 3**

**237555572 237555628 C Arthur1 DNA/Tip100 3436 3377**

**237555638 237555738 C Arthur1 DNA/Tip100 391 291**

**237555755 237556066 + AluSx SINE/Alu 1 312**

**237556073 237556348 + AluJo SINE/Alu 1 287**

**237556540 237556728 C MLT1J LTR/MaLR 202 4**

**237556764 237556803 + (TAGA)n Simple_repeat 4 44**

**237557786 237557872 C Arthur1 DNA/Tip100 210 124**

**237558041 237558335 C AluSp SINE/Alu 306 3**

**237558531 237558749 C L1MD2 LINE/L1 7807 6161**

**237558918 237559039 + FLAM_A SINE/Alu 1 131**

**237559041 237559530 C L1ME3B LINE/L1 5913 5374**

**237559549 237559704 C L1ME3B LINE/L1 5360 5197**

**__________________________________________________________________________________**

**AluYa5_3_32 25674275-25674573 C_INTER_RMD**

**25666324 25667607 C HSMAR2 DNA/Mariner 1302 7**

**25667728 25668078 + L1M4c LINE/L1 2030 2394**

**25668079 25668374 + AluSx SINE/Alu 1 297**

**25668375 25669896 + L1M4c LINE/L1 2395 3995**

**25669897 25670224 C AluJb SINE/Alu 293 1**

**25670225 25672536 + L1MB2 LINE/L1 3996 6406**

**25672816 25672946 + FLAM_A SINE/Alu 4 132**

**25674275 25674573 + AluYa5 SINE/Alu 1 299 ( AluYa5_3_32 ) (R1)**

**25674574 25674732 + AluY SINE/Alu 133 287 (R2)**

**25674733 25674761 + AT_rich Low_complexity 1 29**

**25675104 25675166 + AT_rich Low_complexity 1 63**

**25675952 25676170 C MIR SINE/MIR 243 3**

**25676342 25676462 + MIR SINE/MIR 17 166**

**25676975 25677136 C L2 LINE/L2 3136 2977**

**25677357 25677493 + L1MC5 LINE/L1 7676 7813**

**25677494 25677792 C AluSp SINE/Alu 302 2**

**25677793 25677891 + L1MC5 LINE/L1 7814 7925**

**25678145 25678165 + AT_rich Low_complexity 1 21**

**25678605 25678813 C MIRb SINE/MIR 262 2**

**25680665 25680685 + GC_rich Low_complexity 1 21**

**25681429 25681449 + GC_rich Low_complexity 1 21**

**Ortholog in Chimp 26276765-26276830 Plus Nscore 0.00**

**26268767 26270050 C HSMAR2 DNA/Mariner 1302 7**

**26270171 26270518 + L1M4c LINE/L1 2030 2394**

**26270519 26270814 + AluSx SINE/Alu 1 297**

**26270815 26272336 + L1M4c LINE/L1 2395 3995**

**26272337 26272664 C AluJb SINE/Alu 293 1**

**26272665 26274977 + L1MB2 LINE/L1 3996 6406**

**26275257 26275387 + FLAM_A SINE/Alu 4 132**

**26276716 26277005 + AluY SINE/Alu 1 289 (R12)**

**26277006 26277032 + AT_rich Low_complexity 1 27**

**26277281 26277312 + AT_rich Low_complexity 1 32**

**26277379 26277441 + AT_rich Low_complexity 1 63**

**26278227 26278444 C MIR SINE/MIR 243 3**

**26278616 26278780 + MIR SINE/MIR 17 213**

**26279259 26279410 C L2 LINE/L2 3130 2977**

**26279632 26279767 + L1MC5 LINE/L1 7676 7813**

**26279768 26280068 C AluSp SINE/Alu 304 2**

**26280069 26280167 + L1MC5 LINE/L1 7814 7925**

**26280421 26280441 + AT_rich Low_complexity 1 21**

**26280881 26281089 C MIRb SINE/MIR 262 2**

**26282571 26282590 + (CA)n Simple_repeat 2 21**

**__________________________________________________________________________________**

**AluYa5_3_60 48935363-48935659 C_INTER_RMD**

**48932412 48932711 C AluJo SINE/Alu 302 7**

**48932926 48933069 C AluSg/x SINE/Alu 302 156**

**48933079 48933150 C AluJb SINE/Alu 262 189**

**48933151 48933281 + AluY SINE/Alu 35 165**

**48933282 48933351 + AluYc3 SINE/Alu 212 299**

**48933386 48933690 C AluSg SINE/Alu 310 1**

**48933728 48933756 + AT_rich Low_complexity 1 29**

**48933865 48934169 C AluSx SINE/Alu 307 7**

**48934182 48934346 C AluJb SINE/Alu 307 143**

**48934557 48934679 + AluSg/x SINE/Alu 180 298**

**48934744 48934968 C AluSq SINE/Alu 301 2**

**48935151 48935171 + AT_rich Low_complexity 1 21**

**48935363 48935659 + AluYa5 SINE/Alu 1 297 ( AluYa5_3_60 ) (R1)**

**48935665 48935849 + AluSg/x SINE/Alu 113 295 (R2)**

**48935961 48936257 C AluSp SINE/Alu 298 1**

**48936258 48936279 + AT_rich Low_complexity 1 22**

**48936289 48936567 + AluJo SINE/Alu 1 283**

**48936599 48936727 C FLAM_C SINE/Alu 131 10**

**48936789 48936812 + (TTA)n Simple_repeat 2 25**

**48936813 48936946 C AluJb SINE/Alu 286 134**

**48936947 48937123 C AluSx SINE/Alu 311 137**

**48937124 48937430 C AluSg SINE/Alu 308 1**

**48937431 48937538 C AluSx SINE/Alu 136 29**

**48937539 48937849 C AluY SINE/Alu 311 1**

**Ortholog in Chimp 50075095-50075147 Plus Nscore 0.00**

**50072206 50072501 C AluJo SINE/Alu 298 7**

**50072517 50072617 C AluJb SINE/Alu 291 189**

**50072618 50072748 + AluY SINE/Alu 35 165**

**50073107 50073371 C AluSg SINE/Alu 266 1**

**50073546 50073844 C AluSx SINE/Alu 301 7**

**50073857 50074025 C AluJb SINE/Alu 311 143**

**50074236 50074358 + AluSg/x SINE/Alu 180 298**

**50074425 50074651 C AluSq SINE/Alu 303 2**

**50074834 50074854 + AT_rich Low_complexity 1 21**

**50075046 50075339 + AluSg SINE/Alu 1 292 (R12)**

**50075454 50075759 C AluSp SINE/Alu 307 1**

**50075761 50075781 + AT_rich Low_complexity 1 21**

**50075791 50076150 + AluJo SINE/Alu 1 283**

**50076182 50076310 C FLAM_C SINE/Alu 131 10**

**50076374 50076409 + (TTA)n Simple_repeat 2 37**

**50076410 50076543 C AluJb SINE/Alu 286 134**

**50076544 50076720 C AluSx SINE/Alu 311 137**

**50076721 50077027 C AluSg SINE/Alu 308 1**

**50077028 50077136 C AluSx SINE/Alu 136 28**

**50077137 50077447 C AluY SINE/Alu 311 1**

**__________________________________________________________________________________**

**AluYa5_3_61c 48980639-48980945 C_INTER_RMD**

**48974142 48974410 C L1ME4a LINE/L1 6039 5725**

**48974532 48974834 + AluSx SINE/Alu 1 304**

**48975041 48975244 C MER20 DNA/MER1_type 219 2**

**48975301 48975491 C AluSg/x SINE/Alu 305 115**

**48975978 48976357 C L1ME4a LINE/L1 5713 5287**

**48976399 48976458 C L1ME4a LINE/L1 5292 5241**

**48976459 48976743 C AluSx SINE/Alu 309 1**

**48976744 48976755 C L1ME4a LINE/L1 5240 5233**

**48976756 48977051 C AluSx SINE/Alu 297 1**

**48977052 48977215 C L1ME4a LINE/L1 5232 5082**

**48977508 48977806 + AluY SINE/Alu 1 291**

**48978116 48978410 + AluSx SINE/Alu 1 295**

**48979220 48979275 + L3 LINE/CR1 4431 4486**

**48979334 48979387 + L2 LINE/L2 3101 3155**

**48980488 48980638 C AluSp SINE/Alu 286 134 (R2)**

**48980639 48980945 C AluYa5 SINE/Alu 305 1 ( AluYa5_3_61c ) (R1)**

**48981253 48981323 + MIRb SINE/MIR 20 97**

**48981350 48981609 C AluSc SINE/Alu 269 1**

**48981914 48982173 C AluJb SINE/Alu 310 53**

**48982330 48982628 C AluSx SINE/Alu 298 1**

**48982635 48982791 C FRAM SINE/Alu 162 2**

**48983585 48983674 C MIR SINE/MIR 259 176**

**48983675 48983980 C AluSg SINE/Alu 305 1**

**48984014 48984063 C MIRb SINE/MIR 100 48**

**48984213 48984254 C MER57B LTR/ERV1 368 326**

**48984671 48984782 C AluJo SINE/Alu 236 122**

**48984783 48985091 + AluSq SINE/Alu 1 293**

**48985092 48985159 C AluJo SINE/Alu 121 58**

**48985160 48985451 C AluJb SINE/Alu 289 1**

**48985599 48985732 + AluSc SINE/Alu 1 134**

**48985733 48986036 + AluY SINE/Alu 1 305**

**Ortholog in Chimp 50120617-50120696 Minus Nscore 0.00**

**50114126 50114394 C L1ME4a LINE/L1 6039 5725**

**50114516 50114820 + AluSx SINE/Alu 1 306**

**50115027 50115230 C MER20 DNA/MER1_type 219 2**

**50115285 50115475 C AluSg/x SINE/Alu 305 115**

**50115961 50116340 C L1ME4a LINE/L1 5713 5287**

**50116382 50116441 C L1ME4a LINE/L1 5292 5241**

**50116442 50116726 C AluSx SINE/Alu 309 1**

**50116727 50116737 C L1ME4a LINE/L1 5240 5233**

**50116738 50117034 C AluSx SINE/Alu 298 1**

**50117035 50117198 C L1ME4a LINE/L1 5232 5082**

**50117491 50117790 + AluY SINE/Alu 1 290**

**50118100 50118397 + AluSx SINE/Alu 1 300**

**50119208 50119263 + L3 LINE/CR1 4431 4486**

**50119322 50119375 + L2 LINE/L2 3101 3155**

**50120433 50120745 C AluSp SINE/Alu 312 1 (R12)**

**50121051 50121121 + MIRb SINE/MIR 20 97**

**50121148 50121407 C AluSc SINE/Alu 269 1**

**50121410 50121434 + (T)n Simple_repeat 1 25**

**50121716 50121977 C AluJb SINE/Alu 312 53**

**50122140 50122437 C AluSx SINE/Alu 297 1**

**50122441 50122604 C FRAM SINE/Alu 167 2**

**50123398 50123487 C THER1_MD SINE/MIR 266 176**

**50123488 50123793 C AluSg SINE/Alu 305 1**

**50123827 50123876 C MIRb SINE/MIR 100 48**

**50124026 50124067 C MER57B LTR/ERV1 368 326**

**50124484 50124595 C AluJo SINE/Alu 236 122**

**50124596 50124903 + AluSq SINE/Alu 1 293**

**50124904 50124971 C AluJo SINE/Alu 121 58**

**50124972 50125264 C AluJb SINE/Alu 289 1**

**50125412 50125545 + AluSc SINE/Alu 1 134**

**50125546 50125851 + AluY SINE/Alu 1 307**

**___________________________________________________________________________________________**

**AluYa5_3_166c 133160501-133160833 C_INTER_RMD_M_DISRUPTED**

**133153056 133154792 + SVA Other 1 1386**

**133154793 133155117 C L1M2 LINE/L1 4111 3785**

**133155124 133155575 + L1MA4 LINE/L1 5831 6295**

**133155591 133155789 + L1MEd LINE/L1 941 1147**

**133156031 133156502 C L1PA5 LINE/L1 6154 5682**

**133156673 133156729 C L1M2 LINE/L1 4134 4078**

**133156945 133156968 + (T)n Simple_repeat 1 24**

**133157027 133157470 + LTR16D LTR/ERVL 167 655**

**133157513 133157962 C L1M5 LINE/L1 3886 3445**

**133158043 133158370 + L1ME4a LINE/L1 5605 5934**

**133159185 133159299 C MIRm SINE/MIR 275 154**

**133159667 133159853 C MIRb SINE/MIR 252 39**

**133159878 133160039 + MER5A DNA/MER1_type 1 189**

**133160054 133160135 + MIRb SINE/MIR 37 121**

**133160147 133160491 C AluSg SINE/Alu 310 1 (R2)**

**133160501 133160833 C AluYa5 SINE/Alu 333 1 ( AluYa5_3_166c ) (R1)**

**133160839 133160968 + MIRb SINE/MIR 114 224**

**133161152 133161172 + AT_rich Low_complexity 1 21**

**133161623 133161681 + GA-rich Low_complexity 3 61**

**133161874 133161918 C MIR3 SINE/MIR 201 159**

**133161919 133162530 + LTR26 LTR/ERV1 1 603**

**133162531 133162670 C MIR3 SINE/MIR 158 19**

**133162853 133162945 + MIRb SINE/MIR 88 183**

**133163753 133163821 + L2 LINE/L2 3306 3375**

**133164108 133164168 C L2 LINE/L2 3418 3357**

**133164490 133170911 + L1PA8 LINE/L1 6 6480**

**Ortholog in Chimp 136500448-136500509 Minus Nscore 0.00**

**136493193 136493563 + THE1D LTR/MaLR 4 381**

**136493564 136493635 C L1MB5 LINE/L1 4397 4325**

**136493668 136493778 C L1MB5 LINE/L1 4086 3975**

**136493984 136494202 + MIRb SINE/MIR 23 265**

**136494207 136494303 C Zaphod DNA/Tip100 4030 3934**

**136494588 136494640 C MIRm SINE/MIR 275 220**

**136494731 136494909 + L2 LINE/L2 3136 3304**

**136494911 136495071 C L1M5 LINE/L1 5814 5643**

**136495078 136495723 + MER82 DNA/MER2_type 7 652**

**136495724 136495978 C L1M5 LINE/L1 5645 5389**

**136496047 136496190 C L1M5 LINE/L1 4687 4544**

**136496219 136496459 C L1M5 LINE/L1 4471 4226**

**136496696 136496948 C L1M2 LINE/L1 4137 3911**

**136497047 136497490 + LTR16D LTR/ERVL 167 655**

**136497533 136497982 C L1M5 LINE/L1 3886 3445**

**136498063 136498332 + L1M5 LINE/L1 5605 5859**

**136499202 136499316 C MIRm SINE/MIR 275 154**

**136499684 136499870 C MIRb SINE/MIR 252 39**

**136499895 136500056 + MER5A DNA/MER1_type 1 189**

**136500071 136500151 + MIRb SINE/MIR 37 113**

**136500152 136500495 C AluSg SINE/Alu 309 1 (R12)**

**136500496 136500631 + MIRb SINE/MIR 114 224**

**136500816 136500836 + AT_rich Low_complexity 1 21**

**136501287 136501358 + GA-rich Low_complexity 3 73**

**136501551 136501595 C MIR3 SINE/MIR 201 159**

**136501596 136502212 + LTR26 LTR/ERV1 1 603**

**136502213 136502339 C MIR3 SINE/MIR 158 30**

**136502535 136502627 + MIRb SINE/MIR 88 183**

**136503795 136503856 C L2 LINE/L2 3419 3357**

**136504178 136510620 + L1PA8 LINE/L1 6 6481**

**__________________________________________________________________________________**

**AluYa5_3_174c 137211950-137212253 C_INTER_RMD_M_DISRUPTED**

**137205215 137205343 + MIR SINE/MIR 20 152**

**137205789 137205890 C MLT1I LTR/MaLR 352 244**

**137206185 137206381 + L1ME3B LINE/L1 5957 6171**

**137206574 137206898 + MER46B DNA/MER2_type 26 236**

**137207278 137207355 C MIRb SINE/MIR 262 174**

**137207950 137208163 C MER58C DNA/MER1_type 214 4**

**137210246 137211103 + L2 LINE/L2 20 1130**

**137211104 137211292 + MER5A DNA/MER1_type 1 189**

**137211293 137211772 + L2 LINE/L2 1131 1639**

**137211779 137211949 C AluSg/x SINE/Alu 312 134 (R2)**

**137211950 137212253 C AluYa5 SINE/Alu 308 5 ( AluYa5_3_174c ) (R1)**

**137212258 137212578 + L2 LINE/L2 1627 1947**

**137212554 137212927 + L2 LINE/L2 2989 3407**

**137213504 137213546 + (CA)n Simple_repeat 1 43**

**137214334 137214689 C L1PB1 LINE/L1 6865 6509**

**137214690 137214761 + (TATG)n Simple_repeat 2 73**

**137214762 137218915 C L1PB1 LINE/L1 6508 2204**

**137218989 137219080 C L1M3 LINE/L1 5763 5670**

**Ortholog in Chimp 140630229-140630302 Minus Nscore 0.00**

**140623491 140623619 + THER1_MD SINE/MIR 20 159**

**140624065 140624166 C MLT1I LTR/MaLR 352 244**

**140624461 140624657 + L1ME3B LINE/L1 5957 6171**

**140624850 140625174 + MER46B DNA/MER2_type 26 236**

**140625543 140625631 C MIRb SINE/MIR 263 174**

**140626226 140626439 C MER58C DNA/MER1_type 214 4**

**140628522 140629380 + L2 LINE/L2 20 1130**

**140629381 140629569 + MER5A DNA/MER1_type 1 189**

**140629570 140630054 + L2 LINE/L2 1131 1639**

**140630055 140630351 C AluSg SINE/Alu 309 5 (R12)**

**140630352 140630676 + L2 LINE/L2 1640 1946**

**140630652 140631026 + L2 LINE/L2 2989 3407**

**140631605 140631635 + (CA)n Simple_repeat 1 31**

**140632431 140632786 C L1PB1 LINE/L1 6865 6509**

**140632787 140632850 + (TG)n Simple_repeat 2 65**

**140632851 140637013 C L1PB1 LINE/L1 6508 2204**

**140637087 140637178 C L1M5 LINE/L1 5768 5670**

**__________________________________________________________________________________**

**AluYa5_4_7c 4455005-4455315 C_INTER_RMD_M_DISRUPTED**

**4449668 4449692 + (TG)n Simple_repeat 2 26**

**4449917 4450052 C MIRb SINE/MIR 223 80**

**4450570 4450880 C AluSg SINE/Alu 310 1**

**4450889 4451758 C L1MB5 LINE/L1 6174 5311**

**4451759 4452070 C AluJb SINE/Alu 312 1**

**4452071 4452318 C L1M LINE/L1 5310 5099**

**4452319 4452662 C MER1B DNA/MER1_type 338 1**

**4452663 4453283 C L1M LINE/L1 5098 4449**

**4453285 4453634 + Charlie4a DNA/MER1_type 111 490**

**4454049 4454203 + L2 LINE/L2 2975 3160**

**4454379 4454467 C ORSL DNA/Tip100 253 157**

**4454521 4454815 C AluJb SINE/Alu 288 1**

**4454823 4455004 C AluSg/x SINE/Alu 312 130 (R2)**

**4455005 4455315 C AluYa5 SINE/Alu 310 1 ( AluYa5_4_7c ) (R1)**

**4455316 4455415 C ORSL DNA/Tip100 106 14**

**4455720 4455746 + (CTCA)n Simple_repeat 3 29**

**4455846 4456317 C MLT1D LTR/MaLR 483 1**

**4456327 4456417 + MIRb SINE/MIR 96 190**

**4456649 4456715 C MER5B DNA/MER1_type 78 9**

**4457636 4458040 C MLT1C LTR/MaLR 457 21**

**4458099 4458236 C MIRb SINE/MIR 164 20**

**4458662 4458954 C AluSg SINE/Alu 292 1**

**4458997 4459075 C Charlie4 DNA/MER1_type 1904 1826**

**4460396 4460527 C MIRb SINE/MIR 151 30**

**4461085 4461387 C AluSx SINE/Alu 304 1**

**4461551 4461647 C L2 LINE/L2 3373 3269**

**Ortholog in Chimp 4495838-4495920 Minus Nscore 0.00**

**4490506 4490552 + (TG)n Simple_repeat 2 48**

**4490677 4490742 C L1P4b LINE/L1 722 657**

**4490838 4490911 C MIR SINE/MIR 148 73**

**4491428 4491735 C AluSg SINE/Alu 307 1**

**4491744 4492432 C L1MB5 LINE/L1 6174 5492**

**4492433 4492454 + (TTTTA)n Simple_repeat 4 25**

**4492455 4492624 C L1MB5 LINE/L1 5491 5305**

**4492625 4492932 C AluJb SINE/Alu 308 1**

**4492933 4493146 C L1M LINE/L1 5304 5098**

**4493147 4493490 C MER1B DNA/MER1_type 338 1**

**4493491 4494115 C L1M LINE/L1 5097 4447**

**4494117 4494466 + Charlie4a DNA/MER1_type 111 490**

**4494881 4495034 + L2 LINE/L2 2975 3160**

**4495206 4495351 C ORSL DNA/Tip100 259 107**

**4495352 4495650 C AluJb SINE/Alu 288 1**

**4495654 4495966 C AluSx SINE/Alu 312 1 (R12)**

**4495967 4496079 C ORSL DNA/Tip100 106 1**

**4496386 4496412 + (CTCA)n Simple_repeat 3 29**

**4496511 4496979 C MLT1D LTR/MaLR 483 1**

**4496989 4497075 + MIRb SINE/MIR 96 190**

**4497307 4497373 C MER5B DNA/MER1_type 78 9**

**4498299 4498703 C MLT1C LTR/MaLR 457 21**

**4498762 4498899 C MIRb SINE/MIR 164 20**

**4499325 4499617 C AluSg SINE/Alu 292 1**

**4499664 4499733 C Charlie4 DNA/MER1_type 1895 1826**

**4501049 4501180 C MIRb SINE/MIR 151 30**

**4501738 4502040 C AluSx SINE/Alu 304 1**

**4502204 4502300 C L2 LINE/L2 3373 3269**

**__________________________________________________________________________________**

**AluYa5_4_60 56521566-56521872 C_INTER_RMD_M_DISRUPTED**

**56514557 56514605 + AT_rich Low_complexity 1 49**

**56515564 56515609 + AT_rich Low_complexity 1 46**

**56515728 56516020 + AluJb SINE/Alu 3 299**

**56516021 56516082 C MER5A DNA/MER1_type 128 66**

**56516695 56517017 C L2 LINE/L2 2478 2141**

**56517047 56517171 + MIRm SINE/MIR 84 200**

**56517540 56517836 C AluSq SINE/Alu 294 1**

**56518292 56518432 + L2 LINE/L2 3256 3404**

**56518753 56518876 C L2 LINE/L2 3398 3247**

**56519075 56519352 + AluJb SINE/Alu 1 277**

**56519723 56520022 + AluJb SINE/Alu 1 297**

**56520867 56520954 + AluJo SINE/Alu 109 196**

**56521566 56521872 + AluYa5 SINE/Alu 1 305 ( AluYa5_4_60 ) (R1)**

**56521873 56522048 + AluSg/x SINE/Alu 126 306 (R2)**

**56522406 56522718 + AluSp SINE/Alu 1 313**

**56522819 56523029 + L2 LINE/L2 1622 1809**

**56523030 56523322 C AluY SINE/Alu 292 1**

**56523323 56524215 + L2 LINE/L2 1810 2789**

**56524387 56524685 + AluSx SINE/Alu 1 299**

**56525615 56525763 + L1ME4a LINE/L1 5944 6110**

**56525765 56525797 + AT_rich Low_complexity 1 33**

**56525976 56526276 C MER33 DNA/MER1_type 323 1**

**56527129 56527414 C AluSx SINE/Alu 291 1**

**56527419 56527706 C AluJo SINE/Alu 288 1**

**56527717 56528252 C Charlie2 DNA/MER1_type 2830 2241**

**56528294 56528630 C Charlie2 DNA/MER1_type 325 1**

**56528666 56529389 + L1M3 LINE/L1 3952 4692**

**Ortholog in Chimp 74595672-74595746 Minus Nscore 0.00**

**74588170 74588890 C L1M3 LINE/L1 4692 3952**

**74588926 74589262 + Charlie2 DNA/MER1_type 1 325**

**74589304 74589839 + Charlie2 DNA/MER1_type 2241 2830**

**74589850 74590137 + AluJo SINE/Alu 1 288**

**74590142 74590427 + AluSx SINE/Alu 1 291**

**74591280 74591578 + MER33 DNA/MER1_type 1 323**

**74591757 74591809 + AT_rich Low_complexity 1 53**

**74591824 74591939 C L1ME4a LINE/L1 6052 5944**

**74592869 74593167 C AluSx SINE/Alu 299 1**

**74593339 74594232 C L2 LINE/L2 2789 1810**

**74594233 74594526 + AluY SINE/Alu 1 293**

**74594527 74594737 C L2 LINE/L2 1809 1622**

**74594838 74595143 C AluSp SINE/Alu 306 1**

**74595498 74595792 C AluSx SINE/Alu 300 1 (R12)**

**74596405 74596492 C AluJo SINE/Alu 196 109**

**74597339 74597638 C AluJb SINE/Alu 297 1**

**74598009 74598286 C AluJb SINE/Alu 277 1**

**74598486 74598609 + L2 LINE/L2 3247 3398**

**74598930 74599033 C L2 LINE/L2 3404 3292**

**74599526 74599822 + AluSq SINE/Alu 1 294**

**74600191 74600313 C MIRm SINE/MIR 200 86**

**74600345 74600667 + L2 LINE/L2 2141 2478**

**74601281 74601342 + MER5A DNA/MER1_type 66 128**

**74601343 74601634 C AluJb SINE/Alu 298 3**

**74601753 74601798 + AT_rich Low_complexity 1 46**

**__________________________________________________________________________________**

**AluYa5_4_181c 133333498-133333809 C_INTER_RMD_M_DISRUPTED**

**133331277 133331346 C Alu SINE/Alu 292 223**

**133331347 133331626 C AluSx SINE/Alu 283 4**

**133331678 133331735 + (CA)n Simple_repeat 2 59**

**133332163 133332384 C MER58A DNA/MER1_type 213 1**

**133333181 133333491 C AluSg SINE/Alu 309 1 (R2)**

**133333498 133333809 C AluYa5 SINE/Alu 310 1 ( AluYa5_4_181c ) (R1)**

**133334698 133334747 + AT_rich Low_complexity 1 50**

**133335704 133335779 + AT_rich Low_complexity 1 76**

**133336173 133336247 + (TA)n Simple_repeat 1 76**

**133336276 133336306 + AT_rich Low_complexity 1 31**

**133336904 133336927 + AT_rich Low_complexity 1 24**

**133339719 133339823 + AT_rich Low_complexity 1 70**

**Ortholog in Chimp 135761549-135761610 Minus Nscore 0.00**

**135759410 135759477 C Alu SINE/Alu 290 223**

**135759478 135759757 C AluSx SINE/Alu 283 4**

**135759809 135759858 + (CA)n Simple_repeat 2 51**

**135760286 135760499 C MER58A DNA/MER1_type 213 1**

**135761293 135761598 C AluSg SINE/Alu 298 1 (R12)**

**135762486 135762538 + AT_rich Low_complexity 1 53**

**135763503 135763570 + AT_rich Low_complexity 1 68**

**135763964 135764047 + (TA)n Simple_repeat 2 86**

**135765122 135765142 + AT_rich Low_complexity 1 21**

**135767508 135767612 + AT_rich Low_complexity 1 70**

**__________________________________________________________________________________**

**AluYa5_4_193c 138163281-138163580 C_INTER_RMD_M_DISRUPTED**

**138158043 138158063 + AT_rich Low_complexity 1 21**

**138158139 138158317 + MIRb SINE/MIR 55 245**

**138158572 138158608 + (TG)n Simple_repeat 2 38**

**138159805 138159860 C MLT2B1 LTR/ERVL 562 498**

**138159901 138159944 + (GA)n Simple_repeat 1 44**

**138159970 138160402 C MLT2B1 LTR/ERVL 450 1**

**138160412 138160660 + L1MEc LINE/L1 15 264**

**138160661 138161019 + THE1D LTR/MaLR 2 381**

**138161020 138162260 + L1MEc LINE/L1 265 1641**

**138162346 138162555 C L1MCc LINE/L1 659 437**

**138162746 138162961 C L1MCc LINE/L1 440 241**

**138163223 138163245 + AT_rich Low_complexity 1 23**

**138163281 138163580 C AluYa5 SINE/Alu 296 1 ( AluYa5_4_193c ) (R2)**

**138163587 138163674 C AluY SINE/Alu 123 36 (R1)**

**138163713 138163908 C MIRb SINE/MIR 214 24**

**138164625 138164664 + AT_rich Low_complexity 1 40**

**138166178 138166548 C LTR16C LTR/ERVL 458 65**

**138167072 138167211 C ERVL-E LTR/ERVL 4918 4777**

**138167353 138167446 C ERVL-E LTR/ERVL 4639 4544**

**138167498 138167641 C MLT1H LTR/MaLR 279 133**

**138169131 138169152 + AT_rich Low_complexity 1 22**

**138169219 138170688 C L1PA15 LINE/L1 6157 4656**

**Ortholog in Chimp 140707381-140707509 Minus Nscore 0.00**

**140701612 140701642 + AT_rich Low_complexity 1 31**

**140702060 140702080 + AT_rich Low_complexity 1 21**

**140702156 140702334 + MIRb SINE/MIR 55 245**

**140702589 140702631 + (TG)n Simple_repeat 2 44**

**140703827 140703882 C MLT2B1 LTR/ERVL 562 498**

**140703927 140703968 + (GA)n Simple_repeat 1 42**

**140703994 140704426 C MLT2B1 LTR/ERVL 450 1**

**140704436 140704686 + L1MEc LINE/L1 15 264**

**140704687 140705046 + THE1D LTR/MaLR 1 381**

**140705047 140706309 + L1MEc LINE/L1 265 1641**

**140706395 140706604 C L1MCc LINE/L1 659 437**

**140706795 140707012 C L1MCc LINE/L1 440 239**

**140707272 140707294 + AT_rich Low_complexity 1 23**

**140707330 140707597 C AluY SINE/Alu 298 36 (R12)**

**140707636 140707831 C MIRb SINE/MIR 214 24**

**140708548 140708579 + AT_rich Low_complexity 1 32**

**140710121 140710509 C LTR16C LTR/ERVL 458 65**

**140711019 140711172 C ERVL-E LTR/ERVL 4931 4777**

**140711314 140711407 C ERVL-E LTR/ERVL 4639 4544**

**140711459 140711601 C MLT1H LTR/MaLR 279 133**

**140713087 140713108 + AT_rich Low_complexity 1 22**

**140713175 140714663 C L1PA15 LINE/L1 6157 4656**

**__________________________________________________________________________________**

**AluYa5_5_145c 108743684-108743830 C_INTER_RMD_M_DISRUPTED**

**108736540 108736703 + AluSg/x SINE/Alu 129 294**

**108738096 108738201 + L2 LINE/L2 3243 3359**

**108738828 108739138 + AluSx SINE/Alu 1 313**

**108739904 108739954 + A-rich Low_complexity 1 50**

**108740343 108740496 C MER46C DNA/MER2_type 330 155**

**108740718 108740751 + AT_rich Low_complexity 1 34**

**108741132 108741333 + MIR SINE/MIR 1 210**

**108743684 108743830 C AluYa5 SINE/Alu 309 163 ( AluYa5_5_145c )**

**108744126 108744426 + AluSx SINE/Alu 1 304**

**108744428 108744740 + AluSx SINE/Alu 2 309**

**108745658 108745678 + AT_rich Low_complexity 1 21**

**108746167 108746463 + AluSx SINE/Alu 2 298**

**108746597 108746705 C MIRb SINE/MIR 202 91**

**108746762 108747244 + Charlie7 DNA/MER1_type 6 552**

**108747245 108747551 + AluJb SINE/Alu 7 306**

**108747552 108747668 + Charlie7 DNA/MER1_type 553 671**

**108748424 108748714 C AluSg SINE/Alu 292 1**

**108748725 108749342 C L2 LINE/L2 2879 2198**

**108749346 108749633 C MER46B DNA/MER2_type 235 45**

**108749634 108749706 + AluS SINE/Alu 1 74**

**108749730 108749793 C MER46B DNA/MER2_type 63 2**

**108749796 108750032 C L2 LINE/L2 2192 1907**

**108750094 108750590 C HAL1 LINE/L1 1915 1320**

**Ortholog in Chimp 110576511-110576601 Minus Nscore 0.00**

**110569375 110569544 + AluSg/x SINE/Alu 122 294**

**110570936 110571040 + L2 LINE/L2 3285 3400**

**110571663 110571973 + AluSx SINE/Alu 1 313**

**110572742 110572792 + A-rich Low_complexity 1 50**

**110573182 110573336 C MER46C DNA/MER2_type 330 155**

**110573559 110573592 + AT_rich Low_complexity 1 34**

**110573972 110574173 + MIR SINE/MIR 1 210**

**110576506 110576650 C AluYa5 SINE/Alu 307 163 (OCCUPIED)**

**110576946 110577236 + AluSx SINE/Alu 1 294**

**110577251 110577559 + AluSx SINE/Alu 3 306**

**110578478 110578498 + AT_rich Low_complexity 1 21**

**110578987 110579282 + AluSx SINE/Alu 2 297**

**110579427 110579524 C MIRb SINE/MIR 196 98**

**110579581 110580062 + Charlie7 DNA/MER1_type 6 552**

**110580063 110580369 + AluJb SINE/Alu 7 306**

**110580370 110580486 + Charlie7 DNA/MER1_type 553 671**

**110581239 110581533 C AluSg SINE/Alu 296 1**

**110581544 110582161 C L2 LINE/L2 2879 2198**

**110582165 110582454 C MER46B DNA/MER2_type 235 46**

**110582456 110582528 + AluS SINE/Alu 1 74**

**110582554 110582615 C MER46B DNA/MER2_type 63 4**

**110582634 110582855 C L2 LINE/L2 2172 1907**

**110583276 110583413 C HAL1 LINE/L1 1461 1320**

**__________________________________________________________________________________**

**AluYa5_5_156c 117053181-117053487 C_INTER_RMD_M_DISRUPTED**

**117049061 117049167 + MIR SINE/MIR 52 154**

**117049892 117049970 C MIR SINE/MIR 173 96**

**117050292 117050417 C MIR SINE/MIR 258 131**

**117051297 117051735 C Charlie4a DNA/MER1_type 500 61**

**117051842 117051884 + (TTTTG)n Simple_repeat 2 44**

**117051888 117052183 C AluSc SINE/Alu 279 1**

**117052983 117053180 C AluY SINE/Alu 313 118 (R2)**

**117053181 117053487 C AluYa5 SINE/Alu 307 1 ( AluYa5_5_156c ) (R1)**

**117053604 117053932 C L1M5 LINE/L1 5791 5486**

**117054436 117054459 + (TC)n Simple_repeat 2 25**

**117055863 117055891 + AT_rich Low_complexity 1 29**

**117056013 117056033 + AT_rich Low_complexity 1 21**

**117057786 117058035 C MIRb SINE/MIR 254 6**

**117058280 117058438 C MER5A1 DNA/MER1_type 160 1**

**117058912 117059093 C MLT1H LTR/MaLR 558 375**

**117059141 117059362 C L1M5 LINE/L1 4188 3948**

**117059434 117059634 C MLT1H LTR/MaLR 264 54**

**117059653 117060956 C MLT1H-int LTR/MaLR 1470 2**

**Ortholog in Chimp 119056556-119056639 Minus Nscore 0.00**

**119052425 119052531 + MIR SINE/MIR 52 154**

**119053253 119053334 C MIR SINE/MIR 184 96**

**119053647 119053781 C THER1_MD SINE/MIR 274 138**

**119054660 119055098 C Charlie4a DNA/MER1_type 500 61**

**119055205 119055551 C AluSc SINE/Alu 330 1**

**119056348 119056688 C AluY SINE/Alu 282 4 (R12)**

**119056805 119057133 C L1M5 LINE/L1 5791 5483**

**119059062 119059093 + AT_rich Low_complexity 1 32**

**119060983 119061235 C MIRb SINE/MIR 254 6**

**119061241 119061303 C MADE2 DNA/Mariner 63 1**

**119061480 119061638 C MER5A1 DNA/MER1_type 160 1**

**119062112 119062302 C MLT1H LTR/MaLR 650 380**

**119062319 119062564 C L1P4 LINE/L1 4218 3948**

**119062648 119062848 C MLT1H LTR/MaLR 264 54**

**119062871 119064186 C MLT1H-int LTR/MaLR 1466 2**

**__________________________________________________________________________________**

**AluYa5_5_206 157289104-157289406 C_INTER_RMD_M_DISRUPTED**

**157281999 157282299 + AluSq SINE/Alu 1 300**

**157282339 157282460 C FLAM_C SINE/Alu 124 3**

**157282462 157282507 C MER4D0 LTR/ERV1 311 268**

**157282712 157282996 + AluSq SINE/Alu 1 285**

**157283005 157283184 + (CA)n Simple_repeat 1 180**

**157284326 157284376 + T-rich Low_complexity 1 51**

**157284469 157284611 C MIRb SINE/MIR 220 72**

**157284952 157285068 C MER41B LTR/ERV1 633 517**

**157285069 157285380 + AluSg SINE/Alu 1 310**

**157285381 157285887 C MER41B LTR/ERV1 516 2**

**157286961 157287007 + MIRb SINE/MIR 88 137**

**157287269 157287434 C MER20 DNA/MER1_type 202 32**

**157287436 157287475 + MER53 DNA 1 30**

**157287476 157287755 + AluY SINE/Alu 1 280**

**157287756 157287782 + (CAA)n Simple_repeat 1 27**

**157287791 157287836 + MER53 DNA 31 74**

**157287837 157288138 C AluSx SINE/Alu 299 1**

**157288241 157288541 C AluY SINE/Alu 304 1**

**157288620 157288758 C AluSq/x SINE/Alu 136 1**

**157289031 157289091 C MSTD LTR/MaLR 396 336**

**157289104 157289406 + AluYa5 SINE/Alu 11 310 ( AluYa5_5_206 ) (R1)**

**157289407 157289588 + AluJb SINE/Alu 133 311 (R2)**

**157289591 157289914 C MSTD LTR/MaLR 320 1**

**157289941 157290205 + AluSx SINE/Alu 5 267**

**157290579 157290758 C AluSx SINE/Alu 300 120**

**157291209 157291343 C AluSx SINE/Alu 135 1**

**157291353 157291661 C AluSg SINE/Alu 309 2**

**157291928 157292222 C AluJo SINE/Alu 288 1**

**157292638 157292776 + MIR3 SINE/MIR 20 159**

**157292779 157292938 + MER117 DNA/MER1_type 1 164**

**157293351 157293470 + L1M5 LINE/L1 5184 5308**

**157293471 157293784 + AluSp SINE/Alu 1 315**

**157293785 157293879 + L1M5 LINE/L1 5309 5388**

**157294034 157294113 + L1M5 LINE/L1 5542 5626**

**157294114 157294829 C LTR8 LTR/ERV1 691 1**

**157294830 157294895 + L1M5 LINE/L1 5627 5691**

**157294937 157294968 + AT_rich Low_complexity 1 32**

**157294969 157295256 C AluSg SINE/Alu 288 1**

**157295258 157295560 C AluJb SINE/Alu 303 1**

**157295629 157295662 + AT_rich Low_complexity 1 34**

**157295732 157296031 C AluSx SINE/Alu 300 1**

**157296068 157296122 + Alu SINE/Alu 209 266**

**157296123 157296215 C L1M5 LINE/L1 5489 5394**

**157296216 157296674 C L1PA10 LINE/L1 6166 5705**

**Ortholog in Chimp 159972869-159972929 Plus Nscore 0.00**

**159965713 159966012 + AluSq SINE/Alu 1 299**

**159966013 159966035 C LTR49 LTR/ERV1 28 12**

**159966052 159966174 C FLAM_C SINE/Alu 124 3**

**159966426 159966710 + AluSq SINE/Alu 1 285**

**159966748 159966824 C PRIMA4-int LTR/ERV1 565 488**

**159966856 159966916 C PRIMA4-int LTR/ERV1 559 498**

**159968057 159968107 + T-rich Low_complexity 1 51**

**159968200 159968344 C MIRb SINE/MIR 220 72**

**159968685 159968801 C MER41B LTR/ERV1 633 517**

**159968802 159969113 + AluSg SINE/Alu 1 310**

**159969114 159969620 C MER41B LTR/ERV1 516 2**

**159970695 159970741 + MIRb SINE/MIR 88 137**

**159971003 159971168 C MER20 DNA/MER1_type 202 32**

**159971170 159971209 + MER53 DNA 1 29**

**159971210 159971488 + AluY SINE/Alu 1 279**

**159971489 159971534 + (CAA)n Simple_repeat 3 48**

**159971535 159971584 + MER53 DNA 30 74**

**159971585 159971888 C AluSx SINE/Alu 302 1**

**159971991 159972281 C AluY SINE/Alu 294 1**

**159972342 159972474 C AluSq/x SINE/Alu 136 1**

**159972747 159972819 C MSTD LTR/MaLR 396 321**

**159972820 159973124 + AluJb SINE/Alu 11 311 (R12)**

**159973125 159973450 C MSTD LTR/MaLR 320 1**

**159973477 159973748 + AluSq SINE/Alu 5 274**

**159974115 159974291 C AluSx SINE/Alu 298 121**

**159974744 159974878 C AluSx SINE/Alu 135 1**

**159974888 159975195 C AluSg SINE/Alu 308 2**

**159975464 159975758 C AluJo SINE/Alu 288 1**

**159976147 159976310 + MIR3 SINE/MIR 4 159**

**159976313 159976471 + MER117 DNA/MER1_type 1 166**

**159976885 159977002 + L1M5 LINE/L1 5184 5308**

**159977799 159977906 + L1M5 LINE/L1 5191 5308**

**159977907 159978210 + AluSp SINE/Alu 1 305**

**159978211 159978283 + L1M5 LINE/L1 5309 5367**

**159978465 159978544 + L1M5 LINE/L1 5542 5626**

**159978545 159978872 C LTR8A LTR/ERV1 727 354**

**159979193 159979272 + L1M5 LINE/L1 5542 5626**

**159979273 159979988 C LTR8 LTR/ERV1 691 1**

**__________________________________________________________________________________**

**AluYa5_5_222c 166226720-166227031 C_INTER_RMD_M_DISRUPTED**

**166219683 166219973 + AluSx SINE/Alu 1 295**

**166221144 166221181 + (T)n Simple_repeat 1 38**

**166222665 166222802 + MIRb SINE/MIR 56 196**

**166222803 166222881 C MADE1 DNA/Mariner 80 1**

**166222882 166222927 + MIRb SINE/MIR 197 241**

**166222933 166223117 C MIR SINE/MIR 248 51**

**166224099 166224132 + (TG)n Simple_repeat 2 35**

**166224361 166224610 C MIR SINE/MIR 256 10**

**166225378 166225459 + GA-rich Low_complexity 3 84**

**166226544 166226719 C AluSq SINE/Alu 297 123 (R2)**

**166226720 166227031 C AluYa5 SINE/Alu 310 1 ( AluYa5_5_222c ) (R1)**

**166227457 166227669 + MIRb SINE/MIR 30 262**

**166228092 166228302 + MIR SINE/MIR 8 238**

**166228436 166228741 C AluSg SINE/Alu 310 5**

**166228909 166228991 C MIRb SINE/MIR 125 34**

**166229953 166230189 + (TA)n Simple_repeat 1 253**

**166230200 166230446 C L1ME1 LINE/L1 6102 5849**

**166230447 166230512 C L1PA2 LINE/L1 6155 6090**

**166230513 166230726 C L1ME1 LINE/L1 5848 5664**

**166230900 166231107 C MIRb SINE/MIR 210 13**

**166231119 166231255 C L2 LINE/L2 3418 3283**

**166231477 166231784 + AluSx SINE/Alu 1 307**

**166231918 166232122 C L2 LINE/L2 2883 2683**

**166233049 166233738 + L2 LINE/L2 2051 2831**

**166234012 166234044 + (TTCC)n Simple_repeat 4 36**

**Ortholog in Chimp 169083252-169083321 Minus Nscore 0.00**

**169076855 169076899 + (T)n Simple_repeat 1 45**

**169079194 169079331 + MIRb SINE/MIR 56 196**

**169079332 169079410 C MADE1 DNA/Mariner 80 1**

**169079411 169079456 + MIRb SINE/MIR 197 241**

**169079462 169079646 C MIR SINE/MIR 248 51**

**169080598 169080626 + (GA)n Simple_repeat 1 29**

**169080628 169080661 + (TG)n Simple_repeat 2 35**

**169080891 169081140 C MIR SINE/MIR 256 10**

**169081908 169081989 + GA-rich Low_complexity 3 84**

**169083073 169083370 C AluSq SINE/Alu 299 1 (R12)**

**169083796 169084008 + MIRb SINE/MIR 30 262**

**169084431 169084641 + MIR SINE/MIR 8 238**

**169084768 169085065 C AluSg SINE/Alu 302 5**

**169085233 169085315 C MIRb SINE/MIR 125 34**

**169086241 169086288 C L1ME3 LINE/L1 6155 6109**

**169086295 169086347 + (TA)n Simple_repeat 1 56**

**169086348 169086436 + (TTAA)n Simple_repeat 1 85**

**169086437 169086621 + (TA)n Simple_repeat 1 180**

**169086631 169086897 C L1ME1 LINE/L1 6103 5826**

**169086931 169086978 C L1ME3 LINE/L1 6155 6109**

**169086985 169087050 + (TA)n Simple_repeat 1 70**

**169087051 169087123 + AT_rich Low_complexity 1 73**

**169087124 169087306 + (TA)n Simple_repeat 1 180**

**169087316 169087734 C L1ME1 LINE/L1 6103 5664**

**169087908 169088115 C MIRb SINE/MIR 210 13**

**169088127 169088263 C L2 LINE/L2 3418 3283**

**169088485 169088793 + AluSx SINE/Alu 1 308**

**169088927 169089131 C L2 LINE/L2 2883 2683**

**169090060 169090749 + L2 LINE/L2 2051 2831**

**__________________________________________________________________________________**

**AluYa5_6_17c 11225377-11225693 C_INTER_RMD**

**11215539 11219919 C MER50-int LTR/ERV1 3918 15**

**11219926 11220436 C MER50 LTR/ERV1 516 1**

**11220478 11220771 C AluSx SINE/Alu 295 1**

**11220776 11221024 C L1ME1 LINE/L1 5963 5711**

**11221025 11221336 C AluSx SINE/Alu 302 14**

**11221337 11221793 C L1ME1 LINE/L1 5710 5204**

**11221794 11222095 C AluSx SINE/Alu 301 1**

**11222096 11222164 C L1MD LINE/L1 5203 5127**

**11222187 11222478 C AluSx SINE/Alu 310 21**

**11222485 11222805 C L1ME1 LINE/L1 4203 3892**

**11222806 11223118 C AluSx SINE/Alu 312 1**

**11223119 11223401 C L1ME1 LINE/L1 3891 3589**

**11223414 11223883 C MLT1E1 LTR/MaLR 654 103**

**11223891 11224205 C AluSp SINE/Alu 313 1**

**11224228 11224537 C AluSx SINE/Alu 311 1**

**11224540 11224658 C MLT1E1 LTR/MaLR 129 1**

**11224660 11225193 C L1MC LINE/L1 3580 3008**

**11225199 11225376 C AluSc SINE/Alu 309 132 (R2)**

**11225377 11225693 C AluYa5 SINE/Alu 305 1 ( AluYa5_6_17c ) (R1)**

**11225713 11225965 C L1ME1 LINE/L1 2970 2718**

**11225966 11226254 C AluSx SINE/Alu 303 1**

**11226255 11226381 C AluSq/x SINE/Alu 135 9**

**11226383 11226487 C L1ME1 LINE/L1 2740 2628**

**11226992 11227176 C MIR SINE/MIR 256 56**

**11227464 11227499 + (TC)n Simple_repeat 1 37**

**11227592 11227887 + AluY SINE/Alu 1 296**

**11228461 11228835 C MLT1A0 LTR/MaLR 365 1**

**11230345 11230483 C MER5B DNA/MER1_type 161 24**

**11231146 11231271 + FLAM_C SINE/Alu 1 127**

**11231896 11232081 C L1MA4 LINE/L1 6294 6104**

**11232087 11233014 C L1MA3 LINE/L1 6305 5377**

**Ortholog in Chimp 11302202-11302270 Minus Nscore 0.00**

**11290546 11296766 C MER50-int LTR/ERV1 6074 15**

**11296773 11297283 C MER50 LTR/ERV1 516 1**

**11297325 11297620 C AluSx SINE/Alu 297 1**

**11297625 11297870 C L1ME1 LINE/L1 5963 5711**

**11297871 11298042 C AluSx SINE/Alu 305 134**

**11298043 11298063 + (TTTTA)n Simple_repeat 4 24**

**11298064 11298186 C AluSx SINE/Alu 133 14**

**11298187 11298570 C L1ME1 LINE/L1 5710 5254**

**11298587 11298634 C L1MC LINE/L1 5250 5204**

**11298635 11298936 C AluSx SINE/Alu 301 1**

**11298937 11299006 C L1MC LINE/L1 5203 5126**

**11299028 11299320 C AluSx SINE/Alu 311 21**

**11299331 11299650 C L1MD LINE/L1 4199 3889**

**11299651 11299962 C AluSx SINE/Alu 312 1**

**11299963 11300256 C L1MD LINE/L1 3888 3578**

**11300258 11300712 C MLT1E1 LTR/MaLR 654 103**

**11300724 11301036 C AluSp SINE/Alu 311 1**

**11301037 11301054 C MLT1E1 LTR/MaLR 129 130**

**11301055 11301356 C AluSx SINE/Alu 303 1**

**11301357 11301477 C MLT1E LTR/MaLR 129 1**

**11301479 11302013 C L1M5 LINE/L1 3580 3005**

**11302014 11302319 C AluSc SINE/Alu 307 1 (R12)**

**11302320 11302591 C L1PB LINE/L1 3004 2718**

**11302592 11302880 C AluSx SINE/Alu 303 1**

**11302881 11303007 C AluSq/x SINE/Alu 135 9**

**11303009 11303122 C L1M2 LINE/L1 2740 2620**

**11303301 11303409 C L2 LINE/L2 3353 3248**

**11303616 11303800 C MIR SINE/MIR 256 56**

**11304088 11304122 + (TC)n Simple_repeat 1 36**

**11304942 11305077 + AluY SINE/Alu 163 298**

**11305078 11305177 C L2 LINE/L2 2343 2252**

**11305651 11306025 C MLT1A0 LTR/MaLR 365 1**

**11306129 11306225 + MIR SINE/MIR 64 167**

**11307514 11307651 C MER5B DNA/MER1_type 161 24**

**11308316 11308449 + FLAM_C SINE/Alu 1 133**

**11309066 11309251 C L1MA4 LINE/L1 6294 6104**

**11309257 11310184 C L1MA3 LINE/L1 7724 6796**

**__________________________________________________________________________________**

**AluYa5_6_27c 17812970-17813266 C_INTER_RMD_M_DISRUPTED**

**17808495 17808658 C MIR SINE/MIR 231 6**

**17808928 17809105 + MER58C DNA/MER1_type 10 215**

**17809156 17809449 + AluSp SINE/Alu 9 297**

**17809459 17809591 + AluSg SINE/Alu 2 134**

**17809592 17809894 + AluSg1 SINE/Alu 1 303**

**17809895 17810042 + AluSg SINE/Alu 135 280**

**17810436 17810736 + AluY SINE/Alu 1 300**

**17810739 17810911 + AluSp SINE/Alu 136 308**

**17811128 17811436 + AluSx SINE/Alu 1 309**

**17811852 17811879 + AT_rich Low_complexity 1 28**

**17812221 17812517 + AluY SINE/Alu 1 297**

**17812970 17813266 C AluYa5 SINE/Alu 298 1 ( AluYa5_6_27c )**

**17814675 17814697 + GC_rich Low_complexity 1 23**

**17814963 17815125 + (CCG)n Simple_repeat 3 165**

**17815600 17815761 + MIRb SINE/MIR 49 212**

**17815959 17816094 C MIR3 SINE/MIR 180 41**

**17816174 17816489 C AluSx SINE/Alu 312 1**

**17816503 17816548 + MIRb SINE/MIR 73 117**

**17816549 17816848 C AluSp SINE/Alu 307 1**

**17816849 17816983 + MIRb SINE/MIR 118 250**

**17818086 17818388 + AluSx SINE/Alu 1 295**

**17818401 17818431 + (TA)n Simple_repeat 1 31**

**17818707 17819007 + Tigger3(Golem) DNA/MER2_type 1 321**

**17819055 17819357 + AluJo SINE/Alu 1 303**

**17819371 17819399 + (TTTA)n Simple_repeat 3 32**

**17820006 17820305 + AluSx SINE/Alu 1 299**

**Ortholog in Chimp 18051051-18051354 Minus Nscore 0.00**

**18046615 18046778 C MIR SINE/MIR 231 6**

**18047043 18047220 + MER58C DNA/MER1_type 10 215**

**18047271 18047565 + AluSp SINE/Alu 9 300**

**18047575 18047709 + AluSg SINE/Alu 2 134**

**18047710 18048009 + AluSg1 SINE/Alu 1 300**

**18048010 18048158 + AluSg SINE/Alu 135 281**

**18048277 18048310 + (CACCC)n Simple_repeat 4 36**

**18048563 18048863 + AluY SINE/Alu 1 300**

**18048866 18049038 + AluSp SINE/Alu 136 308**

**18049255 18049559 + AluSx SINE/Alu 1 305**

**18050342 18050642 + AluY SINE/Alu 1 301**

**18051054 18051090 + (A)n Simple_repeat 1 37**

**18051107 18051403 C AluYa5 SINE/Alu 299 1 (OCCUPIED)**

**18052801 18052827 + GC_rich Low_complexity 1 27**

**18053094 18053250 + (CCG)n Simple_repeat 3 159**

**18053724 18053885 + MIRb SINE/MIR 49 212**

**18054083 18054218 C MIR3 SINE/MIR 180 41**

**18054297 18054323 + (TTTC)n Simple_repeat 2 28**

**18054328 18054609 C AluSx SINE/Alu 282 1**

**18054623 18054668 + MIRb SINE/MIR 73 117**

**18054669 18054972 C AluSp SINE/Alu 311 1**

**18054973 18055107 + MIRb SINE/MIR 118 250**

**18056213 18056510 + AluSx SINE/Alu 1 295**

**18056524 18056553 + (TA)n Simple_repeat 1 30**

**18056829 18057130 + Tigger3(Golem) DNA/MER2_type 1 321**

**18057178 18057480 + AluJo SINE/Alu 1 303**

**18057494 18057518 + (TTTA)n Simple_repeat 3 28**

**18058125 18058430 + AluSx SINE/Alu 1 305**

**__________________________________________________________________________________**

**AluYa5_6_55 34786199-34786502 C_INTER_RMD**

**34782706 34782987 C L1ME4a LINE/L1 6119 5827**

**34782988 34783292 C AluJo SINE/Alu 312 5**

**34783293 34783411 C L1ME4a LINE/L1 5826 5709**

**34783418 34783465 C MIRm SINE/MIR 152 106**

**34783526 34783611 C FLAM_A SINE/Alu 127 38**

**34783795 34783922 + L1ME1 LINE/L1 5584 5704**

**34783923 34784219 C AluSx SINE/Alu 297 1**

**34784220 34784658 + L1ME1 LINE/L1 5705 6116**

**34784659 34784957 C AluSx SINE/Alu 299 1**

**34784958 34785013 + L1ME1 LINE/L1 6117 6161**

**34785112 34785140 + AT_rich Low_complexity 1 29**

**34785936 34785971 + (TG)n Simple_repeat 2 37**

**34786199 34786502 + AluYa5 SINE/Alu 1 304 ( AluYa5_6_55 ) (R1)**

**34786503 34786664 + AluY SINE/Alu 133 294 (R2)**

**34786676 34786978 + AluSq SINE/Alu 1 303**

**34786989 34787297 + AluSx SINE/Alu 1 305**

**34787368 34787406 + MER5A DNA/MER1_type 150 188**

**34787448 34787703 C AluJo SINE/Alu 284 3**

**34787773 34788073 + AluY SINE/Alu 1 299**

**34788176 34788491 + L1ME4a LINE/L1 5539 5903**

**34788513 34788526 + AluY SINE/Alu 119 132**

**34788527 34788803 + AluSc SINE/Alu 1 277**

**34788805 34788849 + (CAAAA)n Simple_repeat 2 46**

**34788850 34788918 + AluY SINE/Alu 133 201**

**34788954 34789077 + L1ME4a LINE/L1 5963 6102**

**Ortholog in Chimp 35454993-35455062 Plus Nscore 0.00**

**35451418 35451699 C L1ME4a LINE/L1 6119 5827**

**35451700 35452004 C AluJo SINE/Alu 312 5**

**35452005 35452123 C L1ME4a LINE/L1 5826 5709**

**35452130 35452177 C MIRm SINE/MIR 152 106**

**35452238 35452325 C FLAM_A SINE/Alu 129 38**

**35452509 35452636 + L1ME1 LINE/L1 5584 5704**

**35452637 35452933 C AluSx SINE/Alu 297 1**

**35452934 35453391 + L1ME1 LINE/L1 5705 6116**

**35453392 35453697 C AluSx SINE/Alu 306 1**

**35453698 35453753 + L1ME1 LINE/L1 6117 6161**

**35453853 35453884 + AT_rich Low_complexity 1 32**

**35454679 35454716 + (TG)n Simple_repeat 2 39**

**35454944 35455246 + AluY SINE/Alu 1 295 (R12)**

**35455258 35455567 + AluSq SINE/Alu 1 303**

**35455578 35455893 + AluSx SINE/Alu 1 312**

**35455895 35455999 + MER5A DNA/MER1_type 67 188**

**35456008 35456292 C AluJo SINE/Alu 312 2**

**35456362 35456664 + AluY SINE/Alu 1 300**

**35456771 35457121 + L1ME4a LINE/L1 5539 5954**

**35457122 35457398 + AluSc SINE/Alu 1 277**

**35457400 35457424 + (CAAAA)n Simple_repeat 2 26**

**35457432 35457529 + FLAM_A SINE/Alu 5 129**

**35457530 35457654 + L1ME4a LINE/L1 5961 6101**

**__________________________________________________________________________________**

**AluYa5_6_210c 139878915-139879219 M_INTER_RMD**

**139871991 139872246 C AluJo SINE/Alu 288 1**

**139873702 139873930 C L1ME3B LINE/L1 6226 5976**

**139873981 139874518 C L1ME3B LINE/L1 5978 5393**

**139874519 139874806 + AluY SINE/Alu 2 287**

**139874810 139874852 + (TAAA)n Simple_repeat 2 44**

**139874853 139875042 C L1M LINE/L1 5392 5215**

**139875375 139875568 + L2 LINE/L2 1841 2044**

**139875569 139875670 + MER53 DNA 1 102**

**139876514 139876817 + AluSq SINE/Alu 1 304**

**139876822 139877103 + AluY SINE/Alu 1 280**

**139877774 139877867 C MIRm SINE/MIR 176 66**

**139878915 139879219 C AluYa5 SINE/Alu 310 1 ( AluYa5_6_210c ) (R12)**

**139879314 139879342 + (T)n Simple_repeat 1 29**

**139879838 139879951 + MIRb SINE/MIR 31 153**

**139879961 139880072 + L1M5 LINE/L1 5601 5711**

**139880075 139880202 + L1M5 LINE/L1 5754 5883**

**139880409 139880517 + MIRb SINE/MIR 131 241**

**139880529 139880825 + MARNA DNA/Mariner 263 569**

**139881144 139881171 + (CA)n Simple_repeat 1 28**

**139881187 139881501 + AluSx SINE/Alu 1 324**

**139881510 139881578 + (CA)n Simple_repeat 1 69**

**139881766 139881944 + MIRb SINE/MIR 96 267**

**139881959 139882042 + L2 LINE/L2 3336 3419**

**139883381 139883412 + (TTTTC)n Simple_repeat 3 34**

**139884112 139884214 C MIR SINE/MIR 147 26**

**139884859 139884889 + (CAAAAA)n Simple_repeat 5 35**

**139884900 139885198 C AluY SINE/Alu 303 5**

**Ortholog in Chimp 141919509-141922603 Minus Nscore 0.00**

**141912548 141912803 C AluJo SINE/Alu 288 1**

**141914259 141914487 C L1ME3B LINE/L1 6226 5976**

**141914538 141915075 C L1ME3B LINE/L1 5978 5393**

**141915076 141915363 + AluY SINE/Alu 2 287**

**141915367 141915393 + (TAAA)n Simple_repeat 2 28**

**141915394 141915615 C L1M LINE/L1 5392 5178**

**141915916 141916109 + L2 LINE/L2 1841 2044**

**141916110 141916211 + MER53 DNA 1 102**

**141917055 141917362 + AluSq SINE/Alu 1 308**

**141917367 141917643 + AluY SINE/Alu 1 275**

**141917644 141917668 + (TTTTG)n Simple_repeat 3 27**

**141918318 141918411 C MIRm SINE/MIR 176 66**

**141918606 141918653 + L3_Mars LINE/CR1 3465 3515**

**141919461 141919728 C AluJb SINE/Alu 310 44 (R2)**

**141919784 141920080 + AluSc SINE/Alu 1 302**

**141920102 141920414 C AluY SINE/Alu 311 1**

**141920777 141921276 C L2 LINE/L2 3419 2857**

**141921277 141921581 + AluSx SINE/Alu 1 306**

**141921582 141921802 C L2 LINE/L2 2856 2615**

**141922345 141922652 C AluSc SINE/Alu 307 1 (R1)**

**141923267 141923380 + MIRb SINE/MIR 31 153**

**141923390 141923501 + L1ME4a LINE/L1 5601 5711**

**141923504 141923831 + L1ME4a LINE/L1 5754 6112**

**141923838 141923939 + MIR SINE/MIR 131 234**

**141923943 141924267 + MARNA DNA/Mariner 242 580**

**141924572 141924593 + (CA)n Simple_repeat 1 22**

**141924609 141924920 + AluSx SINE/Alu 1 321**

**141924929 141924996 + (CA)n Simple_repeat 1 67**

**141925145 141925362 + MIRb SINE/MIR 49 267**

**141925377 141925460 + L2 LINE/L2 3336 3419**

**141926799 141926829 + (TTTTC)n Simple_repeat 4 34**

**141927528 141927630 C MIR SINE/MIR 147 26**

**141928277 141928307 + (CAAAAA)n Simple_repeat 5 35**

**141928318 141928608 C AluY SINE/Alu 295 5**

**__________________________________________________________________________________**

**AluYa5_6_226c 151997916-151998227 C_INTER_RMD_M_DISRUPTED**

**151990953 151991205 C MIRb SINE/MIR 252 4**

**151991508 151991860 + MLT1A LTR/MaLR 19 374**

**151992258 151992278 + AT_rich Low_complexity 1 21**

**151992462 151992569 C MIRb SINE/MIR 267 151**

**151992995 151993229 + MIR SINE/MIR 6 262**

**151993349 151993792 + MLT1H2 LTR/MaLR 18 484**

**151994040 151994070 + (GAAA)n Simple_repeat 1 31**

**151994613 151995684 C L1MC1 LINE/L1 6328 5214**

**151995739 151995766 + AT_rich Low_complexity 1 28**

**151995993 151996148 + L2 LINE/L2 3201 3378**

**151996292 151996517 + MIRb SINE/MIR 29 268**

**151997537 151997560 + (TA)n Simple_repeat 2 25**

**151997603 151997680 + (TCTA)n Simple_repeat 3 81**

**151997736 151997915 C AluSq SINE/Alu 301 130**

**151997916 151998227 C AluYa5 SINE/Alu 310 1 ( AluYa5_6_226c )**

**151998228 151998238 C AluSq SINE/Alu 129 121**

**151998440 151998491 + (CA)n Simple_repeat 2 53**

**151998644 151998866 C LTR16B LTR/ERVL 245 13**

**151998867 151998911 + (TCCCC)n Simple_repeat 2 48**

**151998901 151998965 + (TTCC)n Simple_repeat 2 66**

**151998966 151999273 C AluSg SINE/Alu 308 1**

**151999336 151999486 C MER58A DNA/MER1_type 224 45**

**151999552 151999797 C L1M4 LINE/L1 3566 3324**

**152000130 152000366 + MIR SINE/MIR 11 255**

**152000700 152000762 C L1MB1 LINE/L1 5672 5610**

**152000716 152001222 + L1MB1 LINE/L1 5635 6171**

**152001434 152001731 C L1M4 LINE/L1 3246 2963**

**152001848 152002177 C AluJb SINE/Alu 308 1**

**152002202 152003435 C L1MB8 LINE/L1 6165 5039**

**152003436 152003558 C FLAM_A SINE/Alu 124 2**

**152003559 152003838 C L1MB8 LINE/L1 5040 4756**

**152003988 152004392 + MSTB LTR/MaLR 1 426**

**152004407 152004727 C HAL1 LINE/L1 1544 1202**

**152004864 152005155 + AluSx SINE/Alu 5 296**

**152005181 152005316 + MIRb SINE/MIR 32 152**

**Ortholog in Chimp 154407352-154407433 Minus Nscore 0.00**

**154400385 154400637 C MIRb SINE/MIR 252 4**

**154400940 154401292 + MLT1A LTR/MaLR 19 374**

**154401892 154401999 C MIRb SINE/MIR 267 151**

**154402425 154402659 + MIR SINE/MIR 6 262**

**154402779 154403222 + MLT1H2 LTR/MaLR 18 484**

**154403470 154403497 + (GAAA)n Simple_repeat 1 28**

**154404042 154405113 C L1MC1 LINE/L1 6328 5214**

**154405168 154405195 + AT_rich Low_complexity 1 28**

**154405422 154405577 + L2 LINE/L2 3201 3378**

**154405721 154405947 + MIRb SINE/MIR 29 268**

**154406966 154407018 + (TA)n Simple_repeat 2 55**

**154407034 154407116 + (TCTA)n Simple_repeat 3 85**

**154407172 154407480 C AluSq SINE/Alu 301 1 (OCCUPIED)**

**154407693 154407729 + (CA)n Simple_repeat 2 39**

**154407879 154408101 C LTR16B LTR/ERVL 245 13**

**154408133 154408193 + (TTCC)n Simple_repeat 2 62**

**154408194 154408486 C AluSg SINE/Alu 308 1**

**154408549 154408699 C MER58A DNA/MER1_type 224 45**

**154408765 154409010 C L1M4 LINE/L1 3566 3324**

**154409343 154409579 + MIR SINE/MIR 11 255**

**154409913 154409975 C L1MB1 LINE/L1 5672 5610**

**154409929 154410435 + L1MB1 LINE/L1 5635 6171**

**154410647 154410943 C L1M4 LINE/L1 3246 2963**

**154411060 154411389 C AluJb SINE/Alu 308 1**

**154411414 154412647 C L1MB8 LINE/L1 6165 5038**

**154412648 154412770 C FLAM_A SINE/Alu 124 2**

**154412771 154413050 C L1MB8 LINE/L1 5040 4756**

**154413200 154413604 + MSTB LTR/MaLR 1 426**

**154413619 154413939 C HAL1 LINE/L1 1544 1202**

**154414073 154414369 + AluSx SINE/Alu 1 297**

**154414395 154414530 + MIRb SINE/MIR 32 152**

**__________________________________________________________________________________**

**AluYa5_7_7 6464465-6464775 C_INTER_RMD_M_DISRUPTED**

**6460668 6460706 + MER77 LTR/ERVL 578 607**

**6460707 6460750 + MER63 DNA/AcHobo 959 992**

**6460751 6461042 + AluSc SINE/Alu 1 294**

**6461043 6461106 + MER63 DNA/AcHobo 993 1058**

**6461178 6461497 + AluJo SINE/Alu 1 310**

**6461629 6461694 + L1MC5 LINE/L1 7585 7655**

**6461695 6461990 + AluY SINE/Alu 1 293**

**6461991 6462095 + L1MC5 LINE/L1 7656 7785**

**6462099 6462406 + AluSx SINE/Alu 1 306**

**6462625 6462711 + (TA)n Simple_repeat 2 88**

**6462730 6463007 C AluSg SINE/Alu 278 1**

**6463013 6463317 C AluJb SINE/Alu 305 4**

**6463326 6463633 C AluSx SINE/Alu 304 5**

**6463834 6464115 C AluJb SINE/Alu 308 12**

**6464126 6464436 C AluSx SINE/Alu 308 1**

**6464465 6464775 + AluYa5 SINE/Alu 1 310 ( AluYa5_7_7 ) (R1)**

**6464777 6464955 + AluSg/x SINE/Alu 121 299 (R2)**

**6465249 6465552 C AluSx SINE/Alu 307 3**

**6465885 6466194 C AluY SINE/Alu 309 1**

**6466196 6466484 C AluSx SINE/Alu 294 6**

**6466621 6466813 C MIRb SINE/MIR 240 20**

**6467015 6467139 C FLAM_C SINE/Alu 123 2**

**6468957 6468982 + AT_rich Low_complexity 1 26**

**6469605 6469930 + AluJo SINE/Alu 1 311**

**6469935 6470070 + AluSx SINE/Alu 1 136**

**6470071 6470383 + AluSp SINE/Alu 1 313**

**6470384 6470394 + AluSx SINE/Alu 137 136**

**6470395 6470698 + AluSg SINE/Alu 1 304**

**6470699 6470868 + AluSx SINE/Alu 137 307**

**6470882 6471191 C AluSp SINE/Alu 310 1**

**6471338 6471636 C AluY SINE/Alu 301 2**

**6471714 6471933 + L1ME4a LINE/L1 5665 5910**

**Ortholog in Chimp 6466998-6467074 Plus Nscore 0.00**

**6463175 6463213 + MER77 LTR/ERVL 578 607**

**6463214 6463257 + MER63 DNA/AcHobo 959 992**

**6463258 6463550 + AluSc SINE/Alu 1 295**

**6463551 6463614 + MER63 DNA/AcHobo 993 1058**

**6463686 6464007 + AluJo SINE/Alu 1 312**

**6464082 6464112 + (TTTTTG)n Simple_repeat 2 32**

**6464135 6464200 + L1MC5 LINE/L1 7585 7653**

**6464201 6464509 + AluY SINE/Alu 1 306**

**6464510 6464614 + L1MC5 LINE/L1 7654 7785**

**6464618 6464925 + AluSx SINE/Alu 1 306**

**6465142 6465184 + (TA)n Simple_repeat 2 44**

**6465209 6465486 C AluSg SINE/Alu 278 1**

**6465492 6465795 C AluJb SINE/Alu 304 4**

**6465804 6466119 C AluSx SINE/Alu 304 1**

**6466316 6466592 C AluJb SINE/Alu 303 12**

**6466606 6466920 C AluSx SINE/Alu 312 1**

**6466949 6467248 + AluY SINE/Alu 1 301 (R12)**

**6467541 6467845 C AluSx SINE/Alu 308 3**

**6468221 6468530 C AluY SINE/Alu 309 1**

**6468532 6468821 C AluSx SINE/Alu 295 6**

**6468958 6469150 C MIRb SINE/MIR 240 20**

**6469351 6469475 C FLAM_C SINE/Alu 123 2**

**6471293 6471316 + AT_rich Low_complexity 1 24**

**6471939 6472260 + AluJo SINE/Alu 1 307**

**6472271 6472406 + AluSx SINE/Alu 1 136**

**6472407 6472705 + AluSp SINE/Alu 1 299**

**6472706 6473008 + AluSg SINE/Alu 1 304**

**6473009 6473135 + AluSg/x SINE/Alu 137 260**

**6473480 6473690 C AluSx SINE/Alu 211 1**

**6473837 6474135 C AluY SINE/Alu 297 2**

**__________________________________________________________________________________**

**AluYa5_7_93c 72032063-72032372 C_INTER_RMD_M_DISRUPTED**

**72029693 72029798 C MLT2B4 LTR/ERVL 106 2**

**72029834 72030090 C L1MEc LINE/L1 396 131**

**72030253 72030867 C L1M5 LINE/L1 5823 5169**

**72030868 72031161 C AluSx SINE/Alu 292 1**

**72031162 72031229 C L1M5 LINE/L1 5168 5101**

**72031232 72031377 + L2 LINE/L2 2202 2381**

**72031418 72031551 + L2 LINE/L2 1852 1986**

**72031898 72032062 C AluSc SINE/Alu 289 132 (R2)**

**72032063 72032372 C AluYa5 SINE/Alu 309 1 ( AluYa5_7_93c ) (R1)**

**72032420 72032719 C AluY SINE/Alu 298 1**

**72032895 72033084 C L1MB8 LINE/L1 6178 5959**

**72033661 72033691 + GC_rich Low_complexity 1 31**

**72034084 72034164 + (CCCCG)n Simple_repeat 3 82**

**72035902 72036192 + AluSx SINE/Alu 1 295**

**72036284 72036373 C L2 LINE/L2 3400 3307**

**72036398 72036696 + AluJo SINE/Alu 1 294**

**72037169 72037189 + AT_rich Low_complexity 1 21**

**72037249 72037963 + L3 LINE/CR1 3727 4472**

**72037968 72038287 C L1ME1 LINE/L1 6170 5841**

**72038647 72038769 + FLAM_C SINE/Alu 1 123**

**72038771 72038798 + AT_rich Low_complexity 1 28**

**72039121 72039185 + MIRb SINE/MIR 11 77**

**72039186 72039318 + FLAM_C SINE/Alu 1 133**

**72039319 72039478 + MIRb SINE/MIR 78 259**

**Ortholog in Chimp 72810675-72810753 Minus Nscore 0.00**

**72808297 72808401 C MLT2B4 LTR/ERVL 106 3**

**72808438 72808694 C L1MEc LINE/L1 396 131**

**72808857 72809468 C L1M5 LINE/L1 5823 5169**

**72809469 72809758 C AluSx SINE/Alu 289 1**

**72809759 72809826 C L1M5 LINE/L1 5168 5101**

**72809829 72809981 + L2 LINE/L2 2202 2390**

**72810015 72810143 + L2 LINE/L2 1852 1981**

**72810506 72810802 C AluSx SINE/Alu 292 1 (R12)**

**72810848 72811159 C AluY SINE/Alu 299 1**

**72811335 72811524 C L1MB8 LINE/L1 6178 5959**

**72812101 72812134 + GC_rich Low_complexity 1 34**

**72812350 72812655 C AluSx SINE/Alu 296 1**

**72812867 72813056 C L1MB8 LINE/L1 6178 5959**

**72813601 72813697 + (CGG)n Simple_repeat 1 95**

**72814079 72814136 + GC_rich Low_complexity 1 58**

**72815868 72816162 + AluSx SINE/Alu 1 299**

**72816251 72816356 C L2 LINE/L2 3400 3245**

**72816368 72816667 + AluJo SINE/Alu 1 295**

**72817140 72817160 + AT_rich Low_complexity 1 21**

**72817206 72817934 + L3 LINE/CR1 3704 4472**

**__________________________________________________________________________________**

**AluYa5_7_95c 73678435-73678701 C_INTER_RMD_M_DISRUPTED**

**73672027 73672169 + AluSx SINE/Alu 1 143**

**73672170 73672472 + AluSq SINE/Alu 1 293**

**73672473 73672485 + AluSx SINE/Alu 144 143**

**73672486 73672781 + AluSx SINE/Alu 1 296**

**73672782 73672949 + AluSx SINE/Alu 144 301**

**73673018 73673194 C AluSg SINE/Alu 179 2**

**73673200 73673333 C L1M4 LINE/L1 5305 5166**

**73673334 73673627 C AluSp SINE/Alu 294 2**

**73673629 73673923 C AluSx SINE/Alu 295 1**

**73673924 73673938 C L1MC LINE/L1 5163 5150**

**73673956 73674040 + L1M5 LINE/L1 5690 5776**

**73674060 73674130 C MIR SINE/MIR 261 196**

**73674131 73674465 + AluJb SINE/Alu 1 306**

**73674475 73674536 + (GGAGA)n Simple_repeat 4 65**

**73674592 73674906 + AluJo SINE/Alu 1 307**

**73674944 73675172 + MLT1C LTR/MaLR 200 405**

**73675175 73675339 C AluSg/x SINE/Alu 297 133**

**73675361 73675532 + AluSg/x SINE/Alu 135 306**

**73675733 73676048 + AluSx SINE/Alu 3 312**

**73676179 73676503 + AluSx SINE/Alu 1 305**

**73676597 73676892 + AluJo SINE/Alu 26 312**

**73676908 73677012 + FLAM_A SINE/Alu 40 140**

**73677018 73677161 + AluSx SINE/Alu 1 139**

**73677162 73677450 + AluSg SINE/Alu 2 290**

**73677451 73677623 + AluSx SINE/Alu 140 303**

**73677908 73678184 C AluJo SINE/Alu 311 39**

**73678196 73678261 + (TTCC)n Simple_repeat 4 68**

**73678264 73678433 C AluSq SINE/Alu 304 135**

**73678435 73678701 C AluYa5 SINE/Alu 310 52 ( AluYa5_7_95c )**

**73678704 73678817 + CT-rich Low_complexity 3 114**

**73678815 73678853 + (CTTTG)n Simple_repeat 2 40**

**73678919 73678931 + (TA)n Simple_repeat 1 13**

**73678932 73679217 + AluSx SINE/Alu 1 287**

**73679218 73679240 + (TA)n Simple_repeat 2 24**

**73679271 73679584 C AluSx SINE/Alu 311 3**

**73680030 73680208 + MIRb SINE/MIR 20 220**

**73681032 73681339 + AluSx SINE/Alu 1 309**

**73681457 73681784 + AluSx SINE/Alu 1 308**

**73681803 73682091 + AluJb SINE/Alu 3 295**

**73682186 73682499 + AluY SINE/Alu 2 310**

**73682510 73682544 + U2 snRNA 1 35**

**73682565 73682864 + AluJb SINE/Alu 4 309**

**73683144 73683380 + AluSx SINE/Alu 1 294**

**73683384 73683533 + AluJo/FRAM SINE/Alu 137 290**

**73683619 73683661 C L1MB2 LINE/L1 6167 6131**

**73683662 73683952 C AluY SINE/Alu 294 1**

**73683953 73684190 C L1MB2 LINE/L1 6130 5891**

**73684202 73684489 C AluY SINE/Alu 294 27**

**73684522 73684807 C AluJo SINE/Alu 282 1**

**73684901 73684999 + (CA)n Simple_repeat 1 99**

**73685123 73685412 + AluSg SINE/Alu 1 288**

**73685447 73685721 + AluSx SINE/Alu 1 273**

**Ortholog in Chimp 74232274-74232294 Minus Nscore 0.00**

**74225157 74225299 + AluSx SINE/Alu 1 143**

**74225300 74225599 + AluSq SINE/Alu 1 293**

**74225600 74225612 + AluSx SINE/Alu 144 143**

**74225613 74225911 + AluSx SINE/Alu 1 299**

**74225912 74226076 + AluSx SINE/Alu 144 298**

**74226144 74226320 C AluSg SINE/Alu 179 2**

**74226326 74226460 C L1M4 LINE/L1 5305 5166**

**74226461 74226602 C AluSp/q SINE/Alu 299 160**

**74226875 74227015 C AluSp SINE/Alu 143 2**

**74227017 74227311 C AluSx SINE/Alu 295 1**

**74227345 74227429 + L1M5 LINE/L1 5690 5776**

**74227449 74227510 C MIR SINE/MIR 261 199**

**74227520 74227853 + AluJb SINE/Alu 1 305**

**74227863 74227924 + (GGAGA)n Simple_repeat 4 65**

**74227980 74228293 + AluJo SINE/Alu 1 307**

**74228331 74228559 + MLT1C LTR/MaLR 200 405**

**74228562 74228726 C AluSg/x SINE/Alu 297 133**

**74228750 74228919 + AluSg/x SINE/Alu 137 306**

**74229120 74229435 + AluSx SINE/Alu 3 312**

**74229563 74229885 + AluSx SINE/Alu 1 305**

**74229979 74230272 + AluJo SINE/Alu 26 312**

**74230288 74230392 + FLAM_A SINE/Alu 40 140**

**74230398 74230541 + AluSx SINE/Alu 1 139**

**74230542 74230830 + AluSg SINE/Alu 2 290**

**74230831 74231004 + AluSx SINE/Alu 140 307**

**74231287 74231548 C AluJo SINE/Alu 310 54**

**74232084 74232351 C AluSq SINE/Alu 311 41**

**74232354 74232455 + CT-rich Low_complexity 2 102**

**74232461 74232488 + (CTTTG)n Simple_repeat 3 30**

**74232565 74232577 + (TA)n Simple_repeat 2 14**

**74232578 74232863 + AluSx SINE/Alu 1 287**

**74232864 74232884 + (TA)n Simple_repeat 1 21**

**74232918 74233231 C AluSx SINE/Alu 312 3**

**74233679 74233857 + MIRb SINE/MIR 28 229**

**74234683 74234993 + AluSx SINE/Alu 1 312**

**74235111 74235424 + AluSx SINE/Alu 1 310**

**74235443 74235731 + AluJb SINE/Alu 3 295**

**74235826 74236139 + AluY SINE/Alu 2 310**

**74236150 74236184 + U2 snRNA 1 35**

**74236205 74236504 + AluJb SINE/Alu 4 309**

**74236784 74237020 + AluSx SINE/Alu 1 294**

**74237024 74237179 + AluJo/FRAM SINE/Alu 137 290**

**74237262 74237307 C L1MB2 LINE/L1 6170 6131**

**74237308 74237598 C AluY SINE/Alu 294 1**

**74237599 74237836 C L1MB2 LINE/L1 6130 5891**

**74237845 74238136 C AluY SINE/Alu 298 27**

**74238168 74238453 C AluJo SINE/Alu 282 1**

**74238546 74238647 + (CA)n Simple_repeat 2 103**

**74238769 74239089 + AluSg SINE/Alu 1 310**

**74239094 74239368 + AluSx SINE/Alu 1 273**

**__________________________________________________________________________________**

**AluYa5_7_98 75288912-75289206 C_INTER_RMD_M_DISRUPTED**

**75286031 75286079 + MIR3 SINE/MIR 109 159**

**75286192 75286212 + AT_rich Low_complexity 1 21**

**75286254 75286552 + AluSp SINE/Alu 14 313**

**75286558 75286591 + (TA)n Simple_repeat 2 35**

**75286597 75286870 + AluJo SINE/Alu 1 302**

**75287088 75287236 + MIR3 SINE/MIR 54 208**

**75287274 75287453 + (TTC)n Simple_repeat 2 180**

**75287454 75287733 C AluSx SINE/Alu 287 2**

**75287818 75287995 + (TCCA)n Simple_repeat 1 180**

**75288019 75288045 C L2 LINE/L2 3378 3353**

**75288046 75288218 + (TCCA)n Simple_repeat 2 174**

**75288219 75288644 C L2 LINE/L2 3352 2923**

**75288912 75289206 + AluYa5 SINE/Alu 14 306 ( AluYa5_7_98 ) (R1)**

**75289207 75289374 + AluSg/x SINE/Alu 134 301 (R2)**

**75289479 75289651 + MIRb SINE/MIR 4 179**

**75289746 75289805 C MIRb SINE/MIR 182 121**

**75289806 75290107 + AluSx SINE/Alu 1 307**

**75290108 75290232 C MIRb SINE/MIR 121 1**

**75291159 75291442 C AluSx SINE/Alu 282 1**

**75291509 75291586 + L3 LINE/CR1 4297 4374**

**75291742 75291938 + AluJb SINE/Alu 3 145**

**75291939 75292175 + AluSg/x SINE/Alu 75 311**

**75292176 75292339 + AluJb SINE/Alu 146 301**

**75292378 75292429 + MIRb SINE/MIR 27 78**

**75292787 75292833 C Alu SINE/Alu 34 1**

**75292834 75292889 C L1M3 LINE/L1 4181 4126**

**Ortholog in Chimp 75200368-75200432 Plus Nscore 0.00**

**5197747 75197769 + (TA)n Simple_repeat 2 24**

**75197815 75198097 + AluSp SINE/Alu 14 298**

**75198098 75198133 + (TA)n Simple_repeat 2 37**

**75198139 75198412 + AluJo SINE/Alu 1 300**

**75198514 75198539 + (CA)n Simple_repeat 1 26**

**75198643 75198791 + MIR3 SINE/MIR 54 208**

**75198825 75198855 + (TTC)n Simple_repeat 1 31**

**75198856 75199160 C AluSx SINE/Alu 312 2**

**75199245 75199422 + (TCCA)n Simple_repeat 1 180**

**75199443 75199622 + (TCCA)n Simple_repeat 1 180**

**75199627 75200051 C L2 LINE/L2 3357 2923**

**75200319 75200606 + AluSx SINE/Alu 14 302 (R12)**

**75200716 75200883 + MIRb SINE/MIR 9 179**

**75200999 75201037 C MIRb SINE/MIR 159 121**

**75201038 75201253 + AluSq SINE/Alu 1 220**

**75201530 75201819 + AluSx SINE/Alu 16 307**

**75201820 75201944 C MIRb SINE/MIR 130 1**

**75202872 75203155 C AluSx SINE/Alu 282 1**

**75203224 75203301 + L3 LINE/CR1 4297 4374**

**75203457 75203586 + AluJb SINE/Alu 3 144**

**75203588 75203652 C AluS SINE/Alu 78 14**

**75203653 75203880 + AluSg/x SINE/Alu 74 301**

**75203891 75204181 + AluSx SINE/Alu 19 309**

**__________________________________________________________________________________**

**AluYa5_7_202 128514792-128515097 C_INTER_RMD_M_DISRUPTED**

**128512066 128512193 C L1MA10 LINE/L1 6334 6203**

**128512216 128512332 + AluSg/x SINE/Alu 193 311**

**128512387 128512531 C L1ME1 LINE/L1 5997 5849**

**128512546 128512603 C L1M5 LINE/L1 5889 5832**

**128512604 128512664 + L1MB8 LINE/L1 6120 6178**

**128512666 128512976 + L1MD LINE/L1 5429 5715**

**128512977 128513263 C AluSc SINE/Alu 286 1**

**128513264 128513274 + L1MD LINE/L1 5716 5715**

**128513275 128513565 C AluSx SINE/Alu 288 1**

**128513566 128513798 + L1MB7 LINE/L1 5701 5925**

**128513799 128514107 C AluSx SINE/Alu 311 1**

**128514108 128514381 + L1MB7 LINE/L1 5923 6184**

**128514389 128514493 C L1M5 LINE/L1 5812 5704**

**128514508 128514788 + AluJo SINE/Alu 16 296**

**128514792 128515097 + AluYa5 SINE/Alu 1 306 ( AluYa5_7_202 ) (R1)**

**128515098 128515123 + AT_rich Low_complexity 1 26**

**128515126 128515410 + AluY SINE/Alu 1 284 (R2)**

**128515411 128515453 + (TTAAA)n Simple_repeat 3 45**

**128515468 128515593 + FLAM_A SINE/Alu 1 126**

**128515599 128515644 + L1MA9 LINE/L1 6269 6312**

**128515648 128516732 C L1M5 LINE/L1 5711 4619**

**128516768 128516947 + MIR SINE/MIR 28 208**

**128516948 128517409 + MER89 LTR/ERV1 1 559**

**128517795 128517928 + MIR3 SINE/MIR 40 167**

**128517987 128518103 C MER5A1 DNA/MER1_type 115 1**

**Ortholog in Chimp 129493608-129493671 Plus Nscore 0.00**

**129490894 129491021 C L1MA10 LINE/L1 6334 6203**

**129491044 129491159 + AluSg/x SINE/Alu 193 310**

**129491219 129491345 C L1MB7 LINE/L1 6002 5884**

**129491377 129491434 C L1M5 LINE/L1 5889 5832**

**129491435 129491495 + L1MB8 LINE/L1 6120 6178**

**129491497 129491806 + L1MD LINE/L1 5429 5714**

**129491807 129492093 C AluSc SINE/Alu 286 1**

**129492094 129492104 + L1MD LINE/L1 5715 5714**

**129492105 129492395 C AluSx SINE/Alu 288 1**

**129492396 129492623 + L1MB7 LINE/L1 5701 5920**

**129492624 129492937 C AluSx SINE/Alu 312 1**

**129492938 129493211 + L1MB7 LINE/L1 5921 6184**

**129493219 129493323 C L1M5 LINE/L1 5812 5704**

**129493338 129493618 + AluJo SINE/Alu 16 296**

**129493623 129493907 + AluY SINE/Alu 1 283 (R12)**

**129493908 129493930 + (TTAAA)n Simple_repeat 3 25**

**129493945 129494070 + FLAM_A SINE/Alu 1 126**

**129494076 129494121 + L1MA9 LINE/L1 6269 6312**

**129494125 129495207 C L1M5 LINE/L1 5711 4619**

**129495242 129495421 + MIR SINE/MIR 28 208**

**129495422 129495882 + MER89 LTR/ERV1 1 559**

**129496269 129496406 + MIR3 SINE/MIR 36 167**

**129496465 129496581 C MER5A1 DNA/MER1_type 115 1**

**__________________________________________________________________________________**

**AluYa5_8_79c 70520179-70520481 C_INTER_RMD_M_DISRUPTED**

**70515846 70516070 + L1MB5 LINE/L1 5948 6176**

**70516454 70516500 + (CA)n Simple_repeat 1 47**

**70517089 70517382 + AluJb SINE/Alu 1 291**

**70517385 70517679 + AluY SINE/Alu 1 295**

**70517850 70518121 + AluSx SINE/Alu 1 273**

**70518137 70518166 + (CATA)n Simple_repeat 2 31**

**70518532 70519087 C L1MB7 LINE/L1 6184 5648**

**70519282 70519391 C MIR SINE/MIR 155 32**

**70519551 70519780 C MIR SINE/MIR 266 2**

**70519865 70520165 C AluSg SINE/Alu 300 1 (R2)**

**70520179 70520481 C AluYa5 SINE/Alu 303 1 ( AluYa5_8_79c ) (R1)**

**70520649 70520736 + MER117 DNA/MER1_type 4 91**

**70521415 70521692 + AluJo SINE/Alu 1 249**

**70521740 70521836 + FLAM_A SINE/Alu 19 129**

**70522109 70522138 + AT_rich Low_complexity 1 30**

**70523994 70524166 C L1ME4a LINE/L1 5945 5764**

**70524179 70524332 + L2 LINE/L2 2730 2895**

**70524382 70524500 + MER5A1 DNA/MER1_type 38 158**

**70524642 70524999 + MLT1A LTR/MaLR 4 361**

**70525017 70525103 + L2 LINE/L2 3044 3130**

**70525241 70525465 + L1MB5 LINE/L1 5258 5491**

**Ortholog in Chimp 67749348-67749412 Minus Nscore 0.00**

**67745064 67745288 + L1MB5 LINE/L1 5948 6176**

**67745672 67745714 + (CA)n Simple_repeat 1 43**

**67746303 67746597 + AluJb SINE/Alu 1 292**

**67746599 67746892 + AluY SINE/Alu 1 294**

**67747063 67747334 + AluSx SINE/Alu 1 273**

**67747350 67747383 + (CATA)n Simple_repeat 2 35**

**67747749 67748304 C L1MB7 LINE/L1 6184 5648**

**67748507 67748608 C MIR SINE/MIR 141 32**

**67748771 67748998 C MIR SINE/MIR 266 2**

**67749089 67749395 C AluSg SINE/Alu 306 1 (R12)**

**67749566 67749653 + MER117 DNA/MER1_type 4 91**

**67750355 67750632 + AluJo SINE/Alu 1 249**

**67750680 67750775 + FLAM_A SINE/Alu 19 128**

**67752930 67753103 C L1ME4a LINE/L1 5946 5764**

**67753116 67753270 + L2 LINE/L2 2730 2895**

**67753320 67753438 + MER5A1 DNA/MER1_type 38 158**

**67753579 67753936 + MLT1A LTR/MaLR 4 361**

**67753954 67754040 + L2 LINE/L2 3044 3130**

**67754178 67754402 + L1MB5 LINE/L1 5258 5491**

**__________________________________________________________________________________**

**AluYa5_9_112 83765406-83765720 C_INTER_RMD_M_DISRUPTED**

**83761286 83761639 C THE1B LTR/MaLR 362 3**

**83761647 83761681 + AT_rich Low_complexity 1 35**

**83761959 83762007 C L1MC4 LINE/L1 8035 7986**

**83762008 83762413 C MSTB LTR/MaLR 426 1**

**83762414 83762518 C L1MC4 LINE/L1 7985 7892**

**83762519 83762562 + (CA)n Simple_repeat 2 45**

**83762563 83762886 C L1MC4 LINE/L1 7891 7550**

**83764599 83765404 C L1MCa LINE/L1 3226 2407**

**83765406 83765720 + AluYa5 SINE/Alu 1 310 ( AluYa5_9_112 ) (R1)**

**83765721 83765873 + AluSq SINE/Alu 125 276 (R2)**

**83765879 83765911 + (TAAA)n Simple_repeat 1 33**

**83765912 83766145 C L1MCa LINE/L1 2417 2175**

**83766147 83766746 C L1MA9 LINE/L1 6275 5643**

**83766752 83766923 C L1MCb LINE/L1 2826 2621**

**83766924 83767225 C AluSp SINE/Alu 301 1**

**83767226 83767385 C L1MCb LINE/L1 2638 2457**

**83767386 83767681 C AluY SINE/Alu 294 1**

**83767682 83767763 C L1MCb LINE/L1 2456 2380**

**83767764 83768054 C AluJb SINE/Alu 287 1**

**83768055 83768382 C L1MCb LINE/L1 2379 2011**

**83768383 83768439 + (TAGA)n Simple_repeat 4 61**

**Ortholog in Chimp 80873432-80873506 Plus Nscore 0.00**

**80869292 80869645 C THE1B LTR/MaLR 362 3**

**80869965 80870013 C L1MC4 LINE/L1 8035 7986**

**80870014 80870419 C MSTB LTR/MaLR 426 1**

**80870420 80870522 C L1MC4 LINE/L1 7985 7892**

**80870523 80870558 + (CA)n Simple_repeat 2 37**

**80870559 80870882 C L1MC4 LINE/L1 7891 7550**

**80872595 80873382 C L1MCa LINE/L1 3226 2418**

**80873383 80873662 + AluSq SINE/Alu 1 276 (R12)**

**80873668 80873712 + (TAAA)n Simple_repeat 1 45**

**80873713 80873946 C L1MCa LINE/L1 2426 2175**

**80873948 80874547 C L1MA9 LINE/L1 6275 5643**

**80874553 80874724 C L1MCa LINE/L1 2775 2615**

**80874725 80875026 C AluSp SINE/Alu 301 1**

**80875027 80875186 C L1MCa LINE/L1 2638 2457**

**80875187 80875482 C AluY SINE/Alu 294 1**

**80875483 80875564 C L1MCb LINE/L1 2456 2380**

**80875565 80875855 C AluJb SINE/Alu 287 1**

**80875856 80876180 C L1MCb LINE/L1 2379 2011**

**80876181 80876230 + (TAGA)n Simple_repeat 4 53**

**__________________________________________________________________________________**

**AluYa5_9_171c 130155841-130156142 C_INTER_RMD_M_DISRUPTED**

**130150249 130150447 + MIRb SINE/MIR 1 213**

**130150848 130151050 + MIRb SINE/MIR 22 253**

**130151366 130151611 + MIR SINE/MIR 6 261**

**130151602 130151741 + L2 LINE/L2 3239 3389**

**130152065 130152246 + L2 LINE/L2 3184 3375**

**130152774 130152909 + AluSq/x SINE/Alu 1 136**

**130152910 130153214 + AluY SINE/Alu 1 303**

**130153219 130153540 + AluJo SINE/Alu 3 296**

**130153543 130153832 + AluSg SINE/Alu 1 296**

**130153833 130153994 + AluSg/x SINE/Alu 137 291**

**130154005 130154310 + AluY SINE/Alu 1 297**

**130154953 130155029 C MIR3 SINE/MIR 167 93**

**130155770 130155807 + (TTCA)n Simple_repeat 2 39**

**130155841 130156142 C AluYa5 SINE/Alu 302 1 ( AluYa5_9_171c ) (R2)**

**130156143 130156343 C AluSx SINE/Alu 201 1 (R1)**

**130156376 130156578 + MIR SINE/MIR 29 241**

**130156766 130157076 + AluSq SINE/Alu 1 306**

**130158073 130158617 C L2 LINE/L2 3399 2844**

**130158618 130158905 + AluSx SINE/Alu 1 312**

**130158910 130159212 + AluSx SINE/Alu 1 301**

**130159215 130159548 + AluJo SINE/Alu 1 291**

**130159549 130159693 C L2 LINE/L2 2843 2702**

**130160129 130160365 C MLT1H LTR/MaLR 549 339**

**130160366 130160546 + MER3 DNA/MER1_type 1 148**

**130160547 130160846 C AluSx SINE/Alu 297 1**

**130160847 130160898 + MER3 DNA/MER1_type 149 207**

**130160899 130161211 C MLT1H LTR/MaLR 338 19**

**Ortholog in Chimp 128183428-128183480 Minus Nscore 0.00**

**128177787 128177984 + MIRb SINE/MIR 1 213**

**128178385 128178589 + MIRb SINE/MIR 22 253**

**128178905 128179150 + MIR SINE/MIR 6 261**

**128179141 128179281 + L2 LINE/L2 3239 3389**

**128179472 128179786 + L2 LINE/L2 3077 3375**

**128180312 128180447 + AluSq/x SINE/Alu 1 136**

**128180448 128180752 + AluY SINE/Alu 1 303**

**128180757 128181082 + AluJo SINE/Alu 3 296**

**128181085 128181370 + AluSg SINE/Alu 1 292**

**128181371 128181536 + AluSg/x SINE/Alu 137 291**

**128181547 128181847 + AluY SINE/Alu 1 293**

**128182512 128182568 C MIRb SINE/MIR 153 99**

**128183309 128183379 + (TTCA)n Simple_repeat 1 71**

**128183380 128183681 C AluSx SINE/Alu 302 1 (R12)**

**128183682 128183695 + (TTCA)n Simple_repeat 3 16**

**128183714 128183916 + MIR SINE/MIR 29 241**

**128184104 128184416 + AluSq SINE/Alu 1 308**

**128185413 128185955 C L2 LINE/L2 3399 2836**

**128185957 128186090 + AluSq/x SINE/Alu 1 134**

**128186107 128186409 + AluSx SINE/Alu 1 301**

**128186412 128186742 + AluJo SINE/Alu 1 293**

**128186746 128186884 C L2 LINE/L2 2843 2705**

**128187323 128187574 C MLT1H LTR/MaLR 549 339**

**128187575 128187755 + MER3 DNA/MER1_type 1 148**

**128187756 128188059 C AluSx SINE/Alu 301 1**

**128188060 128188111 + MER3 DNA/MER1_type 149 207**

**128188112 128188422 C MLT1H LTR/MaLR 338 19**

**__________________________________________________________________________________**

**AluYa5_9_172c 130266781-130267094 C_INTER_RMD_M_DISRUPTED**

**130264972 130265078 + L1MC4a LINE/L1 5680 5774**

**130265079 130265382 + AluJb SINE/Alu 1 306**

**130265383 130265436 + L1MC4a LINE/L1 5775 5829**

**130265437 130265700 + AluJb SINE/Alu 1 293**

**130265935 130266236 C AluSx SINE/Alu 310 9**

**130266257 130266553 C AluSg SINE/Alu 297 1**

**130266597 130266778 C AluSg/x SINE/Alu 303 121 (R2)**

**130266781 130267094 C AluYa5 SINE/Alu 310 1 ( AluYa5_9_172c ) (R1)**

**130267561 130267855 + AluSc SINE/Alu 1 295**

**130267960 130268270 + AluSx SINE/Alu 3 311**

**130268284 130268587 + AluSx SINE/Alu 1 303**

**130268589 130268668 C L1ME3A LINE/L1 6139 6060**

**130268745 130268766 + (TA)n Simple_repeat 2 23**

**130268796 130269108 + AluY SINE/Alu 1 311**

**130269115 130269157 + (TA)n Simple_repeat 1 43**

**130269158 130269490 C MER7A DNA/MER2_type 344 1**

**Ortholog in Chimp 128296318-128296391 Minus Nscore 0.00**

**128294519 128294615 + L1MC LINE/L1 5680 5773**

**128294616 128294919 + AluJb SINE/Alu 1 306**

**128294920 128294953 + L1MC LINE/L1 5777 5810**

**128294974 128295237 + AluJb SINE/Alu 1 293**

**128295472 128295773 C AluSx SINE/Alu 310 9**

**128295794 128296093 C AluSg SINE/Alu 300 1**

**128296131 128296438 C AluSx SINE/Alu 309 1 (R12)**

**128296906 128296969 + Alu SINE/Alu 2 67**

**128297208 128297471 + AluSc SINE/Alu 37 300**

**128297576 128297879 + AluSx SINE/Alu 3 307**

**128297906 128298206 + AluSx SINE/Alu 1 304**

**128298208 128298287 C L1ME3A LINE/L1 6139 6060**

**128298364 128298383 + (TA)n Simple_repeat 2 21**

**128298413 128298721 + AluY SINE/Alu 1 309**

**128298722 128298752 + (TA)n Simple_repeat 1 31**

**128298753 128299085 C MER7A DNA/MER2_type 344 1**

**__________________________________________________________________________________**

**AluYa5_10_48c 44848456-44848764 C_INTER_RMD_M_DISRUPTED**

**44844448 44844506 + AT_rich Low_complexity 1 59**

**44844507 44844635 + (TA)n Simple_repeat 2 138**

**44844665 44844922 + (TA)n Simple_repeat 1 258**

**44845109 44846249 C L1MEb LINE/L1 1934 688**

**44846250 44846275 + AT_rich Low_complexity 1 26**

**44846310 44846480 C AluJb SINE/Alu 304 125**

**44846481 44846504 + (TTTG)n Simple_repeat 1 24**

**44846505 44846539 C AluJb SINE/Alu 124 89**

**44847074 44848140 C MER11B LTR/ERVK 1222 3**

**44848275 44848451 C AluY SINE/Alu 303 127 (R2)**

**44848456 44848764 C AluYa5 SINE/Alu 309 1 ( AluYa5_10_48c ) (R1)**

**44849122 44849420 C AluJo SINE/Alu 300 1**

**44849664 44850229 + MLT1E LTR/MaLR 1 593**

**44850882 44850947 + CT-rich Low_complexity 4 69**

**44852564 44853335 + L1PA12 LINE/L1 5372 6167**

**44853715 44853892 + L1PB1 LINE/L1 2 182**

**44853884 44856034 + L1PB1 LINE/L1 4780 6858**

**Ortholog in Chimp 45299185-45299254 Minus Nscore 0.00**

**45295195 45295361 + (TA)n Simple_repeat 2 178**

**45295342 45295523 + (TTATA)n Simple_repeat 4 174**

**45295472 45295643 + (TA)n Simple_repeat 2 176**

**45295588 45295813 + (TTATA)n Simple_repeat 1 227**

**45295843 45296984 C L1MEb LINE/L1 1934 688**

**45296985 45297010 + AT_rich Low_complexity 1 26**

**45297045 45297216 C AluJb SINE/Alu 305 126**

**45297217 45297239 + (TTTG)n Simple_repeat 2 24**

**45297240 45297276 C AluJb SINE/Alu 125 88**

**45297810 45298868 C MER11C LTR/ERVK 1057 3**

**45299003 45299303 C AluY SINE/Alu 300 1 (R12)**

**45299660 45299960 C AluJo SINE/Alu 302 1**

**45300205 45300770 + MLT1E LTR/MaLR 1 593**

**45301423 45301488 + CT-rich Low_complexity 4 69**

**45303097 45303873 + L1PA12 LINE/L1 5367 6167**

**45304253 45304430 + L1PB1 LINE/L1 2 182**

**45304422 45306584 + L1PB1 LINE/L1 4780 6867**

**__________________________________________________________________________________**

**AluYa5_10_58 51924521-51924827 C_INTER_RMD_M_DISRUPTED**

**51917734 51917751 C AluSx SINE/Alu 312 284**

**51917798 51918080 C AluSx SINE/Alu 283 2**

**51919460 51919769 + AluSg SINE/Alu 1 310**

**51919795 51919967 + MIR3 SINE/MIR 5 167**

**51920134 51920264 + MER20B DNA/MER1_type 31 164**

**51920304 51920437 + MIRb SINE/MIR 73 202**

**51920441 51920554 + MIRb SINE/MIR 56 186**

**51920831 51921139 + AluJo SINE/Alu 1 301**

**51921314 51922670 + L1PA12 LINE/L1 3368 4728**

**51922671 51922826 + LTR9 LTR/ERV1 457 612**

**51922827 51923569 + L1PA12 LINE/L1 4729 5473**

**51923570 51923863 C AluSg SINE/Alu 292 1**

**51923864 51924508 + L1PA12 LINE/L1 5476 6108**

**51924521 51924827 + AluYa5 SINE/Alu 1 307 ( AluYa5_10_58 ) (R1)**

**51924837 51925037 + AluY SINE/Alu 115 311 (R2)**

**51925324 51925645 + L1MC4a LINE/L1 5636 6017**

**51925744 51925783 + AT_rich Low_complexity 1 40**

**51926489 51926644 + MIRb SINE/MIR 39 191**

**51927277 51927571 + AluSq SINE/Alu 1 295**

**51928073 51928779 C L1MB3 LINE/L1 6180 5456**

**51929008 51929143 + MIRb SINE/MIR 112 248**

**51929653 51929969 + AluSg SINE/Alu 1 316**

**51930294 51930375 C MIR SINE/MIR 232 146**

**Ortholog in Chimp 49267211-49267298 Plus Nscore 0.00**

**49260181 49260294 + MIRb SINE/MIR 56 186**

**49260574 49260896 + AluJo SINE/Alu 1 312**

**49261059 49262413 + L1M2 LINE/L1 3370 4724**

**49262414 49262569 + LTR9 LTR/ERV1 457 612**

**49262570 49263024 + L1M2 LINE/L1 4725 5179**

**49266026 49266212 + L1PA12 LINE/L1 5287 5475**

**49266213 49266506 C AluSg SINE/Alu 292 1**

**49266507 49267155 + L1PA12 LINE/L1 5476 6111**

**49267165 49267495 + AluY SINE/Alu 1 311 (R12)**

**49267785 49268030 + L1MC4a LINE/L1 5636 5906**

**49268205 49268244 + AT_rich Low_complexity 1 40**

**49268951 49269104 + MIRb SINE/MIR 39 191**

**49269737 49270030 + AluSq SINE/Alu 1 294**

**49270532 49271233 C L1MB3 LINE/L1 6180 5456**

**49271399 49271597 + MIRb SINE/MIR 25 248**

**49272107 49272423 + AluSg SINE/Alu 1 316**

**49272748 49272829 C MIR SINE/MIR 232 146**

**__________________________________________________________________________________**

**AluYa5_10_88c 69841981-69842281 C_INTER_RMD_M_DISRUPTED**

**69838819 69839004 C L1PA14 LINE/L1 6150 5962**

**69839005 69839324 C AluJo SINE/Alu 301 1**

**69839726 69840033 + AluSg SINE/Alu 1 304**

**69840044 69840350 + AluJo SINE/Alu 1 300**

**69840428 69840465 C L2 LINE/L2 3165 3128**

**69840627 69840695 + MER112 DNA/MER1_type 8 71**

**69840696 69841019 C AluSx SINE/Alu 312 1**

**69841020 69841177 + MER112 DNA/MER1_type 72 239**

**69841265 69841569 + AluSg SINE/Alu 1 305**

**69841663 69841973 C AluSq SINE/Alu 310 1 (R2)**

**69841981 69842281 C AluYa5 SINE/Alu 301 1 ( AluYa5_10_88c ) (R1)**

**69842755 69842774 + (TTG)n Simple_repeat 2 21**

**69842775 69843056 C AluSq SINE/Alu 290 11**

**69843114 69843138 + (A)n Simple_repeat 1 25**

**69843470 69843764 + AluY SINE/Alu 1 300**

**69843838 69843882 + AT_rich Low_complexity 1 45**

**69844018 69844038 + AT_rich Low_complexity 1 21**

**69845033 69845338 C AluSp SINE/Alu 307 1**

**69845418 69845712 C AluSc SINE/Alu 311 1**

**69845763 69845918 + AluJb SINE/Alu 107 263**

**69845949 69846225 + AluJb SINE/Alu 1 279**

**69846290 69846395 + FLAM_C SINE/Alu 2 107**

**Ortholog in Chimp 67437573-67437634 Minus Nscore 0.00**

**67434484 67434672 C L1PA14 LINE/L1 6150 5959**

**67434679 67434999 C AluJo SINE/Alu 302 1**

**67435401 67435703 + AluSg SINE/Alu 1 299**

**67435713 67436010 + AluJo SINE/Alu 1 302**

**67436088 67436125 C L2 LINE/L2 3165 3128**

**67436287 67436355 + MER112 DNA/MER1_type 8 71**

**67436356 67436677 C AluSx SINE/Alu 312 1**

**67436678 67436835 + MER112 DNA/MER1_type 72 239**

**67436923 67437231 + AluSg SINE/Alu 1 309**

**67437324 67437623 C AluSq SINE/Alu 299 1 (R12)**

**67438097 67438116 + (TTG)n Simple_repeat 2 21**

**67438117 67438398 C AluSq SINE/Alu 290 11**

**67438457 67438482 + (A)n Simple_repeat 1 26**

**67438815 67439110 + AluY SINE/Alu 1 298**

**67439183 67439227 + AT_rich Low_complexity 1 45**

**67439362 67439382 + AT_rich Low_complexity 1 21**

**67440375 67440679 C AluSp SINE/Alu 306 1**

**67440759 67441053 C AluSc SINE/Alu 311 1**

**67441124 67441255 + AluJb SINE/Alu 129 263**

**67441286 67441562 + AluJb SINE/Alu 1 279**

**67441627 67441732 + FLAM_C SINE/Alu 2 107**

**__________________________________________________________________________________**

**AluYa5_11_127c 92587826-92588133 C_INTER_RMD_M_DISRUPTED**

**92580757 92582350 + L1M4c LINE/L1 4 2521**

**92582351 92583218 + MER54A LTR/ERVL 3 899**

**92583219 92584773 + L1M4c LINE/L1 2488 4159**

**92584785 92585461 + L1MA9 LINE/L1 5595 6310**

**92585851 92586148 + AluY SINE/Alu 1 302**

**92587468 92587664 C MIRb SINE/MIR 251 50**

**92587670 92587825 C AluSg/x SINE/Alu 299 134 (R2)**

**92587826 92588133 C AluYa5 SINE/Alu 306 1 ( AluYa5_11_127c ) (R1)**

**92588277 92588297 + (T)n Simple_repeat 1 21**

**92590772 92591166 C L2 LINE/L2 2862 2433**

**92591419 92591714 + AluSx SINE/Alu 1 299**

**92593473 92593512 + AT_rich Low_complexity 1 40**

**92593928 92593999 C MIRb SINE/MIR 250 175**

**Ortholog in Chimp 91745178-91745251 Minus Nscore 0.00**

**91738096 91739689 + L1M4c LINE/L1 4 2521**

**91739690 91740557 + MER54A LTR/ERVL 3 899**

**91740558 91742113 + L1M4c LINE/L1 2488 4159**

**91742125 91742801 + L1MA9 LINE/L1 5595 6310**

**91743191 91743491 + AluY SINE/Alu 1 305**

**91744817 91745013 C MIRb SINE/MIR 251 50**

**91745019 91745300 C AluSg SINE/Alu 296 1 (R12)**

**91747937 91748331 C L2 LINE/L2 2862 2433**

**91748584 91748880 + AluSx SINE/Alu 1 300**

**91750640 91750679 + AT_rich Low_complexity 1 40**

**91751095 91751166 C MIRb SINE/MIR 250 175**

**__________________________________________________________________________________**

**AluYa5_11_160c 113769917-113770106 C_INTER_RMD_M_DISRUPTED**

**113765760 113766062 C AluSx SINE/Alu 303 1**

**113766285 113766595 + AluSx SINE/Alu 1 311**

**113766608 113766731 + FLAM_C SINE/Alu 1 125**

**113766732 113766759 + AT_rich Low_complexity 1 28**

**113766829 113767134 + AluSq SINE/Alu 5 313**

**113767143 113767271 + AluJ SINE/Alu 3 131**

**113767273 113767536 C L1ME4a LINE/L1 6104 5804**

**113767725 113768042 + L1ME3B LINE/L1 5880 6184**

**113768053 113768187 + AluJb SINE/Alu 1 125**

**113768188 113768491 + AluSx SINE/Alu 1 312**

**113768492 113768687 + AluJb SINE/Alu 126 305**

**113768862 113768958 + MER58 DNA/MER1_type 7 104**

**113768970 113769845 + Cheshire DNA/MER1_type 1514 2420**

**113769846 113769899 + MIRb SINE/MIR 168 220**

**113769917 113770106 C AluYa5 SINE/Alu 294 106 ( AluYa5_11_160c )**

**113770107 113770243 + (TTTTC)n Simple_repeat 2 139**

**113770244 113770525 C AluSx SINE/Alu 283 2**

**113770526 113770639 C AluY SINE/Alu 114 1**

**113770650 113770797 C L1ME4a LINE/L1 5775 5638**

**113770981 113771088 C L2 LINE/L2 3378 3240**

**113771841 113771986 C FRAM SINE/Alu 152 4**

**113772169 113772299 + MER103 DNA 7 153**

**113773920 113774092 C L3 LINE/CR1 4452 4265**

**113774137 113774318 + MER105 DNA 4 204**

**113774321 113774458 + MIR SINE/MIR 63 218**

**113774443 113774542 + L2 LINE/L2 3306 3404**

**113774560 113774611 + MIRb SINE/MIR 214 268**

**113774610 113774740 C L3 LINE/CR1 4108 3970**

**Ortholog in Chimp 113196008-113196149 Minus Nscore 0.00**

**113191809 113192111 C AluSx SINE/Alu 303 1**

**113192334 113192645 + AluSx SINE/Alu 1 312**

**113192659 113192785 + FLAM_C SINE/Alu 1 128**

**113192786 113192806 + AT_rich Low_complexity 1 21**

**113192876 113193182 + AluSq SINE/Alu 5 313**

**113193191 113193320 + AluJ SINE/Alu 3 134**

**113193321 113193579 C L1ME4a LINE/L1 6104 5804**

**113193768 113194083 + L1ME3B LINE/L1 5880 6184**

**113194094 113194228 + AluJb SINE/Alu 1 125**

**113194229 113194526 + AluSx SINE/Alu 1 306**

**113194527 113194729 + AluJb SINE/Alu 126 312**

**113194905 113195001 + MER58 DNA/MER1_type 7 104**

**113195013 113195887 + Cheshire DNA/MER1_type 1514 2420**

**113195888 113195941 + MIRb SINE/MIR 168 220**

**113195959 113196148 C AluY SINE/Alu 294 106 (OCCUPIED)**

**113196149 113196176 + (TTTTC)n Simple_repeat 2 29**

**113196177 113196459 C AluSx SINE/Alu 284 2**

**113196460 113196550 C AluY SINE/Alu 105 24**

**113196763 113196913 C L1ME4a LINE/L1 5778 5638**

**113197097 113197204 C L2 LINE/L2 3378 3240**

**113197960 113198105 C FRAM SINE/Alu 152 4**

**113198288 113198418 + MER103 DNA 7 153**

**113200050 113200222 C L3 LINE/CR1 4452 4265**

**113200267 113200449 + MER105 DNA 4 204**

**113200452 113200591 + MIRb SINE/MIR 70 220**

**113200601 113200678 + L2 LINE/L2 3290 3363**

**113200689 113200747 + MIRb SINE/MIR 207 268**

**113200746 113200876 C L3 LINE/CR1 4108 3970**

**__________________________________________________________________________________**

**AluYa5_11_163c 118389383-118389680 C_INTER_RMD_M_DISRUPTED**

**118385362 118385630 C LTR75 LTR/ERVL 305 27**

**118385690 118385743 + L1MC4a LINE/L1 6476 6529**

**118385784 118386089 + AluJb SINE/Alu 1 300**

**118386094 118386224 + L1MC4 LINE/L1 7526 7672**

**118386440 118386607 + L1MC4 LINE/L1 7853 8042**

**118386763 118387066 C MER1B DNA/MER1_type 338 4**

**118387289 118387590 C AluSx SINE/Alu 305 1**

**118388666 118388789 + L2 LINE/L2 3030 3171**

**118388942 118388970 + MIRm SINE/MIR 154 182**

**118389220 118389382 C AluSc SINE/Alu 294 132 (R2)**

**118389383 118389680 C AluYa5 SINE/Alu 300 3 ( AluYa5_11_163c ) (R1)**

**118389724 118390213 + L1ME1 LINE/L1 5237 5861**

**118390223 118390504 + AluSg SINE/Alu 1 292**

**118391070 118391206 + FLAM_C SINE/Alu 7 137**

**118391926 118392199 + AluSx SINE/Alu 1 276**

**118392216 118392335 + AluJo/FLAM SINE/Alu 1 132**

**118392336 118392454 C FLAM_A SINE/Alu 116 1**

**118392566 118392823 C AluSx SINE/Alu 287 19**

**118392917 118393107 + Charlie4 DNA/MER1_type 1751 1958**

**Ortholog in Chimp 117890398-117890468 Minus Nscore 0.00**

**117886361 117886629 C LTR75 LTR/ERVL 305 27**

**117886689 117886742 + L1MC4a LINE/L1 6476 6529**

**117886783 117887090 + AluJb SINE/Alu 1 302**

**117887095 117887225 + L1MC4 LINE/L1 7526 7672**

**117887371 117887609 + L1MC4 LINE/L1 7768 8042**

**117887765 117888068 C MER1B DNA/MER1_type 338 4**

**117888291 117888595 C AluSx SINE/Alu 308 1**

**117889674 117889797 + L2 LINE/L2 3030 3171**

**117889950 117889982 + MIRm SINE/MIR 154 187**

**117890228 117890513 C AluSc SINE/Alu 297 3 (R12)**

**117890514 117890536 + AT_rich Low_complexity 1 23**

**117890554 117891043 + L1ME1 LINE/L1 5235 5861**

**117891053 117891334 + AluSg SINE/Alu 1 292**

**117891904 117892017 + FLAM_C SINE/Alu 7 120**

**117892763 117893036 + AluSx SINE/Alu 1 276**

**117893053 117893172 + AluJo/FLAM SINE/Alu 1 132**

**117893173 117893291 C FLAM_A SINE/Alu 116 1**

**117893405 117893662 C AluSx SINE/Alu 287 19**

**117893756 117893946 + Charlie4 DNA/MER1_type 1751 1958**

**__________________________________________________________________________________**

**AluYa5_12_138c 85700080-85700386 C_INTER_RMD_M_DISRUPTED**

**85696961 85697079 C MIRb SINE/MIR 119 10**

**85697225 85697511 + AluJo SINE/Alu 5 284**

**85697512 85697537 + (TAA)n Simple_repeat 2 27**

**85697540 85697838 + AluY SINE/Alu 1 299**

**85697839 85697861 + AT_rich Low_complexity 1 23**

**85698088 85698108 + AT_rich Low_complexity 1 21**

**85698157 85698258 C L1PB4 LINE/L1 6156 6083**

**85698259 85698639 + MSTA LTR/MaLR 1 428**

**85698640 85698920 C L1PB4 LINE/L1 6082 5781**

**85698921 85698953 + (TG)n Simple_repeat 2 34**

**85698954 85699269 C L1PB4 LINE/L1 5780 5450**

**85699471 85699592 C L2 LINE/L2 3419 3295**

**85699895 85700079 C AluY SINE/Alu 305 124 (R2)**

**85700080 85700386 C AluYa5 SINE/Alu 309 3 ( AluYa5_12_138c ) (R1)**

**85700503 85700889 + MLT1B LTR/MaLR 1 390**

**85703474 85703518 + AT_rich Low_complexity 1 45**

**85703687 85703718 + (TATAA)n Simple_repeat 1 32**

**85704916 85705419 C L1PB4 LINE/L1 6155 5615**

**85705423 85705648 + L1PB4 LINE/L1 5386 5610**

**85705668 85705786 C AluJb SINE/Alu 288 169**

**85706005 85706028 + AT_rich Low_complexity 1 24**

**85706035 85706108 + (TA)n Simple_repeat 1 76**

**85706438 85706522 + LTR67 LTR/ERVL 460 540**

**85707105 85707199 C MIRb SINE/MIR 268 164**

**85707216 85707358 C MLT1K LTR/MaLR 583 443**

**Ortholog in Chimp 87627487-87627554 Minus Nscore 0.00**

**87623372 87623469 + MIRb SINE/MIR 58 158**

**87624194 87624234 + (CAAAA)n Simple_repeat 5 45**

**87624279 87624316 + AT_rich Low_complexity 1 38**

**87624630 87624917 + AluJo SINE/Alu 5 284**

**87624918 87624937 + (TAA)n Simple_repeat 2 21**

**87624940 87625238 + AluY SINE/Alu 1 299**

**87625239 87625261 + AT_rich Low_complexity 1 23**

**87625490 87625510 + AT_rich Low_complexity 1 21**

**87625559 87625660 C L1PB4 LINE/L1 6156 6083**

**87625661 87626041 + MSTA LTR/MaLR 1 428**

**87626042 87626321 C L1PB4 LINE/L1 6082 5781**

**87626322 87626356 + (TG)n Simple_repeat 2 36**

**87626357 87626672 C L1PB4 LINE/L1 5780 5450**

**87626874 87626995 C L2 LINE/L2 3419 3295**

**87627298 87627605 C AluY SINE/Alu 305 1 (R12)**

**87627720 87628106 + MLT1B LTR/MaLR 1 390**

**87630928 87630959 + (TATAA)n Simple_repeat 1 32**

**87632156 87632661 C L1PB4 LINE/L1 6155 5615**

**87632665 87632890 + L1PB4 LINE/L1 5386 5610**

**87632910 87633028 C AluJb SINE/Alu 288 169**

**87633240 87633270 + AT_rich Low_complexity 1 31**

**87633277 87633350 + (TA)n Simple_repeat 1 74**

**87633438 87633507 + AT_rich Low_complexity 1 70**

**87634401 87634499 C THER1_MD SINE/MIR 273 164**

**87634516 87634664 C MLT1K LTR/MaLR 583 437**

**__________________________________________________________________________________**

**AluYa5_12_182c 120319153-120319450 C_INTER_RMD_M_DISRUPTED**

**120312104 120312284 + MIR3 SINE/MIR 5 195**

**120312301 120312596 C AluSq SINE/Alu 299 1**

**120312716 120313045 C AluJo SINE/Alu 300 1**

**120313049 120313121 + MSTB1 LTR/MaLR 358 431**

**120313431 120313453 + (TTTA)n Simple_repeat 2 24**

**120313456 120313741 C AluSg1 SINE/Alu 290 1**

**120313742 120314046 C AluSx SINE/Alu 307 1**

**120314128 120314188 C MIR SINE/MIR 167 105**

**120314735 120314846 + L1MC5 LINE/L1 7803 7913**

**120314979 120315284 C AluY SINE/Alu 305 1 (R2)**

**120315293 120315589 C AluY SINE/Alu 300 1**

**120315660 120315925 C AluJb SINE/Alu 288 3**

**120315935 120316160 C AluJb SINE/Alu 290 64**

**120316162 120316663 C MER51E LTR/ERV1 640 115**

**120316670 120316735 C AluJb SINE/Alu 66 1**

**120316746 120317032 C AluJo SINE/Alu 285 1**

**120317362 120317667 + AluY SINE/Alu 1 306**

**120317914 120318208 + AluSx SINE/Alu 5 299**

**120318217 120318519 + AluSx SINE/Alu 1 306**

**120318523 120318588 C L2 LINE/L2 3366 3292**

**120318722 120318843 C L1MB7 LINE/L1 6175 6054**

**120318844 120319147 C AluJo SINE/Alu 312 1**

**120319153 120319450 C AluYa5 SINE/Alu 309 1 ( AluYa5_12_182c ) (R1)**

**120319451 120319661 C L1MB7 LINE/L1 6053 5873**

**120319662 120319988 C AluSx SINE/Alu 312 1**

**120319989 120320277 C L1MB7 LINE/L1 5872 5592**

**120320278 120320570 + AluY SINE/Alu 1 294**

**120320571 120320789 C L1MB7 LINE/L1 5591 5371**

**120321264 120321546 + AluSq SINE/Alu 1 297**

**120323541 120323560 + (A)n Simple_repeat 1 20**

**120323561 120323662 C L2 LINE/L2 3213 3106**

**120324064 120324109 + L3 LINE/CR1 1989 2037**

**120324342 120324467 + AluSq/x SINE/Alu 1 126**

**120324624 120324651 + (GTTTG)n Simple_repeat 2 29**

**120325301 120325612 + AluY SINE/Alu 1 310**

**120326195 120326492 C AluSx SINE/Alu 307 1**

**Ortholog in Chimp 123029946-123029987 Minus Nscore 0.00**

**123022922 123023222 + AluJb SINE/Alu 1 299**

**123023321 123023630 C AluSg SINE/Alu 308 1**

**123024069 123024365 C AluSx SINE/Alu 296 1**

**123024786 123025094 C AluSq SINE/Alu 313 5**

**123025703 123025992 + AluJo SINE/Alu 1 288**

**123026418 123026562 C AluSp SINE/Alu 147 1**

**123026802 123026979 + MIR3 SINE/MIR 5 195**

**123026996 123027308 C AluSq SINE/Alu 313 1**

**123027428 123027757 C AluJo SINE/Alu 300 1**

**123027761 123027833 + MSTB1 LTR/MaLR 358 431**

**123028143 123028165 + (TTTA)n Simple_repeat 2 24**

**123028168 123028453 C AluSg SINE/Alu 291 1**

**123028454 123028765 C AluSx SINE/Alu 311 1**

**123028846 123028907 C MIR_Mars SINE/MIR 168 108**

**123029451 123029562 + L1MC5 LINE/L1 7803 7913**

**123029696 123029995 C AluY SINE/Alu 311 1 (R12)**

**123030000 123030191 C L1MB7 LINE/L1 6058 5866**

**123030192 123030334 C AluJb SINE/Alu 312 177**

**123030969 123031226 + AluY SINE/Alu 37 295**

**123031231 123031444 C L1MB7 LINE/L1 5597 5371**

**123031922 123032210 + AluSq SINE/Alu 1 303**

**123033324 123033548 + AluSq SINE/Alu 77 306**

**123035555 123035656 C L2 LINE/L2 3213 3106**

**123036333 123036458 + AluSq/x SINE/Alu 1 126**

**__________________________________________________________________________________**

**AluYa5_14_101 90421369-90421678 C_INTER_RMD_M_DISRUPTED**

**90416654 90416928 C L1M4c LINE/L1 2890 2611**

**90416929 90417230 C AluJb SINE/Alu 302 1**

**90417231 90417265 C L1M4c LINE/L1 2610 2586**

**90417266 90417564 + AluSx SINE/Alu 1 297**

**90417565 90417594 C L1M4c LINE/L1 2585 2559**

**90417595 90417897 C AluSq SINE/Alu 303 1**

**90417898 90418911 C L1M4c LINE/L1 2558 1463**

**90418912 90419176 C AluSx SINE/Alu 293 19**

**90419177 90420379 C L1M4c LINE/L1 1462 216**

**90420380 90420608 + MER46A DNA/MER2_type 2 235**

**90420609 90420803 C L1M4c LINE/L1 215 14**

**90420807 90421000 C L1M5 LINE/L1 5769 5565**

**90421068 90421366 + AluJb SINE/Alu 1 299**

**90421369 90421678 + AluYa5 SINE/Alu 2 310 ( AluYa5_14_101 ) (R1)**

**90421684 90421868 + AluY SINE/Alu 127 311 (R2)**

**90422168 90422242 + (CATATA)n Simple_repeat 5 78**

**90422520 90422877 + MLT1B LTR/MaLR 30 390**

**90423285 90423566 C AluJb SINE/Alu 294 21**

**90423579 90423688 C L1MC5 LINE/L1 7715 7609**

**90423737 90423771 + AT_rich Low_complexity 1 35**

**90423772 90424068 + AluJo SINE/Alu 1 308**

**90424090 90424307 C L1MC5 LINE/L1 7571 7330**

**90424731 90425018 + L1M5 LINE/L1 4976 5253**

**90425048 90425108 + L1P LINE/L1 5294 5357**

**90425109 90425414 + AluSx SINE/Alu 1 306**

**90425415 90425524 + L1M5 LINE/L1 5347 5452**

**90425545 90425775 + L1M5 LINE/L1 5519 5777**

**90426983 90427344 + L2 LINE/L2 3003 3418**

**90428093 90428398 C AluSx SINE/Alu 301 1**

**Ortholog in Chimp 91038491-91038568 Plus Nscore 0.00**

**91033588 91033862 C L1M4c LINE/L1 2890 2611**

**91033863 91034160 C AluJb SINE/Alu 298 1**

**91034161 91034195 C L1M4c LINE/L1 2610 2584**

**91034632 91034664 C L1M4c LINE/L1 2588 2559**

**91034665 91034971 C AluSq SINE/Alu 307 1**

**91034972 91035980 C L1M4c LINE/L1 2558 1463**

**91035981 91036248 C AluSx SINE/Alu 296 19**

**91036249 91037454 C L1M4c LINE/L1 1462 216**

**91037455 91037683 + MER46A DNA/MER2_type 2 235**

**91037684 91037878 C L1M4c LINE/L1 215 14**

**91037882 91038098 C L1MD LINE/L1 5769 5540**

**91038143 91038439 + AluJb SINE/Alu 1 298**

**91038442 91038753 + AluY SINE/Alu 2 310 (R12)**

**91039042 91039107 + (TA)n Simple_repeat 1 67**

**91039370 91039743 + MLT1B LTR/MaLR 15 390**

**91040150 91040430 C AluJb SINE/Alu 294 21**

**91040432 91040599 C L1MC5 LINE/L1 7726 7564**

**91040601 91040635 + AT_rich Low_complexity 1 35**

**91040636 91040931 + AluJo SINE/Alu 1 299**

**91040953 91041168 C L1MC5 LINE/L1 7571 7330**

**91041592 91041803 + L1M5 LINE/L1 4976 5191**

**91041909 91041969 + L1P LINE/L1 5294 5357**

**91041970 91042254 + AluSx SINE/Alu 1 302**

**91042255 91042364 + L1M5 LINE/L1 5347 5452**

**91042385 91042617 + L1M5 LINE/L1 5519 5777**

**91043823 91044184 + L2 LINE/L2 3003 3418**

**91044933 91045235 C AluSx SINE/Alu 298 1**

**__________________________________________________________________________________**

**AluYa5_15_89 81072341-81072641 C_INTER_RMD_M_DISRUPTED**

**81068694 81069875 + L1M4 LINE/L1 -261 953**

**81069876 81070000 + FLAM_C SINE/Alu 1 130**

**81070006 81070319 + AluY SINE/Alu 1 311**

**81070320 81071186 + L1M4 LINE/L1 957 2241**

**81071194 81071488 + L1MA9 LINE/L1 5989 6310**

**81071561 81071851 + AluSp SINE/Alu 1 292**

**81071866 81071989 + L1M4 LINE/L1 2346 2467**

**81071990 81072290 + AluSx SINE/Alu 1 299**

**81072291 81072340 + L1M4 LINE/L1 2468 2517**

**81072341 81072641 + AluYa5 SINE/Alu 1 301 ( AluYa5_15_89 )**

**81072656 81072789 + FLAM_C SINE/Alu 1 132**

**81072842 81073110 + L1M4 LINE/L1 2255 2662**

**81073111 81073416 + AluSx SINE/Alu 1 306**

**81073417 81073735 + L1M4 LINE/L1 2504 2811**

**81073717 81074558 C L1M4 LINE/L1 864 1**

**81074591 81075033 + L1M4 LINE/L1 2800 3270**

**81075034 81075318 + AluJb SINE/Alu 12 295**

**81075319 81075583 + L1M4 LINE/L1 3271 3586**

**81075584 81075883 + AluSp SINE/Alu 1 301**

**81075884 81076480 + L1M4 LINE/L1 3587 4221**

**81076481 81076793 + AluSx SINE/Alu 1 312**

**81076794 81077277 + L1M4 LINE/L1 4222 4796**

**81077281 81077499 + AluJo SINE/Alu 1 211**

**81077513 81077720 + L1M5 LINE/L1 3028 3234**

**81077758 81077870 + AluY SINE/Alu 24 132**

**81077922 81078020 + L1M5 LINE/L1 3353 3451**

**81078021 81078326 + AluSx SINE/Alu 1 307**

**81078327 81078360 + L1M5 LINE/L1 3452 3479**

**81078361 81078687 + AluSg SINE/Alu 1 328**

**81078688 81079163 + L1M5 LINE/L1 3480 3950**

**81079186 81079464 C AluJb SINE/Alu 308 2**

**Ortholog in Chimp 80503798-80503860 Plus Nscore 0.00**

**80500159 80501344 + L1M4 LINE/L1 363 1587**

**80501345 80501469 + FLAM_C SINE/Alu 1 130**

**80501475 80501787 + AluY SINE/Alu 1 311**

**80501788 80503023 + L1M4 LINE/L1 1544 2950**

**80503024 80503315 + AluSp SINE/Alu 1 293**

**80503316 80503454 + L1M4 LINE/L1 2951 3091**

**80503455 80503762 + AluSx SINE/Alu 1 303**

**80503763 80503816 + L1M4 LINE/L1 3092 3145**

**80503817 80503949 + FLAM_C SINE/Alu 1 133**

**80503950 80504253 + L1M4 LINE/L1 3146 3553**

**80504254 80504559 + AluSx SINE/Alu 1 306**

**80504560 80504877 + L1M4 LINE/L1 3128 3692**

**80504859 80505707 C L1M4 LINE/L1 864 1**

**80505745 80506187 + L1M4 LINE/L1 2564 3044**

**80506188 80506475 + AluJb SINE/Alu 12 298**

**80506476 80506735 + L1M4 LINE/L1 3905 4205**

**80506736 80507047 + AluSp SINE/Alu 1 313**

**80507048 80507649 + L1M4 LINE/L1 4206 4844**

**80507650 80507962 + AluSx SINE/Alu 1 312**

**80507963 80508436 + L1M4 LINE/L1 4845 5419**

**80508440 80508655 + AluJo SINE/Alu 1 216**

**80508664 80508869 + L1M5 LINE/L1 3028 3234**

**80508907 80509013 + AluY SINE/Alu 24 133**

**80509019 80509042 + (TAAAA)n Simple_repeat 2 25**

**80509078 80509174 + L1M5 LINE/L1 3353 3451**

**80509175 80509475 + AluSx SINE/Alu 1 303**

**80509476 80509511 + L1M5 LINE/L1 3452 3479**

**80509512 80509840 + AluSg SINE/Alu 1 328**

**80509841 80510315 + L1M5 LINE/L1 3480 3950**

**80510337 80510637 C AluJb SINE/Alu 308 2**

**80510857 80511113 C GSAT Satellite/centr 278 1**

**__________________________________________________________________________________**

**AluYa5_16_9c 4765808-4766116 C_INTER_RMD_M_DISRUPTED**

**4762298 4762421 + MER20 DNA/MER1_type 59 182**

**4762423 4762563 + AluJo SINE/Alu 3 142**

**4762565 4762869 + AluJb SINE/Alu 1 299**

**4762876 4762916 + MER41B LTR/ERV1 481 521**

**4762917 4763227 C AluY SINE/Alu 311 1**

**4763228 4763247 + MER41B LTR/ERV1 522 534**

**4763248 4763526 C AluSx SINE/Alu 289 13**

**4763546 4763563 + polypurine Low_complexity 2 19**

**4763564 4763696 + AluJb SINE/Alu 1 135**

**4763697 4763994 + AluSq SINE/Alu 1 299**

**4763995 4764171 + AluJb SINE/Alu 136 310**

**4764172 4764196 + polypurine Low_complexity 20 42**

**4764241 4764324 C L1MC LINE/L1 5654 5570**

**4764335 4764355 + AT_rich Low_complexity 1 21**

**4764356 4765052 C SVA Other 1386 691**

**4765297 4765475 C L2 LINE/L2 3326 3118**

**4765619 4765633 C Alu SINE/Alu 312 285 (R2)**

**4765658 4765807 C AluSg/x SINE/Alu 284 135**

**4765808 4766116 C AluYa5 SINE/Alu 308 1 ( AluYa5_16_9c ) (R1)**

**4766236 4766367 C MIRb SINE/MIR 230 101**

**4766707 4766988 + AluSx SINE/Alu 3 285**

**4766989 4767299 + AluSq SINE/Alu 1 313**

**4767370 4767628 + AluJo SINE/Alu 25 281**

**4768289 4768357 + L2 LINE/L2 3336 3419**

**4768409 4768486 + MER2B DNA/MER2_type 154 329**

**4768521 4768808 + AluSx SINE/Alu 1 289**

**4768809 4768851 + AT_rich Low_complexity 1 43**

**4768860 4769168 + AluSg SINE/Alu 1 309**

**4769289 4769379 + L2 LINE/L2 3303 3389**

**4769456 4769578 C MIR SINE/MIR 177 42**

**Ortholog in Chimp 4928903-4928981 Minus Nscore 0.00**

**4925679 4925801 + MER20 DNA/MER1_type 59 182**

**4925803 4925943 + AluJo SINE/Alu 3 142**

**4925945 4926260 + AluJb SINE/Alu 1 298**

**4926267 4926309 + MER41B LTR/ERV1 481 523**

**4926310 4926620 C AluY SINE/Alu 311 1**

**4926621 4926640 + MER41B LTR/ERV1 524 534**

**4926641 4926875 C AluSx SINE/Alu 289 55**

**4927411 4927522 + MER20 DNA/MER1_type 62 179**

**4927525 4927827 + AluSq SINE/Alu 1 303**

**4927828 4927993 + AluJb SINE/Alu 134 299**

**4927997 4928023 + (GAA)n Simple_repeat 1 27**

**4928074 4928157 C L1MC LINE/L1 5654 5570**

**4928388 4928562 C L2 LINE/L2 3326 3122**

**4928725 4929030 C AluSx SINE/Alu 307 1 (R12)**

**4929150 4929281 C MIRb SINE/MIR 230 101**

**4929619 4929902 + AluSx SINE/Alu 1 285**

**4929903 4930212 + AluSq SINE/Alu 1 312**

**4930285 4930542 + AluJo SINE/Alu 26 281**

**4931205 4931273 + L2 LINE/L2 3336 3419**

**4931316 4931402 + MER2B DNA/MER2_type 146 329**

**4931437 4931722 + AluSx SINE/Alu 1 287**

**4931723 4931767 + AT_rich Low_complexity 1 45**

**4931776 4932085 + AluSg SINE/Alu 1 310**

**4932205 4932305 + L2 LINE/L2 3261 3389**

**4932373 4932475 C MIR SINE/MIR 177 77**

**4932878 4933022 + AluJb SINE/Alu 1 145**

**4933073 4933306 C AluJo SINE/Alu 234 1**

**4933308 4933474 + MER58A DNA/MER1_type 45 211**

**4933620 4933897 + AluSx SINE/Alu 5 282**

**4933899 4934190 + AluSg SINE/Alu 2 293**

**4934246 4934541 C L1MB7 LINE/L1 6122 5838**

**4934569 4934867 C AluSp SINE/Alu 301 1**

**4934868 4935170 C AluSx SINE/Alu 305 1**

**4935197 4935523 C L1MB7 LINE/L1 5828 5504**

**4935531 4935825 C AluSq SINE/Alu 294 1**

**4935832 4936132 C AluSq SINE/Alu 296 1**

**__________________________________________________________________________________**

**AluYa5_16_15 10367259-10367569 C_INTER_RMD_M_DISRUPTED**

**10363483 10363929 C LTR77 LTR/ERV1 444 1**

**10364062 10364397 C AluSx SINE/Alu 331 1**

**10364551 10364630 C LTR24C LTR/ERV1 635 556**

**10364640 10364719 + LTR24C LTR/ERV1 260 340**

**10364720 10365006 + AluSx SINE/Alu 1 300**

**10365018 10365260 C THE1-int LTR/MaLR 1518 1275**

**10365269 10365371 C LTR24C LTR/ERV1 558 466**

**10365372 10365656 C AluSq SINE/Alu 308 1**

**10365657 10365721 C LTR24C LTR/ERV1 465 392**

**10365722 10366031 C AluSp SINE/Alu 309 1**

**10366032 10366096 C LTR24C LTR/ERV1 391 331**

**10366097 10366392 C AluSq SINE/Alu 297 2**

**10366465 10366731 + AluSg SINE/Alu 24 293**

**10366737 10367021 C AluJo SINE/Alu 276 1**

**10367089 10367254 C MLT1F2 LTR/MaLR 567 380**

**10367259 10367569 + AluYa5 SINE/Alu 1 310 ( AluYa5_16_15 ) (R1)**

**10367571 10367772 + AluJb SINE/Alu 129 330 (R2)**

**10367773 10367987 C MLT1F2 LTR/MaLR 380 95**

**10367988 10368277 + AluSp SINE/Alu 1 303**

**10368278 10368367 C MLT1F2 LTR/MaLR 98 11**

**10368584 10368892 + AluY SINE/Alu 1 309**

**10368898 10368951 + MER5B DNA/MER1_type 1 54**

**10368959 10369264 + AluJo SINE/Alu 1 300**

**10369265 10369288 + AT_rich Low_complexity 1 24**

**10369293 10369413 + FLAM_C SINE/Alu 2 122**

**10369495 10369535 C L1MA10 LINE/L1 6328 6288**

**10369801 10369890 C MER5C DNA/MER1_type 324 230**

**Ortholog in Chimp 10656683-10656761 Plus Nscore 0.00**

**10652787 10653232 C LTR77 LTR/ERV1 442 1**

**10653373 10653693 C AluSx SINE/Alu 312 1**

**10653848 10653927 C LTR24C LTR/ERV1 635 556**

**10653937 10654016 + LTR24C LTR/ERV1 260 340**

**10654019 10654303 + AluSx SINE/Alu 3 300**

**10654315 10654557 C THE1-int LTR/MaLR 1518 1275**

**10654569 10654668 C LTR24C LTR/ERV1 555 466**

**10654669 10654952 C AluSq SINE/Alu 307 1**

**10654953 10655017 C LTR24C LTR/ERV1 465 406**

**10655018 10655323 C AluSp SINE/Alu 305 1**

**10655324 10655388 C LTR24C LTR/ERV1 405 331**

**10655389 10655674 C AluSq SINE/Alu 297 14**

**10655845 10656108 + AluSg SINE/Alu 32 298**

**10656114 10656399 C AluJo SINE/Alu 276 1**

**10656469 10656633 C MLT1F LTR/MaLR 536 352**

**10656634 10656966 + AluSx SINE/Alu 1 334 (R12)**

**10656967 10657181 C MLT1F LTR/MaLR 351 91**

**10657182 10657461 + AluSp SINE/Alu 1 293**

**10657462 10657551 C MLT1F LTR/MaLR 90 11**

**10657767 10658076 + AluY SINE/Alu 1 310**

**10658086 10658139 + MER5B DNA/MER1_type 1 54**

**10658147 10658454 + AluJo SINE/Alu 1 302**

**10658458 10658478 + AT_rich Low_complexity 1 21**

**10658483 10658603 + FLAM_C SINE/Alu 2 122**

**10658605 10658652 + MER5B DNA/MER1_type 55 102**

**10658685 10658725 C L1MA10 LINE/L1 6328 6288**

**10658991 10659080 C MER5C DNA/MER1_type 324 230**

**__________________________________________________________________________________**

**AluYa5_16_26 21336740-21337047 C_INTER_RMD_M_DISRUPTED**

**21333018 21333447 + LTR48 LTR/ERV1 325 786**

**21334146 21334259 C MER30 DNA/MER1_type 230 108**

**21334260 21334555 C AluSq SINE/Alu 295 1**

**21334559 21334870 C AluSx SINE/Alu 312 1**

**21334871 21334972 C MER30 DNA/MER1_type 107 6**

**21334996 21335294 C AluSg SINE/Alu 300 1**

**21335532 21335841 + AluY SINE/Alu 1 311**

**21336120 21336336 + L1ME2 LINE/L1 5811 6046**

**21336337 21336652 + AluSp SINE/Alu 1 319**

**21336653 21336738 + L1ME2 LINE/L1 6047 6130**

**21336740 21337047 + AluYa5 SINE/Alu 1 310 ( AluYa5_16_26 ) (R1)**

**21337068 21337246 + AluSg/x SINE/Alu 115 306 (R2)**

**21337319 21337559 C AluJb SINE/Alu 251 15**

**21337809 21337883 + G-rich Low_complexity 3 80**

**21337904 21337941 + GA-rich Low_complexity 1 38**

**21338601 21338879 + L1M5 LINE/L1 2667 2964**

**21339371 21339511 C MER113 DNA/MER1_type 428 291**

**21339514 21339824 + AluSg SINE/Alu 1 310**

**21339892 21340198 + AluSg SINE/Alu 1 302**

**Ortholog in Chimp 74284201-74284276 Minus Nscore 0.00**

**74280428 74280728 C AluSg SINE/Alu 301 1**

**74280796 74281106 C AluSg SINE/Alu 310 1**

**74281109 74281249 + MER113 DNA/MER1_type 291 428**

**74281757 74282212 C L1M5 LINE/L1 2945 2474**

**74283271 74283294 + (TCC)n Simple_repeat 1 24**

**74283308 74283436 + C-rich Low_complexity 4 129**

**74283693 74283933 + AluJb SINE/Alu 15 251**

**74284006 74284325 C AluSc SINE/Alu 298 1 (R12)**

**74284327 74284408 C L1ME2 LINE/L1 6130 6047**

**74284409 74284709 C AluSp SINE/Alu 306 1**

**74284710 74284926 C L1ME2 LINE/L1 6046 5811**

**74285195 74285402 C AluSg/x SINE/Alu 304 97**

**74286186 74286490 + AluSg SINE/Alu 1 305**

**74286504 74286605 + MER30 DNA/MER1_type 3 104**

**74286606 74286917 + AluSx SINE/Alu 1 312**

**74286921 74287214 + AluSq SINE/Alu 1 293**

**74287215 74287330 + MER30 DNA/MER1_type 105 227**

**74288030 74288460 C LTR48 LTR/ERV1 785 325**

**__________________________________________________________________________________**

**AluYa5_16_28c 22439502-22439809 C_INTER_RMD_M_DISRUPTED**

**22434205 22434421 C MIRb SINE/MIR 249 27**

**22434475 22434795 C MLT1I LTR/MaLR 399 74**

**22436019 22436079 C MIRb SINE/MIR 103 44**

**22436354 22436660 C AluSg SINE/Alu 302 1**

**22436751 22437034 C AluSg SINE/Alu 285 1**

**22437037 22437177 + MER113 DNA/MER1_type 291 428**

**22437685 22437947 C L1M5 LINE/L1 2945 2667**

**22438607 22438644 + CT-rich Low_complexity 1 38**

**22438665 22438739 + C-rich Low_complexity 3 80**

**22438989 22439229 + AluJb SINE/Alu 15 251**

**22439302 22439480 C AluSg/x SINE/Alu 306 115 (R2)22439502 22439809 C AluYa5 SINE/Alu 310 1 ( AluYa5_16_28c ) (R1)**

**22439811 22439896 C L1ME2 LINE/L1 6130 6047**

**22439897 22440212 C AluSp SINE/Alu 319 1**

**22440213 22440429 C L1ME2 LINE/L1 6046 5811**

**22440707 22441016 C AluY SINE/Alu 311 1**

**22441255 22441553 + AluSg SINE/Alu 1 300**

**22441577 22441678 + MER30 DNA/MER1_type 3 104**

**22441679 22441990 + AluSx SINE/Alu 1 312**

**22441994 22442289 + AluSq SINE/Alu 1 295**

**22442290 22442403 + MER30 DNA/MER1_type 105 227**

**Ortholog in Chimp 28941748-28941809 Plus Nscore 0.00**

**28936047 28936322 + AluSg1 SINE/Alu 1 276**

**28936323 28936366 + (CA)n Simple_repeat 1 44**

**28936383 28936675 + AluSp SINE/Alu 6 299**

**28936678 28936965 + AluSc SINE/Alu 1 288**

**28936970 28937048 + L1MB2 LINE/L1 6082 6161**

**28938275 28938355 + Alu SINE/Alu 217 298**

**28938801 28939232 + LTR48 LTR/ERV1 325 786**

**28939644 28939696 C AluSq/x SINE/Alu 126 73**

**28939985 28940099 C MER30 DNA/MER1_type 230 115**

**28940100 28940401 C AluSq SINE/Alu 301 1**

**28940408 28940723 C AluSx SINE/Alu 312 1**

**28940724 28940801 C MER30 DNA/MER1_type 108 27**

**28941092 28941308 + L1ME2 LINE/L1 5811 6046**

**28941309 28941612 + AluSp SINE/Alu 1 307**

**28941613 28941697 + L1ME2 LINE/L1 6047 6130**

**28941699 28941980 + AluSc SINE/Alu 1 296 (R12)**

**28942053 28942293 C AluJb SINE/Alu 251 15**

**__________________________________________________________________________________**

**AluYa5_16_37 29417248-29417555 C_INTER_RMD_M_DISRUPTED**

**29410723 29410999 + AluSg1 SINE/Alu 1 277**

**29411037 29411335 + AluSp SINE/Alu 6 304**

**29411336 29411634 + AluSc SINE/Alu 1 299**

**29411640 29411718 + L1MB2 LINE/L1 6082 6161**

**29412084 29412371 C AluSq SINE/Alu 291 1**

**29412789 29413094 + AluSx SINE/Alu 8 308**

**29413540 29413969 + LTR48 LTR/ERV1 325 786**

**29414668 29414781 C MER30 DNA/MER1_type 230 108**

**29414782 29415077 C AluSq SINE/Alu 295 1**

**29415081 29415392 C AluSx SINE/Alu 312 1**

**29415393 29415494 C MER30 DNA/MER1_type 107 6**

**29415518 29415816 C AluSg SINE/Alu 300 1**

**29416056 29416354 + AluY SINE/Alu 1 300**

**29416624 29416840 + L1ME2 LINE/L1 5811 6046**

**29416841 29417160 + AluSp SINE/Alu 1 323**

**29417161 29417246 + L1ME2 LINE/L1 6047 6130**

**29417248 29417555 + AluYa5 SINE/Alu 1 310 ( AluYa5_16_37 ) (R1)**

**29417583 29417761 + AluSg/x SINE/Alu 115 306 (R2)**

**29417834 29418074 C AluJb SINE/Alu 251 15**

**29418323 29418391 + G-rich Low_complexity 3 74**

**29418412 29418449 + GA-rich Low_complexity 1 38**

**29419109 29419371 + L1M5 LINE/L1 2667 2945**

**29419879 29420018 C MER113 DNA/MER1_type 428 292**

**29420020 29420330 + AluSg SINE/Alu 1 310**

**29420398 29420703 + AluSg SINE/Alu 1 301**

**29420978 29421038 + MIRb SINE/MIR 44 103**

**29422262 29422582 + MLT1I LTR/MaLR 74 399**

**29422636 29422874 + MIRb SINE/MIR 27 267**

**29423049 29423332 + AluSg SINE/Alu 1 286**

**29423337 29423379 + (TG)n Simple_repeat 2 44**

**29423695 29423989 C AluSx SINE/Alu 297 1**

**29424491 29424703 + MIRb SINE/MIR 3 212**

**Ortholog in Chimp 70031250-70031325 Plus Nscore 0.00**

**70023955 70024267 C AluSx SINE/Alu 298 1**

**70024494 70024673 + MIRb SINE/MIR 23 210**

**70024708 70024995 + AluJb SINE/Alu 1 293**

**70026800 70027102 + AluSx SINE/Alu 8 305**

**70027548 70027978 + LTR48 LTR/ERV1 325 785**

**70028678 70028787 C MER30 DNA/MER1_type 230 110**

**70028788 70029079 C AluSq SINE/Alu 295 3**

**70029083 70029375 C AluSx SINE/Alu 293 1**

**70029376 70029477 C MER30 DNA/MER1_type 109 8**

**70029496 70029790 C AluSg SINE/Alu 296 1**

**70030024 70030325 + AluY SINE/Alu 1 303**

**70030595 70030811 + L1ME2 LINE/L1 5811 6046**

**70030812 70031114 + AluSp SINE/Alu 1 306**

**70031115 70031199 + L1ME2 LINE/L1 6047 6130**

**70031201 70031526 + AluSc SINE/Alu 1 304 (R12)**

**70031599 70031839 C AluJb SINE/Alu 251 15**

**70033092 70033547 + L1M5 LINE/L1 2474 2945**

**70034055 70034196 C MER113 DNA/MER1_type 428 290**

**70034198 70034508 + AluSg SINE/Alu 1 310**

**70034576 70034882 + AluSg SINE/Alu 1 300**

**70034883 70034904 + (TA)n Simple_repeat 2 23**

**70034905 70034936 + (TG)n Simple_repeat 2 34**

**70035251 70035544 C AluSx SINE/Alu 296 1**

**70036079 70036258 + MIRb SINE/MIR 31 212**

**70036431 70036483 + L2 LINE/L2 3367 3419**

**70036486 70036668 + MIR SINE/MIR 28 212**

**70037458 70037677 C MIRb SINE/MIR 268 38**

**70037875 70037937 + MER101-int LTR/ERV1 3075 3137**

**70037929 70038421 + MER101-int LTR/ERV1 3298 3787**

**__________________________________________________________________________________**

**AluYa5_16_38 30157216-30157523 C_INTER_RMD_M_DISRUPTED**

**30150696 30150975 + AluSg1 SINE/Alu 1 280**

**30151010 30151305 + AluSp SINE/Alu 6 301**

**30151308 30151606 + AluSc SINE/Alu 1 299**

**30151612 30151690 + L1MB2 LINE/L1 6082 6161**

**30152056 30152343 C AluSq SINE/Alu 291 1**

**30152761 30153066 + AluSx SINE/Alu 8 308**

**30153512 30153941 + LTR48 LTR/ERV1 325 786**

**30154640 30154753 C MER30 DNA/MER1_type 230 108**

**30154754 30155049 C AluSq SINE/Alu 295 1**

**30155053 30155364 C AluSx SINE/Alu 312 1**

**30155365 30155466 C MER30 DNA/MER1_type 107 6**

**30155480 30155788 C AluSg SINE/Alu 310 1**

**30156027 30156326 + AluY SINE/Alu 1 301**

**30156596 30156812 + L1ME2 LINE/L1 5811 6046**

**30156813 30157128 + AluSp SINE/Alu 1 319**

**30157129 30157214 + L1ME2 LINE/L1 6047 6130**

**30157216 30157523 + AluYa5 SINE/Alu 1 310 ( AluYa5_16_38 ) (R1)**

**30157552 30157729 + AluSg/x SINE/Alu 115 305 (R2)**

**30157802 30158042 C AluJb SINE/Alu 251 15**

**30158291 30158359 + G-rich Low_complexity 3 74**

**30158380 30158417 + GA-rich Low_complexity 1 38**

**30159077 30159339 + L1M5 LINE/L1 2667 2945**

**30159847 30159987 C MER113 DNA/MER1_type 428 291**

**30159990 30160300 + AluSg SINE/Alu 1 310**

**30160368 30160672 + AluSg SINE/Alu 1 300**

**30160947 30161007 + MIRb SINE/MIR 44 103**

**30162231 30162551 + MLT1I LTR/MaLR 74 399**

**30162605 30162843 + MIRb SINE/MIR 27 267**

**30163018 30163301 + AluSg SINE/Alu 1 286**

**30163306 30163360 + (TG)n Simple_repeat 2 56**

**30163676 30163970 C AluSx SINE/Alu 297 1**

**30164472 30164684 + MIRb SINE/MIR 3 212**

**Ortholog in Chimp 70031250-70031325 Plus Nscore 0.00**

**70023955 70024267 C AluSx SINE/Alu 298 1**

**70024494 70024673 + MIRb SINE/MIR 23 210**

**70024708 70024995 + AluJb SINE/Alu 1 293**

**70026800 70027102 + AluSx SINE/Alu 8 305**

**70027548 70027978 + LTR48 LTR/ERV1 325 785**

**70028678 70028787 C MER30 DNA/MER1_type 230 110**

**70028788 70029079 C AluSq SINE/Alu 295 3**

**70029083 70029375 C AluSx SINE/Alu 293 1**

**70029376 70029477 C MER30 DNA/MER1_type 109 8**

**70029496 70029790 C AluSg SINE/Alu 296 1**

**70030024 70030325 + AluY SINE/Alu 1 303**

**70030595 70030811 + L1ME2 LINE/L1 5811 6046**

**70030812 70031114 + AluSp SINE/Alu 1 306**

**70031115 70031199 + L1ME2 LINE/L1 6047 6130**

**70031201 70031526 + AluSc SINE/Alu 1 304 (R12)**

**70031599 70031839 C AluJb SINE/Alu 251 15**

**70033092 70033547 + L1M5 LINE/L1 2474 2945**

**70034055 70034196 C MER113 DNA/MER1_type 428 290**

**70034198 70034508 + AluSg SINE/Alu 1 310**

**70034576 70034882 + AluSg SINE/Alu 1 300**

**70034883 70034904 + (TA)n Simple_repeat 2 23**

**70034905 70034936 + (TG)n Simple_repeat 2 34**

**70035251 70035544 C AluSx SINE/Alu 296 1**

**70036079 70036258 + MIRb SINE/MIR 31 212**

**70036431 70036483 + L2 LINE/L2 3367 3419**

**70036486 70036668 + MIR SINE/MIR 28 212**

**70037458 70037677 C MIRb SINE/MIR 268 38**

**70037875 70037937 + MER101-int LTR/ERV1 3075 3137**

**70037929 70038421 + MER101-int LTR/ERV1 3298 3787**

**__________________________________________________________________________________**

**AluYa5_16_50 45427192-45427499 C_INTER_RMD_M_DISRUPTED**

**45424031 45424211 C Tigger1 DNA/MER2_type 2418 2228**

**45424225 45424439 + AluJb SINE/Alu 89 305**

**45424440 45424631 C Tigger1 DNA/MER2_type 1957 1720**

**45424632 45424882 + AluJb SINE/Alu 26 278**

**45424883 45425725 C Tigger1 DNA/MER2_type 1719 854**

**45425726 45426045 C AluSx SINE/Alu 312 1**

**45426046 45426335 C Tigger1 DNA/MER2_type 853 553**

**45426322 45426670 C Charlie10 DNA/MER1_type 2100 1744**

**45426672 45426827 + L1M4 LINE/L1 3734 3891**

**45426828 45426982 C AluSg/x SINE/Alu 287 133**

**45426984 45427054 + L1M4 LINE/L1 3884 3952**

**45427055 45427191 + L1MB3 LINE/L1 5481 5614**

**45427192 45427499 + AluYa5 SINE/Alu 1 308 ( AluYa5_16_50 ) (R1)**

**45427500 45427714 + AluSg/x SINE/Alu 87 303 (R2)**

**45427715 45427891 + L1MB3 LINE/L1 5615 5787**

**45427892 45428204 + MER7A DNA/MER2_type 1 346**

**45428205 45428594 + L1MB3 LINE/L1 5788 6183**

**45428595 45428831 + L1M4 LINE/L1 3953 4183**

**45428834 45429122 + AluSg SINE/Alu 1 302**

**45429123 45429203 C Charlie10 DNA/MER1_type 1766 1684**

**45429206 45429501 + AluSc SINE/Alu 1 296**

**45429517 45429638 + FLAM_A SINE/Alu 13 134**

**45429648 45430024 + L1M4 LINE/L1 4112 4516**

**45430026 45430143 + FLAM_C SINE/Alu 1 118**

**45430144 45430163 + (TTTTG)n Simple_repeat 2 21**

**45430174 45430534 + L1M4 LINE/L1 4496 4841**

**45430562 45430775 C MLT1H1 LTR/MaLR 358 142**

**Ortholog in Chimp 46053531-46053565 Plus Nscore 0.00**

**46049718 46049898 C Tigger1 DNA/MER2_type 2418 2228**

**46049912 46050119 + AluJb SINE/Alu 89 298**

**46050120 46050311 C Tigger1 DNA/MER2_type 1957 1720**

**46050312 46050562 + AluJb SINE/Alu 26 278**

**46050563 46051425 C Tigger1 DNA/MER2_type 1719 843**

**46051426 46051450 + (T)n Simple_repeat 1 25**

**46052119 46052338 C AluSq SINE/Alu 216 1**

**46052339 46052628 C Tigger1 DNA/MER2_type 859 552**

**46052615 46052963 C Charlie10 DNA/MER1_type 2100 1744**

**46052965 46053120 + L1M4 LINE/L1 3734 3891**

**46053121 46053275 C AluSg/x SINE/Alu 287 133**

**46053277 46053352 + L1M4 LINE/L1 3884 3958**

**46053353 46053484 + L1MB3 LINE/L1 5483 5614**

**46053485 46053789 + AluSx SINE/Alu 1 308 (R12)**

**46053790 46053966 + L1MB3 LINE/L1 5615 5787**

**46053967 46054279 + MER7A DNA/MER2_type 1 346**

**46054280 46054671 + L1MB3 LINE/L1 5788 6181**

**46054672 46054910 + L1M4 LINE/L1 3959 4183**

**46054913 46055216 + AluSx SINE/Alu 1 304**

**46055217 46055297 C Charlie10 DNA/MER1_type 1766 1684**

**46055300 46055594 + AluSc SINE/Alu 1 295**

**46055610 46055736 + FLAM_A SINE/Alu 13 139**

**46055746 46056122 + L1M4 LINE/L1 4112 4516**

**46056124 46056241 + FLAM_C SINE/Alu 1 118**

**46056242 46056261 + (TTTTG)n Simple_repeat 2 21**

**46056271 46056631 + L1M4 LINE/L1 4496 4841**

**46056770 46056863 C MLT1G LTR/MaLR 239 140**

**__________________________________________________________________________________**

**AluYa5_16_52c 47205499-47205790 C_INTER_RMD_M_DISRUPTED**

**47202151 47202494 + AluSp SINE/Alu 1 304**

**47202618 47202712 + MIR3 SINE/MIR 109 205**

**47202798 47202867 + MIR SINE/MIR 107 187**

**47203766 47204069 + AluSq SINE/Alu 1 303**

**47204801 47204916 + FLAM_A SINE/Alu 1 116**

**47204959 47205050 C MIR SINE/MIR 130 38**

**47205051 47205326 + LTR38C LTR/ERV1 1 279**

**47205327 47205498 C AluSg/x SINE/Alu 305 134 (R2)**

**47205499 47205790 C AluYa5 SINE/Alu 295 5 ( AluYa5_16_52c ) (R1)**

**47205792 47206241 + LTR38C LTR/ERV1 267 711**

**47206751 47206918 + L2 LINE/L2 2779 2952**

**47207001 47207302 C AluSx SINE/Alu 301 1**

**47207316 47207498 C AluSg/x SINE/Alu 312 134**

**47207901 47208198 C AluJo SINE/Alu 296 3**

**47208380 47208633 + AluY SINE/Alu 50 299**

**47208849 47208879 + (TA)n Simple_repeat 2 33**

**47208902 47209000 C L2 LINE/L2 2723 2623**

**47209310 47209518 C MER20 DNA/MER1_type 218 1**

**Ortholog in Chimp 47884748-47884823 Minus Nscore 0.00**

**47881403 47881712 + AluSp SINE/Alu 1 304**

**47881836 47881930 + MIR3 SINE/MIR 109 205**

**47882001 47882085 + MIR_Mars SINE/MIR 94 194**

**47882981 47883289 + AluSx SINE/Alu 1 307**

**47884032 47884147 + FLAM_A SINE/Alu 1 116**

**47884193 47884284 C MIR SINE/MIR 130 38**

**47884285 47884564 + LTR38C LTR/ERV1 1 279**

**47884565 47884872 C AluSx SINE/Alu 312 5 (R12)**

**47884873 47885323 + LTR38C LTR/ERV1 280 711**

**47885850 47886020 + L2 LINE/L2 2779 2950**

**47886100 47886401 C AluSx SINE/Alu 301 1**

**47886421 47886603 C AluSg/x SINE/Alu 312 134**

**47886995 47887292 C AluJo SINE/Alu 296 3**

**47887661 47887912 C AluY SINE/Alu 297 50**

**47887965 47887993 + (TA)n Simple_repeat 2 30**

**47888022 47888120 C L2 LINE/L2 2723 2623**

**47888430 47888638 C MER20 DNA/MER1_type 218 1**

**__________________________________________________________________________________**

**AluYa5_16_67 55131968-55132277 C_INTER_RMD**

**55124910 55125039 C L3 LINE/CR1 4305 4135**

**55125369 55125502 C MER4C LTR/ERV1 435 299**

**55125543 55125858 C MER4C LTR/ERV1 547 3**

**55125863 55126020 C ERVL-B4 LTR/ERVL 1528 1381**

**55126021 55126325 C AluJb SINE/Alu 301 1**

**55126326 55126447 C ERVL-B4 LTR/ERVL 1380 1258**

**55126911 55126932 + AT_rich Low_complexity 1 22**

**55126933 55127315 C L1PB1 LINE/L1 6148 5763**

**55127316 55127706 + MLT1C LTR/MaLR 64 467**

**55127980 55128131 C MIR3 SINE/MIR 184 18**

**55128289 55128952 + L1MB4 LINE/L1 5504 6178**

**55128964 55129233 C AluSq SINE/Alu 302 28**

**55129245 55129375 + AluJb SINE/Alu 1 132**

**55129580 55129679 C L1MC4a LINE/L1 5923 5795**

**55129798 55130111 + AluJo SINE/Alu 1 312**

**55130323 55130539 C MIR SINE/MIR 225 12**

**55130561 55130652 C L2 LINE/L2 3365 3267**

**55130841 55130875 C MIRb SINE/MIR 160 107**

**55130876 55131147 + AluJo SINE/Alu 23 294**

**55131148 55131232 C MIRb SINE/MIR 106 23**

**55131515 55131967 + LTR26 LTR/ERV1 1 448**

**55131968 55132277 + AluYa5 SINE/Alu 1 307 ( AluYa5_16_67 ) (R1)**

**55132279 55132451 + AluSg/x SINE/Alu 134 306 (R2)**

**55132453 55132608 + LTR26 LTR/ERV1 438 603**

**55132625 55132727 C MER113 DNA/MER1_type 514 411**

**55132744 55133039 + AluSq SINE/Alu 1 291**

**55133348 55133479 + MIR SINE/MIR 33 177**

**55133490 55133793 + L2 LINE/L2 3135 3414**

**55134160 55134247 C MIRb SINE/MIR 260 167**

**55134248 55134549 + AluJo SINE/Alu 4 307**

**55134550 55134700 C MIRb SINE/MIR 166 28**

**55134972 55135112 + FLAM_C SINE/Alu 1 143**

**55135130 55135176 + (GAAA)n Simple_repeat 2 48**

**55135259 55135575 + AluSx SINE/Alu 1 282**

**55135699 55135883 + L1MC4 LINE/L1 7382 7575**

**55135884 55136016 + FLAM_C SINE/Alu 2 132**

**55136017 55136360 + L1MC4 LINE/L1 7576 7918**

**55136410 55136508 + L1MC4 LINE/L1 7921 8039**

**55136562 55136615 + T-rich Low_complexity 1 54**

**55136789 55137096 C AluSx SINE/Alu 308 1**

**55137207 55137323 C L1MC4a LINE/L1 5923 5820**

**55137324 55137632 C AluJo SINE/Alu 303 2**

**55137633 55137980 C L1MC4a LINE/L1 5819 5454**

**55137981 55138284 C AluSp SINE/Alu 307 3**

**55138285 55138397 C L1MC4a LINE/L1 5453 5352**

**Ortholog in Chimp 55971835-55971906 Plus Nscore 0.00**

**55964744 55964873 C L3 LINE/CR1 4305 4135**

**55965182 55965201 + (T)n Simple_repeat 1 20**

**55965204 55965337 C MER4C LTR/ERV1 435 299**

**55965405 55965489 C LTR59 LTR/ERV1 433 357**

**55965474 55965697 C MER4C LTR/ERV1 260 3**

**55965702 55965859 C ERVL-B4 LTR/ERVL 1528 1382**

**55965860 55966159 C AluJb SINE/Alu 301 5**

**55966160 55966285 C ERVL-B4 LTR/ERVL 1381 1258**

**55966710 55966731 + AT_rich Low_complexity 1 22**

**55966732 55967114 C L1PB1 LINE/L1 6148 5763**

**55967115 55967505 + MLT1C LTR/MaLR 64 467**

**55967778 55967929 C MIR3 SINE/MIR 184 18**

**55968087 55968752 + L1MB4 LINE/L1 5504 6178**

**55968764 55969035 C AluSq SINE/Alu 304 28**

**55969047 55969177 + AluJb SINE/Alu 1 132**

**55969382 55969481 C L1MC4a LINE/L1 5923 5795**

**55969600 55969955 + AluJo SINE/Alu 1 354**

**55970097 55970350 C MIR SINE/MIR 269 12**

**55970652 55970686 C MIRb SINE/MIR 160 107**

**55970687 55970964 + AluJo SINE/Alu 23 299**

**55970965 55971049 C MIRb SINE/MIR 106 23**

**55971332 55971785 + LTR26 LTR/ERV1 1 448**

**55971786 55972097 + AluSx SINE/Alu 1 304 (R12)**

**55972098 55972252 + LTR26 LTR/ERV1 449 603**

**55972269 55972371 C MER113 DNA/MER1_type 514 411**

**55972388 55972693 + AluSq SINE/Alu 1 305**

**55972897 55972953 + MIRm SINE/MIR 85 140**

**55972986 55973139 + MIR SINE/MIR 15 185**

**55973142 55973445 + L2 LINE/L2 3135 3414**

**55973812 55973899 C MIRb SINE/MIR 260 167**

**55973900 55974201 + AluJo SINE/Alu 4 307**

**55974202 55974385 C MIRb SINE/MIR 166 1**

**55974624 55974764 + FLAM_C SINE/Alu 1 143**

**55974769 55974814 + A-rich Low_complexity 1 46**

**55974896 55975220 + AluSx SINE/Alu 1 325**

**55975221 55975232 + AluSx SINE/Alu 283 293**

**55975348 55975532 + L1MC4 LINE/L1 7382 7575**

**55975537 55975665 + FLAM_C SINE/Alu 6 131**

**55975668 55976011 + L1MC4 LINE/L1 7565 7918**

**55976061 55976159 + L1MC4 LINE/L1 7921 8039**

**55976213 55976269 + T-rich Low_complexity 1 57**

**55976443 55976750 C AluSx SINE/Alu 308 1**

**55976891 55976971 C L1MC4a LINE/L1 5894 5819**

**55976972 55977286 C AluJo SINE/Alu 309 2**

**55977287 55977633 C L1MC4a LINE/L1 5818 5453**

**55977634 55977929 C AluSp SINE/Alu 299 3**

**55977930 55978042 C L1MC4a LINE/L1 5452 5351**

**__________________________________________________________________________________**

**AluYa5_16_69c 55927502-55927795 C_DISRUPTED_M_INTER_RMD**

**55923152 55923249 C L1MB7 LINE/L1 3488 3383**

**55923250 55923499 C AluSg1 SINE/Alu 298 1**

**55923500 55923722 C L1MB7 LINE/L1 3382 3132**

**55923749 55923783 + AT_rich Low_complexity 1 35**

**55924084 55925079 C L1MB7 LINE/L1 3150 2150**

**55925080 55925370 C AluSx SINE/Alu 292 1**

**55925371 55925773 C L1MB7 LINE/L1 2160 1761**

**55925774 55926092 C AluSx SINE/Alu 310 1**

**55926093 55926494 C L1MB7 LINE/L1 1798 1401**

**55926599 55926775 + MLT1A LTR/MaLR 1 175**

**55926820 55927501 C L1MB3 LINE/L1 6062 5374**

**55927502 55927795 C AluYa5 SINE/Alu 299 1 ( AluYa5_16_69c ) (R12)**

**55927796 55927897 C L1M LINE/L1 6233 6141**

**55927898 55927951 + (TA)n Simple_repeat 2 55**

**55927953 55928251 C AluSx SINE/Alu 297 1**

**55928252 55928816 C L1M LINE/L1 6140 5590**

**55928817 55928942 C FLAM_C SINE/Alu 127 2**

**55928943 55929365 C L1M LINE/L1 5589 5177**

**55929366 55929659 C AluJb SINE/Alu 296 1**

**55929660 55930010 C L1M LINE/L1 5174 4821**

**Ortholog in Chimp 56777652-56778354 Minus Nscore 0.00**

**56773259 56773356 C L1MB7 LINE/L1 3492 3383**

**56773357 56773606 C AluSg1 SINE/Alu 298 1**

**56773607 56773829 C L1MB7 LINE/L1 3382 3132**

**56774161 56775180 C L1MB7 LINE/L1 3177 2150**

**56775181 56775471 C AluSx SINE/Alu 292 1**

**56775472 56775874 C L1MB7 LINE/L1 2160 1761**

**56775875 56776193 C AluSx SINE/Alu 310 1**

**56776194 56776595 C L1MB7 LINE/L1 1798 1401**

**56776700 56776876 + MLT1A LTR/MaLR 1 175**

**56776884 56777602 C L1MB2 LINE/L1 6099 5358**

**56777605 56777781 C AluY SINE/Alu 297 119 (R2)**

**56777782 56777808 + (T)n Simple_repeat 1 27**

**56777851 56778216 C L1Pt LINE/L1 6155 5790**

**56778217 56778264 + L1Pt LINE/L1 5733 5780**

**56778265 56778403 C AluY SINE/Alu 142 1 (R1)**

**56778404 56778528 C L1M4 LINE/L1 5373 5256**

**56778529 56778821 C AluSx SINE/Alu 291 1**

**56778822 56779387 C L1M4 LINE/L1 5255 4721**

**56779388 56779513 C FLAM_C SINE/Alu 127 2**

**56779514 56779925 C L1M4 LINE/L1 4720 4331**

**56779926 56780222 C AluJb SINE/Alu 299 1**

**56780223 56780583 C L1M4 LINE/L1 4328 3951**

**56780582 56781106 C L1M4b LINE/L1 2183 1606**

**56781110 56781247 + AluSq/x SINE/Alu 1 138**

**56781360 56781406 + Alu SINE/Alu 244 290**

**56781549 56781658 + L1ME3A LINE/L1 5781 5894**

**56781660 56781811 + AluJb SINE/Alu 137 297**

**56781862 56782158 C AluJb SINE/Alu 301 1**

**56782289 56782598 C AluSg SINE/Alu 309 1**

**56782626 56782731 C L1MC4 LINE/L1 8042 7937**

**56783310 56783411 + MIR_Mars SINE/MIR 96 193**

**56783437 56783555 C MIRb SINE/MIR 191 72**

**56783881 56784301 + MLT1J2 LTR/MaLR 5 450**

**56784531 56784711 C L2 LINE/L2 1953 1750**

**56784712 56784915 + AluSx SINE/Alu 1 203**

**56785170 56785393 C MER30 DNA/MER1_type 227 2**

**__________________________________________________________________________________**

**AluYa5_16_72 57305674-57305973 C_INTER_RMD_M_DISRUPTED**

**57299865 57300043 + AluSg/x SINE/Alu 137 302**

**57300385 57300717 + AluJb SINE/Alu 1 334**

**57300718 57300756 + AluJb SINE/Alu 282 312**

**57301213 57301469 + Tigger5a DNA/MER2_type 26 289**

**57301470 57301601 C AluJo SINE/Alu 135 2**

**57301605 57301923 C AluJb SINE/Alu 308 14**

**57301929 57301990 + Tigger5a DNA/MER2_type 366 449**

**57302005 57302136 + AluY SINE/Alu 1 132**

**57302140 57302398 + AluY SINE/Alu 50 307**

**57302998 57303182 + MER102b DNA/MER1_type 99 295**

**57303635 57303915 + AluSx SINE/Alu 1 284**

**57303916 57303959 + (CAA)n Simple_repeat 2 45**

**57304233 57304532 + AluSp SINE/Alu 1 311**

**57304537 57304578 + (CAAA)n Simple_repeat 4 47**

**57304762 57304892 + FLAM_C SINE/Alu 1 132**

**57304897 57304920 + (CAAAA)n Simple_repeat 2 25**

**57305183 57305402 C MER58A DNA/MER1_type 223 2**

**57305450 57305476 + AT_rich Low_complexity 1 27**

**57305477 57305613 C FLAM_C SINE/Alu 132 2**

**57305674 57305973 + AluYa5 SINE/Alu 1 298 ( AluYa5_16_72 ) (R1)**

**57305974 57306143 + AluSg/x SINE/Alu 134 312 (R2)**

**57306251 57306365 C AluJb SINE/Alu 134 3**

**57306370 57306407 + Charlie9 DNA/MER1_type 2610 2648**

**57306467 57306601 + FLAM_C SINE/Alu 1 131**

**57306829 57306942 + FRAM SINE/Alu 35 148**

**57306973 57307256 C AluSp SINE/Alu 285 1**

**57307965 57307999 + (CA)n Simple_repeat 2 36**

**57308497 57308811 + AluJb SINE/Alu 1 311**

**57309093 57309391 C AluSq SINE/Alu 303 1**

**57310205 57310403 + MIR SINE/MIR 15 248**

**57310881 57311186 + AluSx SINE/Alu 1 309**

**57311325 57311627 + AluSp SINE/Alu 1 303**

**57311984 57312290 C AluSx SINE/Alu 300 1**

**Ortholog in Chimp 58216168-58216250 Plus Nscore 0.00**

**58210324 58210496 + AluSg/x SINE/Alu 137 302**

**58210842 58211149 + AluJb SINE/Alu 1 309**

**58211655 58211911 + Tigger5a DNA/MER2_type 26 289**

**58211912 58212043 C AluJo SINE/Alu 135 2**

**58212047 58212364 C AluJb SINE/Alu 308 14**

**58212370 58212431 + Tigger5a DNA/MER2_type 366 449**

**58212446 58212577 + AluY SINE/Alu 1 132**

**58212581 58212827 + AluY SINE/Alu 50 295**

**58213423 58213608 + MER102b DNA/MER1_type 99 295**

**58214065 58214345 + AluSx SINE/Alu 1 284**

**58214346 58214389 + (CAA)n Simple_repeat 2 45**

**58214664 58214975 + AluSp SINE/Alu 1 313**

**58214981 58215022 + (CAAA)n Simple_repeat 4 47**

**58215206 58215336 + FLAM_C SINE/Alu 1 132**

**58215341 58215369 + (CAAAA)n Simple_repeat 2 30**

**58215633 58215850 C MER58A DNA/MER1_type 221 2**

**58215898 58215924 + AT_rich Low_complexity 1 27**

**58215925 58216061 C FLAM_C SINE/Alu 132 2**

**58216122 58216392 + AluSx SINE/Alu 1 275 (R12)**

**58216400 58216428 + (CAAAA)n Simple_repeat 2 30**

**58216536 58216651 C AluJb SINE/Alu 134 2**

**58216752 58216886 + FLAM_C SINE/Alu 1 131**

**58217114 58217227 + FRAM SINE/Alu 35 148**

**58217258 58217541 C AluSp SINE/Alu 285 1**

**58218251 58218289 + (CA)n Simple_repeat 2 40**

**58218787 58219101 + AluJo SINE/Alu 1 311**

**58219379 58219677 C AluSq SINE/Alu 303 1**

**58220491 58220689 + MIR SINE/MIR 15 248**

**58221167 58221472 + AluSx SINE/Alu 1 309**

**58221611 58221913 + AluSp SINE/Alu 1 303**

**58222270 58222582 C AluSx SINE/Alu 298 1**

**___________________________________________________________________________________________**

**AluYa5_17_2 1170679-1170846 C_DISRUPTED_M_INTER_RMD**

**1166520 1166817 C AluSx SINE/Alu 298 1**

**1167188 1167494 + AluSq SINE/Alu 1 312**

**1167545 1167864 + AluJo SINE/Alu 1 312**

**1168028 1168342 C AluSx SINE/Alu 312 1**

**1168343 1168377 C MIRm SINE/MIR 268 234**

**1168345 1168415 C MIRb SINE/MIR 260 174**

**1168418 1168724 C AluSp SINE/Alu 306 1**

**1168725 1168963 C L1M3 LINE/L1 5732 5497**

**1168964 1169291 C AluSx SINE/Alu 327 1**

**1169297 1169583 C AluJb SINE/Alu 294 1**

**1169589 1169884 C AluY SINE/Alu 298 1**

**1169885 1169926 C L1M3 LINE/L1 5492 5450**

**1170055 1170231 + L1M5 LINE/L1 4490 4670**

**1170232 1170530 + AluSx SINE/Alu 1 299**

**1170531 1170676 + L1M5 LINE/L1 4671 4807**

**1170679 1170846 + AluYa5 SINE/Alu 136 303 ( AluYa5_17_2 ) (Truncated insertion in human)**

**1170847 1170944 + L1M5 LINE/L1 4796 4898**

**1170945 1171254 + AluSx SINE/Alu 1 308**

**1171255 1171560 + L1M5 LINE/L1 4899 5205**

**1171562 1171847 + AluSx SINE/Alu 1 287**

**1171848 1171867 + (GA)n Simple_repeat 2 21**

**1171894 1171961 C MER5A DNA/MER1_type 188 117**

**1172174 1172379 C MIRb SINE/MIR 209 3**

**1172438 1172532 + AluSq SINE/Alu 77 169**

**1172819 1173009 C AluSx SINE/Alu 298 128**

**1173010 1173314 C AluY SINE/Alu 305 1**

**1173315 1173453 C AluSx SINE/Alu 127 1**

**1173596 1173890 + AluSq SINE/Alu 1 299**

**1173900 1173939 + MER5B DNA/MER1_type 1 52**

**1173940 1173984 + (CA)n Simple_repeat 2 46**

**1173985 1174118 + MER5B DNA/MER1_type 53 169**

**Ortholog in Chimp 1281703-1281907 Plus Nscore 0.00**

**1277029 1277335 C AluSx SINE/Alu 307 1**

**1277708 1278014 + AluSq SINE/Alu 3 313**

**1278068 1278386 + AluJo SINE/Alu 1 312**

**1279005 1279316 C AluSx SINE/Alu 312 1**

**1279317 1279351 C MIRm SINE/MIR 268 234**

**1279319 1279389 C MIRb SINE/MIR 260 174**

**1279392 1279694 C AluSp SINE/Alu 302 1**

**1279695 1279933 C L1M3 LINE/L1 5732 5496**

**1279934 1280241 C AluSx SINE/Alu 307 1**

**1280247 1280549 C AluJb SINE/Alu 310 1**

**1280554 1280860 C AluY SINE/Alu 308 1**

**1280861 1280902 C L1M3 LINE/L1 5494 5452**

**1281031 1281207 + L1M5 LINE/L1 4490 4670**

**1281208 1281506 + AluSx SINE/Alu 1 299**

**1281507 1281653 + L1M5 LINE/L1 4671 4796**

**1281654 1281955 + AluY SINE/Alu 1 302 (Full length insertion in Chimp- possibly parallel insertion)**

**1281956 1282053 + L1M5 LINE/L1 4797 4898**

**1282054 1282362 + AluSx SINE/Alu 1 308**

**1282363 1282668 + L1M5 LINE/L1 4899 5205**

**1282670 1282956 + AluSx SINE/Alu 1 287**

**1282957 1282976 + (GA)n Simple_repeat 2 21**

**1283003 1283070 C MER5A DNA/MER1_type 188 117**

**1283268 1283488 C MIRb SINE/MIR 224 3**

**1283547 1283639 + AluSp SINE/Alu 77 169**

**1283926 1284097 C AluY SINE/Alu 299 128**

**1284107 1284135 + (TTG)n Simple_repeat 2 30**

**1284136 1284323 C AluY SINE/Alu 295 108**

**1284969 1285101 C L1P1 LINE/L1 5017 4885**

**1285217 1285308 + AluSp/q SINE/Alu 214 307**

**1285318 1285357 + MER5B DNA/MER1_type 1 60**

**1285358 1285410 + (CA)n Simple_repeat 2 54**

**1285411 1285544 + MER5B DNA/MER1_type 61 170**

**__________________________________________________________________________________**

**AluYa5_17_5 2278492-2278601 C_INTER_RMD_M_DISRUPTED**

**2276275 2276399 C FLAM_A SINE/Alu 122 1**

**2276480 2276492 + L1MC4a LINE/L1 5872 5880**

**2276493 2276802 + AluSg SINE/Alu 1 310**

**2276803 2276896 + L1MC4a LINE/L1 5881 5984**

**2276897 2277210 + AluSx SINE/Alu 1 303**

**2277211 2277296 + L1MC4a LINE/L1 5985 6071**

**2277655 2277778 C FLAM_A SINE/Alu 132 8**

**2277877 2278029 + AluJb SINE/Alu 2 151**

**2278063 2278125 + GA-rich Low_complexity 5 67**

**2278166 2278476 + L1MC4 LINE/L1 6701 7024**

**2278492 2278601 + AluYa5/8 SINE/Alu 201 310 ( AluYa5_17_5 )**

**2278609 2278647 + (TA)n Simple_repeat 1 39**

**2278660 2278797 + FLAM_C SINE/Alu 2 133**

**2278849 2278890 + L1MC5 LINE/L1 6972 7003**

**2278891 2279201 C AluSp SINE/Alu 312 1**

**2279202 2279242 + L1MC5 LINE/L1 7004 7036**

**2279243 2279531 C AluSx SINE/Alu 299 1**

**2279532 2279851 + L1MC5 LINE/L1 7037 7333**

**2279857 2280147 C AluSx SINE/Alu 298 11**

**2280162 2280344 C AluJo SINE/Alu 295 110**

**2280452 2280761 C AluY SINE/Alu 307 1**

**2280765 2280800 + AT_rich Low_complexity 1 36**

**2280972 2281001 + AT_rich Low_complexity 1 30**

**2281002 2281290 C AluSq SINE/Alu 288 1**

**2281292 2281763 C L1ME3A LINE/L1 5881 5371**

**Ortholog in Chimp 2423580-2423635 Plus Nscore 0.00**

**2421292 2421416 C FLAM_A SINE/Alu 121 1**

**2421497 2421509 + L1MC4a LINE/L1 5872 5881**

**2421510 2421821 + AluSg SINE/Alu 1 310**

**2421822 2421914 + L1MC4a LINE/L1 5882 5985**

**2421915 2422232 + AluSx SINE/Alu 1 303**

**2422233 2422318 + L1MC4a LINE/L1 5986 6071**

**2422683 2422807 C FLAM_A SINE/Alu 133 8**

**2422906 2423050 + FLAM_C SINE/Alu 2 143**

**2423102 2423164 + GA-rich Low_complexity 5 67**

**2423205 2423515 + L1MC4 LINE/L1 6701 7024**

**2423516 2423637 + Alu SINE/Alu 188 307 (OCCUPIED)**

**2423648 2423777 + FLAM_C SINE/Alu 2 133**

**2423831 2423872 + L1MC5 LINE/L1 6972 7003**

**2423873 2424183 C AluSp SINE/Alu 312 1**

**2424184 2424224 + L1MC5 LINE/L1 7004 7047**

**2424225 2424404 C AluSc SINE/Alu 299 108**

**2424518 2424823 C AluY SINE/Alu 303 1**

**2424827 2424863 + AT_rich Low_complexity 1 37**

**2425035 2425055 + AT_rich Low_complexity 1 21**

**2425056 2425357 C AluSx SINE/Alu 296 1**

**2425359 2425830 C L1ME3A LINE/L1 5881 5371**

**2425833 2425855 + AT_rich Low_complexity 1 23**

**2426057 2426361 C AluY SINE/Alu 299 1**

**2426398 2426697 + AluJb SINE/Alu 1 301**

**2426699 2426794 + MER85 DNA/PiggyBac 34 139**

**2426896 2427069 C MIR SINE/MIR 262 81**

**2427659 2427730 + MIRb SINE/MIR 6 75**

**2428319 2428627 C AluSx SINE/Alu 295 1**

**__________________________________________________________________________________**

**AluYa5_17_44 28501703-28502003 C_INTER_RMD_M_DISRUPTED**

**28496225 28496302 + LTR50 LTR/ERVL 19 106**

**28496572 28496758 + LTR33A LTR/ERVL 309 504**

**28496775 28496824 + MIRm SINE/MIR 111 157**

**28497368 28497581 C MIRb SINE/MIR 259 21**

**28499390 28499560 C MIRb SINE/MIR 180 3**

**28499623 28499852 + MIR SINE/MIR 9 256**

**28499943 28500254 + AluSx SINE/Alu 1 312**

**28500308 28500419 C L1ME4a LINE/L1 6093 5966**

**28500441 28500577 C L1ME4a LINE/L1 5896 5762**

**28500616 28500712 + MIRm SINE/MIR 172 273**

**28500735 28500953 C L1MC LINE/L1 5569 5351**

**28501703 28502003 + AluYa5 SINE/Alu 5 304 ( AluYa5_17_44 ) (R1)**

**28502004 28502177 + AluSg/x SINE/Alu 126 299 (R2)**

**28502179 28502297 + AluSx SINE/Alu 2 123**

**28502298 28502354 + (CA)n Simple_repeat 2 58**

**28502355 28502529 + AluSx SINE/Alu 124 301**

**28502555 28502628 + MIR SINE/MIR 66 162**

**28502629 28503015 + MSTD LTR/MaLR 5 396**

**28503016 28503111 + MIR SINE/MIR 163 256**

**28503155 28503316 C MIRb SINE/MIR 261 102**

**28503885 28503953 C MER103 DNA 123 55**

**28504208 28504385 C MIRb SINE/MIR 216 10**

**28504818 28505197 + LTR16A LTR/ERVL 30 435**

**Ortholog in Chimp 24072956-24073022 Minus Nscore 0.00**

**24069755 24070134 C LTR16A LTR/ERVL 435 30**

**24070568 24070757 + MIRb SINE/MIR 11 232**

**24070999 24071067 + MER103 DNA 55 123**

**24071636 24071797 + MIRb SINE/MIR 102 261**

**24071841 24071935 C MIR SINE/MIR 256 163**

**24071936 24072322 C MSTD LTR/MaLR 396 5**

**24072323 24072399 C MIR SINE/MIR 162 66**

**24072425 24072600 C AluSx SINE/Alu 304 125**

**24072601 24072657 + (TG)n Simple_repeat 2 58**

**24072658 24072776 C AluSx SINE/Alu 124 3**

**24072778 24073071 C AluSx SINE/Alu 299 5 (R12)**

**24073821 24074039 + L1ME4a LINE/L1 5351 5569**

**24074062 24074158 C MIRm SINE/MIR 273 172**

**24074197 24074333 + L1ME4a LINE/L1 5762 5896**

**24074355 24074466 + L1ME4a LINE/L1 5966 6093**

**24074520 24074831 C AluSx SINE/Alu 312 1**

**24074922 24075151 C MIR SINE/MIR 256 9**

**24075214 24075384 + MIRb SINE/MIR 3 180**

**24077193 24077384 + MIRb SINE/MIR 27 238**

**24077864 24077999 C MIR_Mars SINE/MIR 255 111**

**24078031 24078198 C LTR33A LTR/ERVL 483 309**

**24078468 24078545 C LTR50 LTR/ERVL 106 19**

**__________________________________________________________________________________**

**AluYa5_17_48 32217551-32217858 C_INTER_RMD_M_DISRUPTED**

**32212604 32212827 + MIR SINE/MIR 32 262**

**32212904 32213217 C AluJo SINE/Alu 305 1**

**32213224 32213463 C MIRb SINE/MIR 262 20**

**32213843 32214067 + MIRb SINE/MIR 22 260**

**32214684 32214840 C MIRb SINE/MIR 174 1**

**32215263 32215787 C MLT1K LTR/MaLR 584 14**

**32215918 32216296 C LTR16A1 LTR/ERVL 455 65**

**32216897 32217197 + AluSg SINE/Alu 1 301**

**32217228 32217547 + L1ME4a LINE/L1 5219 5561**

**32217551 32217858 + AluYa5 SINE/Alu 1 308 ( AluYa5_17_48 ) (R1)**

**32217873 32218173 + AluJb SINE/Alu 1 300 (R2)**

**32218174 32218343 + (GGAA)n Simple_repeat 4 172**

**32218362 32218475 + GA-rich Low_complexity 2 118**

**32218476 32218750 + L1ME4a LINE/L1 5559 5829**

**32218751 32219045 + AluJo SINE/Alu 12 300**

**32219046 32219198 + L1ME4a LINE/L1 5830 5995**

**32219409 32219984 C MLT1G LTR/MaLR 581 1**

**32219989 32220272 + MLT1K LTR/MaLR 291 591**

**Ortholog in Chimp 20637589-20637853 Minus Nscore 0.00**

**20635548 20635831 C MLT1K LTR/MaLR 591 291**

**20635836 20636411 + MLT1G LTR/MaLR 1 581**

**20636607 20636774 C L1ME4a LINE/L1 6025 5830**

**20636775 20637075 C AluJo SINE/Alu 300 5**

**20637076 20637343 C L1ME4a LINE/L1 5829 5560**

**20637344 20637401 + (TTCC)n Simple_repeat 1 54**

**20637402 20637588 + CT-rich Low_complexity 1 180**

**20637603 20637899 C AluJb SINE/Alu 297 1 (R12)**

**20637900 20638225 C L1MC LINE/L1 5567 5219**

**20638256 20638554 C AluSg SINE/Alu 299 1**

**20639155 20639533 + LTR16A1 LTR/ERVL 65 455**

**20639664 20640187 + MLT1K LTR/MaLR 14 583**

**20640611 20640767 + MIRb SINE/MIR 1 174**

**20641384 20641627 C MIRb SINE/MIR 260 3**

**20641983 20642227 + MIRb SINE/MIR 15 262**

**20642234 20642544 + AluJo SINE/Alu 1 305**

**20642621 20642853 C MIR SINE/MIR 262 23**

**__________________________________________________________________________________**

**AluYa5_17_77c 55241639-55241948 C_INTER_RMD_M_DISRUPTED**

**55234744 55234879 C AluSq SINE/Alu 265 130**

**55234890 55235187 C AluSp SINE/Alu 300 1**

**55236054 55236088 + AT_rich Low_complexity 1 35**

**55236475 55236780 + AluSx SINE/Alu 1 303**

**55238587 55238875 + AluSq SINE/Alu 1 300**

**55239328 55239639 C AluY SINE/Alu 311 1**

**55239694 55239998 + AluSq SINE/Alu 1 306**

**55240237 55240459 + MIRb SINE/MIR 3 234**

**55241147 55241439 C AluSx SINE/Alu 295 1**

**55241448 55241632 C AluSq SINE/Alu 311 129 (R2)**

**55241639 55241948 C AluYa5 SINE/Alu 310 1 ( AluYa5_17_77c ) (R1)**

**55242288 55242596 C AluSx SINE/Alu 309 1**

**55243237 55243267 + AT_rich Low_complexity 1 31**

**55244098 55244179 + CT-rich Low_complexity 3 84**

**55244796 55245091 + AluSc SINE/Alu 3 298**

**55245439 55245729 + AluSp SINE/Alu 1 298**

**55246118 55246138 + (ATTG)n Simple_repeat 2 22**

**55246139 55246419 C AluSg SINE/Alu 283 1**

**55246465 55246627 C MIRb SINE/MIR 199 20**

**55246681 55246826 C MIRb SINE/MIR 251 94**

**55247584 55247692 + L2 LINE/L2 3303 3415**

**55247715 55248019 + AluY SINE/Alu 1 302**

**55248668 55248800 + AluY SINE/Alu 1 134**

**55248801 55249116 + AluSg SINE/Alu 1 308**

**Ortholog in Chimp 59013011-59013093 Minus Nscore 0.00**

**59006132 59006250 C AluSq SINE/Alu 248 130**

**59006278 59006558 C AluSp SINE/Alu 283 1**

**59007429 59007463 + AT_rich Low_complexity 1 35**

**59007849 59008148 + AluSx SINE/Alu 1 297**

**59009163 59009211 + A-rich Low_complexity 1 49**

**59009961 59010249 + AluSq SINE/Alu 1 300**

**59010701 59011011 C AluY SINE/Alu 310 1**

**59011066 59011377 + AluSp SINE/Alu 1 313**

**59011615 59011837 + MIRb SINE/MIR 3 234**

**59012525 59012821 C AluSx SINE/Alu 299 1**

**59012830 59013139 C AluSq SINE/Alu 306 1 (R12)**

**59013479 59013787 C AluSx SINE/Alu 309 1**

**59014428 59014458 + AT_rich Low_complexity 1 31**

**59015289 59015370 + CT-rich Low_complexity 3 84**

**59015987 59016282 + AluSc SINE/Alu 3 298**

**59016630 59016927 + AluSp SINE/Alu 1 305**

**59017316 59017344 + (ATTG)n Simple_repeat 2 30**

**59017345 59017625 C AluSg SINE/Alu 283 1**

**59017653 59017846 C MIRb SINE/MIR 213 7**

**59017887 59018032 C MIRb SINE/MIR 251 94**

**59018793 59018901 + L2 LINE/L2 3303 3415**

**59018924 59019226 + AluY SINE/Alu 1 301**

**59019875 59020008 + AluY SINE/Alu 1 134**

**59020009 59020325 + AluSg SINE/Alu 1 309**

**__________________________________________________________________________________**

**AluYa5_17_85 59732905-59733217 C_INTER_RMD_M_DISRUPTED**

**59732015 59732324 + AluY SINE/Alu 2 311**

**59732421 59732642 + L1M5 LINE/L1 5579 5824**

**59732772 59732897 C L2 LINE/L2 3415 3273**

**59732905 59733217 + AluYa5 SINE/Alu 1 310 ( AluYa5_17_85 ) (R1)**

**59733218 59733383 + AluSq SINE/Alu 134 299 (R2)**

**59733388 59733445 + (CA)n Simple_repeat 2 61**

**59733546 59733721 + MER5B DNA/MER1_type 2 178**

**59733740 59733902 + MIRb SINE/MIR 41 218**

**59734183 59734457 C AluSx SINE/Alu 304 30**

**59735051 59735336 C AluJb SINE/Alu 296 11**

**59735337 59735369 C FLAM_A SINE/Alu 133 98**

**59735370 59735674 + AluSx SINE/Alu 2 306**

**59735675 59735778 C FLAM_A SINE/Alu 97 2**

**59736041 59736331 C AluSx SINE/Alu 294 4**

**59736340 59736474 C L1MC4 LINE/L1 8042 7911**

**59736475 59736505 + (CA)n Simple_repeat 2 32**

**59736506 59736530 C L1MC4 LINE/L1 7910 7898**

**Ortholog in Chimp 63592619-63592699 Plus Nscore 0.00**

**63591764 63591978 + AluY SINE/Alu 90 302**

**63592086 63592307 + L1M5 LINE/L1 5579 5824**

**63592437 63592562 C L2 LINE/L2 3415 3273**

**63592570 63592871 + AluSq SINE/Alu 1 300 (R12)**

**63592876 63592917 + (CA)n Simple_repeat 2 44**

**63593019 63593194 + MER5B DNA/MER1_type 2 178**

**63593216 63593408 + MIRb SINE/MIR 43 253**

**63593657 63593933 C AluSx SINE/Alu 306 30**

**63594614 63594634 + AT_rich Low_complexity 1 21**

**63594682 63594892 C L1MC4 LINE/L1 1187 957**

**63594893 63595075 C AluSq SINE/Alu 195 11**

**63595076 63595108 C FLAM_A SINE/Alu 133 98**

**63595109 63595411 + AluSx SINE/Alu 2 304**

**__________________________________________________________________________________**

**AluYa5_17_100 71475831-71476131 C_INTER_RMD_M_DISRUPTED**

**71468687 71468996 + AluSx SINE/Alu 1 310**

**71469014 71469163 + AluSx SINE/Alu 1 133**

**71469164 71469458 + AluSx SINE/Alu 1 305**

**71469459 71469558 + AluSx SINE/Alu 134 218**

**71470351 71470460 C MIRm SINE/MIR 170 58**

**71470470 71470525 + L1PB4 LINE/L1 5928 5979**

**71470526 71470830 + AluSx SINE/Alu 1 310**

**71470831 71471001 + L1PB4 LINE/L1 5980 6143**

**71471096 71471384 + AluSc SINE/Alu 1 291**

**71471468 71471568 + L1MD2 LINE/L1 6242 6343**

**71471578 71471875 + AluSq SINE/Alu 1 304**

**71472412 71472433 + AT_rich Low_complexity 1 22**

**71472451 71472733 + L1MD LINE/L1 4025 4331**

**71472734 71473042 + AluY SINE/Alu 4 307**

**71473043 71473140 + L1MD LINE/L1 4332 4434**

**71473309 71473524 + AluJo SINE/Alu 84 293**

**71473525 71473833 + AluSx SINE/Alu 2 310**

**71473834 71473852 + AluSx SINE/Alu 294 311**

**71473853 71474162 + AluSg SINE/Alu 1 310**

**71474163 71474183 + AT_rich Low_complexity 1 21**

**71474198 71474482 + AluSg SINE/Alu 1 287**

**71474682 71474769 + AluYh9 SINE/Alu 231 311**

**71474865 71475167 + AluSc SINE/Alu 1 299**

**71475276 71475429 + (TA)n Simple_repeat 2 155**

**71475430 71475714 C AluSx SINE/Alu 287 1**

**71475831 71476131 + AluYa5 SINE/Alu 1 301 ( AluYa5_17_100 )**

**71476133 71476304 + AluY SINE/Alu 133 304**

**71476317 71476617 + AluSp SINE/Alu 1 302**

**71476831 71477141 C AluSp SINE/Alu 312 1**

**71477511 71477578 + GA-rich Low_complexity 4 70**

**71477579 71477877 + AluSx SINE/Alu 1 299**

**71477878 71477896 + polypurine Low_complexity 71 89**

**71478686 71478984 + AluSp SINE/Alu 1 300**

**71479136 71479434 + AluSx SINE/Alu 1 299**

**71479648 71479692 + (CA)n Simple_repeat 2 47**

**71479732 71480020 + AluY SINE/Alu 1 299**

**71480034 71480344 + AluSq SINE/Alu 1 306**

**71482427 71482753 + AluY SINE/Alu 1 309**

**71482834 71483130 C AluSx SINE/Alu 303 5**

**Ortholog in Chimp 75581162-75581237 Plus Nscore 0.00**

**75574068 75574167 + AluSx SINE/Alu 134 218**

**75574960 75575069 C MIRm SINE/MIR 170 58**

**75575079 75575134 + L1PB4 LINE/L1 5928 5979**

**75575135 75575439 + AluSx SINE/Alu 1 310**

**75575440 75575610 + L1PB4 LINE/L1 5980 6143**

**75575702 75575988 + AluSc SINE/Alu 1 289**

**75576075 75576175 + L1MD2 LINE/L1 6242 6343**

**75576185 75576488 + AluSx SINE/Alu 1 304**

**75577588 75577609 + AT_rich Low_complexity 1 22**

**75577627 75577902 + L1MD LINE/L1 4025 4324**

**75577910 75578213 + AluY SINE/Alu 4 305**

**75578214 75578323 + L1MC LINE/L1 4332 4435**

**75578491 75578706 + AluJo SINE/Alu 84 293**

**75578707 75579016 + AluSx SINE/Alu 2 311**

**75579017 75579036 + AluSx SINE/Alu 294 312**

**75579037 75579346 + AluSg SINE/Alu 1 310**

**75579376 75579663 + AluSg SINE/Alu 1 287**

**75579863 75579950 + Alu SINE/Alu 231 311**

**75580048 75580339 + AluSc SINE/Alu 1 291**

**75580460 75580483 + (TA)n Simple_repeat 2 25**

**75580484 75580776 C AluSg SINE/Alu 292 1**

**75580819 75580999 C AluSx SINE/Alu 183 1**

**75581116 75581428 + AluY SINE/Alu 1 303**

**75581441 75581740 + AluSp SINE/Alu 1 301**

**75581953 75582264 C AluSp SINE/Alu 313 1**

**75582634 75582701 + GA-rich Low_complexity 4 70**

**75582702 75582997 + AluSx SINE/Alu 1 296**

**75582998 75583016 + polypurine Low_complexity 71 89**

**75583806 75584115 + AluSp SINE/Alu 1 311**

**75584267 75584565 + AluSx SINE/Alu 1 299**

**75584779 75584818 + (CA)n Simple_repeat 2 42**

**75584863 75585157 + AluY SINE/Alu 1 306**

**75585171 75585473 + AluSq SINE/Alu 1 305**

**75587846 75588154 + AluY SINE/Alu 5 311**

**75588224 75588524 C AluSx SINE/Alu 307 5**

**__________________________________________________________________________________**

**AluYa5_17_105 77119396-77119691 C_INTER_RMD_M_DISRUPTED**

**77112769 77112786 + (ATGGTG)n Simple_repeat 3 20**

**77112787 77112966 + (TGG)n Simple_repeat 1 180**

**77112967 77113070 + (ATGGTG)n Simple_repeat 1 104**

**77113076 77113196 + (CACCAT)n Simple_repeat 1 115**

**77113208 77113447 + (CACCAT)n Simple_repeat 1 240**

**77119396 77119691 + AluYa5 SINE/Alu 12 305 ( AluYa5_17_105 )**

**77119692 77119713 + (TA)n Simple_repeat 2 23**

**77119715 77119879 + AluSg/x SINE/Alu 129 295**

**77120149 77120306 + MLT1B LTR/MaLR 22 191**

**77122366 77122661 + AluSx SINE/Alu 1 296**

**77124193 77124350 + FRAM SINE/Alu 4 164**

**77125936 77126226 + AluSx SINE/Alu 1 291**

**77126237 77126543 + AluY SINE/Alu 1 306**

**Ortholog in Chimp 81740555-81740626 Plus Nscore 0.00**

**81733470 81733648 + C-rich Low_complexity 3 175**

**81733649 81733715 + (CACCAT)n Simple_repeat 5 71**

**81740506 81740794 + AluSx SINE/Alu 12 298**

**81741063 81741220 + MLT1B LTR/MaLR 22 191**

**81743275 81743570 + AluSx SINE/Alu 1 296**

**81745102 81745260 + FRAM SINE/Alu 4 165**

**81746848 81747136 + AluSx SINE/Alu 3 291**

**81747147 81747453 + AluY SINE/Alu 1 306**

**__________________________________________________________________________________**

**AluYa5_18_50 39707441-39707750 C_INTER_RMD_M_DISRUPTED**

**39701613 39701897 + AluJo SINE/Alu 1 290**

**39702704 39702759 + MIRb SINE/MIR 31 86**

**39702868 39702892 + AT_rich Low_complexity 1 25**

**39704523 39704545 + AT_rich Low_complexity 1 23**

**39704612 39704640 + AT_rich Low_complexity 1 29**

**39704703 39704964 + HAL1b LINE/L1 1062 1330**

**39705208 39705274 + L1MD LINE/L1 4541 4609**

**39705255 39707129 + L1PA3 LINE/L1 4277 6152**

**39707157 39707225 + A-rich Low_complexity 2 72**

**39707346 39707367 + AT_rich Low_complexity 1 22**

**39707441 39707750 + AluYa5 SINE/Alu 1 310 ( AluYa5_18_50 ) (R1)**

**39707769 39708020 + AluY SINE/Alu 1 252 (R2)**

**39708532 39708655 + L2 LINE/L2 3239 3406**

**39708838 39709140 + L1ME4a LINE/L1 5542 5849**

**39709677 39709720 + (TTTA)n Simple_repeat 1 44**

**39710605 39710635 + (CA)n Simple_repeat 2 32**

**39710981 39711306 + LTR33A LTR/ERVL 1 325**

**39712159 39712238 + (TTTTC)n Simple_repeat 1 80**

**39712240 39712414 + CT-rich Low_complexity 1 180**

**39712440 39712624 C FRAM SINE/Alu 169 10**

**39713459 39713672 + MIRb SINE/MIR 2 215**

**39713719 39714132 C MER74A LTR/ERVL 558 122**

**39714710 39714825 + L2 LINE/L2 2783 2905**

**Ortholog in Chimp 40024546-40024610 Plus Nscore 0.00**

**40018728 40019023 + AluJo SINE/Alu 1 299**

**40019981 40020005 + AT_rich Low_complexity 1 25**

**40021636 40021662 + AT_rich Low_complexity 1 27**

**40021725 40021753 + AT_rich Low_complexity 1 29**

**40021816 40022077 + HAL1b LINE/L1 1062 1330**

**40022321 40022389 + L1M5 LINE/L1 4541 4611**

**40022366 40024266 + L1PA3 LINE/L1 4275 6176**

**40024274 40024345 + A-rich Low_complexity 2 72**

**40024466 40024487 + AT_rich Low_complexity 1 22**

**40024561 40024812 + AluY SINE/Alu 1 252 (R12)**

**40024813 40024871 + (TAAA)n Simple_repeat 2 62**

**40025334 40025435 + L2 LINE/L2 3239 3381**

**40025643 40025942 + L1ME4a LINE/L1 5545 5849**

**40026480 40026523 + (TTTA)n Simple_repeat 1 44**

**40027402 40027426 + (CA)n Simple_repeat 2 26**

**40027900 40028096 + LTR33A LTR/ERVL 114 325**

**40028940 40029006 + T-rich Low_complexity 5 74**

**40029007 40029168 + CT-rich Low_complexity 1 164**

**40029176 40029345 C FRAM SINE/Alu 169 24**

**40029726 40029909 + CT-rich Low_complexity 1 180**

**40029921 40030093 C FRAM SINE/Alu 169 22**

**40030708 40030743 + AT_rich Low_complexity 1 36**

**40030744 40030960 C L1PA2 LINE/L1 6155 5939**

**40031229 40031460 + MIR_Mars SINE/MIR 2 237**

**40031492 40031914 C MER74A LTR/ERVL 558 122**

**__________________________________________________________________________________**

**AluYa5_18_58c 43785966-43786273 C_INTER_RMD_M_DISRUPTED**

**43782167 43782439 + AluSx SINE/Alu 1 271**

**43782445 43782467 + (CAAA)n Simple_repeat 2 24**

**43782507 43782635 + MIRb SINE/MIR 34 160**

**43782910 43783043 C MIR SINE/MIR 181 49**

**43783078 43783125 + (TG)n Simple_repeat 1 50**

**43783901 43784052 + MIR3 SINE/MIR 6 159**

**43784963 43785008 C MIRb SINE/MIR 160 112**

**43785132 43785350 + MER58A DNA/MER1_type 1 216**

**43785421 43785803 C L1PA16 LINE/L1 6164 5769**

**43785804 43785965 C AluY SINE/Alu 294 133 (R2)**

**43785966 43786273 C AluYa5 SINE/Alu 308 1 ( AluYa5_18_58c ) (R1)**

**43786274 43786477 C L1PA16 LINE/L1 5786 5583**

**43786478 43786789 C AluSx SINE/Alu 303 1**

**43786790 43786848 C L1PA16 LINE/L1 5582 5524**

**43787594 43787815 C MER30 DNA/MER1_type 230 1**

**43788688 43788734 + (CGGGGG)n Simple_repeat 6 50**

**43788738 43788808 + (CCCCG)n Simple_repeat 1 71**

**43789071 43789111 + L2 LINE/L2 3335 3375**

**43789158 43789508 C L1MC1 LINE/L1 6327 5944**

**43789515 43789594 C MIRb SINE/MIR 195 113**

**43789910 43790255 C MER115 DNA/Tip100 453 123**

**43790266 43790293 + MER53 DNA 1 27**

**43790294 43790631 + MER1B DNA/MER1_type 1 339**

**43790632 43790675 + MER53 DNA 28 63**

**Ortholog in Chimp 44205665-44205742 Minus Nscore 0.00**

**44200823 44201094 + AluSx SINE/Alu 1 271**

**44201100 44201122 + (CAAA)n Simple_repeat 2 24**

**44201160 44201288 + MIRb SINE/MIR 34 160**

**44201563 44201696 C THER1_MD SINE/MIR 188 56**

**44201731 44201786 + (TG)n Simple_repeat 1 58**

**44202186 44202315 + MIRb SINE/MIR 33 160**

**44202590 44202723 C THER1_MD SINE/MIR 188 56**

**44202758 44202803 + (TG)n Simple_repeat 1 48**

**44203579 44203730 + MIR3 SINE/MIR 6 159**

**44204641 44204686 C MIRb SINE/MIR 160 112**

**44204810 44205028 + MER58A DNA/MER1_type 1 216**

**44205099 44205482 C L1PA16 LINE/L1 6164 5787**

**44205483 44205791 C AluY SINE/Alu 309 1 (R12)**

**44205792 44205995 C L1PA16 LINE/L1 5786 5583**

**44205996 44206310 C AluSx SINE/Alu 304 1**

**44206311 44206369 C L1PA16 LINE/L1 5582 5524**

**44207120 44207341 C MER30 DNA/MER1_type 230 1**

**44208214 44208260 + (CGGGGG)n Simple_repeat 6 50**

**44209210 44209250 + L2 LINE/L2 3335 3375**

**44209297 44209647 C L1MC1 LINE/L1 6327 5944**

**44209654 44209733 C MIRb SINE/MIR 195 113**

**44210049 44210393 C MER115 DNA/Tip100 453 123**

**44210404 44210431 + MER53 DNA 1 27**

**44210432 44210769 + MER1B DNA/MER1_type 1 339**

**44210770 44210813 + MER53 DNA 28 63**

**__________________________________________________________________________________**

**AluYa5_19_15 13698730-13699040 C_INTER_RMD_M_DISRUPTED**

**13695069 13695174 + L1MC4 LINE/L1 7364 7484**

**13695182 13695382 + L1MB3 LINE/L1 5936 6143**

**13695383 13695509 + FLAM_C SINE/Alu 2 125**

**13695510 13695558 + L1MB3 LINE/L1 6135 6183**

**13695572 13695870 + AluSx SINE/Alu 1 298**

**13695871 13695973 + AluSg/x SINE/Alu 135 237**

**13695983 13696011 C MIR SINE/MIR 144 116**

**13696012 13696444 C MSTA LTR/MaLR 428 1**

**13696445 13696555 C MIR SINE/MIR 115 8**

**13697046 13697339 + AluSp SINE/Alu 1 294**

**13697356 13697662 + AluJo SINE/Alu 1 299**

**13697782 13698081 C AluSx SINE/Alu 300 1**

**13698157 13698427 C MLT1C LTR/MaLR 453 155**

**13698428 13698726 + AluJo SINE/Alu 1 296**

**13698730 13699040 + AluYa5 SINE/Alu 1 310 ( AluYa5_19_15 ) (R1)**

**13699041 13699065 + (TAA)n Simple_repeat 3 27**

**13699066 13699356 + AluSg SINE/Alu 1 288 (R2)**

**13699359 13699408 + (TAA)n Simple_repeat 2 51**

**13699465 13699752 + AluSx SINE/Alu 1 293**

**13699813 13699957 C MLT1C LTR/MaLR 152 1**

**13700031 13700196 C MLT1J LTR/MaLR 210 59**

**13700248 13700552 + AluSp SINE/Alu 3 311**

**13700563 13700674 C L1MD3 LINE/L1 7907 7787**

**13700678 13700976 + AluSx SINE/Alu 1 302**

**13700998 13701292 + AluSg SINE/Alu 1 295**

**13701295 13701322 + (TAA)n Simple_repeat 2 30**

**13701481 13701668 + AluSg/x SINE/Alu 137 323**

**13701672 13702064 + MER95 LTR/ERV 15 422**

**Ortholog in Chimp 14112965-14113027 Plus Nscore 0.00**

**14108984 14109089 + L1MC4 LINE/L1 7364 7484**

**14109097 14109297 + L1MB3 LINE/L1 5936 6143**

**14109298 14109424 + FLAM_C SINE/Alu 2 125**

**14109425 14109473 + L1MB2 LINE/L1 6123 6171**

**14109487 14109785 + AluSx SINE/Alu 1 298**

**14109786 14109888 + AluSg/x SINE/Alu 135 237**

**14109898 14109926 C MIR SINE/MIR 144 116**

**14109927 14110359 C MSTA LTR/MaLR 428 1**

**14110360 14110470 C MIR SINE/MIR 115 8**

**14110963 14111256 + AluSp SINE/Alu 1 294**

**14111273 14111591 + AluJo SINE/Alu 1 312**

**14111720 14111950 C AluSg/x SINE/Alu 300 77**

**14112178 14112330 C AluSq SINE/Alu 153 1**

**14112406 14112676 C MLT1C LTR/MaLR 453 155**

**14112677 14112975 + AluJo SINE/Alu 1 296**

**14112982 14113292 + AluSg SINE/Alu 1 308 (R12)**

**14113344 14113631 + AluSx SINE/Alu 1 293**

**14113692 14113836 C MLT1C LTR/MaLR 152 1**

**14113910 14114075 C MLT1J2 LTR/MaLR 210 59**

**14114127 14114431 + AluSp SINE/Alu 3 311**

**14114447 14114558 C L1MD3 LINE/L1 7907 7787**

**14114562 14114862 + AluSx SINE/Alu 1 304**

**14114863 14114883 + AT_rich Low_complexity 1 21**

**14114885 14115183 + AluSg SINE/Alu 1 299**

**14115186 14115208 + (TAA)n Simple_repeat 2 24**

**14115372 14115545 + AluSg/x SINE/Alu 137 311**

**14115551 14115946 + MER95 LTR/ERV 17 422**

**__________________________________________________________________________________**

**AluYa5_19_16 14371093-14371404 C_INTER_RMD_M_DISRUPTED**

**14365725 14365823 C MER91A DNA/Tip100 101 1**

**14366831 14367137 + AluSx SINE/Alu 1 307**

**14370059 14370153 + (CA)n Simple_repeat 2 97**

**14370166 14370199 + MIR SINE/MIR 2 32**

**14370200 14370511 + AluY SINE/Alu 1 306**

**14370512 14370638 + MIR SINE/MIR 33 136**

**14370639 14370958 C AluJo SINE/Alu 312 1**

**14370959 14371087 + MIR SINE/MIR 137 252**

**14371093 14371404 + AluYa5 SINE/Alu 1 310 ( AluYa5_19_16 ) (R1)**

**14371405 14371568 + AluSq SINE/Alu 132 297 (R2)**

**14371593 14371651 C Charlie5 DNA/MER1_type 2614 2557**

**14371665 14371820 C AluJo SINE/Alu 287 135**

**14371821 14372131 C AluY SINE/Alu 310 1**

**14372132 14372262 C AluJo SINE/Alu 134 4**

**14372322 14372622 + AluSx SINE/Alu 1 301**

**14372725 14372961 + AluJb SINE/Alu 85 310**

**14373661 14373956 + AluJb SINE/Alu 1 296**

**14373959 14374269 + AluSq SINE/Alu 3 306**

**14374710 14374788 + L2 LINE/L2 3033 3122**

**14374815 14375151 C AluSx SINE/Alu 340 3**

**14375154 14375463 C AluJo SINE/Alu 308 3**

**14375483 14375599 C FLAM_C SINE/Alu 122 11**

**14375612 14375676 + L2 LINE/L2 3243 3290**

**14375677 14375990 C AluSq SINE/Alu 312 1**

**14375991 14376108 + L2 LINE/L2 3291 3414**

**14376679 14376956 C AluSx SINE/Alu 297 1**

**14376957 14377255 C AluSx SINE/Alu 300 1**

**Ortholog in Chimp 14812065-14812144 Plus Nscore 0.00**

**14807051 14807151 C MER91A DNA/Tip100 101 1**

**14808159 14808461 + AluSx SINE/Alu 1 303**

**14810993 14811087 + (CA)n Simple_repeat 2 97**

**14811100 14811133 + MIR SINE/MIR 2 32**

**14811134 14811442 + AluY SINE/Alu 1 307**

**14811443 14811562 + MIR SINE/MIR 33 136**

**14811563 14811881 C AluJo SINE/Alu 311 1**

**14811882 14812006 + MIR SINE/MIR 137 248**

**14812016 14812307 + AluSq SINE/Alu 1 294 (R12)**

**14812400 14812555 C AluJo SINE/Alu 287 135**

**14812556 14812864 C AluY SINE/Alu 308 1**

**14812865 14812995 C AluJo SINE/Alu 134 4**

**14813055 14813355 + AluSx SINE/Alu 1 301**

**14813458 14813692 + AluJb SINE/Alu 85 310**

**14814394 14814693 + AluJb SINE/Alu 1 300**

**14814696 14815009 + AluSq SINE/Alu 3 309**

**14815450 14815528 + L2 LINE/L2 3033 3122**

**14815555 14815891 C AluSx SINE/Alu 340 3**

**14815894 14816203 C AluJo SINE/Alu 308 3**

**14816223 14816340 C FLAM_C SINE/Alu 122 11**

**14816353 14816417 + L2 LINE/L2 3243 3291**

**14816418 14816722 C AluSq SINE/Alu 304 1**

**14816723 14816829 + L2 LINE/L2 3292 3403**

**14817411 14817692 C AluSx SINE/Alu 301 1**

**14817693 14817994 C AluSx SINE/Alu 304 1**

**__________________________________________________________________________________**

**AluYa5_19_28 21305680-21305989 C_INTER_RMD_M_DISRUPTED**

**21301291 21301579 + AluSp SINE/Alu 1 286**

**21301580 21301619 + AT_rich Low_complexity 1 40**

**21301890 21302203 + AluSp SINE/Alu 1 312**

**21303202 21303272 + MER93a LTR/ERV1 1 70**

**21304333 21304627 C AluSp SINE/Alu 293 1**

**21304743 21305048 + AluSq SINE/Alu 5 309**

**21305051 21305094 C MER33 DNA/MER1_type 306 262**

**21305098 21305272 C AluSg/x SINE/Alu 305 131**

**21305309 21305546 C MER33 DNA/MER1_type 255 1**

**21305680 21305989 + AluYa5 SINE/Alu 1 310 ( AluYa5_19_28 ) (R1)**

**21305993 21306168 + AluSg/x SINE/Alu 129 311 (R2)**

**21306628 21307124 C MLT1H LTR/MaLR 549 11**

**21307170 21307262 C MIR SINE/MIR 117 11**

**21307411 21307707 + AluJb SINE/Alu 10 298**

**21307791 21308089 + AluSp SINE/Alu 1 299**

**21308138 21308442 + AluSq SINE/Alu 1 305**

**21308493 21308896 C MER57A LTR/ERV1 401 1**

**21308897 21308993 C MER57A-int LTR/ERV1 7537 7448**

**21308994 21309296 + AluSq SINE/Alu 1 303**

**Ortholog in Chimp 21611177-21611257 Plus Nscore 0.00**

**21606745 21607033 + AluSp SINE/Alu 1 286**

**21607034 21607073 + AT_rich Low_complexity 1 40**

**21607344 21607655 + AluSp SINE/Alu 1 310**

**21608650 21608720 + MER93a LTR/ERV1 1 70**

**21609780 21610074 C AluSp SINE/Alu 293 1**

**21610190 21610498 + AluSq SINE/Alu 5 312**

**21610501 21610544 C MER33 DNA/MER1_type 306 262**

**21610548 21610723 C AluSg/x SINE/Alu 306 131**

**21610760 21610997 C MER33 DNA/MER1_type 255 1**

**21611131 21611422 + AluSg SINE/Alu 1 298 (R12)**

**21611880 21612373 C MLT1H LTR/MaLR 549 11**

**21612419 21612515 C MIRb SINE/MIR 117 11**

**21612664 21612961 + AluJb SINE/Alu 10 298**

**21613045 21613344 + AluSp SINE/Alu 1 300**

**21613393 21613701 + AluSq SINE/Alu 1 309**

**21613752 21614155 C MER57A LTR/ERV1 401 1**

**21614156 21614251 C MER57A-int LTR/ERV1 7537 7448**

**21614252 21614557 + AluSq SINE/Alu 1 306**

**21614558 21614609 C MER57A-int LTR/ERV1 7447 7404**

**21614610 21614676 C LTR54B LTR/ERV1 311 234**

**21614704 21614867 C LTR54B LTR/ERV1 163 3**

**21614868 21615058 C MER57A-int LTR/ERV1 5798 5625**

**21615059 21615205 + FRAM SINE/Alu 1 165**

**21615206 21615306 C MER57A-int LTR/ERV1 5624 5528**

**__________________________________________________________________________________**

**AluYa5_19_57c 46085249-46085439 C_INTER_RMD_M_DISRUPTED**

**46078540 46078607 + (GA)n Simple_repeat 1 68**

**46078614 46078790 + GA-rich Low_complexity 1 172**

**46081375 46081530 C MIRb SINE/MIR 204 35**

**46082651 46082831 + MIRb SINE/MIR 51 260**

**46082832 46082911 + MIR3 SINE/MIR 124 202**

**46083687 46083989 C AluY SINE/Alu 308 1**

**46084145 46084310 C MIRb SINE/MIR 230 70**

**46084337 46084632 C AluSq SINE/Alu 296 1**

**46084925 46085052 C L2 LINE/L2 983 840**

**46085180 46085248 C AluS SINE/Alu 301 233**

**46085249 46085439 C AluYa5 SINE/Alu 300 110 ( AluYa5_19_57c )**

**46085446 46085631 + L1M4c LINE/L1 2410 2599**

**46085632 46085925 + AluSc SINE/Alu 1 297**

**46085926 46085961 + L1M4c LINE/L1 2600 2624**

**46085970 46086068 + AluSg/x SINE/Alu 201 294**

**46086347 46086431 + CT-rich Low_complexity 1 81**

**46086506 46086683 C AluSq SINE/Alu 312 133**

**46086684 46086724 + (TG)n Simple_repeat 2 42**

**46086725 46086850 C AluSq SINE/Alu 132 2**

**46087450 46087548 + MER20B DNA/MER1_type 649 752**

**46087878 46088105 + L1MA6 LINE/L1 6065 6295**

**46088202 46088514 + AluSx SINE/Alu 2 311**

**46088525 46088630 C MIR SINE/MIR 146 29**

**46089437 46089519 C MIR3 SINE/MIR 206 127**

**Ortholog in Chimp 46463477-46463567 Minus Nscore 0.00**

**46456390 46456504 + GA-rich Low_complexity 2 111**

**46456522 46456586 + GA-rich Low_complexity 2 63**

**46456764 46456857 + GA-rich Low_complexity 2 97**

**46459538 46459693 C MIRb SINE/MIR 204 35**

**46460815 46460995 + MIRb SINE/MIR 51 260**

**46460996 46461075 + MIR3 SINE/MIR 124 202**

**46461851 46462153 C AluY SINE/Alu 308 1**

**46462314 46462479 C MIRb SINE/MIR 224 65**

**46462742 46462878 C AluSq/x SINE/Alu 138 1**

**46463427 46463616 C AluYa5 SINE/Alu 299 110**

**46463623 46463808 + L1M4c LINE/L1 2410 2599**

**46463809 46464107 + AluSc SINE/Alu 1 302**

**46464828 46464956 C AluSq/x SINE/Alu 129 1**

**46465556 46465654 + MER20B DNA/MER1_type 649 752**

**46465768 46465801 + (CA)n Simple_repeat 2 35**

**46465988 46466215 + L1MA6 LINE/L1 6065 6295**

**46466312 46466620 + AluSx SINE/Alu 2 312**

**46466631 46466736 C MIR SINE/MIR 146 29**

**46467542 46467668 C MIR3 SINE/MIR 206 85**

**46468051 46468163 C MIR SINE/MIR 240 116**

**46468164 46468469 + AluSq SINE/Alu 1 302**

**46468470 46468557 C MIR SINE/MIR 115 21**

**46468594 46468892 C AluSp SINE/Alu 302 3**

**46469088 46469164 + (TCTA)n Simple_repeat 4 80**

**46469175 46469353 + (TCCA)n Simple_repeat 1 180**

**46469221 46469395 + (TCTA)n Simple_repeat 1 180**

**46469994 46470279 + AluY SINE/Alu 17 301**

**46470288 46470459 + FRAM SINE/Alu 1 169**

**__________________________________________________________________________________**

**AluYa5_19_72 50905043-50905351 C_INTER_RMD_M_DISRUPTED**

**50902695 50902929 C L1MEd LINE/L1 927 660**

**50902930 50903250 + AluSq SINE/Alu 1 311**

**50903251 50903536 + AluSq SINE/Alu 1 301**

**50903537 50903598 C L1MEd LINE/L1 659 597**

**50903930 50904220 C AluY SINE/Alu 306 1**

**50904525 50904821 + AluSx SINE/Alu 1 300**

**50904872 50904991 + MLT1J LTR/MaLR 72 202**

**50905043 50905351 + AluYa5 SINE/Alu 1 306 ( AluYa5_19_72 ) (R1)**

**50905352 50905524 + AluJb SINE/Alu 134 307 (R2)**

**50905808 50905850 + A-rich Low_complexity 1 43**

**50906759 50906798 + C-rich Low_complexity 4 43**

**50908784 50909092 + AluSq SINE/Alu 1 309**

**50909616 50909715 + FRAM SINE/Alu 63 161**

**50909716 50910020 + AluJo SINE/Alu 1 303**

**50910036 50910115 C MIR3 SINE/MIR 159 85**

**50910387 50910586 + MER58A DNA/MER1_type 2 224**

**Ortholog in Chimp 51325585-51325657 Plus Nscore 0.00**

**51323182 51323445 C L1MEd LINE/L1 928 660**

**51323446 51323752 + AluSq SINE/Alu 1 305**

**51323753 51324038 + AluSq SINE/Alu 1 301**

**51324039 51324100 C L1MEd LINE/L1 659 597**

**51324434 51324719 C AluY SINE/Alu 301 1**

**51324856 51324891 C MIRb SINE/MIR 70 35**

**51325020 51325320 + AluSx SINE/Alu 1 304**

**51325367 51325487 + MLT1J LTR/MaLR 72 202**

**51325539 51325849 + AluJo SINE/Alu 1 312 (R12)**

**51327070 51327091 + GC_rich Low_complexity 1 22**

**51329510 51329817 + AluSq SINE/Alu 1 308**

**51330339 51330438 + FRAM SINE/Alu 63 161**

**51330439 51330744 + AluJo SINE/Alu 1 304**

**51330760 51330900 C MIR3 SINE/MIR 159 23**

**51331111 51331319 + MER58A DNA/MER1_type 2 224**

**51331356 51331667 + AluSq SINE/Alu 1 313**

**51331880 51332101 + MER46A DNA/MER2_type 1 236**

**51332527 51332546 + (A)n Simple_repeat 1 20**

**51332569 51332791 C MIR SINE/MIR 261 8**

**__________________________________________________________________________________**

**AluYa5_20_26 17840918-17841164 C_INTER_RMD_M_DISRUPTED**

**17836292 17836568 C MLT1A1 LTR/MaLR 340 23**

**17836614 17836750 + L1MC4a LINE/L1 5771 5909**

**17836758 17837056 C AluSx SINE/Alu 299 1**

**17837107 17837388 + AluJb SINE/Alu 1 286**

**17837430 17837578 C AluSg/x SINE/Alu 271 123**

**17837617 17837729 + AluY SINE/Alu 196 308**

**17837730 17837741 + AluY SINE/Alu 283 291**

**17839156 17839227 + MER66C LTR/ERV1 1 64**

**17839228 17839505 + AluSx SINE/Alu 1 279**

**17839506 17839531 + (CA)n Simple_repeat 1 26**

**17839532 17839809 + MER66C LTR/ERV1 65 487**

**17839900 17840089 + MER66B LTR/ERV1 281 475**

**17840098 17840347 + L2 LINE/L2 3141 3418**

**17840361 17840916 C L2 LINE/L2 2447 1810**

**17840918 17841164 + AluYa5 SINE/Alu 2 310 ( AluYa5_20_26 )**

**17841188 17841210 + (TTTTTA)n Simple_repeat 2 24**

**17841214 17841362 + L1M5 LINE/L1 4706 4853**

**17841375 17841914 C L1MC4 LINE/L1 8021 7445**

**17841921 17842392 C L2 LINE/L2 1825 1385**

**17842393 17842668 + L1MB2 LINE/L1 5534 5823**

**17842669 17842972 + AluSx SINE/Alu 5 309**

**17842973 17843327 + L1MB2 LINE/L1 5824 6170**

**17843328 17843577 C L2 LINE/L2 1384 1152**

**17843655 17843823 + MLT1H LTR/MaLR 107 276**

**17843979 17844027 C L1ME4a LINE/L1 5901 5853**

**17844028 17844328 + AluSx SINE/Alu 1 301**

**17844329 17844516 C L1ME4a LINE/L1 5852 5681**

**Ortholog in Chimp 18107886-18108127 Plus Nscore 0.00**

**18102773 18103056 C MLT1A1 LTR/MaLR 340 23**

**18103102 18103238 + L1MC4a LINE/L1 5771 5909**

**18103246 18103544 C AluSx SINE/Alu 299 1**

**18103550 18103841 C L1M3 LINE/L1 5821 5511**

**18104044 18104301 + AluJb SINE/Alu 29 286**

**18104341 18104494 C AluSg/x SINE/Alu 271 123**

**18104533 18104645 + AluY SINE/Alu 196 308**

**18104646 18104660 + AluY SINE/Alu 283 292**

**18105073 18105259 + L1MC4a LINE/L1 5989 6184**

**18106075 18106146 + MER66C LTR/ERV1 1 64**

**18106147 18106425 + AluSx SINE/Alu 1 280**

**18106426 18106728 + MER66C LTR/ERV1 65 487**

**18106819 18107008 + MER66B LTR/ERV1 281 475**

**18107017 18107266 + L2 LINE/L2 3141 3418**

**18107280 18107835 C L2 LINE/L2 2447 1810**

**18107837 18108080 + AluYa5 SINE/Alu 2 307**

**18108116 18108675 C L1MC4 LINE/L1 8042 7445**

**18108682 18109128 C L2 LINE/L2 1825 1394**

**18109129 18109429 + L1MB2 LINE/L1 5511 5823**

**18109430 18109731 + AluSx SINE/Alu 5 307**

**18109732 18109744 + AluSx SINE/Alu 284 296**

**18109745 18110087 + L1MB2 LINE/L1 5824 6168**

**18110088 18110337 C L2 LINE/L2 1393 1156**

**18110415 18110712 + MLT1H LTR/MaLR 73 367**

**18110729 18110786 C L1ME4a LINE/L1 5910 5853**

**18110787 18111082 + AluSx SINE/Alu 1 296**

**18111083 18111268 C L1ME4a LINE/L1 5852 5681**

**18111474 18111769 + AluSx SINE/Alu 1 286**

**18111781 18112185 + MLT1H LTR/MaLR 105 532**

**18112249 18112336 + L1MCa LINE/L1 3 97**

**18112337 18112618 + AluJo SINE/Alu 1 294**

**18112619 18112886 + L1MCa LINE/L1 98 339**

**18112887 18113191 C AluSx SINE/Alu 300 1**

**18113192 18113272 + L1MCa LINE/L1 340 415**

**18113275 18114011 + L1MC1 LINE/L1 5586 6333**

**18114020 18114317 + AluY SINE/Alu 2 301**

**18114322 18114659 + L1MD1 LINE/L1 3038 3390**

**18114722 18114808 + AluJ/FRAM SINE/Alu 218 302**

**18114811 18115274 + L1MD1 LINE/L1 4437 4905**

**__________________________________________________________________________________**

**AluYa5_20_27c 18540670-18540977 C_INTER_RMD**

**18530168 18535583 + L1PA2 LINE/L1 614 6031**

**18535585 18535841 C L1MC1 LINE/L1 3976 3697**

**18535842 18536102 C AluSx SINE/Alu 266 1**

**18536103 18536345 C L1MC1 LINE/L1 3696 3453**

**18536346 18536657 C AluSx SINE/Alu 312 1**

**18536658 18537036 C L1MC1 LINE/L1 3452 3126**

**18537037 18537093 C U6 snRNA 57 1**

**18537094 18537244 C L1MC1 LINE/L1 3125 2996**

**18537266 18537382 C MER33 DNA/MER1_type 126 1**

**18537644 18537666 + AT_rich Low_complexity 1 23**

**18537790 18538091 C AluJb SINE/Alu 297 5**

**18538906 18539033 C FLAM_A SINE/Alu 130 1**

**18539272 18539571 + AluJo SINE/Alu 1 298**

**18540483 18540504 + AT_rich Low_complexity 1 22**

**18540512 18540667 C AluSg/x SINE/Alu 287 132 (R2)**

**18540670 18540977 C AluYa5 SINE/Alu 308 1 ( AluYa5_20_27c ) (R1)**

**18541021 18541237 C MIR SINE/MIR 251 23**

**18541291 18541602 C AluSx SINE/Alu 312 1**

**18541857 18541955 C L2 LINE/L2 3342 3245**

**18542636 18542748 C AluJb SINE/Alu 116 1**

**18542835 18542927 + MER81 DNA/AcHobo 21 114**

**18543443 18543532 + CT-rich Low_complexity 4 93**

**18543605 18543883 C MLT1G LTR/MaLR 289 19**

**18545157 18545457 + AluJo SINE/Alu 8 289**

**18545963 18546051 + (TA)n Simple_repeat 2 98**

**18546055 18546219 C L1M4 LINE/L1 4850 4685**

**18546324 18546959 C L1MC4a LINE/L1 6031 5352**

**18546982 18547166 C AluJ SINE/Alu 312 133**

**18547181 18547309 + (TA)n Simple_repeat 1 132**

**18547317 18547357 + (CA)n Simple_repeat 2 42**

**18547363 18547656 C AluSq SINE/Alu 294 1**

**18547657 18547788 C AluJ SINE/Alu 134 1**

**18547811 18548061 C L1MC4a LINE/L1 5288 5046**

**Ortholog in Chimp 18814532-18814604 Minus Nscore 0.00**

**18807371 18809119 C L1MC1 LINE/L1 5691 3977**

**18809120 18809414 + AluSx SINE/Alu 1 296**

**18809415 18809672 C L1MC1 LINE/L1 3976 3697**

**18809673 18809933 C AluSx SINE/Alu 266 1**

**18809934 18810175 C L1MC1 LINE/L1 3696 3453**

**18810176 18810484 C AluSx SINE/Alu 309 1**

**18810485 18810857 C L1MC1 LINE/L1 3452 3129**

**18810858 18810914 C U6 snRNA 57 1**

**18810915 18811065 C L1MC1 LINE/L1 3128 2998**

**18811087 18811203 C MER33 DNA/MER1_type 126 1**

**18811465 18811488 + AT_rich Low_complexity 1 24**

**18811612 18811912 C AluJb SINE/Alu 297 5**

**18812761 18812885 C FLAM_A SINE/Alu 127 1**

**18813124 18813423 + AluJo SINE/Alu 1 298**

**18814334 18814354 + AT_rich Low_complexity 1 21**

**18814362 18814650 C AluSg SINE/Alu 285 1 (R12)**

**18814694 18814910 C MIR SINE/MIR 251 23**

**18814964 18815275 C AluSx SINE/Alu 312 1**

**18815529 18815627 C L2 LINE/L2 3342 3245**

**18816307 18816419 C AluJb SINE/Alu 116 1**

**18816506 18816598 + MER81 DNA/AcHobo 21 114**

**18817114 18817149 + (TCCC)n Simple_repeat 2 37**

**18817181 18817205 + (TTCC)n Simple_repeat 2 26**

**18817225 18817275 + CT-rich Low_complexity 3 53**

**18817276 18817554 C MLT1G LTR/MaLR 289 19**

**18818832 18819136 + AluJo SINE/Alu 10 290**

**18819642 18819730 + (TA)n Simple_repeat 2 98**

**18819734 18819898 C L1M4 LINE/L1 4850 4685**

**18820003 18820649 C L1MC4a LINE/L1 6031 5338**

**18820661 18820832 C AluJ SINE/Alu 299 133**

**18820847 18821007 + (TA)n Simple_repeat 1 164**

**18821009 18821303 C AluSq SINE/Alu 295 1**

**18821305 18821435 C AluJ SINE/Alu 131 1**

**18821436 18821709 C L1MC4a LINE/L1 5308 5046**

**__________________________________________________________________________________**

**AluYa5_20_59 41319943-41320256 C_INTER_RMD_M_DISRUPTED**

**41317138 41317296 + L1PA11 LINE/L1 6022 6173**

**41317297 41317362 C MIRb SINE/MIR 69 4**

**41317385 41317478 C L3 LINE/CR1 4152 4053**

**41317681 41317742 C L1MB7 LINE/L1 5950 5888**

**41317755 41317885 + L1MB7 LINE/L1 5990 6122**

**41317886 41318022 C L1MB3 LINE/L1 6182 6041**

**41318023 41318071 C MLT2D LTR/ERVL 560 512**

**41318082 41318548 C Tigger3(Golem) DNA/MER2_type 3028 2561**

**41318644 41318924 C AluSq SINE/Alu 306 1**

**41318928 41318980 + L1MB7 LINE/L1 6114 6165**

**41319012 41319139 + L1MC4 LINE/L1 7522 7641**

**41319263 41319647 + MLT1J LTR/MaLR 103 511**

**41319670 41319845 + MIR SINE/MIR 94 262**

**41319943 41320256 + AluYa5 SINE/Alu 1 310 ( AluYa5_20_59 ) (R1)**

**41320257 41320437 + AluSc SINE/Alu 123 303 (R2)**

**41320548 41320660 + MIRb SINE/MIR 5 118**

**41320663 41320757 + MSTA LTR/MaLR 2 100**

**41320758 41321293 + MLT2A2 LTR/ERVL 1 553**

**41321295 41321598 + MSTA LTR/MaLR 105 428**

**41321599 41321718 + MIR SINE/MIR 109 236**

**41321844 41321916 + (TGGA)n Simple_repeat 4 76**

**41322097 41322397 C AluSc SINE/Alu 301 1**

**41322996 41323100 C MIRb SINE/MIR 154 36**

**Ortholog in Chimp 40569291-40569361 Plus Nscore 0.00**

**40566436 40566594 + L1PA11 LINE/L1 6022 6173**

**40566595 40566660 C MIRb SINE/MIR 69 4**

**40566683 40566776 C L3 LINE/CR1 4152 4053**

**40566978 40567039 C L1MB7 LINE/L1 5950 5888**

**40567043 40567182 + L1MB7 LINE/L1 5980 6122**

**40567183 40567319 C L1MB3 LINE/L1 6182 6041**

**40567320 40567364 C MLT2D LTR/ERVL 560 516**

**40567379 40567842 C Tigger3(Golem) DNA/MER2_type 3028 2561**

**40567897 40567933 C L1M2 LINE/L1 5599 5564**

**40567938 40568225 C AluSq SINE/Alu 313 1**

**40568229 40568281 + L1MB7 LINE/L1 6114 6165**

**40568313 40568438 + L1MC4 LINE/L1 7522 7641**

**40568562 40568946 + MLT1J LTR/MaLR 103 511**

**40568970 40569145 + MIR SINE/MIR 94 262**

**40569243 40569561 + AluSc SINE/Alu 1 319 (R12)**

**40569681 40569786 + MIR SINE/MIR 5 111**

**40569789 40569883 + MSTA LTR/MaLR 2 100**

**40569884 40570419 + MLT2A2 LTR/ERVL 1 553**

**40570421 40570724 + MSTA LTR/MaLR 105 428**

**40570725 40570844 + MIR SINE/MIR 109 236**

**40570970 40571026 + (TGGA)n Simple_repeat 4 60**

**40571208 40571502 C AluSc SINE/Alu 295 1**

**40572103 40572207 C MIRb SINE/MIR 154 36**

**__________________________________________________________________________________**

**AluYa5_20_69 47363772-47364008 C_INTER_RMD_M_DISRUPTED**

**47357615 47357913 C AluSx SINE/Alu 302 3**

**47357952 47357972 + AT_rich Low_complexity 1 21**

**47358019 47358123 C AluJb SINE/Alu 105 2**

**47358140 47358434 C AluY SINE/Alu 296 1**

**47358437 47358546 C AluY SINE/Alu 296 187**

**47358558 47358570 C L1MA10 LINE/L1 6246 6234**

**47358571 47359382 C L1ME1 LINE/L1 6074 5259**

**47359551 47359811 + MLT1K LTR/MaLR 5 279**

**47359828 47360068 + L2 LINE/L2 3096 3359**

**47360087 47360156 C L2 LINE/L2 2642 2577**

**47360166 47360191 + MIRm SINE/MIR 250 275**

**47360211 47360334 C MIRb SINE/MIR 139 15**

**47360374 47360413 C L2 LINE/L2 3376 3334**

**47360414 47360971 C MLT1F2 LTR/MaLR 562 2**

**47360972 47361386 C L2 LINE/L2 3333 2925**

**47361387 47361671 + AluSx SINE/Alu 1 303**

**47361672 47362042 C L2 LINE/L2 2924 2466**

**47362043 47362069 + (TTTTA)n Simple_repeat 3 29**

**47362070 47362093 C L2 LINE/L2 2465 2461**

**47362094 47362402 C AluJb SINE/Alu 308 1**

**47362403 47362723 C L2 LINE/L2 2460 2118**

**47362732 47363092 + MLT1G LTR/MaLR 3 373**

**47363772 47364008 + AluYa5 SINE/Alu 1 236 ( AluYa5_20_69 )**

**47364009 47364311 + AluJb SINE/Alu 17 321**

**47364581 47364871 + AluSq SINE/Alu 1 291**

**47364887 47365188 C L1MC4a LINE/L1 5923 5621**

**47365189 47365485 C AluJo SINE/Alu 311 23**

**47365486 47365724 C L1MC4a LINE/L1 5620 5365**

**47365725 47365869 C HAL1 LINE/L1 2389 2245**

**47365919 47366229 C AluSq SINE/Alu 313 1**

**47366231 47366292 + MER113 DNA/MER1_type 326 387**

**47366303 47367291 C L1MC1 LINE/L1 6331 5323**

**47367292 47367597 C AluSq SINE/Alu 303 1**

**47367598 47367755 C L1M LINE/L1 5323 5186**

**47367773 47367903 + MER113 DNA/MER1_type 388 521**

**47369104 47369291 + MER53 DNA 1 193**

**47369475 47369533 C MIR SINE/MIR 90 27**

**47369727 47369880 C FRAM SINE/Alu 154 1**

**47370100 47370414 + AluJo SINE/Alu 1 315**

**47370429 47370644 C MIRm SINE/MIR 262 41**

**Ortholog in Chimp 46749998-46750211 Plus Nscore 0.00**

**46743894 46744192 C AluSx SINE/Alu 302 3**

**46744298 46744402 C AluJb SINE/Alu 105 2**

**46744419 46744711 C AluY SINE/Alu 295 1**

**46744723 46744823 C AluY SINE/Alu 287 187**

**46744835 46744847 C L1MA10 LINE/L1 6246 6234**

**46744848 46745659 C L1ME1 LINE/L1 6074 5259**

**46745829 46746087 + MLT1K LTR/MaLR 7 279**

**46746104 46746344 + L2 LINE/L2 3096 3359**

**46746363 46746432 C L2 LINE/L2 2642 2577**

**46746442 46746467 + MIRm SINE/MIR 250 275**

**46746487 46746612 C MIRb SINE/MIR 139 10**

**46746651 46746690 C L2 LINE/L2 3376 3334**

**46746691 46747248 C MLT1F2 LTR/MaLR 562 2**

**46747249 46747663 C L2 LINE/L2 3333 2927**

**46747664 46747950 + AluSg SINE/Alu 1 303**

**46747951 46748318 C L2 LINE/L2 2926 2460**

**46748319 46748345 + (TTTTA)n Simple_repeat 3 29**

**46748346 46748366 C L2 LINE/L2 2459 2460**

**46748367 46748678 C AluJb SINE/Alu 311 1**

**46748679 46748999 C L2 LINE/L2 2459 2118**

**46749008 46749539 + MLT1G LTR/MaLR 3 555**

**46750024 46750258 + AluSq SINE/Alu 1 235 (OCCUPIED)**

**46750259 46750523 + AluJb SINE/Alu 17 281**

**46751298 46751588 + AluSq SINE/Alu 1 291**

**46751607 46751918 C L1MC4a LINE/L1 5923 5612**

**46751919 46752206 C AluJo SINE/Alu 304 21**

**46752207 46752435 C L1MC4a LINE/L1 5611 5366**

**46752436 46752580 C HAL1 LINE/L1 2389 2245**

**46752630 46752940 C AluSq SINE/Alu 313 1**

**46753018 46754007 C L1MC1 LINE/L1 6331 5323**

**46754008 46754305 C AluSq SINE/Alu 297 1**

**46754306 46754463 C L1M LINE/L1 5323 5186**

**46754481 46754611 + MER113 DNA/MER1_type 388 521**

**46755811 46755998 + MER53 DNA 1 193**

**46756433 46756586 C FRAM SINE/Alu 154 1**

**46756804 46757116 + AluJo SINE/Alu 1 311**

**46757131 46757346 C MIRm SINE/MIR 262 41**

**__________________________________________________________________________________**

**AluYa5_20_71c 48828055-48828362 C_INTER_RMD_M_DISRUPTED**

**48824426 48824551 C L1ME1 LINE/L1 6161 6031**

**48824607 48824825 C AluJo SINE/Alu 274 56**

**48824826 48825129 C AluSq SINE/Alu 304 2**

**48825130 48825175 C AluJo SINE/Alu 55 17**

**48825177 48825509 C L1M LINE/L1 5122 4788**

**48825520 48825936 + (TA)n Simple_repeat 1 391**

**48825942 48826227 C AluSx SINE/Alu 288 5**

**48826302 48826758 C MLT1D LTR/MaLR 501 3**

**48826770 48826948 C FRAM SINE/Alu 166 1**

**48826953 48826990 + (TTA)n Simple_repeat 2 40**

**48826991 48827272 C AluSx SINE/Alu 285 1**

**48827516 48827762 C L1M LINE/L1 3422 3167**

**48827770 48827889 + (TTCC)n Simple_repeat 4 123**

**48827891 48828054 C AluSc SINE/Alu 289 124 (R2)**

**48828055 48828362 C AluYa5 SINE/Alu 305 1 ( AluYa5_20_71c ) (R1)**

**48828364 48828476 C L1M LINE/L1 3167 3039**

**48828477 48828787 C AluY SINE/Alu 311 1**

**48828788 48828942 C L1M LINE/L1 3038 2869**

**48828943 48829248 + AluJb SINE/Alu 1 303**

**48829249 48829531 C L1M LINE/L1 2869 2530**

**48829758 48829809 C AluYc5 SINE/Alu 291 239**

**48829805 48829853 + AluJ/FLAM SINE/Alu 11 58**

**48829855 48830111 C AluSq SINE/Alu 257 1**

**48830112 48830267 C L1MD LINE/L1 4584 4426**

**48830586 48830882 + AluSq SINE/Alu 2 302**

**48830883 48831047 + AluSg/x SINE/Alu 134 298**

**48831048 48831387 + L1MB8 LINE/L1 5819 6177**

**48831390 48831504 C L2 LINE/L2 3371 3270**

**48831546 48831632 C AluJ/FLAM SINE/Alu 88 2**

**48831677 48831729 + MER5A1 DNA/MER1_type 85 136**

**Ortholog in Chimp 48257371-48257440 Minus Nscore 0.00**

**48253302 48253427 C L1ME1 LINE/L1 6161 6031**

**48253473 48253701 C AluJo SINE/Alu 292 56**

**48253702 48254001 C AluSq SINE/Alu 300 2**

**48254002 48254047 C AluJo SINE/Alu 55 17**

**48254049 48254380 C L1MC LINE/L1 5122 4788**

**48254384 48254480 + (TA)n Simple_repeat 2 101**

**48254481 48254675 C L1MC5 LINE/L1 7770 7572**

**48254676 48254748 + (TA)n Simple_repeat 1 73**

**48254749 48255036 C AluSx SINE/Alu 287 1**

**48255108 48255564 C MLT1D LTR/MaLR 501 3**

**48255576 48255748 C AluJo SINE/Alu 302 135**

**48255759 48255799 + (TTA)n Simple_repeat 2 43**

**48255800 48256081 C AluSx SINE/Alu 285 1**

**48256392 48256560 C L1P3 LINE/L1 3347 3179**

**48256580 48256650 + (TTCC)n Simple_repeat 1 71**

**48257201 48257489 C AluSc SINE/Alu 289 1 (R12)**

**48257492 48257602 C L1M5 LINE/L1 3166 3032**

**48257603 48257913 C AluY SINE/Alu 311 1**

**48257914 48258067 C L1M5 LINE/L1 3031 2870**

**48258068 48258373 + AluJo SINE/Alu 1 303**

**48258374 48258656 C L1M5 LINE/L1 2869 2531**

**48258883 48258935 C AluYc5 SINE/Alu 292 239**

**48258931 48258979 + AluJ/FLAM SINE/Alu 11 58**

**48258981 48259237 C AluSq SINE/Alu 257 1**

**48259712 48260008 + AluSq SINE/Alu 2 302**

**48260009 48260173 + AluSg/x SINE/Alu 134 298**

**48260174 48260513 + L1MB8 LINE/L1 5819 6177**

**48260516 48260630 C L2 LINE/L2 3411 3293**

**48260672 48260758 C AluJ/FLAM SINE/Alu 88 2**

**48260765 48261066 C AluSx SINE/Alu 301 1**

**48261110 48261162 + MER5A1 DNA/MER1_type 85 136**

**__________________________________________________________________________________**

**AluYa5_20_78 54197322-54197632 C_INTER_RMD_M_DISRUPTED**

**54191479 54191639 + L1MC5 LINE/L1 7276 7441**

**54192271 54192388 + L1MC4 LINE/L1 7281 7399**

**54192571 54192769 + MIRb SINE/MIR 3 227**

**54193471 54193892 + MSTA LTR/MaLR 4 428**

**54194430 54194722 + AluSx SINE/Alu 7 298**

**54194761 54194802 C Charlie8 DNA/MER1_type 1428 1387**

**54194799 54195342 C Charlie8 DNA/MER1_type 529 2**

**54195361 54195560 C MER91B DNA/Tip100 190 1**

**54197058 54197321 + L1PB2 LINE/L1 5495 5785**

**54197322 54197632 + AluYa5 SINE/Alu 1 308 ( AluYa5_20_78 ) (R1)**

**54197636 54197798 + AluSp SINE/Alu 137 299 (R2)**

**54197802 54198177 + L1PB2 LINE/L1 5769 6148**

**54198245 54198369 + MIR SINE/MIR 45 192**

**54198668 54198716 C MIRb SINE/MIR 136 88**

**54198766 54198931 C AluSx SINE/Alu 168 3**

**54198933 54199072 + AluSg/x SINE/Alu 168 307**

**54201465 54201786 C MLT1D LTR/MaLR 504 117**

**54202297 54202593 C AluSx SINE/Alu 298 1**

**54203048 54203076 + (TAAA)n Simple_repeat 4 33**

**54203108 54203460 + Zaphod DNA/Tip100 118 520**

**54203462 54203533 C MLT2B1 LTR/ERVL 562 497**

**54203536 54203592 + (TAGA)n Simple_repeat 2 60**

**54203597 54204094 C MLT2B1 LTR/ERVL 532 1**

**54204095 54204441 + Zaphod DNA/Tip100 517 853**

**54204528 54204616 + Charlie10 DNA/MER1_type 5 101**

**Ortholog in Chimp 53806073-53806154 Plus Nscore 0.00**

**53800186 53800347 + L1MC5 LINE/L1 7276 7441**

**53800977 53801094 + L1MC4 LINE/L1 7281 7399**

**53801275 53801524 + MIRb SINE/MIR 2 274**

**53802172 53802593 + MSTA LTR/MaLR 4 428**

**53803127 53803426 + AluSx SINE/Alu 3 301**

**53803465 53803506 C Charlie8 DNA/MER1_type 1428 1387**

**53803503 53803975 C Charlie8 DNA/MER1_type 529 48**

**53804062 53804261 C MER91B DNA/Tip100 190 1**

**53805762 53806025 + L1PB2 LINE/L1 5495 5785**

**53806026 53806338 + AluSp SINE/Alu 1 312 (R12)**

**53806339 53806717 + L1PB2 LINE/L1 5786 6148**

**53806783 53806907 + MIR SINE/MIR 45 192**

**53807209 53807257 C MIRb SINE/MIR 136 88**

**53807307 53807472 C AluSx SINE/Alu 168 3**

**53807474 53807613 + AluSg/x SINE/Alu 168 307**

**53809993 53810314 C MLT1D LTR/MaLR 504 117**

**53810824 53811122 C AluSx SINE/Alu 300 1**

**53811577 53811605 + (TAAA)n Simple_repeat 4 33**

**53811646 53811989 + Zaphod DNA/Tip100 127 520**

**53811991 53812078 C MLT2D LTR/ERVL 562 469**

**53812102 53812600 C MLT2B1 LTR/ERVL 532 1**

**53812601 53812947 + Zaphod DNA/Tip100 517 853**

**53813034 53813122 + Charlie10 DNA/MER1_type 5 101**

**__________________________________________________________________________________**

**AluYa5_22_10c 23726152-23726459 C_INTER_RMD_M_DISRUPTED**

**23722419 23722472 + MIRm SINE/MIR 3 62**

**23722473 23722884 C LTR16A1 LTR/ERVL 457 1**

**23722885 23723017 + MIR3 SINE/MIR 52 196**

**23723043 23723262 C L2 LINE/L2 3378 3126**

**23723691 23723993 C L1ME3 LINE/L1 6151 5841**

**23724014 23724085 C Tigger5 DNA/MER2_type 2399 2324**

**23724086 23724395 + AluJb SINE/Alu 1 298**

**23724405 23724706 + AluY SINE/Alu 1 303**

**23724783 23725168 C L1ME3 LINE/L1 5853 5443**

**23725223 23725494 C AluJb SINE/Alu 279 1**

**23725529 23725826 C AluJb SINE/Alu 301 1**

**23725856 23726149 C AluY SINE/Alu 299 1 (R2)**

**23726152 23726459 C AluYa5 SINE/Alu 308 1 ( AluYa5_22_10c ) (R1)**

**23726460 23726481 + AT_rich Low_complexity 1 22**

**23726495 23726784 C AluSx SINE/Alu 298 1**

**23726785 23726901 C AluJo/FLAM SINE/Alu 128 13**

**23726906 23727139 C L1ME3 LINE/L1 5441 5222**

**23727150 23727254 + (TG)n Simple_repeat 1 105**

**23727256 23727370 C L1ME3 LINE/L1 5240 5120**

**23727860 23727912 + AT_rich Low_complexity 1 53**

**23727934 23727973 + (CATATA)n Simple_repeat 2 41**

**23728232 23728500 C LTR16C LTR/ERVL 491 152**

**23728752 23729039 C AluJb SINE/Alu 295 1**

**23729142 23729297 + MIRb SINE/MIR 103 262**

**Ortholog in Chimp 23636603-23636672 Minus Nscore 0.00**

**23631318 23631552 C MIRb SINE/MIR 273 17**

**23632150 23632561 C LTR16A1 LTR/ERVL 457 1**

**23632562 23632694 + MIR3 SINE/MIR 52 196**

**23632720 23632937 C L2 LINE/L2 3419 3126**

**23633366 23633500 C L1ME3 LINE/L1 6151 6012**

**23634292 23634469 C L1ME3 LINE/L1 6020 5841**

**23634490 23634561 C Tigger5 DNA/MER2_type 2399 2324**

**23634562 23634871 + AluJb SINE/Alu 1 298**

**23634881 23635185 + AluY SINE/Alu 1 305**

**23635262 23635646 C L1ME3 LINE/L1 5853 5442**

**23635680 23635699 + (GA)n Simple_repeat 2 21**

**23635704 23635975 C AluJb SINE/Alu 279 1**

**23636005 23636311 C AluJb SINE/Alu 310 1**

**23636341 23636652 C AluY SINE/Alu 313 1 (R12)**

**23636655 23636676 + AT_rich Low_complexity 1 22**

**23636718 23637000 C AluSq SINE/Alu 261 1**

**23637001 23637116 C AluJo/FLAM SINE/Alu 127 13**

**23637121 23637354 C L1ME3 LINE/L1 5441 5222**

**23637358 23637462 + (TATG)n Simple_repeat 4 108**

**23637465 23637578 C L1ME3 LINE/L1 5240 5121**

**23638078 23638183 + (TA)n Simple_repeat 2 108**

**23638442 23638710 C LTR16C LTR/ERVL 491 152**

**23638962 23639261 C AluJb SINE/Alu 308 1**

**23639364 23639516 + MIRb SINE/MIR 110 268**

**__________________________________________________________________________________**

**AluYa5_22_19 34228318-34228478 C_INTER_RMD_M_DISRUPTED**

**34224536 34224647 + MIR SINE/MIR 145 256**

**34225263 34225560 + AluY SINE/Alu 1 300**

**34225569 34225710 + AluSq/x SINE/Alu 1 136**

**34225717 34225772 + MIR SINE/MIR 151 205**

**34225808 34225862 C MIR3 SINE/MIR 203 147**

**34226023 34226752 + L1MA5 LINE/L1 4126 4856**

**34226864 34227175 C AluSx SINE/Alu 312 1**

**34227356 34227657 + AluSg SINE/Alu 6 307**

**34227731 34227754 + AT_rich Low_complexity 1 24**

**34228318 34228478 + AluYa5 SINE/Alu 150 310 ( AluYa5_22_19 )**

**34228480 34228535 + (TA)n Simple_repeat 2 57**

**34229661 34230076 + L1MA5 LINE/L1 4842 5270**

**34230078 34230162 + AluJ/FLAM SINE/Alu 1 88**

**34230163 34231178 + L1MA5 LINE/L1 5265 6298**

**34231501 34231650 C L1ME1 LINE/L1 6161 6010**

**34231912 34232354 C L1ME1 LINE/L1 6011 5573**

**34233001 34233306 C AluSx SINE/Alu 306 1**

**34233364 34233697 + LTR9 LTR/ERV1 1 300**

**Ortholog in Chimp 34440622-34440741 Plus Nscore 0.00**

**34436797 34436908 + MIR SINE/MIR 145 256**

**34437524 34437830 + AluY SINE/Alu 1 309**

**34437838 34437979 + AluSq/x SINE/Alu 1 136**

**34437986 34438041 + MIR SINE/MIR 151 205**

**34438077 34438131 C MIR3 SINE/MIR 203 147**

**34438082 34438148 C L3b LINE/CR1 4502 4437**

**34438294 34439023 + L1MA5 LINE/L1 4126 4856**

**34439132 34439438 C AluSx SINE/Alu 307 1**

**34439619 34439909 + AluSg SINE/Alu 6 296**

**34439984 34440007 + AT_rich Low_complexity 1 24**

**34440573 34440733 + AluYa5 SINE/Alu 150 310 (OCCUPIED)**

**34440734 34440758 + AT_rich Low_complexity 1 25**

**34441887 34442302 + L1MA5 LINE/L1 4842 5270**

**34442304 34442389 + AluJ/FLAM SINE/Alu 1 88**

**34442390 34443405 + L1MA5 LINE/L1 5265 6298**

**34443728 34443877 C L1ME1 LINE/L1 6161 6010**

**34444227 34444581 C L1ME1 LINE/L1 5958 5573**

**34445227 34445533 C AluSx SINE/Alu 307 1**

**34445591 34445926 + LTR9 LTR/ERV1 1 300**

**34445927 34446214 + AluSx SINE/Alu 1 289**

**34446215 34446483 + LTR9 LTR/ERV1 301 600**

**34446671 34446795 C FLAM_C SINE/Alu 126 2**

**34447669 34447759 + L2 LINE/L2 3290 3383**

**__________________________________________________________________________________**

**AluYa5_22_28 41344207-41344513 C_INTER_RMD**

**41337111 41337273 C FRAM SINE/Alu 163 1**

**41337576 41337856 + AluSx SINE/Alu 34 312**

**41338018 41338142 + L2 LINE/L2 2729 2856**

**41338146 41338392 C MIR SINE/MIR 262 8**

**41338497 41338795 + AluSx SINE/Alu 1 302**

**41339429 41339736 + AluSx SINE/Alu 1 308**

**41340078 41340228 + MIRb SINE/MIR 74 218**

**41340318 41340410 + MIRb SINE/MIR 100 193**

**41341785 41341859 + L2 LINE/L2 3335 3411**

**41341954 41342277 + AluSx SINE/Alu 1 312**

**41342298 41342361 C FRAM/FAM SINE/Alu 63 1**

**41342513 41342811 C AluSx SINE/Alu 298 1**

**41342813 41342921 + MER20B DNA/MER1_type 614 732**

**41342941 41343240 + AluSx SINE/Alu 1 298**

**41343381 41343411 + MIRb SINE/MIR 40 70**

**41343727 41344020 + L2 LINE/L2 2938 3253**

**41344207 41344513 + AluYa5 SINE/Alu 2 308 ( AluYa5_22_28 ) (R1)**

**41344515 41344684 + AluSg/x SINE/Alu 135 309 (R2)**

**41346126 41346353 C MIRm SINE/MIR 225 6**

**41346691 41347001 + L4 LINE/RTE 1637 1958**

**41347134 41347169 + (TG)n Simple_repeat 2 37**

**41347170 41347330 C FAM SINE/Alu 163 1**

**41347883 41348169 C AluSc SINE/Alu 289 1**

**41348824 41349155 + Charlie8 DNA/MER1_type 49 396**

**41349471 41349645 + MIRb SINE/MIR 1 194**

**41350373 41350534 + L2 LINE/L2 3031 3198**

**41351052 41351174 C L1ME4a LINE/L1 6065 5937**

**41351175 41351461 + AluSq SINE/Alu 1 285**

**41351462 41351478 C L1ME4a LINE/L1 5936 5919**

**Ortholog in Chimp 41698211-41698282 Plus Nscore 0.00**

**41691166 41691446 + AluSx SINE/Alu 34 312**

**41691608 41691732 + L2 LINE/L2 2729 2856**

**41691736 41691982 C MIR SINE/MIR 262 8**

**41692087 41692387 + AluSx SINE/Alu 1 302**

**41693021 41693328 + AluSx SINE/Alu 1 308**

**41693646 41693833 + MIRb SINE/MIR 74 259**

**41693886 41693978 + MIRm SINE/MIR 100 193**

**41694142 41694193 + GC_rich Low_complexity 1 52**

**41695352 41695426 + L2 LINE/L2 3335 3411**

**41695521 41695653 + AluSq/x SINE/Alu 1 133**

**41696475 41696774 C AluSx SINE/Alu 298 1**

**41696776 41696884 + MER20B DNA/MER1_type 614 732**

**41696904 41697203 + AluSx SINE/Alu 1 298**

**41697246 41697374 C MIRb SINE/MIR 151 24**

**41697665 41697935 + L2 LINE/L2 2922 3205**

**41698161 41698467 + AluSx SINE/Alu 1 312 (R12)**

**41699915 41700142 C MIRm SINE/MIR 225 6**

**41700483 41700794 + L4 LINE/RTE 1637 1958**

**41700933 41700954 + (TG)n Simple_repeat 2 23**

**41700955 41701115 C FAM SINE/Alu 162 2**

**41701668 41701955 C AluSc SINE/Alu 289 1**

**41702613 41702941 + Charlie8 DNA/MER1_type 49 396**

**41703256 41703421 + MIRb SINE/MIR 1 185**

**41704328 41704350 + GC_rich Low_complexity 1 23**

**41704869 41704991 C L1ME4a LINE/L1 6065 5937**

**41704992 41705278 + AluSq SINE/Alu 1 285**

**41705279 41705295 C L1ME4a LINE/L1 5936 5919**

**__________________________________________________________________________________**

**AluYa5_22_31 46413625-46413888 C_INTER_RMD_M_DISRUPTED**

**46409436 46409842 + MLT2D LTR/ERVL 1 412**

**46410261 46410325 + L2 LINE/L2 3356 3416**

**46410347 46410386 + (GA)n Simple_repeat 2 41**

**46410407 46410624 + MIRb SINE/MIR 16 268**

**46411315 46411677 + MLT1A0 LTR/MaLR 1 365**

**46411710 46412093 + MLT1A LTR/MaLR 1 374**

**46412525 46413204 + L1MC4 LINE/L1 764 1713**

**46413625 46413888 + AluYa5 SINE/Alu 54 310 ( AluYa5_22_31 )**

**46413890 46416124 + L1MC4 LINE/L1 2008 4334**

**46416136 46416160 + (GA)n Simple_repeat 2 26**

**46416211 46417235 + L1MDa LINE/L1 19 1311**

**46417236 46417543 C AluSx SINE/Alu 308 1**

**46417544 46417709 + L1MDa LINE/L1 1312 1477**

**46417710 46417735 + (CAA)n Simple_repeat 2 27**

**46417736 46418473 + L1MDa LINE/L1 1478 2220**

**46418474 46418518 C MADE1 DNA/Mariner 46 1**

**46418519 46418959 + L1MDa LINE/L1 2221 2664**

**46418956 46419372 + L1MDa LINE/L1 3106 3540**

**46419386 46419576 + L1MD LINE/L1 4540 4733**

**46419580 46419803 C L1MB2 LINE/L1 6168 5939**

**46419804 46419901 C Tigger7 DNA/MER2_type 2490 2393**

**46419900 46420151 C Tigger7 DNA/MER2_type 267 1**

**46420153 46420646 C L1MB2 LINE/L1 5940 5451**

**46420646 46421185 + L1MD1 LINE/L1 5707 6241**

**Ortholog in Chimp 46863106-46863383 Plus Nscore 0.00**

**46858874 46859279 + MLT2D LTR/ERVL 1 412**

**46859787 46859822 + (GA)n Simple_repeat 2 37**

**46860755 46861117 + MLT1A0 LTR/MaLR 1 365**

**46861150 46861533 + MLT1A LTR/MaLR 1 374**

**46862123 46862644 + L1MC4 LINE/L1 1147 1713**

**46863065 46863308 + AluY SINE/Alu 54 299 (OCCUPIED)**

**46863572 46865585 + L1MC4 LINE/L1 2177 4309**

**46865692 46866715 + L1MDa LINE/L1 19 1311**

**46866716 46867031 C AluSx SINE/Alu 316 1**

**46867032 46867953 + L1MDa LINE/L1 1312 2226**

**46867954 46868001 C MADE1 DNA/Mariner 49 1**

**46868002 46868443 + L1MDa LINE/L1 2227 2664**

**46868440 46868865 + L1MDa LINE/L1 3106 3539**

**46868880 46869070 + L1MD LINE/L1 4540 4733**

**46869074 46869297 C L1MB2 LINE/L1 6168 5939**

**46869298 46869395 C Tigger7 DNA/MER2_type 2490 2393**

**46869394 46869645 C Tigger7 DNA/MER2_type 267 1**

**46869647 46870140 C L1MB2 LINE/L1 5940 5451**

**46870140 46870679 + L1MD1 LINE/L1 5707 6241**

**__________________________________________________________________________________**

**AluYa5_X_90 99479713-99480018 C_INTER_RMD_M_DISRUPTED**

**99473197 99473580 + THE1D LTR/MaLR 1 381**

**99473894 99474184 + AluSx SINE/Alu 1 298**

**99474384 99474684 + AluJo SINE/Alu 1 312**

**99474686 99474904 + GA-rich Low_complexity 1 220**

**99475430 99475743 C AluSx SINE/Alu 312 1**

**99475753 99475783 + AT_rich Low_complexity 1 31**

**99475898 99475990 + L1MA6 LINE/L1 6105 6199**

**99476410 99476452 + (TG)n Simple_repeat 2 43**

**99476460 99476531 + (TA)n Simple_repeat 2 73**

**99477399 99477447 + L2 LINE/L2 3368 3419**

**99479371 99479605 + L1MA6 LINE/L1 6065 6300**

**99479713 99480018 + AluYa5 SINE/Alu 1 304 ( AluYa5_X_90 ) (R1)**

**99480019 99480197 + AluSg/x SINE/Alu 126 304 (R2)**

**99481563 99481596 + (TG)n Simple_repeat 2 35**

**99481847 99481894 + (CATA)n Simple_repeat 2 50**

**99484068 99484335 C MIR SINE/MIR 262 2**

**99484515 99485027 + MLT2B1 LTR/ERVL 1 520**

**99485780 99486392 + L4 LINE/RTE 661 1361**

**99486393 99486592 C MER20 DNA/MER1_type 219 1**

**99486593 99486613 + L4 LINE/RTE 1362 1383**

**99486621 99486841 + MIRb SINE/MIR 17 256**

**99486864 99487217 + L4 LINE/RTE 1440 1831**

**Ortholog in Chimp 99916731-99916802 Plus Nscore 0.00**

**99909714 99910097 + THE1D LTR/MaLR 1 381**

**99910411 99910700 + AluSx SINE/Alu 1 297**

**99910899 99911064 + AluJo SINE/Alu 1 179**

**99911810 99911867 + (GAAA)n Simple_repeat 4 61**

**99912389 99912699 C AluSx SINE/Alu 311 1**

**99912709 99912739 + AT_rich Low_complexity 1 31**

**99912854 99912968 + L1MA6 LINE/L1 6105 6224**

**99913376 99913422 + (TG)n Simple_repeat 2 47**

**99913430 99913497 + (TA)n Simple_repeat 2 69**

**99916340 99916574 + L1MA6 LINE/L1 6065 6300**

**99916682 99916973 + AluSx SINE/Alu 1 302 (R12)**

**99918342 99918373 + (TG)n Simple_repeat 2 33**

**99918623 99918669 + (CATA)n Simple_repeat 3 50**

**99920845 99921112 C MIR SINE/MIR 262 2**

**99921289 99921801 + MLT2B1 LTR/ERVL 1 520**

**99922554 99922801 + L4 LINE/RTE 661 981**

**99923306 99923394 C MER20 DNA/MER1_type 121 36**

**99923440 99923660 + MIRb SINE/MIR 17 256**

**99923683 99924049 + L4 LINE/RTE 1440 1844**

**__________________________________________________________________________________**

**AluYa5_X_109c 120135833-120136135 C_INTER_RMD_M_DISRUPTED**

**120132739 120133626 C L1MB4 LINE/L1 5097 4222**

**120133619 120133794 C L1MB4 LINE/L1 1776 1212**

**120133861 120133995 + (TA)n Simple_repeat 1 144**

**120133997 120134111 C L1MCa LINE/L1 1959 1839**

**120134170 120134192 + AT_rich Low_complexity 1 23**

**120134354 120134416 C L1MB8 LINE/L1 6127 6065**

**120134417 120134724 C MSTD LTR/MaLR 396 19**

**120134730 120135455 C L1MB7 LINE/L1 6064 5323**

**120135456 120135517 C Alu SINE/Alu 303 242**

**120135638 120135825 C AluY SINE/Alu 306 119 (R2)**

**120135833 120136135 C AluYa5 SINE/Alu 308 6 ( AluYa5_X_109c ) (R1)**

**120136142 120136726 C L1MB7 LINE/L1 5331 4735**

**120136823 120137106 C AluSq SINE/Alu 294 12**

**120137188 120137364 + MLT1D LTR/MaLR 255 437**

**120137387 120137766 C L1PB2 LINE/L1 6206 5843**

**120137767 120138035 C AluY SINE/Alu 310 48**

**120138036 120139236 C L1PB2 LINE/L1 5842 4623**

**120139231 120140899 C L1PB2 LINE/L1 1723 8**

**Ortholog in Chimp 120577402-120577466 Minus Nscore 0.00**

**120574309 120575197 C L1MB4 LINE/L1 5097 4222**

**120575190 120575369 C L1MB4 LINE/L1 1382 1192**

**120575459 120575629 + (TA)n Simple_repeat 2 179**

**120575630 120575682 + AT_rich Low_complexity 1 53**

**120575731 120575753 + AT_rich Low_complexity 1 23**

**120575915 120575977 C L1MB8 LINE/L1 6127 6065**

**120575978 120576285 C MSTD LTR/MaLR 396 19**

**120576291 120577016 C L1MB7 LINE/L1 6064 5323**

**120577017 120577080 C Alu SINE/Alu 305 242**

**120577201 120577520 C AluY SINE/Alu 306 1 (R12)**

**120577522 120578095 C L1MB7 LINE/L1 5331 4747**

**120578203 120578487 C AluSq SINE/Alu 295 12**

**120578569 120578745 + MLT1D LTR/MaLR 255 437**

**120578768 120579130 C L1PB2 LINE/L1 6206 5843**

**120579131 120579399 C AluY SINE/Alu 310 48**

**120579400 120580600 C L1PB2 LINE/L1 5842 4623**

**120580595 120581548 C L1PB2 LINE/L1 1723 728**

**__________________________________________________________________________________**

**AluYa5_1_199c 153681028-153681331 INDEL_CAN (M_INTER_RMD)**

**153677870 153678180 C AluY SINE/Alu 307 1**

**153678181 153678480 C AluJb SINE/Alu 300 1**

**153678550 153678636 + MLT1G3 LTR/MaLR 458 542**

**153678721 153679030 + AluSq SINE/Alu 1 313**

**153679182 153679357 + L2 LINE/L2 3002 3188**

**153679838 153679870 C L1MA10 LINE/L1 6332 6300**

**153679871 153680394 C L1ME1 LINE/L1 6216 5690**

**153680395 153680696 C AluSx SINE/Alu 304 1**

**153680697 153680999 C L1ME1 LINE/L1 5689 5366**

**153681028 153681331 C AluYa5 SINE/Alu 302 1 ( AluYa5_1_199c ) (R12)**

**153681338 153681611 C L1ME1 LINE/L1 5206 4922**

**153681620 153681899 C AluSq SINE/Alu 279 1**

**153681900 153681932 + AT_rich Low_complexity 1 33**

**153681946 153682041 C AluJ/FLAM SINE/Alu 99 2**

**153682043 153682275 C L1ME1 LINE/L1 4730 4540**

**153682276 153682302 + (TG)n Simple_repeat 2 28**

**153682306 153682598 C AluJb SINE/Alu 302 1**

**153682599 153682715 C L1ME1 LINE/L1 4539 4441**

**153682716 153683013 C AluJb SINE/Alu 300 1**

**153683014 153683026 C L1ME1 LINE/L1 4440 4441**

**153683027 153683316 C AluY SINE/Alu 309 2**

**153683317 153683786 C L1ME1 LINE/L1 4440 3914**

**153683804 153683944 C AluSq/x SINE/Alu 141 1**

**153683945 153684737 C L1M1 LINE/L1 5495 4668**

**Ortholog in Chimp 134612051-134612777 Minus Nscore 0.00**

**N positions**

**134608982 134609290 C AluY SINE/Alu 305 1**

**134609291 134609585 C AluJb SINE/Alu 295 1**

**134609655 134609741 + MLT1J LTR/MaLR 504 590**

**134609743 134609813 + L2 LINE/L2 2738 2810**

**134609890 134610061 + AluSq SINE/Alu 124 295**

**134610211 134610326 + L2 LINE/L2 3002 3117**

**134610900 134611421 C L1ME1 LINE/L1 6143 5622**

**134611422 134611719 C AluSx SINE/Alu 300 1**

**134611720 134612022 C L1ME1 LINE/L1 5621 5292**

**134612051 134612355 C AluSx SINE/Alu 303 1 (R2)**

**134612359 134612636 C AluJb SINE/Alu 296 33**

**134612658 134612723 C AluJ/FLAM SINE/Alu 70 7 (R1)**

**134612737 134613057 C L1ME1 LINE/L1 5187 4849**

**134613066 134613345 C AluSq SINE/Alu 279 1**

**134613346 134613378 + AT_rich Low_complexity 1 33**

**134613392 134613487 C AluJ/FLAM SINE/Alu 99 2**

**134613489 134613721 C L1ME1 LINE/L1 4657 4467**

**134613722 134613744 + (TG)n Simple_repeat 2 24**

**134613748 134614039 C AluJb SINE/Alu 302 1**

**134614040 134614155 C L1ME1 LINE/L1 4466 4340**

**134614156 134614449 C AluJb SINE/Alu 296 1**

**134614450 134614471 C L1MEc LINE/L1 4440 4431**

**134614472 134614771 C AluY SINE/Alu 311 2**

**134614772 134615236 C L1MEc LINE/L1 4430 3908**

**134615254 134615394 C AluSq/x SINE/Alu 141 1**

**134615395 134616186 C L1M1 LINE/L1 5495 4669**

**__________________________________________________________________________________**

**AluYa5_1_315c 223328317-223328623 INDEL_CAN (C_INTER_RMD)**

**223323430 223325926 C L1PA12 LINE/L1 5696 3165**

**223325927 223326291 C THE1C LTR/MaLR 375 1**

**223326292 223328002 C L1PA12 LINE/L1 3164 1272**

**223328003 223328305 C AluSc SINE/Alu 308 1 (R12)**

**223328306 223328316 C L1PA12 LINE/L1 1271 1167**

**223328317 223328623 C AluYa5 SINE/Alu 307 1 ( AluYa5_1_315c ) (R1)**

**223328624 223329770 C L1PA12 LINE/L1 1166 4**

**223329789 223329908 C L1M3c LINE/L1 2168 2047**

**223329909 223330019 + FLAM_A SINE/Alu 2 125**

**223330020 223330874 C L1M3c LINE/L1 2053 7**

**223330879 223330924 + MIR SINE/MIR 192 236**

**223331095 223331119 + L1PA6 LINE/L1 5834 5856**

**223331120 223331323 C L1PA6 LINE/L1 6154 5950**

**223331324 223331414 + L1PA6 LINE/L1 5857 5947**

**223331409 223331790 C L1M5 LINE/L1 3991 3569**

**Ortholog in Chimp 205692620-205692632 Minus Nscore 0.00**

**N positions**

**205685877 205690234 C L1PA12 LINE/L1 8155 3725**

**205690235 205690596 C THE1C LTR/MaLR 375 1**

**205690597 205692316 C L1PA12 LINE/L1 3724 1126**

**205692317 205692620 C AluSc SINE/Alu 309 1 (R12)**

**205692621 205693767 C L1PA12 LINE/L1 1160 1**

**205693786 205693905 C L1M3c LINE/L1 2168 2047**

**205693906 205694017 + FLAM_A SINE/Alu 2 126**

**205694018 205694896 C L1M3c LINE/L1 2053 7**

**205694901 205694946 + MIR SINE/MIR 192 236**

**205695117 205695141 + L1PA6 LINE/L1 5834 5856**

**205695142 205695345 C L1PA6 LINE/L1 6154 5950**

**205695346 205695428 + L1PA6 LINE/L1 5857 5947**

**205695423 205695804 C L1M5 LINE/L1 3991 3569**

**__________________________________________________________________________________**

**AluYa5_1_338 235245413-235245713 INDEL_CAN (C_INTER_RMD)**

**235242202 235242438 C MIRb SINE/MIR 257 20**

**235242498 235242536 + CT-rich Low_complexity 2 41**

**235243458 235243479 + (TGAA)n Simple_repeat 1 22**

**235243610 235243782 + MLT1C LTR/MaLR 8 184**

**235243783 235244074 + AluJo SINE/Alu 2 284**

**235244145 235244586 C L1MEc LINE/L1 5378 4932**

**235244587 235244705 + FLAM_A SINE/Alu 1 120**

**235244706 235245016 C AluSq SINE/Alu 297 1**

**235245017 235245175 C L1MEc LINE/L1 4931 4785**

**235245176 235245386 C AluJb SINE/Alu 311 87**

**235245387 235245412 C L1MEc LINE/L1 4784 4761**

**235245413 235245713 + AluYa5 SINE/Alu 1 301 ( AluYa5_1_338 ) (R1)**

**235245723 235246014 + AluSx SINE/Alu 1 297 (R2)**

**235246015 235246563 C L1MEc LINE/L1 4759 4185**

**235246564 235247145 + LTR49 LTR/ERV1 1 593**

**235247146 235248002 C L1MEc LINE/L1 4184 3414**

**235248003 235248300 C AluSx SINE/Alu 299 1**

**235248301 235249384 C L1MD LINE/L1 3340 2172**

**Ortholog in Chimp 217872038-217872048 Plus Nscore 0.00**

**N positions**

**217868839 217869075 C MIRb SINE/MIR 257 20**

**217869135 217869165 + (TC)n Simple_repeat 2 33**

**217870087 217870108 + (TGAA)n Simple_repeat 1 22**

**217870239 217870412 + MLT1C LTR/MaLR 8 184**

**217870413 217870704 + AluJo SINE/Alu 2 284**

**217870774 217871215 C L1MEc LINE/L1 5378 4932**

**217871216 217871334 + FLAM_A SINE/Alu 1 120**

**217871335 217871650 C AluSq SINE/Alu 309 1**

**217871651 217871809 C L1MEc LINE/L1 4931 4788**

**217871810 217872018 C AluJb SINE/Alu 309 87**

**217872019 217872047 C L1MEc LINE/L1 4787 4762**

**217872048 217872339 + AluSx SINE/Alu 1 297 (R12)**

**217872340 217872888 C L1MEc LINE/L1 4759 4185**

**217872889 217873469 + LTR49 LTR/ERV1 1 593**

**217873470 217874326 C L1MEc LINE/L1 4184 3414**

**217874327 217874624 C AluSx SINE/Alu 299 1**

**217874625 217876143 C L1MEc LINE/L1 3413 1886**

**__________________________________________________________________________________**

**AluYa5_2_55c 38806863-38807171 INDEL_CAN (M_INTER_RMD)**

**38803834 38804152 + MER65C LTR/ERV1 1 323**

**38804153 38804465 + AluJo SINE/Alu 1 311**

**38804466 38804569 + MER65C LTR/ERV1 324 397**

**38804570 38804864 C AluSx SINE/Alu 296 1**

**38804865 38804927 + MER65C LTR/ERV1 398 461**

**38805900 38806176 + AluJb SINE/Alu 2 277**

**38806186 38806211 + (CA)n Simple_repeat 2 27**

**38806227 38806290 + FRAM/FAM SINE/Alu 13 76**

**38806354 38806651 C AluSx SINE/Alu 312 7**

**38806863 38807171 C AluYa5 SINE/Alu 309 1 ( AluYa5_2_55c ) (R12)**

**38807274 38807551 + AluSq SINE/Alu 1 292**

**38807596 38807881 + AluSx SINE/Alu 17 302**

**38808093 38808399 + AluSg SINE/Alu 1 309**

**38808758 38809050 + AluSx SINE/Alu 1 294**

**38809061 38809361 + AluSx SINE/Alu 1 313**

**38809668 38809800 C MER2B DNA/MER2_type 333 201**

**38809844 38810008 C AluJb SINE/Alu 292 123**

**38810009 38810036 + (TTTA)n Simple_repeat 2 29**

**38810037 38810079 C AluJb SINE/Alu 122 82**

**Ortholog in Chimp 39590429-39591420 Minus Nscore 0.00**

**N positions**

**39587387 39587705 + MER65C LTR/ERV1 1 323**

**39587706 39588018 + AluJo SINE/Alu 1 312**

**39588019 39588117 + MER65C LTR/ERV1 324 397**

**39588118 39588412 C AluSx SINE/Alu 296 1**

**39588413 39588475 + MER65C LTR/ERV1 398 461**

**39589449 39589725 + AluJb SINE/Alu 2 277**

**39589735 39589771 + (CA)n Simple_repeat 2 38**

**39589788 39589851 + FRAM/FAM SINE/Alu 13 76**

**39589914 39590217 C AluSx SINE/Alu 312 7**

**39590524 39590643 + L1MC4 LINE/L1 2011 2139**

**39590644 39590913 C AluJb SINE/Alu 305 37 (R2)**

**39590914 39590951 + L1MC4 LINE/L1 2140 2173**

**39591012 39591043 + (TA)n Simple_repeat 2 33**

**39591118 39591420 C AluJo SINE/Alu 300 1 (R1)**

**39591523 39591801 + AluSq SINE/Alu 1 293**

**39591846 39592133 + AluSx SINE/Alu 17 304**

**39592345 39592651 + AluSg SINE/Alu 1 309**

**39593010 39593305 + AluSx SINE/Alu 1 297**

**39593314 39593614 + AluSx SINE/Alu 1 313**

**39593922 39594054 C MER2B DNA/MER2_type 333 201**

**39594097 39594258 C AluJb SINE/Alu 292 129**

**39594262 39594301 + AT_rich Low_complexity 1 40**

**__________________________________________________________________________________**

**AluYa5_2_121c 77211459-77211767 INDEL_CAN (C_INTER_RMD)**

**77208497 77208801 + AluSg SINE/Alu 1 305**

**77209030 77209056 + (TAGA)n Simple_repeat 2 28**

**77209283 77209569 C AluSq SINE/Alu 284 5**

**77211145 77211444 C AluY SINE/Alu 300 1 (R2)**

**77211459 77211767 C AluYa5 SINE/Alu 309 1 ( AluYa5_2_121c ) (R1)**

**77211774 77211796 + AT_rich Low_complexity 1 23**

**77211830 77211949 C MIR SINE/MIR 241 111**

**77212442 77212498 C MLT1A1 LTR/MaLR 408 364**

**77212499 77213103 C L1MA4A LINE/L1 6301 5680**

**77213104 77213454 C MLT1A1 LTR/MaLR 363 6**

**77213479 77213561 + GA-rich Low_complexity 5 87**

**Ortholog in Chimp 78797934-78797949 Minus Nscore 0.00**

**N positions**

**78795053 78795083 + (TAGA)n Simple_repeat 2 32**

**78795311 78795480 C AluSq SINE/Alu 283 121**

**78795542 78795568 + (TAGA)n Simple_repeat 2 28**

**78795796 78796077 C AluSq SINE/Alu 283 5**

**78797635 78797934 C AluY SINE/Alu 300 1 (R12)**

**78797947 78797969 + AT_rich Low_complexity 1 23**

**78798003 78798122 C MIR SINE/MIR 241 111**

**78798615 78798671 C MLT1A1 LTR/MaLR 408 364**

**78798672 78799276 C L1MA4A LINE/L1 6302 5680**

**78799277 78799627 C MLT1A1 LTR/MaLR 363 6**

**78799652 78799734 + GA-rich Low_complexity 5 87**

**78800849 78800927 + (TA)n Simple_repeat 2 82**

**___________________________________________________________________________________________**

**AluYa5_4_256c 184540695-184541003 INDEL_CAN (C_INTER_RMD)**

**184537419 184537704 + AluY SINE/Alu 9 294**

**184537719 184537861 + MER21B LTR/ERV1 322 492**

**184538090 184538209 + MLT1C LTR/MaLR 178 314**

**184538276 184538363 + AluJ/FLAM SINE/Alu 2 89**

**184540369 184540679 C AluSg SINE/Alu 309 1 (R2)**

**184540695 184541003 C AluYa5 SINE/Alu 309 1 ( AluYa5_4_256c ) (R1)**

**184541439 184541504 C L2 LINE/L2 3347 3275**

**184541657 184543529 + MER52A LTR/ERV1 1 1755**

**184543607 184543690 C L1PA13 LINE/L1 5799 5716**

**184543691 184544055 + L1PA13 LINE/L1 5797 6163**

**Ortholog in Chimp 188033002-188033018 Minus Nscore 0.00**

**N positions**

**188030090 188030209 + MLT1A0 LTR/MaLR 178 314**

**188030275 188030318 + Alu SINE/Alu 1 44**

**188032694 188033002 C AluSg SINE/Alu 309 1 (R12)**

**188033433 188033498 C L2 LINE/L2 3347 3275**

**188033651 188035527 + MER52A LTR/ERV1 1 1755**

**188035605 188035688 C L1P4 LINE/L1 5799 5716**

**188035689 188036050 + L1PREC2 LINE/L1 5797 6160**

**__________________________________________________________________________________**

**AluYa5_5_240 176319042-176319344 INDEL_CAN (C_INTER_RMD)**

**176317421 176317526 + L3 LINE/CR1 4384 4488**

**176317905 176318086 + MIRb SINE/MIR 19 216**

**176319042 176319344 + AluYa5 SINE/Alu 1 303 ( AluYa5_5_240 ) (R1)**

**176319357 176319651 + AluSp SINE/Alu 1 295 (R2)**

**176319652 176319859 C MIRb SINE/MIR 251 27**

**176320216 176321244 + SVA Other 1 868**

**176321258 176322777 + SVA Other 433 1386**

**Ortholog in Chimp 179326881-179326894 Plus Nscore 0.00**

**N positions**

**179325246 179325357 + L3 LINE/CR1 4376 4488**

**179325721 179325949 + MIRb SINE/MIR 1 252**

**179326676 179326725 + GA-rich Low_complexity 4 55**

**179326894 179327188 + AluSp SINE/Alu 1 295 (R12)**

**179327189 179327396 C MIRb SINE/MIR 251 27**

**179327778 179328072 + AluSx SINE/Alu 1 295**

**179328846 179329198 + L1ME4a LINE/L1 5494 5921**

**179329672 179329970 + AluY SINE/Alu 1 297**

**__________________________________________________________________________________**

**AluYa5_6_54 34679123-34679395 INDEL_CAN (M_INTER_RMD)**

**34675373 34676144 + L2 LINE/L2 2267 3081**

**34676624 34676836 + MIRb SINE/MIR 3 219**

**34676840 34676963 + HAL1 LINE/L1 2385 2507**

**34676976 34677028 + MIRb SINE/MIR 208 263**

**34678986 34679120 + AluSq/x SINE/Alu 1 135**

**34679123 34679395 + AluYa5 SINE/Alu 16 288 ( AluYa5_6_54 ) (R12)**

**34679500 34679559 + L2 LINE/L2 3225 3284**

**34680780 34681091 + AluSq SINE/Alu 1 313**

**34681101 34681413 + AluSx SINE/Alu 1 312**

**Ortholog in Chimp 35342398-35343918 Plus Nscore 0.00**

**N positions**

**35338649 35339420 + L2 LINE/L2 2267 3081**

**35339900 35340121 + MIRb SINE/MIR 3 228**

**35340150 35340239 + HAL1 LINE/L1 2418 2507**

**35340252 35340304 + MIRb SINE/MIR 208 263**

**35342261 35342574 + AluSx SINE/Alu 1 312 (R1)**

**35342984 35343086 + MIRb SINE/MIR 53 158**

**35343630 35343921 + AluJo SINE/Alu 1 292 (R2)**

**35344023 35344082 + L2 LINE/L2 3225 3284**

**35345256 35345567 + AluSq SINE/Alu 1 313**

**35345572 35345884 + AluSx SINE/Alu 1 312**

**__________________________________________________________________________________**

**AluYa5_6_61c 42061341-42061644 INDEL_CAN (C_INTER_RMD)**

**42058006 42058763 + L1MC1 LINE/L1 4325 5094**

**42058769 42058811 C L1MA9 LINE/L1 6306 6263**

**42058812 42059101 + AluSq SINE/Alu 1 299**

**42059102 42059268 C L1MA9 LINE/L1 6262 6085**

**42059269 42059579 + AluJb SINE/Alu 1 299**

**42059580 42060054 C L1MA9 LINE/L1 6084 5610**

**42060055 42060362 C AluSq SINE/Alu 308 2**

**42060363 42061020 C L1MA9 LINE/L1 5609 4999**

**42061021 42061330 C AluSq SINE/Alu 309 1 (R2)**

**42061341 42061644 C AluYa5 SINE/Alu 304 1 ( AluYa5_6_61c ) (R1)**

**42061645 42062556 C L1MA9 LINE/L1 4991 4068**

**42063194 42063234 + (TC)n Simple_repeat 2 42**

**42063235 42063272 + (TG)n Simple_repeat 2 40**

**42063273 42063517 C AluJo SINE/Alu 286 1**

**42063750 42064047 + AluSp SINE/Alu 1 304**

**42064343 42064407 + L1MC1 LINE/L1 5080 5145**

**42064408 42064714 C AluY SINE/Alu 306 1**

**Ortholog in Chimp 42945970-42945981 Minus Nscore 0.00**

**N positions**

**42942864 42943152 + AluSq SINE/Alu 1 298**

**42943153 42943204 C L1MA9 LINE/L1 6262 6199**

**42943742 42943892 C L1MA9 LINE/L1 6231 6084**

**42943893 42944203 + AluJb SINE/Alu 1 299**

**42944204 42944678 C L1MA9 LINE/L1 6083 5609**

**42944679 42945004 C AluSq SINE/Alu 308 2**

**42945005 42945662 C L1MA9 LINE/L1 5608 5007**

**42945663 42945970 C AluSq SINE/Alu 307 1 (R12)**

**42945971 42946879 C L1MA9 LINE/L1 5006 4068**

**42947517 42947551 + (TC)n Simple_repeat 2 36**

**42947552 42947581 + (TG)n Simple_repeat 2 32**

**42947582 42947616 C AluJo SINE/Alu 274 240**

**42947622 42947828 C AluJo SINE/Alu 206 1**

**42948061 42948364 + AluSp SINE/Alu 1 310**

**42948716 42948779 + L1MC1 LINE/L1 5080 5144**

**42948780 42949088 C AluY SINE/Alu 308 1**

**__________________________________________________________________________________**

**AluYa5_7_10c 8130267-8130573 INDEL_CAN (M_INTER_RMD)**

**8127426 8127455 + (CAGC)n Simple_repeat 3 32**

**8127465 8127579 C L2 LINE/L2 3353 3234**

**8127580 8128063 + L1MA3 LINE/L1 5819 6304**

**8128064 8128322 C L2 LINE/L2 3233 3025**

**8129987 8130228 + MIR SINE/MIR 13 262**

**8130267 8130573 C AluYa5 SINE/Alu 308 1 ( AluYa5_7_10c ) (R12)**

**8130943 8131241 C AluSx SINE/Alu 297 3**

**8131300 8131911 + L2 LINE/L2 2786 3417**

**8133133 8133446 C AluSx SINE/Alu 297 1**

**Ortholog in Chimp 8139014-8140018 Minus Nscore 0.00**

**N positions**

**8136209 8136347 C L2 LINE/L2 3378 3234**

**8136348 8136830 + L1MA3 LINE/L1 5819 6304**

**8136831 8137089 C L2 LINE/L2 3233 3025**

**8138206 8138309 + GA-rich Low_complexity 3 105**

**8138734 8138975 + MIR SINE/MIR 13 262**

**8139085 8139236 C MIR3 SINE/MIR 156 2 (R2)**

**8139245 8139546 C AluJb SINE/Alu 294 1**

**8139725 8140018 C AluSc SINE/Alu 289 1 (R1)**

**8140383 8140679 C AluSx SINE/Alu 295 3**

**8140738 8141348 + L2 LINE/L2 2786 3417**

**8142572 8142890 C AluSx SINE/Alu 296 1**

**__________________________________________________________________________________**

**__________________________________________________________________________________**

**AluYa5_10_79c 64454907-64455216 INDEL_CAN (C_INTER_RMD)**

**64452139 64452685 + MER41A LTR/ERV1 1 552**

**64453376 64453922 C MER41A LTR/ERV1 554 1**

**64454201 64454284 + L2 LINE/L2 3331 3415**

**64454404 64454579 + FRAM SINE/Alu 1 173**

**64454593 64454883 C AluSx SINE/Alu 305 6 (R2)**

**64454907 64455216 C AluYa5 SINE/Alu 310 1 ( AluYa5_10_79c ) (R1)**

**64455223 64455347 C FLAM_C SINE/Alu 124 1**

**64455385 64455674 + L1MEe LINE/L1 366 672**

**64455721 64457423 + L1MEe LINE/L1 775 2490**

**64457436 64457737 C MLT1F1 LTR/MaLR 303 1**

**64457847 64457951 + L1M5 LINE/L1 5486 5589**

**64458038 64458302 + MLT1J LTR/MaLR 82 368**

**Ortholog in Chimp 61921249-61921264 Minus Nscore 0.00**

**N positions**

**61918480 61919027 + MER41A LTR/ERV1 1 552**

**61919712 61920285 C MER41A LTR/ERV1 554 1**

**61920564 61920647 + L2 LINE/L2 3331 3415**

**61920767 61920942 + FRAM SINE/Alu 1 173**

**61920958 61921244 C AluSx SINE/Alu 301 6 (R12)**

**61921259 61921383 C FLAM_C SINE/Alu 124 1**

**61921421 61923461 + L1MEe LINE/L1 366 2501**

**61923465 61923766 C MLT1F1 LTR/MaLR 303 1**

**61923779 61923986 + L1M5 LINE/L1 5350 5592**

**61924067 61924331 + MLT1J LTR/MaLR 82 368**

**__________________________________________________________________________________**

**AluYa5_11_77 61907370-61907679 INDEL_CAN (C_INTER_RMD)**

**61900174 61905147 C HERVK LTR/ERVK 5717 743**

**61905219 61905977 C HERVK LTR/ERVK 758 1**

**61905978 61906054 C LTR5B LTR/ERVK 1002 926**

**61906055 61906186 + AluJo/FLAM SINE/Alu 1 132**

**61906187 61906247 + AluYd8 SINE/Alu 121 181**

**61906249 61907139 C LTR5_Hs LTR/ERVK 891 1**

**61907146 61907369 + Zaphod DNA/Tip100 3415 3639**

**61907370 61907679 + AluYa5 SINE/Alu 1 310 ( AluYa5_11_77 ) (R1)**

**61907688 61907977 + AluSx SINE/Alu 1 298 (R2)**

**61907978 61908048 + Zaphod DNA/Tip100 3648 3732**

**61908049 61908101 + (TG)n Simple_repeat 2 54**

**61908102 61908190 + Zaphod DNA/Tip100 3733 3822**

**61908191 61908270 + AluY SINE/Alu 216 295**

**61908273 61908558 + AluSg1 SINE/Alu 3 286**

**61908562 61908681 + L1MA9 LINE/L1 6126 6251**

**61908682 61908978 + AluSx SINE/Alu 1 297**

**61908979 61908994 + L1MA9 LINE/L1 6252 6267**

**61909030 61909159 + Zaphod DNA/Tip100 3882 4031**

**61909393 61909518 C FLAM_A SINE/Alu 124 1**

**61909631 61909927 + AluSq SINE/Alu 1 298**

**61909930 61910237 + AluSg SINE/Alu 1 308**

**61910255 61910289 + Alu SINE/Alu 51 85**

**61910295 61910308 C L1M5 LINE/L1 5733 5720**

**61910309 61910658 + MER44A DNA/MER2_type 1 339**

**61910659 61910927 C L1M5 LINE/L1 5719 5445**

**Ortholog in Chimp 60759577-60759586 Plus Nscore 0.00**

**N positions**

**60752380 60757361 C HERVK LTR/ERVK 5721 743**

**60757437 60758195 C HERVK LTR/ERVK 758 1**

**60758196 60758272 C LTR5B LTR/ERVK 1002 926**

**60758273 60758404 + AluJo/FLAM SINE/Alu 1 132**

**60758405 60758465 + AluYd8 SINE/Alu 121 181**

**60758467 60759363 C LTR5_Hs LTR/ERVK 891 1**

**60759370 60759585 + Zaphod DNA/Tip100 3415 3651**

**60759586 60759874 + AluSx SINE/Alu 1 297 (R12)**

**60759875 60759946 + Zaphod DNA/Tip100 3652 3728**

**60759947 60759999 + (TG)n Simple_repeat 2 54**

**60760000 60760088 + Zaphod DNA/Tip100 3729 3823**

**60760089 60760168 + AluY SINE/Alu 216 295**

**60760169 60760457 + AluSg1 SINE/Alu 1 287**

**60760461 60760580 + L1MA9 LINE/L1 6126 6251**

**60760581 60760877 + AluSx SINE/Alu 1 297**

**60760878 60760893 + L1MA9 LINE/L1 6252 6267**

**60760929 60761058 + Zaphod DNA/Tip100 3882 4031**

**60761094 60761175 C L1M5 LINE/L1 5460 5372**

**60761276 60761411 C FLAM_A SINE/Alu 130 1**

**60761528 60761824 + AluSq SINE/Alu 1 298**

**60761827 60762134 + AluSg SINE/Alu 1 308**

**60762152 60762186 + Alu SINE/Alu 51 85**

**60762192 60762205 C L1M5 LINE/L1 5733 5720**

**60762206 60762510 + MER44A DNA/MER2_type 1 339**

**60762511 60762784 C L1M5 LINE/L1 5719 5445**

**__________________________________________________________________________________**

**AluYa5_17_40 25134929-25135237 INDEL_CAN (C_INTER_RMD)**

**25131956 25132254 C AluY SINE/Alu 301 1**

**25132613 25132921 C AluSg SINE/Alu 309 1**

**25132928 25132992 + (TTCTC)n Simple_repeat 5 66**

**25132994 25133288 C AluSx SINE/Alu 292 1**

**25133446 25133539 C L2 LINE/L2 3373 3256**

**25133551 25133615 + L2 LINE/L2 2708 2775**

**25133665 25133728 C LTR8 LTR/ERV1 589 524**

**25133730 25134029 C AluSx SINE/Alu 300 1**

**25134030 25134164 C AluSq/x SINE/Alu 135 1**

**25134171 25134362 C LTR8A LTR/ERV1 577 383**

**25134363 25134395 C MLT2A1 LTR/ERVL 550 499**

**25134396 25134484 + (TA)n Simple_repeat 1 94**

**25134485 25134857 C MLT2A1 LTR/ERVL 390 2**

**25134858 25134927 C LTR8A LTR/ERV1 382 314**

**25134929 25135237 + AluYa5 SINE/Alu 1 309 ( AluYa5_17_40 ) (R1)**

**25135249 25135540 + AluSx SINE/Alu 1 294 (R2)**

**25135584 25135759 + AluSg/x SINE/Alu 126 299**

**25135760 25135809 C LTR8A LTR/ERV1 320 271**

**25136171 25136299 + L3b LINE/CR1 4172 4309**

**25136636 25136942 C AluSg SINE/Alu 304 1**

**25136950 25137247 C AluY SINE/Alu 298 1**

**25137386 25137708 + MER61A LTR/ERV1 1 343**

**25137814 25138069 + AluJo SINE/Alu 1 284**

**25138113 25138875 C L1MB3 LINE/L1 6182 5399**

**Ortholog in Chimp 27523914-27523926 Minus Nscore 0.00**

**N positions**

**27520241 27521004 + L1MB3 LINE/L1 5399 6182**

**27521048 27521303 C AluJo SINE/Alu 284 1**

**27521410 27521732 C MER61A LTR/ERV1 343 1**

**27521871 27522177 + AluY SINE/Alu 1 307**

**27522185 27522490 + AluSg SINE/Alu 1 304**

**27522827 27522955 C L3b LINE/CR1 4309 4172**

**27523343 27523392 + LTR8A LTR/ERV1 271 320**

**27523393 27523581 C AluSg/x SINE/Alu 312 126**

**27523625 27523914 C AluSx SINE/Alu 292 1 (R12)**

**27523915 27523983 + LTR8A LTR/ERV1 313 382**

**27523984 27524438 + MLT2A1 LTR/ERVL 1 543**

**27524452 27524645 + LTR8 LTR/ERV1 385 534**

**27524647 27524781 + AluSq/x SINE/Alu 1 135**

**27524782 27525080 + AluSx SINE/Alu 1 299**

**27525082 27525145 + LTR8 LTR/ERV1 524 589**

**27525201 27525265 C L2 LINE/L2 2775 2708**

**27525277 27525370 + L2 LINE/L2 3256 3373**

**27525526 27525820 + AluSx SINE/Alu 1 292**

**27525822 27525886 + (GAGAA)n Simple_repeat 5 66**

**27525893 27526198 + AluSg SINE/Alu 1 306**

**27526557 27526864 + AluY SINE/Alu 1 310**

**__________________________________________________________________________________**

**AluYa5_1_226 166133576-166133887 INDEL_CAN (C_INTER_RMD)**

**166130501 166130762 + MER21B LTR/ERV1 434 714**

**166130765 166131059 + AluSc SINE/Alu 1 296**

**166131388 166131522 C L2 LINE/L2 2484 2352**

**166131586 166131658 C MER96 DNA/MER1_type? 82 10**

**166131676 166131990 C L2 LINE/L2 2302 1963**

**166132949 166133081 + L1MC4a LINE/L1 7300 7425**

**166133082 166133366 + AluSx SINE/Alu 17 290 (R1)**

**166133367 166133391 + (TA)n Simple_repeat 2 26**

**166133434 166133575 + L1MD3 LINE/L1 7417 7726**

**166133576 166133887 + AluYa5 SINE/Alu 1 309 ( AluYa5_1_226 ) (R2)**

**166133888 166134146 + L1MD3 LINE/L1 7727 7982**

**166134181 166134459 + AluSg1 SINE/Alu 1 278**

**166136402 166136693 C AluSq SINE/Alu 293 1**

**166136872 166136943 + MIR SINE/MIR 100 175**

**Ortholog in Chimp 147262767-147262907 Plus Nscore 0.00**

**N positions**

**147260103 147260399 + AluSc SINE/Alu 6 303**

**147260727 147260797 C L2 LINE/L2 2484 2412**

**147260925 147260997 C MER96 DNA/hAT 82 10**

**147261026 147261329 C L2 LINE/L2 2290 1963**

**147262290 147262424 + L1MC4a LINE/L1 7298 7425**

**147262425 147262729 + AluSx SINE/Alu 17 298 (R12)**

**147262730 147262752 + (TA)n Simple_repeat 1 23**

**147262753 147262780 + (GA)n Simple_repeat 2 30**

**147262781 147263166 + L1MC4a LINE/L1 7426 7808**

**147263201 147263479 + AluSg1 SINE/Alu 1 278**

**147265485 147265776 C AluSq SINE/Alu 293 1**

**__________________________________________________________________________________**

**Supplementary file 3 - Recombination mediated deletions – AluYa5 Human Vs Celera comparison**

Repeat masker annotation of the locus in the main genome and its flanks is listed first, followed by the Repeat masker annotation of the identified ortholog in the comparative genome and its flanks.

R1,R2 – PUTATIVE REGIONS OF HOMOLOGY WHICH RECOMBINE TO GIVE R12 (M_INTER_RMD or C_INTER_RMD).

In case of disruptions, R1 and R2 represent disrupted halves of the parent repeat, R represents undisrupted repeat and D represents the disruptive sequence. (M_DISRUPTED OR C_DISRUPTED).

In case of intra element recombination (M_INTRA_RMD or C_INTRA_RMD), R12 represent the 2 resulting copies arising out of intra element recombination and R represents the intact repeat.

**AluYa5_2_67c C_INTER_RMD**

**2398 7.9 0.0 0.0 chr2 43508318 43508620 (199442529) + AluY SINE/Alu 1 303 (8) 785**

**209 12.7 6.7 1.7 chr2 43508621 43508655 (199442494) + (TA)n Simple_repeat 2 37 (0) 786**

**2098 11.5 1.4 0.3 chr2 43508969 43509257 (199441892) + AluSx SINE/Alu 1 292 (20) 787**

**2264 10.9 0.3 0.0 chr2 43509751 43510044 (199441105) C AluSx SINE/Alu (12) 300 6 788**

**981 16.6 1.2 0.0 chr2 43510530 43510698 (199440451) + AluJo SINE/Alu 1 171 (141) 789**

**1104 19.3 0.5 2.3 chr2 43511335 43511551 (199439598) C MER20 DNA/MER1_type (6) 213 1 790**

**790 21.8 4.9 0.4 chr2 43511880 43512105 (199439044) C L1ME2 LINE/L1 (21) 6143 5908 791**

**1468 7.1 0.0 0.0 chr2 43512106 43512289 (199438860) C AluY SINE/Alu (9) 302 119 792 (R2)**

**1684 2.6 0.0 0.0 chr2 43512297 43512488 (199438661) C AluYa5 SINE/Alu (0) 310 119 793**

**2862 0.7 0.0 0.0 chr2 43512489 43512798 (199438351) C AluYa5 SINE/Alu (0) 310 1 794 (AluYa5_2_67c) (R1)**

**1097 7.5 0.0 0.0 chr2 43512799 43512931 (199438218) C AluY SINE/Alu (177) 134 2 793**

**1710 20.9 6.9 2.7 chr2 43512937 43513823 (199437326) C L1ME2 LINE/L1 (262) 5902 4930 791**

**2203 11.8 0.7 0.3 chr2 43513892 43514197 (199436952) + AluSx SINE/Alu 2 308 (4) 795**

**687 24.7 8.4 8.4 chr2 43514199 43514329 (199436820) + L1M5 LINE/L1 4508 4627 (1519) 796**

**2211 12.0 0.0 0.3 chr2 43514330 43514631 (199436518) C AluSx SINE/Alu (11) 301 1 797**

**687 24.7 8.4 8.4 chr2 43514632 43514870 (199436279) + L1M5 LINE/L1 4628 4878 (1268) 796**

**Ortholog in Celera**

**1043 16.6 1.2 0.0 gi|89161198:43496242-43500311 242 410 (3660) + AluJo SINE/Alu 1 171 (141) 1**

**1101 18.9 0.5 2.4 gi|89161198:43496242-43500311 1047 1263 (2807) C MER20 DNA/hAT-Charlie (6) 213 1 2**

**735 21.3 7.5 0.4 gi|89161198:43496242-43500311 1592 1817 (2253) C L1ME2z LINE/L1 (295) 6149 5908 3**

**2724 3.6 0.0 0.0 gi|89161198:43496242-43500311 1818 2119 (1951) C AluYa5 SINE/Alu (8) 302 1 4 (R12)**

**1116 6.8 0.0 0.0 gi|89161198:43496242-43500311 2120 2252 (1818) C AluYk11 SINE/Alu (178) 134 2 5**

**1535 21.1 12.3 2.9 gi|89161198:43496242-43500311 2274 3144 (926) C L1ME2z LINE/L1 (558) 5886 4930 3**

**2257 11.8 0.7 0.3 gi|89161198:43496242-43500311 3213 3518 (552) + AluSx SINE/Alu 2 308 (4) 6**

**643 20.3 5.1 10.2 gi|89161198:43496242-43500311 3520 3650 (420) + L1M5 LINE/L1 4508 4627 (1519) 7**

**2256 11.9 0.0 0.3 gi|89161198:43496242-43500311 3651 3952 (118) C AluSx SINE/Alu (11) 301 1 8**

**643 20.3 5.1 10.2 gi|89161198:43496242-43500311 3953 4056 (14) + L1M5 LINE/L1 4628 4732 (1414) 7**

**____________________________________________________________________________________**

**AluYa5_2_181 OCCUPIED**

**1909 18.0 0.0 0.3 chr2 118618185 118618480 (124332669) C AluJb SINE/Alu (17) 295 1 135**

**228 19.7 2.6 7.8 chr2 118618484 118618560 (124332589) C HAL1 LINE/L1 (810) 1697 1625 136**

**376 11.8 4.0 0.0 chr2 118618571 118618646 (124332503) + CT-rich Low_complexity 1 79 (0) 137**

**437 26.2 10.2 1.0 chr2 118618647 118618843 (124332306) C HAL1 LINE/L1 (844) 1663 1449 136**

**2348 4.9 0.0 0.7 chr2 118619099 118619388 (124331761) C L1PA8 LINE/L1 (0) 6172 5885 138**

**254 27.4 22.2 1.9 chr2 118619416 118619523 (124331626) C MARNA DNA/Mariner (438) 148 19 139**

**420 27.5 4.3 0.0 chr2 118619650 118619787 (124331362) C MIRb SINE/MIR (109) 159 16 140**

**1953 12.1 0.0 0.0 chr2 118621191 118621466 (124329683) + AluSg SINE/Alu 28 303 (0) 141**

**1932 20.3 6.6 1.6 chr2 118621785 118622224 (124328925) C MLT1C LTR/MaLR (0) 467 6 142**

**1311 0.7 0.0 0.0 chr2 118622610 118622752 (124328397) + AluYa5/8 SINE/Alu 168 310 (0) 143**

**204 6.7 0.0 0.0 chr2 118623312 118623341 (124327808) + (T)n Simple_repeat 1 30 (0) 144**

**1838 25.2 9.7 1.0 chr2 118624977 118625577 (124325572) C L1ME3A LINE/L1 (155) 6015 5363 145**

**364 34.6 10.4 3.2 chr2 118625644 118626019 (124325130) + L3 LINE/CR1 3874 4276 (213) 146**

**243 27.2 0.0 0.0 chr2 118627645 118627725 (124323424) + (CA)n Simple_repeat 1 81 (0) 147**

**195 32.8 3.3 0.0 chr2 118628764 118628824 (124322325) C L2 LINE/L2 (273) 3105 3043 148**

**189 31.9 0.0 0.0 chr2 118628854 118628900 (124322249) C MER5B DNA/MER1_type (6) 172 126 149**

**411 26.6 2.8 3.5 chr2 118628911 118629054 (124322095) + L1MEb LINE/L1 143 285 (5924) 150**

**1142 29.3 7.5 4.8 chr2 118629787 118631128 (124320021) + L1MEb LINE/L1 721 2103 (4398) 150**

**1468 24.2 12.7 0.8 chr2 118631134 118631653 (124319496) + L1M5 LINE/L1 4577 5158 (988) 151**

**17028 20.0 3.6 2.0 chr2 118631645 118634912 (124316237) + L1MEb LINE/L1 976 4341 (2035) 152**

**3133 15.2 8.4 3.7 chr2 118634913 118635252 (124315897) C LTR49 LTR/ERV1 (0) 595 231 153**

**1708 17.7 0.0 5.5 chr2 118635253 118635580 (124315569) C AluJo SINE/Alu (0) 312 3 154**

**Ortholog in Celera**

**381 27.9 4.7 0.0 gi|89161198:112230569-112236651 1 129 (5954) C MIRb SINE/MIR (118) 150 16 1**

**1954 12.1 0.0 0.0 gi|89161198:112230569-112236651 1533 1808 (4275) + AluSg SINE/Alu 28 310 (0) 2**

**1913 20.3 6.6 1.5 gi|89161198:112230569-112236651 2127 2566 (3517) C MLT1C LTR/ERVL-MaLR (0) 467 6 3**

**1236 0.8 0.0 0.0 gi|89161198:112230569-112236651 2952 3082 (3001) + AluYa8 SINE/Alu 168 298 (12) 4 (OCCUPIED)**

**204 6.7 0.0 0.0 gi|89161198:112230569-112236651 3628 3657 (2426) + (T)n Simple_repeat 1 30 (0) 5**

**1370 27.1 3.4 0.2 gi|89161198:112230569-112236651 5293 5677 (406) C L1ME3A LINE/L1 (158) 6015 5619 6**

**518 23.6 8.5 1.0 gi|89161198:112230569-112236651 5695 5882 (201) C L1ME3A LINE/L1 (584) 5578 5377 6**

**_______________________________________________________________________________________**

**AluYa5_5_222c**

**357 29.1 6.5 5.4 chr5 166222665 166222802 (14635064) + MIRb SINE/MIR 56 196 (72) 174**

**626 3.8 1.3 0.0 chr5 166222803 166222881 (14634985) C MADE1 DNA/Mariner (0) 80 1 175**

**357 29.1 6.5 5.4 chr5 166222882 166222927 (14634939) + MIRb SINE/MIR 197 241 (27) 174**

**256 34.4 9.7 2.7 chr5 166222933 166223117 (14634749) C MIR SINE/MIR (14) 248 51 176**

**231 8.8 0.0 0.0 chr5 166224099 166224132 (14633734) + (TG)n Simple_repeat 2 35 (0) 177**

**605 30.3 5.2 6.4 chr5 166224361 166224610 (14633256) C MIR SINE/MIR (6) 256 10 178**

**256 29.6 1.2 1.2 chr5 166225378 166225459 (14632407) + GA-rich Low_complexity 3 84 (0) 179**

**1335 9.1 0.0 0.6 chr5 166226544 166226719 (14631147) C AluSq SINE/Alu (16) 297 123 180**

**2550 5.8 0.0 0.6 chr5 166226720 166227031 (14630835) C AluYa5 SINE/Alu (0) 310 1 181 (AluYa5_5_222C)**

**618 23.5 11.3 2.5 chr5 166227457 166227669 (14630197) + MIRb SINE/MIR 30 262 (0) 182**

**494 34.3 9.9 0.5 chr5 166228092 166228302 (14629564) + MIR SINE/MIR 8 238 (24) 183**

**2423 8.8 0.0 0.0 chr5 166228436 166228741 (14629125) C AluSg SINE/Alu (0) 310 5 184**

**215 33.7 10.8 0.0 chr5 166228909 166228991 (14628875) C MIRb SINE/MIR (143) 125 34 185**

**642 8.7 6.9 0.0 chr5 166229953 166230189 (14627677) + (TA)n Simple_repeat 1 253 (0) 186**

**834 27.0 4.5 9.5 chr5 166230200 166230446 (14627420) C L1ME1 LINE/L1 (64) 6102 5849 187**

**509 1.5 0.0 0.0 chr5 166230447 166230512 (14627354) C L1PA2 LINE/L1 (0) 6155 6090 188**

**834 27.0 4.5 9.5 chr5 166230513 166230726 (14627140) C L1ME1 LINE/L1 (318) 5848 5664 187**

**436 33.3 1.4 6.2 chr5 166230900 166231107 (14626759) C MIRb SINE/MIR (58) 210 13 189**

**224 32.3 4.4 5.1 chr5 166231119 166231255 (14626611) C L2 LINE/L2 (1) 3418 3283 190**

**231 8.8 0.0 0.0 gi|89161208:162329963-162336032 373 406 (5664) + (TG)n Simple_repeat 2 35 (0) 1**

**595 30.4 5.2 6.5 gi|89161208:162329963-162336032 635 884 (5186) C MIR SINE/MIR (6) 256 10 2**

**246 29.7 0.0 3.7 gi|89161208:162329963-162336032 1652 1735 (4335) + A-rich Low_complexity 6 86 (0) 3**

**300 34.3 4.7 1.7 gi|89161208:162329963-162336032 1781 1949 (4121) + Tigger10 DNA/TcMar-Tigger 501 674 (1169) 4**

**380 31.9 0.7 0.7 gi|89161208:162329963-162336032 2089 2224 (3846) + Tigger10 DNA/TcMar-Tigger 904 1039 (804) 5**

**225 21.4 1.3 5.3 gi|89161208:162329963-162336032 2305 2383 (3687) + Tigger10 DNA/TcMar-Tigger 1169 1244 (599) 6**

**513 33.6 0.9 6.0 gi|89161208:162329963-162336032 2572 2821 (3249) + Tigger10 DNA/TcMar-Tigger 1449 1698 (145) 7**

**2261 11.1 0.0 0.3 gi|89161208:162329963-162336032 2822 3119 (2951) C AluSc8 SINE/Alu (15) 297 1 8**

**513 33.6 0.9 6.0 gi|89161208:162329963-162336032 3120 3203 (2867) + Tigger10 DNA/TcMar-Tigger 1699 1767 (76) 7**

**260 27.8 0.8 5.8 gi|89161208:162329963-162336032 3261 3386 (2684) + Tigger15a DNA/TcMar-Tigger 472 591 (124) 9**

**604 23.5 11.3 2.3 gi|89161208:162329963-162336032 3545 3757 (2313) + MIRb SINE/MIR 30 262 (0) 10**

**477 34.3 9.9 0.4 gi|89161208:162329963-162336032 4180 4390 (1680) + MIR SINE/MIR 8 238 (24) 11**

**2505 8.5 0.0 0.0 gi|89161208:162329963-162336032 4527 4831 (1239) C AluSg SINE/Alu (1) 309 5 12**

**243 12.2 2.0 0.0 gi|89161208:162329963-162336032 6007 6055 (15) C L1ME2z LINE/L1 (280) 6164 6115 13**

**__________________________________________________________________________________**

**AluYa5_6_204 OCCUPIED**

**1241 19.5 8.6 14.4 chr6 135624879 135625163 (35274829) + MLT2D LTR/ERVL 88 392 (22) 300**

**317 12.5 0.2 1.6 chr6 135625174 135625237 (35274755) + MLT2B2 LTR/ERVL 503 567 (0) 300**

**335 33.3 4.7 3.0 chr6 135625568 135625802 (35274190) C L2 LINE/L2 (0) 3419 3181 302**

**1594 21.7 7.4 0.3 chr6 135626002 135626339 (35273653) C MLT1A0 LTR/MaLR (2) 363 2 303**

**249 31.2 2.6 0.0 chr6 135626501 135626577 (35273415) + L2 LINE/L2 3299 3377 (1) 304**

**878 19.6 0.5 7.8 chr6 135627159 135627363 (35272629) + MER53 DNA 2 191 (2) 305**

**2228 1.3 0.0 0.0 chr6 135627830 135628066 (35271926) + AluYa5 SINE/Alu 1 237 (73) 306 (AluYa5_6_204)**

**180 0.0 0.0 0.0 chr6 135628072 135628091 (35271901) + (A)n Simple_repeat 1 20 (0) 307**

**222 31.5 4.1 8.2 chr6 135628653 135628700 (35271292) + L2 LINE/L2 3298 3348 (71) 308**

**2144 15.5 0.5 9.8 chr6 135628701 135629017 (35270975) C MLT1B LTR/MaLR (2) 388 101 309**

**1758 16.7 4.8 0.3 chr6 135629018 135629311 (35270681) C AluJo SINE/Alu (5) 307 1 310**

**2144 15.5 0.5 9.8 chr6 135629312 135629421 (35270571) C MLT1B LTR/MaLR (290) 100 1 309**

**222 31.5 4.1 8.2 chr6 135629422 135629469 (35270523) + L2 LINE/L2 3349 3388 (31) 308**

**Ortholog in Celera**

**1106 20.5 11.4 2.5 gi|89161209:136323514-136329746 8 291 (5942) + MLT2D LTR/ERVL 88 537 (22) 1**

**321 12.5 0.2 1.6 gi|89161209:136323514-136329746 296 359 (5874) + MLT2D LTR/ERVL 503 567 (0) 1**

**449 34.8 4.8 0.8 gi|89161209:136323514-136329746 690 918 (5315) C L2a LINE/L2 (0) 3426 3189 2**

**1581 21.7 7.4 0.3 gi|89161209:136323514-136329746 1124 1461 (4772) C MLT1A0 LTR/ERVL-MaLR (2) 363 2 3**

**287 30.3 2.9 3.2 gi|89161209:136323514-136329746 1589 1699 (4534) + L2b LINE/L2 3283 3386 (1) 4**

**853 19.7 0.5 8.4 gi|89161209:136323514-136329746 2281 2485 (3748) + MER53 DNA/hAT 2 191 (2) 5**

**2810 0.7 0.0 0.0 gi|89161209:136323514-136329746 2952 3247 (2986) + AluYa5 SINE/Alu 1 296 (14) 6 (OCCUPIED)**

**249 33.2 2.9 12.7 gi|89161209:136323514-136329746 3801 3856 (2377) + L2b LINE/L2 3246 3305 (70) 7**

**2066 15.4 0.5 14.9 gi|89161209:136323514-136329746 3857 4173 (2060) C MLT1B LTR/ERVL-MaLR (2) 388 100 8**

**1840 16.6 0.0 0.0 gi|89161209:136323514-136329746 4174 4451 (1782) C AluJr SINE/Alu (5) 307 30 9**

**2066 15.4 0.5 14.9 gi|89161209:136323514-136329746 4452 4577 (1656) C MLT1B LTR/ERVL-MaLR (291) 99 1 8**

**249 33.2 2.9 12.7 gi|89161209:136323514-136329746 4578 4658 (1575) + L2b LINE/L2 3306 3370 (5) 7**

**________________________________________________________________________________________**

**AluYa5_16_26 C_INTER_RMD**

**1982 11.0 1.0 1.4 chr16 21334260 21334555 (67492699) C AluSq SINE/Alu (18) 295 1 472**

**1959 14.7 0.0 0.0 chr16 21334559 21334870 (67492384) C AluSx SINE/Alu (0) 312 1 473**

**1212 16.9 4.1 0.9 chr16 21334871 21334972 (67492282) C MER30 DNA/MER1_type (123) 107 6 471**

**2137 11.0 0.3 0.0 chr16 21334996 21335294 (67491960) C AluSg SINE/Alu (10) 300 1 474**

**2315 9.0 0.3 0.0 chr16 21335532 21335841 (67491413) + AluY SINE/Alu 1 311 (0) 475**

**453 23.9 13.3 8.8 chr16 21336120 21336336 (67490918) + L1ME2 LINE/L1 5811 6046 (118) 476**

**2026 10.0 1.1 0.0 chr16 21336337 21336652 (67490602) + AluSp SINE/Alu 1 319 (0) 477**

**453 23.6 14.6 9.3 chr16 21336653 21336738 (67490516) + L1ME2 LINE/L1 6047 6130 (34) 476**

**2523 4.2 0.7 0.0 chr16 21336740 21337047 (67490207) + AluYa5 SINE/Alu 1 310 (0) 478 (R1)**

**1250 7.8 7.3 0.0 chr16 21337068 21337246 (67490008) + AluSg/x SINE/Alu 115 306 (6) 479 (R2)**

**1333 16.4 0.0 1.7 chr16 21337319 21337559 (67489695) C AluJb SINE/Alu (61) 251 15 480**

**333 13.3 4.0 0.0 chr16 21337809 21337883 (67489371) + G-rich Low_complexity 3 80 (0) 481**

**245 15.8 0.0 0.0 chr16 21337904 21337941 (67489313) + GA-rich Low_complexity 1 38 (0) 482**

**367 28.4 9.7 2.9 chr16 21338601 21338879 (67488375) + L1M5 LINE/L1 2667 2964 (3182) 483**

**298 28.5 0.7 2.8 chr16 21339371 21339511 (67487743) C MER113 DNA/MER1_type (93) 428 291 484**

**2156 10.0 0.3 0.6 chr16 21339514 21339824 (67487430) + AluSg SINE/Alu 1 310 (0) 485**

**2364 7.3 0.0 1.6 chr16 21339892 21340198 (67487056) + AluSg SINE/Alu 1 302 (8) 486**

**Ortholog in Celera**

**1205 16.4 4.1 0.9 gi|89161194:12205350-12211461 400 513 (5599) C MER30 DNA/hAT-Charlie (0) 230 108 1**

**2168 10.2 1.0 1.7 gi|89161194:12205350-12211461 514 808 (5304) C AluSx1 SINE/Alu (19) 293 1 2**

**2181 14.4 0.0 0.0 gi|89161194:12205350-12211461 812 1123 (4989) C AluSx1 SINE/Alu (0) 312 1 3**

**1205 16.4 4.1 0.9 gi|89161194:12205350-12211461 1124 1225 (4887) C MER30 DNA/hAT-Charlie (123) 107 6 1**

**2325 9.5 0.3 0.0 gi|89161194:12205350-12211461 1244 1538 (4574) C AluSg4 SINE/Alu (16) 296 1 4**

**2512 7.6 0.3 0.0 gi|89161194:12205350-12211461 1773 2075 (4037) + AluSc8 SINE/Alu 1 304 (8) 5**

**403 27.1 5.4 4.5 gi|89161194:12205350-12211461 2345 2561 (3551) + L1ME3A LINE/L1 5811 6045 (399) 6**

**2147 10.0 1.1 0.0 gi|89161194:12205350-12211461 2562 2864 (3248) + AluSp SINE/Alu 1 313 (0) 7**

**400 29.0 11.8 4.0 gi|89161194:12205350-12211461 2865 2950 (3162) + L1ME3A LINE/L1 6046 6136 (308) 6**

**1934 6.4 0.6 20.1 gi|89161194:12205350-12211461 2952 3308 (2804) + AluSc SINE/Alu 1 299 (10) 8 (R12)**

**1467 16.2 0.0 1.7 gi|89161194:12205350-12211461 3386 3626 (2486) C AluJb SINE/Alu (61) 251 15 9**

**279 14.5 4.3 0.0 gi|89161194:12205350-12211461 3874 3942 (2170) + G-rich Low_complexity 3 74 (0) 10**

**204 9.7 0.0 0.0 gi|89161194:12205350-12211461 3963 3993 (2119) + (GGA)n Simple_repeat 1 31 (0) 11**

**394 31.8 7.9 4.2 gi|89161194:12205350-12211461 4462 4917 (1195) + L1M5 LINE/L1 2474 2945 (3201) 12**

**302 25.8 1.4 3.6 gi|89161194:12205350-12211461 5425 5565 (547) C MER113 DNA/hAT-Charlie (93) 428 291 13**

**2260 8.8 0.3 0.0 gi|89161194:12205350-12211461 5568 5851 (261) + AluSg SINE/Alu 1 285 (25) 14**

**1376 7.4 0.0 0.0 gi|89161194:12205350-12211461 5950 6112 (0) + AluSg SINE/Alu 1 163 (147) 15**

**____________________________________________________________________________________**

**AluYa5_16_28c C_INTER_RMD**

**2498 7.3 0.0 1.6 chr16 22436354 22436660 (66390594) C AluSg SINE/Alu (8) 302 1 733**

**2275 8.4 0.3 0.0 chr16 22436751 22437034 (66390220) C AluSg SINE/Alu (25) 285 1 734**

**305 28.5 0.7 2.8 chr16 22437037 22437177 (66390077) + MER113 DNA/MER1_type 291 428 (93) 735**

**401 27.9 9.1 3.0 chr16 22437685 22437947 (66389307) C L1M5 LINE/L1 (3201) 2945 2667 736**

**245 15.8 0.0 0.0 chr16 22438607 22438644 (66388610) + CT-rich Low_complexity 1 38 (0) 737**

**333 13.3 4.0 0.0 chr16 22438665 22438739 (66388515) + C-rich Low_complexity 3 80 (0) 738**

**1419 16.4 0.0 1.7 chr16 22438989 22439229 (66388025) + AluJb SINE/Alu 15 251 (61) 739**

**1323 7.8 7.3 0.0 chr16 22439302 22439480 (66387774) C AluSg/x SINE/Alu (6) 306 115 740 (R2)**

**2657 4.2 0.7 0.0 chr16 22439502 22439809 (66387445) C AluYa5 SINE/Alu (0) 310 1 741 (R1)**

**454 23.6 14.6 9.3 chr16 22439811 22439896 (66387358) C L1ME2 LINE/L1 (34) 6130 6047 742**

**2149 10.0 1.1 0.0 chr16 22439897 22440212 (66387042) C AluSp SINE/Alu (0) 319 1 743**

**454 23.9 13.3 8.8 chr16 22440213 22440429 (66386825) C L1ME2 LINE/L1 (118) 6046 5811 742**

**2315 9.0 0.3 0.0 chr16 22440707 22441016 (66386238) C AluY SINE/Alu (0) 311 1 744**

**2137 11.0 0.3 0.0 chr16 22441255 22441553 (66385701) + AluSg SINE/Alu 1 300 (10) 745**

**1212 16.9 4.1 0.9 chr16 22441577 22441678 (66385576) + MER30 DNA/MER1_type 3 104 (126) 746**

**1959 14.7 0.0 0.0 chr16 22441679 22441990 (66385264) + AluSx SINE/Alu 1 312 (0) 747**

**1982 11.0 1.0 1.4 chr16 22441994 22442289 (66384965) + AluSq SINE/Alu 1 295 (18) 748**

**1212 16.9 4.1 0.9 chr16 22442290 22442403 (66384851) + MER30 DNA/MER1_type 105 227 (3) 746**

**Ortholog in Celera**

**1376 7.4 0.0 0.0 gi|89161194:12205350-12211461 1 163 (5949) C AluSg SINE/Alu (147) 163 1 1**

**2260 8.8 0.3 0.0 gi|89161194:12205350-12211461 262 545 (5567) C AluSg SINE/Alu (25) 285 1 2**

**302 25.8 1.4 3.6 gi|89161194:12205350-12211461 548 688 (5424) + MER113 DNA/hAT-Charlie 291 428 (93) 3**

**391 31.8 7.9 4.2 gi|89161194:12205350-12211461 1196 1651 (4461) C L1M5 LINE/L1 (3201) 2945 2474 4**

**277 30.1 0.0 0.0 gi|89161194:12205350-12211461 2113 2195 (3917) + CT-rich Low_complexity 97 179 (0) 5**

**274 4.5 4.5 0.0 gi|89161194:12205350-12211461 2196 2239 (3873) + (CCCCCT)n Simple_repeat 3 48 (0) 6**

**1467 16.2 0.0 1.7 gi|89161194:12205350-12211461 2487 2727 (3385) + AluJb SINE/Alu 15 251 (61) 7**

**1934 6.4 0.6 20.1 gi|89161194:12205350-12211461 2805 3161 (2951) C AluSc SINE/Alu (10) 299 1 8 (R12)**

**400 27.9 11.8 4.0 gi|89161194:12205350-12211461 3163 3248 (2864) C L1ME3A LINE/L1 (308) 6136 6046 9**

**2147 10.0 1.1 0.0 gi|89161194:12205350-12211461 3249 3551 (2561) C AluSp SINE/Alu (0) 313 1 10**

**403 26.7 5.4 4.5 gi|89161194:12205350-12211461 3552 3768 (2344) C L1ME3A LINE/L1 (399) 6045 5811 9**

**2512 7.6 0.3 0.0 gi|89161194:12205350-12211461 4038 4340 (1772) C AluSc8 SINE/Alu (8) 304 1 11**

**2325 9.5 0.3 0.0 gi|89161194:12205350-12211461 4575 4869 (1243) + AluSg4 SINE/Alu 1 296 (16) 12**

**1205 16.4 4.1 0.9 gi|89161194:12205350-12211461 4888 4989 (1123) + MER30 DNA/hAT-Charlie 3 104 (126) 13**

**2181 14.4 0.0 0.0 gi|89161194:12205350-12211461 4990 5301 (811) + AluSx1 SINE/Alu 1 312 (0) 14**

**2168 10.2 1.0 1.7 gi|89161194:12205350-12211461 5305 5599 (513) + AluSx1 SINE/Alu 1 293 (19) 15**

**1205 16.4 4.1 0.9 gi|89161194:12205350-12211461 5600 5713 (399) + MER30 DNA/hAT-Charlie 105 227 (3) 13**

**__________________________________________________________________________________**

**AluYa5_16_37 C_INTER_RMD**

**2016 10.3 1.0 1.4 chr16 29414782 29415077 (59412177) C AluSq SINE/Alu (18) 295 1 516**

**1936 15.1 0.0 0.0 chr16 29415081 29415392 (59411862) C AluSx SINE/Alu (0) 312 1 517**

**1212 16.9 4.1 0.9 chr16 29415393 29415494 (59411760) C MER30 DNA/MER1_type (123) 107 6 515**

**2137 11.0 0.3 0.0 chr16 29415518 29415816 (59411438) C AluSg SINE/Alu (10) 300 1 518**

**2228 9.4 0.3 0.0 chr16 29416056 29416354 (59410900) + AluY SINE/Alu 1 300 (11) 519**

**453 23.9 13.3 8.8 chr16 29416624 29416840 (59410414) + L1ME2 LINE/L1 5811 6046 (118) 520**

**2039 9.6 1.1 0.0 chr16 29416841 29417160 (59410094) + AluSp SINE/Alu 1 323 (0) 521**

**453 23.6 14.6 9.3 chr16 29417161 29417246 (59410008) + L1ME2 LINE/L1 6047 6130 (34) 520**

**2539 3.9 0.7 0.0 chr16 29417248 29417555 (59409699) + AluYa5 SINE/Alu 1 310 (0) 522 (R1)**

**1250 7.8 7.3 0.0 chr16 29417583 29417761 (59409493) + AluSg/x SINE/Alu 115 306 (6) 523 (R2)**

**1320 16.9 0.0 1.7 chr16 29417834 29418074 (59409180) C AluJb SINE/Alu (61) 251 15 524**

**279 14.5 4.3 0.0 chr16 29418323 29418391 (59408863) + G-rich Low_complexity 3 74 (0) 525**

**245 15.8 0.0 0.0 chr16 29418412 29418449 (59408805) + GA-rich Low_complexity 1 38 (0) 526**

**381 27.9 9.1 3.0 chr16 29419109 29419371 (59407883) + L1M5 LINE/L1 2667 2945 (3201) 527**

**293 28.7 0.7 2.9 chr16 29419879 29420018 (59407236) C MER113 DNA/MER1_type (93) 428 292 528**

**2156 10.0 0.3 0.6 chr16 29420020 29420330 (59406924) + AluSg SINE/Alu 1 310 (0) 529**

**2297 8.3 0.0 1.6 chr16 29420398 29420703 (59406551) + AluSg SINE/Alu 1 301 (9) 530**

**Ortholog in Celera**

**2213 14.0 0.0 0.0 gi|89161194:54535534-54541595 831 1138 (4924) C AluSx1 SINE/Alu (4) 308 1 3**

**1236 16.0 4.1 0.0 gi|89161194:54535534-54541595 1139 1240 (4822) C MER30 DNA/hAT-Charlie (123) 107 6 1**

**2357 10.2 0.3 0.0 gi|89161194:54535534-54541595 1254 1557 (4505) C AluSg4 SINE/Alu (7) 305 1 4**

**2345 9.6 0.3 0.0 gi|89161194:54535534-54541595 1791 2083 (3979) + AluSc8 SINE/Alu 1 294 (18) 5**

**379 29.3 4.2 3.4 gi|89161194:54535534-54541595 2345 2561 (3501) + L1ME3A LINE/L1 5811 6045 (399) 6**

**2157 10.0 1.1 0.0 gi|89161194:54535534-54541595 2562 2864 (3198) + AluSp SINE/Alu 1 313 (0) 7**

**331 31.0 11.1 3.3 gi|89161194:54535534-54541595 2865 2950 (3112) + L1ME3A LINE/L1 6046 6136 (308) 6**

**2154 7.4 5.3 0.3 gi|89161194:54535534-54541595 2952 3235 (2827) + AluSc SINE/Alu 1 298 (11) 8 (R12)**

**1477 16.2 0.0 1.7 gi|89161194:54535534-54541595 3308 3548 (2514) C AluJb SINE/Alu (61) 251 15 9**

**227 17.9 3.6 0.0 gi|89161194:54535534-54541595 3804 3859 (2203) + G-rich Low_complexity 1 58 (0) 10**

**231 3.6 0.0 0.0 gi|89161194:54535534-54541595 3875 3902 (2160) + (GGA)n Simple_repeat 1 28 (0) 11**

**341 24.9 10.3 3.9 gi|89161194:54535534-54541595 4550 4812 (1250) + L1M5 LINE/L1 2667 2945 (3201) 12**

**282 26.7 1.4 2.9 gi|89161194:54535534-54541595 5320 5458 (604) C MER113 DNA/hAT-Charlie (93) 428 292 13**

**2205 11.8 0.3 1.3 gi|89161194:54535534-54541595 5460 5774 (288) + AluSg4 SINE/Alu 1 312 (0) 14**

**1890 6.8 0.0 0.0 gi|89161194:54535534-54541595 5842 6062 (0) + AluSg SINE/Alu 1 221 (89) 15**

**____________________________________________________________________________________**

**AlluYa5_16_38 C_INTER_RMD**

**2157 10.3 1.0 1.4 chr16 30154754 30155049 (58672205) C AluSq SINE/Alu (18) 295 1 120**

**2116 14.7 0.0 0.0 chr16 30155053 30155364 (58671890) C AluSx SINE/Alu (0) 312 1 121**

**1226 16.9 4.1 0.9 chr16 30155365 30155466 (58671788) C MER30 DNA/MER1_type (123) 107 6 119**

**2285 12.0 0.3 0.0 chr16 30155480 30155788 (58671466) C AluSg SINE/Alu (0) 310 1 122**

**2384 9.3 0.3 0.0 chr16 30156027 30156326 (58670928) + AluY SINE/Alu 1 301 (10) 123**

**401 24.2 13.1 8.6 chr16 30156596 30156812 (58670442) + L1ME2 LINE/L1 5811 6046 (118) 124**

**2150 10.0 1.1 0.0 chr16 30156813 30157128 (58670126) + AluSp SINE/Alu 1 319 (0) 125**

**401 23.6 14.6 9.3 chr16 30157129 30157214 (58670040) + L1ME2 LINE/L1 6047 6130 (34) 124**

**2684 3.9 0.7 0.0 chr16 30157216 30157523 (58669731) + AluYa5 SINE/Alu 1 310 (0) 126 (AluYa5_16_38) (R1)**

**1334 7.9 7.3 0.0 chr16 30157552 30157729 (58669525) + AluSg/x SINE/Alu 115 305 (7) 127 (R2)**

**1448 16.4 0.0 1.7 chr16 30157802 30158042 (58669212) C AluJb SINE/Alu (61) 251 15 128**

**279 14.5 4.3 0.0 chr16 30158291 30158359 (58668895) + G-rich Low_complexity 3 74 (0) 129**

**245 15.8 0.0 0.0 chr16 30158380 30158417 (58668837) + GA-rich Low_complexity 1 38 (0) 130**

**343 27.9 9.1 3.0 chr16 30159077 30159339 (58667915) + L1M5 LINE/L1 2667 2945 (3201) 131**

**289 28.5 0.7 2.8 chr16 30159847 30159987 (58667267) C MER113 DNA/MER1_type (93) 428 291 132**

**2305 10.0 0.3 0.6 chr16 30159990 30160300 (58666954) + AluSg SINE/Alu 1 310 (0) 133**

**2439 8.3 0.0 1.6 chr16 30160368 30160672 (58666582) + AluSg SINE/Alu 1 300 (10) 134**

**196 25.9 3.3 4.9 chr16 30160947 30161007 (58666247) + MIRb SINE/MIR 44 103 (165) 135**

**Ortholog in Celera**

**2212 10.9 1.0 1.7 gi|89161194:54535534-54541595 526 829 (5233) C AluSx1 SINE/Alu (10) 302 1 2**

**2213 14.0 0.0 0.0 gi|89161194:54535534-54541595 831 1138 (4924) C AluSx1 SINE/Alu (4) 308 1 3**

**1236 16.0 4.1 0.0 gi|89161194:54535534-54541595 1139 1240 (4822) C MER30 DNA/hAT-Charlie (123) 107 6 1**

**2357 10.2 0.3 0.0 gi|89161194:54535534-54541595 1254 1557 (4505) C AluSg4 SINE/Alu (7) 305 1 4**

**2345 9.6 0.3 0.0 gi|89161194:54535534-54541595 1791 2083 (3979) + AluSc8 SINE/Alu 1 294 (18) 5**

**379 29.3 4.2 3.4 gi|89161194:54535534-54541595 2345 2561 (3501) + L1ME3A LINE/L1 5811 6045 (399) 6**

**2157 10.0 1.1 0.0 gi|89161194:54535534-54541595 2562 2864 (3198) + AluSp SINE/Alu 1 313 (0) 7**

**331 31.0 11.1 3.3 gi|89161194:54535534-54541595 2865 2950 (3112) + L1ME3A LINE/L1 6046 6136 (308) 6**

**2154 7.4 5.3 0.3 gi|89161194:54535534-54541595 2952 3235 (2827) + AluSc SINE/Alu 1 298 (11) 8 (R12)**

**1477 16.2 0.0 1.7 gi|89161194:54535534-54541595 3308 3548 (2514) C AluJb SINE/Alu (61) 251 15 9**

**227 17.9 3.6 0.0 gi|89161194:54535534-54541595 3804 3859 (2203) + G-rich Low_complexity 1 58 (0) 10**

**231 3.6 0.0 0.0 gi|89161194:54535534-54541595 3875 3902 (2160) + (GGA)n Simple_repeat 1 28 (0) 11**

**341 24.9 10.3 3.9 gi|89161194:54535534-54541595 4550 4812 (1250) + L1M5 LINE/L1 2667 2945 (3201) 12**

**282 26.7 1.4 2.9 gi|89161194:54535534-54541595 5320 5458 (604) C MER113 DNA/hAT-Charlie (93) 428 292 13**

**2205 11.8 0.3 1.3 gi|89161194:54535534-54541595 5460 5774 (288) + AluSg4 SINE/Alu 1 312 (0) 14**

**1890 6.8 0.0 0.0 gi|89161194:54535534-54541595 5842 6062 (0) + AluSg SINE/Alu 1 221 (89) 15**

**__________________________________________________________________________________**

**AluYa5_16_67 C_INTER_RMD**

**260 24.0 29.0 0.0 chr16 55129580 55129679 (33697575) C L1MC4a LINE/L1 (1885) 5923 5795 283**

**1878 15.8 0.6 1.3 chr16 55129798 55130111 (33697143) + AluJo SINE/Alu 1 312 (0) 284**

**828 25.8 0.5 1.8 chr16 55130323 55130539 (33696715) C MIR SINE/MIR (37) 225 12 285**

**189 32.6 10.9 3.3 chr16 55130561 55130652 (33696602) C L2 LINE/L2 (13) 3365 3267 286**

**190 27.4 18.2 3.3 chr16 55130841 55130875 (33696379) C MIRb SINE/MIR (108) 160 107 287**

**1710 16.2 0.4 0.4 chr16 55130876 55131147 (33696107) + AluJo SINE/Alu 23 294 (18) 288**

**190 27.4 18.2 3.3 chr16 55131148 55131232 (33696022) C MIRb SINE/MIR (162) 106 23 287**

**2561 13.9 3.5 4.6 chr16 55131515 55131967 (33695287) + LTR26 LTR/ERV1 1 448 (155) 289**

**2540 5.5 0.0 1.0 chr16 55131968 55132277 (33694977) + AluYa5 SINE/Alu 1 307 (3) 290 (AluYa5_16_67) (R1)**

**1184 13.9 0.0 0.0 chr16 55132279 55132451 (33694803) + AluSg/x SINE/Alu 134 306 (6) 291 (R2)**

**974 13.5 6.4 0.0 chr16 55132453 55132608 (33694646) + LTR26 LTR/ERV1 438 603 (0) 289**

**303 22.0 3.9 2.9 chr16 55132625 55132727 (33694527) C MER113 DNA/MER1_type (7) 514 411 292**

**2071 12.1 0.3 2.0 chr16 55132744 55133039 (33694215) + AluSq SINE/Alu 1 291 (22) 293**

**448 22.7 9.8 0.0 chr16 55133348 55133479 (33693775) + MIR SINE/MIR 33 177 (85) 294**

**399 27.6 6.2 14.1 chr16 55133490 55133793 (33693461) + L2 LINE/L2 3135 3414 (5) 295**

**395 25.6 7.7 11.4 chr16 55134160 55134247 (33693007) C MIRb SINE/MIR (8) 260 167 296**

**1778 16.6 1.0 0.3 chr16 55134248 55134549 (33692705) + AluJo SINE/Alu 4 307 (5) 297**

**395 25.7 7.2 10.3 chr16 55134550 55134700 (33692554) C MIRb SINE/MIR (102) 166 28 296**

**Ortholog in Celera**

**243 28.6 4.3 0.0 gi|89161194:41069529-41075600 565 634 (5438) C L1MC4a LINE/L1 (1959) 5923 5851 3**

**1957 15.3 0.6 1.3 gi|89161194:41069529-41075600 783 1095 (4977) + AluJr SINE/Alu 1 311 (1) 4**

**924 30.0 0.4 1.6 gi|89161194:41069529-41075600 1270 1523 (4549) C MIR SINE/MIR (0) 262 12 5**

**220 27.5 13.7 6.2 gi|89161194:41069529-41075600 1545 1728 (4344) C L2b LINE/L2 (13) 3374 3182 6**

**222 26.4 18.2 2.9 gi|89161194:41069529-41075600 1825 1859 (4213) C MIRb SINE/MIR (108) 160 112 7**

**1710 16.2 0.4 0.4 gi|89161194:41069529-41075600 1860 2131 (3941) + AluJo SINE/Alu 23 294 (18) 8**

**222 26.4 18.2 2.9 gi|89161194:41069529-41075600 2132 2216 (3856) C MIRb SINE/MIR (157) 111 23 7**

**3242 15.3 4.3 5.6 gi|89161194:41069529-41075600 2499 2951 (3121) + LTR26 LTR/ERV1 1 448 (155) 9**

**2269 10.9 0.0 2.3 gi|89161194:41069529-41075600 2952 3263 (2809) + AluSx SINE/Alu 1 305 (7) 10 (R12)**

**3242 15.3 4.3 5.6 gi|89161194:41069529-41075600 3264 3420 (2652) + LTR26 LTR/ERV1 449 603 (0) 9**

**416 21.7 9.9 4.9 gi|89161194:41069529-41075600 3437 3555 (2517) C MER113A DNA/hAT-Charlie (7) 307 183 11**

**2250 11.9 0.0 0.3 gi|89161194:41069529-41075600 3556 3858 (2214) + AluSq2 SINE/Alu 1 302 (10) 12**

**416 21.7 9.9 4.9 gi|89161194:41069529-41075600 3859 3990 (2082) C MER113A DNA/hAT-Charlie (132) 182 44 11**

**190 20.8 1.8 3.7 gi|89161194:41069529-41075600 4064 4118 (1954) + MIR3 SINE/MIR 81 134 (74) 13**

**506 24.0 11.0 0.0 gi|89161194:41069529-41075600 4151 4304 (1768) + MIR SINE/MIR 15 185 (77) 14**

**440 33.1 4.3 10.4 gi|89161194:41069529-41075600 4307 4610 (1462) + L2a LINE/L2 3135 3421 (5) 15**

**470 26.6 2.4 13.0 gi|89161194:41069529-41075600 4985 5064 (1008) C MIRb SINE/MIR (28) 240 171 16**

**1838 16.6 1.0 0.3 gi|89161194:41069529-41075600 5065 5366 (706) + AluJo SINE/Alu 4 307 (5) 17**

**470 26.6 2.4 13.0 gi|89161194:41069529-41075600 5367 5497 (575) C MIRb SINE/MIR (98) 170 49 16**

**__________________________________________________________________________________**

**AluYa5_19_28 C_INTER_RMD**

**26 5.0 0.0 0.0 chr19 21301580 21301619 (42510032) + AT_rich Low_complexity 1 40 (0) 301**

**2579 6.4 0.0 0.6 chr19 21301890 21302203 (42509448) + AluSp SINE/Alu 1 312 (1) 302**

**314 18.8 1.4 2.8 chr19 21303202 21303272 (42508379) + MER93a LTR/ERV1 1 70 (332) 303**

**2446 6.1 0.0 0.7 chr19 21304333 21304627 (42507024) C AluSp SINE/Alu (20) 293 1 304**

**2132 11.2 0.7 1.0 chr19 21304743 21305048 (42506603) + AluSq SINE/Alu 5 309 (4) 305**

**293 9.1 2.3 0.0 chr19 21305051 21305094 (42506557) C MER33 DNA/MER1_type (18) 306 262 306**

**1204 12.6 0.0 0.0 chr19 21305098 21305272 (42506379) C AluSg/x SINE/Alu (7) 305 131 307**

**744 27.4 7.6 0.4 chr19 21305309 21305546 (42506105) C MER33 DNA/MER1_type (69) 255 1 306**

**2892 0.7 0.0 0.0 chr19 21305680 21305989 (42505662) + AluYa5 SINE/Alu 1 310 (0) 308 (R1) (AluYa5_19_28)**

**1310 8.0 4.0 0.0 chr19 21305993 21306168 (42505483) + AluSg/x SINE/Alu 129 311 (1) 309 (R2)**

**1336 28.4 9.9 1.4 chr19 21306628 21307124 (42504527) C MLT1H LTR/MaLR (0) 549 11 310**

**299 26.9 15.1 0.0 chr19 21307170 21307262 (42504389) C MIR SINE/MIR (145) 117 11 311**

**1525 21.1 0.0 2.7 chr19 21307411 21307707 (42503944) + AluJb SINE/Alu 10 298 (14) 312**

**1747 17.4 0.3 0.3 chr19 21307791 21308089 (42503562) + AluSp SINE/Alu 1 299 (14) 313**

**Ortholog in Celera**

**875 10.6 0.8 0.0 gi|89161197:21345803-21351883 419 541 (5540) + MER57E3 LTR/ERV1 1 124 (363) 1**

**1333 15.7 0.0 0.9 gi|89161197:21345803-21351883 549 773 (5308) + MER57E3 LTR/ERV1 265 487 (0) 1**

**2454 6.1 0.0 0.7 gi|89161197:21345803-21351883 1608 1902 (4179) C AluSp SINE/Alu (20) 293 1 2**

**2164 10.8 0.7 1.0 gi|89161197:21345803-21351883 2018 2323 (3758) + AluSq SINE/Alu 5 309 (4) 3**

**289 9.1 2.3 0.0 gi|89161197:21345803-21351883 2326 2369 (3712) C MER33 DNA/hAT-Charlie (18) 306 262 4**

**1226 12.6 0.0 0.0 gi|89161197:21345803-21351883 2373 2547 (3534) C AluSx1 SINE/Alu (7) 305 131 5**

**715 27.4 7.6 0.4 gi|89161197:21345803-21351883 2584 2821 (3260) C MER33 DNA/hAT-Charlie (69) 255 1 4**

**2427 6.7 2.3 0.0 gi|89161197:21345803-21351883 2955 3254 (2827) + AluSg SINE/Alu 1 307 (3) 6 (R12)**

**1112 30.5 4.3 0.9 gi|89161197:21345803-21351883 3714 4210 (1871) C MLT1H LTR/ERVL-MaLR (0) 549 11 7**

**267 25.6 6.3 1.2 gi|89161197:21345803-21351883 4262 4340 (1741) C MIRb SINE/MIR (156) 112 30 8**

**1586 20.5 0.0 2.8 gi|89161197:21345803-21351883 4497 4793 (1288) + AluJb SINE/Alu 10 298 (14) 9**

**1802 17.4 0.3 0.3 gi|89161197:21345803-21351883 4877 5174 (907) + AluSp SINE/Alu 1 298 (15) 10**

**2234 11.9 0.3 0.3 gi|89161197:21345803-21351883 5223 5525 (556) + AluSq2 SINE/Alu 1 303 (9) 11**

**1672 23.0 1.2 2.0 gi|89161197:21345803-21351883 5576 5979 (102) C MER57B2 LTR/ERV1 (2) 401 1 12**

**437 19.6 0.0 4.1 gi|89161197:21345803-21351883 5980 6080 (1) C MER57-int LTR/ERV1 (0) 7537 7441 12**

**___________________________________________________________________________________**

**AluYa5_22_19 OCCUPIED**

**956 11.8 0.0 4.2 chr22 34225569 34225710 (15465722) + AluSq/x SINE/Alu 1 136 (176) 633**

**250 21.8 0.0 1.8 chr22 34225717 34225772 (15465660) + MIR SINE/MIR 151 205 (57) 634**

**211 23.6 3.6 0.0 chr22 34225808 34225862 (15465570) C MIR3 SINE/MIR (5) 203 147 635**

**3731 15.2 1.9 1.8 chr22 34226023 34226752 (15464680) + L1MA5 LINE/L1 4126 4856 (1287) 636**

**2102 14.7 0.0 0.0 chr22 34226864 34227175 (15464257) C AluSx SINE/Alu (0) 312 1 637**

**2485 9.3 0.0 0.0 chr22 34227356 34227657 (15463775) + AluSg SINE/Alu 6 307 (3) 638**

**24 0.0 0.0 0.0 chr22 34227731 34227754 (15463678) + AT_rich Low_complexity 1 24 (0) 639**

**1370 5.6 0.0 0.0 chr22 34228318 34228478 (15462954) + AluYa5 SINE/Alu 150 310 (0) 640 (AluYa5_22_19)**

**504 0.0 0.0 0.0 chr22 34228480 34228535 (15462897) + (TA)n Simple_repeat 2 57 (0) 641**

**2256 14.6 4.1 1.0 chr22 34229661 34230076 (15461356) + L1MA5 LINE/L1 4842 5270 (873) 636**

**520 12.9 3.5 0.0 chr22 34230078 34230162 (15461270) + AluJ/FLAM SINE/Alu 1 88 (224) 642**

**5511 15.3 3.4 1.6 chr22 34230163 34231178 (15460254) + L1MA5 LINE/L1 5265 6298 (2) 636**

**764 22.0 1.3 0.0 chr22 34231501 34231650 (15459782) C L1ME1 LINE/L1 (5) 6161 6010 643**

**1635 18.2 7.5 8.3 chr22 34231912 34232354 (15459078) C L1ME1 LINE/L1 (155) 6011 5573 643**

**2212 13.4 0.0 0.0 chr22 34233001 34233306 (15458126) C AluSx SINE/Alu (6) 306 1 644**

**Ortholog in Celera**

**1574 8.2 1.0 0.0 gi|89161202:19698730-19704833 1 194 (5910) + AluSx3 SINE/Alu 105 300 (12) 1**

**972 10.1 0.0 4.5 gi|89161202:19698730-19704833 203 341 (5763) + AluSz SINE/Alu 1 133 (179) 2**

**256 22.2 0.5 1.6 gi|89161202:19698730-19704833 342 406 (5698) + MIR SINE/MIR 150 205 (57) 3**

**210 29.7 3.0 4.5 gi|89161202:19698730-19704833 447 513 (5591) C L3b LINE/CR1 (6) 4502 4437 4**

**3664 15.2 1.9 1.8 gi|89161202:19698730-19704833 657 1386 (4718) + L1MA5 LINE/L1 4126 4856 (1287) 5**

**2178 14.4 0.0 0.0 gi|89161202:19698730-19704833 1498 1809 (4295) C AluSx1 SINE/Alu (0) 312 1 6**

**2471 9.3 0.0 0.0 gi|89161202:19698730-19704833 1990 2291 (3813) + AluSg SINE/Alu 6 307 (3) 7**

**24 41.7 0.0 0.0 gi|89161202:19698730-19704833 2366 2389 (3715) + AT_rich Low_complexity 1 24 (0) 8**

**1333 5.9 0.0 0.0 gi|89161202:19698730-19704833 2952 3104 (3000) + AluYa5 SINE/Alu 150 302 (8) 9 (OCCUPIED)**

**23 56.5 0.0 0.0 gi|89161202:19698730-19704833 3105 3127 (2977) + AT_rich Low_complexity 1 23 (0) 10**

**2196 14.6 4.1 0.9 gi|89161202:19698730-19704833 4249 4664 (1440) + L1MA5 LINE/L1 4842 5270 (873) 5**

**630 11.9 3.6 0.0 gi|89161202:19698730-19704833 4667 4750 (1354) + AluJr SINE/Alu 2 88 (224) 11**

**3253 18.5 4.0 1.3 gi|89161202:19698730-19704833 4751 5766 (338) + L1MA5 LINE/L1 5262 6298 (2) 5**

**Supplementary file 14 - Recombination mediated deletions – AluYa5 elements, Human Vs HuRef comparison**

Repeat masker annotation of the locus in the main genome and its flanks is listed first, followed by the Repeat masker annotation of the identified ortholog in the comparative genome and its flanks.

R1,R2 – PUTATIVE REGIONS OF HOMOLOGY WHICH RECOMBINE TO GIVE R12 (M_INTER_RMD or C_INTER_RMD).

In case of disruptions, R1 and R2 represent disrupted halves of the parent repeat, R represents undisrupted repeat and D represents the disruptive sequence. (M_DISRUPTED OR C_DISRUPTED).

In case of intra element recombination (M_INTRA_RMD or C_INTRA_RMD), R12 represent the 2 resulting copies arising out of intra element recombination and R represents the intact repeat.

AluYa5_2_67c C_INTER_RMD

2398 7.9 0.0 0.0 chr2 43508318 43508620 (199442529) + AluY SINE/Alu 1 303 (8) 785

209 12.7 6.7 1.7 chr2 43508621 43508655 (199442494) + (TA)n Simple_repeat 2 37 (0) 786

2098 11.5 1.4 0.3 chr2 43508969 43509257 (199441892) + AluSx SINE/Alu 1 292 (20) 787

2264 10.9 0.3 0.0 chr2 43509751 43510044 (199441105) C AluSx SINE/Alu (12) 300 6 788

981 16.6 1.2 0.0 chr2 43510530 43510698 (199440451) + AluJo SINE/Alu 1 171 (141) 789

1104 19.3 0.5 2.3 chr2 43511335 43511551 (199439598) C MER20 DNA/MER1_type (6) 213 1 790

790 21.8 4.9 0.4 chr2 43511880 43512105 (199439044) C L1ME2 LINE/L1 (21) 6143 5908 791

1468 7.1 0.0 0.0 chr2 43512106 43512289 (199438860) C AluY SINE/Alu (9) 302 119 792 (R2)

1684 2.6 0.0 0.0 chr2 43512297 43512488 (199438661) C AluYa5 SINE/Alu (0) 310 119 793

2862 0.7 0.0 0.0 chr2 43512489 43512798 (199438351) C AluYa5 SINE/Alu (0) 310 1 794 (AluYa5_2_67c) (R1)

1097 7.5 0.0 0.0 chr2 43512799 43512931 (199438218) C AluY SINE/Alu (177) 134 2 793

1710 20.9 6.9 2.7 chr2 43512937 43513823 (199437326) C L1ME2 LINE/L1 (262) 5902 4930 791

2203 11.8 0.7 0.3 chr2 43513892 43514197 (199436952) + AluSx SINE/Alu 2 308 (4) 795

687 24.7 8.4 8.4 chr2 43514199 43514329 (199436820) + L1M5 LINE/L1 4508 4627 (1519) 796

2211 12.0 0.0 0.3 chr2 43514330 43514631 (199436518) C AluSx SINE/Alu (11) 301 1 797

687 24.7 8.4 8.4 chr2 43514632 43514870 (199436279) + L1M5 LINE/L1 4628 4878 (1268) 796

Ortholog in HuRef

2390 10.9 0.3 0.0 gi|157724517:43391678-43397747 464 756 (5314) C AluSx SINE/Alu (13) 299 6 1

1102 16.6 1.2 0.0 gi|157724517:43391678-43397747 1242 1410 (4660) + AluJo SINE/Alu 1 171 (141) 2

1187 18.4 0.5 2.4 gi|157724517:43391678-43397747 2047 2263 (3807) C MER20 DNA/hAT-Charlie (6) 213 1 3

855 20.9 7.5 0.4 gi|157724517:43391678-43397747 2592 2817 (3253) C L1ME2z LINE/L1 (295) 6149 5908 4

2847 3.6 0.0 0.0 gi|157724517:43391678-43397747 2818 3119 (2951) C AluYa5 SINE/Alu (8) 302 1 5 (R12)

1173 6.8 0.0 0.0 gi|157724517:43391678-43397747 3120 3252 (2818) C AluYk11 SINE/Alu (178) 134 2 6

1770 21.0 12.8 2.9 gi|157724517:43391678-43397747 3274 4144 (1926) C L1ME2z LINE/L1 (558) 5886 4930 4

2378 11.8 0.7 0.3 gi|157724517:43391678-43397747 4213 4518 (1552) + AluSx SINE/Alu 2 308 (4) 7

818 24.7 8.4 8.4 gi|157724517:43391678-43397747 4520 4650 (1420) + L1M5 LINE/L1 4508 4627 (1519) 8

2373 11.9 0.0 0.3 gi|157724517:43391678-43397747 4651 4952 (1118) C AluSx SINE/Alu (11) 301 1 9

818 24.7 8.4 8.4 gi|157724517:43391678-43397747 4953 5191 (879) + L1M5 LINE/L1 4628 4878 (1268) 8

4899 13.8 2.4 5.4 gi|157724517:43391678-43397747 5265 6070 (0) + LTR1 LTR/ERV1 1 783 (0) 10

____________________________________________________________________________________

Alu_3_139c OCCUPIED

2406 7.0 4.5 2.1 chr3 117307328 117307708 (82194119) C HERVH LTR/ERV1 (0) 7713 7324 387

1261 13.9 1.8 1.4 chr3 117307709 117307927 (82193900) C HERVH LTR/ERV1 (1894) 5819 5600 387

3629 13.9 7.3 0.3 chr3 117307929 117308547 (82193280) C HERVH LTR/ERV1 (2564) 5149 4488 387

3149 10.8 0.8 0.8 chr3 117308550 117309051 (82192776) C HERVH LTR/ERV1 (3720) 3993 3492 387

743 16.9 2.0 0.0 chr3 117309042 117309189 (82192638) C HERVH LTR/ERV1 (4423) 3290 3140 387

16672 9.2 1.2 2.9 chr3 117309184 117312335 (82189492) C HERVH LTR/ERV1 (4726) 2987 1 387

2563 8.6 12.6 2.8 chr3 117312336 117312765 (82189062) C LTR7Y LTR/ERV1 (0) 472 1 387

189 0.0 0.0 0.0 chr3 117314938 117314958 (82186869) + (TA)n Simple_repeat 2 22 (0) 388

2757 1.6 0.0 0.0 chr3 117314959 117315261 (82186566) C AluYa5 SINE/Alu (7) 303 1 389 (Alu_3_139c)

218 30.2 0.0 0.0 chr3 117315294 117315356 (82186471) + (TTA)n Simple_repeat 2 64 (0) 390

498 21.1 0.9 1.9 chr3 117315543 117315648 (82186179) + MER92C LTR/ERV1 1 105 (449) 391

229 27.4 0.0 0.0 chr3 117315771 117315832 (82185995) + MER92C LTR/ERV1 493 554 (0) 391

650 27.9 6.2 1.8 chr3 117316777 117317002 (82184825) C MIR SINE/MIR (8) 254 19 392

1189 27.9 7.4 4.1 chr3 117317016 117317948 (82183879) C HAL1 LINE/L1 (695) 1812 813 393

1965 10.7 0.0 7.3 chr3 117317952 117318253 (82183574) + AluSc SINE/Alu 1 280 (29) 394

267 3.1 0.0 0.0 chr3 117318254 117318285 (82183542) + (TAA)n Simple_repeat 2 33 (0) 395

1336 22.1 10.2 0.6 chr3 117318306 117318647 (82183180) + MLT1H1 LTR/MaLR 181 555 (0) 396

438 22.4 2.8 1.8 chr3 117318731 117318839 (82182988) C HAL1 LINE/L1 (1695) 812 703 393

2606 18.4 3.2 1.1 chr3 117318871 117319437 (82182390) C L1MC3 LINE/L1 (1) 7784 7206 397

1218 28.3 10.2 8.0 chr3 117319493 117320157 (82181670) C HAL1 LINE/L1 (1816) 691 12 393

Ortholog in HuRef

3061 6.6 0.0 0.0 gi|157731950:113204942-113211201 1 394 (5866) C HERVH-int LTR/ERV1 (7319) 394 1 1

2502 8.3 12.6 2.5 gi|157731950:113204942-113211201 395 824 (5436) C LTR7Y LTR/ERV1 (0) 472 1 1

2904 1.9 0.0 0.0 gi|157731950:113204942-113211201 3001 3309 (2951) C AluYa5 SINE/Alu (1) 309 1 2 (OCCUPIED)

218 30.2 0.0 0.0 gi|157731950:113204942-113211201 3342 3404 (2856) + (TTA)n Simple_repeat 2 64 (0) 3

534 21.3 1.0 2.1 gi|157731950:113204942-113211201 3591 3715 (2545) + MER92C LTR/ERV1 1 123 (513) 4

244 27.4 0.0 0.0 gi|157731950:113204942-113211201 3819 3880 (2380) + MER92C LTR/ERV1 493 554 (0) 4

641 27.9 6.2 1.7 gi|157731950:113204942-113211201 4824 5049 (1211) C MIR SINE/MIR (8) 254 19 5

1102 29.6 10.2 2.9 gi|157731950:113204942-113211201 5063 5995 (265) C HAL1 LINE/L1 (695) 1812 813 6

1816 9.2 0.0 8.7 gi|157731950:113204942-113211201 5999 6260 (0) + AluSc8 SINE/Alu 1 241 (71) 7

_____________________________________________________________________________________

AluYa5_3_205c OCCUPIED

1409 30.7 18.0 4.0 chr3 158503182 158503341 (40998486) + L2 LINE/L2 2814 2991 (428) 27

263 29.6 2.3 0.0 chr3 158503344 158503431 (40998396) + Charlie7 DNA/MER1_type 12 101 (2511) 29

412 16.2 9.5 6.3 chr3 158503422 158503579 (40998248) + Charlie7 DNA/MER1_type 2427 2589 (23) 29

202 23.9 0.0 0.0 chr3 158503616 158503661 (40998166) + T-rich Low_complexity 1 46 (0) 30

193 20.0 0.0 0.0 chr3 158503706 158503740 (40998087) + L2 LINE/L2 3132 3166 (253) 31

238 23.8 25.8 1.6 chr3 158503795 158503922 (40997905) + L2 LINE/L2 3261 3419 (0) 31

26 0.0 0.0 0.0 chr3 158504497 158504522 (40997305) + AT_rich Low_complexity 1 26 (0) 32

234 21.4 15.3 1.2 chr3 158505241 158505325 (40996502) C L1ME4a LINE/L1 (52) 6069 5973 33

416 7.7 0.0 1.5 chr3 158505520 158505585 (40996242) + (TCTA)n Simple_repeat 2 66 (0) 34

2841 0.0 0.0 0.3 chr3 158505586 158505892 (40995935) C AluYa5 SINE/Alu (4) 306 1 35

835 26.5 13.4 2.1 chr3 158505947 158506327 (40995500) C L1ME4a LINE/L1 (318) 5803 5380 33

703 30.2 5.0 7.0 chr3 158506702 158507143 (40994684) C L1ME3B LINE/L1 (157) 6083 5651 36

1822 12.8 11.8 0.2 chr3 158507246 158507559 (40994268) C L1PB3 LINE/L1 (31) 6123 5777 37

316 26.3 3.0 0.0 chr3 158507710 158507808 (40994019) C L1ME3B LINE/L1 (662) 5578 5477 36

828 32.0 9.7 5.9 chr3 158507805 158508851 (40992976) C L1ME3B LINE/L1 (1061) 5085 3999 36

488 36.6 7.1 0.9 chr3 158508926 158509275 (40992552) C L1ME4a LINE/L1 (1302) 4844 4473 33

597 29.8 2.8 0.0 chr3 158509277 158509454 (40992373) + MIR SINE/MIR 80 262 (0) 38

382 26.7 2.9 1.9 chr3 158509588 158509690 (40992137) C L2 LINE/L2 (21) 3398 3295 39

312 31.1 13.1 0.0 chr3 158509880 158510001 (40991826) C L2 LINE/L2 (1) 3377 3240 40

33 2.5 0.0 0.0 chr3 158510123 158510162 (40991665) + AT_rich Low_complexity 1 40 (0) 41

Ortholog in HuRef

962 31.1 6.2 2.5 gi|157731950:154416095-154422431 24 294 (6043) + L2a LINE/L2 2544 2813 (606) 1

1692 19.0 3.0 2.5 gi|157731950:154416095-154422431 295 655 (5682) + MLT1A0 LTR/ERVL-MaLR 1 363 (2) 2

962 31.1 6.2 2.5 gi|157731950:154416095-154422431 656 815 (5522) + L2a LINE/L2 2814 2991 (428) 1

727 21.4 4.4 5.3 gi|157731950:154416095-154422431 818 1044 (5293) + Charlie7a DNA/hAT-Charlie 12 236 (36) 3

202 23.9 0.0 0.0 gi|157731950:154416095-154422431 1090 1135 (5202) + T-rich Low_complexity 135 180 (0) 4

199 23.6 18.9 1.8 gi|157731950:154416095-154422431 1302 1396 (4941) + L2a LINE/L2 3316 3426 (0) 1

312 9.1 0.0 0.0 gi|157731950:154416095-154422431 1981 2024 (4313) + (TA)n Simple_repeat 1 44 (0) 5

21 52.4 0.0 0.0 gi|157731950:154416095-154422431 2310 2330 (4007) + AT_rich Low_complexity 1 21 (0) 6

232 24.7 14.1 0.0 gi|157731950:154416095-154422431 2735 2819 (3518) C L1ME4a LINE/L1 (53) 6071 5975 7

2814 1.0 0.0 0.3 gi|157731950:154416095-154422431 3087 3386 (2951) C AluYa5 SINE/Alu (11) 299 1 8 (OCCUPIED)

809 27.5 10.9 3.0 gi|157731950:154416095-154422431 3441 3818 (2519) C L1ME4a LINE/L1 (318) 5806 5386 7

1129 26.2 5.0 7.5 gi|157731950:154416095-154422431 4160 4637 (1700) C L1ME3C LINE/L1 (9) 6114 5648 9

1398 12.5 11.8 0.2 gi|157731950:154416095-154422431 4740 5053 (1284) C L1PB3 LINE/L1 (31) 6123 5777 10

478 35.7 1.7 1.7 gi|157731950:154416095-154422431 5299 5589 (748) C L1ME3C LINE/L1 (1061) 5085 4795 9

707 32.1 3.7 6.3 gi|157731950:154416095-154422431 5676 6330 (7) C L1ME3C LINE/L1 (1495) 4651 4013 9

__________________________________________________________________________________________

AluYa5_5_222c

357 29.1 6.5 5.4 chr5 166222665 166222802 (14635064) + MIRb SINE/MIR 56 196 (72) 174

626 3.8 1.3 0.0 chr5 166222803 166222881 (14634985) C MADE1 DNA/Mariner (0) 80 1 175

357 29.1 6.5 5.4 chr5 166222882 166222927 (14634939) + MIRb SINE/MIR 197 241 (27) 174

256 34.4 9.7 2.7 chr5 166222933 166223117 (14634749) C MIR SINE/MIR (14) 248 51 176

231 8.8 0.0 0.0 chr5 166224099 166224132 (14633734) + (TG)n Simple_repeat 2 35 (0) 177

605 30.3 5.2 6.4 chr5 166224361 166224610 (14633256) C MIR SINE/MIR (6) 256 10 178

256 29.6 1.2 1.2 chr5 166225378 166225459 (14632407) + GA-rich Low_complexity 3 84 (0) 179

1335 9.1 0.0 0.6 chr5 166226544 166226719 (14631147) C AluSq SINE/Alu (16) 297 123 180

2550 5.8 0.0 0.6 chr5 166226720 166227031 (14630835) C AluYa5 SINE/Alu (0) 310 1 181 (AluYa5_5_222C)

618 23.5 11.3 2.5 chr5 166227457 166227669 (14630197) + MIRb SINE/MIR 30 262 (0) 182

494 34.3 9.9 0.5 chr5 166228092 166228302 (14629564) + MIR SINE/MIR 8 238 (24) 183

2423 8.8 0.0 0.0 chr5 166228436 166228741 (14629125) C AluSg SINE/Alu (0) 310 5 184

215 33.7 10.8 0.0 chr5 166228909 166228991 (14628875) C MIRb SINE/MIR (143) 125 34 185

642 8.7 6.9 0.0 chr5 166229953 166230189 (14627677) + (TA)n Simple_repeat 1 253 (0) 186

834 27.0 4.5 9.5 chr5 166230200 166230446 (14627420) C L1ME1 LINE/L1 (64) 6102 5849 187

509 1.5 0.0 0.0 chr5 166230447 166230512 (14627354) C L1PA2 LINE/L1 (0) 6155 6090 188

834 27.0 4.5 9.5 chr5 166230513 166230726 (14627140) C L1ME1 LINE/L1 (318) 5848 5664 187

436 33.3 1.4 6.2 chr5 166230900 166231107 (14626759) C MIRb SINE/MIR (58) 210 13 189

224 32.3 4.4 5.1 chr5 166231119 166231255 (14626611) C L2 LINE/L2 (1) 3418 3283 190

Ortholog in HuRef

231 8.8 0.0 0.0 gi|157734151:161391926-161397995 373 406 (5664) + (TG)n Simple_repeat 2 35 (0) 1

595 30.4 5.2 6.5 gi|157734151:161391926-161397995 635 884 (5186) C MIR SINE/MIR (6) 256 10 2

246 29.7 0.0 3.7 gi|157734151:161391926-161397995 1652 1735 (4335) + A-rich Low_complexity 6 86 (0) 3

300 34.3 4.7 1.7 gi|157734151:161391926-161397995 1781 1949 (4121) + Tigger10 DNA/TcMar-Tigger 501 674 (1169) 4

380 31.9 0.7 0.7 gi|157734151:161391926-161397995 2089 2224 (3846) + Tigger10 DNA/TcMar-Tigger 904 1039 (804) 5

225 21.4 1.3 5.3 gi|157734151:161391926-161397995 2305 2383 (3687) + Tigger10 DNA/TcMar-Tigger 1169 1244 (599) 6

513 33.6 0.9 6.0 gi|157734151:161391926-161397995 2572 2821 (3249) + Tigger10 DNA/TcMar-Tigger 1449 1698 (145) 7

2261 11.1 0.0 0.3 gi|157734151:161391926-161397995 2822 3119 (2951) C AluSc8 SINE/Alu (15) 297 1 8

513 33.6 0.9 6.0 gi|157734151:161391926-161397995 3120 3203 (2867) + Tigger10 DNA/TcMar-Tigger 1699 1767 (76) 7

260 27.8 0.8 5.8 gi|157734151:161391926-161397995 3261 3386 (2684) + Tigger15a DNA/TcMar-Tigger 472 591 (124) 9

604 23.5 11.3 2.3 gi|157734151:161391926-161397995 3545 3757 (2313) + MIRb SINE/MIR 30 262 (0) 10

477 34.3 9.9 0.4 gi|157734151:161391926-161397995 4180 4390 (1680) + MIR SINE/MIR 8 238 (24) 11

2505 8.5 0.0 0.0 gi|157734151:161391926-161397995 4527 4831 (1239) C AluSg SINE/Alu (1) 309 5 12

243 12.2 2.0 0.0 gi|157734151:161391926-161397995 6007 6055 (15) C L1ME2z LINE/L1 (280) 6164 6115 13

___________________________________________________________________________________

AluYa5_6_204 OCCUPIED

1241 19.5 8.6 14.4 chr6 135624879 135625163 (35274829) + MLT2D LTR/ERVL 88 392 (22) 300

317 12.5 0.2 1.6 chr6 135625174 135625237 (35274755) + MLT2B2 LTR/ERVL 503 567 (0) 300

335 33.3 4.7 3.0 chr6 135625568 135625802 (35274190) C L2 LINE/L2 (0) 3419 3181 302

1594 21.7 7.4 0.3 chr6 135626002 135626339 (35273653) C MLT1A0 LTR/MaLR (2) 363 2 303

249 31.2 2.6 0.0 chr6 135626501 135626577 (35273415) + L2 LINE/L2 3299 3377 (1) 304

878 19.6 0.5 7.8 chr6 135627159 135627363 (35272629) + MER53 DNA 2 191 (2) 305

2228 1.3 0.0 0.0 chr6 135627830 135628066 (35271926) + AluYa5 SINE/Alu 1 237 (73) 306 (AluYa5_6_204)

180 0.0 0.0 0.0 chr6 135628072 135628091 (35271901) + (A)n Simple_repeat 1 20 (0) 307

222 31.5 4.1 8.2 chr6 135628653 135628700 (35271292) + L2 LINE/L2 3298 3348 (71) 308

2144 15.5 0.5 9.8 chr6 135628701 135629017 (35270975) C MLT1B LTR/MaLR (2) 388 101 309

1758 16.7 4.8 0.3 chr6 135629018 135629311 (35270681) C AluJo SINE/Alu (5) 307 1 310

2144 15.5 0.5 9.8 chr6 135629312 135629421 (35270571) C MLT1B LTR/MaLR (290) 100 1 309

222 31.5 4.1 8.2 chr6 135629422 135629469 (35270523) + L2 LINE/L2 3349 3388 (31) 308

Ortholog in HuRef

1106 20.5 11.4 2.5 gi|157734152:133147049-133153281 8 291 (5942) + MLT2D LTR/ERVL 88 537 (22) 1

321 12.5 0.2 1.6 gi|157734152:133147049-133153281 296 359 (5874) + MLT2D LTR/ERVL 503 567 (0) 1

449 34.8 4.8 0.8 gi|157734152:133147049-133153281 690 918 (5315) C L2a LINE/L2 (0) 3426 3189 2

1581 21.7 7.4 0.3 gi|157734152:133147049-133153281 1124 1461 (4772) C MLT1A0 LTR/ERVL-MaLR (2) 363 2 3

287 30.3 2.9 3.2 gi|157734152:133147049-133153281 1589 1699 (4534) + L2b LINE/L2 3283 3386 (1) 4

873 19.2 0.5 8.4 gi|157734152:133147049-133153281 2281 2485 (3748) + MER53 DNA/hAT 2 191 (2) 5

2810 0.7 0.0 0.0 gi|157734152:133147049-133153281 2952 3247 (2986) + AluYa5 SINE/Alu 1 296 (14) 6 (OCCUPIED)

249 33.2 2.9 12.7 gi|157734152:133147049-133153281 3801 3856 (2377) + L2b LINE/L2 3246 3305 (70) 7

2066 15.4 0.5 14.9 gi|157734152:133147049-133153281 3857 4173 (2060) C MLT1B LTR/ERVL-MaLR (2) 388 100 8

1840 16.6 0.0 0.0 gi|157734152:133147049-133153281 4174 4451 (1782) C AluJr SINE/Alu (5) 307 30 9

2066 15.4 0.5 14.9 gi|157734152:133147049-133153281 4452 4577 (1656) C MLT1B LTR/ERVL-MaLR (291) 99 1 8

249 33.2 2.9 12.7 gi|157734152:133147049-133153281 4578 4658 (1575) + L2b LINE/L2 3306 3370 (5) 7

___________________________________________________________________________________________

AluYa5_7_82 OCCUPIED

1643 18.2 5.7 6.9 chr7 63604890 63605325 (95216099) + L1MA7 LINE/L1 5773 6203 (88) 2

1030 6.6 8.1 0.0 chr7 63605332 63605467 (95215957) C AluSp SINE/Alu (19) 294 148 3

406 16.7 0.0 0.0 chr7 63605468 63605545 (95215879) + L1MA7 LINE/L1 6211 6288 (3) 2

495 0.0 0.0 0.0 chr7 63605638 63605692 (95215732) + (CA)n Simple_repeat 2 56 (0) 4

231 13.3 0.0 0.0 chr7 63605916 63605960 (95215464) + (TA)n Simple_repeat 1 45 (0) 5

735 10.1 0.0 0.0 chr7 63605962 63606060 (95215364) C AluSg/x SINE/Alu (16) 296 198 6

7246 18.0 4.7 1.6 chr7 63606431 63607910 (95213514) + LTR25-int LTR/ERV1 262 1833 (5355) 7

2204 20.7 2.2 5.4 chr7 63607906 63608441 (95212983) + LTR25-int LTR/ERV1 2427 2945 (4243) 7

2776 1.9 0.0 0.0 chr7 63608442 63608748 (95212676) + AluYa5 SINE/Alu 1 307 (3) 8

631 23.1 7.3 11.4 chr7 63608767 63608802 (95212622) + LTR25-int LTR/ERV1 3182 3214 (3974) 7

2179 12.2 0.0 0.0 chr7 63608803 63609104 (95212320) + AluSg SINE/Alu 1 302 (8) 9

10694 19.4 6.6 8.8 chr7 63609105 63609589 (95211835) + LTR25-int LTR/ERV1 3215 3773 (3415) 7

2266 10.8 0.0 0.0 chr7 63609590 63609885 (95211539) + AluSq SINE/Alu 1 296 (17) 10

10694 17.3 6.2 7.1 chr7 63609886 63609980 (95211444) + LTR25-int LTR/ERV1 3774 3870 (3318) 7

2310 9.1 0.0 2.2 chr7 63609981 63610294 (95211130) + AluSc SINE/Alu 1 307 (2) 11

10694 17.3 6.2 7.1 chr7 63610295 63611463 (95209961) + LTR25-int LTR/ERV1 3871 5131 (2057) 7

2206 10.6 0.0 0.3 chr7 63611464 63611756 (95209668) C AluSc SINE/Alu (17) 292 1 12

Ortholog in HuRef

370 14.7 0.0 0.0 gi|157734172:60349008-60355264 1 68 (6189) + L1MA7 LINE/L1 6221 6288 (3) 1

333 0.0 0.0 0.0 gi|157734172:60349008-60355264 161 197 (6060) + (CA)n Simple_repeat 2 38 (0) 2

267 30.0 1.8 2.7 gi|157734172:60349008-60355264 205 317 (5940) + Charlie25 DNA/hAT-Charlie 1678 1789 (735) 3

285 11.8 0.0 0.0 gi|157734172:60349008-60355264 421 471 (5786) + (TA)n Simple_repeat 1 51 (0) 4

797 9.1 0.0 0.0 gi|157734172:60349008-60355264 473 571 (5686) C AluSz6 SINE/Alu (16) 296 198 5

5498 17.9 4.8 1.6 gi|157734172:60349008-60355264 942 2422 (3835) + LTR25-int LTR/ERV1 262 1833 (5355) 6

2210 20.7 2.2 5.2 gi|157734172:60349008-60355264 2418 2951 (3306) + LTR25-int LTR/ERV1 2427 2945 (4243) 6 *

2696 1.8 0.0 0.0 gi|157734172:60349008-60355264 2952 3265 (2992) + AluYa5 SINE/Alu 1 310 (0) 7 (OCCUPIED)

588 24.6 4.7 9.2 gi|157734172:60349008-60355264 3266 3296 (2961) + LTR25-int LTR/ERV1 3187 3214 (3974) 6

2293 11.6 0.0 0.0 gi|157734172:60349008-60355264 3297 3598 (2659) + AluSg4 SINE/Alu 1 302 (10) 8

2285 21.1 4.6 7.2 gi|157734172:60349008-60355264 3599 4080 (2177) + LTR25-int LTR/ERV1 3215 3773 (3415) 6

2371 10.1 0.0 0.0 gi|157734172:60349008-60355264 4081 4376 (1881) + AluSq2 SINE/Alu 1 296 (16) 9

2537 25.1 7.3 5.4 gi|157734172:60349008-60355264 4377 4471 (1786) + LTR25-int LTR/ERV1 1053 1149 (4068) 6

2366 8.9 0.0 2.3 gi|157734172:60349008-60355264 4472 4784 (1473) + AluSc SINE/Alu 1 306 (3) 10

3337 17.2 6.7 2.3 gi|157734172:60349008-60355264 4785 5942 (315) + LTR25-int LTR/ERV1 4108 5128 (2060) 6

2285 10.6 0.3 0.0 gi|157734172:60349008-60355264 5954 6246 (11) C AluSc8 SINE/Alu (18) 294 1 11

___________________________________________________________________________________

AluYa5_8_19 C_INTER_RMD

1624 10.4 0.0 0.0 chr8 17554431 17554641 (128720185) + AluSg/x SINE/Alu 89 299 (13) 495

617 18.0 0.9 0.9 chr8 17556604 17556715 (128718111) + FLAM_C SINE/Alu 9 120 (23) 496

244 21.8 13.8 2.5 chr8 17556906 17556985 (128717841) + L2 LINE/L2 3285 3373 (5) 497

1755 11.9 0.0 2.8 chr8 17557277 17557526 (128717300) + AluSx SINE/Alu 47 289 (23) 498

2214 11.7 0.0 0.0 chr8 17558035 17558334 (128716492) + AluSx SINE/Alu 1 300 (12) 499

2507 5.1 4.0 0.0 chr8 17558579 17558874 (128715952) + AluY SINE/Alu 1 308 (3) 500 (R1)

21 7.1 0.0 0.0 chr8 17558910 17558951 (128715875) + AT_rich Low_complexity 1 42 (0) 501

1277 19.1 10.1 5.2 chr8 17559027 17559075 (128715751) + AluJb SINE/Alu 1 50 (252) 502

2750 1.4 0.0 0.0 chr8 17559076 17559370 (128715456) + AluYa5 SINE/Alu 1 295 (15) 503 (AluYa5_8_19) (R2)

1277 19.1 10.1 5.2 chr8 17559371 17559607 (128715219) + AluJb SINE/Alu 51 301 (1) 502

652 22.3 0.6 0.6 chr8 17559637 17559812 (128715014) + (TA)n Simple_repeat 1 176 (0) 504

306 0.0 0.0 0.0 chr8 17559814 17559847 (128714979) + (CA)n Simple_repeat 2 35 (0) 505

1655 12.7 0.8 0.0 chr8 17559848 17560083 (128714743) C AluSg SINE/Alu (33) 277 40 506

363 28.4 11.0 7.8 chr8 17560816 17561006 (128713820) C MIRb SINE/MIR (68) 200 4 507

28 2.9 0.0 0.0 chr8 17561159 17561193 (128713633) + AT_rich Low_complexity 1 35 (0) 508

2218 10.4 0.3 1.3 chr8 17561204 17561504 (128713322) + AluSc SINE/Alu 1 298 (11) 509

Ortholog in HuRef

647 18.8 1.7 0.8 gi|157734173:16055872-16062034 932 1048 (5115) + AluJo SINE/Alu 1 118 (194) 1

255 26.7 1.7 0.0 gi|157734173:16055872-16062034 1261 1320 (4843) + L2a LINE/L2 3361 3421 (5) 2

1803 12.0 0.0 2.8 gi|157734173:16055872-16062034 1614 1872 (4291) + AluSx1 SINE/Alu 47 298 (14) 3

2255 11.3 0.0 0.0 gi|157734173:16055872-16062034 2372 2671 (3492) + AluSx1 SINE/Alu 1 300 (12) 4

2704 1.7 0.0 0.3 gi|157734173:16055872-16062034 2917 3212 (2951) + AluYa5 SINE/Alu 1 295 (15) 5 (R12)

1153 17.1 11.3 3.5 gi|157734173:16055872-16062034 3213 3451 (2712) + AluJb SINE/Alu 46 302 (10) 6

306 29.3 0.0 0.0 gi|157734173:16055872-16062034 3483 3598 (2565) + (TATATG)n Simple_repeat 5 120 (0) 7

315 28.9 3.5 5.7 gi|157734173:16055872-16062034 3599 3685 (2478) C L1MCa LINE/L1 (4536) 1972 1887 8

1663 12.7 0.8 0.0 gi|157734173:16055872-16062034 3686 3921 (2242) C AluSg SINE/Alu (33) 277 40 9

315 28.9 3.5 5.7 gi|157734173:16055872-16062034 3922 3976 (2187) C L1MCa LINE/L1 (4622) 1886 1834 8

416 27.8 11.0 7.6 gi|157734173:16055872-16062034 4654 4844 (1319) C MIRb SINE/MIR (68) 200 4 10

28 57.1 0.0 0.0 gi|157734173:16055872-16062034 4997 5031 (1132) + AT_rich Low_complexity 1 35 (0) 11

2228 10.3 0.3 1.3 gi|157734173:16055872-16062034 5042 5342 (821) + AluSc SINE/Alu 1 298 (11) 12

_______________________________________________________________________________________

AluYa5_9_103c

925 22.6 13.0 5.3 chr9 79418741 79419062 (60854190) C MLT1J2 LTR/MaLR (0) 450 104 524

450 0.0 0.0 0.0 chr9 79420439 79420488 (60852764) + (CA)n Simple_repeat 2 51 (0) 525

189 0.0 0.0 0.0 chr9 79420665 79420685 (60852567) + (TTG)n Simple_repeat 1 21 (0) 526

2363 9.5 0.0 0.3 chr9 79421601 79421905 (60851347) C AluY SINE/Alu (7) 304 1 527

822 30.2 15.5 3.4 chr9 79422021 79422860 (60850392) + L2 LINE/L2 2474 3405 (14) 528

204 31.9 3.2 5.6 chr9 79423098 79423186 (60850066) C MIR3 SINE/MIR (2) 206 120 529

760 18.6 23.0 10.1 chr9 79423187 79423317 (60849935) + MER33 DNA/MER1_type 1 190 (134) 530

360 0.0 0.0 0.0 chr9 79423318 79423357 (60849895) + (TA)n Simple_repeat 2 41 (0) 531

2906 0.3 0.0 0.0 chr9 79423359 79423668 (60849584) C AluYa5 SINE/Alu (0) 310 1 532

760 18.6 23.0 10.1 chr9 79423669 79423821 (60849431) + MER33 DNA/MER1_type 191 324 (0) 530

204 27.9 8.3 6.0 chr9 79423822 79423905 (60849347) C MIR SINE/MIR (89) 125 40 533

2024 13.3 2.0 0.0 chr9 79423943 79424236 (60849016) C AluSq SINE/Alu (13) 300 1 534

673 24.1 7.3 1.8 chr9 79424259 79424493 (60848759) + MIR SINE/MIR 16 262 (0) 535

225 9.4 0.0 0.0 chr9 79424823 79424854 (60848398) + (CA)n Simple_repeat 1 32 (0) 536

384 30.4 8.8 2.0 chr9 79424915 79425062 (60848190) C MIR3 SINE/MIR (12) 196 39 537

2271 10.5 0.0 0.7 chr9 79425304 79425609 (60847643) + AluSg SINE/Alu 2 305 (5) 538

22 0.0 0.0 0.0 chr9 79425639 79425660 (60847592) + AT_rich Low_complexity 1 22 (0) 539

1845 18.1 0.0 0.3 chr9 79425664 79425968 (60847284) C AluJb SINE/Alu (7) 305 2 540

916 17.8 1.3 1.3 chr9 79426667 79426825 (60846427) C FRAM SINE/Alu (17) 159 1 541

Ortholog in HuRef

2387 9.5 0.0 0.3 gi|157734174:50062865-50069108 1265 1569 (4675) C AluY SINE/Alu (7) 304 1 4

634 30.8 7.0 2.6 gi|157734174:50062865-50069108 1685 2084 (4160) + L2b LINE/L2 2474 2890 (529) 5

249 33.7 5.9 8.2 gi|157734174:50062865-50069108 2102 2524 (3720) + L2b LINE/L2 2948 3361 (14) 5

650 18.4 23.1 8.6 gi|157734174:50062865-50069108 2851 2981 (3263) + MER33 DNA/hAT-Charlie 1 190 (134) 6

180 0.0 0.0 0.0 gi|157734174:50062865-50069108 2982 3001 (3243) + (TA)n Simple_repeat 2 21 (0) 7

2778 0.3 0.0 0.0 gi|157734174:50062865-50069108 3002 3293 (2951) C AluYa5 SINE/Alu (18) 292 1 8 (OCCUPIED)

650 18.4 23.1 8.6 gi|157734174:50062865-50069108 3294 3446 (2798) + MER33 DNA/hAT-Charlie 191 324 (0) 6

2032 13.3 2.0 0.0 gi|157734174:50062865-50069108 3568 3861 (2383) C AluSq2 SINE/Alu (12) 300 1 9

618 26.5 3.5 1.0 gi|157734174:50062865-50069108 3921 4118 (2126) + MIR SINE/MIR 60 262 (0) 10

225 9.4 0.0 0.0 gi|157734174:50062865-50069108 4448 4479 (1765) + (CA)n Simple_repeat 1 32 (0) 11

326 30.3 8.8 1.9 gi|157734174:50062865-50069108 4540 4687 (1557) C MIR3 SINE/MIR (12) 196 39 12

2289 10.5 0.0 0.7 gi|157734174:50062865-50069108 4929 5232 (1012) + AluSg SINE/Alu 2 303 (7) 13

22 40.9 0.0 0.0 gi|157734174:50062865-50069108 5262 5283 (961) + AT_rich Low_complexity 1 22 (0) 14

1908 18.0 0.0 0.3 gi|157734174:50062865-50069108 5287 5591 (653) C AluJb SINE/Alu (7) 305 2 15

__________________________________________________________________________________________________________________________

AluYa5_9_172c C_INTER_RMD

503 29.4 6.6 10.6 chr9 130264972 130265078 (10008174) + L1MC4a LINE/L1 5680 5774 (2034) 113

1958 15.6 1.3 0.7 chr9 130265079 130265382 (10007870) + AluJb SINE/Alu 1 306 (6) 116

503 29.4 6.6 10.6 chr9 130265383 130265436 (10007816) + L1MC4a LINE/L1 5775 5829 (1979) 113

1441 18.9 11.0 0.0 chr9 130265437 130265700 (10007552) + AluJb SINE/Alu 1 293 (19) 117

2371 10.6 0.0 0.0 chr9 130265935 130266236 (10007016) C AluSx SINE/Alu (2) 310 9 118

2510 8.4 0.0 0.0 chr9 130266257 130266553 (10006699) C AluSg SINE/Alu (13) 297 1 119

1290 11.1 1.1 0.6 chr9 130266597 130266778 (10006474) C AluSg/x SINE/Alu (9) 303 121 120 (R2)

2738 2.9 0.0 1.3 chr9 130266781 130267094 (10006158) C AluYa5 SINE/Alu (0) 310 1 121 (AluYa5_9_172C) (R1)

2502 7.1 0.0 0.0 chr9 130267561 130267855 (10005397) + AluSc SINE/Alu 1 295 (14) 122

2066 13.0 0.3 1.0 chr9 130267960 130268270 (10004982) + AluSx SINE/Alu 3 311 (1) 123

1838 18.5 0.0 0.3 chr9 130268284 130268587 (10004665) + AluSx SINE/Alu 1 303 (9) 124

374 23.8 0.0 0.0 chr9 130268589 130268668 (10004584) C L1ME3A LINE/L1 (31) 6139 6060 125

198 0.0 0.0 0.0 chr9 130268745 130268766 (10004486) + (TA)n Simple_repeat 2 23 (0) 126

2753 4.5 0.0 0.6 chr9 130268796 130269108 (10004144) + AluY SINE/Alu 1 311 (0) 127

387 0.0 0.0 0.0 chr9 130269115 130269157 (10004095) + (TA)n Simple_repeat 1 43 (0) 128

1919 15.1 4.2 0.3 chr9 130269158 130269490 (10003762) C MER7A DNA/MER2_type (2) 344 1 129

2310 11.6 0.0 0.0 chr9 130269565 130269867 (10003385) + AluSx SINE/Alu 3 305 (7) 130

Ortholog in HuRef

614 16.3 4.7 9.1 gi|157734174:100836717-100842788 1195 1301 (4771) + L1MC4a LINE/L1 5685 5779 (345) 2

1960 15.5 1.3 0.7 gi|157734174:100836717-100842788 1302 1605 (4467) + AluJb SINE/Alu 1 306 (6) 5

614 14.2 4.1 8.6 gi|157734174:100836717-100842788 1606 1659 (4413) + L1MC4a LINE/L1 5775 5829 (2053) 2

1438 18.9 11.0 0.0 gi|157734174:100836717-100842788 1660 1923 (4149) + AluJb SINE/Alu 1 293 (19) 6

2327 9.9 0.0 0.0 gi|157734174:100836717-100842788 2158 2459 (3613) C AluSz6 SINE/Alu (2) 310 9 7

2478 7.4 0.3 0.0 gi|157734174:100836717-100842788 2480 2776 (3296) C AluSx4 SINE/Alu (14) 298 1 8

2234 11.7 0.7 0.3 gi|157734174:100836717-100842788 2814 3121 (2951) C AluSz SINE/Alu (3) 309 1 9 (R12)

2458 7.1 0.0 0.0 gi|157734174:100836717-100842788 3589 3882 (2190) + AluSc SINE/Alu 2 295 (14) 10

2105 12.9 0.3 1.0 gi|157734174:100836717-100842788 3987 4297 (1775) + AluSx SINE/Alu 3 311 (1) 11

1839 16.9 0.0 0.7 gi|157734174:100836717-100842788 4311 4606 (1466) + AluSx4 SINE/Alu 1 294 (18) 12

391 22.5 0.0 0.0 gi|157734174:100836717-100842788 4615 4694 (1378) C L1ME3A LINE/L1 (35) 6138 6059 13

198 0.0 0.0 0.0 gi|157734174:100836717-100842788 4771 4792 (1280) + (TA)n Simple_repeat 2 23 (0) 14

2729 4.5 0.0 0.6 gi|157734174:100836717-100842788 4822 5134 (938) + AluY SINE/Alu 1 311 (0) 15

315 0.0 0.0 0.0 gi|157734174:100836717-100842788 5136 5170 (902) + (TA)n Simple_repeat 1 35 (0) 16

2011 15.1 4.2 0.3 gi|157734174:100836717-100842788 5171 5503 (569) C Tigger3a DNA/TcMar-Tigger (2) 346 1 17

2311 10.7 0.0 0.0 gi|157734174:100836717-100842788 5578 5876 (196) + AluSx1 SINE/Alu 3 301 (11) 18

_______________________________________________________________________________________________________________________________

AluYa5_16_26 C_INTER_RMD

1212 16.9 4.1 0.9 chr16 21334146 21334259 (67492995) C MER30 DNA/MER1_type (0) 230 108 471

1982 11.0 1.0 1.4 chr16 21334260 21334555 (67492699) C AluSq SINE/Alu (18) 295 1 472

1959 14.7 0.0 0.0 chr16 21334559 21334870 (67492384) C AluSx SINE/Alu (0) 312 1 473

1212 16.9 4.1 0.9 chr16 21334871 21334972 (67492282) C MER30 DNA/MER1_type (123) 107 6 471

2137 11.0 0.3 0.0 chr16 21334996 21335294 (67491960) C AluSg SINE/Alu (10) 300 1 474

2315 9.0 0.3 0.0 chr16 21335532 21335841 (67491413) + AluY SINE/Alu 1 311 (0) 475

453 23.9 13.3 8.8 chr16 21336120 21336336 (67490918) + L1ME2 LINE/L1 5811 6046 (118) 476

2026 10.0 1.1 0.0 chr16 21336337 21336652 (67490602) + AluSp SINE/Alu 1 319 (0) 477

453 23.6 14.6 9.3 chr16 21336653 21336738 (67490516) + L1ME2 LINE/L1 6047 6130 (34) 476

2523 4.2 0.7 0.0 chr16 21336740 21337047 (67490207) + AluYa5 SINE/Alu 1 310 (0) 478 (AluYa5_16_26) (R1)

1250 7.8 7.3 0.0 chr16 21337068 21337246 (67490008) + AluSg/x SINE/Alu 115 306 (6) 479 (R2)

1333 16.4 0.0 1.7 chr16 21337319 21337559 (67489695) C AluJb SINE/Alu (61) 251 15 480

333 13.3 4.0 0.0 chr16 21337809 21337883 (67489371) + G-rich Low_complexity 3 80 (0) 481

245 15.8 0.0 0.0 chr16 21337904 21337941 (67489313) + GA-rich Low_complexity 1 38 (0) 482

367 28.4 9.7 2.9 chr16 21338601 21338879 (67488375) + L1M5 LINE/L1 2667 2964 (3182) 483

298 28.5 0.7 2.8 chr16 21339371 21339511 (67487743) C MER113 DNA/MER1_type (93) 428 291 484

2156 10.0 0.3 0.6 chr16 21339514 21339824 (67487430) + AluSg SINE/Alu 1 310 (0) 485

2364 7.3 0.0 1.6 chr16 21339892 21340198 (67487056) + AluSg SINE/Alu 1 302 (8) 486

208 24.1 3.3 4.9 chr16 21340473 21340533 (67486721) + MIRb SINE/MIR 44 103 (165) 487

676 30.4 4.0 2.5 chr16 21341757 21342077 (67485177) + MLT1I LTR/MaLR 74 399 (10) 417

Ortholog in HuRef

413 16.5 0.0 0.0 gi|157713457:14861653-14867764 39 117 (5995) C AluJb SINE/Alu (1) 311 233 1

296 26.5 1.4 3.6 gi|157713457:14861653-14867764 354 494 (5618) C MER113 DNA/hAT-Charlie (93) 428 291 2

2271 10.6 0.3 0.7 gi|157713457:14861653-14867764 497 807 (5305) + AluSg SINE/Alu 1 310 (0) 3

2289 10.0 0.0 1.7 gi|157713457:14861653-14867764 875 1173 (4939) + AluSg SINE/Alu 1 294 (16) 4

198 28.6 1.5 4.7 gi|157713457:14861653-14867764 1445 1510 (4602) + MIR SINE/MIR 38 101 (161) 5

2007 10.8 1.1 0.0 gi|157713457:14861653-14867764 2589 2865 (3247) + AluSp SINE/Alu 28 307 (6) 6

242 24.7 5.9 0.0 gi|157713457:14861653-14867764 2866 2950 (3162) + L1ME2z LINE/L1 6047 6136 (308) 7

1840 7.3 0.6 20.1 gi|157713457:14861653-14867764 2952 3308 (2804) + AluSc SINE/Alu 1 299 (10) 8 (R12)

1277 16.5 0.4 1.7 gi|157713457:14861653-14867764 3381 3620 (2492) C AluJb SINE/Alu (61) 251 15 1

275 25.7 5.2 2.9 gi|157713457:14861653-14867764 4601 4736 (1376) C MER113 DNA/hAT-Charlie (93) 428 290 9

2260 10.6 0.3 0.7 gi|157713457:14861653-14867764 4743 5053 (1059) + AluSg SINE/Alu 1 310 (0) 10

2232 9.9 1.0 0.0 gi|157713457:14861653-14867764 5121 5414 (698) + AluSx4 SINE/Alu 1 297 (15) 11

354 2.3 0.0 0.0 gi|157713457:14861653-14867764 5425 5467 (645) + (TG)n Simple_repeat 2 44 (0) 12

2101 13.2 1.0 0.0 gi|157713457:14861653-14867764 5783 6078 (34) C AluSz6 SINE/Alu (13) 299 1 13

___________________________________________________________________________________________

AluYa5_16_28c C_INTER_RMD

2498 7.3 0.0 1.6 chr16 22436354 22436660 (66390594) C AluSg SINE/Alu (8) 302 1 733

2275 8.4 0.3 0.0 chr16 22436751 22437034 (66390220) C AluSg SINE/Alu (25) 285 1 734

305 28.5 0.7 2.8 chr16 22437037 22437177 (66390077) + MER113 DNA/MER1_type 291 428 (93) 735

401 27.9 9.1 3.0 chr16 22437685 22437947 (66389307) C L1M5 LINE/L1 (3201) 2945 2667 736

245 15.8 0.0 0.0 chr16 22438607 22438644 (66388610) + CT-rich Low_complexity 1 38 (0) 737

333 13.3 4.0 0.0 chr16 22438665 22438739 (66388515) + C-rich Low_complexity 3 80 (0) 738

1419 16.4 0.0 1.7 chr16 22438989 22439229 (66388025) + AluJb SINE/Alu 15 251 (61) 739

1323 7.8 7.3 0.0 chr16 22439302 22439480 (66387774) C AluSg/x SINE/Alu (6) 306 115 740 (R2)

2657 4.2 0.7 0.0 chr16 22439502 22439809 (66387445) C AluYa5 SINE/Alu (0) 310 1 741 (R1)

454 23.6 14.6 9.3 chr16 22439811 22439896 (66387358) C L1ME2 LINE/L1 (34) 6130 6047 742

2149 10.0 1.1 0.0 chr16 22439897 22440212 (66387042) C AluSp SINE/Alu (0) 319 1 743

454 23.9 13.3 8.8 chr16 22440213 22440429 (66386825) C L1ME2 LINE/L1 (118) 6046 5811 742

2315 9.0 0.3 0.0 chr16 22440707 22441016 (66386238) C AluY SINE/Alu (0) 311 1 744

2137 11.0 0.3 0.0 chr16 22441255 22441553 (66385701) + AluSg SINE/Alu 1 300 (10) 745

1212 16.9 4.1 0.9 chr16 22441577 22441678 (66385576) + MER30 DNA/MER1_type 3 104 (126) 746

1959 14.7 0.0 0.0 chr16 22441679 22441990 (66385264) + AluSx SINE/Alu 1 312 (0) 747

1982 11.0 1.0 1.4 chr16 22441994 22442289 (66384965) + AluSq SINE/Alu 1 295 (18) 748

1212 16.9 4.1 0.9 chr16 22442290 22442403 (66384851) + MER30 DNA/MER1_type 105 227 (3) 746

Ortholog in HuRef

2101 13.2 1.0 0.0 gi|157713457:14861653-14867764 35 330 (5782) + AluSz6 SINE/Alu 1 299 (13) 1

354 2.3 0.0 0.0 gi|157713457:14861653-14867764 646 688 (5424) + (CA)n Simple_repeat 2 44 (0) 2

2232 9.9 1.0 0.0 gi|157713457:14861653-14867764 699 992 (5120) C AluSx4 SINE/Alu (15) 297 1 3

2260 10.6 0.3 0.7 gi|157713457:14861653-14867764 1060 1370 (4742) C AluSg SINE/Alu (0) 310 1 4

275 25.7 5.2 2.9 gi|157713457:14861653-14867764 1377 1512 (4600) + MER113 DNA/hAT-Charlie 290 428 (93) 5

1277 16.5 0.4 1.7 gi|157713457:14861653-14867764 2493 2732 (3380) + AluJb SINE/Alu 15 251 (61) 6

1840 7.3 0.6 20.1 gi|157713457:14861653-14867764 2805 3161 (2951) C AluSc SINE/Alu (10) 299 1 7 (R12)

242 24.7 5.9 0.0 gi|157713457:14861653-14867764 3163 3247 (2865) C L1ME2z LINE/L1 (308) 6136 6047 8

2007 10.8 1.1 0.0 gi|157713457:14861653-14867764 3248 3524 (2588) C AluSp SINE/Alu (6) 307 28 9

198 28.6 1.5 4.7 gi|157713457:14861653-14867764 4603 4668 (1444) C MIR SINE/MIR (161) 101 38 10

2289 10.0 0.0 1.7 gi|157713457:14861653-14867764 4940 5238 (874) C AluSg SINE/Alu (16) 294 1 11

2271 10.6 0.3 0.7 gi|157713457:14861653-14867764 5306 5616 (496) C AluSg SINE/Alu (0) 310 1 12

296 26.5 1.4 3.6 gi|157713457:14861653-14867764 5619 5759 (353) + MER113 DNA/hAT-Charlie 291 428 (93) 13

413 16.5 0.0 0.0 gi|157713457:14861653-14867764 5996 6074 (38) + AluJb SINE/Alu 233 311 (1) 6

_____________________________________________________________________________________

AluYa5_16_67 C_INTER_RMD

260 24.0 29.0 0.0 chr16 55129580 55129679 (33697575) C L1MC4a LINE/L1 (1885) 5923 5795 283

1878 15.8 0.6 1.3 chr16 55129798 55130111 (33697143) + AluJo SINE/Alu 1 312 (0) 284

828 25.8 0.5 1.8 chr16 55130323 55130539 (33696715) C MIR SINE/MIR (37) 225 12 285

189 32.6 10.9 3.3 chr16 55130561 55130652 (33696602) C L2 LINE/L2 (13) 3365 3267 286

190 27.4 18.2 3.3 chr16 55130841 55130875 (33696379) C MIRb SINE/MIR (108) 160 107 287

1710 16.2 0.4 0.4 chr16 55130876 55131147 (33696107) + AluJo SINE/Alu 23 294 (18) 288

190 27.4 18.2 3.3 chr16 55131148 55131232 (33696022) C MIRb SINE/MIR (162) 106 23 287

2561 13.9 3.5 4.6 chr16 55131515 55131967 (33695287) + LTR26 LTR/ERV1 1 448 (155) 289

2540 5.5 0.0 1.0 chr16 55131968 55132277 (33694977) + AluYa5 SINE/Alu 1 307 (3) 290 (AluYa5_16_67) (R1)

1184 13.9 0.0 0.0 chr16 55132279 55132451 (33694803) + AluSg/x SINE/Alu 134 306 (6) 291 (R2)

974 13.5 6.4 0.0 chr16 55132453 55132608 (33694646) + LTR26 LTR/ERV1 438 603 (0) 289

303 22.0 3.9 2.9 chr16 55132625 55132727 (33694527) C MER113 DNA/MER1_type (7) 514 411 292

2071 12.1 0.3 2.0 chr16 55132744 55133039 (33694215) + AluSq SINE/Alu 1 291 (22) 293

448 22.7 9.8 0.0 chr16 55133348 55133479 (33693775) + MIR SINE/MIR 33 177 (85) 294

399 27.6 6.2 14.1 chr16 55133490 55133793 (33693461) + L2 LINE/L2 3135 3414 (5) 295

395 25.6 7.7 11.4 chr16 55134160 55134247 (33693007) C MIRb SINE/MIR (8) 260 167 296

1778 16.6 1.0 0.3 chr16 55134248 55134549 (33692705) + AluJo SINE/Alu 4 307 (5) 297

395 25.7 7.2 10.3 chr16 55134550 55134700 (33692554) C MIRb SINE/MIR (102) 166 28 296

Ortholog in HuRef

243 28.6 4.3 0.0 gi|157713457:42441759-42447830 565 634 (5438) C L1MC4a LINE/L1 (1959) 5923 5851 3

1957 15.3 0.6 1.3 gi|157713457:42441759-42447830 783 1095 (4977) + AluJr SINE/Alu 1 311 (1) 4

924 30.0 0.4 1.6 gi|157713457:42441759-42447830 1270 1523 (4549) C MIR SINE/MIR (0) 262 12 5

220 27.5 13.7 6.2 gi|157713457:42441759-42447830 1545 1728 (4344) C L2b LINE/L2 (13) 3374 3182 6

222 26.4 18.2 2.9 gi|157713457:42441759-42447830 1825 1859 (4213) C MIRb SINE/MIR (108) 160 112 7

1710 16.2 0.4 0.4 gi|157713457:42441759-42447830 1860 2131 (3941) + AluJo SINE/Alu 23 294 (18) 8

222 26.4 18.2 2.9 gi|157713457:42441759-42447830 2132 2216 (3856) C MIRb SINE/MIR (157) 111 23 7

3242 15.3 4.3 5.6 gi|157713457:42441759-42447830 2499 2951 (3121) + LTR26 LTR/ERV1 1 448 (155) 9

2269 10.9 0.0 2.3 gi|157713457:42441759-42447830 2952 3263 (2809) + AluSx SINE/Alu 1 305 (7) 10 (R12)

3242 15.3 4.3 5.6 gi|157713457:42441759-42447830 3264 3420 (2652) + LTR26 LTR/ERV1 449 603 (0) 9

416 21.7 9.9 4.9 gi|157713457:42441759-42447830 3437 3555 (2517) C MER113A DNA/hAT-Charlie (7) 307 183 11

2250 11.9 0.0 0.3 gi|157713457:42441759-42447830 3556 3858 (2214) + AluSq2 SINE/Alu 1 302 (10) 12

416 21.7 9.9 4.9 gi|157713457:42441759-42447830 3859 3990 (2082) C MER113A DNA/hAT-Charlie (132) 182 44 11

190 20.8 1.8 3.7 gi|157713457:42441759-42447830 4064 4118 (1954) + MIR3 SINE/MIR 81 134 (74) 13

506 24.0 11.0 0.0 gi|157713457:42441759-42447830 4151 4304 (1768) + MIR SINE/MIR 15 185 (77) 14

440 33.1 4.3 10.4 gi|157713457:42441759-42447830 4307 4610 (1462) + L2a LINE/L2 3135 3421 (5) 15

464 25.2 7.7 11.8 gi|157713457:42441759-42447830 4977 5064 (1008) C MIRb SINE/MIR (8) 260 171 16

1834 16.9 1.0 0.3 gi|157713457:42441759-42447830 5065 5366 (706) + AluJo SINE/Alu 4 307 (5) 17

464 25.2 7.7 11.8 gi|157713457:42441759-42447830 5367 5497 (575) C MIRb SINE/MIR (98) 170 49 16

__________________________________________________________________________________

AluYa5_17_48

1819 16.4 0.3 3.2 chr17 32212904 32213217 (46561525) C AluJo SINE/Alu (7) 305 1 751

707 29.7 2.9 1.7 chr17 32213224 32213463 (46561279) C MIRb SINE/MIR (6) 262 20 752

402 33.0 8.0 1.8 chr17 32213843 32214067 (46560675) + MIRb SINE/MIR 22 260 (8) 753

398 30.1 11.5 0.6 chr17 32214684 32214840 (46559902) C MIRb SINE/MIR (94) 174 1 754

807 28.1 13.7 5.0 chr17 32215263 32215787 (46558955) C MLT1K LTR/MaLR (7) 584 14 755

1419 22.2 5.8 2.6 chr17 32215918 32216296 (46558446) C LTR16A1 LTR/ERVL (2) 455 65 756

2384 10.6 0.0 0.0 chr17 32216897 32217197 (46557545) + AluSg SINE/Alu 1 301 (9) 757

594 28.9 11.6 1.8 chr17 32217228 32217547 (46557195) + L1ME4a LINE/L1 5219 5561 (579) 758

2906 1.0 0.0 0.0 chr17 32217551 32217858 (46556884) + AluYa5 SINE/Alu 1 308 (2) 759 (AluYa5_17_48) (R1)

1957 18.6 0.0 0.6 chr17 32217873 32218173 (46556569) + AluJb SINE/Alu 1 300 (12) 760 (R2)

1049 11.8 0.0 0.6 chr17 32218174 32218343 (46556399) + (GGAA)n Simple_repeat 4 172 (0) 761

443 14.3 4.4 1.8 chr17 32218362 32218475 (46556267) + GA-rich Low_complexity 2 118 (0) 762

1071 28.8 4.0 2.1 chr17 32218476 32218750 (46555992) + L1ME4a LINE/L1 5559 5829 (292) 758

2027 14.2 0.0 2.0 chr17 32218751 32219045 (46555697) + AluJo SINE/Alu 12 300 (12) 763

1071 28.8 4.0 2.1 chr17 32219046 32219198 (46555544) + L1ME4a LINE/L1 5830 5995 (126) 758

1409 24.6 10.7 4.0 chr17 32219409 32219984 (46554758) C MLT1G LTR/MaLR (14) 581 1 764

362 30.1 10.2 4.2 chr17 32219989 32220272 (46554470) + MLT1K LTR/MaLR 291 591 (0) 765

1696 17.7 0.7 3.2 chr17 32220300 32220322 (46554420) C AluJo SINE/Alu (0) 312 279 766

Ortholog in HuRef

413 30.1 11.5 0.6 gi|157713538:31076867-31083131 84 240 (6025) C MIRb SINE/MIR (94) 174 1 1

582 23.0 11.9 1.5 gi|157713538:31076867-31083131 664 848 (5417) C MLT1K LTR/ERVL-MaLR (8) 587 384 2

393 30.4 5.2 0.0 gi|157713538:31076867-31083131 1053 1187 (5078) C MLT1K LTR/ERVL-MaLR (440) 155 14 2

1513 23.2 5.5 2.5 gi|157713538:31076867-31083131 1318 1702 (4563) C LTR16A1 LTR/ERVL (2) 454 59 3

2355 10.6 0.0 0.0 gi|157713538:31076867-31083131 2297 2598 (3667) + AluSg SINE/Alu 1 302 (8) 4

775 24.5 12.2 3.8 gi|157713538:31076867-31083131 2629 2954 (3311) + L1M5 LINE/L1 5219 5570 (554) 5

1971 17.5 0.0 0.0 gi|157713538:31076867-31083131 2955 3251 (3014) + AluJb SINE/Alu 1 297 (15) 6 (R12)

810 17.0 0.6 1.1 gi|157713538:31076867-31083131 3257 3434 (2831) + GA-rich Low_complexity 4 180 (0) 7

457 12.7 2.5 2.5 gi|157713538:31076867-31083131 3451 3531 (2734) + GA-rich Low_complexity 2 82 (0) 7

1080 24.6 0.8 0.4 gi|157713538:31076867-31083131 3532 3796 (2469) + L1ME4a LINE/L1 5562 5827 (297) 8

2033 13.5 0.0 2.1 gi|157713538:31076867-31083131 3808 4103 (2162) + AluJr SINE/Alu 12 301 (11) 9

1563 24.3 8.0 7.1 gi|157713538:31076867-31083131 4467 5042 (1223) C MLT1G3 LTR/ERVL-MaLR (14) 581 1 10

370 28.4 11.1 3.7 gi|157713538:31076867-31083131 5079 5330 (935) + MLT1K LTR/ERVL-MaLR 326 595 (0) 11

927 6.3 2.1 1.4 gi|157713538:31076867-31083131 5391 5534 (731) + (TTTC)n Simple_repeat 3 147 (0) 12

1567 17.3 1.7 3.5 gi|157713538:31076867-31083131 5535 5817 (448) C AluJb SINE/Alu (22) 280 1 13

1670 19.2 1.7 1.0 gi|157713538:31076867-31083131 5882 6168 (97) C AluJb SINE/Alu (22) 290 2 14

__________________________________________________________________________________

AluYa5_19_28 C_INTER_RMD

26 5.0 0.0 0.0 chr19 21301580 21301619 (42510032) + AT_rich Low_complexity 1 40 (0) 301

2579 6.4 0.0 0.6 chr19 21301890 21302203 (42509448) + AluSp SINE/Alu 1 312 (1) 302

314 18.8 1.4 2.8 chr19 21303202 21303272 (42508379) + MER93a LTR/ERV1 1 70 (332) 303

2446 6.1 0.0 0.7 chr19 21304333 21304627 (42507024) C AluSp SINE/Alu (20) 293 1 304

2132 11.2 0.7 1.0 chr19 21304743 21305048 (42506603) + AluSq SINE/Alu 5 309 (4) 305

293 9.1 2.3 0.0 chr19 21305051 21305094 (42506557) C MER33 DNA/MER1_type (18) 306 262 306

1204 12.6 0.0 0.0 chr19 21305098 21305272 (42506379) C AluSg/x SINE/Alu (7) 305 131 307

744 27.4 7.6 0.4 chr19 21305309 21305546 (42506105) C MER33 DNA/MER1_type (69) 255 1 306

2892 0.7 0.0 0.0 chr19 21305680 21305989 (42505662) + AluYa5 SINE/Alu 1 310 (0) 308 (R1) (AluYa5_19_28)

1310 8.0 4.0 0.0 chr19 21305993 21306168 (42505483) + AluSg/x SINE/Alu 129 311 (1) 309 (R2)

1336 28.4 9.9 1.4 chr19 21306628 21307124 (42504527) C MLT1H LTR/MaLR (0) 549 11 310

299 26.9 15.1 0.0 chr19 21307170 21307262 (42504389) C MIR SINE/MIR (145) 117 11 311

1525 21.1 0.0 2.7 chr19 21307411 21307707 (42503944) + AluJb SINE/Alu 10 298 (14) 312

1747 17.4 0.3 0.3 chr19 21307791 21308089 (42503562) + AluSp SINE/Alu 1 299 (14) 313

Ortholog in HuRef

875 10.6 0.8 0.0 gi|157718668:21000652-21006732 720 842 (5239) + MER57E3 LTR/ERV1 1 124 (363) 1

1333 15.7 0.0 0.9 gi|157718668:21000652-21006732 850 1074 (5007) + MER57E3 LTR/ERV1 265 487 (0) 1

2454 6.1 0.0 0.7 gi|157718668:21000652-21006732 1909 2203 (3878) C AluSp SINE/Alu (20) 293 1 2

708 27.5 7.6 0.4 gi|157718668:21000652-21006732 2585 2821 (3260) C MER33 DNA/hAT-Charlie (70) 254 1 3

2427 6.7 2.3 0.0 gi|157718668:21000652-21006732 2955 3254 (2827) + AluSg SINE/Alu 1 307 (3) 4 (R12)

1112 30.5 4.3 0.9 gi|157718668:21000652-21006732 3714 4210 (1871) C MLT1H LTR/ERVL-MaLR (0) 549 11 5

267 25.6 6.3 1.2 gi|157718668:21000652-21006732 4262 4340 (1741) C MIRb SINE/MIR (156) 112 30 6

1586 20.5 0.0 2.8 gi|157718668:21000652-21006732 4497 4793 (1288) + AluJb SINE/Alu 10 298 (14) 7

1787 17.8 0.3 0.3 gi|157718668:21000652-21006732 4877 5174 (907) + AluSp SINE/Alu 1 298 (15) 8

2234 11.9 0.3 0.3 gi|157718668:21000652-21006732 5223 5525 (556) + AluSq2 SINE/Alu 1 303 (9) 9

1672 23.0 1.2 2.0 gi|157718668:21000652-21006732 5576 5979 (102) C MER57B2 LTR/ERV1 (2) 401 1 10

437 19.6 0.0 4.1 gi|157718668:21000652-21006732 5980 6080 (1) C MER57-int LTR/ERV1 (0) 7537 7441 10

____________________________________________________________________________________________________________

AluYa5_22_19 OCCUPIED

956 11.8 0.0 4.2 chr22 34225569 34225710 (15465722) + AluSq/x SINE/Alu 1 136 (176) 633

250 21.8 0.0 1.8 chr22 34225717 34225772 (15465660) + MIR SINE/MIR 151 205 (57) 634

211 23.6 3.6 0.0 chr22 34225808 34225862 (15465570) C MIR3 SINE/MIR (5) 203 147 635

3731 15.2 1.9 1.8 chr22 34226023 34226752 (15464680) + L1MA5 LINE/L1 4126 4856 (1287) 636

2102 14.7 0.0 0.0 chr22 34226864 34227175 (15464257) C AluSx SINE/Alu (0) 312 1 637

2485 9.3 0.0 0.0 chr22 34227356 34227657 (15463775) + AluSg SINE/Alu 6 307 (3) 638

24 0.0 0.0 0.0 chr22 34227731 34227754 (15463678) + AT_rich Low_complexity 1 24 (0) 639

1370 5.6 0.0 0.0 chr22 34228318 34228478 (15462954) + AluYa5 SINE/Alu 150 310 (0) 640 (AluYa5_22_19)

504 0.0 0.0 0.0 chr22 34228480 34228535 (15462897) + (TA)n Simple_repeat 2 57 (0) 641

2256 14.6 4.1 1.0 chr22 34229661 34230076 (15461356) + L1MA5 LINE/L1 4842 5270 (873) 636

520 12.9 3.5 0.0 chr22 34230078 34230162 (15461270) + AluJ/FLAM SINE/Alu 1 88 (224) 642

5511 15.3 3.4 1.6 chr22 34230163 34231178 (15460254) + L1MA5 LINE/L1 5265 6298 (2) 636

764 22.0 1.3 0.0 chr22 34231501 34231650 (15459782) C L1ME1 LINE/L1 (5) 6161 6010 643

1635 18.2 7.5 8.3 chr22 34231912 34232354 (15459078) C L1ME1 LINE/L1 (155) 6011 5573 643

2212 13.4 0.0 0.0 chr22 34233001 34233306 (15458126) C AluSx SINE/Alu (6) 306 1 644

Ortholog in HuRef

1574 8.2 1.0 0.0 gi|157729478:18855430-18861534 1 194 (5911) + AluSx3 SINE/Alu 105 300 (12) 1

972 10.1 0.0 4.5 gi|157729478:18855430-18861534 203 341 (5764) + AluSz SINE/Alu 1 133 (179) 2

256 22.2 0.5 1.6 gi|157729478:18855430-18861534 342 406 (5699) + MIR SINE/MIR 150 205 (57) 3

210 29.7 3.0 4.5 gi|157729478:18855430-18861534 447 513 (5592) C L3b LINE/CR1 (6) 4502 4437 4

3664 15.2 1.9 1.8 gi|157729478:18855430-18861534 657 1386 (4719) + L1MA5 LINE/L1 4126 4856 (1287) 5

2178 14.4 0.0 0.0 gi|157729478:18855430-18861534 1498 1809 (4296) C AluSx1 SINE/Alu (0) 312 1 6

2471 9.3 0.0 0.0 gi|157729478:18855430-18861534 1990 2291 (3814) + AluSg SINE/Alu 6 307 (3) 7

24 41.7 0.0 0.0 gi|157729478:18855430-18861534 2366 2389 (3716) + AT_rich Low_complexity 1 24 (0) 8

1341 5.8 0.0 0.0 gi|157729478:18855430-18861534 2952 3105 (3000) + AluYa5 SINE/Alu 150 303 (7) 9 (OCCUPIED)

23 56.5 0.0 0.0 gi|157729478:18855430-18861534 3106 3128 (2977) + AT_rich Low_complexity 1 23 (0) 10

2196 14.6 4.1 0.9 gi|157729478:18855430-18861534 4250 4665 (1440) + L1MA5 LINE/L1 4842 5270 (873) 5

630 11.9 3.6 0.0 gi|157729478:18855430-18861534 4668 4751 (1354) + AluJr SINE/Alu 2 88 (224) 11

3253 18.5 4.0 1.3 gi|157729478:18855430-18861534 4752 5767 (338) + L1MA5 LINE/L1 5262 6298 (2) 5
