# Supplementary material for: RISCI - Repeat Induced Sequence Changes Identifier: a comprehensive, comparative genomics-based, in silico subtractive hybridization pipeline to identify repeat induced sequence changes in closely related genomes
Source: BMC Bioinformatics. 2010 Dec 26;11:609. doi: 10.1186/1471-2105-11-609 (PMC3024322; doi:10.1186/1471-2105-11-609)
Supplement: Additional file 9 — Pairwise alignment and RepeatMasker annotation of repeat locus in main genome and the ortholog in comparative genome for truncated AluYa5. Summary of pair-wise alignments between the AluYa5 loci in the reference genome and the identified orthologs in the comparative genome and its RepeatMasker annotation to confirm RISCI annotation. [file 1471-2105-11-609-S9.DOC]

**Additional file 9 - AluYa5 comparison with Chimpanzee genome**

**The file summarizes the blast2 result of the identified ortholog with the AluYa5 repeat and also gives the repeat masker annotation of the identified orthologous locus, along with N details.**

**OFC – Ortholog start coordinate, OLC – ortholog end coordinate, RFC – Repeat start coordinate, RLC – Repeat end coordinate**

**1. AluYa5_10_100**

**Ortholog annotation INDEL_PTS Length 6107 nscore 7.91 NPOSITIONS 3095 3577 ;**

**Repeat length (main genome) 308**

**Blast2 Results -**

**OFC OLC RFC RLC**

**11 61 11 61**

**254 287 240 273**

**779 1053 22 293**

**3606 3655 12 61**

**3749 3867 155 273**

**5660 5952 1 292**

**Ortholog Repeat Masker annotation**

**SW perc perc perc query position in query matching repeat position in repeat**

**score div. del. ins. sequence begin end (left) repeat class/family begin end (left) ID**

**1661 17.5 0.3 4.8 AluYa5_10_100 1 294 (5813) + AluJo SINE/Alu 1 281 (31) 1**

**2317 11.2 0.0 0.0 AluYa5_10_100 407 711 (5396) C AluSx SINE/Alu (7) 305 1 2**

**2124 10.7 0.7 1.4 AluYa5_10_100 759 1053 (5054) + AluSx SINE/Alu 2 294 (18) 3**

**2008 11.9 5.1 0.7 AluYa5_10_100 1197 1491 (4616) C AluSx SINE/Alu (2) 310 3 4**

**1998 27.2 24.4 2.1 AluYa5_10_100 1786 2309 (3798) C L2a LINE/L2 (297) 3129 2489 5**

**1811 18.7 0.3 0.0 AluYa5_10_100 2310 2609 (3498) C AluJo SINE/Alu (7) 305 5 6**

**1998 26.9 26.7 2.0 AluYa5_10_100 2610 3061 (3046) C L2a LINE/L2 (931) 2488 1925 5**

**1902 14.9 0.4 0.0 AluYa5_10_100 3595 3870 (2237) + AluJo SINE/Alu 1 277 (35) 7**

**1479 24.9 7.8 6.3 AluYa5_10_100 4049 4841 (1266) + L1MC4 LINE/L1 7198 8002 (40) 8**

**1827 25.5 15.1 3.8 AluYa5_10_100 4880 5659 (448) C L2a LINE/L2 (1443) 1976 1107 5**

**2432 8.9 0.3 0.0 AluYa5_10_100 5660 5963 (144) + AluSp SINE/Alu 1 305 (8) 9**

**1827 25.5 15.1 3.8 AluYa5_10_100 5964 6002 (105) C L2a LINE/L2 (2313) 1106 1064 5**

**______________________________________________________________________________________**

**2. AluYa5_10_117c**

**Ortholog annotation C_INTER_RMD_M_DISRUPTED Length 265 nscore 0.00 NPOSITIONS NA**

**Repeat length (main genome) 301**

**Blast2 Results -**

**OFC OLC RFC RLC**

**1 264 1 266**

**Ortholog Repeat Masker annotation**

**SW perc perc perc query position in query matching repeat position in repeat**

**score div. del. ins. sequence begin end (left) repeat class/family begin end (left) ID**

**2046 10.6 0.4 0.0 AluYa5_10_117c 1 265 (0) + AluSg SINE/Alu 2 267 (43) 1**

**__________________________________________________________________________________**

**3. AluYa5_10_20**

**Ortholog annotation INDEL_CAN Length 320 nscore 0.00 NPOSITIONS NA**

**Repeat length (main genome) 274**

**Blast2 Results -**

**OFC OLC RFC RLC**

**11 95 11 95**

**178 288 145 257**

**Ortholog Repeat Masker annotation**

**SW perc perc perc query position in query matching repeat position in repeat**

**score div. del. ins. sequence begin end (left) repeat class/family begin end (left) ID**

**1892 11.3 1.7 3.7 AluYa5_10_20 5 304 (16) + AluY SINE/Alu 5 311 (0) 1**

**1892 11.3 1.7 3.7 AluYa5_10_20 305 317 (3) + AluY SINE/Alu 281 292 (19) 1**

**__________________________________________________________________________________**

**4. AluYa5_10_28c**

**Ortholog annotation C_INTER_RMD Length 285 nscore 0.00 NPOSITIONS NA**

**Repeat length (main genome) 298**

**Blast2 Results -**

**OFC OLC RFC RLC**

**1 285 1 285**

**Ortholog Repeat Masker annotation**

**SW perc perc perc query position in query matching repeat position in repeat**

**score div. del. ins. sequence begin end (left) repeat class/family begin end (left) ID**

**1967 7.0 4.6 4.2 AluYa5_10_28c 1 285 (0) + AluY SINE/Alu 8 293 (18) 1**

**__________________________________________________________________________________**

**5. AluYa5_10_33c**

**Ortholog annotation INDEL_PTS Length 1241 nscore 18.78 NPOSITIONS 561 793 ;**

**Repeat length (main genome) 302**

**Blast2 Results -**

**OFC OLC RFC RLC**

**11 301 11 302**

**Ortholog Repeat Masker annotation**

**SW perc perc perc query position in query matching repeat position in repeat**

**score div. del. ins. sequence begin end (left) repeat class/family begin end (left) ID**

**2354 9.2 0.7 0.0 AluYa5_10_33c 1 306 (935) + AluSx SINE/Alu 2 309 (3) 1**

**1308 18.8 0.8 2.4 AluYa5_10_33c 315 560 (681) + AluJo SINE/Alu 1 242 (70) 2**

**22 0.0 0.0 0.0 AluYa5_10_33c 911 932 (309) + AT_rich Low_complexity 1 22 (0) 3**

**__________________________________________________________________________________**

**6. AluYa5_10_48c**

**Ortholog annotation C_INTER_RMD_M_DISRUPTED Length 123 nscore 0.00 NPOSITIONS NA**

**Repeat length (main genome) 309**

**Blast2 Results -**

**OFC OLC RFC RLC**

**1 123 1 123**

**Ortholog Repeat Masker annotation**

**SW perc perc perc query position in query matching repeat position in repeat**

**score div. del. ins. sequence begin end (left) repeat class/family begin end (left) ID**

**1065 7.3 0.0 0.0 AluYa5_10_48c 1 123 (0) + AluY SINE/Alu 1 123 (188) 1**

**__________________________________________________________________________________**

**7. AluYa5_10_50c**

**Ortholog annotation INDEL_CAN Length 1928 nscore 0.00 NPOSITIONS NA**

**Repeat length (main genome) 298**

**Blast2 Results -**

**OFC OLC RFC RLC**

**1 298 1 297**

**339 448 153 259**

**Ortholog Repeat Masker annotation**

**SW perc perc perc query position in query matching repeat position in repeat**

**score div. del. ins. sequence begin end (left) repeat class/family begin end (left) ID**

**2196 12.0 0.6 0.6 AluYa5_10_50c 1 311 (1617) + AluSx SINE/Alu 2 312 (0) 1**

**1056 14.8 1.1 1.7 AluYa5_10_50c 312 490 (1438) + AluSg/x SINE/Alu 128 305 (7) 2**

**36 0.0 0.0 0.0 AluYa5_10_50c 491 526 (1402) + AT_rich Low_complexity 1 36 (0) 3**

**690 26.3 26.3 2.5 AluYa5_10_50c 547 1098 (830) + HAL1 LINE/L1 1346 2029 (478) 4**

**389 29.9 0.8 2.5 AluYa5_10_50c 1523 1642 (286) + MIR SINE/MIR 110 227 (35) 5**

**__________________________________________________________________________________**

**8. AluYa5_10_54c**

**Ortholog annotation INDEL_CAN Length 1928 nscore 0.00 NPOSITIONS NA**

**Repeat length (main genome) 298**

**Blast2 Results -**

**OFC OLC RFC RLC**

**1 298 1 297**

**339 448 153 259**

**Ortholog Repeat Masker annotation**

**SW perc perc perc query position in query matching repeat position in repeat**

**score div. del. ins. sequence begin end (left) repeat class/family begin end (left) ID**

**2196 12.0 0.6 0.6 AluYa5_10_54c 1 311 (1617) + AluSx SINE/Alu 2 312 (0) 1**

**1056 14.8 1.1 1.7 AluYa5_10_54c 312 490 (1438) + AluSg/x SINE/Alu 128 305 (7) 2**

**36 0.0 0.0 0.0 AluYa5_10_54c 491 526 (1402) + AT_rich Low_complexity 1 36 (0) 3**

**690 26.3 26.3 2.5 AluYa5_10_54c 547 1098 (830) + HAL1 LINE/L1 1346 2029 (478) 4**

**389 29.9 0.8 2.5 AluYa5_10_54c 1523 1642 (286) + MIR SINE/MIR 110 227 (35) 5**

**__________________________________________________________________________________**

**9. AluYa5_10_58**

**Ortholog annotation C_INTER_RMD_M_DISRUPTED Length 133 nscore 0.00 NPOSITIONS NA**

**Repeat length (main genome) 307**

**Blast2 Results -**

**OFC OLC RFC RLC**

**1 123 1 123**

**20 53 154 187**

**Ortholog Repeat Masker annotation**

**SW perc perc perc query position in query matching repeat position in repeat**

**score div. del. ins. sequence begin end (left) repeat class/family begin end (left) ID**

**1048 6.9 0.0 2.3 AluYa5_10_58 1 133 (0) + AluY SINE/Alu 1 130 (181) 1**

**__________________________________________________________________________________**

**10. AluYa5_10_95c**

**Ortholog annotation INDEL_PTS Length 3320 nscore 0.30 NPOSITIONS 3311 3320 ;**

**Repeat length (main genome) 305**

**Blast2 Results -**

**OFC OLC RFC RLC**

**12 299 12 298**

**936 964 238 266**

**1009 1296 1 285**

**Ortholog Repeat Masker annotation**

**SW perc perc perc query position in query matching repeat position in repeat**

**score div. del. ins. sequence begin end (left) repeat class/family begin end (left) ID**

**2253 10.0 0.0 0.0 AluYa5_10_95c 1 299 (3021) + AluSx SINE/Alu 1 299 (13) 1**

**2813 16.0 3.6 1.2 AluYa5_10_95c 346 859 (2461) C LTR26B LTR/ERV1 (0) 531 16 2**

**814 15.2 0.0 0.0 AluYa5_10_95c 863 987 (2333) + FRAM SINE/Alu 30 154 (22) 3**

**1913 13.1 0.0 0.7 AluYa5_10_95c 1009 1320 (2000) + AluSx SINE/Alu 1 312 (0) 4**

**945 24.4 3.9 6.7 AluYa5_10_95c 1337 1578 (1742) C L1M5 LINE/L1 (3182) 2964 2728 5**

**1384 19.2 8.4 0.3 AluYa5_10_95c 1579 1865 (1455) C AluJb SINE/Alu (2) 310 1 6**

**945 24.4 3.9 6.7 AluYa5_10_95c 1866 1952 (1368) C L1M5 LINE/L1 (3419) 2727 2644 5**

**235 19.1 0.0 0.0 AluYa5_10_95c 2075 2116 (1204) C MLT1J LTR/MaLR (350) 162 121 7**

**2992 17.3 9.1 5.5 AluYa5_10_95c 2217 2909 (411) + LTR8A LTR/ERV1 1 718 (9) 8**

**372 24.6 11.4 0.8 AluYa5_10_95c 2936 3058 (262) + L2b LINE/L2 3029 3164 (211) 9**

**1256 9.6 2.1 0.0 AluYa5_10_95c 3062 3310 (10) C AluSq SINE/Alu (0) 313 121 10**

**__________________________________________________________________________________**

**11. AluYa5_10_98c**

**Ortholog annotation INDEL_PTS Length 2386 nscore 0.42 NPOSITIONS 2377 2386 ;**

**Repeat length (main genome) 309**

**Blast2 Results -**

**OFC OLC RFC RLC**

**91 142 11 62**

**238 525 12 298**

**1245 1275 22 52**

**1328 1357 102 131**

**1689 1733 12 56**

**1698 1730 155 187**

**1782 1969 103 288**

**Ortholog Repeat Masker annotation**

**SW perc perc perc query position in query matching repeat position in repeat**

**score div. del. ins. sequence begin end (left) repeat class/family begin end (left) ID**

**796 18.2 0.0 0.8 AluYa5_10_98c 81 213 (2173) + FLAM_C SINE/Alu 1 132 (1) 1**

**2247 13.2 0.0 0.0 AluYa5_10_98c 227 537 (1849) + AluSx SINE/Alu 1 311 (1) 2**

**391 20.6 5.2 0.0 AluYa5_10_98c 548 644 (1742) + L1MEe LINE/L1 935 1036 (5083) 3**

**2152 7.5 4.6 0.3 AluYa5_10_98c 659 941 (1445) C AluSx SINE/Alu (17) 295 1 4**

**415 22.1 10.6 4.2 AluYa5_10_98c 944 1085 (1301) + L1MEe LINE/L1 1856 2006 (4143) 3**

**1832 14.5 3.2 0.4 AluYa5_10_98c 1227 1503 (883) + AluJb SINE/Alu 2 286 (26) 5**

**198 10.3 0.0 0.0 AluYa5_10_98c 1633 1661 (725) + (T)n Simple_repeat 1 29 (0) 6**

**1927 16.7 0.0 0.9 AluYa5_10_98c 1678 1992 (394) + AluJb SINE/Alu 1 312 (0) 7**

**25 0.0 0.0 0.0 AluYa5_10_98c 2095 2119 (267) + AT_rich Low_complexity 1 25 (0) 8**

**__________________________________________________________________________________**

**12. AluYa5_11_127c**

**Ortholog annotation C_INTER_RMD_M_DISRUPTED Length 127 nscore 0.00 NPOSITIONS NA**

**Repeat length (main genome) 308**

**Blast2 Results -**

**OFC OLC RFC RLC**

**1 80 1 80**

**Ortholog Repeat Masker annotation**

**SW perc perc perc query position in query matching repeat position in repeat**

**score div. del. ins. sequence begin end (left) repeat class/family begin end (left) ID**

**983 9.4 3.1 0.0 AluYa5_11_127c 1 127 (0) + AluSg SINE/Alu 1 131 (179) 1**

**__________________________________________________________________________________**

**13. AluYa5_11_12c**

**Ortholog annotation INDEL_CAN Length 2297 nscore 0.00 NPOSITIONS NA**

**Repeat length (main genome) 331**

**Blast2 Results -**

**OFC OLC RFC RLC**

**1 296 1 298**

**537 827 10 298**

**Ortholog Repeat Masker annotation**

**SW perc perc perc query position in query matching repeat position in repeat**

**score div. del. ins. sequence begin end (left) repeat class/family begin end (left) ID**

**2126 12.0 0.3 0.7 AluYa5_11_12c 1 303 (1994) + AluSg SINE/Alu 2 303 (7) 1**

**2443 6.9 0.0 1.6 AluYa5_11_12c 527 835 (1462) + AluSg SINE/Alu 1 304 (6) 2**

**382 28.2 7.8 2.4 AluYa5_11_12c 1199 1287 (1010) + MIRc SINE/MIR 74 168 (100) 3**

**1639 14.8 5.3 3.0 AluYa5_11_12c 1288 1463 (834) C AluSx SINE/Alu (5) 307 127 4**

**189 0.0 0.0 0.0 AluYa5_11_12c 1464 1484 (813) + (TG)n Simple_repeat 2 22 (0) 5**

**1639 14.8 5.3 3.0 AluYa5_11_12c 1485 1607 (690) C AluSx SINE/Alu (186) 126 1 4**

**382 28.2 7.8 2.4 AluYa5_11_12c 1608 1684 (613) + MIRc SINE/MIR 169 249 (19) 3**

**22 3.5 0.0 0.0 AluYa5_11_12c 2269 2297 (0) + AT_rich Low_complexity 1 29 (0) 6**

**__________________________________________________________________________________**

**14. AluYa5_11_135c**

**Ortholog annotation INDEL_CAN Length 54 nscore 0.00 NPOSITIONS NA**

**Repeat length (main genome) 305**

**Blast2 Results -**

**OFC OLC RFC RLC**

**no hits found**

**Ortholog Repeat Masker annotation**

**There were no repetitive sequences detected in /home/vipin/WHOLE_GENOME_CG/AluYa5_CHR/Chimp/CONFIRMATION/AluYa5_INDEL_SEQUENCES/AluYa5_11_135c**

**__________________________________________________________________________________**

**15. AluYa5_11_152c**

**Ortholog annotation INDEL_PTS Length 189 nscore 0.00 NPOSITIONS NA**

**Repeat length (main genome) 299**

**Blast2 Results -**

**OFC OLC RFC RLC**

**no hits found**

**Ortholog Repeat Masker annotation**

**SW perc perc perc query position in query matching repeat position in repeat**

**score div. del. ins. sequence begin end (left) repeat class/family begin end (left) ID**

**1395 4.9 1.1 0.0 AluYa5_11_152c 2 186 (3) C L1P3 LINE/L1 (709) 5463 5277 1**

**__________________________________________________________________________________**

**16. AluYa5_11_160c**

**Ortholog annotation C_INTER_RMD_M_DISRUPTED Length 190 nscore 0.00 NPOSITIONS NA**

**Repeat length (main genome) 190**

**Blast2 Results -**

**OFC OLC RFC RLC**

**1 190 1 190**

**Ortholog Repeat Masker annotation**

**SW perc perc perc query position in query matching repeat position in repeat**

**score div. del. ins. sequence begin end (left) repeat class/family begin end (left) ID**

**1531 5.3 0.5 1.1 AluYa5_11_160c 1 190 (0) + AluY SINE/Alu 106 294 (17) 1**

**__________________________________________________________________________________**

**17. AluYa5_11_163c**

**Ortholog annotation C_INTER_RMD_M_DISRUPTED Length 120 nscore 0.00 NPOSITIONS NA**

**Repeat length (main genome) 298**

**Blast2 Results -**

**OFC OLC RFC RLC**

**1 79 1 79**

**88 120 97 129**

**Ortholog Repeat Masker annotation**

**SW perc perc perc query position in query matching repeat position in repeat**

**score div. del. ins. sequence begin end (left) repeat class/family begin end (left) ID**

**928 7.5 7.5 0.0 AluYa5_11_163c 1 120 (0) + AluYg SINE/Alu 3 131 (180) 1**

**__________________________________________________________________________________**

**18. AluYa5_11_165**

**Ortholog annotation INDEL_CAN Length 978 nscore 1.02 NPOSITIONS 195 204 ;**

**Repeat length (main genome) 280**

**Blast2 Results -**

**OFC OLC RFC RLC**

**706 978 9 279**

**Ortholog Repeat Masker annotation**

**SW perc perc perc query position in query matching repeat position in repeat**

**score div. del. ins. sequence begin end (left) repeat class/family begin end (left) ID**

**2361 8.5 0.3 1.3 AluYa5_11_165 668 978 (0) + AluSg SINE/Alu 1 308 (2) 1**

**__________________________________________________________________________________**

**19. AluYa5_11_22**

**Ortholog annotation INDEL_PTS Length 1599 nscore 0.00 NPOSITIONS NA**

**Repeat length (main genome) 309**

**Blast2 Results -**

**OFC OLC RFC RLC**

**12 125 12 123**

**159 297 162 301**

**Ortholog Repeat Masker annotation**

**SW perc perc perc query position in query matching repeat position in repeat**

**score div. del. ins. sequence begin end (left) repeat class/family begin end (left) ID**

**2231 11.3 0.6 1.0 AluYa5_11_22 1 314 (1285) + AluSq SINE/Alu 1 313 (0) 1**

**1273 11.4 0.0 1.7 AluYa5_11_22 1393 1571 (28) C AluSg/x SINE/Alu (10) 302 127 2**

**__________________________________________________________________________________**

**20. AluYa5_11_6**

**Ortholog annotation C_INTER_RMD Length 256 nscore 0.00 NPOSITIONS NA**

**Repeat length (main genome) 300**

**Blast2 Results -**

**OFC OLC RFC RLC**

**143 246 186 289**

**Ortholog Repeat Masker annotation**

**SW perc perc perc query position in query matching repeat position in repeat**

**score div. del. ins. sequence begin end (left) repeat class/family begin end (left) ID**

**1056 8.4 0.0 0.0 AluYa5_11_6 1 142 (114) + L1P1 LINE/L1 3608 3749 (2397) 1**

**900 8.1 0.0 0.0 AluYa5_11_6 143 253 (3) + AluY SINE/Alu 185 295 (16) 2**

**__________________________________________________________________________________**

**21. AluYa5_11_83**

**Ortholog annotation INDEL_CAN Length 1069 nscore 0.00 NPOSITIONS NA**

**Repeat length (main genome) 302**

**Blast2 Results -**

**OFC OLC RFC RLC**

**1 52 1 52**

**138 202 121 185**

**Ortholog Repeat Masker annotation**

**SW perc perc perc query position in query matching repeat position in repeat**

**score div. del. ins. sequence begin end (left) repeat class/family begin end (left) ID**

**1855 12.6 0.7 5.3 AluYa5_11_83 1 301 (768) + AluJb SINE/Alu 1 287 (25) 1**

**22 0.0 0.0 0.0 AluYa5_11_83 302 323 (746) + AT_rich Low_complexity 1 22 (0) 2**

**__________________________________________________________________________________**

**22. AluYa5_12_124**

**Ortholog annotation C_INTER_RMD_M_DISRUPTED Length 302 nscore 0.00 NPOSITIONS NA**

**Repeat length (main genome) 314**

**Blast2 Results -**

**OFC OLC RFC RLC**

**8 302 19 313**

**Ortholog Repeat Masker annotation**

**SW perc perc perc query position in query matching repeat position in repeat**

**score div. del. ins. sequence begin end (left) repeat class/family begin end (left) ID**

**2177 6.8 0.0 0.8 AluYa5_12_124 8 302 (0) + AluY SINE/Alu 20 311 (0) 1**

**__________________________________________________________________________________**

**23. AluYa5_12_138c**

**Ortholog annotation C_INTER_RMD_M_DISRUPTED Length 121 nscore 0.00 NPOSITIONS NA**

**Repeat length (main genome) 307**

**Blast2 Results -**

**OFC OLC RFC RLC**

**1 121 1 121**

**Ortholog Repeat Masker annotation**

**SW perc perc perc query position in query matching repeat position in repeat**

**score div. del. ins. sequence begin end (left) repeat class/family begin end (left) ID**

**1065 5.8 0.0 0.0 AluYa5_12_138c 1 121 (0) + AluY SINE/Alu 3 123 (188) 1**

**__________________________________________________________________________________**

**24. AluYa5_12_139c**

**Ortholog annotation INDEL_PTS Length 366 nscore 0.00 NPOSITIONS NA**

**Repeat length (main genome) 308**

**Blast2 Results -**

**OFC OLC RFC RLC**

**52 359 1 308**

**Ortholog Repeat Masker annotation**

**SW perc perc perc query position in query matching repeat position in repeat**

**score div. del. ins. sequence begin end (left) repeat class/family begin end (left) ID**

**2873 1.3 0.0 0.0 AluYa5_12_139c 52 362 (4) + AluY SINE/Alu 1 311 (0) 1**

**__________________________________________________________________________________**

**25. AluYa5_12_163c**

**Ortholog annotation INDEL_CAN Length 284 nscore 0.00 NPOSITIONS NA**

**Repeat length (main genome) 295**

**Blast2 Results -**

**OFC OLC RFC RLC**

**1 278 1 278**

**Ortholog Repeat Masker annotation**

**SW perc perc perc query position in query matching repeat position in repeat**

**score div. del. ins. sequence begin end (left) repeat class/family begin end (left) ID**

**2257 6.0 5.3 0.3 AluYa5_12_163c 1 284 (0) + AluY SINE/Alu 2 299 (12) 1**

**__________________________________________________________________________________**

**26. AluYa5_12_168c**

**Ortholog annotation INDEL_CAN Length 280 nscore 0.00 NPOSITIONS NA**

**Repeat length (main genome) 269**

**Blast2 Results -**

**OFC OLC RFC RLC**

**1 269 1 269**

**Ortholog Repeat Masker annotation**

**SW perc perc perc query position in query matching repeat position in repeat**

**score div. del. ins. sequence begin end (left) repeat class/family begin end (left) ID**

**2049 8.7 1.1 1.4 AluYa5_12_168c 1 280 (0) + AluYa5 SINE/Alu 27 305 (5) 1**

**__________________________________________________________________________________**

**27. AluYa5_12_179**

**Ortholog annotation INDEL_CAN Length 295 nscore 0.00 NPOSITIONS NA**

**Repeat length (main genome) 303**

**Blast2 Results -**

**OFC OLC RFC RLC**

**1 295 1 295**

**Ortholog Repeat Masker annotation**

**SW perc perc perc query position in query matching repeat position in repeat**

**score div. del. ins. sequence begin end (left) repeat class/family begin end (left) ID**

**2259 9.6 0.3 1.0 AluYa5_12_179 1 295 (0) + AluSc SINE/Alu 1 293 (16) 1**

**__________________________________________________________________________________**

**28. AluYa5_12_35c**

**Ortholog annotation INDEL_CAN Length 297 nscore 0.00 NPOSITIONS NA**

**Repeat length (main genome) 309**

**Blast2 Results -**

**OFC OLC RFC RLC**

**no hits found**

**Ortholog Repeat Masker annotation**

**SW perc perc perc query position in query matching repeat position in repeat**

**score div. del. ins. sequence begin end (left) repeat class/family begin end (left) ID**

**548 12.8 0.5 8.2 AluYa5_12_35c 19 213 (84) + GA-rich Low_complexity 1 180 (0) 1**

**__________________________________________________________________________________**

**29. AluYa5_12_39c**

**Ortholog annotation INDEL_CAN Length 102 nscore 0.00 NPOSITIONS NA**

**Repeat length (main genome) 310**

**Blast2 Results -**

**OFC OLC RFC RLC**

**no hits found**

**Ortholog Repeat Masker annotation**

**SW perc perc perc query position in query matching repeat position in repeat**

**score div. del. ins. sequence begin end (left) repeat class/family begin end (left) ID**

**707 6.0 5.0 0.0 AluYa5_12_39c 2 101 (1) C LTR12C LTR/ERV1 (213) 1365 1261 1**

**__________________________________________________________________________________**

**30. AluYa5_12_40**

**Ortholog annotation C_INTER_RMD_M_DISRUPTED Length 258 nscore 0.00 NPOSITIONS NA**

**Repeat length (main genome) 284**

**Blast2 Results -**

**OFC OLC RFC RLC**

**1 250 1 251**

**Ortholog Repeat Masker annotation**

**SW perc perc perc query position in query matching repeat position in repeat**

**score div. del. ins. sequence begin end (left) repeat class/family begin end (left) ID**

**1988 7.6 0.4 0.4 AluYa5_12_40 1 250 (8) + AluY SINE/Alu 35 284 (27) 1**

**__________________________________________________________________________________**

**31. AluYa5_12_71**

**Ortholog annotation INDEL_CAN Length 934 nscore 13.28 NPOSITIONS 1 124 ;**

**Repeat length (main genome) 307**

**Blast2 Results -**

**OFC OLC RFC RLC**

**178 348 121 292**

**647 700 11 64**

**843 934 204 295**

**Ortholog Repeat Masker annotation**

**SW perc perc perc query position in query matching repeat position in repeat**

**score div. del. ins. sequence begin end (left) repeat class/family begin end (left) ID**

**1712 10.3 0.5 0.0 AluYa5_12_71 125 348 (586) + AluSc SINE/Alu 66 290 (19) 1**

**843 10.8 7.5 0.0 AluYa5_12_71 349 468 (466) C AluSq/x SINE/Alu (182) 130 2 2**

**823 11.1 4.0 0.0 AluYa5_12_71 510 635 (299) C MER41B LTR/ERV1 (0) 634 504 3**

**2004 15.2 0.0 0.7 AluYa5_12_71 637 934 (0) + AluJb SINE/Alu 1 296 (16) 4**

**__________________________________________________________________________________**

**32. AluYa5_13_11**

**Ortholog annotation INDEL_CAN Length 265 nscore 0.00 NPOSITIONS NA**

**Repeat length (main genome) 250**

**Blast2 Results -**

**OFC OLC RFC RLC**

**1 245 1 245**

**Ortholog Repeat Masker annotation**

**SW perc perc perc query position in query matching repeat position in repeat**

**score div. del. ins. sequence begin end (left) repeat class/family begin end (left) ID**

**1701 6.2 23.4 0.0 AluYa5_13_11 1 244 (21) + AluY SINE/Alu 1 301 (0) 1**

**__________________________________________________________________________________**

**33. AluYa5_13_131c**

**Ortholog annotation INDEL_PTS Length 136 nscore 0.00 NPOSITIONS NA**

**Repeat length (main genome) 299**

**Blast2 Results -**

**OFC OLC RFC RLC**

**no hits found**

**Ortholog Repeat Masker annotation**

**There were no repetitive sequences detected in /home/vipin/WHOLE_GENOME_CG/AluYa5_CHR/Chimp/CONFIRMATION/AluYa5_INDEL_SEQUENCES/AluYa5_13_131c**

**__________________________________________________________________________________**

**34. AluYa5_13_133c**

**Ortholog annotation INDEL_CAN Length 902 nscore 0.00 NPOSITIONS NA**

**Repeat length (main genome) 308**

**Blast2 Results -**

**OFC OLC RFC RLC**

**no hits found**

**Ortholog Repeat Masker annotation**

**SW perc perc perc query position in query matching repeat position in repeat**

**score div. del. ins. sequence begin end (left) repeat class/family begin end (left) ID**

**5079 16.8 1.2 1.6 AluYa5_13_133c 1 902 (0) + L1MA9 LINE/L1 5366 6264 (48) 1**

**__________________________________________________________________________________**

**35. AluYa5_13_160**

**Ortholog annotation C_INTER_RMD_M_DISRUPTED Length 379 nscore 0.00 NPOSITIONS NA**

**Repeat length (main genome) 286**

**Blast2 Results -**

**OFC OLC RFC RLC**

**1 287 1 286**

**Ortholog Repeat Masker annotation**

**SW perc perc perc query position in query matching repeat position in repeat**

**score div. del. ins. sequence begin end (left) repeat class/family begin end (left) ID**

**2427 6.8 0.3 0.7 AluYa5_13_160 1 297 (82) + AluY SINE/Alu 13 308 (3) 1**

**__________________________________________________________________________________**

**36. AluYa5_13_6c**

**Ortholog annotation INDEL_CAN Length 162 nscore 0.00 NPOSITIONS NA**

**Repeat length (main genome) 175**

**Blast2 Results -**

**OFC OLC RFC RLC**

**1 162 1 162**

**Ortholog Repeat Masker annotation**

**SW perc perc perc query position in query matching repeat position in repeat**

**score div. del. ins. sequence begin end (left) repeat class/family begin end (left) ID**

**1287 8.0 0.0 0.0 AluYa5_13_6c 1 162 (0) + AluYa5 SINE/Alu 133 294 (16) 1**

**__________________________________________________________________________________**

**37. AluYa5_13_94c**

**Ortholog annotation INDEL_PTS Length 1947 nscore 8.27 NPOSITIONS 1787 1947 ;**

**Repeat length (main genome) 289**

**Blast2 Results -**

**OFC OLC RFC RLC**

**Ortholog Repeat Masker annotation**

**SW perc perc perc query position in query matching repeat position in repeat**

**score div. del. ins. sequence begin end (left) repeat class/family begin end (left) ID**

**207 19.4 6.1 1.0 AluYa5_13_94c 1360 1458 (489) + (TTATA)n Simple_repeat 4 107 (0) 1**

**189 28.0 15.8 4.1 AluYa5_13_94c 1493 1738 (209) C L2 LINE/L2 (1065) 2354 2080 2**

**__________________________________________________________________________________**

**38. AluYa5_14_101**

**Ortholog annotation C_INTER_RMD_M_DISRUPTED Length 126 nscore 0.00 NPOSITIONS NA**

**Repeat length (main genome) 310**

**Blast2 Results -**

**OFC OLC RFC RLC**

**1 126 1 126**

**21 52 156 187**

**Ortholog Repeat Masker annotation**

**SW perc perc perc query position in query matching repeat position in repeat**

**score div. del. ins. sequence begin end (left) repeat class/family begin end (left) ID**

**1006 8.0 0.0 0.8 AluYa5_14_101 1 126 (0) + AluSc SINE/Alu 2 126 (183) 1**

**__________________________________________________________________________________**

**39. AluYa5_14_108**

**Ortholog annotation INDEL_CAN Length 1468 nscore 0.00 NPOSITIONS NA**

**Repeat length (main genome) 310**

**Blast2 Results -**

**OFC OLC RFC RLC**

**3 263 3 264**

**264 290 276 302**

**Ortholog Repeat Masker annotation**

**SW perc perc perc query position in query matching repeat position in repeat**

**score div. del. ins. sequence begin end (left) repeat class/family begin end (left) ID**

**2109 10.7 3.8 0.0 AluYa5_14_108 1 290 (1178) + AluSg SINE/Alu 1 301 (9) 1**

**333 32.4 2.0 2.0 AluYa5_14_108 523 670 (798) C MIRc SINE/MIR (119) 149 2 2**

**535 29.7 6.3 2.5 AluYa5_14_108 998 1235 (233) + MIRb SINE/MIR 10 256 (12) 3**

**536 19.7 1.0 0.0 AluYa5_14_108 1241 1347 (121) C LTR16C LTR/ERVL (1) 537 399 4**

**673 18.4 0.0 0.9 AluYa5_14_108 1351 1465 (3) + AluJo/FLAM SINE/Alu 5 118 (184) 5**

**__________________________________________________________________________________**

**40. AluYa5_14_116c**

**Ortholog annotation INDEL_PTS Length 292 nscore 0.00 NPOSITIONS NA**

**Repeat length (main genome) 308**

**Blast2 Results -**

**OFC OLC RFC RLC**

**no hits found**

**Ortholog Repeat Masker annotation**

**There were no repetitive sequences detected in /home/vipin/WHOLE_GENOME_CG/AluYa5_CHR/Chimp/CONFIRMATION/AluYa5_INDEL_SEQUENCES/AluYa5_14_116c**

**__________________________________________________________________________________**

**41. AluYa5_14_2**

**Ortholog annotation INDEL_CAN Length 9818 nscore 12.68 NPOSITIONS 8338 9582 ;**

**Repeat length (main genome) 295**

**Blast2 Results -**

**OFC OLC RFC RLC**

**684 719 175 210**

**1735 1853 26 143**

**1872 1994 162 284**

**2008 2258 1 253**

**3080 3375 1 295**

**5446 5713 30 295**

**5760 6042 12 293**

**8291 8326 102 137**

**9583 9818 59 294**

**Ortholog Repeat Masker annotation**

**SW perc perc perc query position in query matching repeat position in repeat**

**score div. del. ins. sequence begin end (left) repeat class/family begin end (left) ID**

**1978 13.9 1.8 0.0 AluYa5_14_2 6 335 (9483) + L1MC LINE/L1 5330 5665 (668) 1**

**543 16.5 0.0 0.0 AluYa5_14_2 676 772 (9046) + FLAM_A SINE/Alu 33 129 (13) 2**

**361 14.5 6.6 0.0 AluYa5_14_2 878 953 (8865) + L1ME4a LINE/L1 5986 6066 (58) 3**

**25 3.1 0.0 0.0 AluYa5_14_2 1483 1514 (8304) + AT_rich Low_complexity 1 32 (0) 4**

**1934 13.3 0.0 0.0 AluYa5_14_2 1709 1994 (7824) + AluSx SINE/Alu 1 286 (26) 5**

**2115 7.1 3.7 1.0 AluYa5_14_2 2007 2304 (7514) + AluSg SINE/Alu 1 306 (4) 6**

**2308 8.6 0.3 0.0 AluYa5_14_2 3079 3382 (6436) + AluSq SINE/Alu 1 305 (8) 7**

**2094 12.2 0.0 0.0 AluYa5_14_2 3660 3955 (5863) C AluSx SINE/Alu (16) 296 1 8**

**1009 28.6 6.7 3.2 AluYa5_14_2 4527 4999 (4819) C L1M5 LINE/L1 (453) 5787 5304 9**

**1953 10.3 0.4 0.4 AluYa5_14_2 5443 5715 (4103) + AluSq SINE/Alu 28 300 (13) 10**

**2324 8.1 0.7 0.3 AluYa5_14_2 5748 6046 (3772) + AluSp SINE/Alu 1 300 (13) 11**

**1838 11.4 0.7 1.4 AluYa5_14_2 6351 6626 (3192) C AluSg1 SINE/Alu (19) 290 17 12**

**259 28.7 2.3 1.1 AluYa5_14_2 7064 7151 (2667) C L1M5 LINE/L1 (502) 5738 5650 13**

**21 0.0 0.0 0.0 AluYa5_14_2 7494 7514 (2304) + AT_rich Low_complexity 1 21 (0) 14**

**2250 11.2 0.0 0.7 AluYa5_14_2 7515 7811 (2007) C AluSx SINE/Alu (17) 295 1 15**

**444 10.6 0.0 1.5 AluYa5_14_2 8271 8337 (1481) + AluJb SINE/Alu 85 150 (162) 16**

**2051 2.5 0.0 0.0 AluYa5_14_2 9583 9818 (0) + AluYa5 SINE/Alu 60 295 (15) 17**

**__________________________________________________________________________________**

**42. AluYa5_14_20**

**Ortholog annotation INDEL_CAN Length 505 nscore 0.00 NPOSITIONS NA**

**Repeat length (main genome) 149**

**Blast2 Results -**

**OFC OLC RFC RLC**

**358 505 1 148**

**Ortholog Repeat Masker annotation**

**SW perc perc perc query position in query matching repeat position in repeat**

**score div. del. ins. sequence begin end (left) repeat class/family begin end (left) ID**

**1193 8.1 0.0 0.0 AluYa5_14_20 358 505 (0) + AluYa5 SINE/Alu 154 301 (9) 1**

**__________________________________________________________________________________**

**43. AluYa5_14_3**

**Ortholog annotation INDEL_CAN Length 750 nscore 0.00 NPOSITIONS NA**

**Repeat length (main genome) 300**

**Blast2 Results -**

**OFC OLC RFC RLC**

**98 155 242 299**

**328 399 12 85**

**454 749 2 298**

**Ortholog Repeat Masker annotation**

**SW perc perc perc query position in query matching repeat position in repeat**

**score div. del. ins. sequence begin end (left) repeat class/family begin end (left) ID**

**955 18.2 0.0 0.0 AluYa5_14_3 3 161 (589) + AluJb SINE/Alu 147 305 (7) 1**

**908 11.9 3.7 0.0 AluYa5_14_3 317 450 (300) + AluSg1 SINE/Alu 1 139 (170) 2**

**2584 5.4 0.0 0.0 AluYa5_14_3 453 749 (1) + AluY SINE/Alu 1 297 (14) 3**

**__________________________________________________________________________________**

**44. AluYa5_14_35c**

**Ortholog annotation INDEL_CAN Length 308 nscore 0.00 NPOSITIONS NA**

**Repeat length (main genome) 301**

**Blast2 Results -**

**OFC OLC RFC RLC**

**2 294 1 294**

**Ortholog Repeat Masker annotation**

**SW perc perc perc query position in query matching repeat position in repeat**

**score div. del. ins. sequence begin end (left) repeat class/family begin end (left) ID**

**2668 4.6 0.3 0.0 AluYa5_14_35c 2 308 (0) + AluY SINE/Alu 1 308 (3) 1**

**__________________________________________________________________________________**

**45. AluYa5_14_69c**

**Ortholog annotation INDEL_PTS Length 66 nscore 0.00 NPOSITIONS NA**

**Repeat length (main genome) 306**

**Blast2 Results -**

**OFC OLC RFC RLC**

**no hits found**

**Ortholog Repeat Masker annotation**

**There were no repetitive sequences detected in /home/vipin/WHOLE_GENOME_CG/AluYa5_CHR/Chimp/CONFIRMATION/AluYa5_INDEL_SEQUENCES/AluYa5_14_69c**

**__________________________________________________________________________________**

**46. AluYa5_14_79c**

**Ortholog annotation INDEL_CAN Length 64 nscore 0.00 NPOSITIONS NA**

**Repeat length (main genome) 29**

**Blast2 Results -**

**OFC OLC RFC RLC**

**39 64 1 26**

**Ortholog Repeat Masker annotation**

**SW perc perc perc query position in query matching repeat position in repeat**

**score div. del. ins. sequence begin end (left) repeat class/family begin end (left) ID**

**340 14.1 15.6 0.0 AluYa5_14_79c 1 64 (0) + Alu SINE/Alu 212 285 (15) 1**

**__________________________________________________________________________________**

**47. AluYa5_14_85**

**Ortholog annotation INDEL_CAN Length 1217 nscore 0.00 NPOSITIONS NA**

**Repeat length (main genome) 312**

**Blast2 Results -**

**OFC OLC RFC RLC**

**923 1214 3 295**

**1055 1118 1 64**

**Ortholog Repeat Masker annotation**

**SW perc perc perc query position in query matching repeat position in repeat**

**score div. del. ins. sequence begin end (left) repeat class/family begin end (left) ID**

**191 34.8 13.6 4.2 AluYa5_14_85 475 636 (581) C L2 LINE/L2 (469) 2950 2773 1**

**432 0.0 0.0 0.0 AluYa5_14_85 637 684 (533) + (TG)n Simple_repeat 1 48 (0) 2**

**191 34.8 13.6 4.2 AluYa5_14_85 685 875 (342) C L2 LINE/L2 (647) 2772 2564 1**

**2334 9.5 0.3 0.0 AluYa5_14_85 921 1214 (3) + AluSp SINE/Alu 1 295 (18) 3**

**__________________________________________________________________________________**

**48. AluYa5_14_93**

**Ortholog annotation INDEL_CAN Length 297 nscore 0.00 NPOSITIONS NA**

**Repeat length (main genome) 304**

**Blast2 Results -**

**OFC OLC RFC RLC**

**1 297 1 297**

**Ortholog Repeat Masker annotation**

**SW perc perc perc query position in query matching repeat position in repeat**

**score div. del. ins. sequence begin end (left) repeat class/family begin end (left) ID**

**2290 10.5 0.0 0.3 AluYa5_14_93 1 297 (0) + AluSc SINE/Alu 1 296 (13) 1**

**__________________________________________________________________________________**

**49. AluYa5_14_98c**

**Ortholog annotation INDEL_CAN Length 2613 nscore 0.00 NPOSITIONS NA**

**Repeat length (main genome) 233**

**Blast2 Results -**

**OFC OLC RFC RLC**

**700 754 11 65**

**1510 1727 12 230**

**Ortholog Repeat Masker annotation**

**SW perc perc perc query position in query matching repeat position in repeat**

**score div. del. ins. sequence begin end (left) repeat class/family begin end (left) ID**

**879 23.9 15.1 3.9 AluYa5_14_98c 630 690 (1923) C MER57E1 LTR/ERV1 (0) 372 304 1**

**686 14.4 0.6 16.5 AluYa5_14_98c 691 848 (1765) + FLAM_C SINE/Alu 1 133 (0) 2**

**879 23.9 15.1 3.9 AluYa5_14_98c 849 1117 (1496) C MER57E1 LTR/ERV1 (69) 303 5 1**

**658 31.5 5.2 2.1 AluYa5_14_98c 1184 1498 (1115) + L1M5 LINE/L1 4067 4393 (1753) 3**

**2077 13.7 0.0 0.0 AluYa5_14_98c 1499 1797 (816) + AluSg SINE/Alu 1 299 (11) 4**

**658 31.5 5.2 2.1 AluYa5_14_98c 1798 1808 (805) + L1M5 LINE/L1 4394 4405 (1741) 3**

**1375 22.6 1.0 2.2 AluYa5_14_98c 1809 2120 (493) + AluJo SINE/Alu 3 310 (2) 5**

**658 31.5 5.2 2.1 AluYa5_14_98c 2121 2217 (396) + L1M5 LINE/L1 4406 4504 (1642) 3**

**288 14.3 5.2 9.1 AluYa5_14_98c 2382 2458 (155) + L1M5 LINE/L1 5642 5715 (458) 6**

**758 17.2 2.6 0.7 AluYa5_14_98c 2462 2613 (0) C L1M4 LINE/L1 (1571) 4575 4421 7**

**__________________________________________________________________________________**

**50. AluYa5_15_100c**

**Ortholog annotation INDEL_CAN Length 343 nscore 0.00 NPOSITIONS NA**

**Repeat length (main genome) 304**

**Blast2 Results -**

**OFC OLC RFC RLC**

**no hits found**

**Ortholog Repeat Masker annotation**

**SW perc perc perc query position in query matching repeat position in repeat**

**score div. del. ins. sequence begin end (left) repeat class/family begin end (left) ID**

**651 23.0 0.0 9.4 AluYa5_15_100c 151 342 (1) + MLT1E2 LTR/MaLR 6 179 (448) 1**

**__________________________________________________________________________________**

**51. AluYa5_15_40c**

**Ortholog annotation INDEL_CAN Length 623 nscore 0.00 NPOSITIONS NA**

**Repeat length (main genome) 295**

**Blast2 Results -**

**OFC OLC RFC RLC**

**1 303 1 294**

**Ortholog Repeat Masker annotation**

**SW perc perc perc query position in query matching repeat position in repeat**

**score div. del. ins. sequence begin end (left) repeat class/family begin end (left) ID**

**2381 8.1 0.0 3.5 AluYa5_15_40c 1 319 (304) + AluSg SINE/Alu 2 309 (1) 1**

**1690 11.0 4.0 0.3 AluYa5_15_40c 322 623 (0) + L1M4 LINE/L1 3117 3429 (2717) 2**

**__________________________________________________________________________________**

**52. AluYa5_15_43c**

**Ortholog annotation INDEL_CAN Length 242 nscore 0.00 NPOSITIONS NA**

**Repeat length (main genome) 254**

**Blast2 Results -**

**OFC OLC RFC RLC**

**15 239 15 239**

**Ortholog Repeat Masker annotation**

**SW perc perc perc query position in query matching repeat position in repeat**

**score div. del. ins. sequence begin end (left) repeat class/family begin end (left) ID**

**443 8.3 1.7 0.0 AluYa5_15_43c 1 60 (182) + AluY SINE/Alu 2 62 (249) 1**

**1534 5.0 0.6 0.0 AluYa5_15_43c 61 242 (0) + AluY SINE/Alu 116 298 (13) 1**

**__________________________________________________________________________________**

**53. AluYa5_15_44**

**Ortholog annotation INDEL_CAN Length 72 nscore 0.00 NPOSITIONS NA**

**Repeat length (main genome) 310**

**Blast2 Results -**

**OFC OLC RFC RLC**

**no hits found**

**Ortholog Repeat Masker annotation**

**There were no repetitive sequences detected in /home/vipin/WHOLE_GENOME_CG/AluYa5_CHR/Chimp/CONFIRMATION/AluYa5_INDEL_SEQUENCES/AluYa5_15_44**

**__________________________________________________________________________________**

**54. AluYa5_15_55**

**Ortholog annotation C_INTER_RMD Length 293 nscore 0.00 NPOSITIONS NA**

**Repeat length (main genome) 308**

**Blast2 Results -**

**OFC OLC RFC RLC**

**1 293 1 289**

**Ortholog Repeat Masker annotation**

**SW perc perc perc query position in query matching repeat position in repeat**

**score div. del. ins. sequence begin end (left) repeat class/family begin end (left) ID**

**2362 7.9 0.0 1.0 AluYa5_15_55 1 293 (0) + AluSx SINE/Alu 1 290 (22) 1**

**__________________________________________________________________________________**

**55. AluYa5_15_82c**

**Ortholog annotation INDEL_CAN Length 425 nscore 0.00 NPOSITIONS NA**

**Repeat length (main genome) 315**

**Blast2 Results -**

**OFC OLC RFC RLC**

**1 285 1 290**

**Ortholog Repeat Masker annotation**

**SW perc perc perc query position in query matching repeat position in repeat**

**score div. del. ins. sequence begin end (left) repeat class/family begin end (left) ID**

**2171 11.2 0.0 0.0 AluYa5_15_82c 1 285 (140) + AluY SINE/Alu 1 285 (26) 1**

**378 15.2 0.0 0.0 AluYa5_15_82c 298 356 (69) + HSMAR1 DNA/Mariner 1229 1287 (0) 2**

**__________________________________________________________________________________**

**56. AluYa5_15_98**

**Ortholog annotation INDEL_CAN Length 320 nscore 0.00 NPOSITIONS NA**

**Repeat length (main genome) 301**

**Blast2 Results -**

**OFC OLC RFC RLC**

**1 308 1 301**

**Ortholog Repeat Masker annotation**

**SW perc perc perc query position in query matching repeat position in repeat**

**score div. del. ins. sequence begin end (left) repeat class/family begin end (left) ID**

**2204 10.1 0.6 3.8 AluYa5_15_98 1 320 (0) + AluY SINE/Alu 1 310 (1) 1**

**__________________________________________________________________________________**

**57. AluYa5_15_99c**

**Ortholog annotation INDEL_CAN Length 2391 nscore 0.00 NPOSITIONS NA**

**Repeat length (main genome) 310**

**Blast2 Results -**

**OFC OLC RFC RLC**

**Ortholog Repeat Masker annotation**

**SW perc perc perc query position in query matching repeat position in repeat**

**score div. del. ins. sequence begin end (left) repeat class/family begin end (left) ID**

**3349 25.7 8.0 3.4 AluYa5_15_99c 7 1361 (1030) + L1MEf LINE/L1 653 2069 (4173) 1**

**1407 25.1 7.0 0.5 AluYa5_15_99c 1534 2042 (349) + L1MEf LINE/L1 3479 4041 (2105) 1**

**569 16.1 9.2 2.8 AluYa5_15_99c 2114 2254 (137) + L1ME2z LINE/L1 5814 5963 (481) 2**

**486 25.7 3.5 0.7 AluYa5_15_99c 2245 2385 (6) + L1MEf LINE/L1 4039 4183 (1963) 1 ***

**__________________________________________________________________________________**

**58. AluYa5_16_100**

**Ortholog annotation INDEL_PTS Length 3797 nscore 0.00 NPOSITIONS NA**

**Repeat length (main genome) 310**

**Blast2 Results -**

**OFC OLC RFC RLC**

**1 307 1 301**

**Ortholog Repeat Masker annotation**

**SW perc perc perc query position in query matching repeat position in repeat**

**score div. del. ins. sequence begin end (left) repeat class/family begin end (left) ID**

**2332 8.0 0.0 1.9 AluYa5_16_100 1 307 (3490) + AluY SINE/Alu 1 301 (10) 1**

**690 18.1 0.0 0.0 AluYa5_16_100 531 646 (3151) C FLAM_C SINE/Alu (15) 128 13 2**

**__________________________________________________________________________________**

**59. AluYa5_16_108**

**Ortholog annotation C_INTER_RMD_M_DISRUPTED Length 305 nscore 0.00 NPOSITIONS NA**

**Repeat length (main genome) 316**

**Blast2 Results -**

**OFC OLC RFC RLC**

**8 305 8 300**

**Ortholog Repeat Masker annotation**

**SW perc perc perc query position in query matching repeat position in repeat**

**score div. del. ins. sequence begin end (left) repeat class/family begin end (left) ID**

**2224 8.3 0.0 5.2 AluYa5_16_108 1 305 (0) + AluYa5 SINE/Alu 2 290 (20) 1**

**__________________________________________________________________________________**

**60. AluYa5_16_112c**

**Ortholog annotation C_INTER_RMD_M_DISRUPTED Length 214 nscore 14.02 NPOSITIONS 180 209 ;**

**Repeat length (main genome) 309**

**Blast2 Results -**

**OFC OLC RFC RLC**

**11 179 11 179**

**Ortholog Repeat Masker annotation**

**SW perc perc perc query position in query matching repeat position in repeat**

**score div. del. ins. sequence begin end (left) repeat class/family begin end (left) ID**

**1396 10.6 0.0 0.0 AluYa5_16_112c 1 179 (35) + AluY SINE/Alu 1 179 (132) 1**

**__________________________________________________________________________________**

**61. AluYa5_16_15**

**Ortholog annotation C_INTER_RMD_M_DISRUPTED Length 127 nscore 0.00 NPOSITIONS NA**

**Repeat length (main genome) 311**

**Blast2 Results -**

**OFC OLC RFC RLC**

**1 127 1 127**

**Ortholog Repeat Masker annotation**

**SW perc perc perc query position in query matching repeat position in repeat**

**score div. del. ins. sequence begin end (left) repeat class/family begin end (left) ID**

**868 11.1 1.6 0.8 AluYa5_16_15 1 127 (0) + AluSq/x SINE/Alu 1 128 (184) 1**

**__________________________________________________________________________________**

**62. AluYa5_16_17**

**Ortholog annotation C_INTER_RMD_M_DISRUPTED Length 288 nscore 0.00 NPOSITIONS NA**

**Repeat length (main genome) 307**

**Blast2 Results -**

**OFC OLC RFC RLC**

**1 288 1 288**

**Ortholog Repeat Masker annotation**

**SW perc perc perc query position in query matching repeat position in repeat**

**score div. del. ins. sequence begin end (left) repeat class/family begin end (left) ID**

**2405 7.3 0.3 0.0 AluYa5_16_17 1 288 (0) + AluSq SINE/Alu 3 291 (22) 1**

**__________________________________________________________________________________**

**63. AluYa5_16_1c**

**Ortholog annotation INDEL_CAN Length 1682 nscore 4.22 NPOSITIONS 244 314 ;**

**Repeat length (main genome) 304**

**Blast2 Results -**

**OFC OLC RFC RLC**

**316 456 146 286**

**Ortholog Repeat Masker annotation**

**SW perc perc perc query position in query matching repeat position in repeat**

**score div. del. ins. sequence begin end (left) repeat class/family begin end (left) ID**

**1204 7.0 0.0 0.0 AluYa5_16_1c 315 456 (1226) + AluY SINE/Alu 145 286 (25) 1**

**3756 12.2 3.1 3.3 AluYa5_16_1c 476 580 (1102) + Tigger1 DNA/MER2_type 869 974 (1444) 2**

**1998 12.5 0.0 3.3 AluYa5_16_1c 581 886 (796) C AluSq SINE/Alu (13) 300 5 3**

**2340 9.3 0.0 0.0 AluYa5_16_1c 896 1195 (487) C AluSg SINE/Alu (9) 301 2 4**

**3756 12.2 3.1 3.3 AluYa5_16_1c 1196 1682 (0) + Tigger1 DNA/MER2_type 985 1470 (948) 2**

**__________________________________________________________________________________**

**64. AluYa5_16_26**

**Ortholog annotation C_INTER_RMD_M_DISRUPTED Length 126 nscore 0.00 NPOSITIONS NA**

**Repeat length (main genome) 308**

**Blast2 Results -**

**OFC OLC RFC RLC**

**1 126 1 126**

**Ortholog Repeat Masker annotation**

**SW perc perc perc query position in query matching repeat position in repeat**

**score div. del. ins. sequence begin end (left) repeat class/family begin end (left) ID**

**1058 4.8 1.6 0.0 AluYa5_16_26 1 126 (0) + AluSc SINE/Alu 1 128 (181) 1**

**__________________________________________________________________________________**

**65. AluYa5_16_28c**

**Ortholog annotation C_INTER_RMD_M_DISRUPTED Length 110 nscore 0.00 NPOSITIONS NA**

**Repeat length (main genome) 308**

**Blast2 Results -**

**OFC OLC RFC RLC**

**1 107 1 107**

**Ortholog Repeat Masker annotation**

**SW perc perc perc query position in query matching repeat position in repeat**

**score div. del. ins. sequence begin end (left) repeat class/family begin end (left) ID**

**878 5.5 1.8 0.0 AluYa5_16_28c 1 110 (0) + AluSc SINE/Alu 1 112 (197) 1**

**__________________________________________________________________________________**

**66. AluYa5_16_37**

**Ortholog annotation C_INTER_RMD_M_DISRUPTED Length 124 nscore 0.00 NPOSITIONS NA**

**Repeat length (main genome) 308**

**Blast2 Results -**

**OFC OLC RFC RLC**

**1 124 1 124**

**Ortholog Repeat Masker annotation**

**SW perc perc perc query position in query matching repeat position in repeat**

**score div. del. ins. sequence begin end (left) repeat class/family begin end (left) ID**

**1047 4.0 1.6 0.0 AluYa5_16_37 1 124 (0) + AluSc SINE/Alu 1 126 (183) 1**

**__________________________________________________________________________________**

**67. AluYa5_16_38**

**Ortholog annotation C_INTER_RMD_M_DISRUPTED Length 124 nscore 0.00 NPOSITIONS NA**

**Repeat length (main genome) 308**

**Blast2 Results -**

**OFC OLC RFC RLC**

**1 124 1 124**

**Ortholog Repeat Masker annotation**

**SW perc perc perc query position in query matching repeat position in repeat**

**score div. del. ins. sequence begin end (left) repeat class/family begin end (left) ID**

**1047 4.0 1.6 0.0 AluYa5_16_38 1 124 (0) + AluSc SINE/Alu 1 126 (183) 1**

**__________________________________________________________________________________**

**68. AluYa5_16_50**

**Ortholog annotation C_INTER_RMD_M_DISRUPTED Length 84 nscore 0.00 NPOSITIONS NA**

**Repeat length (main genome) 308**

**Blast2 Results -**

**OFC OLC RFC RLC**

**1 64 1 64**

**Ortholog Repeat Masker annotation**

**SW perc perc perc query position in query matching repeat position in repeat**

**score div. del. ins. sequence begin end (left) repeat class/family begin end (left) ID**

**581 13.2 0.0 0.0 AluYa5_16_50 2 84 (0) + AluS SINE/Alu 2 84 (228) 1**

**__________________________________________________________________________________**

**69. AluYa5_16_52c**

**Ortholog annotation C_INTER_RMD_M_DISRUPTED Length 129 nscore 0.00 NPOSITIONS NA**

**Repeat length (main genome) 292**

**Blast2 Results -**

**OFC OLC RFC RLC**

**5 129 5 129**

**Ortholog Repeat Masker annotation**

**SW perc perc perc query position in query matching repeat position in repeat**

**score div. del. ins. sequence begin end (left) repeat class/family begin end (left) ID**

**915 13.9 0.0 0.0 AluYa5_16_52c 1 129 (0) + AluSq/x SINE/Alu 5 133 (179) 1**

**__________________________________________________________________________________**

**70. AluYa5_16_67**

**Ortholog annotation C_INTRA_RMD Length 139 nscore 0.00 NPOSITIONS NA**

**Repeat length (main genome) 310**

**Blast2 Results -**

**OFC OLC RFC RLC**

**1 125 1 125**

**Ortholog Repeat Masker annotation**

**SW perc perc perc query position in query matching repeat position in repeat**

**score div. del. ins. sequence begin end (left) repeat class/family begin end (left) ID**

**1084 9.0 0.0 0.0 AluYa5_16_67 1 134 (5) + AluSq/x SINE/Alu 1 134 (178) 1**

**__________________________________________________________________________________**

**71. AluYa5_16_69c**

**Ortholog annotation C_DISRUPTED_M_INTER_RMD Length 801 nscore 0.00 NPOSITIONS NA**

**Repeat length (main genome) 294**

**Blast2 Results -**

**OFC OLC RFC RLC**

**1 139 1 139**

**631 799 124 292**

**Ortholog Repeat Masker annotation**

**SW perc perc perc query position in query matching repeat position in repeat**

**score div. del. ins. sequence begin end (left) repeat class/family begin end (left) ID**

**1047 10.8 2.2 0.0 AluYa5_16_69c 1 139 (662) + AluY SINE/Alu 1 142 (168) 1**

**428 0.0 0.0 0.0 AluYa5_16_69c 140 187 (614) C L1PA2 LINE/L1 (375) 5780 5733 2**

**3253 1.6 0.0 0.0 AluYa5_16_69c 188 553 (248) + L1PA2 LINE/L1 5790 6155 (0) 2**

**243 0.0 0.0 0.0 AluYa5_16_69c 596 622 (179) + (A)n Simple_repeat 1 27 (0) 3**

**1394 7.3 1.1 0.0 AluYa5_16_69c 623 799 (2) + AluY SINE/Alu 119 297 (14) 1**

**__________________________________________________________________________________**

**72. AluYa5_16_72**

**Ortholog annotation C_INTER_RMD_M_DISRUPTED Length 135 nscore 0.00 NPOSITIONS NA**

**Repeat length (main genome) 300**

**Blast2 Results -**

**OFC OLC RFC RLC**

**1 134 1 134**

**Ortholog Repeat Masker annotation**

**SW perc perc perc query position in query matching repeat position in repeat**

**score div. del. ins. sequence begin end (left) repeat class/family begin end (left) ID**

**1005 11.3 0.0 0.0 AluYa5_16_72 1 133 (2) + AluSq/x SINE/Alu 1 133 (179) 1**

**__________________________________________________________________________________**

**73. AluYa5_16_9c**

**Ortholog annotation C_INTER_RMD_M_DISRUPTED Length 132 nscore 0.00 NPOSITIONS NA**

**Repeat length (main genome) 309**

**Blast2 Results -**

**OFC OLC RFC RLC**

**1 132 1 132**

**Ortholog Repeat Masker annotation**

**SW perc perc perc query position in query matching repeat position in repeat**

**score div. del. ins. sequence begin end (left) repeat class/family begin end (left) ID**

**1104 5.3 0.8 0.0 AluYa5_16_9c 1 132 (0) + AluSq/x SINE/Alu 1 133 (179) 1**

**__________________________________________________________________________________**

**74. AluYa5_17_100**

**Ortholog annotation INDEL_PTS Length 8810 nscore 8.62 NPOSITIONS 1 479 ; 3876 3885 ; 8541 8810 ;**

**Repeat length (main genome) 301**

**Blast2 Results -**

**OFC OLC RFC RLC**

**985 1276 12 301**

**1782 2073 10 300**

**2115 2399 12 297**

**2454 2730 12 288**

**2939 2998 240 296**

**3126 3406 12 293**

**4183 4491 1 299**

**4508 4803 1 295**

**5775 6006 7 235**

**6884 7174 12 301**

**7349 7632 16 298**

**7938 8219 9 301**

**8249 8537 12 300**

**Ortholog Repeat Masker annotation**

**SW perc perc perc query position in query matching repeat position in repeat**

**score div. del. ins. sequence begin end (left) repeat class/family begin end (left) ID**

**22 0.0 0.0 0.0 AluYa5_17_100 655 676 (8134) + AT_rich Low_complexity 1 22 (0) 1**

**364 29.3 11.2 4.1 AluYa5_17_100 694 960 (7850) + L1MD LINE/L1 4025 4310 (1836) 2**

**2507 5.0 0.0 0.7 AluYa5_17_100 977 1280 (7530) + AluY SINE/Alu 4 305 (6) 3**

**1145 17.1 0.4 3.8 AluYa5_17_100 1558 1773 (7037) + AluJo SINE/Alu 84 293 (19) 4**

**2394 10.3 0.0 0.0 AluYa5_17_100 1774 2083 (6727) + AluSx SINE/Alu 2 311 (1) 5**

**1145 17.1 0.4 3.8 AluYa5_17_100 2084 2103 (6707) + AluJo SINE/Alu 294 312 (0) 4**

**2401 9.7 0.0 0.7 AluYa5_17_100 2104 2413 (6397) + AluSg SINE/Alu 1 308 (2) 6**

**2195 10.5 0.0 0.3 AluYa5_17_100 2443 2730 (6080) + AluSg SINE/Alu 1 287 (23) 7**

**452 11.1 0.0 8.0 AluYa5_17_100 2930 3017 (5793) + Alu SINE/Alu 231 311 (0) 8**

**2222 10.3 0.0 0.3 AluYa5_17_100 3115 3406 (5404) + AluSc SINE/Alu 1 291 (18) 9**

**216 0.0 0.0 0.0 AluYa5_17_100 3527 3550 (5260) + (TA)n Simple_repeat 2 25 (0) 10**

**2244 9.9 0.0 0.3 AluYa5_17_100 3551 3843 (4967) C AluSg SINE/Alu (18) 292 1 11**

**1299 12.2 1.1 0.0 AluYa5_17_100 3886 4066 (4744) C AluSx SINE/Alu (129) 183 1 12**

**2108 10.9 0.0 3.2 AluYa5_17_100 4183 4495 (4315) + AluY SINE/Alu 1 303 (8) 13**

**2374 8.3 0.3 0.0 AluYa5_17_100 4508 4807 (4003) + AluSp SINE/Alu 1 301 (12) 14**

**2066 12.3 0.3 0.0 AluYa5_17_100 5020 5331 (3479) C AluSp SINE/Alu (0) 313 1 15**

**247 22.1 0.0 2.3 AluYa5_17_100 5701 5768 (3042) + GA-rich Low_complexity 4 71 (0) 16**

**2055 11.7 1.7 1.7 AluYa5_17_100 5769 6064 (2746) + AluSx SINE/Alu 1 296 (16) 17**

**247 22.1 0.0 2.3 AluYa5_17_100 6065 6083 (2727) + polypurine Low_complexity 72 89 (0) 18**

**2565 6.1 0.3 0.0 AluYa5_17_100 6873 7182 (1628) + AluSp SINE/Alu 1 311 (2) 19**

**2254 10.4 0.0 0.0 AluYa5_17_100 7334 7632 (1178) + AluSx SINE/Alu 1 299 (13) 20**

**182 15.0 2.5 0.0 AluYa5_17_100 7846 7885 (925) + (CA)n Simple_repeat 2 42 (0) 21**

**2304 5.8 3.7 0.0 AluYa5_17_100 7930 8224 (586) + AluY SINE/Alu 1 306 (5) 22**

**2273 9.9 0.7 0.0 AluYa5_17_100 8238 8540 (270) + AluSq SINE/Alu 1 305 (8) 23**

**__________________________________________________________________________________**

**75. AluYa5_17_104**

**Ortholog annotation INDEL_CAN Length 296 nscore 0.00 NPOSITIONS NA**

**Repeat length (main genome) 310**

**Blast2 Results -**

**OFC OLC RFC RLC**

**1 294 1 294**

**Ortholog Repeat Masker annotation**

**SW perc perc perc query position in query matching repeat position in repeat**

**score div. del. ins. sequence begin end (left) repeat class/family begin end (left) ID**

**2569 6.1 0.0 0.0 AluYa5_17_104 1 294 (2) + AluY SINE/Alu 1 294 (17) 1**

**__________________________________________________________________________________**

**76. AluYa5_17_105**

**Ortholog annotation C_INTER_RMD_M_DISRUPTED Length 120 nscore 0.00 NPOSITIONS NA**

**Repeat length (main genome) 296**

**Blast2 Results -**

**OFC OLC RFC RLC**

**1 116 1 116**

**Ortholog Repeat Masker annotation**

**SW perc perc perc query position in query matching repeat position in repeat**

**score div. del. ins. sequence begin end (left) repeat class/family begin end (left) ID**

**972 9.2 0.0 0.0 AluYa5_17_105 1 120 (0) + AluSq/x SINE/Alu 12 131 (181) 1**

**__________________________________________________________________________________**

**77. AluYa5_17_2**

**Ortholog annotation C_DISRUPTED_M_INTER_RMD Length 301 nscore 0.00 NPOSITIONS NA**

**Repeat length (main genome) 168**

**Blast2 Results -**

**OFC OLC RFC RLC**

**1 55 1 55**

**153 301 19 168**

**Ortholog Repeat Masker annotation**

**SW perc perc perc query position in query matching repeat position in repeat**

**score div. del. ins. sequence begin end (left) repeat class/family begin end (left) ID**

**2608 6.0 0.0 0.0 AluYa5_17_2 1 301 (0) + AluY SINE/Alu 2 302 (9) 1**

**__________________________________________________________________________________**

**78. AluYa5_17_38c**

**Ortholog annotation C_INTER_RMD Length 299 nscore 0.00 NPOSITIONS NA**

**Repeat length (main genome) 310**

**Blast2 Results -**

**OFC OLC RFC RLC**

**12 294 12 295**

**Ortholog Repeat Masker annotation**

**SW perc perc perc query position in query matching repeat position in repeat**

**score div. del. ins. sequence begin end (left) repeat class/family begin end (left) ID**

**2291 9.5 0.7 0.0 AluYa5_17_38c 1 294 (5) + AluSx SINE/Alu 1 296 (16) 1**

**__________________________________________________________________________________**

**79. AluYa5_17_40**

**Ortholog annotation INDEL_PTS Length 7136 nscore 0.00 NPOSITIONS NA**

**Repeat length (main genome) 309**

**Blast2 Results -**

**OFC OLC RFC RLC**

**456 738 8 291**

**785 967 128 308**

**3070 3123 11 64**

**Ortholog Repeat Masker annotation**

**SW perc perc perc query position in query matching repeat position in repeat**

**score div. del. ins. sequence begin end (left) repeat class/family begin end (left) ID**

**2552 8.2 6.9 2.6 AluYa5_17_40 2 379 (6757) C MLT2A1 LTR/ERVL (50) 394 1 1**

**470 12.9 1.4 0.0 AluYa5_17_40 380 448 (6688) C LTR8A LTR/ERV1 (345) 382 313 2**

**2147 12.4 0.7 0.0 AluYa5_17_40 449 738 (6398) + AluSx SINE/Alu 1 292 (20) 3**

**1397 9.9 22.1 0.9 AluYa5_17_40 745 970 (6166) + AluSg/x SINE/Alu 39 312 (0) 4**

**325 16.4 1.8 0.0 AluYa5_17_40 971 1025 (6111) C LTR8A LTR/ERV1 (407) 320 265 2**

**184 33.9 8.5 1.6 AluYa5_17_40 1408 1536 (5600) + L3b LINE/CR1 146 283 (199) 5**

**2301 10.8 0.0 0.7 AluYa5_17_40 1873 2178 (4958) C AluSg SINE/Alu (6) 304 1 6**

**2518 6.5 0.0 0.0 AluYa5_17_40 2186 2492 (4644) C AluY SINE/Alu (4) 307 1 7**

**1728 16.8 6.8 0.6 AluYa5_17_40 2631 2953 (4183) + MER61A LTR/ERV1 1 343 (0) 8**

**1328 16.6 12.1 1.2 AluYa5_17_40 3060 3315 (3821) + AluJo SINE/Alu 1 284 (18) 9**

**5085 12.6 4.4 2.2 AluYa5_17_40 3359 4122 (3014) C L1MB3 LINE/L1 (1) 6182 5401 10**

**1705 17.5 1.4 1.0 AluYa5_17_40 4123 4416 (2720) C AluJo SINE/Alu (17) 295 1 11**

**5085 12.6 4.4 2.2 AluYa5_17_40 4417 4507 (2629) C L1MB3 LINE/L1 (783) 5400 5308 10**

**1973 19.7 8.0 3.3 AluYa5_17_40 4509 4973 (2163) + MLT2B1 LTR/ERVL 1 534 (20) 12**

**504 8.6 0.0 0.0 AluYa5_17_40 4988 5057 (2079) + (TCCC)n Simple_repeat 3 72 (0) 13**

**354 16.7 0.0 4.0 AluYa5_17_40 5078 5152 (1984) + Ricksha_c DNA/MuDR 851 922 (1126) 14**

**3093 19.8 3.0 0.8 AluYa5_17_40 5164 5884 (1252) + MLT-int LTR/MaLR 58 894 (477) 15**

**286 22.2 1.8 11.6 AluYa5_17_40 5863 5974 (1162) + MLT1A-int LTR/MaLR 657 757 (978) 16 ***

**2063 23.7 1.9 0.0 AluYa5_17_40 6141 6554 (582) + MLT1A-int LTR/MaLR 1314 1735 (0) 16**

**1437 20.1 7.8 4.0 AluYa5_17_40 6555 6902 (234) + MLT1A LTR/MaLR 1 361 (13) 16**

**763 14.4 7.6 2.7 AluYa5_17_40 6908 7097 (39) C L1MB3 LINE/L1 (843) 5303 5105 10**

**__________________________________________________________________________________**

**80. AluYa5_17_44**

**Ortholog annotation C_INTER_RMD_M_DISRUPTED Length 120 nscore 0.00 NPOSITIONS NA**

**Repeat length (main genome) 301**

**Blast2 Results -**

**OFC OLC RFC RLC**

**1 120 1 120**

**17 48 152 183**

**Ortholog Repeat Masker annotation**

**SW perc perc perc query position in query matching repeat position in repeat**

**score div. del. ins. sequence begin end (left) repeat class/family begin end (left) ID**

**918 10.0 0.8 0.0 AluYa5_17_44 1 120 (0) + AluSq/x SINE/Alu 5 125 (187) 1**

**__________________________________________________________________________________**

**81. AluYa5_17_47c**

**Ortholog annotation INDEL_CAN Length 1301 nscore 0.77 NPOSITIONS 1 10 ;**

**Repeat length (main genome) 300**

**Blast2 Results -**

**OFC OLC RFC RLC**

**138 372 65 300**

**760 1051 11 300**

**886 919 2 35**

**Ortholog Repeat Masker annotation**

**SW perc perc perc query position in query matching repeat position in repeat**

**score div. del. ins. sequence begin end (left) repeat class/family begin end (left) ID**

**284 9.5 0.0 0.0 AluYa5_17_47c 92 133 (1168) + L1M4c LINE/L1 17 58 (6326) 1**

**1996 7.2 0.4 0.8 AluYa5_17_47c 134 385 (916) + AluSp SINE/Alu 63 313 (0) 2**

**2644 19.3 2.7 3.1 AluYa5_17_47c 409 750 (551) + L1M3 LINE/L1 4426 4767 (1373) 3**

**2391 9.5 0.0 0.0 AluYa5_17_47c 751 1056 (245) + AluSp SINE/Alu 2 307 (6) 4**

**2644 19.3 2.7 3.1 AluYa5_17_47c 1057 1301 (0) + L1M3 LINE/L1 4768 5011 (1129) 3**

**__________________________________________________________________________________**

**82. AluYa5_17_48**

**Ortholog annotation C_INTER_RMD_M_DISRUPTED Length 310 nscore 0.00 NPOSITIONS NA**

**Repeat length (main genome) 308**

**Blast2 Results -**

**OFC OLC RFC RLC**

**17 63 17 63**

**Ortholog Repeat Masker annotation**

**SW perc perc perc query position in query matching repeat position in repeat**

**score div. del. ins. sequence begin end (left) repeat class/family begin end (left) ID**

**1955 17.5 0.0 0.0 AluYa5_17_48 1 297 (13) + AluJb SINE/Alu 1 297 (15) 1**

**__________________________________________________________________________________**

**83. AluYa5_17_49**

**Ortholog annotation INDEL_PTS Length 3904 nscore 0.00 NPOSITIONS NA**

**Repeat length (main genome) 309**

**Blast2 Results -**

**OFC OLC RFC RLC**

**1 275 1 274**

**571 870 5 309**

**3410 3707 1 299**

**Ortholog Repeat Masker annotation**

**SW perc perc perc query position in query matching repeat position in repeat**

**score div. del. ins. sequence begin end (left) repeat class/family begin end (left) ID**

**2115 9.1 0.0 0.4 AluYa5_17_49 1 275 (3629) + AluY SINE/Alu 1 274 (37) 1**

**213 6.5 0.0 0.0 AluYa5_17_49 283 313 (3591) + (TAAA)n Simple_repeat 2 32 (0) 2**

**2197 10.5 1.3 0.0 AluYa5_17_49 567 872 (3032) + AluSg SINE/Alu 1 310 (0) 3**

**252 32.8 0.0 0.0 AluYa5_17_49 1080 1146 (2758) + MIRc SINE/MIR 168 234 (34) 4**

**206 32.5 13.2 0.0 AluYa5_17_49 1199 1312 (2592) + L2b LINE/L2 3048 3176 (199) 5**

**224 26.2 0.0 1.6 AluYa5_17_49 1450 1511 (2393) C MIRc SINE/MIR (37) 231 171 6 ***

**441 35.6 7.4 0.0 AluYa5_17_49 1508 1656 (2248) + L2b LINE/L2 3216 3375 (0) 5**

**2423 6.1 2.7 0.8 AluYa5_17_49 1971 2426 (1478) + L1PA8 LINE/L1 5135 5593 (561) 7**

**4568 4.4 0.2 0.5 AluYa5_17_49 2698 3267 (637) + L1PA8 LINE/L1 5598 6165 (7) 7**

**25 0.0 0.0 0.0 AluYa5_17_49 3268 3292 (612) + AT_rich Low_complexity 1 25 (0) 8**

**2374 6.7 0.3 0.0 AluYa5_17_49 3410 3707 (197) + AluY SINE/Alu 1 299 (12) 9**

**__________________________________________________________________________________**

**84. AluYa5_17_5**

**Ortholog annotation C_INTER_RMD_M_DISRUPTED Length 104 nscore 0.00 NPOSITIONS NA**

**Repeat length (main genome) 110**

**Blast2 Results -**

**OFC OLC RFC RLC**

**1 95 1 95**

**Ortholog Repeat Masker annotation**

**SW perc perc perc query position in query matching repeat position in repeat**

**score div. del. ins. sequence begin end (left) repeat class/family begin end (left) ID**

**730 8.4 0.0 0.0 AluYa5_17_5 1 95 (9) + Alu SINE/Alu 201 295 (15) 1**

**__________________________________________________________________________________**

**85. AluYa5_17_51**

**Ortholog annotation INDEL_PTS Length 1227 nscore 0.00 NPOSITIONS NA**

**Repeat length (main genome) 306**

**Blast2 Results -**

**OFC OLC RFC RLC**

**928 1206 6 284**

**Ortholog Repeat Masker annotation**

**SW perc perc perc query position in query matching repeat position in repeat**

**score div. del. ins. sequence begin end (left) repeat class/family begin end (left) ID**

**2319 10.3 0.0 1.0 AluYa5_17_51 923 1227 (0) + AluSx SINE/Alu 1 302 (10) 1**

**__________________________________________________________________________________**

**86. AluYa5_17_58**

**Ortholog annotation INDEL_PTS Length 7274 nscore 7.81 NPOSITIONS 3179 3746 ;**

**Repeat length (main genome) 310**

**Blast2 Results -**

**OFC OLC RFC RLC**

**97 394 5 305**

**672 713 5 46**

**752 969 90 308**

**2148 2400 6 258**

**2279 2312 2 35**

**3763 3983 5 228**

**4038 4090 5 57**

**4141 4334 102 296**

**4888 5122 1 235**

**5130 5185 255 310**

**Ortholog Repeat Masker annotation**

**SW perc perc perc query position in query matching repeat position in repeat**

**score div. del. ins. sequence begin end (left) repeat class/family begin end (left) ID**

**637 27.8 11.8 1.0 AluYa5_17_58 6 93 (7181) + HAL1 LINE/L1 1852 1949 (558) 1**

**2214 11.6 0.7 0.0 AluYa5_17_58 94 394 (6880) + AluSg SINE/Alu 2 304 (6) 2**

**637 27.8 11.8 1.0 AluYa5_17_58 395 618 (6656) + HAL1 LINE/L1 1950 2198 (309) 1 ***

**822 15.1 0.6 6.4 AluYa5_17_58 614 667 (6607) + L1M4 LINE/L1 3801 3852 (2294) 3**

**2150 11.3 2.3 0.0 AluYa5_17_58 668 969 (6305) + AluSx SINE/Alu 1 309 (3) 4**

**822 15.1 0.6 6.4 AluYa5_17_58 970 1070 (6204) + L1M4 LINE/L1 3853 3947 (2199) 3**

**283 28.6 9.0 9.0 AluYa5_17_58 1081 1174 (6100) + L2c LINE/L2 3031 3125 (262) 5**

**2275 12.0 0.0 0.0 AluYa5_17_58 1175 1475 (5799) C AluSx SINE/Alu (11) 301 1 6**

**283 28.6 9.0 9.0 AluYa5_17_58 1476 1540 (5734) + L2c LINE/L2 3126 3191 (196) 5**

**2631 5.8 0.0 0.0 AluYa5_17_58 1541 1849 (5425) C AluY SINE/Alu (2) 309 1 7**

**283 28.6 9.0 9.0 AluYa5_17_58 1850 2011 (5263) + L2c LINE/L2 3192 3353 (34) 5**

**2345 8.6 0.7 0.3 AluYa5_17_58 2143 2444 (4830) + AluSp SINE/Alu 1 303 (10) 8**

**250 36.4 4.7 3.1 AluYa5_17_58 2451 2708 (4566) C L2 LINE/L2 (337) 3082 2821 9**

**2450 7.2 0.3 0.0 AluYa5_17_58 2728 3019 (4255) C AluSp SINE/Alu (20) 293 1 10**

**1801 9.1 0.9 0.0 AluYa5_17_58 3759 3989 (3285) + AluSc SINE/Alu 1 233 (76) 11**

**243 0.0 0.0 0.0 AluYa5_17_58 4006 4032 (3242) + (TAAA)n Simple_repeat 2 28 (0) 12**

**2305 7.5 1.7 2.7 AluYa5_17_58 4034 4334 (2940) + AluSp SINE/Alu 1 298 (15) 13**

**2004 10.0 4.4 0.4 AluYa5_17_58 4888 5182 (2092) + AluSg SINE/Alu 1 310 (0) 14**

**186 21.1 0.0 0.0 AluYa5_17_58 5206 5243 (2031) + MIRc SINE/MIR 113 150 (118) 15**

**1625 16.2 4.9 0.3 AluYa5_17_58 5974 6258 (1016) C AluJb SINE/Alu (14) 298 1 16**

**__________________________________________________________________________________**

**87. AluYa5_17_73**

**Ortholog annotation INDEL_PTS Length 4052 nscore 0.00 NPOSITIONS NA**

**Repeat length (main genome) 302**

**Blast2 Results -**

**OFC OLC RFC RLC**

**15 306 15 302**

**431 717 13 298**

**771 986 15 229**

**2944 3003 240 299**

**3262 3455 1 193**

**3502 3530 239 267**

**3764 4052 1 285**

**Ortholog Repeat Masker annotation**

**SW perc perc perc query position in query matching repeat position in repeat**

**score div. del. ins. sequence begin end (left) repeat class/family begin end (left) ID**

**2204 13.0 0.0 1.0 AluYa5_17_73 1 310 (3742) + AluSx SINE/Alu 1 307 (5) 1**

**6443 14.8 1.2 3.5 AluYa5_17_73 311 418 (3634) + L1M2 LINE/L1 2257 2362 (3781) 2**

**2481 8.7 0.3 0.0 AluYa5_17_73 419 717 (3335) + AluSp SINE/Alu 1 300 (13) 3**

**6443 14.8 1.2 3.5 AluYa5_17_73 718 756 (3296) + L1M2 LINE/L1 2363 2401 (3742) 2**

**2322 9.4 0.3 0.0 AluYa5_17_73 757 1043 (3009) + AluSq SINE/Alu 1 288 (25) 4**

**6443 14.8 1.2 3.5 AluYa5_17_73 1044 1924 (2128) + L1M2 LINE/L1 2402 3265 (2878) 2**

**1596 16.9 3.6 2.3 AluYa5_17_73 1925 2232 (1820) + AluJo SINE/Alu 1 312 (0) 5**

**6443 14.8 1.2 3.5 AluYa5_17_73 2233 2478 (1574) + L1M2 LINE/L1 3266 3504 (2639) 2**

**969 16.6 0.0 0.0 AluYa5_17_73 2482 2626 (1426) + AluJ SINE/Alu 1 145 (167) 6**

**1024 20.1 0.0 0.0 AluYa5_17_73 2825 3003 (1049) + AluJ SINE/Alu 122 300 (12) 6**

**2751 10.8 3.7 1.2 AluYa5_17_73 3004 3261 (791) + L1M2 LINE/L1 3497 3762 (2381) 2**

**2030 13.0 0.0 1.4 AluYa5_17_73 3262 3550 (502) + AluSp SINE/Alu 1 285 (28) 7**

**369 0.0 0.0 0.0 AluYa5_17_73 3551 3591 (461) + (CAA)n Simple_repeat 2 42 (0) 8**

**2751 10.8 3.7 1.2 AluYa5_17_73 3592 3762 (290) + L1M2 LINE/L1 3763 3938 (2205) 2**

**2126 9.8 0.3 1.4 AluYa5_17_73 3764 4052 (0) + AluSx SINE/Alu 1 286 (26) 9**

**__________________________________________________________________________________**

**88. AluYa5_17_77c**

**Ortholog annotation C_INTER_RMD_M_DISRUPTED Length 128 nscore 0.00 NPOSITIONS NA**

**Repeat length (main genome) 310**

**Blast2 Results -**

**OFC OLC RFC RLC**

**1 128 1 126**

**Ortholog Repeat Masker annotation**

**SW perc perc perc query position in query matching repeat position in repeat**

**score div. del. ins. sequence begin end (left) repeat class/family begin end (left) ID**

**1044 9.4 0.0 0.0 AluYa5_17_77c 1 128 (0) + AluSq/x SINE/Alu 1 128 (184) 1**

**__________________________________________________________________________________**

**89. AluYa5_17_85**

**Ortholog annotation C_INTER_RMD_M_DISRUPTED Length 134 nscore 0.00 NPOSITIONS NA**

**Repeat length (main genome) 313**

**Blast2 Results -**

**OFC OLC RFC RLC**

**1 134 1 134**

**Ortholog Repeat Masker annotation**

**SW perc perc perc query position in query matching repeat position in repeat**

**score div. del. ins. sequence begin end (left) repeat class/family begin end (left) ID**

**1056 7.5 0.0 0.8 AluYa5_17_85 1 134 (0) + AluSq/x SINE/Alu 1 133 (179) 1**

**__________________________________________________________________________________**

**90. AluYa5_17_87**

**Ortholog annotation INDEL_CAN Length 1697 nscore 0.00 NPOSITIONS NA**

**Repeat length (main genome) 303**

**Blast2 Results -**

**OFC OLC RFC RLC**

**1 295 1 294**

**Ortholog Repeat Masker annotation**

**SW perc perc perc query position in query matching repeat position in repeat**

**score div. del. ins. sequence begin end (left) repeat class/family begin end (left) ID**

**2415 6.8 0.0 0.3 AluYa5_17_87 1 295 (1402) + AluY SINE/Alu 1 294 (17) 1**

**229 20.8 2.1 0.0 AluYa5_17_87 809 856 (841) + L2b LINE/L2 3312 3360 (15) 2**

**320 14.9 0.0 0.0 AluYa5_17_87 876 922 (775) + Alu SINE/Alu 1 47 (255) 3**

**338 21.1 6.4 4.3 AluYa5_17_87 954 1047 (650) C MamRep1879 DNA/hAT? (0) 216 121 4**

**181 28.8 3.5 5.9 AluYa5_17_87 1218 1302 (395) + MIRb SINE/MIR 117 199 (69) 5**

**278 22.2 7.4 0.0 AluYa5_17_87 1306 1386 (311) + Kanga1 DNA/Tc2 3 89 (1656) 6**

**1867 11.0 0.4 0.4 AluYa5_17_87 1397 1678 (19) C AluSx SINE/Alu (0) 312 28 7**

**__________________________________________________________________________________**

**91. AluYa5_17_95**

**Ortholog annotation INDEL_CAN Length 1183 nscore 0.00 NPOSITIONS NA**

**Repeat length (main genome) 302**

**Blast2 Results -**

**OFC OLC RFC RLC**

**no hits found**

**Ortholog Repeat Masker annotation**

**SW perc perc perc query position in query matching repeat position in repeat**

**score div. del. ins. sequence begin end (left) repeat class/family begin end (left) ID**

**207 39.4 3.0 0.8 AluYa5_17_95 88 220 (963) C MIRb SINE/MIR (100) 168 33 1**

**3846 4.8 0.0 0.0 AluYa5_17_95 550 1031 (152) + L1PA5 LINE/L1 5695 6154 (0) 2**

**__________________________________________________________________________________**

**92. AluYa5_17_98c**

**Ortholog annotation INDEL_CAN Length 3447 nscore 18.62 NPOSITIONS 72 552 ; 586 587 ; 589 589 ; 591 591 ; 611 612 ; 615 615 ; 620 620 ; 625 625 ; 628 628 ; 652 652 ; 2617 2736 ; 3211 3240 ;**

**Repeat length (main genome) 286**

**Blast2 Results -**

**OFC OLC RFC RLC**

**850 880 1 31**

**2339 2453 155 270**

**2737 2870 153 286**

**3241 3447 80 286**

**Ortholog Repeat Masker annotation**

**SW perc perc perc query position in query matching repeat position in repeat**

**score div. del. ins. sequence begin end (left) repeat class/family begin end (left) ID**

**573 29.6 0.0 0.0 AluYa5_17_98c 555 686 (2761) C AluSq/x SINE/Alu (179) 133 2 1**

**262 7.2 0.0 0.0 AluYa5_17_98c 848 887 (2560) + Alu SINE/Alu 1 35 (267) 2**

**257 28.7 1.0 8.2 AluYa5_17_98c 949 1051 (2396) C MER5B DNA/MER1_type (3) 175 91 3**

**1995 13.9 0.3 0.7 AluYa5_17_98c 1165 1468 (1979) C AluSx SINE/Alu (7) 305 3 4**

**628 19.8 0.0 4.1 AluYa5_17_98c 1676 1796 (1651) C Tigger1 DNA/MER2_type (1) 2417 2302 5**

**992 21.7 2.9 1.0 AluYa5_17_98c 1797 2001 (1446) + AluJb SINE/Alu 86 294 (18) 6**

**259 15.2 10.2 0.0 AluYa5_17_98c 2005 2063 (1384) C Tigger1 DNA/MER2_type (122) 2296 2232 5**

**762 20.4 2.4 8.2 AluYa5_17_98c 2084 2291 (1156) C AluJo SINE/Alu (32) 280 85 7**

**854 14.5 0.0 0.0 AluYa5_17_98c 2339 2469 (978) + AluSg/x SINE/Alu 154 284 (25) 8**

**192 7.7 0.0 0.0 AluYa5_17_98c 2489 2514 (933) + (TATG)n Simple_repeat 2 27 (0) 9**

**1016 10.4 0.0 0.0 AluYa5_17_98c 2737 2870 (577) + Alu SINE/Alu 153 286 (24) 10**

**323 9.6 1.9 0.0 AluYa5_17_98c 2872 2923 (524) + (TA)n Simple_repeat 2 54 (0) 11**

**611 11.7 0.0 0.0 AluYa5_17_98c 3113 3206 (241) C AluJ/FRAM SINE/Alu (2) 310 217 12**

**1658 8.2 0.0 0.0 AluYa5_17_98c 3241 3447 (0) + AluY SINE/Alu 80 286 (25) 13**

**__________________________________________________________________________________**

**93. AluYa5_18_106**

**Ortholog annotation INDEL_PTS Length 4049 nscore 0.00 NPOSITIONS NA**

**Repeat length (main genome) 300**

**Blast2 Results -**

**OFC OLC RFC RLC**

**Ortholog Repeat Masker annotation**

**SW perc perc perc query position in query matching repeat position in repeat**

**score div. del. ins. sequence begin end (left) repeat class/family begin end (left) ID**

**23 5.4 0.0 0.0 AluYa5_18_106 364 400 (3649) + AT_rich Low_complexity 1 37 (0) 1**

**436 31.8 5.3 6.9 AluYa5_18_106 416 733 (3316) C L2c LINE/L2 (0) 3387 3075 2**

**362 36.2 8.9 2.1 AluYa5_18_106 763 1089 (2960) C L2c LINE/L2 (410) 3009 2661 2**

**331 35.5 4.0 2.4 AluYa5_18_106 1123 1246 (2803) + L3 LINE/CR1 4363 4488 (1) 3**

**24 6.7 0.0 0.0 AluYa5_18_106 2986 3030 (1019) + AT_rich Low_complexity 1 45 (0) 4**

**623 18.2 0.0 0.0 AluYa5_18_106 3828 3937 (112) C MER31A LTR/ERV1 (120) 365 256 5**

**__________________________________________________________________________________**

**94. AluYa5_18_17c**

**Ortholog annotation INDEL_CAN Length 273 nscore 0.00 NPOSITIONS NA**

**Repeat length (main genome) 281**

**Blast2 Results -**

**OFC OLC RFC RLC**

**1 273 1 272**

**Ortholog Repeat Masker annotation**

**SW perc perc perc query position in query matching repeat position in repeat**

**score div. del. ins. sequence begin end (left) repeat class/family begin end (left) ID**

**2004 6.6 6.6 0.7 AluYa5_18_17c 1 273 (0) + AluYd8 SINE/Alu 1 289 (10) 1**

**__________________________________________________________________________________**

**95. AluYa5_18_49**

**Ortholog annotation INDEL_CAN Length 267 nscore 0.00 NPOSITIONS NA**

**Repeat length (main genome) 278**

**Blast2 Results -**

**OFC OLC RFC RLC**

**1 267 1 266**

**Ortholog Repeat Masker annotation**

**SW perc perc perc query position in query matching repeat position in repeat**

**score div. del. ins. sequence begin end (left) repeat class/family begin end (left) ID**

**2096 7.9 0.0 0.4 AluYa5_18_49 1 267 (0) + AluYd8 SINE/Alu 20 285 (14) 1**

**__________________________________________________________________________________**

**96. AluYa5_18_58c**

**Ortholog annotation C_INTER_RMD_M_DISRUPTED Length 132 nscore 0.00 NPOSITIONS NA**

**Repeat length (main genome) 308**

**Blast2 Results -**

**OFC OLC RFC RLC**

**5 132 5 132**

**Ortholog Repeat Masker annotation**

**SW perc perc perc query position in query matching repeat position in repeat**

**score div. del. ins. sequence begin end (left) repeat class/family begin end (left) ID**

**1186 4.5 0.0 0.0 AluYa5_18_58c 1 132 (0) + AluY SINE/Alu 1 132 (179) 1**

**__________________________________________________________________________________**

**97. AluYa5_19_10**

**Ortholog annotation INDEL_CAN Length 563 nscore 0.00 NPOSITIONS NA**

**Repeat length (main genome) 313**

**Blast2 Results -**

**OFC OLC RFC RLC**

**1 309 1 307**

**Ortholog Repeat Masker annotation**

**SW perc perc perc query position in query matching repeat position in repeat**

**score div. del. ins. sequence begin end (left) repeat class/family begin end (left) ID**

**2298 10.5 0.0 1.3 AluYa5_19_10 1 309 (254) + AluSq SINE/Alu 2 306 (7) 1**

**188 33.3 7.6 10.3 AluYa5_19_10 373 556 (7) C L2b LINE/L2 (74) 3301 3123 2**

**__________________________________________________________________________________**

**98. AluYa5_19_16**

**Ortholog annotation C_INTER_RMD_M_DISRUPTED Length 130 nscore 0.00 NPOSITIONS NA**

**Repeat length (main genome) 312**

**Blast2 Results -**

**OFC OLC RFC RLC**

**1 130 1 130**

**20 54 156 190**

**Ortholog Repeat Masker annotation**

**SW perc perc perc query position in query matching repeat position in repeat**

**score div. del. ins. sequence begin end (left) repeat class/family begin end (left) ID**

**1028 10.0 0.0 0.0 AluYa5_19_16 1 130 (0) + AluSq/x SINE/Alu 1 130 (182) 1**

**__________________________________________________________________________________**

**99. AluYa5_19_28**

**Ortholog annotation C_INTER_RMD_M_DISRUPTED Length 126 nscore 0.00 NPOSITIONS NA**

**Repeat length (main genome) 310**

**Blast2 Results -**

**OFC OLC RFC RLC**

**1 126 1 126**

**Ortholog Repeat Masker annotation**

**SW perc perc perc query position in query matching repeat position in repeat**

**score div. del. ins. sequence begin end (left) repeat class/family begin end (left) ID**

**1033 6.3 0.0 0.0 AluYa5_19_28 1 126 (0) + AluSg SINE/Alu 1 126 (184) 1**

**__________________________________________________________________________________**

**100. AluYa5_19_36c**

**Ortholog annotation INDEL_PTS Length 725 nscore 0.00 NPOSITIONS NA**

**Repeat length (main genome) 299**

**Blast2 Results -**

**OFC OLC RFC RLC**

**168 199 155 186**

**263 300 246 285**

**Ortholog Repeat Masker annotation**

**SW perc perc perc query position in query matching repeat position in repeat**

**score div. del. ins. sequence begin end (left) repeat class/family begin end (left) ID**

**1687 17.3 1.1 1.4 AluYa5_19_36c 14 300 (425) + AluJo SINE/Alu 1 286 (26) 1**

**__________________________________________________________________________________**

**101. AluYa5_19_57c**

**Ortholog annotation C_INTER_RMD_M_DISRUPTED Length 139 nscore 0.00 NPOSITIONS NA**

**Repeat length (main genome) 191**

**Blast2 Results -**

**OFC OLC RFC RLC**

**1 139 1 139**

**Ortholog Repeat Masker annotation**

**SW perc perc perc query position in query matching repeat position in repeat**

**score div. del. ins. sequence begin end (left) repeat class/family begin end (left) ID**

**1123 7.9 0.0 0.0 AluYa5_19_57c 1 139 (0) + AluYa5 SINE/Alu 110 248 (62) 1**

**__________________________________________________________________________________**

**102. AluYa5_19_60c**

**Ortholog annotation INDEL_PTS Length 247 nscore 0.00 NPOSITIONS NA**

**Repeat length (main genome) 106**

**Blast2 Results -**

**OFC OLC RFC RLC**

**no hits found**

**Ortholog Repeat Masker annotation**

**There were no repetitive sequences detected in /home/vipin/WHOLE_GENOME_CG/AluYa5_CHR/Chimp/CONFIRMATION/AluYa5_INDEL_SEQUENCES/AluYa5_19_60c**

**__________________________________________________________________________________**

**103. AluYa5_19_68c**

**Ortholog annotation INDEL_CAN Length 306 nscore 0.00 NPOSITIONS NA**

**Repeat length (main genome) 289**

**Blast2 Results -**

**OFC OLC RFC RLC**

**1 278 1 278**

**Ortholog Repeat Masker annotation**

**SW perc perc perc query position in query matching repeat position in repeat**

**score div. del. ins. sequence begin end (left) repeat class/family begin end (left) ID**

**2181 9.8 4.4 0.0 AluYa5_19_68c 1 296 (10) + AluYa5 SINE/Alu 1 309 (1) 1**

**__________________________________________________________________________________**

**104. AluYa5_19_72**

**Ortholog annotation INDEL_PTS Length 3428 nscore 17.33 NPOSITIONS 1501 1510 ; 2785 3368 ;**

**Repeat length (main genome) 309**

**Blast2 Results -**

**OFC OLC RFC RLC**

**1 52 1 52**

**22 57 156 191**

**240 266 240 266**

**Ortholog Repeat Masker annotation**

**SW perc perc perc query position in query matching repeat position in repeat**

**score div. del. ins. sequence begin end (left) repeat class/family begin end (left) ID**

**1870 14.4 1.9 2.6 AluYa5_19_72 1 313 (3115) + AluJo SINE/Alu 1 311 (1) 1**

**22 0.0 0.0 0.0 AluYa5_19_72 1532 1553 (1875) + GC_rich Low_complexity 1 22 (0) 2**

**__________________________________________________________________________________**

**105. AluYa5_19_74c**

**Ortholog annotation C_INTER_RMD_M_DISRUPTED Length 307 nscore 0.00 NPOSITIONS NA**

**Repeat length (main genome) 290**

**Blast2 Results -**

**OFC OLC RFC RLC**

**1 290 1 290**

**Ortholog Repeat Masker annotation**

**SW perc perc perc query position in query matching repeat position in repeat**

**score div. del. ins. sequence begin end (left) repeat class/family begin end (left) ID**

**2488 6.9 0.3 0.0 AluYa5_19_74c 1 303 (4) + AluY SINE/Alu 1 304 (7) 1**

**__________________________________________________________________________________**

**106. AluYa5_1_10c**

**Ortholog annotation INDEL_CAN Length 296 nscore 0.00 NPOSITIONS NA**

**Repeat length (main genome) 285**

**Blast2 Results -**

**OFC OLC RFC RLC**

**1 278 1 277**

**271 296 260 285**

**Ortholog Repeat Masker annotation**

**SW perc perc perc query position in query matching repeat position in repeat**

**score div. del. ins. sequence begin end (left) repeat class/family begin end (left) ID**

**2061 5.4 9.2 0.0 AluYa5_1_10c 1 293 (3) + AluY SINE/Alu 1 311 (0) 1**

**__________________________________________________________________________________**

**107. AluYa5_1_138c**

**Ortholog annotation INDEL_CAN Length 292 nscore 0.00 NPOSITIONS NA**

**Repeat length (main genome) 301**

**Blast2 Results -**

**OFC OLC RFC RLC**

**1 292 1 292**

**Ortholog Repeat Masker annotation**

**SW perc perc perc query position in query matching repeat position in repeat**

**score div. del. ins. sequence begin end (left) repeat class/family begin end (left) ID**

**2330 7.2 3.1 0.0 AluYa5_1_138c 1 292 (0) + AluY SINE/Alu 1 301 (10) 1**

**__________________________________________________________________________________**

**108. AluYa5_1_187**

**Ortholog annotation INDEL_CAN Length 487 nscore 0.00 NPOSITIONS NA**

**Repeat length (main genome) 301**

**Blast2 Results -**

**OFC OLC RFC RLC**

**no hits found**

**Ortholog Repeat Masker annotation**

**SW perc perc perc query position in query matching repeat position in repeat**

**score div. del. ins. sequence begin end (left) repeat class/family begin end (left) ID**

**26 0.0 0.0 0.0 AluYa5_1_187 17 42 (445) + AT_rich Low_complexity 1 26 (0) 1**

**__________________________________________________________________________________**

**109. AluYa5_1_19**

**Ortholog annotation C_INTER_RMD_M_DISRUPTED Length 116 nscore 0.00 NPOSITIONS NA**

**Repeat length (main genome) 310**

**Blast2 Results -**

**OFC OLC RFC RLC**

**1 107 1 107**

**Ortholog Repeat Masker annotation**

**SW perc perc perc query position in query matching repeat position in repeat**

**score div. del. ins. sequence begin end (left) repeat class/family begin end (left) ID**

**984 6.0 0.9 0.0 AluYa5_1_19 1 116 (0) + AluY SINE/Alu 1 117 (194) 1**

**__________________________________________________________________________________**

**110. AluYa5_1_190**

**Ortholog annotation C_INTER_RMD_M_DISRUPTED Length 235 nscore 0.00 NPOSITIONS NA**

**Repeat length (main genome) 236**

**Blast2 Results -**

**OFC OLC RFC RLC**

**1 235 1 235**

**Ortholog Repeat Masker annotation**

**SW perc perc perc query position in query matching repeat position in repeat**

**score div. del. ins. sequence begin end (left) repeat class/family begin end (left) ID**

**1698 12.4 0.0 0.4 AluYa5_1_190 1 235 (0) + AluSq SINE/Alu 3 236 (77) 1**

**__________________________________________________________________________________**

**111. AluYa5_1_199c**

**Ortholog annotation INDEL_CAN Length 727 nscore 0.00 NPOSITIONS NA**

**Repeat length (main genome) 304**

**Blast2 Results -**

**OFC OLC RFC RLC**

**61 103 11 53**

**435 727 13 304**

**Ortholog Repeat Masker annotation**

**SW perc perc perc query position in query matching repeat position in repeat**

**score div. del. ins. sequence begin end (left) repeat class/family begin end (left) ID**

**386 15.6 0.0 3.0 AluYa5_1_199c 55 120 (607) + AluJ/FLAM SINE/Alu 7 70 (232) 1**

**1199 22.0 0.0 5.0 AluYa5_1_199c 142 419 (308) + AluJb SINE/Alu 33 296 (16) 2**

**1893 17.2 0.0 0.7 AluYa5_1_199c 423 727 (0) + AluSx SINE/Alu 1 303 (9) 3**

**__________________________________________________________________________________**

**112. AluYa5_1_2**

**Ortholog annotation INDEL_PTS Length 1365 nscore 0.00 NPOSITIONS NA**

**Repeat length (main genome) 308**

**Blast2 Results -**

**OFC OLC RFC RLC**

**10 305 10 304**

**317 600 12 295**

**606 746 130 270**

**Ortholog Repeat Masker annotation**

**SW perc perc perc query position in query matching repeat position in repeat**

**score div. del. ins. sequence begin end (left) repeat class/family begin end (left) ID**

**2204 12.4 0.0 0.0 AluYa5_1_2 7 305 (1060) + AluSx SINE/Alu 7 305 (7) 1**

**2267 10.6 0.0 0.3 AluYa5_1_2 306 608 (757) + AluSg SINE/Alu 1 302 (8) 2**

**1070 12.6 1.3 0.0 AluYa5_1_2 609 759 (606) + AluSg/x SINE/Alu 134 286 (26) 3**

**1150 18.7 7.6 0.0 AluYa5_1_2 805 1066 (299) C L1M3 LINE/L1 (1903) 4237 3956 4**

**254 16.4 0.0 4.3 AluYa5_1_2 1070 1139 (226) C Alu SINE/Alu (0) 300 234 5**

**965 8.3 0.0 0.0 AluYa5_1_2 1140 1259 (106) C AluSq/x SINE/Alu (192) 120 1 6**

**455 19.2 0.0 5.7 AluYa5_1_2 1261 1365 (0) C L1M3 LINE/L1 (2183) 3957 3859 4**

**__________________________________________________________________________________**

**113. AluYa5_1_204c**

**Ortholog annotation INDEL_CAN Length 1748 nscore 0.00 NPOSITIONS NA**

**Repeat length (main genome) 295**

**Blast2 Results -**

**OFC OLC RFC RLC**

**1455 1748 1 292**

**Ortholog Repeat Masker annotation**

**SW perc perc perc query position in query matching repeat position in repeat**

**score div. del. ins. sequence begin end (left) repeat class/family begin end (left) ID**

**257 34.5 4.8 0.0 AluYa5_1_204c 304 387 (1361) + MER102b DNA/MER1_type 2 89 (252) 1**

**1737 15.2 0.4 0.4 AluYa5_1_204c 422 686 (1062) C AluJb SINE/Alu (47) 265 1 2**

**737 28.6 5.2 4.0 AluYa5_1_204c 712 747 (1001) C L1M5 LINE/L1 (1200) 4946 4910 3**

**2324 8.2 0.0 0.3 AluYa5_1_204c 748 1039 (709) C AluY SINE/Alu (20) 291 1 4**

**737 28.6 5.2 4.0 AluYa5_1_204c 1040 1253 (495) C L1M5 LINE/L1 (1237) 4909 4693 3**

**344 15.9 3.3 3.3 AluYa5_1_204c 1261 1351 (397) C Alu SINE/Alu (1) 299 209 5**

**325 26.3 1.0 5.0 AluYa5_1_204c 1352 1451 (297) C L1M5 LINE/L1 (1487) 4659 4564 3**

**2288 10.3 0.3 0.0 AluYa5_1_204c 1455 1745 (3) + AluSq SINE/Alu 1 292 (21) 6**

**__________________________________________________________________________________**

**114. AluYa5_1_205**

**Ortholog annotation INDEL_CAN Length 122 nscore 0.00 NPOSITIONS NA**

**Repeat length (main genome) 108**

**Blast2 Results -**

**OFC OLC RFC RLC**

**1 106 1 106**

**Ortholog Repeat Masker annotation**

**SW perc perc perc query position in query matching repeat position in repeat**

**score div. del. ins. sequence begin end (left) repeat class/family begin end (left) ID**

**941 3.6 0.0 0.0 AluYa5_1_205 1 112 (10) + AluYa5/8 SINE/Alu 199 310 (0) 1**

**__________________________________________________________________________________**

**115. AluYa5_1_212c**

**Ortholog annotation INDEL_CAN Length 306 nscore 0.00 NPOSITIONS NA**

**Repeat length (main genome) 305**

**Blast2 Results -**

**OFC OLC RFC RLC**

**1 306 1 300**

**Ortholog Repeat Masker annotation**

**SW perc perc perc query position in query matching repeat position in repeat**

**score div. del. ins. sequence begin end (left) repeat class/family begin end (left) ID**

**2237 10.9 0.0 1.3 AluYa5_1_212c 1 306 (0) + AluSq SINE/Alu 1 302 (11) 1**

**__________________________________________________________________________________**

**116. AluYa5_1_216c**

**Ortholog annotation C_INTER_RMD_M_DISRUPTED Length 132 nscore 0.00 NPOSITIONS NA**

**Repeat length (main genome) 310**

**Blast2 Results -**

**OFC OLC RFC RLC**

**1 132 1 132**

**Ortholog Repeat Masker annotation**

**SW perc perc perc query position in query matching repeat position in repeat**

**score div. del. ins. sequence begin end (left) repeat class/family begin end (left) ID**

**990 10.6 0.8 0.0 AluYa5_1_216c 1 132 (0) + AluSq/x SINE/Alu 1 133 (179) 1**

**__________________________________________________________________________________**

**117. AluYa5_1_223c**

**Ortholog annotation INDEL_CAN Length 6424 nscore 0.00 NPOSITIONS NA**

**Repeat length (main genome) 281**

**Blast2 Results -**

**OFC OLC RFC RLC**

**5454 5726 5 276**

**Ortholog Repeat Masker annotation**

**SW perc perc perc query position in query matching repeat position in repeat**

**score div. del. ins. sequence begin end (left) repeat class/family begin end (left) ID**

**679 10.4 0.0 1.0 AluYa5_1_223c 62 158 (6266) C AluSq/x SINE/Alu (183) 129 34 1**

**199 30.2 9.2 1.1 AluYa5_1_223c 1362 1448 (4976) + L3 LINE/CR1 3053 3146 (1343) 2**

**879 31.4 4.2 0.3 AluYa5_1_223c 1471 1850 (4574) C Tigger8 DNA/MER2_type (5) 661 267 3**

**21 0.0 0.0 0.0 AluYa5_1_223c 1914 1934 (4490) + AT_rich Low_complexity 1 21 (0) 4**

**1871 26.3 7.3 8.0 AluYa5_1_223c 1969 2415 (4009) C HAL1 LINE/L1 (109) 2398 1954 5**

**1949 13.6 0.0 3.3 AluYa5_1_223c 2416 2719 (3705) C AluJb SINE/Alu (18) 294 1 6**

**1871 27.5 7.8 6.7 AluYa5_1_223c 2720 3581 (2843) C HAL1 LINE/L1 (554) 1953 976 5**

**2278 10.8 0.3 0.0 AluYa5_1_223c 3582 3887 (2537) C AluSx SINE/Alu (5) 307 1 7**

**1808 29.7 8.8 4.4 AluYa5_1_223c 3888 4497 (1927) C HAL1 LINE/L1 (1532) 975 339 5**

**408 28.3 5.5 7.5 AluYa5_1_223c 4601 4799 (1625) C HAL1 LINE/L1 (2312) 195 1 5**

**21 7.1 0.0 0.0 AluYa5_1_223c 4810 4851 (1573) + AT_rich Low_complexity 1 42 (0) 8**

**2220 13.1 0.0 0.0 AluYa5_1_223c 5421 5726 (698) + AluSx SINE/Alu 1 306 (6) 9**

**333 31.6 7.6 8.7 AluYa5_1_223c 5762 6049 (375) C Charlie23a DNA/MER1_type (54) 285 1 10**

**__________________________________________________________________________________**

**118. AluYa5_1_225c**

**Ortholog annotation C_INTER_RMD_M_DISRUPTED Length 281 nscore 0.00 NPOSITIONS NA**

**Repeat length (main genome) 310**

**Blast2 Results -**

**OFC OLC RFC RLC**

**1 281 1 281**

**Ortholog Repeat Masker annotation**

**SW perc perc perc query position in query matching repeat position in repeat**

**score div. del. ins. sequence begin end (left) repeat class/family begin end (left) ID**

**2176 10.7 0.0 0.0 AluYa5_1_225c 1 281 (0) + AluY SINE/Alu 1 281 (30) 1**

**__________________________________________________________________________________**

**119. AluYa5_1_226**

**Ortholog annotation INDEL_CAN Length 140 nscore 0.00 NPOSITIONS NA**

**Repeat length (main genome) 312**

**Blast2 Results -**

**OFC OLC RFC RLC**

**no hits found**

**Ortholog Repeat Masker annotation**

**SW perc perc perc query position in query matching repeat position in repeat**

**score div. del. ins. sequence begin end (left) repeat class/family begin end (left) ID**

**620 16.5 5.0 0.0 AluYa5_1_226 11 131 (9) + L1MC4a LINE/L1 7479 7605 (277) 1**

**__________________________________________________________________________________**

**120. AluYa5_1_277**

**Ortholog annotation C_INTER_RMD_M_DISRUPTED Length 132 nscore 0.00 NPOSITIONS NA**

**Repeat length (main genome) 302**

**Blast2 Results -**

**OFC OLC RFC RLC**

**1 132 1 132**

**Ortholog Repeat Masker annotation**

**SW perc perc perc query position in query matching repeat position in repeat**

**score div. del. ins. sequence begin end (left) repeat class/family begin end (left) ID**

**1125 6.8 0.0 0.0 AluYa5_1_277 1 132 (0) + AluSq/x SINE/Alu 1 132 (180) 1**

**__________________________________________________________________________________**

**121. AluYa5_1_286c**

**Ortholog annotation C_INTER_RMD_M_DISRUPTED Length 135 nscore 0.00 NPOSITIONS NA**

**Repeat length (main genome) 306**

**Blast2 Results -**

**OFC OLC RFC RLC**

**1 132 1 132**

**Ortholog Repeat Masker annotation**

**SW perc perc perc query position in query matching repeat position in repeat**

**score div. del. ins. sequence begin end (left) repeat class/family begin end (left) ID**

**1184 5.3 0.0 0.0 AluYa5_1_286c 1 132 (3) + AluY SINE/Alu 1 132 (179) 1**

**__________________________________________________________________________________**

**122. AluYa5_1_289c**

**Ortholog annotation INDEL_CAN Length 308 nscore 0.00 NPOSITIONS NA**

**Repeat length (main genome) 311**

**Blast2 Results -**

**OFC OLC RFC RLC**

**1 308 1 308**

**Ortholog Repeat Masker annotation**

**SW perc perc perc query position in query matching repeat position in repeat**

**score div. del. ins. sequence begin end (left) repeat class/family begin end (left) ID**

**2625 5.5 0.0 0.3 AluYa5_1_289c 1 308 (0) + AluY SINE/Alu 1 307 (4) 1**

**__________________________________________________________________________________**

**123. AluYa5_1_300**

**Ortholog annotation INDEL_PTS Length 839 nscore 0.00 NPOSITIONS NA**

**Repeat length (main genome) 32**

**Blast2 Results -**

**OFC OLC RFC RLC**

**1 32 1 32**

**Ortholog Repeat Masker annotation**

**SW perc perc perc query position in query matching repeat position in repeat**

**score div. del. ins. sequence begin end (left) repeat class/family begin end (left) ID**

**431 11.3 0.0 0.0 AluYa5_1_300 1 62 (777) + Alu SINE/Alu 233 294 (16) 1**

**222 30.6 16.3 2.0 AluYa5_1_300 619 765 (74) C MIRc SINE/MIR (49) 219 52 2**

**__________________________________________________________________________________**

**124. AluYa5_1_95**

**Ortholog annotation INDEL_PTS Length 68 nscore 0.00 NPOSITIONS NA**

**Repeat length (main genome) 309**

**Blast2 Results -**

**OFC OLC RFC RLC**

**no hits found**

**Ortholog Repeat Masker annotation**

**SW perc perc perc query position in query matching repeat position in repeat**

**score div. del. ins. sequence begin end (left) repeat class/family begin end (left) ID**

**289 24.6 0.0 0.0 AluYa5_1_95 12 68 (0) + (TA)n Simple_repeat 1 57 (0) 1**

**__________________________________________________________________________________**

**125. AluYa5_20_17**

**Ortholog annotation INDEL_CAN Length 424 nscore 0.00 NPOSITIONS NA**

**Repeat length (main genome) 312**

**Blast2 Results -**

**OFC OLC RFC RLC**

**5 285 5 289**

**Ortholog Repeat Masker annotation**

**SW perc perc perc query position in query matching repeat position in repeat**

**score div. del. ins. sequence begin end (left) repeat class/family begin end (left) ID**

**2307 8.8 0.0 1.1 AluYa5_20_17 1 307 (117) + AluSc SINE/Alu 1 309 (0) 1**

**__________________________________________________________________________________**

**126. AluYa5_20_26**

**Ortholog annotation C_INTER_RMD_M_DISRUPTED Length 290 nscore 0.00 NPOSITIONS NA**

**Repeat length (main genome) 247**

**Blast2 Results -**

**OFC OLC RFC RLC**

**1 235 1 235**

**Ortholog Repeat Masker annotation**

**SW perc perc perc query position in query matching repeat position in repeat**

**score div. del. ins. sequence begin end (left) repeat class/family begin end (left) ID**

**1531 10.1 26.5 0.0 AluYa5_20_26 1 238 (52) + AluSx SINE/Alu 2 302 (0) 1**

**__________________________________________________________________________________**

**127. AluYa5_20_27c**

**Ortholog annotation C_INTRA_RMD Length 131 nscore 0.00 NPOSITIONS NA**

**Repeat length (main genome) 308**

**Blast2 Results -**

**OFC OLC RFC RLC**

**1 131 1 131**

**22 56 156 190**

**Ortholog Repeat Masker annotation**

**SW perc perc perc query position in query matching repeat position in repeat**

**score div. del. ins. sequence begin end (left) repeat class/family begin end (left) ID**

**1109 6.1 0.0 0.0 AluYa5_20_27c 1 131 (0) + AluSg1 SINE/Alu 1 131 (178) 1**

**__________________________________________________________________________________**

**128. AluYa5_20_45c**

**Ortholog annotation INDEL_CAN Length 5041 nscore 0.40 NPOSITIONS 3720 3729 ; 4571 4580 ;**

**Repeat length (main genome) 310**

**Blast2 Results -**

**OFC OLC RFC RLC**

**1 30 271 300**

**1308 1585 10 288**

**3288 3516 10 238**

**3730 3913 104 288**

**4581 4876 1 295**

**Ortholog Repeat Masker annotation**

**SW perc perc perc query position in query matching repeat position in repeat**

**score div. del. ins. sequence begin end (left) repeat class/family begin end (left) ID**

**348 25.0 17.2 1.6 AluYa5_20_45c 117 238 (4803) C MIRc SINE/MIR (99) 169 29 1**

**230 22.2 0.0 0.0 AluYa5_20_45c 772 816 (4225) + Charlie8 DNA/MER1_type 27 71 (2357) 2**

**2365 6.1 0.3 0.0 AluYa5_20_45c 1299 1594 (3447) + AluY SINE/Alu 1 297 (14) 3**

**982 24.6 15.3 2.2 AluYa5_20_45c 1768 2211 (2830) + LTR79 LTR/ERVL 21 522 (5) 4**

**195 12.8 18.8 2.1 AluYa5_20_45c 2436 2483 (2558) + L3 LINE/CR1 3679 3734 (755) 5**

**228 13.9 0.0 0.0 AluYa5_20_45c 2499 2534 (2507) + MER63B DNA/AcHobo 43 78 (358) 6 ***

**563 19.8 8.3 0.0 AluYa5_20_45c 2533 2653 (2388) + MER63B DNA/AcHobo 303 433 (3) 6**

**992 18.1 15.0 0.4 AluYa5_20_45c 2779 3011 (2030) + MLT1D LTR/MaLR 239 505 (0) 7**

**334 24.4 9.8 5.8 AluYa5_20_45c 3096 3269 (1772) + L1MC5 LINE/L1 7410 7590 (371) 8**

**1726 10.2 0.4 0.0 AluYa5_20_45c 3279 3514 (1527) + AluSx SINE/Alu 1 237 (75) 9**

**1424 7.8 0.5 0.0 AluYa5_20_45c 3730 3922 (1119) + AluY SINE/Alu 104 297 (14) 10**

**925 24.2 15.9 2.3 AluYa5_20_45c 4096 4523 (518) + LTR79 LTR/ERVL 21 506 (21) 11**

**2301 9.0 0.3 0.0 AluYa5_20_45c 4581 4891 (150) + AluSp SINE/Alu 1 312 (1) 12**

**__________________________________________________________________________________**

**129. AluYa5_20_47**

**Ortholog annotation INDEL_CAN Length 286 nscore 0.00 NPOSITIONS NA**

**Repeat length (main genome) 273**

**Blast2 Results -**

**OFC OLC RFC RLC**

**1 273 1 273**

**Ortholog Repeat Masker annotation**

**SW perc perc perc query position in query matching repeat position in repeat**

**score div. del. ins. sequence begin end (left) repeat class/family begin end (left) ID**

**2334 5.2 4.2 0.0 AluYa5_20_47 1 286 (0) + AluYa5 SINE/Alu 12 309 (1) 1**

**__________________________________________________________________________________**

**130. AluYa5_20_59**

**Ortholog annotation C_INTER_RMD_M_DISRUPTED Length 121 nscore 0.00 NPOSITIONS NA**

**Repeat length (main genome) 314**

**Blast2 Results -**

**OFC OLC RFC RLC**

**1 121 1 121**

**Ortholog Repeat Masker annotation**

**SW perc perc perc query position in query matching repeat position in repeat**

**score div. del. ins. sequence begin end (left) repeat class/family begin end (left) ID**

**1033 7.4 0.0 0.0 AluYa5_20_59 1 121 (0) + AluSc SINE/Alu 1 121 (188) 1**

**__________________________________________________________________________________**

**131. AluYa5_20_62**

**Ortholog annotation INDEL_CAN Length 279 nscore 0.00 NPOSITIONS NA**

**Repeat length (main genome) 310**

**Blast2 Results -**

**OFC OLC RFC RLC**

**1 70 1 70**

**81 277 99 297**

**Ortholog Repeat Masker annotation**

**SW perc perc perc query position in query matching repeat position in repeat**

**score div. del. ins. sequence begin end (left) repeat class/family begin end (left) ID**

**2037 10.1 6.5 0.0 AluYa5_20_62 1 277 (2) + AluSg1 SINE/Alu 1 295 (14) 1**

**__________________________________________________________________________________**

**132. AluYa5_20_66c**

**Ortholog annotation INDEL_CAN Length 194 nscore 0.00 NPOSITIONS NA**

**Repeat length (main genome) 182**

**Blast2 Results -**

**OFC OLC RFC RLC**

**1 182 1 182**

**Ortholog Repeat Masker annotation**

**SW perc perc perc query position in query matching repeat position in repeat**

**score div. del. ins. sequence begin end (left) repeat class/family begin end (left) ID**

**1449 7.6 0.0 0.0 AluYa5_20_66c 1 185 (9) + AluY SINE/Alu 127 311 (0) 1**

**__________________________________________________________________________________**

**133. AluYa5_20_69**

**Ortholog annotation C_INTER_RMD_M_DISRUPTED Length 261 nscore 0.00 NPOSITIONS NA**

**Repeat length (main genome) 237**

**Blast2 Results -**

**OFC OLC RFC RLC**

**26 261 1 236**

**Ortholog Repeat Masker annotation**

**SW perc perc perc query position in query matching repeat position in repeat**

**score div. del. ins. sequence begin end (left) repeat class/family begin end (left) ID**

**1800 11.4 0.0 0.0 AluYa5_20_69 26 261 (0) + AluSq SINE/Alu 1 236 (77) 1**

**__________________________________________________________________________________**

**134. AluYa5_20_71c**

**Ortholog annotation C_INTER_RMD_M_DISRUPTED Length 126 nscore 0.00 NPOSITIONS NA**

**Repeat length (main genome) 308**

**Blast2 Results -**

**OFC OLC RFC RLC**

**1 123 1 123**

**21 54 158 191**

**Ortholog Repeat Masker annotation**

**SW perc perc perc query position in query matching repeat position in repeat**

**score div. del. ins. sequence begin end (left) repeat class/family begin end (left) ID**

**984 9.8 0.0 0.0 AluYa5_20_71c 1 123 (3) + AluSc SINE/Alu 1 123 (186) 1**

**__________________________________________________________________________________**

**135. AluYa5_20_78**

**Ortholog annotation C_INTER_RMD_M_DISRUPTED Length 133 nscore 0.00 NPOSITIONS NA**

**Repeat length (main genome) 311**

**Blast2 Results -**

**OFC OLC RFC RLC**

**1 133 1 133**

**Ortholog Repeat Masker annotation**

**SW perc perc perc query position in query matching repeat position in repeat**

**score div. del. ins. sequence begin end (left) repeat class/family begin end (left) ID**

**1131 6.8 0.0 0.0 AluYa5_20_78 1 133 (0) + AluSp SINE/Alu 1 133 (180) 1**

**__________________________________________________________________________________**

**136. AluYa5_20_82**

**Ortholog annotation C_INTER_RMD_M_DISRUPTED Length 312 nscore 0.00 NPOSITIONS NA**

**Repeat length (main genome) 303**

**Blast2 Results -**

**OFC OLC RFC RLC**

**1 301 1 303**

**Ortholog Repeat Masker annotation**

**SW perc perc perc query position in query matching repeat position in repeat**

**score div. del. ins. sequence begin end (left) repeat class/family begin end (left) ID**

**2546 6.8 0.0 0.0 AluYa5_20_82 1 308 (4) + AluYa5 SINE/Alu 2 309 (1) 1**

**__________________________________________________________________________________**

**137. AluYa5_21_6c**

**Ortholog annotation INDEL_CAN Length 164 nscore 0.00 NPOSITIONS NA**

**Repeat length (main genome) 299**

**Blast2 Results -**

**OFC OLC RFC RLC**

**no hits found**

**Ortholog Repeat Masker annotation**

**SW perc perc perc query position in query matching repeat position in repeat**

**score div. del. ins. sequence begin end (left) repeat class/family begin end (left) ID**

**288 24.3 3.5 3.5 AluYa5_21_6c 50 164 (0) + L1M5 LINE/L1 5015 5129 (1017) 1**

**__________________________________________________________________________________**

**138. AluYa5_22_19**

**Ortholog annotation C_INTER_RMD_M_DISRUPTED Length 168 nscore 0.00 NPOSITIONS NA**

**Repeat length (main genome) 161**

**Blast2 Results -**

**OFC OLC RFC RLC**

**1 158 1 158**

**Ortholog Repeat Masker annotation**

**SW perc perc perc query position in query matching repeat position in repeat**

**score div. del. ins. sequence begin end (left) repeat class/family begin end (left) ID**

**1310 6.8 0.0 0.0 AluYa5_22_19 1 161 (7) + AluYa5 SINE/Alu 150 310 (0) 1**

**__________________________________________________________________________________**

**139. AluYa5_22_24**

**Ortholog annotation INDEL_PTS Length 4095 nscore 0.00 NPOSITIONS NA**

**Repeat length (main genome) 302**

**Blast2 Results -**

**OFC OLC RFC RLC**

**1 299 1 302**

**977 1259 11 289**

**Ortholog Repeat Masker annotation**

**SW perc perc perc query position in query matching repeat position in repeat**

**score div. del. ins. sequence begin end (left) repeat class/family begin end (left) ID**

**2094 11.3 1.3 0.0 AluYa5_22_24 1 301 (3794) + AluSx SINE/Alu 1 305 (7) 1**

**734 21.4 4.5 1.9 AluYa5_22_24 303 459 (3636) + AluJo/FRAM SINE/Alu 136 296 (16) 2**

**2123 10.0 0.3 1.4 AluYa5_22_24 967 1259 (2836) + AluSx SINE/Alu 1 290 (22) 3**

**2379 8.0 0.7 0.0 AluYa5_22_24 1956 2255 (1840) C AluSp SINE/Alu (10) 303 2 4**

**1845 14.1 0.0 5.5 AluYa5_22_24 2328 2619 (1476) C AluSx SINE/Alu (36) 276 1 5**

**375 26.9 14.6 1.3 AluYa5_22_24 2886 3043 (1052) C L1MD LINE/L1 (748) 5398 5220 6**

**1909 15.4 1.6 0.7 AluYa5_22_24 3520 3827 (268) C AluSx SINE/Alu (1) 311 1 7**

**__________________________________________________________________________________**

**140. AluYa5_22_28**

**Ortholog annotation C_INTRA_RMD Length 131 nscore 0.00 NPOSITIONS NA**

**Repeat length (main genome) 307**

**Blast2 Results -**

**OFC OLC RFC RLC**

**5 131 5 129**

**Ortholog Repeat Masker annotation**

**SW perc perc perc query position in query matching repeat position in repeat**

**score div. del. ins. sequence begin end (left) repeat class/family begin end (left) ID**

**1004 12.2 0.0 0.0 AluYa5_22_28 1 131 (0) + AluSq/x SINE/Alu 2 132 (180) 1**

**__________________________________________________________________________________**

**141. AluYa5_22_31**

**Ortholog annotation C_INTER_RMD_M_DISRUPTED Length 318 nscore 0.00 NPOSITIONS NA**

**Repeat length (main genome) 264**

**Blast2 Results -**

**OFC OLC RFC RLC**

**1 241 1 248**

**Ortholog Repeat Masker annotation**

**SW perc perc perc query position in query matching repeat position in repeat**

**score div. del. ins. sequence begin end (left) repeat class/family begin end (left) ID**

**1953 8.6 0.8 0.0 AluYa5_22_31 1 244 (74) + AluY SINE/Alu 54 299 (12) 1**

**__________________________________________________________________________________**

**142. AluYa5_2_101c**

**Ortholog annotation INDEL_CAN Length 283 nscore 0.00 NPOSITIONS NA**

**Repeat length (main genome) 297**

**Blast2 Results -**

**OFC OLC RFC RLC**

**1 283 1 283**

**Ortholog Repeat Masker annotation**

**SW perc perc perc query position in query matching repeat position in repeat**

**score div. del. ins. sequence begin end (left) repeat class/family begin end (left) ID**

**2482 2.1 4.2 0.0 AluYa5_2_101c 1 283 (0) + AluYa5 SINE/Alu 1 295 (15) 1**

**__________________________________________________________________________________**

**143. AluYa5_2_109**

**Ortholog annotation C_INTER_RMD_M_DISRUPTED Length 122 nscore 0.00 NPOSITIONS NA**

**Repeat length (main genome) 310**

**Blast2 Results -**

**OFC OLC RFC RLC**

**1 122 1 120**

**Ortholog Repeat Masker annotation**

**SW perc perc perc query position in query matching repeat position in repeat**

**score div. del. ins. sequence begin end (left) repeat class/family begin end (left) ID**

**1015 7.4 0.0 0.0 AluYa5_2_109 1 122 (0) + AluSq/x SINE/Alu 1 122 (190) 1**

**__________________________________________________________________________________**

**144. AluYa5_2_121c**

**Ortholog annotation INDEL_PTS Length 2454 nscore 0.41 NPOSITIONS 2445 2454 ;**

**Repeat length (main genome) 309**

**Blast2 Results -**

**OFC OLC RFC RLC**

**1 300 1 300**

**1874 2106 21 256**

**Ortholog Repeat Masker annotation**

**SW perc perc perc query position in query matching repeat position in repeat**

**score div. del. ins. sequence begin end (left) repeat class/family begin end (left) ID**

**2463 6.3 0.0 0.0 AluYa5_2_121c 1 300 (2154) + AluY SINE/Alu 1 300 (11) 1**

**1856 13.1 1.4 2.5 AluYa5_2_121c 1858 2139 (315) + AluSq SINE/Alu 5 283 (30) 2**

**243 0.0 0.0 0.0 AluYa5_2_121c 2367 2393 (61) + (TCTA)n Simple_repeat 2 28 (0) 3**

**__________________________________________________________________________________**

**145. AluYa5_2_14c**

**Ortholog annotation INDEL_CAN Length 5618 nscore 0.00 NPOSITIONS NA**

**Repeat length (main genome) 302**

**Blast2 Results -**

**OFC OLC RFC RLC**

**1 301 1 300**

**1778 2012 65 301**

**2263 2542 12 287**

**3229 3269 12 52**

**3348 3629 5 285**

**3731 3760 241 270**

**4648 4938 1 290**

**Ortholog Repeat Masker annotation**

**SW perc perc perc query position in query matching repeat position in repeat**

**score div. del. ins. sequence begin end (left) repeat class/family begin end (left) ID**

**2480 8.0 0.0 0.0 AluYa5_2_14c 1 301 (5317) + AluSx SINE/Alu 1 301 (11) 1**

**187 35.4 8.1 9.0 AluYa5_2_14c 981 1312 (4306) + L2b LINE/L2 2984 3312 (63) 2**

**26 3.0 0.0 0.0 AluYa5_2_14c 1646 1678 (3940) + AT_rich Low_complexity 1 33 (0) 3**

**1713 10.2 0.4 0.0 AluYa5_2_14c 1768 2017 (3601) + AluSx SINE/Alu 57 312 (0) 4**

**2192 10.3 0.3 2.0 AluYa5_2_14c 2252 2557 (3061) + AluSg SINE/Alu 1 301 (9) 5**

**217 31.0 4.2 0.0 AluYa5_2_14c 2567 2637 (2981) C MIR3 SINE/MIR (45) 163 90 6**

**495 14.6 21.8 0.0 AluYa5_2_14c 3107 3216 (2402) + L1M1 LINE/L1 5233 5366 (780) 7**

**1216 16.9 18.0 0.0 AluYa5_2_14c 3218 3343 (2275) + AluJo SINE/Alu 1 150 (162) 8**

**2449 6.9 0.0 0.7 AluYa5_2_14c 3344 3627 (1991) + AluSx SINE/Alu 1 284 (28) 9**

**210 3.7 0.0 0.0 AluYa5_2_14c 3628 3654 (1964) + (TAA)n Simple_repeat 2 28 (0) 10**

**1216 16.9 18.0 0.0 AluYa5_2_14c 3661 3788 (1830) + AluJo SINE/Alu 151 301 (11) 8**

**2425 7.8 0.0 0.3 AluYa5_2_14c 4648 4944 (674) + AluY SINE/Alu 1 296 (15) 11**

**267 14.3 4.1 0.0 AluYa5_2_14c 5057 5105 (513) C Alu SINE/Alu (15) 285 235 12**

**2215 11.7 0.0 0.0 AluYa5_2_14c 5192 5473 (145) C AluSx SINE/Alu (28) 284 3 13**

**__________________________________________________________________________________**

**146. AluYa5_2_154c**

**Ortholog annotation INDEL_CAN Length 496 nscore 0.00 NPOSITIONS NA**

**Repeat length (main genome) 309**

**Blast2 Results -**

**OFC OLC RFC RLC**

**no hits found**

**Ortholog Repeat Masker annotation**

**SW perc perc perc query position in query matching repeat position in repeat**

**score div. del. ins. sequence begin end (left) repeat class/family begin end (left) ID**

**38 3.9 0.0 0.0 AluYa5_2_154c 138 189 (307) + AT_rich Low_complexity 1 52 (0) 1**

**__________________________________________________________________________________**

**147. AluYa5_2_186c**

**Ortholog annotation INDEL_CAN Length 281 nscore 0.00 NPOSITIONS NA**

**Repeat length (main genome) 289**

**Blast2 Results -**

**OFC OLC RFC RLC**

**4 278 4 278**

**Ortholog Repeat Masker annotation**

**SW perc perc perc query position in query matching repeat position in repeat**

**score div. del. ins. sequence begin end (left) repeat class/family begin end (left) ID**

**2300 6.4 4.3 0.0 AluYa5_2_186c 1 281 (0) + AluYa5 SINE/Alu 9 301 (9) 1**

**__________________________________________________________________________________**

**148. AluYa5_2_192c**

**Ortholog annotation INDEL_CAN Length 241 nscore 0.00 NPOSITIONS NA**

**Repeat length (main genome) 269**

**Blast2 Results -**

**OFC OLC RFC RLC**

**1 241 1 241**

**Ortholog Repeat Masker annotation**

**SW perc perc perc query position in query matching repeat position in repeat**

**score div. del. ins. sequence begin end (left) repeat class/family begin end (left) ID**

**1409 11.2 24.5 0.0 AluYa5_2_192c 1 241 (0) + AluSx SINE/Alu 1 300 (2) 1**

**__________________________________________________________________________________**

**149. AluYa5_2_201c**

**Ortholog annotation C_INTER_RMD_M_DISRUPTED Length 97 nscore 0.00 NPOSITIONS NA**

**Repeat length (main genome) 275**

**Blast2 Results -**

**OFC OLC RFC RLC**

**1 95 1 95**

**Ortholog Repeat Masker annotation**

**SW perc perc perc query position in query matching repeat position in repeat**

**score div. del. ins. sequence begin end (left) repeat class/family begin end (left) ID**

**842 4.2 0.0 0.0 AluYa5_2_201c 1 95 (2) + AluSc SINE/Alu 36 130 (179) 1**

**__________________________________________________________________________________**

**150. AluYa5_2_250**

**Ortholog annotation C_INTER_RMD_M_DISRUPTED Length 156 nscore 7.05 NPOSITIONS 1 10 ; 131 131 ;**

**Repeat length (main genome) 303**

**Blast2 Results -**

**OFC OLC RFC RLC**

**11 152 157 298**

**Ortholog Repeat Masker annotation**

**SW perc perc perc query position in query matching repeat position in repeat**

**score div. del. ins. sequence begin end (left) repeat class/family begin end (left) ID**

**1229 4.9 0.0 0.0 AluYa5_2_250 11 154 (2) + AluY SINE/Alu 156 299 (12) 1**

**__________________________________________________________________________________**

**151. AluYa5_2_327**

**Ortholog annotation INDEL_CAN Length 173 nscore 0.00 NPOSITIONS NA**

**Repeat length (main genome) 153**

**Blast2 Results -**

**OFC OLC RFC RLC**

**7 151 7 150**

**Ortholog Repeat Masker annotation**

**SW perc perc perc query position in query matching repeat position in repeat**

**score div. del. ins. sequence begin end (left) repeat class/family begin end (left) ID**

**1234 8.0 0.6 0.0 AluYa5_2_327 1 162 (11) + AluY SINE/Alu 149 311 (0) 1**

**__________________________________________________________________________________**

**152. AluYa5_2_341**

**Ortholog annotation C_INTER_RMD_M_DISRUPTED Length 131 nscore 0.00 NPOSITIONS NA**

**Repeat length (main genome) 309**

**Blast2 Results -**

**OFC OLC RFC RLC**

**1 131 1 131**

**Ortholog Repeat Masker annotation**

**SW perc perc perc query position in query matching repeat position in repeat**

**score div. del. ins. sequence begin end (left) repeat class/family begin end (left) ID**

**1068 9.9 0.0 0.0 AluYa5_2_341 1 131 (0) + AluSq/x SINE/Alu 2 132 (180) 1**

**__________________________________________________________________________________**

**153. AluYa5_2_345c**

**Ortholog annotation INDEL_CAN Length 318 nscore 0.00 NPOSITIONS NA**

**Repeat length (main genome) 310**

**Blast2 Results -**

**OFC OLC RFC RLC**

**1 313 1 310**

**Ortholog Repeat Masker annotation**

**SW perc perc perc query position in query matching repeat position in repeat**

**score div. del. ins. sequence begin end (left) repeat class/family begin end (left) ID**

**2488 7.5 0.0 1.3 AluYa5_2_345c 1 313 (5) + AluY SINE/Alu 1 309 (2) 1**

**__________________________________________________________________________________**

**154. AluYa5_2_346**

**Ortholog annotation C_INTER_RMD_M_DISRUPTED Length 132 nscore 0.00 NPOSITIONS NA**

**Repeat length (main genome) 298**

**Blast2 Results -**

**OFC OLC RFC RLC**

**2 132 2 132**

**18 49 158 189**

**Ortholog Repeat Masker annotation**

**SW perc perc perc query position in query matching repeat position in repeat**

**score div. del. ins. sequence begin end (left) repeat class/family begin end (left) ID**

**906 8.5 0.0 3.3 AluYa5_2_346 12 132 (0) + AluSq/x SINE/Alu 15 131 (181) 1**

**__________________________________________________________________________________**

**155. AluYa5_2_354c**

**Ortholog annotation C_INTRA_RMD Length 89 nscore 0.00 NPOSITIONS NA**

**Repeat length (main genome) 262**

**Blast2 Results -**

**OFC OLC RFC RLC**

**1 89 1 89**

**Ortholog Repeat Masker annotation**

**SW perc perc perc query position in query matching repeat position in repeat**

**score div. del. ins. sequence begin end (left) repeat class/family begin end (left) ID**

**632 11.2 0.0 0.0 AluYa5_2_354c 1 89 (0) + AluSg SINE/Alu 43 131 (179) 1**

**__________________________________________________________________________________**

**156. AluYa5_2_359c**

**Ortholog annotation INDEL_PTS Length 155 nscore 0.00 NPOSITIONS NA**

**Repeat length (main genome) 271**

**Blast2 Results -**

**OFC OLC RFC RLC**

**19 136 121 238**

**Ortholog Repeat Masker annotation**

**SW perc perc perc query position in query matching repeat position in repeat**

**score div. del. ins. sequence begin end (left) repeat class/family begin end (left) ID**

**1073 15.6 0.0 0.0 AluYa5_2_359c 1 154 (1) + AluSg/x SINE/Alu 138 291 (21) 1**

**__________________________________________________________________________________**

**157. AluYa5_2_3c**

**Ortholog annotation INDEL_CAN Length 80 nscore 0.00 NPOSITIONS NA**

**Repeat length (main genome) 308**

**Blast2 Results -**

**OFC OLC RFC RLC**

**no hits found**

**Ortholog Repeat Masker annotation**

**There were no repetitive sequences detected in /home/vipin/WHOLE_GENOME_CG/AluYa5_CHR/Chimp/CONFIRMATION/AluYa5_INDEL_SEQUENCES/AluYa5_2_3c**

**__________________________________________________________________________________**

**158. AluYa5_2_43**

**Ortholog annotation C_INTER_RMD_M_DISRUPTED Length 139 nscore 0.00 NPOSITIONS NA**

**Repeat length (main genome) 313**

**Blast2 Results -**

**OFC OLC RFC RLC**

**1 139 1 139**

**Ortholog Repeat Masker annotation**

**SW perc perc perc query position in query matching repeat position in repeat**

**score div. del. ins. sequence begin end (left) repeat class/family begin end (left) ID**

**1020 8.3 0.0 5.0 AluYa5_2_43 1 139 (0) + AluSq/x SINE/Alu 2 133 (179) 1**

**__________________________________________________________________________________**

**159. AluYa5_2_54c**

**Ortholog annotation INDEL_PTS Length 1738 nscore 2.19 NPOSITIONS 1123 1125 ; 1128 1128 ; 1134 1135 ; 1154 1154 ; 1577 1577 ; 1709 1738 ;**

**Repeat length (main genome) 290**

**Blast2 Results -**

**OFC OLC RFC RLC**

**1 76 1 74**

**146 292 145 290**

**Ortholog Repeat Masker annotation**

**SW perc perc perc query position in query matching repeat position in repeat**

**score div. del. ins. sequence begin end (left) repeat class/family begin end (left) ID**

**2065 14.2 0.0 0.0 AluYa5_2_54c 1 296 (1442) + AluSq SINE/Alu 12 307 (6) 1**

**676 15.9 1.2 1.9 AluYa5_2_54c 957 1116 (622) + CT-rich Low_complexity 3 161 (0) 2**

**1233 22.4 0.3 0.7 AluYa5_2_54c 1173 1455 (283) C AluSx SINE/Alu (30) 282 1 3**

**__________________________________________________________________________________**

**160. AluYa5_2_55c**

**Ortholog annotation INDEL_CAN Length 992 nscore 0.00 NPOSITIONS NA**

**Repeat length (main genome) 309**

**Blast2 Results -**

**OFC OLC RFC RLC**

**1 180 1 180**

**693 777 220 304**

**Ortholog Repeat Masker annotation**

**SW perc perc perc query position in query matching repeat position in repeat**

**score div. del. ins. sequence begin end (left) repeat class/family begin end (left) ID**

**1998 14.2 0.3 1.3 AluYa5_2_55c 1 300 (692) + AluJo SINE/Alu 1 297 (15) 1**

**192 12.5 0.0 0.0 AluYa5_2_55c 378 409 (583) + (TA)n Simple_repeat 2 33 (0) 2**

**1805 14.5 0.0 0.4 AluYa5_2_55c 508 777 (215) + AluJb SINE/Alu 37 305 (7) 3**

**__________________________________________________________________________________**

**161. AluYa5_2_62**

**Ortholog annotation C_INTER_RMD_M_DISRUPTED Length 319 nscore 0.00 NPOSITIONS NA**

**Repeat length (main genome) 305**

**Blast2 Results -**

**OFC OLC RFC RLC**

**1 77 1 75**

**105 195 103 195**

**279 319 252 292**

**Ortholog Repeat Masker annotation**

**SW perc perc perc query position in query matching repeat position in repeat**

**score div. del. ins. sequence begin end (left) repeat class/family begin end (left) ID**

**1613 18.3 2.1 0.0 AluYa5_2_62 1 279 (40) + AluSx SINE/Alu 1 285 (27) 1**

**__________________________________________________________________________________**

**162. AluYa5_2_66c**

**Ortholog annotation INDEL_CAN Length 4727 nscore 0.21 NPOSITIONS 21 30 ;**

**Repeat length (main genome) 192**

**Blast2 Results -**

**OFC OLC RFC RLC**

**3040 3197 25 182**

**Ortholog Repeat Masker annotation**

**SW perc perc perc query position in query matching repeat position in repeat**

**score div. del. ins. sequence begin end (left) repeat class/family begin end (left) ID**

**364 24.4 1.3 1.3 AluYa5_2_66c 145 223 (4504) C MER68-int LTR/ERVL (476) 2514 2436 1**

**2289 22.3 4.9 2.2 AluYa5_2_66c 528 1060 (3667) C HERVL40-int LTR/ERVL (876) 4654 4108 2**

**1993 17.3 0.0 0.0 AluYa5_2_66c 1072 1383 (3344) C AluSx SINE/Alu (0) 312 1 3**

**315 12.2 0.0 0.0 AluYa5_2_66c 1384 1432 (3295) + (TCCC)n Simple_repeat 1 49 (0) 4**

**2013 10.4 0.4 1.8 AluYa5_2_66c 1439 1711 (3016) C AluSp SINE/Alu (44) 269 1 5**

**4071 15.0 4.1 6.7 AluYa5_2_66c 1778 2583 (2144) C LTR1 LTR/ERV1 (0) 785 1 6**

**858 25.0 9.9 5.3 AluYa5_2_66c 2657 2895 (1832) C L1M5 LINE/L1 (1268) 4878 4627 7**

**2148 12.9 0.0 0.3 AluYa5_2_66c 2896 3197 (1530) + AluSx SINE/Alu 1 301 (11) 8**

**858 25.0 9.9 5.3 AluYa5_2_66c 3198 3210 (1517) C L1M5 LINE/L1 (1520) 4626 4613 7**

**225 0.0 0.0 0.0 AluYa5_2_66c 3211 3235 (1492) + (CA)n Simple_repeat 2 26 (0) 9**

**858 25.0 9.9 5.3 AluYa5_2_66c 3236 3336 (1391) C L1M5 LINE/L1 (1534) 4612 4508 7**

**210 3.7 0.0 0.0 AluYa5_2_66c 3338 3364 (1363) + (TTTG)n Simple_repeat 2 28 (0) 10**

**2179 10.0 0.7 0.4 AluYa5_2_66c 3367 3647 (1080) C AluSx SINE/Alu (29) 283 2 11**

**1792 21.4 12.6 3.2 AluYa5_2_66c 3716 4589 (138) + L1ME2z LINE/L1 4930 5886 (558) 12**

**963 7.1 0.0 0.0 AluYa5_2_66c 4611 4723 (4) + AluY SINE/Alu 2 114 (197) 13**

**__________________________________________________________________________________**

**163. AluYa5_2_71c**

**Ortholog annotation C_INTER_RMD_M_DISRUPTED Length 121 nscore 0.00 NPOSITIONS NA**

**Repeat length (main genome) 310**

**Blast2 Results -**

**OFC OLC RFC RLC**

**1 121 1 121**

**Ortholog Repeat Masker annotation**

**SW perc perc perc query position in query matching repeat position in repeat**

**score div. del. ins. sequence begin end (left) repeat class/family begin end (left) ID**

**1020 8.3 0.0 0.0 AluYa5_2_71c 1 121 (0) + AluSg SINE/Alu 1 121 (189) 1**

**__________________________________________________________________________________**

**164. AluYa5_3_103**

**Ortholog annotation INDEL_CAN Length 195 nscore 0.00 NPOSITIONS NA**

**Repeat length (main genome) 184**

**Blast2 Results -**

**OFC OLC RFC RLC**

**1 184 1 184**

**Ortholog Repeat Masker annotation**

**SW perc perc perc query position in query matching repeat position in repeat**

**score div. del. ins. sequence begin end (left) repeat class/family begin end (left) ID**

**1542 6.7 0.0 0.5 AluYa5_3_103 1 194 (1) + AluY SINE/Alu 119 311 (0) 1**

**__________________________________________________________________________________**

**165. AluYa5_3_111**

**Ortholog annotation INDEL_CAN Length 227 nscore 0.00 NPOSITIONS NA**

**Repeat length (main genome) 310**

**Blast2 Results -**

**OFC OLC RFC RLC**

**no hits found**

**Ortholog Repeat Masker annotation**

**SW perc perc perc query position in query matching repeat position in repeat**

**score div. del. ins. sequence begin end (left) repeat class/family begin end (left) ID**

**1053 16.9 0.0 0.0 AluYa5_3_111 6 227 (0) C ALR/Alpha Satellite/centr (32) 222 1 1**

**__________________________________________________________________________________**

**166. AluYa5_3_165c**

**Ortholog annotation INDEL_PTS Length 383 nscore 0.00 NPOSITIONS NA**

**Repeat length (main genome) 331**

**Blast2 Results -**

**OFC OLC RFC RLC**

**no hits found**

**Ortholog Repeat Masker annotation**

**SW perc perc perc query position in query matching repeat position in repeat**

**score div. del. ins. sequence begin end (left) repeat class/family begin end (left) ID**

**3043 2.3 0.0 0.0 AluYa5_3_165c 26 373 (10) + L1PA2 LINE/L1 5808 6155 (0) 1**

**__________________________________________________________________________________**

**167. AluYa5_3_171**

**Ortholog annotation C_INTER_RMD_M_DISRUPTED Length 158 nscore 0.00 NPOSITIONS NA**

**Repeat length (main genome) 181**

**Blast2 Results -**

**OFC OLC RFC RLC**

**1 158 1 158**

**Ortholog Repeat Masker annotation**

**SW perc perc perc query position in query matching repeat position in repeat**

**score div. del. ins. sequence begin end (left) repeat class/family begin end (left) ID**

**1367 4.4 0.0 0.0 AluYa5_3_171 1 158 (0) + AluY SINE/Alu 130 287 (24) 1**

**__________________________________________________________________________________**

**168. AluYa5_3_174c**

**Ortholog annotation C_INTER_RMD_M_DISRUPTED Length 127 nscore 0.00 NPOSITIONS NA**

**Repeat length (main genome) 304**

**Blast2 Results -**

**OFC OLC RFC RLC**

**1 127 1 127**

**Ortholog Repeat Masker annotation**

**SW perc perc perc query position in query matching repeat position in repeat**

**score div. del. ins. sequence begin end (left) repeat class/family begin end (left) ID**

**1091 6.3 0.0 0.0 AluYa5_3_174c 1 127 (0) + AluSg SINE/Alu 5 131 (179) 1**

**__________________________________________________________________________________**

**169. AluYa5_3_175**

**Ortholog annotation INDEL_CAN Length 269 nscore 0.00 NPOSITIONS NA**

**Repeat length (main genome) 286**

**Blast2 Results -**

**OFC OLC RFC RLC**

**1 269 1 269**

**240 269 252 281**

**240 269 256 285**

**240 266 260 286**

**Ortholog Repeat Masker annotation**

**SW perc perc perc query position in query matching repeat position in repeat**

**score div. del. ins. sequence begin end (left) repeat class/family begin end (left) ID**

**1635 8.4 17.1 0.0 AluYa5_3_175 1 269 (0) + AluYa8 SINE/Alu 1 310 (0) 1**

**__________________________________________________________________________________**

**170. AluYa5_3_223c**

**Ortholog annotation C_INTER_RMD_M_DISRUPTED Length 188 nscore 0.00 NPOSITIONS NA**

**Repeat length (main genome) 168**

**Blast2 Results -**

**OFC OLC RFC RLC**

**1 168 1 168**

**Ortholog Repeat Masker annotation**

**SW perc perc perc query position in query matching repeat position in repeat**

**score div. del. ins. sequence begin end (left) repeat class/family begin end (left) ID**

**1442 8.0 0.5 0.0 AluYa5_3_223c 1 188 (0) + AluY SINE/Alu 123 311 (0) 1**

**__________________________________________________________________________________**

**171. AluYa5_3_235c**

**Ortholog annotation C_INTER_RMD_M_DISRUPTED Length 335 nscore 0.00 NPOSITIONS NA**

**Repeat length (main genome) 348**

**Blast2 Results -**

**OFC OLC RFC RLC**

**1 335 1 338**

**Ortholog Repeat Masker annotation**

**SW perc perc perc query position in query matching repeat position in repeat**

**score div. del. ins. sequence begin end (left) repeat class/family begin end (left) ID**

**1301 14.6 3.0 14.3 AluYa5_3_235c 1 335 (0) + AluYb9 SINE/Alu 1 297 (21) 1**

**__________________________________________________________________________________**

**172. AluYa5_3_245**

**Ortholog annotation INDEL_CAN Length 462 nscore 0.00 NPOSITIONS NA**

**Repeat length (main genome) 313**

**Blast2 Results -**

**OFC OLC RFC RLC**

**no hits found**

**Ortholog Repeat Masker annotation**

**SW perc perc perc query position in query matching repeat position in repeat**

**score div. del. ins. sequence begin end (left) repeat class/family begin end (left) ID**

**24 3.2 0.0 0.0 AluYa5_3_245 179 209 (253) + AT_rich Low_complexity 1 31 (0) 1**

**300 26.4 14.3 6.0 AluYa5_3_245 314 446 (16) C MLT1J LTR/MaLR (81) 431 288 2**

**__________________________________________________________________________________**

**173. AluYa5_3_256**

**Ortholog annotation INDEL_CAN Length 166 nscore 0.00 NPOSITIONS NA**

**Repeat length (main genome) 144**

**Blast2 Results -**

**OFC OLC RFC RLC**

**1 143 1 143**

**Ortholog Repeat Masker annotation**

**SW perc perc perc query position in query matching repeat position in repeat**

**score div. del. ins. sequence begin end (left) repeat class/family begin end (left) ID**

**1174 8.1 0.0 0.0 AluYa5_3_256 1 148 (18) + AluY SINE/Alu 164 311 (0) 1**

**__________________________________________________________________________________**

**174. AluYa5_3_32**

**Ortholog annotation C_INTRA_RMD Length 128 nscore 0.00 NPOSITIONS NA**

**Repeat length (main genome) 299**

**Blast2 Results -**

**OFC OLC RFC RLC**

**1 128 1 131**

**20 56 154 190**

**Ortholog Repeat Masker annotation**

**SW perc perc perc query position in query matching repeat position in repeat**

**score div. del. ins. sequence begin end (left) repeat class/family begin end (left) ID**

**974 7.1 3.1 0.8 AluYa5_3_32 1 128 (0) + AluY SINE/Alu 1 131 (180) 1**

**__________________________________________________________________________________**

**175. AluYa5_3_36**

**Ortholog annotation INDEL_PTS Length 827 nscore 1.21 NPOSITIONS 12 21 ;**

**Repeat length (main genome) 313**

**Blast2 Results -**

**OFC OLC RFC RLC**

**692 815 1 122**

**Ortholog Repeat Masker annotation**

**SW perc perc perc query position in query matching repeat position in repeat**

**score div. del. ins. sequence begin end (left) repeat class/family begin end (left) ID**

**406 13.2 11.8 0.0 AluYa5_3_36 37 104 (723) C AluJ/FLAM SINE/Alu (222) 80 5 1**

**1142 11.2 12.8 7.6 AluYa5_3_36 110 164 (663) + Tigger3b DNA/MER2_type 525 583 (648) 2**

**1900 16.3 0.3 0.0 AluYa5_3_36 165 458 (369) C AluJb SINE/Alu (9) 303 9 3**

**1142 11.2 12.8 7.6 AluYa5_3_36 459 691 (136) + Tigger3b DNA/MER2_type 584 828 (403) 2**

**1119 7.3 0.7 0.0 AluYa5_3_36 692 827 (0) + AluSq/x SINE/Alu 1 137 (175) 4**

**__________________________________________________________________________________**

**176. AluYa5_3_41c**

**Ortholog annotation INDEL_CAN Length 97 nscore 0.00 NPOSITIONS NA**

**Repeat length (main genome) 104**

**Blast2 Results -**

**OFC OLC RFC RLC**

**1 97 1 93**

**Ortholog Repeat Masker annotation**

**SW perc perc perc query position in query matching repeat position in repeat**

**score div. del. ins. sequence begin end (left) repeat class/family begin end (left) ID**

**754 3.1 0.0 1.0 AluYa5_3_41c 1 97 (0) + AluSg/x SINE/Alu 206 301 (11) 1**

**__________________________________________________________________________________**

**177. AluYa5_3_44c**

**Ortholog annotation INDEL_CAN Length 244 nscore 12.30 NPOSITIONS 148 177 ;**

**Repeat length (main genome) 310**

**Blast2 Results -**

**OFC OLC RFC RLC**

**no hits found**

**Ortholog Repeat Masker annotation**

**There were no repetitive sequences detected in /home/vipin/WHOLE_GENOME_CG/AluYa5_CHR/Chimp/CONFIRMATION/AluYa5_INDEL_SEQUENCES/AluYa5_3_44c**

**__________________________________________________________________________________**

**178. AluYa5_3_60**

**Ortholog annotation C_INTER_RMD_M_DISRUPTED Length 104 nscore 0.00 NPOSITIONS NA**

**Repeat length (main genome) 297**

**Blast2 Results -**

**OFC OLC RFC RLC**

**1 85 1 85**

**Ortholog Repeat Masker annotation**

**SW perc perc perc query position in query matching repeat position in repeat**

**score div. del. ins. sequence begin end (left) repeat class/family begin end (left) ID**

**827 9.6 0.0 0.0 AluYa5_3_60 1 104 (0) + AluSg SINE/Alu 1 104 (206) 1**

**__________________________________________________________________________________**

**179. AluYa5_3_61c**

**Ortholog annotation C_INTER_RMD_M_DISRUPTED Length 132 nscore 0.00 NPOSITIONS NA**

**Repeat length (main genome) 307**

**Blast2 Results -**

**OFC OLC RFC RLC**

**1 132 1 132**

**Ortholog Repeat Masker annotation**

**SW perc perc perc query position in query matching repeat position in repeat**

**score div. del. ins. sequence begin end (left) repeat class/family begin end (left) ID**

**1103 8.3 0.0 0.0 AluYa5_3_61c 1 132 (0) + AluSp SINE/Alu 1 132 (181) 1**

**__________________________________________________________________________________**

**180. AluYa5_4_193c**

**Ortholog annotation C_INTER_RMD_M_DISRUPTED Length 177 nscore 0.00 NPOSITIONS NA**

**Repeat length (main genome) 300**

**Blast2 Results -**

**OFC OLC RFC RLC**

**1 176 124 300**

**Ortholog Repeat Masker annotation**

**SW perc perc perc query position in query matching repeat position in repeat**

**score div. del. ins. sequence begin end (left) repeat class/family begin end (left) ID**

**1497 2.9 0.0 2.3 AluYa5_4_193c 1 177 (0) + AluY SINE/Alu 124 296 (15) 1**

**__________________________________________________________________________________**

**181. AluYa5_4_194c**

**Ortholog annotation INDEL_CAN Length 4584 nscore 0.00 NPOSITIONS NA**

**Repeat length (main genome) 308**

**Blast2 Results -**

**OFC OLC RFC RLC**

**1 290 1 297**

**Ortholog Repeat Masker annotation**

**SW perc perc perc query position in query matching repeat position in repeat**

**score div. del. ins. sequence begin end (left) repeat class/family begin end (left) ID**

**2275 5.6 3.1 1.0 AluYa5_4_194c 1 290 (4294) + AluY SINE/Alu 1 296 (15) 1**

**199 12.2 9.8 0.0 AluYa5_4_194c 383 464 (4120) + (TATAA)n Simple_repeat 1 90 (0) 2**

**289 27.9 8.3 3.5 AluYa5_4_194c 1246 1390 (3194) + L2a LINE/L2 3261 3412 (14) 3**

**662 32.2 9.5 1.5 AluYa5_4_194c 2022 2798 (1786) + L1M5 LINE/L1 2956 3794 (2352) 4**

**661 25.0 7.3 9.4 AluYa5_4_194c 2875 2923 (1661) + L1MC4 LINE/L1 7710 7758 (284) 5**

**2122 20.5 8.8 0.2 AluYa5_4_194c 2924 3421 (1163) C MER34 LTR/ERV1 (0) 542 2 6**

**661 25.0 7.3 9.4 AluYa5_4_194c 3422 3711 (873) + L1MC4 LINE/L1 7759 8042 (0) 5**

**636 24.8 0.7 1.4 AluYa5_4_194c 3788 3933 (651) + MER5B DNA/MER1_type 2 146 (32) 7**

**1316 23.7 4.8 4.2 AluYa5_4_194c 3934 4290 (294) + MLT1A LTR/MaLR 1 359 (15) 8**

**204 17.9 0.0 2.5 AluYa5_4_194c 4300 4339 (245) C MER5B DNA/MER1_type (139) 39 1 9**

**__________________________________________________________________________________**

**182. AluYa5_4_220c**

**Ortholog annotation INDEL_CAN Length 69 nscore 0.00 NPOSITIONS NA**

**Repeat length (main genome) 309**

**Blast2 Results -**

**OFC OLC RFC RLC**

**no hits found**

**Ortholog Repeat Masker annotation**

**There were no repetitive sequences detected in /home/vipin/WHOLE_GENOME_CG/AluYa5_CHR/Chimp/CONFIRMATION/AluYa5_INDEL_SEQUENCES/AluYa5_4_220c**

**__________________________________________________________________________________**

**183. AluYa5_4_245**

**Ortholog annotation INDEL_CAN Length 954 nscore 0.00 NPOSITIONS NA**

**Repeat length (main genome) 312**

**Blast2 Results -**

**OFC OLC RFC RLC**

**662 954 12 302**

**788 821 2 35**

**Ortholog Repeat Masker annotation**

**SW perc perc perc query position in query matching repeat position in repeat**

**score div. del. ins. sequence begin end (left) repeat class/family begin end (left) ID**

**2320 9.6 0.0 1.0 AluYa5_4_245 651 954 (0) + AluSx SINE/Alu 1 301 (11) 1**

**__________________________________________________________________________________**

**184. AluYa5_4_256c**

**Ortholog annotation INDEL_PTS Length 2686 nscore 18.21 NPOSITIONS 2196 2684 ;**

**Repeat length (main genome) 309**

**Blast2 Results -**

**OFC OLC RFC RLC**

**1 308 1 309**

**Ortholog Repeat Masker annotation**

**SW perc perc perc query position in query matching repeat position in repeat**

**score div. del. ins. sequence begin end (left) repeat class/family begin end (left) ID**

**2172 12.4 0.0 0.7 AluYa5_4_256c 1 309 (2377) + AluSg SINE/Alu 1 307 (3) 1**

**__________________________________________________________________________________**

**185. AluYa5_4_25c**

**Ortholog annotation INDEL_CAN Length 423 nscore 0.00 NPOSITIONS NA**

**Repeat length (main genome) 212**

**Blast2 Results -**

**OFC OLC RFC RLC**

**156 357 11 212**

**Ortholog Repeat Masker annotation**

**SW perc perc perc query position in query matching repeat position in repeat**

**score div. del. ins. sequence begin end (left) repeat class/family begin end (left) ID**

**392 17.1 2.7 7.1 AluYa5_4_25c 1 144 (279) + L1P4a LINE/L1 2473 2634 (4234) 1**

**1700 14.2 0.0 0.4 AluYa5_4_25c 146 392 (31) + AluSc SINE/Alu 1 246 (63) 2**

**198 7.1 0.0 0.0 AluYa5_4_25c 396 423 (0) + (A)n Simple_repeat 1 28 (0) 3**

**__________________________________________________________________________________**

**186. AluYa5_4_40c**

**Ortholog annotation INDEL_CAN Length 314 nscore 0.00 NPOSITIONS NA**

**Repeat length (main genome) 308**

**Blast2 Results -**

**OFC OLC RFC RLC**

**1 309 1 308**

**Ortholog Repeat Masker annotation**

**SW perc perc perc query position in query matching repeat position in repeat**

**score div. del. ins. sequence begin end (left) repeat class/family begin end (left) ID**

**2267 9.2 0.0 0.0 AluYa5_4_40c 1 314 (0) + AluY SINE/Alu 1 311 (0) 1**

**__________________________________________________________________________________**

**187. AluYa5_4_60**

**Ortholog annotation C_INTER_RMD_M_DISRUPTED Length 125 nscore 0.00 NPOSITIONS NA**

**Repeat length (main genome) 307**

**Blast2 Results -**

**OFC OLC RFC RLC**

**1 125 1 125**

**Ortholog Repeat Masker annotation**

**SW perc perc perc query position in query matching repeat position in repeat**

**score div. del. ins. sequence begin end (left) repeat class/family begin end (left) ID**

**962 12.0 0.0 0.0 AluYa5_4_60 1 125 (0) + AluSq/x SINE/Alu 1 125 (187) 1**

**__________________________________________________________________________________**

**188. AluYa5_4_7c**

**Ortholog annotation C_INTER_RMD_M_DISRUPTED Length 129 nscore 0.00 NPOSITIONS NA**

**Repeat length (main genome) 311**

**Blast2 Results -**

**OFC OLC RFC RLC**

**1 129 1 129**

**Ortholog Repeat Masker annotation**

**SW perc perc perc query position in query matching repeat position in repeat**

**score div. del. ins. sequence begin end (left) repeat class/family begin end (left) ID**

**950 10.8 0.0 0.0 AluYa5_4_7c 1 129 (0) + AluSq/x SINE/Alu 1 129 (183) 1**

**__________________________________________________________________________________**

**189. AluYa5_4_84**

**Ortholog annotation INDEL_CAN Length 364 nscore 0.00 NPOSITIONS NA**

**Repeat length (main genome) 305**

**Blast2 Results -**

**OFC OLC RFC RLC**

**no hits found**

**Ortholog Repeat Masker annotation**

**There were no repetitive sequences detected in /home/vipin/WHOLE_GENOME_CG/AluYa5_CHR/Chimp/CONFIRMATION/AluYa5_INDEL_SEQUENCES/AluYa5_4_84**

**__________________________________________________________________________________**

**190. AluYa5_4_90**

**Ortholog annotation INDEL_PTS Length 6879 nscore 0.00 NPOSITIONS NA**

**Repeat length (main genome) 305**

**Blast2 Results -**

**OFC OLC RFC RLC**

**1599 1881 12 292**

**Ortholog Repeat Masker annotation**

**SW perc perc perc query position in query matching repeat position in repeat**

**score div. del. ins. sequence begin end (left) repeat class/family begin end (left) ID**

**2215 10.7 0.0 0.0 AluYa5_4_90 1 281 (6598) C AluSx SINE/Alu (31) 281 1 1**

**1305 20.1 0.0 0.5 AluYa5_4_90 517 736 (6143) C MER58A DNA/MER1_type (5) 219 1 2**

**1968 13.1 0.3 0.3 AluYa5_4_90 1232 1515 (5364) C AluSq SINE/Alu (15) 298 15 3**

**2196 11.3 0.0 0.3 AluYa5_4_90 1588 1881 (4998) + AluSx SINE/Alu 1 293 (19) 4**

**2371 15.1 5.8 1.5 AluYa5_4_90 1943 2352 (4527) C MSTA LTR/MaLR (0) 428 1 5**

**739 17.7 7.5 15.0 AluYa5_4_90 2819 3044 (3835) C MER6B DNA/MER2_type (0) 210 2 6**

**1692 16.8 2.5 0.4 AluYa5_4_90 4444 4718 (2161) C AluJb SINE/Alu (31) 281 1 7**

**942 17.5 8.3 2.1 AluYa5_4_90 4721 4913 (1966) C AluJo SINE/Alu (17) 295 91 8**

**1819 13.6 6.4 0.3 AluYa5_4_90 4935 5229 (1650) C AluSq SINE/Alu (0) 313 1 9**

**1756 16.5 1.0 0.7 AluYa5_4_90 5232 5524 (1355) C AluSx SINE/Alu (18) 294 1 10**

**2493 6.9 0.3 0.0 AluYa5_4_90 5538 5842 (1037) C AluY SINE/Alu (5) 306 1 11**

**2617 5.8 0.0 0.0 AluYa5_4_90 5853 6160 (719) C AluY SINE/Alu (3) 308 1 12**

**__________________________________________________________________________________**

**191. AluYa5_5_10**

**Ortholog annotation INDEL_PTS Length 8352 nscore 7.23 NPOSITIONS 7749 8352 ;**

**Repeat length (main genome) 302**

**Blast2 Results -**

**OFC OLC RFC RLC**

**Ortholog Repeat Masker annotation**

**SW perc perc perc query position in query matching repeat position in repeat**

**score div. del. ins. sequence begin end (left) repeat class/family begin end (left) ID**

**436 7.0 0.0 0.0 AluYa5_5_10 1 57 (8295) C AluY SINE/Alu (242) 57 1 1**

**2124 26.9 2.3 2.5 AluYa5_5_10 64 519 (7833) + L1ME3A LINE/L1 5523 5979 (194) 2**

**189 0.0 0.0 0.0 AluYa5_5_10 520 540 (7812) + (TTG)n Simple_repeat 1 21 (0) 3**

**2105 11.2 0.0 0.0 AluYa5_5_10 541 818 (7534) C AluSc SINE/Alu (31) 278 1 4**

**2124 26.9 2.3 2.5 AluYa5_5_10 819 1008 (7344) + L1ME3A LINE/L1 5980 6169 (4) 2**

**716 24.6 9.3 6.1 AluYa5_5_10 1010 1162 (7190) + L1MC4 LINE/L1 1893 2051 (4164) 5**

**438 2.0 0.0 0.0 AluYa5_5_10 1163 1213 (7139) + (TA)n Simple_repeat 1 51 (0) 6**

**716 24.6 9.3 6.1 AluYa5_5_10 1214 1500 (6852) + L1MC4 LINE/L1 2052 2347 (3868) 5**

**654 18.9 0.6 5.7 AluYa5_5_10 1503 1659 (6693) C L1MC3 LINE/L1 (875) 6910 6762 7**

**2506 21.8 1.5 0.2 AluYa5_5_10 1695 2228 (6124) + L1MC3 LINE/L1 6994 7534 (251) 8**

**288 32.5 5.7 0.6 AluYa5_5_10 2240 2397 (5955) + L1MC4a LINE/L1 6663 6828 (1054) 9**

**310 33.9 5.9 1.6 AluYa5_5_10 4446 4631 (3721) + L2b LINE/L2 3181 3374 (1) 10**

**181 32.0 5.9 1.0 AluYa5_5_10 5022 5122 (3230) + MIR SINE/MIR 38 143 (119) 11**

**28 0.0 0.0 0.0 AluYa5_5_10 5744 5771 (2581) + AT_rich Low_complexity 1 28 (0) 12**

**699 16.9 45.8 0.0 AluYa5_5_10 6069 6301 (2051) C AluJb SINE/Alu (0) 312 1 13**

**24 0.0 0.0 0.0 AluYa5_5_10 6315 6338 (2014) + AT_rich Low_complexity 1 24 (0) 14**

**1925 15.2 0.0 3.6 AluYa5_5_10 6369 6676 (1676) C AluJb SINE/Alu (15) 297 1 15**

**23 3.3 0.0 0.0 AluYa5_5_10 7437 7466 (886) + AT_rich Low_complexity 1 30 (0) 16**

**__________________________________________________________________________________**

**192. AluYa5_5_100**

**Ortholog annotation INDEL_PTS Length 1579 nscore 1.27 NPOSITIONS 9 18 ; 1570 1579 ;**

**Repeat length (main genome) 233**

**Blast2 Results -**

**OFC OLC RFC RLC**

**243 473 1 232**

**1013 1147 85 220**

**Ortholog Repeat Masker annotation**

**SW perc perc perc query position in query matching repeat position in repeat**

**score div. del. ins. sequence begin end (left) repeat class/family begin end (left) ID**

**2459 7.8 0.3 0.0 AluYa5_5_100 173 481 (1098) + AluY SINE/Alu 1 310 (1) 1**

**1836 15.2 0.7 2.6 AluYa5_5_100 855 1159 (420) + AluJb SINE/Alu 5 303 (9) 2**

**1530 17.6 4.9 1.0 AluYa5_5_100 1255 1558 (21) C MLT1A0 LTR/MaLR (0) 365 50 3**

**__________________________________________________________________________________**

**193. AluYa5_5_144**

**Ortholog annotation INDEL_PTS Length 98 nscore 0.00 NPOSITIONS NA**

**Repeat length (main genome) 300**

**Blast2 Results -**

**OFC OLC RFC RLC**

**no hits found**

**Ortholog Repeat Masker annotation**

**There were no repetitive sequences detected in /home/vipin/WHOLE_GENOME_CG/AluYa5_CHR/Chimp/CONFIRMATION/AluYa5_INDEL_SEQUENCES/AluYa5_5_144**

**__________________________________________________________________________________**

**194. AluYa5_5_145c**

**Ortholog annotation C_INTER_RMD_M_DISRUPTED Length 139 nscore 0.00 NPOSITIONS NA**

**Repeat length (main genome) 147**

**Blast2 Results -**

**OFC OLC RFC RLC**

**1 139 1 139**

**Ortholog Repeat Masker annotation**

**SW perc perc perc query position in query matching repeat position in repeat**

**score div. del. ins. sequence begin end (left) repeat class/family begin end (left) ID**

**1172 5.0 0.0 0.0 AluYa5_5_145c 1 139 (0) + AluYa5 SINE/Alu 163 301 (9) 1**

**__________________________________________________________________________________**

**195. AluYa5_5_156c**

**Ortholog annotation C_INTER_RMD_M_DISRUPTED Length 132 nscore 0.00 NPOSITIONS NA**

**Repeat length (main genome) 307**

**Blast2 Results -**

**OFC OLC RFC RLC**

**7 132 7 129**

**Ortholog Repeat Masker annotation**

**SW perc perc perc query position in query matching repeat position in repeat**

**score div. del. ins. sequence begin end (left) repeat class/family begin end (left) ID**

**1105 5.4 0.0 2.3 AluYa5_5_156c 1 132 (0) + AluY SINE/Alu 1 129 (182) 1**

**__________________________________________________________________________________**

**196. AluYa5_5_175c**

**Ortholog annotation INDEL_CAN Length 4476 nscore 0.00 NPOSITIONS NA**

**Repeat length (main genome) 309**

**Blast2 Results -**

**OFC OLC RFC RLC**

**3244 3512 10 280**

**4354 4408 10 64**

**Ortholog Repeat Masker annotation**

**SW perc perc perc query position in query matching repeat position in repeat**

**score div. del. ins. sequence begin end (left) repeat class/family begin end (left) ID**

**2782 12.3 1.5 1.3 AluYa5_5_175c 14 227 (4249) + L1MA2 LINE/L1 3777 3991 (2155) 1**

**4238 6.5 1.0 3.9 AluYa5_5_175c 228 433 (4043) C LTR19B LTR/ERV1 (0) 580 380 2**

**1575 13.8 9.5 2.5 AluYa5_5_175c 434 707 (3769) C AluSx SINE/Alu (19) 293 1 3**

**4238 6.5 1.0 3.9 AluYa5_5_175c 708 1097 (3379) C LTR19B LTR/ERV1 (201) 379 1 2**

**2782 12.3 1.5 1.3 AluYa5_5_175c 1098 1345 (3131) + L1MA2 LINE/L1 3992 4240 (1906) 1**

**6239 12.3 3.0 2.3 AluYa5_5_175c 1525 1969 (2507) + L1MA2 LINE/L1 4240 4688 (1458) 1**

**1981 13.2 7.0 2.0 AluYa5_5_175c 1970 2324 (2152) + THE1C LTR/MaLR 1 373 (2) 4**

**6239 11.5 2.4 2.3 AluYa5_5_175c 2325 3234 (1242) + L1MA2 LINE/L1 4689 5598 (706) 1**

**2014 15.4 0.3 0.0 AluYa5_5_175c 3235 3539 (937) + AluSg SINE/Alu 1 306 (4) 5**

**5154 10.0 1.3 2.2 AluYa5_5_175c 3540 4042 (434) + L1MA2 LINE/L1 5599 6097 (207) 1**

**989 12.6 5.7 0.6 AluYa5_5_175c 4141 4315 (161) + L1MA2 LINE/L1 6121 6304 (0) 1**

**984 9.8 0.8 0.0 AluYa5_5_175c 4345 4476 (0) + AluSq/x SINE/Alu 1 133 (179) 6**

**__________________________________________________________________________________**

**197. AluYa5_5_184**

**Ortholog annotation INDEL_PTS Length 615 nscore 0.00 NPOSITIONS NA**

**Repeat length (main genome) 296**

**Blast2 Results -**

**OFC OLC RFC RLC**

**no hits found**

**Ortholog Repeat Masker annotation**

**SW perc perc perc query position in query matching repeat position in repeat**

**score div. del. ins. sequence begin end (left) repeat class/family begin end (left) ID**

**5111 3.3 0.3 2.3 AluYa5_5_184 1 615 (0) + SVA_D Other 612 1214 (172) 1**

**__________________________________________________________________________________**

**198. AluYa5_5_206**

**Ortholog annotation C_INTRA_RMD Length 122 nscore 0.00 NPOSITIONS NA**

**Repeat length (main genome) 303**

**Blast2 Results -**

**OFC OLC RFC RLC**

**1 122 1 122**

**12 42 149 179**

**Ortholog Repeat Masker annotation**

**SW perc perc perc query position in query matching repeat position in repeat**

**score div. del. ins. sequence begin end (left) repeat class/family begin end (left) ID**

**929 8.3 0.0 0.8 AluYa5_5_206 1 122 (0) + AluJb SINE/Alu 11 131 (181) 1**

**__________________________________________________________________________________**

**199. AluYa5_5_222c**

**Ortholog annotation C_INTER_RMD_M_DISRUPTED Length 120 nscore 0.00 NPOSITIONS NA**

**Repeat length (main genome) 312**

**Blast2 Results -**

**OFC OLC RFC RLC**

**1 120 1 120**

**Ortholog Repeat Masker annotation**

**SW perc perc perc query position in query matching repeat position in repeat**

**score div. del. ins. sequence begin end (left) repeat class/family begin end (left) ID**

**952 10.0 0.0 0.0 AluYa5_5_222c 1 120 (0) + AluSc SINE/Alu 1 120 (189) 1**

**__________________________________________________________________________________**

**200. AluYa5_5_233c**

**Ortholog annotation INDEL_CAN Length 311 nscore 0.00 NPOSITIONS NA**

**Repeat length (main genome) 302**

**Blast2 Results -**

**OFC OLC RFC RLC**

**5 299 5 299**

**Ortholog Repeat Masker annotation**

**SW perc perc perc query position in query matching repeat position in repeat**

**score div. del. ins. sequence begin end (left) repeat class/family begin end (left) ID**

**2816 2.3 0.0 0.0 AluYa5_5_233c 1 308 (3) + AluYa5 SINE/Alu 1 308 (2) 1**

**__________________________________________________________________________________**

**201. AluYa5_5_240**

**Ortholog annotation INDEL_PTS Length 825 nscore 0.00 NPOSITIONS NA**

**Repeat length (main genome) 303**

**Blast2 Results -**

**OFC OLC RFC RLC**

**1 258 1 258**

**Ortholog Repeat Masker annotation**

**SW perc perc perc query position in query matching repeat position in repeat**

**score div. del. ins. sequence begin end (left) repeat class/family begin end (left) ID**

**2293 9.9 0.3 0.3 AluYa5_5_240 1 295 (530) + AluSp SINE/Alu 1 295 (18) 1**

**574 28.8 9.6 1.4 AluYa5_5_240 296 503 (322) C MIRb SINE/MIR (17) 251 27 2**

**__________________________________________________________________________________**

**202. AluYa5_5_94c**

**Ortholog annotation INDEL_CAN Length 1193 nscore 0.00 NPOSITIONS NA**

**Repeat length (main genome) 157**

**Blast2 Results -**

**OFC OLC RFC RLC**

**1038 1193 1 156**

**Ortholog Repeat Masker annotation**

**SW perc perc perc query position in query matching repeat position in repeat**

**score div. del. ins. sequence begin end (left) repeat class/family begin end (left) ID**

**192 12.5 0.0 0.0 AluYa5_5_94c 1 32 (1161) + (A)n Simple_repeat 1 32 (0) 1**

**1508 17.0 2.5 13.7 AluYa5_5_94c 33 162 (1031) + L1M4 LINE/L1 4364 4479 (1667) 2**

**1869 17.1 1.0 0.7 AluYa5_5_94c 163 457 (736) + AluSx SINE/Alu 3 298 (14) 3**

**1508 17.0 2.5 13.7 AluYa5_5_94c 458 644 (549) + L1M4 LINE/L1 4480 4646 (1500) 2**

**719 20.9 0.0 0.0 AluYa5_5_94c 645 773 (420) + FLAM_C SINE/Alu 1 129 (4) 4**

**1508 17.0 2.5 13.7 AluYa5_5_94c 774 891 (302) + L1M4 LINE/L1 4647 4751 (1395) 2**

**2458 6.3 0.3 0.3 AluYa5_5_94c 892 1193 (0) + AluY SINE/Alu 1 302 (9) 5**

**__________________________________________________________________________________**

**203. AluYa5_6_142**

**Ortholog annotation INDEL_CAN Length 305 nscore 0.00 NPOSITIONS NA**

**Repeat length (main genome) 295**

**Blast2 Results -**

**OFC OLC RFC RLC**

**1 289 1 289**

**Ortholog Repeat Masker annotation**

**SW perc perc perc query position in query matching repeat position in repeat**

**score div. del. ins. sequence begin end (left) repeat class/family begin end (left) ID**

**2509 4.0 4.0 0.3 AluYa5_6_142 1 300 (5) + AluY SINE/Alu 1 311 (0) 1**

**__________________________________________________________________________________**

**204. AluYa5_6_167**

**Ortholog annotation INDEL_CAN Length 3071 nscore 0.00 NPOSITIONS NA**

**Repeat length (main genome) 300**

**Blast2 Results -**

**OFC OLC RFC RLC**

**935 1084 1 151**

**1088 1212 168 295**

**1570 1620 12 62**

**Ortholog Repeat Masker annotation**

**SW perc perc perc query position in query matching repeat position in repeat**

**score div. del. ins. sequence begin end (left) repeat class/family begin end (left) ID**

**1110 16.5 1.5 4.4 AluYa5_6_167 5 207 (2864) C AluJo SINE/Alu (115) 197 1 1**

**896 24.3 1.4 0.0 AluYa5_6_167 263 480 (2591) + HAL1 LINE/L1 1638 1858 (649) 2**

**503 25.4 0.0 2.5 AluYa5_6_167 505 625 (2446) C MLT1I LTR/MaLR (0) 411 294 3**

**2053 9.9 5.1 0.0 AluYa5_6_167 934 1227 (1844) + AluSc SINE/Alu 1 309 (0) 4**

**410 31.1 14.2 0.0 AluYa5_6_167 1281 1558 (1513) + L1M5 LINE/L1 4589 4906 (1240) 5**

**817 15.2 0.0 1.5 AluYa5_6_167 1559 1692 (1379) + FLAM_C SINE/Alu 2 133 (0) 6**

**365 30.1 6.2 6.6 AluYa5_6_167 1693 1993 (1078) + L1M5 LINE/L1 4884 5183 (963) 5**

**1735 22.9 6.1 2.6 AluYa5_6_167 2005 2497 (574) + L1M5 LINE/L1 4791 5300 (846) 7**

**3601 9.5 7.2 0.0 AluYa5_6_167 2538 3064 (7) + L1PA13 LINE/L1 5588 6152 (11) 8**

**__________________________________________________________________________________**

**205. AluYa5_6_17c**

**Ortholog annotation C_INTRA_RMD Length 130 nscore 0.00 NPOSITIONS NA**

**Repeat length (main genome) 317**

**Blast2 Results -**

**OFC OLC RFC RLC**

**1 122 1 122**

**Ortholog Repeat Masker annotation**

**SW perc perc perc query position in query matching repeat position in repeat**

**score div. del. ins. sequence begin end (left) repeat class/family begin end (left) ID**

**979 10.8 0.8 0.0 AluYa5_6_17c 1 130 (0) + AluSc SINE/Alu 1 131 (178) 1**

**__________________________________________________________________________________**

**206. AluYa5_6_205**

**Ortholog annotation INDEL_CAN Length 159 nscore 0.00 NPOSITIONS NA**

**Repeat length (main genome) 303**

**Blast2 Results -**

**OFC OLC RFC RLC**

**no hits found**

**Ortholog Repeat Masker annotation**

**There were no repetitive sequences detected in /home/vipin/WHOLE_GENOME_CG/AluYa5_CHR/Chimp/CONFIRMATION/AluYa5_INDEL_SEQUENCES/AluYa5_6_205**

**__________________________________________________________________________________**

**207. AluYa5_6_210c**

**Ortholog annotation C_DISRUPTED_M_INTER_RMD Length 3193 nscore 0.00 NPOSITIONS NA**

**Repeat length (main genome) 305**

**Blast2 Results -**

**OFC OLC RFC RLC**

**1 308 1 304**

**2249 2550 11 305**

**3040 3068 158 186**

**3128 3192 241 304**

**Ortholog Repeat Masker annotation**

**SW perc perc perc query position in query matching repeat position in repeat**

**score div. del. ins. sequence begin end (left) repeat class/family begin end (left) ID**

**2573 6.8 0.0 0.3 AluYa5_6_210c 1 308 (2885) + AluSc SINE/Alu 1 307 (2) 1**

**553 29.5 15.3 5.0 AluYa5_6_210c 851 1071 (2122) + L2a LINE/L2 2615 2859 (560) 2**

**2315 10.2 0.7 0.3 AluYa5_6_210c 1072 1376 (1817) C AluSx SINE/Alu (6) 306 1 3**

**553 30.5 16.8 3.8 AluYa5_6_210c 1377 1876 (1317) + L2a LINE/L2 2860 3426 (0) 2**

**2450 8.4 0.0 0.6 AluYa5_6_210c 2239 2551 (642) + AluY SINE/Alu 1 311 (0) 4**

**2312 9.1 1.7 0.0 AluYa5_6_210c 2573 2869 (324) C AluSc SINE/Alu (7) 302 1 5**

**1965 12.7 0.0 0.4 AluYa5_6_210c 2925 3192 (1) + AluJb SINE/Alu 44 310 (2) 6**

**__________________________________________________________________________________**

**208. AluYa5_6_226c**

**Ortholog annotation C_INTER_RMD_M_DISRUPTED Length 129 nscore 0.00 NPOSITIONS NA**

**Repeat length (main genome) 312**

**Blast2 Results -**

**OFC OLC RFC RLC**

**1 129 1 129**

**Ortholog Repeat Masker annotation**

**SW perc perc perc query position in query matching repeat position in repeat**

**score div. del. ins. sequence begin end (left) repeat class/family begin end (left) ID**

**1059 9.3 0.0 0.0 AluYa5_6_226c 1 129 (0) + AluSq/x SINE/Alu 1 129 (183) 1**

**__________________________________________________________________________________**

**209. AluYa5_6_245**

**Ortholog annotation INDEL_CAN Length 778 nscore 0.00 NPOSITIONS NA**

**Repeat length (main genome) 336**

**Blast2 Results -**

**OFC OLC RFC RLC**

**no hits found**

**Ortholog Repeat Masker annotation**

**There were no repetitive sequences detected in /home/vipin/WHOLE_GENOME_CG/AluYa5_CHR/Chimp/CONFIRMATION/AluYa5_INDEL_SEQUENCES/AluYa5_6_245**

**__________________________________________________________________________________**

**210. AluYa5_6_27c**

**Ortholog annotation C_INTER_RMD_M_DISRUPTED Length 352 nscore 0.00 NPOSITIONS NA**

**Repeat length (main genome) 297**

**Blast2 Results -**

**OFC OLC RFC RLC**

**1 292 1 293**

**Ortholog Repeat Masker annotation**

**SW perc perc perc query position in query matching repeat position in repeat**

**score div. del. ins. sequence begin end (left) repeat class/family begin end (left) ID**

**2287 6.8 1.4 0.7 AluYa5_6_27c 1 297 (55) + AluYa5 SINE/Alu 1 299 (11) 1**

**333 0.0 0.0 0.0 AluYa5_6_27c 314 350 (2) + (T)n Simple_repeat 1 37 (0) 2**

**__________________________________________________________________________________**

**211. AluYa5_6_41c**

**Ortholog annotation INDEL_CAN Length 5529 nscore 0.00 NPOSITIONS NA**

**Repeat length (main genome) 301**

**Blast2 Results -**

**OFC OLC RFC RLC**

**1 43 1 43**

**151 281 155 284**

**1287 1317 12 42**

**1353 1394 240 281**

**1445 1736 12 301**

**1779 2064 14 301**

**4281 4566 11 295**

**Ortholog Repeat Masker annotation**

**SW perc perc perc query position in query matching repeat position in repeat**

**score div. del. ins. sequence begin end (left) repeat class/family begin end (left) ID**

**1692 17.9 1.8 0.4 AluYa5_6_41c 1 281 (5248) + AluJb SINE/Alu 1 285 (27) 1**

**21 0.0 0.0 0.0 AluYa5_6_41c 282 302 (5227) + AT_rich Low_complexity 1 21 (0) 2**

**1608 19.5 5.2 0.9 AluYa5_6_41c 778 1267 (4262) + L1MCa LINE/L1 1625 2165 (4247) 3**

**616 22.1 0.0 2.3 AluYa5_6_41c 1276 1405 (4124) + FLAM_C SINE/Alu 1 127 (6) 4**

**2411 7.7 0.3 0.6 AluYa5_6_41c 1435 1746 (3783) + AluSx SINE/Alu 2 312 (0) 5**

**2203 10.6 0.7 0.0 AluYa5_6_41c 1766 2068 (3461) + AluY SINE/Alu 1 305 (6) 6**

**216 0.0 0.0 0.0 AluYa5_6_41c 2070 2093 (3436) + (GA)n Simple_repeat 1 24 (0) 7**

**14962 15.5 3.2 0.6 AluYa5_6_41c 2094 4277 (1252) + L1MCa LINE/L1 2705 4945 (1386) 3**

**2212 11.1 0.0 0.0 AluYa5_6_41c 4278 4574 (955) + AluSx SINE/Alu 8 304 (8) 8**

**14962 15.4 3.8 0.6 AluYa5_6_41c 4575 5321 (208) + L1MCa LINE/L1 4946 5708 (821) 3**

**566 31.7 1.6 1.6 AluYa5_6_41c 5329 5514 (15) C MLT1G1 LTR/MaLR (314) 265 80 9**

**__________________________________________________________________________________**

**212. AluYa5_6_49c**

**Ortholog annotation INDEL_CAN Length 3321 nscore 10.69 NPOSITIONS 324 678 ;**

**Repeat length (main genome) 284**

**Blast2 Results -**

**OFC OLC RFC RLC**

**3038 3321 1 284**

**Ortholog Repeat Masker annotation**

**SW perc perc perc query position in query matching repeat position in repeat**

**score div. del. ins. sequence begin end (left) repeat class/family begin end (left) ID**

**2017 7.5 4.3 1.2 AluYa5_6_49c 1 322 (2999) C L1PBa LINE/L1 (4915) 1948 1617 1**

**2958 10.0 2.9 0.2 AluYa5_6_49c 783 1263 (2058) C L1PBa LINE/L1 (5076) 1787 1294 1**

**3742 15.6 5.7 1.7 AluYa5_6_49c 1265 2085 (1236) C L1PBa LINE/L1 (5731) 1131 1 1**

**452 20.4 1.1 0.0 AluYa5_6_49c 2152 2244 (1077) + MLT1D LTR/MaLR 1 94 (411) 2**

**228 11.1 0.0 0.0 AluYa5_6_49c 2689 2724 (597) + (TTA)n Simple_repeat 2 37 (0) 3**

**1653 16.7 0.7 2.8 AluYa5_6_49c 2725 3013 (308) C AluJb SINE/Alu (29) 283 1 4**

**2369 7.0 0.0 0.0 AluYa5_6_49c 3038 3321 (0) + AluY SINE/Alu 1 284 (27) 5**

**__________________________________________________________________________________**

**213. AluYa5_6_50c**

**Ortholog annotation INDEL_PTS Length 833 nscore 1.20 NPOSITIONS 251 260 ;**

**Repeat length (main genome) 182**

**Blast2 Results -**

**OFC OLC RFC RLC**

**678 751 51 124**

**Ortholog Repeat Masker annotation**

**SW perc perc perc query position in query matching repeat position in repeat**

**score div. del. ins. sequence begin end (left) repeat class/family begin end (left) ID**

**621 23.2 4.9 0.7 AluYa5_6_50c 107 249 (584) + AluSc SINE/Alu 2 150 (159) 1**

**499 20.8 0.0 0.0 AluYa5_6_50c 269 369 (464) C AluJ/FLAM SINE/Alu (210) 102 2 2**

**1673 14.6 0.0 2.5 AluYa5_6_50c 538 812 (21) + AluSx SINE/Alu 23 290 (22) 3**

**__________________________________________________________________________________**

**214. AluYa5_6_54**

**Ortholog annotation INDEL_CAN Length 1520 nscore 0.00 NPOSITIONS NA**

**Repeat length (main genome) 273**

**Blast2 Results -**

**OFC OLC RFC RLC**

**80 124 200 244**

**1389 1520 141 272**

**Ortholog Repeat Masker annotation**

**SW perc perc perc query position in query matching repeat position in repeat**

**score div. del. ins. sequence begin end (left) repeat class/family begin end (left) ID**

**1162 11.6 0.0 0.6 AluYa5_6_54 1 165 (1355) + AluSg/x SINE/Alu 137 300 (12) 1**

**469 22.3 1.9 0.0 AluYa5_6_54 587 721 (799) + MIRb SINE/MIR 53 234 (34) 2**

**1838 16.7 0.3 0.3 AluYa5_6_54 1233 1520 (0) + AluJo SINE/Alu 1 288 (24) 3**

**__________________________________________________________________________________**

**215. AluYa5_6_55**

**Ortholog annotation C_INTRA_RMD Length 139 nscore 0.00 NPOSITIONS NA**

**Repeat length (main genome) 304**

**Blast2 Results -**

**OFC OLC RFC RLC**

**1 132 1 132**

**Ortholog Repeat Masker annotation**

**SW perc perc perc query position in query matching repeat position in repeat**

**score div. del. ins. sequence begin end (left) repeat class/family begin end (left) ID**

**1068 9.1 0.0 0.0 AluYa5_6_55 1 132 (7) + AluY SINE/Alu 1 132 (179) 1**

**__________________________________________________________________________________**

**216. AluYa5_6_61c**

**Ortholog annotation INDEL_PTS Length 2766 nscore 18.33 NPOSITIONS 2260 2766 ;**

**Repeat length (main genome) 304**

**Blast2 Results -**

**OFC OLC RFC RLC**

**11 307 11 304**

**976 1018 11 53**

**1050 1290 65 304**

**Ortholog Repeat Masker annotation**

**SW perc perc perc query position in query matching repeat position in repeat**

**score div. del. ins. sequence begin end (left) repeat class/family begin end (left) ID**

**2448 7.8 0.0 0.3 AluYa5_6_61c 1 308 (2458) + AluSq SINE/Alu 1 307 (6) 1**

**4557 16.4 1.4 4.5 AluYa5_6_61c 309 966 (1800) + L1MA9 LINE/L1 5002 5639 (529) 2**

**2115 7.5 0.0 6.3 AluYa5_6_61c 967 1292 (1474) + AluSq SINE/Alu 2 313 (0) 3**

**4557 16.9 2.2 4.8 AluYa5_6_61c 1293 1774 (992) + L1MA9 LINE/L1 5614 6085 (227) 2**

**1825 15.0 0.3 3.6 AluYa5_6_61c 1775 2078 (688) C AluJb SINE/Alu (18) 294 1 4**

**4557 16.9 2.2 4.8 AluYa5_6_61c 2079 2229 (537) + L1MA9 LINE/L1 6086 6231 (81) 2**

**__________________________________________________________________________________**

**217. AluYa5_7_10c**

**Ortholog annotation INDEL_CAN Length 1005 nscore 0.00 NPOSITIONS NA**

**Repeat length (main genome) 307**

**Blast2 Results -**

**OFC OLC RFC RLC**

**5 194 5 194**

**240 294 238 292**

**635 774 153 292**

**Ortholog Repeat Masker annotation**

**SW perc perc perc query position in query matching repeat position in repeat**

**score div. del. ins. sequence begin end (left) repeat class/family begin end (left) ID**

**1956 10.8 0.7 2.4 AluYa5_7_10c 1 294 (711) + AluSc SINE/Alu 1 289 (20) 1**

**1517 19.1 2.0 4.6 AluYa5_7_10c 473 774 (231) + AluJb SINE/Alu 1 294 (18) 2**

**243 25.7 7.2 5.3 AluYa5_7_10c 783 934 (71) + MIR3 SINE/MIR 2 156 (52) 3**

**__________________________________________________________________________________**

**218. AluYa5_7_114c**

**Ortholog annotation INDEL_PTS Length 8195 nscore 3.17 NPOSITIONS 6394 6403 ; 6460 6460 ; 6462 6463 ; 6473 6473 ; 6475 6475 ; 6502 6502 ; 6515 6515 ; 7279 7521 ;**

**Repeat length (main genome) 311**

**Blast2 Results -**

**OFC OLC RFC RLC**

**319 378 5 64**

**428 626 99 298**

**Ortholog Repeat Masker annotation**

**SW perc perc perc query position in query matching repeat position in repeat**

**score div. del. ins. sequence begin end (left) repeat class/family begin end (left) ID**

**2142 9.7 0.0 4.5 AluYa5_7_114c 315 626 (7569) + AluSx SINE/Alu 1 298 (14) 1**

**22459 8.1 1.2 0.2 AluYa5_7_114c 1233 5901 (2294) C L1PA7 LINE/L1 (4) 6150 1452 2**

**3905 6.9 0.0 0.4 AluYa5_7_114c 5902 6408 (1787) C L1PA4 LINE/L1 (0) 6155 5651 3**

**5303 12.6 1.8 0.3 AluYa5_7_114c 6409 7278 (917) C L1P2 LINE/L1 (2736) 3410 2528 4**

**4711 8.5 1.9 0.0 AluYa5_7_114c 7522 8194 (1) C L1P2 LINE/L1 (137) 2138 1453 4**

**__________________________________________________________________________________**

**219. AluYa5_7_148c**

**Ortholog annotation INDEL_CAN Length 755 nscore 1.46 NPOSITIONS 524 533 ; 599 599 ;**

**Repeat length (main genome) 294**

**Blast2 Results -**

**OFC OLC RFC RLC**

**Ortholog Repeat Masker annotation**

**SW perc perc perc query position in query matching repeat position in repeat**

**score div. del. ins. sequence begin end (left) repeat class/family begin end (left) ID**

**752 18.1 0.0 0.0 AluYa5_7_148c 537 669 (86) C AluSg/x SINE/Alu (7) 305 173 1**

**437 11.7 0.0 0.0 AluYa5_7_148c 670 729 (26) C AluS SINE/Alu (233) 69 10 2**

**__________________________________________________________________________________**

**220. AluYa5_7_187**

**Ortholog annotation INDEL_CAN Length 289 nscore 0.00 NPOSITIONS NA**

**Repeat length (main genome) 318**

**Blast2 Results -**

**OFC OLC RFC RLC**

**2 277 2 277**

**Ortholog Repeat Masker annotation**

**SW perc perc perc query position in query matching repeat position in repeat**

**score div. del. ins. sequence begin end (left) repeat class/family begin end (left) ID**

**2222 6.9 4.3 0.0 AluYa5_7_187 1 276 (13) + AluY SINE/Alu 1 288 (23) 1**

**__________________________________________________________________________________**

**221. AluYa5_7_194**

**Ortholog annotation INDEL_CAN Length 315 nscore 0.00 NPOSITIONS NA**

**Repeat length (main genome) 321**

**Blast2 Results -**

**OFC OLC RFC RLC**

**1 307 1 305**

**Ortholog Repeat Masker annotation**

**SW perc perc perc query position in query matching repeat position in repeat**

**score div. del. ins. sequence begin end (left) repeat class/family begin end (left) ID**

**2503 7.1 0.0 1.6 AluYa5_7_194 1 315 (0) + AluY SINE/Alu 1 310 (1) 1**

**__________________________________________________________________________________**

**222. AluYa5_7_22**

**Ortholog annotation INDEL_PTS Length 1741 nscore 0.00 NPOSITIONS NA**

**Repeat length (main genome) 313**

**Blast2 Results -**

**OFC OLC RFC RLC**

**no hits found**

**Ortholog Repeat Masker annotation**

**SW perc perc perc query position in query matching repeat position in repeat**

**score div. del. ins. sequence begin end (left) repeat class/family begin end (left) ID**

**11997 10.2 1.0 0.0 AluYa5_7_22 6 1741 (0) C L1P3 LINE/L1 (1796) 4350 2598 1**

**__________________________________________________________________________________**

**223. AluYa5_7_36c**

**Ortholog annotation INDEL_PTS Length 8247 nscore 10.69 NPOSITIONS 1 882 ;**

**Repeat length (main genome) 309**

**Blast2 Results -**

**OFC OLC RFC RLC**

**7263 7539 22 298**

**Ortholog Repeat Masker annotation**

**SW perc perc perc query position in query matching repeat position in repeat**

**score div. del. ins. sequence begin end (left) repeat class/family begin end (left) ID**

**2481 18.0 3.2 3.4 AluYa5_7_36c 925 1364 (6883) + MLT1D LTR/MaLR 6 446 (59) 1**

**1469 19.2 7.7 3.1 AluYa5_7_36c 1365 1713 (6534) C MLT1A0 LTR/MaLR (0) 365 1 2**

**2481 18.0 3.2 3.4 AluYa5_7_36c 1714 1773 (6474) + MLT1D LTR/MaLR 447 505 (0) 1**

**1776 16.3 0.3 1.9 AluYa5_7_36c 2481 2787 (5460) C AluJo SINE/Alu (10) 302 1 3**

**300 24.7 12.4 4.1 AluYa5_7_36c 2837 3030 (5217) C Tigger14a DNA/MER2_type (26) 298 89 4**

**32 8.1 0.0 2.6 AluYa5_7_36c 4245 4358 (3889) + AT_rich Low_complexity 1 111 (0) 5**

**219 33.0 3.2 0.0 AluYa5_7_36c 6226 6319 (1928) + L3b LINE/CR1 27 123 (359) 6**

**1990 9.3 7.6 0.0 AluYa5_7_36c 6728 7016 (1231) C AluSp SINE/Alu (0) 313 3 7**

**2122 11.6 0.0 0.3 AluYa5_7_36c 7261 7555 (692) + AluSq SINE/Alu 20 313 (0) 8**

**__________________________________________________________________________________**

**224. AluYa5_7_6c**

**Ortholog annotation C_INTER_RMD_M_DISRUPTED Length 308 nscore 0.00 NPOSITIONS NA**

**Repeat length (main genome) 293**

**Blast2 Results -**

**OFC OLC RFC RLC**

**1 293 1 293**

**Ortholog Repeat Masker annotation**

**SW perc perc perc query position in query matching repeat position in repeat**

**score div. del. ins. sequence begin end (left) repeat class/family begin end (left) ID**

**2220 7.7 0.3 0.7 AluYa5_7_6c 1 278 (30) + AluY SINE/Alu 1 279 (32) 1**

**189 0.0 0.0 0.0 AluYa5_7_6c 279 299 (9) + (CAAAA)n Simple_repeat 5 25 (0) 2**

**__________________________________________________________________________________**

**225. AluYa5_7_7**

**Ortholog annotation C_INTER_RMD_M_DISRUPTED Length 125 nscore 0.00 NPOSITIONS NA**

**Repeat length (main genome) 311**

**Blast2 Results -**

**OFC OLC RFC RLC**

**1 125 1 126**

**Ortholog Repeat Masker annotation**

**SW perc perc perc query position in query matching repeat position in repeat**

**score div. del. ins. sequence begin end (left) repeat class/family begin end (left) ID**

**1011 8.0 0.8 0.0 AluYa5_7_7 1 125 (0) + AluY SINE/Alu 1 126 (185) 1**

**__________________________________________________________________________________**

**226. AluYa5_7_70**

**Ortholog annotation INDEL_CAN Length 321 nscore 0.00 NPOSITIONS NA**

**Repeat length (main genome) 303**

**Blast2 Results -**

**OFC OLC RFC RLC**

**1 303 2 303**

**Ortholog Repeat Masker annotation**

**SW perc perc perc query position in query matching repeat position in repeat**

**score div. del. ins. sequence begin end (left) repeat class/family begin end (left) ID**

**2796 2.9 0.0 0.0 AluYa5_7_70 1 309 (12) + AluYa5 SINE/Alu 2 310 (0) 1**

**__________________________________________________________________________________**

**227. AluYa5_7_82**

**Ortholog annotation INDEL_CAN Length 568 nscore 0.00 NPOSITIONS NA**

**Repeat length (main genome) 307**

**Blast2 Results -**

**OFC OLC RFC RLC**

**76 374 6 306**

**Ortholog Repeat Masker annotation**

**SW perc perc perc query position in query matching repeat position in repeat**

**score div. del. ins. sequence begin end (left) repeat class/family begin end (left) ID**

**948 24.1 1.7 8.0 AluYa5_7_82 2 73 (495) + MER52-int LTR/ERV1 4058 4125 (2966) 1**

**2262 10.3 0.7 0.0 AluYa5_7_82 74 374 (194) + AluSg SINE/Alu 4 306 (4) 2**

**948 24.2 1.5 7.3 AluYa5_7_82 375 557 (11) + MER52-int LTR/ERV1 4126 4310 (4609) 1**

**__________________________________________________________________________________**

**228. AluYa5_7_87c**

**Ortholog annotation INDEL_CAN Length 3623 nscore 0.00 NPOSITIONS NA**

**Repeat length (main genome) 308**

**Blast2 Results -**

**OFC OLC RFC RLC**

**682 981 5 297**

**1045 1094 12 61**

**1326 1356 156 186**

**1414 1472 240 298**

**1572 1688 13 127**

**1700 1729 6 35**

**1773 1871 200 298**

**2228 2282 10 64**

**2317 2357 1 41**

**2420 2610 102 291**

**2605 2741 127 265**

**3000 3139 127 267**

**3197 3239 6 48**

**3328 3358 16 46**

**3445 3505 127 187**

**3567 3598 242 273**

**Ortholog Repeat Masker annotation**

**SW perc perc perc query position in query matching repeat position in repeat**

**score div. del. ins. sequence begin end (left) repeat class/family begin end (left) ID**

**2287 10.3 1.3 0.0 AluYa5_7_87c 2 303 (3320) C AluSx SINE/Alu (6) 306 1 1**

**2476 6.4 0.0 2.6 AluYa5_7_87c 677 981 (2642) + AluY SINE/Alu 1 297 (14) 2**

**829 14.3 0.0 0.0 AluYa5_7_87c 1043 1154 (2469) + FLAM_C SINE/Alu 10 121 (22) 3**

**1878 17.6 0.0 1.3 AluYa5_7_87c 1175 1479 (2144) + AluJo SINE/Alu 6 306 (6) 4**

**2294 11.3 0.0 4.0 AluYa5_7_87c 1560 1882 (1741) + AluSx SINE/Alu 1 310 (2) 5**

**1424 13.5 16.7 2.3 AluYa5_7_87c 2219 2316 (1307) + AluSx SINE/Alu 1 113 (199) 6**

**2295 9.2 0.3 0.7 AluYa5_7_87c 2317 2610 (1013) + AluSp SINE/Alu 1 293 (20) 7**

**1424 13.5 16.7 2.3 AluYa5_7_87c 2611 2769 (854) + AluSx SINE/Alu 114 295 (17) 6**

**1484 11.4 0.5 1.5 AluYa5_7_87c 2955 3159 (464) + AluJb SINE/Alu 86 288 (24) 8**

**26 0.0 0.0 0.0 AluYa5_7_87c 3160 3185 (438) + AT_rich Low_complexity 1 26 (0) 9**

**910 15.0 0.0 0.8 AluYa5_7_87c 3194 3327 (296) + AluJo SINE/Alu 3 135 (177) 10**

**1516 16.7 1.0 6.1 AluYa5_7_87c 3328 3620 (3) + AluJo SINE/Alu 16 293 (19) 11**

**__________________________________________________________________________________**

**229. AluYa5_7_93c**

**Ortholog annotation C_INTER_RMD_M_DISRUPTED Length 132 nscore 0.00 NPOSITIONS NA**

**Repeat length (main genome) 310**

**Blast2 Results -**

**OFC OLC RFC RLC**

**1 132 1 132**

**Ortholog Repeat Masker annotation**

**SW perc perc perc query position in query matching repeat position in repeat**

**score div. del. ins. sequence begin end (left) repeat class/family begin end (left) ID**

**1082 8.3 0.8 0.0 AluYa5_7_93c 1 132 (0) + AluSq/x SINE/Alu 1 133 (179) 1**

**__________________________________________________________________________________**

**230. AluYa5_7_98**

**Ortholog annotation C_INTER_RMD_M_DISRUPTED Length 118 nscore 0.00 NPOSITIONS NA**

**Repeat length (main genome) 295**

**Blast2 Results -**

**OFC OLC RFC RLC**

**1 118 1 118**

**Ortholog Repeat Masker annotation**

**SW perc perc perc query position in query matching repeat position in repeat**

**score div. del. ins. sequence begin end (left) repeat class/family begin end (left) ID**

**974 8.5 0.0 0.0 AluYa5_7_98 1 118 (0) + AluSp SINE/Alu 14 131 (182) 1**

**__________________________________________________________________________________**

**231. AluYa5_7_99c**

**Ortholog annotation INDEL_PAC Length 646 nscore 0.00 NPOSITIONS NA**

**Repeat length (main genome) 308**

**Blast2 Results -**

**OFC OLC RFC RLC**

**no hits found**

**Ortholog Repeat Masker annotation**

**SW perc perc perc query position in query matching repeat position in repeat**

**score div. del. ins. sequence begin end (left) repeat class/family begin end (left) ID**

**25 3.1 0.0 0.0 AluYa5_7_99c 1 32 (614) + AT_rich Low_complexity 1 32 (0) 1**

**756 16.1 0.6 0.0 AluYa5_7_99c 33 523 (123) + (TA)n Simple_repeat 1 493 (0) 2**

**22 0.0 0.0 0.0 AluYa5_7_99c 524 545 (101) + AT_rich Low_complexity 1 22 (0) 3**

**285 15.0 3.0 0.0 AluYa5_7_99c 546 645 (1) + (TA)n Simple_repeat 1 103 (0) 4**

**__________________________________________________________________________________**

**232. AluYa5_8_109c**

**Ortholog annotation INDEL_PTS Length 3312 nscore 0.30 NPOSITIONS 3296 3305 ;**

**Repeat length (main genome) 296**

**Blast2 Results -**

**OFC OLC RFC RLC**

**Ortholog Repeat Masker annotation**

**SW perc perc perc query position in query matching repeat position in repeat**

**score div. del. ins. sequence begin end (left) repeat class/family begin end (left) ID**

**644 29.5 2.4 1.8 AluYa5_8_109c 3 367 (2945) + L1M5 LINE/L1 4235 4620 (1526) 1**

**1693 26.8 7.6 3.9 AluYa5_8_109c 604 1642 (1670) + L1M5 LINE/L1 4632 5709 (2173) 1**

**21 0.0 0.0 0.0 AluYa5_8_109c 1697 1717 (1595) + AT_rich Low_complexity 1 21 (0) 2**

**187 31.0 6.9 0.0 AluYa5_8_109c 1803 1860 (1452) C L2b LINE/L2 (0) 3375 3314 3**

**23 3.3 0.0 0.0 AluYa5_8_109c 2913 2942 (370) + AT_rich Low_complexity 1 30 (0) 4**

**__________________________________________________________________________________**

**233. AluYa5_8_112c**

**Ortholog annotation INDEL_CAN Length 54 nscore 0.00 NPOSITIONS NA**

**Repeat length (main genome) 21**

**Blast2 Results -**

**OFC OLC RFC RLC**

**Ortholog Repeat Masker annotation**

**SW perc perc perc query position in query matching repeat position in repeat**

**score div. del. ins. sequence begin end (left) repeat class/family begin end (left) ID**

**189 0.0 0.0 0.0 AluYa5_8_112c 30 50 (4) + (CAAA)n Simple_repeat 4 24 (0) 1**

**__________________________________________________________________________________**

**234. AluYa5_8_118**

**Ortholog annotation INDEL_CAN Length 537 nscore 0.00 NPOSITIONS NA**

**Repeat length (main genome) 302**

**Blast2 Results -**

**OFC OLC RFC RLC**

**1 298 1 298**

**Ortholog Repeat Masker annotation**

**SW perc perc perc query position in query matching repeat position in repeat**

**score div. del. ins. sequence begin end (left) repeat class/family begin end (left) ID**

**2498 7.2 0.3 0.3 AluYa5_8_118 1 307 (230) + AluY SINE/Alu 1 307 (4) 1**

**__________________________________________________________________________________**

**235. AluYa5_8_123**

**Ortholog annotation INDEL_CAN Length 1635 nscore 0.00 NPOSITIONS NA**

**Repeat length (main genome) 305**

**Blast2 Results -**

**OFC OLC RFC RLC**

**597 630 11 44**

**1069 1366 6 303**

**Ortholog Repeat Masker annotation**

**SW perc perc perc query position in query matching repeat position in repeat**

**score div. del. ins. sequence begin end (left) repeat class/family begin end (left) ID**

**629 12.5 0.0 0.0 AluYa5_8_123 5 92 (1543) + LTR13A LTR/ERVK 879 966 (0) 1**

**955 21.5 1.0 1.0 AluYa5_8_123 103 304 (1331) C L1M5 LINE/L1 (621) 5558 5357 2**

**823 18.2 0.0 0.7 AluYa5_8_123 587 730 (905) + FLAM_C SINE/Alu 1 143 (0) 3**

**2531 5.0 0.3 0.0 AluYa5_8_123 1065 1366 (269) + AluY SINE/Alu 1 303 (8) 4**

**302 23.5 19.4 5.6 AluYa5_8_123 1449 1592 (43) + MIRb SINE/MIR 105 268 (0) 5**

**__________________________________________________________________________________**

**236. AluYa5_8_155**

**Ortholog annotation INDEL_PTS Length 2721 nscore 0.00 NPOSITIONS NA**

**Repeat length (main genome) 301**

**Blast2 Results -**

**OFC OLC RFC RLC**

**Ortholog Repeat Masker annotation**

**SW perc perc perc query position in query matching repeat position in repeat**

**score div. del. ins. sequence begin end (left) repeat class/family begin end (left) ID**

**813 27.5 0.0 3.4 AluYa5_8_155 47 283 (2438) + L2a LINE/L2 3198 3426 (0) 1**

**322 28.0 0.0 0.0 AluYa5_8_155 297 371 (2350) C MIRb SINE/MIR (90) 178 104 2**

**292 25.6 4.2 5.3 AluYa5_8_155 767 861 (1860) C L2b LINE/L2 (0) 3375 3282 3**

**251 22.4 21.9 0.0 AluYa5_8_155 1140 1249 (1472) C MIRb SINE/MIR (93) 175 39 4**

**261 31.2 16.1 0.0 AluYa5_8_155 1272 1364 (1357) C MIRb SINE/MIR (74) 194 87 5**

**33 0.0 0.0 0.0 AluYa5_8_155 1446 1478 (1243) + AT_rich Low_complexity 1 33 (0) 6**

**625 12.4 13.3 0.0 AluYa5_8_155 1479 1583 (1138) C FLAM_C SINE/Alu (14) 129 11 7**

**1831 20.0 9.9 1.2 AluYa5_8_155 1775 2500 (221) C Zaphod DNA/Tip100 (315) 3716 2928 8**

**__________________________________________________________________________________**

**237. AluYa5_8_23c**

**Ortholog annotation INDEL_CAN Length 982 nscore 9.37 NPOSITIONS 298 389 ;**

**Repeat length (main genome) 310**

**Blast2 Results -**

**OFC OLC RFC RLC**

**Ortholog Repeat Masker annotation**

**SW perc perc perc query position in query matching repeat position in repeat**

**score div. del. ins. sequence begin end (left) repeat class/family begin end (left) ID**

**281 11.8 2.0 0.0 AluYa5_8_23c 2 52 (930) + (GGAA)n Simple_repeat 2 53 (0) 1**

**190 9.5 0.0 4.5 AluYa5_8_23c 126 169 (813) + (GAGAA)n Simple_repeat 3 44 (0) 2**

**1143 1.4 0.0 2.0 AluYa5_8_23c 565 712 (270) + (GGAA)n Simple_repeat 3 147 (0) 3**

**__________________________________________________________________________________**

**238. AluYa5_8_29**

**Ortholog annotation INDEL_CAN Length 141 nscore 0.00 NPOSITIONS NA**

**Repeat length (main genome) 158**

**Blast2 Results -**

**OFC OLC RFC RLC**

**1 137 1 137**

**Ortholog Repeat Masker annotation**

**SW perc perc perc query position in query matching repeat position in repeat**

**score div. del. ins. sequence begin end (left) repeat class/family begin end (left) ID**

**1215 4.3 0.0 0.0 AluYa5_8_29 1 141 (0) + AluYa5 SINE/Alu 151 291 (19) 1**

**__________________________________________________________________________________**

**239. AluYa5_8_92**

**Ortholog annotation INDEL_PTS Length 78 nscore 0.00 NPOSITIONS NA**

**Repeat length (main genome) 298**

**Blast2 Results -**

**OFC OLC RFC RLC**

**no hits found**

**Ortholog Repeat Masker annotation**

**There were no repetitive sequences detected in /home/vipin/WHOLE_GENOME_CG/AluYa5_CHR/Chimp/CONFIRMATION/AluYa5_INDEL_SEQUENCES/AluYa5_8_92**

**__________________________________________________________________________________**

**240. AluYa5_9_112**

**Ortholog annotation C_INTER_RMD_M_DISRUPTED Length 126 nscore 0.00 NPOSITIONS NA**

**Repeat length (main genome) 315**

**Blast2 Results -**

**OFC OLC RFC RLC**

**1 126 1 126**

**Ortholog Repeat Masker annotation**

**SW perc perc perc query position in query matching repeat position in repeat**

**score div. del. ins. sequence begin end (left) repeat class/family begin end (left) ID**

**914 9.0 0.8 3.2 AluYa5_9_112 1 126 (0) + AluSq/x SINE/Alu 1 123 (189) 1**

**__________________________________________________________________________________**

**241. AluYa5_9_11c**

**Ortholog annotation INDEL_CAN Length 300 nscore 0.00 NPOSITIONS NA**

**Repeat length (main genome) 308**

**Blast2 Results -**

**OFC OLC RFC RLC**

**1 300 1 300**

**Ortholog Repeat Masker annotation**

**SW perc perc perc query position in query matching repeat position in repeat**

**score div. del. ins. sequence begin end (left) repeat class/family begin end (left) ID**

**2492 7.3 0.0 0.0 AluYa5_9_11c 1 300 (0) + AluY SINE/Alu 1 300 (11) 1**

**__________________________________________________________________________________**

**242. AluYa5_9_12c**

**Ortholog annotation INDEL_CAN Length 947 nscore 0.00 NPOSITIONS NA**

**Repeat length (main genome) 310**

**Blast2 Results -**

**OFC OLC RFC RLC**

**no hits found**

**Ortholog Repeat Masker annotation**

**SW perc perc perc query position in query matching repeat position in repeat**

**score div. del. ins. sequence begin end (left) repeat class/family begin end (left) ID**

**5991 9.5 4.6 2.3 AluYa5_9_12c 3 946 (1) + L1P4 LINE/L1 4352 5316 (830) 1**

**__________________________________________________________________________________**

**243. AluYa5_9_130c**

**Ortholog annotation INDEL_PTS Length 257 nscore 0.00 NPOSITIONS NA**

**Repeat length (main genome) 310**

**Blast2 Results -**

**OFC OLC RFC RLC**

**no hits found**

**Ortholog Repeat Masker annotation**

**SW perc perc perc query position in query matching repeat position in repeat**

**score div. del. ins. sequence begin end (left) repeat class/family begin end (left) ID**

**1499 6.3 0.0 0.0 AluYa5_9_130c 1 190 (67) C L1PA10 LINE/L1 (269) 5899 5710 1**

**32 0.0 0.0 0.0 AluYa5_9_130c 198 229 (28) + AT_rich Low_complexity 1 32 (0) 2**

**__________________________________________________________________________________**

**244. AluYa5_9_171c**

**Ortholog annotation C_INTER_RMD_M_DISRUPTED Length 102 nscore 0.00 NPOSITIONS NA**

**Repeat length (main genome) 302**

**Blast2 Results -**

**OFC OLC RFC RLC**

**1 98 201 298**

**Ortholog Repeat Masker annotation**

**SW perc perc perc query position in query matching repeat position in repeat**

**score div. del. ins. sequence begin end (left) repeat class/family begin end (left) ID**

**698 10.9 0.0 0.0 AluYa5_9_171c 1 101 (1) + AluSg/x SINE/Alu 202 302 (10) 1**

**__________________________________________________________________________________**

**245. AluYa5_9_172c**

**Ortholog annotation C_INTER_RMD_M_DISRUPTED Length 120 nscore 0.00 NPOSITIONS NA**

**Repeat length (main genome) 314**

**Blast2 Results -**

**OFC OLC RFC RLC**

**1 118 1 118**

**Ortholog Repeat Masker annotation**

**SW perc perc perc query position in query matching repeat position in repeat**

**score div. del. ins. sequence begin end (left) repeat class/family begin end (left) ID**

**966 10.0 0.0 0.0 AluYa5_9_172c 1 120 (0) + AluSq/x SINE/Alu 1 120 (192) 1**

**__________________________________________________________________________________**

**246. AluYa5_9_175c**

**Ortholog annotation INDEL_CAN Length 80 nscore 0.00 NPOSITIONS NA**

**Repeat length (main genome) 299**

**Blast2 Results -**

**OFC OLC RFC RLC**

**1 78 17 95**

**Ortholog Repeat Masker annotation**

**SW perc perc perc query position in query matching repeat position in repeat**

**score div. del. ins. sequence begin end (left) repeat class/family begin end (left) ID**

**625 7.5 1.2 0.0 AluYa5_9_175c 1 80 (0) + AluY SINE/Alu 17 97 (214) 1**

**__________________________________________________________________________________**

**247. AluYa5_9_178c**

**Ortholog annotation INDEL_PTS Length 290 nscore 0.00 NPOSITIONS NA**

**Repeat length (main genome) 160**

**Blast2 Results -**

**OFC OLC RFC RLC**

**1 160 1 160**

**Ortholog Repeat Masker annotation**

**SW perc perc perc query position in query matching repeat position in repeat**

**score div. del. ins. sequence begin end (left) repeat class/family begin end (left) ID**

**1013 11.3 0.0 3.8 AluYa5_9_178c 1 157 (133) + Alu SINE/Alu 157 307 (3) 1**

**__________________________________________________________________________________**

**248. AluYa5_9_181**

**Ortholog annotation INDEL_CAN Length 3537 nscore 0.00 NPOSITIONS NA**

**Repeat length (main genome) 313**

**Blast2 Results -**

**OFC OLC RFC RLC**

**Ortholog Repeat Masker annotation**

**SW perc perc perc query position in query matching repeat position in repeat**

**score div. del. ins. sequence begin end (left) repeat class/family begin end (left) ID**

**7226 15.2 11.5 1.7 AluYa5_9_181 1 869 (2668) C L1MD LINE/L1 (2232) 3914 2959 1**

**1714 16.7 4.5 0.0 AluYa5_9_181 870 1035 (2502) C AluSx SINE/Alu (11) 301 127 2**

**186 7.1 0.0 0.0 AluYa5_9_181 1036 1063 (2474) + (TTTA)n Simple_repeat 2 29 (0) 3**

**1714 16.7 4.5 0.0 AluYa5_9_181 1064 1184 (2353) C AluSx SINE/Alu (186) 126 1 2**

**2132 11.6 1.0 0.0 AluYa5_9_181 1187 1479 (2058) C AluSx SINE/Alu (16) 296 1 4**

**7226 15.2 6.3 2.3 AluYa5_9_181 1480 2281 (1256) C L1MD LINE/L1 (3188) 2958 2122 1**

**659 13.5 2.9 0.0 AluYa5_9_181 2282 2385 (1152) C MLT1C LTR/MaLR (0) 467 361 5**

**384 29.9 6.5 4.8 AluYa5_9_181 2383 2568 (969) C LTR16E1 LTR/ERVL (105) 433 245 6 ***

**__________________________________________________________________________________**

**249. AluYa5_9_186**

**Ortholog annotation INDEL_PTS Length 2114 nscore 5.11 NPOSITIONS 1 98 ; 1452 1461 ;**

**Repeat length (main genome) 86**

**Blast2 Results -**

**OFC OLC RFC RLC**

**99 184 1 86**

**Ortholog Repeat Masker annotation**

**SW perc perc perc query position in query matching repeat position in repeat**

**score div. del. ins. sequence begin end (left) repeat class/family begin end (left) ID**

**712 1.2 0.0 0.0 AluYa5_9_186 99 184 (1930) + AluYa5/8 SINE/Alu 213 298 (12) 1**

**2906 10.2 1.1 0.2 AluYa5_9_186 188 639 (1475) + L1PB LINE/L1 2771 3226 (2920) 2**

**26 8.2 0.0 0.0 AluYa5_9_186 642 702 (1412) + AT_rich Low_complexity 1 61 (0) 3**

**3299 11.1 0.2 1.2 AluYa5_9_186 980 1465 (649) + L1PB LINE/L1 3215 3695 (2451) 2**

**2876 10.8 1.1 0.4 AluYa5_9_186 1473 1935 (179) + L1PB LINE/L1 2761 3226 (2920) 4**

**25 3.1 0.0 0.0 AluYa5_9_186 1938 1969 (145) + AT_rich Low_complexity 1 32 (0) 5**

**__________________________________________________________________________________**

**250. AluYa5_9_5**

**Ortholog annotation INDEL_CAN Length 295 nscore 0.00 NPOSITIONS NA**

**Repeat length (main genome) 311**

**Blast2 Results -**

**OFC OLC RFC RLC**

**1 295 1 296**

**Ortholog Repeat Masker annotation**

**SW perc perc perc query position in query matching repeat position in repeat**

**score div. del. ins. sequence begin end (left) repeat class/family begin end (left) ID**

**2518 5.1 0.0 0.0 AluYa5_9_5 1 295 (0) + AluY SINE/Alu 1 295 (16) 1**

**__________________________________________________________________________________**

**251. AluYa5_9_61**

**Ortholog annotation INDEL_PTS Length 138 nscore 0.00 NPOSITIONS NA**

**Repeat length (main genome) 311**

**Blast2 Results -**

**OFC OLC RFC RLC**

**no hits found**

**Ortholog Repeat Masker annotation**

**SW perc perc perc query position in query matching repeat position in repeat**

**score div. del. ins. sequence begin end (left) repeat class/family begin end (left) ID**

**619 10.9 9.4 0.0 AluYa5_9_61 1 138 (0) + L1M2 LINE/L1 3070 3220 (2923) 1**

**__________________________________________________________________________________**

**252. AluYa5_9_65**

**Ortholog annotation INDEL_PTS Length 138 nscore 0.00 NPOSITIONS NA**

**Repeat length (main genome) 311**

**Blast2 Results -**

**OFC OLC RFC RLC**

**no hits found**

**Ortholog Repeat Masker annotation**

**SW perc perc perc query position in query matching repeat position in repeat**

**score div. del. ins. sequence begin end (left) repeat class/family begin end (left) ID**

**619 10.9 9.4 0.0 AluYa5_9_65 1 138 (0) + L1M2 LINE/L1 3070 3220 (2923) 1**

**__________________________________________________________________________________**

**253. AluYa5_9_70**

**Ortholog annotation INDEL_PTS Length 134 nscore 0.00 NPOSITIONS NA**

**Repeat length (main genome) 305**

**Blast2 Results -**

**OFC OLC RFC RLC**

**no hits found**

**Ortholog Repeat Masker annotation**

**SW perc perc perc query position in query matching repeat position in repeat**

**score div. del. ins. sequence begin end (left) repeat class/family begin end (left) ID**

**596 11.2 9.7 0.0 AluYa5_9_70 1 134 (0) + L1M2 LINE/L1 3070 3216 (2927) 1**

**__________________________________________________________________________________**

**254. AluYa5_X_106c**

**Ortholog annotation INDEL_PTS Length 6561 nscore 0.15 NPOSITIONS 1368 1377 ;**

**Repeat length (main genome) 347**

**Blast2 Results -**

**OFC OLC RFC RLC**

**5864 6127 1 260**

**Ortholog Repeat Masker annotation**

**SW perc perc perc query position in query matching repeat position in repeat**

**score div. del. ins. sequence begin end (left) repeat class/family begin end (left) ID**

**11095 4.5 1.2 0.1 AluYa5_X_106c 1 1367 (5194) C L1P1 LINE/L1 (1583) 4563 3182 1**

**12002 4.9 1.9 0.2 AluYa5_X_106c 1556 3397 (3164) C L1P1 LINE/L1 (2416) 3730 1858 2**

**2279 8.7 0.7 2.0 AluYa5_X_106c 3398 3702 (2859) C AluSq SINE/Alu (11) 302 2 3**

**6748 4.0 0.6 0.8 AluYa5_X_106c 3703 4128 (2433) C L1P1 LINE/L1 (4298) 1857 1433 2**

**5444 5.9 0.0 0.0 AluYa5_X_106c 4128 4809 (1752) + L1P1 LINE/L1 746 1427 (4728) 2 ***

**695 19.2 2.6 0.0 AluYa5_X_106c 4803 4958 (1603) + L1ME3B LINE/L1 5652 5811 (362) 4 ***

**261 22.6 4.5 8.9 AluYa5_X_106c 4959 5070 (1491) + LTR40a LTR/ERVL 41 147 (372) 5**

**252 0.0 0.0 0.0 AluYa5_X_106c 5375 5402 (1159) + (TG)n Simple_repeat 2 29 (0) 6**

**1239 15.1 1.0 1.5 AluYa5_X_106c 5403 5604 (957) C AluJo SINE/Alu (27) 285 85 7**

**461 6.5 3.2 0.0 AluYa5_X_106c 5662 5723 (838) + LTR40b LTR/ERVL 394 457 (5) 8**

**492 22.8 5.7 0.8 AluYa5_X_106c 5730 5853 (708) + L1ME3B LINE/L1 5810 5939 (301) 4**

**2189 12.2 0.3 2.2 AluYa5_X_106c 5864 6182 (379) + AluSq SINE/Alu 1 313 (0) 9**

**953 15.7 1.3 0.0 AluYa5_X_106c 6260 6418 (143) C FRAM SINE/Alu (0) 176 16 10**

**257 9.1 0.0 0.0 AluYa5_X_106c 6517 6560 (1) C AluYc5 SINE/Alu (7) 292 249 11**

**__________________________________________________________________________________**

**255. AluYa5_X_109c**

**Ortholog annotation C_INTER_RMD_M_DISRUPTED Length 120 nscore 0.00 NPOSITIONS NA**

**Repeat length (main genome) 303**

**Blast2 Results -**

**OFC OLC RFC RLC**

**1 120 1 120**

**Ortholog Repeat Masker annotation**

**SW perc perc perc query position in query matching repeat position in repeat**

**score div. del. ins. sequence begin end (left) repeat class/family begin end (left) ID**

**1084 5.0 0.0 0.0 AluYa5_X_109c 1 120 (0) + AluY SINE/Alu 6 125 (186) 1**

**__________________________________________________________________________________**

**256. AluYa5_X_132c**

**Ortholog annotation INDEL_PTS Length 89 nscore 0.00 NPOSITIONS NA**

**Repeat length (main genome) 309**

**Blast2 Results -**

**OFC OLC RFC RLC**

**Ortholog Repeat Masker annotation**

**There were no repetitive sequences detected in /home/vipin/WHOLE_GENOME_CG/AluYa5_CHR/Chimp/CONFIRMATION/AluYa5_INDEL_SEQUENCES/AluYa5_X_132c**

**__________________________________________________________________________________**

**257. AluYa5_X_16c**

**Ortholog annotation INDEL_CAN Length 289 nscore 3.46 NPOSITIONS 1 10 ;**

**Repeat length (main genome) 181**

**Blast2 Results -**

**OFC OLC RFC RLC**

**109 286 1 178**

**Ortholog Repeat Masker annotation**

**SW perc perc perc query position in query matching repeat position in repeat**

**score div. del. ins. sequence begin end (left) repeat class/family begin end (left) ID**

**543 20.4 3.1 0.0 AluYa5_X_16c 11 108 (181) C AluJb SINE/Alu (111) 201 101 1**

**1403 8.8 0.0 0.0 AluYa5_X_16c 109 289 (0) + AluY SINE/Alu 130 310 (1) 2**

**__________________________________________________________________________________**

**258. AluYa5_X_19**

**Ortholog annotation INDEL_CAN Length 294 nscore 0.00 NPOSITIONS NA**

**Repeat length (main genome) 302**

**Blast2 Results -**

**OFC OLC RFC RLC**

**5 294 5 294**

**Ortholog Repeat Masker annotation**

**SW perc perc perc query position in query matching repeat position in repeat**

**score div. del. ins. sequence begin end (left) repeat class/family begin end (left) ID**

**2370 5.8 3.1 0.7 AluYa5_X_19 1 294 (0) + AluYa5 SINE/Alu 1 301 (9) 1**

**__________________________________________________________________________________**

**259. AluYa5_X_31**

**Ortholog annotation INDEL_CAN Length 125 nscore 0.00 NPOSITIONS NA**

**Repeat length (main genome) 308**

**Blast2 Results -**

**OFC OLC RFC RLC**

**no hits found**

**Ortholog Repeat Masker annotation**

**SW perc perc perc query position in query matching repeat position in repeat**

**score div. del. ins. sequence begin end (left) repeat class/family begin end (left) ID**

**201 25.0 0.0 0.0 AluYa5_X_31 11 70 (55) + (TA)n Simple_repeat 2 61 (0) 1**

**__________________________________________________________________________________**

**260. AluYa5_X_56c**

**Ortholog annotation INDEL_PTS Length 3016 nscore 0.33 NPOSITIONS 1705 1714 ;**

**Repeat length (main genome) 310**

**Blast2 Results -**

**OFC OLC RFC RLC**

**Ortholog Repeat Masker annotation**

**SW perc perc perc query position in query matching repeat position in repeat**

**score div. del. ins. sequence begin end (left) repeat class/family begin end (left) ID**

**9557 9.7 0.8 1.3 AluYa5_X_56c 1 141 (2875) + L1PB LINE/L1 2219 2359 (3787) 1**

**2295 9.3 0.3 0.0 AluYa5_X_56c 142 431 (2585) C AluSg SINE/Alu (19) 291 1 2**

**9557 9.7 0.8 1.3 AluYa5_X_56c 432 1716 (1300) + L1PB LINE/L1 2360 3638 (2508) 1**

**7875 8.8 0.7 0.7 AluYa5_X_56c 1715 2865 (151) + L1PB LINE/L1 2550 3700 (2446) 3 ***

**904 8.7 0.5 0.0 AluYa5_X_56c 2863 3007 (9) C L1PB LINE/L1 (2151) 3995 3839 4 ***

**__________________________________________________________________________________**

**261. AluYa5_X_64c**

**Ortholog annotation INDEL_CAN Length 223 nscore 4.48 NPOSITIONS 1 10 ;**

**Repeat length (main genome) 307**

**Blast2 Results -**

**OFC OLC RFC RLC**

**no hits found**

**Ortholog Repeat Masker annotation**

**SW perc perc perc query position in query matching repeat position in repeat**

**score div. del. ins. sequence begin end (left) repeat class/family begin end (left) ID**

**761 20.0 0.0 1.1 AluYa5_X_64c 37 213 (10) + L1PREC2 LINE/L1 2210 2384 (3762) 1**

**__________________________________________________________________________________**

**262. AluYa5_X_71c**

**Ortholog annotation INDEL_PTS Length 329 nscore 0.00 NPOSITIONS NA**

**Repeat length (main genome) 306**

**Blast2 Results -**

**OFC OLC RFC RLC**

**no hits found**

**Ortholog Repeat Masker annotation**

**SW perc perc perc query position in query matching repeat position in repeat**

**score div. del. ins. sequence begin end (left) repeat class/family begin end (left) ID**

**2459 7.3 0.0 0.0 AluYa5_X_71c 1 328 (1) C L1P1 LINE/L1 (805) 5341 5014 1**

**__________________________________________________________________________________**

**263. AluYa5_X_75**

**Ortholog annotation INDEL_CAN Length 1967 nscore 0.00 NPOSITIONS NA**

**Repeat length (main genome) 271**

**Blast2 Results -**

**OFC OLC RFC RLC**

**no hits found**

**Ortholog Repeat Masker annotation**

**SW perc perc perc query position in query matching repeat position in repeat**

**score div. del. ins. sequence begin end (left) repeat class/family begin end (left) ID**

**4418 22.1 3.6 1.6 AluYa5_X_75 13 1967 (0) + L1M1 LINE/L1 2785 4842 (2720) 1**

**__________________________________________________________________________________**

**264. AluYa5_X_90**

**Ortholog annotation C_INTER_RMD_M_DISRUPTED Length 124 nscore 0.00 NPOSITIONS NA**

**Repeat length (main genome) 306**

**Blast2 Results -**

**OFC OLC RFC RLC**

**1 124 1 124**

**Ortholog Repeat Masker annotation**

**SW perc perc perc query position in query matching repeat position in repeat**

**score div. del. ins. sequence begin end (left) repeat class/family begin end (left) ID**

**1077 5.7 0.0 0.0 AluYa5_X_90 1 124 (0) + AluSq/x SINE/Alu 1 124 (188) 1**

**__________________________________________________________________________________**

**265. AluYa5_X_96c**

**Ortholog annotation INDEL_PTS Length 89 nscore 0.00 NPOSITIONS NA**

**Repeat length (main genome) 310**

**Blast2 Results -**

**OFC OLC RFC RLC**

**no hits found**

**Ortholog Repeat Masker annotation**

**SW perc perc perc query position in query matching repeat position in repeat**

**score div. del. ins. sequence begin end (left) repeat class/family begin end (left) ID**

**607 8.2 0.0 0.0 AluYa5_X_96c 3 87 (2) C L1PREC2 LINE/L1 (2574) 3572 3488 1**

**__________________________________________________________________________________**

**266. AluYa5_Y_3**

**Ortholog annotation INDEL_CAN Length 227 nscore 0.00 NPOSITIONS NA**

**Repeat length (main genome) 212**

**Blast2 Results -**

**OFC OLC RFC RLC**

**1 202 1 202**

**Ortholog Repeat Masker annotation**

**SW perc perc perc query position in query matching repeat position in repeat**

**score div. del. ins. sequence begin end (left) repeat class/family begin end (left) ID**

**1497 6.8 0.5 1.6 AluYa5_Y_3 1 184 (43) + AluYa5 SINE/Alu 99 282 (28) 1**

**297 0.0 0.0 0.0 AluYa5_Y_3 185 217 (10) + (CAA)n Simple_repeat 1 33 (0) 2**

**__________________________________________________________________________________**

**267. AluYa5_Y_4c**

**Ortholog annotation INDEL_PTS Length 133 nscore 0.00 NPOSITIONS NA**

**Repeat length (main genome) 310**

**Blast2 Results -**

**OFC OLC RFC RLC**

**no hits found**

**Ortholog Repeat Masker annotation**

**There were no repetitive sequences detected in /home/vipin/WHOLE_GENOME_CG/AluYa5_CHR/Chimp/CONFIRMATION/AluYa5_INDEL_SEQUENCES/AluYa5_Y_4c**

**__________________________________________________________________________________**

**268. AluYa5_Y_63c**

**Ortholog annotation INDEL_CAN Length 112 nscore 0.00 NPOSITIONS NA**

**Repeat length (main genome) 303**

**Blast2 Results -**

**OFC OLC RFC RLC**

**no hits found**

**Ortholog Repeat Masker annotation**

**There were no repetitive sequences detected in /home/vipin/WHOLE_GENOME_CG/AluYa5_CHR/Chimp/CONFIRMATION/AluYa5_INDEL_SEQUENCES/AluYa5_Y_63c**

**__________________________________________________________________________________**

**Supplementary file 4 - AluYa5 comparison with Celera genome.**

**The file summarizes the blast2 result of the identified ortholog with the Alu repeat and also gives the repeat masker annotation of the identified orthologous locus, along with N details.**

**1. AluYa5_10_109c**

**Ortholog annotation INDEL_PTS Length 9558 nscore 4.79 NPOSITIONS 9101 9558 ;**

**Repeat length (main genome) 310**

**Blast2 Results -**

**OFC OLC RFC RLC**

**2150 2433 12 293**

**2459 2493 23 57**

**2565 2607 122 164**

**2755 2781 38 64**

**2960 3012 243 297**

**4729 4815 1 85**

**4839 4868 102 131**

**4913 5028 163 278**

**Ortholog Repeat Masker annotation**

**SW perc perc perc query position in query matching repeat position in repeat**

**score div. del. ins. sequence begin end (left) repeat class/family begin end (left) ID**

**964 20.6 2.0 6.3 AluYa5_10_109c 237 490 (9068) + MER96B DNA/hAT 165 407 (10) 1**

**765 15.7 0.0 0.0 AluYa5_10_109c 560 680 (8878) C FLAM_C SINE/Alu (21) 122 2 2**

**6224 13.5 1.4 0.1 AluYa5_10_109c 1138 2122 (7436) C L1MA4 LINE/L1 (3) 6297 5300 3**

**2064 12.0 0.0 0.3 AluYa5_10_109c 2141 2433 (7125) + AluSx SINE/Alu 3 294 (18) 4**

**1223 15.8 0.4 4.6 AluYa5_10_109c 2438 2676 (6882) + AluJb SINE/Alu 1 229 (83) 5**

**1751 15.7 1.6 0.7 AluYa5_10_109c 2718 3024 (6534) + AluJo SINE/Alu 1 310 (2) 6**

**2096 10.3 0.0 1.0 AluYa5_10_109c 3162 3455 (6103) C AluSx SINE/Alu (21) 291 1 7**

**252 23.8 6.1 2.4 AluYa5_10_109c 3534 3615 (5943) + L1M1 LINE/L1 5498 5582 (720) 8**

**283 33.3 5.6 4.3 AluYa5_10_109c 3684 3987 (5571) + L1ME3B LINE/L1 5633 5940 (300) 9**

**186 4.3 0.0 0.0 AluYa5_10_109c 4117 4139 (5419) + (GGGGA)n Simple_repeat 1 23 (0) 10**

**278 32.5 4.0 0.8 AluYa5_10_109c 4320 4443 (5115) C MER113 DNA/MER1_type (364) 157 30 11**

**1957 10.0 0.3 9.1 AluYa5_10_109c 4729 5069 (4489) + AluSx SINE/Alu 1 311 (1) 12**

**296 25.8 1.5 0.0 AluYa5_10_109c 6651 6716 (2842) + MER5B DNA/MER1_type 5 71 (107) 13**

**702 18.3 9.7 1.3 AluYa5_10_109c 7052 7206 (2352) C MIR SINE/MIR (81) 181 14 14**

**348 29.4 19.8 4.7 AluYa5_10_109c 7598 8111 (1447) + L2 LINE/L2 2477 3068 (351) 15**

**252 21.8 11.3 3.2 AluYa5_10_109c 8668 8781 (777) C L2a LINE/L2 (2) 3385 3257 16**

**2113 8.2 0.3 1.4 AluYa5_10_109c 8784 9086 (472) C AluSx SINE/Alu (0) 312 2 17**

**__________________________________________________________________________________**

**2. AluYa5_11_26**

**Ortholog annotation INDEL_PAC Length 552 nscore 3.62 NPOSITIONS 490 509 ;**

**Repeat length (main genome) 311**

**Blast2 Results -**

**OFC OLC RFC RLC**

**1 215 1 215**

**282 310 283 311**

**283 311 283 311**

**284 312 283 311**

**285 313 283 311**

**286 314 283 311**

**287 315 283 311**

**288 316 283 311**

**289 317 283 311**

**290 318 283 311**

**291 319 283 311**

**292 320 283 311**

**293 321 283 311**

**294 322 283 311**

**295 323 283 311**

**296 324 283 311**

**297 325 283 311**

**Ortholog Repeat Masker annotation**

**SW perc perc perc query position in query matching repeat position in repeat**

**score div. del. ins. sequence begin end (left) repeat class/family begin end (left) ID**

**2276 8.4 0.3 0.3 AluYa5_11_26 1 310 (242) + AluYa5 SINE/Alu 1 310 (0) 1**

**__________________________________________________________________________________**

**3. AluYa5_11_73**

**Ortholog annotation C_DISRUPTED_M_INTER_RMD Length 1277 nscore 3.13 NPOSITIONS 195 214 ; 1040 1059 ;**

**Repeat length (main genome) 279**

**Blast2 Results -**

**OFC OLC RFC RLC**

**1 194 1 194**

**331 362 1 32**

**1060 1277 61 278**

**Ortholog Repeat Masker annotation**

**SW perc perc perc query position in query matching repeat position in repeat**

**score div. del. ins. sequence begin end (left) repeat class/family begin end (left) ID**

**1838 0.5 0.0 0.0 AluYa5_11_73 1 194 (1083) + AluYa5 SINE/Alu 28 221 (89) 1**

**323 27.5 5.5 0.0 AluYa5_11_73 216 306 (971) C MLT1A1 LTR/MaLR (312) 96 1 2**

**582 20.8 0.6 11.2 AluYa5_11_73 329 642 (635) + AluYa5 SINE/Alu 26 306 (4) 3**

**232 17.9 2.6 0.0 AluYa5_11_73 681 758 (519) + (TAA)n Simple_repeat 3 82 (0) 4**

**283 22.2 0.0 0.0 AluYa5_11_73 819 872 (405) + MLT1F-int LTR/MaLR 1202 1255 (234) 5**

**2032 0.9 0.0 0.0 AluYa5_11_73 1060 1277 (0) + AluYa5 SINE/Alu 88 305 (5) 6**

**__________________________________________________________________________________**

**4. AluYa5_11_97c**

**Ortholog annotation INDEL_CAN Length 820 nscore 2.44 NPOSITIONS 288 307 ;**

**Repeat length (main genome) 305**

**Blast2 Results -**

**OFC OLC RFC RLC**

**1 287 1 286**

**Ortholog Repeat Masker annotation**

**SW perc perc perc query position in query matching repeat position in repeat**

**score div. del. ins. sequence begin end (left) repeat class/family begin end (left) ID**

**2651 1.1 0.0 0.3 AluYa5_11_97c 1 287 (533) + AluYa5 SINE/Alu 1 286 (24) 1**

**180 0.0 0.0 0.0 AluYa5_11_97c 748 767 (53) + (C)n Simple_repeat 1 20 (0) 2**

**216 0.0 0.0 0.0 AluYa5_11_97c 797 820 (0) + (A)n Simple_repeat 1 24 (0) 3**

**__________________________________________________________________________________**

**5. AluYa5_12_101c**

**Ortholog annotation C_INTER_RMD_M_DISRUPTED Length 136 nscore 14.71 NPOSITIONS 83 102 ;**

**Repeat length (main genome) 301**

**Blast2 Results -**

**OFC OLC RFC RLC**

**2 82 1 82**

**21 53 154 187**

**103 136 268 301**

**Ortholog Repeat Masker annotation**

**SW perc perc perc query position in query matching repeat position in repeat**

**score div. del. ins. sequence begin end (left) repeat class/family begin end (left) ID**

**733 20.6 0.8 1.5 AluYa5_12_101c 2 134 (2) + Alu SINE/Alu 1 132 (178) 1**

**__________________________________________________________________________________**

**6. AluYa5_12_169**

**Ortholog annotation C_DISRUPTED_M_INTER_RMD Length 728 nscore 2.75 NPOSITIONS 430 449 ;**

**Repeat length (main genome) 310**

**Blast2 Results -**

**OFC OLC RFC RLC**

**1 309 1 309**

**450 728 31 309**

**Ortholog Repeat Masker annotation**

**SW perc perc perc query position in query matching repeat position in repeat**

**score div. del. ins. sequence begin end (left) repeat class/family begin end (left) ID**

**2942 0.3 0.0 0.0 AluYa5_12_169 1 309 (419) + AluYa5 SINE/Alu 1 309 (1) 1**

**2646 0.4 0.0 0.0 AluYa5_12_169 450 728 (0) + AluYa5 SINE/Alu 31 309 (1) 2**

**__________________________________________________________________________________**

**7. AluYa5_13_113c**

**Ortholog annotation C_DISRUPTED_M_INTER_RMD Length 1013 nscore 1.97 NPOSITIONS 266 285 ;**

**Repeat length (main genome) 302**

**Blast2 Results -**

**OFC OLC RFC RLC**

**1 265 1 265**

**711 1013 1 302**

**Ortholog Repeat Masker annotation**

**SW perc perc perc query position in query matching repeat position in repeat**

**score div. del. ins. sequence begin end (left) repeat class/family begin end (left) ID**

**2529 7.3 0.0 0.0 AluYa5_13_113c 1 288 (725) + AluYa5 SINE/Alu 1 288 (22) 1**

**40 5.9 0.0 0.0 AluYa5_13_113c 642 709 (304) + AT_rich Low_complexity 1 68 (0) 2**

**2860 0.0 0.0 0.3 AluYa5_13_113c 711 1013 (0) + AluYa5 SINE/Alu 1 302 (8) 3**

**__________________________________________________________________________________**

**8. AluYa5_14_98c**

**Ortholog annotation INDEL_CAN Length 2959 nscore 0.00 NPOSITIONS NA**

**Repeat length (main genome) 233**

**Blast2 Results -**

**OFC OLC RFC RLC**

**1 233 1 233**

**1028 1082 11 65**

**1849 2066 12 230**

**Ortholog Repeat Masker annotation**

**SW perc perc perc query position in query matching repeat position in repeat**

**score div. del. ins. sequence begin end (left) repeat class/family begin end (left) ID**

**2801 1.0 0.0 0.0 AluYa5_14_98c 1 304 (2655) + AluYa5 SINE/Alu 1 304 (6) 1**

**180 0.0 0.0 0.0 AluYa5_14_98c 305 324 (2635) + (TAA)n Simple_repeat 2 21 (0) 2**

**305 10.4 0.0 0.0 AluYa5_14_98c 958 1005 (1954) C MER57E1 LTR/ERV1 (0) 411 364 3**

**716 19.6 4.7 1.2 AluYa5_14_98c 1020 1189 (1770) + FRAM SINE/Alu 1 176 (0) 4**

**774 24.1 18.6 2.6 AluYa5_14_98c 1192 1455 (1504) C MER57E1 LTR/ERV1 (62) 310 5 3**

**733 31.5 4.6 1.7 AluYa5_14_98c 1522 1837 (1122) + L1M5 LINE/L1 4067 4393 (1753) 5**

**2040 14.4 0.0 0.0 AluYa5_14_98c 1838 2136 (823) + AluSg SINE/Alu 1 299 (11) 6**

**733 31.5 4.6 1.7 AluYa5_14_98c 2137 2147 (812) + L1M5 LINE/L1 4394 4405 (1741) 5**

**1379 22.7 1.0 2.2 AluYa5_14_98c 2148 2458 (501) + AluJo SINE/Alu 3 309 (3) 7**

**733 31.5 4.6 1.7 AluYa5_14_98c 2459 2542 (417) + L1M5 LINE/L1 4406 4491 (1655) 5**

**236 15.0 23.3 0.0 AluYa5_14_98c 2722 2753 (206) + L1M5 LINE/L1 5642 5682 (491) 8**

**183 4.2 0.0 0.0 AluYa5_14_98c 2754 2777 (182) + (CAA)n Simple_repeat 2 25 (0) 9**

**236 15.0 23.3 0.0 AluYa5_14_98c 2778 2804 (155) + L1M5 LINE/L1 5683 5715 (458) 8**

**779 16.6 2.6 0.7 AluYa5_14_98c 2808 2959 (0) C L1M4 LINE/L1 (1571) 4575 4421 10**

**__________________________________________________________________________________**

**9. AluYa5_15_89**

**Ortholog annotation INDEL_PTS Length 3876 nscore 0.00 NPOSITIONS NA**

**Repeat length (main genome) 301**

**Blast2 Results -**

**OFC OLC RFC RLC**

**Ortholog Repeat Masker annotation**

**SW perc perc perc query position in query matching repeat position in repeat**

**score div. del. ins. sequence begin end (left) repeat class/family begin end (left) ID**

**1767 17.5 2.6 3.9 AluYa5_15_89 3 305 (3571) C L1M4c LINE/L1 (3584) 3422 3123 1**

**2296 10.5 0.3 0.3 AluYa5_15_89 306 611 (3265) C AluSx SINE/Alu (6) 306 1 2**

**1767 18.6 4.3 6.5 AluYa5_15_89 612 932 (2944) C L1M4c LINE/L1 (4006) 3328 2879 1**

**769 18.9 0.0 1.5 AluYa5_15_89 933 1066 (2810) C FLAM_C SINE/Alu (1) 132 1 3**

**456 20.4 6.5 16.3 AluYa5_15_89 1067 1120 (2756) C L1M4c LINE/L1 (4204) 3130 3082 1**

**2002 11.8 1.0 1.7 AluYa5_15_89 1121 1421 (2455) C AluSx SINE/Alu (13) 299 1 4**

**456 20.4 6.5 16.3 AluYa5_15_89 1422 1545 (2331) C L1M4c LINE/L1 (4253) 3081 2970 1**

**2201 10.7 0.3 0.0 AluYa5_15_89 1560 1850 (2026) C AluSp SINE/Alu (21) 292 1 5**

**892 16.7 15.6 6.4 AluYa5_15_89 1923 2217 (1659) C L1MA9 LINE/L1 (2) 6310 5989 6**

**3019 22.8 8.7 3.3 AluYa5_15_89 2223 3091 (785) C L1M4c LINE/L1 (4646) 2547 1590 1**

**2338 9.0 0.3 1.3 AluYa5_15_89 3092 3405 (471) C AluY SINE/Alu (0) 311 1 7**

**619 20.0 4.0 0.0 AluYa5_15_89 3411 3535 (341) C FLAM_C SINE/Alu (3) 130 1 8**

**3019 21.4 8.8 2.8 AluYa5_15_89 3536 3876 (0) C L1M4c LINE/L1 (5425) 1583 1221 1**

**__________________________________________________________________________________**

**10. AluYa5_16_26**

**Ortholog annotation C_INTER_RMD_M_DISRUPTED Length 161 nscore 0.00 NPOSITIONS NA**

**Repeat length (main genome) 308**

**Blast2 Results -**

**OFC OLC RFC RLC**

**1 146 1 147**

**Ortholog Repeat Masker annotation**

**SW perc perc perc query position in query matching repeat position in repeat**

**score div. del. ins. sequence begin end (left) repeat class/family begin end (left) ID**

**1236 4.8 1.4 0.0 AluYa5_16_26 1 146 (15) + AluSc SINE/Alu 1 148 (161) 1**

**__________________________________________________________________________________**

**11. AluYa5_16_28c**

**Ortholog annotation C_INTER_RMD_M_DISRUPTED Length 160 nscore 0.00 NPOSITIONS NA**

**Repeat length (main genome) 308**

**Blast2 Results -**

**OFC OLC RFC RLC**

**1 146 1 147**

**Ortholog Repeat Masker annotation**

**SW perc perc perc query position in query matching repeat position in repeat**

**score div. del. ins. sequence begin end (left) repeat class/family begin end (left) ID**

**1236 4.8 1.4 0.0 AluYa5_16_28c 1 146 (14) + AluSc SINE/Alu 1 148 (161) 1**

**__________________________________________________________________________________**

**12. AluYa5_16_37**

**Ortholog annotation C_INTER_RMD_M_DISRUPTED Length 110 nscore 0.00 NPOSITIONS NA**

**Repeat length (main genome) 308**

**Blast2 Results -**

**OFC OLC RFC RLC**

**1 110 1 110**

**Ortholog Repeat Masker annotation**

**SW perc perc perc query position in query matching repeat position in repeat**

**score div. del. ins. sequence begin end (left) repeat class/family begin end (left) ID**

**884 5.5 1.8 0.0 AluYa5_16_37 1 110 (0) + AluSc SINE/Alu 1 112 (197) 1**

**__________________________________________________________________________________**

**13. AluYa5_16_38**

**Ortholog annotation C_INTER_RMD_M_DISRUPTED Length 110 nscore 0.00 NPOSITIONS NA**

**Repeat length (main genome) 308**

**Blast2 Results -**

**OFC OLC RFC RLC**

**1 110 1 110**

**Ortholog Repeat Masker annotation**

**SW perc perc perc query position in query matching repeat position in repeat**

**score div. del. ins. sequence begin end (left) repeat class/family begin end (left) ID**

**884 5.5 1.8 0.0 AluYa5_16_38 1 110 (0) + AluSc SINE/Alu 1 112 (197) 1**

**__________________________________________________________________________________**

**14. AluYa5_16_67**

**Ortholog annotation C_INTER_RMD_M_DISRUPTED Length 138 nscore 0.00 NPOSITIONS NA**

**Repeat length (main genome) 310**

**Blast2 Results -**

**OFC OLC RFC RLC**

**1 125 1 125**

**Ortholog Repeat Masker annotation**

**SW perc perc perc query position in query matching repeat position in repeat**

**score div. del. ins. sequence begin end (left) repeat class/family begin end (left) ID**

**1100 6.8 0.8 0.0 AluYa5_16_67 1 133 (5) + AluSq/x SINE/Alu 1 134 (178) 1**

**__________________________________________________________________________________**

**15. AluYa5_17_28c**

**Ortholog annotation INDEL_PTS Length 3690 nscore 0.00 NPOSITIONS NA**

**Repeat length (main genome) 303**

**Blast2 Results -**

**OFC OLC RFC RLC**

**134 411 12 290**

**2949 3068 5 131**

**3080 3108 103 131**

**3130 3164 159 193**

**3170 3229 240 298**

**Ortholog Repeat Masker annotation**

**SW perc perc perc query position in query matching repeat position in repeat**

**score div. del. ins. sequence begin end (left) repeat class/family begin end (left) ID**

**2652 16.8 6.9 4.2 AluYa5_17_28c 3 122 (3568) + L1MB8 LINE/L1 5484 5607 (571) 1**

**2251 11.1 0.0 0.0 AluYa5_17_28c 123 411 (3279) + AluSg SINE/Alu 1 289 (21) 2**

**2652 16.8 6.9 4.2 AluYa5_17_28c 412 925 (2765) + L1MB8 LINE/L1 5608 6135 (43) 1**

**1207 13.3 0.0 0.0 AluYa5_17_28c 926 1083 (2607) C AluSg/x SINE/Alu (21) 291 134 3**

**2284 9.5 1.0 0.0 AluYa5_17_28c 1084 1377 (2313) C AluSq SINE/Alu (16) 297 1 4**

**2193 10.5 0.3 0.7 AluYa5_17_28c 1654 1983 (1707) C AluSq SINE/Alu (0) 313 1 5**

**2630 6.1 0.0 0.0 AluYa5_17_28c 2012 2321 (1369) C AluY SINE/Alu (1) 310 1 6**

**1234 17.1 8.4 3.5 AluYa5_17_28c 2945 3229 (461) + AluSx SINE/Alu 1 299 (13) 7**

**243 0.0 0.0 0.0 AluYa5_17_28c 3262 3288 (402) + (TAAA)n Simple_repeat 2 28 (0) 8**

**1455 17.2 0.0 0.0 AluYa5_17_28c 3394 3625 (65) C AluJb SINE/Alu (41) 271 40 9**

**__________________________________________________________________________________**

**16. AluYa5_17_47c**

**Ortholog annotation INDEL_PTS Length 9317 nscore 0.00 NPOSITIONS NA**

**Repeat length (main genome) 300**

**Blast2 Results -**

**OFC OLC RFC RLC**

**2307 2346 9 48**

**2356 2580 65 291**

**2429 2459 5 35**

**Ortholog Repeat Masker annotation**

**SW perc perc perc query position in query matching repeat position in repeat**

**score div. del. ins. sequence begin end (left) repeat class/family begin end (left) ID**

**325 17.0 20.2 0.0 AluYa5_17_47c 137 230 (9087) + LTR45C LTR/ERV1 1 113 (426) 1**

**3551 13.4 2.3 0.0 AluYa5_17_47c 233 756 (8561) + LTR45C LTR/ERV1 4 539 (0) 2**

**331 34.9 7.8 0.7 AluYa5_17_47c 907 1059 (8258) + L2 LINE/L2 2667 2830 (589) 3**

**414 27.4 17.7 0.0 AluYa5_17_47c 1083 1268 (8049) + L1ME4a LINE/L1 5700 5918 (206) 4**

**2287 9.6 0.7 0.3 AluYa5_17_47c 1552 1845 (7472) C AluSg SINE/Alu (15) 295 1 5**

**965 22.9 9.8 0.3 AluYa5_17_47c 1888 2172 (7145) C LTR16C LTR/ERVL (0) 489 178 6**

**2163 9.7 3.4 1.0 AluYa5_17_47c 2299 2591 (6726) + AluSx SINE/Alu 1 300 (12) 7**

**318 28.6 6.1 0.0 AluYa5_17_47c 2612 2709 (6608) + MIRc SINE/MIR 90 193 (75) 8**

**324 0.0 0.0 0.0 AluYa5_17_47c 2715 2750 (6567) + (TG)n Simple_repeat 2 37 (0) 9**

**1161 17.1 28.8 3.0 AluYa5_17_47c 2753 3047 (6270) + MLT1A LTR/MaLR 4 374 (0) 10**

**696 18.6 0.9 0.0 AluYa5_17_47c 3494 3606 (5711) + MER81 DNA/AcHobo 1 114 (0) 11**

**592 27.2 14.5 2.1 AluYa5_17_47c 5310 5598 (3719) + MLT1K LTR/MaLR 20 344 (251) 12**

**2189 13.3 0.0 0.3 AluYa5_17_47c 6063 6372 (2945) C AluSg SINE/Alu (1) 309 1 13**

**264 29.5 2.2 1.5 AluYa5_17_47c 7291 7424 (1893) + (TGGA)n Simple_repeat 1 135 (0) 14**

**2323 9.6 0.3 0.0 AluYa5_17_47c 8596 8887 (430) C AluSc SINE/Alu (16) 293 1 15**

**22 5.6 0.0 0.0 AluYa5_17_47c 8895 8930 (387) + AT_rich Low_complexity 1 36 (0) 16**

**1710 20.7 1.6 2.6 AluYa5_17_47c 8934 9310 (7) C L1MB5 LINE/L1 (8) 6166 5794 17**

**__________________________________________________________________________________**

**17. AluYa5_17_49**

**Ortholog annotation INDEL_PTS Length 9317 nscore 0.00 NPOSITIONS NA**

**Repeat length (main genome) 309**

**Blast2 Results -**

**OFC OLC RFC RLC**

**2307 2346 9 48**

**2356 2580 65 291**

**2429 2459 5 35**

**Ortholog Repeat Masker annotation**

**SW perc perc perc query position in query matching repeat position in repeat**

**score div. del. ins. sequence begin end (left) repeat class/family begin end (left) ID**

**325 17.0 20.2 0.0 AluYa5_17_49 137 230 (9087) + LTR45C LTR/ERV1 1 113 (426) 1**

**3551 13.4 2.3 0.0 AluYa5_17_49 233 756 (8561) + LTR45C LTR/ERV1 4 539 (0) 2**

**331 34.9 7.8 0.7 AluYa5_17_49 907 1059 (8258) + L2 LINE/L2 2667 2830 (589) 3**

**414 27.4 17.7 0.0 AluYa5_17_49 1083 1268 (8049) + L1ME4a LINE/L1 5700 5918 (206) 4**

**2287 9.6 0.7 0.3 AluYa5_17_49 1552 1845 (7472) C AluSg SINE/Alu (15) 295 1 5**

**965 22.9 9.8 0.3 AluYa5_17_49 1888 2172 (7145) C LTR16C LTR/ERVL (0) 489 178 6**

**2163 9.7 3.4 1.0 AluYa5_17_49 2299 2591 (6726) + AluSx SINE/Alu 1 300 (12) 7**

**318 28.6 6.1 0.0 AluYa5_17_49 2612 2709 (6608) + MIRc SINE/MIR 90 193 (75) 8**

**324 0.0 0.0 0.0 AluYa5_17_49 2715 2750 (6567) + (TG)n Simple_repeat 2 37 (0) 9**

**1161 17.1 28.8 3.0 AluYa5_17_49 2753 3047 (6270) + MLT1A LTR/MaLR 4 374 (0) 10**

**696 18.6 0.9 0.0 AluYa5_17_49 3494 3606 (5711) + MER81 DNA/AcHobo 1 114 (0) 11**

**592 27.2 14.5 2.1 AluYa5_17_49 5310 5598 (3719) + MLT1K LTR/MaLR 20 344 (251) 12**

**2189 13.3 0.0 0.3 AluYa5_17_49 6063 6372 (2945) C AluSg SINE/Alu (1) 309 1 13**

**264 29.5 2.2 1.5 AluYa5_17_49 7291 7424 (1893) + (TGGA)n Simple_repeat 1 135 (0) 14**

**2323 9.6 0.3 0.0 AluYa5_17_49 8596 8887 (430) C AluSc SINE/Alu (16) 293 1 15**

**22 5.6 0.0 0.0 AluYa5_17_49 8895 8930 (387) + AT_rich Low_complexity 1 36 (0) 16**

**1710 20.7 1.6 2.6 AluYa5_17_49 8934 9310 (7) C L1MB5 LINE/L1 (8) 6166 5794 17**

**__________________________________________________________________________________**

**18. AluYa5_17_55c**

**Ortholog annotation C_DISRUPTED_M_INTER_RMD Length 708 nscore 2.82 NPOSITIONS 417 436 ;**

**Repeat length (main genome) 310**

**Blast2 Results -**

**OFC OLC RFC RLC**

**1 281 1 282**

**437 705 39 307**

**Ortholog Repeat Masker annotation**

**SW perc perc perc query position in query matching repeat position in repeat**

**score div. del. ins. sequence begin end (left) repeat class/family begin end (left) ID**

**2595 0.7 0.4 0.0 AluYa5_17_55c 1 281 (427) + AluYa5 SINE/Alu 1 282 (28) 1**

**2547 0.7 0.0 0.0 AluYa5_17_55c 437 708 (0) + AluYa5 SINE/Alu 39 310 (0) 2**

**__________________________________________________________________________________**

**19. AluYa5_18_12**

**Ortholog annotation C_DISRUPTED_M_INTER_RMD Length 1164 nscore 1.72 NPOSITIONS 837 856 ;**

**Repeat length (main genome) 310**

**Blast2 Results -**

**OFC OLC RFC RLC**

**1 303 1 303**

**426 485 127 186**

**857 1162 2 307**

**Ortholog Repeat Masker annotation**

**SW perc perc perc query position in query matching repeat position in repeat**

**score div. del. ins. sequence begin end (left) repeat class/family begin end (left) ID**

**2833 1.3 0.0 0.0 AluYa5_18_12 1 303 (861) + AluYa5 SINE/Alu 1 303 (7) 1**

**1184 17.9 4.0 1.8 AluYa5_18_12 306 532 (632) + AluJb SINE/Alu 1 232 (80) 2**

**2860 1.3 0.0 0.0 AluYa5_18_12 857 1162 (2) + AluYa5 SINE/Alu 2 307 (3) 3**

**__________________________________________________________________________________**

**20. AluYa5_18_41c**

**Ortholog annotation C_INTER_RMD_M_DISRUPTED Length 328 nscore 6.10 NPOSITIONS 309 328 ;**

**Repeat length (main genome) 310**

**Blast2 Results -**

**OFC OLC RFC RLC**

**1 308 1 307**

**Ortholog Repeat Masker annotation**

**SW perc perc perc query position in query matching repeat position in repeat**

**score div. del. ins. sequence begin end (left) repeat class/family begin end (left) ID**

**2862 0.7 0.0 0.3 AluYa5_18_41c 1 308 (20) + AluYa5 SINE/Alu 1 307 (3) 1**

**__________________________________________________________________________________**

**21. AluYa5_18_50**

**Ortholog annotation INDEL_CAN Length 8700 nscore 0.00 NPOSITIONS NA**

**Repeat length (main genome) 310**

**Blast2 Results -**

**OFC OLC RFC RLC**

**184 468 12 297**

**1512 1763 14 264**

**4494 4519 23 48**

**4575 4768 102 298**

**Ortholog Repeat Masker annotation**

**SW perc perc perc query position in query matching repeat position in repeat**

**score div. del. ins. sequence begin end (left) repeat class/family begin end (left) ID**

**315 0.0 0.0 0.0 AluYa5_18_50 4 38 (8662) + (TAAA)n Simple_repeat 2 36 (0) 1**

**656 15.0 12.7 2.7 AluYa5_18_50 40 149 (8551) C FLAM_C SINE/Alu (19) 124 4 2**

**2333 9.8 0.0 0.0 AluYa5_18_50 173 468 (8232) + AluSg SINE/Alu 1 296 (14) 3**

**230 35.9 0.0 2.5 AluYa5_18_50 688 767 (7933) C MIRc SINE/MIR (36) 232 155 4**

**745 29.7 2.7 2.2 AluYa5_18_50 775 998 (7702) C MIRb SINE/MIR (31) 237 13 5**

**2153 13.6 0.0 0.0 AluYa5_18_50 1499 1807 (6893) + AluSq SINE/Alu 1 309 (4) 6**

**2203 15.3 1.8 2.8 AluYa5_18_50 1888 2278 (6422) C MLT1B LTR/MaLR (0) 390 4 7**

**25 0.0 0.0 0.0 AluYa5_18_50 2572 2596 (6104) + AT_rich Low_complexity 1 25 (0) 8**

**1015 26.3 17.9 8.2 AluYa5_18_50 2604 3129 (5571) + MLT1G1 LTR/MaLR 1 577 (2) 9**

**2186 14.6 2.4 1.2 AluYa5_18_50 3643 3981 (4719) + THE1B LTR/MaLR 17 359 (5) 10**

**1869 15.2 1.0 0.3 AluYa5_18_50 4472 4775 (3925) + AluSx SINE/Alu 1 306 (6) 11**

**2196 16.2 3.3 0.0 AluYa5_18_50 4900 5233 (3467) + Tigger3a DNA/MER2_type 2 346 (2) 12**

**408 37.3 1.1 0.0 AluYa5_18_50 5274 5450 (3250) + Tigger12 DNA/MER2_type 54 232 (1727) 13**

**346 32.5 2.4 0.0 AluYa5_18_50 5469 5594 (3106) + Tigger12 DNA/MER2_type 1816 1944 (15) 13**

**208 25.4 5.1 0.0 AluYa5_18_50 5689 5747 (2953) + MIR SINE/MIR 15 76 (186) 14**

**263 27.0 5.6 0.0 AluYa5_18_50 5996 6084 (2616) + MamRep605 Unknown 617 710 (166) 15**

**1633 20.6 9.5 3.5 AluYa5_18_50 6220 6587 (2113) + MLT1B LTR/MaLR 1 390 (0) 16**

**463 23.0 10.9 3.8 AluYa5_18_50 6880 7084 (1616) C MIR SINE/MIR (1) 261 51 17**

**628 19.9 0.7 2.1 AluYa5_18_50 7085 7228 (1472) C L1MB8 LINE/L1 (1) 6177 6036 18**

**282 31.2 5.7 10.2 AluYa5_18_50 7229 7276 (1424) C MIRm SINE/MIR (226) 50 5 17**

**955 15.4 21.9 1.7 AluYa5_18_50 7335 7571 (1129) C AluJo SINE/Alu (7) 305 21 19**

**612 28.6 7.2 4.7 AluYa5_18_50 7574 7892 (808) + L1ME3B LINE/L1 5610 5936 (304) 20**

**618 9.7 6.1 1.7 AluYa5_18_50 8572 8693 (7) + MLT1A0 LTR/MaLR 1 141 (233) 21**

**__________________________________________________________________________________**

**22. AluYa5_18_80c**

**Ortholog annotation C_DISRUPTED_M_INTER_RMD Length 626 nscore 3.19 NPOSITIONS 100 119 ;**

**Repeat length (main genome) 310**

**Blast2 Results -**

**OFC OLC RFC RLC**

**1 99 1 99**

**353 626 37 310**

**Ortholog Repeat Masker annotation**

**SW perc perc perc query position in query matching repeat position in repeat**

**score div. del. ins. sequence begin end (left) repeat class/family begin end (left) ID**

**888 2.0 0.0 0.0 AluYa5_18_80c 1 99 (527) + AluY SINE/Alu 1 99 (211) 1**

**1862 14.8 0.0 0.0 AluYa5_18_80c 317 626 (0) + AluYa5 SINE/Alu 1 310 (0) 2**

**__________________________________________________________________________________**

**23. AluYa5_19_28**

**Ortholog annotation C_INTER_RMD_M_DISRUPTED Length 126 nscore 0.00 NPOSITIONS NA**

**Repeat length (main genome) 310**

**Blast2 Results -**

**OFC OLC RFC RLC**

**1 126 1 126**

**Ortholog Repeat Masker annotation**

**SW perc perc perc query position in query matching repeat position in repeat**

**score div. del. ins. sequence begin end (left) repeat class/family begin end (left) ID**

**1084 4.8 0.0 0.0 AluYa5_19_28 1 126 (0) + AluSg SINE/Alu 1 126 (184) 1**

**__________________________________________________________________________________**

**24. AluYa5_22_19**

**Ortholog annotation C_INTER_RMD_M_DISRUPTED Length 152 nscore 0.00 NPOSITIONS NA**

**Repeat length (main genome) 161**

**Blast2 Results -**

**OFC OLC RFC RLC**

**1 152 1 152**

**Ortholog Repeat Masker annotation**

**SW perc perc perc query position in query matching repeat position in repeat**

**score div. del. ins. sequence begin end (left) repeat class/family begin end (left) ID**

**1294 5.9 0.0 0.0 AluYa5_22_19 1 152 (0) + AluYa5 SINE/Alu 150 301 (9) 1**

**__________________________________________________________________________________**

**25. AluYa5_2_181**

**Ortholog annotation C_INTER_RMD_M_DISRUPTED Length 131 nscore 0.00 NPOSITIONS NA**

**Repeat length (main genome) 143**

**Blast2 Results -**

**OFC OLC RFC RLC**

**1 131 1 131**

**Ortholog Repeat Masker annotation**

**SW perc perc perc query position in query matching repeat position in repeat**

**score div. del. ins. sequence begin end (left) repeat class/family begin end (left) ID**

**1218 0.8 0.0 0.0 AluYa5_2_181 1 131 (0) + AluYa5/8 SINE/Alu 168 298 (12) 1**

**__________________________________________________________________________________**

**26. AluYa5_2_59**

**Ortholog annotation INDEL_CAN Length 966 nscore 0.00 NPOSITIONS NA**

**Repeat length (main genome) 109**

**Blast2 Results -**

**OFC OLC RFC RLC**

**847 955 1 109**

**Ortholog Repeat Masker annotation**

**SW perc perc perc query position in query matching repeat position in repeat**

**score div. del. ins. sequence begin end (left) repeat class/family begin end (left) ID**

**1031 0.9 0.0 0.0 AluYa5_2_59 847 960 (6) + AluYa5/8 SINE/Alu 197 310 (0) 1**

**__________________________________________________________________________________**

**27. AluYa5_2_67c**

**Ortholog annotation C_INTER_RMD_M_DISRUPTED Length 118 nscore 0.00 NPOSITIONS NA**

**Repeat length (main genome) 310**

**Blast2 Results -**

**OFC OLC RFC RLC**

**1 118 1 118**

**Ortholog Repeat Masker annotation**

**SW perc perc perc query position in query matching repeat position in repeat**

**score div. del. ins. sequence begin end (left) repeat class/family begin end (left) ID**

**1123 0.0 0.0 0.0 AluYa5_2_67c 1 118 (0) + AluYa5/8 SINE/Alu 1 118 (192) 1**

**__________________________________________________________________________________**

**28. AluYa5_3_54**

**Ortholog annotation INDEL_CAN Length 506 nscore 3.95 NPOSITIONS 1 20 ;**

**Repeat length (main genome) 304**

**Blast2 Results -**

**OFC OLC RFC RLC**

**36 75 22 61**

**244 506 40 303**

**Ortholog Repeat Masker annotation**

**SW perc perc perc query position in query matching repeat position in repeat**

**score div. del. ins. sequence begin end (left) repeat class/family begin end (left) ID**

**632 20.7 0.7 3.6 AluYa5_3_54 21 160 (346) + FLAM_C SINE/Alu 7 142 (1) 1**

**430 8.9 0.0 0.0 AluYa5_3_54 168 223 (283) C MLT1B LTR/MaLR (295) 95 40 2**

**2280 6.7 0.3 0.0 AluYa5_3_54 224 506 (0) + AluY SINE/Alu 19 302 (9) 3**

**__________________________________________________________________________________**

**29. AluYa5_3_94c**

**Ortholog annotation C_INTER_RMD_M_DISRUPTED Length 259 nscore 7.72 NPOSITIONS 1 20 ;**

**Repeat length (main genome) 310**

**Blast2 Results -**

**OFC OLC RFC RLC**

**32 259 83 310**

**Ortholog Repeat Masker annotation**

**SW perc perc perc query position in query matching repeat position in repeat**

**score div. del. ins. sequence begin end (left) repeat class/family begin end (left) ID**

**2066 2.5 0.0 0.8 AluYa5_3_94c 21 259 (0) + AluYa5 SINE/Alu 73 309 (1) 1**

**__________________________________________________________________________________**

**30. AluYa5_4_120c**

**Ortholog annotation INDEL_CAN Length 424 nscore 4.72 NPOSITIONS 380 399 ;**

**Repeat length (main genome) 313**

**Blast2 Results -**

**OFC OLC RFC RLC**

**1 313 1 313**

**Ortholog Repeat Masker annotation**

**SW perc perc perc query position in query matching repeat position in repeat**

**score div. del. ins. sequence begin end (left) repeat class/family begin end (left) ID**

**2743 2.6 0.0 1.3 AluYa5_4_120c 1 313 (111) + AluYa5 SINE/Alu 1 309 (1) 1**

**__________________________________________________________________________________**

**31. AluYa5_4_132c**

**Ortholog annotation C_INTER_RMD Length 281 nscore 0.00 NPOSITIONS NA**

**Repeat length (main genome) 296**

**Blast2 Results -**

**OFC OLC RFC RLC**

**1 281 1 281**

**Ortholog Repeat Masker annotation**

**SW perc perc perc query position in query matching repeat position in repeat**

**score div. del. ins. sequence begin end (left) repeat class/family begin end (left) ID**

**2647 0.7 0.0 0.0 AluYa5_4_132c 1 281 (0) + AluYa5 SINE/Alu 1 281 (29) 1**

**__________________________________________________________________________________**

**32. AluYa5_4_168c**

**Ortholog annotation INDEL_CAN Length 129 nscore 0.00 NPOSITIONS NA**

**Repeat length (main genome) 310**

**Blast2 Results -**

**OFC OLC RFC RLC**

**1 129 40 168**

**Ortholog Repeat Masker annotation**

**SW perc perc perc query position in query matching repeat position in repeat**

**score div. del. ins. sequence begin end (left) repeat class/family begin end (left) ID**

**1213 0.8 0.0 0.0 AluYa5_4_168c 1 129 (0) + AluYa5 SINE/Alu 40 168 (142) 1**

**__________________________________________________________________________________**

**33. AluYa5_4_194c**

**Ortholog annotation INDEL_CAN Length 4586 nscore 3.23 NPOSITIONS 2002 2149 ;**

**Repeat length (main genome) 308**

**Blast2 Results -**

**OFC OLC RFC RLC**

**1 289 1 296**

**Ortholog Repeat Masker annotation**

**SW perc perc perc query position in query matching repeat position in repeat**

**score div. del. ins. sequence begin end (left) repeat class/family begin end (left) ID**

**2298 5.2 3.1 1.0 AluYa5_4_194c 1 289 (4297) + AluY SINE/Alu 1 295 (16) 1**

**199 12.2 9.8 0.0 AluYa5_4_194c 382 463 (4123) + (TATAA)n Simple_repeat 1 90 (0) 2**

**274 27.8 10.9 3.2 AluYa5_4_194c 1238 1393 (3193) + L2a LINE/L2 3245 3412 (14) 3**

**481 31.6 9.2 1.9 AluYa5_4_194c 2162 2802 (1784) + L1M5 LINE/L1 3107 3794 (2352) 4**

**748 25.1 6.5 7.7 AluYa5_4_194c 2879 2927 (1659) + L1MC4 LINE/L1 7710 7758 (284) 5**

**2099 20.7 8.8 0.2 AluYa5_4_194c 2928 3425 (1161) C MER34 LTR/ERV1 (0) 542 2 6**

**748 25.1 6.5 7.7 AluYa5_4_194c 3426 3712 (874) + L1MC4 LINE/L1 7759 8042 (0) 5**

**689 23.4 0.7 1.4 AluYa5_4_194c 3790 3935 (651) + MER5B DNA/MER1_type 2 146 (32) 7**

**1355 23.5 5.3 4.8 AluYa5_4_194c 3936 4292 (294) + MLT1A LTR/MaLR 1 359 (15) 8**

**204 17.9 0.0 2.5 AluYa5_4_194c 4302 4341 (245) C MER5B DNA/MER1_type (139) 39 1 9**

**__________________________________________________________________________________**

**34. AluYa5_4_226c**

**Ortholog annotation C_DISRUPTED_M_INTER_RMD Length 597 nscore 3.35 NPOSITIONS 363 382 ;**

**Repeat length (main genome) 310**

**Blast2 Results -**

**OFC OLC RFC RLC**

**1 301 1 310**

**383 597 96 310**

**Ortholog Repeat Masker annotation**

**SW perc perc perc query position in query matching repeat position in repeat**

**score div. del. ins. sequence begin end (left) repeat class/family begin end (left) ID**

**2107 6.3 3.3 0.3 AluYa5_4_226c 1 301 (296) + AluYa5 SINE/Alu 1 310 (0) 1**

**1972 1.9 0.0 0.0 AluYa5_4_226c 383 597 (0) + AluYa5 SINE/Alu 96 310 (0) 2**

**__________________________________________________________________________________**

**35. AluYa5_4_245**

**Ortholog annotation INDEL_CAN Length 962 nscore 0.00 NPOSITIONS NA**

**Repeat length (main genome) 312**

**Blast2 Results -**

**OFC OLC RFC RLC**

**673 962 23 311**

**Ortholog Repeat Masker annotation**

**SW perc perc perc query position in query matching repeat position in repeat**

**score div. del. ins. sequence begin end (left) repeat class/family begin end (left) ID**

**2325 11.0 0.0 0.6 AluYa5_4_245 651 962 (0) + AluSx SINE/Alu 1 310 (2) 1**

**__________________________________________________________________________________**

**36. AluYa5_5_222c**

**Ortholog annotation C_INTER_RMD_M_DISRUPTED Length 120 nscore 0.00 NPOSITIONS NA**

**Repeat length (main genome) 312**

**Blast2 Results -**

**OFC OLC RFC RLC**

**1 120 1 120**

**Ortholog Repeat Masker annotation**

**SW perc perc perc query position in query matching repeat position in repeat**

**score div. del. ins. sequence begin end (left) repeat class/family begin end (left) ID**

**916 10.8 0.0 0.0 AluYa5_5_222c 1 120 (0) + AluSc SINE/Alu 1 120 (189) 1**

**__________________________________________________________________________________**

**37. AluYa5_5_242c**

**Ortholog annotation C_DISRUPTED_M_INTER_RMD Length 615 nscore 3.25 NPOSITIONS 291 310 ;**

**Repeat length (main genome) 306**

**Blast2 Results -**

**OFC OLC RFC RLC**

**1 281 1 283**

**311 612 3 303**

**Ortholog Repeat Masker annotation**

**SW perc perc perc query position in query matching repeat position in repeat**

**score div. del. ins. sequence begin end (left) repeat class/family begin end (left) ID**

**2248 9.2 0.4 0.0 AluYa5_5_242c 1 281 (334) + AluSg SINE/Alu 1 282 (28) 1**

**2809 0.7 0.0 0.3 AluYa5_5_242c 311 612 (3) + AluYa5 SINE/Alu 3 303 (7) 2**

**__________________________________________________________________________________**

**38. AluYa5_5_73**

**Ortholog annotation INDEL_CAN Length 574 nscore 3.48 NPOSITIONS 252 271 ;**

**Repeat length (main genome) 304**

**Blast2 Results -**

**OFC OLC RFC RLC**

**284 574 12 303**

**Ortholog Repeat Masker annotation**

**SW perc perc perc query position in query matching repeat position in repeat**

**score div. del. ins. sequence begin end (left) repeat class/family begin end (left) ID**

**2406 5.2 0.3 0.0 AluYa5_5_73 284 574 (0) + AluYa5 SINE/Alu 12 303 (7) 1**

**__________________________________________________________________________________**

**39. AluYa5_6_204**

**Ortholog annotation C_INTER_RMD_M_DISRUPTED Length 281 nscore 0.00 NPOSITIONS NA**

**Repeat length (main genome) 237**

**Blast2 Results -**

**OFC OLC RFC RLC**

**1 237 1 237**

**Ortholog Repeat Masker annotation**

**SW perc perc perc query position in query matching repeat position in repeat**

**score div. del. ins. sequence begin end (left) repeat class/family begin end (left) ID**

**2661 0.7 0.0 0.0 AluYa5_6_204 1 281 (0) + AluYa5 SINE/Alu 1 281 (29) 1**

**__________________________________________________________________________________**

**40. AluYa5_6_52**

**Ortholog annotation INDEL_CAN Length 386 nscore 0.00 NPOSITIONS NA**

**Repeat length (main genome) 310**

**Blast2 Results -**

**OFC OLC RFC RLC**

**Ortholog Repeat Masker annotation**

**SW perc perc perc query position in query matching repeat position in repeat**

**score div. del. ins. sequence begin end (left) repeat class/family begin end (left) ID**

**2269 13.1 2.6 4.9 AluYa5_6_52 1 386 (0) C LTR12 LTR/ERV1 (285) 541 165 1**

**__________________________________________________________________________________**

**41. AluYa5_7_227**

**Ortholog annotation INDEL_CAN Length 912 nscore 2.19 NPOSITIONS 535 554 ;**

**Repeat length (main genome) 303**

**Blast2 Results -**

**OFC OLC RFC RLC**

**625 912 15 302**

**Ortholog Repeat Masker annotation**

**SW perc perc perc query position in query matching repeat position in repeat**

**score div. del. ins. sequence begin end (left) repeat class/family begin end (left) ID**

**2707 0.3 0.0 0.0 AluYa5_7_227 625 908 (4) + AluYa5 SINE/Alu 15 298 (12) 1**

**__________________________________________________________________________________**

**42. AluYa5_X_62**

**Ortholog annotation INDEL_CAN Length 2239 nscore 0.00 NPOSITIONS NA**

**Repeat length (main genome) 310**

**Blast2 Results -**

**OFC OLC RFC RLC**

**1205 1498 5 296**

**Ortholog Repeat Masker annotation**

**SW perc perc perc query position in query matching repeat position in repeat**

**score div. del. ins. sequence begin end (left) repeat class/family begin end (left) ID**

**506 37.1 8.4 2.3 AluYa5_X_62 872 1133 (1106) C L2b LINE/L2 (74) 3301 3024 1**

**2560 6.4 0.0 0.0 AluYa5_X_62 1201 1498 (741) + AluSp SINE/Alu 1 298 (15) 2**

**1102 0.8 0.0 0.0 AluYa5_X_62 2116 2239 (0) + L1PA5 LINE/L1 5775 5898 (256) 3**

**__________________________________________________________________________________**

**43. AluYa5_X_7**

**Ortholog annotation INDEL_CAN Length 666 nscore 3.00 NPOSITIONS 1 20 ;**

**Repeat length (main genome) 302**

**Blast2 Results -**

**OFC OLC RFC RLC**

**366 666 1 301**

**Ortholog Repeat Masker annotation**

**SW perc perc perc query position in query matching repeat position in repeat**

**score div. del. ins. sequence begin end (left) repeat class/family begin end (left) ID**

**194 30.7 20.6 1.9 AluYa5_X_7 27 235 (431) + MIR SINE/MIR 15 262 (0) 1**

**2854 0.7 0.0 0.0 AluYa5_X_7 366 666 (0) + AluYa5 SINE/Alu 1 301 (9) 2**

**__________________________________________________________________________________**

**44. AluYa5_X_75**

**Ortholog annotation INDEL_CAN Length 1967 nscore 0.00 NPOSITIONS NA**

**Repeat length (main genome) 271**

**Blast2 Results -**

**OFC OLC RFC RLC**

**no hits found**

**Ortholog Repeat Masker annotation**

**SW perc perc perc query position in query matching repeat position in repeat**

**score div. del. ins. sequence begin end (left) repeat class/family begin end (left) ID**

**4334 22.6 6.3 1.3 AluYa5_X_75 1 1967 (0) + L1M1 LINE/L1 2774 4842 (2720) 1**

**__________________________________________________________________________________**

**Supplementary file 4 - AluYa5 comparison with HuRef genome.**

**The file summarizes the blast2 result of the identified ortholog with the AluYa5 repeat and also gives the repeat masker annotation of the identified orthologous locus, along with N details.**

**1. AluYa5_10_109c**

**Ortholog annotation INDEL_CAN Length 9359 nscore 6.18 NPOSITIONS 8743 9320 ;**

**Repeat length (main genome) 310**

**Blast2 Results -**

**OFC OLC RFC RLC**

**2150 2433 12 293**

**2459 2493 23 57**

**2565 2607 122 164**

**2755 2781 38 64**

**2960 3012 243 297**

**4729 4815 1 85**

**4839 4868 102 131**

**4913 5028 163 278**

**9321 9359 259 297**

**Ortholog Repeat Masker annotation**

**SW perc perc perc query position in query matching repeat position in repeat**

**score div. del. ins. sequence begin end (left) repeat class/family begin end (left) ID**

**964 20.6 2.0 6.3 AluYa5_10_109c 237 490 (8869) + MER96B DNA/hAT 165 407 (10) 1**

**765 15.7 0.0 0.0 AluYa5_10_109c 560 680 (8679) C FLAM_C SINE/Alu (21) 122 2 2**

**6224 13.5 1.4 0.1 AluYa5_10_109c 1138 2122 (7237) C L1MA4 LINE/L1 (3) 6297 5300 3**

**2043 12.3 0.0 0.3 AluYa5_10_109c 2141 2433 (6926) + AluSx SINE/Alu 3 294 (18) 4**

**1223 15.8 0.4 4.6 AluYa5_10_109c 2438 2676 (6683) + AluJb SINE/Alu 1 229 (83) 5**

**1751 15.7 1.6 0.7 AluYa5_10_109c 2718 3024 (6335) + AluJo SINE/Alu 1 310 (2) 6**

**2096 10.3 0.0 1.0 AluYa5_10_109c 3162 3455 (5904) C AluSx SINE/Alu (21) 291 1 7**

**252 23.8 6.1 2.4 AluYa5_10_109c 3534 3615 (5744) + L1M1 LINE/L1 5498 5582 (720) 8**

**283 33.3 5.6 4.3 AluYa5_10_109c 3684 3987 (5372) + L1ME3B LINE/L1 5633 5940 (300) 9**

**186 4.3 0.0 0.0 AluYa5_10_109c 4117 4139 (5220) + (GGGGA)n Simple_repeat 1 23 (0) 10**

**278 32.5 4.0 0.8 AluYa5_10_109c 4320 4443 (4916) C MER113 DNA/MER1_type (364) 157 30 11**

**1957 10.0 0.3 9.1 AluYa5_10_109c 4729 5069 (4290) + AluSx SINE/Alu 1 311 (1) 12**

**296 25.8 1.5 0.0 AluYa5_10_109c 6651 6716 (2643) + MER5B DNA/MER1_type 5 71 (107) 13**

**702 18.3 9.7 1.3 AluYa5_10_109c 7052 7206 (2153) C MIR SINE/MIR (81) 181 14 14**

**348 29.4 19.8 4.7 AluYa5_10_109c 7598 8111 (1248) + L2 LINE/L2 2477 3068 (351) 15**

**213 29.2 6.2 0.0 AluYa5_10_109c 8668 8732 (627) C L2a LINE/L2 (2) 3424 3356 16**

**302 0.0 0.0 0.0 AluYa5_10_109c 9321 9359 (0) + AluYc5 SINE/Alu 247 285 (14) 17**

**__________________________________________________________________________________**

**2. AluYa5_10_19c**

**Ortholog annotation C_INTER_RMD_M_DISRUPTED Length 345 nscore 5.80 NPOSITIONS 1 20 ;**

**Repeat length (main genome) 310**

**Blast2 Results -**

**OFC OLC RFC RLC**

**47 345 12 310**

**Ortholog Repeat Masker annotation**

**SW perc perc perc query position in query matching repeat position in repeat**

**score div. del. ins. sequence begin end (left) repeat class/family begin end (left) ID**

**2875 1.9 0.0 0.0 AluYa5_10_19c 36 345 (0) + AluYa5 SINE/Alu 1 310 (0) 1**

**__________________________________________________________________________________**

**3. AluYa5_10_41**

**Ortholog annotation C_INTER_RMD_M_DISRUPTED Length 278 nscore 7.19 NPOSITIONS 259 278 ;**

**Repeat length (main genome) 305**

**Blast2 Results -**

**OFC OLC RFC RLC**

**1 258 1 258**

**Ortholog Repeat Masker annotation**

**SW perc perc perc query position in query matching repeat position in repeat**

**score div. del. ins. sequence begin end (left) repeat class/family begin end (left) ID**

**2466 0.0 0.0 0.0 AluYa5_10_41 1 258 (20) + AluYa5 SINE/Alu 2 259 (51) 1**

**__________________________________________________________________________________**

**4. AluYa5_10_88c**

**Ortholog annotation C_INTER_RMD_M_DISRUPTED Length 324 nscore 6.17 NPOSITIONS 305 324 ;**

**Repeat length (main genome) 301**

**Blast2 Results -**

**OFC OLC RFC RLC**

**1 293 1 293**

**Ortholog Repeat Masker annotation**

**SW perc perc perc query position in query matching repeat position in repeat**

**score div. del. ins. sequence begin end (left) repeat class/family begin end (left) ID**

**2847 0.3 0.0 0.0 AluYa5_10_88c 1 300 (24) + AluYa5 SINE/Alu 1 300 (10) 1**

**__________________________________________________________________________________**

**5. AluYa5_11_26**

**Ortholog annotation C_INTER_RMD_M_DISRUPTED Length 292 nscore 6.85 NPOSITIONS 273 292 ;**

**Repeat length (main genome) 311**

**Blast2 Results -**

**OFC OLC RFC RLC**

**1 272 1 272**

**Ortholog Repeat Masker annotation**

**SW perc perc perc query position in query matching repeat position in repeat**

**score div. del. ins. sequence begin end (left) repeat class/family begin end (left) ID**

**2544 0.0 0.0 0.4 AluYa5_11_26 1 272 (20) + AluYa5 SINE/Alu 1 271 (39) 1**

**__________________________________________________________________________________**

**6. AluYa5_12_108**

**Ortholog annotation C_INTER_RMD Length 196 nscore 10.20 NPOSITIONS 4 23 ;**

**Repeat length (main genome) 263**

**Blast2 Results -**

**OFC OLC RFC RLC**

**24 195 90 261**

**Ortholog Repeat Masker annotation**

**SW perc perc perc query position in query matching repeat position in repeat**

**score div. del. ins. sequence begin end (left) repeat class/family begin end (left) ID**

**1586 1.7 0.0 0.0 AluYa5_12_108 24 195 (1) + AluYa5 SINE/Alu 134 305 (5) 1**

**__________________________________________________________________________________**

**7. AluYa5_12_180c**

**Ortholog annotation C_DISRUPTED_M_INTER_RMD Length 896 nscore 0.00 NPOSITIONS NA**

**Repeat length (main genome) 305**

**Blast2 Results -**

**OFC OLC RFC RLC**

**1 295 1 298**

**604 889 12 298**

**Ortholog Repeat Masker annotation**

**SW perc perc perc query position in query matching repeat position in repeat**

**score div. del. ins. sequence begin end (left) repeat class/family begin end (left) ID**

**2137 11.6 1.4 0.3 AluYa5_12_180c 1 295 (601) + AluSx SINE/Alu 1 298 (14) 1**

**1733 9.5 16.2 0.0 AluYa5_12_180c 297 549 (347) C AluSc SINE/Alu (15) 294 1 2**

**2788 0.7 0.0 0.0 AluYa5_12_180c 593 889 (7) + AluYa5 SINE/Alu 1 297 (13) 3**

**__________________________________________________________________________________**

**8. AluYa5_12_99**

**Ortholog annotation C_INTER_RMD_M_DISRUPTED Length 292 nscore 6.85 NPOSITIONS 1 20 ;**

**Repeat length (main genome) 300**

**Blast2 Results -**

**OFC OLC RFC RLC**

**21 292 28 299**

**Ortholog Repeat Masker annotation**

**SW perc perc perc query position in query matching repeat position in repeat**

**score div. del. ins. sequence begin end (left) repeat class/family begin end (left) ID**

**2610 0.0 0.0 0.0 AluYa5_12_99 21 292 (0) + AluYa5 SINE/Alu 28 299 (11) 1**

**__________________________________________________________________________________**

**9. AluYa5_13_23**

**Ortholog annotation INDEL_CAN Length 335 nscore 5.97 NPOSITIONS 1 20 ;**

**Repeat length (main genome) 307**

**Blast2 Results -**

**OFC OLC RFC RLC**

**21 326 2 307**

**Ortholog Repeat Masker annotation**

**SW perc perc perc query position in query matching repeat position in repeat**

**score div. del. ins. sequence begin end (left) repeat class/family begin end (left) ID**

**2914 0.7 0.0 0.0 AluYa5_13_23 21 329 (6) + AluYa5 SINE/Alu 2 310 (0) 1**

**__________________________________________________________________________________**

**10. AluYa5_13_55c**

**Ortholog annotation C_INTER_RMD_M_DISRUPTED Length 333 nscore 6.61 NPOSITIONS 312 333 ;**

**Repeat length (main genome) 310**

**Blast2 Results -**

**OFC OLC RFC RLC**

**1 306 1 306**

**Ortholog Repeat Masker annotation**

**SW perc perc perc query position in query matching repeat position in repeat**

**score div. del. ins. sequence begin end (left) repeat class/family begin end (left) ID**

**2908 0.3 0.0 0.3 AluYa5_13_55c 1 311 (22) + AluYa5 SINE/Alu 1 310 (0) 1**

**__________________________________________________________________________________**

**11. AluYa5_14_100**

**Ortholog annotation INDEL_CAN Length 810 nscore 2.47 NPOSITIONS 1 20 ;**

**Repeat length (main genome) 294**

**Blast2 Results -**

**OFC OLC RFC RLC**

**32 225 101 294**

**525 810 9 293**

**Ortholog Repeat Masker annotation**

**SW perc perc perc query position in query matching repeat position in repeat**

**score div. del. ins. sequence begin end (left) repeat class/family begin end (left) ID**

**1623 8.9 0.0 0.5 AluYa5_14_100 21 234 (576) + AluSg/x SINE/Alu 92 304 (8) 1**

**436 25.5 4.7 0.0 AluYa5_14_100 256 361 (449) + L2a LINE/L2 3316 3426 (0) 2**

**262 17.7 6.5 0.0 AluYa5_14_100 455 516 (294) C L1ME2z LINE/L1 (1) 6443 6378 3**

**2515 5.5 0.0 0.3 AluYa5_14_100 517 810 (0) + AluY SINE/Alu 1 293 (18) 4**

**__________________________________________________________________________________**

**12. AluYa5_14_3**

**Ortholog annotation C_DISRUPTED_M_INTER_RMD Length 1066 nscore 0.00 NPOSITIONS NA**

**Repeat length (main genome) 300**

**Blast2 Results -**

**OFC OLC RFC RLC**

**1 300 1 300**

**414 471 242 299**

**644 758 12 131**

**770 1066 2 299**

**Ortholog Repeat Masker annotation**

**SW perc perc perc query position in query matching repeat position in repeat**

**score div. del. ins. sequence begin end (left) repeat class/family begin end (left) ID**

**2858 0.3 0.0 0.3 AluYa5_14_3 1 306 (760) + AluYa5 SINE/Alu 1 305 (5) 1**

**1002 19.3 0.0 0.0 AluYa5_14_3 307 477 (589) + AluJb SINE/Alu 135 305 (7) 2**

**969 10.3 3.7 0.0 AluYa5_14_3 633 768 (298) + AluSg1 SINE/Alu 1 141 (168) 3**

**2549 6.4 0.0 0.0 AluYa5_14_3 769 1066 (0) + AluY SINE/Alu 1 298 (13) 4**

**__________________________________________________________________________________**

**13. AluYa5_14_55c**

**Ortholog annotation C_INTER_RMD_M_DISRUPTED Length 361 nscore 18.84 NPOSITIONS 294 361 ;**

**Repeat length (main genome) 308**

**Blast2 Results -**

**OFC OLC RFC RLC**

**1 293 1 293**

**Ortholog Repeat Masker annotation**

**SW perc perc perc query position in query matching repeat position in repeat**

**score div. del. ins. sequence begin end (left) repeat class/family begin end (left) ID**

**2787 0.7 0.0 0.0 AluYa5_14_55c 1 293 (68) + AluYa5 SINE/Alu 1 293 (17) 1**

**__________________________________________________________________________________**

**14. AluYa5_14_77c**

**Ortholog annotation C_INTER_RMD_M_DISRUPTED Length 295 nscore 6.78 NPOSITIONS 1 20 ;**

**Repeat length (main genome) 299**

**Blast2 Results -**

**OFC OLC RFC RLC**

**21 295 25 299**

**Ortholog Repeat Masker annotation**

**SW perc perc perc query position in query matching repeat position in repeat**

**score div. del. ins. sequence begin end (left) repeat class/family begin end (left) ID**

**2341 6.2 0.0 0.0 AluYa5_14_77c 21 295 (0) + AluYa5 SINE/Alu 25 299 (11) 1**

**__________________________________________________________________________________**

**15. AluYa5_14_98c**

**Ortholog annotation INDEL_CAN Length 2961 nscore 0.00 NPOSITIONS NA**

**Repeat length (main genome) 233**

**Blast2 Results -**

**OFC OLC RFC RLC**

**1 233 1 233**

**1028 1082 11 65**

**1850 2067 12 230**

**Ortholog Repeat Masker annotation**

**SW perc perc perc query position in query matching repeat position in repeat**

**score div. del. ins. sequence begin end (left) repeat class/family begin end (left) ID**

**2843 0.7 0.0 0.0 AluYa5_14_98c 1 306 (2655) + AluYa5 SINE/Alu 1 306 (4) 1**

**305 10.4 0.0 0.0 AluYa5_14_98c 958 1005 (1956) C MER57E1 LTR/ERV1 (0) 411 364 2**

**716 19.6 4.7 1.2 AluYa5_14_98c 1020 1189 (1772) + FRAM SINE/Alu 1 176 (0) 3**

**779 24.8 18.1 2.6 AluYa5_14_98c 1192 1456 (1505) C MER57E1 LTR/ERV1 (62) 310 5 2**

**733 31.5 4.6 1.7 AluYa5_14_98c 1523 1838 (1123) + L1M5 LINE/L1 4067 4393 (1753) 4**

**2040 14.4 0.0 0.0 AluYa5_14_98c 1839 2137 (824) + AluSg SINE/Alu 1 299 (11) 5**

**733 31.5 4.6 1.7 AluYa5_14_98c 2138 2148 (813) + L1M5 LINE/L1 4394 4405 (1741) 4**

**1386 22.6 1.0 2.2 AluYa5_14_98c 2149 2460 (501) + AluJo SINE/Alu 3 310 (2) 6**

**733 31.5 4.6 1.7 AluYa5_14_98c 2461 2544 (417) + L1M5 LINE/L1 4406 4491 (1655) 4**

**236 15.0 23.3 0.0 AluYa5_14_98c 2724 2755 (206) + L1M5 LINE/L1 5642 5682 (491) 7**

**183 4.2 0.0 0.0 AluYa5_14_98c 2756 2779 (182) + (CAA)n Simple_repeat 2 25 (0) 8**

**236 15.0 23.3 0.0 AluYa5_14_98c 2780 2806 (155) + L1M5 LINE/L1 5683 5715 (458) 7**

**779 16.6 2.6 0.7 AluYa5_14_98c 2810 2961 (0) C L1M4 LINE/L1 (1571) 4575 4421 9**

**__________________________________________________________________________________**

**16. AluYa5_16_26**

**Ortholog annotation C_INTER_RMD_M_DISRUPTED Length 161 nscore 0.00 NPOSITIONS NA**

**Repeat length (main genome) 308**

**Blast2 Results -**

**OFC OLC RFC RLC**

**1 147 1 148**

**Ortholog Repeat Masker annotation**

**SW perc perc perc query position in query matching repeat position in repeat**

**score div. del. ins. sequence begin end (left) repeat class/family begin end (left) ID**

**1192 6.1 1.4 0.0 AluYa5_16_26 1 147 (14) + AluSc SINE/Alu 1 149 (160) 1**

**__________________________________________________________________________________**

**17. AluYa5_16_28c**

**Ortholog annotation C_INTER_RMD_M_DISRUPTED Length 160 nscore 0.00 NPOSITIONS NA**

**Repeat length (main genome) 308**

**Blast2 Results -**

**OFC OLC RFC RLC**

**1 147 1 148**

**Ortholog Repeat Masker annotation**

**SW perc perc perc query position in query matching repeat position in repeat**

**score div. del. ins. sequence begin end (left) repeat class/family begin end (left) ID**

**1192 6.1 1.4 0.0 AluYa5_16_28c 1 147 (13) + AluSc SINE/Alu 1 149 (160) 1**

**__________________________________________________________________________________**

**18. AluYa5_16_37**

**Ortholog annotation C_INTER_RMD_M_DISRUPTED Length 160 nscore 0.00 NPOSITIONS NA**

**Repeat length (main genome) 308**

**Blast2 Results -**

**OFC OLC RFC RLC**

**1 147 1 148**

**Ortholog Repeat Masker annotation**

**SW perc perc perc query position in query matching repeat position in repeat**

**score div. del. ins. sequence begin end (left) repeat class/family begin end (left) ID**

**1192 6.1 1.4 0.0 AluYa5_16_37 1 147 (13) + AluSc SINE/Alu 1 149 (160) 1**

**__________________________________________________________________________________**

**19. AluYa5_16_38**

**Ortholog annotation C_INTER_RMD_M_DISRUPTED Length 160 nscore 0.00 NPOSITIONS NA**

**Repeat length (main genome) 308**

**Blast2 Results -**

**OFC OLC RFC RLC**

**1 147 1 148**

**Ortholog Repeat Masker annotation**

**SW perc perc perc query position in query matching repeat position in repeat**

**score div. del. ins. sequence begin end (left) repeat class/family begin end (left) ID**

**1192 6.1 1.4 0.0 AluYa5_16_38 1 147 (13) + AluSc SINE/Alu 1 149 (160) 1**

**__________________________________________________________________________________**

**20. AluYa5_16_41c**

**Ortholog annotation INDEL_CAN Length 1391 nscore 0.00 NPOSITIONS NA**

**Repeat length (main genome) 278**

**Blast2 Results -**

**OFC OLC RFC RLC**

**no hits found**

**Ortholog Repeat Masker annotation**

**SW perc perc perc query position in query matching repeat position in repeat**

**score div. del. ins. sequence begin end (left) repeat class/family begin end (left) ID**

**3714 14.8 1.6 6.1 AluYa5_16_41c 1 705 (686) C HERVH-int LTR/ERV1 (3901) 3812 3140 1 ***

**4429 12.8 0.0 2.0 AluYa5_16_41c 685 1391 (0) C HERVH-int LTR/ERV1 (4718) 2995 2303 1**

**__________________________________________________________________________________**

**21. AluYa5_16_4c**

**Ortholog annotation INDEL_CAN Length 92 nscore 0.00 NPOSITIONS NA**

**Repeat length (main genome) 310**

**Blast2 Results -**

**OFC OLC RFC RLC**

**5 92 214 301**

**Ortholog Repeat Masker annotation**

**SW perc perc perc query position in query matching repeat position in repeat**

**score div. del. ins. sequence begin end (left) repeat class/family begin end (left) ID**

**725 4.4 0.0 0.0 AluYa5_16_4c 2 92 (0) + AluYa5/8 SINE/Alu 210 300 (10) 1**

**__________________________________________________________________________________**

**22. AluYa5_16_67**

**Ortholog annotation C_INTER_RMD_M_DISRUPTED Length 138 nscore 0.00 NPOSITIONS NA**

**Repeat length (main genome) 310**

**Blast2 Results -**

**OFC OLC RFC RLC**

**1 125 1 125**

**Ortholog Repeat Masker annotation**

**SW perc perc perc query position in query matching repeat position in repeat**

**score div. del. ins. sequence begin end (left) repeat class/family begin end (left) ID**

**1100 6.8 0.8 0.0 AluYa5_16_67 1 133 (5) + AluSq/x SINE/Alu 1 134 (178) 1**

**__________________________________________________________________________________**

**23. AluYa5_17_101c**

**Ortholog annotation C_INTER_RMD_M_DISRUPTED Length 241 nscore 8.30 NPOSITIONS 222 241 ;**

**Repeat length (main genome) 276**

**Blast2 Results -**

**OFC OLC RFC RLC**

**1 218 1 218**

**Ortholog Repeat Masker annotation**

**SW perc perc perc query position in query matching repeat position in repeat**

**score div. del. ins. sequence begin end (left) repeat class/family begin end (left) ID**

**1575 8.2 13.6 0.5 AluYa5_17_101c 1 221 (20) + AluSg1 SINE/Alu 1 250 (59) 1**

**__________________________________________________________________________________**

**24. AluYa5_17_24c**

**Ortholog annotation C_INTER_RMD_M_DISRUPTED Length 300 nscore 6.67 NPOSITIONS 1 20 ;**

**Repeat length (main genome) 306**

**Blast2 Results -**

**OFC OLC RFC RLC**

**22 296 28 302**

**Ortholog Repeat Masker annotation**

**SW perc perc perc query position in query matching repeat position in repeat**

**score div. del. ins. sequence begin end (left) repeat class/family begin end (left) ID**

**2486 1.8 0.0 0.4 AluYa5_17_24c 22 299 (1) + AluYa5 SINE/Alu 28 304 (6) 1**

**__________________________________________________________________________________**

**25. AluYa5_17_47c**

**Ortholog annotation INDEL_PTS Length 9327 nscore 0.00 NPOSITIONS NA**

**Repeat length (main genome) 300**

**Blast2 Results -**

**OFC OLC RFC RLC**

**2315 2354 9 48**

**2364 2588 65 291**

**2437 2467 5 35**

**Ortholog Repeat Masker annotation**

**SW perc perc perc query position in query matching repeat position in repeat**

**score div. del. ins. sequence begin end (left) repeat class/family begin end (left) ID**

**325 17.0 20.2 0.0 AluYa5_17_47c 145 238 (9089) + LTR45C LTR/ERV1 1 113 (426) 1**

**3551 13.4 2.3 0.0 AluYa5_17_47c 241 764 (8563) + LTR45C LTR/ERV1 4 539 (0) 2**

**331 34.9 7.8 0.7 AluYa5_17_47c 915 1067 (8260) + L2 LINE/L2 2667 2830 (589) 3**

**414 27.4 17.7 0.0 AluYa5_17_47c 1091 1276 (8051) + L1ME4a LINE/L1 5700 5918 (206) 4**

**2287 9.6 0.7 0.3 AluYa5_17_47c 1560 1853 (7474) C AluSg SINE/Alu (15) 295 1 5**

**965 22.9 9.8 0.3 AluYa5_17_47c 1896 2180 (7147) C LTR16C LTR/ERVL (0) 489 178 6**

**2163 9.7 3.4 1.0 AluYa5_17_47c 2307 2599 (6728) + AluSx SINE/Alu 1 300 (12) 7**

**318 28.6 6.1 0.0 AluYa5_17_47c 2620 2717 (6610) + MIRc SINE/MIR 90 193 (75) 8**

**342 0.0 0.0 0.0 AluYa5_17_47c 2723 2760 (6567) + (TG)n Simple_repeat 2 39 (0) 9**

**1161 17.1 28.8 3.0 AluYa5_17_47c 2763 3057 (6270) + MLT1A LTR/MaLR 4 374 (0) 10**

**696 18.6 0.9 0.0 AluYa5_17_47c 3504 3616 (5711) + MER81 DNA/AcHobo 1 114 (0) 11**

**592 27.2 14.5 2.1 AluYa5_17_47c 5320 5608 (3719) + MLT1K LTR/MaLR 20 344 (251) 12**

**2189 13.3 0.0 0.3 AluYa5_17_47c 6073 6382 (2945) C AluSg SINE/Alu (1) 309 1 13**

**264 29.5 2.2 1.5 AluYa5_17_47c 7301 7434 (1893) + (TGGA)n Simple_repeat 1 135 (0) 14**

**2323 9.6 0.3 0.0 AluYa5_17_47c 8606 8897 (430) C AluSc SINE/Alu (16) 293 1 15**

**22 5.6 0.0 0.0 AluYa5_17_47c 8905 8940 (387) + AT_rich Low_complexity 1 36 (0) 16**

**1710 20.7 1.6 2.6 AluYa5_17_47c 8944 9320 (7) C L1MB5 LINE/L1 (8) 6166 5794 17**

**__________________________________________________________________________________**

**26. AluYa5_17_48**

**Ortholog annotation C_INTER_RMD_M_DISRUPTED Length 310 nscore 0.00 NPOSITIONS NA**

**Repeat length (main genome) 308**

**Blast2 Results -**

**OFC OLC RFC RLC**

**17 63 17 63**

**Ortholog Repeat Masker annotation**

**SW perc perc perc query position in query matching repeat position in repeat**

**score div. del. ins. sequence begin end (left) repeat class/family begin end (left) ID**

**1955 17.5 0.0 0.0 AluYa5_17_48 1 297 (13) + AluJb SINE/Alu 1 297 (15) 1**

**__________________________________________________________________________________**

**27. AluYa5_17_49**

**Ortholog annotation INDEL_PTS Length 9327 nscore 0.00 NPOSITIONS NA**

**Repeat length (main genome) 309**

**Blast2 Results -**

**OFC OLC RFC RLC**

**2315 2354 9 48**

**2364 2588 65 291**

**2437 2467 5 35**

**Ortholog Repeat Masker annotation**

**SW perc perc perc query position in query matching repeat position in repeat**

**score div. del. ins. sequence begin end (left) repeat class/family begin end (left) ID**

**325 17.0 20.2 0.0 AluYa5_17_49 145 238 (9089) + LTR45C LTR/ERV1 1 113 (426) 1**

**3551 13.4 2.3 0.0 AluYa5_17_49 241 764 (8563) + LTR45C LTR/ERV1 4 539 (0) 2**

**331 34.9 7.8 0.7 AluYa5_17_49 915 1067 (8260) + L2 LINE/L2 2667 2830 (589) 3**

**414 27.4 17.7 0.0 AluYa5_17_49 1091 1276 (8051) + L1ME4a LINE/L1 5700 5918 (206) 4**

**2287 9.6 0.7 0.3 AluYa5_17_49 1560 1853 (7474) C AluSg SINE/Alu (15) 295 1 5**

**965 22.9 9.8 0.3 AluYa5_17_49 1896 2180 (7147) C LTR16C LTR/ERVL (0) 489 178 6**

**2163 9.7 3.4 1.0 AluYa5_17_49 2307 2599 (6728) + AluSx SINE/Alu 1 300 (12) 7**

**318 28.6 6.1 0.0 AluYa5_17_49 2620 2717 (6610) + MIRc SINE/MIR 90 193 (75) 8**

**342 0.0 0.0 0.0 AluYa5_17_49 2723 2760 (6567) + (TG)n Simple_repeat 2 39 (0) 9**

**1161 17.1 28.8 3.0 AluYa5_17_49 2763 3057 (6270) + MLT1A LTR/MaLR 4 374 (0) 10**

**696 18.6 0.9 0.0 AluYa5_17_49 3504 3616 (5711) + MER81 DNA/AcHobo 1 114 (0) 11**

**592 27.2 14.5 2.1 AluYa5_17_49 5320 5608 (3719) + MLT1K LTR/MaLR 20 344 (251) 12**

**2189 13.3 0.0 0.3 AluYa5_17_49 6073 6382 (2945) C AluSg SINE/Alu (1) 309 1 13**

**264 29.5 2.2 1.5 AluYa5_17_49 7301 7434 (1893) + (TGGA)n Simple_repeat 1 135 (0) 14**

**2323 9.6 0.3 0.0 AluYa5_17_49 8606 8897 (430) C AluSc SINE/Alu (16) 293 1 15**

**22 5.6 0.0 0.0 AluYa5_17_49 8905 8940 (387) + AT_rich Low_complexity 1 36 (0) 16**

**1710 20.7 1.6 2.6 AluYa5_17_49 8944 9320 (7) C L1MB5 LINE/L1 (8) 6166 5794 17**

**__________________________________________________________________________________**

**28. AluYa5_17_50c**

**Ortholog annotation C_INTER_RMD_M_DISRUPTED Length 178 nscore 11.24 NPOSITIONS 1 20 ;**

**Repeat length (main genome) 156**

**Blast2 Results -**

**OFC OLC RFC RLC**

**23 178 1 156**

**Ortholog Repeat Masker annotation**

**SW perc perc perc query position in query matching repeat position in repeat**

**score div. del. ins. sequence begin end (left) repeat class/family begin end (left) ID**

**1239 6.4 1.9 0.0 AluYa5_17_50c 23 178 (0) + AluYa5 SINE/Alu 151 309 (1) 1**

**__________________________________________________________________________________**

**29. AluYa5_17_61c**

**Ortholog annotation INDEL_CAN Length 744 nscore 2.69 NPOSITIONS 575 594 ;**

**Repeat length (main genome) 310**

**Blast2 Results -**

**OFC OLC RFC RLC**

**597 744 150 297**

**Ortholog Repeat Masker annotation**

**SW perc perc perc query position in query matching repeat position in repeat**

**score div. del. ins. sequence begin end (left) repeat class/family begin end (left) ID**

**1381 0.7 0.0 0.0 AluYa5_17_61c 597 744 (0) + AluYa5 SINE/Alu 150 297 (13) 1**

**__________________________________________________________________________________**

**30. AluYa5_18_16**

**Ortholog annotation C_INTER_RMD_M_DISRUPTED Length 321 nscore 6.23 NPOSITIONS 1 20 ;**

**Repeat length (main genome) 299**

**Blast2 Results -**

**OFC OLC RFC RLC**

**24 321 1 298**

**Ortholog Repeat Masker annotation**

**SW perc perc perc query position in query matching repeat position in repeat**

**score div. del. ins. sequence begin end (left) repeat class/family begin end (left) ID**

**2721 2.0 0.0 0.0 AluYa5_18_16 24 321 (0) + AluYa5 SINE/Alu 1 298 (12) 1**

**__________________________________________________________________________________**

**31. AluYa5_18_50**

**Ortholog annotation INDEL_CAN Length 8701 nscore 0.00 NPOSITIONS NA**

**Repeat length (main genome) 310**

**Blast2 Results -**

**OFC OLC RFC RLC**

**184 468 12 297**

**1512 1758 14 259**

**4494 4519 23 48**

**4575 4768 102 298**

**Ortholog Repeat Masker annotation**

**SW perc perc perc query position in query matching repeat position in repeat**

**score div. del. ins. sequence begin end (left) repeat class/family begin end (left) ID**

**315 0.0 0.0 0.0 AluYa5_18_50 4 38 (8663) + (TAAA)n Simple_repeat 2 36 (0) 1**

**656 15.0 12.7 2.7 AluYa5_18_50 40 149 (8552) C FLAM_C SINE/Alu (19) 124 4 2**

**2333 9.8 0.0 0.0 AluYa5_18_50 173 468 (8233) + AluSg SINE/Alu 1 296 (14) 3**

**230 35.9 0.0 2.5 AluYa5_18_50 688 767 (7934) C MIRc SINE/MIR (36) 232 155 4**

**745 29.7 2.7 2.2 AluYa5_18_50 775 998 (7703) C MIRb SINE/MIR (31) 237 13 5**

**2123 13.9 0.0 0.0 AluYa5_18_50 1499 1807 (6894) + AluSq SINE/Alu 1 309 (4) 6**

**2203 15.3 1.8 2.8 AluYa5_18_50 1888 2278 (6423) C MLT1B LTR/MaLR (0) 390 4 7**

**25 0.0 0.0 0.0 AluYa5_18_50 2572 2596 (6105) + AT_rich Low_complexity 1 25 (0) 8**

**1015 26.3 17.9 8.2 AluYa5_18_50 2604 3129 (5572) + MLT1G1 LTR/MaLR 1 577 (2) 9**

**2186 14.6 2.4 1.2 AluYa5_18_50 3643 3981 (4720) + THE1B LTR/MaLR 17 359 (5) 10**

**1869 15.2 1.0 0.3 AluYa5_18_50 4472 4775 (3926) + AluSx SINE/Alu 1 306 (6) 11**

**2196 16.2 3.3 0.0 AluYa5_18_50 4900 5233 (3468) + Tigger3a DNA/MER2_type 2 346 (2) 12**

**408 37.3 1.1 0.0 AluYa5_18_50 5274 5450 (3251) + Tigger12 DNA/MER2_type 54 232 (1727) 13**

**329 28.1 1.1 0.0 AluYa5_18_50 5469 5557 (3144) + Tigger12 DNA/MER2_type 1816 1905 (54) 13**

**208 25.4 5.1 0.0 AluYa5_18_50 5690 5748 (2953) + MIR SINE/MIR 15 76 (186) 14**

**263 27.0 5.6 0.0 AluYa5_18_50 5997 6085 (2616) + MamRep605 Unknown 617 710 (166) 15**

**1672 20.0 9.5 3.5 AluYa5_18_50 6221 6588 (2113) + MLT1B LTR/MaLR 1 390 (0) 16**

**463 23.0 10.9 3.8 AluYa5_18_50 6881 7085 (1616) C MIR SINE/MIR (1) 261 51 17**

**628 19.9 0.7 2.1 AluYa5_18_50 7086 7229 (1472) C L1MB8 LINE/L1 (1) 6177 6036 18**

**282 31.2 5.7 10.2 AluYa5_18_50 7230 7277 (1424) C MIRm SINE/MIR (226) 50 5 17**

**955 15.4 21.9 1.7 AluYa5_18_50 7336 7572 (1129) C AluJo SINE/Alu (7) 305 21 19**

**612 28.6 7.2 4.7 AluYa5_18_50 7575 7893 (808) + L1ME3B LINE/L1 5610 5936 (304) 20**

**618 9.7 6.1 1.7 AluYa5_18_50 8573 8694 (7) + MLT1A0 LTR/MaLR 1 141 (233) 21**

**__________________________________________________________________________________**

**32. AluYa5_19_28**

**Ortholog annotation C_INTER_RMD_M_DISRUPTED Length 126 nscore 0.00 NPOSITIONS NA**

**Repeat length (main genome) 310**

**Blast2 Results -**

**OFC OLC RFC RLC**

**1 126 1 126**

**Ortholog Repeat Masker annotation**

**SW perc perc perc query position in query matching repeat position in repeat**

**score div. del. ins. sequence begin end (left) repeat class/family begin end (left) ID**

**1084 4.8 0.0 0.0 AluYa5_19_28 1 126 (0) + AluSg SINE/Alu 1 126 (184) 1**

**__________________________________________________________________________________**

**33. AluYa5_19_9**

**Ortholog annotation C_INTER_RMD_M_DISRUPTED Length 364 nscore 16.76 NPOSITIONS 304 364 ;**

**Repeat length (main genome) 310**

**Blast2 Results -**

**OFC OLC RFC RLC**

**1 303 1 303**

**Ortholog Repeat Masker annotation**

**SW perc perc perc query position in query matching repeat position in repeat**

**score div. del. ins. sequence begin end (left) repeat class/family begin end (left) ID**

**2874 0.7 0.0 0.0 AluYa5_19_9 1 303 (61) + AluYa5 SINE/Alu 1 303 (7) 1**

**__________________________________________________________________________________**

**34. AluYa5_1_181**

**Ortholog annotation INDEL_PTS Length 3232 nscore 0.00 NPOSITIONS NA**

**Repeat length (main genome) 155**

**Blast2 Results -**

**OFC OLC RFC RLC**

**Ortholog Repeat Masker annotation**

**SW perc perc perc query position in query matching repeat position in repeat**

**score div. del. ins. sequence begin end (left) repeat class/family begin end (left) ID**

**10548 14.9 2.4 1.7 AluYa5_1_181 3 2280 (952) C L1P3 LINE/L1 (4201) 2282 10 1**

**688 16.5 1.6 0.0 AluYa5_1_181 2289 2409 (823) + L1MC LINE/L1 5630 5752 (592) 2**

**25 0.0 0.0 0.0 AluYa5_1_181 2468 2492 (740) + AT_rich Low_complexity 1 25 (0) 3**

**484 29.2 6.0 0.0 AluYa5_1_181 2564 2731 (501) C MLT1N2 LTR/MaLR (10) 552 375 4**

**296 8.5 0.0 0.0 AluYa5_1_181 3183 3229 (3) C AluYc5 SINE/Alu (6) 293 247 5**

**__________________________________________________________________________________**

**35. AluYa5_1_183**

**Ortholog annotation INDEL_PTS Length 3232 nscore 0.00 NPOSITIONS NA**

**Repeat length (main genome) 156**

**Blast2 Results -**

**OFC OLC RFC RLC**

**Ortholog Repeat Masker annotation**

**SW perc perc perc query position in query matching repeat position in repeat**

**score div. del. ins. sequence begin end (left) repeat class/family begin end (left) ID**

**10548 14.9 2.4 1.7 AluYa5_1_183 3 2280 (952) C L1P3 LINE/L1 (4201) 2282 10 1**

**688 16.5 1.6 0.0 AluYa5_1_183 2289 2409 (823) + L1MC LINE/L1 5630 5752 (592) 2**

**25 0.0 0.0 0.0 AluYa5_1_183 2468 2492 (740) + AT_rich Low_complexity 1 25 (0) 3**

**484 29.2 6.0 0.0 AluYa5_1_183 2564 2731 (501) C MLT1N2 LTR/MaLR (10) 552 375 4**

**296 8.5 0.0 0.0 AluYa5_1_183 3183 3229 (3) C AluYc5 SINE/Alu (6) 293 247 5**

**__________________________________________________________________________________**

**36. AluYa5_1_224**

**Ortholog annotation C_INTER_RMD_M_DISRUPTED Length 311 nscore 6.43 NPOSITIONS 289 308 ;**

**Repeat length (main genome) 285**

**Blast2 Results -**

**OFC OLC RFC RLC**

**1 286 1 285**

**Ortholog Repeat Masker annotation**

**SW perc perc perc query position in query matching repeat position in repeat**

**score div. del. ins. sequence begin end (left) repeat class/family begin end (left) ID**

**2704 1.1 0.0 0.0 AluYa5_1_224 1 287 (24) + AluYa5 SINE/Alu 13 299 (11) 1**

**__________________________________________________________________________________**

**37. AluYa5_1_226**

**Ortholog annotation INDEL_CAN Length 442 nscore 0.00 NPOSITIONS NA**

**Repeat length (main genome) 312**

**Blast2 Results -**

**OFC OLC RFC RLC**

**148 441 15 310**

**Ortholog Repeat Masker annotation**

**SW perc perc perc query position in query matching repeat position in repeat**

**score div. del. ins. sequence begin end (left) repeat class/family begin end (left) ID**

**576 17.9 5.1 0.0 AluYa5_1_226 1 117 (325) + L1MC4a LINE/L1 7483 7605 (277) 1**

**2854 1.0 0.0 0.0 AluYa5_1_226 135 441 (1) + AluYa5 SINE/Alu 1 307 (3) 2**

**__________________________________________________________________________________**

**38. AluYa5_1_313**

**Ortholog annotation INDEL_PTS Length 847 nscore 2.36 NPOSITIONS 827 847 ;**

**Repeat length (main genome) 201**

**Blast2 Results -**

**OFC OLC RFC RLC**

**1 201 1 201**

**Ortholog Repeat Masker annotation**

**SW perc perc perc query position in query matching repeat position in repeat**

**score div. del. ins. sequence begin end (left) repeat class/family begin end (left) ID**

**1941 0.5 0.0 0.0 AluYa5_1_313 1 205 (642) + AluYa5 SINE/Alu 105 309 (1) 1**

**__________________________________________________________________________________**

**39. AluYa5_1_38c**

**Ortholog annotation INDEL_CAN Length 535 nscore 3.74 NPOSITIONS 335 354 ;**

**Repeat length (main genome) 296**

**Blast2 Results -**

**OFC OLC RFC RLC**

**365 535 126 296**

**397 426 24 53**

**Ortholog Repeat Masker annotation**

**SW perc perc perc query position in query matching repeat position in repeat**

**score div. del. ins. sequence begin end (left) repeat class/family begin end (left) ID**

**1453 7.3 0.0 0.0 AluYa5_1_38c 358 535 (0) + AluYa5 SINE/Alu 119 296 (14) 1**

**__________________________________________________________________________________**

**40. AluYa5_1_62c**

**Ortholog annotation INDEL_CAN Length 198 nscore 10.10 NPOSITIONS 1 20 ;**

**Repeat length (main genome) 184**

**Blast2 Results -**

**OFC OLC RFC RLC**

**25 186 22 181**

**171 198 157 184**

**Ortholog Repeat Masker annotation**

**SW perc perc perc query position in query matching repeat position in repeat**

**score div. del. ins. sequence begin end (left) repeat class/family begin end (left) ID**

**1243 8.3 0.0 1.2 AluYa5_1_62c 21 190 (8) + AluYa5 SINE/Alu 143 310 (0) 1**

**__________________________________________________________________________________**

**41. AluYa5_1_72c**

**Ortholog annotation C_INTER_RMD_M_DISRUPTED Length 295 nscore 14.58 NPOSITIONS 253 295 ;**

**Repeat length (main genome) 273**

**Blast2 Results -**

**OFC OLC RFC RLC**

**3 252 3 252**

**Ortholog Repeat Masker annotation**

**SW perc perc perc query position in query matching repeat position in repeat**

**score div. del. ins. sequence begin end (left) repeat class/family begin end (left) ID**

**2339 0.8 0.0 0.0 AluYa5_1_72c 3 252 (43) + AluYa5 SINE/Alu 38 287 (23) 1**

**__________________________________________________________________________________**

**42. AluYa5_20_2**

**Ortholog annotation C_INTER_RMD_M_DISRUPTED Length 290 nscore 6.90 NPOSITIONS 1 20 ;**

**Repeat length (main genome) 306**

**Blast2 Results -**

**OFC OLC RFC RLC**

**21 290 36 305**

**Ortholog Repeat Masker annotation**

**SW perc perc perc query position in query matching repeat position in repeat**

**score div. del. ins. sequence begin end (left) repeat class/family begin end (left) ID**

**2485 1.9 0.0 0.0 AluYa5_20_2 21 290 (0) + AluYa5 SINE/Alu 38 307 (3) 1**

**__________________________________________________________________________________**

**43. AluYa5_20_46c**

**Ortholog annotation C_INTER_RMD_M_DISRUPTED Length 325 nscore 6.15 NPOSITIONS 2 21 ;**

**Repeat length (main genome) 302**

**Blast2 Results -**

**OFC OLC RFC RLC**

**24 325 1 302**

**Ortholog Repeat Masker annotation**

**SW perc perc perc query position in query matching repeat position in repeat**

**score div. del. ins. sequence begin end (left) repeat class/family begin end (left) ID**

**2816 1.3 0.0 0.0 AluYa5_20_46c 24 325 (0) + AluYa5 SINE/Alu 2 303 (7) 1**

**__________________________________________________________________________________**

**44. AluYa5_20_71c**

**Ortholog annotation INDEL_CAN Length 756 nscore 2.65 NPOSITIONS 1 20 ;**

**Repeat length (main genome) 308**

**Blast2 Results -**

**OFC OLC RFC RLC**

**35 321 12 301**

**449 756 1 308**

**Ortholog Repeat Masker annotation**

**SW perc perc perc query position in query matching repeat position in repeat**

**score div. del. ins. sequence begin end (left) repeat class/family begin end (left) ID**

**2443 7.7 0.3 0.3 AluYa5_20_71c 24 334 (422) + AluY SINE/Alu 1 311 (0) 1**

**2718 3.3 0.0 1.0 AluYa5_20_71c 449 756 (0) + AluYa5 SINE/Alu 1 305 (5) 2**

**__________________________________________________________________________________**

**45. AluYa5_20_81**

**Ortholog annotation C_INTER_RMD_M_DISRUPTED Length 320 nscore 6.25 NPOSITIONS 1 20 ;**

**Repeat length (main genome) 297**

**Blast2 Results -**

**OFC OLC RFC RLC**

**25 320 1 296**

**Ortholog Repeat Masker annotation**

**SW perc perc perc query position in query matching repeat position in repeat**

**score div. del. ins. sequence begin end (left) repeat class/family begin end (left) ID**

**2814 0.3 0.0 0.0 AluYa5_20_81 25 320 (0) + AluYa5 SINE/Alu 1 296 (14) 1**

**__________________________________________________________________________________**

**46. AluYa5_21_28**

**Ortholog annotation C_INTER_RMD_M_DISRUPTED Length 284 nscore 7.04 NPOSITIONS 1 20 ;**

**Repeat length (main genome) 309**

**Blast2 Results -**

**OFC OLC RFC RLC**

**23 284 47 308**

**Ortholog Repeat Masker annotation**

**SW perc perc perc query position in query matching repeat position in repeat**

**score div. del. ins. sequence begin end (left) repeat class/family begin end (left) ID**

**2241 2.7 0.4 0.4 AluYa5_21_28 23 284 (0) + AluYa5 SINE/Alu 47 308 (2) 1**

**__________________________________________________________________________________**

**47. AluYa5_22_19**

**Ortholog annotation C_INTER_RMD_M_DISRUPTED Length 153 nscore 0.00 NPOSITIONS NA**

**Repeat length (main genome) 161**

**Blast2 Results -**

**OFC OLC RFC RLC**

**1 153 1 153**

**Ortholog Repeat Masker annotation**

**SW perc perc perc query position in query matching repeat position in repeat**

**score div. del. ins. sequence begin end (left) repeat class/family begin end (left) ID**

**1301 5.9 0.0 0.0 AluYa5_22_19 1 153 (0) + AluYa5 SINE/Alu 150 302 (8) 1**

**__________________________________________________________________________________**

**48. AluYa5_22_3c**

**Ortholog annotation C_INTER_RMD_M_DISRUPTED Length 326 nscore 6.13 NPOSITIONS 307 326 ;**

**Repeat length (main genome) 310**

**Blast2 Results -**

**OFC OLC RFC RLC**

**1 306 1 306**

**Ortholog Repeat Masker annotation**

**SW perc perc perc query position in query matching repeat position in repeat**

**score div. del. ins. sequence begin end (left) repeat class/family begin end (left) ID**

**2756 2.6 0.0 0.0 AluYa5_22_3c 1 306 (20) + AluYa5 SINE/Alu 1 306 (4) 1**

**__________________________________________________________________________________**

**49. AluYa5_2_215**

**Ortholog annotation INDEL_CAN Length 1090 nscore 1.83 NPOSITIONS 791 810 ;**

**Repeat length (main genome) 304**

**Blast2 Results -**

**OFC OLC RFC RLC**

**811 1090 24 303**

**Ortholog Repeat Masker annotation**

**SW perc perc perc query position in query matching repeat position in repeat**

**score div. del. ins. sequence begin end (left) repeat class/family begin end (left) ID**

**569 11.0 0.0 0.0 AluYa5_2_215 12 102 (988) C Alu SINE/Alu (219) 91 1 1**

**2571 1.4 0.0 0.4 AluYa5_2_215 811 1090 (0) + AluYa5 SINE/Alu 24 302 (8) 2**

**__________________________________________________________________________________**

**50. AluYa5_2_275**

**Ortholog annotation C_INTER_RMD_M_DISRUPTED Length 279 nscore 7.17 NPOSITIONS 260 279 ;**

**Repeat length (main genome) 303**

**Blast2 Results -**

**OFC OLC RFC RLC**

**1 258 1 258**

**Ortholog Repeat Masker annotation**

**SW perc perc perc query position in query matching repeat position in repeat**

**score div. del. ins. sequence begin end (left) repeat class/family begin end (left) ID**

**2369 1.2 0.0 0.4 AluYa5_2_275 1 258 (21) + AluYa5 SINE/Alu 1 257 (53) 1**

**__________________________________________________________________________________**

**51. AluYa5_2_335**

**Ortholog annotation C_INTER_RMD_M_DISRUPTED Length 331 nscore 6.04 NPOSITIONS 1 20 ;**

**Repeat length (main genome) 306**

**Blast2 Results -**

**OFC OLC RFC RLC**

**27 331 1 305**

**Ortholog Repeat Masker annotation**

**SW perc perc perc query position in query matching repeat position in repeat**

**score div. del. ins. sequence begin end (left) repeat class/family begin end (left) ID**

**2807 2.3 0.0 0.0 AluYa5_2_335 27 331 (0) + AluYa5 SINE/Alu 1 305 (5) 1**

**__________________________________________________________________________________**

**52. AluYa5_2_359c**

**Ortholog annotation C_INTER_RMD_M_DISRUPTED Length 277 nscore 7.22 NPOSITIONS 258 277 ;**

**Repeat length (main genome) 271**

**Blast2 Results -**

**OFC OLC RFC RLC**

**1 257 1 257**

**Ortholog Repeat Masker annotation**

**SW perc perc perc query position in query matching repeat position in repeat**

**score div. del. ins. sequence begin end (left) repeat class/family begin end (left) ID**

**2395 1.6 0.0 0.0 AluYa5_2_359c 1 257 (20) + AluYa5 SINE/Alu 35 291 (19) 1**

**__________________________________________________________________________________**

**53. AluYa5_2_35c**

**Ortholog annotation C_INTER_RMD_M_DISRUPTED Length 321 nscore 6.23 NPOSITIONS 302 321 ;**

**Repeat length (main genome) 310**

**Blast2 Results -**

**OFC OLC RFC RLC**

**1 301 1 301**

**Ortholog Repeat Masker annotation**

**SW perc perc perc query position in query matching repeat position in repeat**

**score div. del. ins. sequence begin end (left) repeat class/family begin end (left) ID**

**2859 0.3 0.0 0.0 AluYa5_2_35c 1 301 (20) + AluYa5 SINE/Alu 1 301 (9) 1**

**__________________________________________________________________________________**

**54. AluYa5_2_47c**

**Ortholog annotation C_INTER_RMD_M_DISRUPTED Length 339 nscore 15.34 NPOSITIONS 1 52 ;**

**Repeat length (main genome) 309**

**Blast2 Results -**

**OFC OLC RFC RLC**

**53 339 23 309**

**Ortholog Repeat Masker annotation**

**SW perc perc perc query position in query matching repeat position in repeat**

**score div. del. ins. sequence begin end (left) repeat class/family begin end (left) ID**

**2461 4.5 0.0 0.0 AluYa5_2_47c 53 339 (0) + AluYa5 SINE/Alu 23 309 (1) 1**

**__________________________________________________________________________________**

**55. AluYa5_2_59**

**Ortholog annotation INDEL_CAN Length 965 nscore 0.00 NPOSITIONS NA**

**Repeat length (main genome) 109**

**Blast2 Results -**

**OFC OLC RFC RLC**

**847 955 1 109**

**Ortholog Repeat Masker annotation**

**SW perc perc perc query position in query matching repeat position in repeat**

**score div. del. ins. sequence begin end (left) repeat class/family begin end (left) ID**

**1031 0.9 0.0 0.0 AluYa5_2_59 847 960 (5) + AluYa5/8 SINE/Alu 197 310 (0) 1**

**__________________________________________________________________________________**

**56. AluYa5_2_66c**

**Ortholog annotation INDEL_CAN Length 186 nscore 0.00 NPOSITIONS NA**

**Repeat length (main genome) 192**

**Blast2 Results -**

**OFC OLC RFC RLC**

**2 185 1 184**

**Ortholog Repeat Masker annotation**

**SW perc perc perc query position in query matching repeat position in repeat**

**score div. del. ins. sequence begin end (left) repeat class/family begin end (left) ID**

**1533 6.0 0.0 0.0 AluYa5_2_66c 1 185 (1) + AluYa5 SINE/Alu 118 302 (8) 1**

**__________________________________________________________________________________**

**57. AluYa5_2_67c**

**Ortholog annotation C_INTER_RMD_M_DISRUPTED Length 118 nscore 0.00 NPOSITIONS NA**

**Repeat length (main genome) 310**

**Blast2 Results -**

**OFC OLC RFC RLC**

**1 118 1 118**

**Ortholog Repeat Masker annotation**

**SW perc perc perc query position in query matching repeat position in repeat**

**score div. del. ins. sequence begin end (left) repeat class/family begin end (left) ID**

**1123 0.0 0.0 0.0 AluYa5_2_67c 1 118 (0) + AluYa5/8 SINE/Alu 1 118 (192) 1**

**__________________________________________________________________________________**

**58. AluYa5_2_87**

**Ortholog annotation C_INTER_RMD_M_DISRUPTED Length 368 nscore 19.29 NPOSITIONS 1 71 ;**

**Repeat length (main genome) 294**

**Blast2 Results -**

**OFC OLC RFC RLC**

**77 368 1 293**

**Ortholog Repeat Masker annotation**

**SW perc perc perc query position in query matching repeat position in repeat**

**score div. del. ins. sequence begin end (left) repeat class/family begin end (left) ID**

**2698 1.0 0.3 0.0 AluYa5_2_87 77 368 (0) + AluYa5 SINE/Alu 1 293 (17) 1**

**__________________________________________________________________________________**

**59. AluYa5_3_139c**

**Ortholog annotation C_INTER_RMD_M_DISRUPTED Length 308 nscore 0.00 NPOSITIONS NA**

**Repeat length (main genome) 303**

**Blast2 Results -**

**OFC OLC RFC RLC**

**1 303 1 303**

**Ortholog Repeat Masker annotation**

**SW perc perc perc query position in query matching repeat position in repeat**

**score div. del. ins. sequence begin end (left) repeat class/family begin end (left) ID**

**2854 1.9 0.0 0.0 AluYa5_3_139c 1 308 (0) + AluYa5 SINE/Alu 1 308 (2) 1**

**__________________________________________________________________________________**

**60. AluYa5_3_152c**

**Ortholog annotation C_INTER_RMD_M_DISRUPTED Length 311 nscore 8.36 NPOSITIONS 1 26 ;**

**Repeat length (main genome) 301**

**Blast2 Results -**

**OFC OLC RFC RLC**

**27 311 17 301**

**Ortholog Repeat Masker annotation**

**SW perc perc perc query position in query matching repeat position in repeat**

**score div. del. ins. sequence begin end (left) repeat class/family begin end (left) ID**

**2665 1.4 0.0 0.0 AluYa5_3_152c 27 311 (0) + AluYa5 SINE/Alu 17 301 (9) 1**

**__________________________________________________________________________________**

**61. AluYa5_3_205c**

**Ortholog annotation C_INTER_RMD_M_DISRUPTED Length 385 nscore 0.00 NPOSITIONS NA**

**Repeat length (main genome) 307**

**Blast2 Results -**

**OFC OLC RFC RLC**

**1 300 1 300**

**Ortholog Repeat Masker annotation**

**SW perc perc perc query position in query matching repeat position in repeat**

**score div. del. ins. sequence begin end (left) repeat class/family begin end (left) ID**

**2752 1.0 0.0 0.3 AluYa5_3_205c 1 300 (85) + AluYa5 SINE/Alu 1 299 (11) 1**

**213 14.6 0.0 2.4 AluYa5_3_205c 301 342 (43) + (TAGA)n Simple_repeat 1 41 (0) 2**

**__________________________________________________________________________________**

**62. AluYa5_3_5**

**Ortholog annotation C_INTER_RMD_M_DISRUPTED Length 331 nscore 6.04 NPOSITIONS 1 20 ;**

**Repeat length (main genome) 305**

**Blast2 Results -**

**OFC OLC RFC RLC**

**28 331 1 304**

**Ortholog Repeat Masker annotation**

**SW perc perc perc query position in query matching repeat position in repeat**

**score div. del. ins. sequence begin end (left) repeat class/family begin end (left) ID**

**2638 4.0 0.0 0.0 AluYa5_3_5 28 331 (0) + AluYa5 SINE/Alu 1 304 (6) 1**

**__________________________________________________________________________________**

**63. AluYa5_3_50**

**Ortholog annotation C_DISRUPTED_M_INTER_RMD Length 1750 nscore 2.29 NPOSITIONS 302 321 ; 1450 1469 ;**

**Repeat length (main genome) 295**

**Blast2 Results -**

**OFC OLC RFC RLC**

**1 295 1 295**

**1470 1750 14 294**

**Ortholog Repeat Masker annotation**

**SW perc perc perc query position in query matching repeat position in repeat**

**score div. del. ins. sequence begin end (left) repeat class/family begin end (left) ID**

**2838 0.0 0.0 0.0 AluYa5_3_50 1 296 (1454) + AluYa5 SINE/Alu 1 296 (14) 1**

**2165 16.2 2.8 6.2 AluYa5_3_50 322 504 (1246) + L1MB3 LINE/L1 5713 5890 (293) 2**

**2422 8.0 0.0 0.3 AluYa5_3_50 505 806 (944) C AluSx SINE/Alu (11) 301 1 3**

**2165 16.2 2.8 6.2 AluYa5_3_50 807 1090 (660) + L1MB3 LINE/L1 5891 6164 (19) 2**

**2315 4.9 0.0 0.0 AluYa5_3_50 1184 1449 (301) C AluY SINE/Alu (18) 293 28 4**

**2705 0.0 0.0 0.0 AluYa5_3_50 1470 1750 (0) + AluYa5 SINE/Alu 14 294 (16) 5**

**__________________________________________________________________________________**

**64. AluYa5_4_132c**

**Ortholog annotation C_INTER_RMD Length 281 nscore 0.00 NPOSITIONS NA**

**Repeat length (main genome) 296**

**Blast2 Results -**

**OFC OLC RFC RLC**

**1 281 1 281**

**Ortholog Repeat Masker annotation**

**SW perc perc perc query position in query matching repeat position in repeat**

**score div. del. ins. sequence begin end (left) repeat class/family begin end (left) ID**

**2647 0.7 0.0 0.0 AluYa5_4_132c 1 281 (0) + AluYa5 SINE/Alu 1 281 (29) 1**

**__________________________________________________________________________________**

**65. AluYa5_4_168c**

**Ortholog annotation INDEL_CAN Length 129 nscore 0.00 NPOSITIONS NA**

**Repeat length (main genome) 310**

**Blast2 Results -**

**OFC OLC RFC RLC**

**1 129 40 168**

**Ortholog Repeat Masker annotation**

**SW perc perc perc query position in query matching repeat position in repeat**

**score div. del. ins. sequence begin end (left) repeat class/family begin end (left) ID**

**1215 0.8 0.0 0.0 AluYa5_4_168c 1 129 (0) + AluYa5 SINE/Alu 40 168 (142) 1**

**__________________________________________________________________________________**

**66. AluYa5_4_194c**

**Ortholog annotation INDEL_CAN Length 4583 nscore 0.00 NPOSITIONS NA**

**Repeat length (main genome) 308**

**Blast2 Results -**

**OFC OLC RFC RLC**

**1 289 1 296**

**Ortholog Repeat Masker annotation**

**SW perc perc perc query position in query matching repeat position in repeat**

**score div. del. ins. sequence begin end (left) repeat class/family begin end (left) ID**

**2298 5.2 3.1 1.0 AluYa5_4_194c 1 289 (4294) + AluY SINE/Alu 1 295 (16) 1**

**199 12.2 9.8 0.0 AluYa5_4_194c 382 463 (4120) + (TATAA)n Simple_repeat 1 90 (0) 2**

**274 27.8 10.9 3.2 AluYa5_4_194c 1238 1393 (3190) + L2a LINE/L2 3245 3412 (14) 3**

**643 32.1 9.8 1.6 AluYa5_4_194c 2025 2799 (1784) + L1M5 LINE/L1 2956 3794 (2352) 4**

**748 25.1 6.5 7.7 AluYa5_4_194c 2876 2924 (1659) + L1MC4 LINE/L1 7710 7758 (284) 5**

**2099 20.7 8.8 0.2 AluYa5_4_194c 2925 3422 (1161) C MER34 LTR/ERV1 (0) 542 2 6**

**748 25.1 6.5 7.7 AluYa5_4_194c 3423 3709 (874) + L1MC4 LINE/L1 7759 8042 (0) 5**

**689 23.4 0.7 1.4 AluYa5_4_194c 3787 3932 (651) + MER5B DNA/MER1_type 2 146 (32) 7**

**1355 23.5 5.3 4.8 AluYa5_4_194c 3933 4289 (294) + MLT1A LTR/MaLR 1 359 (15) 8**

**204 17.9 0.0 2.5 AluYa5_4_194c 4299 4338 (245) C MER5B DNA/MER1_type (139) 39 1 9**

**__________________________________________________________________________________**

**67. AluYa5_4_245**

**Ortholog annotation INDEL_CAN Length 962 nscore 0.00 NPOSITIONS NA**

**Repeat length (main genome) 312**

**Blast2 Results -**

**OFC OLC RFC RLC**

**673 962 23 311**

**Ortholog Repeat Masker annotation**

**SW perc perc perc query position in query matching repeat position in repeat**

**score div. del. ins. sequence begin end (left) repeat class/family begin end (left) ID**

**2325 11.0 0.0 0.6 AluYa5_4_245 651 962 (0) + AluSx SINE/Alu 1 310 (2) 1**

**__________________________________________________________________________________**

**68. AluYa5_4_3**

**Ortholog annotation INDEL_CAN Length 503 nscore 3.98 NPOSITIONS 212 231 ;**

**Repeat length (main genome) 301**

**Blast2 Results -**

**OFC OLC RFC RLC**

**232 503 29 300**

**Ortholog Repeat Masker annotation**

**SW perc perc perc query position in query matching repeat position in repeat**

**score div. del. ins. sequence begin end (left) repeat class/family begin end (left) ID**

**2579 0.7 0.0 0.0 AluYa5_4_3 232 502 (1) + AluYa5 SINE/Alu 29 299 (11) 1**

**__________________________________________________________________________________**

**69. AluYa5_5_10**

**Ortholog annotation INDEL_CAN Length 334 nscore 5.99 NPOSITIONS 1 20 ;**

**Repeat length (main genome) 302**

**Blast2 Results -**

**OFC OLC RFC RLC**

**22 323 1 302**

**Ortholog Repeat Masker annotation**

**SW perc perc perc query position in query matching repeat position in repeat**

**score div. del. ins. sequence begin end (left) repeat class/family begin end (left) ID**

**2707 3.9 0.0 0.0 AluYa5_5_10 22 331 (3) + AluYa5 SINE/Alu 1 310 (0) 1**

**__________________________________________________________________________________**

**70. AluYa5_5_182c**

**Ortholog annotation C_INTER_RMD_M_DISRUPTED Length 340 nscore 11.18 NPOSITIONS 1 38 ;**

**Repeat length (main genome) 296**

**Blast2 Results -**

**OFC OLC RFC RLC**

**45 340 1 296**

**Ortholog Repeat Masker annotation**

**SW perc perc perc query position in query matching repeat position in repeat**

**score div. del. ins. sequence begin end (left) repeat class/family begin end (left) ID**

**2772 1.4 0.0 0.0 AluYa5_5_182c 45 340 (0) + AluYa5 SINE/Alu 1 296 (14) 1**

**__________________________________________________________________________________**

**71. AluYa5_5_222c**

**Ortholog annotation C_INTER_RMD_M_DISRUPTED Length 120 nscore 0.00 NPOSITIONS NA**

**Repeat length (main genome) 312**

**Blast2 Results -**

**OFC OLC RFC RLC**

**1 120 1 120**

**Ortholog Repeat Masker annotation**

**SW perc perc perc query position in query matching repeat position in repeat**

**score div. del. ins. sequence begin end (left) repeat class/family begin end (left) ID**

**916 10.8 0.0 0.0 AluYa5_5_222c 1 120 (0) + AluSc SINE/Alu 1 120 (189) 1**

**__________________________________________________________________________________**

**72. AluYa5_6_177**

**Ortholog annotation C_INTER_RMD_M_DISRUPTED Length 293 nscore 6.83 NPOSITIONS 274 293 ;**

**Repeat length (main genome) 304**

**Blast2 Results -**

**OFC OLC RFC RLC**

**1 273 1 273**

**Ortholog Repeat Masker annotation**

**SW perc perc perc query position in query matching repeat position in repeat**

**score div. del. ins. sequence begin end (left) repeat class/family begin end (left) ID**

**2566 1.1 0.0 0.0 AluYa5_6_177 1 273 (20) + AluYa5 SINE/Alu 1 273 (37) 1**

**__________________________________________________________________________________**

**73. AluYa5_6_204**

**Ortholog annotation C_INTER_RMD_M_DISRUPTED Length 281 nscore 0.00 NPOSITIONS NA**

**Repeat length (main genome) 237**

**Blast2 Results -**

**OFC OLC RFC RLC**

**1 237 1 237**

**Ortholog Repeat Masker annotation**

**SW perc perc perc query position in query matching repeat position in repeat**

**score div. del. ins. sequence begin end (left) repeat class/family begin end (left) ID**

**2661 0.7 0.0 0.0 AluYa5_6_204 1 281 (0) + AluYa5 SINE/Alu 1 281 (29) 1**

**__________________________________________________________________________________**

**74. AluYa5_6_38**

**Ortholog annotation C_INTER_RMD_M_DISRUPTED Length 344 nscore 5.81 NPOSITIONS 11 30 ;**

**Repeat length (main genome) 306**

**Blast2 Results -**

**OFC OLC RFC RLC**

**36 343 1 304**

**Ortholog Repeat Masker annotation**

**SW perc perc perc query position in query matching repeat position in repeat**

**score div. del. ins. sequence begin end (left) repeat class/family begin end (left) ID**

**2933 0.3 0.0 0.0 AluYa5_6_38 36 343 (1) + AluYa5 SINE/Alu 1 308 (2) 1**

**__________________________________________________________________________________**

**75. AluYa5_6_49c**

**Ortholog annotation C_INTER_RMD_M_DISRUPTED Length 124 nscore 16.13 NPOSITIONS 1 20 ;**

**Repeat length (main genome) 284**

**Blast2 Results -**

**OFC OLC RFC RLC**

**27 124 187 284**

**Ortholog Repeat Masker annotation**

**SW perc perc perc query position in query matching repeat position in repeat**

**score div. del. ins. sequence begin end (left) repeat class/family begin end (left) ID**

**829 6.7 1.0 0.0 AluYa5_6_49c 21 124 (0) + AluY SINE/Alu 180 284 (27) 1**

**__________________________________________________________________________________**

**76. AluYa5_6_52**

**Ortholog annotation INDEL_CAN Length 373 nscore 0.00 NPOSITIONS NA**

**Repeat length (main genome) 310**

**Blast2 Results -**

**OFC OLC RFC RLC**

**no hits found**

**Ortholog Repeat Masker annotation**

**SW perc perc perc query position in query matching repeat position in repeat**

**score div. del. ins. sequence begin end (left) repeat class/family begin end (left) ID**

**2319 14.9 1.6 0.6 AluYa5_6_52 1 365 (8) C LTR12 LTR/ERV1 (285) 541 173 1**

**__________________________________________________________________________________**

**77. AluYa5_7_161**

**Ortholog annotation INDEL_CAN Length 608 nscore 3.29 NPOSITIONS 283 302 ;**

**Repeat length (main genome) 309**

**Blast2 Results -**

**OFC OLC RFC RLC**

**1 280 1 280**

**303 602 10 309**

**Ortholog Repeat Masker annotation**

**SW perc perc perc query position in query matching repeat position in repeat**

**score div. del. ins. sequence begin end (left) repeat class/family begin end (left) ID**

**2630 1.4 0.0 0.0 AluYa5_7_161 1 280 (328) + AluYa5 SINE/Alu 2 281 (29) 1**

**1938 11.3 0.0 0.0 AluYa5_7_161 303 602 (6) + AluYa5 SINE/Alu 11 310 (0) 2**

**__________________________________________________________________________________**

**78. AluYa5_7_204c**

**Ortholog annotation C_INTER_RMD_M_DISRUPTED Length 352 nscore 18.18 NPOSITIONS 1 64 ;**

**Repeat length (main genome) 315**

**Blast2 Results -**

**OFC OLC RFC RLC**

**65 352 28 315**

**Ortholog Repeat Masker annotation**

**SW perc perc perc query position in query matching repeat position in repeat**

**score div. del. ins. sequence begin end (left) repeat class/family begin end (left) ID**

**2624 0.4 0.0 1.7 AluYa5_7_204c 65 352 (0) + AluYa5 SINE/Alu 28 310 (0) 1**

**__________________________________________________________________________________**

**79. AluYa5_7_82**

**Ortholog annotation C_INTER_RMD_M_DISRUPTED Length 305 nscore 0.00 NPOSITIONS NA**

**Repeat length (main genome) 307**

**Blast2 Results -**

**OFC OLC RFC RLC**

**1 293 1 293**

**Ortholog Repeat Masker annotation**

**SW perc perc perc query position in query matching repeat position in repeat**

**score div. del. ins. sequence begin end (left) repeat class/family begin end (left) ID**

**2778 2.3 0.0 0.0 AluYa5_7_82 1 304 (1) + AluYa5 SINE/Alu 1 304 (6) 1**

**__________________________________________________________________________________**

**80. AluYa5_8_19**

**Ortholog annotation C_INTER_RMD_M_DISRUPTED Length 210 nscore 0.00 NPOSITIONS NA**

**Repeat length (main genome) 295**

**Blast2 Results -**

**OFC OLC RFC RLC**

**3 210 87 294**

**Ortholog Repeat Masker annotation**

**SW perc perc perc query position in query matching repeat position in repeat**

**score div. del. ins. sequence begin end (left) repeat class/family begin end (left) ID**

**1923 1.0 0.0 0.0 AluYa5_8_19 3 208 (2) + AluYa5 SINE/Alu 87 292 (18) 1**

**__________________________________________________________________________________**

**81. AluYa5_9_103c**

**Ortholog annotation C_INTER_RMD_M_DISRUPTED Length 292 nscore 0.00 NPOSITIONS NA**

**Repeat length (main genome) 310**

**Blast2 Results -**

**OFC OLC RFC RLC**

**1 292 1 292**

**Ortholog Repeat Masker annotation**

**SW perc perc perc query position in query matching repeat position in repeat**

**score div. del. ins. sequence begin end (left) repeat class/family begin end (left) ID**

**2773 0.3 0.0 0.0 AluYa5_9_103c 1 292 (0) + AluYa5 SINE/Alu 1 292 (18) 1**

**__________________________________________________________________________________**

**82. AluYa5_9_172c**

**Ortholog annotation C_INTER_RMD_M_DISRUPTED Length 120 nscore 0.00 NPOSITIONS NA**

**Repeat length (main genome) 314**

**Blast2 Results -**

**OFC OLC RFC RLC**

**1 87 1 87**

**Ortholog Repeat Masker annotation**

**SW perc perc perc query position in query matching repeat position in repeat**

**score div. del. ins. sequence begin end (left) repeat class/family begin end (left) ID**

**928 11.7 0.0 0.0 AluYa5_9_172c 1 120 (0) + AluSq/x SINE/Alu 1 120 (192) 1**

**__________________________________________________________________________________**

**83. AluYa5_9_61**

**Ortholog annotation INDEL_PTS Length 138 nscore 0.00 NPOSITIONS NA**

**Repeat length (main genome) 311**

**Blast2 Results -**

**OFC OLC RFC RLC**

**no hits found**

**Ortholog Repeat Masker annotation**

**SW perc perc perc query position in query matching repeat position in repeat**

**score div. del. ins. sequence begin end (left) repeat class/family begin end (left) ID**

**607 11.6 9.4 0.0 AluYa5_9_61 1 138 (0) + L1M2 LINE/L1 3070 3220 (2923) 1**

**__________________________________________________________________________________**

**84. AluYa5_9_63c**

**Ortholog annotation INDEL_PTS Length 5081 nscore 3.94 NPOSITIONS 1 100 ; 4516 4615 ;**

**Repeat length (main genome) 304**

**Blast2 Results -**

**OFC OLC RFC RLC**

**3504 3621 11 126**

**3515 3543 156 184**

**3685 3714 155 184**

**Ortholog Repeat Masker annotation**

**SW perc perc perc query position in query matching repeat position in repeat**

**score div. del. ins. sequence begin end (left) repeat class/family begin end (left) ID**

**316 18.6 7.0 0.0 AluYa5_9_63c 108 193 (4888) + Charlie23a DNA/MER1_type 160 251 (88) 1**

**1795 22.4 7.0 0.3 AluYa5_9_63c 194 758 (4323) + L1MC4 LINE/L1 7255 7902 (140) 2**

**24 5.3 0.0 0.0 AluYa5_9_63c 920 957 (4124) + AT_rich Low_complexity 1 38 (0) 3**

**2164 11.8 0.0 0.0 AluYa5_9_63c 985 1280 (3801) C AluSx SINE/Alu (16) 296 1 4**

**1569 16.9 3.8 0.0 AluYa5_9_63c 1434 1700 (3381) C AluJo SINE/Alu (17) 295 19 5**

**2035 11.6 12.5 0.3 AluYa5_9_63c 1703 2031 (3050) + MER57B1 LTR/ERV1 5 373 (28) 6**

**3568 18.3 4.8 1.4 AluYa5_9_63c 2032 2836 (2245) + L1M4 LINE/L1 4316 5148 (998) 7**

**386 18.7 0.0 0.0 AluYa5_9_63c 2835 2909 (2172) + L1MA7 LINE/L1 6210 6284 (7) 8 ***

**1910 14.9 0.3 0.0 AluYa5_9_63c 2933 3221 (1860) C AluSx SINE/Alu (22) 290 1 9**

**1191 17.5 4.8 0.0 AluYa5_9_63c 3222 3489 (1592) + L1M4 LINE/L1 5159 5437 (741) 7**

**1089 13.0 0.0 16.3 AluYa5_9_63c 3494 3714 (1367) + AluSq SINE/Alu 1 185 (128) 10**

**__________________________________________________________________________________**

**85. AluYa5_9_65**

**Ortholog annotation INDEL_PTS Length 138 nscore 0.00 NPOSITIONS NA**

**Repeat length (main genome) 311**

**Blast2 Results -**

**OFC OLC RFC RLC**

**no hits found**

**Ortholog Repeat Masker annotation**

**SW perc perc perc query position in query matching repeat position in repeat**

**score div. del. ins. sequence begin end (left) repeat class/family begin end (left) ID**

**607 11.6 9.4 0.0 AluYa5_9_65 1 138 (0) + L1M2 LINE/L1 3070 3220 (2923) 1**

**__________________________________________________________________________________**

**86. AluYa5_9_70**

**Ortholog annotation INDEL_PTS Length 134 nscore 0.00 NPOSITIONS NA**

**Repeat length (main genome) 305**

**Blast2 Results -**

**OFC OLC RFC RLC**

**no hits found**

**Ortholog Repeat Masker annotation**

**SW perc perc perc query position in query matching repeat position in repeat**

**score div. del. ins. sequence begin end (left) repeat class/family begin end (left) ID**

**584 11.9 9.7 0.0 AluYa5_9_70 1 134 (0) + L1M2 LINE/L1 3070 3216 (2927) 1**

**__________________________________________________________________________________**

**87. AluYa5_9_72c**

**Ortholog annotation INDEL_PTS Length 5081 nscore 3.94 NPOSITIONS 1 100 ; 4516 4615 ;**

**Repeat length (main genome) 300**

**Blast2 Results -**

**OFC OLC RFC RLC**

**3504 3621 11 126**

**3515 3543 156 184**

**3685 3714 155 184**

**Ortholog Repeat Masker annotation**

**SW perc perc perc query position in query matching repeat position in repeat**

**score div. del. ins. sequence begin end (left) repeat class/family begin end (left) ID**

**316 18.6 7.0 0.0 AluYa5_9_72c 108 193 (4888) + Charlie23a DNA/MER1_type 160 251 (88) 1**

**1795 22.4 7.0 0.3 AluYa5_9_72c 194 758 (4323) + L1MC4 LINE/L1 7255 7902 (140) 2**

**24 5.3 0.0 0.0 AluYa5_9_72c 920 957 (4124) + AT_rich Low_complexity 1 38 (0) 3**

**2164 11.8 0.0 0.0 AluYa5_9_72c 985 1280 (3801) C AluSx SINE/Alu (16) 296 1 4**

**1569 16.9 3.8 0.0 AluYa5_9_72c 1434 1700 (3381) C AluJo SINE/Alu (17) 295 19 5**

**2035 11.6 12.5 0.3 AluYa5_9_72c 1703 2031 (3050) + MER57B1 LTR/ERV1 5 373 (28) 6**

**3568 18.3 4.8 1.4 AluYa5_9_72c 2032 2836 (2245) + L1M4 LINE/L1 4316 5148 (998) 7**

**386 18.7 0.0 0.0 AluYa5_9_72c 2835 2909 (2172) + L1MA7 LINE/L1 6210 6284 (7) 8 ***

**1910 14.9 0.3 0.0 AluYa5_9_72c 2933 3221 (1860) C AluSx SINE/Alu (22) 290 1 9**

**1191 17.5 4.8 0.0 AluYa5_9_72c 3222 3489 (1592) + L1M4 LINE/L1 5159 5437 (741) 7**

**1089 13.0 0.0 16.3 AluYa5_9_72c 3494 3714 (1367) + AluSq SINE/Alu 1 185 (128) 10**

**__________________________________________________________________________________**

**88. AluYa5_9_82**

**Ortholog annotation INDEL_PTS Length 5081 nscore 3.94 NPOSITIONS 1 100 ; 4516 4615 ;**

**Repeat length (main genome) 302**

**Blast2 Results -**

**OFC OLC RFC RLC**

**3504 3621 11 126**

**3515 3543 156 184**

**3685 3714 155 184**

**Ortholog Repeat Masker annotation**

**SW perc perc perc query position in query matching repeat position in repeat**

**score div. del. ins. sequence begin end (left) repeat class/family begin end (left) ID**

**316 18.6 7.0 0.0 AluYa5_9_82 108 193 (4888) + Charlie23a DNA/MER1_type 160 251 (88) 1**

**1795 22.4 7.0 0.3 AluYa5_9_82 194 758 (4323) + L1MC4 LINE/L1 7255 7902 (140) 2**

**24 5.3 0.0 0.0 AluYa5_9_82 920 957 (4124) + AT_rich Low_complexity 1 38 (0) 3**

**2164 11.8 0.0 0.0 AluYa5_9_82 985 1280 (3801) C AluSx SINE/Alu (16) 296 1 4**

**1569 16.9 3.8 0.0 AluYa5_9_82 1434 1700 (3381) C AluJo SINE/Alu (17) 295 19 5**

**2035 11.6 12.5 0.3 AluYa5_9_82 1703 2031 (3050) + MER57B1 LTR/ERV1 5 373 (28) 6**

**3568 18.3 4.8 1.4 AluYa5_9_82 2032 2836 (2245) + L1M4 LINE/L1 4316 5148 (998) 7**

**386 18.7 0.0 0.0 AluYa5_9_82 2835 2909 (2172) + L1MA7 LINE/L1 6210 6284 (7) 8 ***

**1910 14.9 0.3 0.0 AluYa5_9_82 2933 3221 (1860) C AluSx SINE/Alu (22) 290 1 9**

**1191 17.5 4.8 0.0 AluYa5_9_82 3222 3489 (1592) + L1M4 LINE/L1 5159 5437 (741) 7**

**1089 13.0 0.0 16.3 AluYa5_9_82 3494 3714 (1367) + AluSq SINE/Alu 1 185 (128) 10**

**__________________________________________________________________________________**

**89. AluYa5_9_85**

**Ortholog annotation INDEL_PTS Length 5081 nscore 3.94 NPOSITIONS 1 100 ; 4516 4615 ;**

**Repeat length (main genome) 302**

**Blast2 Results -**

**OFC OLC RFC RLC**

**3504 3621 11 126**

**3515 3543 156 184**

**3685 3714 155 184**

**Ortholog Repeat Masker annotation**

**SW perc perc perc query position in query matching repeat position in repeat**

**score div. del. ins. sequence begin end (left) repeat class/family begin end (left) ID**

**316 18.6 7.0 0.0 AluYa5_9_85 108 193 (4888) + Charlie23a DNA/MER1_type 160 251 (88) 1**

**1795 22.4 7.0 0.3 AluYa5_9_85 194 758 (4323) + L1MC4 LINE/L1 7255 7902 (140) 2**

**24 5.3 0.0 0.0 AluYa5_9_85 920 957 (4124) + AT_rich Low_complexity 1 38 (0) 3**

**2164 11.8 0.0 0.0 AluYa5_9_85 985 1280 (3801) C AluSx SINE/Alu (16) 296 1 4**

**1569 16.9 3.8 0.0 AluYa5_9_85 1434 1700 (3381) C AluJo SINE/Alu (17) 295 19 5**

**2035 11.6 12.5 0.3 AluYa5_9_85 1703 2031 (3050) + MER57B1 LTR/ERV1 5 373 (28) 6**

**3568 18.3 4.8 1.4 AluYa5_9_85 2032 2836 (2245) + L1M4 LINE/L1 4316 5148 (998) 7**

**386 18.7 0.0 0.0 AluYa5_9_85 2835 2909 (2172) + L1MA7 LINE/L1 6210 6284 (7) 8 ***

**1910 14.9 0.3 0.0 AluYa5_9_85 2933 3221 (1860) C AluSx SINE/Alu (22) 290 1 9**

**1191 17.5 4.8 0.0 AluYa5_9_85 3222 3489 (1592) + L1M4 LINE/L1 5159 5437 (741) 7**

**1089 13.0 0.0 16.3 AluYa5_9_85 3494 3714 (1367) + AluSq SINE/Alu 1 185 (128) 10**

**__________________________________________________________________________________**

**90. AluYa5_9_94c**

**Ortholog annotation C_DISRUPTED_M_INTER_RMD Length 502 nscore 3.98 NPOSITIONS 168 187 ;**

**Repeat length (main genome) 310**

**Blast2 Results -**

**OFC OLC RFC RLC**

**1 53 1 53**

**193 502 1 310**

**Ortholog Repeat Masker annotation**

**SW perc perc perc query position in query matching repeat position in repeat**

**score div. del. ins. sequence begin end (left) repeat class/family begin end (left) ID**

**1113 15.0 0.0 0.0 AluYa5_9_94c 1 167 (335) + AluSx SINE/Alu 1 167 (145) 1**

**2803 1.3 0.0 0.7 AluYa5_9_94c 193 502 (0) + AluYa5 SINE/Alu 1 308 (2) 2**

**__________________________________________________________________________________**

**91. AluYa5_X_105c**

**Ortholog annotation C_INTER_RMD_M_DISRUPTED Length 334 nscore 5.99 NPOSITIONS 1 20 ;**

**Repeat length (main genome) 310**

**Blast2 Results -**

**OFC OLC RFC RLC**

**27 334 3 310**

**Ortholog Repeat Masker annotation**

**SW perc perc perc query position in query matching repeat position in repeat**

**score div. del. ins. sequence begin end (left) repeat class/family begin end (left) ID**

**2622 4.9 0.0 0.0 AluYa5_X_105c 27 334 (0) + AluYa5 SINE/Alu 3 310 (0) 1**

**__________________________________________________________________________________**

**92. AluYa5_X_29c**

**Ortholog annotation C_INTER_RMD_M_DISRUPTED Length 172 nscore 11.63 NPOSITIONS 153 172 ;**

**Repeat length (main genome) 151**

**Blast2 Results -**

**OFC OLC RFC RLC**

**1 151 1 151**

**Ortholog Repeat Masker annotation**

**SW perc perc perc query position in query matching repeat position in repeat**

**score div. del. ins. sequence begin end (left) repeat class/family begin end (left) ID**

**1324 3.3 0.0 0.0 AluYa5_X_29c 1 151 (21) + AluYa5 SINE/Alu 155 305 (5) 1**

**__________________________________________________________________________________**

**93. AluYa5_X_37c**

**Ortholog annotation C_INTER_RMD_M_DISRUPTED Length 336 nscore 7.44 NPOSITIONS 1 25 ;**

**Repeat length (main genome) 272**

**Blast2 Results -**

**OFC OLC RFC RLC**

**65 336 1 272**

**Ortholog Repeat Masker annotation**

**SW perc perc perc query position in query matching repeat position in repeat**

**score div. del. ins. sequence begin end (left) repeat class/family begin end (left) ID**

**2519 1.1 0.0 0.0 AluYa5_X_37c 65 336 (0) + AluYa5 SINE/Alu 39 310 (0) 1**

**__________________________________________________________________________________**

**94. AluYa5_X_47c**

**Ortholog annotation INDEL_PTS Length 5138 nscore 15.59 NPOSITIONS 3 596 ; 2607 2813 ;**

**Repeat length (main genome) 310**

**Blast2 Results -**

**OFC OLC RFC RLC**

**2345 2606 1 259**

**3142 3180 15 53**

**3148 3185 155 192**

**3720 3920 99 300**

**3931 4230 11 310**

**Ortholog Repeat Masker annotation**

**SW perc perc perc query position in query matching repeat position in repeat**

**score div. del. ins. sequence begin end (left) repeat class/family begin end (left) ID**

**725 10.8 0.0 0.0 AluYa5_X_47c 598 699 (4439) C AluS SINE/Alu (210) 102 1 1**

**472 12.5 0.0 0.0 AluYa5_X_47c 703 774 (4364) + U3 snRNA 1 72 (145) 2**

**431 28.0 8.2 0.6 AluYa5_X_47c 837 994 (4144) C MLT1I LTR/MaLR (234) 177 8 3**

**244 20.0 22.1 1.2 AluYa5_X_47c 1208 1293 (3845) + MIR SINE/MIR 158 261 (1) 4**

**2079 12.0 0.3 0.3 AluYa5_X_47c 1728 2020 (3118) C AluSx SINE/Alu (15) 297 5 5**

**189 28.6 2.1 5.2 AluYa5_X_47c 2073 2168 (2970) + L2 LINE/L2 2489 2581 (838) 6**

**331 24.3 0.0 0.0 AluYa5_X_47c 2270 2339 (2799) + MER5A1 DNA/MER1_type 2 71 (95) 7**

**2036 9.2 0.0 0.8 AluYa5_X_47c 2345 2606 (2532) + AluSx SINE/Alu 1 260 (52) 8**

**2169 6.6 0.0 0.0 AluYa5_X_47c 2814 3071 (2067) C AluY SINE/Alu (53) 258 1 9**

**327 18.3 0.0 0.0 AluYa5_X_47c 3072 3131 (2007) + MER5A1 DNA/MER1_type 103 162 (4) 7**

**1723 14.2 1.4 1.4 AluYa5_X_47c 3138 3422 (1716) + AluJo SINE/Alu 11 295 (17) 10**

**187 27.1 2.1 0.0 AluYa5_X_47c 3506 3553 (1585) + MER5B DNA/MER1_type 1 49 (129) 11**

**2215 12.2 0.0 0.0 AluYa5_X_47c 3620 3921 (1217) + AluSx SINE/Alu 1 302 (10) 12**

**2411 7.8 0.3 0.0 AluYa5_X_47c 3925 4231 (907) + AluY SINE/Alu 4 311 (0) 13**

**21 0.0 0.0 0.0 AluYa5_X_47c 4291 4311 (827) + AT_rich Low_complexity 1 21 (0) 14**

**767 13.5 0.0 0.0 AluYa5_X_47c 4321 4431 (707) C FLAM_C SINE/Alu (20) 123 13 15**

**1956 13.4 0.7 0.3 AluYa5_X_47c 4438 4728 (410) C AluJb SINE/Alu (16) 296 5 16**

**1435 4.2 1.5 4.6 AluYa5_X_47c 4775 4793 (345) C AluSq SINE/Alu (0) 313 295 17**

**734 8.3 3.7 0.0 AluYa5_X_47c 4794 4902 (236) C L1PB1 LINE/L1 (0) 6151 6039 18**

**1435 4.2 1.5 4.6 AluYa5_X_47c 4903 5079 (59) C AluSq SINE/Alu (19) 294 123 17**

**323 12.1 0.0 0.0 AluYa5_X_47c 5081 5138 (0) C AluSc SINE/Alu (0) 299 242 19**

**__________________________________________________________________________________**

**95. AluYa5_X_4c**

**Ortholog annotation INDEL_CAN Length 864 nscore 2.31 NPOSITIONS 6 25 ;**

**Repeat length (main genome) 282**

**Blast2 Results -**

**OFC OLC RFC RLC**

**583 864 1 282**

**Ortholog Repeat Masker annotation**

**SW perc perc perc query position in query matching repeat position in repeat**

**score div. del. ins. sequence begin end (left) repeat class/family begin end (left) ID**

**2652 1.1 0.0 0.0 AluYa5_X_4c 583 864 (0) + AluYa5 SINE/Alu 22 303 (7) 1**

**__________________________________________________________________________________**

**96. AluYa5_Y_19c**

**Ortholog annotation INDEL_PTS Length 2442 nscore 0.00 NPOSITIONS NA**

**Repeat length (main genome) 310**

**Blast2 Results -**

**OFC OLC RFC RLC**

**no hits found**

**Ortholog Repeat Masker annotation**

**SW perc perc perc query position in query matching repeat position in repeat**

**score div. del. ins. sequence begin end (left) repeat class/family begin end (left) ID**

**1001 10.6 0.6 0.3 AluYa5_Y_19c 456 2439 (3) C BSR/Beta Satellite (47) 1989 1 1**

**__________________________________________________________________________________**

**97. AluYa5_Y_30**

**Ortholog annotation C_INTER_RMD_M_DISRUPTED Length 336 nscore 5.95 NPOSITIONS 1 20 ;**

**Repeat length (main genome) 310**

**Blast2 Results -**

**OFC OLC RFC RLC**

**28 336 1 309**

**Ortholog Repeat Masker annotation**

**SW perc perc perc query position in query matching repeat position in repeat**

**score div. del. ins. sequence begin end (left) repeat class/family begin end (left) ID**

**2827 2.6 0.0 0.0 AluYa5_Y_30 28 336 (0) + AluYa5 SINE/Alu 1 309 (1) 1**

**__________________________________________________________________________________**

**98. AluYa5_Y_63c**

**Ortholog annotation INDEL_PTS Length 4320 nscore 5.60 NPOSITIONS 3711 3952 ;**

**Repeat length (main genome) 303**

**Blast2 Results -**

**OFC OLC RFC RLC**

**Ortholog Repeat Masker annotation**

**SW perc perc perc query position in query matching repeat position in repeat**

**score div. del. ins. sequence begin end (left) repeat class/family begin end (left) ID**

**214 18.6 0.0 0.0 AluYa5_Y_63c 840 882 (3438) + A-rich Low_complexity 2 44 (0) 1**

**609 27.4 8.5 1.0 AluYa5_Y_63c 963 1161 (3159) + L1MB5 LINE/L1 5949 6162 (12) 2**

**29 0.0 0.0 0.0 AluYa5_Y_63c 1383 1411 (2909) + AT_rich Low_complexity 1 29 (0) 3**

**25 6.5 0.0 0.0 AluYa5_Y_63c 1955 2000 (2320) + AT_rich Low_complexity 1 46 (0) 4**

**553 25.8 1.0 9.4 AluYa5_Y_63c 2441 2641 (1679) C AluJo SINE/Alu (117) 195 12 5**

**461 29.1 0.9 0.0 AluYa5_Y_63c 2667 2776 (1544) C FLAM_C SINE/Alu (10) 123 13 6**

**__________________________________________________________________________________**

**99. AluYa5_Y_65c**

**Ortholog annotation INDEL_CAN Length 988 nscore 2.02 NPOSITIONS 50 69 ;**

**Repeat length (main genome) 303**

**Blast2 Results -**

**OFC OLC RFC RLC**

**432 486 10 64**

**502 735 65 297**

**578 605 6 33**

**740 988 5 253**

**Ortholog Repeat Masker annotation**

**SW perc perc perc query position in query matching repeat position in repeat**

**score div. del. ins. sequence begin end (left) repeat class/family begin end (left) ID**

**2335 10.2 3.4 0.0 AluYa5_Y_65c 70 422 (566) + L1M1 LINE/L1 3426 3790 (2356) 1**

**2189 10.4 0.0 4.8 AluYa5_Y_65c 423 735 (253) + AluSx SINE/Alu 1 298 (14) 2**

**2346 1.2 0.0 0.0 AluYa5_Y_65c 736 988 (0) + AluYa5 SINE/Alu 1 253 (57) 3**

**__________________________________________________________________________________**

**100. AluYa5_Y_66c**

**Ortholog annotation C_INTER_RMD_M_DISRUPTED Length 326 nscore 6.13 NPOSITIONS 307 326 ;**

**Repeat length (main genome) 301**

**Blast2 Results -**

**OFC OLC RFC RLC**

**1 300 1 300**

**Ortholog Repeat Masker annotation**

**SW perc perc perc query position in query matching repeat position in repeat**

**score div. del. ins. sequence begin end (left) repeat class/family begin end (left) ID**

**2876 0.0 0.0 0.0 AluYa5_Y_66c 1 300 (26) + AluYa5 SINE/Alu 1 300 (10) 1**

**__________________________________________________________________________________**

**101. AluYa5_Y_8c**

**Ortholog annotation C_INTER_RMD_M_DISRUPTED Length 283 nscore 7.07 NPOSITIONS 264 283 ;**

**Repeat length (main genome) 308**

**Blast2 Results -**

**OFC OLC RFC RLC**

**1 263 1 263**

**Ortholog Repeat Masker annotation**

**SW perc perc perc query position in query matching repeat position in repeat**

**score div. del. ins. sequence begin end (left) repeat class/family begin end (left) ID**

**2433 0.8 0.0 0.4 AluYa5_Y_8c 1 263 (20) + AluYa5 SINE/Alu 1 262 (48) 1**

**__________________________________________________________________________________**
