# Supplementary material for: RISCI - Repeat Induced Sequence Changes Identifier: a comprehensive, comparative genomics-based, in silico subtractive hybridization pipeline to identify repeat induced sequence changes in closely related genomes
Source: BMC Bioinformatics. 2010 Dec 26;11:609. doi: 10.1186/1471-2105-11-609 (PMC3024322; doi:10.1186/1471-2105-11-609)
Supplement: Additional file 10 — 3' flank transduction results for AluYa5 (Reference human vs chimpanzee). Output file of the 3' flank transduction confirmation module of RISCI - includes the putative transduced flank in EMBL format, RepeatMasker annotation for the same and BLAST hits in reference and comparative genome. [file 1471-2105-11-609-S10.DOC]

**Additional file 10 – 3’ flank transduction results for AluYa5**

Compiled 3’ flank trnsduction file generated by RISCI for AluYa5 reference human vs Chimpanzee comparisons.

The putative transduced flank is printed in EMBL format, followed by RepeatMasker annotation, the number of blast hits obtained in the main genome, non redundandant hits if any, number of blast hits obtained in the comparative genome and the non redundant hits, if any.

1 . AluYa5_10_120

SEQUENCE IN EMBL FORMAT

ID AluYa5_10_120; SV 1; linear; unassigned DNA; STD; UNC; 2327 BP.

XX

DE PTS length 2327

XX

SQ Sequence 2327 BP; 795 A; 475 C; 419 G; 638 T; 0 other;

TCTTTAATCC ATCTTGAATT GATTTTTGTA TAAGGTGTAA GGAAGGGATC CAGTTTCAGC 60

TTTCTACATA TGGCTAGCCA GTTTTCCCAG CACCATTTAT TAAATAGGGA ATCCTTTCCC 120

CATTGCTTGT TTTTCTCAGG TTTGTCAAAG ATCAGATAGT TGTAGGTATG CGGCGTTATT 180

TCTGAGGGCT CTGTTCTGTT CCATTGATCT ATATCTCTGT TTTGGTACCA GTACCATGCT 240

GTTTTGGTTA CTGTAGCCTT GTAGTATAGT TTGAAGTCAG GTAGTGTGAT GCCTCCAGCT 300

TTGTTCTTTT GGCTTAGGAT TGACTTGATG ATGCGGGCTC TTTTTTGGTT CCATATGAAC 360

TTTAAAGTAG TTTTCTCCAA TTCTGTGAAG AAAGTCATTG GTAGCTTGAT GGAGATGGCA 420

TTGAATCTGT AAATTACCTT GGGCAGTATG GCCATTTTCA CAATATTGAT TCTTCCTACC 480

CATGAGCATG GAATGTTCTT ccatttgttt gtatcctctt ttatttcctt gagcagtggt 540

ttgtagttct ccttgaagag gtccttcaca tcccttgtaa gttggattcc taggtatttt 600

attctctttg aagcaattgt gaatgggagt tcactcatga ttcggctctc tgtttgtctg 660

ttgttggtgt ataagaatgc ttgtgatttt tgtacattga ttttgtatcc tgagactttg 720

ctgaagttgc ttatcagctt aaggagattt tgggctgaga cgatggggtt ttctagataa 780

acaatcatgt cgtctgcaaa cagggacaat ttgacttcct cttttcctaa ttgaATCCCC 840

TTTATTTCCT TCTCCTGCCT GATTGCCCTG GCCAGAACTT CCAAATCAAC AGAATATACA 900

TTTTTTTCAG CACCACACCA CAcctattcc aaaattgacc acatagttgg aagtaaagct 960

ctcctcagca aatgtaaaag aacagaaatt ataacaaact atctctcaga ccacagtgca 1020

atcaaactag aactcaggat taagaatctc actcaaaact gctcaactac atggaaactg 1080

aacaacctgc tcctgaatga ctactgggta tataacgaaa tgaaggcaga aataaagatg 1140

ttctttgaaa ccaacgagaa caaagacaca acataccaga atctctggga cgcattcaaa 1200

gcagtgtgta gagggaaatt tatagcacta aatgcccaca agagaaagca ggaaagatcc 1260

aaaattgaca ccctaacatc acaattaaaa gaactagaaa agcaagagca aacacattca 1320

aaagctagca gaaggcaaga aataactaaa atcagagcag aactgaagga aatagagaca 1380

caaaaaaccc ttcaaaaaat caatgaatcc aggagctggt tttttgaaag gatcaacaaa 1440

attgatagac cactagcaag actaataaag aaaaaaagag agaagaatca aatagacaca 1500

ataaaaaatg ataaagggga tatcaccact gatcccacag aaatacaaac taccatcaga 1560

gaatactaca aacacctcta cacaaataaa ctagaaaatc tagaagaaat ggatacattc 1620

ctcgacacat acactctccc aagactaaac caggaagaag ttgaatctct gaatagacca 1680

ataacaggag ctgaaattgt ggcaataatc aatagtttac caaccaaaaa gagtccggga 1740

ccagatggat tcacagccga attctaccag aggtacaagg aggaactggt accattcctt 1800

ctgaaactat tccaatcaat agaaaaagag ggaatcctcc ctaactcatt ttatgaggcc 1860

agcatcattc tgataccaaa gccgggcaga gacacaacca aaaaagagaa ttttagacca 1920

atatccttga tgaacattca tgcaaaaatc ctcaataaaa tactggcaaa ccgaatccag 1980

cagcacatca aaaagcttat ccaccatgat caagtgggct tcatccctgg gatgcaaggc 2040

tggttcaata tatgcaaatc aataaatgta atccagcata taaacagagc caaagacaaa 2100

aaccacatca ttatctcaat agatgcagaa aaagcccttg acaaaattca acaacccttc 2160

atgctaaaaa ctctcaataa attaggtatt gatgggatgt atttcaaaat aataagagct 2220

atctatgaca aacccacagc caatatcata ctgaatgggc aaaaactgga agcattccct 2280

ttgAAAACTG GCACAAGACA GGGATGCCCT CTCTCACCGC TCCTATT 2327

//

REPEAT MASKER ANNOTATION

SW perc perc perc query position in query matching repeat position in repeat

score div. del. ins. sequence begin end (left) repeat class/family begin end (left) ID

7733 1.2 0.0 0.0 AluYa5_10_120 1 884 (1443) C L1P1 LINE/L1 (1140) 5006 4123 1 *

12427 1.2 0.4 0.0 AluYa5_10_120 873 2327 (0) + L1P1 LINE/L1 2652 4112 (2034) 1

PTS BLAST RESULTS

GENOME FILENAME CHR CONTIG ORIEN E-VAL LEN1 LEN2 QFC QSC SFC SSC

human AluYa5_10_120 4978

human AluYa5_10_120 10 NC_000010 Plus 0.0 2327 2327 1 2327 96567784 96570110

Chimp AluYa5_10_120 7305

__________________________________________________________________________________

2 . AluYa5_10_28c

SEQUENCE IN EMBL FORMAT

ID AluYa5_10_28c; SV 1; linear; unassigned DNA; STD; UNC; 96 BP.

XX

DE PTS length 96

XX

SQ Sequence 96 BP; 32 A; 15 C; 35 G; 14 T; 0 other;

AGAGAGAGAG AGAGAGAGAG AGAGAAAACA GGCAAACAGG TTGGGTACGG GTACGGTGGC 60

TTACGCCTGT AATCCCAGTA CTTTGGGAGG CCGAAA 96

//

REPEAT MASKER ANNOTATION

SW perc perc perc query position in query matching repeat position in repeat

score div. del. ins. sequence begin end (left) repeat class/family begin end (left) ID

225 0.0 0.0 0.0 AluYa5_10_28c 1 25 (71) + (GA)n Simple_repeat 2 26 (0) 1

374 8.5 0.0 0.0 AluYa5_10_28c 48 94 (2) + Alu SINE/Alu 4 50 (252) 2

PTS BLAST RESULTS

GENOME FILENAME CHR CONTIG ORIEN E-VAL LEN1 LEN2 QFC QSC SFC SSC

human AluYa5_10_28c 740

human AluYa5_10_28c 10 NC_000010 Minus 9e-47 96 96 1 96 27262941 27262846

Chimp AluYa5_10_28c 796

Chimp AluYa5_10_28c 10 NC_006477 Minus 3e-28 82 88 9 96 27512497 27512416

Chimp AluYa5_10_28c 10 NC_006477 Minus 3e-28 82 88 9 96 27513582 27513501

__________________________________________________________________________________

3 . AluYa5_10_7c

SEQUENCE IN EMBL FORMAT

ID AluYa5_10_7c; SV 1; linear; unassigned DNA; STD; UNC; 113 BP.

XX

DE PTS length 113

XX

SQ Sequence 113 BP; 36 A; 25 C; 23 G; 29 T; 0 other;

TGCAGTCAGC CTCTAGAAGC TTGAGAATCA AGGACCCCCG GAAGTAATGC AGACCTACCA 60

ACATCCGGGT TTTAGACTTA TGACCTTCAG ATATGTGAGA AAATACATTA TTT 113

//

REPEAT MASKER ANNOTATION

SW perc perc perc query position in query matching repeat position in repeat

score div. del. ins. sequence begin end (left) repeat class/family begin end (left) ID

247 31.4 0.9 3.8 AluYa5_10_7c 4 109 (4) + MLT1B LTR/MaLR 226 328 (62) 1

PTS BLAST RESULTS

GENOME FILENAME CHR CONTIG ORIEN E-VAL LEN1 LEN2 QFC QSC SFC SSC

human AluYa5_10_7c 1

human AluYa5_10_7c 10 NC_000010 Minus 8e-57 113 113 1 113 9559828 9559716

Chimp AluYa5_10_7c 0

__________________________________________________________________________________

4 . AluYa5_12_108

SEQUENCE IN EMBL FORMAT

ID AluYa5_12_108; SV 1; linear; unassigned DNA; STD; UNC; 458 BP.

XX

DE PTS length 458

XX

SQ Sequence 458 BP; 165 A; 120 C; 53 G; 120 T; 0 other;

GAGTCATCAC CACTCCCTAA TCTCAAGTAC CCAGGGACAC AAACACTGCG GAAGGCCGCA 60

GGGTCCTCTG CATAGGAAAA CCAGAGACCT TTGTTCACTT GTTTATCTGC TGACCCTCCC 120

TCCACTATTG TCCTATGACC CTGCCAAATC CCCCTCTGTG AGAAACACCC AAGAATGATC 180

AATAAAAAAA TAAAAATAAA AATAAAAATA AACAAAAACA AAACTGGACA CCCTACTACC 240

CATACCCAGT TTAAGATACA GATTACAACC AACACCGTTA AAGCCCTTTT GCATGCCCTT 300

CTCCATCCCA GCCCCCTCCT AAATTTTGTT TATAATGATC TCGCTTTTCT TCATAATTTT 360

ACCTCCAAAA TATGCATCTG TAAACAATAT GCTGTTTTTG CAAGCTTTTG AACATTATAT 420

AAAATAAATC ATACTGCATA TAAAAAAATA AAATAAAA 458

//

REPEAT MASKER ANNOTATION

SW perc perc perc query position in query matching repeat position in repeat

score div. del. ins. sequence begin end (left) repeat class/family begin end (left) ID

1821 2.5 0.0 0.0 AluYa5_12_108 1 202 (256) + SVA_D Other 1184 1385 (1) 1

454 22.1 8.2 0.7 AluYa5_12_108 297 442 (16) C L1ME3B LINE/L1 (183) 6057 5901 2

PTS BLAST RESULTS

GENOME FILENAME CHR CONTIG ORIEN E-VAL LEN1 LEN2 QFC QSC SFC SSC

human AluYa5_12_108 260

human AluYa5_12_108 12 NC_000012 Plus 0.0 458 458 1 458 74623248 74623705

human AluYa5_12_108 19 NC_000019 Plus 0.0 437 453 1 453 6307297 6307745

human AluYa5_12_108 1 NC_000001 Plus 0.0 436 453 1 453 24052955 24053404

Chimp AluYa5_12_108 225

Chimp AluYa5_12_108 2A NC_006469 Minus e-117 219 220 220 439 87431931 87431712

__________________________________________________________________________________

5 . AluYa5_14_48c

SEQUENCE IN EMBL FORMAT

ID AluYa5_14_48c; SV 1; linear; unassigned DNA; STD; UNC; 1290 BP.

XX

DE PTS length 1290

XX

SQ Sequence 1290 BP; 254 A; 264 C; 267 G; 505 T; 0 other;

CCTGCTCCTG GATTCATATA ATTTTTGGAG GGTTTTTCAT GTCTCTATCT CATTCAATTC 60

TTCTCTGATC TTAGTTATTT CTTGTCTTCT GCTAGCTTTT GGATTAGTTT GCTCTTGCCT 120

CTCTAGCTCT TTTAATTATG ATGTTAAAGT GTCAATGTGA GATCTTTCTA GCTTTCTGAG 180

GTAGGCATTT AGTGCTATAA ATTTTCTTCT TAACACTGCT TTAGCTGTGT CTCAGGGATT 240

CTGCTGTGTT GTCTCTTTGT ACTCATTGGT TTCAAAGAAC TTCTTGATTT CTGCCTTAAT 300

TTCATTATTT ACACAGGAGT CATTCAGGAG GAGGTTGTTC AATTTCCATG AAATTGTGTG 360

CTTTTGAGTG AGTTTCTTAA TCTTGAGTTC AAATTTGATT GCATTGTGGT CTGAGAGACT 420

GTTATGATTT CAGTTATTTT GCATTTATTG AGGAGTATTT TACTTCCAAT TGTGTGGTCG 480

ATTTTAGAAT AAGTGCCATG agcactgaga agaatgtaca ttctgttgat ttggggtaga 540

gagttctgta gacgtctacc aggtacactt gatccagagc tgagttcaag tcctgaatat 600

ccttgttaat tttctgtctt gttgatctgt ctaatactga ctggggtgtt aaagtctccc 660

actatcattg tgtgggagtc tgtctctttg taggtctcta agaacttgtt ttattgggtg 720

cccctgtatt gggtgcatat atatttataa tagttagctc ttcttcttga attgttcctt 780

ttaccattat gtaatgccct ctttatcctt tttgatcTTT GTCGGTTTAA AGTCTGTTTT 840

GTTAGAGCCT AGGATTGCAA CCCCTGCTTT TTTTTTTTTA TTTCTGAGAT GCAGTCTTGC 900

TCTCTCACCC AGGCTGGagt gcagtggcac gaacttagct cactgcaacc tctgcctccc 960

agttcaagag attctcctgc ctcagcttcc ctagtagcta ggattacagg tgcccaccgc 1020

catgcccagc taatttttgt attttttgta gagacggggt ttcaccatgt tggccagcct 1080

ggtcaagagc tgaactcctg acctctggtg atccgccccc cTCAGCCTCC CAAGGTGCTG 1140

GGATTACAGG TGTGAGCCAC CATGCCTGGC CACCCCTGCT TTTTTTTTGC TTTCCATTTA 1200

CTTGGTAAAT TTTCCTCCAA Ccttttattt ggagcctgtg tgtgtctttg cacataagtt 1260

gggtctcctg aatacagcac atcgatgggt 1290

//

REPEAT MASKER ANNOTATION

SW perc perc perc query position in query matching repeat position in repeat

score div. del. ins. sequence begin end (left) repeat class/family begin end (left) ID

6872 9.0 1.5 1.0 AluYa5_14_48c 1 867 (423) C L1P3 LINE/L1 (2942) 3204 2332 1

2086 11.7 0.3 1.6 AluYa5_14_48c 868 1171 (119) C AluSx SINE/Alu (12) 300 1 2

6872 9.0 1.5 1.0 AluYa5_14_48c 1172 1290 (0) C L1P3 LINE/L1 (3815) 2331 2213 1

PTS BLAST RESULTS

GENOME FILENAME CHR CONTIG ORIEN E-VAL LEN1 LEN2 QFC QSC SFC SSC

human AluYa5_14_48c 78

human AluYa5_14_48c 14 NC_000014 Minus 0.0 1290 1290 1 1290 50671152 50669863

human AluYa5_14_48c 14 NC_000014 Minus 3e-40 226 271 893 1162 83240839 83240574

human AluYa5_14_48c X NC_000023 Minus 4e-52 222 259 909 1166 53550506 53550253

human AluYa5_14_48c 2 NC_000002 Plus 6e-51 221 257 905 1160 215510205 215510455

human AluYa5_14_48c 2 NC_000002 Plus 2e-35 180 210 871 1077 32694208 32694416

human AluYa5_14_48c 2 NC_000002 Plus 2e-32 216 262 900 1160 73275356 73275612

Chimp AluYa5_14_48c 72

Chimp AluYa5_14_48c 14 NC_006481 Minus 0.0 550 564 727 1290 50341857 50341296

Chimp AluYa5_14_48c 14 NC_006481 Plus 0.0 759 885 5 874 86047003 86047886

Chimp AluYa5_14_48c 14 NC_006481 Plus 0.0 760 889 4 874 88492850 88493737

Chimp AluYa5_14_48c 14 NC_006481 Minus 2e-29 242 297 869 1164 77429556 77429266

Chimp AluYa5_14_48c 2B NC_006470 Minus 3e-37 192 226 938 1162 168244574 168244355

Chimp AluYa5_14_48c X NC_006491 Minus 1e-49 221 259 909 1166 53933088 53932835

Chimp AluYa5_14_48c X NC_006491 Minus 4e-46 231 273 900 1171 17790549 17790283

__________________________________________________________________________________

6 . AluYa5_18_101c

SEQUENCE IN EMBL FORMAT

ID AluYa5_18_101c; SV 1; linear; unassigned DNA; STD; UNC; 21 BP.

XX

DE PTS length 21

XX

SQ Sequence 21 BP; 8 A; 2 C; 4 G; 7 T; 0 other;

TGAAAATACA TGGATTGACT T 21

//

REPEAT MASKER ANNOTATION

There were no repetitive sequences detected in /home/vipin/WHOLE_GENOME_CG/AluYa5_CHR/Chimp/CONFIRMATION/AluYa5_3PTS/AluYa5_18_101c

PTS BLAST RESULTS

GENOME FILENAME CHR CONTIG ORIEN E-VAL LEN1 LEN2 QFC QSC SFC SSC

human AluYa5_18_101c 20

human AluYa5_18_101c 18 NC_000018 Minus 0.003 21 21 1 21 68223963 68223943

Chimp AluYa5_18_101c 4

Chimp AluYa5_18_101c 2A NC_006469 Plus 0.16 18 18 1 18 86889579 86889596

Chimp AluYa5_18_101c X NC_006491 Minus 0.65 17 17 5 21 137005539 137005523

Chimp AluYa5_18_101c 13 NC_006480 Minus 0.65 17 17 5 21 108067768 108067752

Chimp AluYa5_18_101c 13 NC_006480 Minus 2.6 16 16 1 16 69750919 69750904

__________________________________________________________________________________

7 . AluYa5_18_28c

SEQUENCE IN EMBL FORMAT

ID AluYa5_18_28c; SV 1; linear; unassigned DNA; STD; UNC; 143 BP.

XX

DE PTS length 143

XX

SQ Sequence 143 BP; 52 A; 19 C; 20 G; 52 T; 0 other;

GAACTTAGCT TTGAAGACAA TGCATTCTTA ATATTTCAAA CACAGAAGCT TTAAGAAAAG 60

AACTAATTTT TAAAAGTTTC ACATCATTTG TGACATTATA AATCAGCTTT TCTTGGTAGT 120

ATTCCAGAAA GTTATGGTTA ATT 143

//

REPEAT MASKER ANNOTATION

There were no repetitive sequences detected in /home/vipin/WHOLE_GENOME_CG/AluYa5_CHR/Chimp/CONFIRMATION/AluYa5_3PTS/AluYa5_18_28c

PTS BLAST RESULTS

GENOME FILENAME CHR CONTIG ORIEN E-VAL LEN1 LEN2 QFC QSC SFC SSC

human AluYa5_18_28c 2

human AluYa5_18_28c 18 NC_000018 Minus 1e-74 143 143 1 143 27130381 27130239

human AluYa5_18_28c 18 NC_000018 Minus 3e-69 134 134 10 143 27130238 27130105

Chimp AluYa5_18_28c 1

Chimp AluYa5_18_28c 18 NC_006485 Minus 8e-70 141 143 1 143 27194027 27193885

__________________________________________________________________________________

8 . AluYa5_19_5

SEQUENCE IN EMBL FORMAT

ID AluYa5_19_5; SV 1; linear; unassigned DNA; STD; UNC; 189 BP.

XX

DE PTS length 189

XX

SQ Sequence 189 BP; 58 A; 38 C; 67 G; 26 T; 0 other;

TTAGCCGGGC ATGGTGGTGG GTGCCTGTAG TCCCAGCTAC TCGGGAGGCT GAGGCAGGAG 60

AATGGCATGA ACCTGGGAGG CAGAGCTTGC AGTGAGCCGA GATCGCGCCA CTGCCCTCCA 120

GCCTGGGCGA CAGAATGAGA CTGTCTCAAA AAAAAAAAAA AGAAGAAGAA GGAGAAGGAG 180

AAGGAGAAG 189

//

REPEAT MASKER ANNOTATION

SW perc perc perc query position in query matching repeat position in repeat

score div. del. ins. sequence begin end (left) repeat class/family begin end (left) ID

1346 5.6 1.2 0.0 AluYa5_19_5 1 160 (29) + AluY SINE/Alu 133 294 (17) 1

240 3.5 0.0 0.0 AluYa5_19_5 161 189 (0) + (GGAGAA)n Simple_repeat 3 31 (0) 2

PTS BLAST RESULTS

GENOME FILENAME CHR CONTIG ORIEN E-VAL LEN1 LEN2 QFC QSC SFC SSC

human AluYa5_19_5 722

human AluYa5_19_5 19 NC_000019 Plus e-102 189 189 1 189 4845547 4845735

Chimp AluYa5_19_5 568

Chimp AluYa5_19_5 1 NC_006468 Minus 1e-44 162 184 1 182 173795372 173795189

__________________________________________________________________________________

9 . AluYa5_2_130c

SEQUENCE IN EMBL FORMAT

ID AluYa5_2_130c; SV 1; linear; unassigned DNA; STD; UNC; 1964 BP.

XX

DE PTS length 1964

XX

SQ Sequence 1964 BP; 703 A; 381 C; 395 G; 485 T; 0 other;

TTCTGTGAAG AAAGTCATTG GTAGCTTGAT GGGGATGGCA TTGAATCTGT AAATTACCTT 60

GGGCAGTATG GCCATTTTCA CGATATTGAT TCTTCCTACC CATGAGCATG GAATGTTCTT 120

CCATTTGTTT GTGTCCTCTT TTATTTCCTT GAGCAGTGGT TTGTAGTTCT CCTTGAAGAG 180

GTCCTTCACA TCCCTTGTAA GTTGGATTCC TAGGTATTTT ATTCTCTTTG AAGCAATTGT 240

GAATGGGAGT TCACCCATGA TTTGGCTCTC TGTTTGTCTG TTGTTGGTGT ATAAGAATGC 300

TTGTGATTTT TGTACATTGA TTTTGTATCC TGAGACTTTG CTGAAGTTGC TTATCAGCTT 360

AAGGAGATTT TGGGCTGAGA CGATGGGGTT TTCTAGATAA ACAATCATTT CTTCACAGAA 420

TTGGAAAAAA CTACTTTAAA GTTCATATGG AACCAAAAAA GAGCCCGCAT CGCCAAGTCA 480

ATCCTAAGCC AAAAGAACAA agctggaggc atcacactac ctgacttcaa actatactac 540

aaggctacag taaccaaaac agcaaggtac tggtaccaaa acagagatat agatcaatgg 600

aacagaacag agccctcaga aataatgccg catatctaca actatctgat ctttgacaaa 660

cctgagaaaa acaagcaatg gggaaaggat tccctattta ataaatggtg ctgggaaaac 720

tggctagcca tatgtagaaa gctgaaactg gatcccttcc ttacacctta tacaaaaatc 780

aattcaagat ggattaaaga tttaaacgtt agacctaaaa ccataaaaac cctagaagaa 840

aacctaggca ttaccattca ggacataggc gtgggcaagg acttcatgtc caaaacacca 900

aaagcaatgg caataaaagc caaaattgac aaatgggatc taattaaact gaagagcttc 960

tgcacagcaa aagaaactac catcagagtg aacaggcaac ctacaacatg ggagaaaatt 1020

ttcacaacct actcatctga caaagggcta atatccagaa tctacaatga actcaaacaa 1080

atttacaaga aaaaaacaaa caaccccatc aaaaagtggg cgaaggacat gaacagacac 1140

ttctcaaaag aagacattta tgcagccaaa aaacacatga agaaatgctc atcatcactg 1200

gccatcagag aaatgcaaat caaaaccact acgagatatc atctcacacc agttagaatg 1260

gcaatcatta aaaagtcagg aaacaacagg tgctggagag gatgtggaga aatagggaca 1320

cttttacact gttggtggga ctgtaaacta gttcaaccat tgtggaagtc agtgtggcga 1380

ttcctcaggg atctagaact agaaatacca tttgacccag ccatcccatt actgggtata 1440

tacccaaagg gctataaatc atgctgctat aaagacacat gcacacgtat gtttattgcg 1500

gcactattca caatagcaaa gacttggaac caacccaaat gtccaacaat gatagactgg 1560

attaagaaaa tgtggcacat atacaccatg gaatactatg ctgccataaa aaatgatgag 1620

ttcatatcct ttgtagggac atggatgaaa ttggaaacca tcattctcag taaactatcg 1680

caagaacaaa aaaccaaaca ccgcatattc tcactcatag gtgggaattg aacaatgaga 1740

tcacatggac acaggaaggg gaatatcaca ctctggggac tgtggtgggg ttgggggagg 1800

ggggagggat agcattggga gagataccta atgctagatg acacattagt gggtgcagcg 1860

caccagcatg gcacatgtat acatatgtaa ctaaccTGCA CAATGTGCAC ATGTACCCTA 1920

AAACTTAGAG TATAATAAAA AAAAAATAAA TAAAAAAAAA AATA 1964

//

REPEAT MASKER ANNOTATION

SW perc perc perc query position in query matching repeat position in repeat

score div. del. ins. sequence begin end (left) repeat class/family begin end (left) ID

3705 0.5 0.0 0.0 AluYa5_2_130c 1 414 (1550) C L1HS LINE/L1 (1520) 4626 4213 1 *

8099 0.9 0.1 0.0 AluYa5_2_130c 402 1949 (15) + L1HS LINE/L1 4607 6155 (0) 1

PTS BLAST RESULTS

GENOME FILENAME CHR CONTIG ORIEN E-VAL LEN1 LEN2 QFC QSC SFC SSC

human AluYa5_2_130c 8726

human AluYa5_2_130c 2 NC_000002 Minus 0.0 1964 1964 1 1964 81690247 81688284

Chimp AluYa5_2_130c 9472

__________________________________________________________________________________

10 . AluYa5_2_177c

SEQUENCE IN EMBL FORMAT

ID AluYa5_2_177c; SV 1; linear; unassigned DNA; STD; UNC; 165 BP.

XX

DE PTS length 165

XX

SQ Sequence 165 BP; 44 A; 43 C; 56 G; 22 T; 0 other;

TTAGCCGGGC GCGGTGGCGG GCGCCTGTAG TCCCAGCTAC TCGGGAGGCT GAGGCAGGAG 60

AATGGCGTGA ACCCGGGAAG CGGAGCTTGC AGTGAGCCGA GATTGCGCCA CTGCACTCCA 120

GCCTGGGCGA CAGAGCGAGA CTCCATCTCA AAAAAAAAAA AAAAA 165

//

REPEAT MASKER ANNOTATION

SW perc perc perc query position in query matching repeat position in repeat

score div. del. ins. sequence begin end (left) repeat class/family begin end (left) ID

1501 2.4 0.0 0.0 AluYa5_2_177c 1 165 (0) + AluY SINE/Alu 133 297 (14) 1

PTS BLAST RESULTS

GENOME FILENAME CHR CONTIG ORIEN E-VAL LEN1 LEN2 QFC QSC SFC SSC

human AluYa5_2_177c 4422

human AluYa5_2_177c 5 NC_000005 Plus 1e-87 165 165 1 165 26121457 26121621

Chimp AluYa5_2_177c 3073

__________________________________________________________________________________

11 . AluYa5_2_67c

SEQUENCE IN EMBL FORMAT

ID AluYa5_2_67c; SV 1; linear; unassigned DNA; STD; UNC; 199 BP.

XX

DE PTS length 199

XX

SQ Sequence 199 BP; 73 A; 44 C; 56 G; 26 T; 0 other;

AAAAGTACAA AAAATTATCC GGGCGTGGTG GCGGGCGCCT GTAGTCCCAG CTACTTGGGA 60

GGCTGAGGCA GGAGAATGGC GTGAACCCGC GAGGCGGAGC TTGCAGTGAG CCGAGATCCC 120

GCCACTGCAT TCCAGCCTGG GCGACAGAGC GAGACTCCGT CTCAAAAAAA AAAAAAAAAA 180

AAAAAAAAAA AAAAAAAAA 199

//

REPEAT MASKER ANNOTATION

SW perc perc perc query position in query matching repeat position in repeat

score div. del. ins. sequence begin end (left) repeat class/family begin end (left) ID

1710 2.6 0.0 0.0 AluYa5_2_67c 1 192 (7) + AluYa5 SINE/Alu 119 310 (0) 1

PTS BLAST RESULTS

GENOME FILENAME CHR CONTIG ORIEN E-VAL LEN1 LEN2 QFC QSC SFC SSC

human AluYa5_2_67c 1852

human AluYa5_2_67c 2 NC_000002 Minus e-108 199 199 1 199 43512488 43512290

Chimp AluYa5_2_67c 1094

__________________________________________________________________________________

12 . AluYa5_4_132c

SEQUENCE IN EMBL FORMAT

ID AluYa5_4_132c; SV 1; linear; unassigned DNA; STD; UNC; 310 BP.

XX

DE PTS length 310

XX

SQ Sequence 310 BP; 92 A; 79 C; 96 G; 43 T; 0 other;

AGCCGGGCGC GGTGGCTCAC GCCTGTAATC CCAGCACTTT GGGAGGCCGA GGCGGGCGGA 60

TCACGAGGTC AGGAGATCGA GACCATCCTG GCTAACACGG TGAAACCCCG TCTCTACTAA 120

AAATACAAAA AAATTAGCCG GGCGTGGTGG CGGGCGCCTG TAGTCCCAGC TACTCGGGAG 180

GCTGAGGCAG GAGAATGGCG TGAACCCGGG AGGCGGAGCT TGCAGTGAGC CGAGATTGCG 240

CCACTGCACT CCAGCCTGGG CAACAGAGCG AGACTCCGTC TCAAAAAAAA AAAAAAAAAA 300

AAAAAAAAAA 310

//

REPEAT MASKER ANNOTATION

SW perc perc perc query position in query matching repeat position in repeat

score div. del. ins. sequence begin end (left) repeat class/family begin end (left) ID

2890 0.7 0.0 0.3 AluYa5_4_132c 1 310 (0) + AluY SINE/Alu 1 309 (2) 1

PTS BLAST RESULTS

GENOME FILENAME CHR CONTIG ORIEN E-VAL LEN1 LEN2 QFC QSC SFC SSC

human AluYa5_4_132c 8675

human AluYa5_4_132c 4 NC_000004 Minus e-174 310 310 1 310 107639249 107638940

Chimp AluYa5_4_132c 3884

__________________________________________________________________________________

13 . AluYa5_4_168c

SEQUENCE IN EMBL FORMAT

ID AluYa5_4_168c; SV 1; linear; unassigned DNA; STD; UNC; 175 BP.

XX

DE PTS length 175

XX

SQ Sequence 175 BP; 55 A; 42 C; 55 G; 23 T; 0 other;

AATTAGCCGG ACATGGTGGC GGGCACCTGT AGTCCCAGCT ACTTGGGAGG CTGAGGCAGG 60

AGAATGGCGT GAACCCGGGA GGCGGAGCTT GCAGTGAGCC GAGATCGCGC CACTGCACTC 120

CAGCCTGGGC GACAGAGCGA GACTCCGTCT CAAAAAAAAA AAAAAAAAAA AAAAA 175

//

REPEAT MASKER ANNOTATION

SW perc perc perc query position in query matching repeat position in repeat

score div. del. ins. sequence begin end (left) repeat class/family begin end (left) ID

1601 2.3 0.0 0.0 AluYa5_4_168c 1 175 (0) + AluY SINE/Alu 131 305 (6) 1

PTS BLAST RESULTS

GENOME FILENAME CHR CONTIG ORIEN E-VAL LEN1 LEN2 QFC QSC SFC SSC

human AluYa5_4_168c 4565

human AluYa5_4_168c 4 NC_000004 Minus 1e-93 175 175 1 175 122667675 122667501

Chimp AluYa5_4_168c 3054

__________________________________________________________________________________

14 . AluYa5_4_196c

SEQUENCE IN EMBL FORMAT

ID AluYa5_4_196c; SV 1; linear; unassigned DNA; STD; UNC; 2175 BP.

XX

DE PTS length 2175

XX

SQ Sequence 2175 BP; 547 A; 536 C; 516 G; 576 T; 0 other;

GTAGTAGACT GATTTCATCT TGATAGTCCG GGTCAGTCAC CCCAGCCAAC ACTGTAACTC 60

CCTTCTTAGA CTGTTGACTT AAAGGTAGGA GGAGCCCCAA GTGTCCAGGT GACAATCTTA 120

ACTTCCTGTT TAATGGAATT GCTGTTGTGT CTCCTGGTGG CAGTGTTCCT CCCTTTGAAA 180

CTAAGACCTC TAAGCCAGCA GAACGTAATG TCATGGGAAC AGGAAGCAAA ACTTTTGCTA 240

GTGACTCTCT AAGGGTGATG ATGAGTGGTG CCACTTCCAC CCCTTGATTC CTGGACCTGT 300

GAATCCTGGC TATGAGAGAA ATAGTATCAT ATATTGGACG CTGATTCAGG GTATACATGG 360

CCTTCTGGAG AACTTTGCCC CAGGCCTGCA AAGTGTTGTC ACCTGGTTGA TGTTGTAATT 420

GTGACTTCAA AAGGCCATTC CGTTCTATCA TTCCAGCTGC TTCAGGATGA CGGGGGAACA 480

TGGTAAGACC AGTGAATTCC atgagcatga gcccactgtc acacttcttt agccataaag 540

tgagtgcctt ggtcagaggc aatgctgtgt ggaataccat gacagtggat aagacattct 600

gtgagtccac agatagtagt cttggcagaa gcattgcatg caggataggc aaaccgatat 660

ctggaataag tgtttattcc agtgaggaca aacctctacc ctttacatga taaaagaggt 720

ccaatatcat caacatgcca tcgggtagct ggctggttac cccaaggaat ggtgccattt 780

cgagggctca gtgttggtct ctgctgctgg caaattgggc actctgcagt ggccgtagcc 840

aggtcagcct tggtgggtgg aagtccatgt tcccgagccc atatgtaacc tccatccctg 900

ccaccatggc cactttgttc gtgggcccat tgggcaatga caggcgtaac tggggaaaga 960

ggctgagtat gcacagaagg gctcatccta ttcacttgat tattaaaatc cttctctgct 1020

gagatcaccc attggtgagc actcacatgg gatacaaata tcttcacagt ttttgaccac 1080

tcagagagat acatccacat atctctttcc cagatttctt tgtcaccaat tttccaatta 1140

tgcttcttcc atgtccctga ccatccagcc aaaccattag ctacagccca tgaataagta 1200

tataatcgca catctggcca tttctccttc catacaaagt gcacaactag gtgtactgct 1260

taaagttctg cccactggga agatttcctt tcactgctgt ccttcaggga tgtcctagaa 1320

aggggctgta gtgctacagc tgtccacttt tgggtagtgc ctacatatcg tgcagaatca 1380

tccgtgaacc gggccctagt cttttcttcc tctgtcagct gatcataggg aactccccat 1440

gaggccatcg gtgcaggctc gggagataag tcagggtggc aggagtggag accgtgggca 1500

tttgagccac ttcctcatgt aacttatttg tgccttcagg acctgctcga gcctgatcac 1560

gtatatacca cttccatttg atgatggaat gctgctgtgc atgacccact ttacggctag 1620

atgggtcaga aggcacccag ttaatgatag tcagttcagg ttgaatggtg acttgatgac 1680

acatagtcaa acgttccgtt tccgccaaag cccagtaaca ggccaaaagc tgtctctcag 1740

aaggagaata gttatctgca gaagatgtca gggccttgct ccaaaatcct agaggcctct 1800

actgtgattc acctatgggg gactgccaaa ggctccaaac agcatccctt gaggtgtcac 1860

tgacacctca agcaccattg gatctgctgg gtcatatggc ccaagtggca gagcagcttg 1920

cacagcagtc tggacatgtt gtagagcctt ctcctggacc ccagtcaaaa ctggcagcct 1980

ttcgggtcac tcagtaaatg ggccagagta acacaccgaa atgaggaatg tgttgcctcc 2040

aaaatccaaa tgggcccact aggcgttgtt cctctttctt ggtataggag gggccaaatg 2100

cagcaactta tccttcactt tagaaggaac atctcgacag gacccacact actggacccc 2160

tagaaatttt actga 2175

//

REPEAT MASKER ANNOTATION

SW perc perc perc query position in query matching repeat position in repeat

score div. del. ins. sequence begin end (left) repeat class/family begin end (left) ID

16755 6.9 0.9 0.1 AluYa5_4_196c 1 2175 (0) C HERVL-int LTR/ERVL (611) 5043 2853 1

PTS BLAST RESULTS

GENOME FILENAME CHR CONTIG ORIEN E-VAL LEN1 LEN2 QFC QSC SFC SSC

human AluYa5_4_196c 30

human AluYa5_4_196c 4 NC_000004 Minus 0.0 2175 2175 1 2175 140588262 140586088

Chimp AluYa5_4_196c 20

Chimp AluYa5_4_196c 18 NC_006485 Minus 0.0 1931 2188 1 2175 13906489 13904310

Chimp AluYa5_4_196c 2B NC_006470 Plus 0.0 1890 2191 1 2168 158670967 158673151

Chimp AluYa5_4_196c 2B NC_006470 Minus 0.0 1892 2196 1 2175 141956258 141954073

Chimp AluYa5_4_196c 10 NC_006477 Plus 0.0 1920 2191 1 2173 84070101 84072283

Chimp AluYa5_4_196c 10 NC_006477 Minus 0.0 1877 2192 3 2175 85165654 85163470

__________________________________________________________________________________

15 . AluYa5_5_140

SEQUENCE IN EMBL FORMAT

ID AluYa5_5_140; SV 1; linear; unassigned DNA; STD; UNC; 338 BP.

XX

DE PTS length 338

XX

SQ Sequence 338 BP; 119 A; 56 C; 89 G; 74 T; 0 other;

GGATGAGTTC ATGTCCTTTG TAGGGACATG GATGAAATTG GAAATCATCA TTCTCAGTAA 60

ACTATCGCAA GAACAAAAAA CCAAACACCG CATATTCTCA CTCATAGGTG GGAATTGAAC 120

AATGAGATCA CATGGACACA GGAAGGGGAA TATCACACTC TGGGGACGGT TGTGGGGTGG 180

GGGGAGGGGG GAGGGATAGC ATTGGGAGAT ATACCTAATG CTAGATGACG AGTTAGTGGG 240

TGCAGTGCGC CAGCATGGCA CATGTATACA TATGTAACTA ACCTGCACAA TGTGCACATG 300

TACCCTAAAA CTTAAAGTAT AATAAAAAAA AAAAAAGA 338

//

REPEAT MASKER ANNOTATION

SW perc perc perc query position in query matching repeat position in repeat

score div. del. ins. sequence begin end (left) repeat class/family begin end (left) ID

3009 1.2 0.0 0.0 AluYa5_5_140 2 336 (2) + L1PA2 LINE/L1 5821 6155 (0) 1

PTS BLAST RESULTS

GENOME FILENAME CHR CONTIG ORIEN E-VAL LEN1 LEN2 QFC QSC SFC SSC

human AluYa5_5_140 1424

human AluYa5_5_140 5 NC_000005 Plus 0.0 338 338 1 338 104680324 104680661

Chimp AluYa5_5_140 2287

__________________________________________________________________________________

16 . AluYa5_5_181c

SEQUENCE IN EMBL FORMAT

ID AluYa5_5_181c; SV 1; linear; unassigned DNA; STD; UNC; 259 BP.

XX

DE PTS length 259

XX

SQ Sequence 259 BP; 85 A; 52 C; 59 G; 63 T; 0 other;

AGTCAGGAAA CAACAGGTGC TGGAGAGGAT GTGGAGAAAT AGGAACACTT TTACACTGTT 60

GGTGGGACTG TAAACTAGTT CAACCATTGT GGAAGTCAGT GTGGCGATTC CTCAGGGATC 120

TAGAACTAGA AATACCATTT GACCCAGCCA TCCCATTACT GGGTATATAC CCAAAGGACT 180

ATAAATCATG CTGCTATAAA GACACATGCA CACGTATGTT TATTGCGGCA CTATTCACAA 240

TAGCAAAGAC TTGGAACCA 259

//

REPEAT MASKER ANNOTATION

SW perc perc perc query position in query matching repeat position in repeat

score div. del. ins. sequence begin end (left) repeat class/family begin end (left) ID

2427 0.0 0.0 0.0 AluYa5_5_181c 1 259 (0) + L1P1 LINE/L1 5480 5738 (417) 1

PTS BLAST RESULTS

GENOME FILENAME CHR CONTIG ORIEN E-VAL LEN1 LEN2 QFC QSC SFC SSC

human AluYa5_5_181c 2463

human AluYa5_5_181c Y NC_000024 Plus e-143 259 259 1 259 18487108 18487366

Chimp AluYa5_5_181c 1491

__________________________________________________________________________________

17 . AluYa5_5_213

SEQUENCE IN EMBL FORMAT

ID AluYa5_5_213; SV 1; linear; unassigned DNA; STD; UNC; 23 BP.

XX

DE PTS length 23

XX

SQ Sequence 23 BP; 15 A; 1 C; 4 G; 3 T; 0 other;

GAAAGAAAAG AACAAATAAA TGT 23

//

REPEAT MASKER ANNOTATION

There were no repetitive sequences detected in /home/vipin/WHOLE_GENOME_CG/AluYa5_CHR/Chimp/CONFIRMATION/AluYa5_3PTS/AluYa5_5_213

PTS BLAST RESULTS

GENOME FILENAME CHR CONTIG ORIEN E-VAL LEN1 LEN2 QFC QSC SFC SSC

human AluYa5_5_213 71

human AluYa5_5_213 5 NC_000005 Plus 2e-04 23 23 1 23 163356562 163356584

Chimp AluYa5_5_213 55

__________________________________________________________________________________

18 . AluYa5_5_60

SEQUENCE IN EMBL FORMAT

ID AluYa5_5_60; SV 1; linear; unassigned DNA; STD; UNC; 2669 BP.

XX

DE PTS length 2669

XX

SQ Sequence 2669 BP; 920 A; 541 C; 414 G; 794 T; 0 other;

GAGCTCCAAA TATCCACTTT CAGGTACTAC AAGAGGAGAG TTTCAAAACT GCTCAATCAA 60

AACAAAGGTT CATTTCTGTT AGTTGAACAC ACATCACAAA GAATTTTCTC CAAATGCTTC 120

TGTGTGGTTT TTATGTGAAG ATAATTCCTT TTCCACCATA GTCCACAAAG CATTCCAAAT 180

ATCCAGTTGC AGATTCTACA AAAGAATGTT TCCAAACTCC TCAATGAAAA TAAAGGTTCA 240

AGTCCATGAG ATGAATGCAC ACATCACAAA GAAGATTCTC AGAATGCTTC TGTCTAGTTT 300

TTATGTGAAG ATATTTCATT TTCCACCATA GGCCTCAAAG CACTCAAATA TCCATTTGAA 360

GAATCTAGAA GAAGTACGTT TCATAACTGC TCCATGAGAA CAAAGGCTCA ACTCTGGGAG 420

ATGAATGCAC ACATCAGAAA GAAATTTGCC AGAATGCTGC TGTCTAGTTT TTGTATGAAG 480

TTATTTCCTT TTCCACAATA ggcctgaaag cgctccaaat atccacatga agaatatata 540

aatagagtgt atcaaaaata ctcaattgaa agaaatgttc aactctgtga gatgaatgca 600

cacatcacaa agatgtttct cagaatgctt cttgtgtagt ttttatgtga agacatttcc 660

tttcccacct taggctgaaa aggtctccat aaatacactt gaagattcta caaaaagaga 720

gtttcaaaac tgcttaatca aaagaaaatt tcaactctgt gagatgaatg cacacatcac 780

gaagaagttt ctctgaatgc ttctgtctag tttttatgtg gagatatatc cttttccacc 840

acaggcctca tagtgctcca aatatccact tgcagattct acaaaaagaa cgtatcagta 900

ctgctcaatg aaaacaaaag ttcaactctg tgggttgaat gcacacatca gaaaaaagtt 960

tgtcataatg ctgctgtcta gtttttatgt gaagttattt ccttttccaa aagaggcctc 1020

aaagccctcc aaatatccac ttgcagattc tacaaaaaga gtgtttcaaa actgctaaat 1080

gtaaagaaaa gttcaacatt gtgagatgaa tgcacccatc acaaataagt ttctcagaat 1140

gcttctgtct agtgtttatg tatagatatt tgcttttcca caatagacca caaaggcctc 1200

caaatatcca cttgcagatt ctacaaaaag agaatttcga aactgctcag tcaaaagaaa 1260

tgttcaactc tgtgagttgg atgcatacat cacaaagaat tttctcagaa tgcttctgtg 1320

tagtttttat gtgaagatat ttccttttcc accaaaggcc tcaaagcgct ccaaatacca 1380

atttgcagaa actacaaaaa gattgtttca aaactgatca acgaaaacaa agtttcaact 1440

ctggaagatg aatgcacaga tcagaaagaa gtttgtcaga atgcttctgt ctagttttta 1500

tgtgaagata ttacccttcc caacataggc caaaaacggc tccaaatatc cacttacaga 1560

ttccataaaa agaaagtttc aaaactgctc aatcaaaata tatgttcaac tctgtgaatg 1620

gaccgcacac atcacaaagc agtttctcag aatgcttctt tctagctttt atgtgaagat 1680

attttctttt gcaccatagg tcccaaaacg caccaaatat ccacttgcag atccttcaaa 1740

aagagtgttt caaaactgtt caaggaaaag aaagattcaa ctctgtgaga tgaatgcaac 1800

catcacaaaa ttttttctaa gaatgcttct gtctagtttt tatgtaaaca tatttccttt 1860

tccacaatag gccgcaaagg tctccaaata tccatttgca gattctacaa aaagagagtt 1920

tcaaaactgc tcaatcaaag gaaatgttca actctgtgag ttgaaagcac acatcacaga 1980

gaagtttctc agaatgcttc catctagttt ttatatgaag atatttcctt ttccaccata 2040

ggcctcaaag tgctcaaatt atctacttgt agattctata aaaagaatgt ttcaaaactg 2100

ctcaatcaaa agaaaagttc agctctgtga gttgaacgca cacatcacaa agaagtttct 2160

cagaatgctt atgtgtagtc tttatgtgaa tatatatctt ttccaccata ggcctcaaag 2220

tgcttcaaat atccacttgc agattcaaga aaaagagtgt tttgaaactg ttgaatgaaa 2280

aaaaaaggtt caactctgtg agatgaatgc acacatcaca aagtagtttc tcagcatgct 2340

tctgtgtact ttttatgtga agttatttcc tttcccacca tagcccttaa agggctccaa 2400

acatccactt gcagattctt caaaaagaga gtttcaaaac tgctcattca aaatataggt 2460

tcaacactgt gattggaatg cccacatcac aaagtagttt ctcagaatgc ttctgcctag 2520

ttttgatgtg aagatatttc cttatccaac atagccttcc aaacactcca aatatcccct 2580

agcagattct aaaaaaagca gtgtttcaaa actcctctat gtaaaaaatt gttcatctct 2640

gagacatgaa tgcacacatc acaattaag 2669

//

REPEAT MASKER ANNOTATION

SW perc perc perc query position in query matching repeat position in repeat

score div. del. ins. sequence begin end (left) repeat class/family begin end (left) ID

1130 16.4 0.1 0.1 AluYa5_5_60 1 2664 (5) C ALR/Alpha Satellite/centr (53) 2664 1 1

PTS BLAST RESULTS

GENOME FILENAME CHR CONTIG ORIEN E-VAL LEN1 LEN2 QFC QSC SFC SSC

human AluYa5_5_60 52

human AluYa5_5_60 5 NC_000005 Plus 0.0 2669 2669 1 2669 49522369 49525037

human AluYa5_5_60 5 NC_000005 Plus 0.0 1500 1809 3 1807 49559749 49561552

human AluYa5_5_60 5 NC_000005 Plus 0.0 1332 1604 3 1602 49560091 49561689

human AluYa5_5_60 5 NC_000005 Plus 0.0 1401 1702 3 1700 49559237 49560935

human AluYa5_5_60 5 NC_000005 Plus 0.0 1440 1762 3 1759 49508573 49510333

human AluYa5_5_60 5 NC_000005 Plus 0.0 1498 1842 721 2554 49528521 49530351

human AluYa5_5_60 5 NC_000005 Plus e-176 1102 1354 1262 2613 49512571 49513917

human AluYa5_5_60 16 NC_000016 Plus 0.0 1665 1986 569 2553 33902483 33904466

human AluYa5_5_60 16 NC_000016 Minus 0.0 2048 2512 40 2543 35081114 35078611

human AluYa5_5_60 16 NC_000016 Minus 0.0 1752 2136 412 2542 35087230 35085099

human AluYa5_5_60 16 NC_000016 Plus 0.0 1476 1783 811 2591 33911232 33913013

human AluYa5_5_60 16 NC_000016 Minus 0.0 870 1044 1567 2609 33821706 33820667

human AluYa5_5_60 X NC_000023 Minus 0.0 1891 2288 251 2536 61702199 61699916

human AluYa5_5_60 X NC_000023 Minus 0.0 1732 2096 127 2218 61808085 61805998

human AluYa5_5_60 X NC_000023 Minus 0.0 1726 2137 422 2554 61716143 61714023

human AluYa5_5_60 X NC_000023 Minus 0.0 1372 1686 934 2617 61713934 61712257

human AluYa5_5_60 X NC_000023 Minus e-174 1260 1567 3 1563 61736915 61735351

Chimp AluYa5_5_60 52

Chimp AluYa5_5_60 3 NC_006490 Plus 0.0 1989 2370 176 2541 97519185 97521545

Chimp AluYa5_5_60 3 NC_006490 Plus 0.0 2055 2510 36 2538 97523136 97525634

Chimp AluYa5_5_60 3 NC_006490 Plus 0.0 1779 2151 408 2554 92773938 92776083

Chimp AluYa5_5_60 3 NC_006490 Plus 0.0 1500 1802 865 2664 97490265 97492061

Chimp AluYa5_5_60 3 NC_006490 Plus 0.0 1573 1938 730 2664 92764719 92766645

Chimp AluYa5_5_60 3 NC_006490 Plus 0.0 1186 1446 4 1443 97504478 97505919

Chimp AluYa5_5_60 3 NC_006490 Plus e-130 1202 1514 3 1512 92838747 92840256

Chimp AluYa5_5_60 11 NC_006478 Plus 0.0 1762 2097 569 2664 50778285 50780370

Chimp AluYa5_5_60 11 NC_006478 Plus 0.0 1829 2184 482 2664 50777527 50779687

Chimp AluYa5_5_60 11 NC_006478 Plus 0.0 1717 2097 4 2095 50773394 50775479

Chimp AluYa5_5_60 11 NC_006478 Minus 0.0 1218 1484 313 1794 50760894 50759416

Chimp AluYa5_5_60 8 NC_006475 Minus 0.0 1830 2207 408 2613 44042404 44040205

Chimp AluYa5_5_60 8 NC_006475 Minus 0.0 1381 1682 5 1682 44042298 44040621

Chimp AluYa5_5_60 8 NC_006475 Minus 0.0 1090 1318 247 1563 44038300 44036984

__________________________________________________________________________________

19 . AluYa5_7_65c

SEQUENCE IN EMBL FORMAT

ID AluYa5_7_65c; SV 1; linear; unassigned DNA; STD; UNC; 315 BP.

XX

DE PTS length 315

XX

SQ Sequence 315 BP; 84 A; 84 C; 102 G; 45 T; 0 other;

TAGCAAACAC CGGCCGGGCG CGGTGGCTCA CGCCTGTAAT CCCAGCACTT TGGGAGGCCG 60

AGGCGGGTGG ATCACGAGGT CAGGAGATCG AGACCATCCT GGCTAACAAG GTGAAACCCC 120

GTCTCTACTA AAAATACAAA AAATTAGCCG GGCGCGGTGG CGGGCGCCTG TAGTCCCAGC 180

TACTCGGGAG GCTGAGGCAG GAGAATGGCG TGAACCCGGG AAGCGGAGCT TGCAGTGAGC 240

CGAGATTGCG CCACTGCAGT CCGCAGTCCG GCCTGGGCGA CAGAGCGAGA CTCCGTCTCA 300

AAAAAAAAAA AAAAA 315

//

REPEAT MASKER ANNOTATION

SW perc perc perc query position in query matching repeat position in repeat

score div. del. ins. sequence begin end (left) repeat class/family begin end (left) ID

2872 0.7 0.0 0.0 AluYa5_7_65c 12 315 (0) + AluYb8 SINE/Alu 1 304 (14) 1

PTS BLAST RESULTS

GENOME FILENAME CHR CONTIG ORIEN E-VAL LEN1 LEN2 QFC QSC SFC SSC

human AluYa5_7_65c 4969

human AluYa5_7_65c 7 NC_000007 Minus e-177 315 315 1 315 46333006 46332692

Chimp AluYa5_7_65c 3216

__________________________________________________________________________________

20 . AluYa5_9_149

SEQUENCE IN EMBL FORMAT

ID AluYa5_9_149; SV 1; linear; unassigned DNA; STD; UNC; 875 BP.

XX

DE PTS length 875

XX

SQ Sequence 875 BP; 224 A; 127 C; 117 G; 407 T; 0 other;

CCTTATAAAT TTATTTCAGC TTATTTAATA GTCCAGATTA TGGTTGATCT TGGTGACTGT 60

TGTATGTGCT CCATAGTATG TTGAAGTTTG GATCTCCCCC ATTACATATA AATTTGTATA 120

TTTGATGGAT TTGCTAAAGT CTTAGAGGCT CTGTTAATTT TTCTTAATTC TTTTTTTCTT 180

TCTGTTCTCA AACTGGATAA TTTCCTATCA CCAAACTATC ATTTAATAAT AAGATAGCAC 240

ATCACAACTG AAGGAAGACT TTTACAACTT AACTTTTATT TCTCTTTGTG CAATTTGTGT 300

CATACATTTT ACTCATGTAT ATTTTATGAC ACCCATAATA GTTTTTTTTG TTTTACATGG 360

TCAATTATGT TTTAAAACAT TTTTATTAGA AAAAAGTCTT CTATATTTAC CCATATATTT 420

TCTCTATTTC AGGTGATTCT CATCTCTTTG TGCACATTCA AATTTTTGTC TAGAATCATT 480

TTTTTGTCTA AAGTTGTTTT taagcattta gctcaaattt gcttcaaatt aattatgtta 540

gcttttgttc atatgaaaaa agtttttact tagcctttat ttttaaagaa tctttctatt 600

tcttatggaa ttctagattg acaattattt ttctttttag ggctttaaag atgttccatt 660

gtgttttgct ttccacggtt tctaatgaaa aatatgtgtt aattatcttt attccttttt 720

acataaggta tgcttttttt ctagttgctt ttaaggtttg tttccctctt tattttgttt 780

ttacaattca attatgacgt gcctgcgggt gaatctgtat atgtatgtat ttcttttttc 840

ctgattctca ttgcacattt ggatgagtag gtgca 875

//

REPEAT MASKER ANNOTATION

SW perc perc perc query position in query matching repeat position in repeat

score div. del. ins. sequence begin end (left) repeat class/family begin end (left) ID

226 26.9 0.0 0.0 AluYa5_9_149 2 68 (807) C L1MEf LINE/L1 (3381) 2938 2872 1

369 21.5 1.0 2.9 AluYa5_9_149 87 205 (670) C L1M4 LINE/L1 (5109) 1537 1421 2

960 26.6 8.2 2.0 AluYa5_9_149 206 811 (64) C L1MEf LINE/L1 (4300) 2100 1463 1

PTS BLAST RESULTS

GENOME FILENAME CHR CONTIG ORIEN E-VAL LEN1 LEN2 QFC QSC SFC SSC

human AluYa5_9_149 1

human AluYa5_9_149 9 NC_000009 Plus 0.0 875 875 1 875 108064258 108065132

Chimp AluYa5_9_149 0

__________________________________________________________________________________

21 . AluYa5_9_26

SEQUENCE IN EMBL FORMAT

ID AluYa5_9_26; SV 1; linear; unassigned DNA; STD; UNC; 1226 BP.

XX

DE PTS length 1226

XX

SQ Sequence 1226 BP; 493 A; 264 C; 220 G; 249 T; 0 other;

TACCTAAGAA TCCAACTTAC CAGGGATGTG AAGGACCTCT TCAAGGAGAA CTACAAACCA 60

CTGCTCAAGG AAATAAAAGA GGATACAAAC AAATGGAAGA ACATTCCATG CTCATGGGTA 120

GGAAGAATCA ATATCATGAA AATGGCCATA CTGCCAAAGG TAATTTACAG ATTCAATGCC 180

ATCCCCATCA AGCTACCAAT GACTTTCTTC ACAGAATTGG AAAAAACTAC TTTAAACTTC 240

ATATGGAACC AAACAAGAGC CCGCATCGCC AAGTCAATCC TAAGGCAAAA GAACAAAGCT 300

GGAGGCATCA CACTACTTGA CTTCAAACTA TACTACAAGG CTACAGTAAC CAAAACAGCA 360

TGGTACTGGT ACCAAAACAG CGATATTGAT CAATGGAACA GAACAGAGCC CTCAGAAATA 420

ACACCGCATA TCTACAACTA TCTGATCTTT GACAAACCTG ACAAAAACAA GCAATGGGGA 480

AAGGATTCCC TATTTAATAA atggtgctgg gaaaactggc tagccatatg tagaaagctg 540

aaactggatc ccttccttac accttataca aaaatcaatt caagatggat taaagactta 600

aatgttagac ctaaaaccat aaaagcccta gaagaaaacc taggcaacac cattcaggac 660

ataggcatgg gcaaggactt catgtctaaa acaccaaaag caatggcaac aaaagccaaa 720

attgacaaat gggatctcat taaactaaag agcttctgca cagcaaaaga aattaccatc 780

agagtgaaca ggcaatctac aaaatgggag aaaatttttg caagctactc atctgaccaa 840

ggggtaatat ccagaatcta caatgaactc aaacaaattt acaagaaaaa aacaaacaac 900

ccgatcaaaa agtgggcgaa ggatatgaac agacacttct caaaagaaga catttatgca 960

gccaaaaaac acatgaaaaa atgctcacca tcactggcca tcagagaaat gcaaatcaaa 1020

accacaatga gataccatct cacaccattt agaatggcga tcattaaaaa gtcaggaaac 1080

aacaggtgct ggagaggatg tggagaaata ggaacacttt tacactgttg gtgggactgt 1140

aaactagttc aacccttgtg gaagtcagtg tggcgattcc tcagggatct agaactagaa 1200

ataccatttg acccagccat cccatt 1226

//

REPEAT MASKER ANNOTATION

SW perc perc perc query position in query matching repeat position in repeat

score div. del. ins. sequence begin end (left) repeat class/family begin end (left) ID

8306 2.7 0.0 0.0 AluYa5_9_26 1 1226 (0) + L1P1 LINE/L1 4411 5636 (519) 1

PTS BLAST RESULTS

GENOME FILENAME CHR CONTIG ORIEN E-VAL LEN1 LEN2 QFC QSC SFC SSC

human AluYa5_9_26 5905

human AluYa5_9_26 9 NC_000009 Plus 0.0 1226 1226 1 1226 18170554 18171779

Chimp AluYa5_9_26 3560

__________________________________________________________________________________

22 . AluYa5_X_111

SEQUENCE IN EMBL FORMAT

ID AluYa5_X_111; SV 1; linear; unassigned DNA; STD; UNC; 378 BP.

XX

DE PTS length 378

XX

SQ Sequence 378 BP; 138 A; 81 C; 77 G; 82 T; 0 other;

AGGACACGAA CAGACACTTC TCAAAAGAAG ACATTTATGC AGCCAAAAAA CACATGAAAA 60

AATGCTCACC ATCACGGGCC ATCAGAGAAA TGCAAATCAA AACCGCAATG AGATACCATC 120

TCACACCAGT TCGAATGGCG ATCATTACAA AGTCAGGAAA CAACAGGTGC TGGAGAGGAT 180

GTGGAGAAAT AGGAACACTT TTACACTGTT GGTGGGAATG TAAACTACTT CAACCATTGT 240

GGAAGTCAGT GTGGCAATTC CTCAGGGATC TAGAACTAGA AATACCATTT GACCCAGCCA 300

TCCCATTACT GGGTATATAC CCAAAGGACT ATAAATCATG CTGCTATAAA GACACATGCA 360

CACGTATGTT TATTGTGG 378

//

REPEAT MASKER ANNOTATION

SW perc perc perc query position in query matching repeat position in repeat

score div. del. ins. sequence begin end (left) repeat class/family begin end (left) ID

3296 2.6 0.0 0.0 AluYa5_X_111 1 378 (0) + L1P1 LINE/L1 5330 5707 (448) 1

PTS BLAST RESULTS

GENOME FILENAME CHR CONTIG ORIEN E-VAL LEN1 LEN2 QFC QSC SFC SSC

human AluYa5_X_111 2902

human AluYa5_X_111 X NC_000023 Plus 0.0 378 378 1 378 121490948 121491325

Chimp AluYa5_X_111 3256

__________________________________________________________________________________

23 . AluYa5_Y_19c

SEQUENCE IN EMBL FORMAT

ID AluYa5_Y_19c; SV 1; linear; unassigned DNA; STD; UNC; 41 BP.

XX

DE PTS length 41

XX

SQ Sequence 41 BP; 6 A; 9 C; 12 G; 14 T; 0 other;

GTGATGTAAC TCTTGTCTAG GCTCTGCCTA CAGGGGCTTT G 41

//

REPEAT MASKER ANNOTATION

SW perc perc perc query position in query matching repeat position in repeat

score div. del. ins. sequence begin end (left) repeat class/family begin end (left) ID

345 2.4 0.0 0.0 AluYa5_Y_19c 1 41 (0) C BSR/Beta Satellite (74) 62 22 1

PTS BLAST RESULTS

GENOME FILENAME CHR CONTIG ORIEN E-VAL LEN1 LEN2 QFC QSC SFC SSC

human AluYa5_Y_19c 158

human AluYa5_Y_19c Y NC_000024 Minus 2e-14 41 41 1 41 11946880 11946840

Chimp AluYa5_Y_19c 44

__________________________________________________________________________________
