# Supplementary material for: RISCI - Repeat Induced Sequence Changes Identifier: a comprehensive, comparative genomics-based, in silico subtractive hybridization pipeline to identify repeat induced sequence changes in closely related genomes
Source: BMC Bioinformatics. 2010 Dec 26;11:609. doi: 10.1186/1471-2105-11-609 (PMC3024322; doi:10.1186/1471-2105-11-609)
Supplement: Additional file 15 — RISCI validation. Alu loci mentioned in Sen et al and annotated as INDELs by RISCI. RepeatMasker annotation of the repeat locus and in flanks in the reference genome and of the identified ortholog and its flanks in the comparative genomes to identify putative regions of homology where recombination takes place [file 1471-2105-11-609-S15.DOC]

**Additional file 15**

**Alu_Recombination – loci annotated as INDEL_CAN or OCCUPIED by RISCI.**

**RepeatMasker annotation of the locus and 1.5kb flank in human genome (Hg18) and the identified ortholog in Chimpanzee genome (Pantro 2) are mentioned.**

**R1 and R2 – PUTATIVE REGIONS OF HOMOLOGY IN CHIMPANZEE GENOME WHERE RECOMBINATION TAKES PLACE IN THE HUMAN GENOME**

**AluYc2_1_5 11652576-11652872 INDEL_CAN**

**11651046 11651208 C FRAM SINE/Alu 160 1**

**11651616 11651832 + L2 LINE/L2 3015 3247**

**11651833 11652126 + AluSp SINE/Alu 6 299**

**11652127 11652221 + L2 LINE/L2 3248 3359**

**11652232 11652382 + (TA)n Simple_repeat 2 152**

**11652405 11652575 C AluSq SINE/Alu 176 6**

**11652576 11652872 + AluY SINE/Alu 2 297 ( AluYc2_1_5 )**

**11653247 11653282 + AT_rich Low_complexity 1 36**

**11653290 11653400 C FLAM_C SINE/Alu 111 1**

**Ortholog in Chimp 11869324-11871174 Plus Nscore 0**

**N positions**

**11867788 11867950 C FRAM SINE/Alu 160 1**

**11868358 11868574 + L2 LINE/L2 3015 3247**

**11868575 11868868 + AluSp SINE/Alu 6 299**

**11868869 11868961 + L2 LINE/L2 3248 3359**

**11868972 11869134 + (TA)n Simple_repeat 1 163**

**11869156 11869328 C AluSq SINE/Alu 173 1**

**11869331 11869429 C AluSg/x SINE/Alu 307 212**

**11869432 11869504 + (TA)n Simple_repeat 2 75**

**11869508 11869643 C FLAM_C SINE/Alu 143 11**

**11869774 11870080 + AluSx SINE/Alu 1 307**

**11870083 11870364 + AluSx SINE/Alu 1 279**

**11870365 11870402 + (CA)n Simple_repeat 1 38**

**11870440 11870502 C MIRb SINE/MIR 152 86**

**11870877 11871171 + AluY SINE/Alu 1 294**

**11871546 11871580 + AT_rich Low_complexity 1 35**

**11871589 11871699 C FLAM_C SINE/Alu 111 1**

**__________________________________________________________________________________**

**AluSx_1_12c 28141751-28142054 INDEL_CAN**

**28140371 28140472 + MIRb SINE/MIR 119 221**

**28140524 28140905 C L2 LINE/L2 3417 3016**

**28141086 28141152 + (CA)n Simple_repeat 1 67**

**28141458 28141579 + MIR SINE/MIR 16 138**

**28141580 28141748 C AluSg/x SINE/Alu 299 135**

**28141751 28142054 C AluSx SINE/Alu 312 6 ( AluSx_1_12c )**

**28142070 28142202 + L1MEe LINE/L1 830 963**

**28142241 28142374 + MIRb SINE/MIR 50 207**

**28142815 28143134 + AluSx SINE/Alu 1 306**

**28143451 28143751 C AluJo SINE/Alu 302 3**

**Ortholog in Chimp 28200304-28200888 Minus Nscore 0**

**N positions**

**28198923 28199041 + MIRb SINE/MIR 112 225**

**28199076 28199457 C L2 LINE/L2 3417 3016**

**28199638 28199704 + (CA)n Simple_repeat 1 67**

**28200132 28200310 C AluSx SINE/Alu 299 137**

**28200311 28200623 C AluSc SINE/Alu 309 1 (R2)**

**28200624 28200760 C AluSx SINE/Alu 136 1**

**28200762 28200888 C AluJo SINE/Alu 135 6 (R1)**

**28200904 28201036 + L1MEe LINE/L1 830 963**

**28201075 28201209 + MIRb SINE/MIR 50 207**

**28201652 28201980 + AluSx SINE/Alu 1 309**

**__________________________________________________________________________________**

**AluSq_1_37 209490963-209491226 INDEL_CAN**

**209489526 209489589 + AluJo SINE/Alu 12 75**

**209489591 209490234 C LTR8 LTR/ERV1 644 1**

**209490235 209490283 + AluJo SINE/Alu 82 135**

**209490298 209490467 + AluJo SINE/Alu 141 300**

**209490646 209490669 + AT_rich Low_complexity 1 24**

**209490963 209491226 + AluSq SINE/Alu 1 280 ( AluSq_1_37 )**

**209491369 209491680 + AluSx SINE/Alu 2 320**

**209491752 209491779 + (TG)n Simple_repeat 2 29**

**209491811 209492187 + L2 LINE/L2 1891 2305**

**209492188 209492529 + MLT1A0 LTR/MaLR 3 365**

**209492530 209492662 + L2 LINE/L2 2306 2453**

**209492663 209492959 C AluSp SINE/Alu 303 2**

**Ortholog in Chimp 191712122-191713070 Plus Nscore 0**

**N positions**

**191710684 191710747 + AluJo SINE/Alu 12 75**

**191710749 191711392 C LTR8 LTR/ERV1 644 1**

**191711393 191711442 + AluJo SINE/Alu 82 135**

**191711453 191711626 + AluJo SINE/Alu 137 300**

**191711824 191711946 C HAL1b LINE/L1 1530 1412**

**191712122 191712414 + AluSg SINE/Alu 1 294 (R1)**

**191712415 191712438 + (TA)n Simple_repeat 2 25**

**191712782 191713055 + AluSq SINE/Alu 2 291 (R2)**

**191713214 191713532 + AluSx SINE/Alu 2 328**

**191713604 191713629 + (TG)n Simple_repeat 2 27**

**191713661 191714038 + L2 LINE/L2 1891 2305**

**191714039 191714380 + MLT1A0 LTR/MaLR 3 365**

**191714381 191714513 + L2 LINE/L2 2306 2453**

**191714514 191714811 C AluSp SINE/Alu 304 2**

**__________________________________________________________________________________**

**AluSq_2_48c 34208446-34208738 INDEL_CAN**

**34206363 34207074 C L1PA4 LINE/L1 6155 5440**

**34207651 34207766 + AluJo SINE/Alu 5 134**

**34208446 34208738 C AluSq SINE/Alu 309 7 ( AluSq_2_48c )**

**34208739 34208987 C AluSp SINE/Alu 240 1**

**34209172 34209832 + MER65C-int LTR/ERV1 5610 6274**

**Ortholog in Chimp 34913101-34914036 Minus Nscore 0**

**N positions**

**34911018 34911729 C L1PA4 LINE/L1 6155 5440**

**34912305 34912420 + AluJo SINE/Alu 5 134**

**34913101 34913399 C AluSq SINE/Alu 301 1**

**34913969 34914283 C AluSp SINE/Alu 307 3**

**34914468 34915128 + MER65C-int LTR/ERV1 5610 6274**

**__________________________________________________________________________________**

**AluY_2_52c 48044434-48044754 INDEL_CAN**

**48041249 48043584 + L1MA1 LINE/L1 5381 7717**

**48043587 48044102 C L1MCa LINE/L1 1838 1445**

**48044175 48044380 + L1M5 LINE/L1 5175 5384**

**48044434 48044754 C AluY SINE/Alu 321 1 ( AluY_2_52c )**

**48045618 48046242 + MER51A LTR/ERV1 1 634**

**Ortholog in Chimp 49119791-49121728 Minus Nscore 0**

**N positions**

**49116613 49118942 + L1MA1 LINE/L1 5381 7712**

**49118947 49119171 C L1MCa LINE/L1 1838 1611**

**49119164 49119289 + L1M1 LINE/L1 4565 4700**

**49119294 49119461 C L1MCa LINE/L1 1615 1445**

**49119674 49119789 + L1MC LINE/L1 5315 5432**

**49119790 49120093 C AluY SINE/Alu 302 1 (R1)**

**49120094 49120383 + L1M5 LINE/L1 5433 5752**

**49121373 49121406 + (TCCC)n Simple_repeat 3 36**

**49121417 49121728 C AluY SINE/Alu 310 1 (R2)**

**49122591 49123215 + MER51A LTR/ERV1 1 634**

**__________________________________________________________________________________**

**AluSc_2_57c 96595354-96595664 INDEL_CAN**

**96594407 96594427 + (TG)n Simple_repeat 1 21**

**96594987 96595139 + LTR67 LTR/ERVL 33 189**

**96595354 96595664 C AluSc SINE/Alu 309 1 ( AluSc_2_57c )**

**96595665 96596086 + MER31-int LTR/ERV1 2516 2936**

**96596087 96596548 + MER66B LTR/ERV1 1 486**

**96596549 96596571 + MER31-int LTR/ERV1 2937 2955**

**96596572 96596868 C AluSg SINE/Alu 297 1**

**96596869 96597154 C AluSg SINE/Alu 301 1**

**96597155 96597248 + MER31-int LTR/ERV1 2956 3069**

**Ortholog in Chimp 97512341-97513708 Minus Nscore 0**

**N positions**

**97511350 97511370 + (TTG)n Simple_repeat 1 21**

**97511374 97511410 + (TG)n Simple_repeat 1 37**

**97511970 97512122 + LTR67 LTR/ERVL 33 189**

**97512336 97512639 C AluSc SINE/Alu 302 1 (R2)**

**97512651 97512808 C FAM SINE/Alu 168 10**

**97512843 97512902 + MER31-int LTR/ERV1 1062 1123**

**97513002 97513282 C AluJo SINE/Alu 307 3**

**97513334 97513362 + AT_rich Low_complexity 1 29**

**97513415 97513708 C AluSc SINE/Alu 293 1 (R1)**

**97513709 97514129 + MER31-int LTR/ERV1 2517 2936**

**97514130 97514591 + MER66B LTR/ERV1 1 486**

**97514592 97514614 + MER31-int LTR/ERV1 2937 2955**

**97514615 97514910 C AluSg SINE/Alu 296 1**

**97514911 97515187 C AluSg SINE/Alu 300 8**

**97515188 97515289 + MER31-int LTR/ERV1 2956 3069**

**__________________________________________________________________________________**

**AluY_2_60 100542796-100543106 INDEL_CAN**

**100541271 100541405 C L1MC4a LINE/L1 6073 5948**

**100541406 100541712 C AluSx SINE/Alu 306 1**

**100541713 100541784 C L1MC4a LINE/L1 5947 5888**

**100541846 100542660 C L1MC4a LINE/L1 5090 4152**

**100542796 100543106 + AluY SINE/Alu 1 311 ( AluY_2_60 )**

**100543107 100543482 C LOR1b LTR/ERV1 408 1**

**100543550 100543587 + AluSg SINE/Alu 1 38**

**100543589 100543851 + AluSg SINE/Alu 13 295**

**100543852 100544163 + AluSq SINE/Alu 1 306**

**100544221 100544332 C MER41B LTR/ERV1 113 2**

**Ortholog in Chimp 101570373-101571388 Plus Nscore 0**

**N positions**

**101568842 101568979 C L1MC4a LINE/L1 6073 5948**

**101568980 101569289 C AluSx SINE/Alu 309 1**

**101569290 101569361 C L1MC4a LINE/L1 5947 5888**

**101569423 101570237 C L1MC4a LINE/L1 5090 4152**

**101570373 101570668 + AluY SINE/Alu 1 297 (R1)**

**101570747 101571024 C MER41C LTR/ERV1 520 260**

**101571025 101571089 C LOR1b LTR/ERV1 461 397**

**101571090 101571389 + AluY SINE/Alu 1 300 (R2)**

**101571390 101571764 C LOR1b LTR/ERV1 396 2**

**101571765 101571870 C MER41C LTR/ERV1 259 192**

**101571871 101572135 + AluSg SINE/Alu 13 295**

**101572136 101572456 + AluSq SINE/Alu 1 306**

**101572457 101572627 C MER41C LTR/ERV1 191 6**

**__________________________________________________________________________________**

**AluY_2_70 229678758-229679066 INDEL_CAN**

**229677698 229677814 + MIR SINE/MIR 108 232**

**229678601 229678709 C MIRb SINE/MIR 231 107**

**229678758 229679066 + AluY SINE/Alu 1 310 ( AluY_2_70 )**

**229679394 229679755 + MLT1A0 LTR/MaLR 6 365**

**229679806 229680273 + MLT1C LTR/MaLR 6 467**

**229680464 229680487 + (A)n Simple_repeat 1 24**

**Ortholog in Chimp 235261995-235265651 Plus Nscore 0**

**N positions**

**235260871 235261064 + MIR SINE/MIR 81 265**

**235261048 235261128 + MIRb SINE/MIR 76 147**

**235261838 235261946 C MIRb SINE/MIR 231 107**

**235261995 235262311 + AluSx SINE/Alu 1 316 (R1)**

**235262765 235262942 + Charlie5 DNA/MER1_type 2403 2584**

**235262986 235263129 C MIR SINE/MIR 221 63**

**235263133 235263443 + AluY SINE/Alu 1 311**

**235263648 235264010 + MSTB1 LTR/MaLR 52 432**

**235265336 235265644 + AluY SINE/Alu 1 311 (R2)**

**235265979 235266340 + MLT1A0 LTR/MaLR 6 365**

**235266391 235266858 + MLT1C LTR/MaLR 6 467**

**235267049 235267074 + (A)n Simple_repeat 1 26**

**__________________________________________________________________________________**

**AluSq_3_75 10289305-10289433 INDEL_CAN**

**10288008 10288287 C AluSx SINE/Alu 288 1**

**10288393 10288713 C AluSx SINE/Alu 302 1**

**10288791 10288952 C FRAM SINE/Alu 166 1**

**10288969 10289286 C AluSx SINE/Alu 289 2**

**10289305 10289433 + AluSq/x SINE/Alu 14 142 ( AluSq_3_75 )**

**10289437 10289722 + AluJb SINE/Alu 1 291**

**10289912 10290147 + L3 LINE/CR1 1953 2201**

**10290159 10290296 + FLAM_C SINE/Alu 1 132**

**10290647 10290675 + AT_rich Low_complexity 1 29**

**Ortholog in Chimp 10566073-10566346 Plus Nscore 0**

**N positions**

**10564772 10565064 C AluSx SINE/Alu 301 1**

**10565171 10565479 C AluSx SINE/Alu 302 1**

**10565558 10565719 C FRAM SINE/Alu 166 1**

**10565736 10566054 C AluSx SINE/Alu 289 2**

**10566073 10566287 + AluSx SINE/Alu 14 218 (R1)**

**10566288 10566349 + AluSq/x SINE/Alu 84 145 (R2)**

**10566350 10566645 + AluJb SINE/Alu 1 291**

**10566650 10566788 + L3_Mars LINE/CR1 1556 1695**

**10566835 10567070 + L3 LINE/CR1 1878 2126**

**10567082 10567219 + FLAM_C SINE/Alu 1 132**

**10567570 10567598 + AT_rich Low_complexity 1 29**

**__________________________________________________________________________________**

**AluY_5_116c 31941874-31942048 INDEL_CAN**

**31940389 31940580 C AluY SINE/Alu 301 111**

**31940830 31941146 C AluSx SINE/Alu 302 1**

**31941874 31942048 C AluY SINE/Alu 310 136 ( AluY_5_116c )**

**31942130 31942156 + (TTG)n Simple_repeat 1 27**

**31942157 31942239 C AluJ SINE/Alu 281 182**

**31942240 31942372 C AluJ SINE/Alu 133 1**

**31942424 31942725 + AluSx SINE/Alu 1 302**

**31943005 31943349 C MLT1A LTR/MaLR 374 1**

**Ortholog in Chimp 83875072-83875652 Plus Nscore 0**

**N positions**

**83873754 83874111 + MLT1A LTR/MaLR 1 374**

**83874391 83874692 C AluSx SINE/Alu 302 1**

**83874744 83874962 + AluJb SINE/Alu 1 284**

**83874963 83874988 + (CAA)n Simple_repeat 2 27**

**83875071 83875362 + AluSx SINE/Alu 1 294**

**83875366 83875652 + AluY SINE/Alu 1 310**

**83876380 83876697 + AluSx SINE/Alu 1 302**

**83876948 83877143 + AluY SINE/Alu 111 305**

**__________________________________________________________________________________**

**AluSp_5_117c 36598166-36598498 INDEL_CAN**

**36596439 36596743 + AluSx SINE/Alu 1 305**

**36596913 36597207 C AluSx SINE/Alu 296 1**

**36597211 36597277 C L2 LINE/L2 2937 2871**

**36597287 36597427 C MER5A1 DNA/MER1_type 160 1**

**36598166 36598498 C AluSp SINE/Alu 334 1 ( AluSp_5_117c )**

**36598645 36598757 C L2 LINE/L2 3419 3262**

**36598849 36599067 + L1M3 LINE/L1 5191 5428**

**36599137 36599433 C AluSg SINE/Alu 297 1**

**36599985 36600286 C AluSx SINE/Alu 303 3**

**Ortholog in Chimp 79110839-79113508 Plus Nscore 0.37**

**N positions 79112635-79112644;**

**79109048 79109351 + AluSx SINE/Alu 1 303**

**79109903 79110201 + AluSg SINE/Alu 1 299**

**79110271 79110507 C L1P LINE/L1 5428 5189**

**79110580 79110689 + L2 LINE/L2 3262 3375**

**79110789 79110838 + L2 LINE/L2 2774 2824**

**79110839 79111148 + AluSp SINE/Alu 1 311 (R1)**

**79111149 79111664 + L2 LINE/L2 2825 3415**

**79111866 79111891 + AT_rich Low_complexity 1 26**

**79112011 79112266 + AluSx SINE/Alu 27 282**

**79112279 79112406 + L1ME4a LINE/L1 5991 6121**

**79112430 79112631 + AluSx SINE/Alu 1 203**

**79112647 79112671 + AT_rich Low_complexity 1 25**

**79112791 79113046 + AluSx SINE/Alu 27 282**

**79113204 79113508 + AluSq SINE/Alu 1 306 (R2)**

**79114247 79114386 + MER5A1 DNA/MER1_type 1 160**

**79114396 79114462 + L2 LINE/L2 2871 2937**

**79114466 79114760 + AluSx SINE/Alu 1 296**

**79114930 79115234 C AluSx SINE/Alu 305 1**

**__________________________________________________________________________________**

**AluSx_5_118 55281840-55282152 INDEL_CAN**

**55280638 55280762 + L1ME4a LINE/L1 5956 6098**

**55280780 55281079 + AluJo SINE/Alu 1 302**

**55281168 55281204 + AT_rich Low_complexity 1 37**

**55281840 55282152 + AluSx SINE/Alu 1 312 ( AluSx_5_118 )**

**55282286 55282568 + AluJb SINE/Alu 1 312**

**Ortholog in Chimp 59940444-59941110 Minus Nscore 0**

**N positions**

**59939840 59940034 + L2 LINE/L2 1850 2051**

**59940035 59940310 C AluJb SINE/Alu 305 1**

**59940311 59940441 + L2 LINE/L2 2052 2209**

**59940442 59940748 C AluY SINE/Alu 303 2 (R2)**

**59940749 59940805 + L2 LINE/L2 2210 2256**

**59940806 59941110 C AluSq SINE/Alu 306 1 (R1)**

**59941111 59941195 + L2 LINE/L2 2257 2353**

**59941741 59941777 + AT_rich Low_complexity 1 37**

**59941866 59942165 C AluJo SINE/Alu 302 1**

**59942183 59942307 C L1ME4a LINE/L1 6098 5956**

**__________________________________________________________________________________**

**AluSq_5_124c 76146126-76146442 INDEL_CAN**

**76143150 76145255 + SVA Other 1 1375**

**76145256 76145279 + AT_rich Low_complexity 1 24**

**76145285 76145593 + AluSg SINE/Alu 3 308**

**76146126 76146442 C AluSq SINE/Alu 313 1 ( AluSq_5_124c )**

**76146460 76146747 C AluSx SINE/Alu 296 1**

**76146760 76147040 C AluJo SINE/Alu 289 1**

**76147041 76147352 C L2 LINE/L2 2565 2254**

**76147353 76147656 + AluJb SINE/Alu 2 304**

**76147657 76147946 C L2 LINE/L2 2253 1968**

**Ortholog in Chimp 39028997-39029867 Plus Nscore 0**

**N positions**

**39027465 39027768 + L2 LINE/L2 1968 2267**

**39027769 39028080 C AluJb SINE/Alu 312 2**

**39028081 39028392 + L2 LINE/L2 2268 2565**

**39028393 39028670 + AluJo SINE/Alu 1 287**

**39028671 39028684 + L2 LINE/L2 2566 2570**

**39028685 39028980 + AluSx SINE/Alu 1 304**

**39028981 39028996 + L2 LINE/L2 2571 2570**

**39028997 39029304 + AluSq SINE/Alu 1 311 (R1)**

**39029305 39029575 + L2 LINE/L2 2571 2854**

**39029576 39029867 + AluSp SINE/Alu 2 303 (R2)**

**39029868 39030032 + L2 LINE/L2 2855 3008**

**39030398 39030702 C AluSg SINE/Alu 304 3**

**39031117 39031419 + MER61A LTR/ERV1 1 343**

**__________________________________________________________________________________**

**AluSg_5_128 125947971-125948270 INDEL_CAN**

**125946792 125947100 + AluSq SINE/Alu 3 311**

**125947101 125947124 + AT_rich Low_complexity 1 24**

**125947145 125947440 + AluSx SINE/Alu 1 294**

**125947971 125948270 + AluSg SINE/Alu 1 300 ( AluSg_5_128 )**

**125948278 125948508 C L2 LINE/L2 2683 2433**

**125948608 125948904 + AluSq SINE/Alu 1 298**

**125949086 125949145 + L1MC LINE/L1 5552 5608**

**125949146 125949437 C AluSx SINE/Alu 305 17**

**125949438 125949609 + L1MC LINE/L1 5609 5793**

**Ortholog in Chimp 128129287-128130557 Plus Nscore 0**

**N positions**

**128128112 128128416 + AluSq SINE/Alu 3 307**

**128128417 128128437 + AT_rich Low_complexity 1 21**

**128128461 128128756 + AluSx SINE/Alu 1 294**

**128129287 128129586 + AluSg SINE/Alu 1 300 (R1)**

**128129709 128129925 C L2 LINE/L2 2944 2727**

**128129926 128130222 + AluSq SINE/Alu 1 297**

**128130223 128130256 C L2 LINE/L2 2726 2692**

**128130257 128130557 + AluY SINE/Alu 1 301 (R2)**

**128130558 128130795 C L2 LINE/L2 2691 2434**

**128130895 128131184 + AluSq SINE/Alu 1 291**

**128131627 128131905 C AluSx SINE/Alu 292 17**

**128131910 128132077 + L1MC LINE/L1 5607 5793**

**__________________________________________________________________________________**

**AluSg_6_139c 1126760-1126982 INDEL_CAN**

**1125690 1125719 + AT_rich Low_complexity 1 30**

**1125954 1126115 + L1MEb LINE/L1 306 467**

**1126285 1126758 + L1MEb LINE/L1 1186 1767**

**1126760 1126982 C AluSg/x SINE/Alu 307 88 ( AluSg_6_139c )**

**1127052 1127253 C AluJb SINE/Alu 303 107**

**1127263 1127389 + MIRb SINE/MIR 131 268**

**1128150 1128383 C MIRb SINE/MIR 256 9**

**Ortholog in Chimp 1148065-1149143 Minus Nscore 0**

**N positions**

**1147261 1147420 + L1MEb LINE/L1 306 467**

**1147590 1148063 + L1MEb LINE/L1 1186 1767**

**1148065 1148296 C AluSg/x SINE/Alu 297 68 (R2)**

**1148923 1149143 C AluSg/x SINE/Alu 306 88 (R1)**

**1149213 1149412 C AluJb SINE/Alu 303 107**

**1149422 1149549 + MIRb SINE/MIR 131 268**

**1150310 1150543 C MIRb SINE/MIR 256 9**

**__________________________________________________________________________________**

**AluY_6_146c 35547210-35547497 INDEL_CAN**

**35546897 35547046 C AluSp SINE/Alu 297 127**

**35547210 35547497 C AluY SINE/Alu 294 1 ( AluY_6_146c )**

**35547547 35547688 + AluSg/x SINE/Alu 134 280**

**35548182 35548305 + MIRb SINE/MIR 36 170**

**35548318 35548439 + MER53 DNA 2 192**

**35548585 35548892 C AluY SINE/Alu 298 1**

**Ortholog in Chimp 36231699-36232620 Minus Nscore 0**

**N positions**

**36231369 36231525 C AluSp SINE/Alu 296 121**

**36231699 36231998 C AluY SINE/Alu 305 1 (R1)**

**36232041 36232296 C AluSq SINE/Alu 275 11**

**36232315 36232620 C AluY SINE/Alu 304 1 (R2)**

**36232670 36232811 + AluSg/x SINE/Alu 134 280**

**36233301 36233422 + MIRb SINE/MIR 36 170**

**36233435 36233556 + MER53 DNA 2 192**

**36233709 36234012 C AluY SINE/Alu 297 1**

**__________________________________________________________________________________**

**AluSq_6_147 35789925-35790236 INDEL_CAN**

**35788685 35788861 + AluJb SINE/Alu 136 312**

**35789125 35789309 C MIR SINE/MIR 262 59**

**35789631 35789716 + L2 LINE/L2 3071 3165**

**35789925 35790236 + AluSq SINE/Alu 1 312 ( AluSq_6_147 )**

**35790291 35790585 + AluSq SINE/Alu 2 299**

**35790667 35790696 + AT_rich Low_complexity 1 30**

**35790706 35790953 C AluSx SINE/Alu 257 2**

**35790994 35791175 + MIR SINE/MIR 25 204**

**35791283 35791848 C L2 LINE/L2 3113 2509**

**Ortholog in Chimp 36480283-36481262 Plus Nscore 0**

**N positions**

**36479045 36479219 + AluJb SINE/Alu 136 310**

**36479483 36479667 C MIR SINE/MIR 262 59**

**36479989 36480074 + L2 LINE/L2 3071 3165**

**36480283 36480441 + AluSx SINE/Alu 1 159 (R1)**

**36480442 36480549 C AluSq SINE/Alu 210 103**

**36480557 36480710 + AluSx SINE/Alu 157 311**

**36480758 36480919 + FRAM SINE/Alu 1 166**

**36480929 36481243 + AluSq SINE/Alu 1 313 (R2)**

**36481317 36481609 + AluSq SINE/Alu 2 297**

**36481691 36481720 + AT_rich Low_complexity 1 30**

**36481730 36481977 C AluSx SINE/Alu 257 2**

**36482077 36482285 + MIR SINE/MIR 1 205**

**36482392 36482965 C L2 LINE/L2 3113 2497**

**__________________________________________________________________________________**

**AluJb_6_148 36504751-36505041 INDEL_CAN**

**36503254 36503580 C MER33 DNA/MER1_type 324 8**

**36503920 36504160 + AluY SINE/Alu 1 242**

**36504502 36504557 C AluYc3 SINE/Alu 283 230**

**36504558 36504710 C AluSx SINE/Alu 153 1**

**36504751 36505041 + AluJb SINE/Alu 12 297 ( AluJb_6_148 )**

**36505248 36505559 C AluSq SINE/Alu 313 1**

**36505672 36505974 + AluSx SINE/Alu 1 295**

**36506046 36506499 + MLT1D LTR/MaLR 1 505**

**Ortholog in Chimp 37214664-37215352 Plus Nscore 0**

**N positions**

**37213159 37213480 C MER33 DNA/MER1_type 324 8**

**37213820 37214089 + AluY SINE/Alu 1 300**

**37214412 37214470 C AluSx SINE/Alu 296 240**

**37214471 37214623 C AluSx SINE/Alu 153 1**

**37214664 37214967 + AluJb SINE/Alu 12 310 (R1)**

**37215056 37215353 + AluSx SINE/Alu 1 298 (R2)**

**37215551 37215851 C AluSq SINE/Alu 312 1**

**37215965 37216265 + AluSx SINE/Alu 1 293**

**37216337 37216790 + MLT1D LTR/MaLR 1 505**

**37216832 37217004 C AluSx SINE/Alu 303 157**

**__________________________________________________________________________________**

**AluSq_7_163 47596519-47596825 INDEL_CAN**

**47595193 47595496 + AluSx SINE/Alu 1 311**

**47595510 47595806 + AluSx SINE/Alu 1 297**

**47596042 47596109 + CT-rich Low_complexity 6 71**

**47596135 47596292 + CT-rich Low_complexity 2 161**

**47596315 47596447 + CT-rich Low_complexity 2 136**

**47596519 47596825 + AluSq SINE/Alu 1 309 ( AluSq_7_163 )**

**47596845 47597150 + AluSp SINE/Alu 1 307**

**47597311 47597491 + FRAM SINE/Alu 1 175**

**47597510 47597671 + MIR SINE/MIR 81 228**

**Ortholog in Chimp 48369861-48370950 Plus Nscore 0**

**N positions**

**48368531 48368834 + AluSx SINE/Alu 1 311**

**48368848 48369148 + AluSx SINE/Alu 1 301**

**48369384 48369451 + CT-rich Low_complexity 2 71**

**48369477 48369634 + CT-rich Low_complexity 2 161**

**48369657 48369789 + CT-rich Low_complexity 2 136**

**48369861 48370155 + AluSg SINE/Alu 1 295 (R1)**

**48370167 48370461 + AluSx SINE/Alu 1 295**

**48370656 48370965 + AluSp SINE/Alu 1 311 (R2)**

**48370970 48371275 + AluSp SINE/Alu 1 307**

**48371436 48371616 + FRAM SINE/Alu 1 175**

**48371620 48371825 + MIR SINE/MIR 74 274**

**48371978 48372093 C MER5A DNA/MER1_type 188 67**

**__________________________________________________________________________________**

**AluSq_7_164c 62575196-62575500 OCCUPIED**

**62573567 62573908 + L1M4 LINE/L1 5167 5519**

**62573985 62574109 C AluJ SINE/Alu 291 177**

**62574159 62574191 C L1M4 LINE/L1 5251 5219**

**62574208 62574376 C AluJ SINE/Alu 175 3**

**62575196 62575500 C AluSq SINE/Alu 305 1 ( AluSq_7_164c )**

**62575539 62575823 C AluSx SINE/Alu 296 1**

**62575880 62576014 + FLAM_C SINE/Alu 1 137**

**62576018 62576306 + AluY SINE/Alu 33 321**

**Ortholog in Chimp 58280929-58281233 Plus Nscore 0**

**N positions**

**58280141 58280409 C AluY SINE/Alu 301 33**

**58280413 58280547 C FLAM_C SINE/Alu 137 1**

**58280604 58280889 + AluSx SINE/Alu 1 297**

**58280929 58281234 + AluSq SINE/Alu 1 306 (OCCUPIED)**

**58282054 58282222 + AluJ SINE/Alu 3 175**

**58282239 58282271 + L1M4 LINE/L1 5219 5251**

**58282321 58282443 + AluJ SINE/Alu 177 293**

**58282520 58282844 C L1M4 LINE/L1 5519 5167**

**__________________________________________________________________________________**

**AluSq_7_180 148347589-148347842 OCCUPIED**

**148345973 148346274 + AluY SINE/Alu 1 302**

**148346275 148346406 + MER5A DNA/MER1_type 76 189**

**148346461 148346756 C AluSx SINE/Alu 296 1**

**148346813 148346876 + (CACCC)n Simple_repeat 3 67**

**148347298 148347437 + MSTB2 LTR/MaLR 3 134**

**148347463 148347588 + AluSq/x SINE/Alu 1 126**

**148347589 148347842 + AluSq SINE/Alu 36 290 ( AluSq_7_180 )**

**148347852 148347975 + Charlie1 DNA/MER1_type 2629 2758**

**148348010 148348285 C AluSx SINE/Alu 276 1**

**148349100 148349146 + CT-rich Low_complexity 2 48**

**148349234 148349536 + AluSg SINE/Alu 1 303**

**Ortholog in Chimp 149658220-149658473 Plus Nscore 0**

**N positions**

**149657243 149657407 + MSTB1 LTR/MaLR 3 201**

**149657408 149657711 + AluSq SINE/Alu 1 304**

**149657712 149657943 + MSTB1 LTR/MaLR 202 432**

**149657993 149658102 + MIRb SINE/MIR 1 133**

**149658108 149658217 + AluJb SINE/Alu 16 125**

**149658220 149658473 + AluSq SINE/Alu 36 290 (OCCUPIED)**

**149658483 149658600 + Charlie1 DNA/MER1_type 2629 2754**

**149658636 149658914 C AluSx SINE/Alu 279 1**

**149659729 149659775 + CT-rich Low_complexity 2 48**

**149659863 149660154 + AluSg SINE/Alu 1 292**

**__________________________________________________________________________________**

**AluY_8_188 12960787-12961098 INDEL_CAN**

**12959416 12959535 + FRAM SINE/Alu 42 161**

**12959690 12959999 C AluY SINE/Alu 311 1**

**12960387 12960476 + MIR3 SINE/MIR 116 205**

**12960611 12960756 + FRAM SINE/Alu 1 146**

**12960787 12961098 + AluY SINE/Alu 1 311 ( AluY_8_188 )**

**12961900 12962233 + AluJo SINE/Alu 1 286**

**12962351 12962553 C THE1D LTR/MaLR 381 165**

**12962554 12962863 + AluY SINE/Alu 3 312**

**Ortholog in Chimp 9020216-9020681 Plus Nscore 0**

**N positions**

**9019152 9019431 C AluY SINE/Alu 281 1**

**9019818 9019907 + MIR3 SINE/MIR 116 205**

**9020042 9020215 + AluJb SINE/Alu 1 145**

**9020216 9020513 + AluY SINE/Alu 1 297 (R1)**

**9020514 9020681 + AluJb SINE/Alu 146 299 (R2)**

**9021483 9021818 + AluJo SINE/Alu 1 286**

**9021936 9022138 C THE1D LTR/MaLR 381 165**

**9022139 9022453 + AluY SINE/Alu 3 317**

**__________________________________________________________________________________**

**AluSg_9_202c 1082540-1082850 INDEL_CAN**

**1079944 1081130 + L1MC4 LINE/L1 6806 8032**

**1081192 1081392 + AluJo SINE/Alu 87 311**

**1081977 1082038 C L1MB3 LINE/L1 6176 6115**

**1082039 1082531 + Tigger2b_Pri DNA/MER2_type 6 516**

**1082540 1082850 C AluSg SINE/Alu 310 2 ( AluSg_9_202c )**

**1082851 1083535 C MER49 LTR/ERV1 881 5**

**1083538 1084027 + Tigger2b_Pri DNA/MER2_type 572 1068**

**1084030 1084685 C L1MB3 LINE/L1 6119 5417**

**Ortholog in Chimp 1106713-1107449 Minus Nscore 0**

**N positions**

**1104150 1105303 + L1MC4 LINE/L1 6806 8032**

**1105365 1105566 + AluJo SINE/Alu 87 312**

**1106150 1106199 C L1MB3 LINE/L1 6176 6125**

**1106207 1106704 + Tigger2b_Pri DNA/MER2_type 1 516**

**1106705 1107015 C AluY SINE/Alu 311 1 (R2)**

**1107016 1107090 + Tigger2b_Pri DNA/MER2_type 517 576**

**1107100 1107136 C MER49 LTR/ERV1 913 882**

**1107137 1107449 C AluSg SINE/Alu 312 2 (R1)**

**1107450 1108134 C MER49 LTR/ERV1 881 5**

**1108143 1108621 + Tigger2b_Pri DNA/MER2_type 577 1068**

**1108624 1109206 C L1MB3 LINE/L1 6119 5535**

**__________________________________________________________________________________**

**AluSx_9_203 16802925-16803207 INDEL_CAN**

**16801267 16801573 + AluSq SINE/Alu 1 306**

**16802789 16802809 + AT_rich Low_complexity 1 21**

**16802925 16803207 + AluSx SINE/Alu 1 296 ( AluSx_9_203 )**

**16803265 16803562 C AluY SINE/Alu 298 1**

**Ortholog in Chimp 17216670-17217680 Plus Nscore 40.3**

**N positions 17217271-17217677;**

**17215012 17215302 + AluSq SINE/Alu 1 290**

**17215303 17215324 + (CAA)n Simple_repeat 2 23**

**17216534 17216554 + AT_rich Low_complexity 1 21**

**17216670 17216975 + AluY SINE/Alu 1 306 (R1)**

**17216988 17217270 + AluSx SINE/Alu 1 296 (R2)**

**17217737 17218036 C AluY SINE/Alu 300 1**

**__________________________________________________________________________________**

**AluSx_9_215 131093731-131094006 INDEL_CAN**

**131092262 131092669 + AluJb SINE/Alu 1 402**

**131093149 131093277 + L2 LINE/L2 3291 3418**

**131093530 131093610 C L2 LINE/L2 3160 3080**

**131093661 131093726 C L1MEc LINE/L1 3598 3532**

**131093731 131094006 + AluSx SINE/Alu 11 301 ( AluSx_9_215 )**

**131094033 131094339 C L1MEc LINE/L1 2973 2495**

**131094340 131094652 C AluSx SINE/Alu 312 1**

**131094653 131094721 C L1MEc LINE/L1 2494 2427**

**131094734 131094872 C AluJo SINE/Alu 136 2**

**131094897 131095057 + AluSg/x SINE/Alu 134 294**

**131095058 131095199 C L1MD2 LINE/L1 6342 6195**

**131095201 131095441 C L1MD2 LINE/L1 6142 5885**

**131095442 131095785 C AluJo SINE/Alu 301 2**

**Ortholog in Chimp 129147311-129147869 Plus Nscore 0**

**N positions**

**129145921 129146238 + AluJb SINE/Alu 1 312**

**129146729 129146857 + L2 LINE/L2 3291 3418**

**129147241 129147306 C L1MEc LINE/L1 3598 3532**

**129147311 129147462 + AluSx SINE/Alu 11 170 (R1)**

**129147490 129147514 + AT_rich Low_complexity 1 25**

**129147584 129147869 + AluSx SINE/Alu 1 301 (R2)**

**129147896 129148092 C L1MEc LINE/L1 2973 2774**

**129148201 129148500 C AluSx SINE/Alu 299 1**

**129148501 129148569 C L1MEc LINE/L1 2493 2426**

**129148574 129148595 + AT_rich Low_complexity 1 22**

**129148596 129148721 C FLAM_C SINE/Alu 126 1**

**129148745 129148905 + AluSg/x SINE/Alu 134 294**

**129148906 129149046 C L1MD2 LINE/L1 6342 6195**

**129149048 129149289 C L1MD2 LINE/L1 6142 5885**

**129149290 129149634 C AluJo SINE/Alu 301 2**

**__________________________________________________________________________________**

**AluSq_9_219 134225813-134225988 INDEL_CAN**

**134224684 134224828 C L1MEd LINE/L1 3306 3163**

**134224829 134225141 C AluJo SINE/Alu 309 1**

**134225142 134225518 C L1MEd LINE/L1 3162 2784**

**134225520 134225812 + AluSg SINE/Alu 1 295**

**134225813 134225988 + AluSq SINE/Alu 134 312 ( AluSq_9_219 )**

**134226001 134226386 C L1MEd LINE/L1 2797 2477**

**134226387 134226686 C AluSp SINE/Alu 300 1**

**134226687 134227030 C L1MEd LINE/L1 2483 2132**

**134227031 134227345 C AluY SINE/Alu 298 1**

**134227346 134227379 C L1MEd LINE/L1 2131 2098**

**134227406 134227490 C LTR43B LTR/ERV1 572 489**

**Ortholog in Chimp 132409802-132410295 Plus Nscore 0**

**N positions**

**132408658 132408808 C L1M4c LINE/L1 3306 3159**

**132408809 132409127 C AluJo SINE/Alu 311 1**

**132409128 132409504 C L1M4c LINE/L1 3158 2784**

**132409506 132409801 + AluSg SINE/Alu 1 298**

**132409803 132409973 + AluSq SINE/Alu 135 305 (R1)**

**132409985 132410295 + AluSx SINE/Alu 1 311 (R2)**

**132410308 132410689 C L1M4c LINE/L1 2797 2477**

**132410690 132410987 C AluSp SINE/Alu 298 1**

**132410988 132411313 C L1M4c LINE/L1 2483 2148**

**132411330 132411642 C AluY SINE/Alu 301 5**

**__________________________________________________________________________________**

**AluSq_10_224c 15125472-15125767 OCCUPIED**

**15123882 15124046 + L2 LINE/L2 2086 2257**

**15124157 15124457 C AluSq SINE/Alu 300 1**

**15124458 15124634 C L1MA1 LINE/L1 6260 6076**

**15124638 15124767 + L2 LINE/L2 2563 2693**

**15124771 15124854 + MIR SINE/MIR 2 84**

**15124855 15125164 C AluY SINE/Alu 307 1**

**15125165 15125296 + MIR SINE/MIR 85 213**

**15125472 15125767 C AluSq SINE/Alu 311 6 ( AluSq_10_224c )**

**15126040 15126440 C L1MEe LINE/L1 2655 2205**

**15126450 15126538 C AluS SINE/Alu 306 221**

**15126539 15127055 C L1MEe LINE/L1 2111 1529**

**Ortholog in Chimp 15635451-15635746 Minus Nscore 0**

**N positions**

**15634115 15634415 C AluSq SINE/Alu 300 1**

**15634416 15634597 C L1MA1 LINE/L1 6260 6076**

**15634744 15634817 + MIR SINE/MIR 12 84**

**15634818 15635138 C AluY SINE/Alu 303 1**

**15635139 15635274 + MIR SINE/MIR 85 213**

**15635450 15635746 C AluSq SINE/Alu 312 6 (OCCUPIED)**

**15635976 15636003 + AT_rich Low_complexity 1 28**

**15636019 15636419 C L1MEe LINE/L1 2655 2205**

**15636429 15636517 C AluS SINE/Alu 306 221**

**15636518 15637035 C L1MEe LINE/L1 2111 1529**

**__________________________________________________________________________________**

**AluSg_10_229c 37101729-37102038 INDEL_CAN**

**37101275 37101467 C MIR SINE/MIR 226 28**

**37101476 37101704 + AluJo SINE/Alu 71 292**

**37101729 37102038 C AluSg SINE/Alu 310 1 ( AluSg_10_229c )**

**37102039 37102132 C AluSc SINE/Alu 134 41**

**37102136 37102418 C L1MEe LINE/L1 2320 2024**

**37102469 37102797 C L1P4 LINE/L1 4504 4210**

**37102802 37103152 C L1MEe LINE/L1 1775 1312**

**37103216 37103490 C AluSx SINE/Alu 277 1**

**37103515 37104046 C L1MEe LINE/L1 1196 662**

**Ortholog in Chimp 37595031-37595497 Minus Nscore 0**

**N positions**

**37594578 37594769 C MIR SINE/MIR 226 28**

**37594778 37595007 + AluJo SINE/Alu 71 293**

**37595029 37595192 C AluSc SINE/Alu 295 135 (R2)**

**37595193 37595497 C AluSg SINE/Alu 304 1 (R1)**

**37595498 37595591 C AluSc SINE/Alu 134 41**

**37595595 37595870 C L1MEe LINE/L1 2320 2024**

**37595921 37596251 C L1M2 LINE/L1 4501 4207**

**37596256 37596603 C L1MEe LINE/L1 1775 1300**

**37596656 37596930 C AluSx SINE/Alu 277 1**

**37596955 37597441 C L1MEe LINE/L1 1196 713**

**__________________________________________________________________________________**

**AluSx_10_239 126511122-126511422 OCCUPIED**

**126510402 126510717 C AluSx SINE/Alu 311 1**

**126511122 126511422 + AluSx SINE/Alu 1 304 ( AluSx_10_239 )**

**126511425 126511734 + AluSg SINE/Alu 1 310**

**126511744 126512036 + AluJb SINE/Alu 1 295**

**126512113 126512224 C MIRb SINE/MIR 140 22**

**126512228 126512328 + L1ME4a LINE/L1 5998 6110**

**126512701 126512880 + MIR SINE/MIR 23 197**

**Ortholog in Chimp 125767949-125768249 Plus Nscore 0**

**N positions**

**125767232 125767544 C AluSx SINE/Alu 303 1**

**125767949 125768249 + AluSx SINE/Alu 1 304 (OCCUPIED)**

**125768252 125768558 + AluSg SINE/Alu 1 309**

**125768572 125768865 + AluJb SINE/Alu 1 295**

**125768942 125769054 C MIRb SINE/MIR 140 22**

**125769058 125769158 + L1ME4a LINE/L1 5998 6110**

**125769531 125769710 + MIR SINE/MIR 23 197**

**__________________________________________________________________________________**

**AluJb_11_261 107489002-107489297 INDEL_CAN**

**107487446 107487743 C AluJo SINE/Alu 287 1**

**107487847 107487961 + AluJo SINE/Alu 6 135**

**107487963 107488287 + L2 LINE/L2 2036 2419**

**107488505 107488528 + AT_rich Low_complexity 1 24**

**107489002 107489297 + AluJb SINE/Alu 1 308 ( AluJb_11_261 )**

**107489298 107489457 + AluSg/x SINE/Alu 137 298**

**107489458 107489902 C MER65D LTR/ERV1 453 1**

**107490112 107490549 C MLT1C LTR/MaLR 466 6**

**107490555 107490837 C MLT1C-int LTR/MaLR 1371 1121**

**Ortholog in Chimp 106821516-106822334 Plus Nscore 0**

**N positions**

**106819973 106820270 C AluJo SINE/Alu 287 1**

**106820374 106820488 + AluJo SINE/Alu 6 135**

**106820490 106820819 + L2 LINE/L2 2036 2419**

**106821516 106821809 + AluJb SINE/Alu 1 306 (R1)**

**106821834 106821858 + (TTTTTG)n Simple_repeat 3 28**

**106821873 106821898 C MER65D LTR/ERV1 473 454**

**106821899 106822032 + AluSg SINE/Alu 1 131**

**106822033 106822331 + AluY SINE/Alu 1 298 (R2)**

**106822332 106822494 + AluSg SINE/Alu 132 296**

**106822495 106822939 C MER65D LTR/ERV1 453 3**

**106823177 106823614 C MLT1C LTR/MaLR 466 6**

**106823620 106823902 C MLT1C-int LTR/MaLR 1371 1121**

**__________________________________________________________________________________**

**AluSp_11_264 118629440-118629585 INDEL_CAN**

**118628494 118628619 + AluSq SINE/Alu 1 126**

**118628620 118628649 + (CAAA)n Simple_repeat 4 33**

**118628650 118628833 + AluSq SINE/Alu 127 312**

**118629074 118629159 + L1MA7 LINE/L1 6202 6291**

**118629257 118629387 + FLAM_A SINE/Alu 1 132**

**118629440 118629585 + AluSp SINE/Alu 137 282 ( AluSp_11_264 )**

**118629586 118629606 + (TAA)n Simple_repeat 1 21**

**118629613 118629776 + FRAM SINE/Alu 2 166**

**118629793 118629925 + FLAM_C SINE/Alu 2 133**

**118629988 118630114 + FLAM_C SINE/Alu 2 130**

**118630123 118630195 + L1MB4 LINE/L1 6109 6180**

**118630244 118630517 C MER7A DNA/MER2_type 346 67**

**118630518 118630821 C AluSx SINE/Alu 304 1**

**Ortholog in Chimp 118138733-118139144 Plus Nscore 0**

**N positions**

**118137789 118138124 + AluSq SINE/Alu 1 311**

**118138365 118138446 + L1MA7 LINE/L1 6202 6286**

**118138549 118138680 + FLAM_A SINE/Alu 1 133**

**118138732 118138863 + AluJo SINE/Alu 1 131 (R1)**

**118138864 118139144 + AluSq SINE/Alu 1 282 (R2)**

**118139145 118139168 + (TAA)n Simple_repeat 1 24**

**118139169 118139340 + AluJo SINE/Alu 132 304**

**118139350 118139482 + FLAM_C SINE/Alu 2 133**

**118139548 118139674 + FLAM_C SINE/Alu 2 130**

**118139683 118139747 + L1MB4 LINE/L1 6109 6175**

**118139804 118140077 C MER7A DNA/MER2_type 346 67**

**118140078 118140380 C AluSx SINE/Alu 304 1**

**__________________________________________________________________________________**

**AluSg_11_266 133430562-133430859 INDEL_CAN**

**133429204 133429489 + AluSq SINE/Alu 2 294**

**133429497 133429805 + AluJo SINE/Alu 1 310**

**133430073 133430296 C LTR16A1 LTR/ERVL 457 205**

**133430427 133430561 + AluSq/x SINE/Alu 1 135**

**133430562 133430859 + AluSg SINE/Alu 1 299 ( AluSg_11_266 )**

**133431218 133431528 + AluSx SINE/Alu 1 312**

**133431618 133432240 C L1MEe LINE/L1 2006 1377**

**133432344 133432646 + AluY SINE/Alu 1 302**

**Ortholog in Chimp 133219137-133219601 Plus Nscore 0**

**N positions**

**133217783 133218067 + AluSq SINE/Alu 2 293**

**133218075 133218383 + AluJo SINE/Alu 1 310**

**133218651 133218989 C LTR16A1 LTR/ERVL 457 74**

**133219002 133219292 + AluSx SINE/Alu 1 303 (R1)**

**133219303 133219604 + AluSg SINE/Alu 1 302 (R2)**

**133219963 133220125 + AluSx SINE/Alu 1 165**

**133220305 133220506 + AluSc SINE/Alu 108 309**

**133220601 133221224 C L1MEe LINE/L1 2006 1326**

**__________________________________________________________________________________**

**AluY_12_267 6701854-6702159 INDEL_CAN**

**6700286 6700464 C MIR3 SINE/MIR 203 6**

**6700493 6700817 C AluSx SINE/Alu 312 1**

**6700825 6701148 C AluSx SINE/Alu 312 1**

**6701173 6701319 + MIR SINE/MIR 34 191**

**6701334 6701635 + AluSg SINE/Alu 1 304**

**6701645 6701743 + AluJ/FRAM SINE/Alu 214 312**

**6701854 6702159 + AluY SINE/Alu 1 307 ( AluY_12_267 )**

**6702262 6702335 + CT-rich Low_complexity 3 78**

**6702456 6702581 + MIRb SINE/MIR 80 222**

**6702730 6702782 + MIRm SINE/MIR 101 153**

**6702891 6702980 + MIRb SINE/MIR 168 265**

**Ortholog in Chimp 6944680-6945187 Plus Nscore 0**

**N positions**

**6943119 6943297 C MIR3 SINE/MIR 203 6**

**6943329 6943650 C AluSx SINE/Alu 311 1**

**6943665 6943992 C AluSx SINE/Alu 311 1**

**6944017 6944160 + MIR SINE/MIR 34 191**

**6944175 6944473 + AluSg SINE/Alu 1 301**

**6944483 6944578 + AluJ/FRAM SINE/Alu 214 309**

**6944680 6944985 + AluY SINE/Alu 1 307 (R1)**

**6944993 6945187 + AluSp SINE/Alu 109 304 (R2)**

**6945290 6945363 + CT-rich Low_complexity 3 81**

**6945503 6945609 + MIRb SINE/MIR 103 222**

**6945758 6945810 + MIRm SINE/MIR 101 153**

**6945934 6946009 + THER1_MD SINE/MIR 182 269**

**__________________________________________________________________________________**

**AluSx_12_272c 92284735-92285052 INDEL_CAN**

**92283171 92283480 C AluY SINE/Alu 310 1**

**92284735 92285052 C AluSx SINE/Alu 310 1 ( AluSx_12_272c )**

**92285053 92285330 C AluY SINE/Alu 296 1**

**92285369 92285682 + AluJb SINE/Alu 1 314**

**92285683 92285712 + AT_rich Low_complexity 1 30**

**92285915 92286200 + AluSc SINE/Alu 1 295**

**Ortholog in Chimp 94313575-94314546 Minus Nscore 0**

**N positions**

**94312009 94312319 C AluY SINE/Alu 311 1**

**94313205 94313301 C THER1_MD SINE/MIR 213 127**

**94313582 94313767 C AluSg/x SINE/Alu 310 130 (R2)**

**94313798 94314085 + AluJb SINE/Alu 1 299**

**94314232 94314546 C AluSx SINE/Alu 312 1 (R1)**

**94314547 94314716 C AluSc SINE/Alu 296 111**

**94314774 94315089 + AluJb SINE/Alu 1 314**

**94315090 94315119 + AT_rich Low_complexity 1 30**

**94315322 94315611 + AluSc SINE/Alu 1 299**

**__________________________________________________________________________________**

**AluY_13_281c 30412460-30412769 INDEL_CAN**

**30410825 30411099 + L2 LINE/L2 2026 2326**

**30412460 30412769 C AluY SINE/Alu 310 1 ( AluY_13_281c )**

**30412879 30413096 + AluSg/x SINE/Alu 87 304**

**30413129 30413334 + MIRb SINE/MIR 13 237**

**30413634 30418000 C L1PB3 LINE/L1 6869 2395**

**Ortholog in Chimp 30667602-30668245 Minus Nscore 0**

**N positions**

**30665965 30666239 + L2 LINE/L2 2026 2326**

**30667602 30667889 C AluY SINE/Alu 298 11 (R2)**

**30667969 30668245 C AluY SINE/Alu 279 1 (R1)**

**30668355 30668574 + AluSg/x SINE/Alu 87 306**

**30668605 30668815 + MIRb SINE/MIR 13 237**

**30669116 30674331 C L1PB3 LINE/L1 6210 800**

**__________________________________________________________________________________**

**AluSp_14_288 22731859-22732170 INDEL_CAN**

**22730121 22730425 + AluSx SINE/Alu 1 310**

**22730426 22730538 + L2 LINE/L2 3181 3310**

**22730559 22730816 + L1MC5 LINE/L1 7654 7929**

**22730823 22730897 + L2 LINE/L2 3326 3401**

**22731066 22731745 + L1PA5 LINE/L1 5473 6152**

**22731859 22732170 + AluSp SINE/Alu 1 312 ( AluSp_14_288 )**

**22732175 22732805 + HAL1 LINE/L1 1487 2172**

**22732895 22733190 C AluSq SINE/Alu 296 1**

**22733193 22733504 + L1M5 LINE/L1 2250 2574**

**22733505 22733806 + AluSx SINE/Alu 1 305**

**Ortholog in Chimp 22146586-22148290 Plus Nscore 0**

**N positions**

**22144849 22145150 + AluSx SINE/Alu 1 307**

**22145151 22145263 + L2 LINE/L2 3181 3310**

**22145284 22145541 + L1MC5 LINE/L1 7654 7929**

**22145550 22145626 + L2 LINE/L2 3325 3401**

**22145795 22146476 + L1PA5 LINE/L1 5473 6154**

**22146586 22146896 + AluSp SINE/Alu 1 311 (R1)**

**22146897 22146919 + (TA)n Simple_repeat 1 23**

**22147257 22147318 C L2 LINE/L2 3371 3303**

**22147410 22147983 + HAL1 LINE/L1 856 1486**

**22147984 22148290 + AluSp SINE/Alu 1 310 (R2)**

**22148291 22148705 + HAL1 LINE/L1 1487 1932**

**22148715 22149015 + AluSp SINE/Alu 1 305**

**22149020 22149652 + HAL1 LINE/L1 1487 2172**

**22149738 22150033 C AluSq SINE/Alu 296 1**

**__________________________________________________________________________________**

**AluY_15_301c 21258637-21258953 INDEL_CAN**

**21258123 21258427 C AluY SINE/Alu 305 3**

**21258637 21258953 C AluY SINE/Alu 311 1 ( AluY_15_301c )**

**21259236 21259793 + L2 LINE/L2 1707 2296**

**21259892 21260046 + MER58B DNA/MER1_type 167 340**

**Ortholog in Chimp 20958606-20959325 Minus Nscore 0**

**N positions**

**20958095 20958396 C AluY SINE/Alu 302 3**

**20958608 20958908 C AluSg1 SINE/Alu 297 1 (R2)**

**20959019 20959325 C AluY SINE/Alu 301 1 (R1)**

**20959609 20960162 + L2 LINE/L2 1707 2296**

**20960260 20960414 + MER58B DNA/MER1_type 167 340**

**__________________________________________________________________________________**

**AluY_15_319 81696435-81696678 INDEL_CAN**

**81693301 81695032 + ERVL-E LTR/ERVL 2652 4608**

**81695033 81695074 + (TG)n Simple_repeat 2 43**

**81695158 81695688 + ERVL-E LTR/ERVL 4725 5316**

**81695781 81695988 + ERVL-E LTR/ERVL 5443 5655**

**81695995 81696111 + LTR50 LTR/ERVL 1 117**

**81696112 81696407 C AluSx SINE/Alu 298 1**

**81696435 81696678 + AluY SINE/Alu 62 305 ( AluY_15_319 )**

**81696679 81696839 + AluY SINE/Alu 133 308**

**81696845 81697671 + L1M2 LINE/L1 1720 2578**

**81697685 81698208 + L1M4c LINE/L1 2027 2556**

**Ortholog in Chimp 81133747-81137556 Plus Nscore 0**

**N positions**

**81132006 81132343 + ERVL-E LTR/ERVL 4263 4608**

**81132344 81132385 + (TG)n Simple_repeat 2 43**

**81132469 81133015 + ERVL-E LTR/ERVL 4725 5333**

**81133092 81133299 + ERVL-E LTR/ERVL 5443 5655**

**81133306 81133421 + LTR50 LTR/ERVL 1 116**

**81133423 81133719 C AluSx SINE/Alu 299 1**

**81133747 81133987 + AluY SINE/Alu 62 302 (R1)**

**81134007 81135142 + MER11A LTR/ERVK 1 1266**

**81135151 81135336 C Charlie7 DNA/MER1_type 2574 2386**

**81135352 81135639 C Charlie7 DNA/MER1_type 608 349**

**81135643 81136052 + L1M3c LINE/L1 5 432**

**81136826 81136887 + L1M3c LINE/L1 1101 1164**

**81136900 81137290 + L1M4c LINE/L1 1160 1567**

**81137300 81137473 + L1MA4A LINE/L1 6125 6301**

**81137476 81137718 + AluY SINE/Alu 55 309 (R2)**

**81137732 81139093 + L1M3c LINE/L1 1222 2524**

**__________________________________________________________________________________**

**AluY_16_323c 224079-224389 INDEL_CAN**

**222585 222876 + AluSg SINE/Alu 1 290**

**222912 222956 + AT_rich Low_complexity 1 45**

**223082 223382 C AluY SINE/Alu 300 1**

**223506 223784 + AluJb SINE/Alu 2 280**

**223786 224071 C AluSp SINE/Alu 299 14**

**224079 224389 C AluY SINE/Alu 311 1 ( AluY_16_323c )**

**224552 224623 + GA-rich Low_complexity 2 71**

**225714 225860 C L2 LINE/L2 3411 3264**

**225872 226042 + AluSq SINE/Alu 1 172**

**Ortholog in Chimp 226264-227325 Minus Nscore 0**

**N positions**

**224588 224906 + AluSg SINE/Alu 1 310**

**224944 224993 + AT_rich Low_complexity 1 50**

**225120 225256 C AluY SINE/Alu 300 164**

**225296 225571 C AluJb SINE/Alu 276 1**

**225695 225973 + AluJb SINE/Alu 2 280**

**225975 226256 C AluSp SINE/Alu 295 14**

**226264 226566 C AluY SINE/Alu 303 1 (R1)**

**226567 226872 C AluSg SINE/Alu 309 1**

**226895 227015 C FLAM_C SINE/Alu 118 2**

**227025 227325 C AluY SINE/Alu 301 1 (R2)**

**227831 228132 C AluSg SINE/Alu 305 1**

**228133 228275 C AluJo SINE/Alu 136 2**

**228285 228582 C AluY SINE/Alu 298 1**

**228673 228837 + GA-rich Low_complexity 1 157**

**__________________________________________________________________________________**

**AluSx_16_331c 7156978-7157287 INDEL_CAN**

**7155446 7155730 C AluSp SINE/Alu 286 1**

**7155931 7155981 + (CA)n Simple_repeat 1 51**

**7156014 7156315 C AluSx SINE/Alu 302 1**

**7156978 7157287 C AluSx SINE/Alu 312 1 ( AluSx_16_331c )**

**7157288 7157670 + MLT1C LTR/MaLR 81 467**

**7157673 7157889 + MER58A DNA/MER1_type 2 223**

**Ortholog in Chimp 7388979-7389840 Minus Nscore 0**

**N positions**

**7387475 7387759 C AluSp SINE/Alu 286 1**

**7388015 7388316 C AluSx SINE/Alu 302 1**

**7388918 7388972 + MER113 DNA/MER1_type 122 177**

**7388973 7389273 C AluSx SINE/Alu 305 1 (R2)**

**7389274 7389418 + MER113 DNA/MER1_type 178 336**

**7389504 7389522 + MLT1C LTR/MaLR 71 89**

**7389523 7389556 + (TTG)n Simple_repeat 2 35**

**7389557 7389840 C AluSx SINE/Alu 284 1 (R1)**

**7389841 7390223 + MLT1C LTR/MaLR 90 467**

**7390226 7390443 + MER58A DNA/MER1_type 2 223**

**__________________________________________________________________________________**

**AluY_16_336 21952372-21952664 INDEL_CAN**

**21950175 21951005 C L1MB8 LINE/L1 6177 5332**

**21951006 21951313 C AluSx SINE/Alu 308 1**

**21951314 21951365 C L1M LINE/L1 5331 5281**

**21951366 21951669 C AluJo SINE/Alu 302 1**

**21951670 21952208 C L1M LINE/L1 4992 4438**

**21952209 21952370 C AluSg/x SINE/Alu 301 140**

**21952372 21952664 + AluY SINE/Alu 15 305 ( AluY_16_336 )**

**21952665 21952869 C Tigger5a DNA/MER2_type 235 7**

**21952878 21953535 C L1MCa LINE/L1 2458 1639**

**21953568 21953855 C AluJb SINE/Alu 312 1**

**21953860 21954627 C L1MCa LINE/L1 974 2**

**Ortholog in Chimp 22288568-22289521 Plus Nscore 0**

**N positions**

**22286371 22287200 C L1MB8 LINE/L1 6177 5332**

**22287201 22287509 C AluSx SINE/Alu 307 1**

**22287510 22287561 C L1M LINE/L1 5749 5699**

**22287562 22287865 C AluJo SINE/Alu 302 1**

**22287866 22288404 C L1M LINE/L1 5410 4856**

**22288405 22288700 C AluSx SINE/Alu 301 5**

**22288710 22288745 + AT_rich Low_complexity 1 36**

**22288771 22288823 C Tigger5a DNA/MER2_type 346 308**

**22288824 22289128 + AluJb SINE/Alu 1 305**

**22289129 22289215 C Tigger5a DNA/MER2_type 307 236**

**22289216 22289521 + AluY SINE/Alu 1 305**

**22289522 22289726 C Tigger5a DNA/MER2_type 235 7**

**22289732 22290366 C L1M LINE/L1 2341 1639**

**22290414 22290647 C AluJb SINE/Alu 309 55**

**22290648 22290720 C L1M LINE/L1 745 673**

**22290723 22291419 C L1MCa LINE/L1 731 2**

**__________________________________________________________________________________**

**AluSq_16_345 70376699-70377003 INDEL_CAN**

**70375127 70375330 C Charlie8 DNA/MER1_type 211 1**

**70375411 70375559 + L4 LINE/RTE 1519 1671**

**70375610 70375756 C MIR SINE/MIR 189 20**

**70375961 70376258 C AluSx SINE/Alu 302 5**

**70376376 70376697 C Charlie5 DNA/MER1_type 2551 2247**

**70376699 70377003 + AluSq SINE/Alu 1 307 ( AluSq_16_345 )**

**70377007 70377299 + AluY SINE/Alu 1 295**

**70377750 70377871 + AluJo/FLAM SINE/Alu 3 124**

**70377872 70378162 C AluSc SINE/Alu 291 1**

**70378185 70378434 + AluJo SINE/Alu 1 288**

**70378445 70378748 + AluSg SINE/Alu 3 306**

**Ortholog in Chimp 71650502-71651256 Plus Nscore 0**

**N positions**

**71648932 71649135 C Charlie8 DNA/MER1_type 211 1**

**71649216 71649364 + L4 LINE/RTE 1519 1671**

**71649415 71649561 C MIR SINE/MIR 189 20**

**71649764 71650062 C AluSx SINE/Alu 303 5**

**71650179 71650501 C Charlie5 DNA/MER1_type 2551 2261**

**71650502 71650793 + AluSx SINE/Alu 1 294 (R1)**

**71650794 71650853 C Charlie5 DNA/MER1_type 2260 2205**

**71650839 71650918 C Charlie5 DNA/MER1_type 89 7**

**71650959 71651256 + AluSq SINE/Alu 1 298 (R2)**

**71651260 71651552 + AluY SINE/Alu 1 295**

**71652005 71652125 + AluJb SINE/Alu 3 123**

**71652126 71652415 C AluSc SINE/Alu 294 5**

**71652442 71652691 + AluJo SINE/Alu 1 288**

**71652702 71652998 + AluSg SINE/Alu 3 299**

**__________________________________________________________________________________**

**AluSx_16_347 72878810-72879115 INDEL_CAN**

**72877210 72877346 C L2 LINE/L2 3418 3275**

**72877365 72877435 C L2 LINE/L2 2418 2347**

**72877443 72877707 + AluJo SINE/Alu 43 299**

**72877824 72878125 + AluSq SINE/Alu 1 300**

**72878208 72878495 + AluSx SINE/Alu 1 299**

**72878670 72878749 + FLAM SINE/Alu 50 132**

**72878810 72879115 + AluSx SINE/Alu 1 306 ( AluSx_16_347 )**

**72879119 72879290 + AluSp SINE/Alu 130 301**

**72879802 72879954 C AluSx SINE/Alu 153 1**

**Ortholog in Chimp 74187937-74188363 Plus Nscore 0**

**N positions**

**74186318 74186580 C L2 LINE/L2 2611 2347**

**74186588 74186852 + AluJo SINE/Alu 43 299**

**74186969 74187269 + AluSq SINE/Alu 1 299**

**74187352 74187620 + AluSx SINE/Alu 1 300**

**74187797 74187876 + FLAM SINE/Alu 50 132**

**74187937 74188231 + AluSx SINE/Alu 1 295 (R1)**

**74188237 74188544 + AluSp SINE/Alu 1 307 (R2)**

**74189050 74189202 C AluSx SINE/Alu 153 1**

**__________________________________________________________________________________**

**AluSg_16_350 80017422-80017602 OCCUPIED**

**80015692 80015981 + AluJb SINE/Alu 2 297**

**80016323 80016610 + AluJb SINE/Alu 1 292**

**80016632 80016986 + L1MB3 LINE/L1 5601 5955**

**80016987 80017117 + AluSg SINE/Alu 1 134**

**80017118 80017421 + AluY SINE/Alu 1 290**

**80017422 80017602 + AluSg SINE/Alu 135 303 ( AluSg_16_350 )**

**80017603 80017828 + L1MB3 LINE/L1 5956 6176**

**80017926 80018223 C AluY SINE/Alu 299 1**

**80018241 80018320 C L2 LINE/L2 3370 3289**

**80018356 80018547 + MIRb SINE/MIR 67 261**

**80018775 80018851 + MIR SINE/MIR 88 168**

**80018852 80019094 C AluJb SINE/Alu 324 87**

**80019100 80019262 C L1MC5 LINE/L1 7925 7759**

**Ortholog in Chimp 81552266-81552446 Plus Nscore 0**

**N positions**

**81550537 81550825 + AluJb SINE/Alu 2 299**

**81551167 81551455 + AluJb SINE/Alu 1 293**

**81551477 81551831 + L1MB3 LINE/L1 5601 5955**

**81551832 81551962 + AluSg SINE/Alu 1 134**

**81551963 81552265 + AluY SINE/Alu 1 290**

**81552266 81552445 + AluSg SINE/Alu 135 302 (OCCUPIED)**

**81552446 81552671 + L1MB3 LINE/L1 5956 6176**

**81552769 81553070 C AluY SINE/Alu 302 1**

**81553088 81553167 C L2 LINE/L2 3370 3289**

**81553203 81553394 + MIRb SINE/MIR 67 261**

**81553623 81553697 + MIR SINE/MIR 88 166**

**81553698 81553923 C AluJb SINE/Alu 307 87**

**81553929 81554091 C L1MC5 LINE/L1 7925 7759**

**__________________________________________________________________________________**

**AluSx_17_353 1255830-1255958 OCCUPIED**

**1254107 1254406 C AluSx SINE/Alu 303 1**

**1254644 1254945 + AluSg SINE/Alu 1 302**

**1254946 1255144 C L2 LINE/L2 2094 1881**

**1255830 1255958 + AluSx SINE/Alu 3 131 ( AluSx_17_353 )**

**1255959 1256274 + AluSg SINE/Alu 1 310**

**1256275 1256380 + AluSx SINE/Alu 132 231**

**1256381 1256510 + AluSg/x SINE/Alu 171 300**

**1256562 1256872 + AluJo SINE/Alu 7 310**

**1256901 1257182 + AluJo SINE/Alu 11 297**

**1257183 1257473 + AluSg SINE/Alu 1 292**

**Ortholog in Chimp 1367493-1367621 Plus Nscore 0**

**N positions**

**1365989 1366295 + AluSg SINE/Alu 1 307**

**1366296 1366398 C L2 LINE/L2 2094 1982**

**1367493 1367696 + AluSx SINE/Alu 3 212 (R1)**

**1367697 1367750 + AluSq/x SINE/Alu 78 131 (R2)**

**1367751 1368052 + AluSg SINE/Alu 1 296**

**1368098 1368401 + AluJo SINE/Alu 7 310**

**1368423 1368704 + AluJo SINE/Alu 11 297**

**1368705 1368962 + AluSg SINE/Alu 1 258**

**__________________________________________________________________________________**

**AluY_17_363 10009045-10009363 INDEL_CAN**

**10007809 10008023 + MIRb SINE/MIR 27 266**

**10008065 10008192 C MER5B DNA/MER1_type 178 7**

**10008203 10008501 + AluSc SINE/Alu 1 298**

**10008559 10008750 + FAM SINE/Alu 1 49**

**10008754 10009042 + AluJo SINE/Alu 2 300**

**10009045 10009363 + AluY SINE/Alu 1 311 ( AluY_17_363 )**

**10010054 10010362 C AluJo SINE/Alu 311 3**

**10010586 10010762 C L1ME4a LINE/L1 6027 5849**

**Ortholog in Chimp 46279690-46280167 Minus Nscore 0**

**N positions**

**46278697 46279006 + AluJo SINE/Alu 3 312**

**46279690 46280009 C AluY SINE/Alu 311 1 (R2)**

**46280010 46280303 C AluSq SINE/Alu 293 1 (R1)**

**46280306 46280456 C FLAM_C SINE/Alu 143 1**

**46280460 46280651 C FAM SINE/Alu 49 1**

**46280709 46281010 C AluSc SINE/Alu 301 1**

**46281021 46281148 + MER5B DNA/MER1_type 7 178**

**46281190 46281369 C MIRb SINE/MIR 266 75**

**__________________________________________________________________________________**

**AluSq_17_369c 18996479-18996615 OCCUPIED**

**18995112 18995346 + AluSg/x SINE/Alu 83 306**

**18995617 18995916 C AluSx SINE/Alu 304 3**

**18995919 18996167 C AluSx SINE/Alu 304 56**

**18996183 18996477 C AluSx SINE/Alu 295 1**

**18996479 18996615 C AluSq/x SINE/Alu 137 1 ( AluSq_17_369c )**

**18997008 18997299 + AluSx SINE/Alu 1 292**

**18997302 18997607 + AluY SINE/Alu 1 283**

**18997679 18997843 C FRAM SINE/Alu 172 6**

**18998019 18998330 + AluSg SINE/Alu 1 310**

**Ortholog in Chimp 37068465-37068601 Minus Nscore 0**

**N positions**

**37066827 37067061 + AluSg/x SINE/Alu 83 305**

**37067332 37067631 C AluSx SINE/Alu 283 3**

**37067634 37067875 C AluSx SINE/Alu 295 56**

**37067891 37068187 C AluSx SINE/Alu 297 1**

**37068189 37068325 C AluSq/x SINE/Alu 137 1**

**37068193 37068463 C AluSx SINE/Alu 299 1**

**37068469 37068601 C FLAM_C SINE/Alu 133 1 (OCCUPIED)**

**37068994 37069285 + AluSx SINE/Alu 1 292**

**37069288 37069588 + AluY SINE/Alu 1 305**

**37069657 37069821 C FRAM SINE/Alu 172 6**

**37069997 37070292 + AluSg SINE/Alu 1 294**

**__________________________________________________________________________________**

**AluSc_17_373 27033712-27033817 INDEL_CAN**

**27031858 27032298 + L1ME3B LINE/L1 647 1080**

**27032299 27032601 + AluY SINE/Alu 1 302**

**27032602 27032675 + L1ME3B LINE/L1 1081 1150**

**27032685 27033026 + L1ME3B LINE/L1 2861 3205**

**27033023 27033146 + L1M4 LINE/L1 4963 5085**

**27033147 27033421 C AluSx SINE/Alu 306 1**

**27033422 27033699 + L1M4 LINE/L1 5086 5366**

**27033712 27033817 + AluSc SINE/Alu 35 136 ( AluSc_17_373 )**

**27033942 27034073 + L1MA10 LINE/L1 6196 6327**

**27034075 27034610 + L1ME3B LINE/L1 3172 3719**

**27034611 27034911 C AluJo SINE/Alu 312 2**

**27034912 27035116 + L1ME3B LINE/L1 3720 4009**

**27035117 27035399 + AluJb SINE/Alu 10 292**

**Ortholog in Chimp 25586514-25586931 Minus Nscore 0**

**N positions**

**25584919 25585211 C AluJb SINE/Alu 292 1**

**25585212 25585418 C L1ME3B LINE/L1 4013 3721**

**25585419 25585719 + AluJo SINE/Alu 2 312**

**25585720 25586256 C L1ME3B LINE/L1 3720 3172**

**25586258 25586389 C L1MA10 LINE/L1 6327 6196**

**25586514 25586647 C AluSc SINE/Alu 136 7 (R2)**

**25586658 25586931 C AluSx SINE/Alu 311 35 (R1)**

**25586939 25587221 C L1M4 LINE/L1 5369 5086**

**25587222 25587488 + AluSx SINE/Alu 1 306**

**25587489 25587612 C L1M4 LINE/L1 5085 4963**

**25587609 25587948 C L1ME3B LINE/L1 3205 2861**

**25587958 25588017 C L1ME3B LINE/L1 1150 1091**

**25588018 25588329 C AluY SINE/Alu 311 1**

**25588330 25588766 C L1ME3B LINE/L1 1090 647**

**__________________________________________________________________________________**

**AluSx_17_380c 40963413-40963730 INDEL_CAN**

**40961575 40962230 + L1M4 LINE/L1 1174 1812**

**40962231 40962521 + AluJb SINE/Alu 2 290**

**40962522 40962741 + L1MB7 LINE/L1 1813 2025**

**40962743 40963050 + AluSx SINE/Alu 2 309**

**40963061 40963233 + L1MB7 LINE/L1 2253 2429**

**40963234 40963405 C AluJo SINE/Alu 173 1**

**40963413 40963730 C AluSx SINE/Alu 312 1 ( AluSx_17_380c )**

**40963801 40963841 C L1P4 LINE/L1 5751 5711**

**40963836 40964341 + L1MB7 LINE/L1 5081 5591**

**40964523 40965577 + L1MB7 LINE/L1 5594 6663**

**Ortholog in Chimp 11974234-11975562 Plus Nscore 0**

**N positions**

**11972389 11973442 C L1MB7 LINE/L1 6663 5594**

**11973624 11974128 C L1MB7 LINE/L1 5591 5081**

**11974123 11974163 + L1P4 LINE/L1 5711 5751**

**11974234 11974510 + AluSx SINE/Alu 1 277 (R2)**

**11974511 11974629 C AluSq/x SINE/Alu 120 2**

**11974737 11975251 C L1MB7 LINE/L1 4265 3758**

**11975257 11975562 + AluJo SINE/Alu 7 308 (R1)**

**11975570 11975741 + AluJo SINE/Alu 1 173**

**11975742 11975915 C L1MB7 LINE/L1 2429 2252**

**11975925 11976233 C AluSx SINE/Alu 309 1**

**11976234 11976327 C L1MB7 LINE/L1 2025 1930**

**11976810 11976948 C L1MB7 LINE/L1 1961 1813**

**11976966 11977142 C AluJb SINE/Alu 290 120**

**__________________________________________________________________________________**

**AluSx_17_390c 73650641-73650959 INDEL_CAN**

**73649096 73649185 + Charlie7 DNA/MER1_type 2513 2611**

**73649307 73649371 C MIR SINE/MIR 93 29**

**73649838 73649990 C MER5B DNA/MER1_type 176 6**

**73650211 73650270 C L2 LINE/L2 3373 3310**

**73650370 73650462 + MER5A DNA/MER1_type 11 105**

**73650528 73650638 + MIRb SINE/MIR 86 202**

**73650641 73650959 C AluSx SINE/Alu 312 1 ( AluSx_17_390c )**

**Ortholog in Chimp 77812499-77812973 Minus Nscore 0**

**N positions**

**77810952 77811041 + Charlie7 DNA/MER1_type 2513 2611**

**77811163 77811227 C MIR SINE/MIR 93 29**

**77811694 77811846 C MER5B DNA/MER1_type 176 6**

**77812069 77812147 C L2 LINE/L2 3414 3332**

**77812228 77812320 + MER5A DNA/MER1_type 11 105**

**77812386 77812496 + MIRb SINE/MIR 86 202**

**77812499 77812659 C AluSq SINE/Alu 299 134 (R2)**

**77812660 77812973 C AluSq SINE/Alu 313 1 (R1)**

**__________________________________________________________________________________**

**AluSq_17_393c 76595100-76595224 INDEL_CAN**

**76593465 76593623 + AluSc SINE/Alu 1 115**

**76593624 76593944 + AluSg SINE/Alu 1 308**

**76593945 76593957 + AluSc SINE/Alu 116 125**

**76593958 76594272 + AluSx SINE/Alu 1 312**

**76594273 76594456 + AluSc SINE/Alu 126 309**

**76594471 76595099 C L1ME1 LINE/L1 5698 5003**

**76595100 76595224 C AluSq/x SINE/Alu 129 1 ( AluSq_17_393c )**

**76595225 76596036 C L1ME1 LINE/L1 5002 4104**

**76596045 76596256 + AluY SINE/Alu 1 213**

**76596258 76596408 + AluJo/FRAM SINE/Alu 145 295**

**Ortholog in Chimp 81182909-81183754 Minus Nscore 0**

**N positions**

**81181274 81181437 + AluSx SINE/Alu 2 118**

**81181438 81181760 + AluSg SINE/Alu 1 310**

**81181761 81181773 + AluSx SINE/Alu 119 118**

**81181774 81182070 + AluSx SINE/Alu 3 312**

**81182071 81182255 + AluSx SINE/Alu 119 302**

**81182280 81182902 C L1M3 LINE/L1 5698 5009**

**81182913 81183075 C AluJo SINE/Alu 287 129 (R2)**

**81183076 81183378 C AluSx SINE/Alu 312 9**

**81183379 81183429 C AluJo SINE/Alu 128 84**

**81183440 81183754 C AluSx SINE/Alu 312 1 (R1)**

**81183757 81184563 C L1M3 LINE/L1 4954 4102**

**81184572 81184866 + AluY SINE/Alu 1 296**

**81184972 81185027 + T-rich Low_complexity 3 57**

**81185108 81185563 + L3 LINE/CR1 3370 3948**

**__________________________________________________________________________________**

**AluYc2_17_394 76596045-76596256 INDEL_CAN**

**76594471 76595099 C L1ME1 LINE/L1 5698 5003**

**76595100 76595224 C AluSq/x SINE/Alu 129 1**

**76595225 76596036 C L1ME1 LINE/L1 5002 4104**

**76596045 76596256 + AluY SINE/Alu 1 213 ( AluYc2_17_394 )**

**76596258 76596408 + AluJo/FRAM SINE/Alu 145 295**

**76596816 76597108 C AluSx SINE/Alu 292 1**

**76597287 76597598 C AluSx SINE/Alu 312 1**

**Ortholog in Chimp 81184572-81186417 Plus Nscore 0**

**N positions**

**81182913 81183075 C AluJo SINE/Alu 287 129**

**81183076 81183378 C AluSx SINE/Alu 312 9**

**81183379 81183429 C AluJo SINE/Alu 128 84**

**81183440 81183754 C AluSx SINE/Alu 312 1**

**81183757 81184563 C L1M3 LINE/L1 4954 4102**

**81184572 81184866 + AluY SINE/Alu 1 296**

**81184972 81185027 + T-rich Low_complexity 3 57**

**81185108 81185563 + L3 LINE/CR1 3370 3948**

**81185658 81185968 + AluSx SINE/Alu 1 312**

**81185995 81186101 + L2 LINE/L2 3274 3419**

**81186278 81186569 + AluJo SINE/Alu 2 295**

**81186980 81187268 C AluSx SINE/Alu 289 1**

**81187447 81187758 C AluSx SINE/Alu 312 1**

**81187848 81188367 + L1MEa LINE/L1 102 619**

**__________________________________________________________________________________**

**AluSc_17_395c 76639784-76640086 INDEL_CAN**

**76639105 76639215 + (CA)n Simple_repeat 1 111**

**76639694 76639773 + L2 LINE/L2 2727 2805**

**76639784 76640086 C AluSc SINE/Alu 309 1 ( AluSc_17_395c )**

**76640089 76640384 C AluJb SINE/Alu 296 1**

**Ortholog in Chimp 81231039-81231665 Minus Nscore 0**

**N positions**

**81230357 81230410 + (CA)n Simple_repeat 1 54**

**81230949 81231028 + L2 LINE/L2 2727 2805**

**81231039 81231341 C AluSc SINE/Alu 309 1 (R2)**

**81231345 81231385 + (TTTA)n Simple_repeat 4 44**

**81231386 81231665 C AluY SINE/Alu 279 1**

**81231668 81231963 C AluJb SINE/Alu 296 1 (R1)**

**__________________________________________________________________________________**

**AluSp_18_402 41881553-41881664 INDEL_CAN**

**41879925 41880117 + L1M LINE/L1 4737 4932**

**41880381 41880541 + MER5A DNA/MER1_type 18 183**

**41881223 41881250 + AT_rich Low_complexity 1 28**

**41881262 41881546 + AluSg SINE/Alu 6 271**

**41881553 41881664 + AluSp/q SINE/Alu 182 293 ( AluSp_18_402 )**

**41881730 41881927 + L1MC4 LINE/L1 7164 7352**

**41881928 41882432 + MLT1D LTR/MaLR 1 505**

**41882433 41882972 + L1MC4 LINE/L1 7353 7914**

**41882973 41883020 + (TG)n Simple_repeat 1 48**

**41883021 41883137 + L1MC4 LINE/L1 7915 8042**

**41883156 41883446 + L1PA13 LINE/L1 5874 6163**

**Ortholog in Chimp 42264881-42268817 Plus Nscore 0**

**N positions**

**42263254 42263445 + L1M5 LINE/L1 4738 4932**

**42263709 42263869 + MER5A DNA/MER1_type 18 183**

**42264551 42264578 + AT_rich Low_complexity 1 28**

**42264585 42264900 + AluSg SINE/Alu 1 297**

**42264906 42265047 C MIRb SINE/MIR 258 119**

**42265174 42265305 + MIRb SINE/MIR 22 157**

**42266268 42266333 C MER41B LTR/ERV1 546 481**

**42266430 42266489 + L1ME1 LINE/L1 4545 4605**

**42266539 42266709 + L1MC4a LINE/L1 6284 6505**

**42266710 42266910 + MER63A DNA/AcHobo 1 209**

**42266911 42267309 + L1MC4a LINE/L1 6506 6934**

**42267310 42267623 + AluSx SINE/Alu 1 312**

**42267624 42267777 + L1MC4a LINE/L1 6935 7077**

**42267781 42267871 + L1ME1 LINE/L1 5670 5752**

**42267872 42268179 + AluJb SINE/Alu 1 309**

**42268180 42268525 + L1ME1 LINE/L1 5753 6110**

**42268526 42268820 + AluSp SINE/Alu 1 296**

**42268886 42269083 + L1MC4 LINE/L1 7164 7352**

**42269084 42269588 + MLT1D LTR/MaLR 1 505**

**42269589 42270127 + L1MC4 LINE/L1 7353 7907**

**42270128 42270156 + (TG)n Simple_repeat 1 29**

**42270157 42270275 + L1MC4 LINE/L1 7908 8041**

**42270294 42270586 + L1PA13 LINE/L1 5874 6163**

**__________________________________________________________________________________**

**AluSc_19_407 4835874-4836158 OCCUPIED**

**4834339 4834616 C AluY SINE/Alu 309 1**

**4834669 4834863 + L2 LINE/L2 2975 3173**

**4834945 4835239 C AluSq SINE/Alu 296 2**

**4835241 4835509 C AluSx SINE/Alu 304 30**

**4835569 4835870 + AluSp SINE/Alu 1 303**

**4835874 4836158 + AluSc SINE/Alu 6 290 ( AluSc_19_407 )**

**4836386 4836405 + (TTTAA)n Simple_repeat 3 22**

**4836434 4836723 + AluSc SINE/Alu 1 290**

**4836944 4836992 + AT_rich Low_complexity 1 49**

**4836995 4837306 + AluY SINE/Alu 1 310**

**4837308 4837425 C AluSq/x SINE/Alu 118 2**

**4837449 4837555 C L2 LINE/L2 2916 2809**

**4837586 4837652 C MER53 DNA 188 122**

**Ortholog in Chimp 4970098-4970386 Plus Nscore 0**

**N positions**

**4968556 4968835 C AluY SINE/Alu 311 1**

**4968888 4969063 + L2 LINE/L2 2975 3154**

**4969164 4969460 C AluSq SINE/Alu 297 2**

**4969462 4969733 C AluSx SINE/Alu 305 30**

**4969793 4970094 + AluSp SINE/Alu 1 303**

**4970098 4970391 + AluSg SINE/Alu 6 295 (OCCUPIED)**

**4970615 4970639 + (TTTAA)n Simple_repeat 3 27**

**4970668 4970980 + AluY SINE/Alu 1 310**

**4970982 4971098 C AluSq/x SINE/Alu 118 2**

**4971122 4971228 C L2 LINE/L2 2916 2809**

**4971259 4971325 C MER53 DNA 188 122**

**4971344 4971364 + (TC)n Simple_repeat 2 22**

**4971378 4971667 C AluSq SINE/Alu 290 2**

**4971669 4971940 C AluSx SINE/Alu 301 30**

**__________________________________________________________________________________**

**AluY_19_408 5788494-5788807 INDEL_CAN**

**5786908 5787204 C AluSx SINE/Alu 292 1**

**5787218 5787526 C AluSq SINE/Alu 309 1**

**5787558 5787878 + AluJo SINE/Alu 1 305**

**5787879 5787908 + AluJo SINE/Alu 284 312**

**5787974 5788042 + Charlie5 DNA/MER1_type 2246 2313**

**5788043 5788348 C AluSx SINE/Alu 306 1**

**5788349 5788491 + Charlie5 DNA/MER1_type 2314 2460**

**5788494 5788807 + AluY SINE/Alu 1 312 ( AluY_19_408 )**

**5790293 5790573 C L2 LINE/L2 3092 2773**

**Ortholog in Chimp 5946571-5948462 Plus Nscore 0**

**N positions**

**5944967 5945263 C AluSx SINE/Alu 292 1**

**5945277 5945588 C AluSq SINE/Alu 312 1**

**5945620 5945952 + AluJo SINE/Alu 1 309**

**5945953 5945980 + AluJo SINE/Alu 284 310**

**5946039 5946119 + Charlie5 DNA/MER1_type 2234 2313**

**5946120 5946425 C AluSx SINE/Alu 306 1**

**5946426 5946568 + Charlie5 DNA/MER1_type 2314 2460**

**5946571 5946702 + AluSq/x SINE/Alu 1 132 (R1)**

**5946705 5946998 + AluJb SINE/Alu 1 305**

**5947001 5947307 + AluSq SINE/Alu 1 309**

**5947310 5947479 + Charlie5 DNA/MER1_type 2416 2582**

**5947543 5947853 + AluSx SINE/Alu 1 312**

**5947922 5948154 + AluSq SINE/Alu 1 296**

**5948158 5948466 + AluY SINE/Alu 1 306 (R2)**

**5949947 5950227 C L2 LINE/L2 3092 2773**

**__________________________________________________________________________________**

**AluY_19_412 8111795-8112101 INDEL_CAN**

**8110325 8110388 + L3 LINE/CR1 4337 4400**

**8111073 8111352 + AluSx SINE/Alu 8 289**

**8111437 8111650 C MIR SINE/MIR 252 22**

**8111795 8112101 + AluY SINE/Alu 1 304 ( AluY_19_412 )**

**8112102 8112277 + AluSg/x SINE/Alu 134 310**

**Ortholog in Chimp 8368732-8369628 Plus Nscore 0**

**N positions**

**8367258 8367321 + L3 LINE/CR1 4337 4400**

**8368006 8368285 + AluSx SINE/Alu 8 289**

**8368370 8368583 C MIR SINE/MIR 252 22**

**8368732 8369036 + AluSp SINE/Alu 1 306 (R1)**

**8369392 8369492 + MIR SINE/MIR 29 147**

**8369496 8369802 + AluSx SINE/Alu 1 308 (R2)**

**__________________________________________________________________________________**

**AluSx_19_423 22608329-22608623 INDEL_CAN**

**22606564 22606877 C AluSp SINE/Alu 311 1**

**22607017 22607321 C AluY SINE/Alu 298 1**

**22607671 22607841 C MER5B DNA/MER1_type 178 1**

**22607967 22608263 + AluSx SINE/Alu 1 297**

**22608329 22608623 + AluSx SINE/Alu 1 294 ( AluSx_19_423 )**

**22609553 22609886 C MER93a LTR/ERV1 359 19**

**Ortholog in Chimp 22710451-22711975 Minus Nscore 0**

**N positions**

**22709195 22709521 + MER93a LTR/ERV1 19 359**

**22710444 22710744 C AluSg SINE/Alu 299 1**

**22710805 22711090 C AluSx SINE/Alu 296 5**

**22711124 22711180 C Charlie5 DNA/MER1_type 2569 2514**

**22711194 22711486 + AluSg SINE/Alu 5 299**

**22711493 22711795 + AluSg SINE/Alu 1 303**

**22711948 22712223 C AluSx SINE/Alu 276 1**

**22712348 22712512 + MER5B DNA/MER1_type 1 164**

**22712699 22712754 C L2 LINE/L2 3355 3297**

**22712879 22713168 + AluSq SINE/Alu 4 293**

**22713171 22713481 + MER61E LTR/ERV1 304 779**

**__________________________________________________________________________________**

**AluY_19_432 51681056-51681359 INDEL_CAN**

**51679554 51679731 + AluSx SINE/Alu 130 297**

**51679732 51679991 + MER65D LTR/ERV1 209 473**

**51679995 51680114 + L2 LINE/L2 3258 3373**

**51680241 51680525 + AluSx SINE/Alu 3 284**

**51680526 51680548 + (TAA)n Simple_repeat 2 24**

**51680569 51680777 C L1MC4a LINE/L1 5963 5741**

**51681056 51681359 + AluY SINE/Alu 1 303 ( AluY_19_432 )**

**51681360 51681518 + AluSq SINE/Alu 134 292**

**51681580 51681710 + (CA)n Simple_repeat 2 131**

**51682336 51682492 + MIR SINE/MIR 6 164**

**51682620 51682791 C MER5B DNA/MER1_type 178 3**

**51682830 51683000 C MER5A DNA/MER1_type 187 1**

**Ortholog in Chimp 52093760-52097034 Plus Nscore 0**

**N positions**

**52092252 52092429 + AluSx SINE/Alu 130 297**

**52092430 52092689 + MER65D LTR/ERV1 209 473**

**52092693 52092812 + L2 LINE/L2 3258 3373**

**52092939 52093223 + AluSx SINE/Alu 3 284**

**52093224 52093252 + (TAA)n Simple_repeat 2 30**

**52093273 52093481 C L1MC4a LINE/L1 5963 5741**

**52093760 52094029 + AluY SINE/Alu 1 308 (R1)**

**52094337 52094743 C MER77 LTR/ERVL 606 180**

**52094744 52095044 + AluSq SINE/Alu 1 302**

**52095045 52095227 C MER77 LTR/ERVL 179 28**

**52095228 52095523 C AluSc SINE/Alu 291 1**

**52095524 52095555 C MER77 LTR/ERVL 27 1**

**52095558 52095933 + MSTA LTR/MaLR 1 428**

**52096359 52096387 + (CA)n Simple_repeat 1 29**

**52096399 52096543 + (CA)n Simple_repeat 1 144**

**52096568 52096711 + AluSq SINE/Alu 1 124**

**52096712 52097022 + AluY SINE/Alu 1 310 (R2)**

**52097023 52097193 + AluSq SINE/Alu 125 292**

**52097255 52097385 + (CA)n Simple_repeat 2 131**

**52098011 52098214 + MIR SINE/MIR 6 214**

**52098369 52098467 + MER5B DNA/MER1_type 90 176**

**52098506 52098676 C MER5A DNA/MER1_type 187 1**

**__________________________________________________________________________________**

**AluSg_19_436 57443800-57444107 INDEL_CAN**

**57442040 57442467 C MER77 LTR/ERVL 596 184**

**57442476 57442726 + AluSc SINE/Alu 1 251**

**57443103 57443514 C MER74C LTR/ERVL 454 3**

**57443666 57443797 + AluSp SINE/Alu 1 132**

**57443800 57444107 + AluSg SINE/Alu 1 309 ( AluSg_19_436 )**

**57444265 57444369 C MLT1C LTR/MaLR 106 1**

**57444647 57444689 + (TG)n Simple_repeat 2 44**

**57444830 57444853 + AT_rich Low_complexity 1 24**

**57444883 57445188 C AluSx SINE/Alu 305 1**

**57445278 57445305 + AT_rich Low_complexity 1 28**

**57445309 57445785 + MLT2B1 LTR/ERVL 1 510**

**Ortholog in Chimp 57908800-57909917 Plus Nscore 0**

**N positions**

**57907029 57907466 C MER77 LTR/ERVL 603 182**

**57907473 57907723 + AluSc SINE/Alu 1 251**

**57908103 57908514 C MER74C LTR/ERVL 454 3**

**57908666 57908949 + AluSp SINE/Alu 1 285 (R1)**

**57908950 57908981 + (TAA)n Simple_repeat 2 33**

**57909063 57909356 C AluSx SINE/Alu 309 1**

**57909611 57909918 + AluSg SINE/Alu 2 310 (R2)**

**57910066 57910170 C MLT1C LTR/MaLR 106 1**

**57910446 57910468 + (TG)n Simple_repeat 2 24**

**57910611 57910634 + AT_rich Low_complexity 1 24**

**57910664 57910958 C AluSx SINE/Alu 294 1**

**57911048 57911075 + AT_rich Low_complexity 1 28**

**57911081 57911555 + MLT2B1 LTR/ERVL 3 510**

**__________________________________________________________________________________**

**AluSx_20_449 34703693-34704014 INDEL_CAN**

**34702211 34702322 + MER3 DNA/MER1_type 15 126**

**34702323 34702629 C AluSq SINE/Alu 304 5**

**34702630 34702698 + MER3 DNA/MER1_type 127 208**

**34703693 34704014 + AluSx SINE/Alu 1 312 ( AluSx_20_449 )**

**34704073 34704142 + MIRb SINE/MIR 105 174**

**34704246 34704395 C AluJo SINE/Alu 275 126**

**34704396 34704701 C AluSp SINE/Alu 305 1**

**34704702 34704829 C AluJo SINE/Alu 125 1**

**34704847 34704984 + L2 LINE/L2 3261 3413**

**34705178 34705297 C L2 LINE/L2 3369 3243**

**34705487 34705765 + AluSx SINE/Alu 1 279**

**Ortholog in Chimp 33797116-33797905 Plus Nscore 0**

**N positions**

**33795620 33795731 + MER3 DNA/MER1_type 15 126**

**33795732 33796053 C AluSq SINE/Alu 300 5**

**33796054 33796122 + MER3 DNA/MER1_type 127 208**

**33797116 33797421 + AluSx SINE/Alu 1 296 (R1)**

**33797496 33797581 C AluSg/x SINE/Alu 191 131**

**33797597 33797905 + AluSx SINE/Alu 26 312 (R2)**

**33797964 33798033 + MIRb SINE/MIR 105 174**

**33798137 33798286 C AluJo SINE/Alu 275 126**

**33798287 33798592 C AluSp SINE/Alu 305 1**

**33798593 33798720 C AluJo SINE/Alu 125 1**

**33798741 33798875 + L2 LINE/L2 3264 3413**

**33799065 33799184 C L2 LINE/L2 3369 3243**

**33799324 33799369 + MIR SINE/MIR 108 154**

**33799374 33799652 + AluSx SINE/Alu 1 279**

**__________________________________________________________________________________**

**AluSx_20_452 39148035-39148339 INDEL_CAN**

**39146982 39147228 + L1M5 LINE/L1 5032 5298**

**39147270 39147475 + L1M5 LINE/L1 5366 5585**

**39148035 39148339 + AluSx SINE/Alu 1 305 ( AluSx_20_452 )**

**39148516 39148553 + L2 LINE/L2 3382 3419**

**39149746 39149775 + AT_rich Low_complexity 1 30**

**Ortholog in Chimp 38351911-38352514 Plus Nscore 0**

**N positions**

**38350851 38351097 + L1M5 LINE/L1 5032 5298**

**38351142 38351348 + L1M5 LINE/L1 5365 5585**

**38351911 38352218 + AluSx SINE/Alu 1 308 (R1)**

**38352219 38352516 + AluSg SINE/Alu 8 305 (R2)**

**38352692 38352729 + L2 LINE/L2 3382 3419**

**38353921 38353950 + AT_rich Low_complexity 1 30**

**__________________________________________________________________________________**

**AluJb_20_458c 56958335-56958603 OCCUPIED**

**56956865 56958076 + HSMAR1 DNA/Mariner 57 1284**

**56958079 56958130 + AT_rich Low_complexity 1 52**

**56958089 56958298 + (TA)n Simple_repeat 1 220**

**56958335 56958603 C AluJb SINE/Alu 281 5 ( AluJb_20_458c )**

**56958941 56959093 + AluJb SINE/Alu 2 135**

**56959307 56959641 C AluSx SINE/Alu 325 1**

**56959727 56960546 + L1M LINE/L1 2639 3501**

**Ortholog in Chimp 56663661-56663929 Minus Nscore 0**

**N positions**

**56661557 56662766 + HSMAR1 DNA/Mariner 59 1284**

**56662810 56663090 + (TA)n Simple_repeat 1 288**

**56663091 56663362 C AluJb SINE/Alu 284 5**

**56663457 56663624 + (TA)n Simple_repeat 2 180**

**56663658 56663929 C AluJo SINE/Alu 284 5 (OCCUPIED)**

**56664266 56664419 + AluJb SINE/Alu 1 135**

**56664420 56664451 + AT_rich Low_complexity 1 32**

**56664632 56664966 C AluSx SINE/Alu 325 1**

**56665050 56665411 + L1M LINE/L1 2639 3011**

**56665412 56665451 + (TA)n Simple_repeat 2 41**

**__________________________________________________________________________________**

**AluSg_21_460c 19463381-19463551 INDEL_CAN**

**19462194 19462264 + A-rich Low_complexity 2 72**

**19462895 19463016 + (TA)n Simple_repeat 1 126**

**19463381 19463551 C AluSg/x SINE/Alu 307 137 ( AluSg_21_460c )**

**Ortholog in Chimp 19255809-19256991 Minus Nscore 0**

**N positions**

**19254612 19254684 + GA-rich Low_complexity 3 75**

**19255317 19255401 + (TATATG)n Simple_repeat 4 90**

**19255410 19255440 + (TA)n Simple_repeat 1 30**

**19255809 19256120 C AluSx SINE/Alu 307 1**

**19256574 19256599 + AT_rich Low_complexity 1 26**

**19256699 19256992 C AluSx SINE/Alu 297 1**

**__________________________________________________________________________________**

**AluJb_22_470 28145512-28145819 INDEL_CAN**

**28143928 28144216 C AluSg1 SINE/Alu 298 1**

**28144368 28144482 + Charlie8 DNA/MER1_type 33 145**

**28144483 28144786 C AluSg SINE/Alu 304 1**

**28144787 28144845 + Charlie8 DNA/MER1_type 146 193**

**28145457 28145508 + Alu SINE/Alu 1 52**

**28145512 28145819 + AluJb SINE/Alu 1 312 ( AluJb_22_470 )**

**28146322 28146609 C AluSg SINE/Alu 296 9**

**28146616 28146929 C AluSx SINE/Alu 312 1**

**28146934 28147131 C MIRb SINE/MIR 247 27**

**28147278 28147439 + AluSg/x SINE/Alu 122 306**

**Ortholog in Chimp 28273125-28273674 Plus Nscore 0**

**N positions**

**28273070 28273124 + AluJb SINE/Alu 1 52**

**28273125 28273418 + AluSc SINE/Alu 1 294 (R1)**

**28273419 28273679 + AluJb SINE/Alu 53 304 (R2)**

**28274177 28274470 C AluSg SINE/Alu 296 3**

**28274471 28274769 C AluSx SINE/Alu 299 1**

**28274774 28274971 C MIRb SINE/MIR 247 27**

**28275105 28275279 + AluSc SINE/Alu 103 303**

**__________________________________________________________________________________**

**AluSg_22_473 32569279-32569459 INDEL_CAN**

**32567776 32567879 C MIRb SINE/MIR 144 46**

**32568301 32568330 + AT_rich Low_complexity 1 30**

**32568651 32568808 + MER3 DNA/MER1_type 1 194**

**32568898 32568945 + (CAAAA)n Simple_repeat 3 50**

**32568957 32569126 + L2 LINE/L2 2996 3165**

**32569279 32569459 + AluSg/x SINE/Alu 137 317 ( AluSg_22_473 )**

**32569826 32570095 + AluSq SINE/Alu 43 311**

**32570619 32570707 + MIRm SINE/MIR 76 171**

**32570711 32571005 C AluSp SINE/Alu 298 1**

**Ortholog in Chimp 32750462-32751544 Plus Nscore 0**

**N positions**

**32748980 32749098 C MIRb SINE/MIR 144 26**

**32749505 32749534 + AT_rich Low_complexity 1 30**

**32749855 32750012 + MER3 DNA/MER1_type 1 194**

**32750102 32750129 + (CAAAA)n Simple_repeat 3 30**

**32750140 32750309 + L2 LINE/L2 2996 3165**

**32750461 32750765 + AluSx SINE/Alu 1 299**

**32750771 32750987 + MIR SINE/MIR 30 251**

**32751235 32751544 + AluSx SINE/Alu 1 309**

**32751911 32752024 + AluSq/x SINE/Alu 43 150**

**32752674 32752948 C AluSp SINE/Alu 278 1**

**__________________________________________________________________________________**

**AluSp_X_480 419516-419818 INDEL_CAN**

**418310 418600 C AluSx SINE/Alu 291 1**

**418894 419188 C AluJb SINE/Alu 293 1**

**419516 419818 + AluSp SINE/Alu 1 303 ( AluSp_X_480 )**

**419829 420146 + AluSq SINE/Alu 1 313**

**420524 420809 C AluSx SINE/Alu 286 1**

**420821 420953 C AluSq/x SINE/Alu 133 1**

**421001 421307 C AluSc SINE/Alu 305 1**

**Ortholog in Chimp 392371-393439 Plus Nscore 53.93**

**N positions 392375-392950;**

**391260 391553 C AluSx SINE/Alu 294 1**

**391847 392143 C AluJb SINE/Alu 295 1**

**392951 393127 + AluSq SINE/Alu 125 301 (R1)**

**393138 393437 + AluSp SINE/Alu 1 301**

**393448 393760 + AluSq SINE/Alu 1 313 (R2)**

**393761 393779 + AluSq SINE/Alu 288 302**

**__________________________________________________________________________________**
