# Supplementary material for: RISCI - Repeat Induced Sequence Changes Identifier: a comprehensive, comparative genomics-based, in silico subtractive hybridization pipeline to identify repeat induced sequence changes in closely related genomes
Source: BMC Bioinformatics. 2010 Dec 26;11:609. doi: 10.1186/1471-2105-11-609 (PMC3024322; doi:10.1186/1471-2105-11-609)
Supplement: Additional file 17 — Additional figures. Figures S1 - Alignment signatures for M_DISRUPTED, S2 - RISCI facilitates precise demarcation of transposon boundaries, S3 - Variation in RepeatMasker annotated boundaries and RISCI predicted boundary, S4 - The non repeat tag forms a part of upstream query for most loci. [file 1471-2105-11-609-S17.DOC]

Additional file 17 – Additional Figures

**
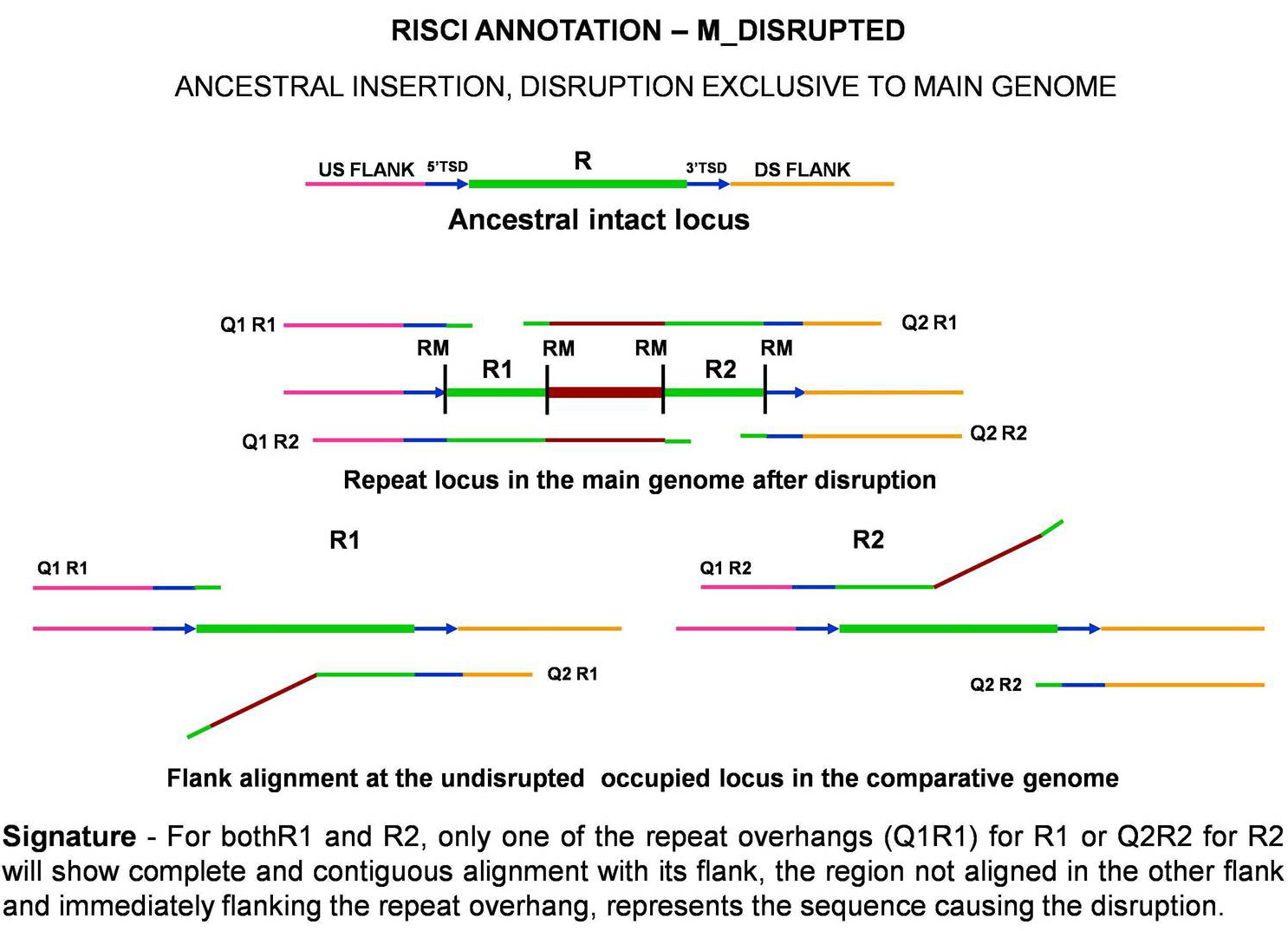
**

**Figure S1 - Alignment signatures for M_DISRUPTED** - Disruption of the ancestral occupied locus in reference or main genome. The original repeat is disrupted into R1 and R2 by a sequence represented by the brown line. The orthologous locus in the comparative genome remains intact. Upon alignment at the orthologous locus in the comparative genome only one of the repeat overhangs, Q1R1 for R and Q2R2 for R2 aligns completely and contiguously with its flank. The region that does not align in the other flank and immediately flanking the repeat overhang represents the sequence causing disruption. Green lines – repeat sequence, blue lines – target site duplications, pink line – 5’ flank of the intact repeat, orange line -3’ flank of the original repeat.


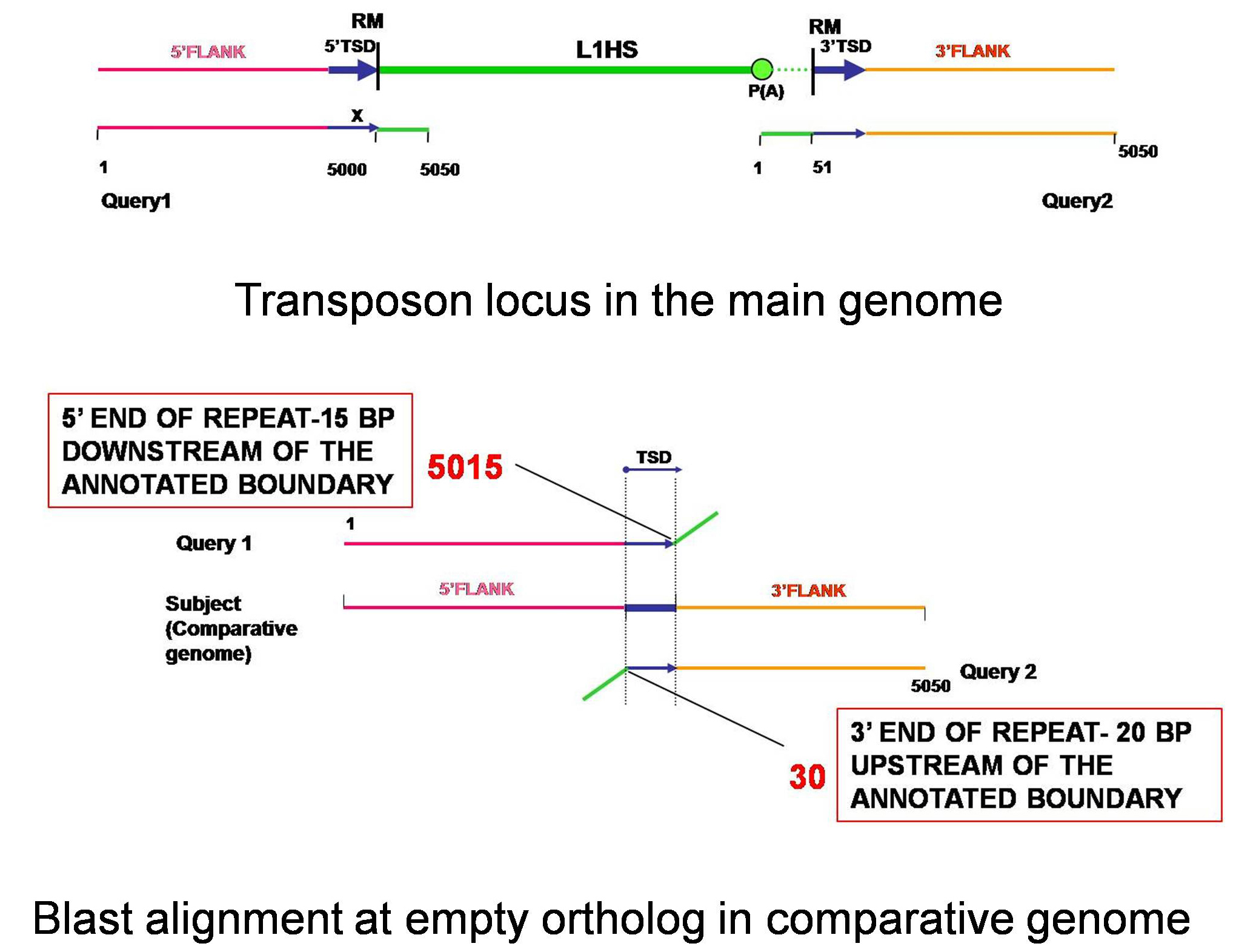


**Figure S2** **– Precise identification of transposon boundaries by RISCI and improvement over Repeat Masker annotation** – Flanks with 50 base repeat overhangs as per RepeatMasker annotated boundaries are aligned at the empty orthologous locus in the comparative genome. The region of overlap between the queries gives the target site duplication sequence. If the TSD begins from the 51st base (counting from the 3’ end) of the upstream query and 51^st^ base (counting from the 5’ end) in the downstream query, the RepeatMasker annotation of the transposon boundary is perfect. Deviations from this indicate imprecise annotation of transposon boundaries. Pink line – 5’ flank, blue arrows – target site duplications, Green line – transposon, Orange line – 3’ flank

**
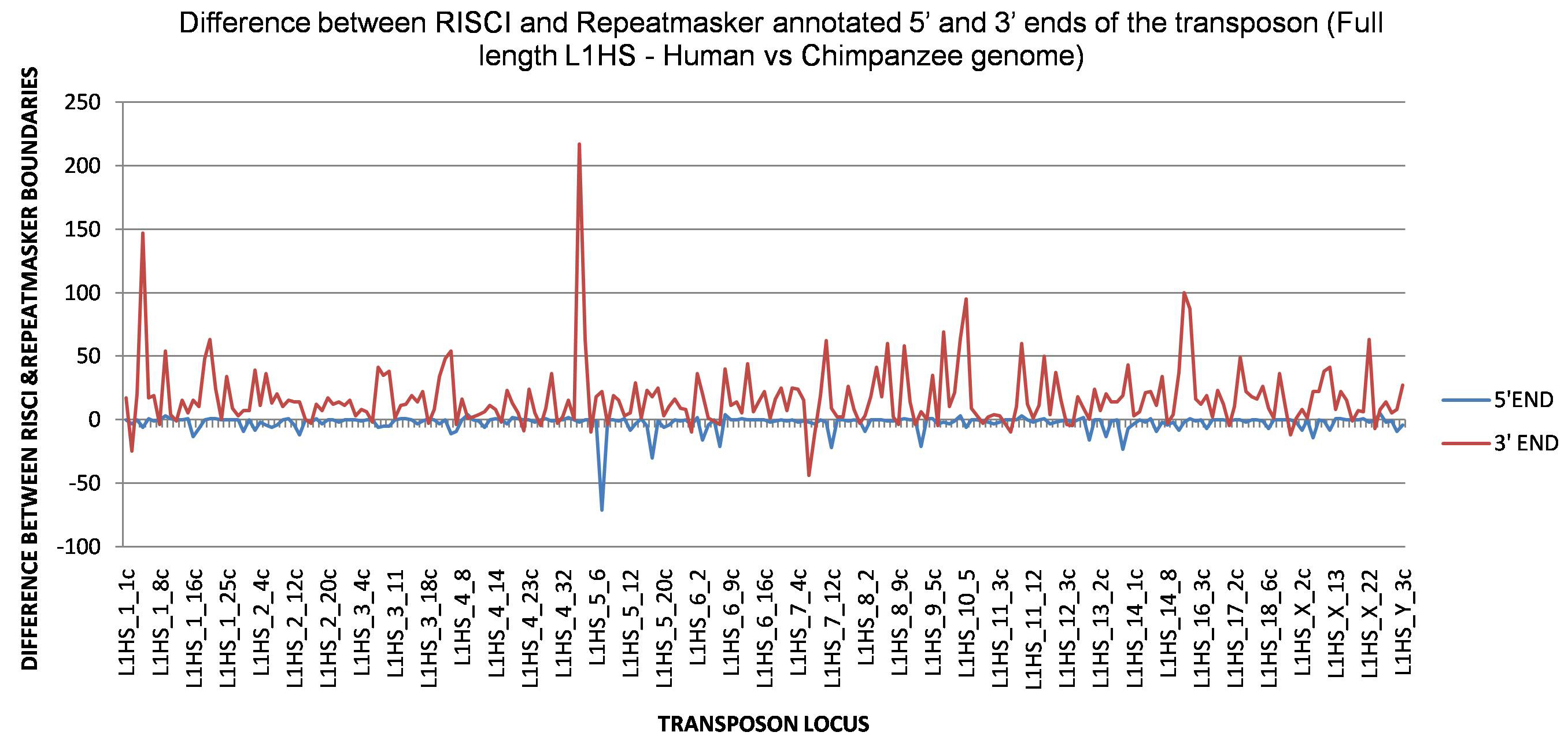
**

**Figure S3** – **Extent of deviation between RISCI and RepeatMasker annotated boundaries for full length L1HS elements -** Loci inferred as CAN or PAC in comparison with chimpanzee genome were considered. The difference between RISCI and RepeatMasker annotated boundaries are plotted for each locus. Peaks below zero indicate overestimation and above zero indicate underestimation of the boundaries by RepeatMasker. As is clearly seen, the 5’ boundary is more precisely annotated by RepeatMasker than the 3’ boundary.

**
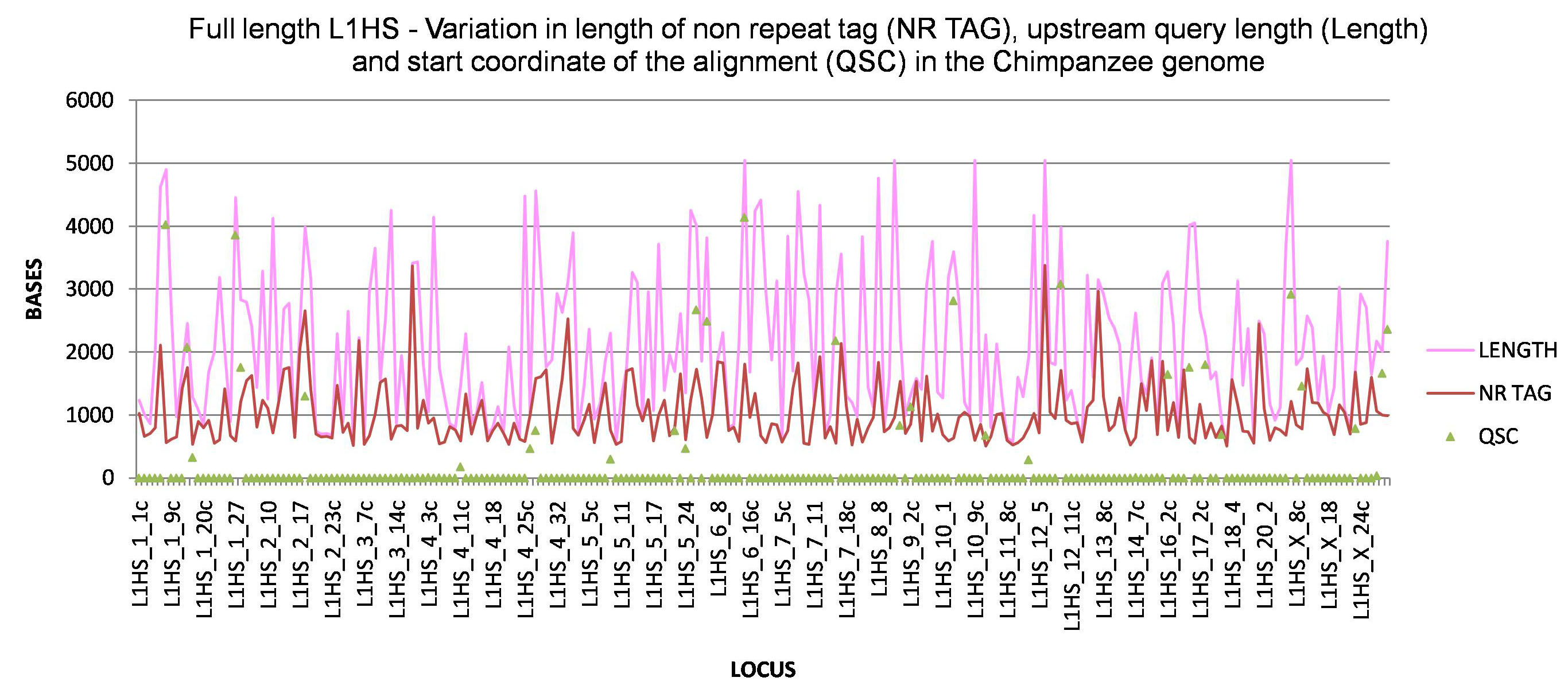
**

**Figure S4 – Non repeat tag at 5’ end of upstream flank mostly forms a part of the aligned flank sequence -** Variation in length of non repeat tag, length of the upstream query and the start coordinate of the alignment in the chimpanzee genome. The non repeat tag forms the 5’ end of the query. As can be seen, for most loci the alignment starts from 1, the entire non repeat tag aligning at the identified orthologous locus in the comparative genome. In 9 of the 234 loci shown here, the non repeat tag aligns only partially at the identified orthologous locus. In another 16 loci, the non repeat tag does not align with the identified orthologous in the chimpanzee genome.
